# Supplementary material for: Multi-omics signatures of the human early life exposome
Source: Nat Commun. 2022 Nov 21;13:7024. doi: 10.1038/s41467-022-34422-2 (PMC9678903; doi:10.1038/s41467-022-34422-2)
Supplement: Supplementary file 3 — Description of Additional Supplementary Files [file 41467_2022_34422_MOESM3_ESM.pdf]

Description of Additional Supplementary Files

File Name: Supplementary Data 1
Description: Methods: HELIX sample, exposome, omics, lists of proteins and serum and urinary metabolites, multiple testing correction and public catalogues

File Name: Supplementary Data 2
Description: ExWAS: results of the association between pregnancy and childhood exposomes and child molecular phenotypes, ordered by p-value

File Name: Supplementary Data 3
Description: Overlap of exposome-omics associations between pregnancy and childhood periods

File Name: Supplementary Data 4
Description: Results of the multi-exposure models for the pregnancy and childhood exposomes and child molecular phenotypes

File Name: Supplementary Data 5
Description: Node attributes of the integrative network analysis of the pregnancy and childhood ExWAS

File Name: Supplementary Data 6
Description: Overlap of CpGs associated with the pregnancy and childhood exposomes with CpGs associated with traits and exposures in the EWAS Catalog and EWAS Atlas

File Name: Supplementary Data 7
Description: Functional enrichment analyses of molecular features associated with the pregnancy and childhood exposomes

File Name: Supplementary Data 8
Description: CpGs associated with maternal smoking, maternal cotinine and/or maternal Cd levels

File Name: Supplementary Data 9
Description: Overlap of exposome-metabolite associations between urine and serum matrices

File Name: Supplementary Data 10
Description: Overlap of exposome-omics associations across molecular layers in blood

File Name: Supplementary Data 11
Description: Forest plots by cohort of the 1,170 significant associations. For each cohort, point estimate of the association is represented bounded by the 95% confidence interval (CI) with a

horizontal line and the cohort weight as a grey square. The 95% CI from the fixed and random effects meta-analysis are shown as diamonds. For all omics, except DNA methylation, effect size is reported as a log2 fold change (log2FC) of the molecular phenotype levels between categories of discrete exposure variables or for interquartile range (IQR) of continuous exposure variables. For DNA methylation, it is reported as a difference in methylation levels between categories of discrete exposure variables or for IQR of continuous exposure variables. MD: mean difference. Heterogeneity is reported (I2). A) Pregnancy exposome vs blood DNA methylation (n=1173). B) Pregnancy exposome vs blood transcriptome (n=1007). C) Pregnancy exposome vs blood miRNA expression (n=941). D) Pregnancy exposome vs plasma proteins (n=1170). E) Pregnancy exposome vs serum metabolites (n=1198). F) Pregnancy exposome vs urinary metabolites (n=1198). G) Childhood exposome vs blood DNA methylation (n=1173). H) Childhood exposome vs blood transcriptome (n=1007). I) Childhood exposome vs blood miRNA expression (n=941). J) Childhood exposome vs plasma proteins (n=1170). K) Childhood exposome vs serum metabolites (n=1198). L) Childhood exposome vs urinary metabolites (n=1198).
