# Supplementary figures and images for: Multi-omics signatures of the human early life exposome
Source: Nat Commun. 2022 Nov 21;13:7024. doi: 10.1038/s41467-022-34422-2 (PMC9678903; doi:10.1038/s41467-022-34422-2)

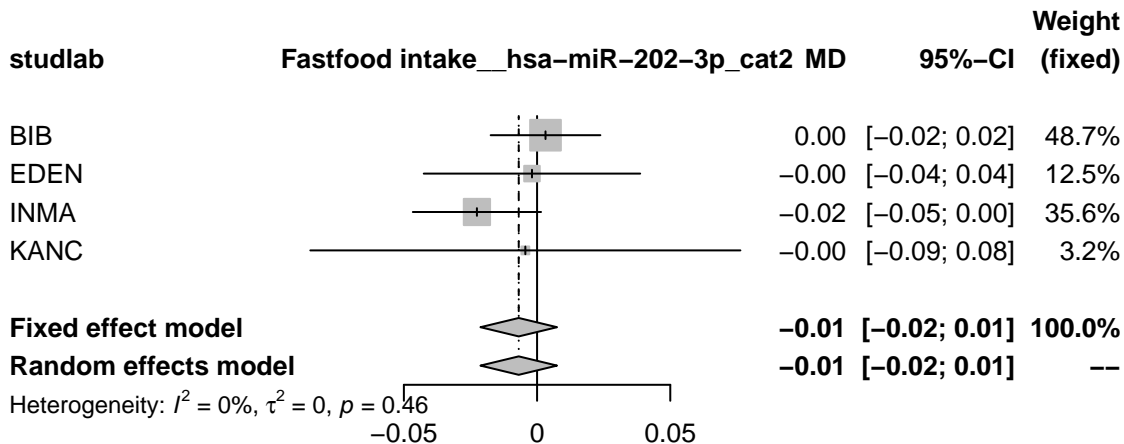

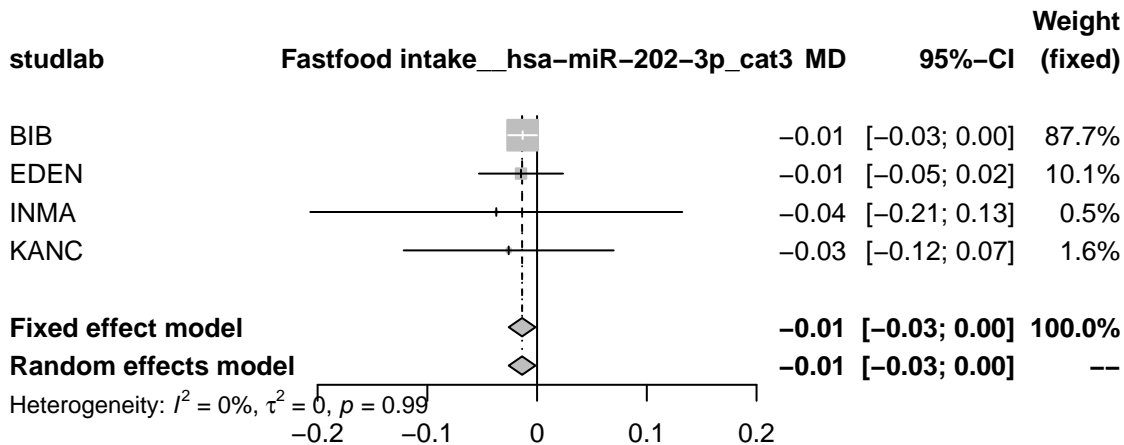

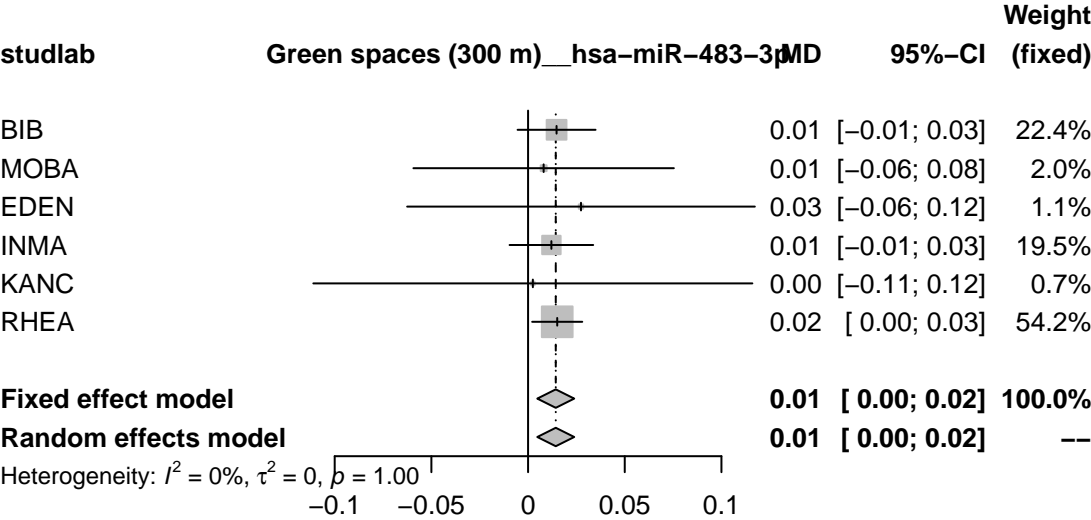

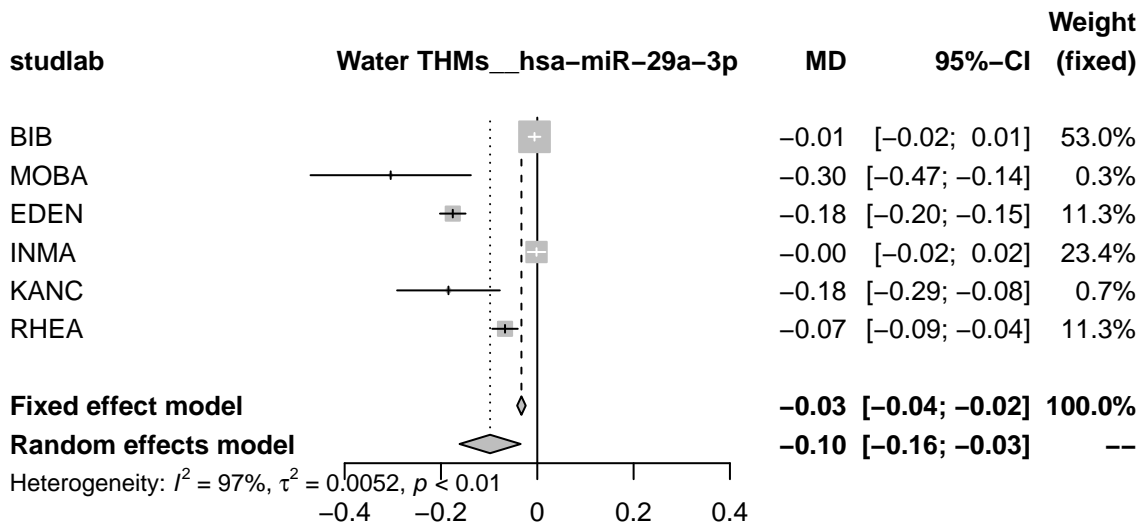

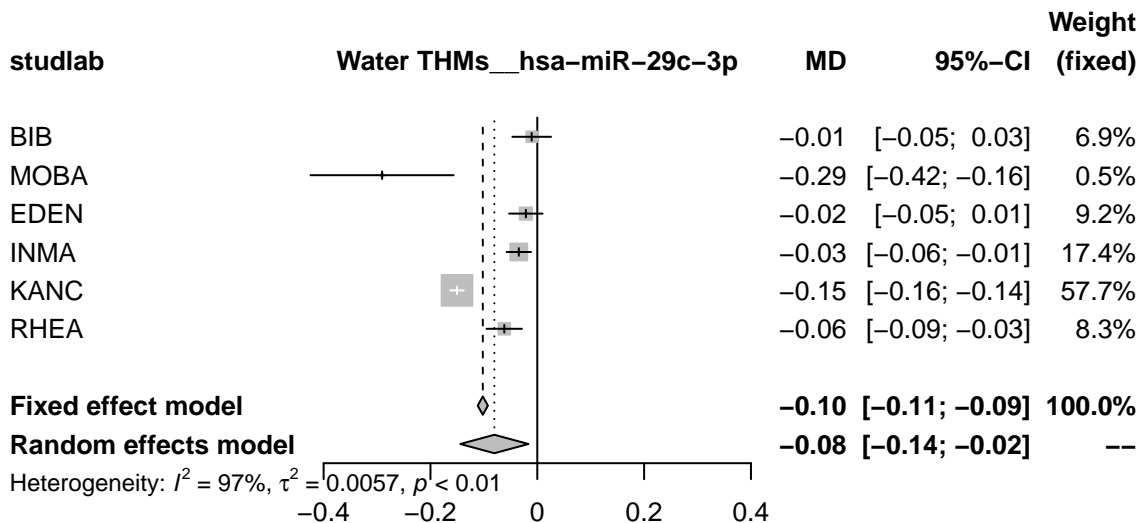

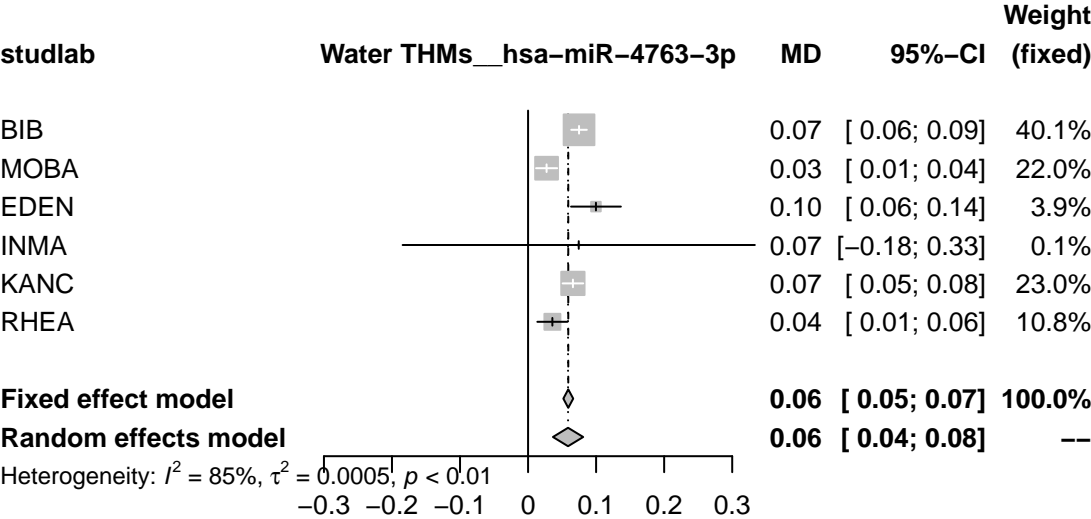

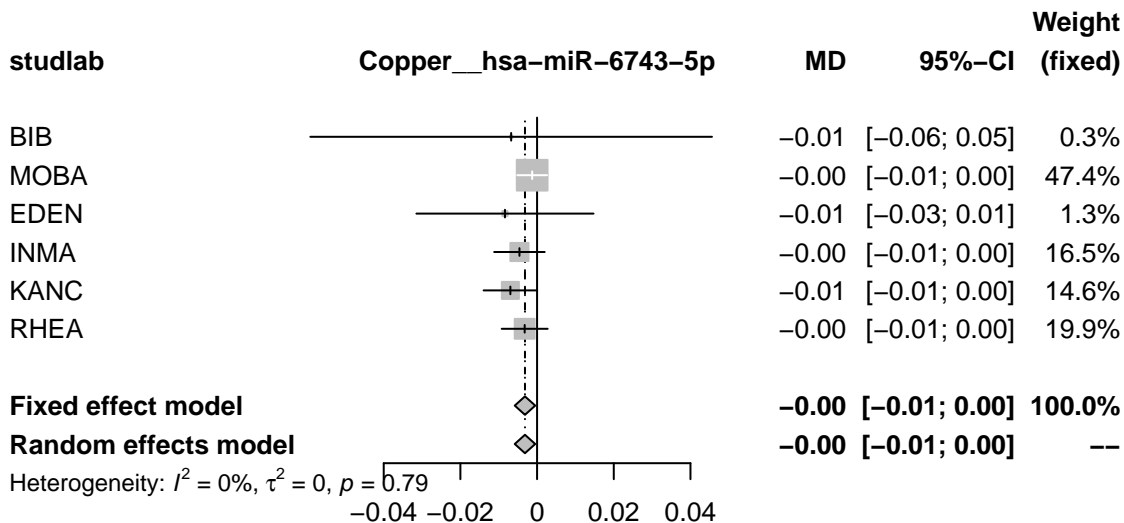

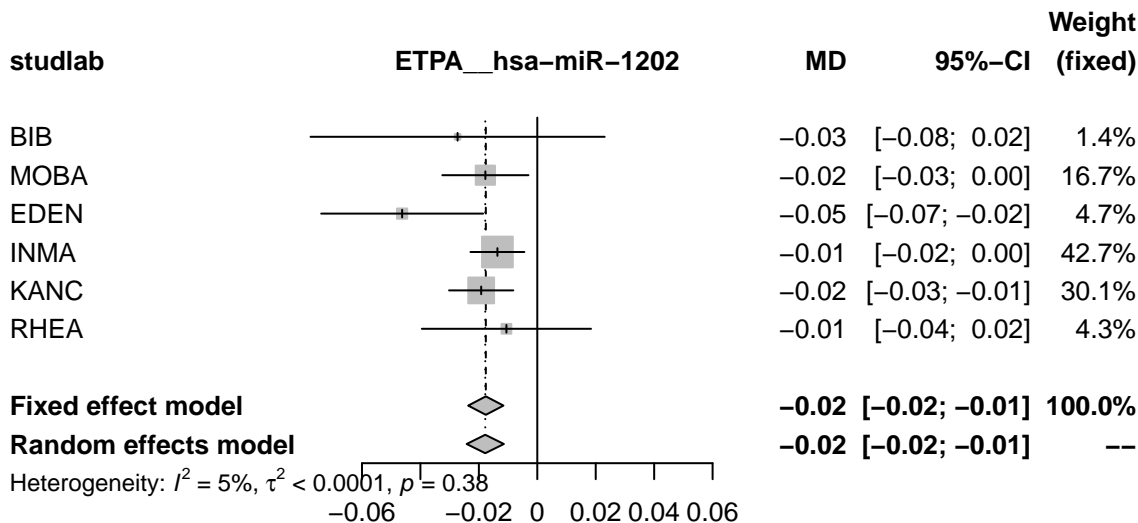

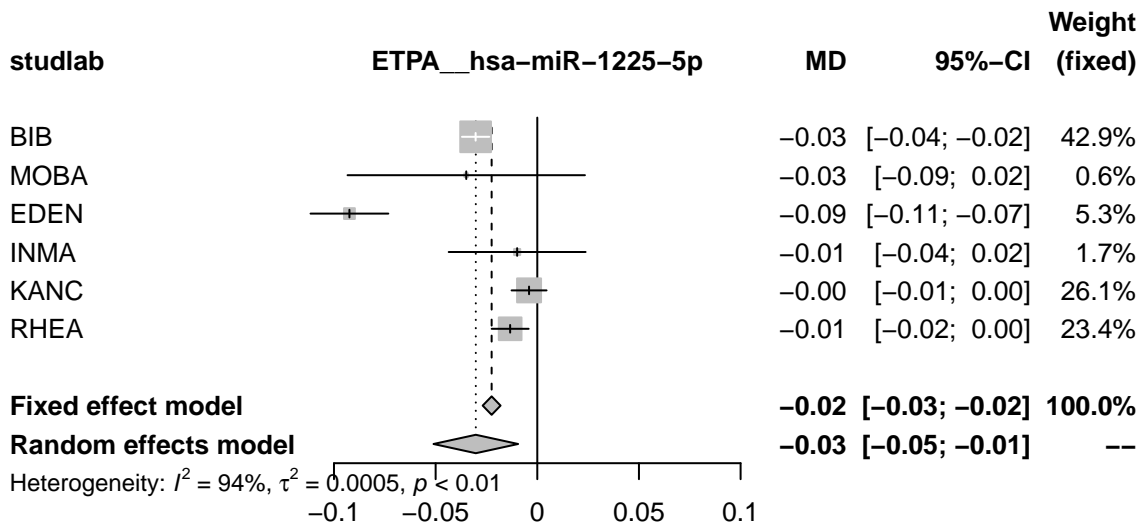

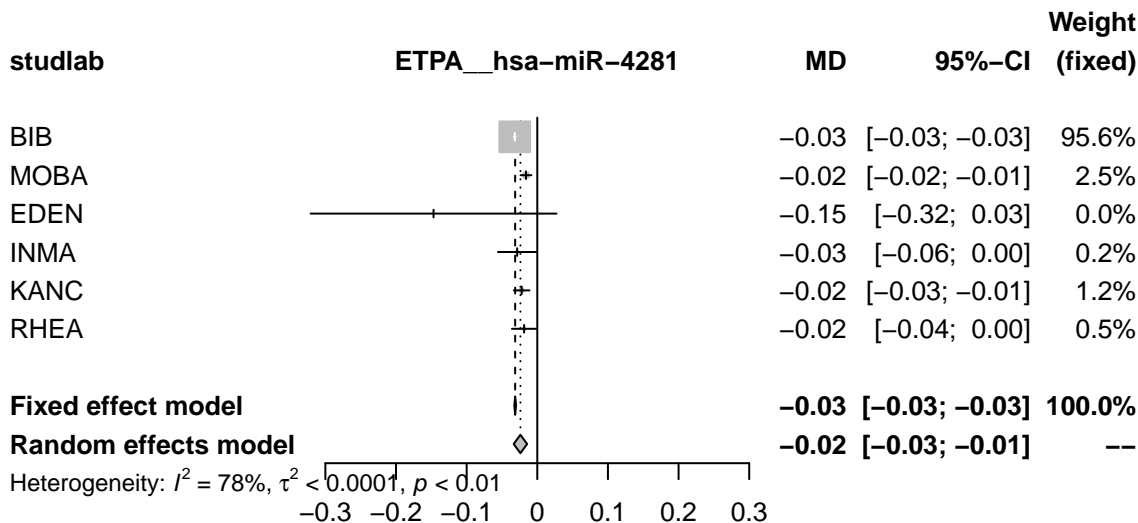

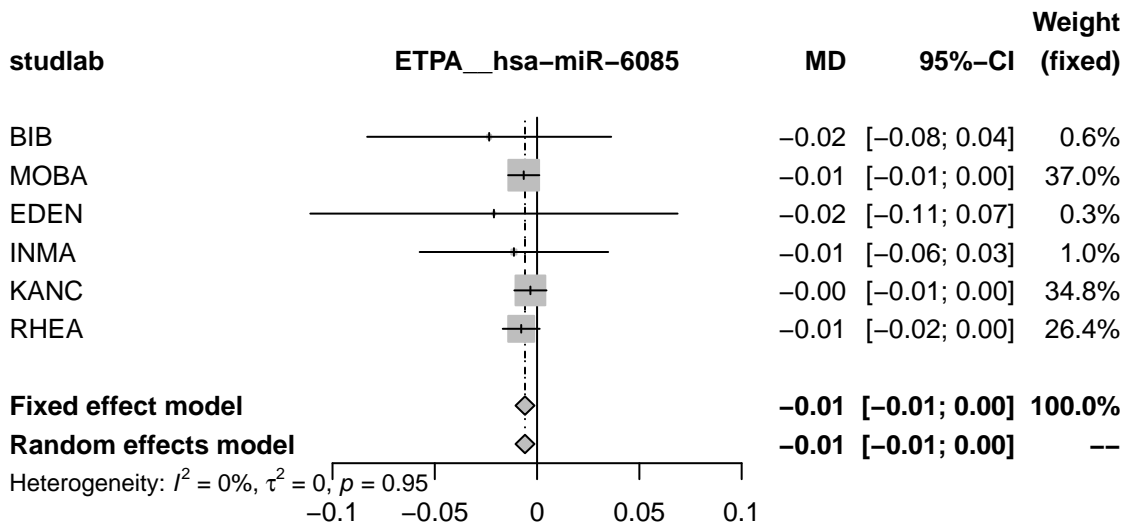

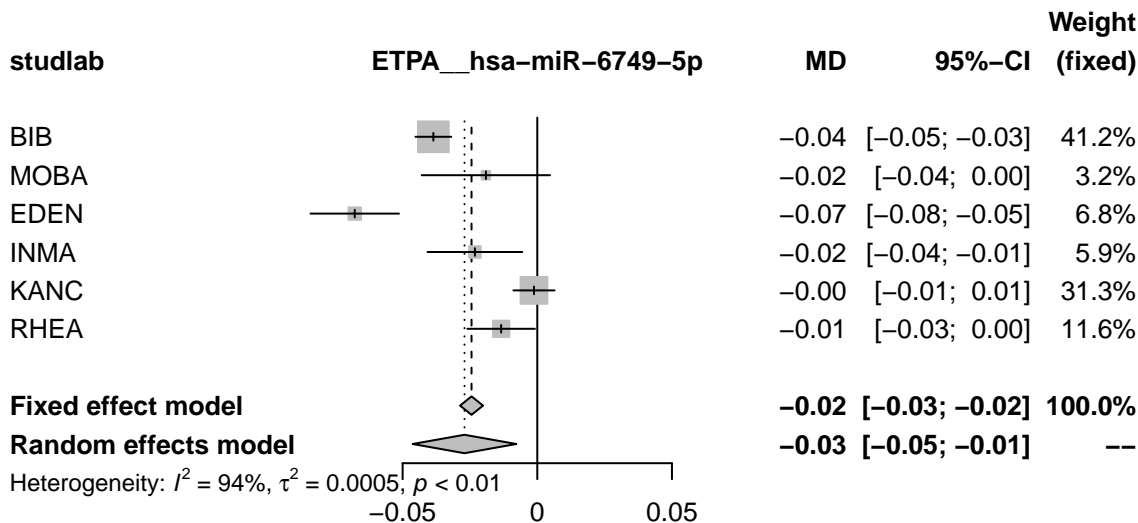

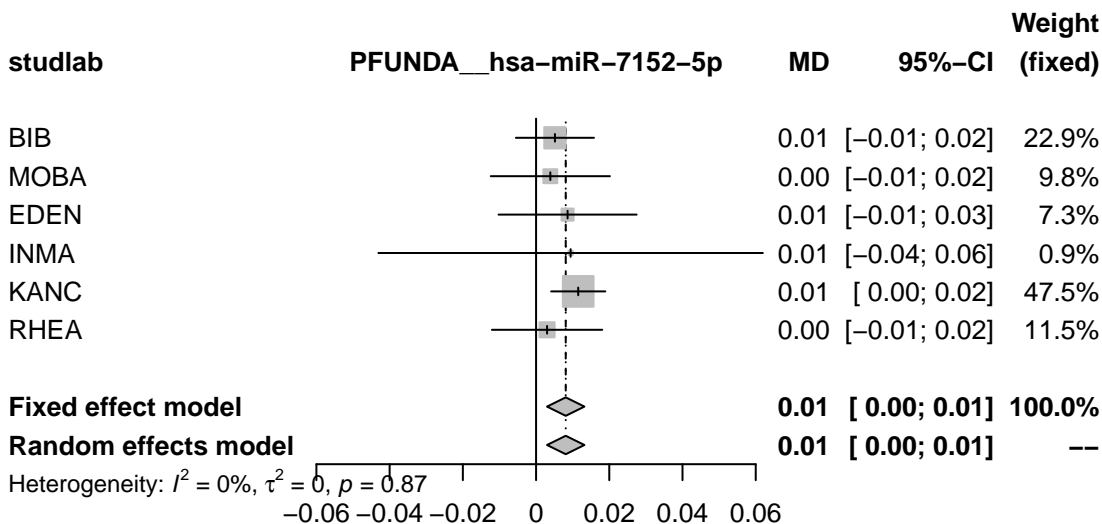

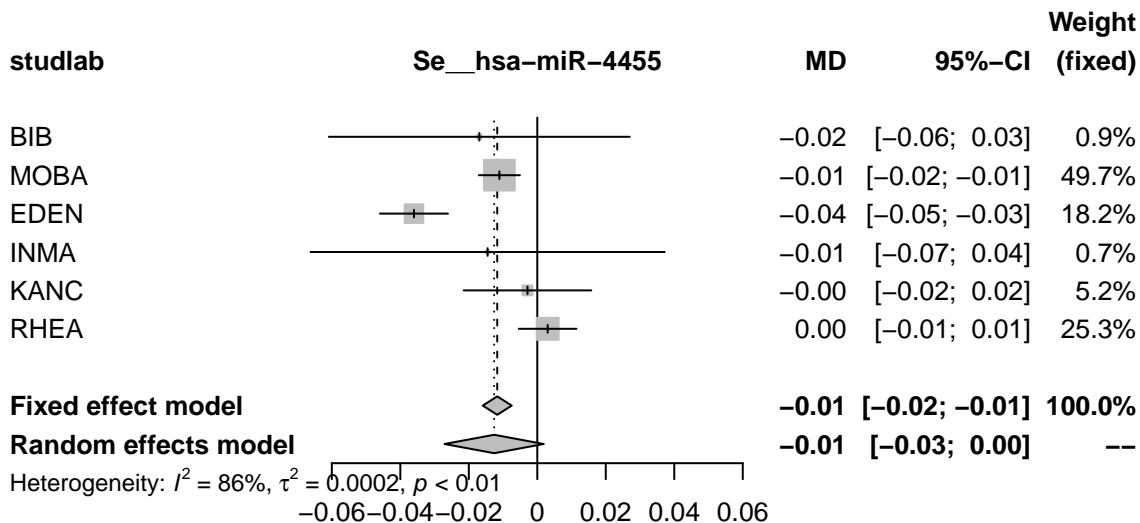

Supplement: Supplementary file 14 — Supplementary Dataset 11 [file 41467_2022_34422_MOESM14_ESM.zip › HELIX_ExpOmics_FigS2_Forestplots/HELIX_ExpOmics_FigS2C_mirna_preg.pdf]

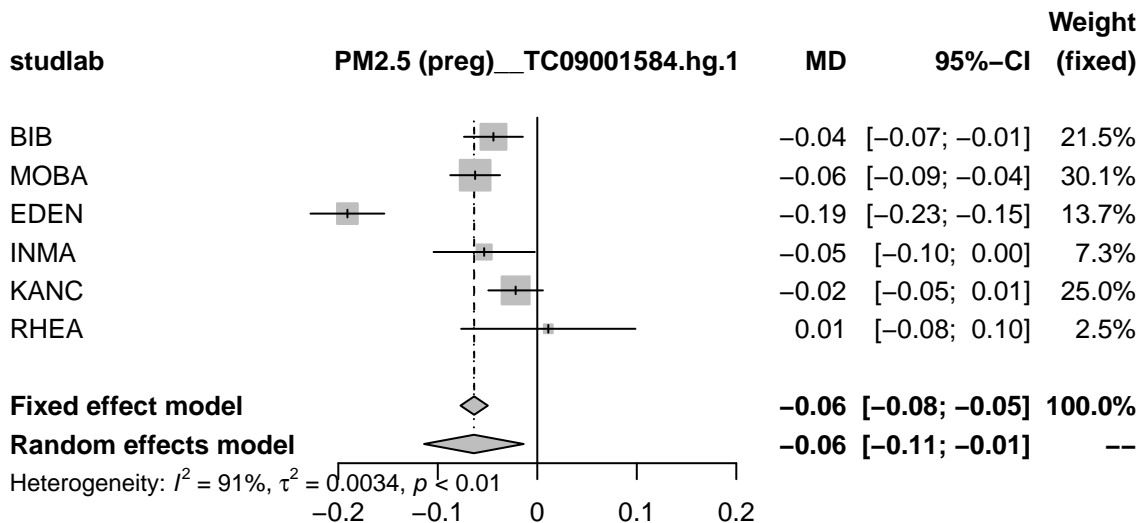

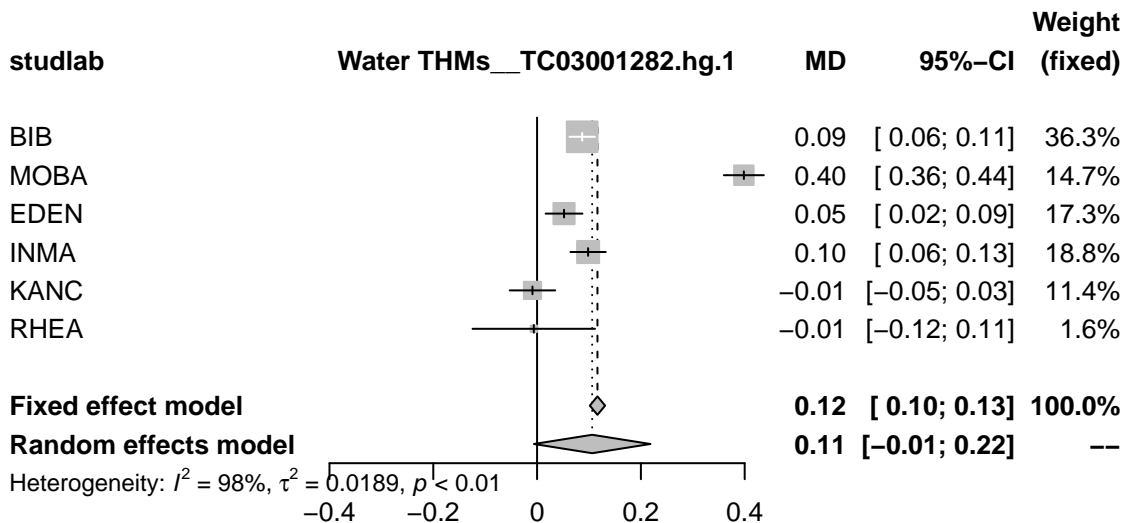

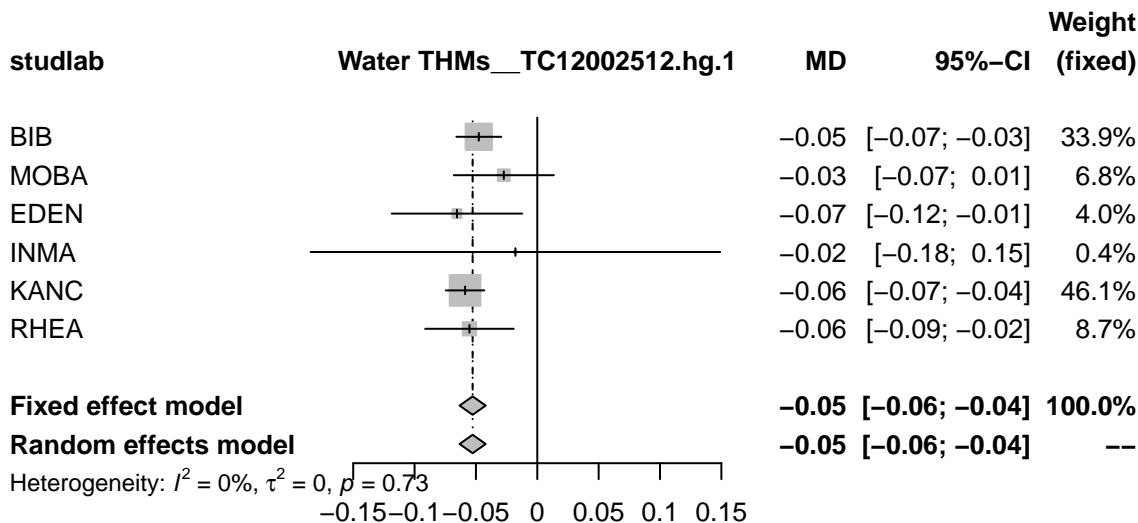

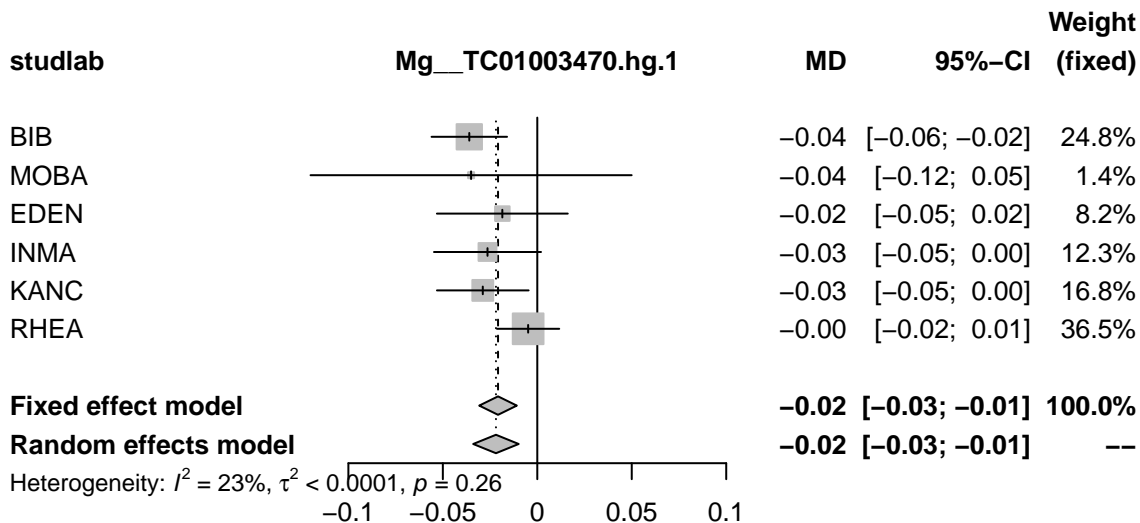

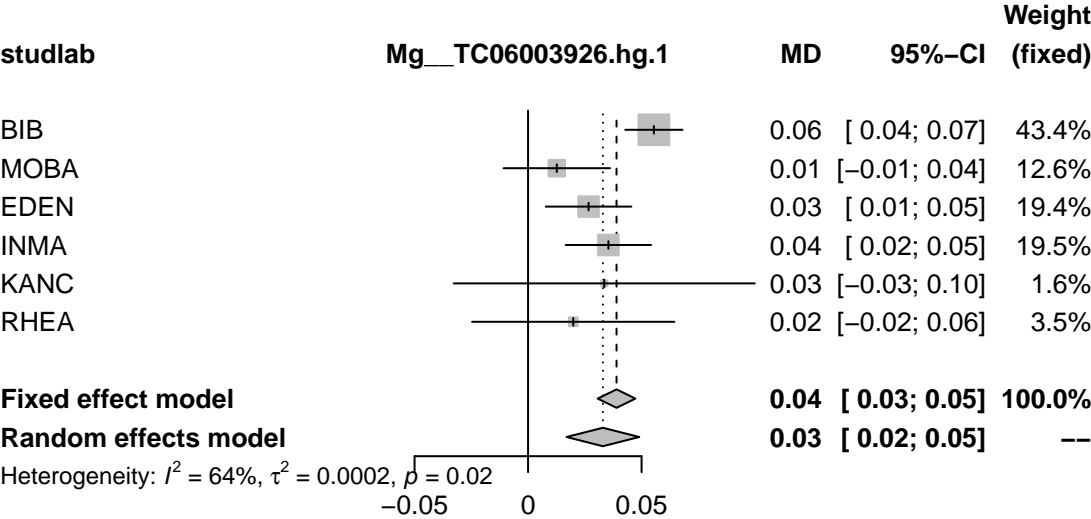

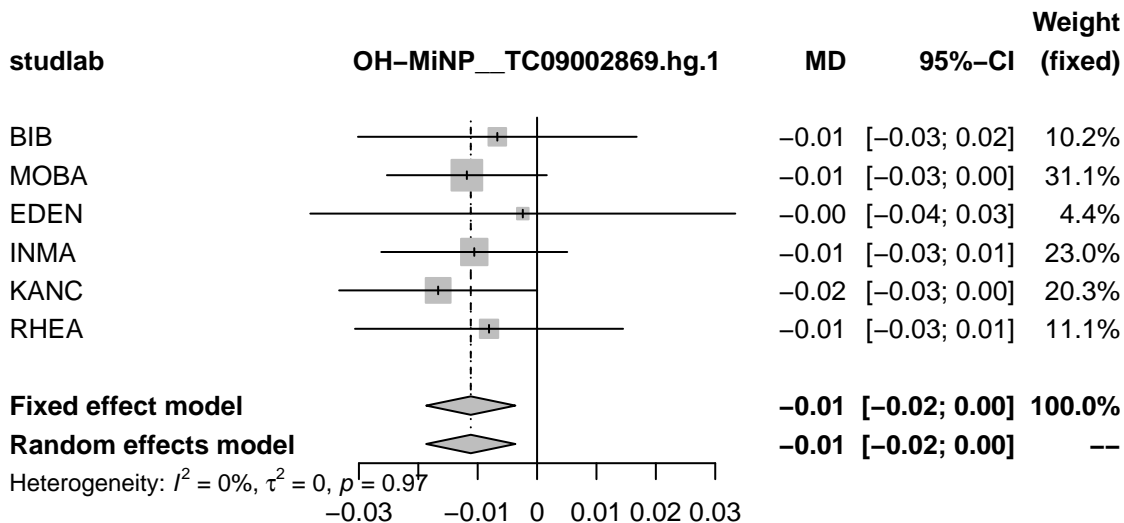

Supplement: Supplementary file 14 — Supplementary Dataset 11 [file 41467_2022_34422_MOESM14_ESM.zip › HELIX_ExpOmics_FigS2_Forestplots/HELIX_ExpOmics_FigS2B_trans_preg.pdf]

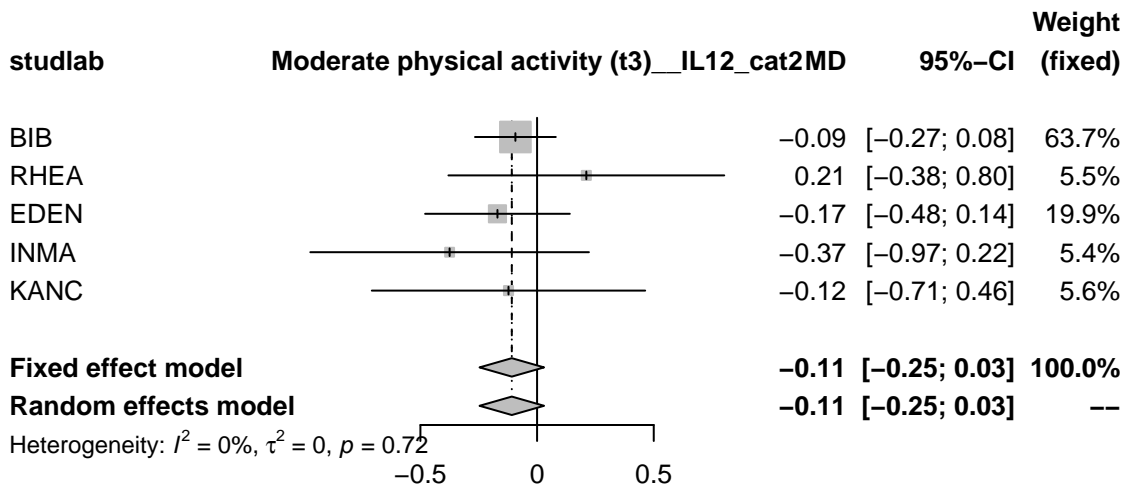

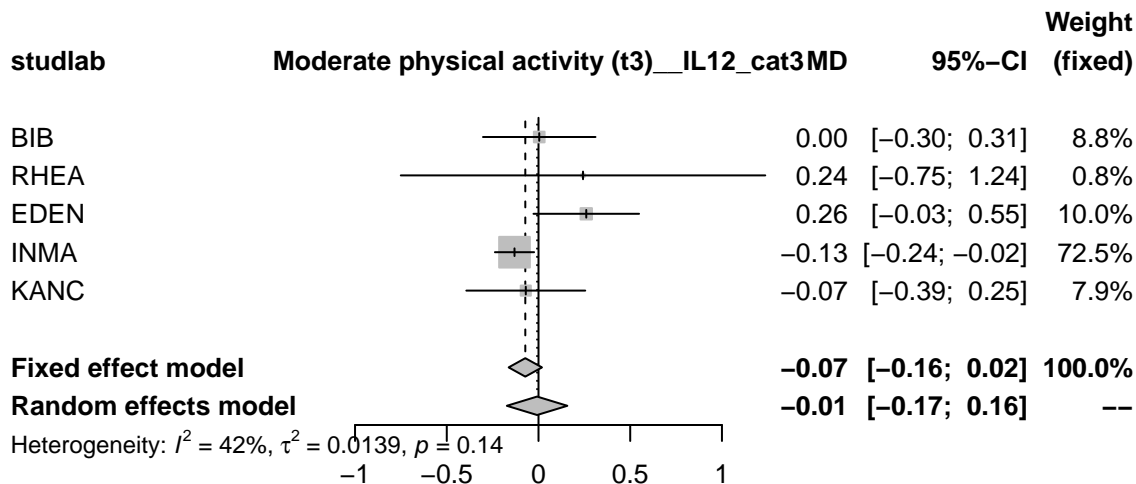

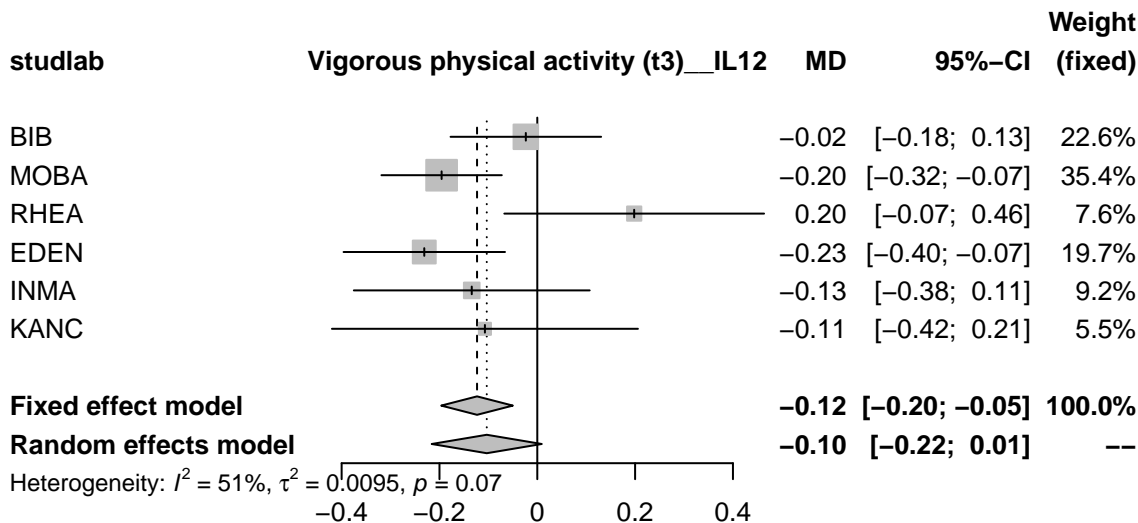

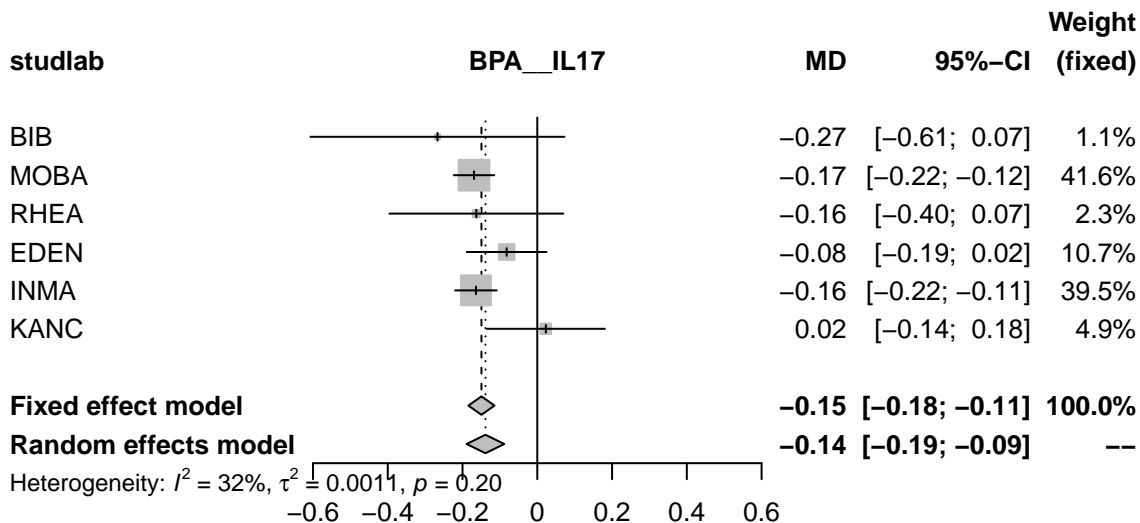

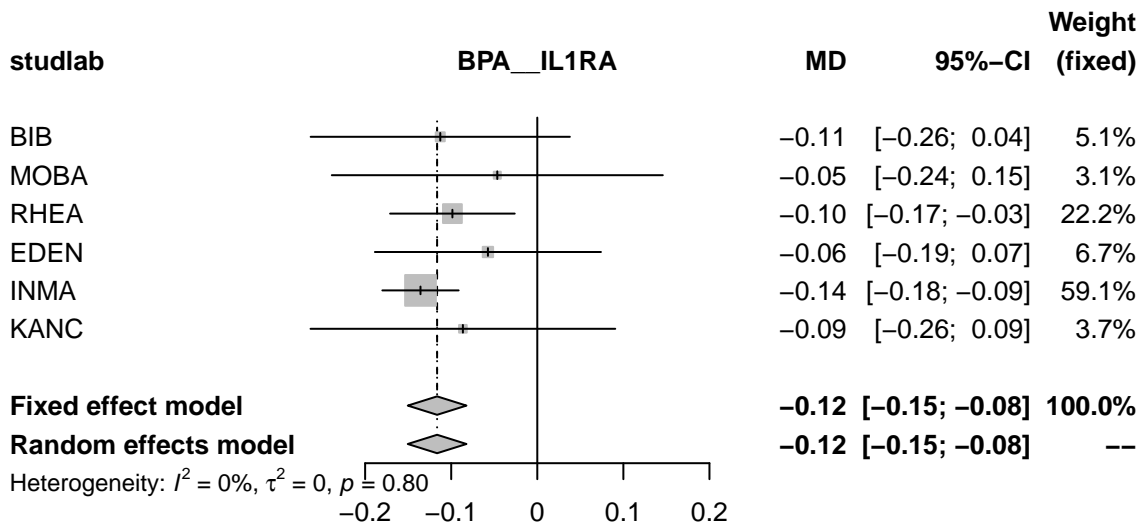

Supplement: Supplementary file 14 — Supplementary Dataset 11 [file 41467_2022_34422_MOESM14_ESM.zip › HELIX_ExpOmics_FigS2_Forestplots/HELIX_ExpOmics_FigS2D_prot_preg.pdf]

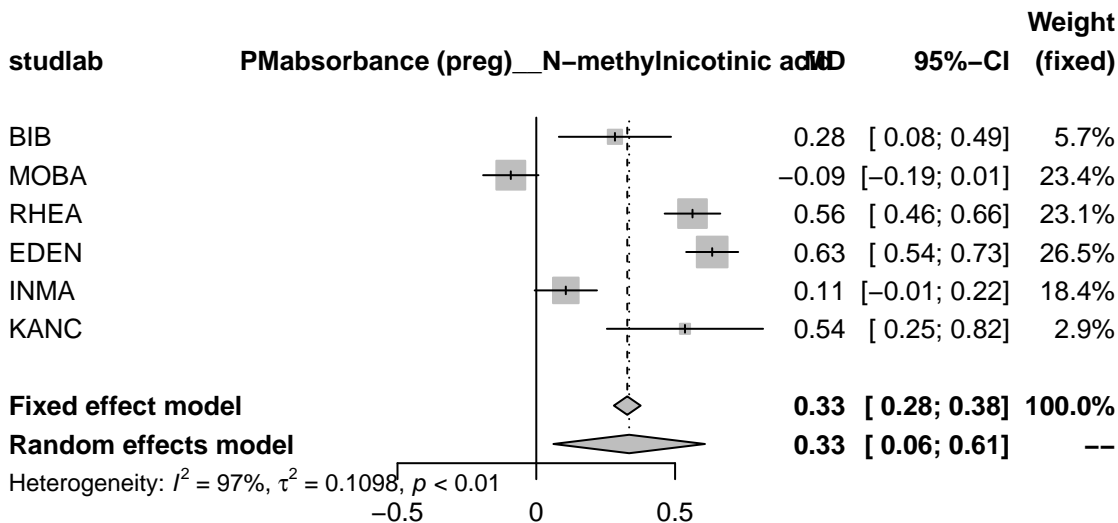

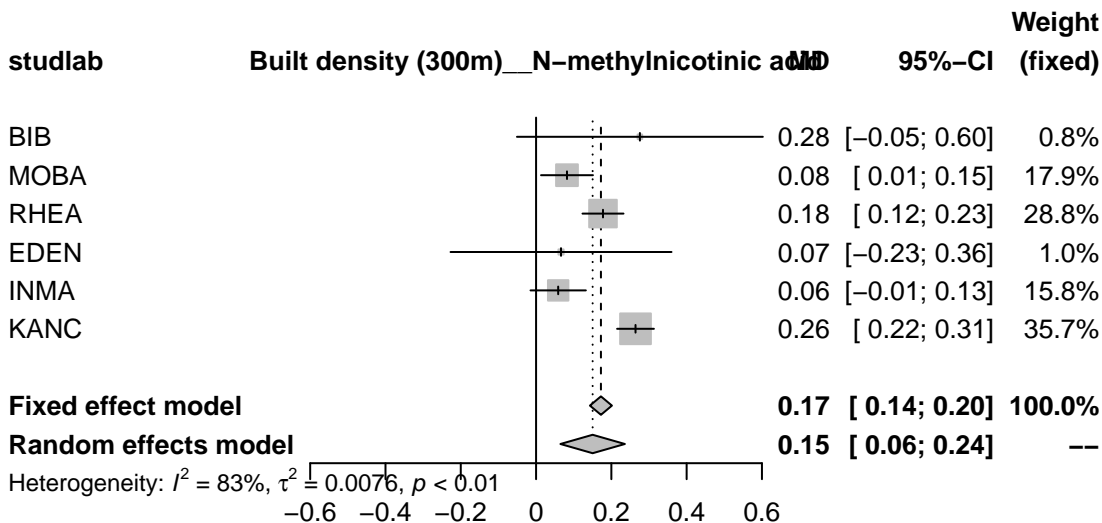

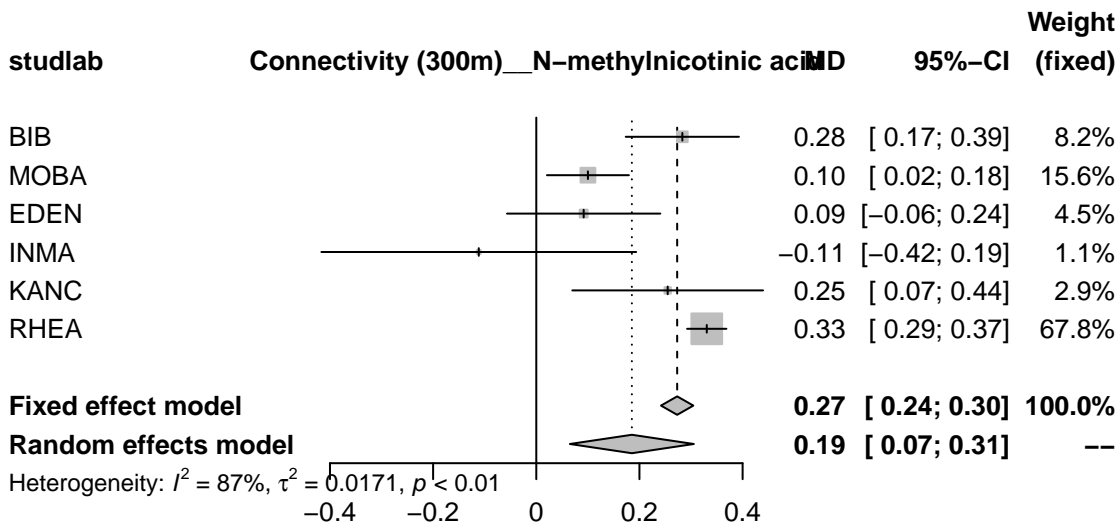

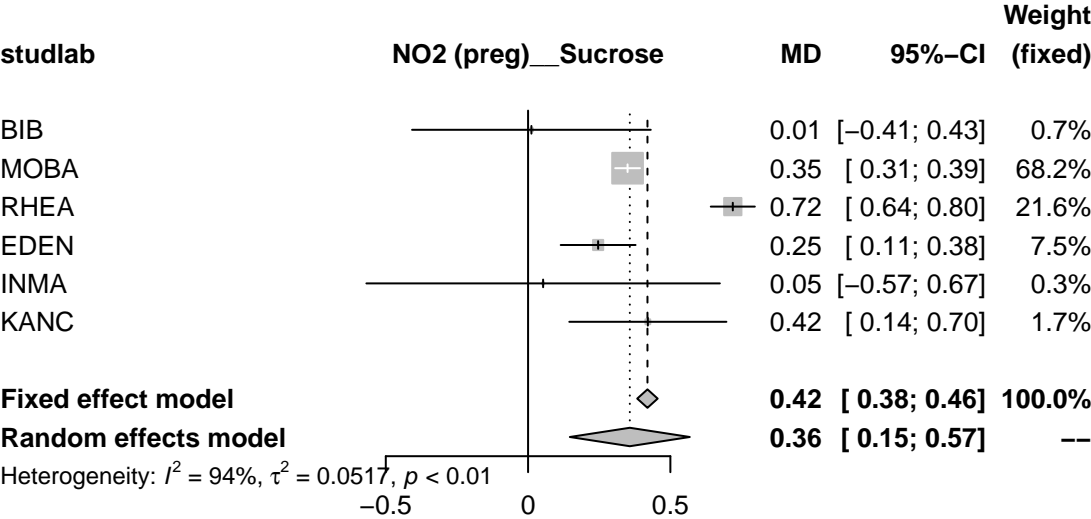

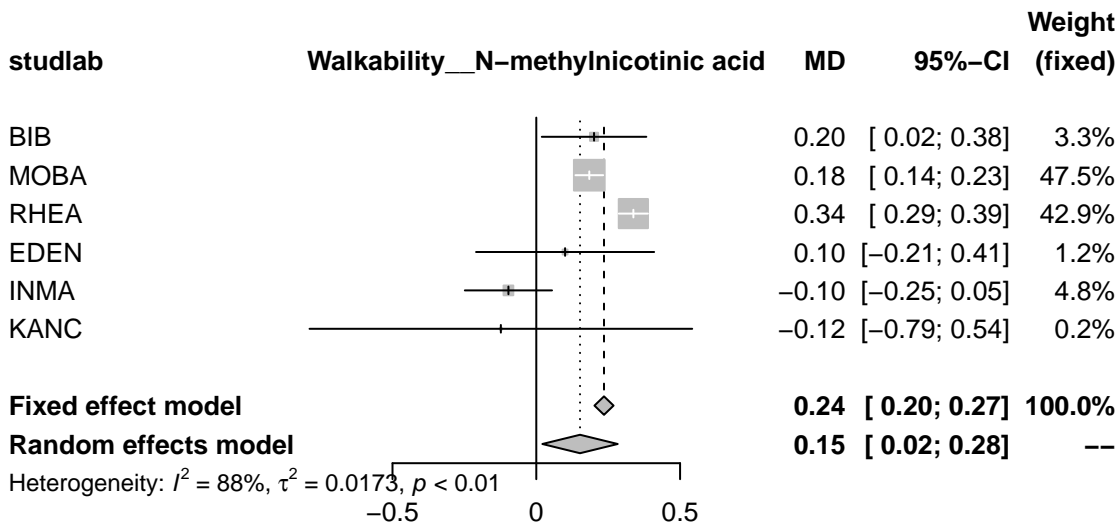

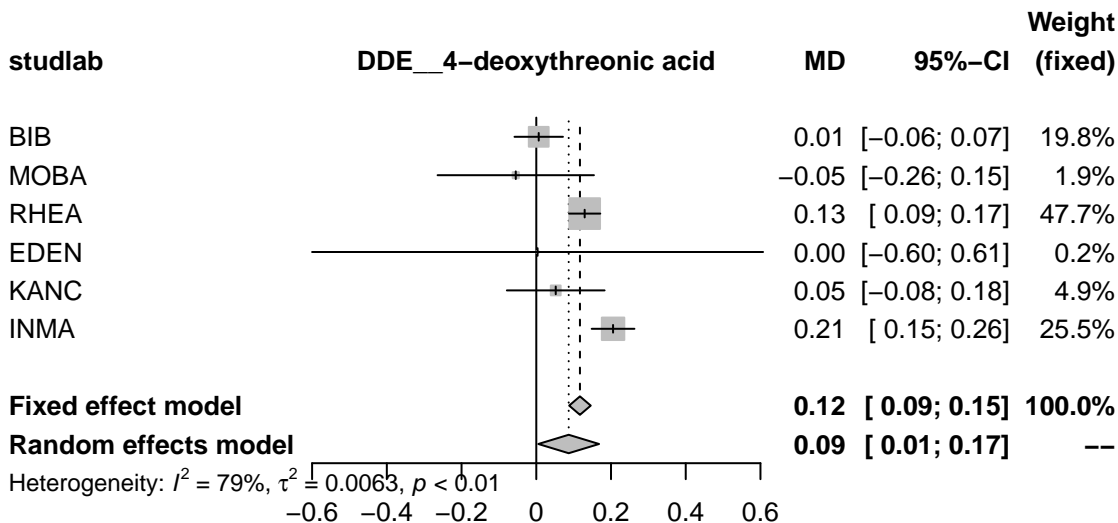

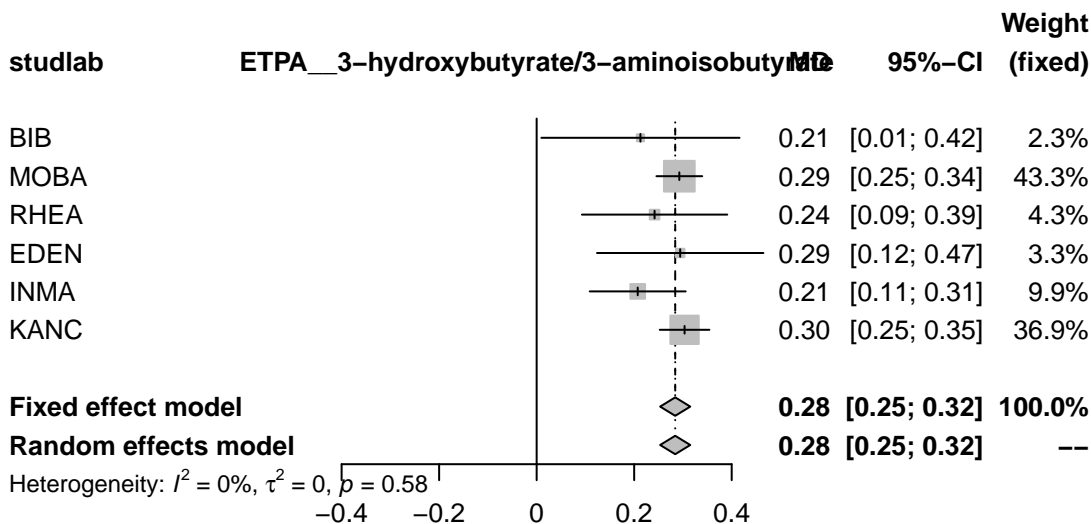

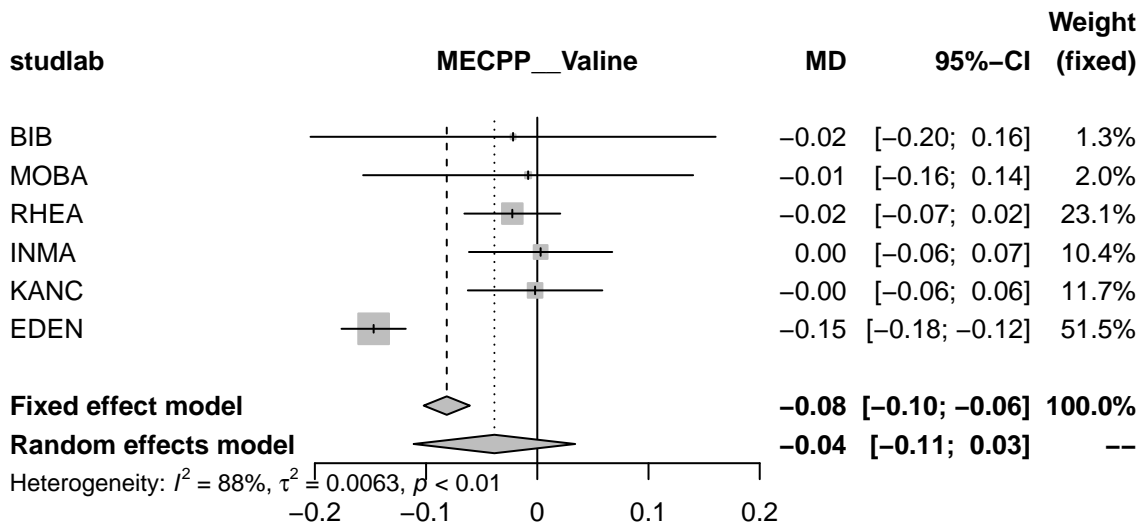

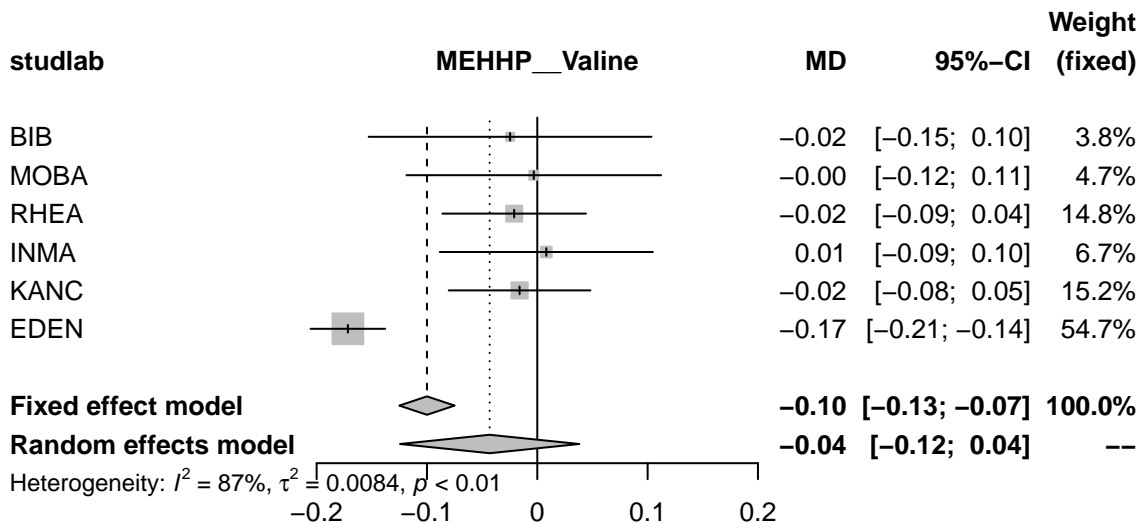

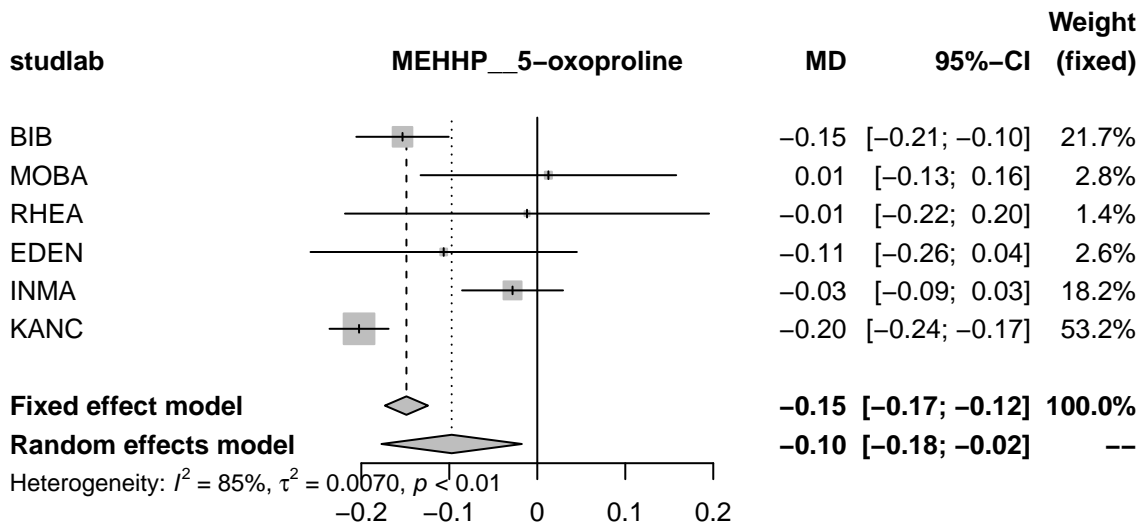

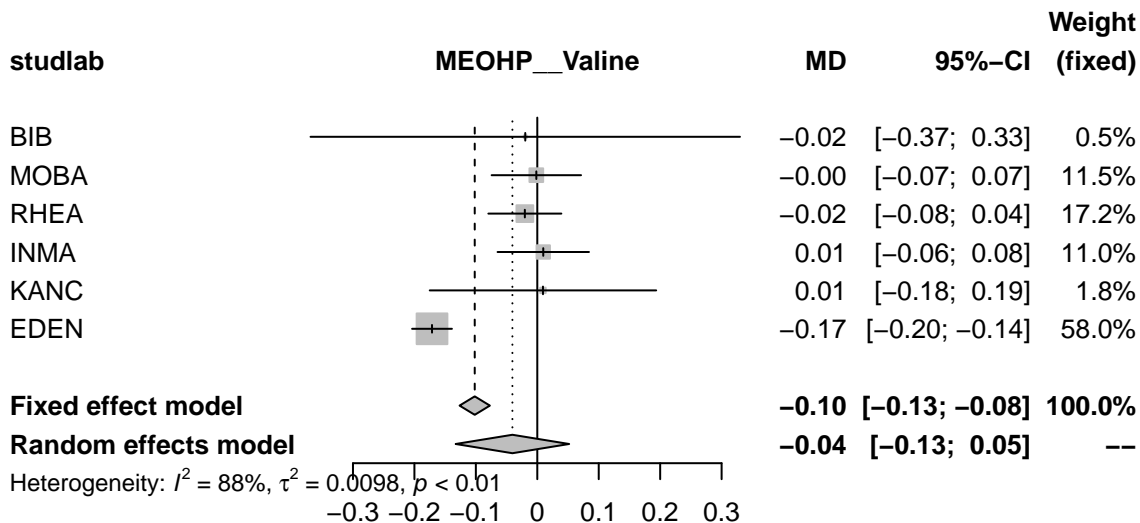

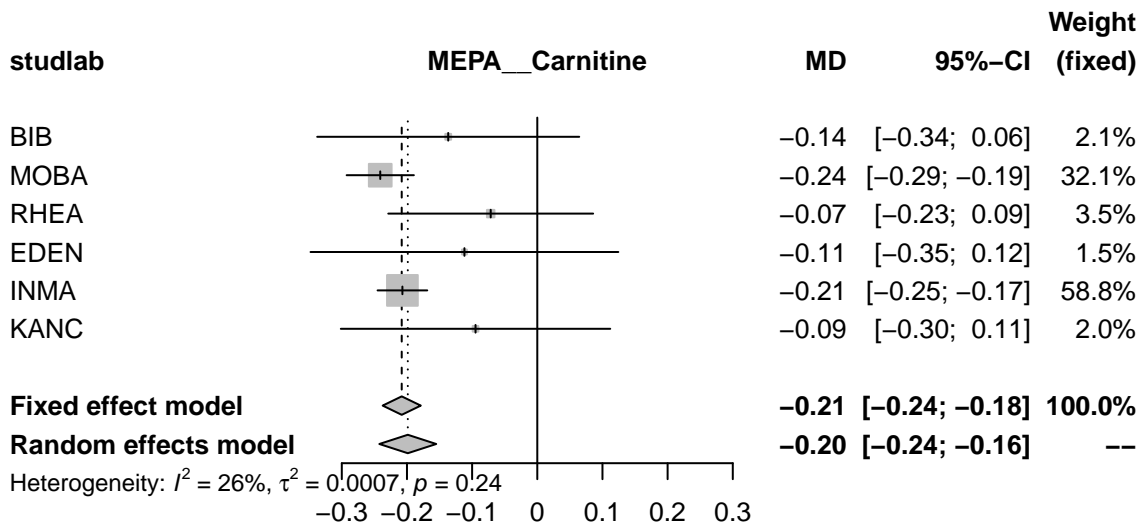

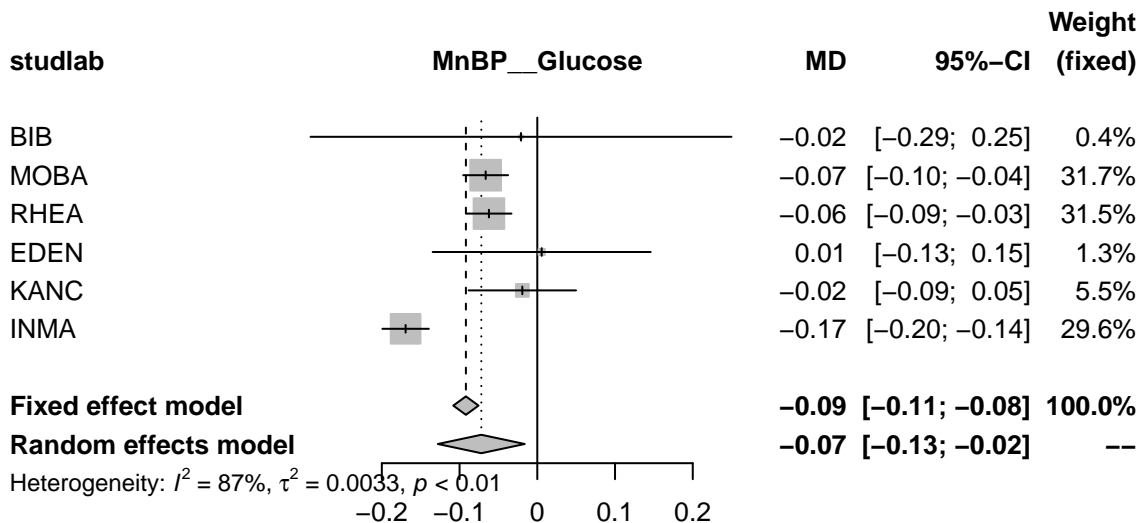

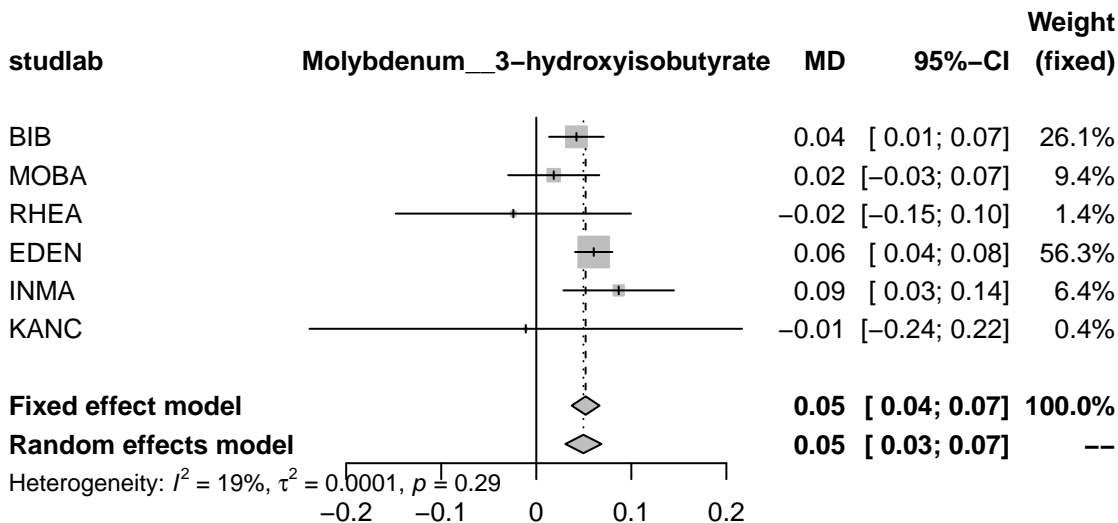

Supplement: Supplementary file 14 — Supplementary Dataset 11 [file 41467_2022_34422_MOESM14_ESM.zip › HELIX_ExpOmics_FigS2_Forestplots/HELIX_ExpOmics_FigS2F_met_u_preg.pdf]

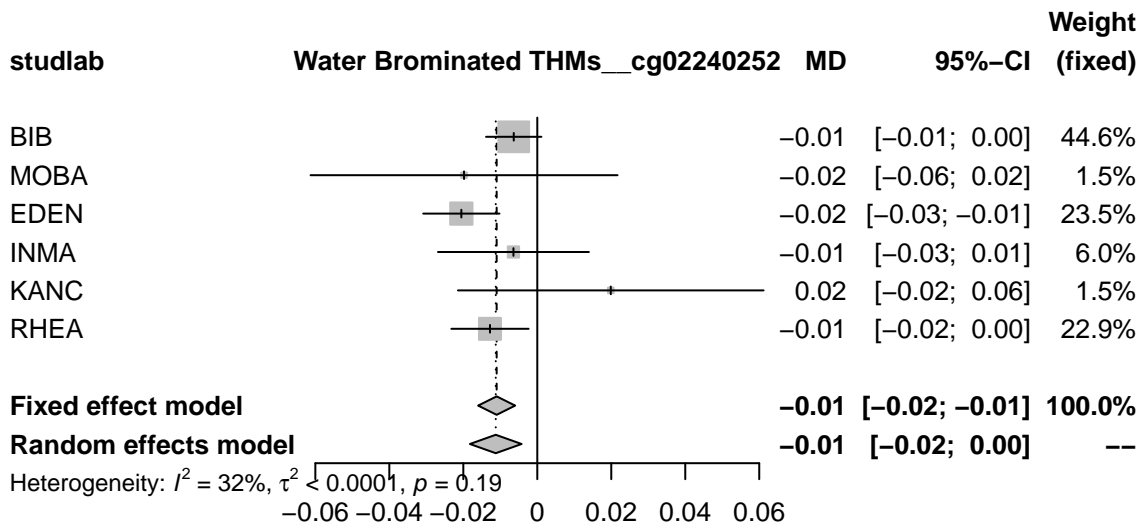

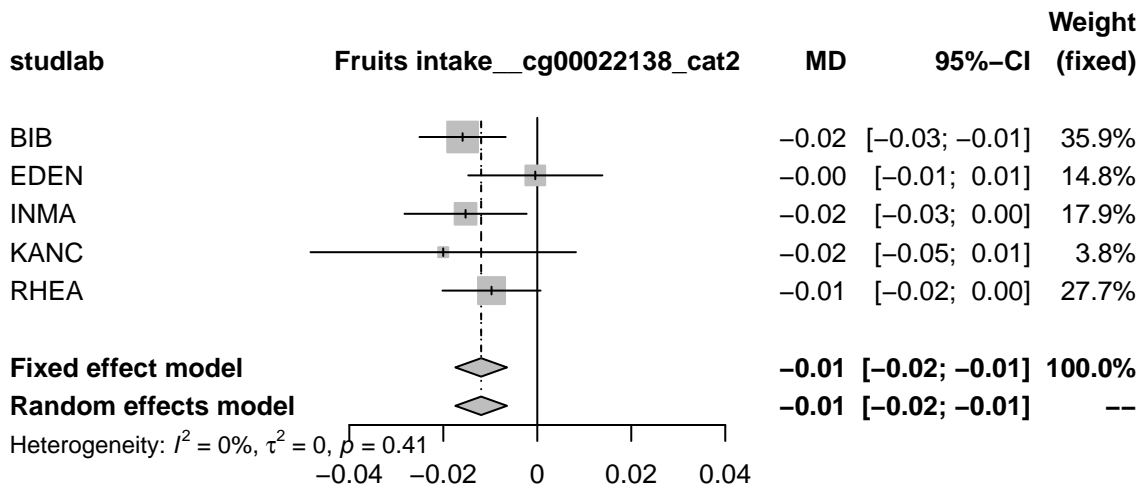

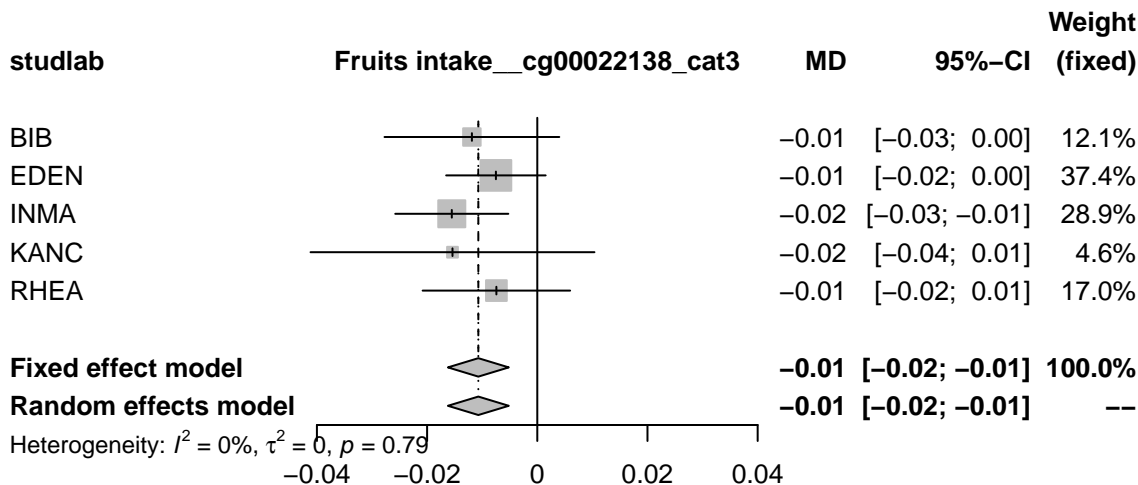

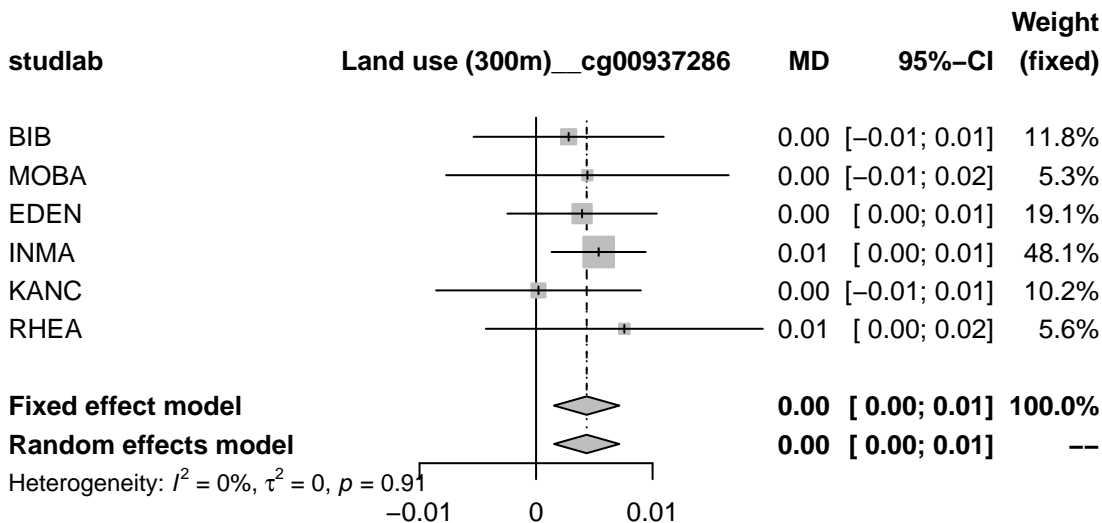

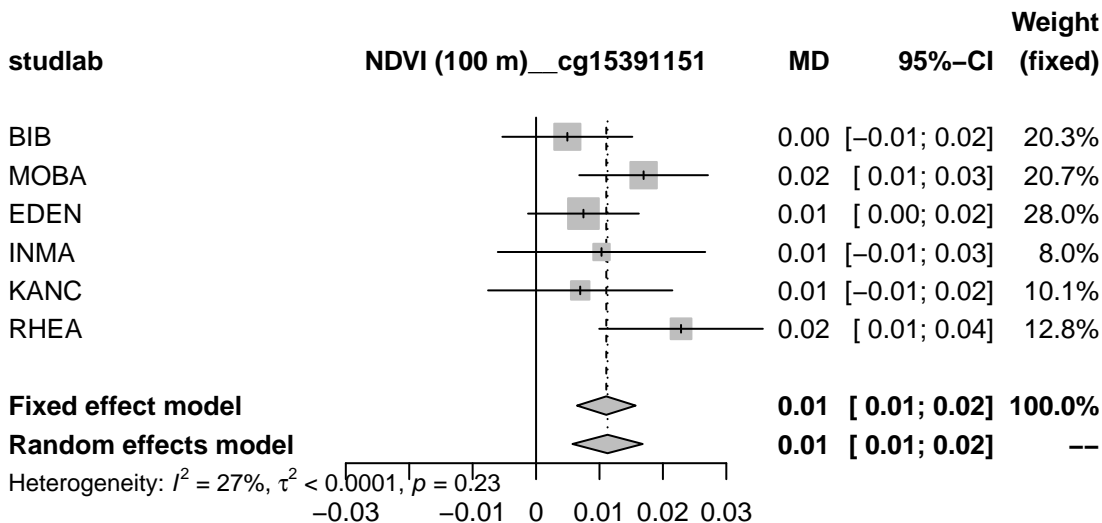

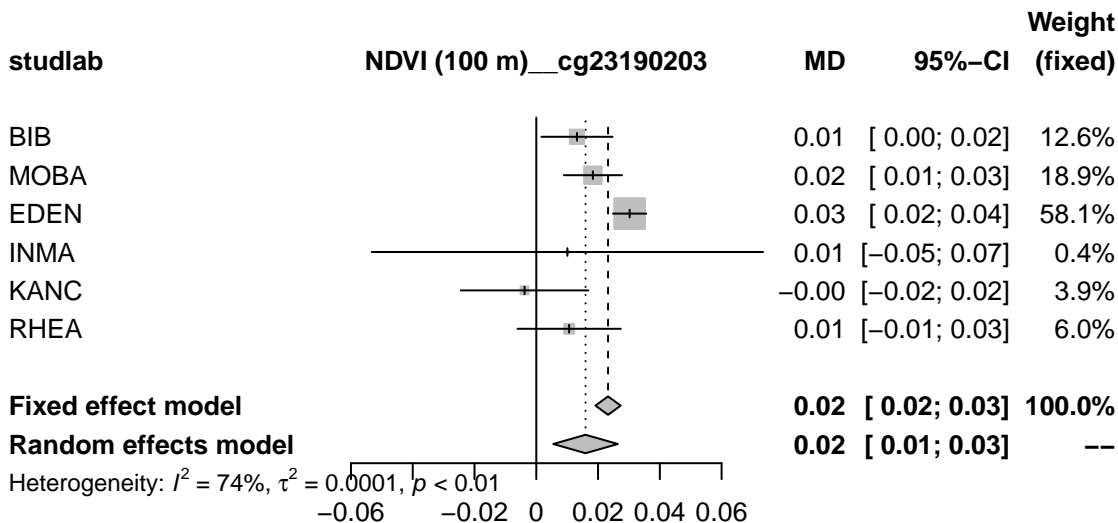

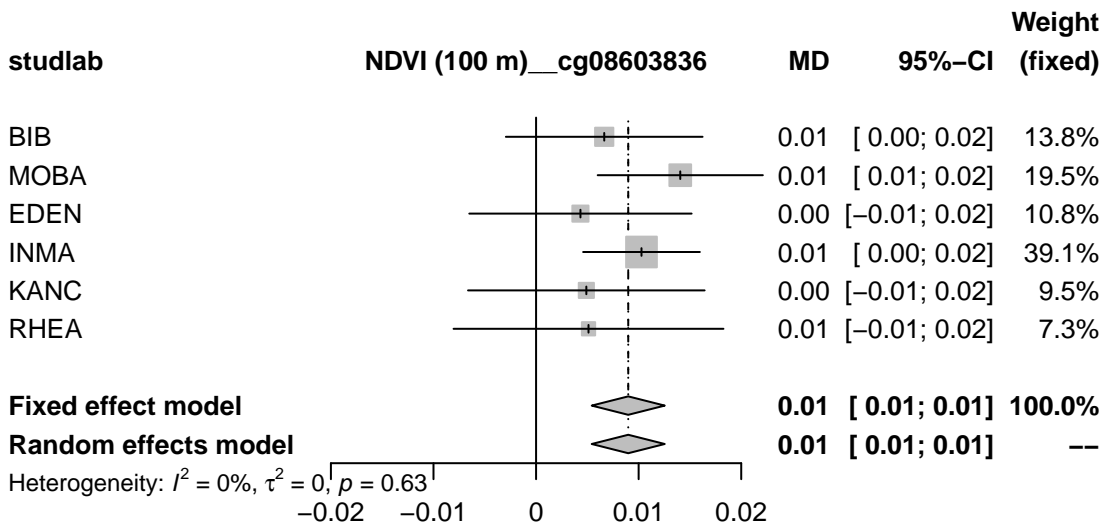

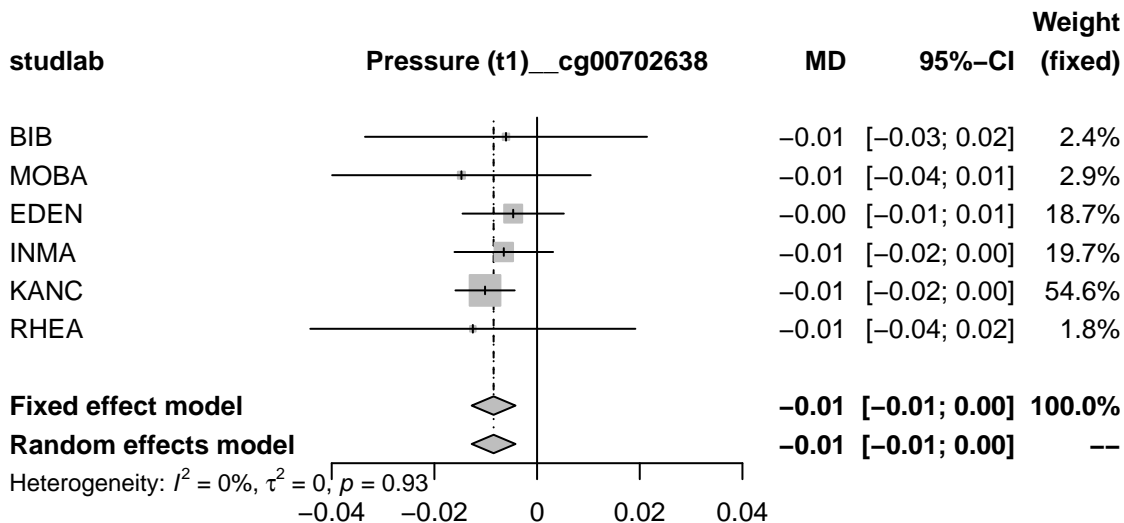

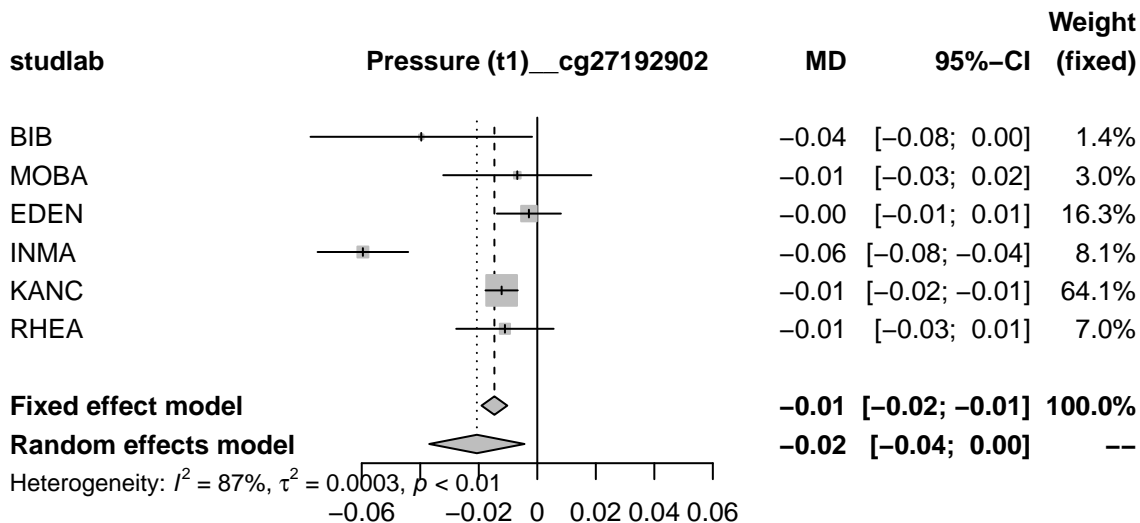

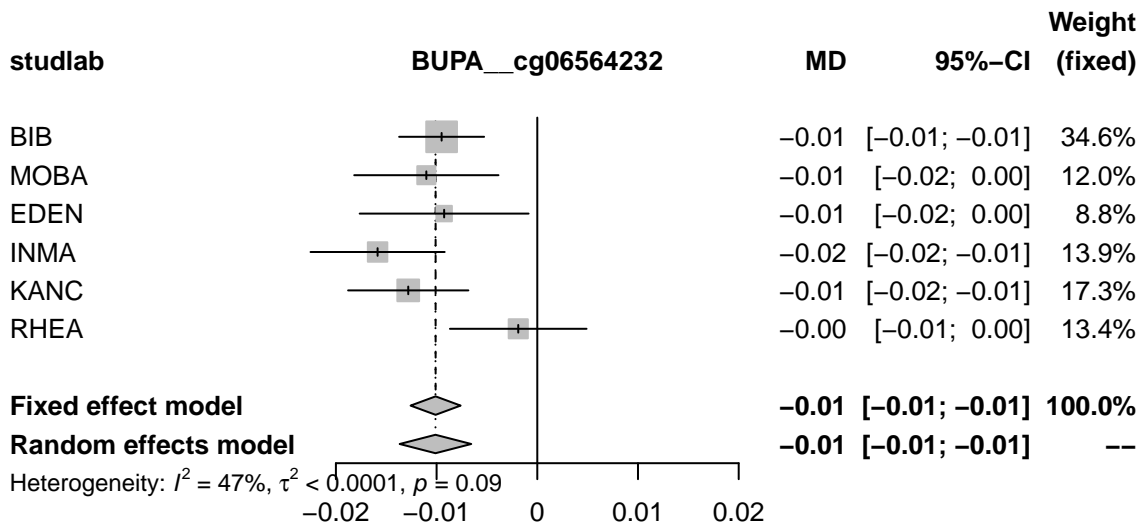

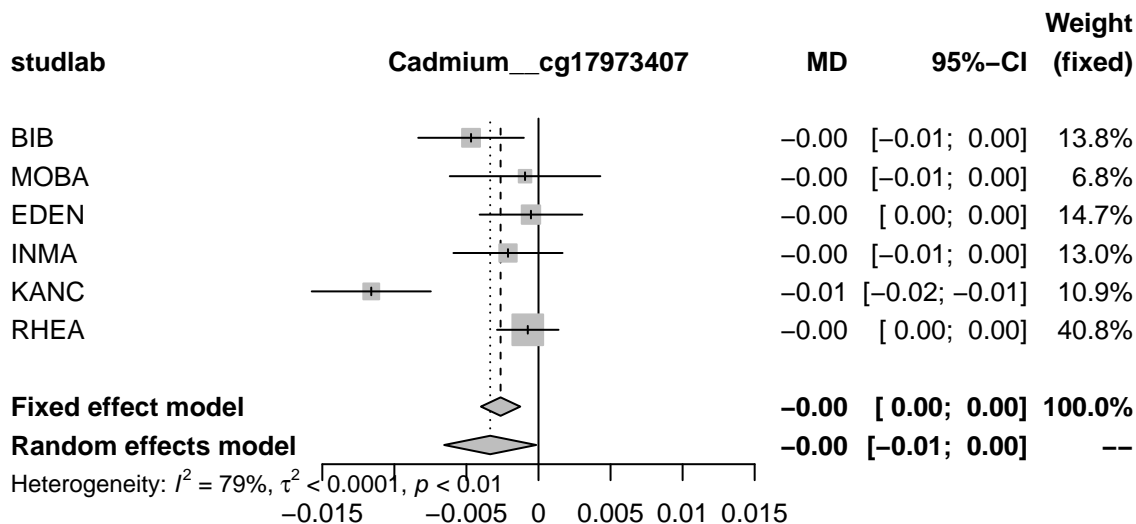

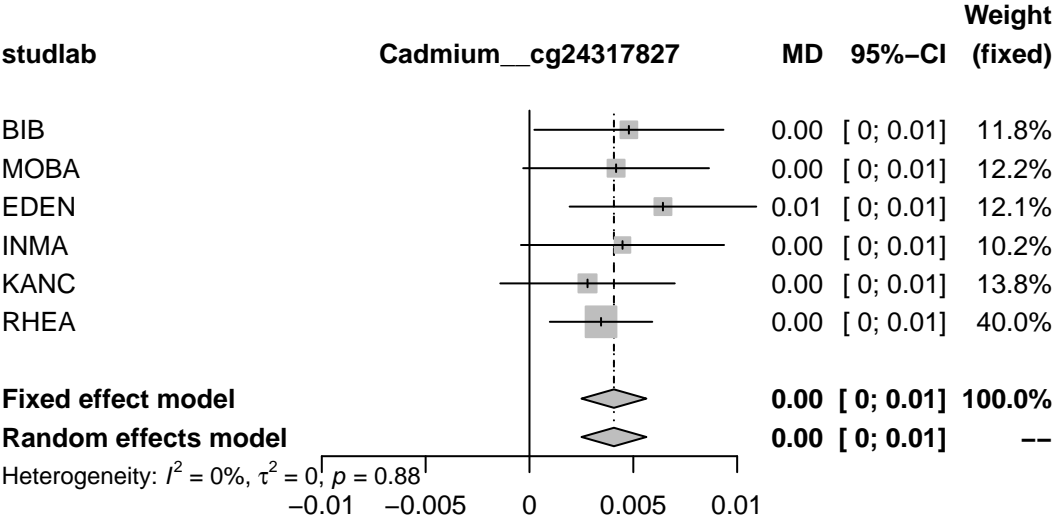

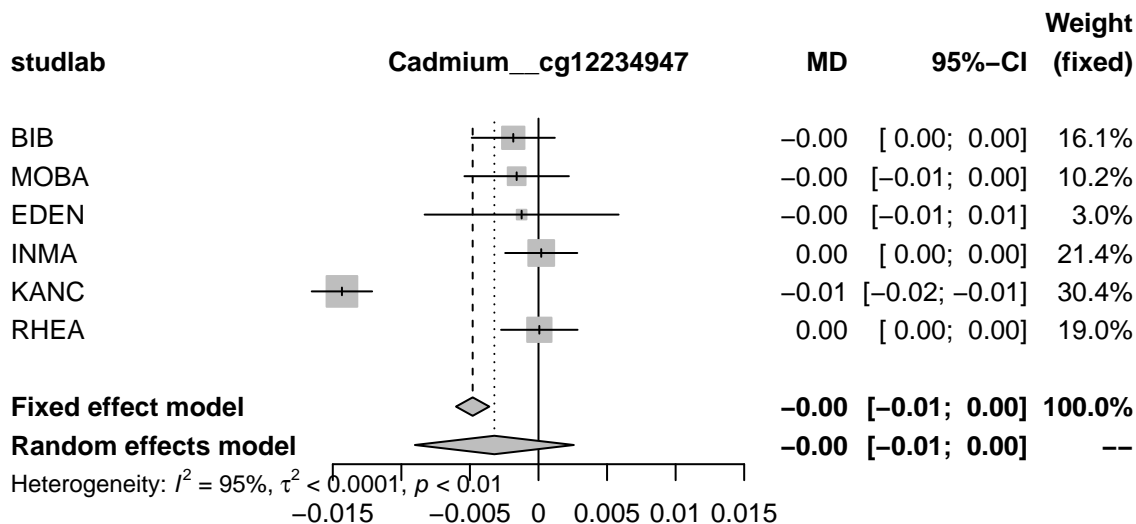

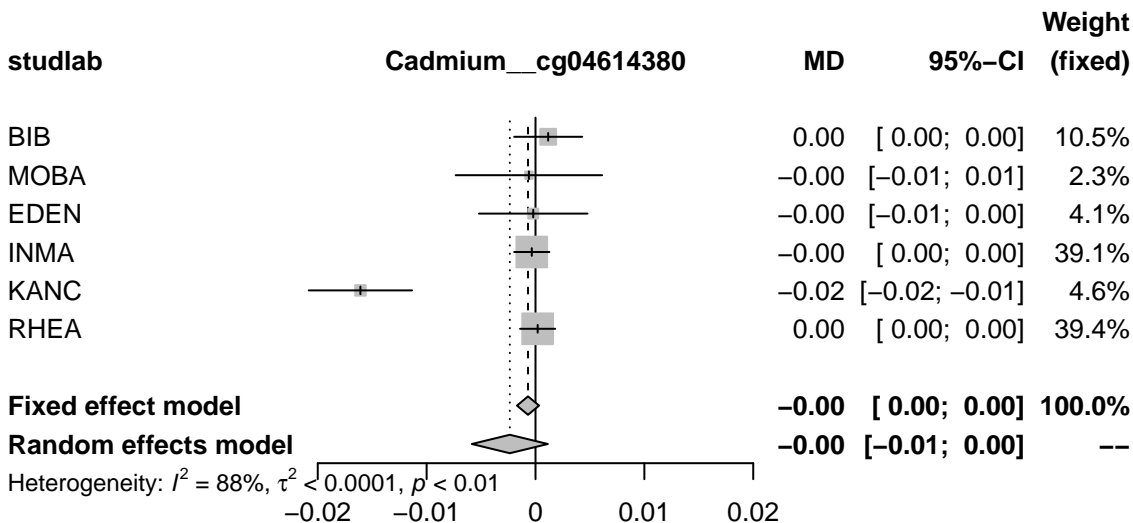

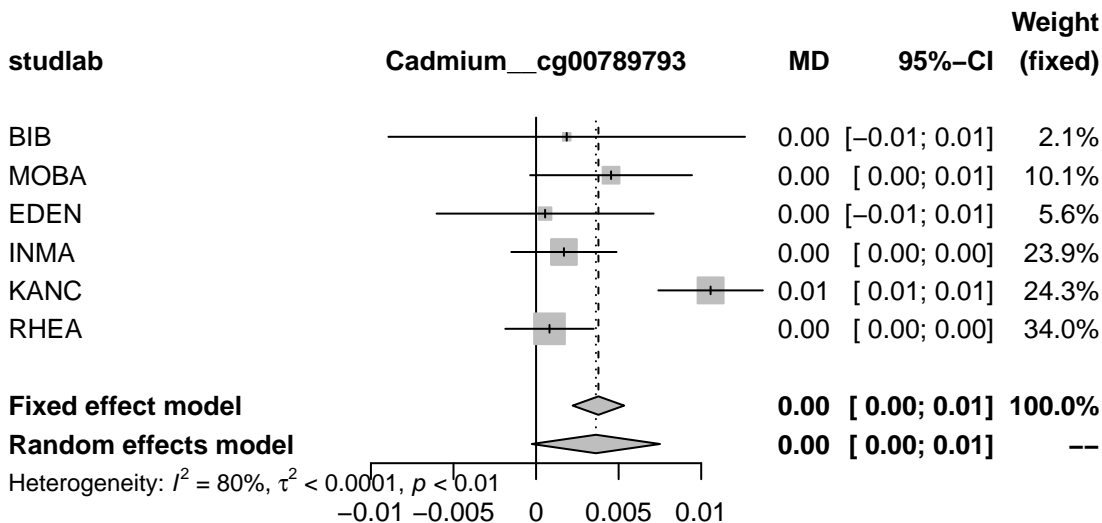

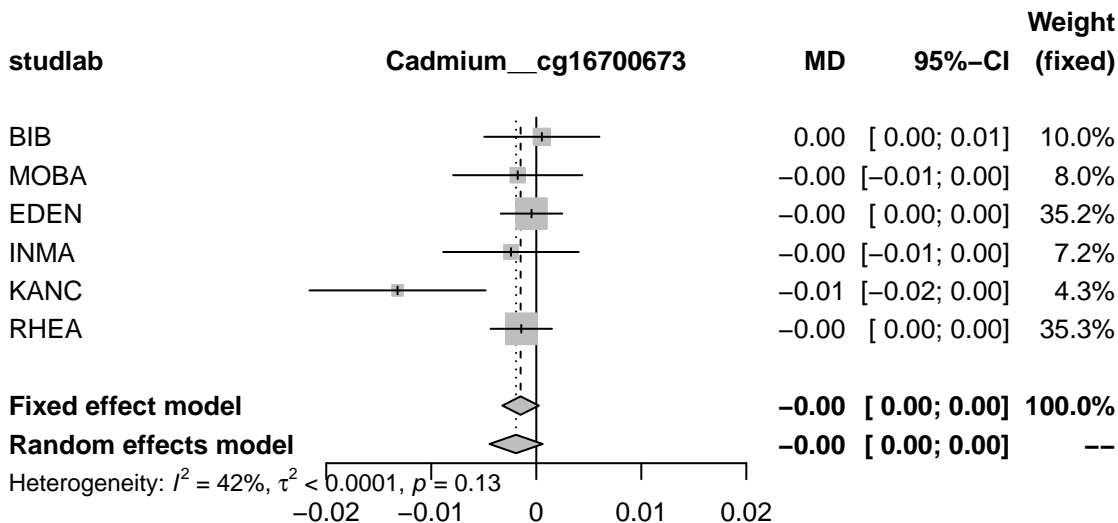

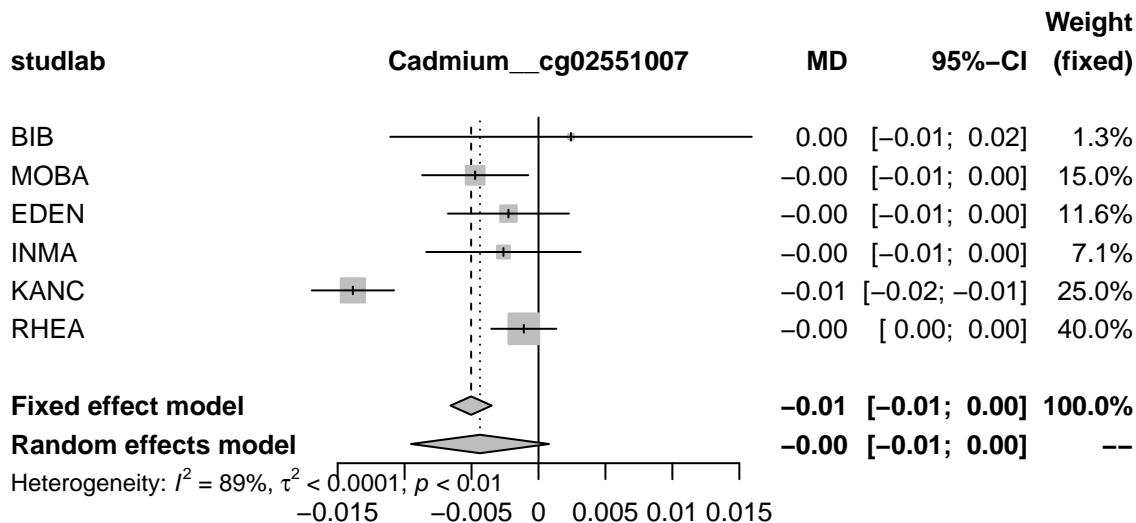

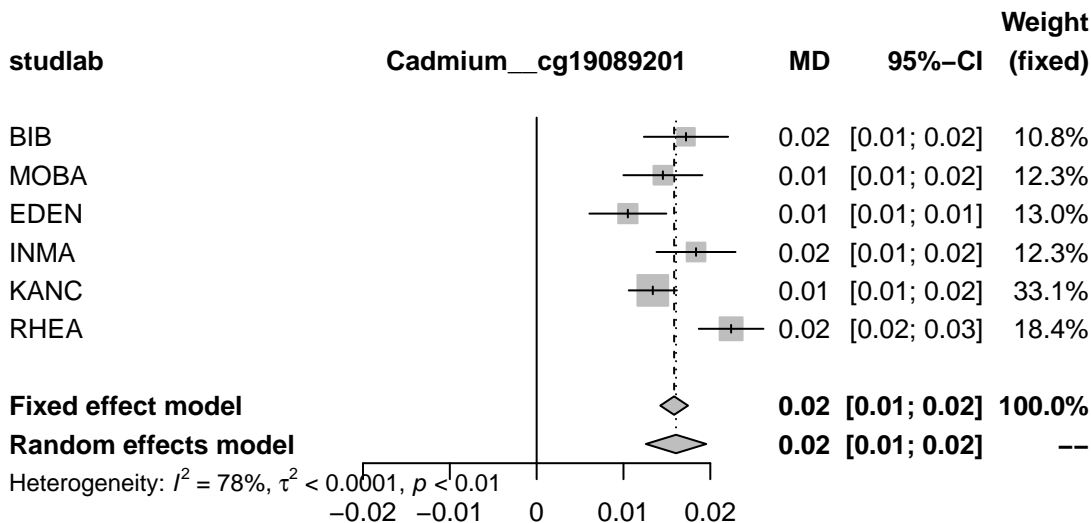

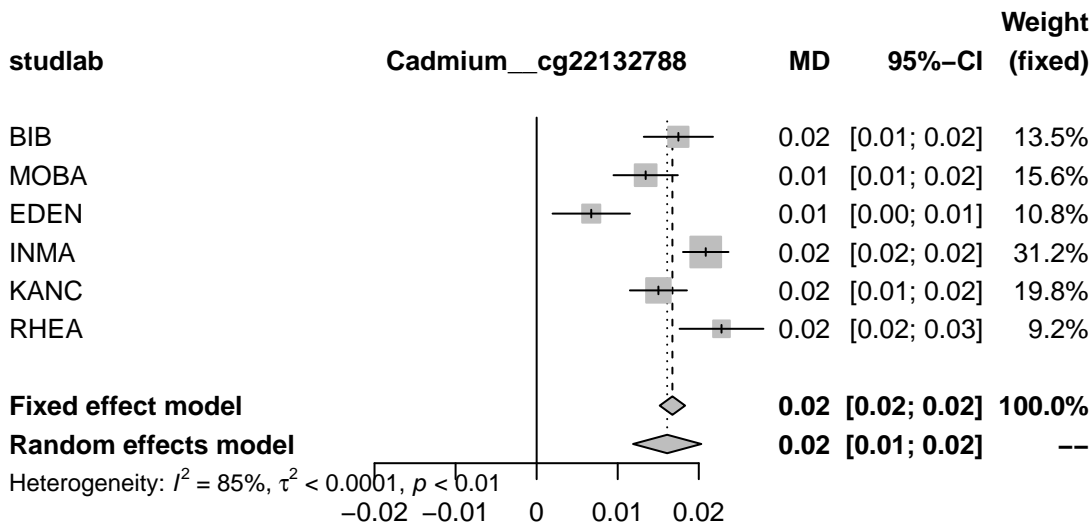

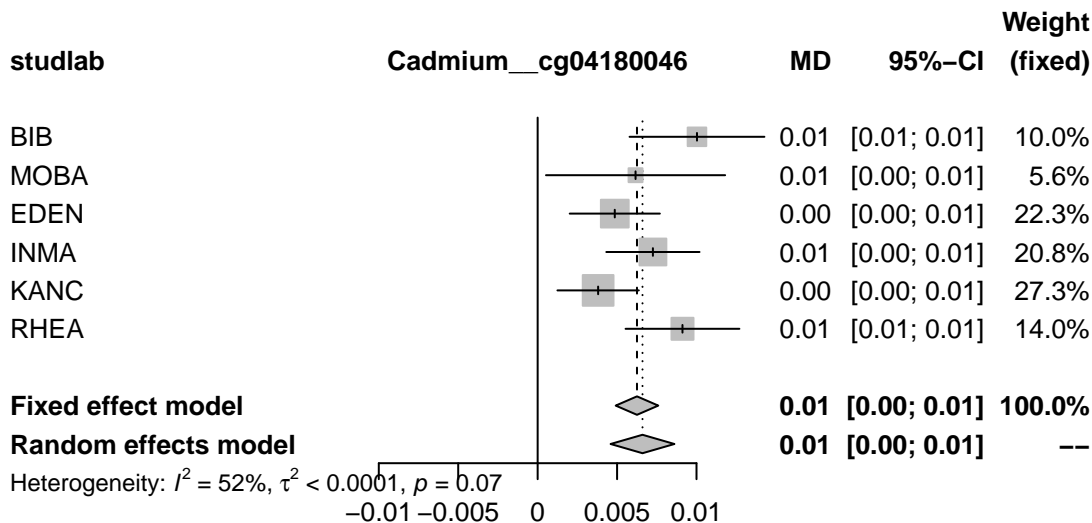

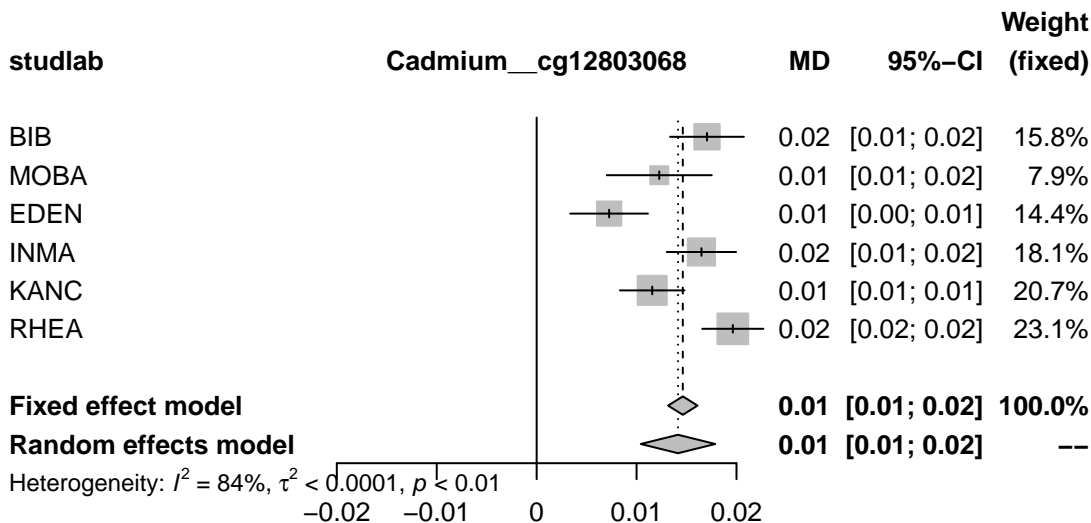

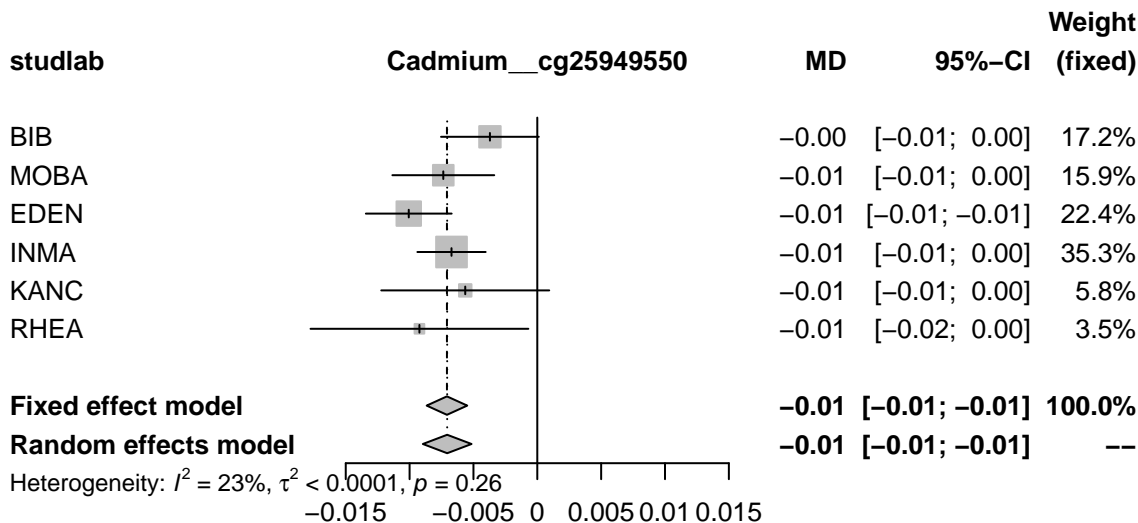

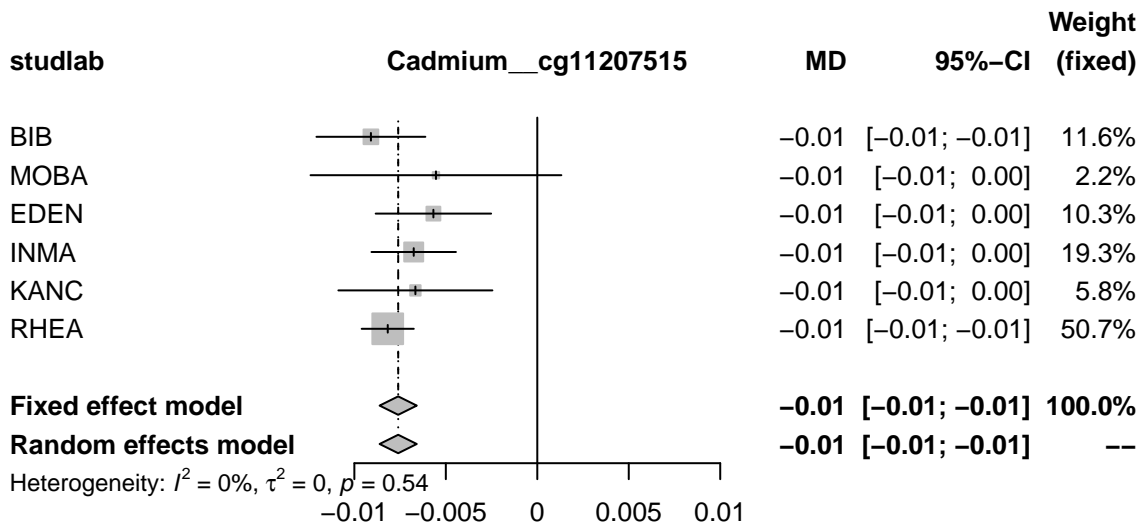

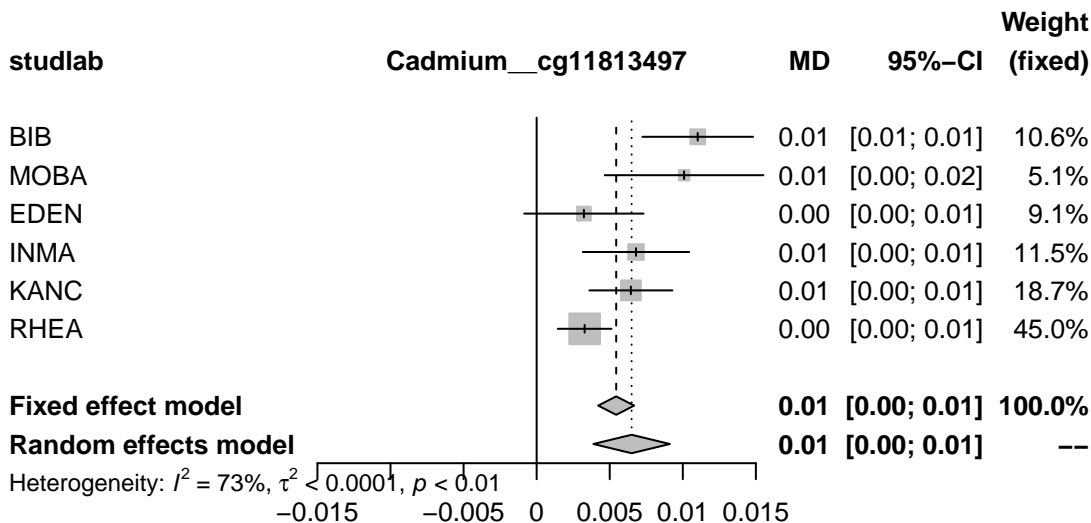

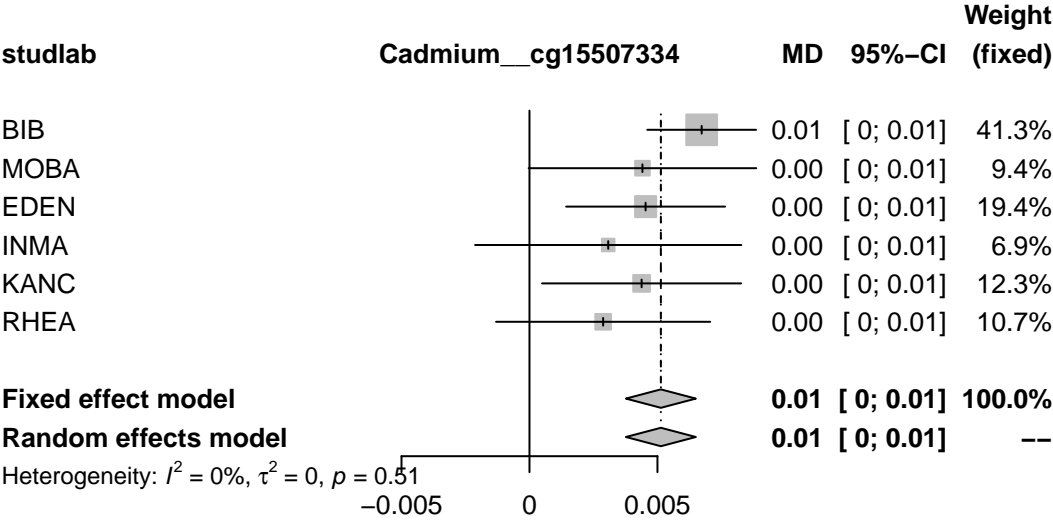

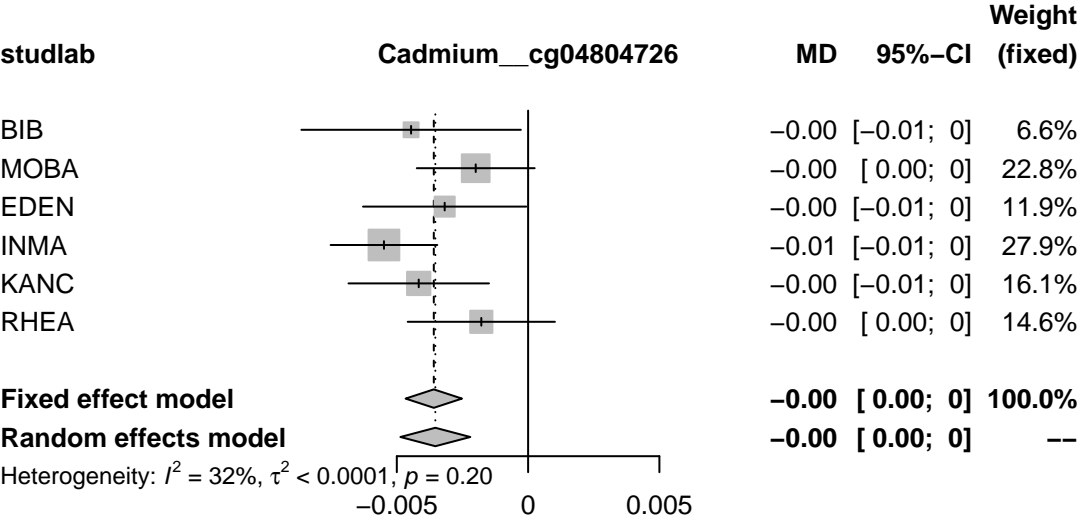

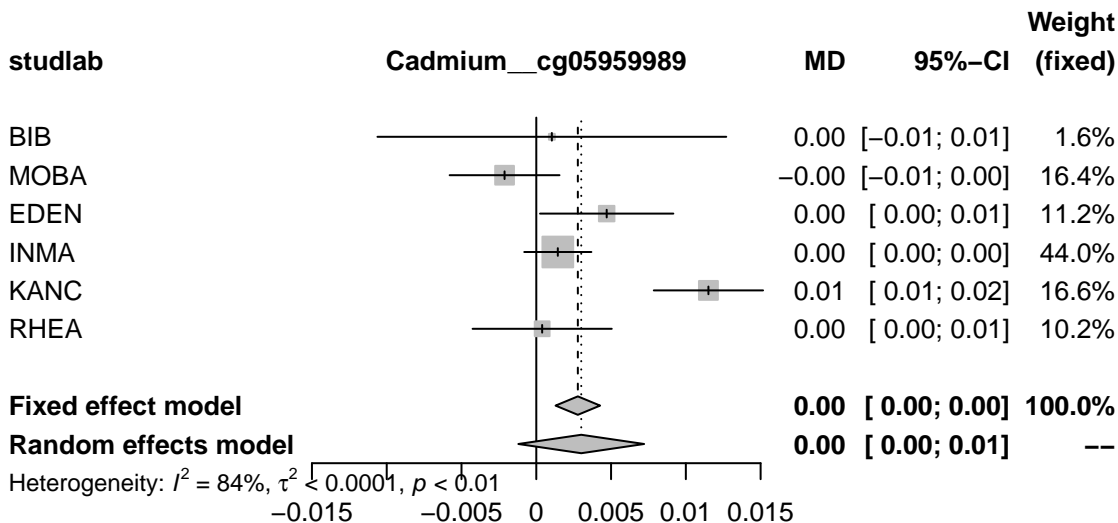

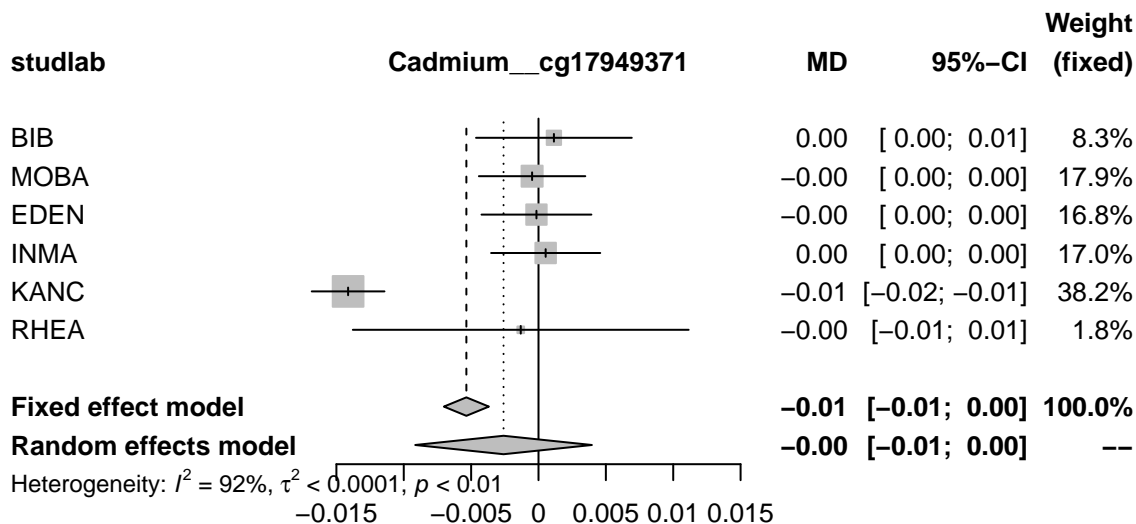

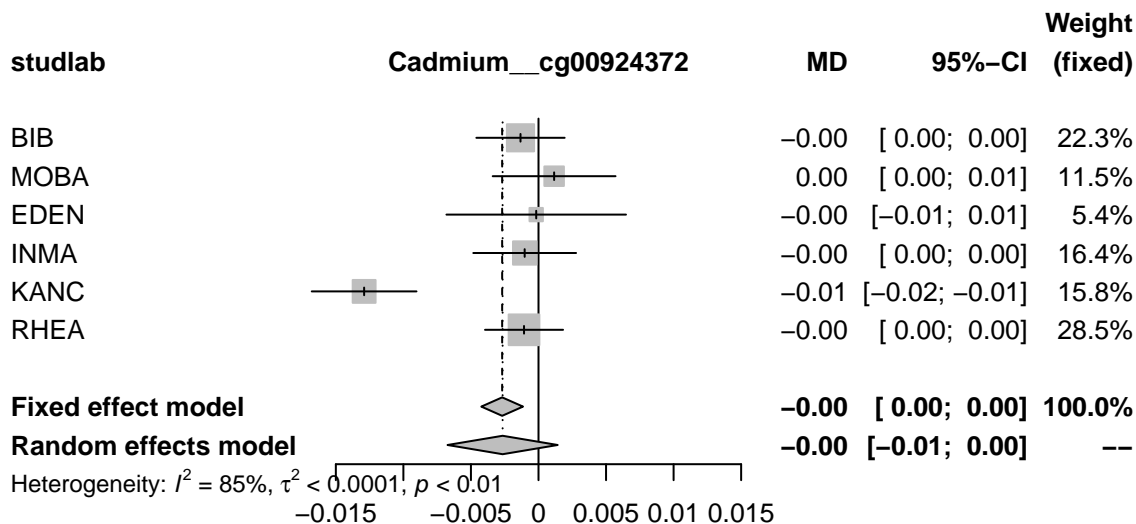

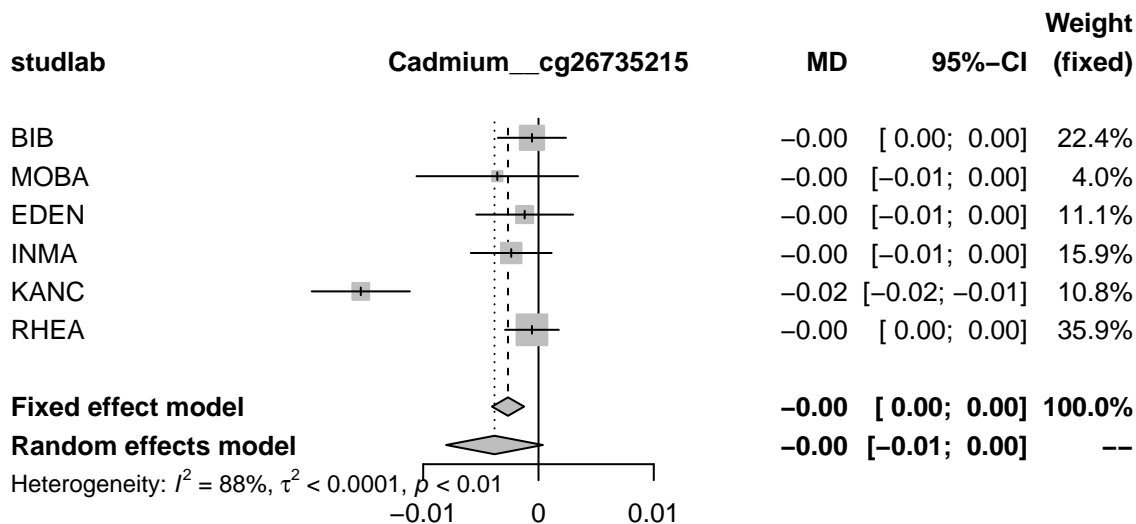

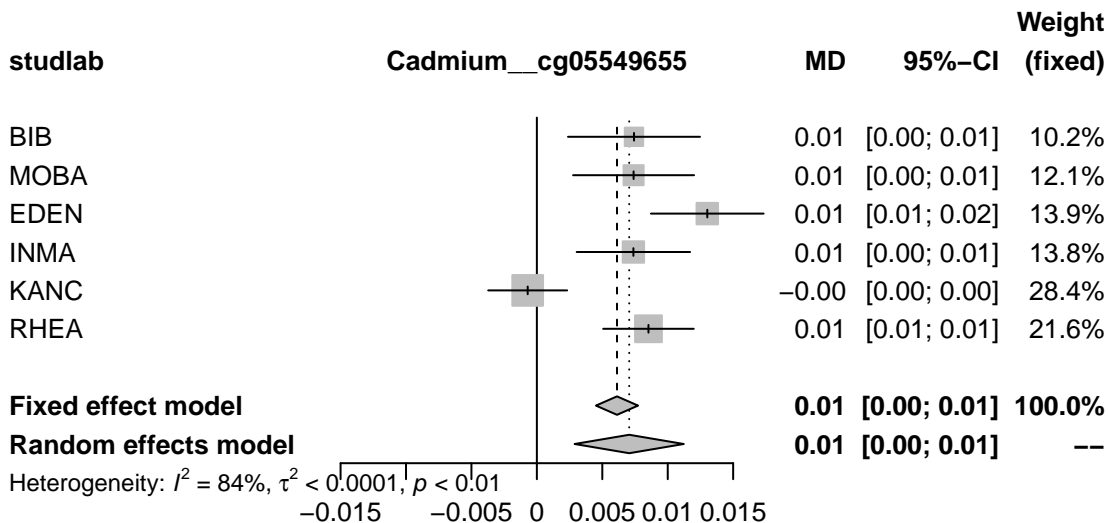

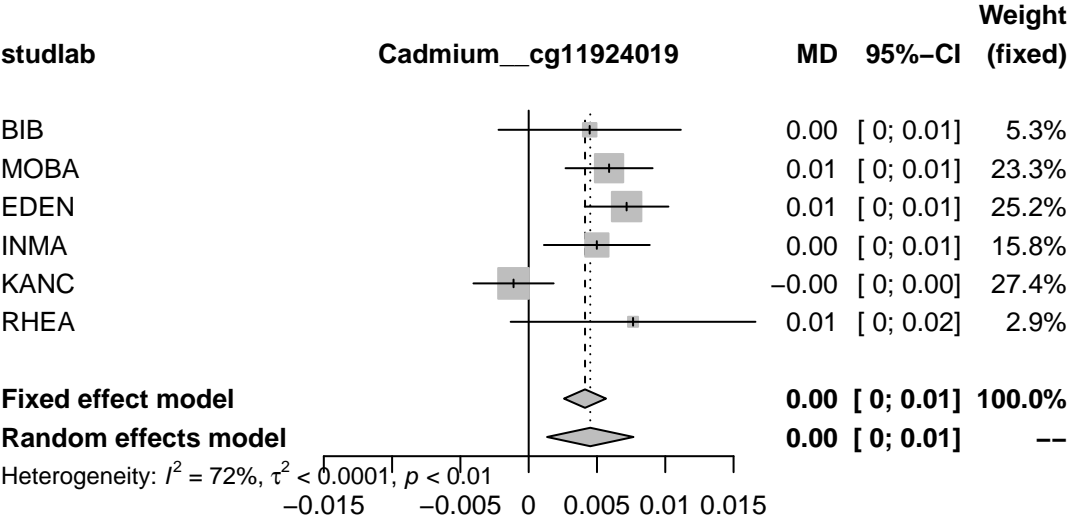

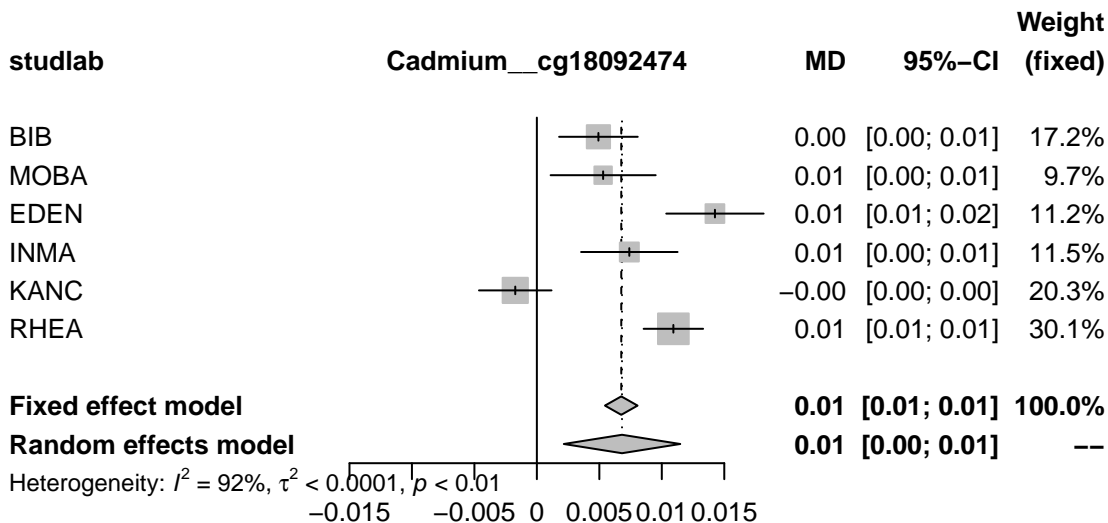

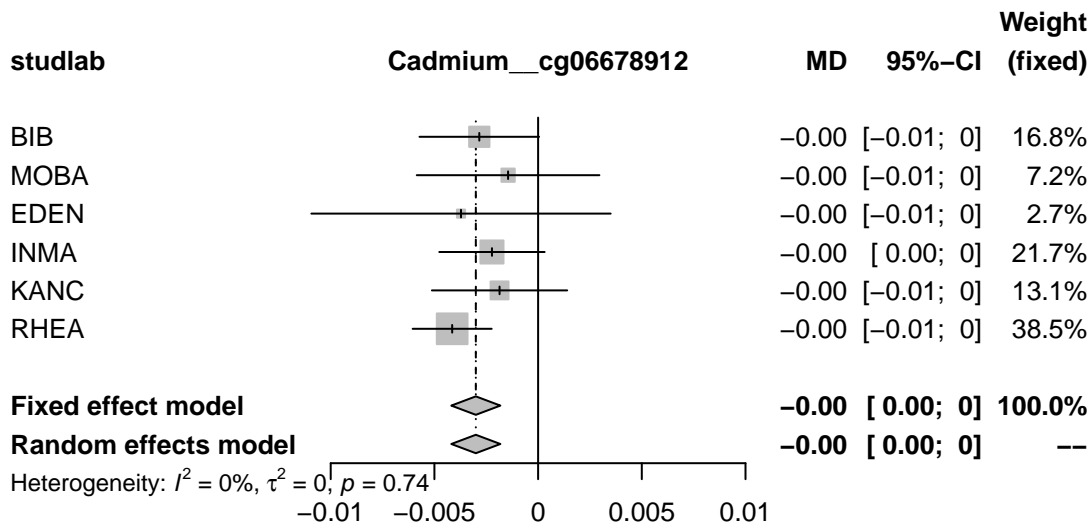

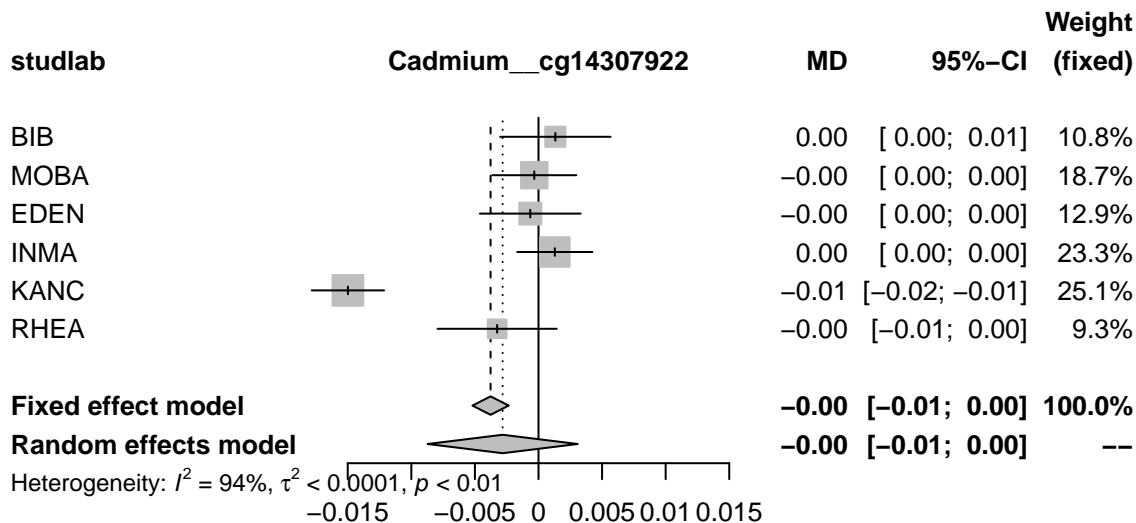

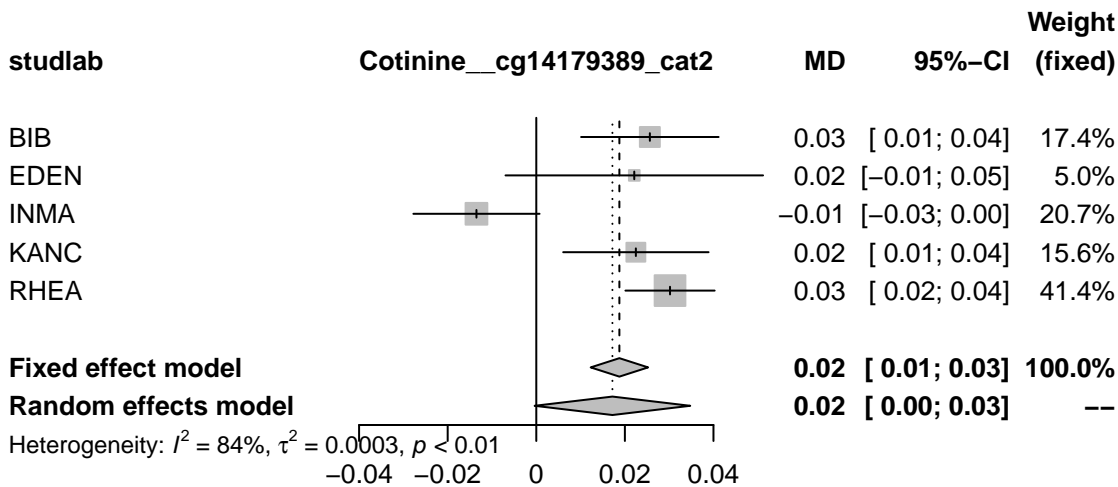

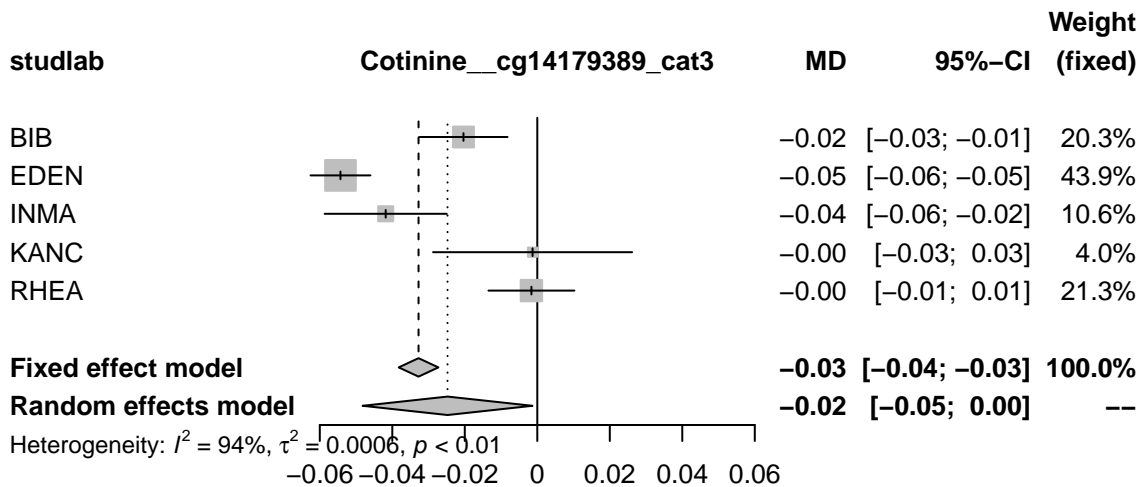

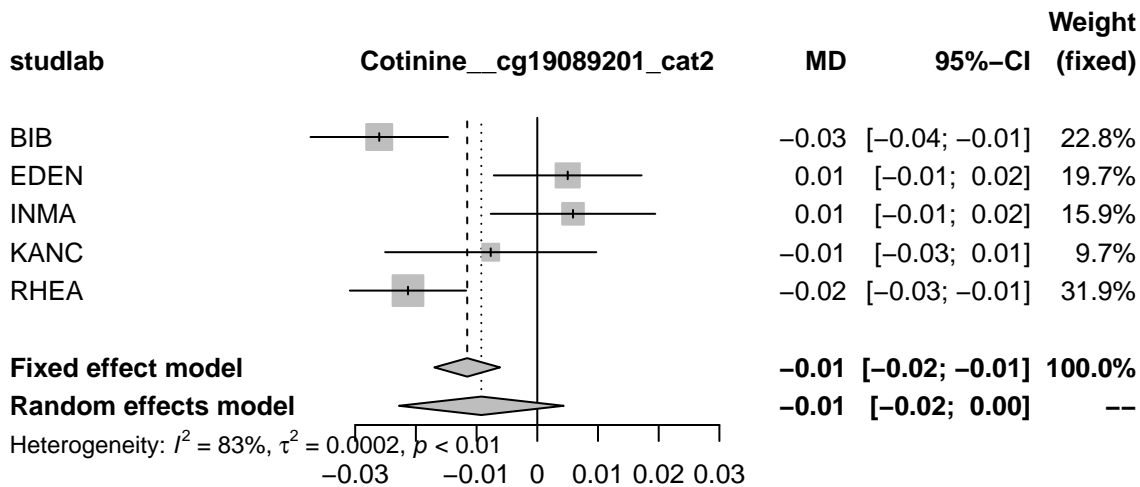

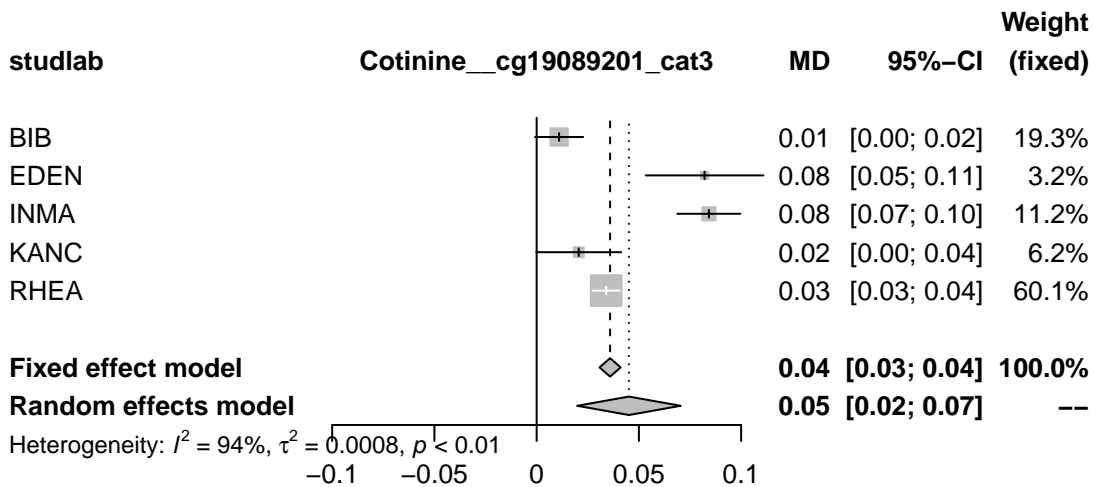

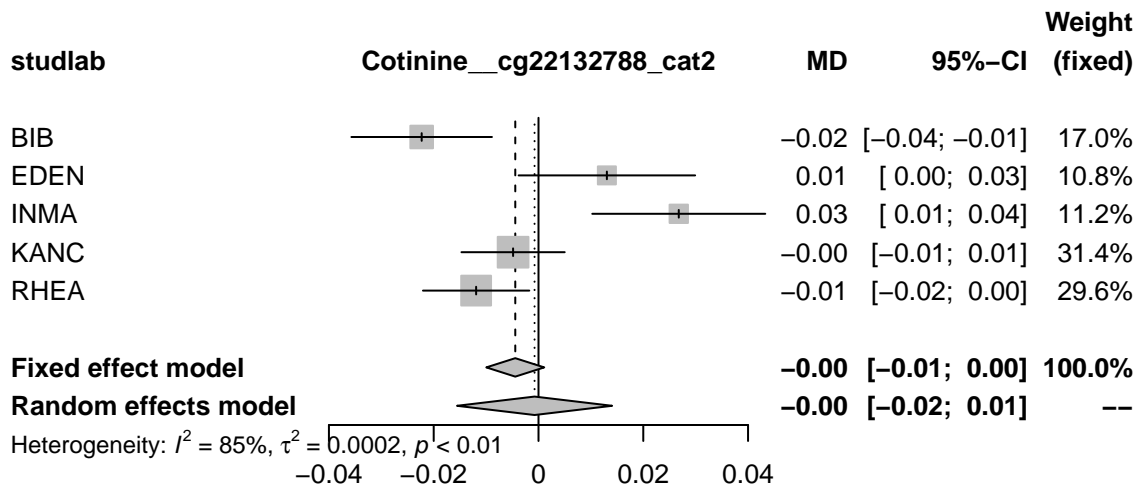

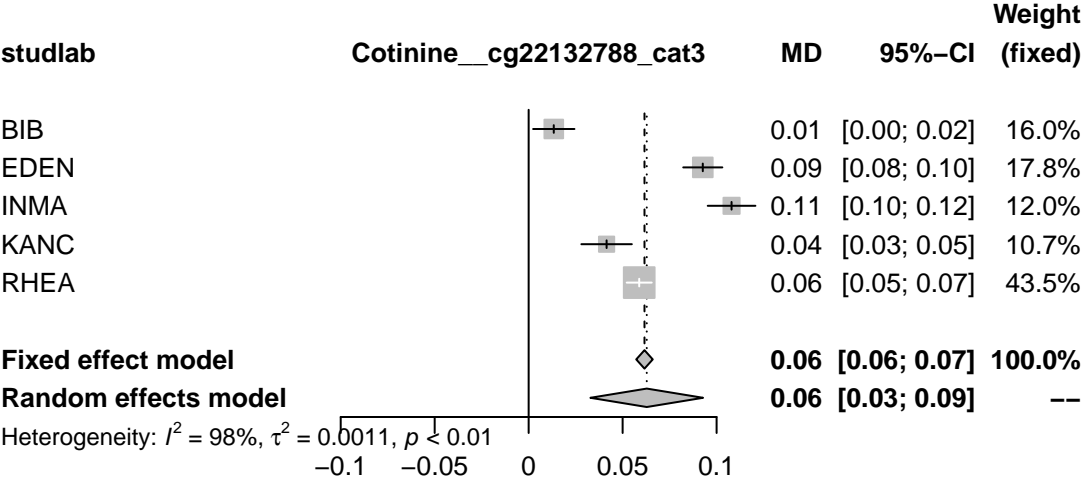

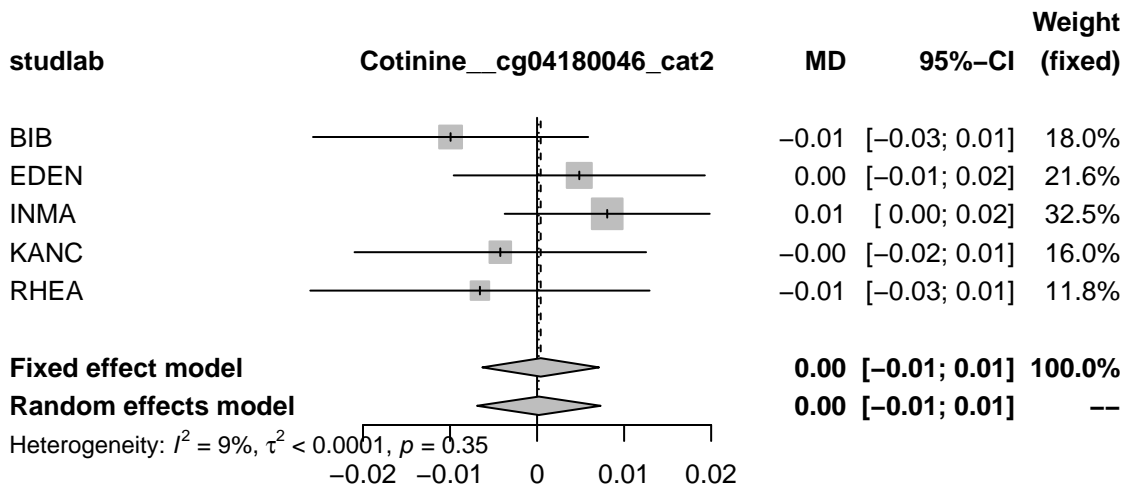

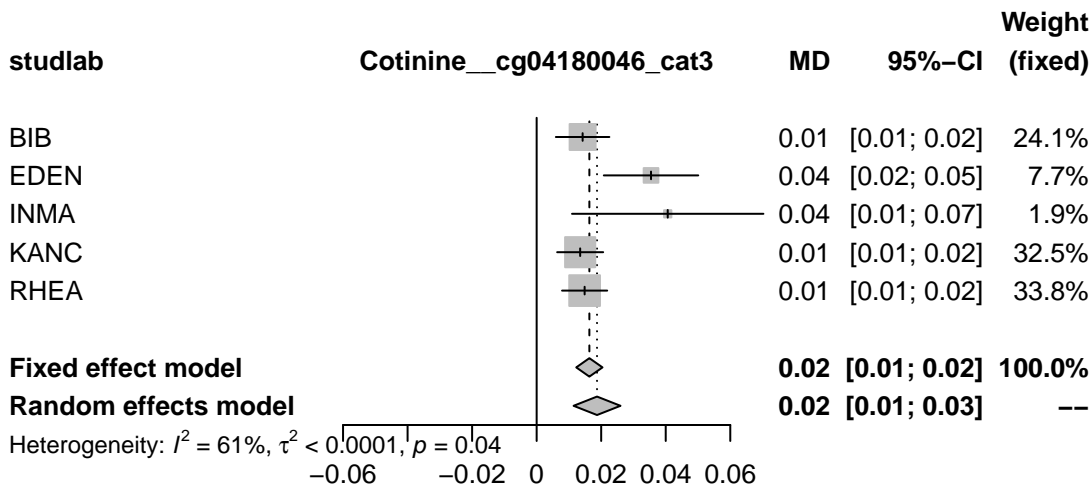

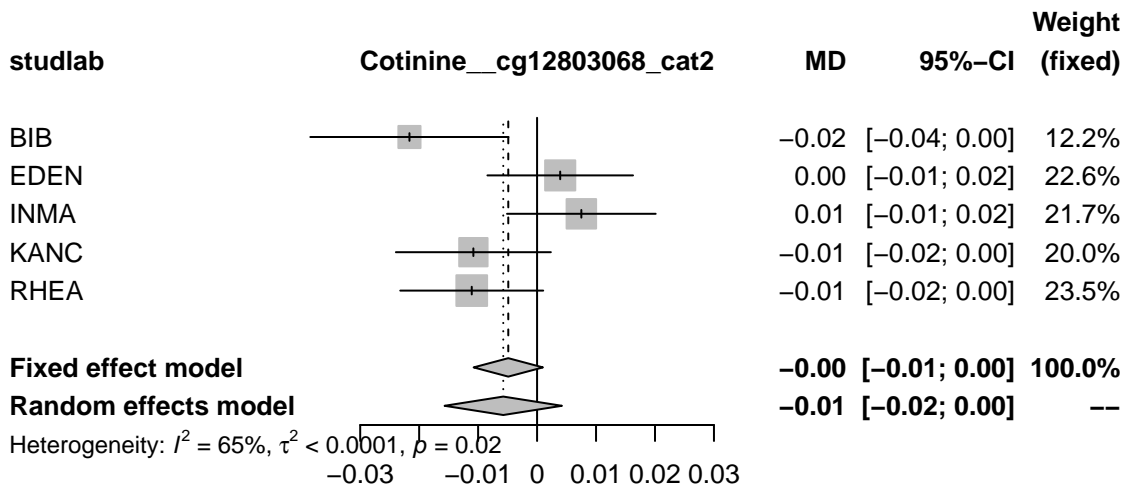

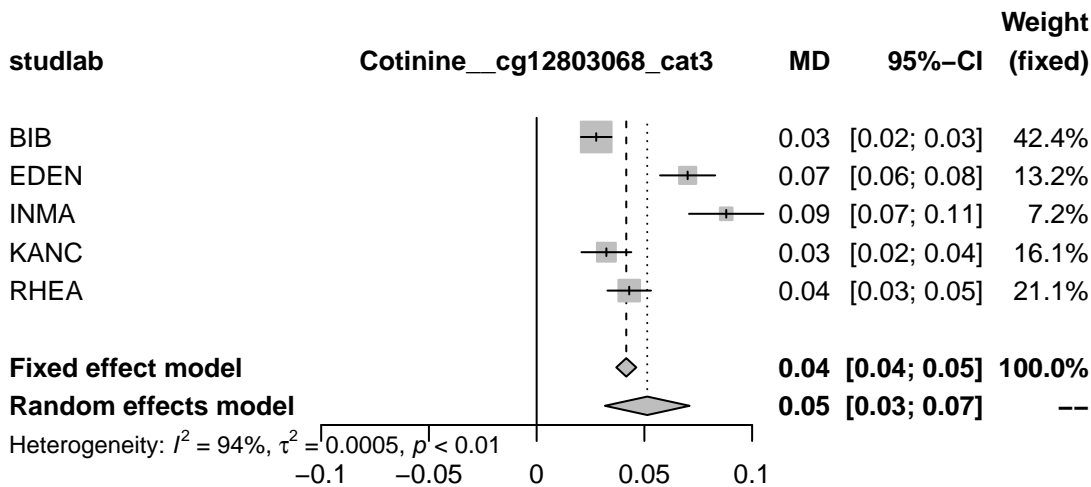

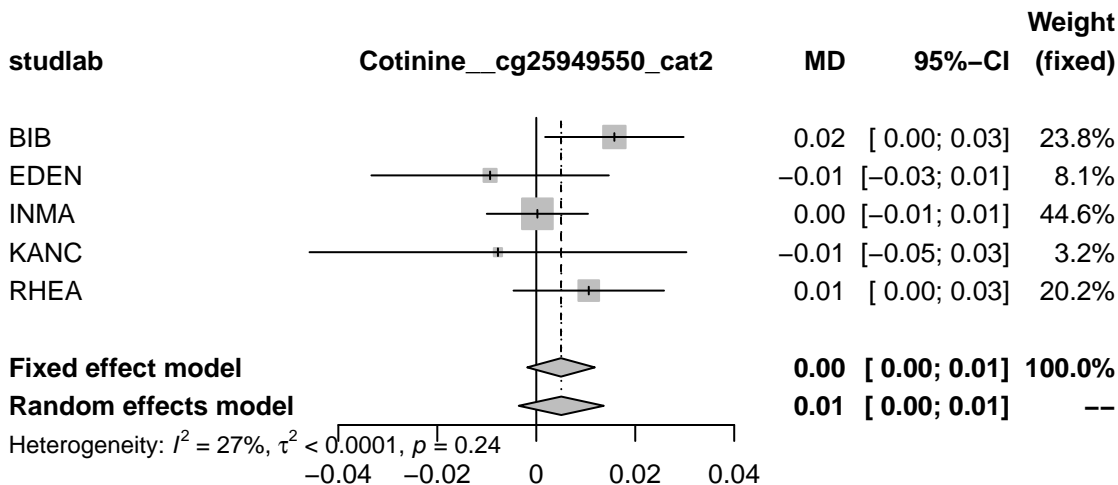

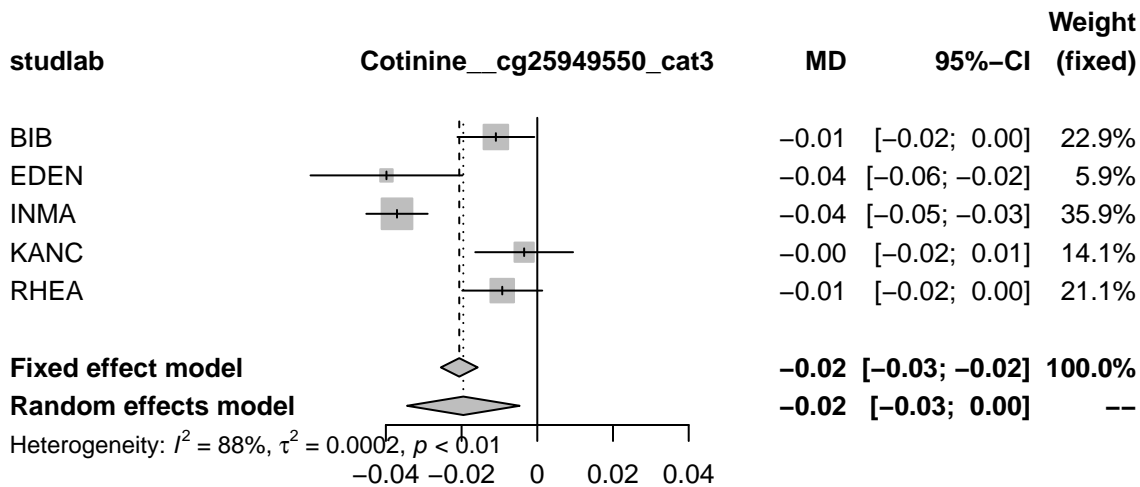

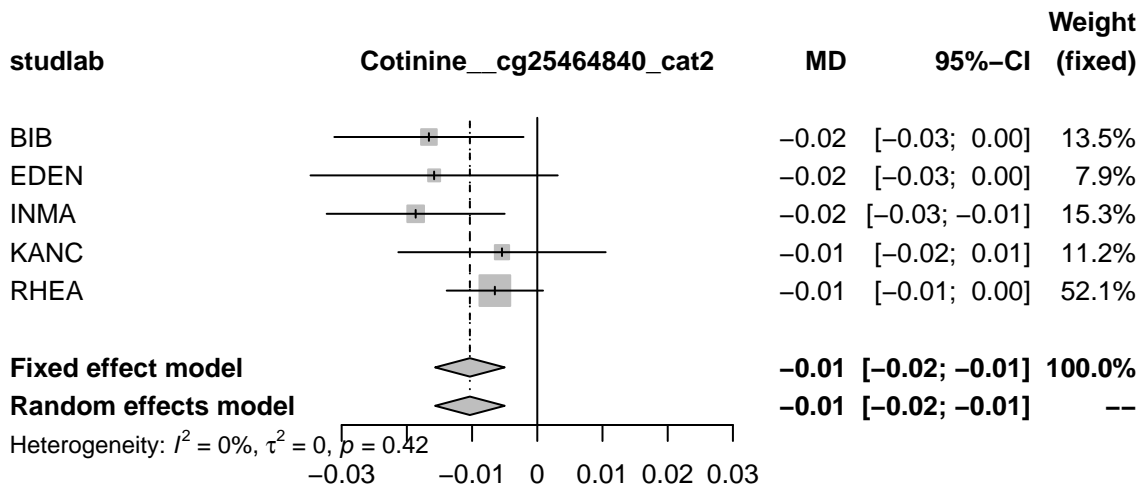

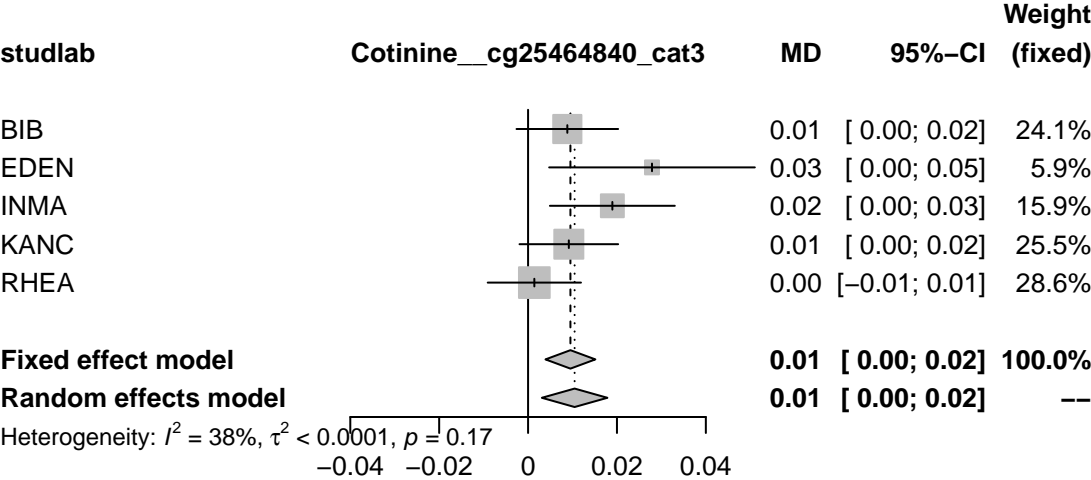

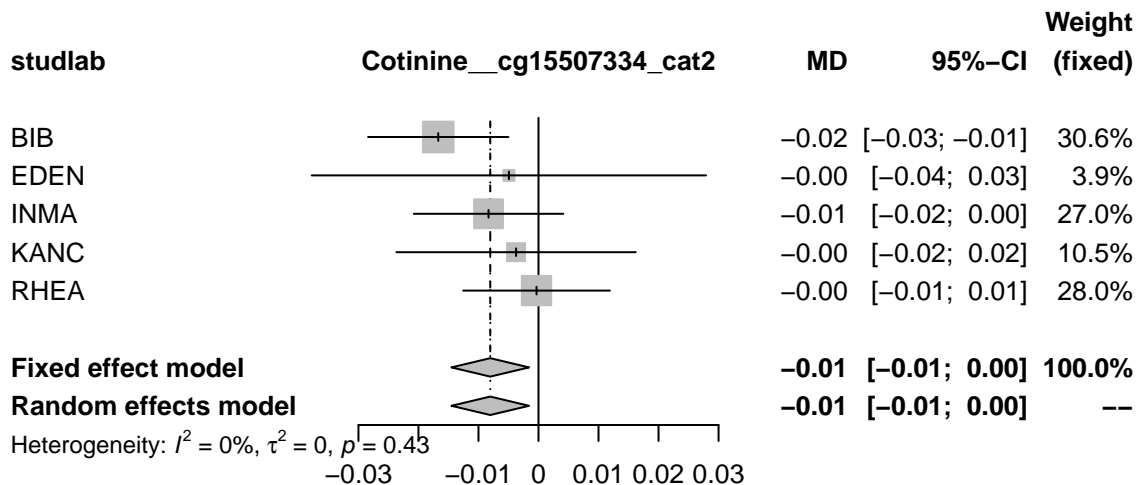

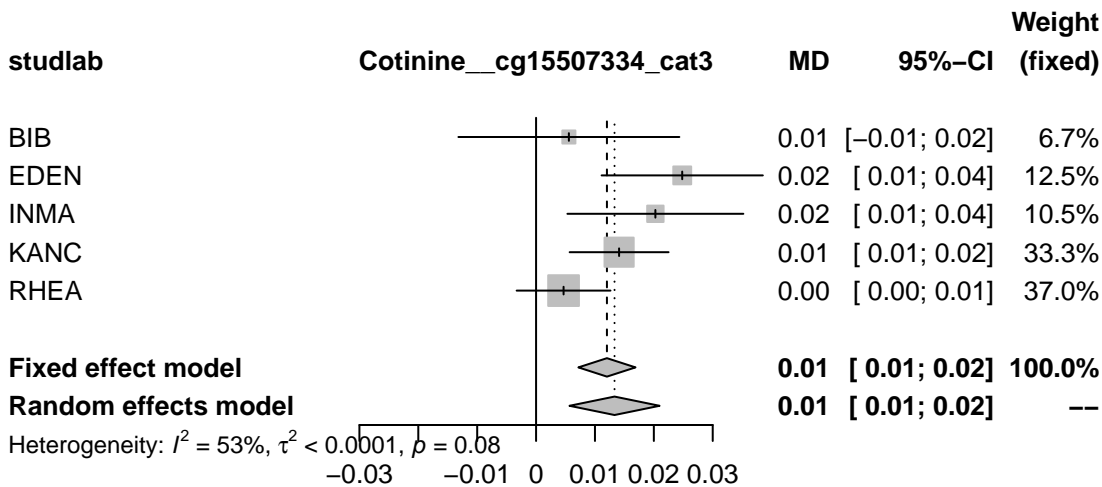

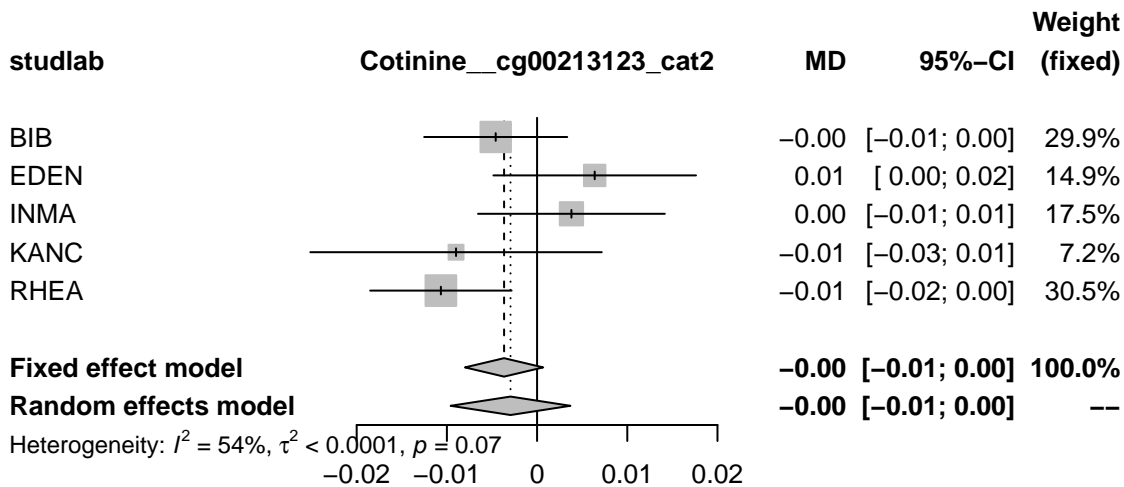

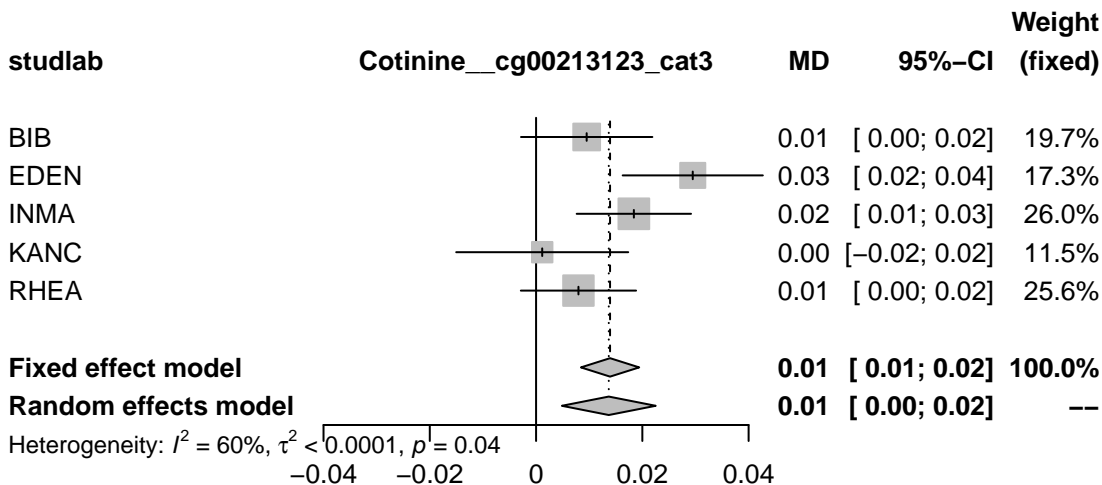

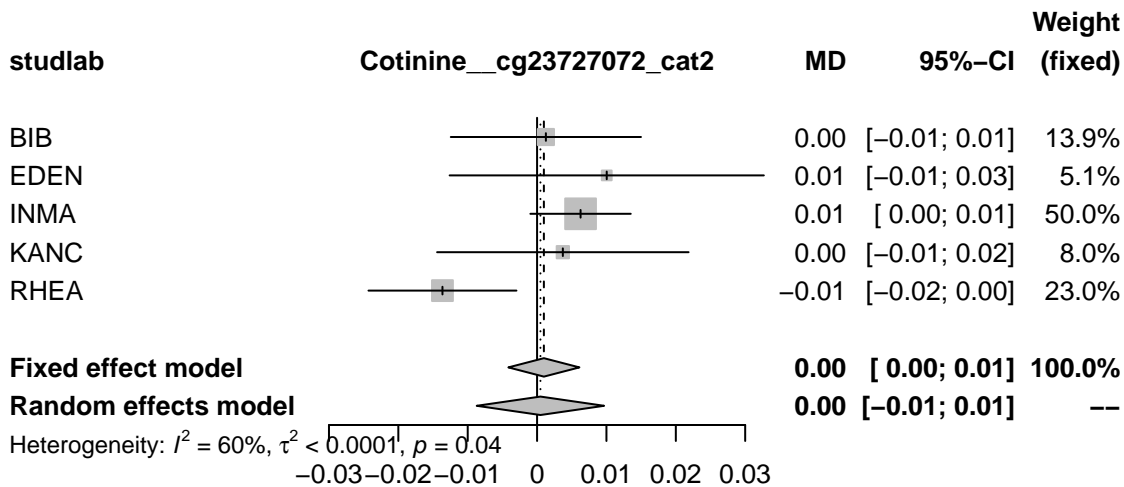

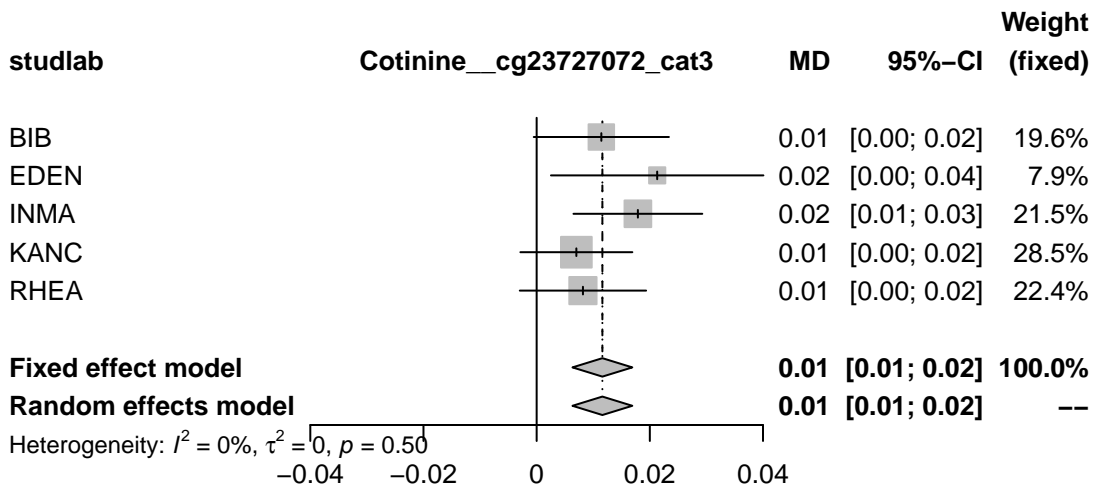

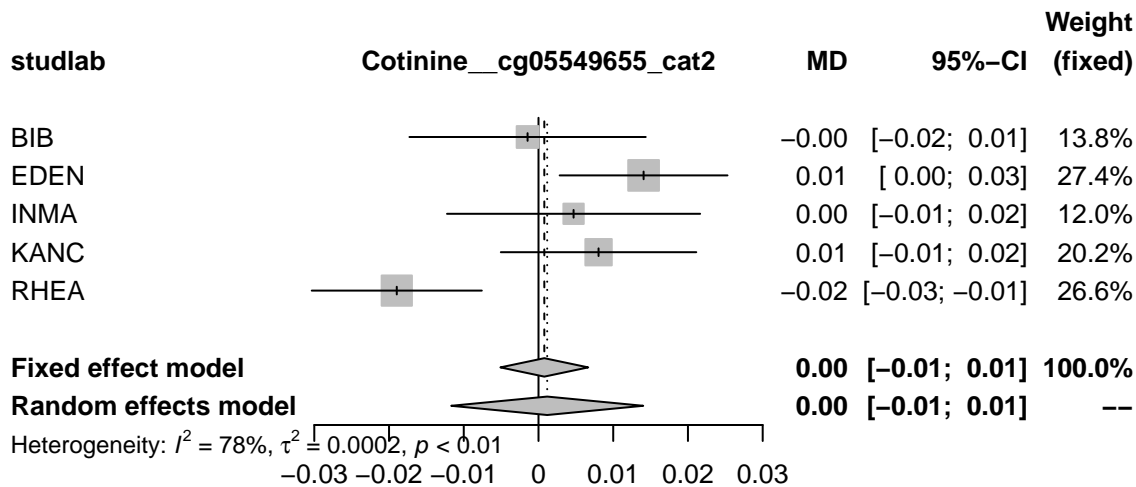

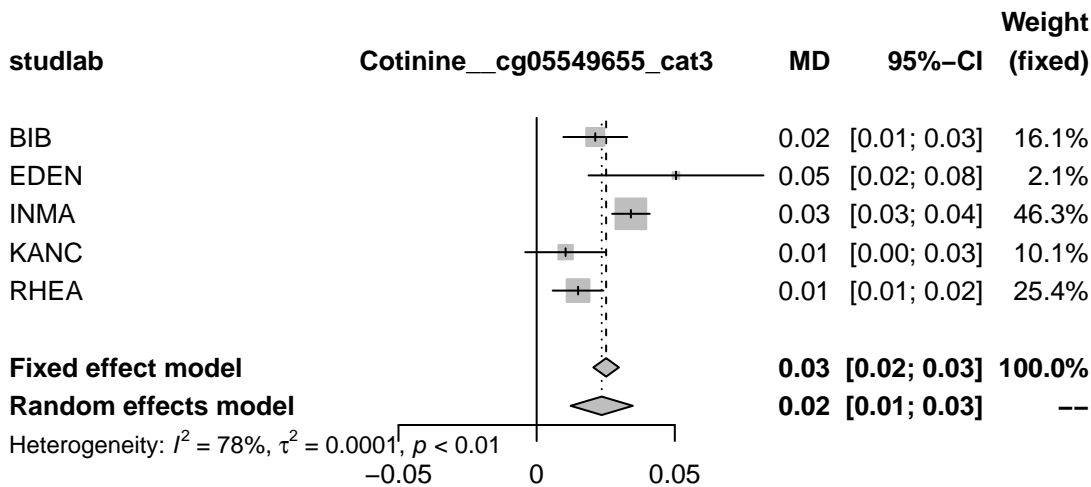

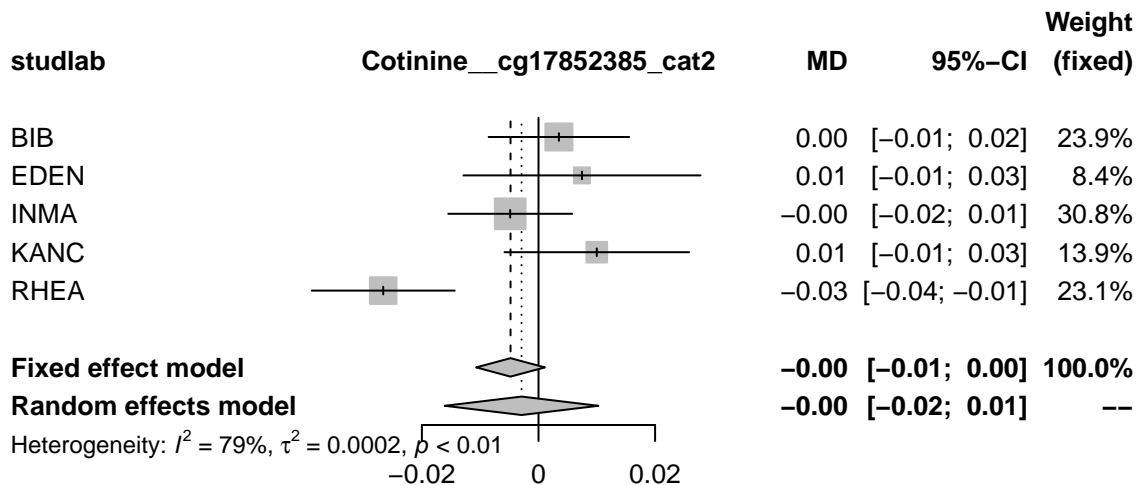

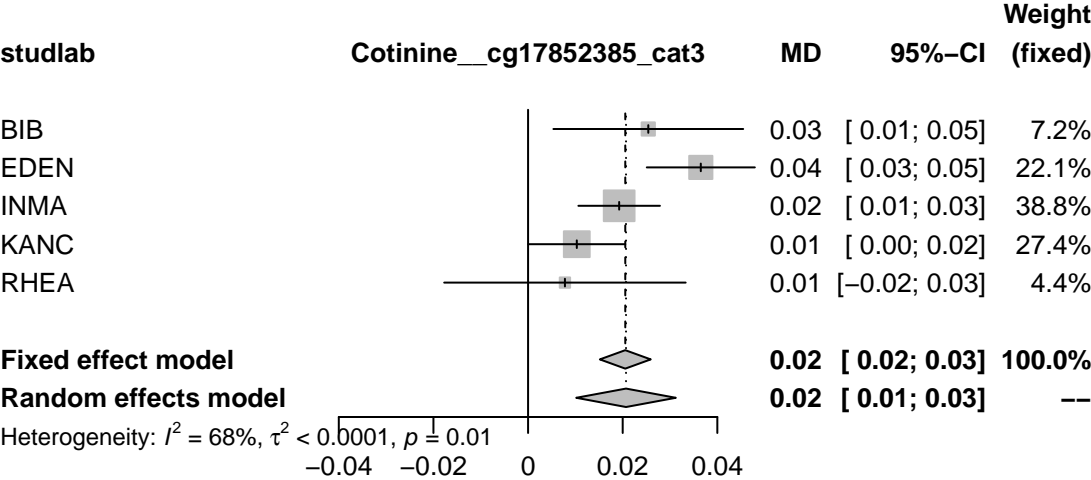

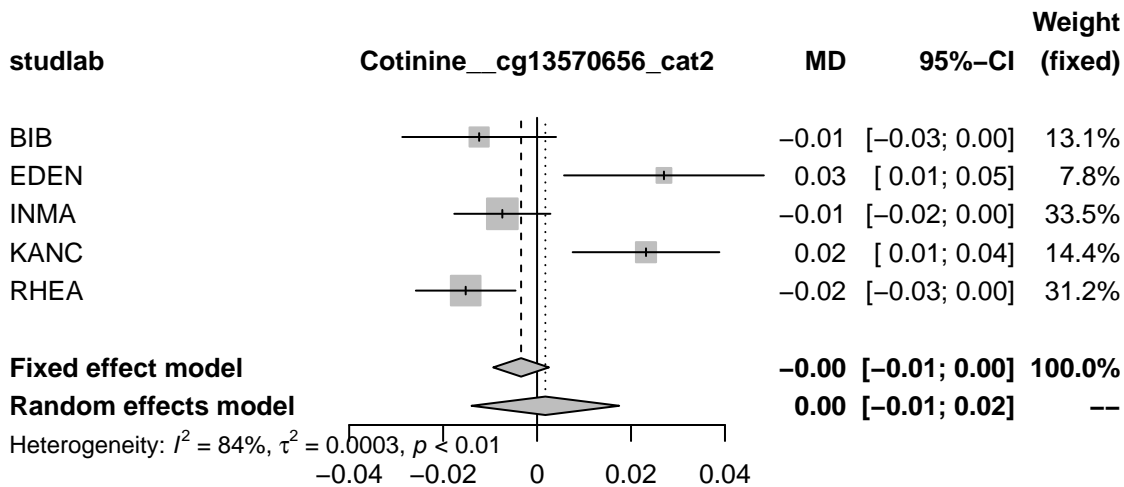

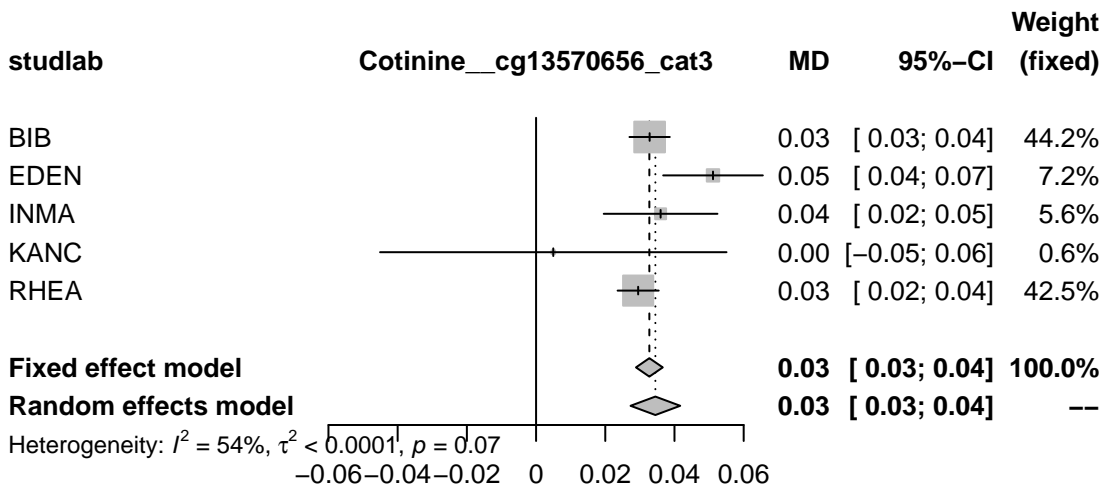

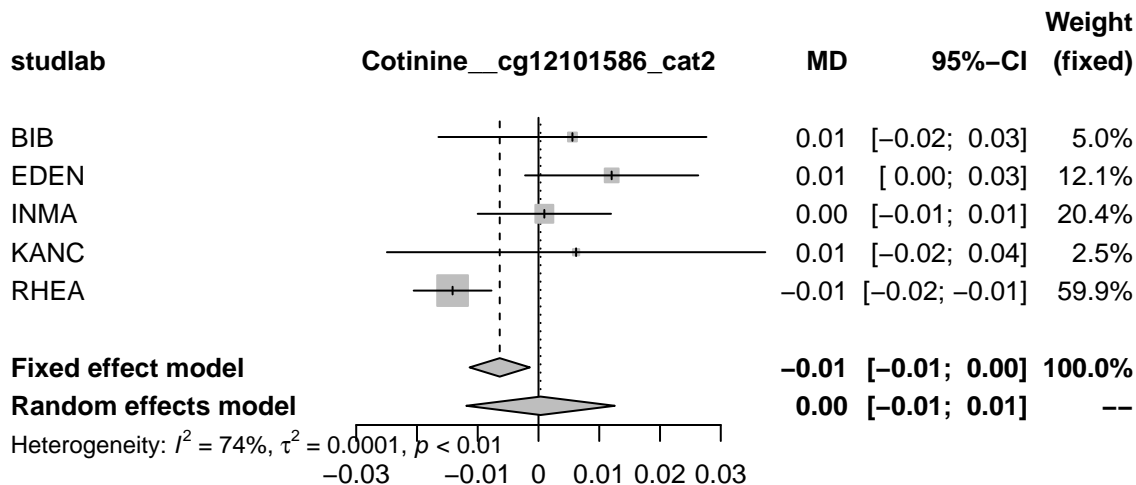

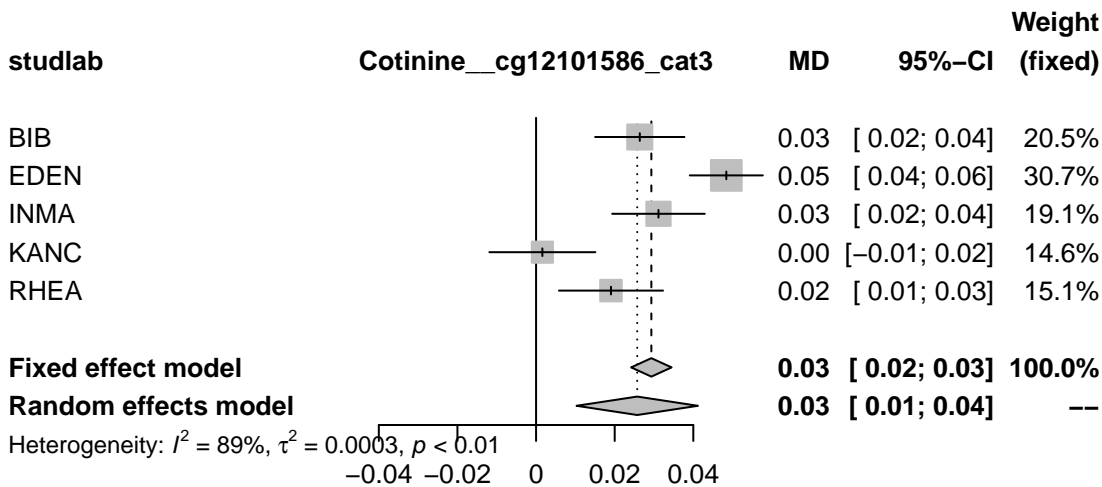

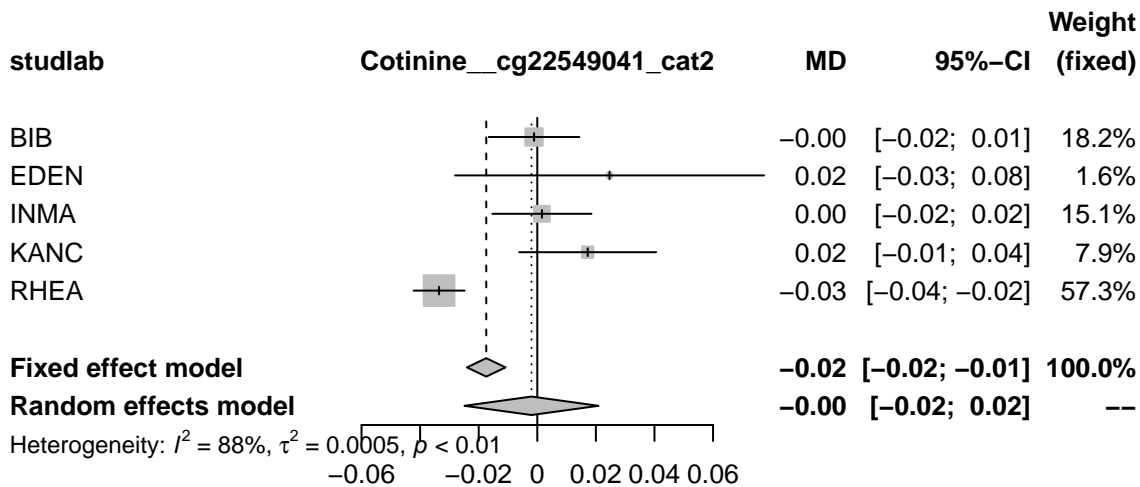

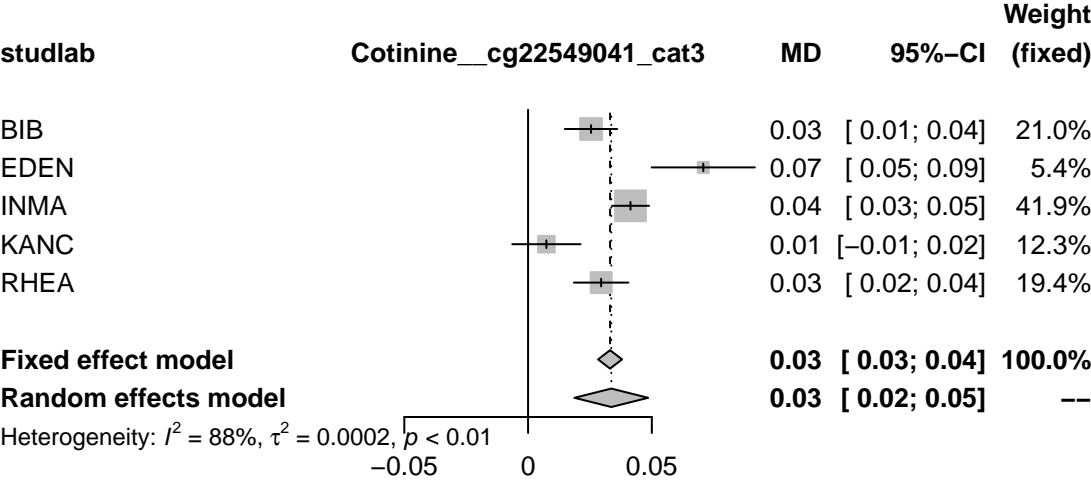

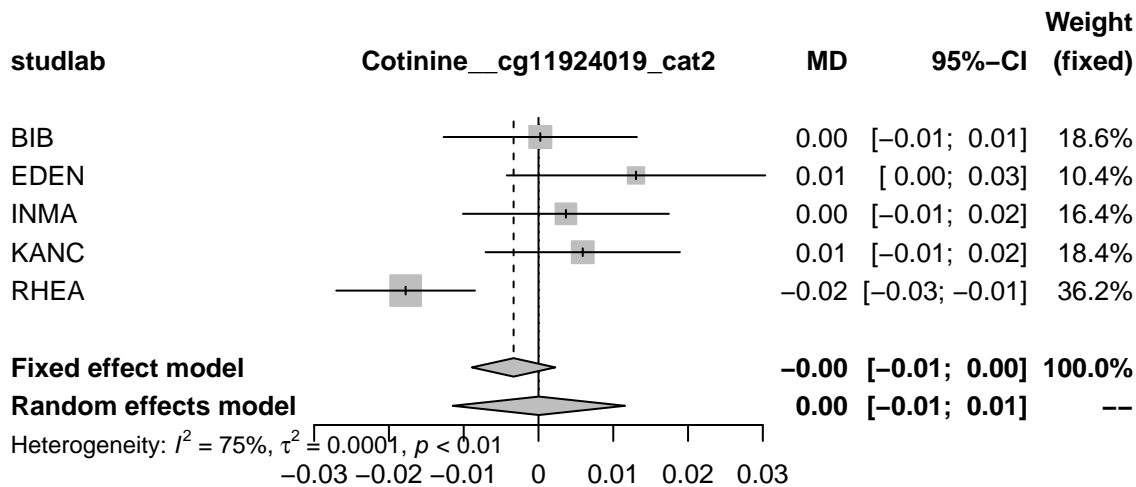

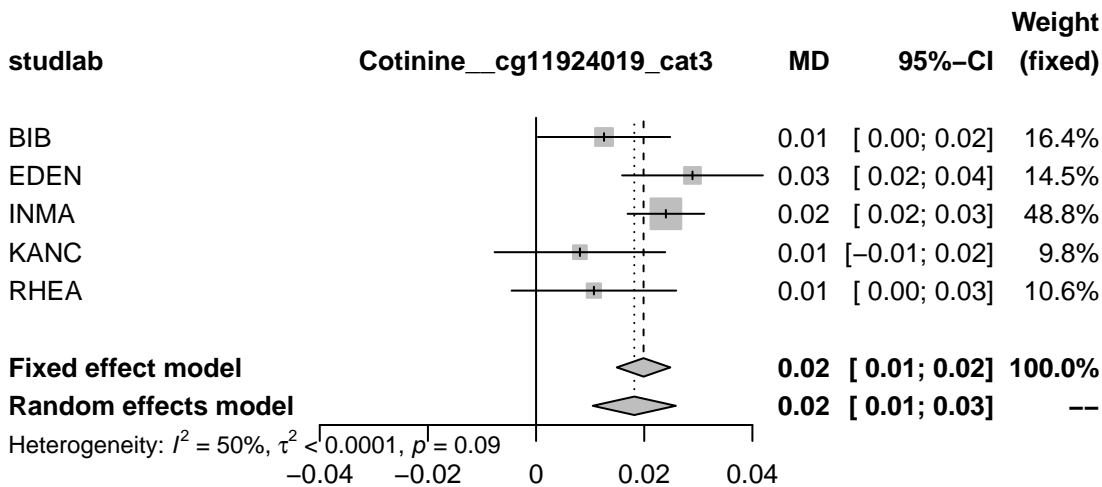

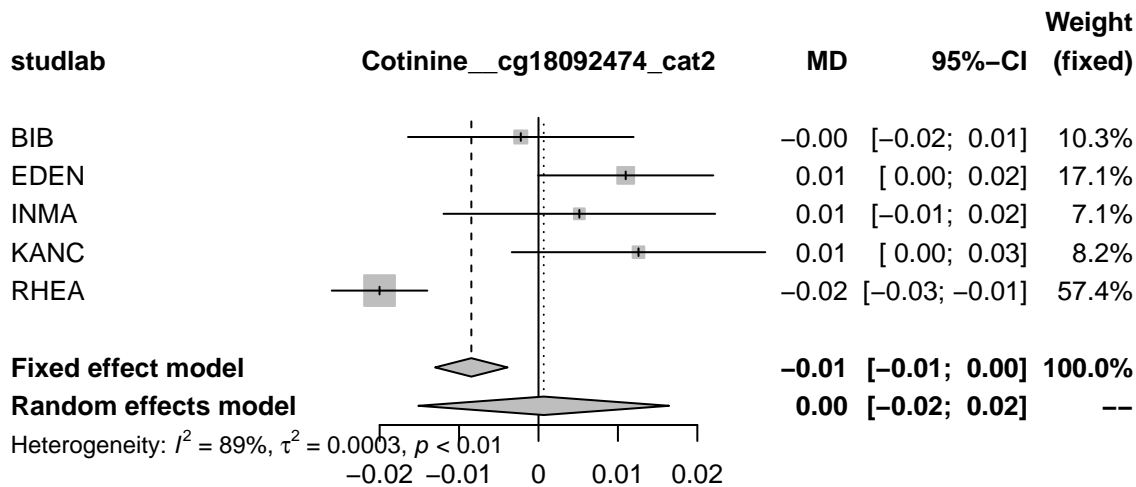

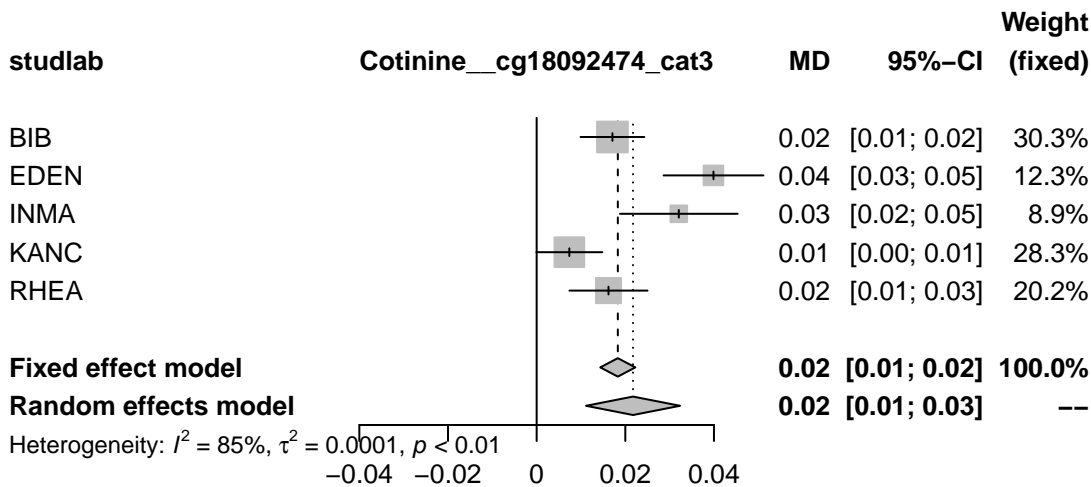

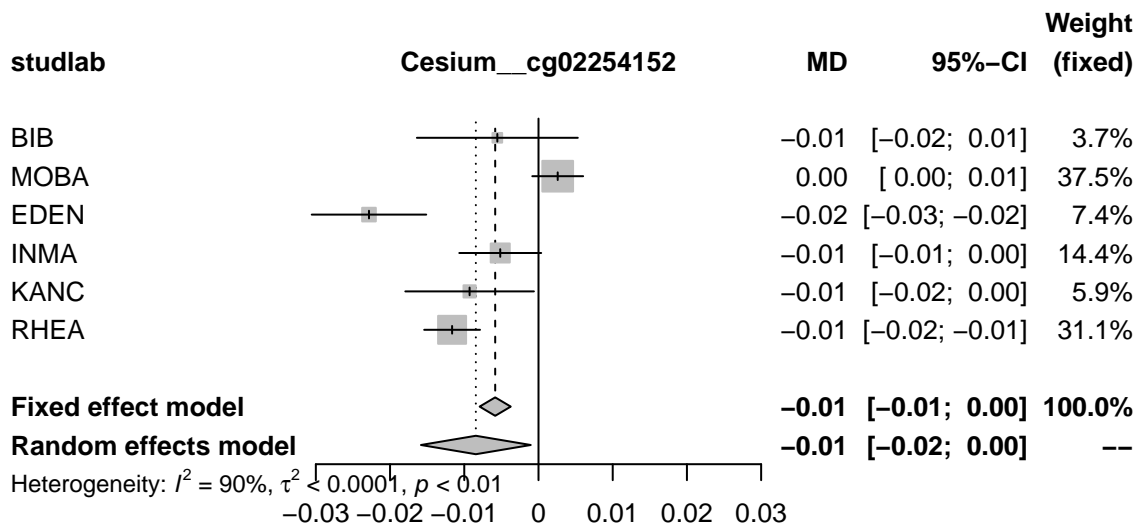

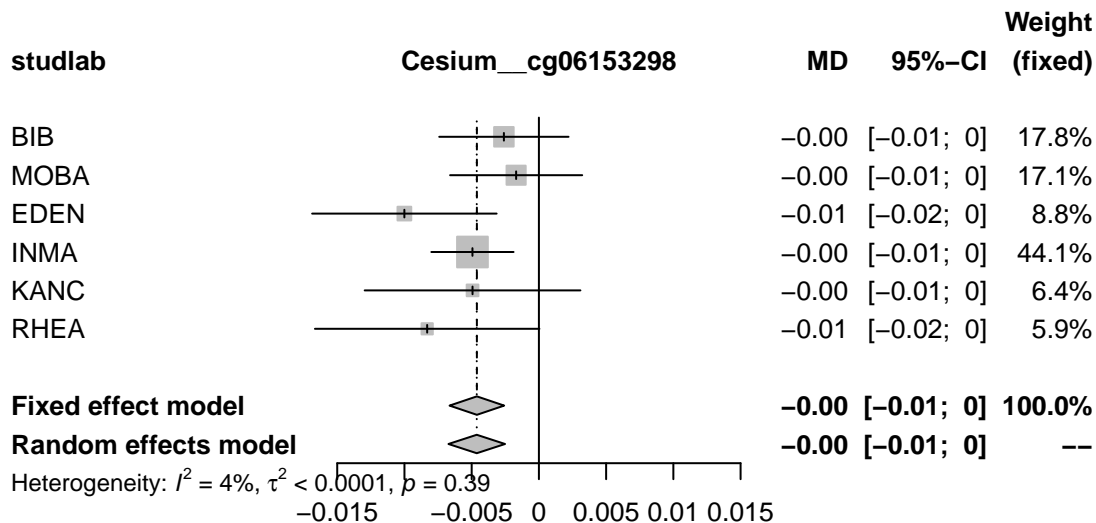

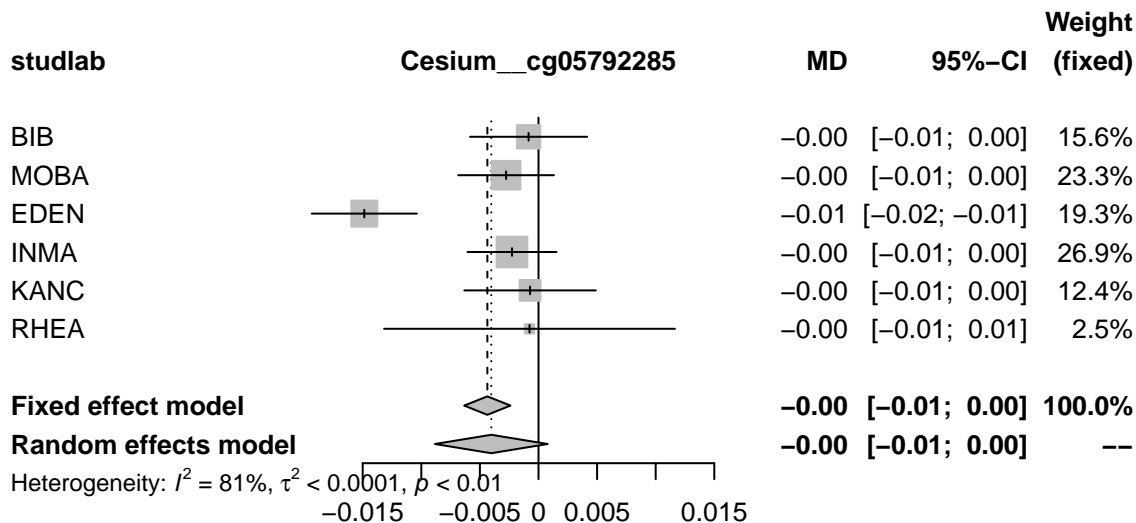

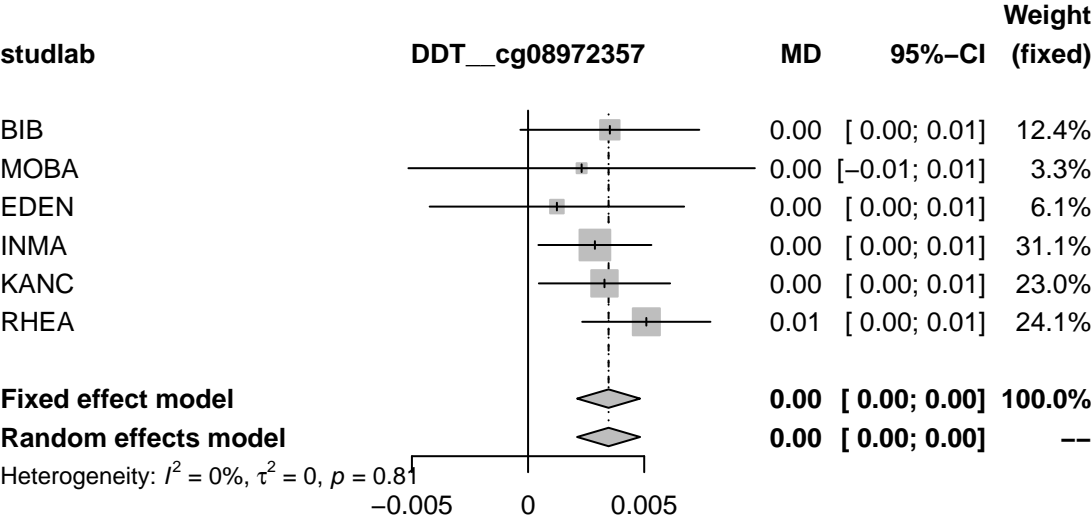

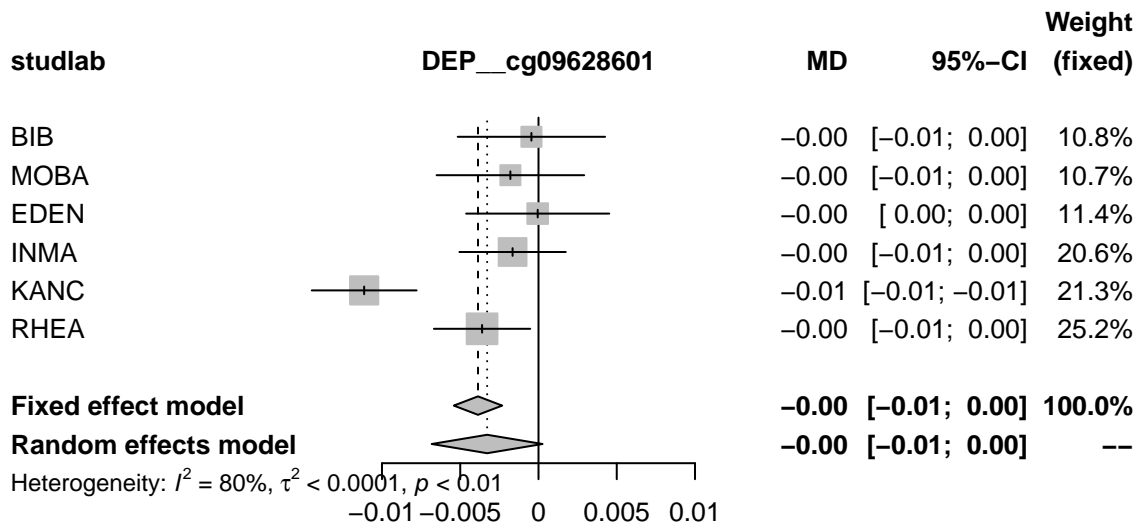

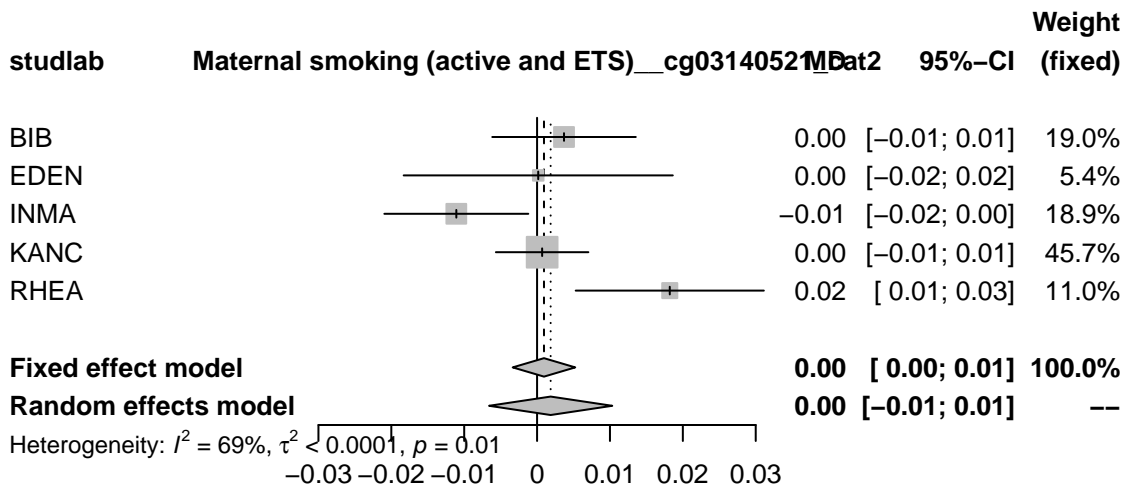

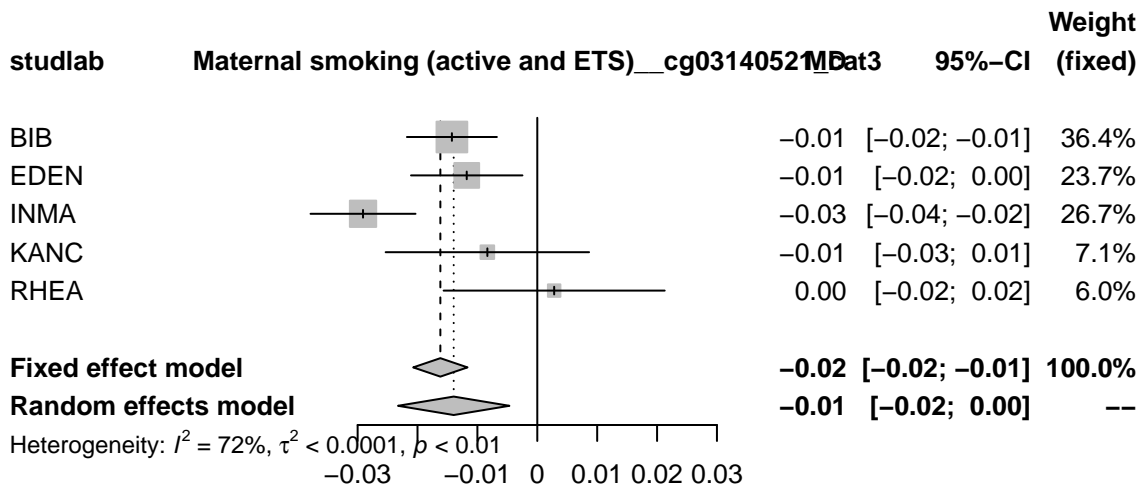

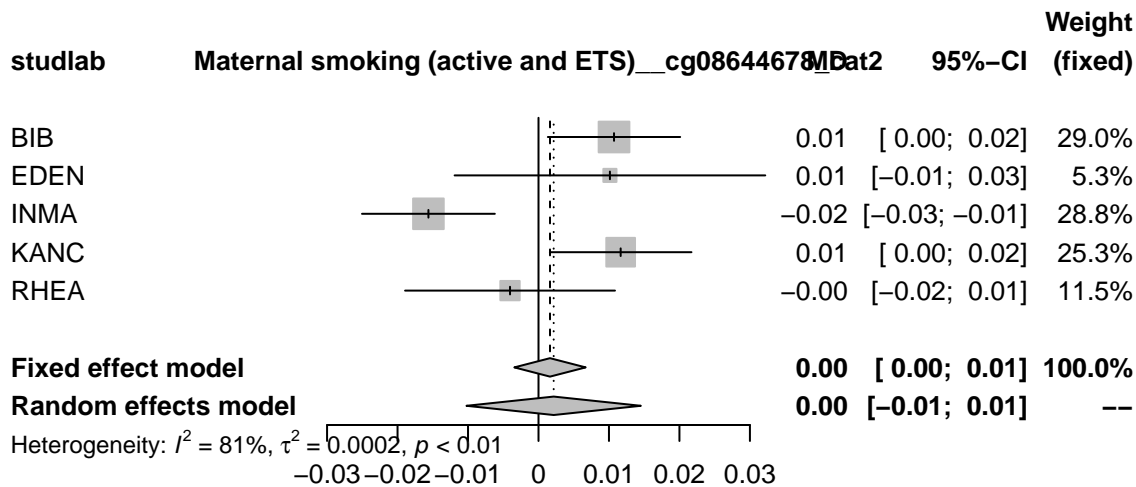

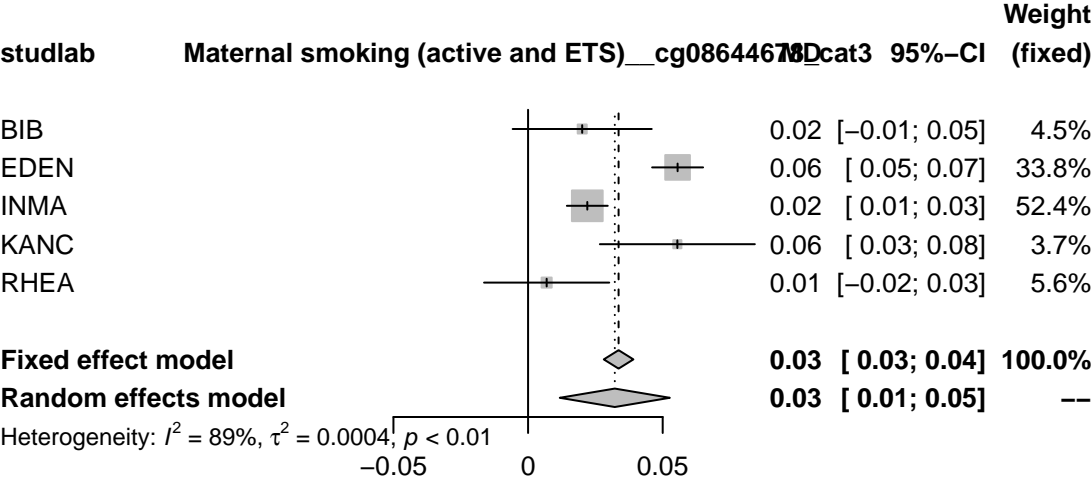

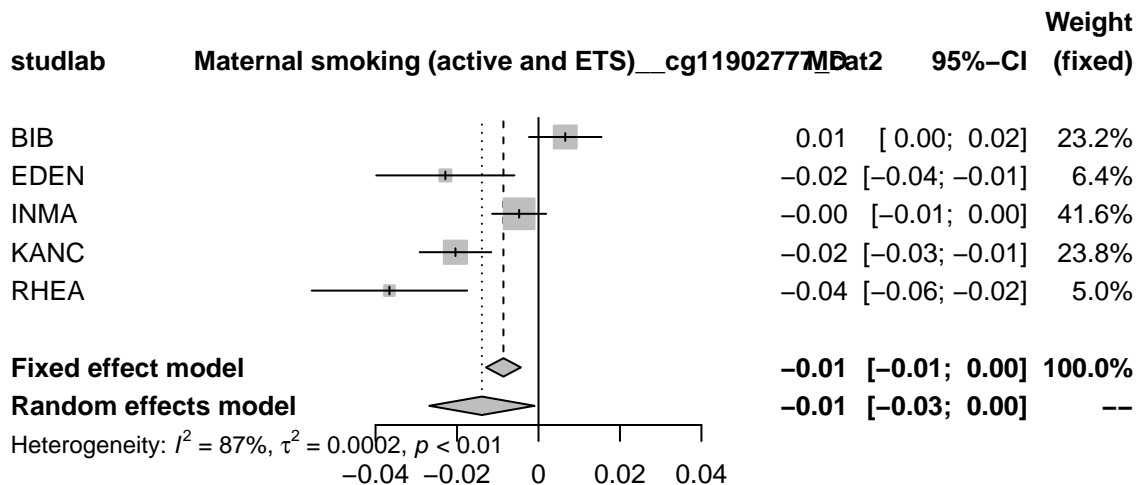

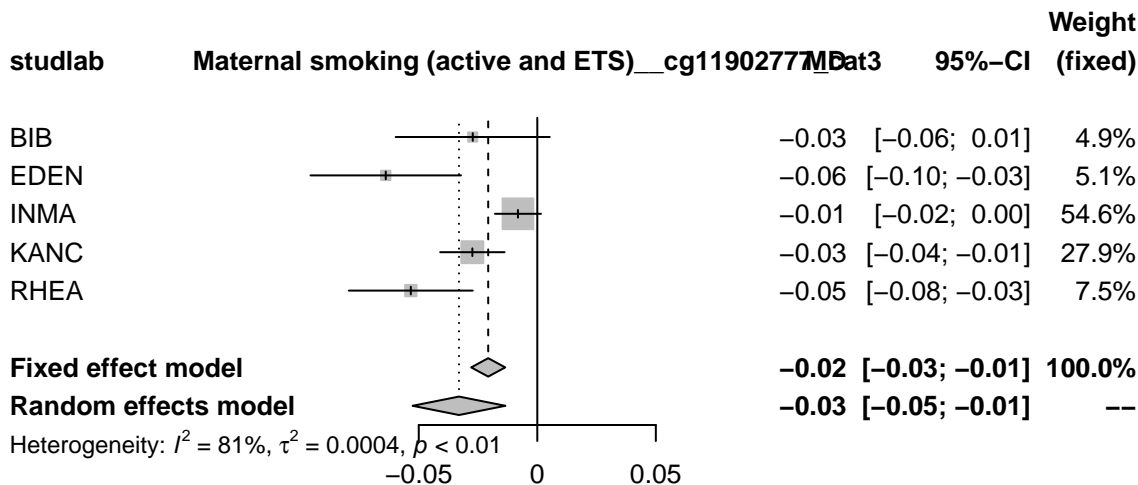

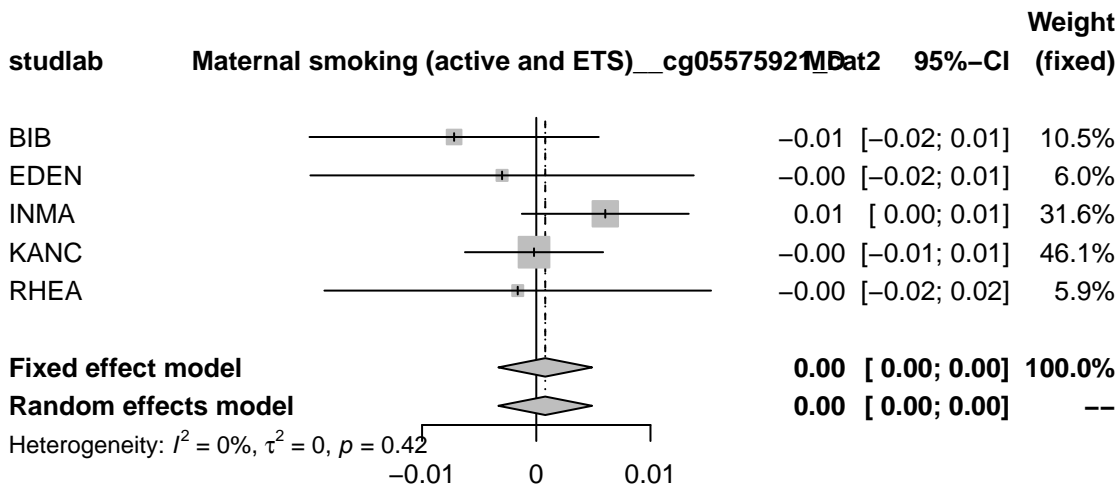

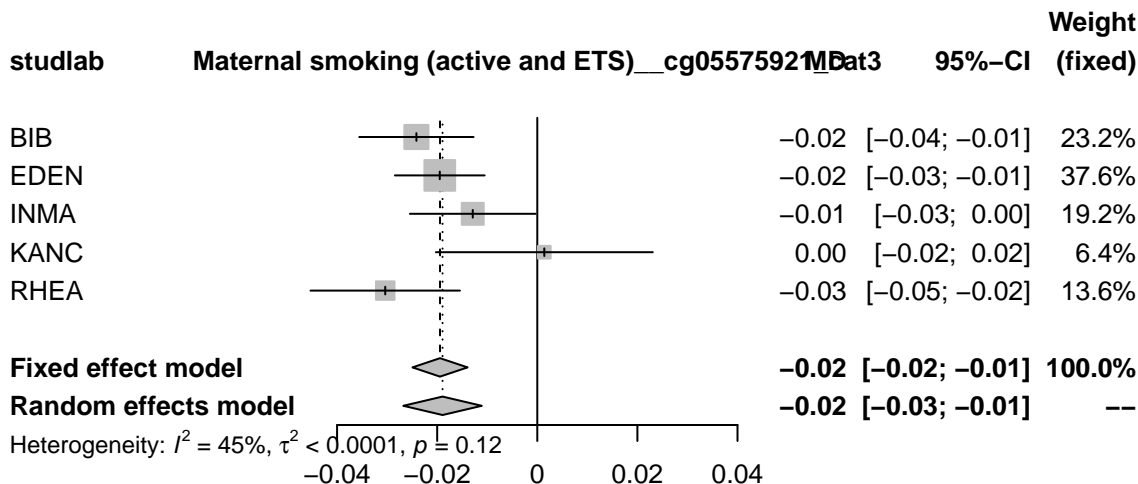

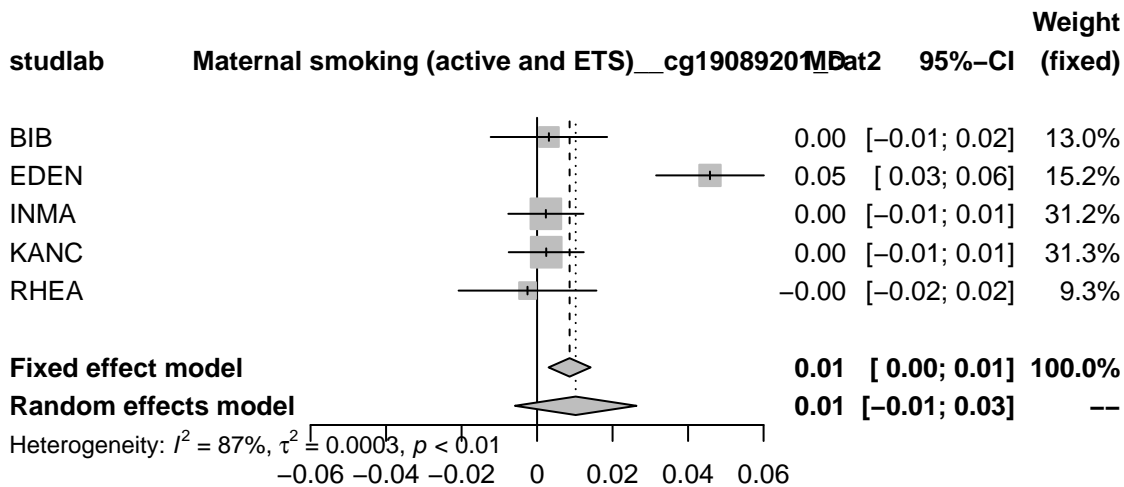

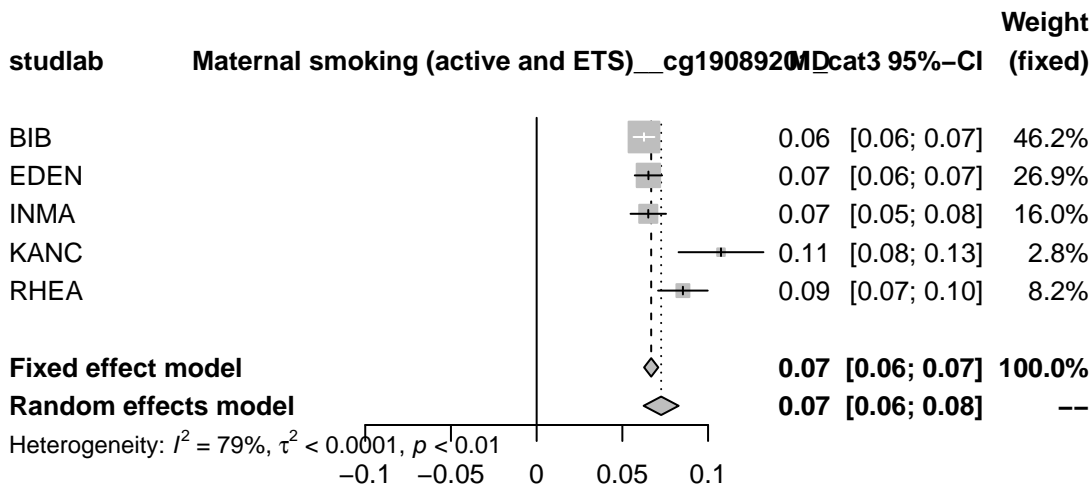

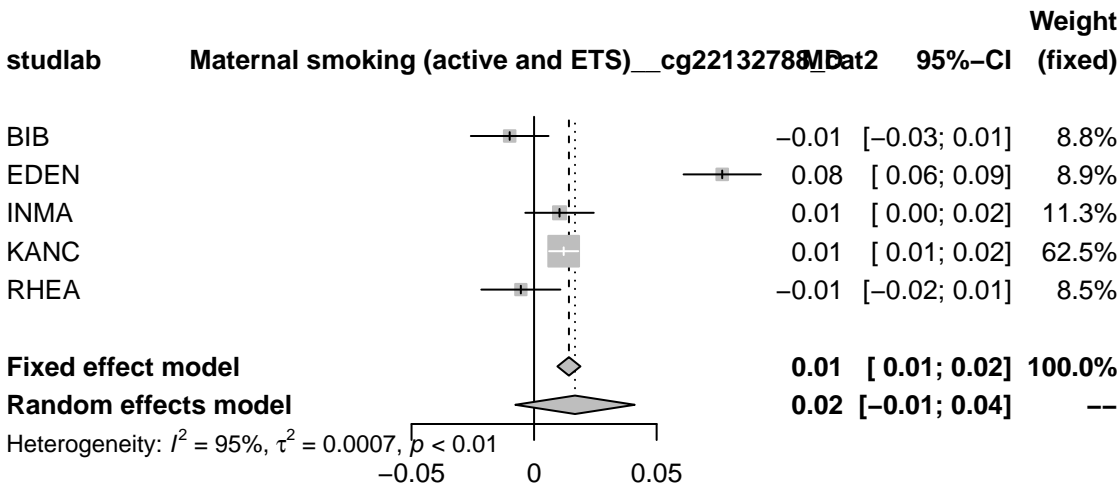

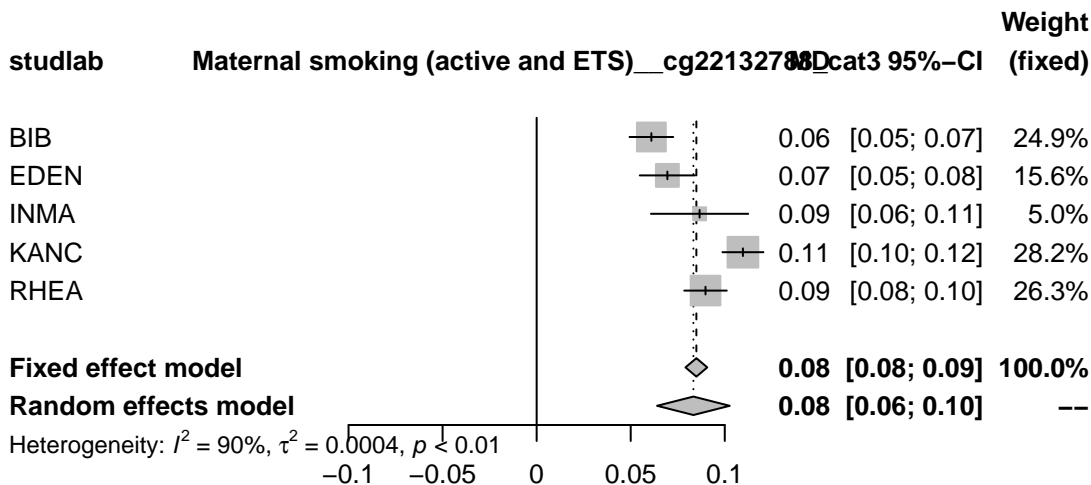

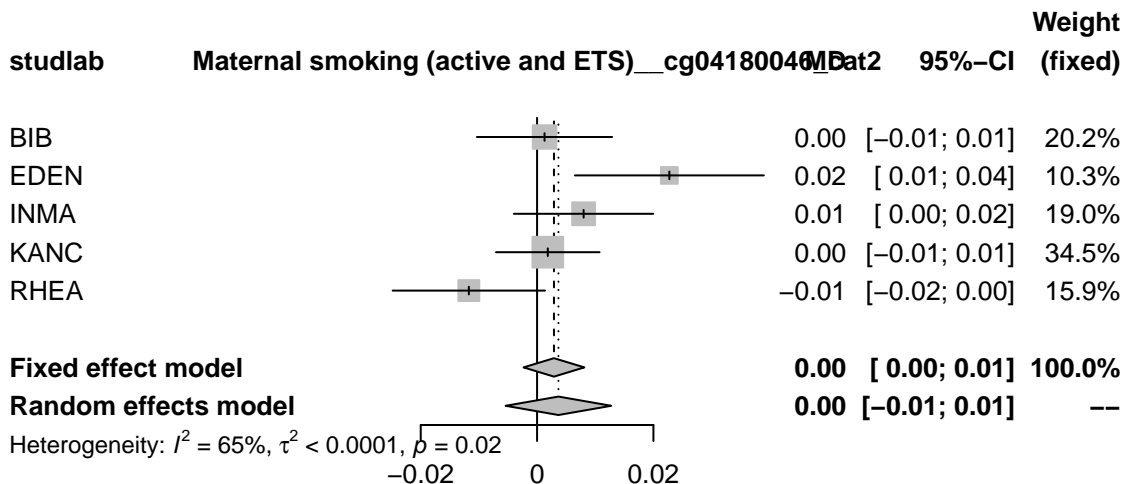

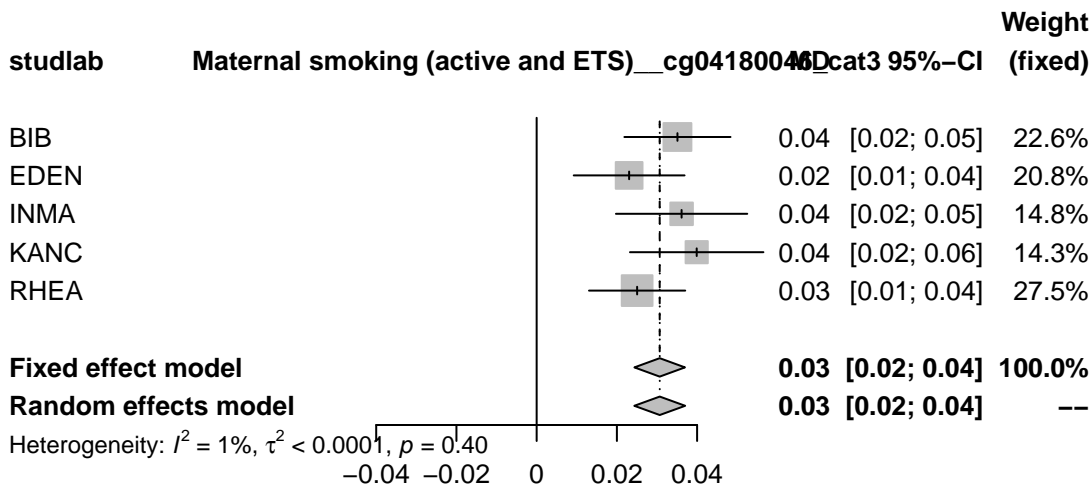

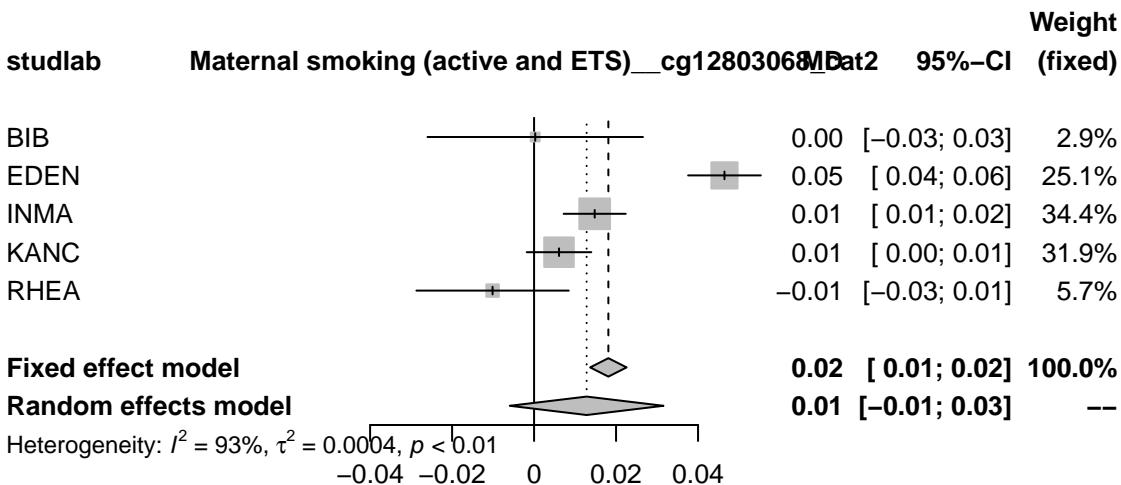

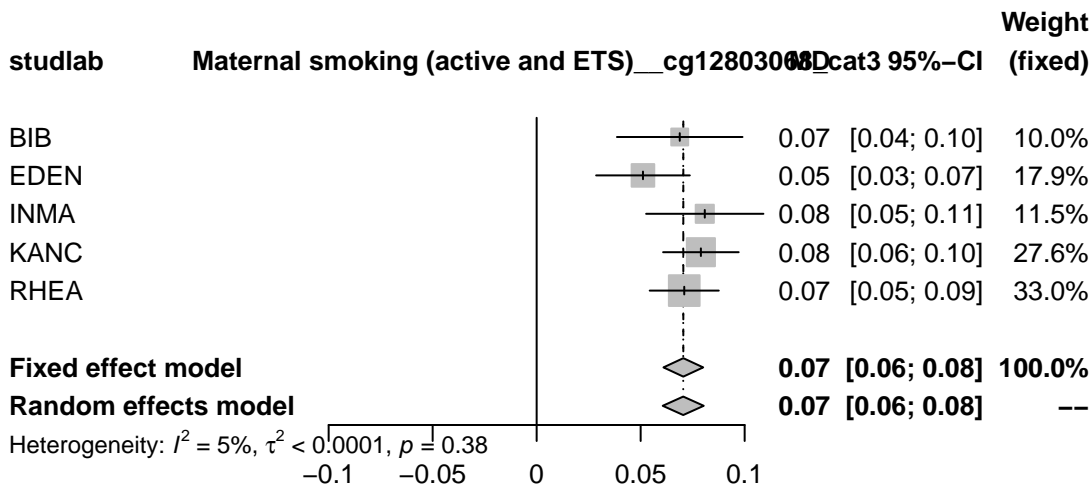

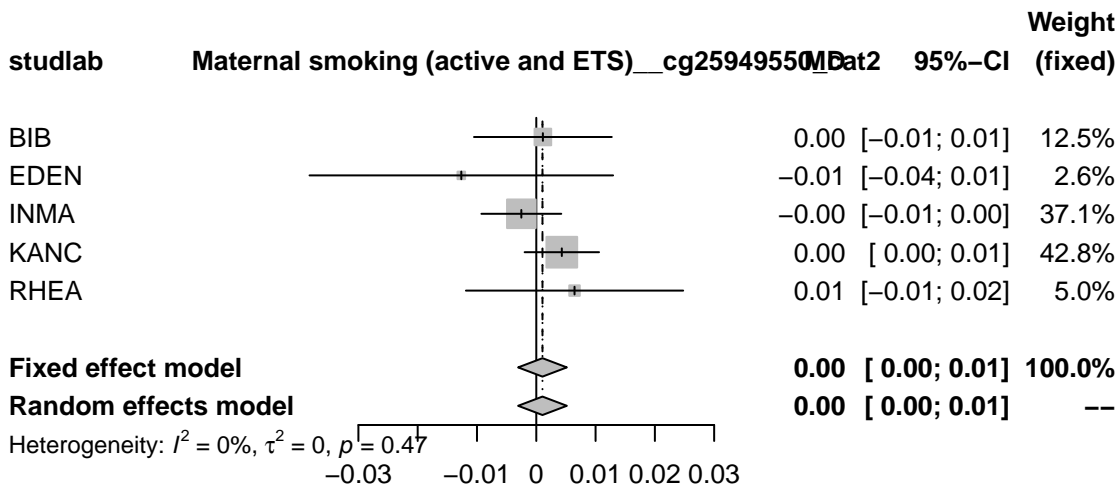

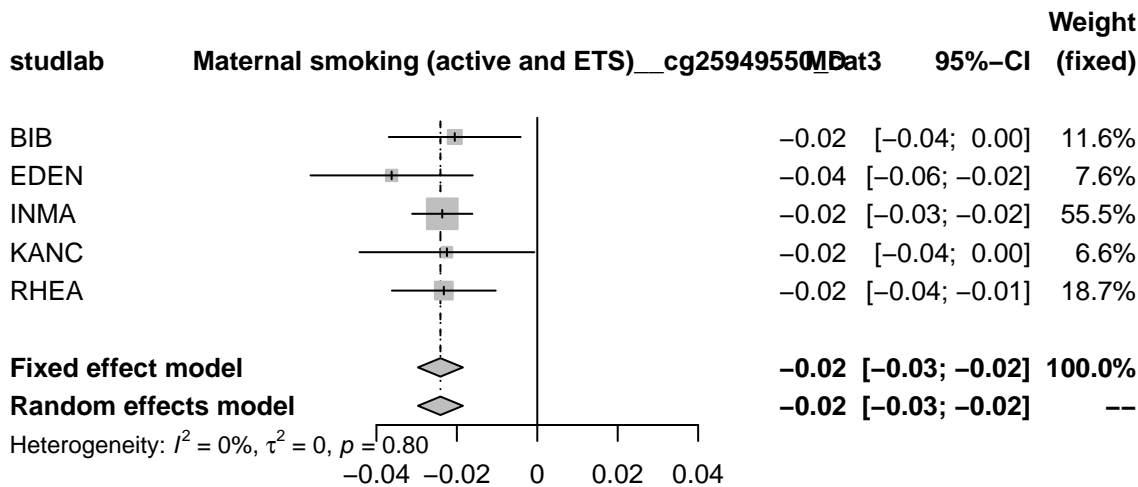

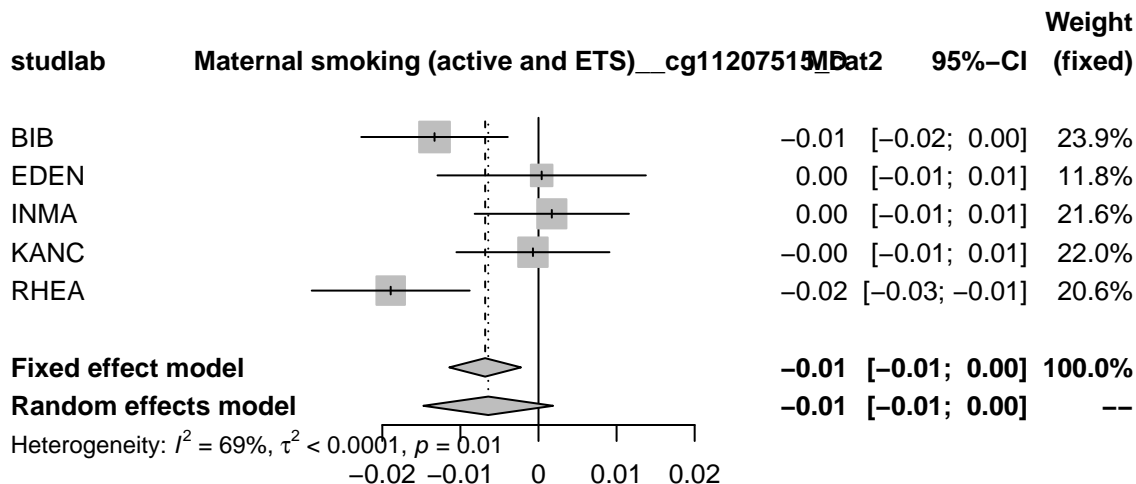

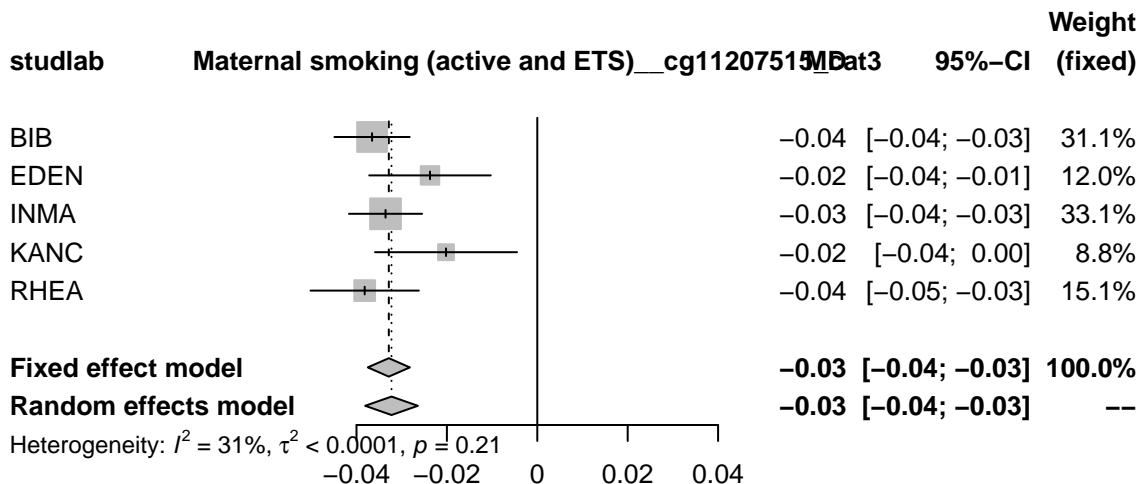

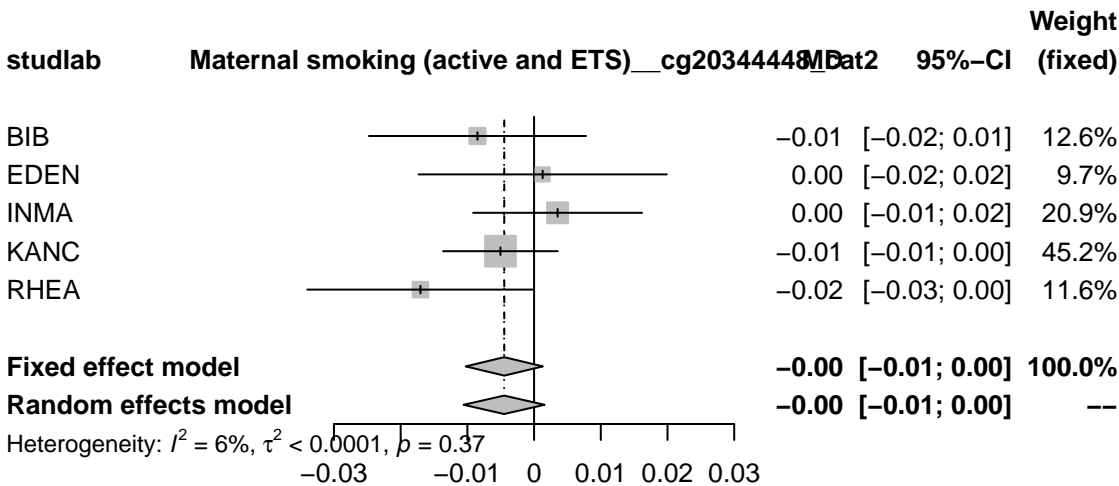

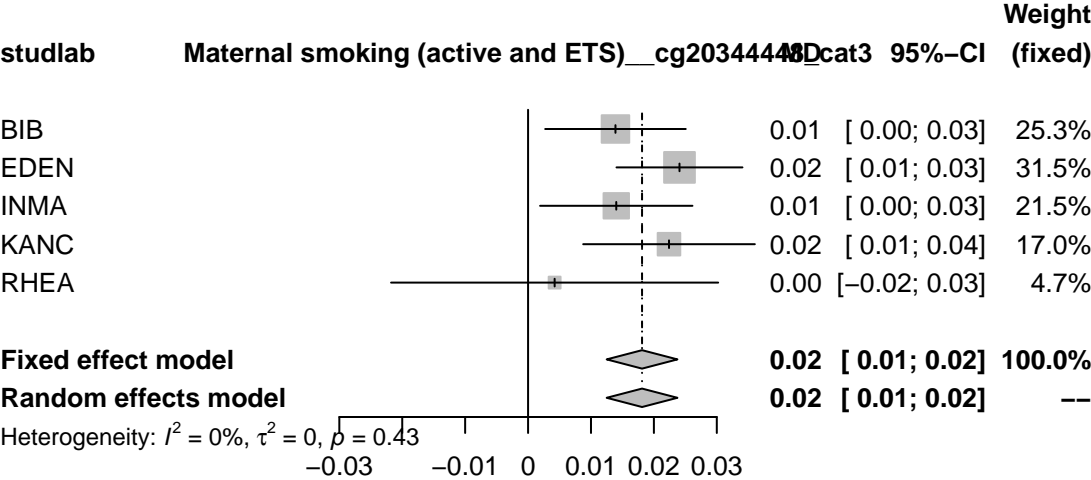

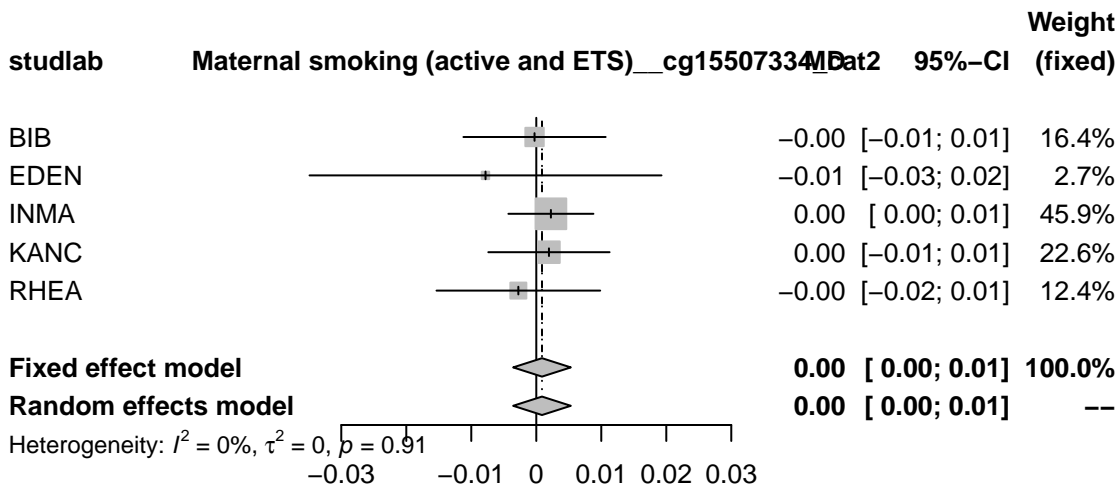

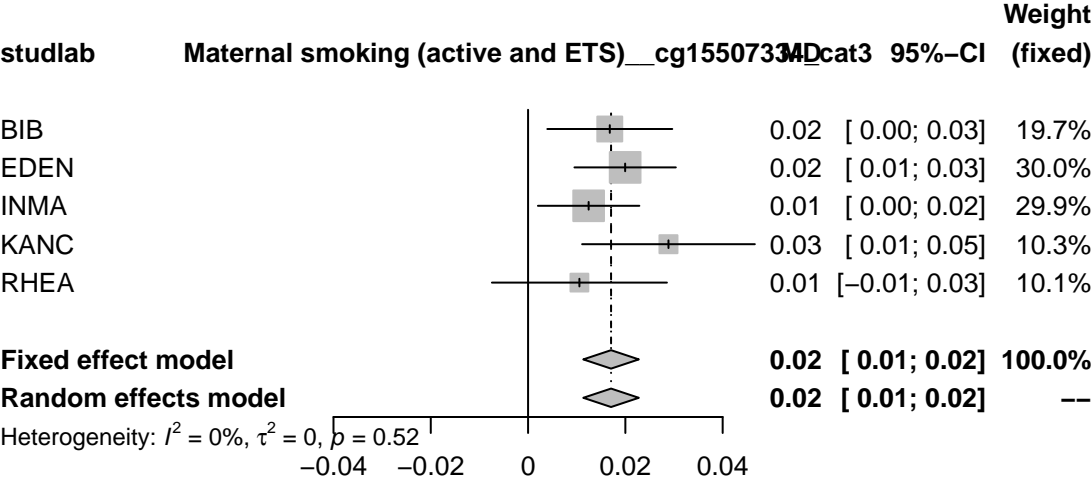

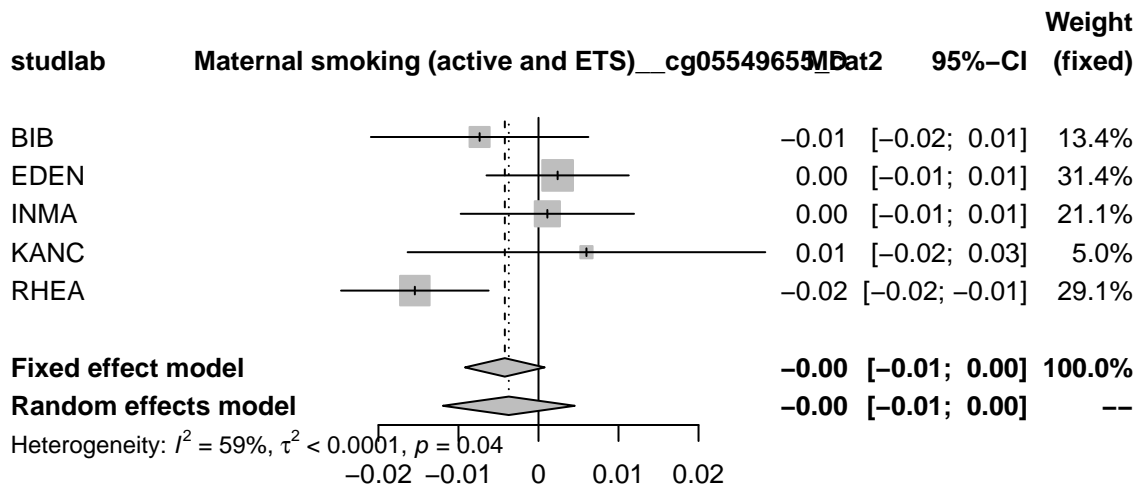

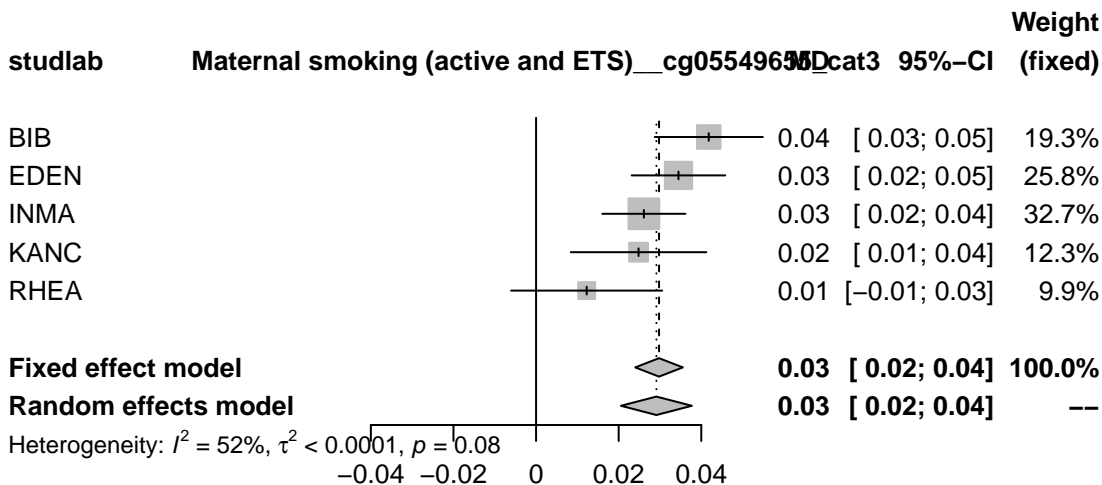

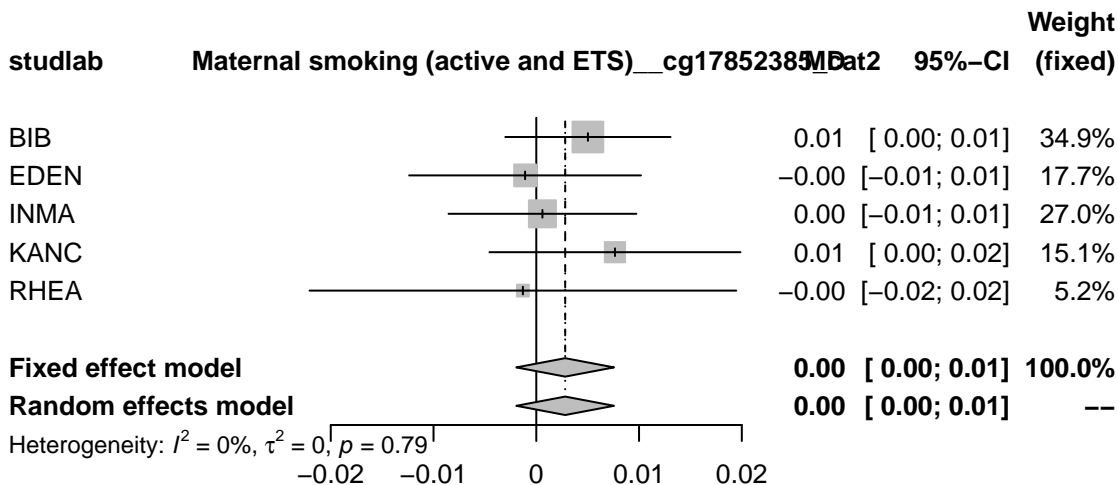

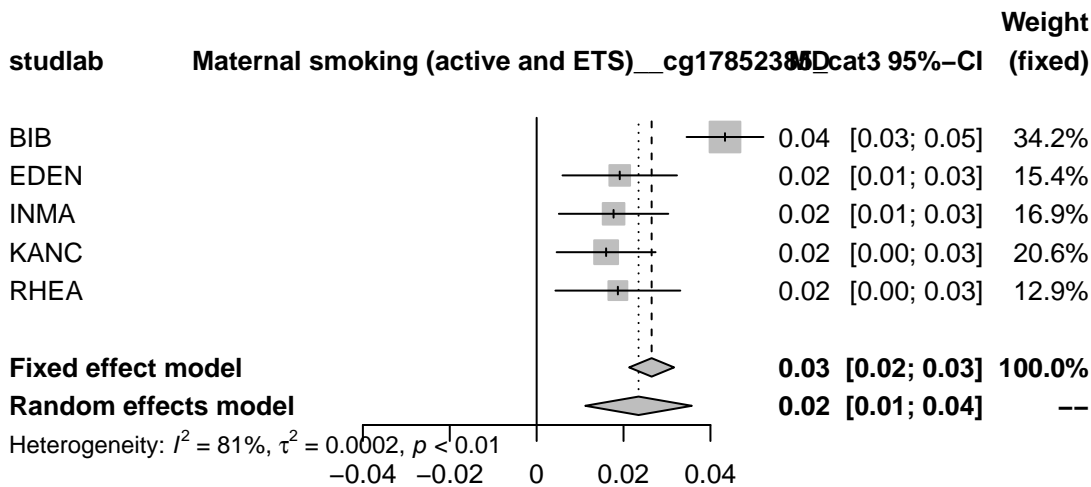

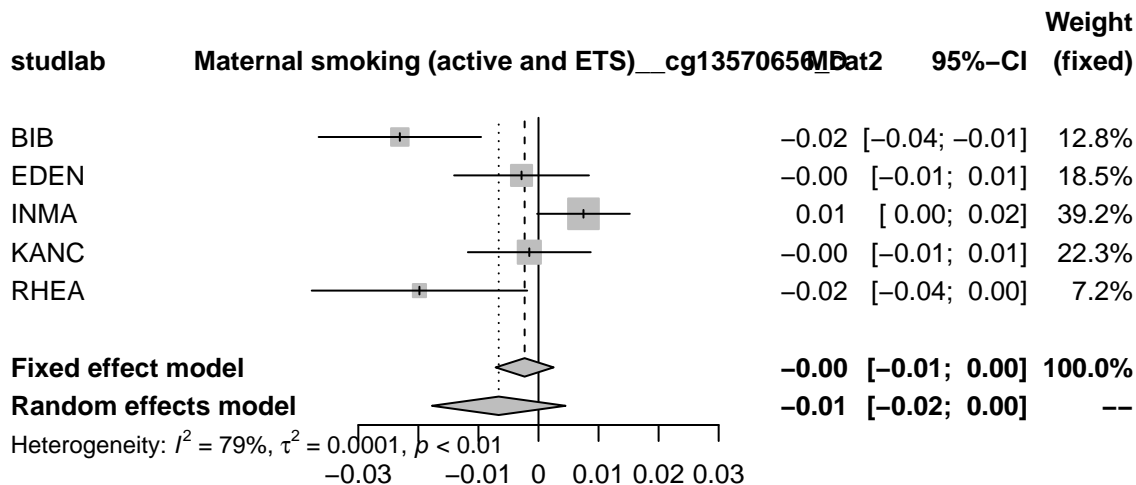

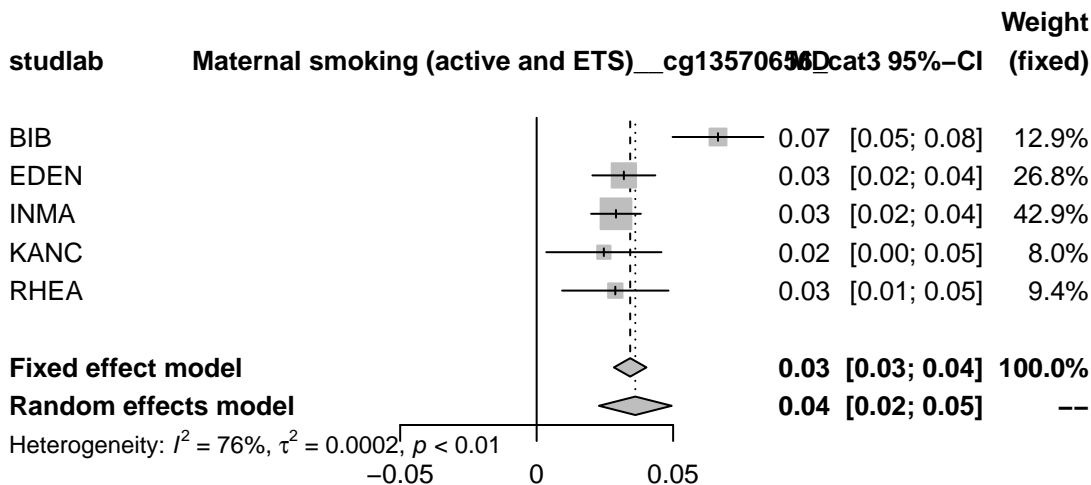

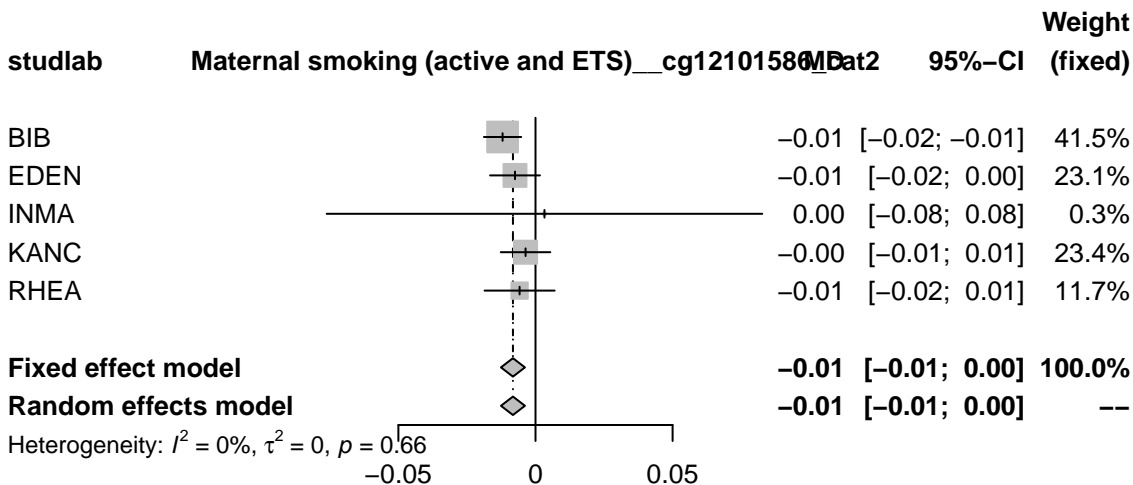

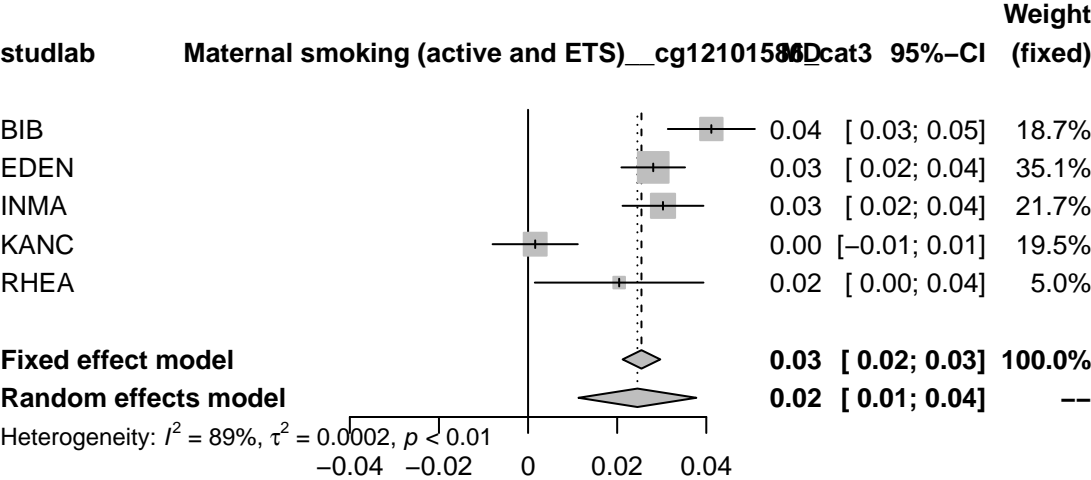

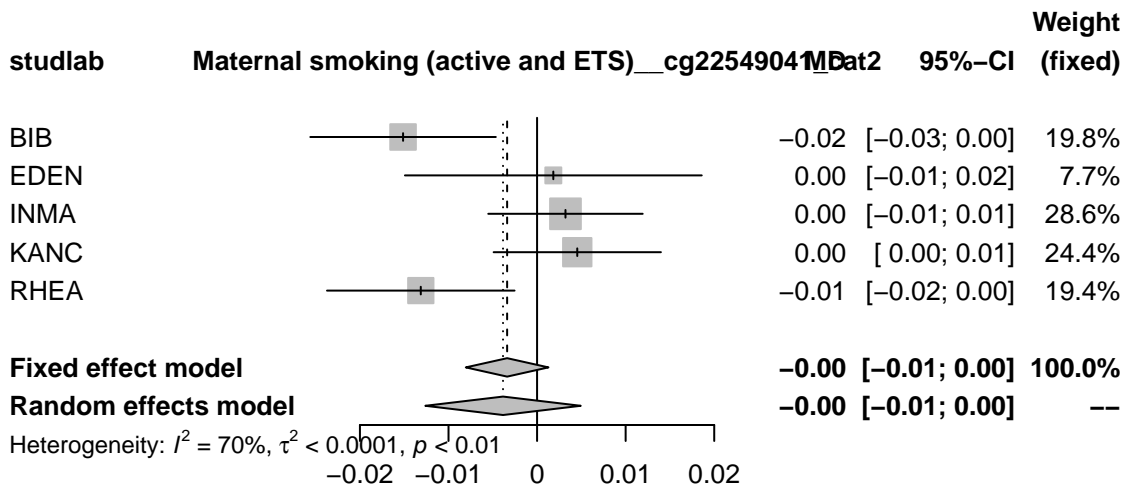

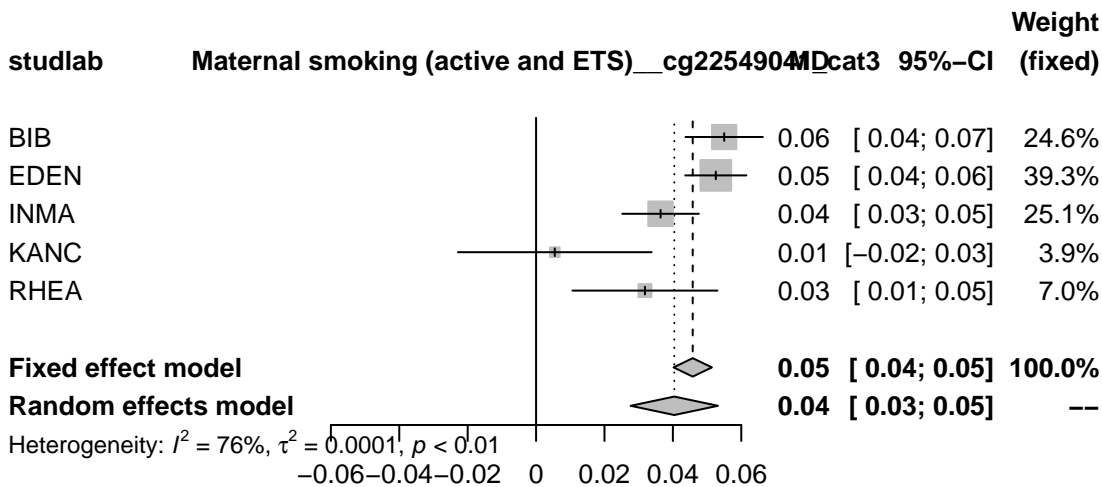

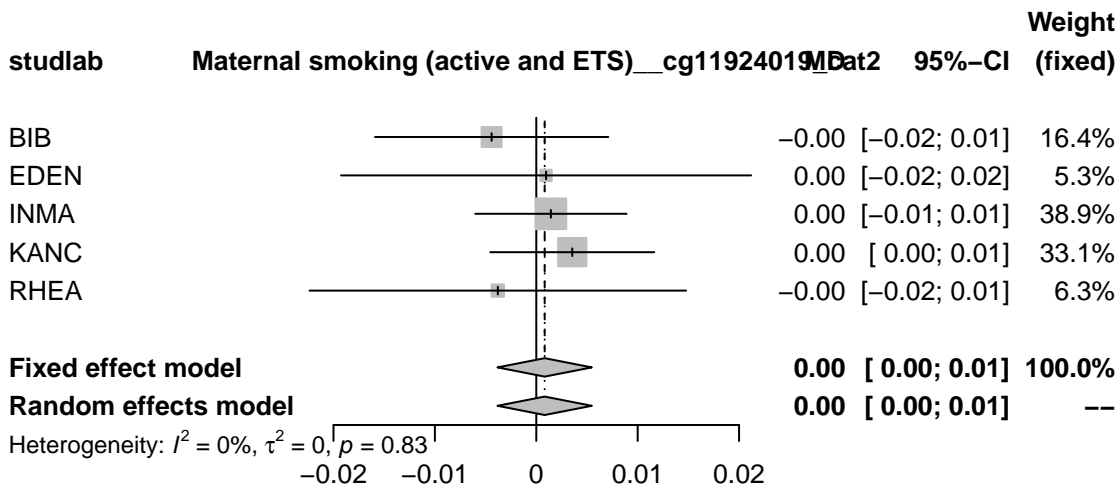

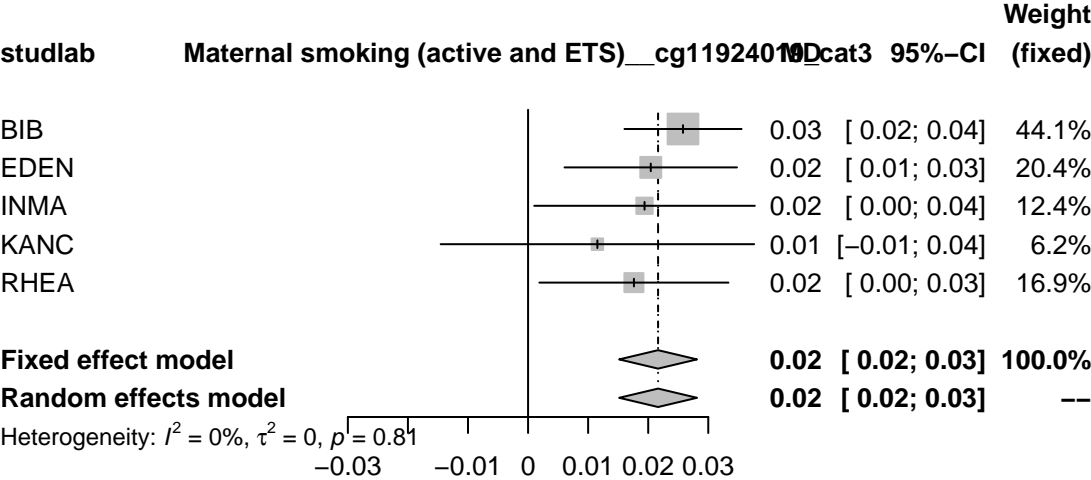

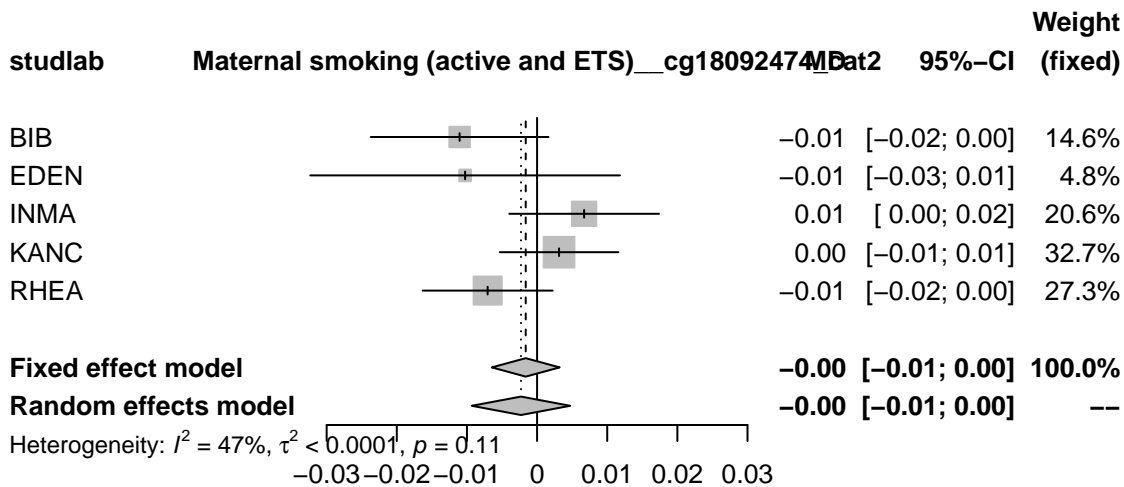

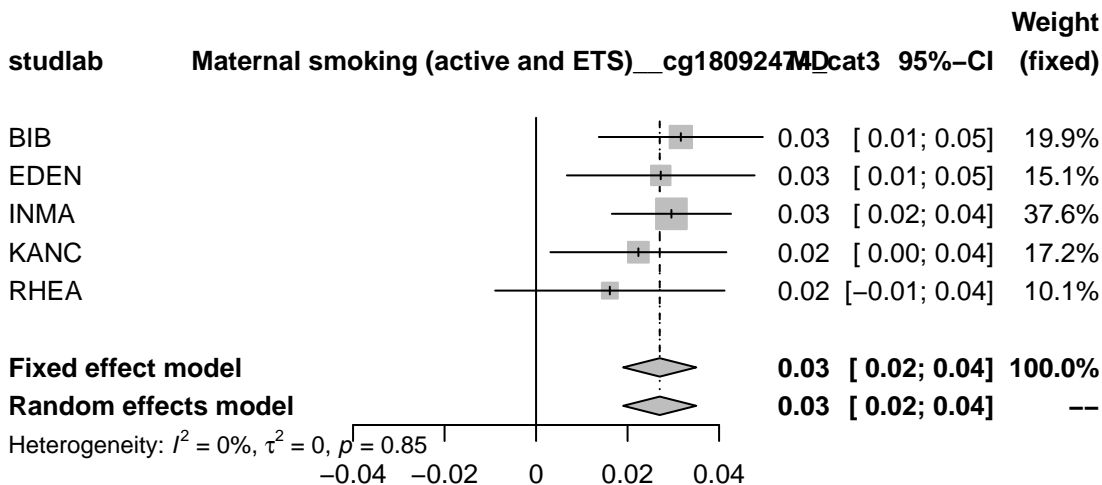

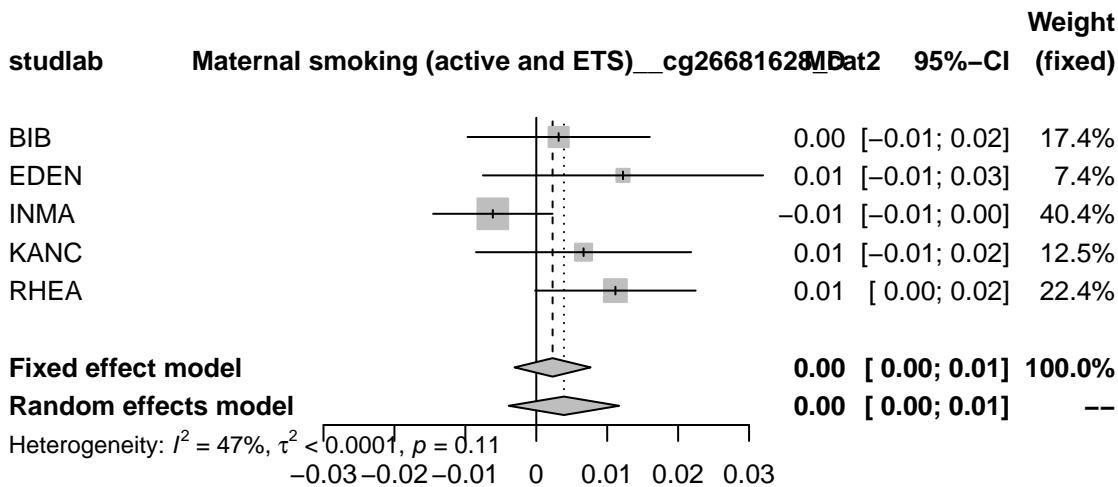

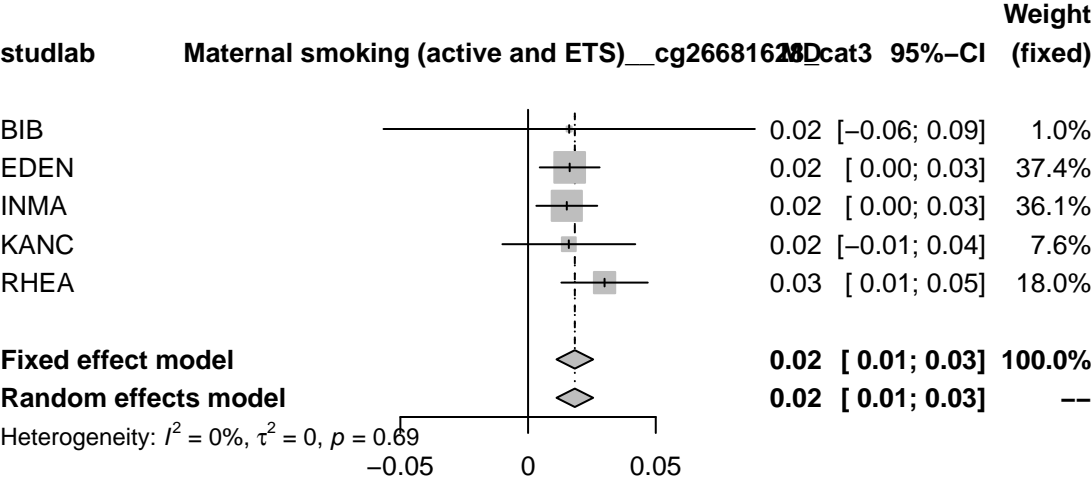

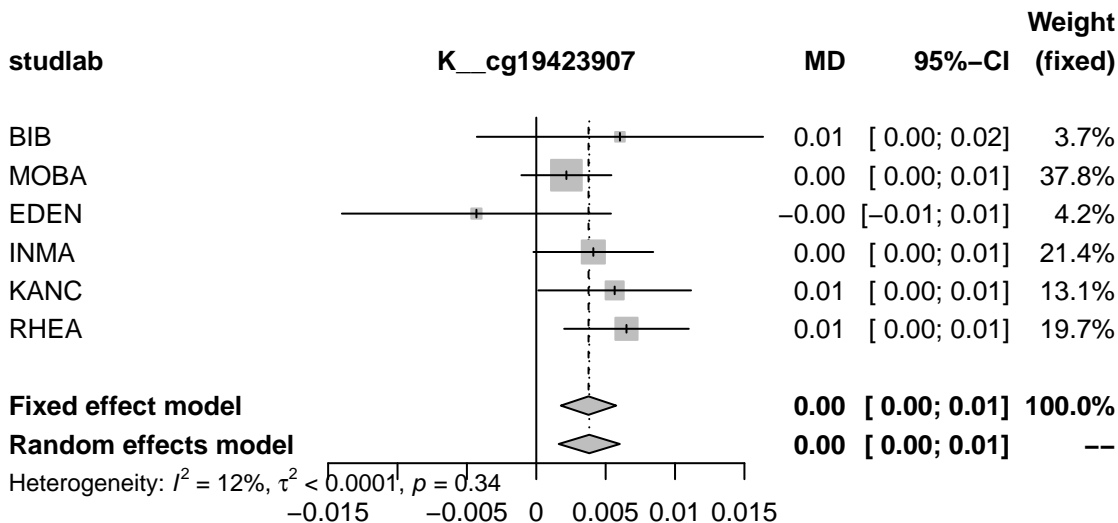

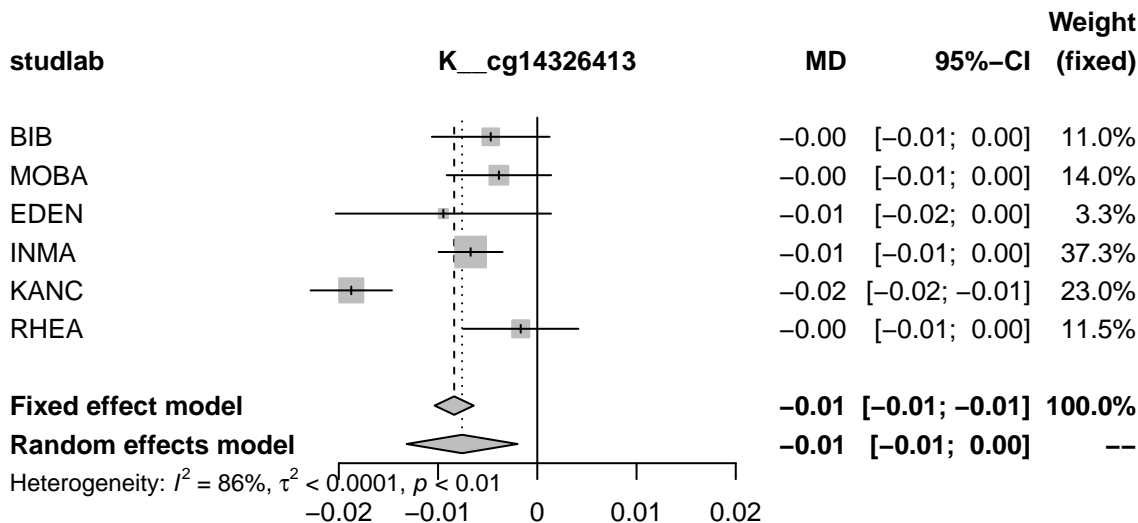

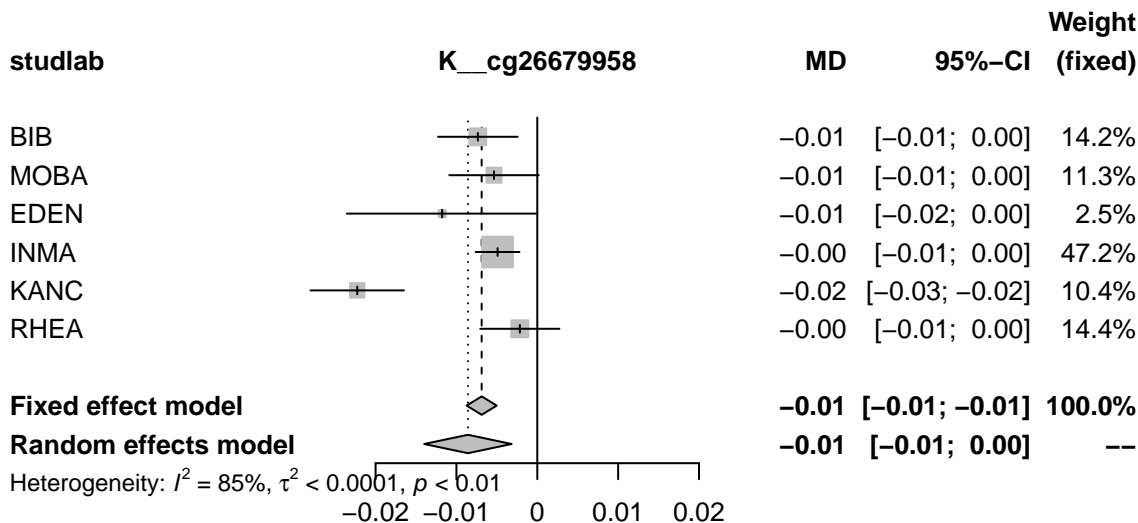

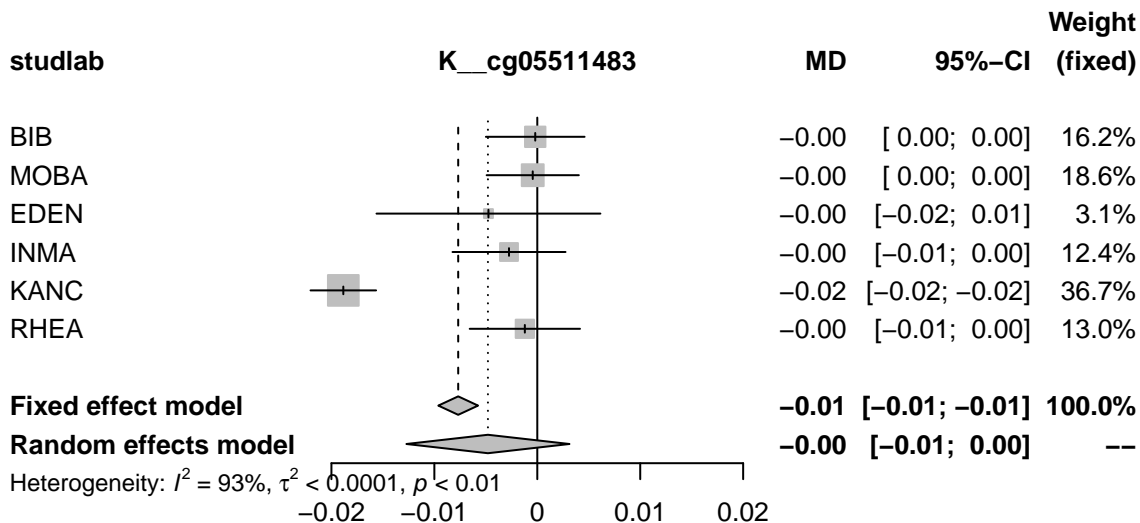

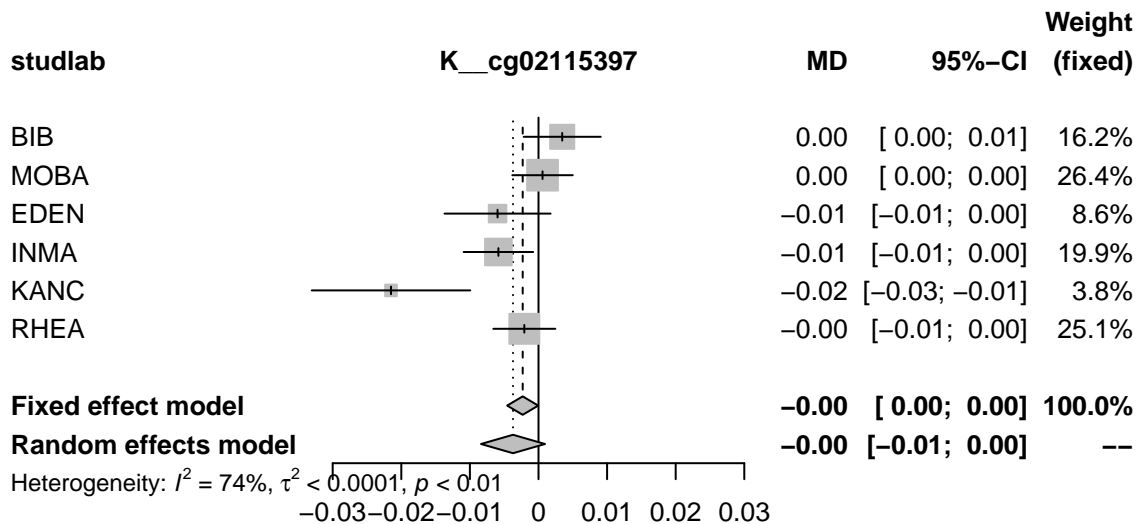

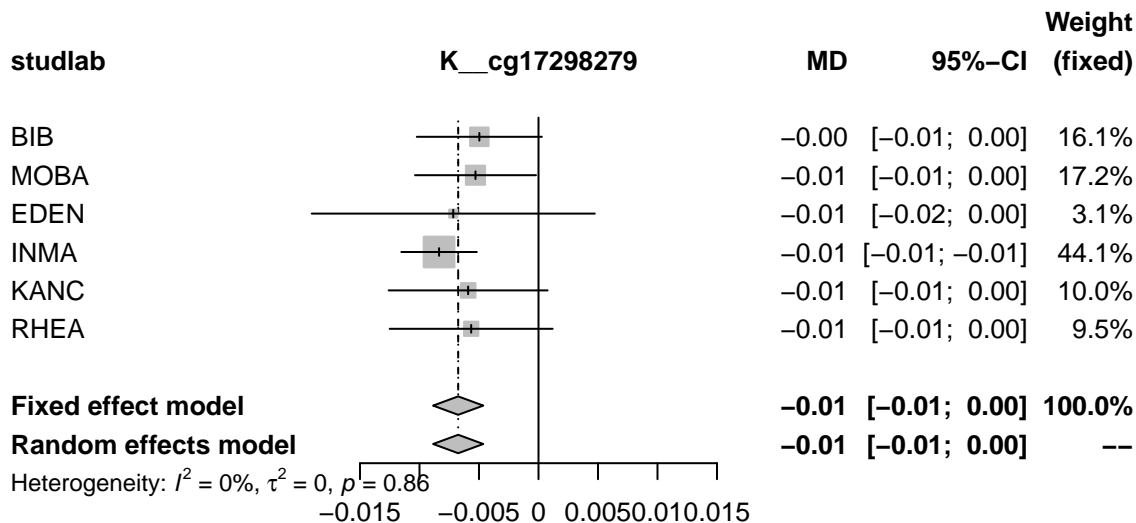

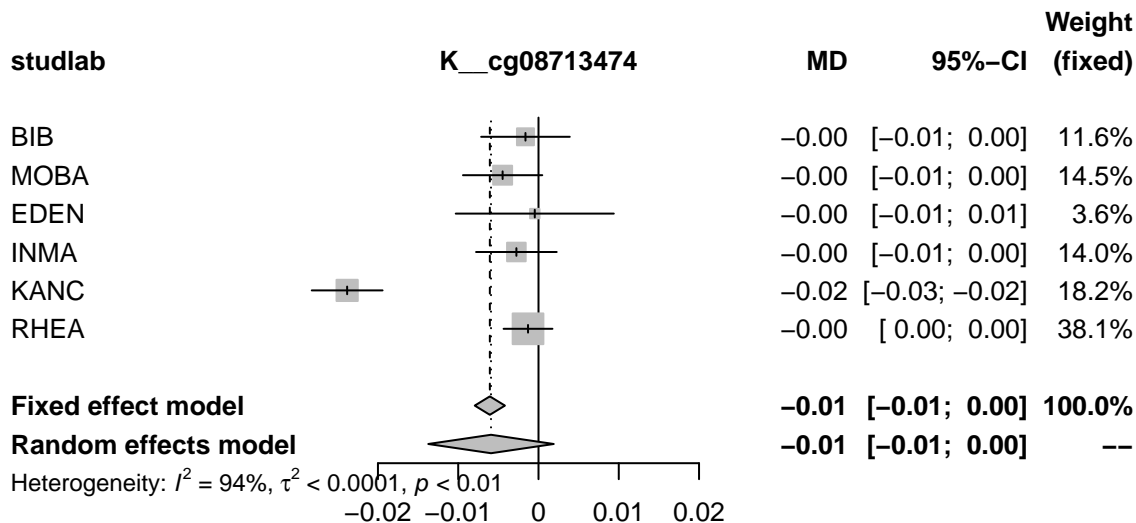

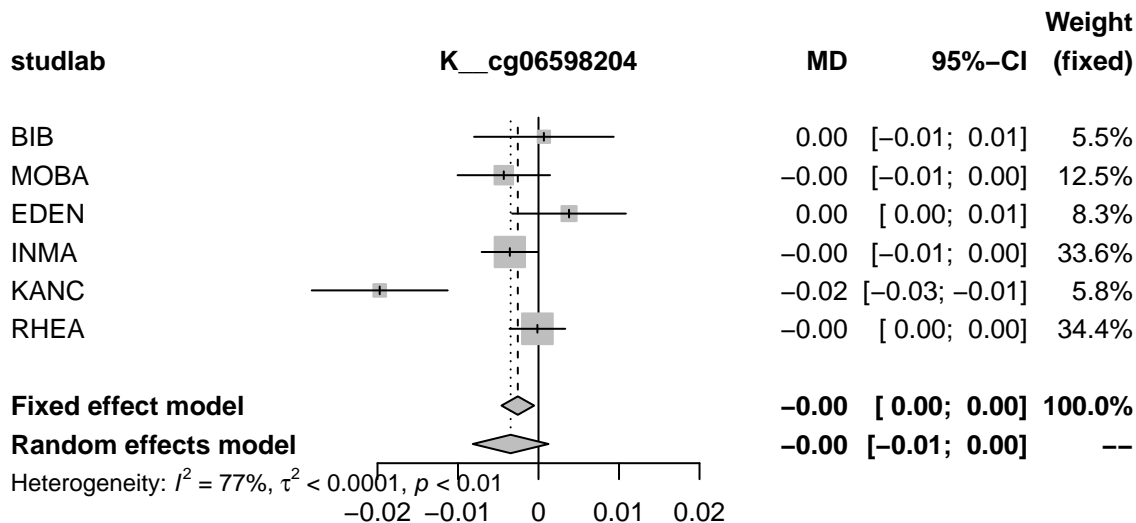

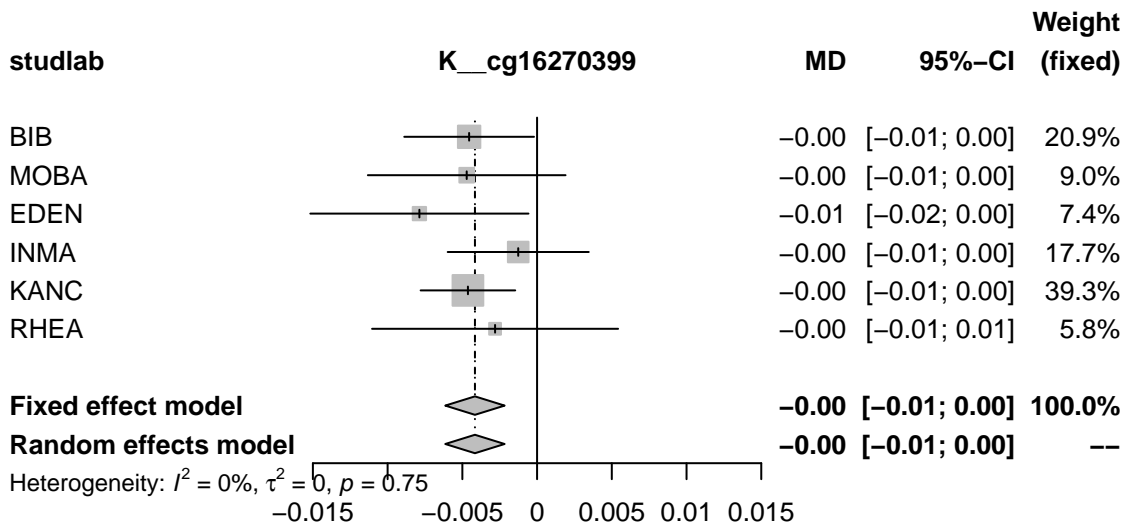

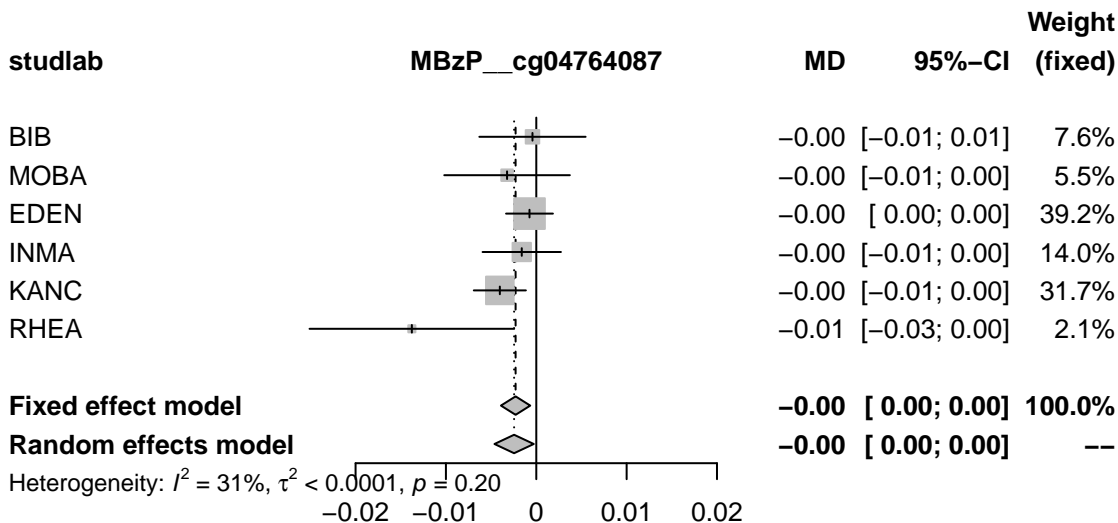

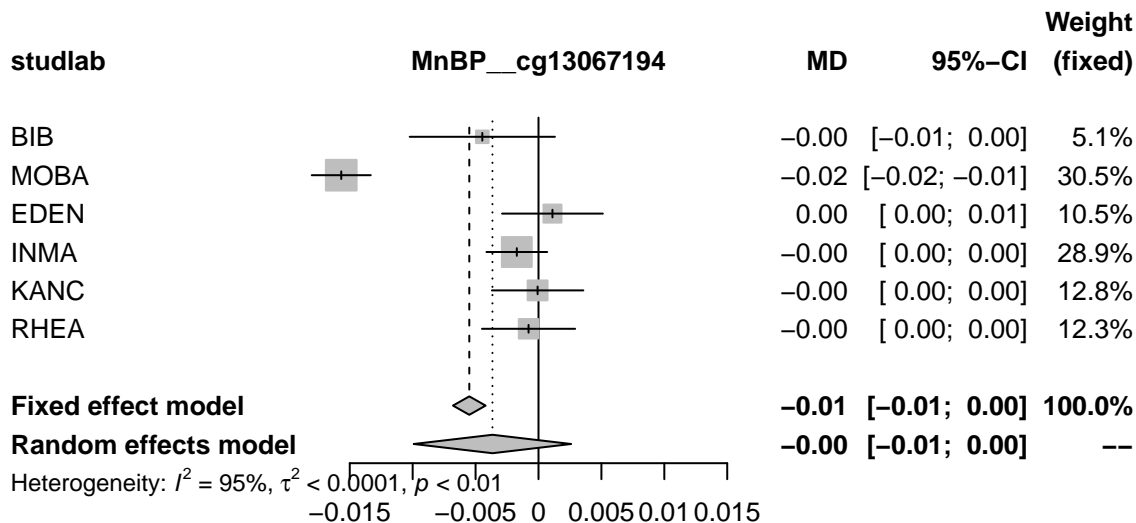

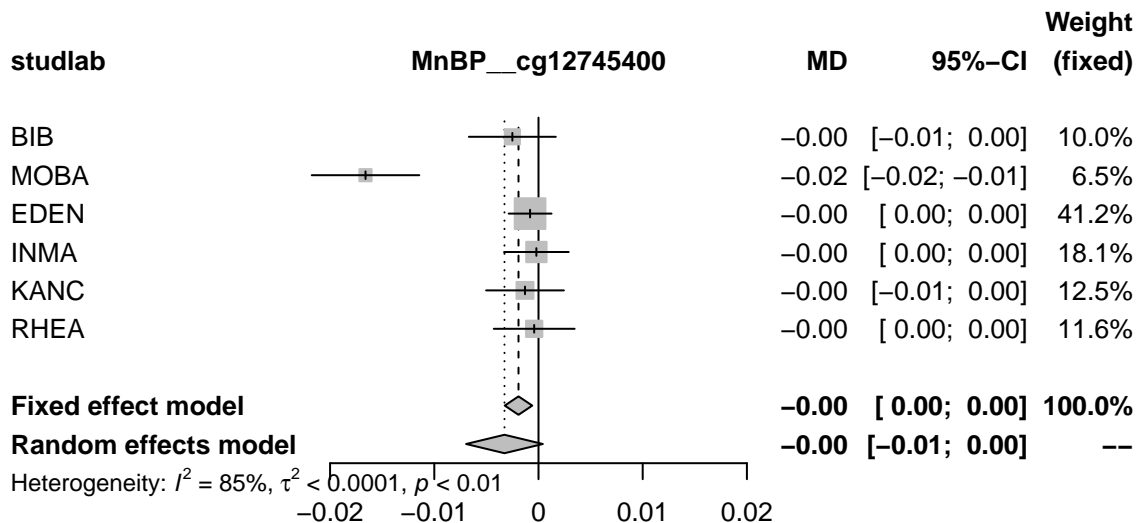

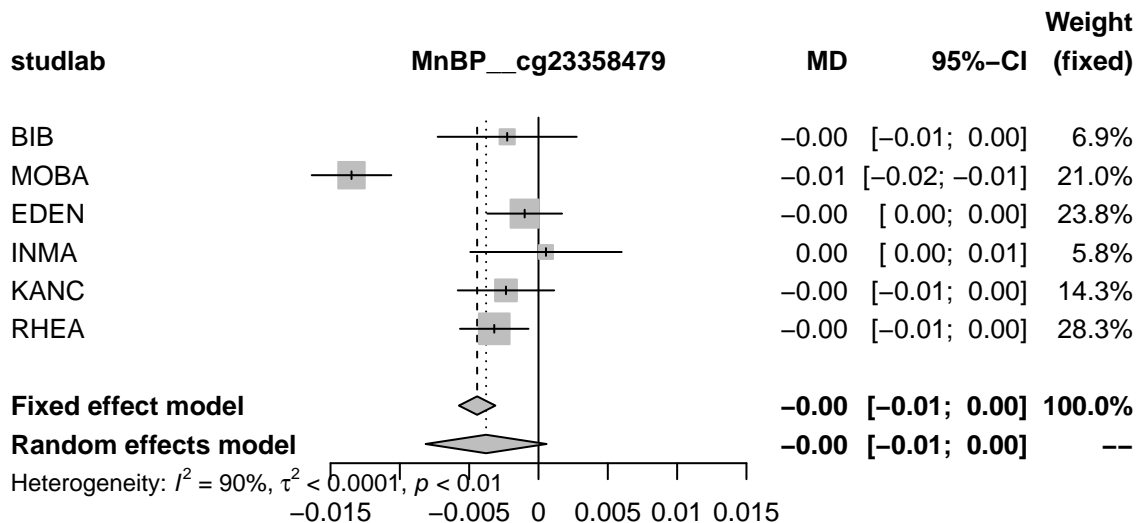

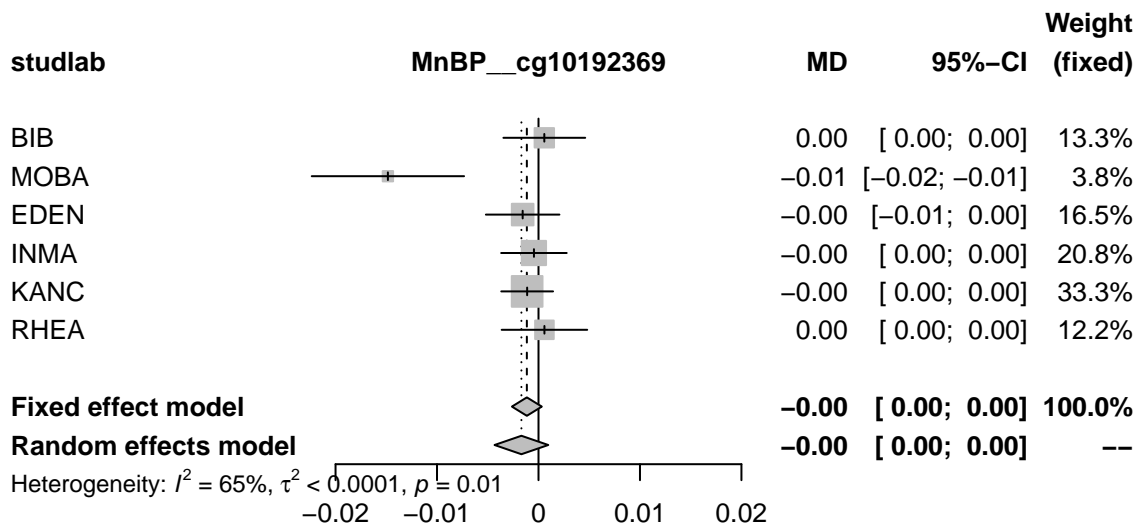

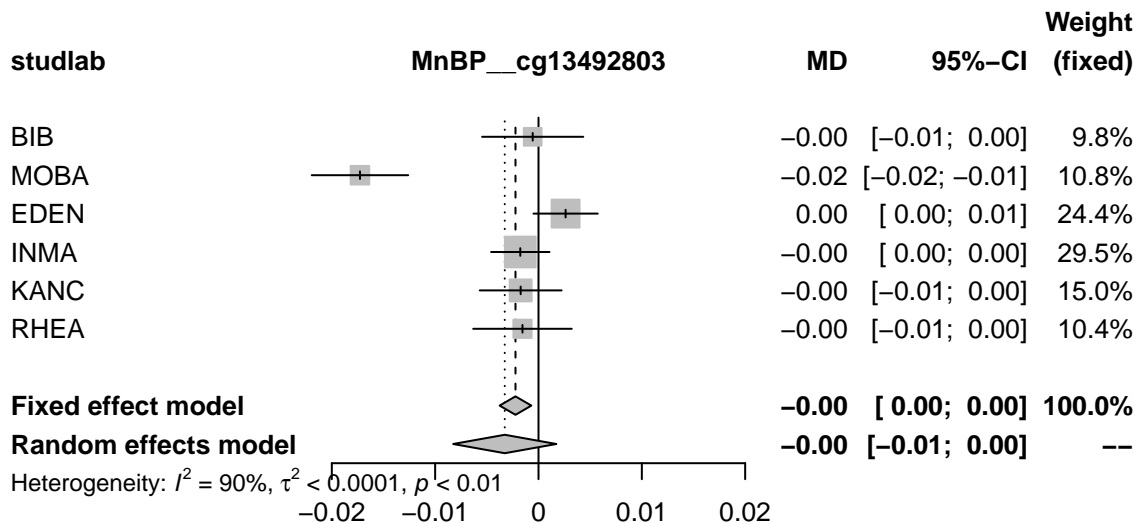

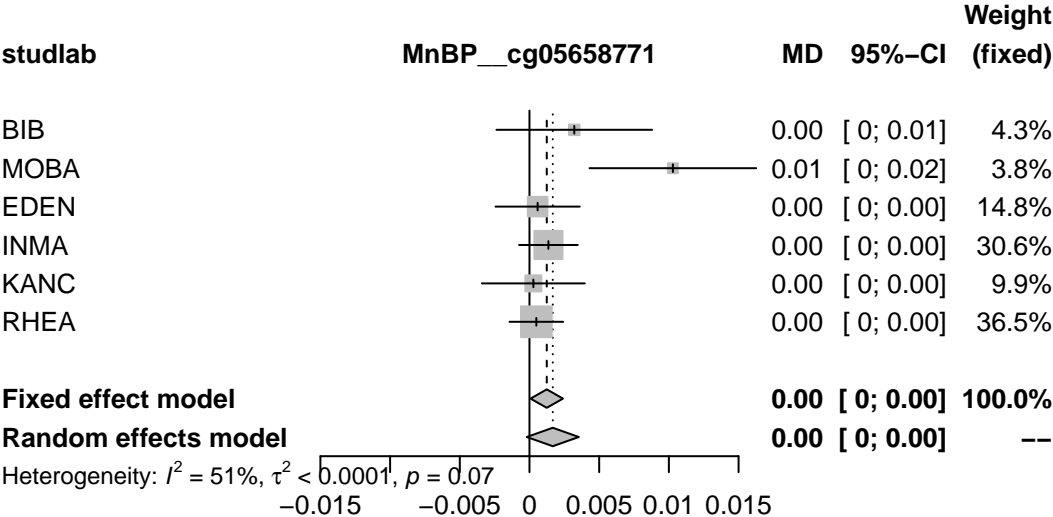

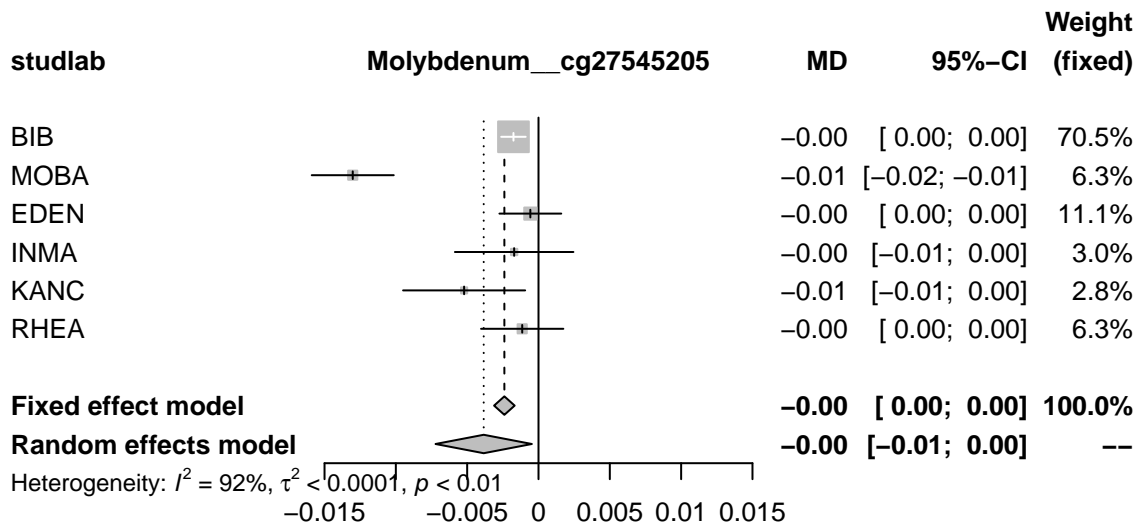

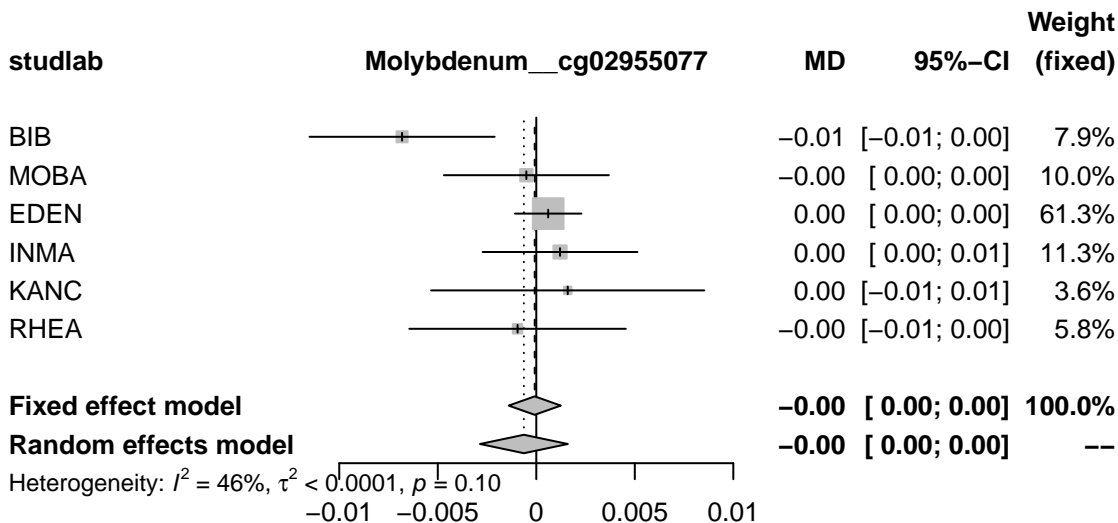

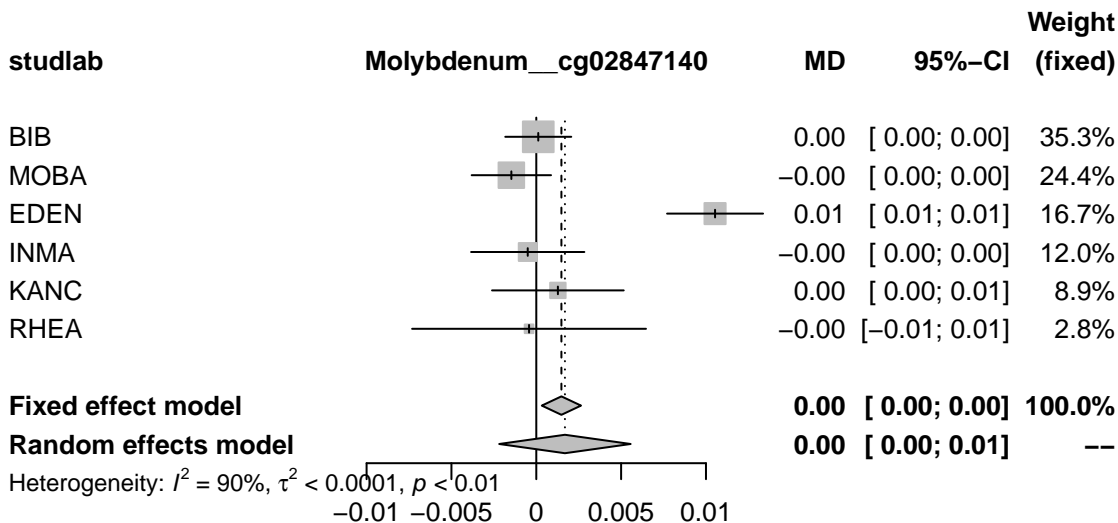

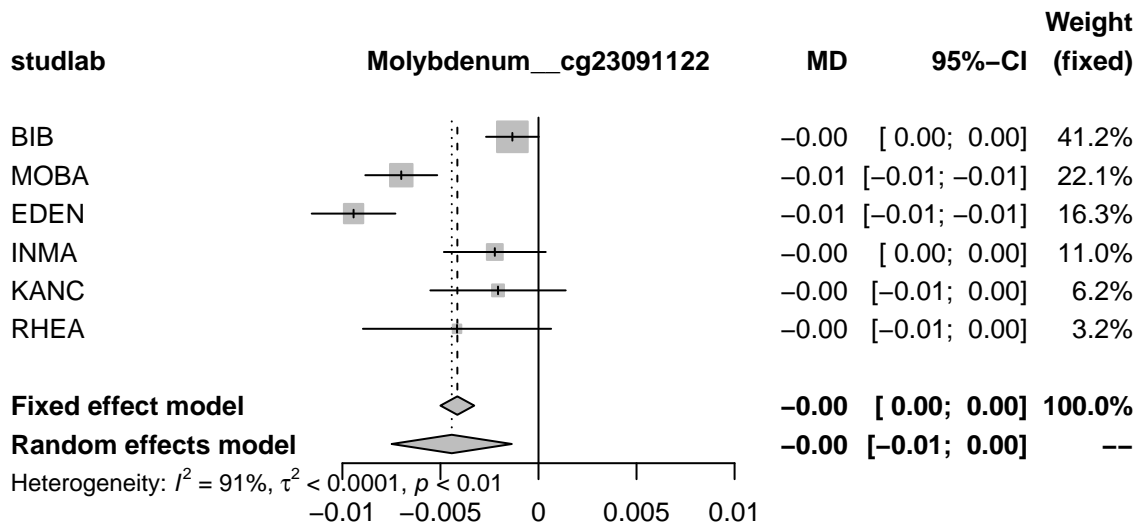

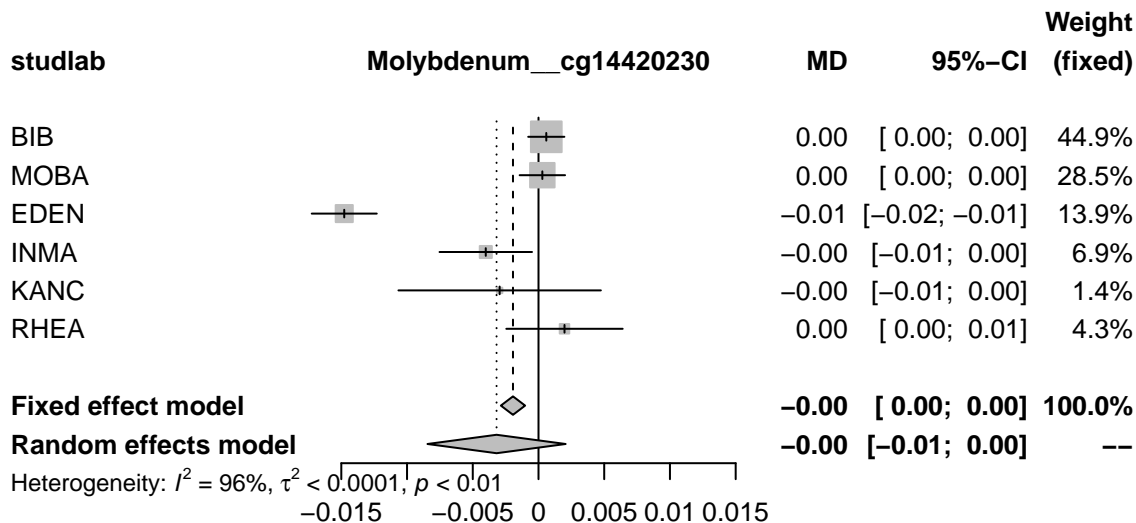

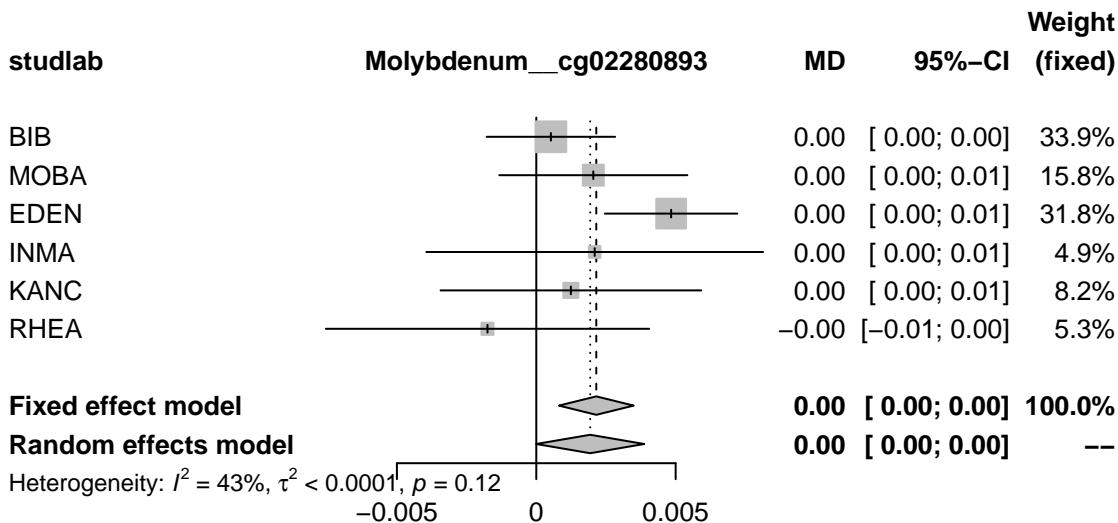

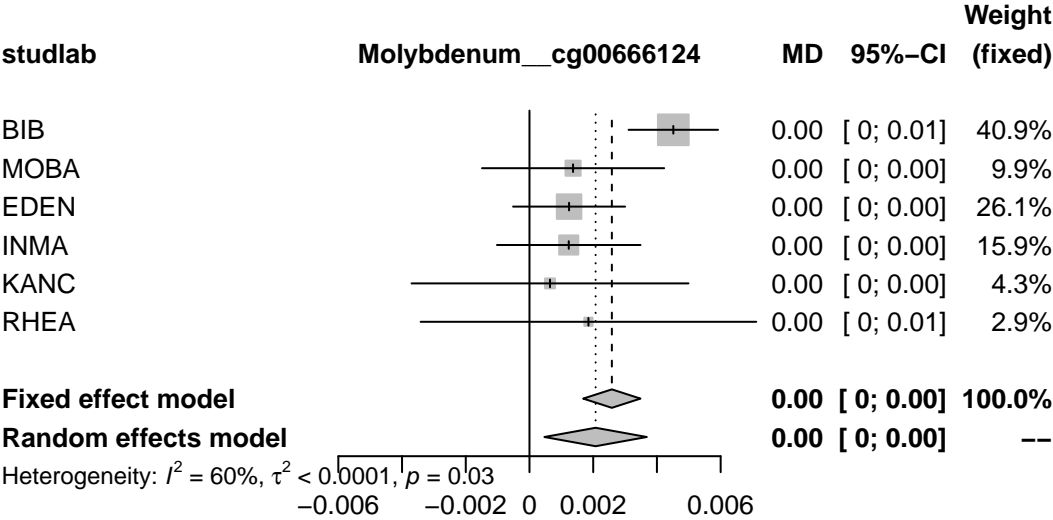

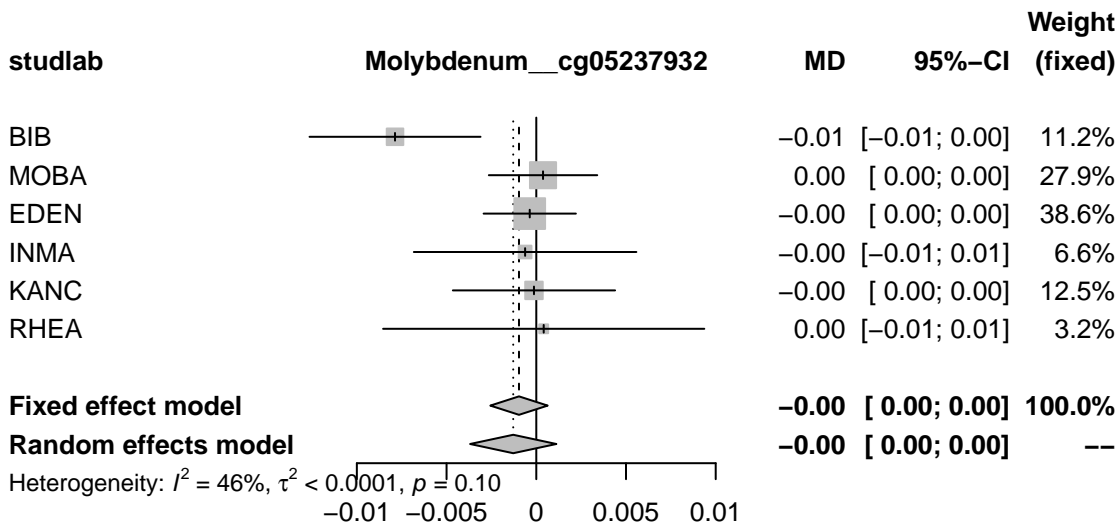

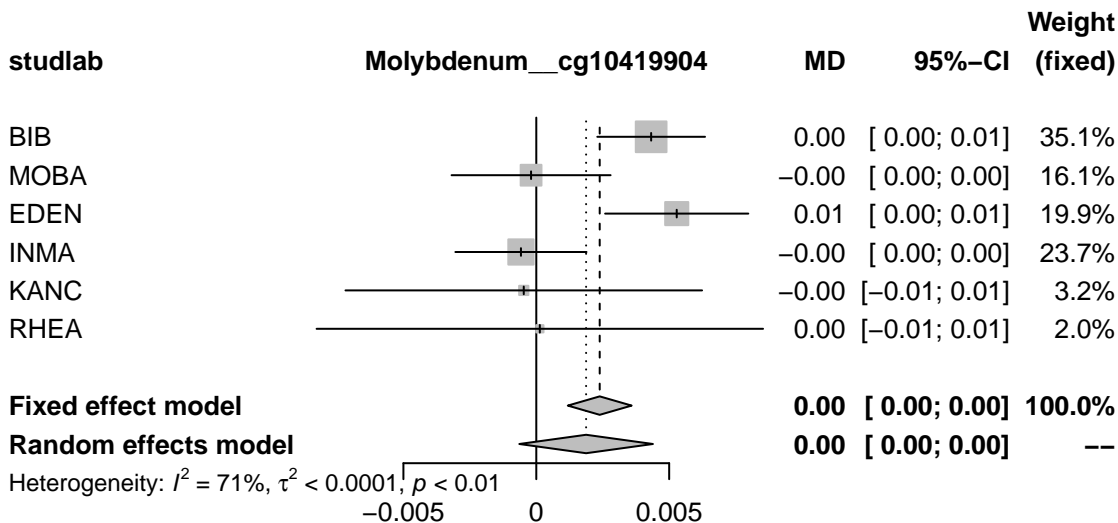

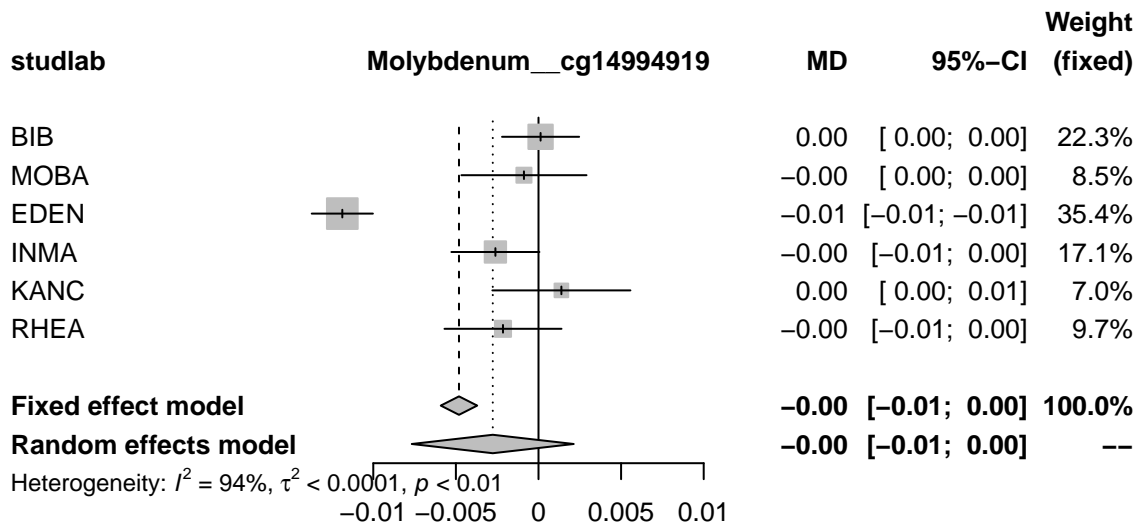

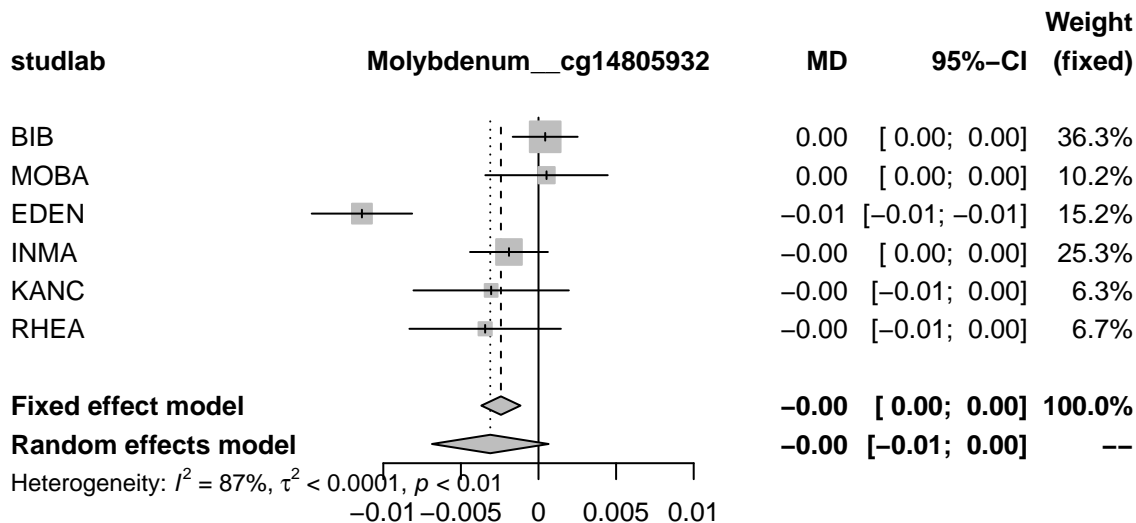

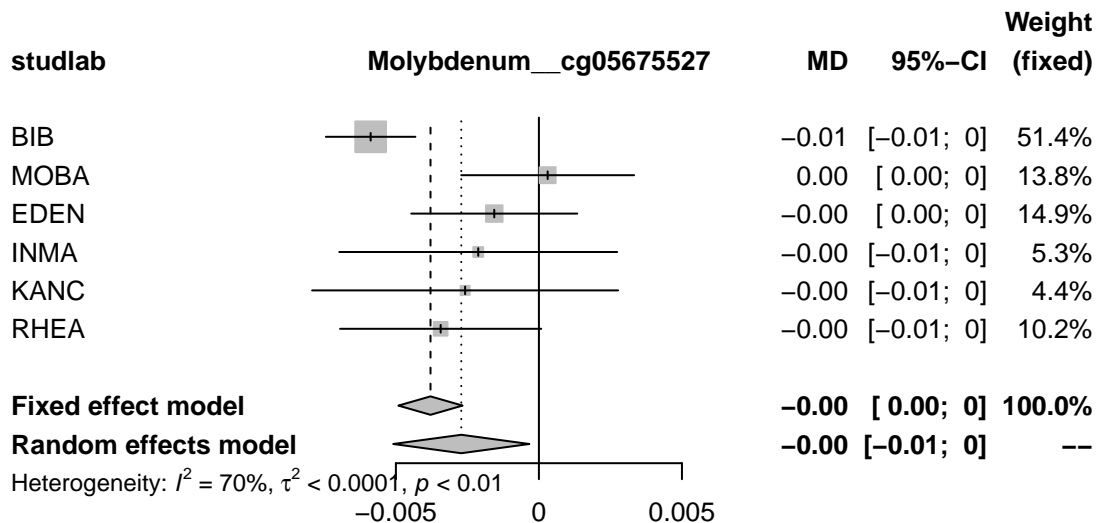

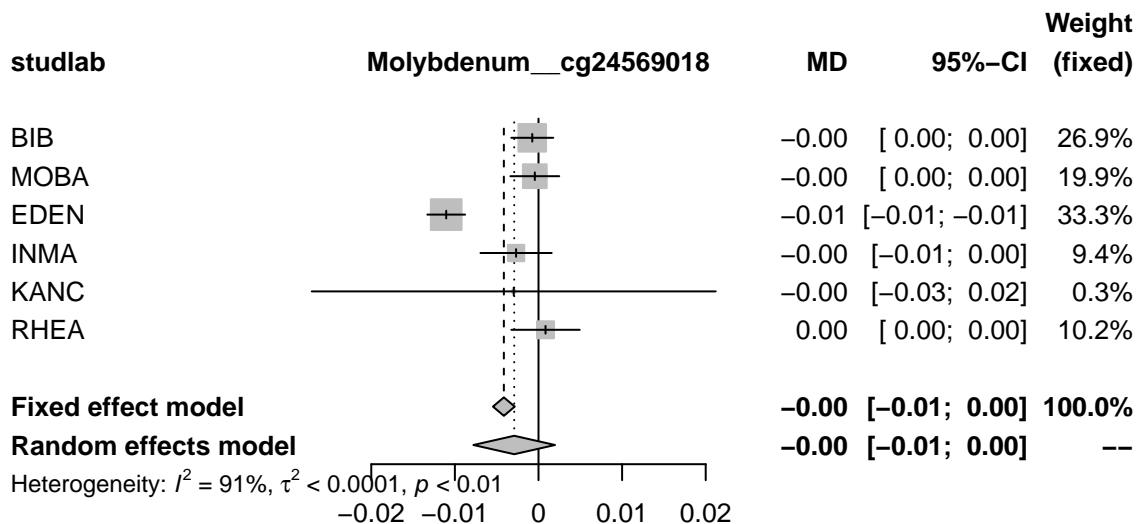

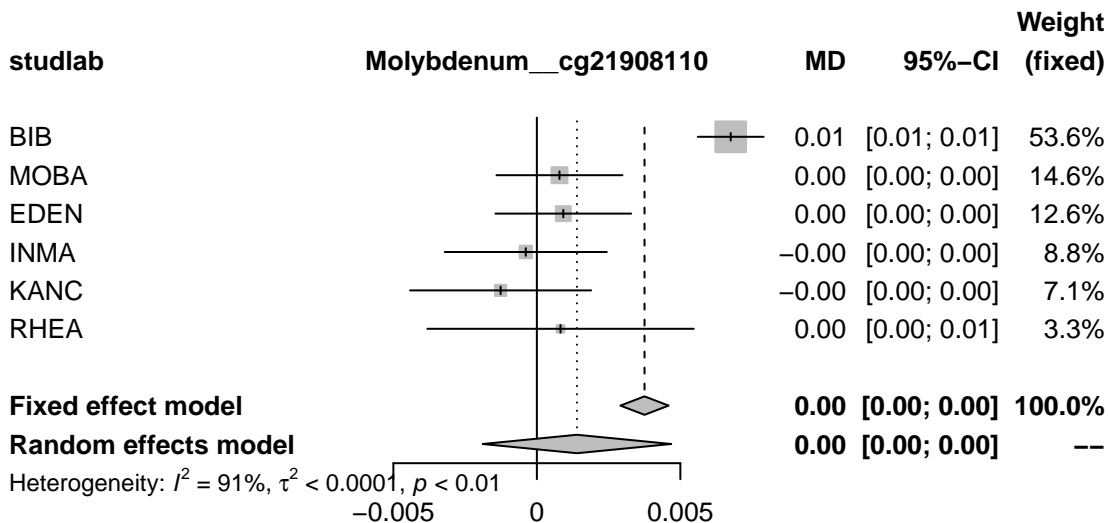

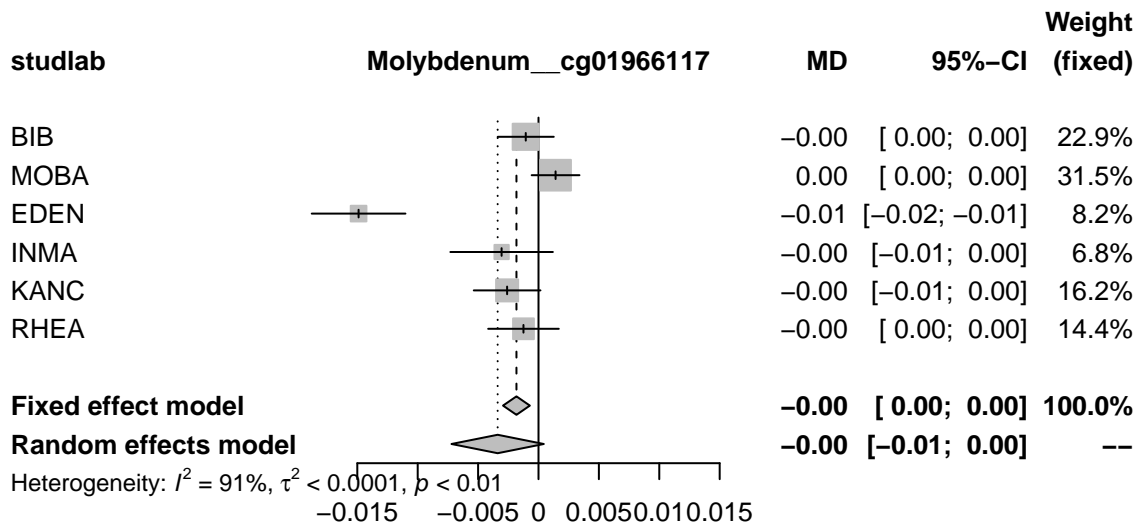

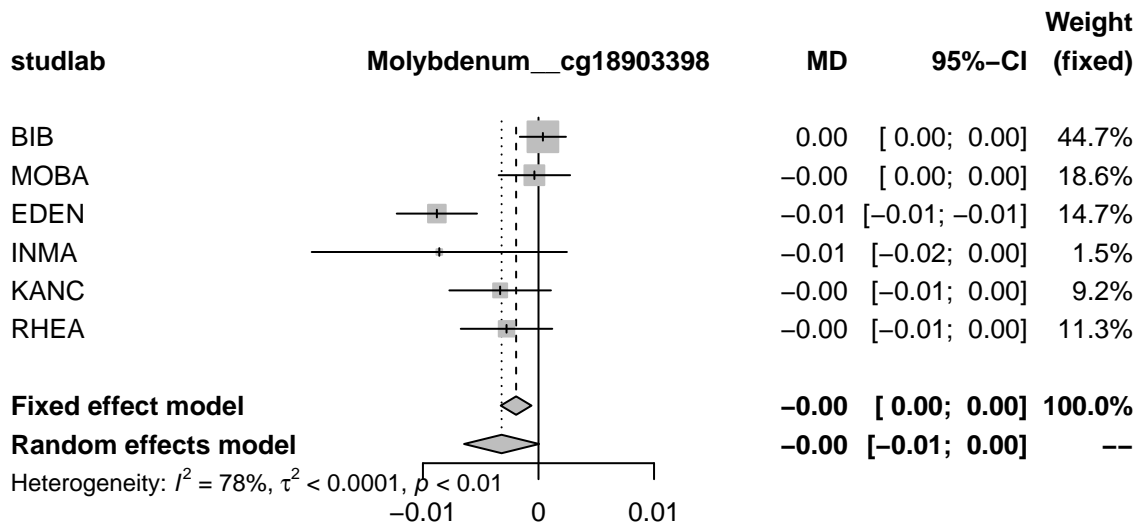

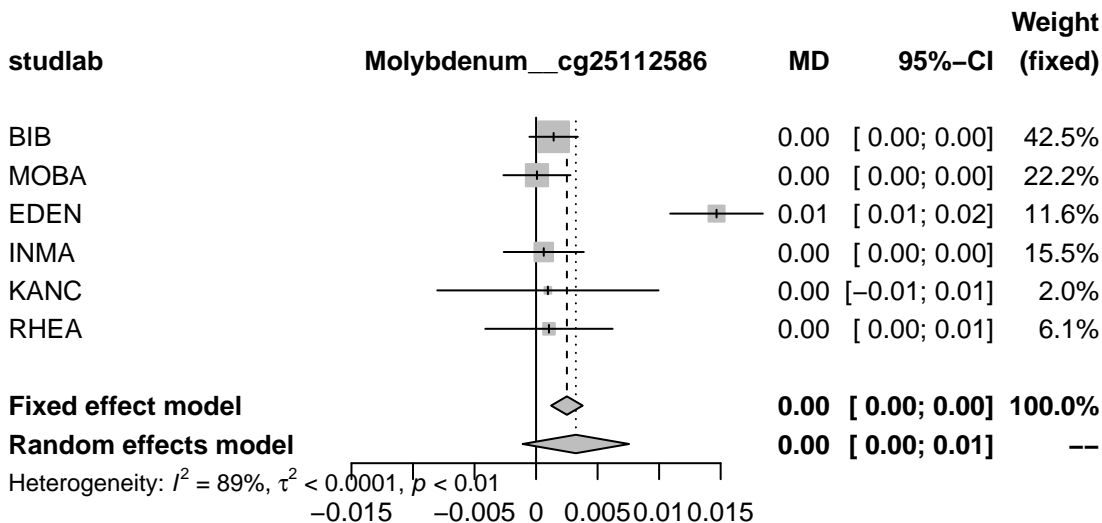

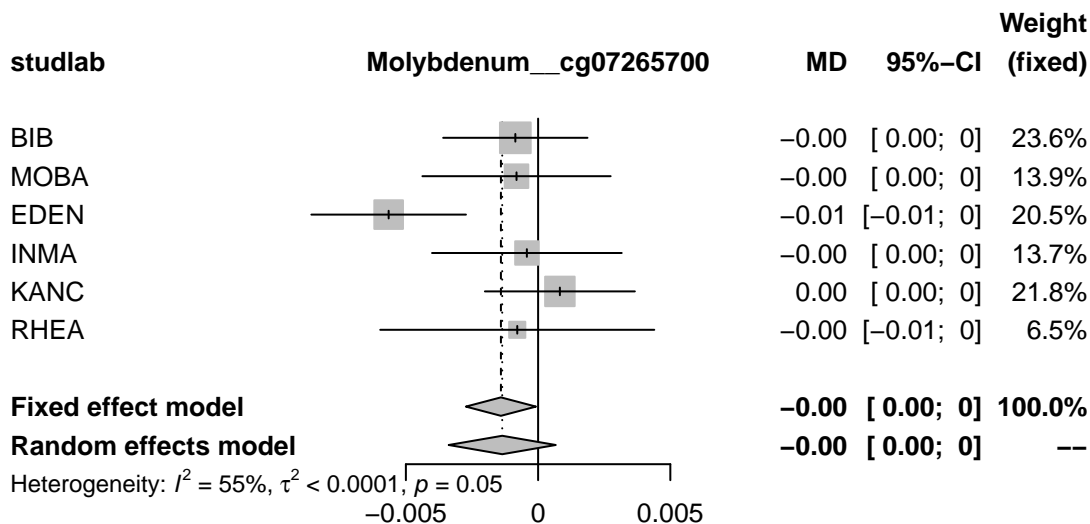

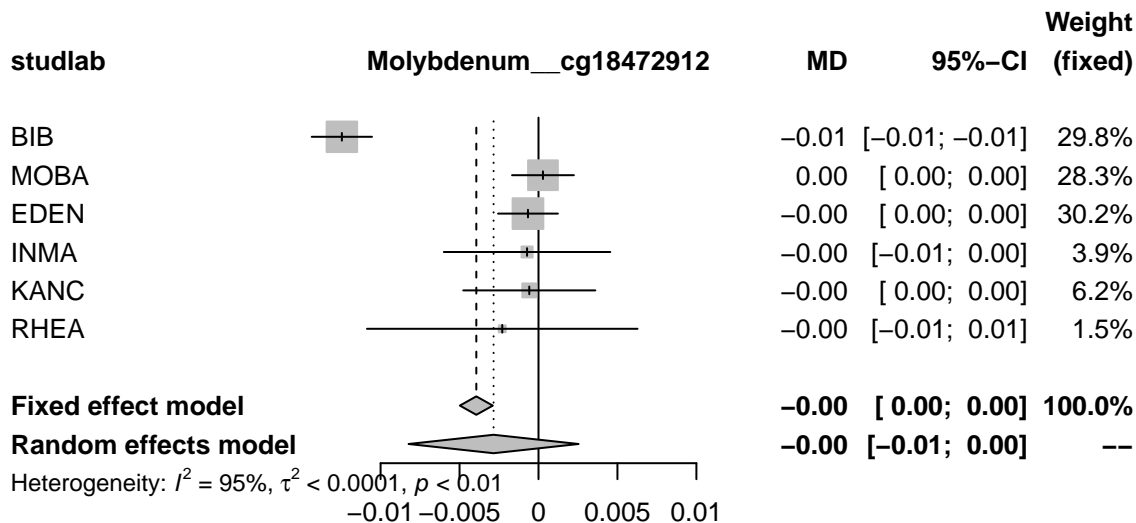

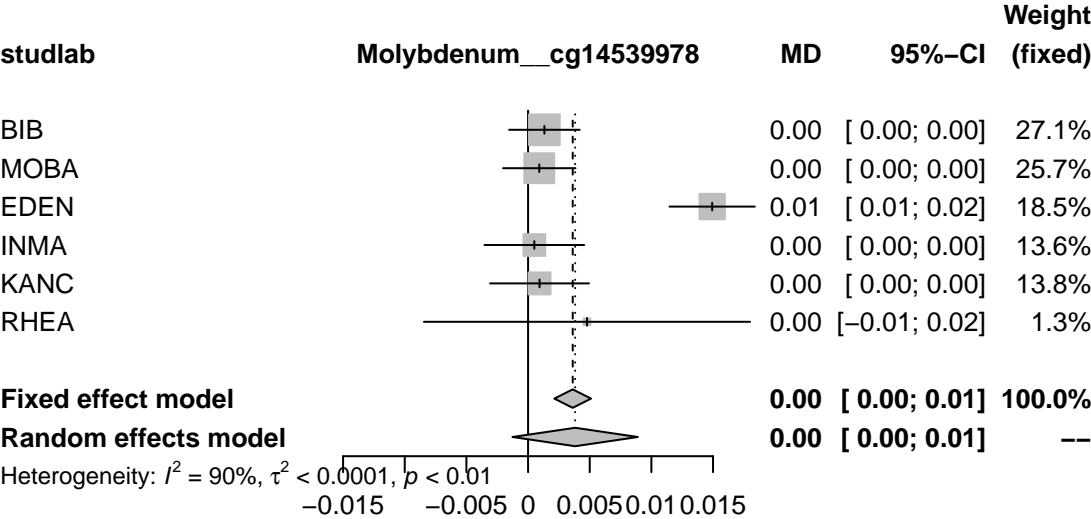

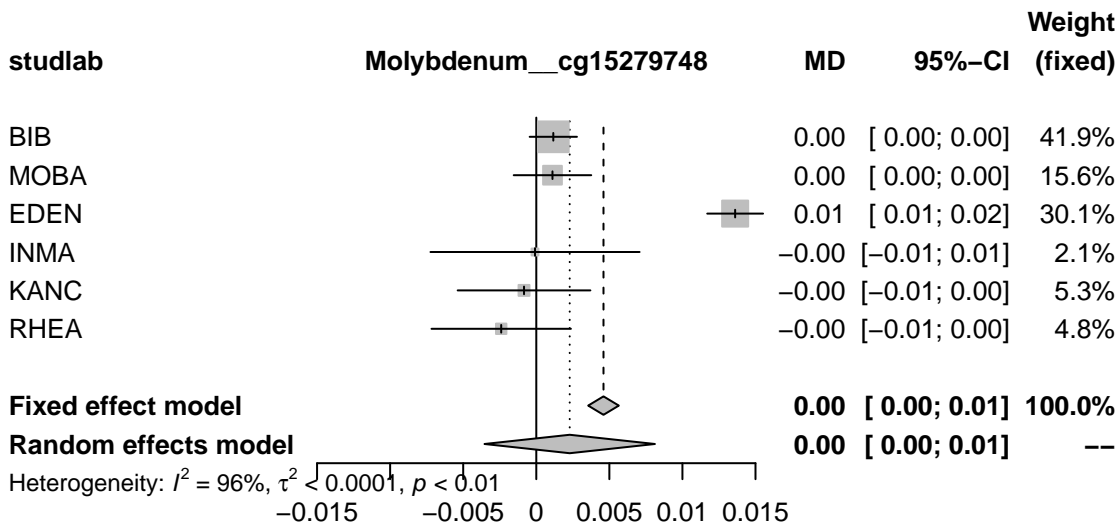

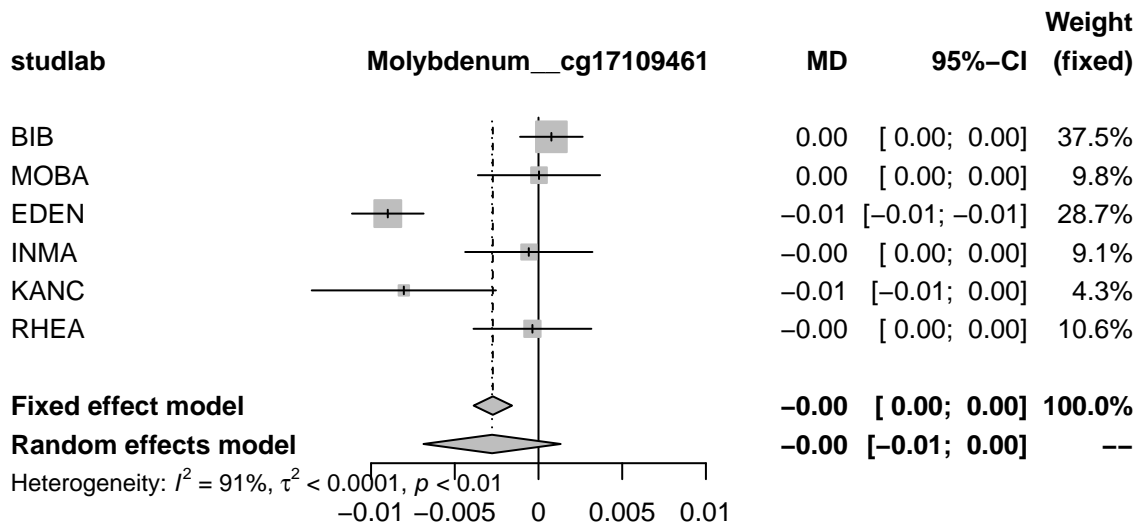

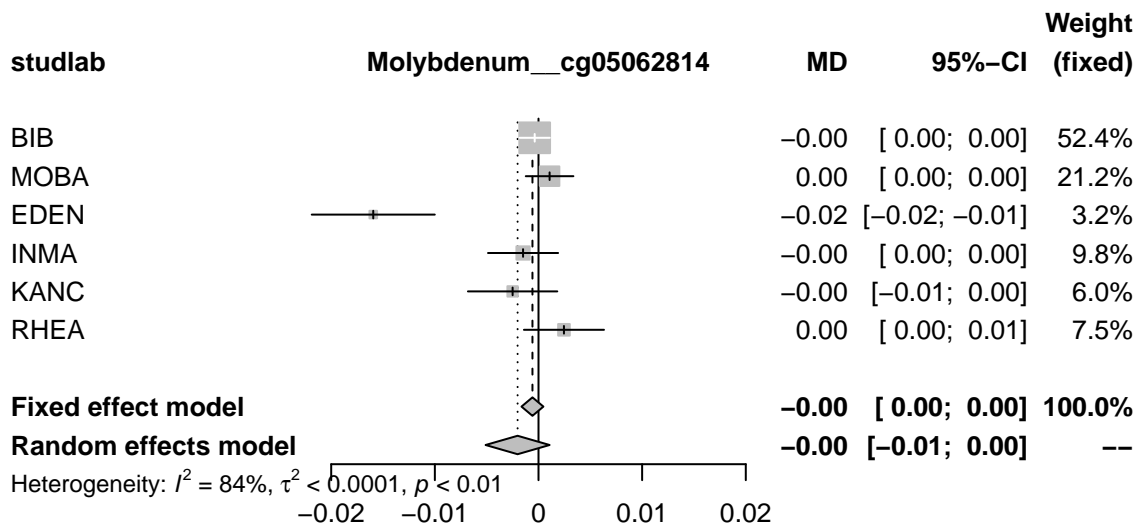

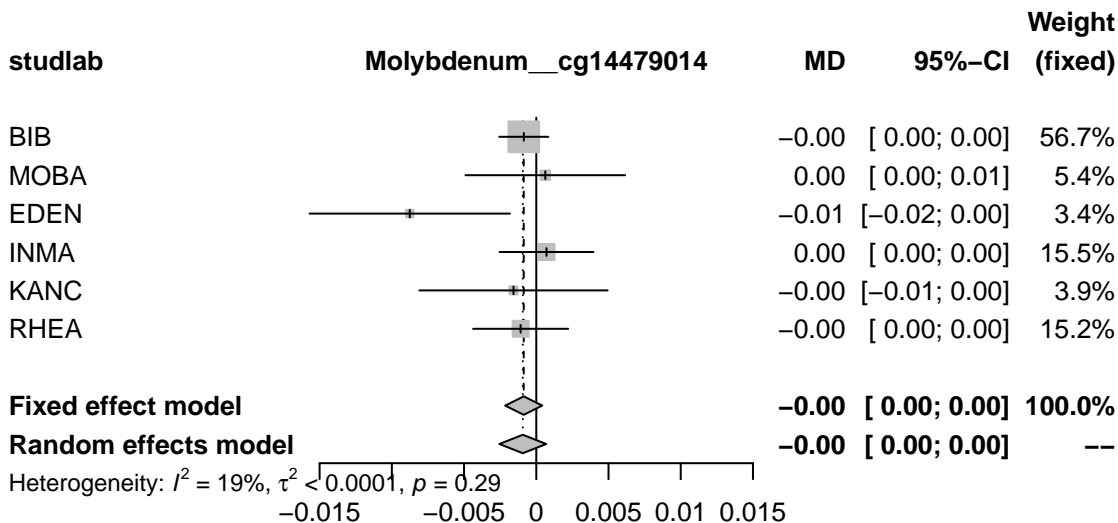

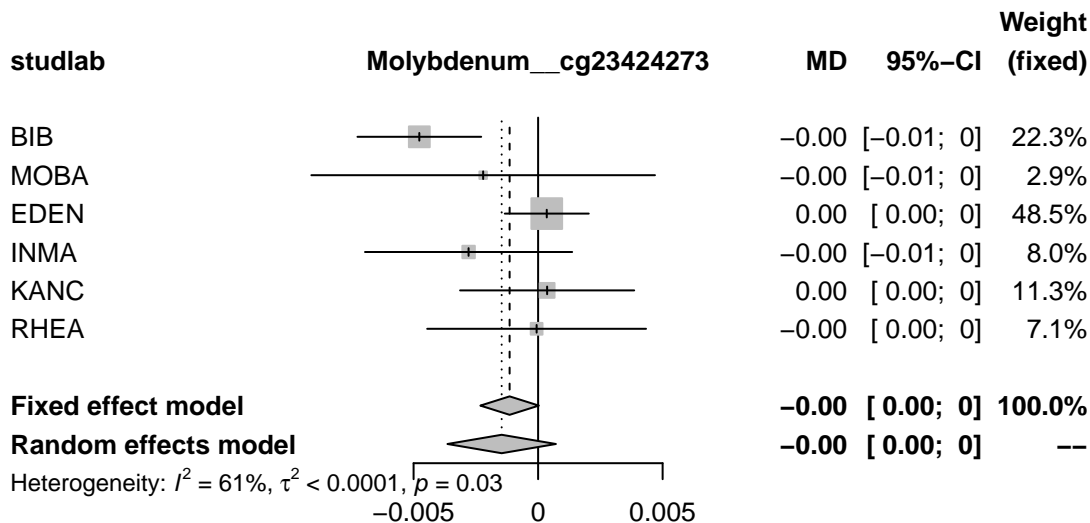

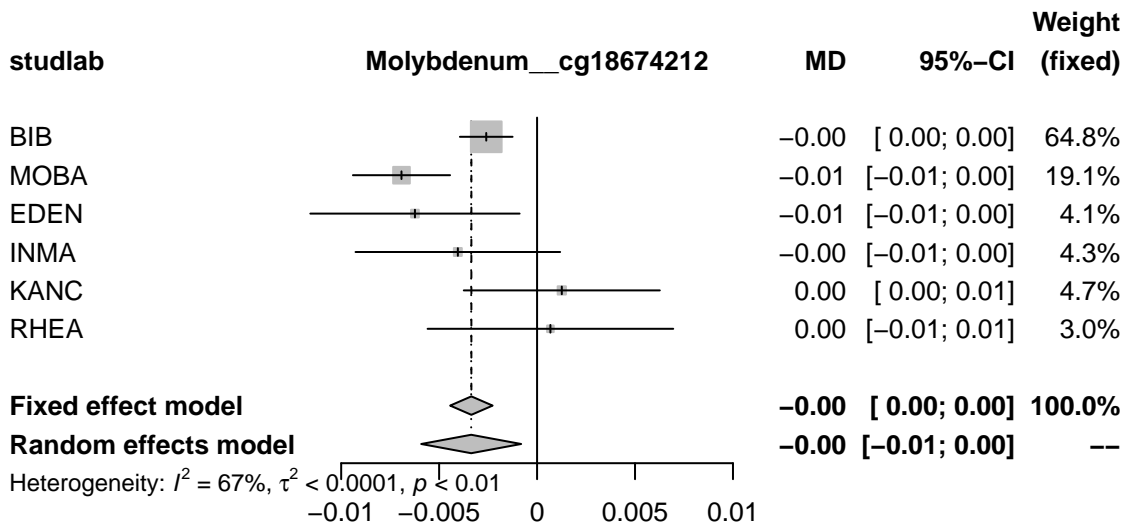

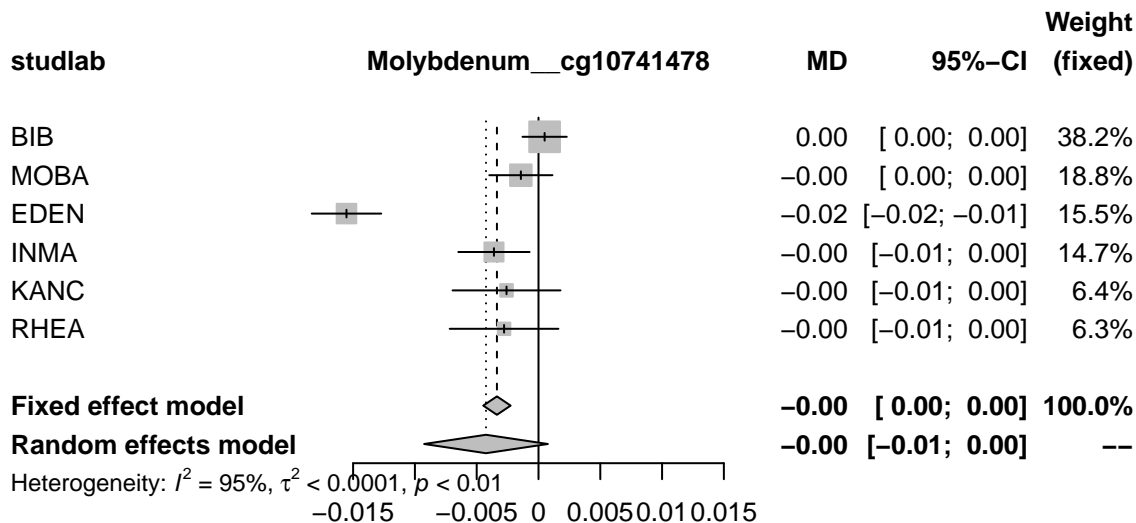

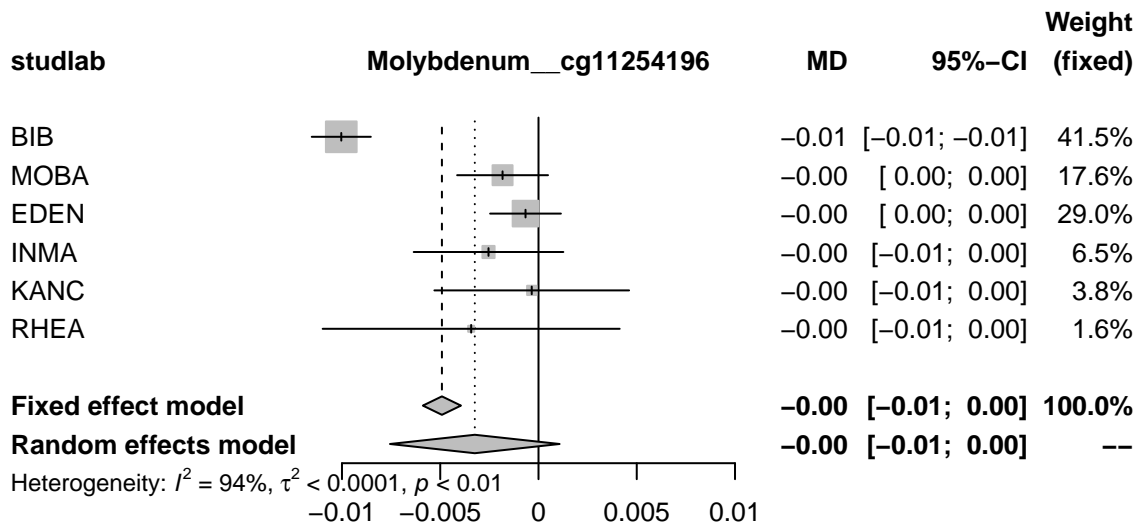

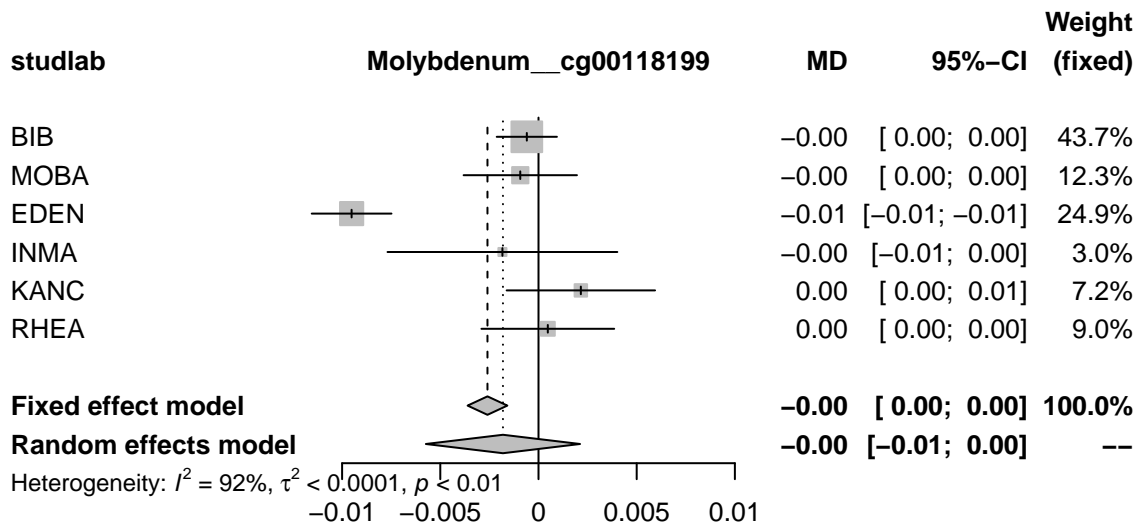

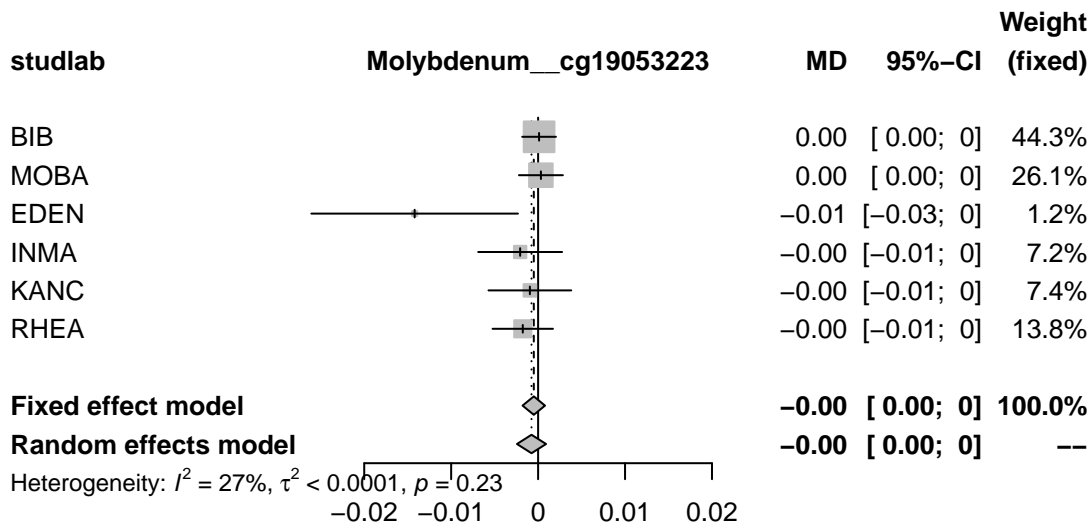

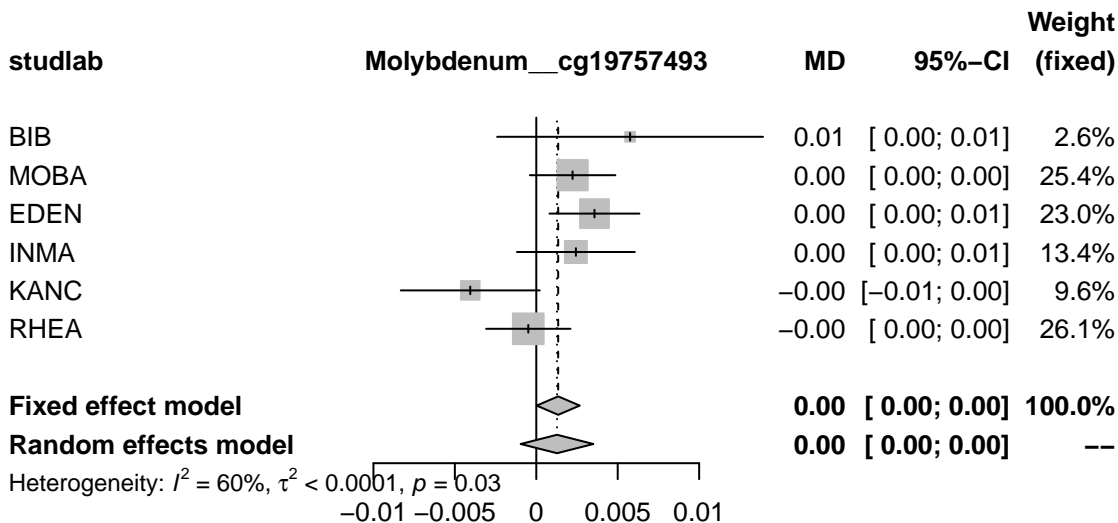

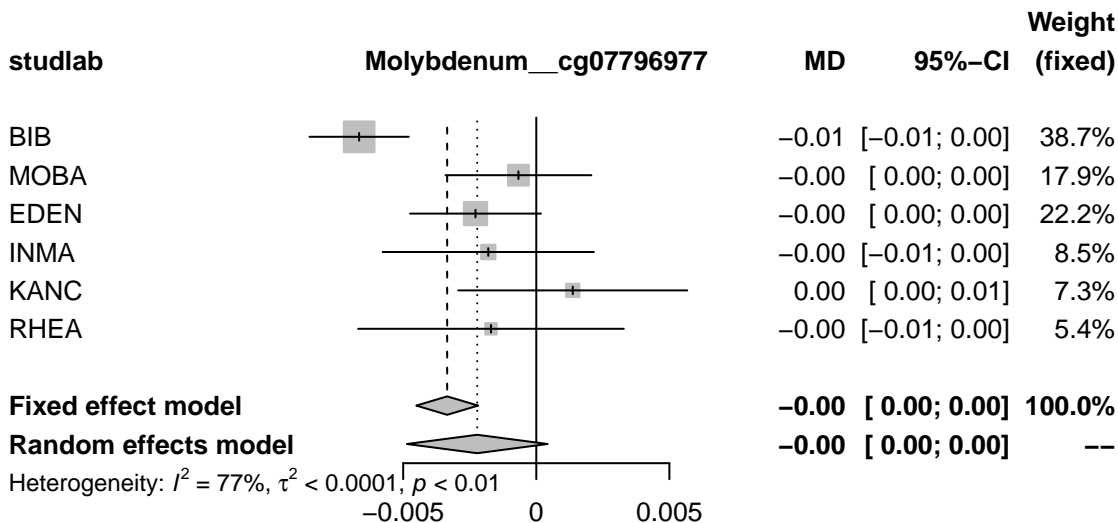

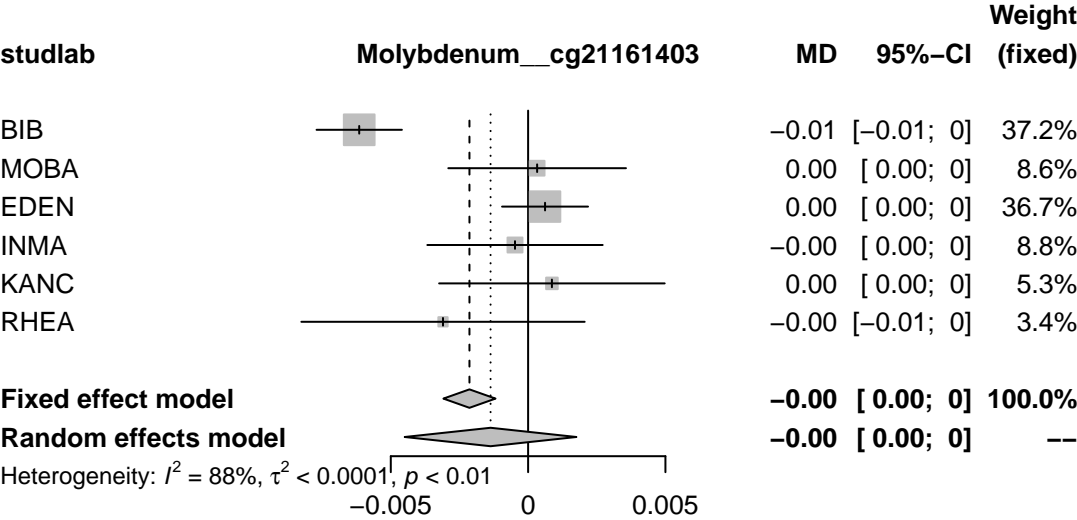

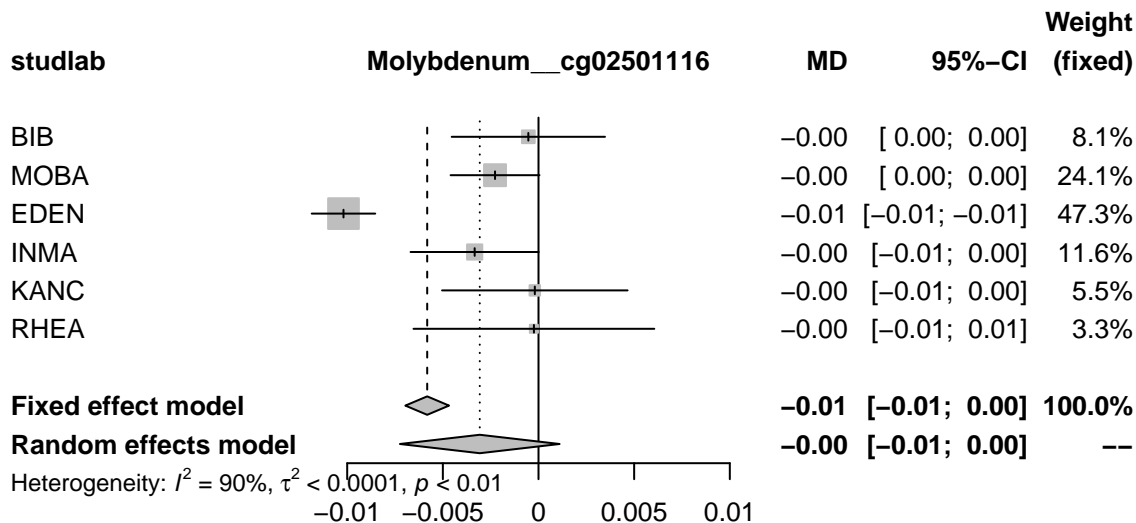

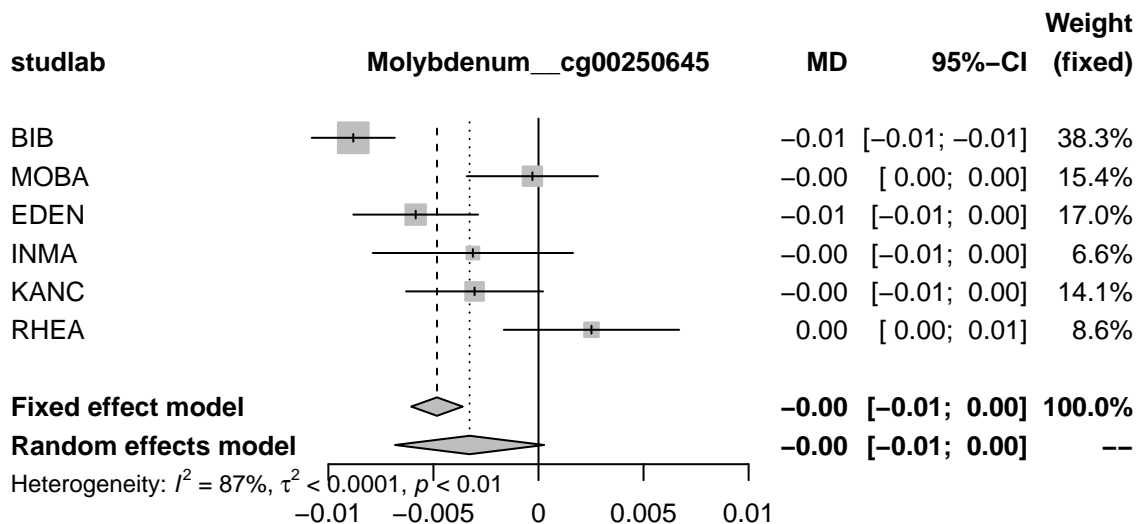

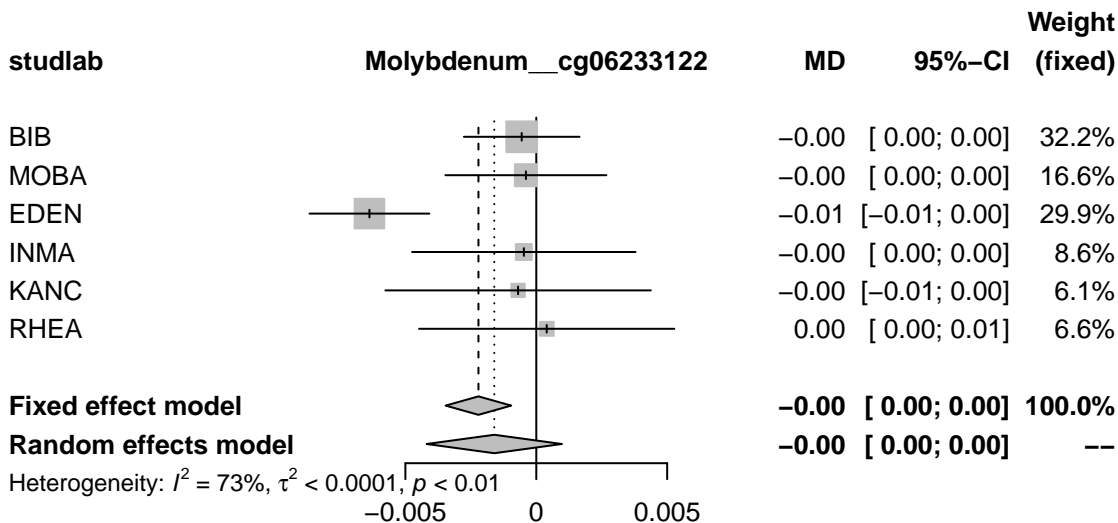

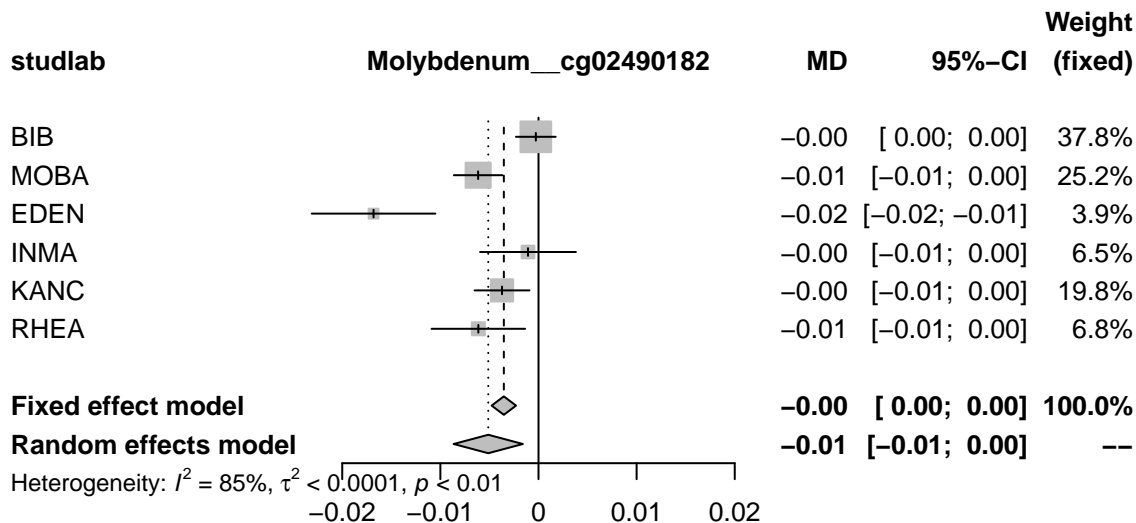

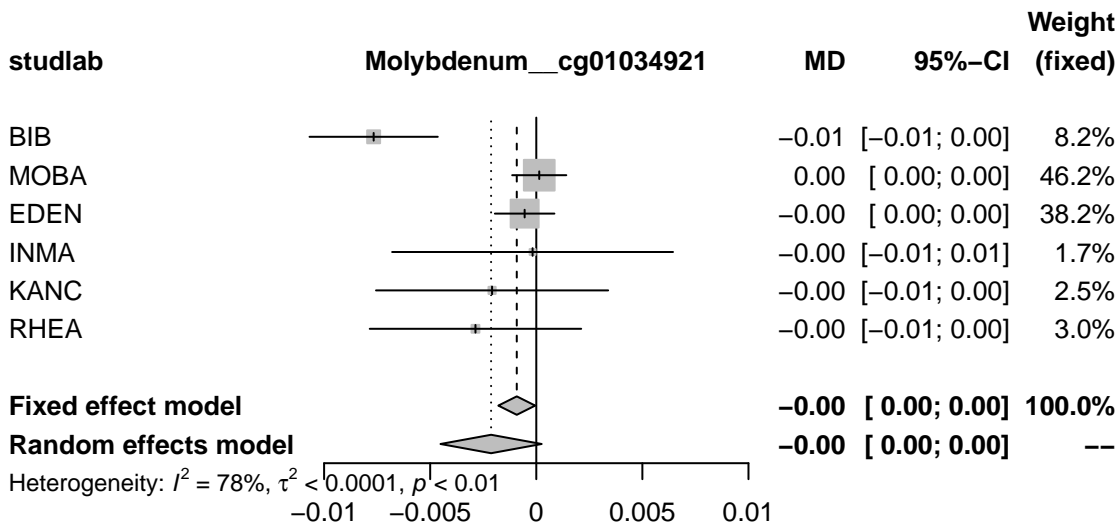

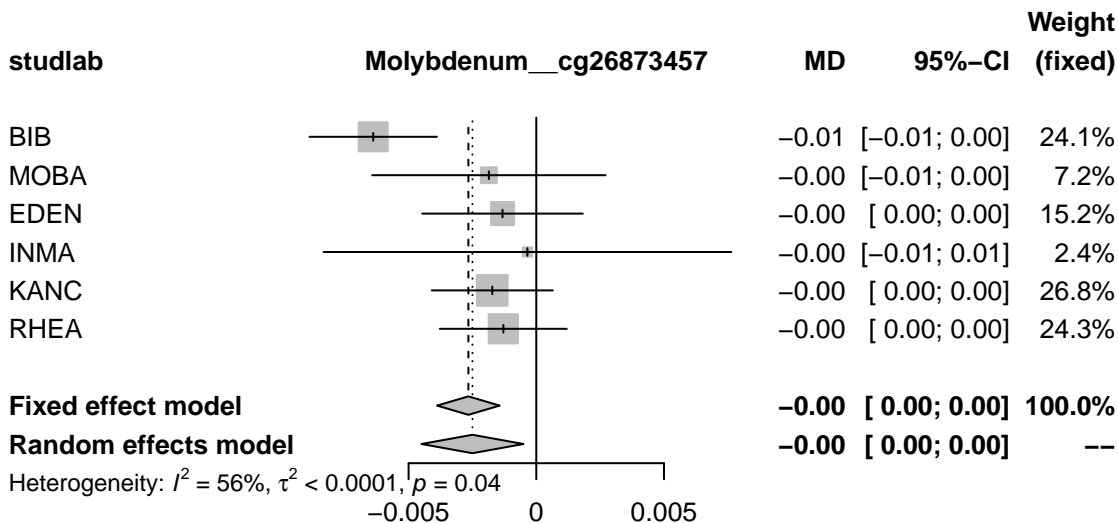

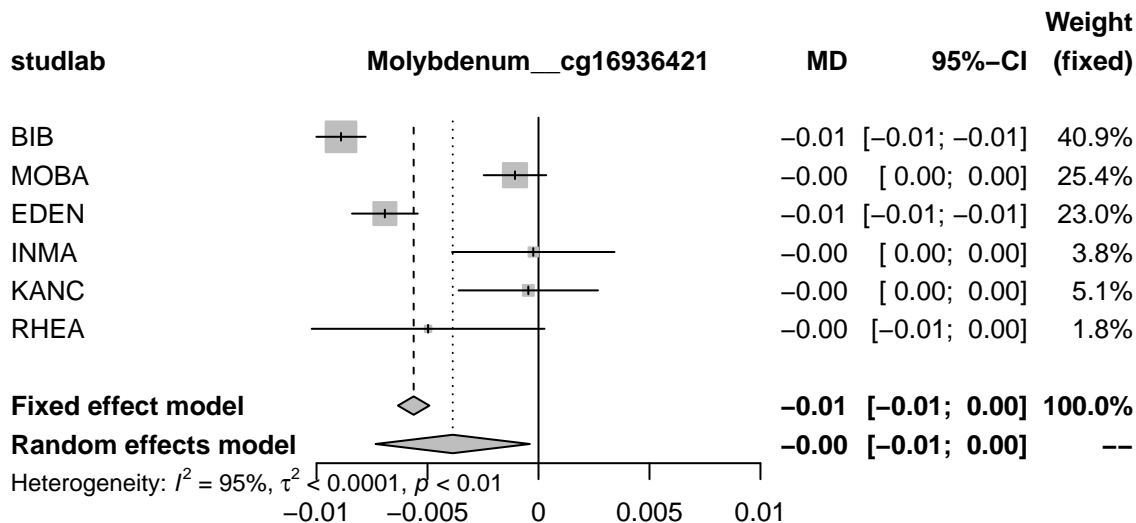

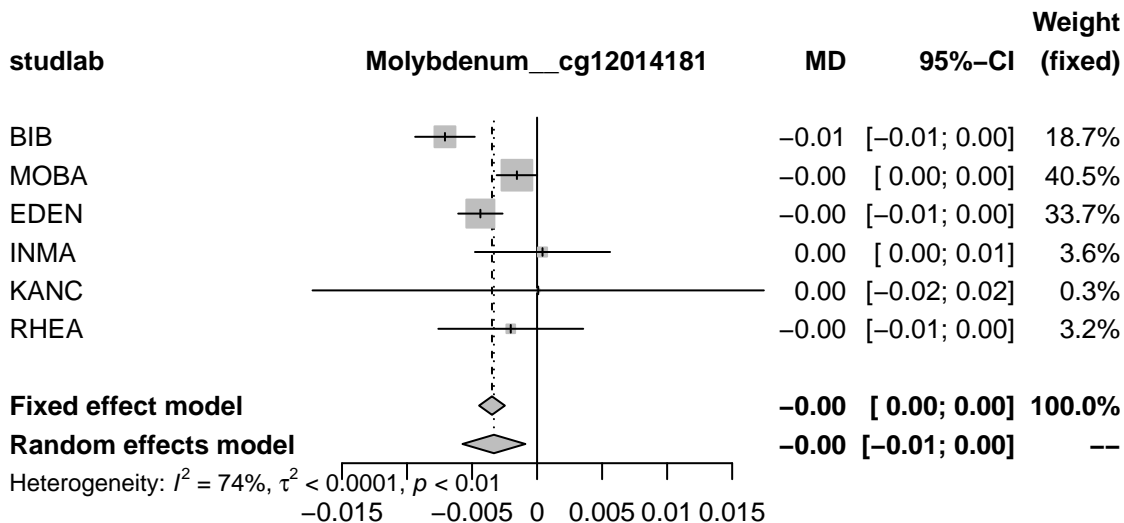

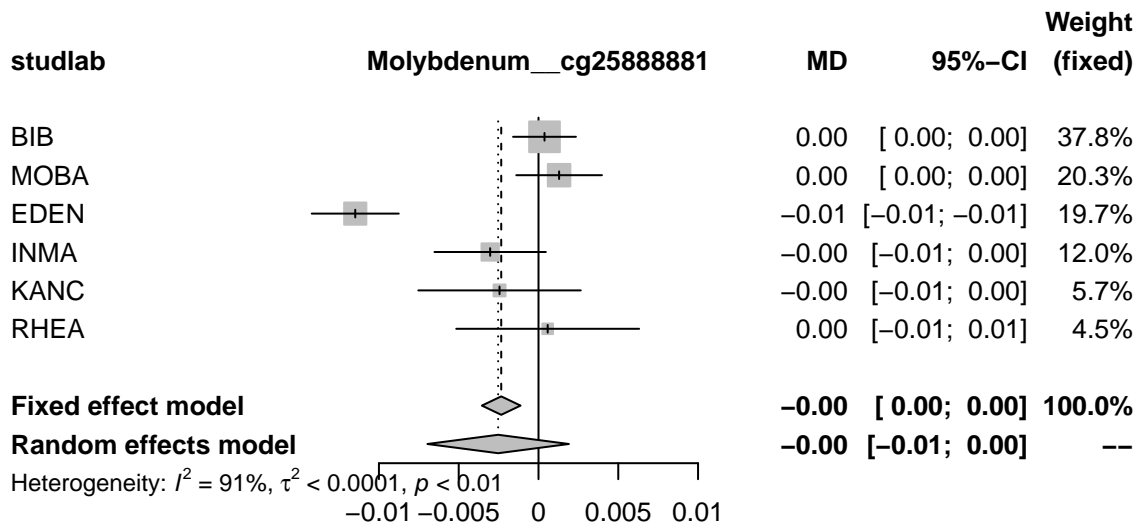

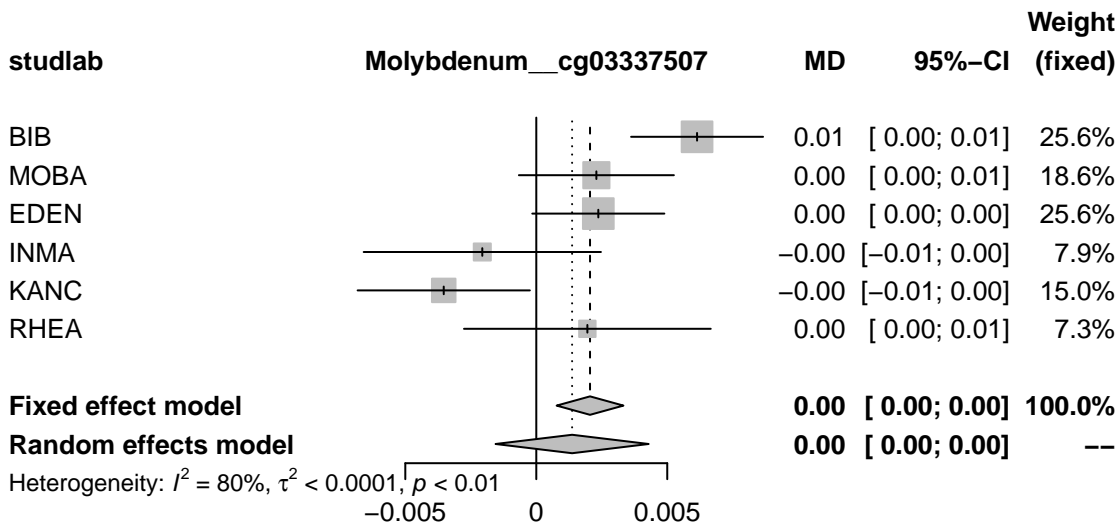

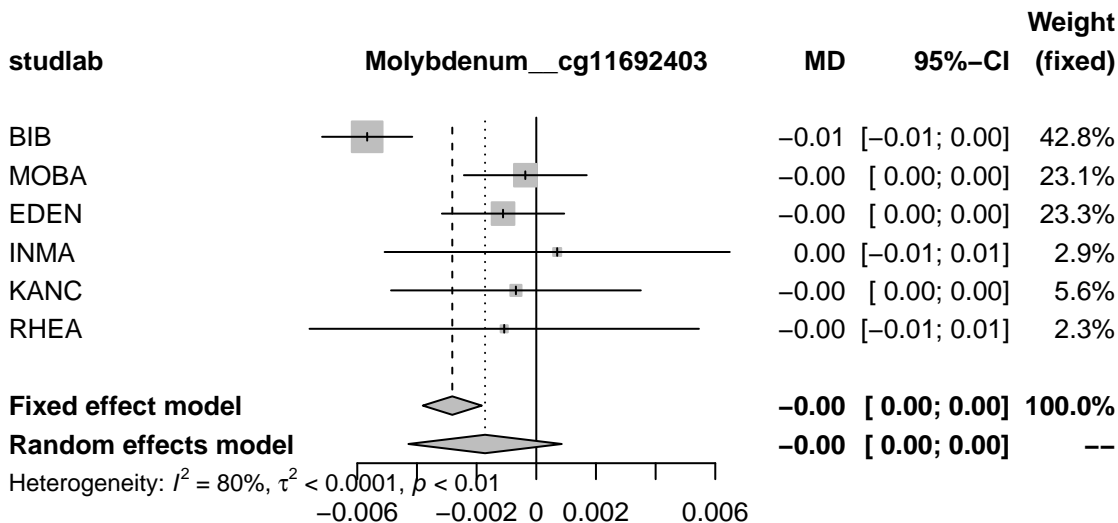

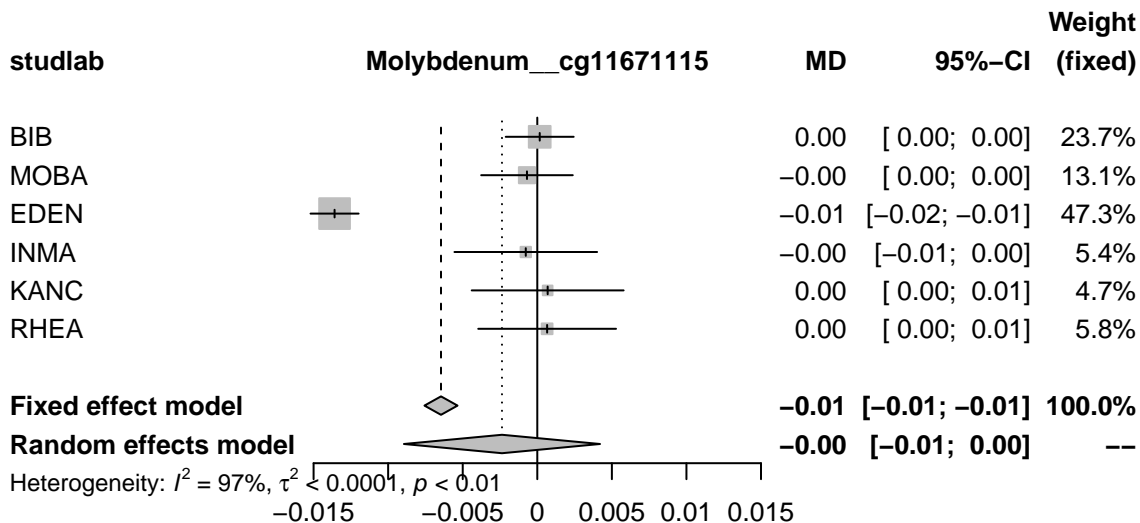

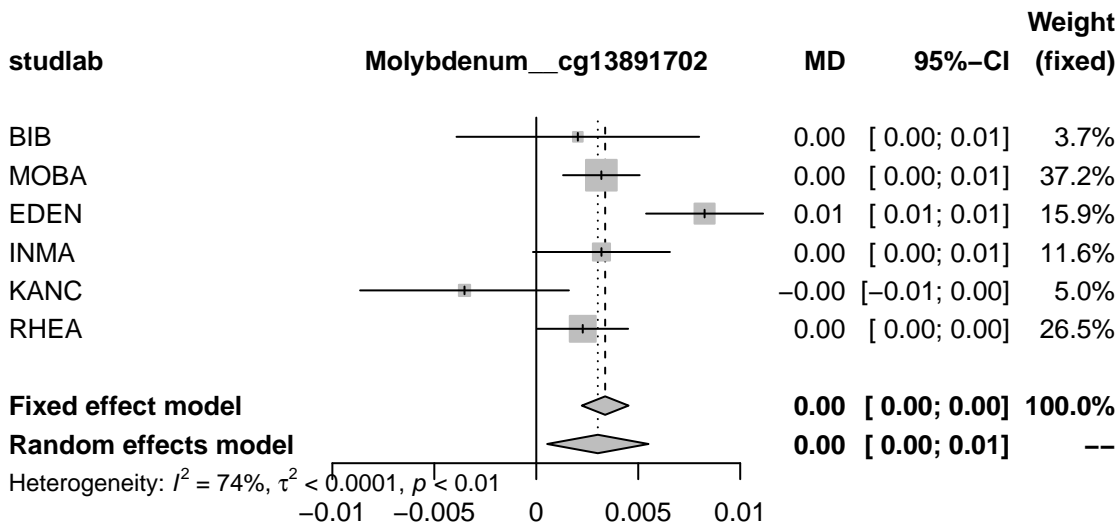

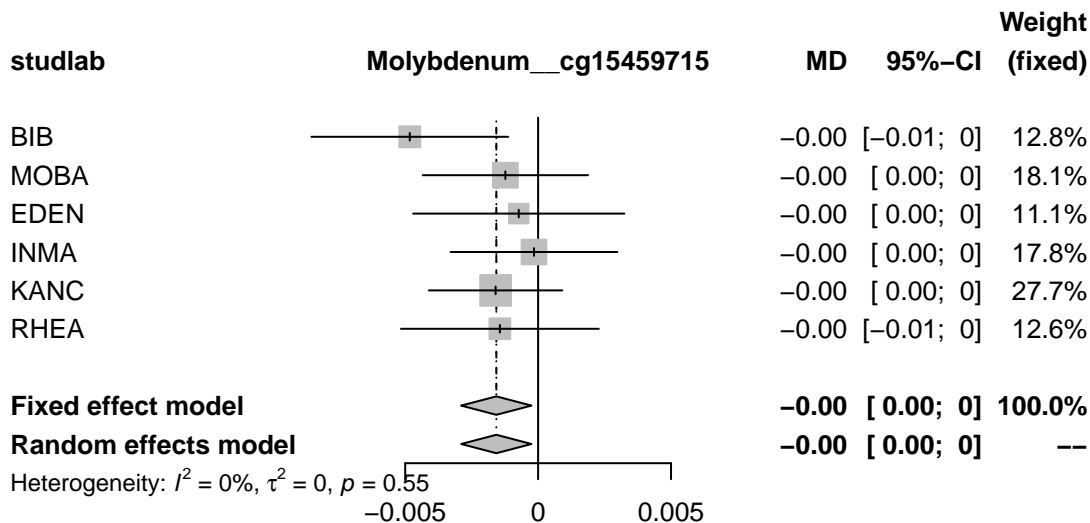

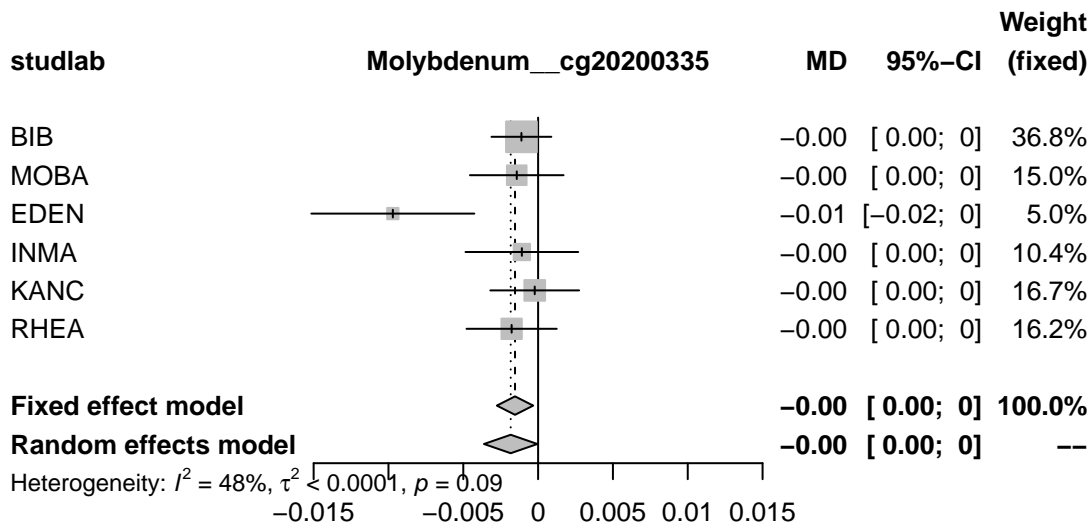

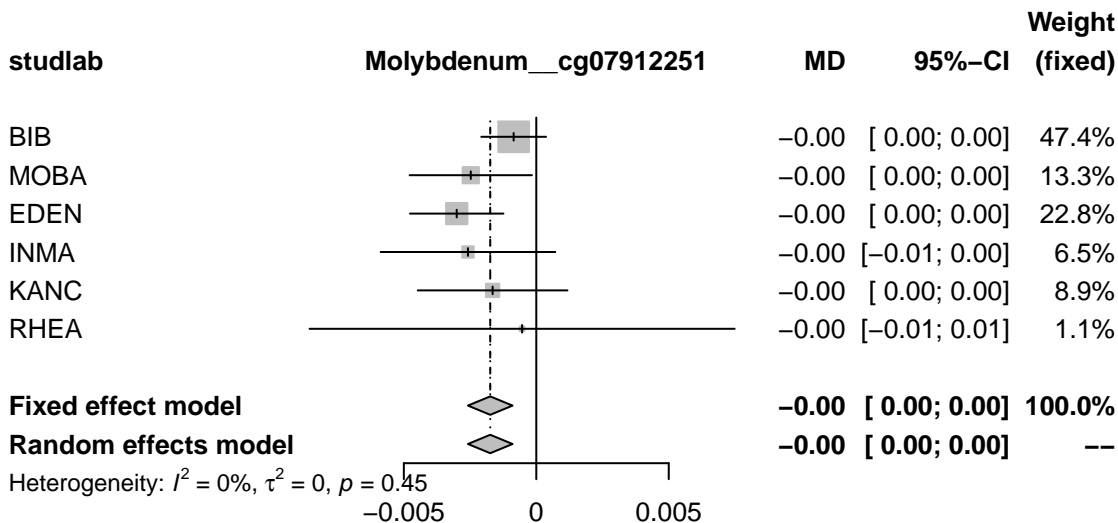

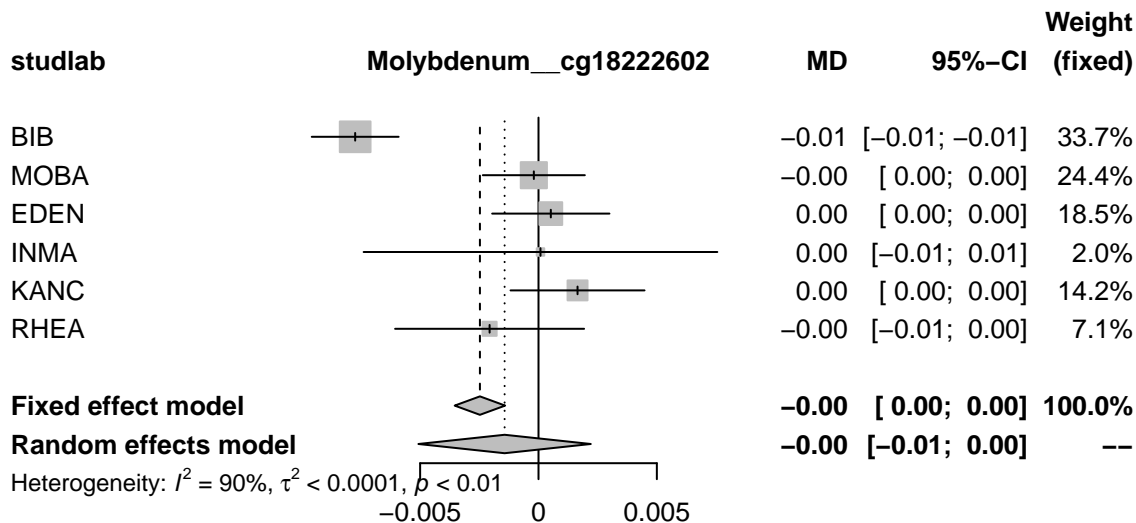

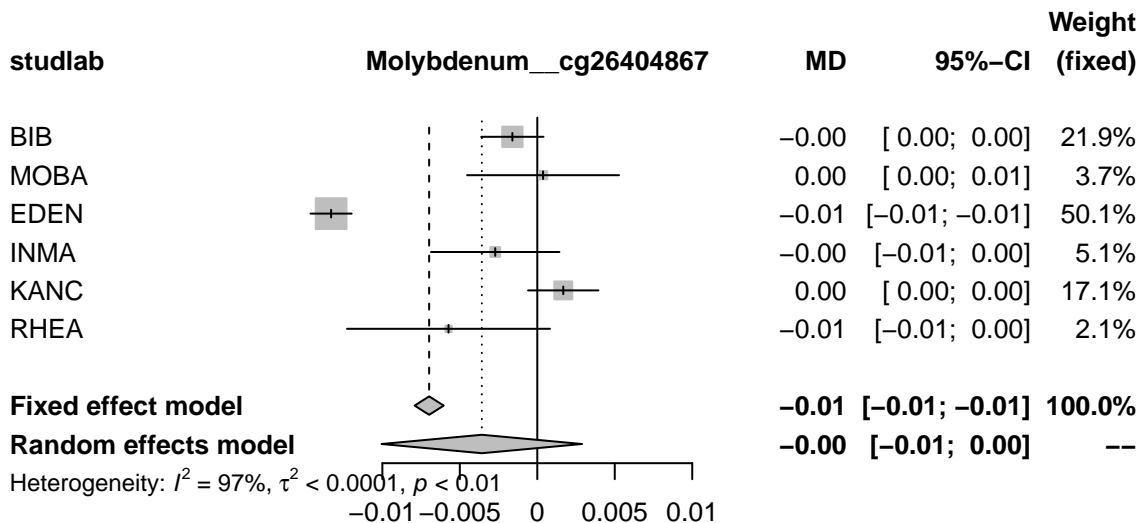

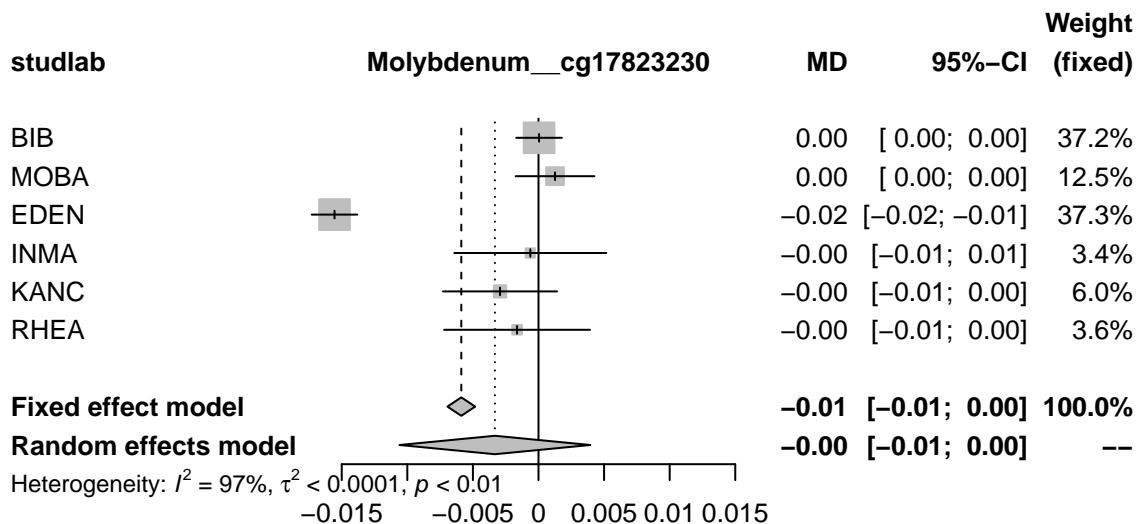

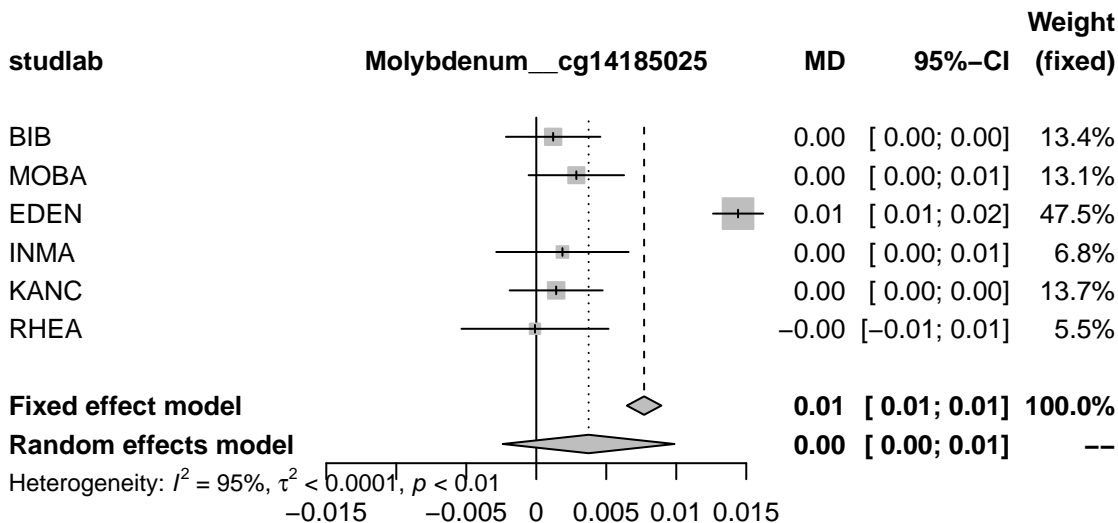

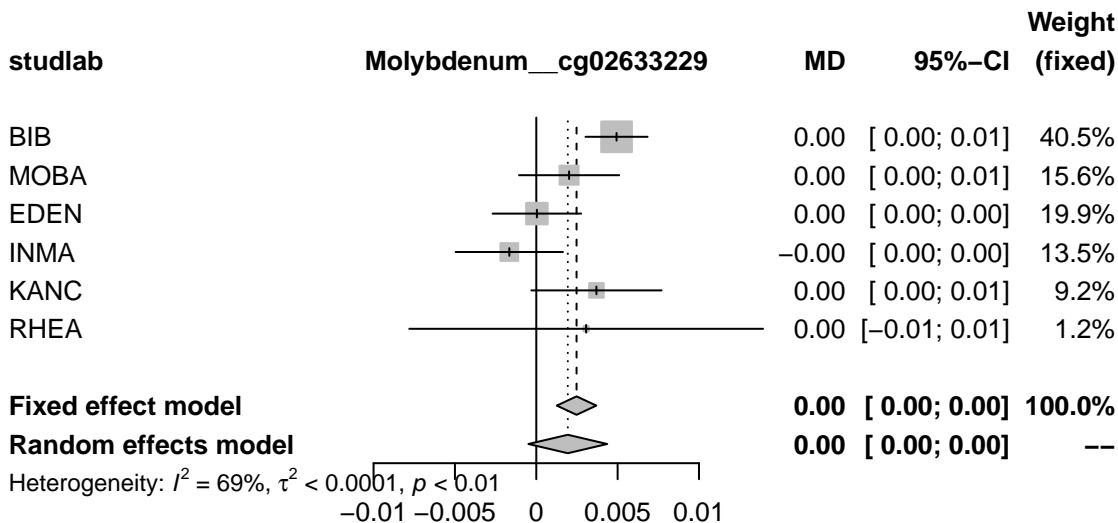

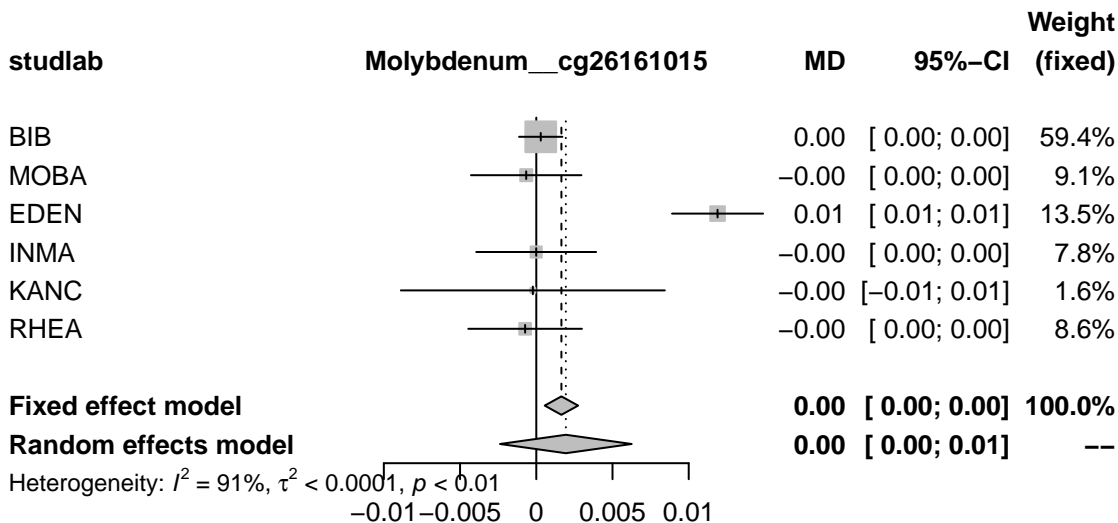

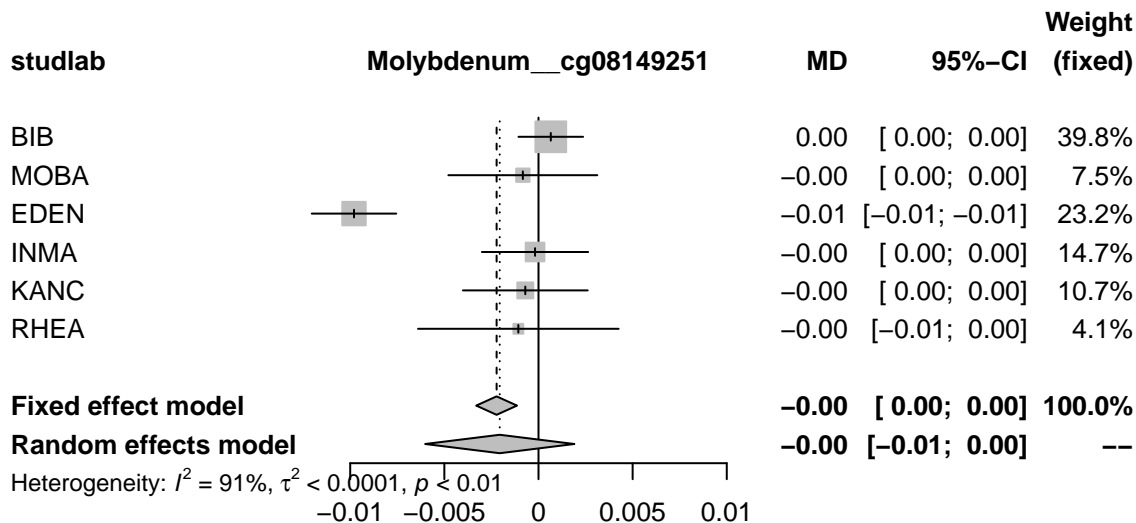

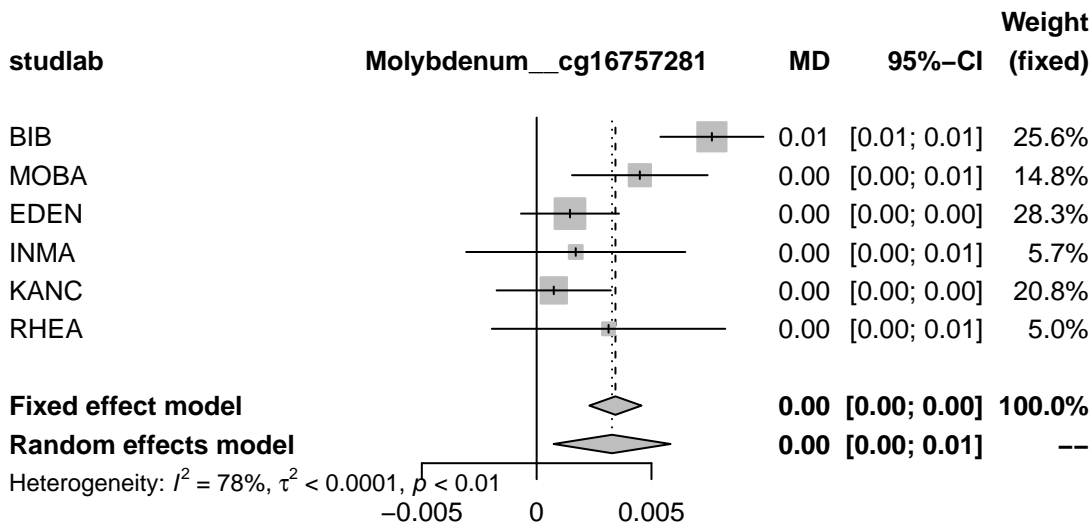

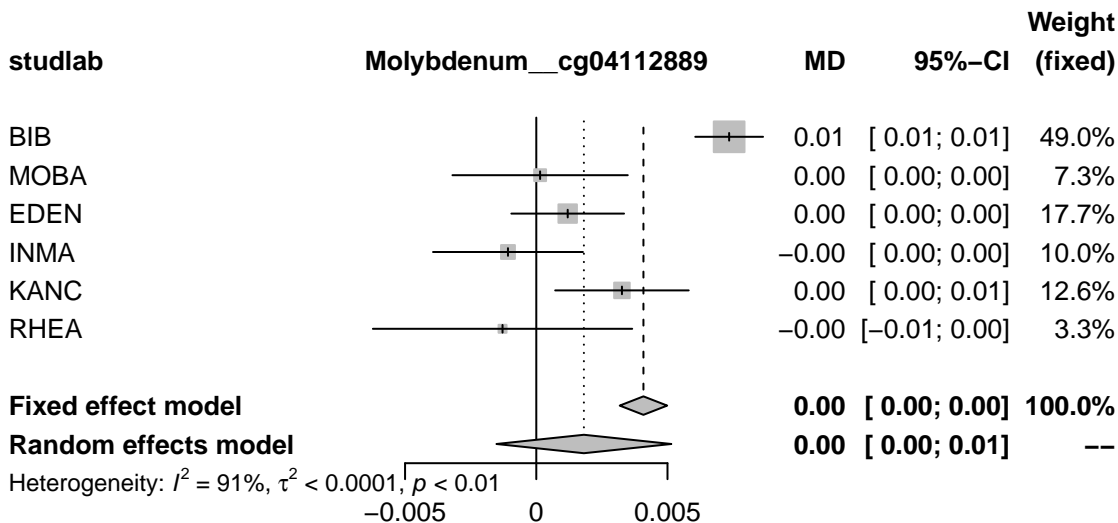

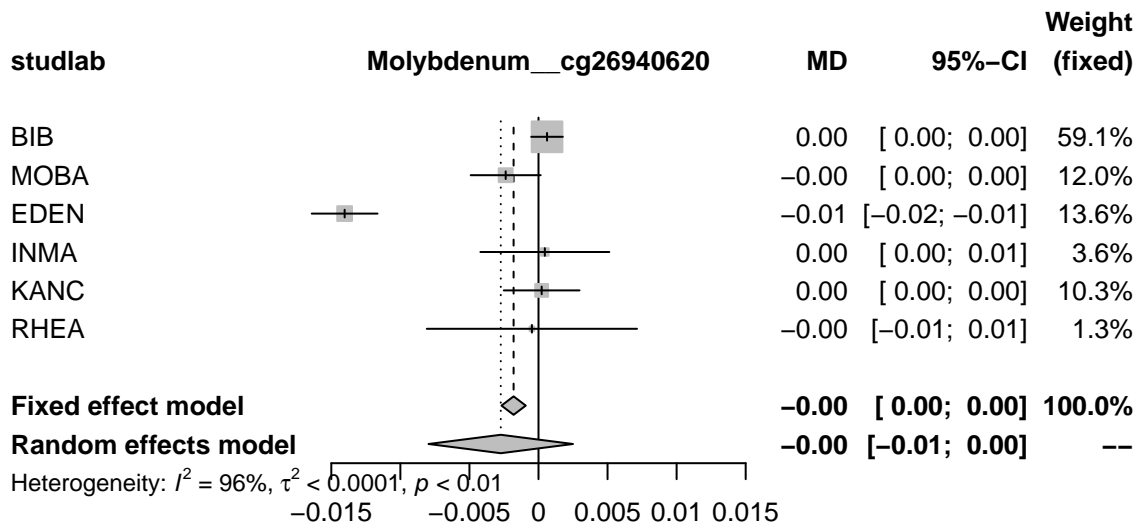

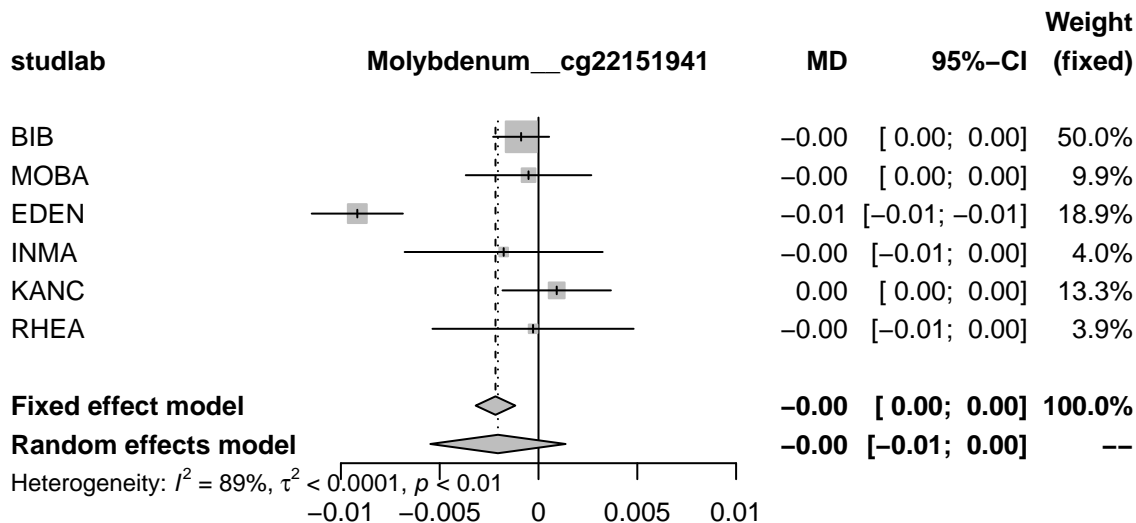

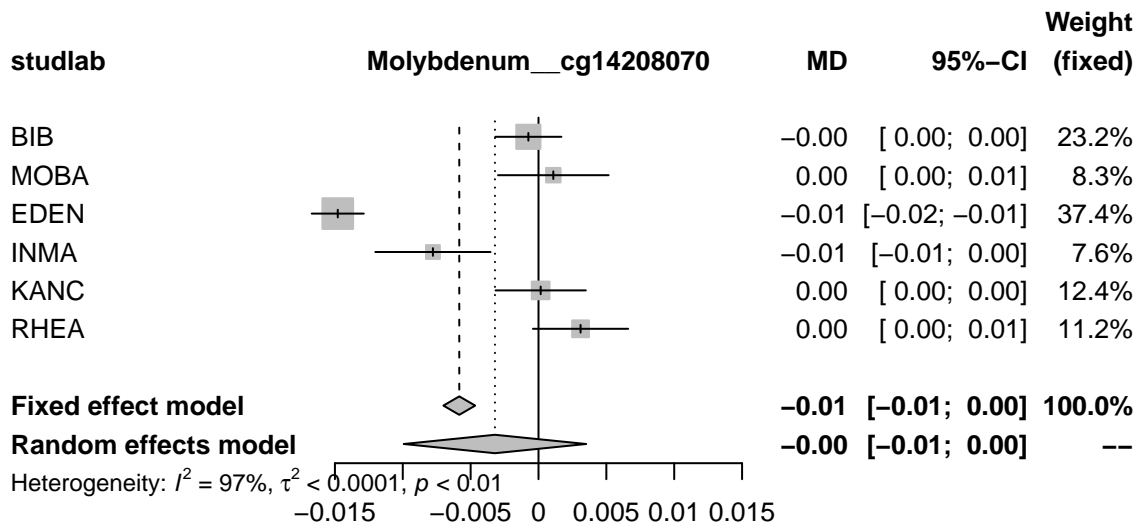

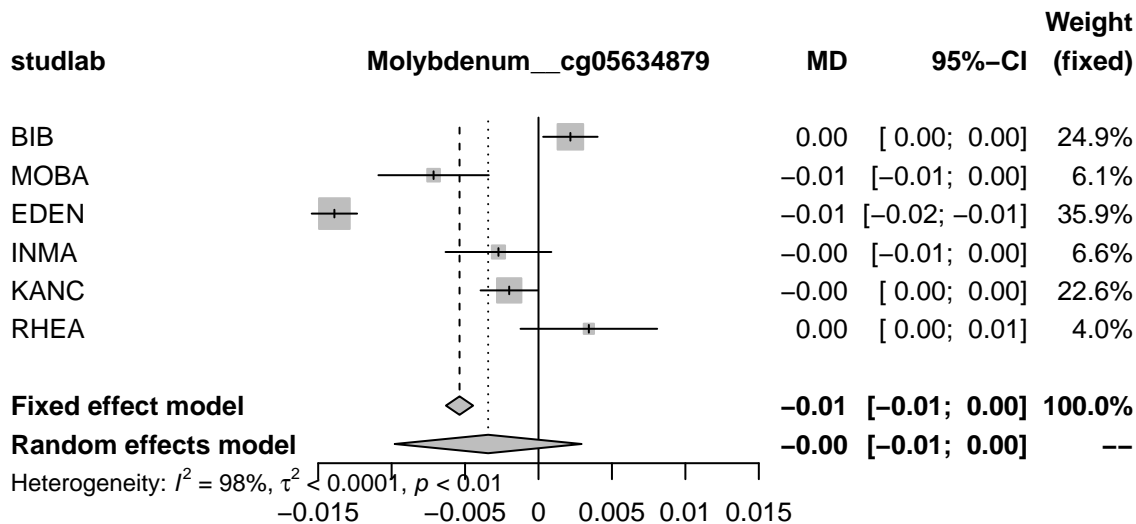

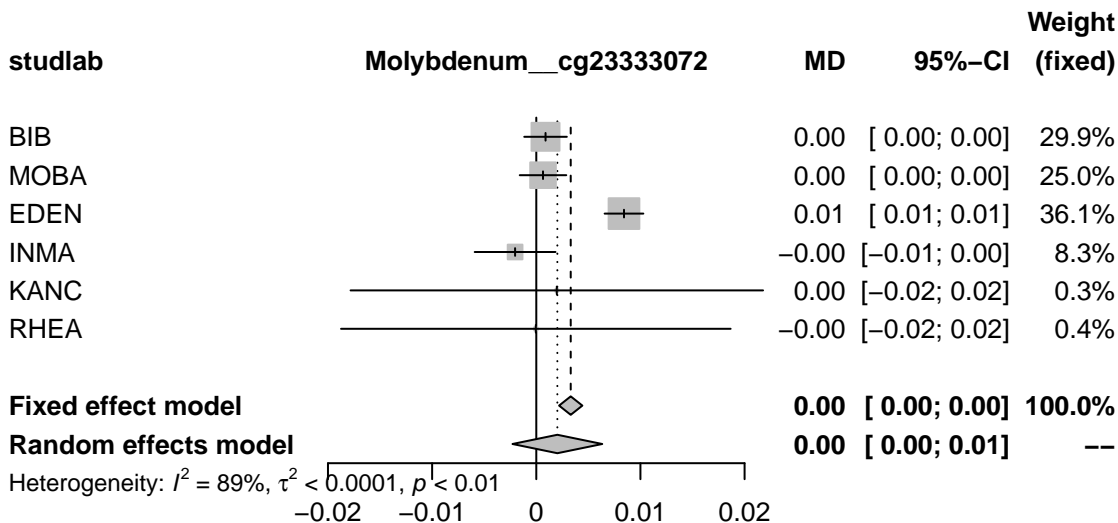

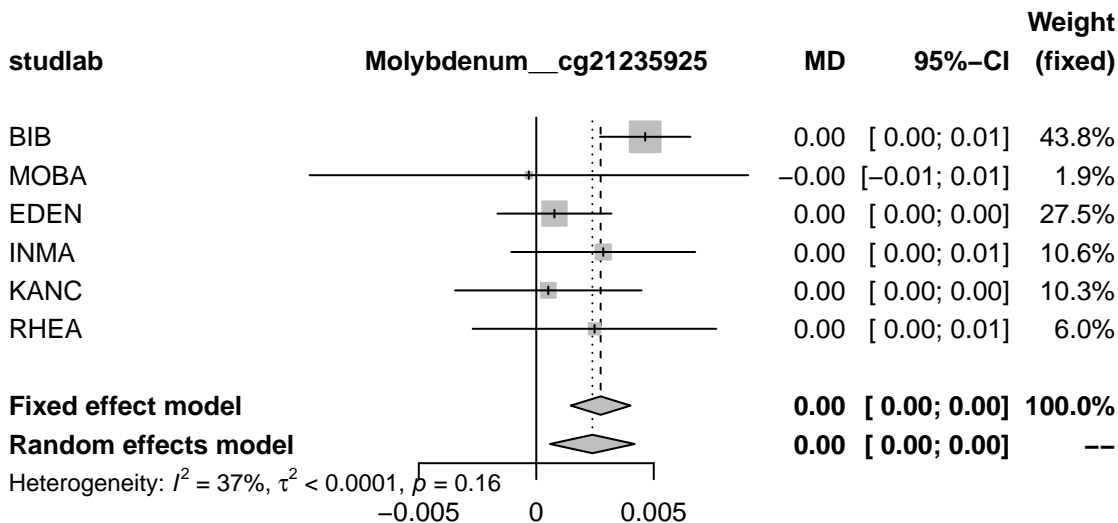

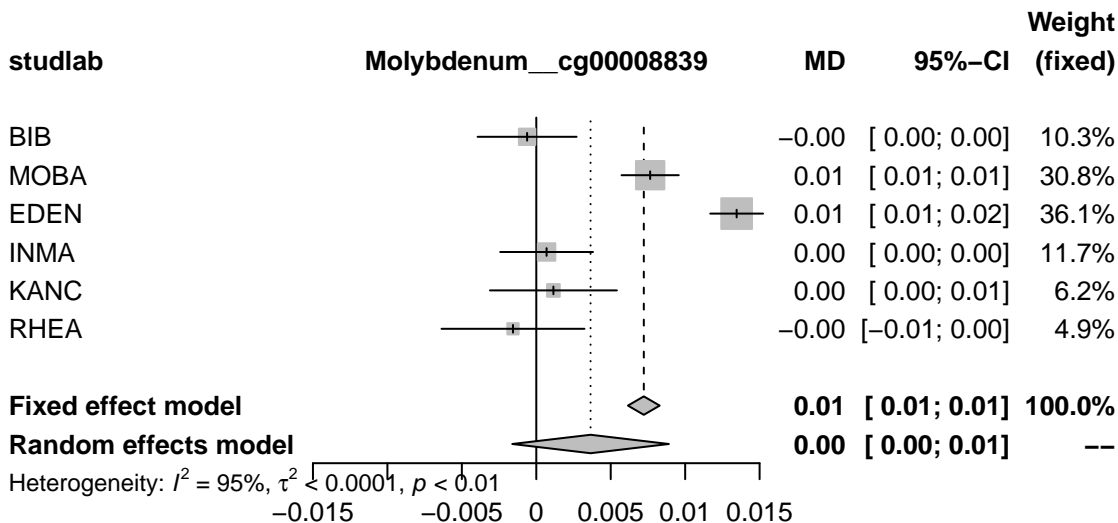

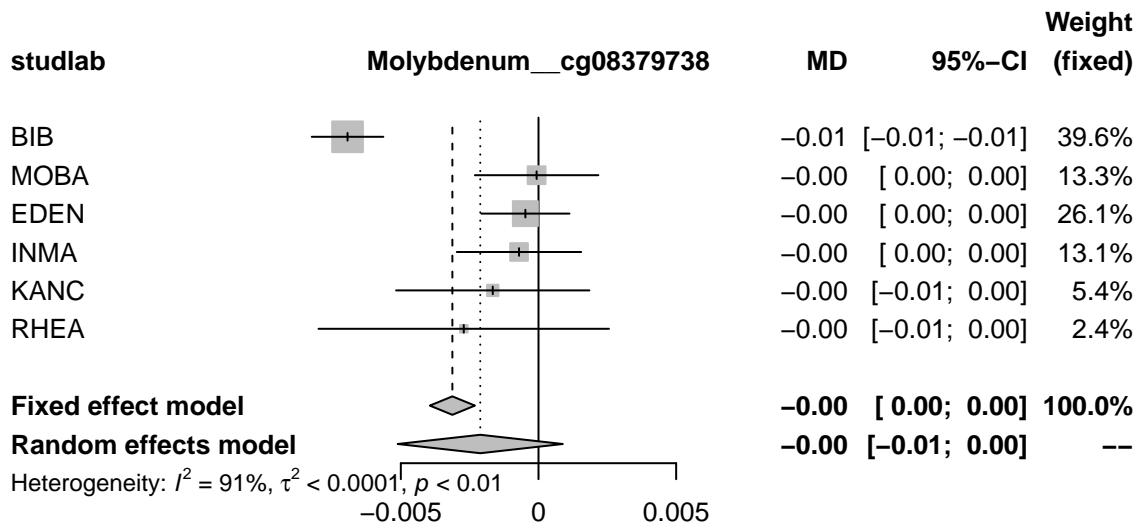

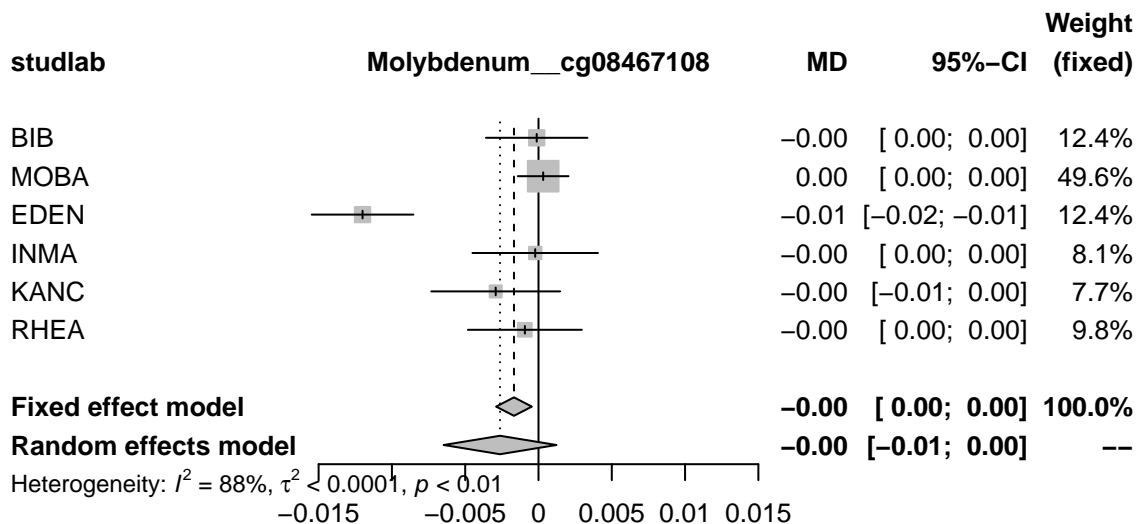

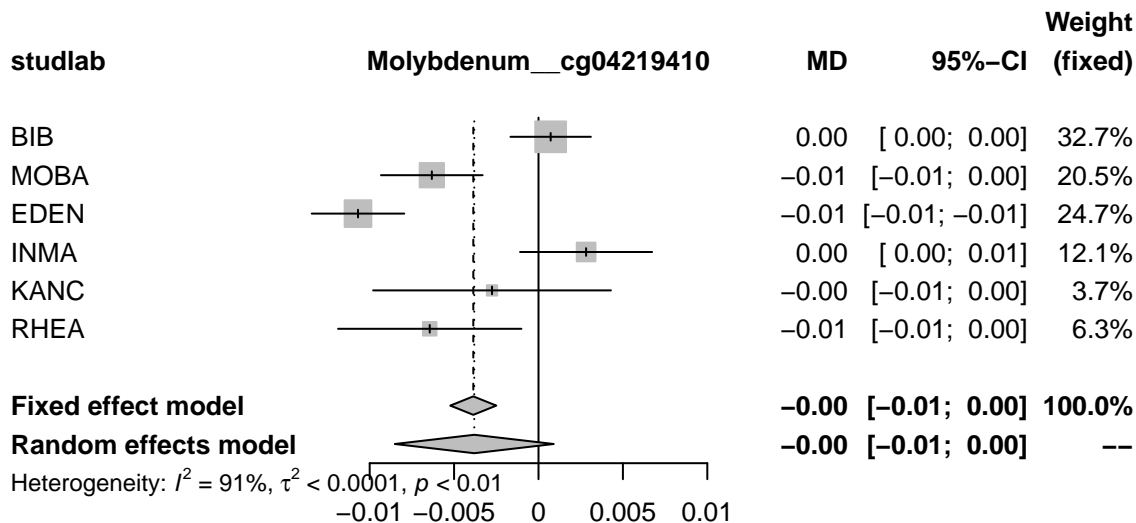

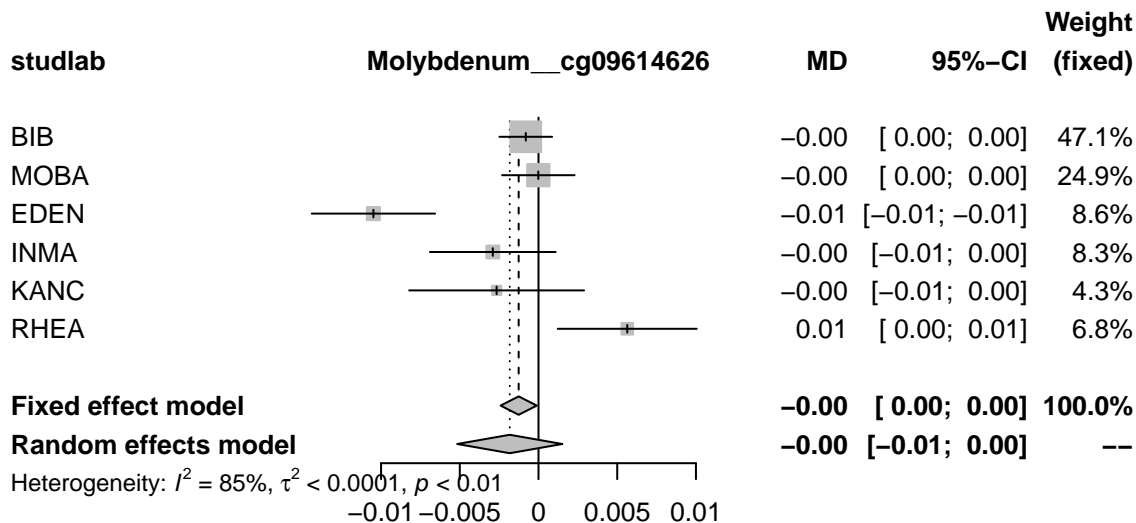

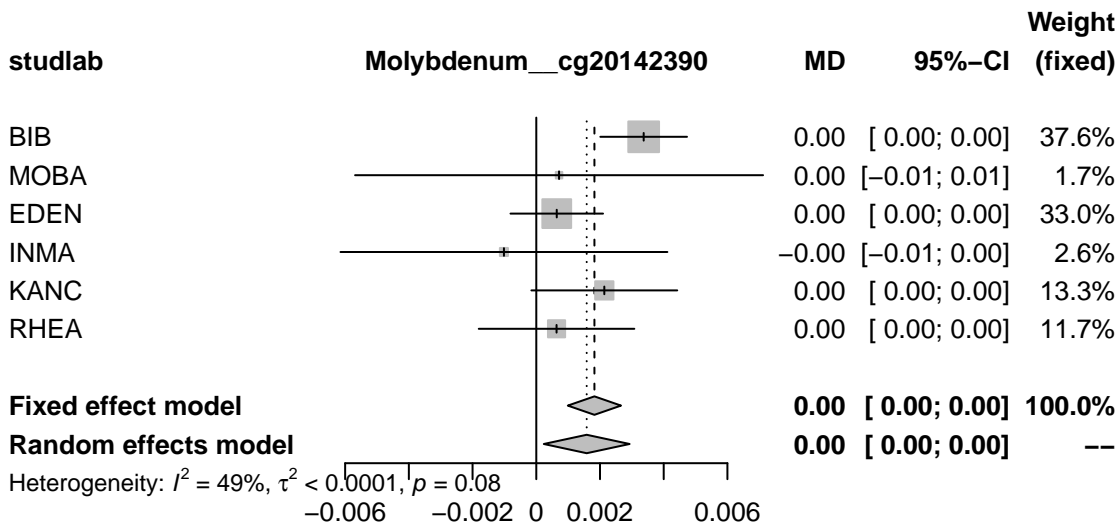

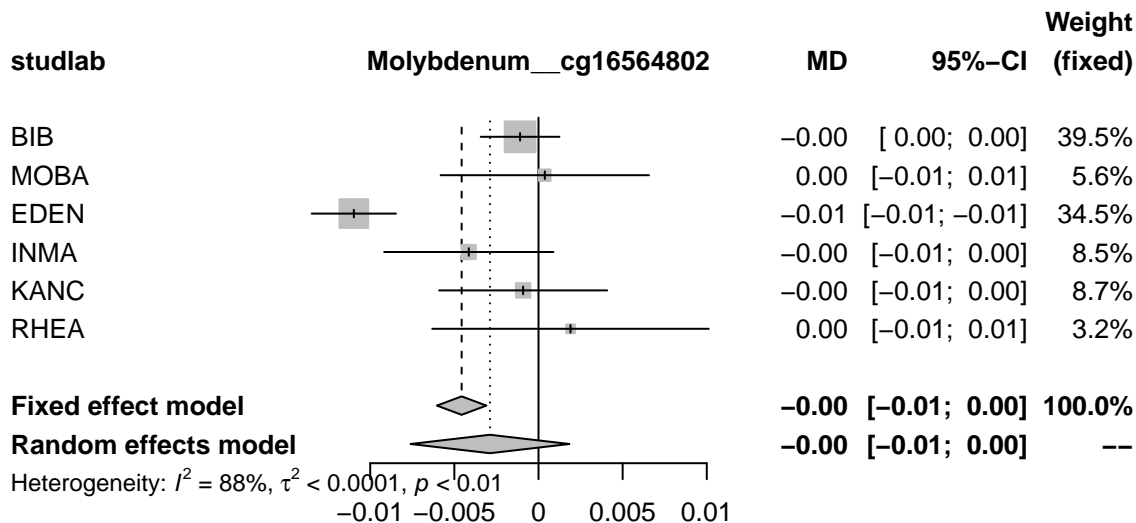

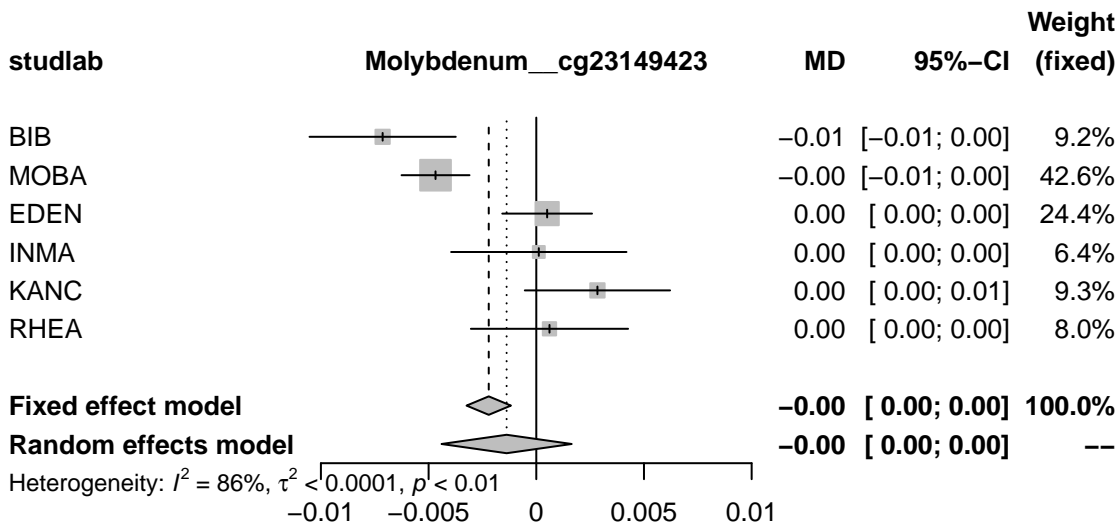

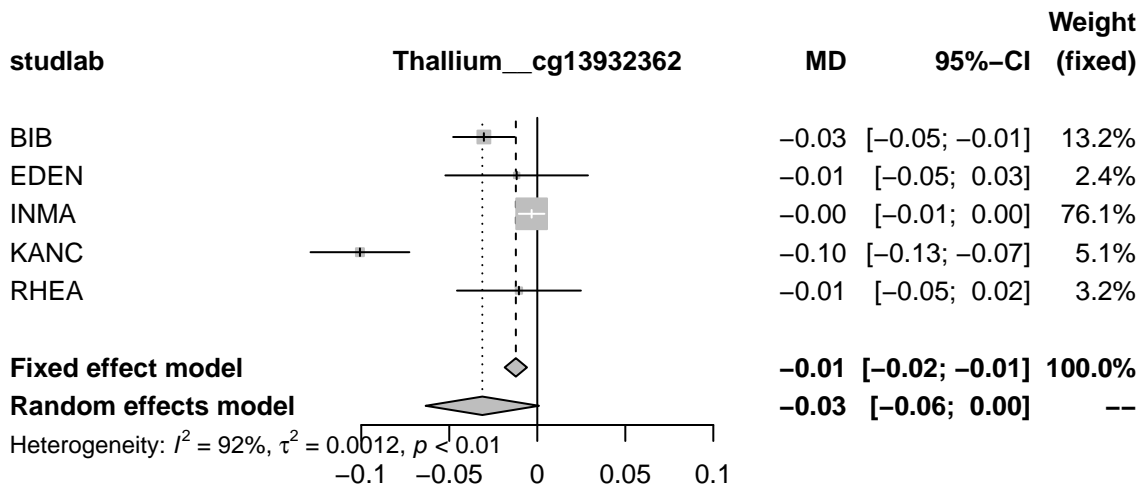

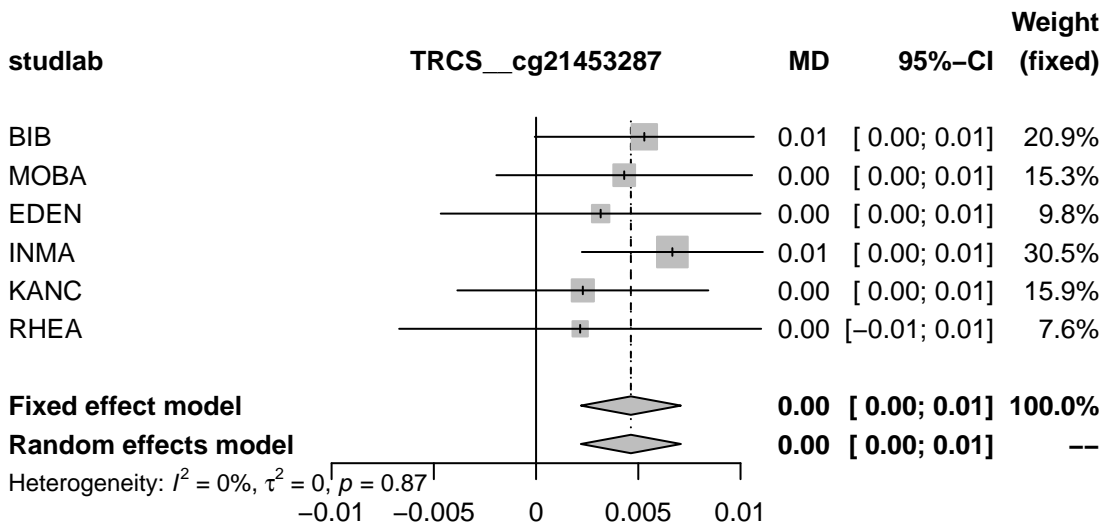

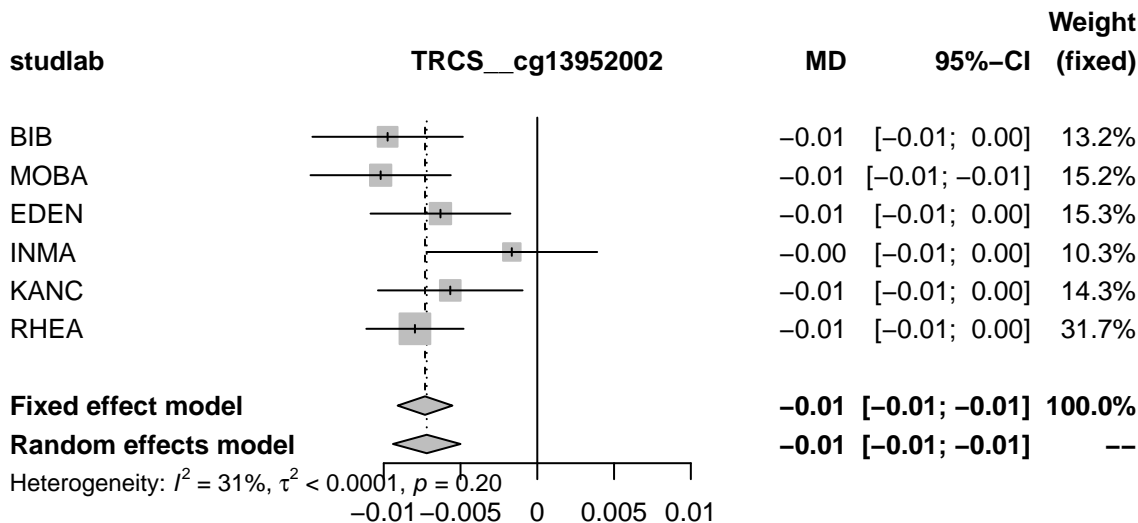

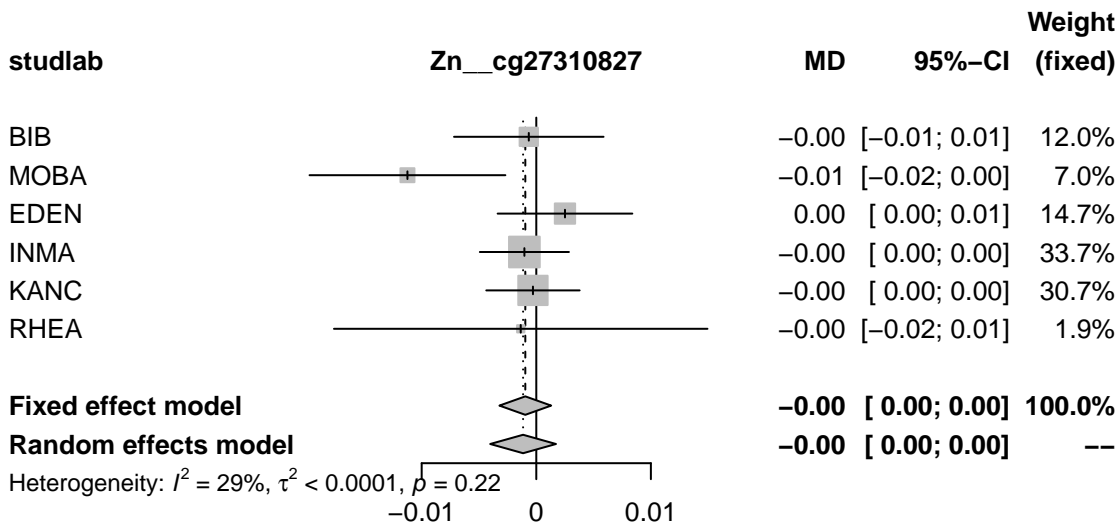

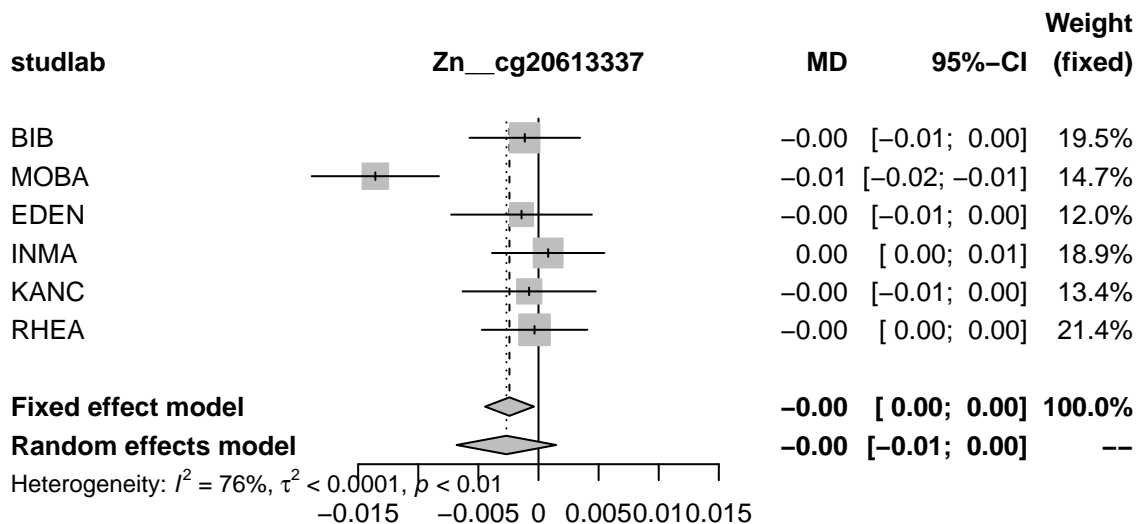

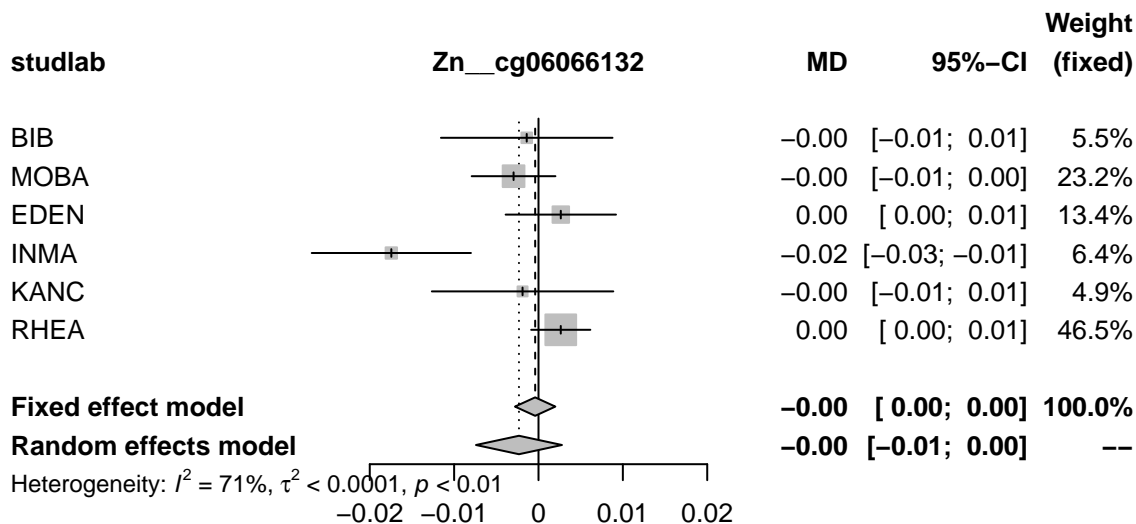

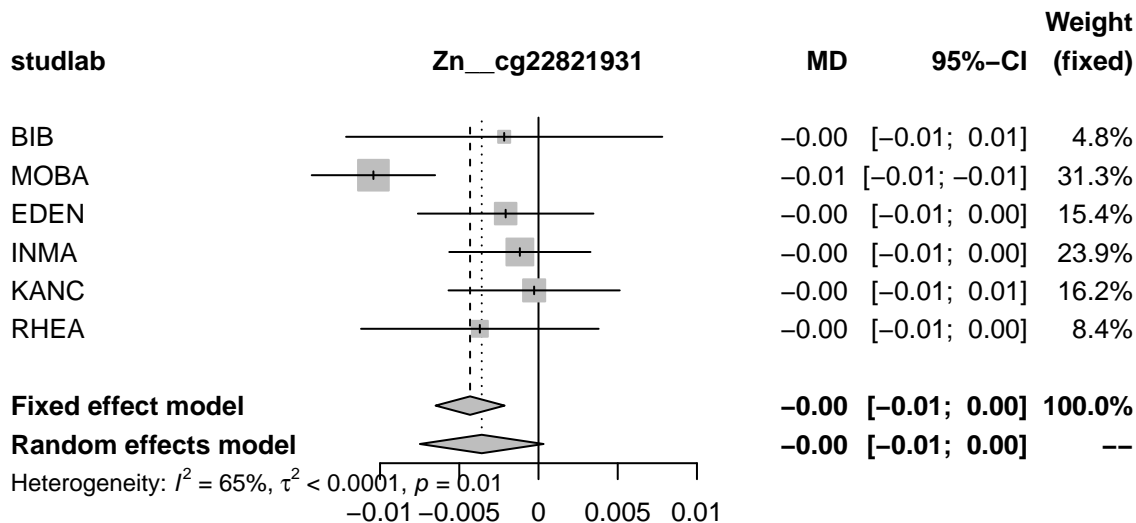

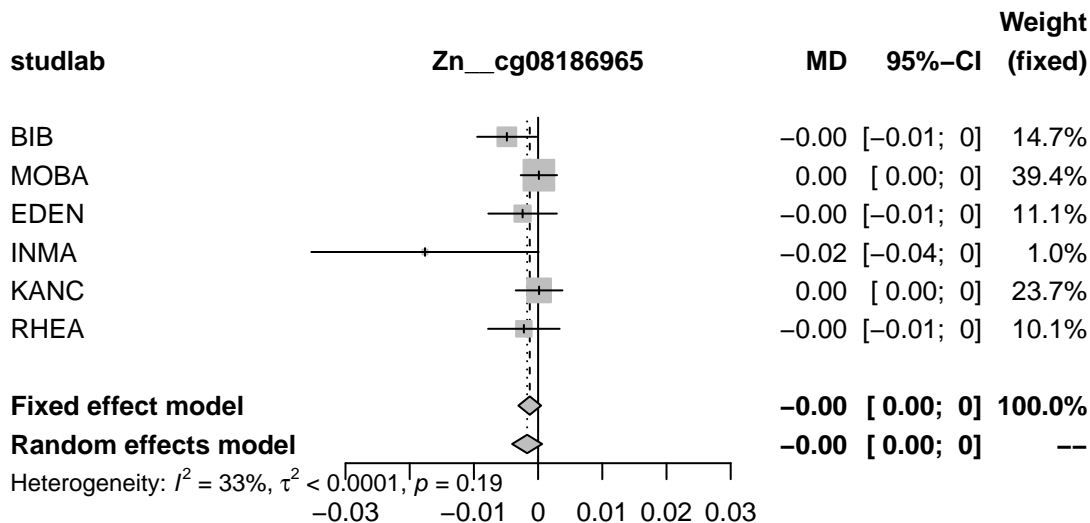

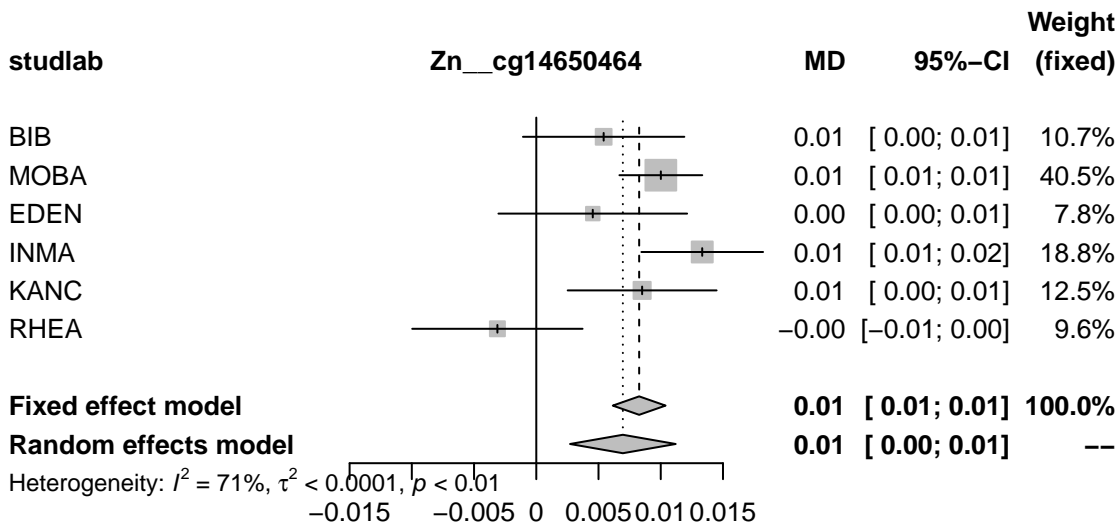

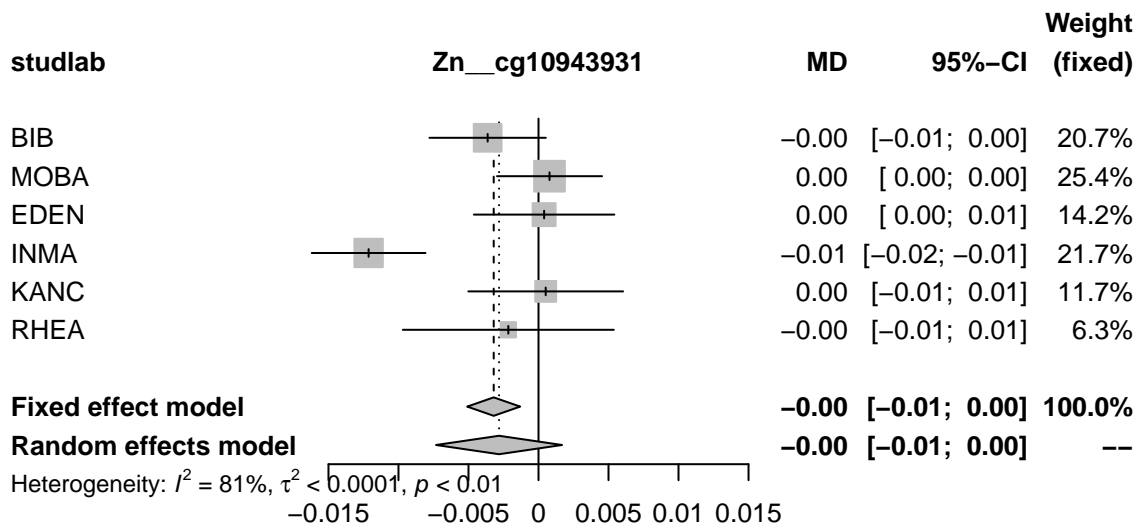

Supplement: Supplementary file 14 — Supplementary Dataset 11 [file 41467_2022_34422_MOESM14_ESM.zip › HELIX_ExpOmics_FigS2_Forestplots/HELIX_ExpOmics_FigS2A_meth_preg.pdf]

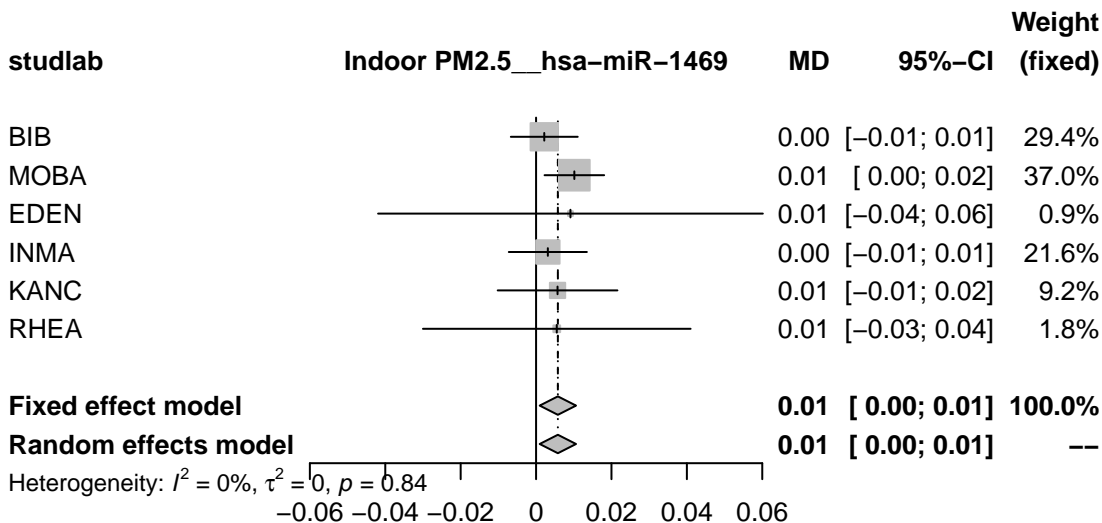

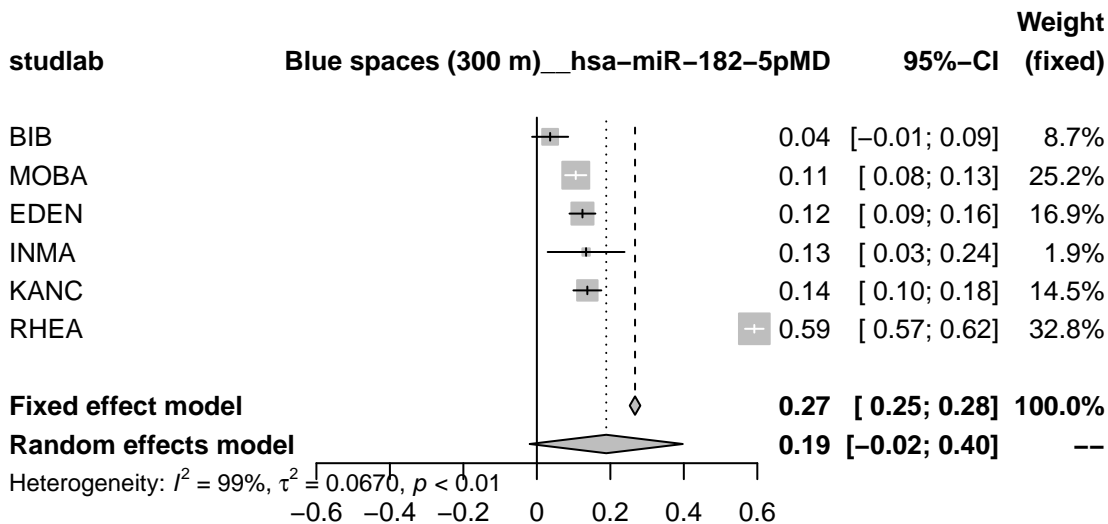

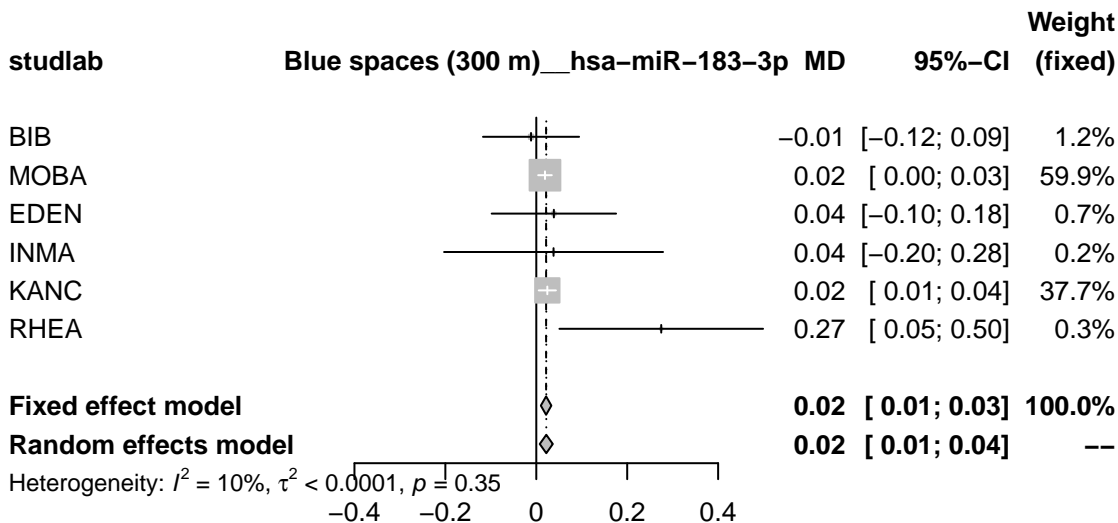

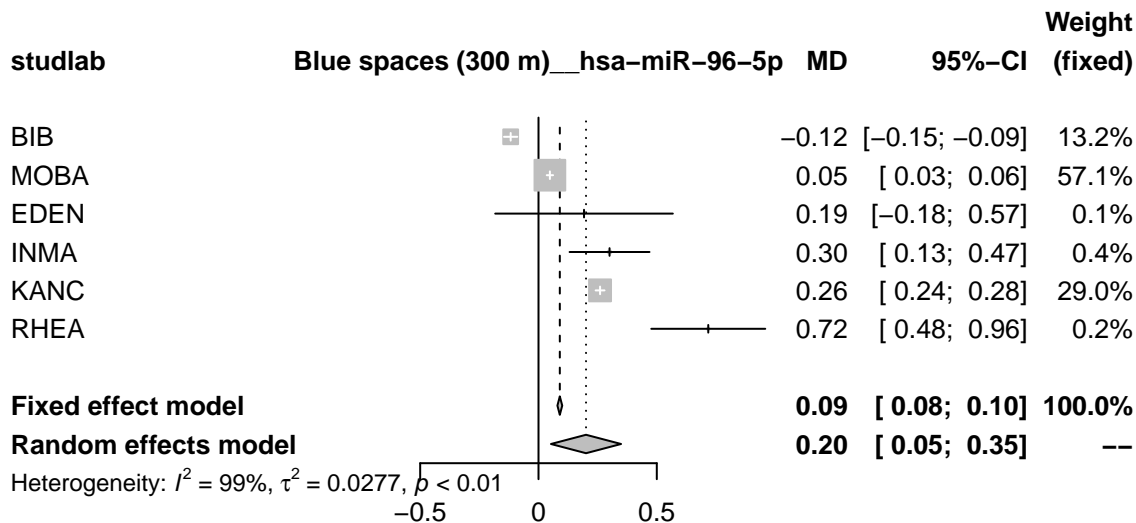

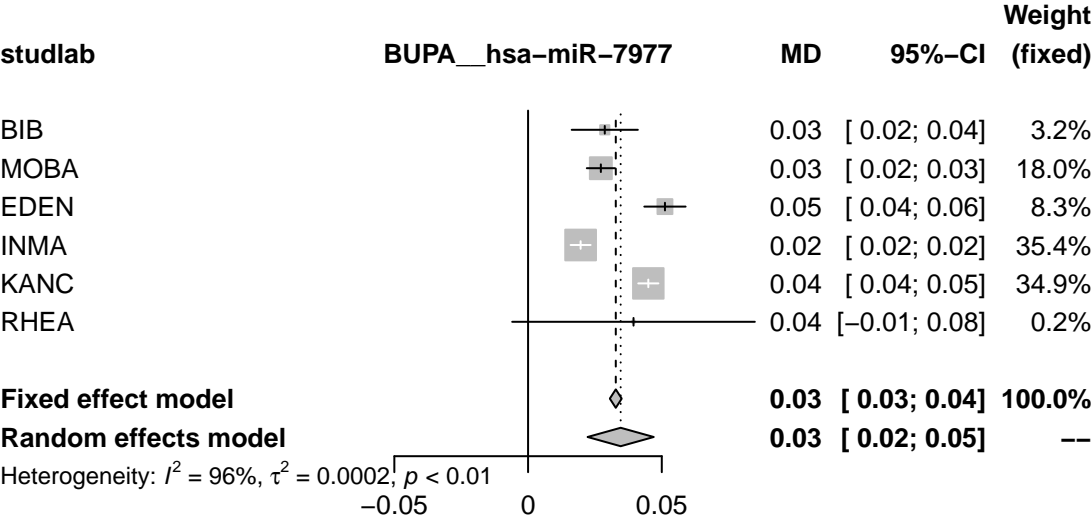

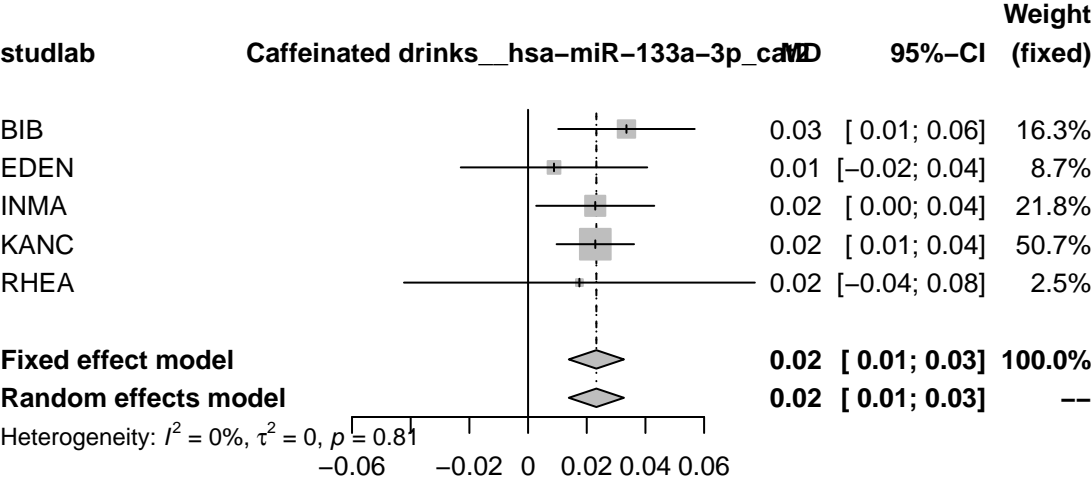

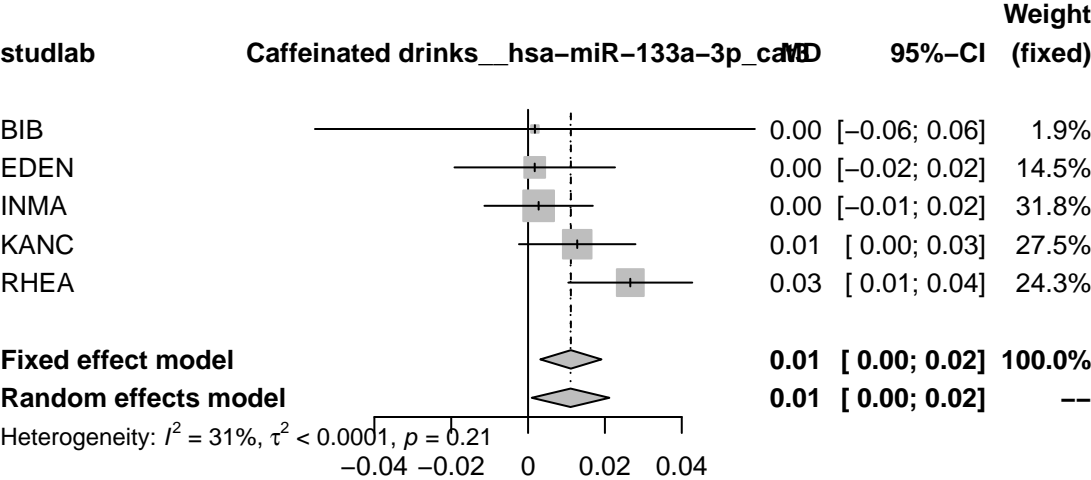

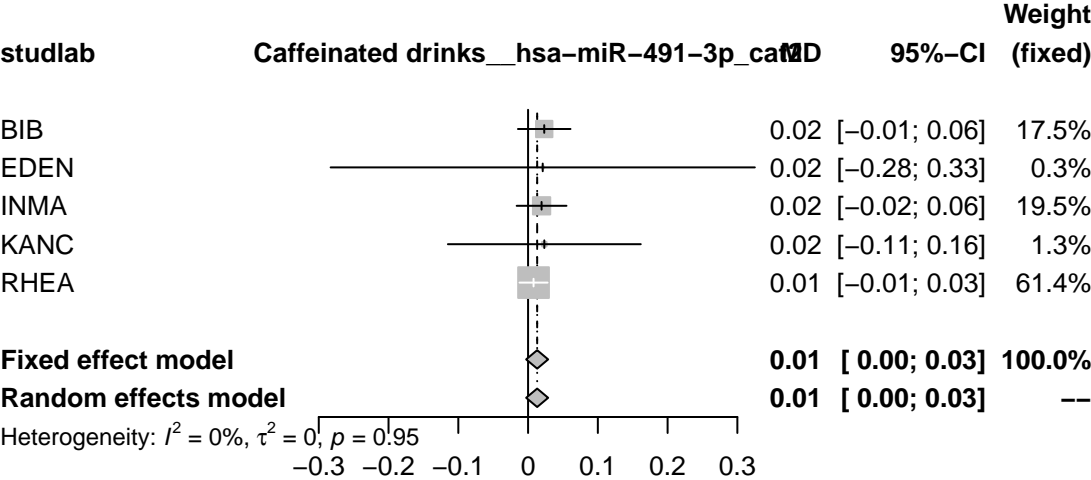

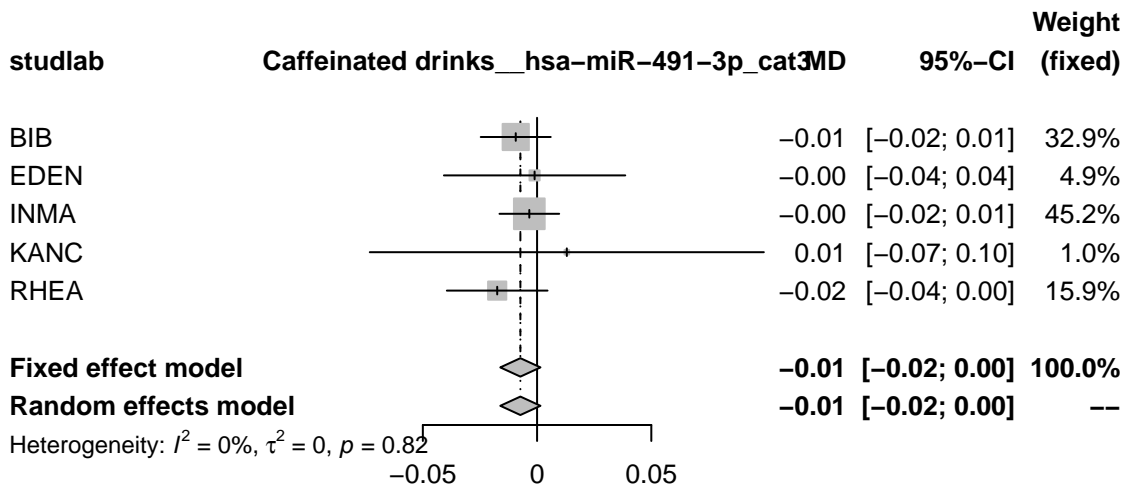

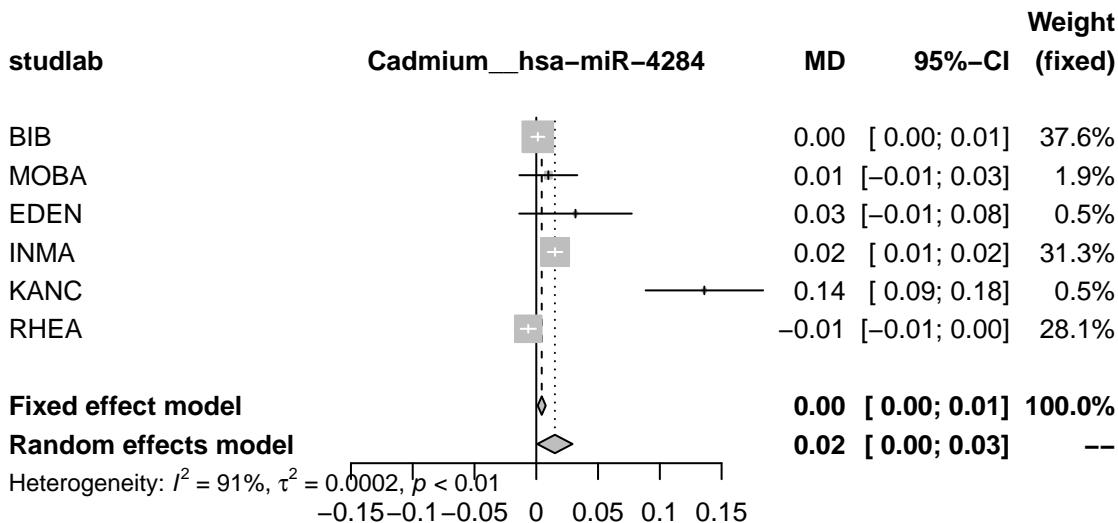

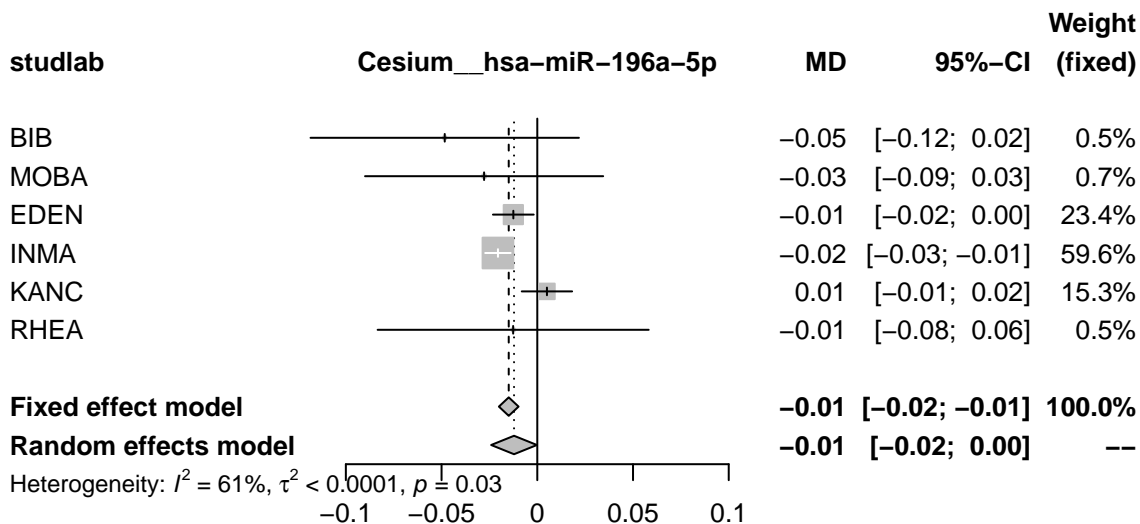

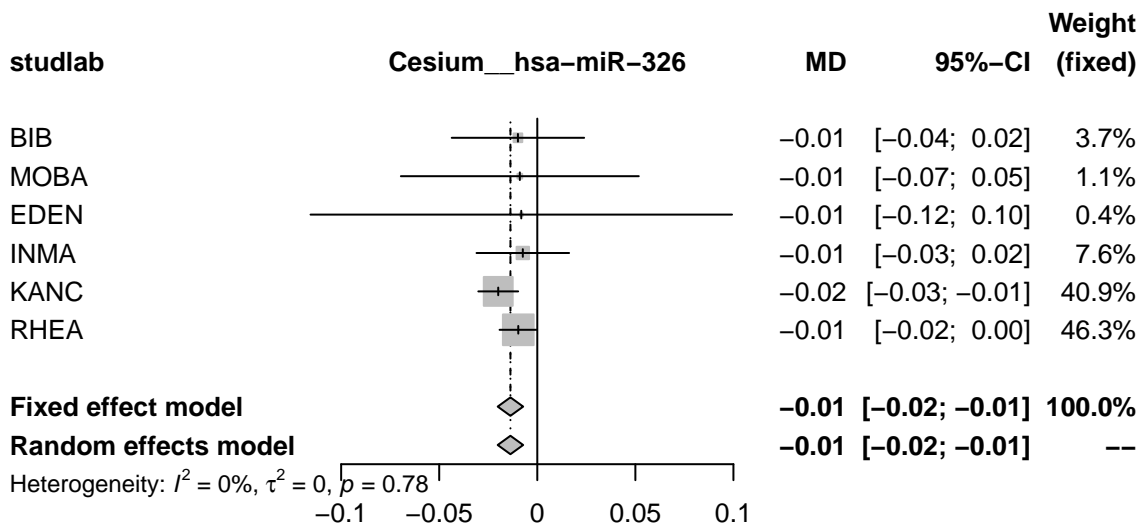

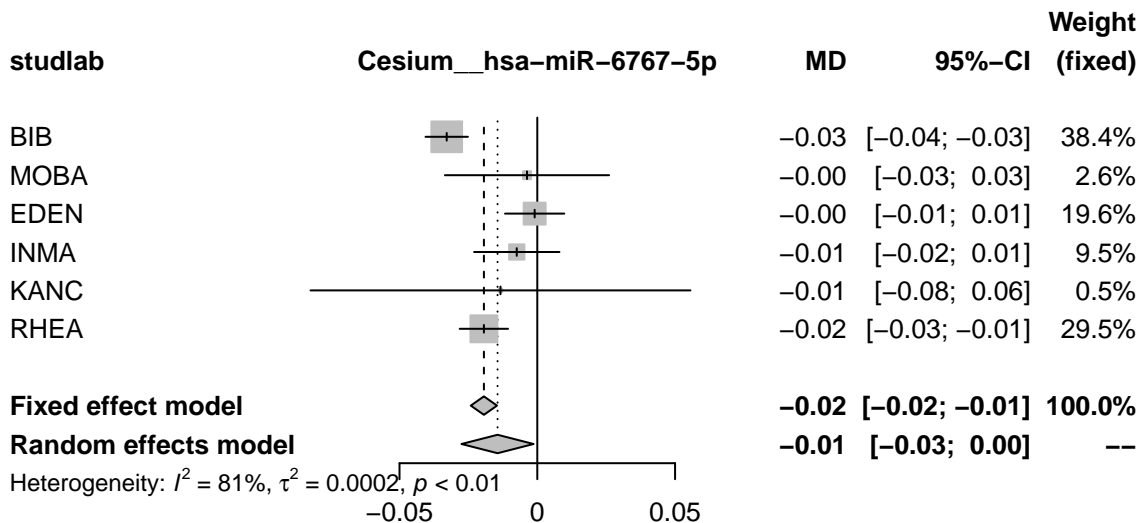

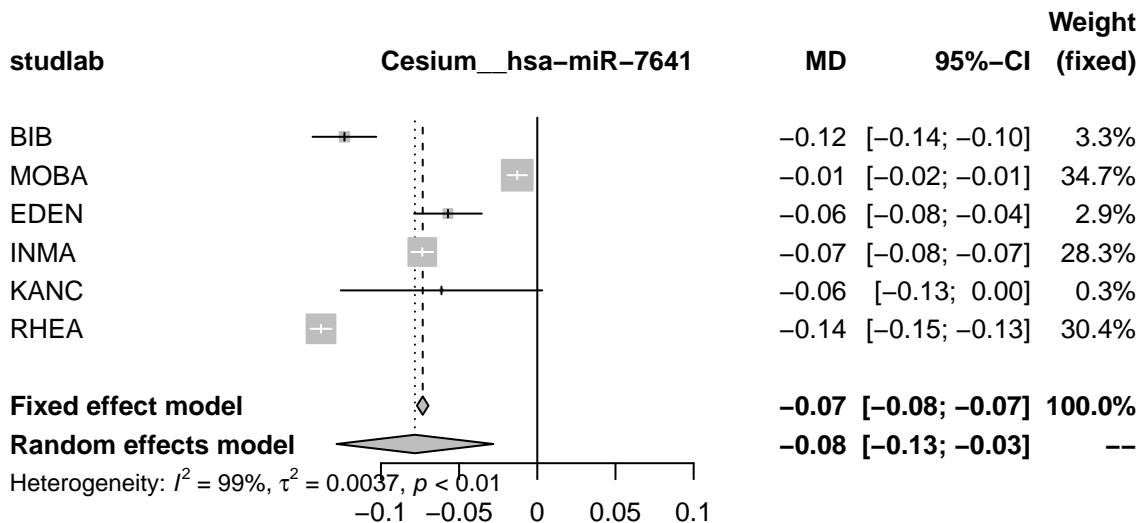

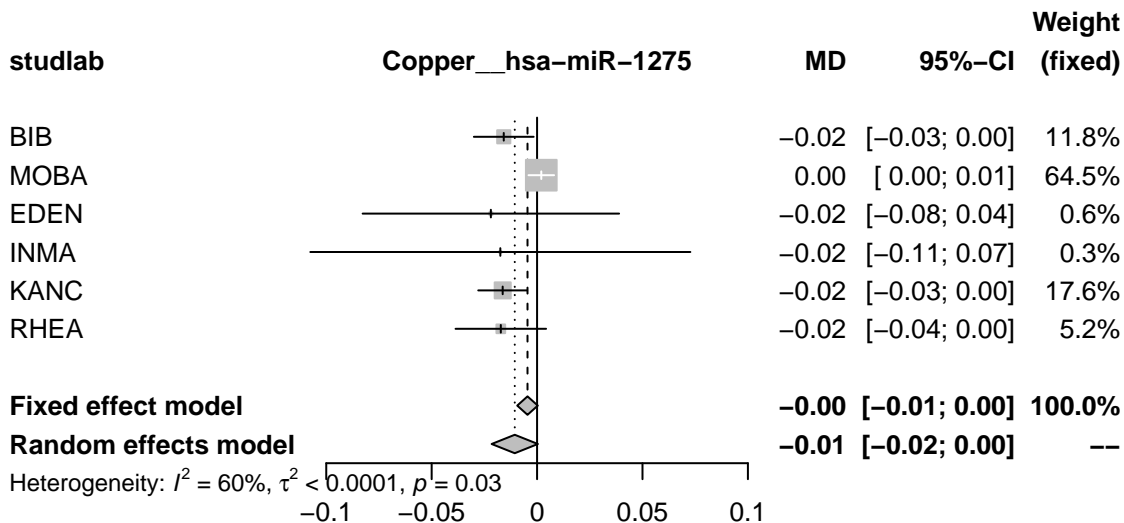

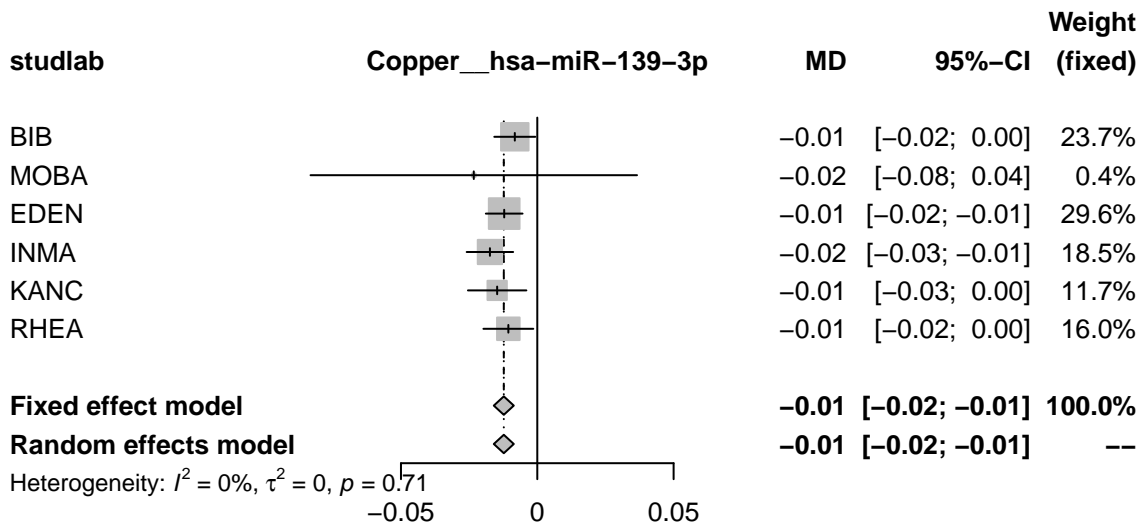

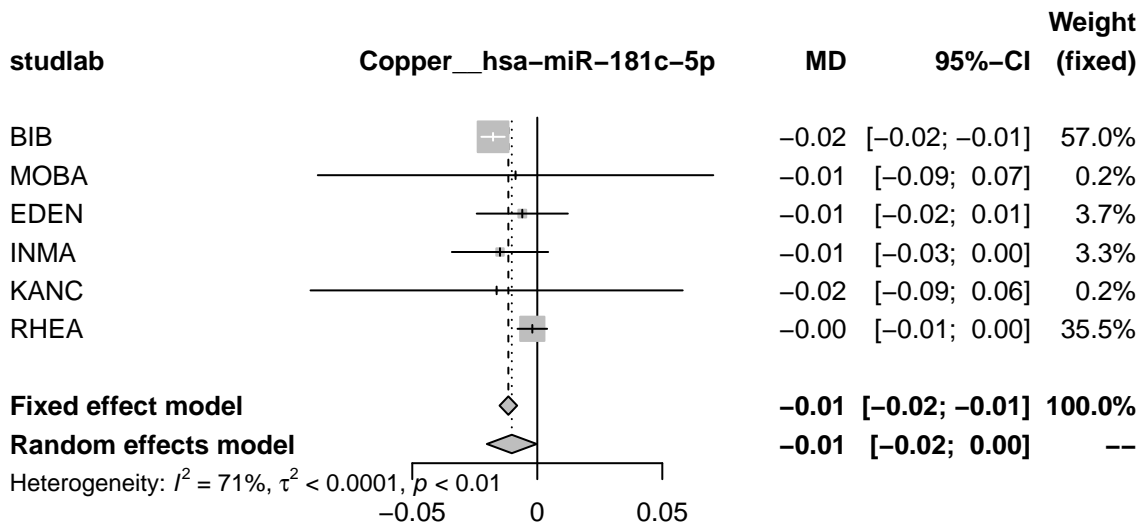

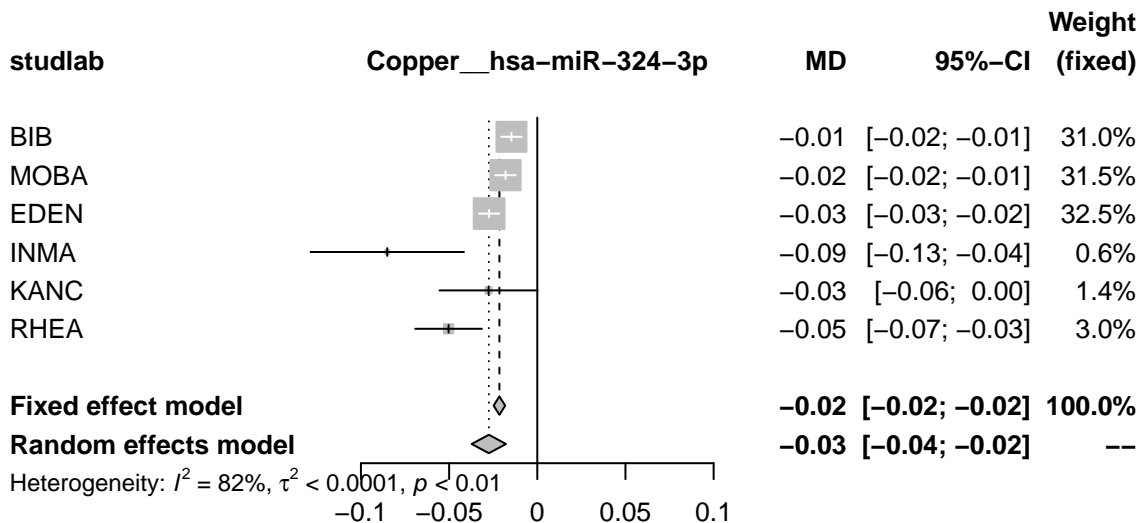

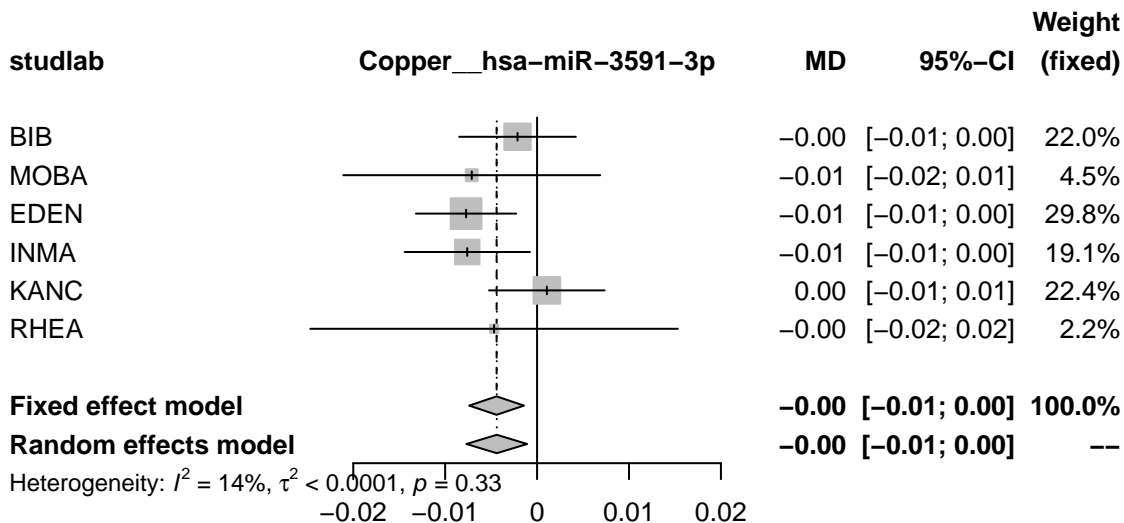

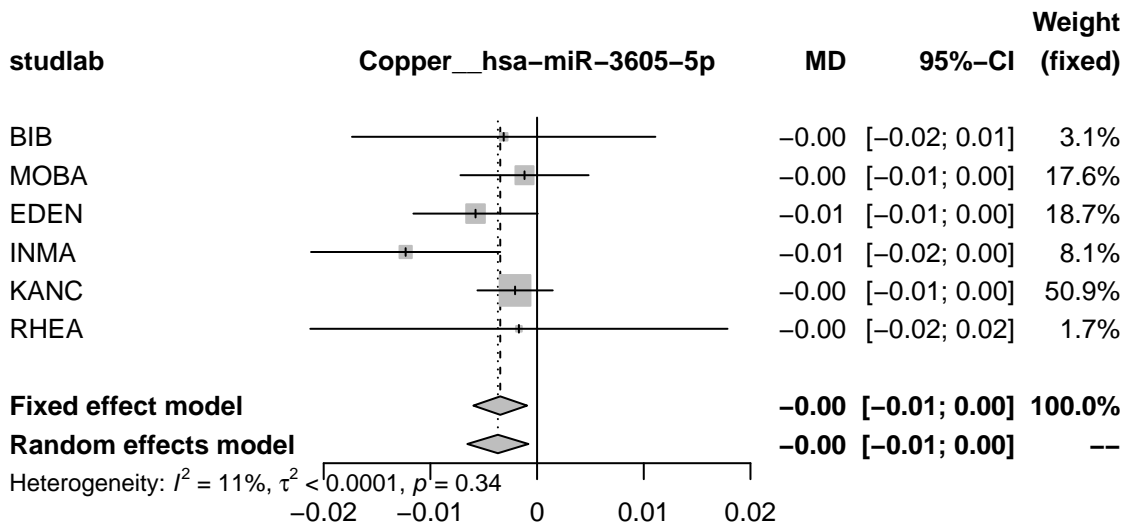

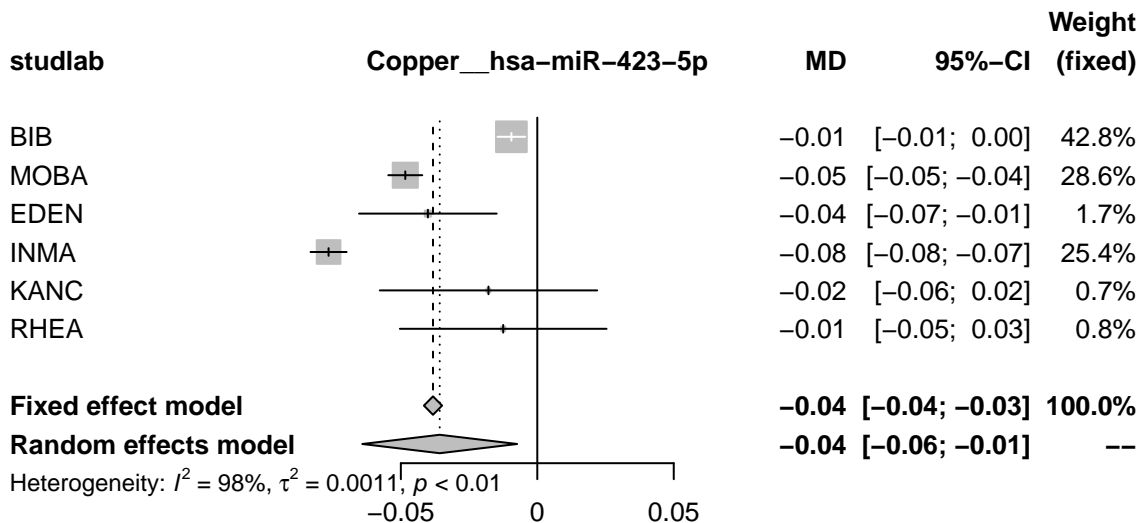

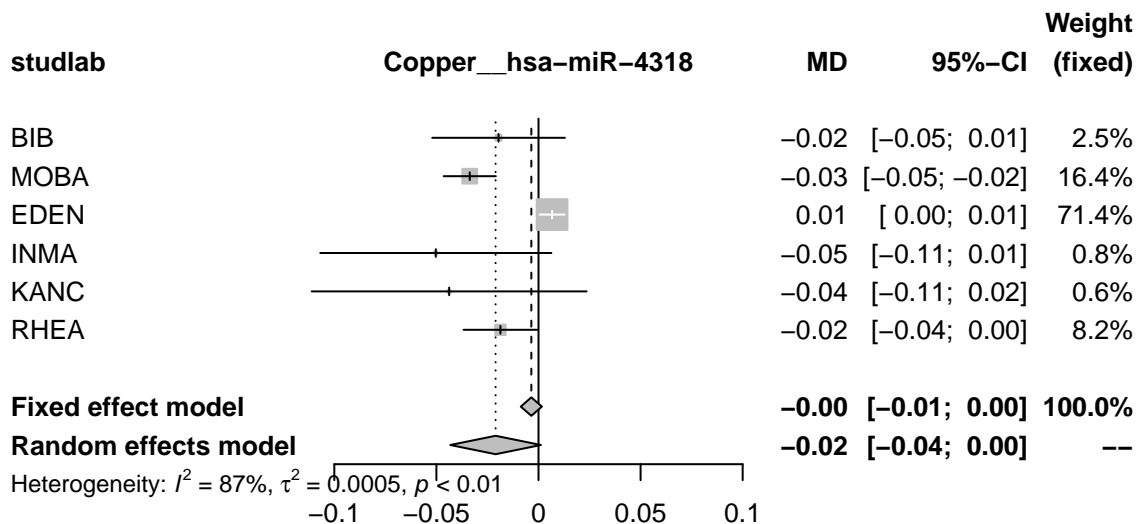

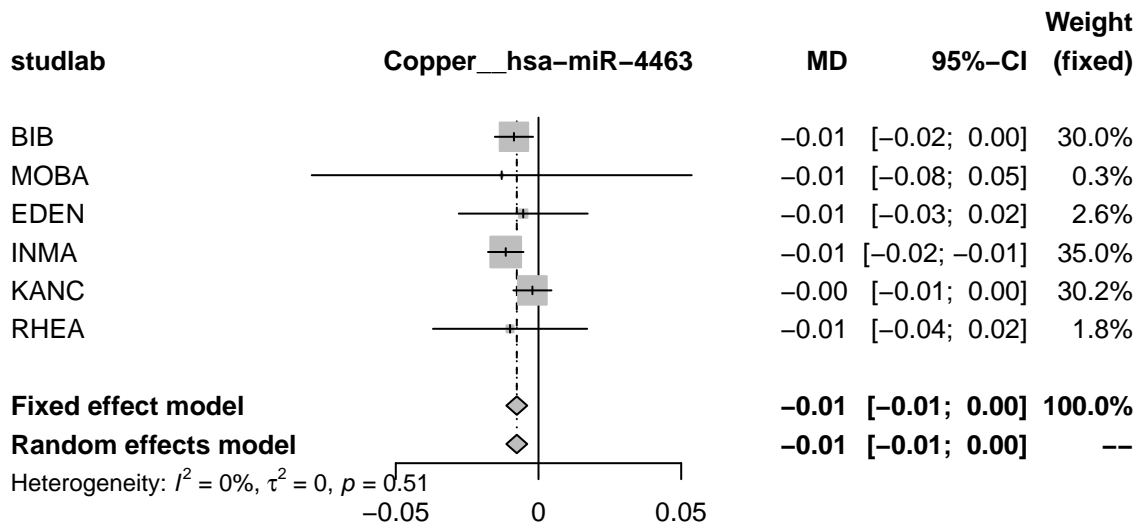

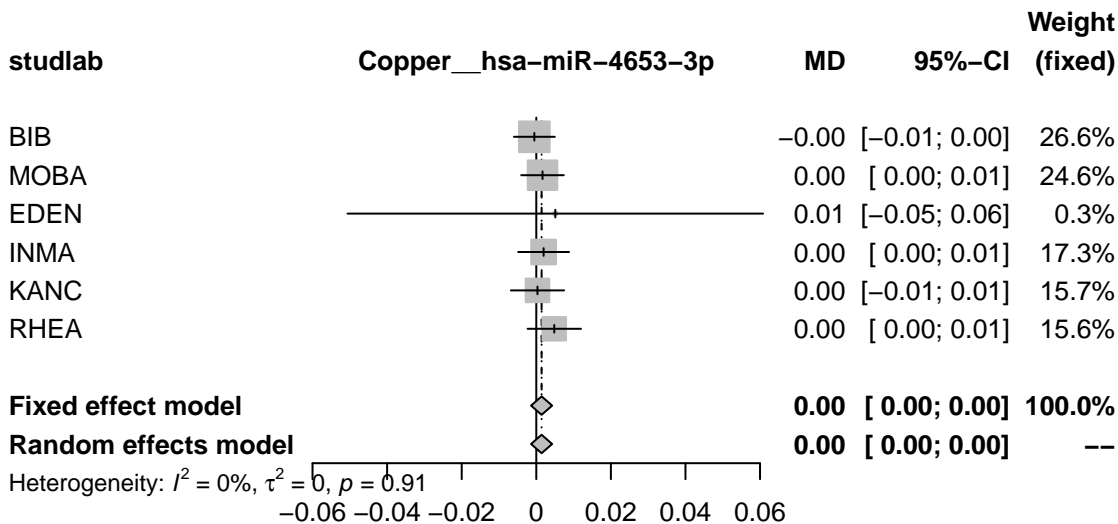

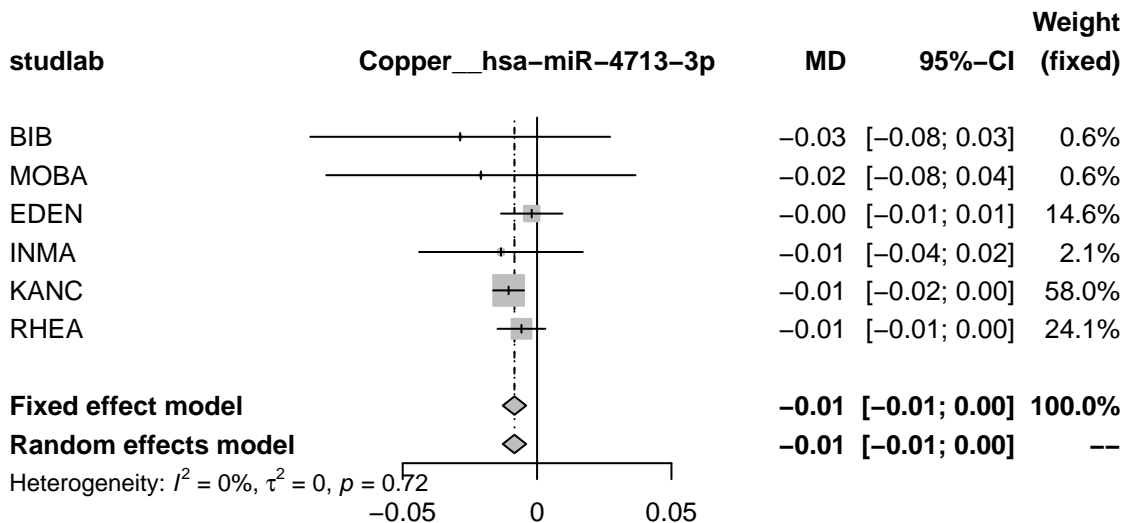

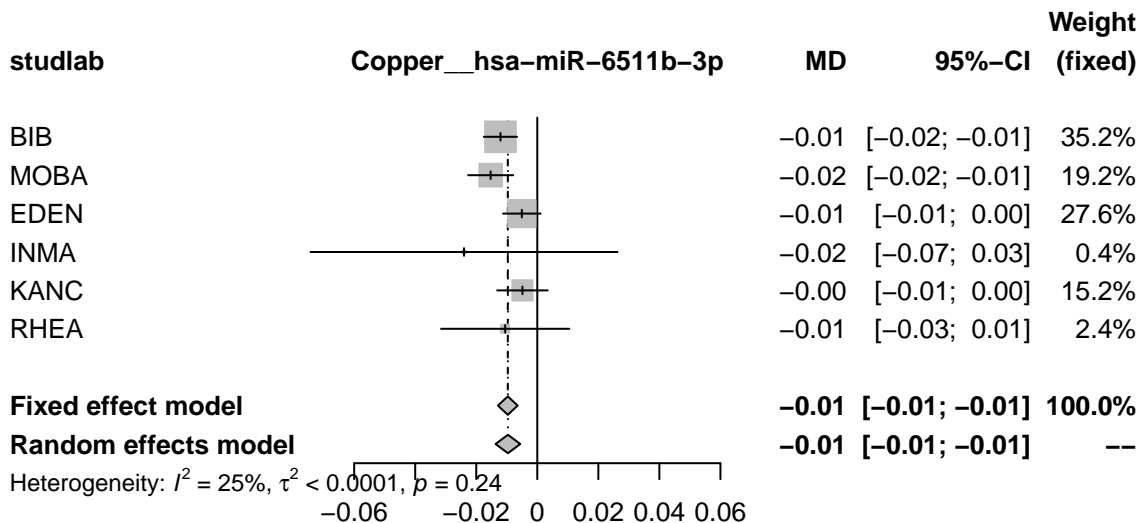

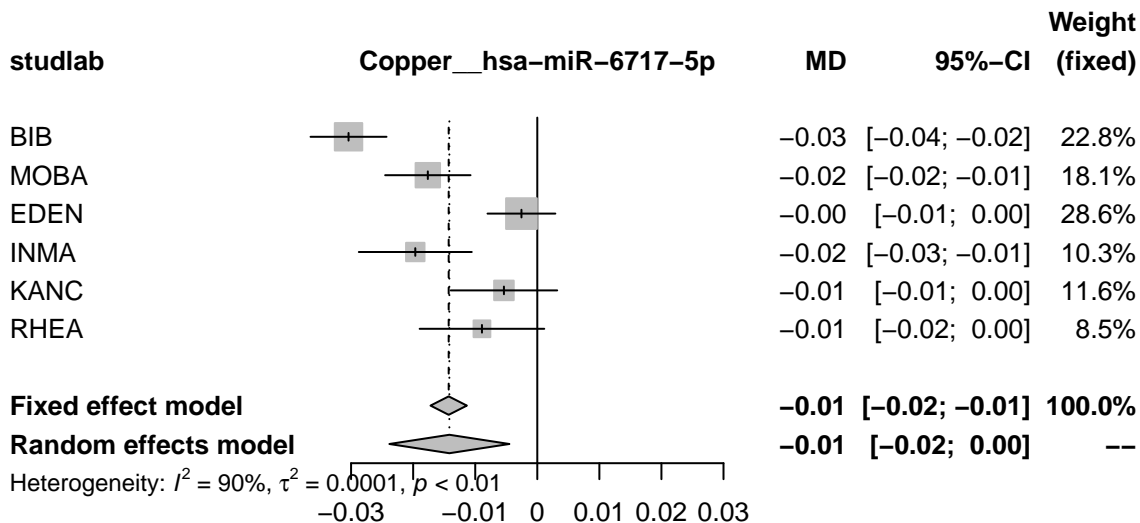

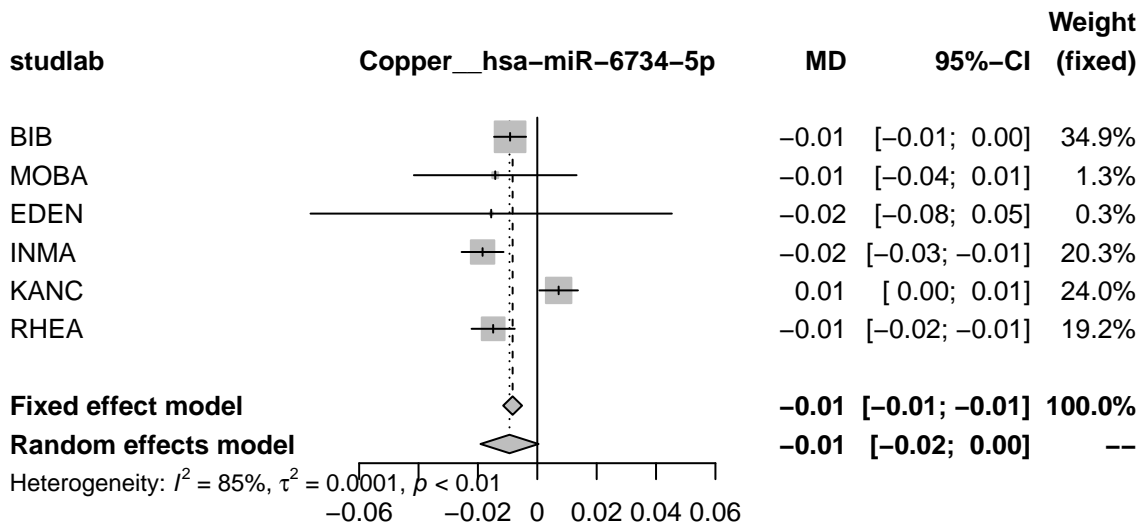

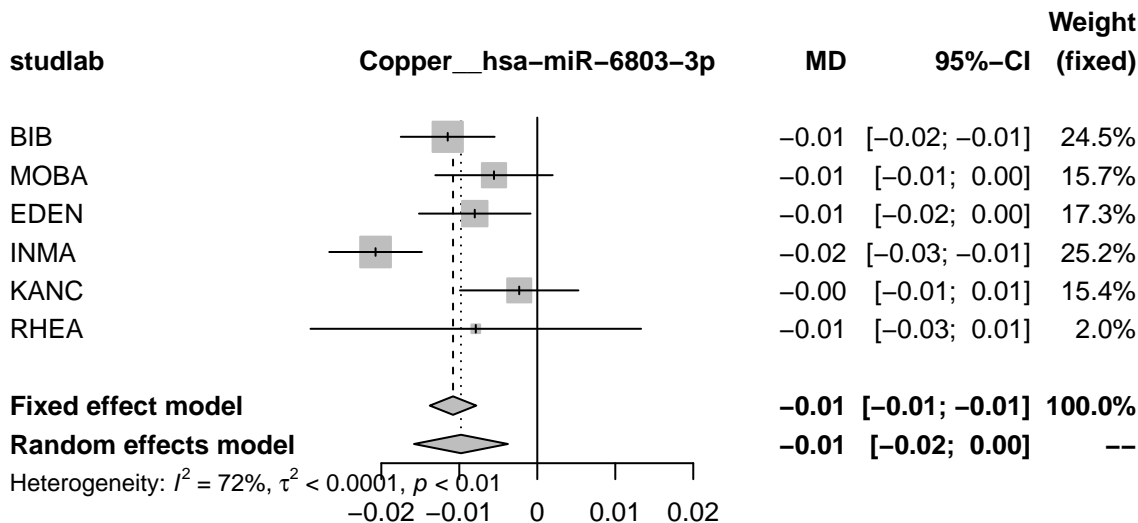

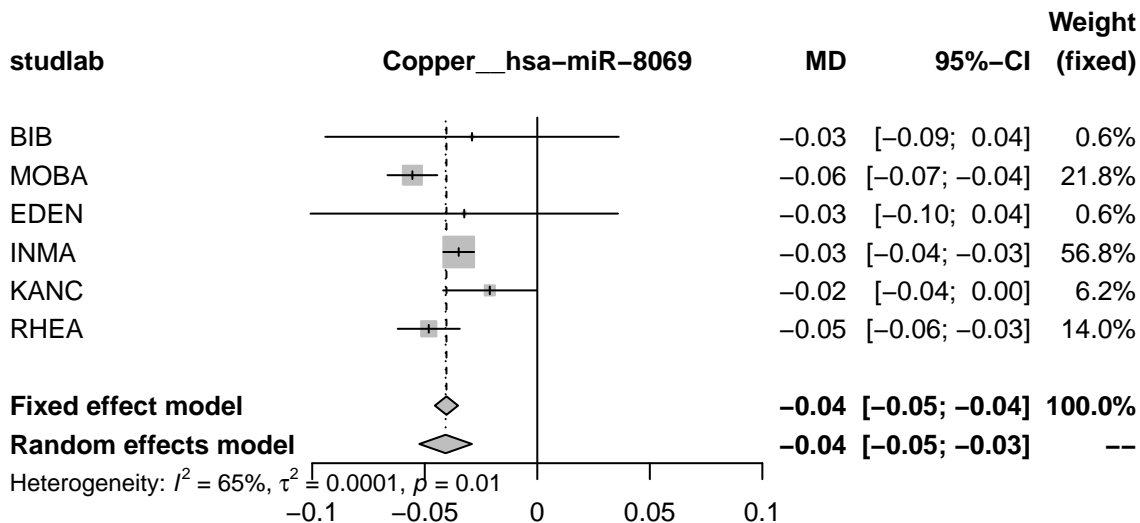

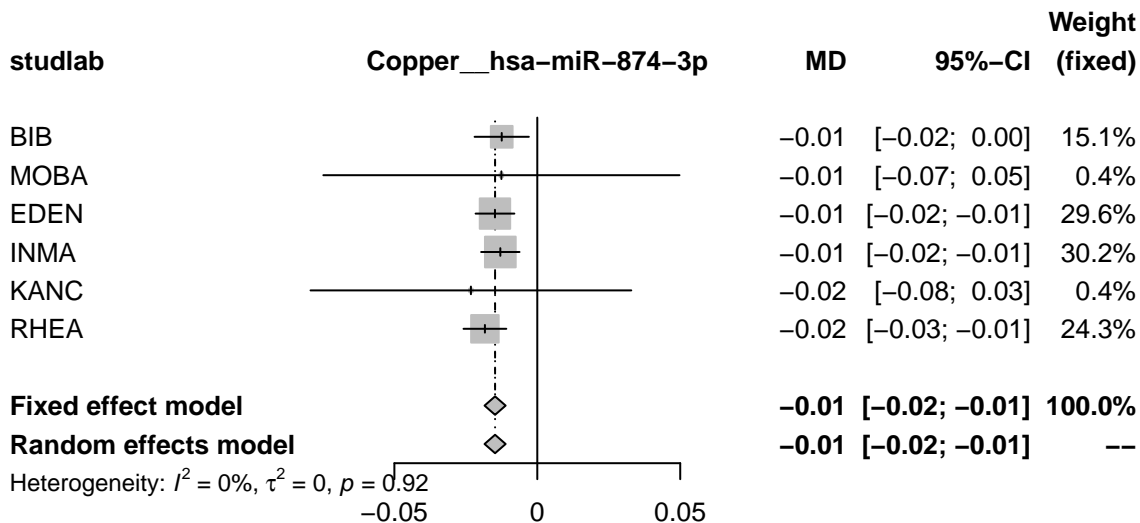

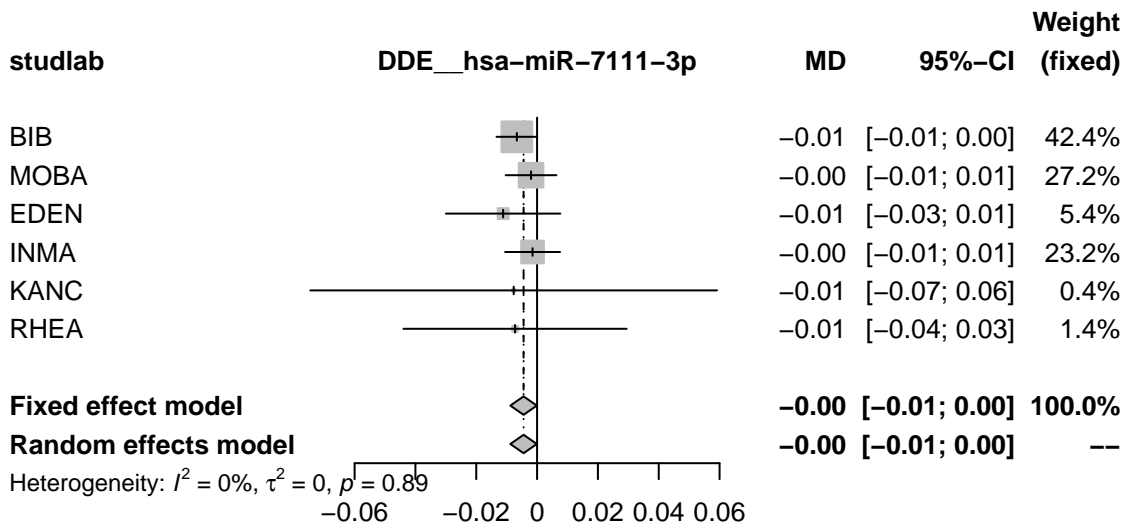

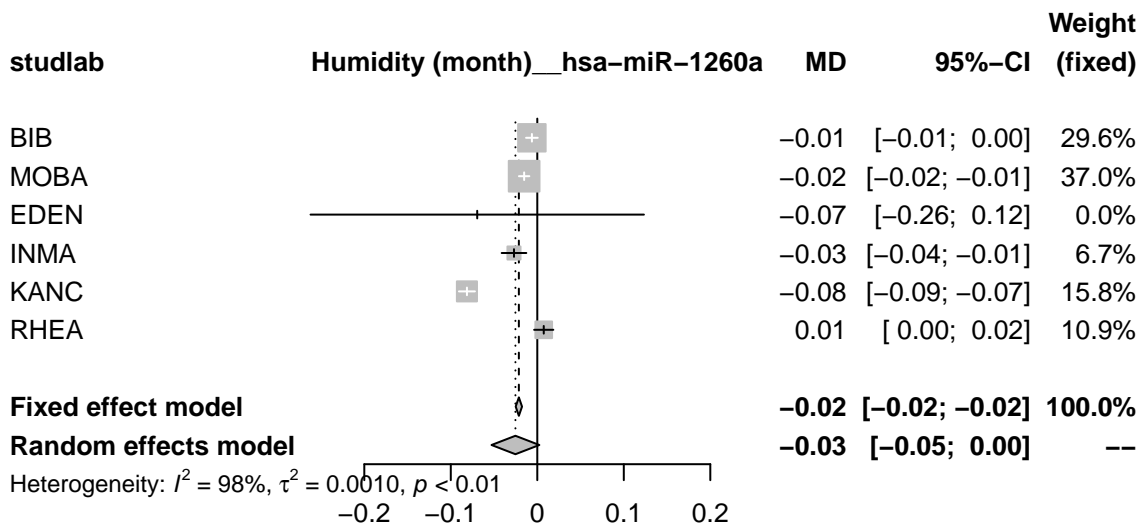

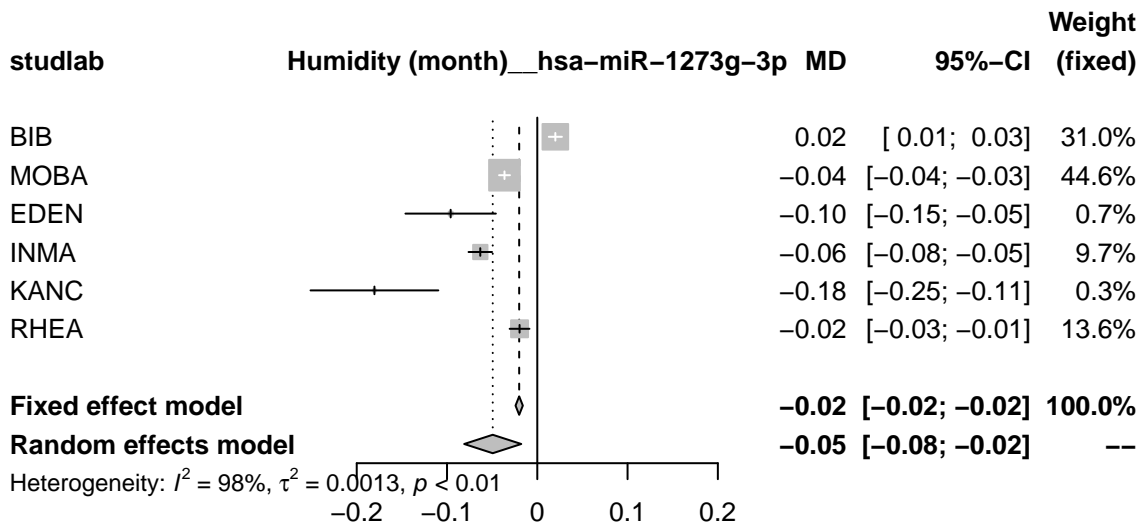

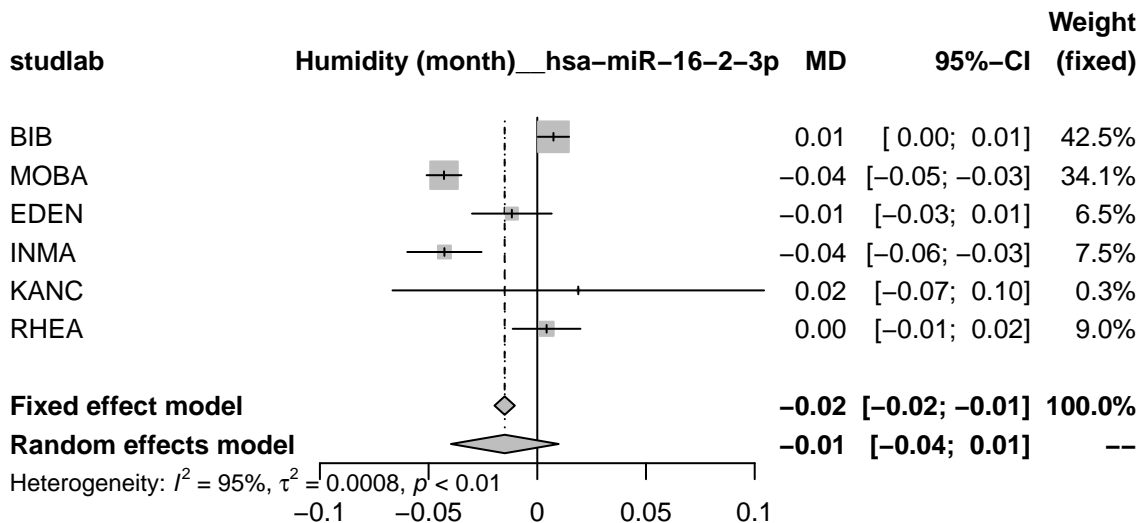

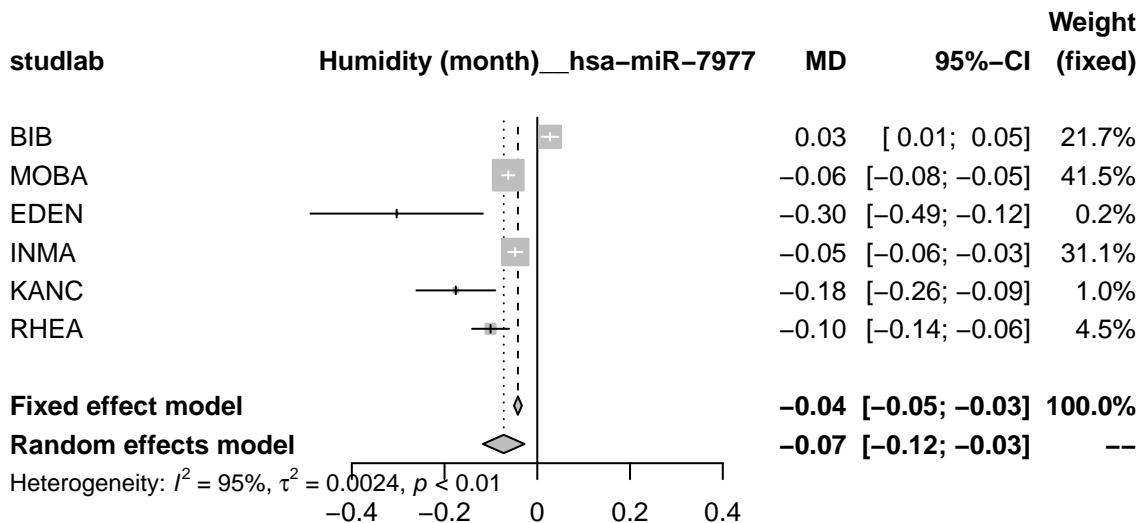

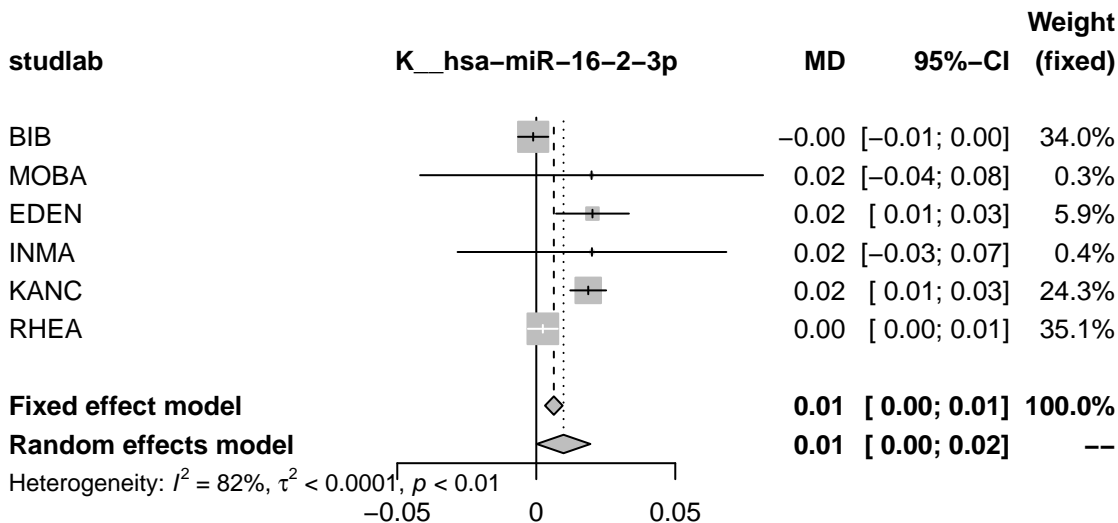

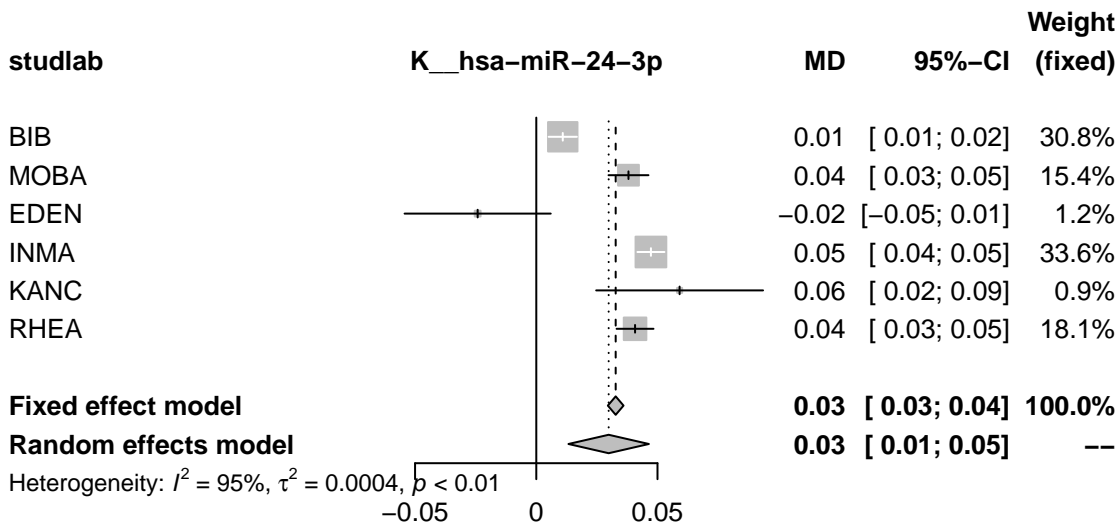

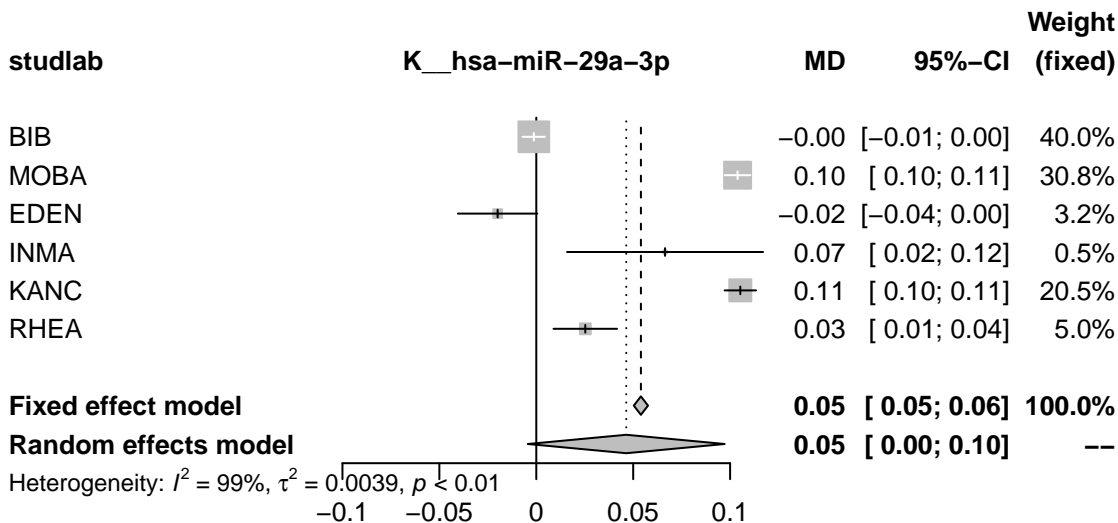

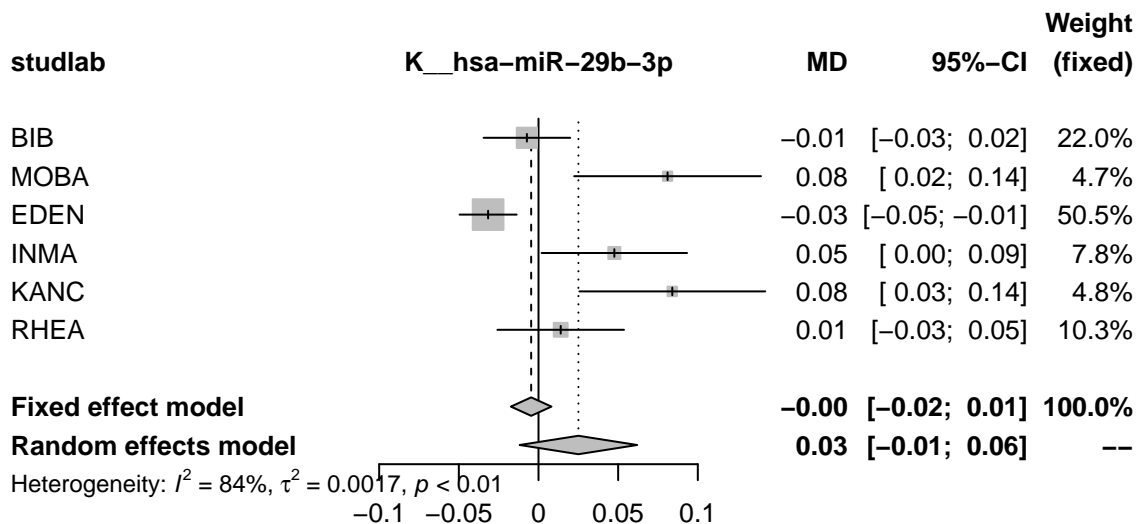

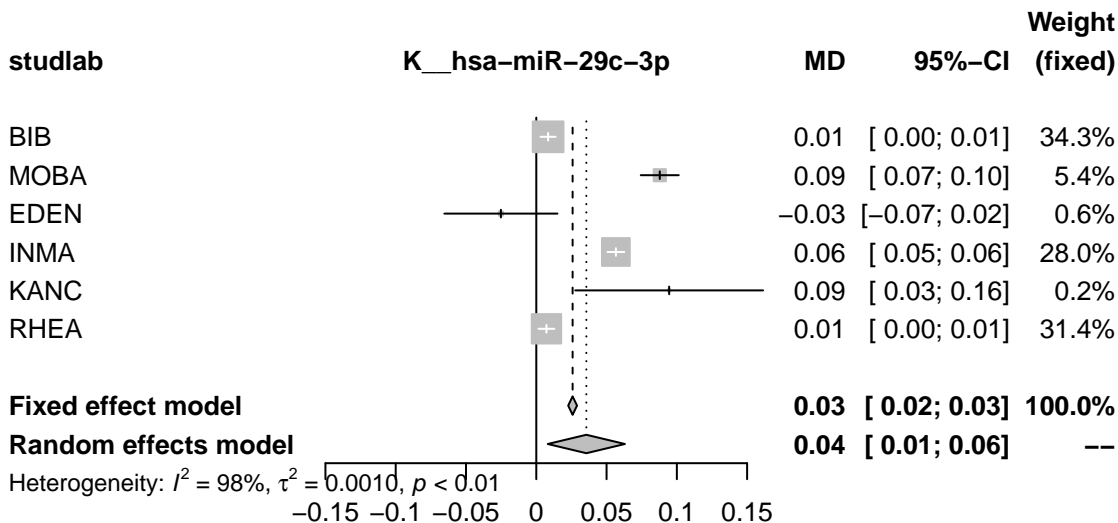

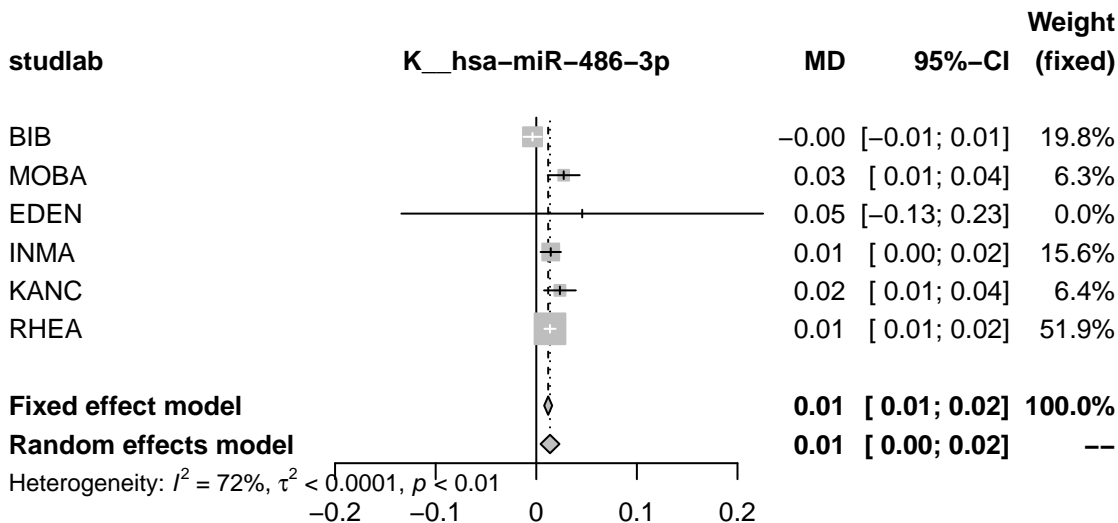

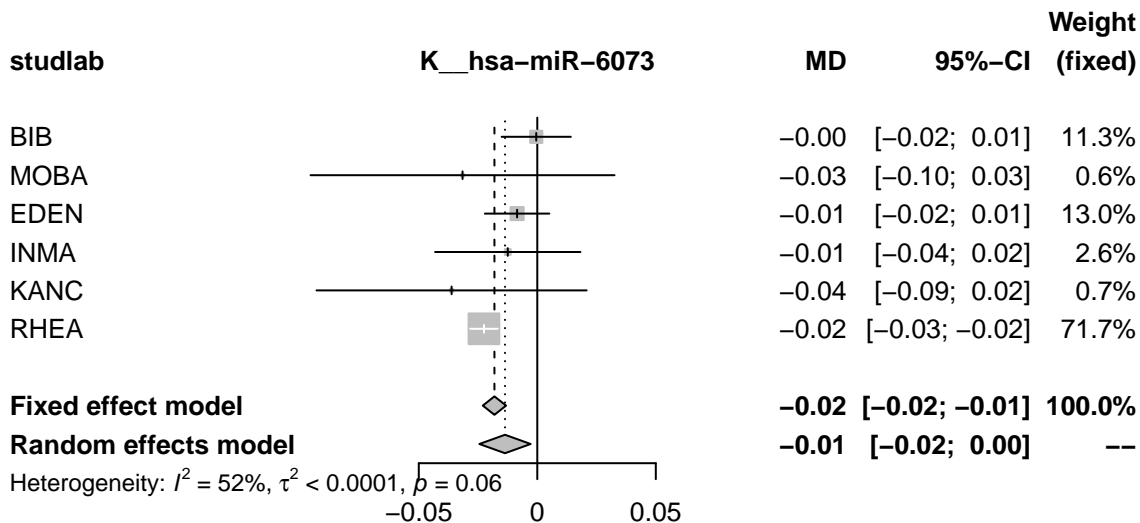

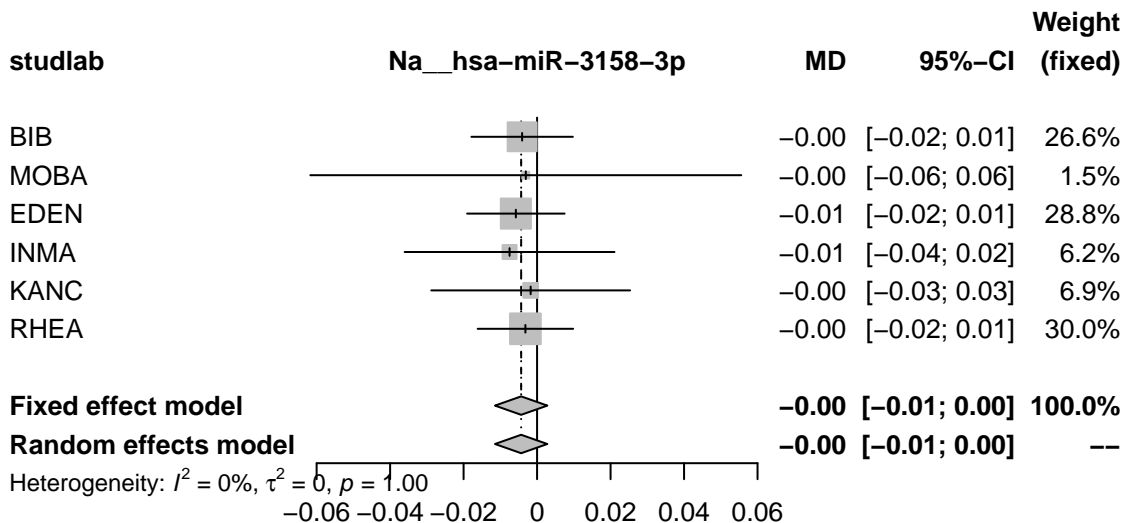

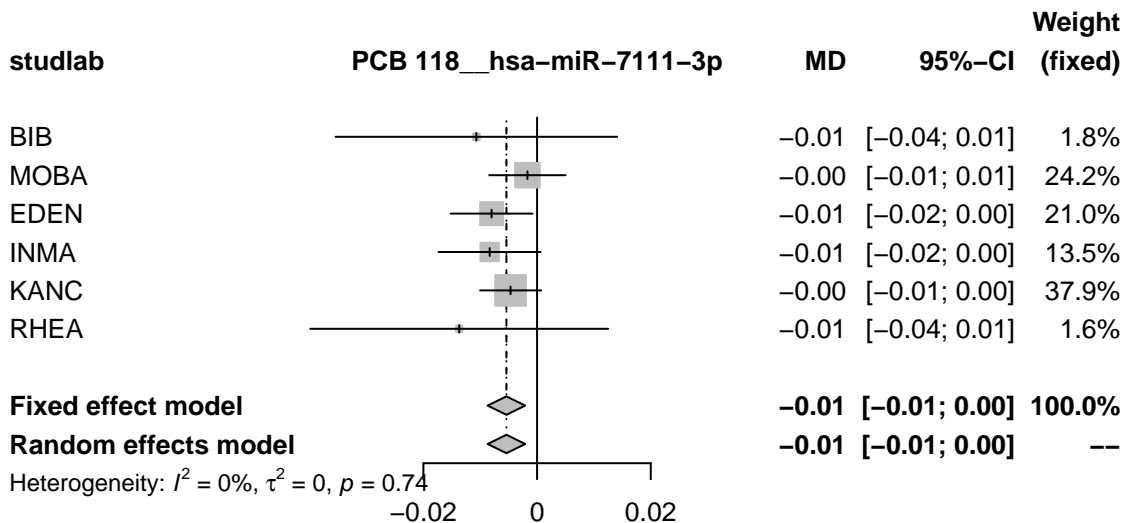

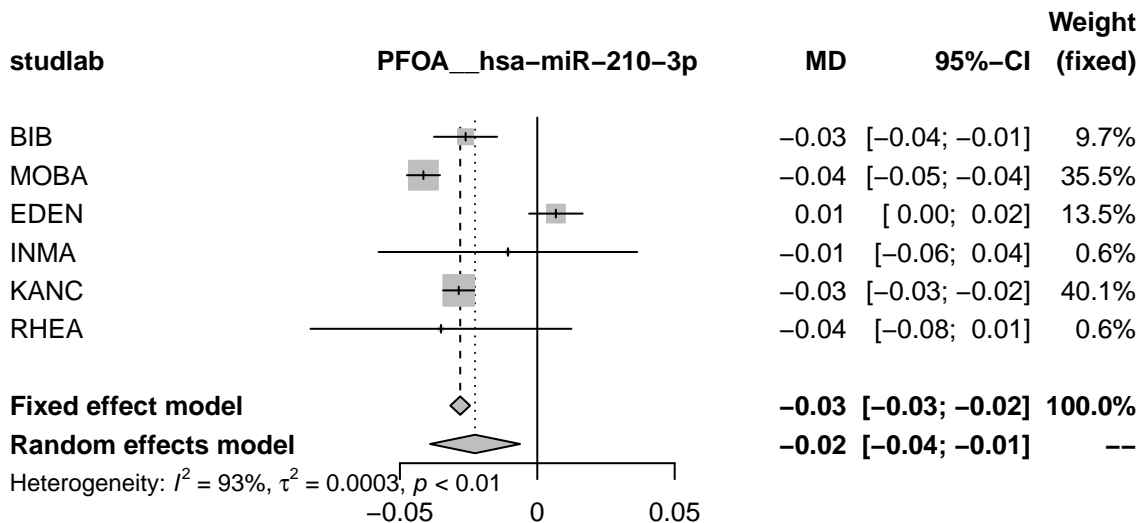

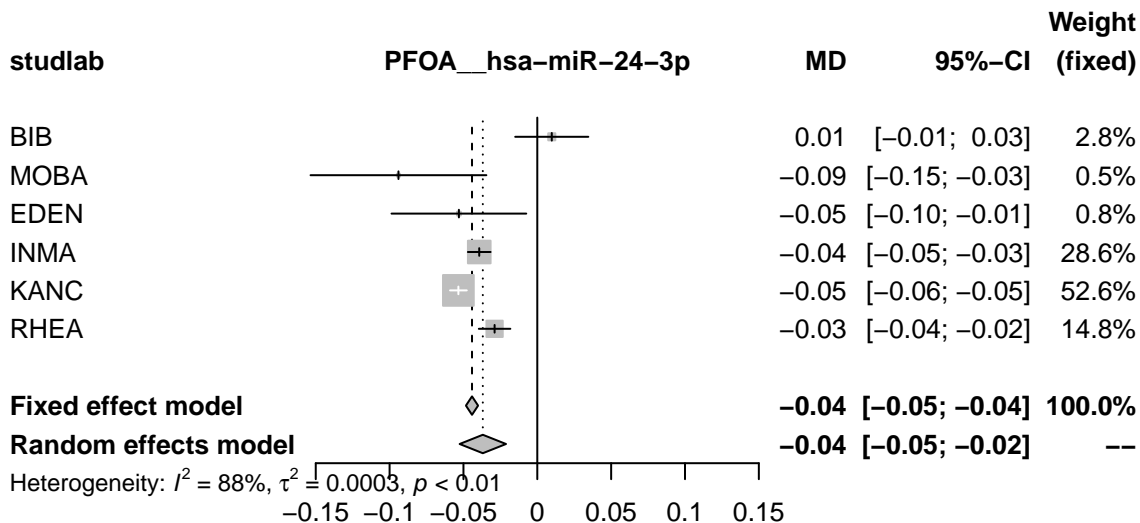

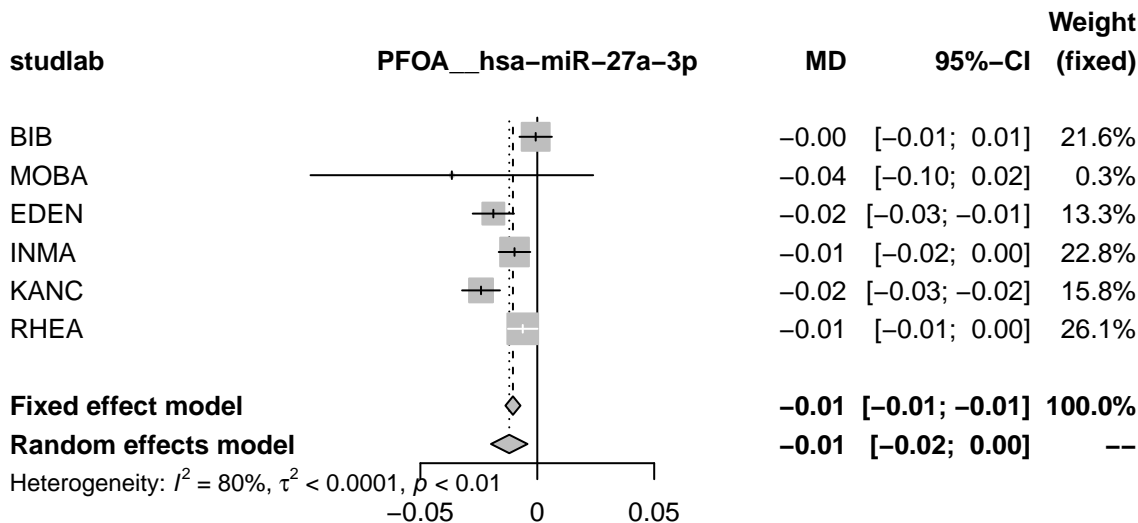

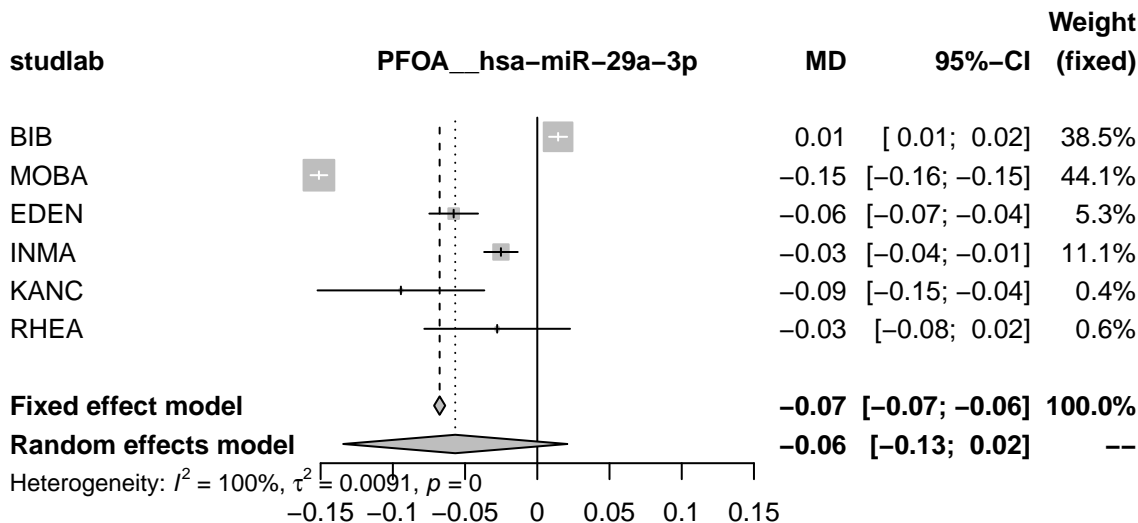

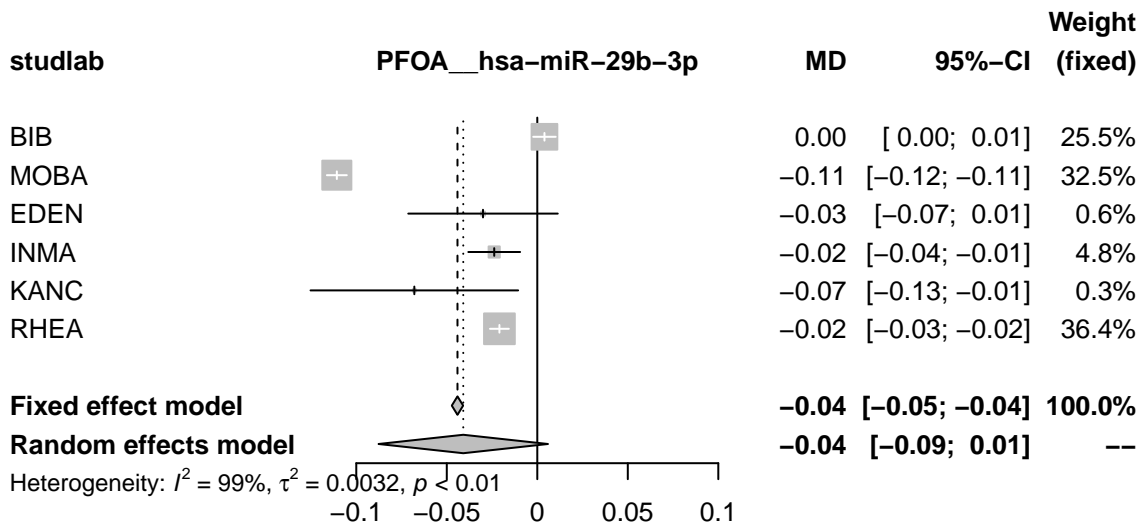

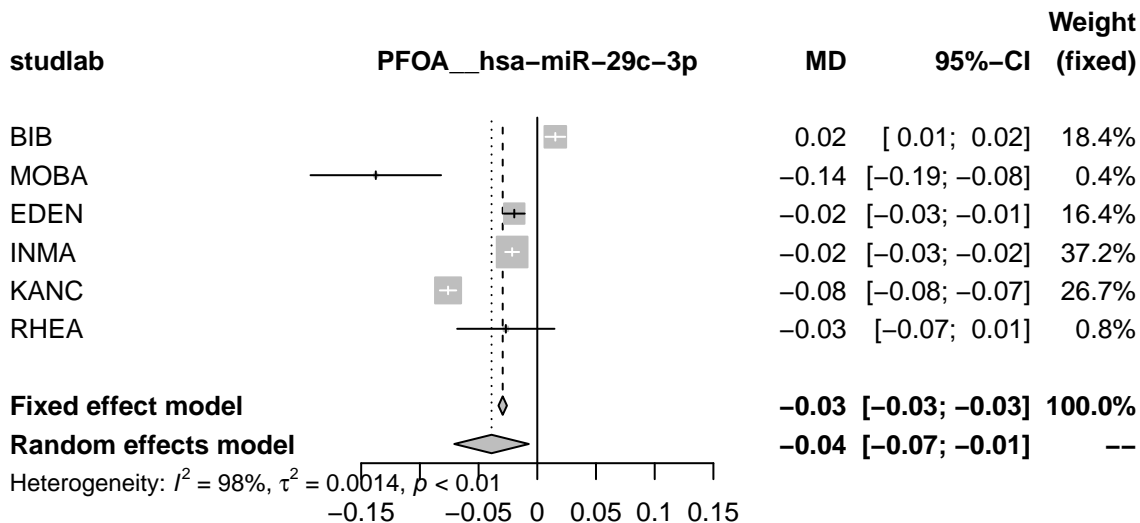

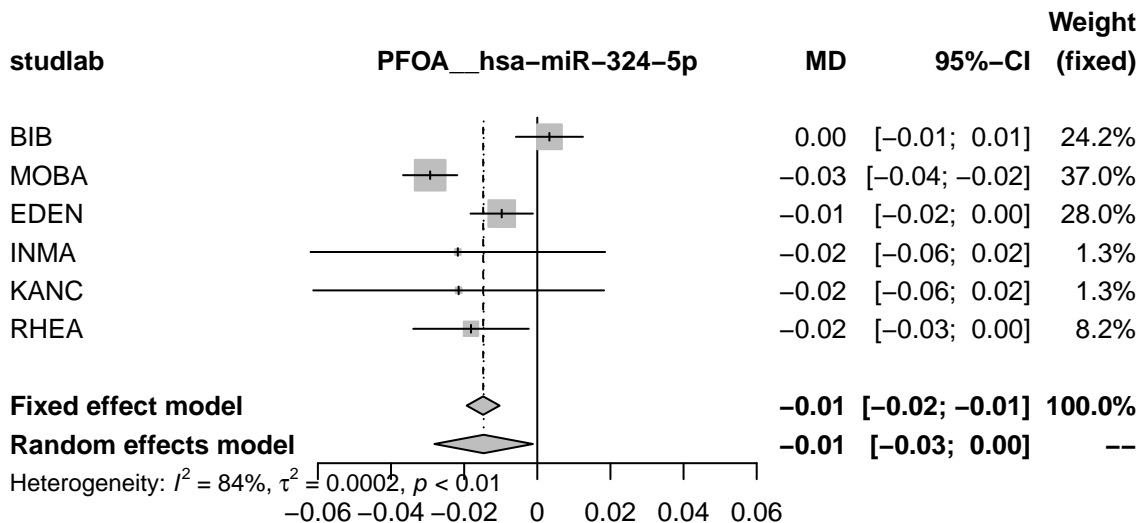

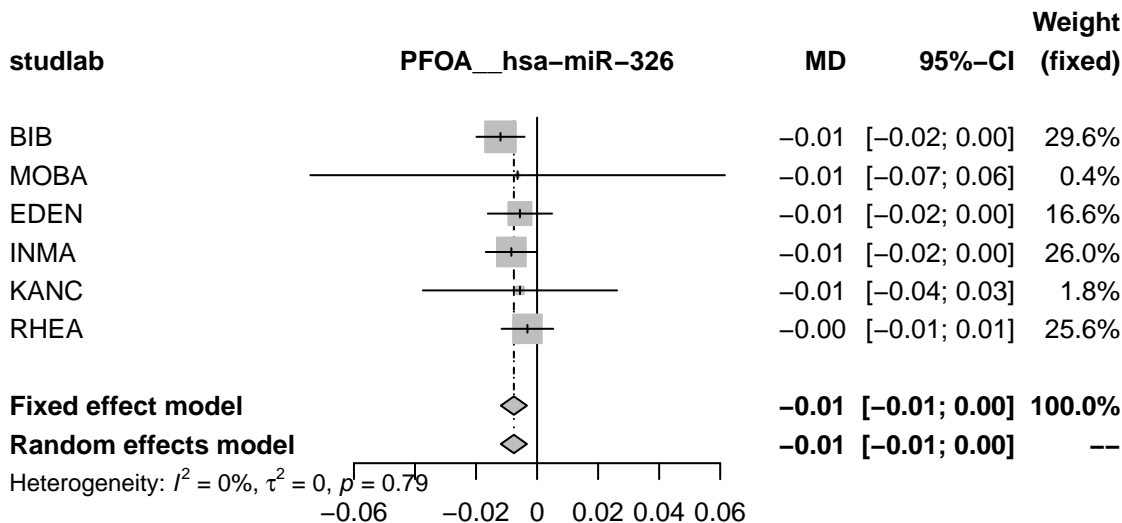

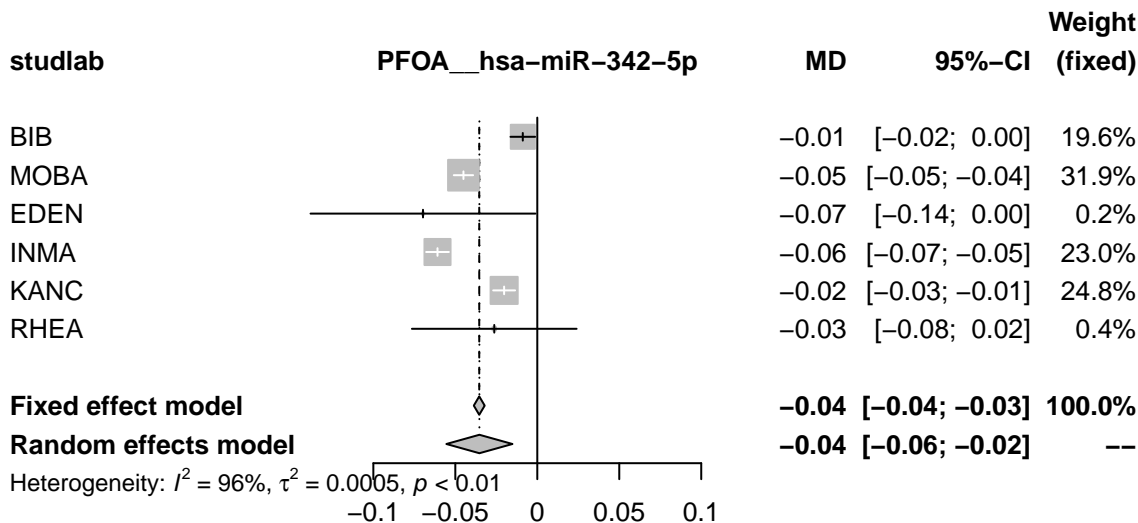

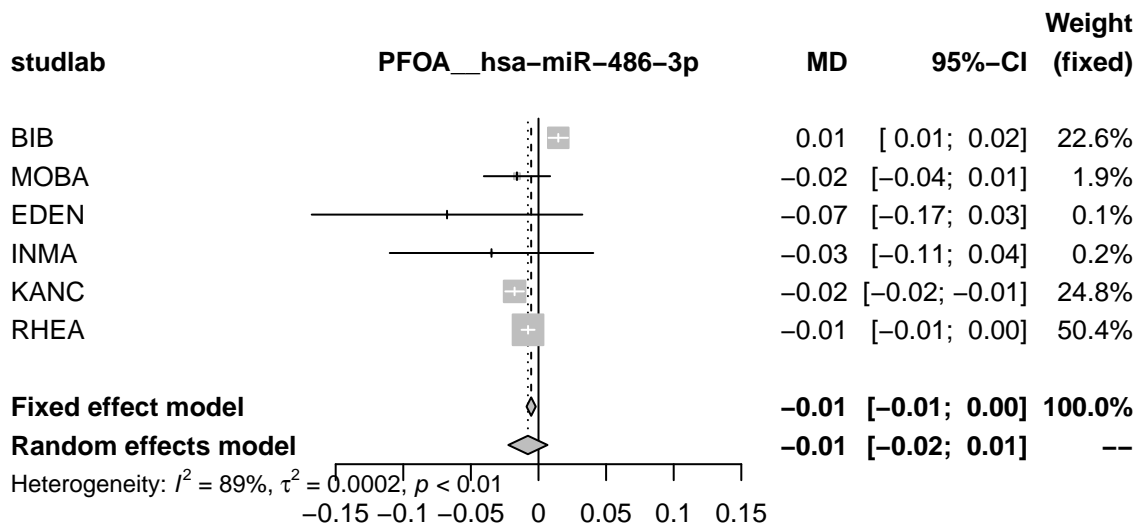

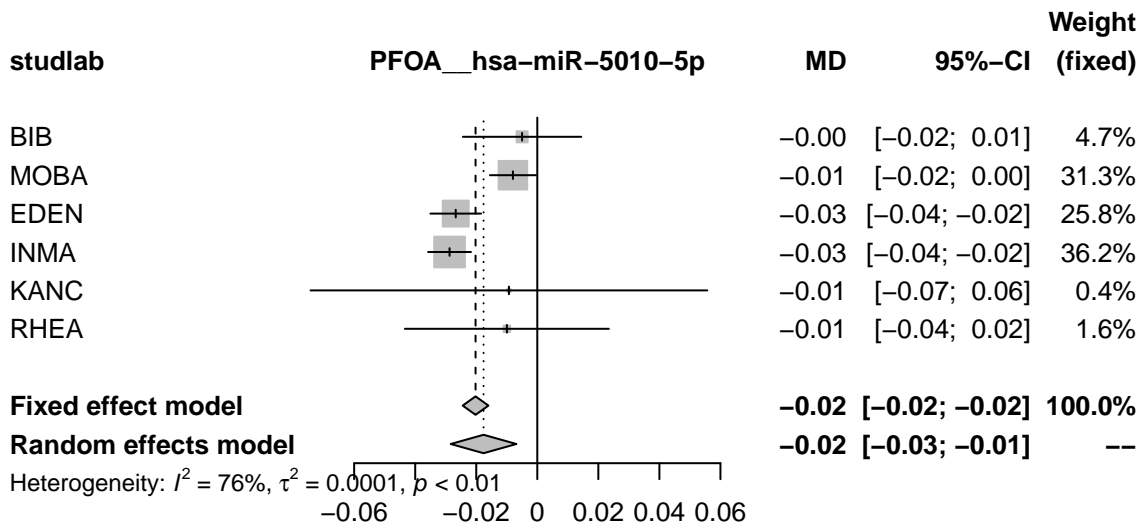

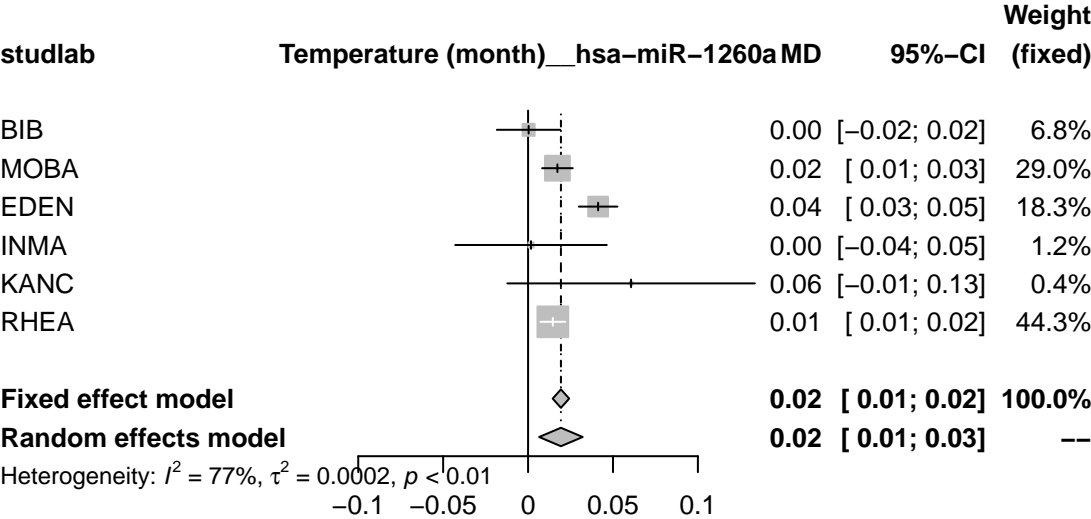

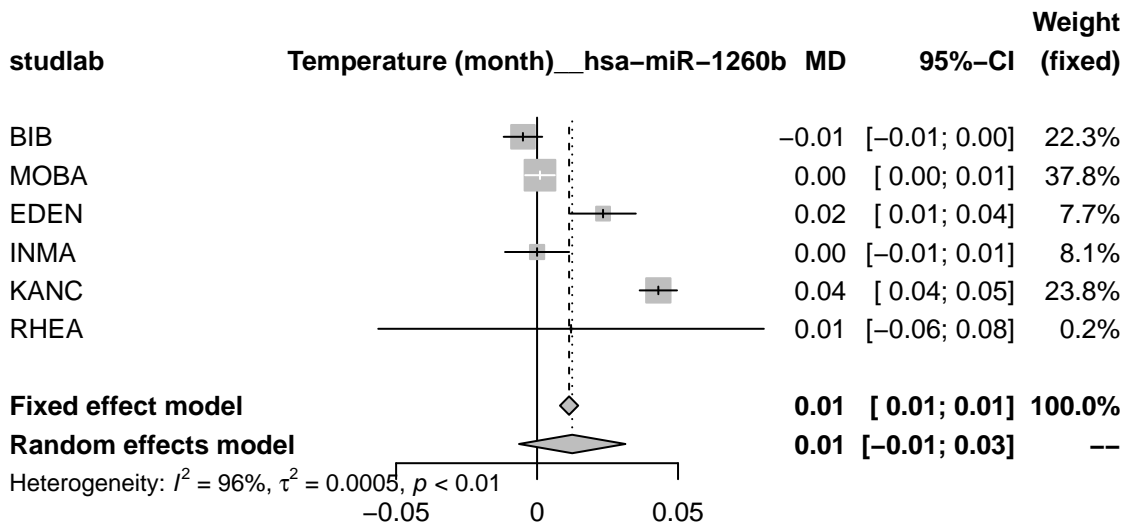

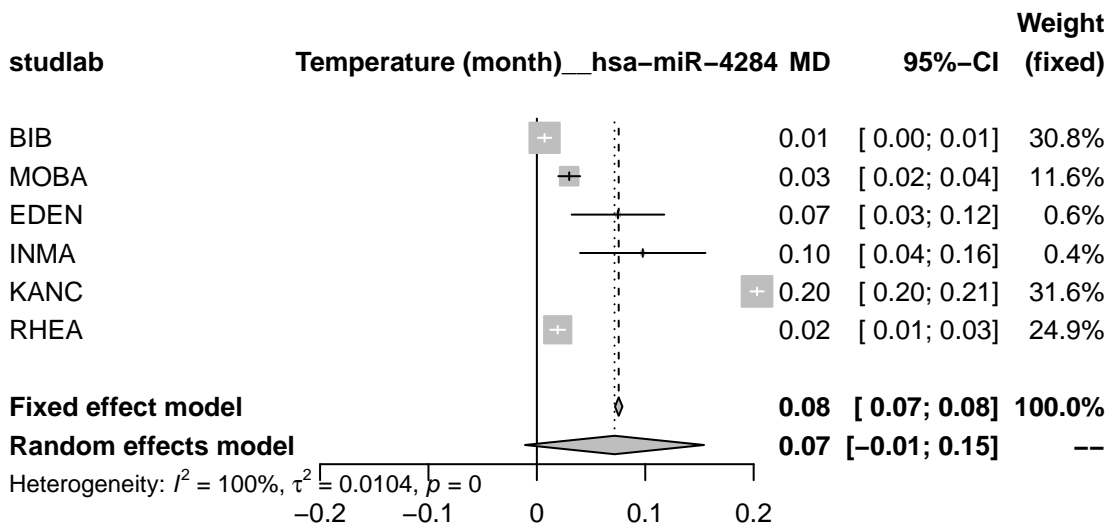

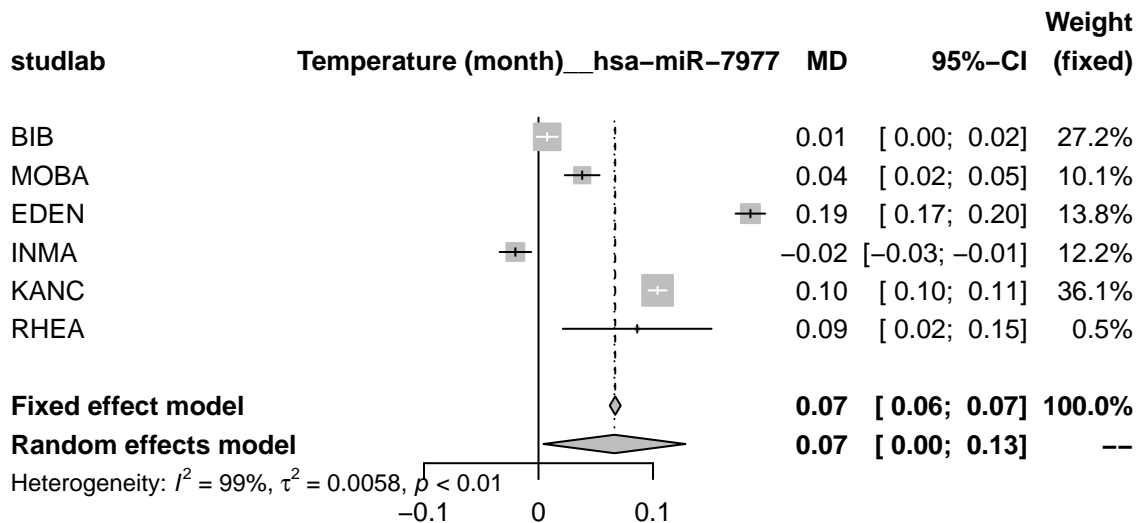

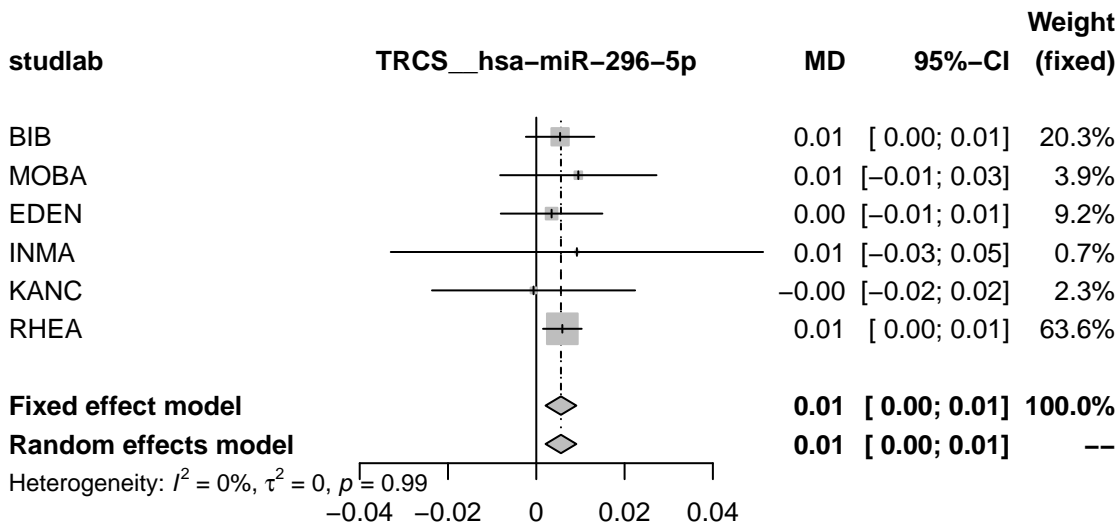

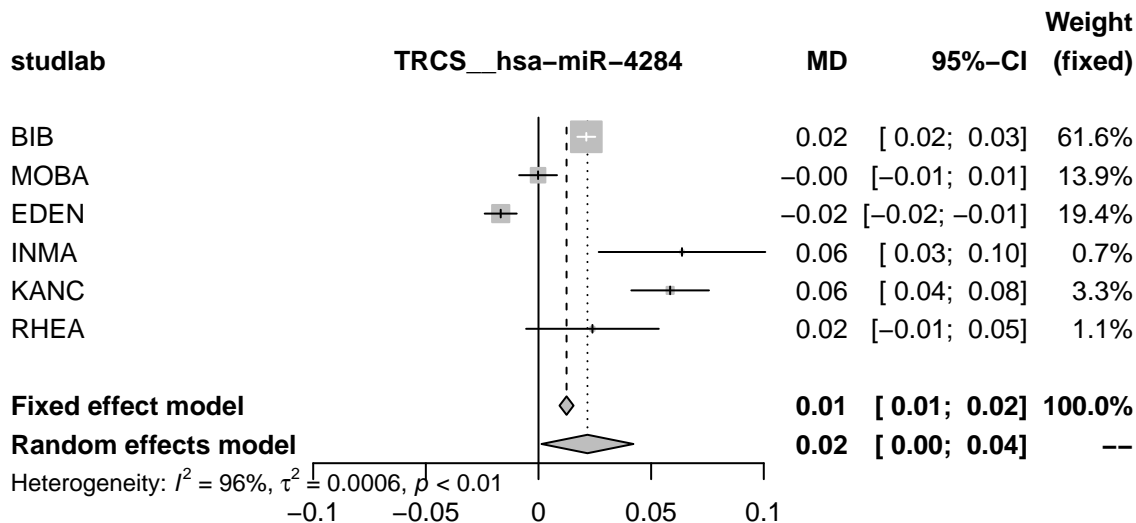

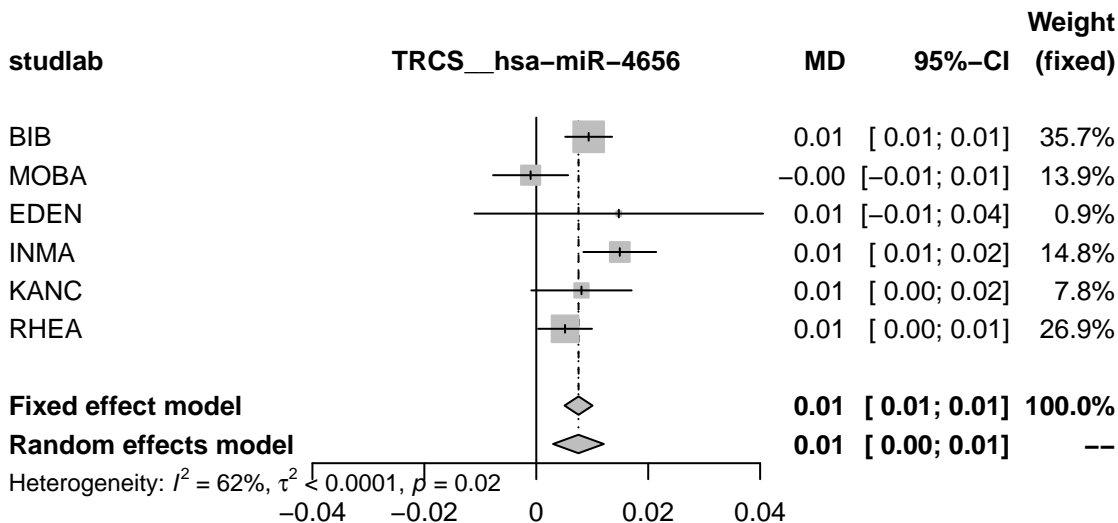

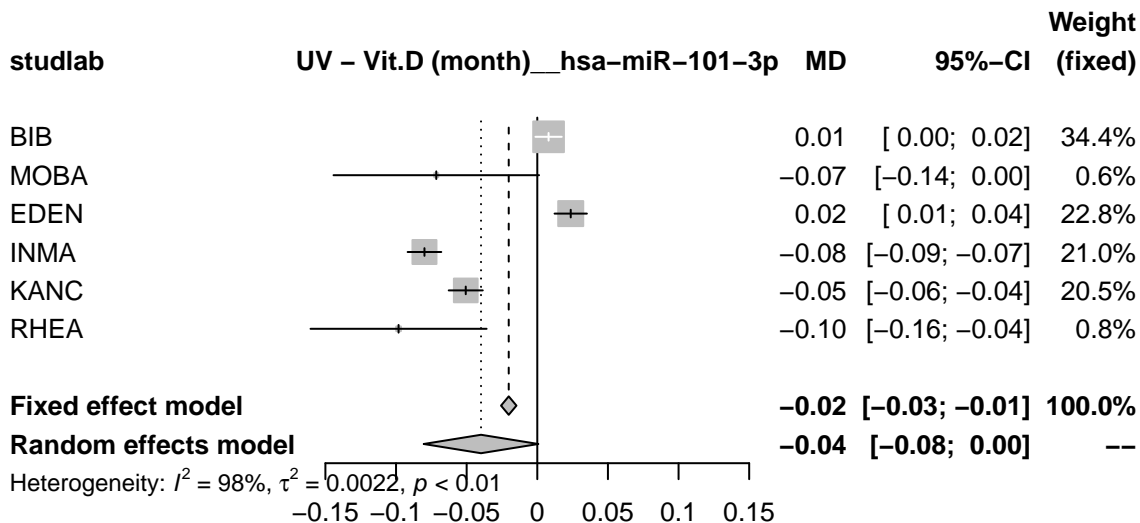

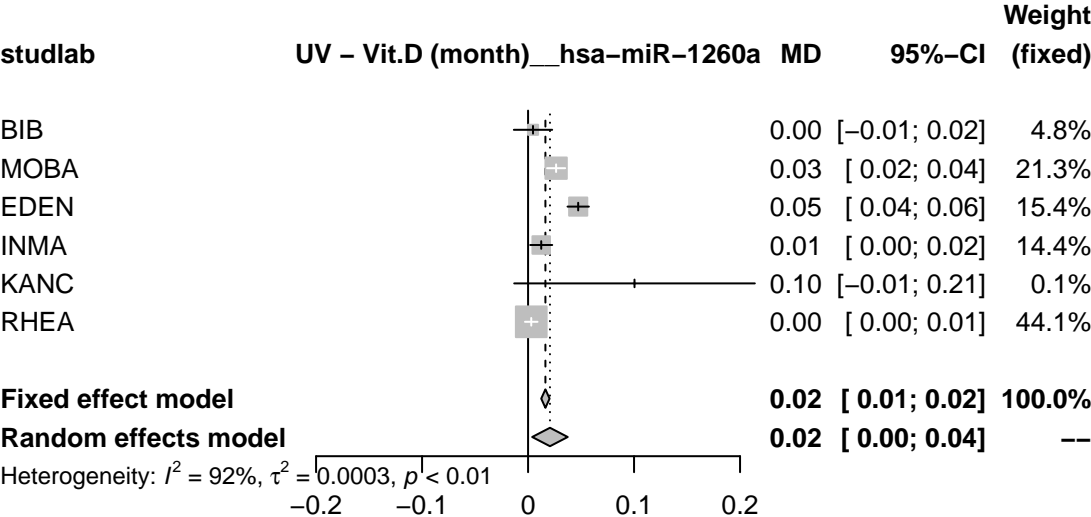

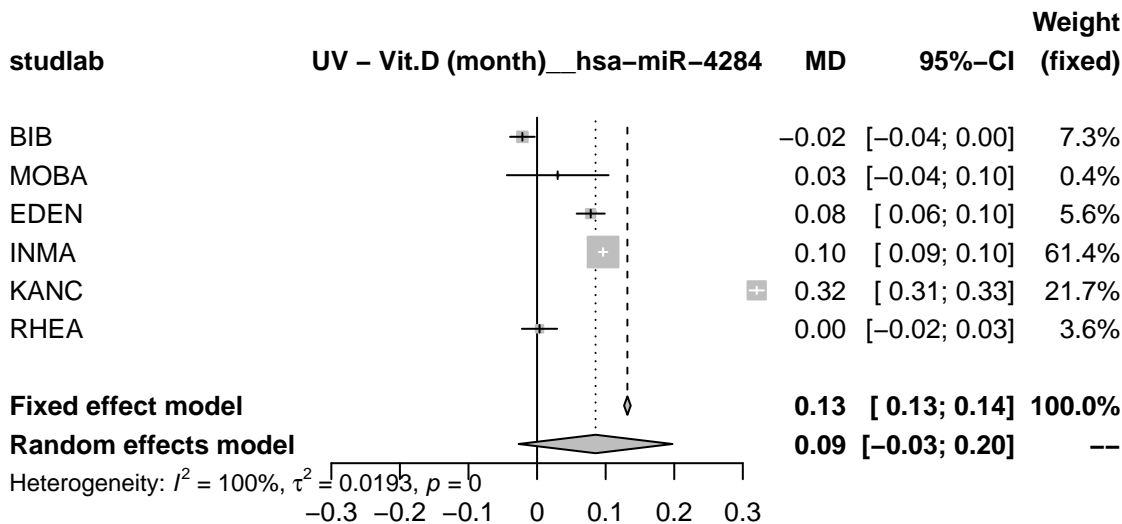

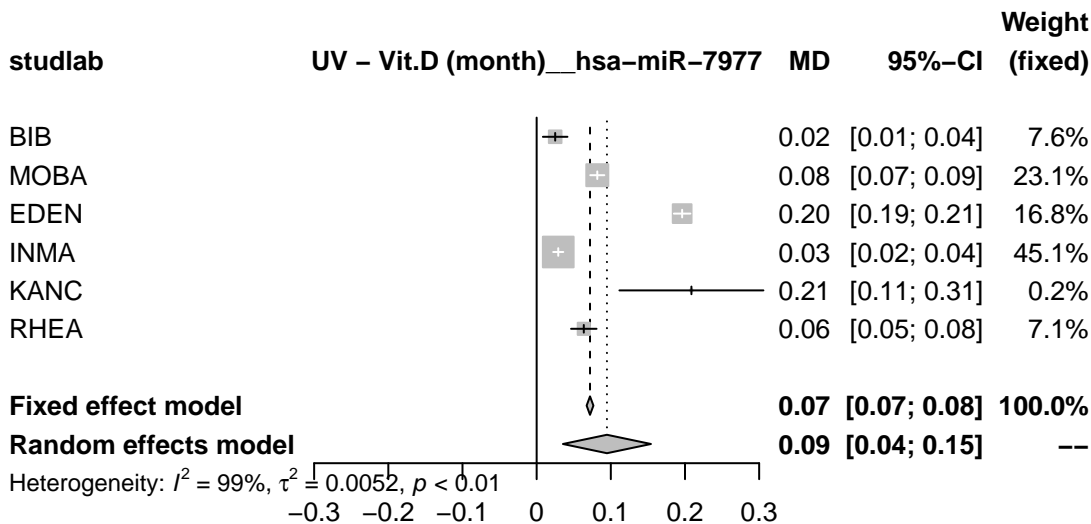

Supplement: Supplementary file 14 — Supplementary Dataset 11 [file 41467_2022_34422_MOESM14_ESM.zip › HELIX_ExpOmics_FigS2_Forestplots/HELIX_ExpOmics_FigS2I_mirna_post.pdf]

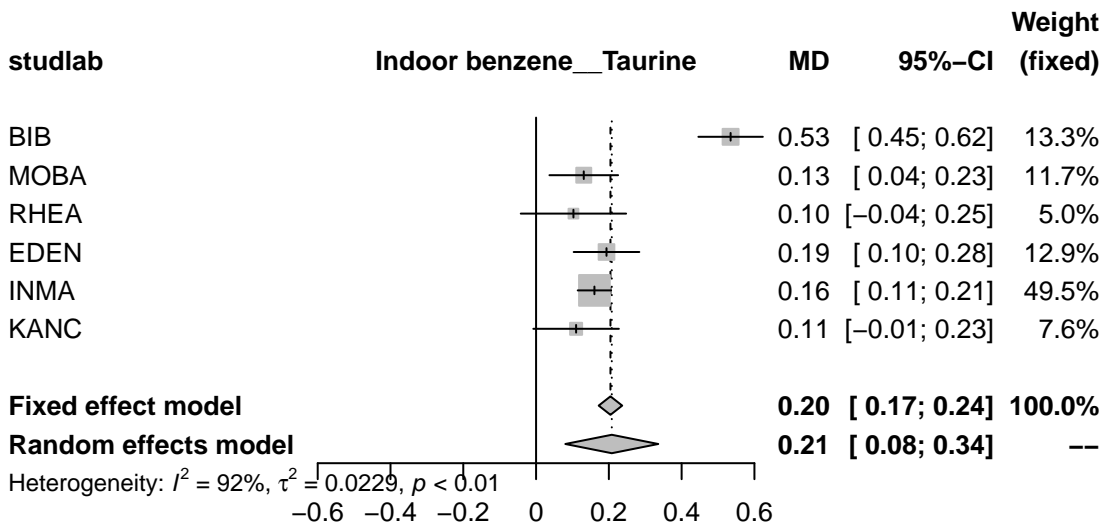

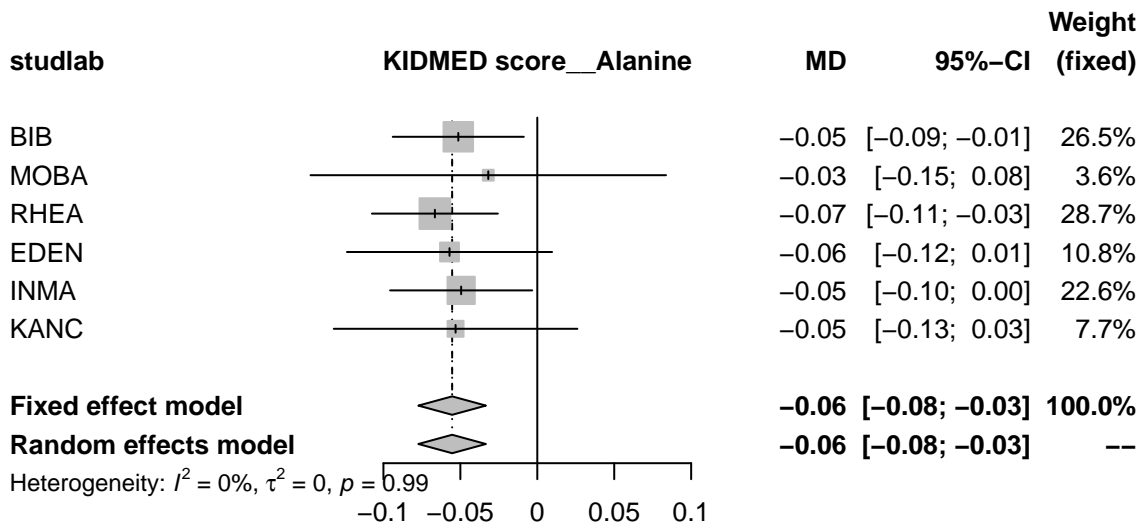

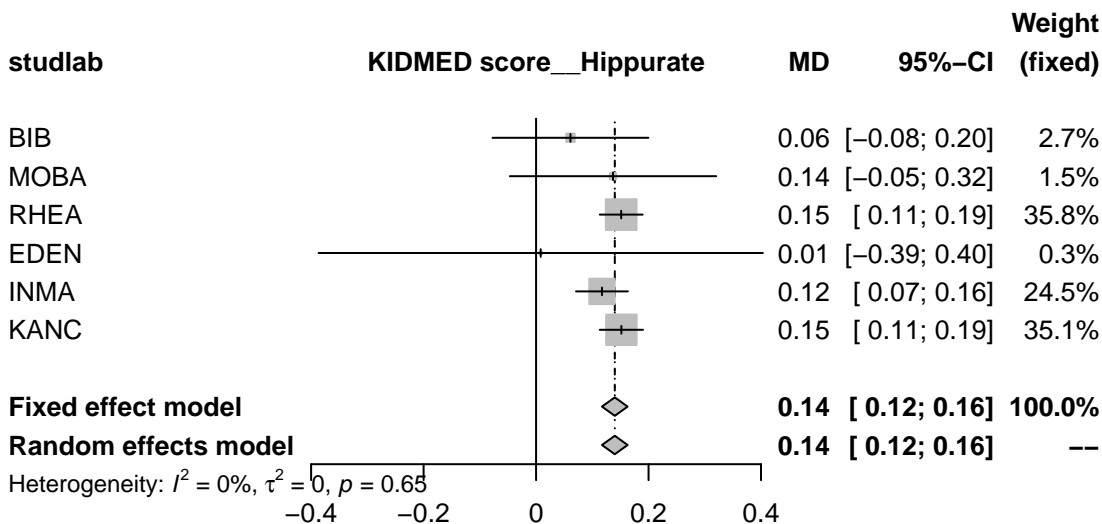

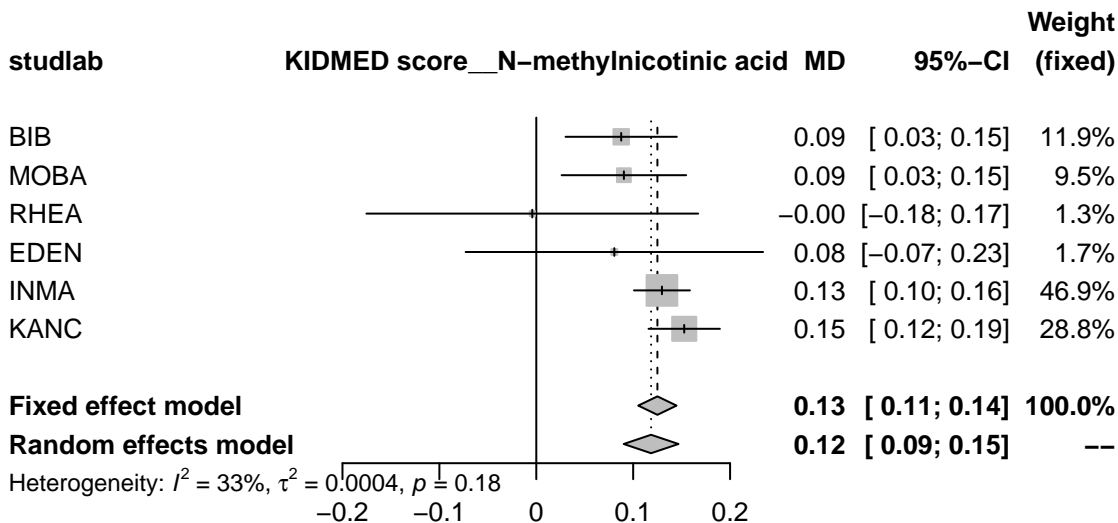

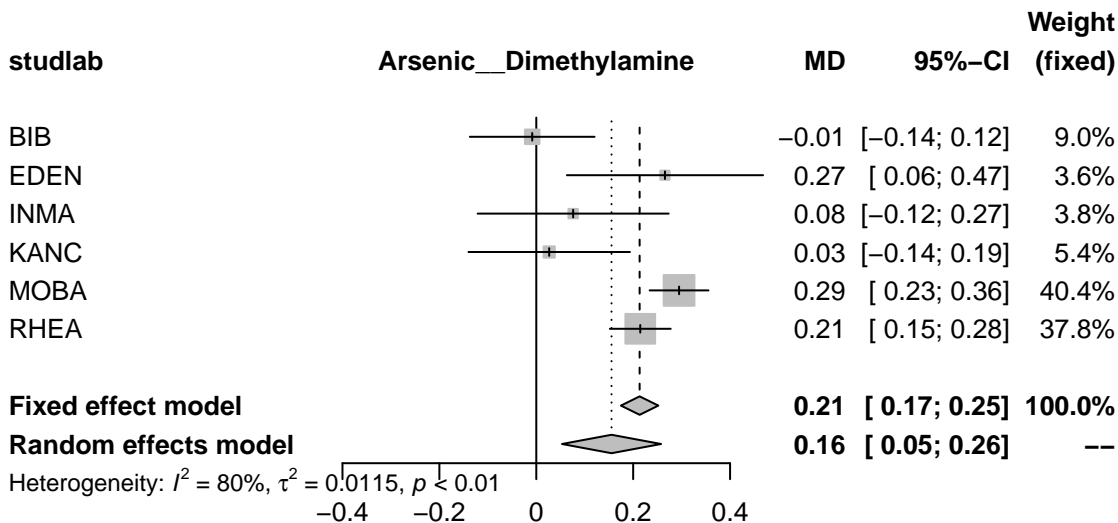

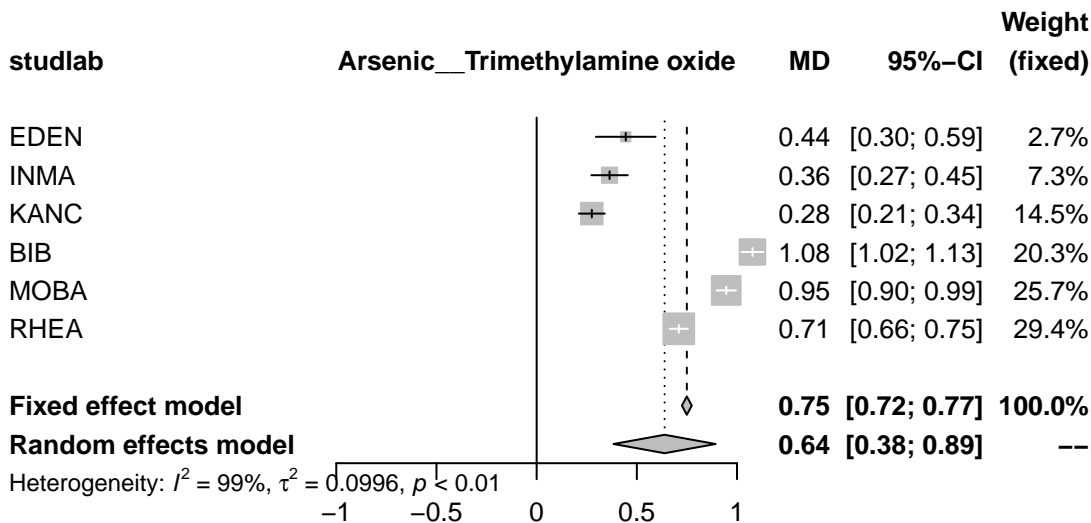

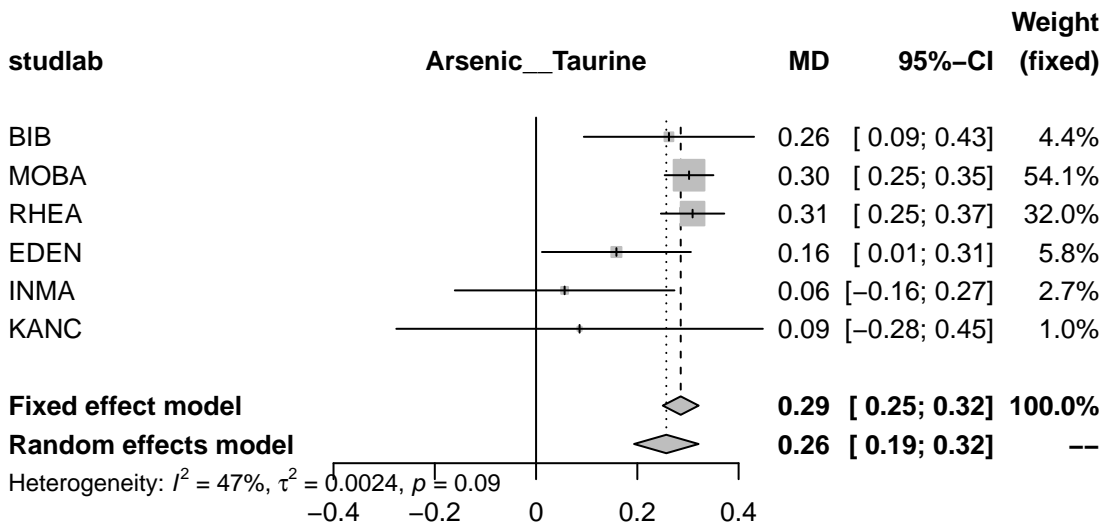

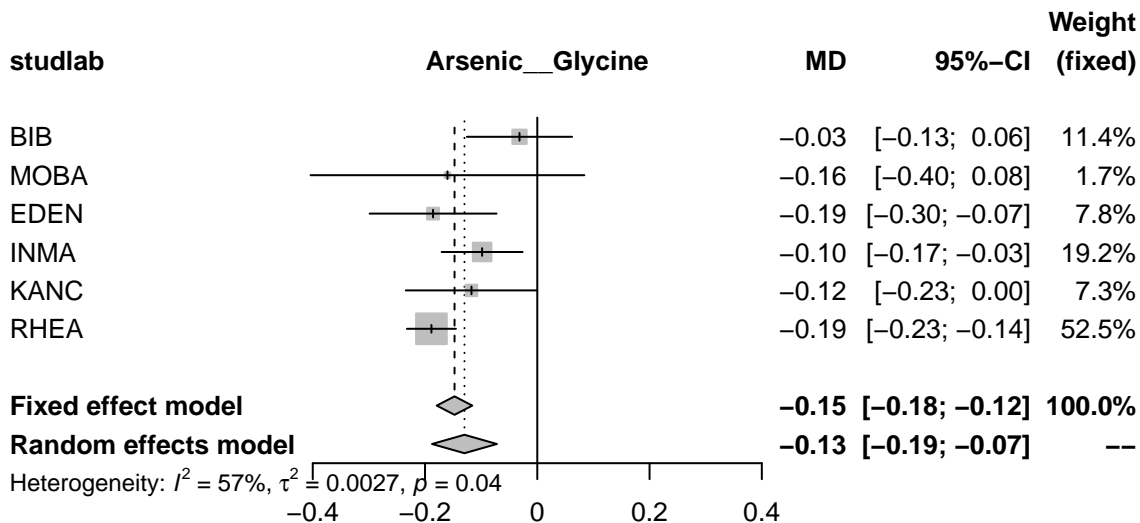

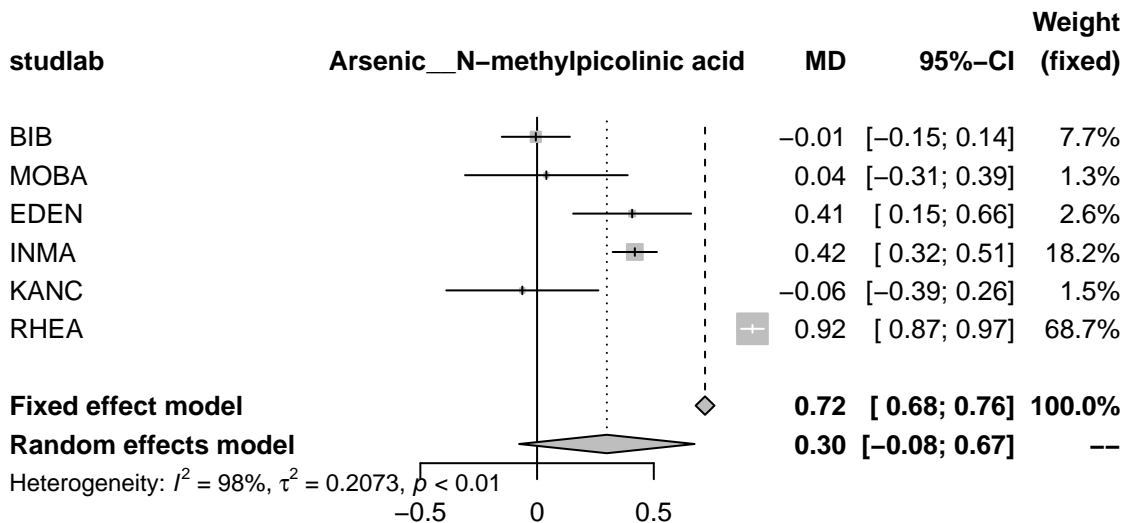

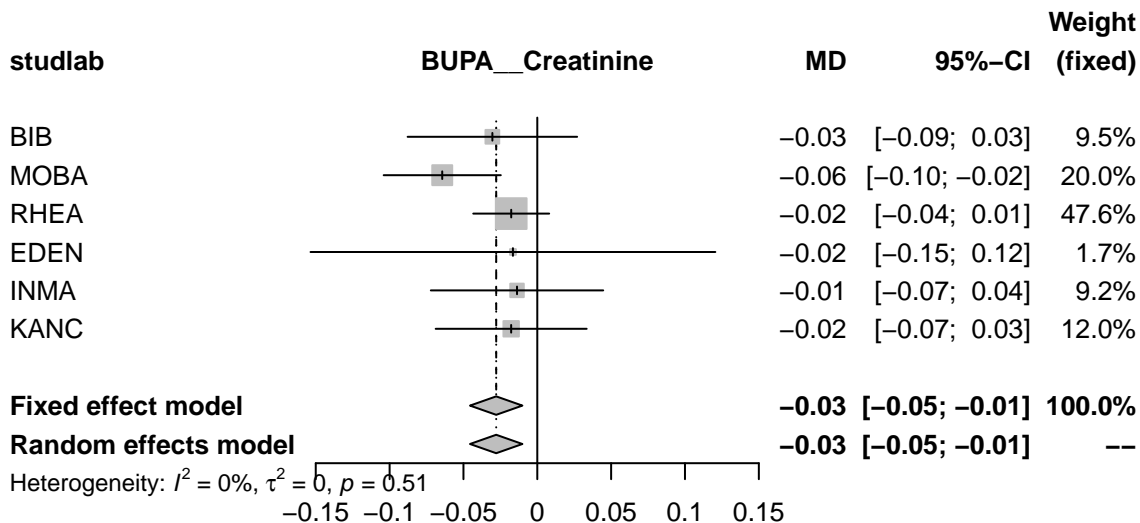

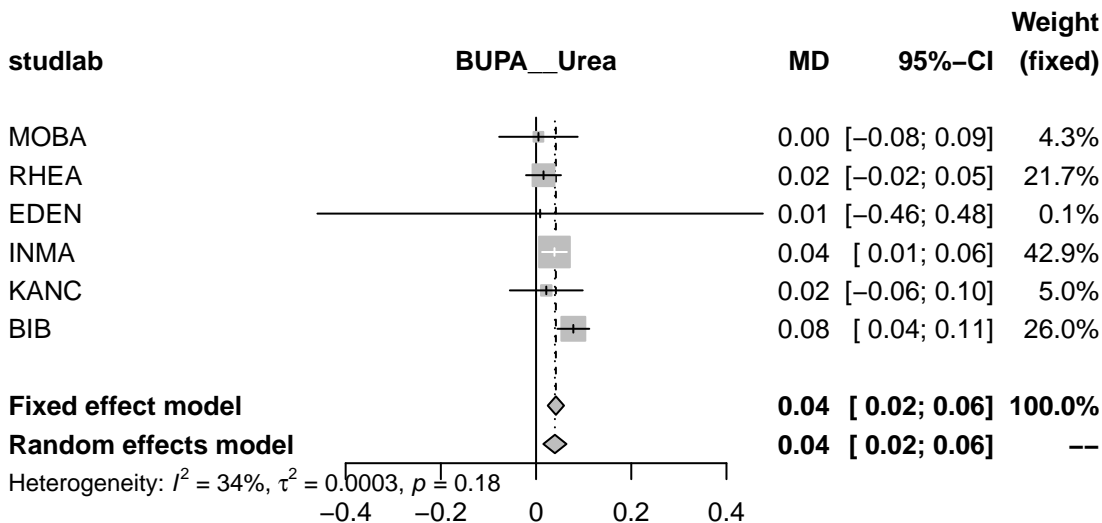

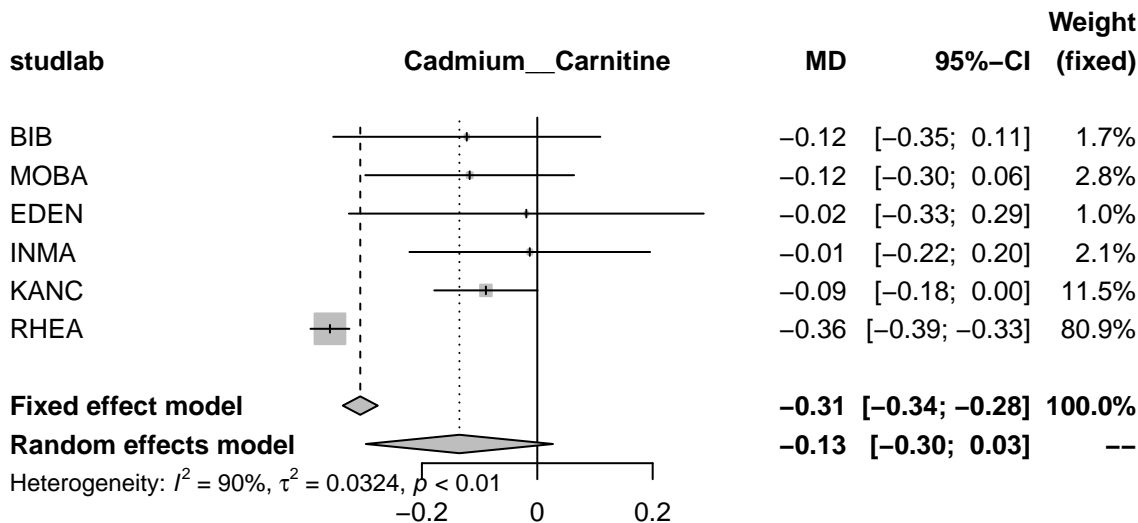

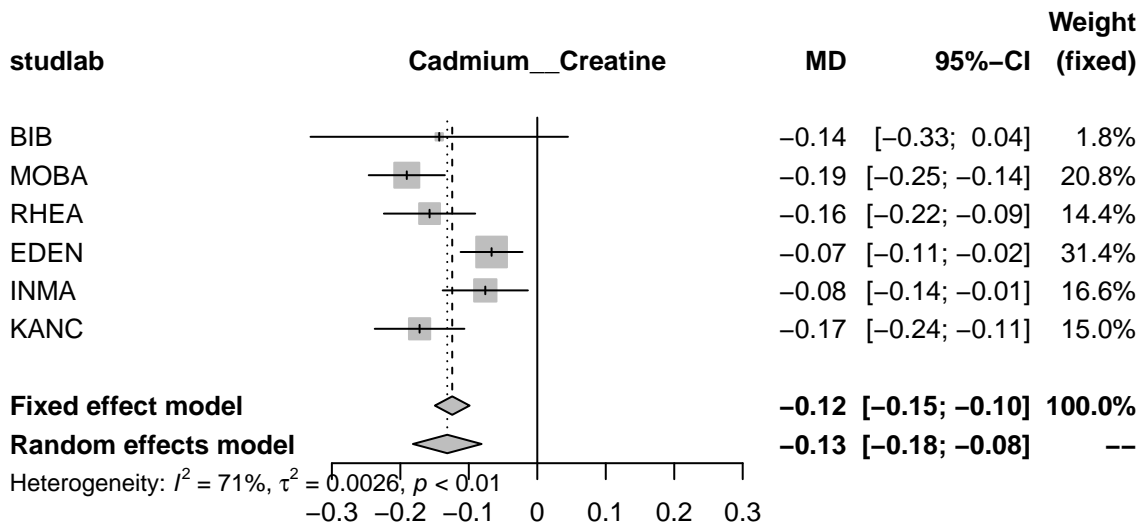

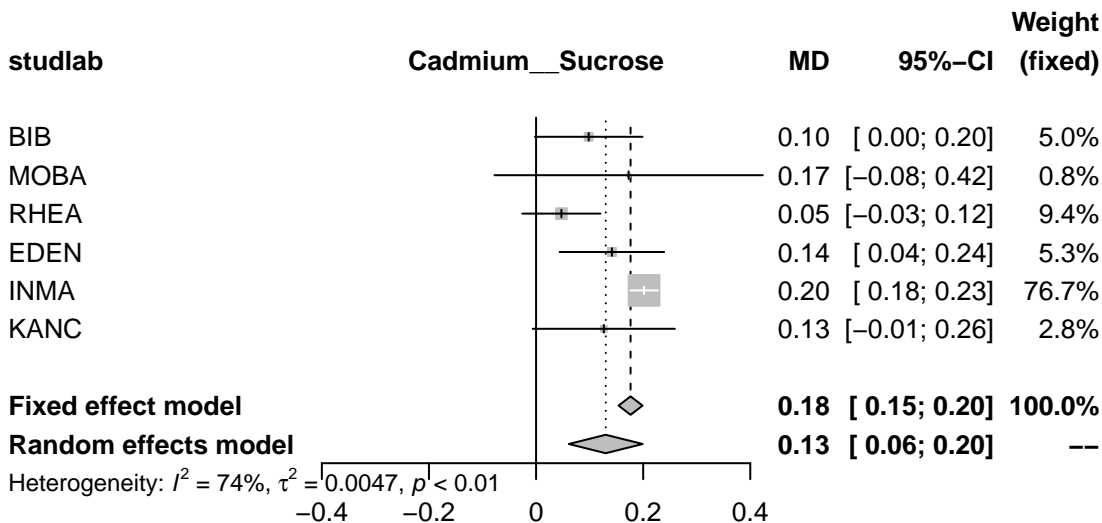

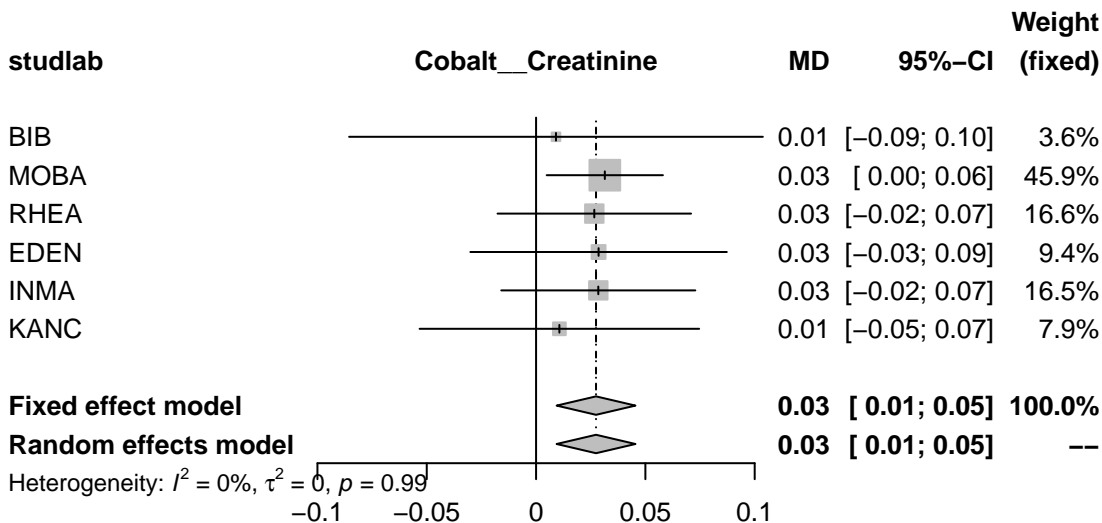

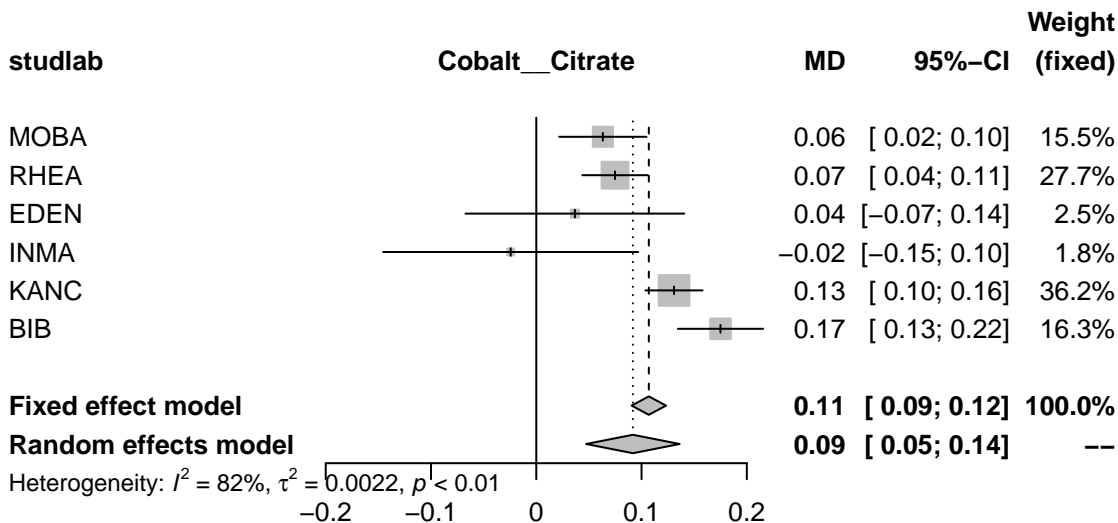

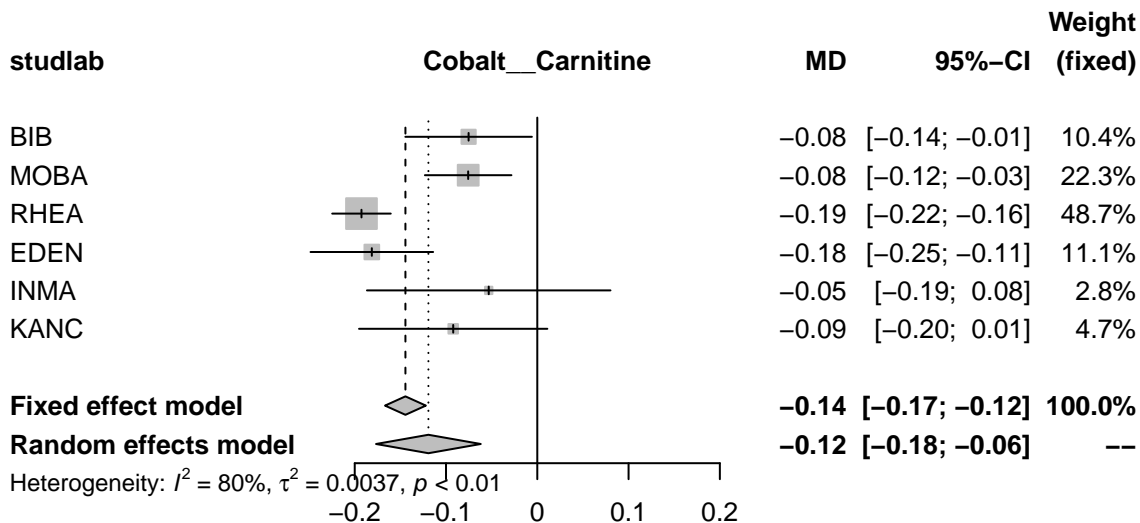

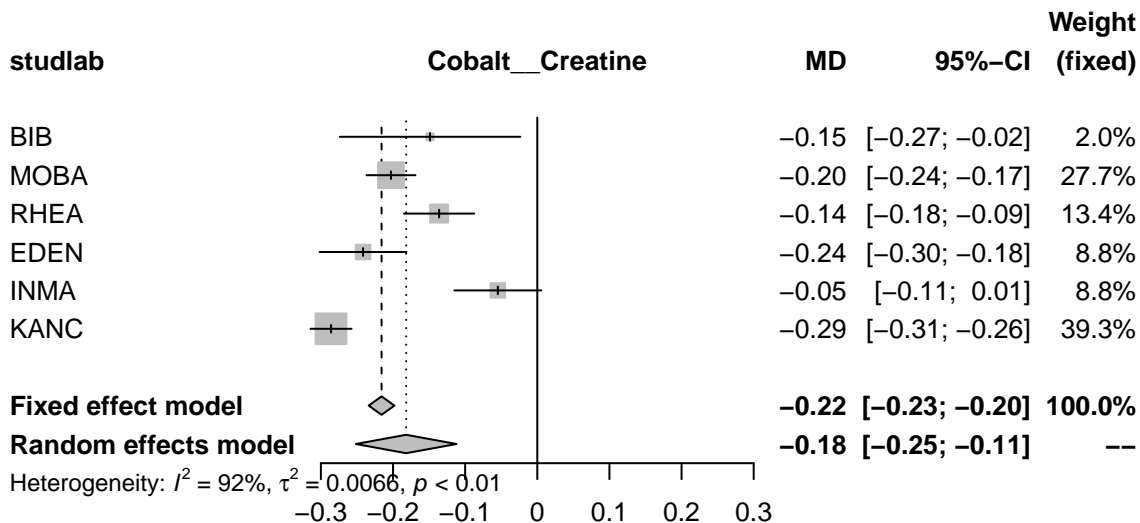

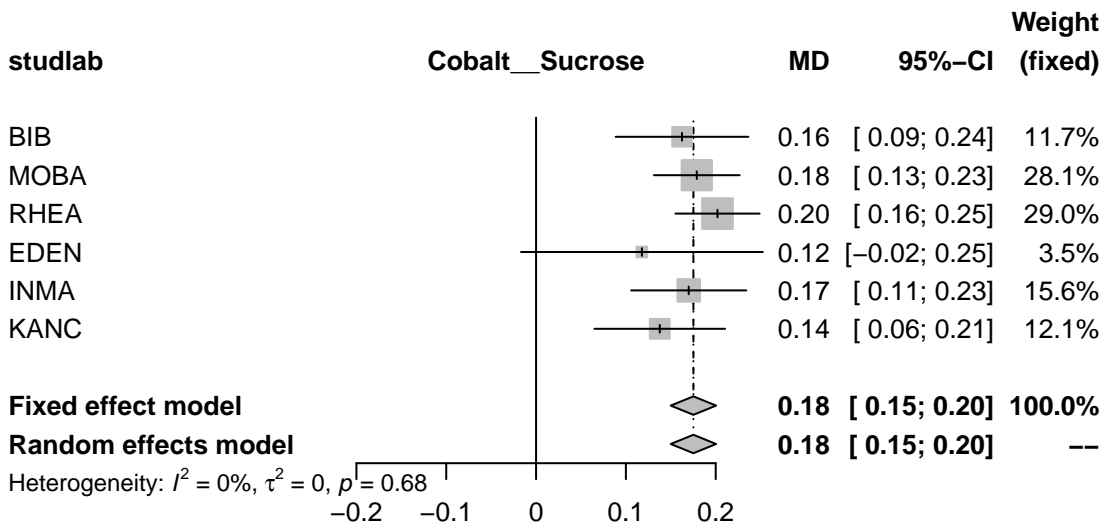

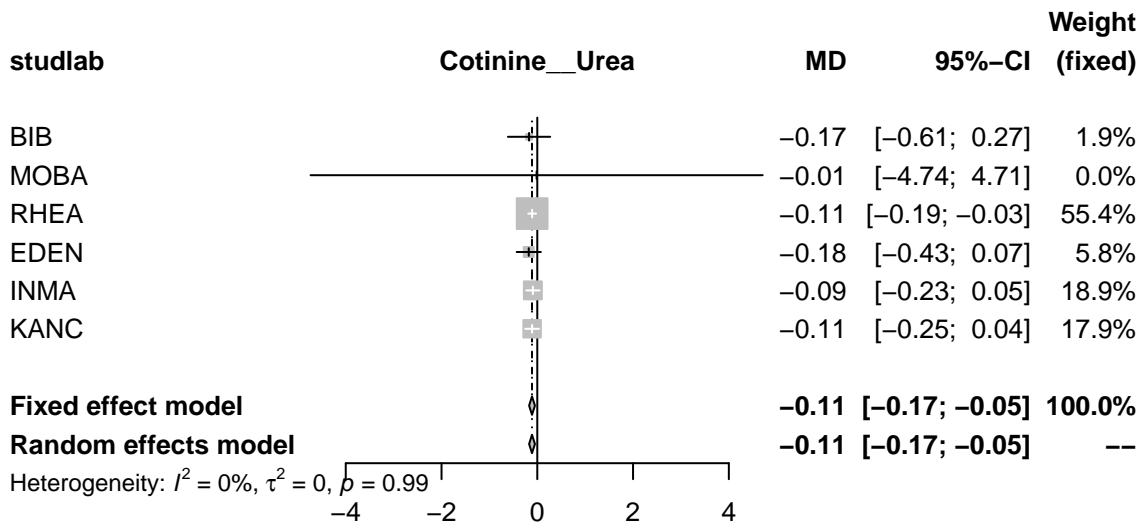

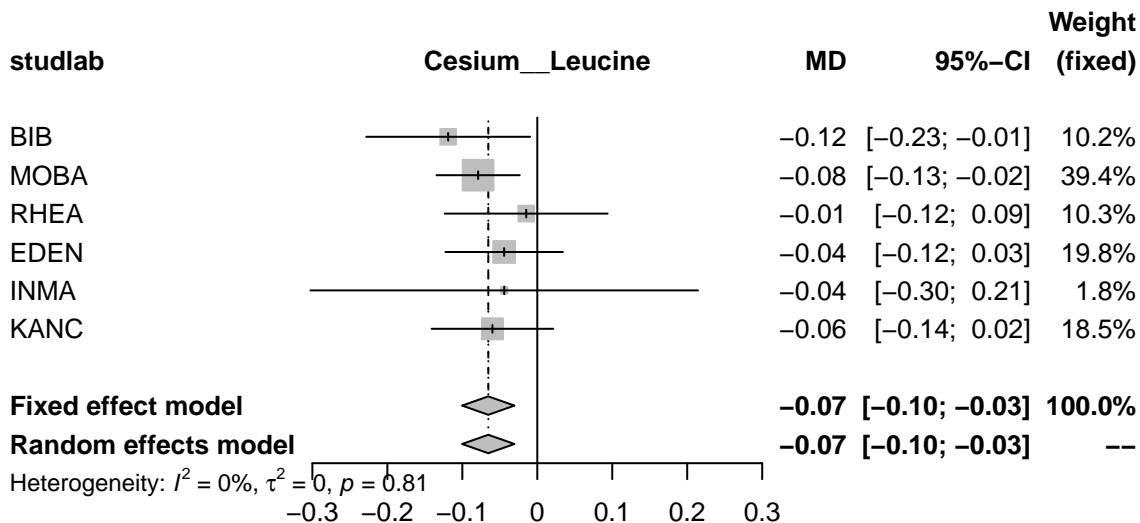

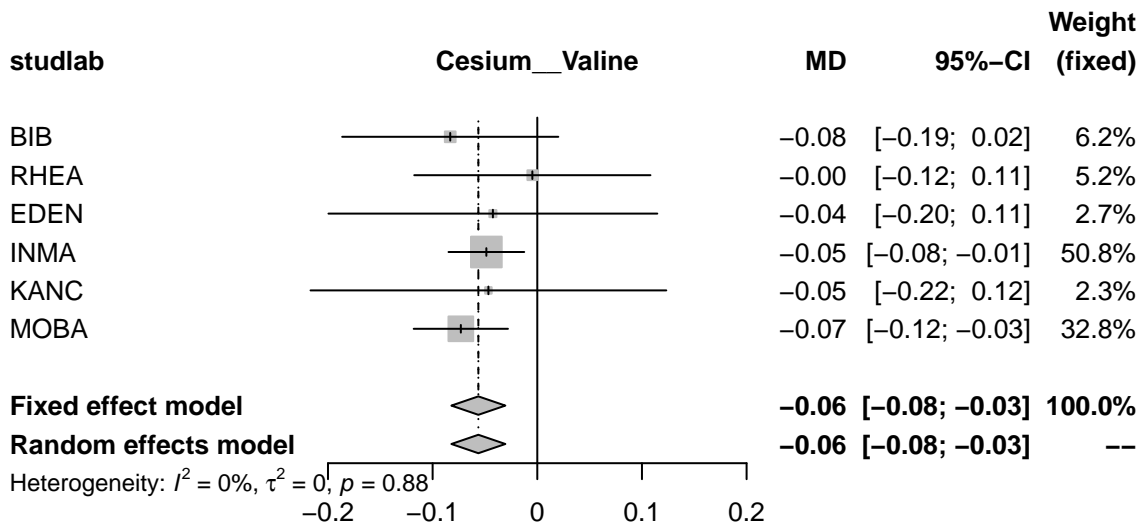

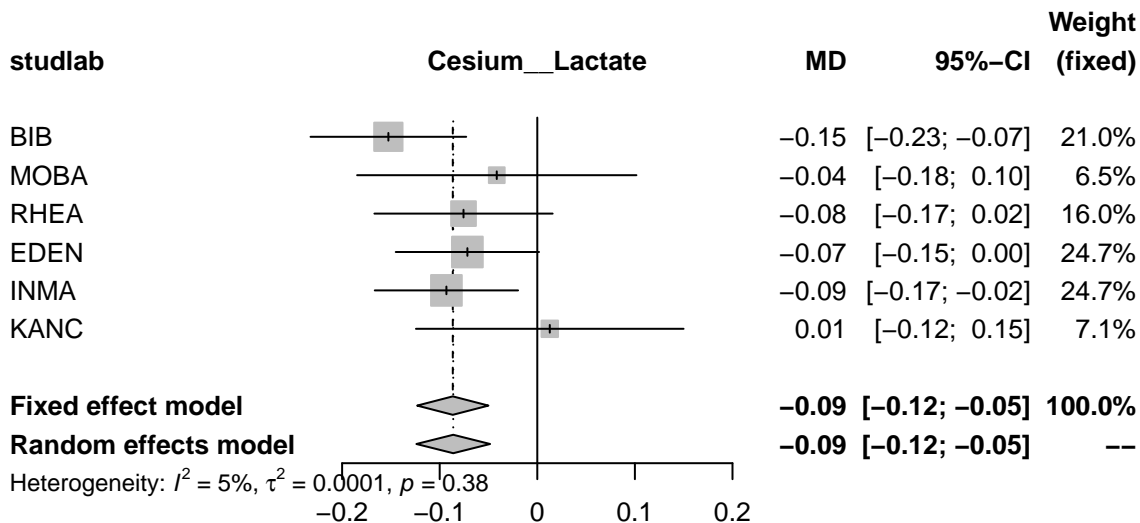

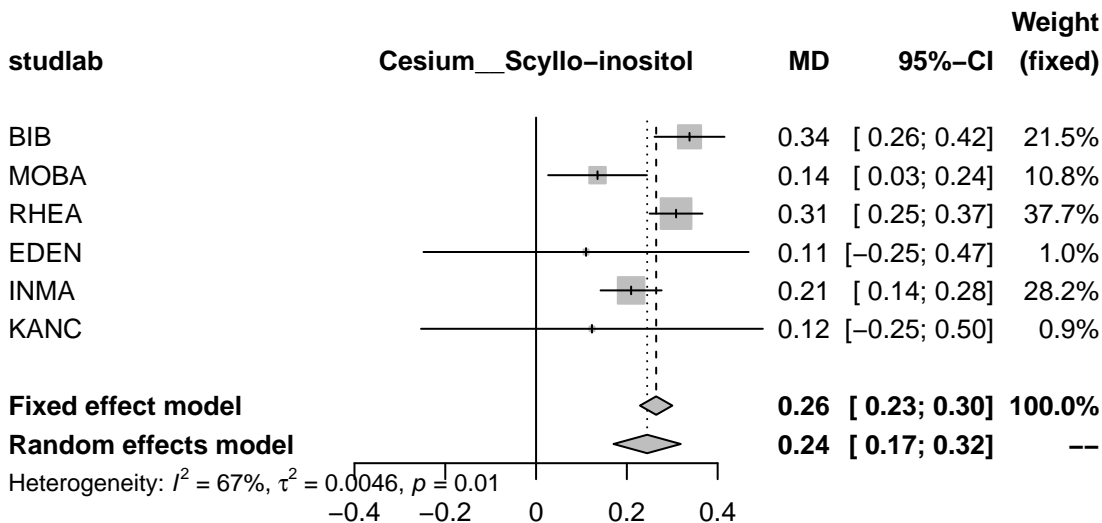

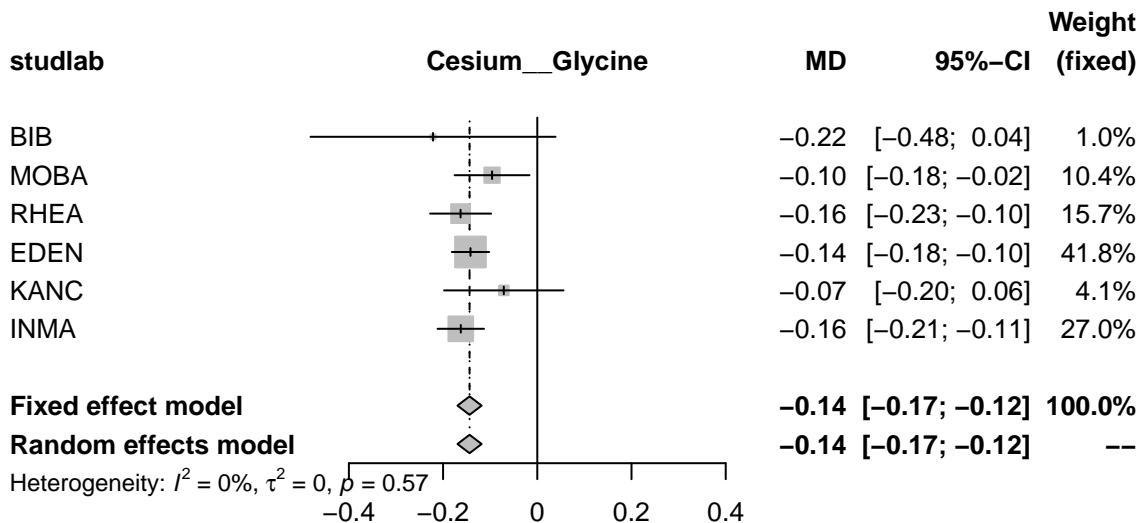

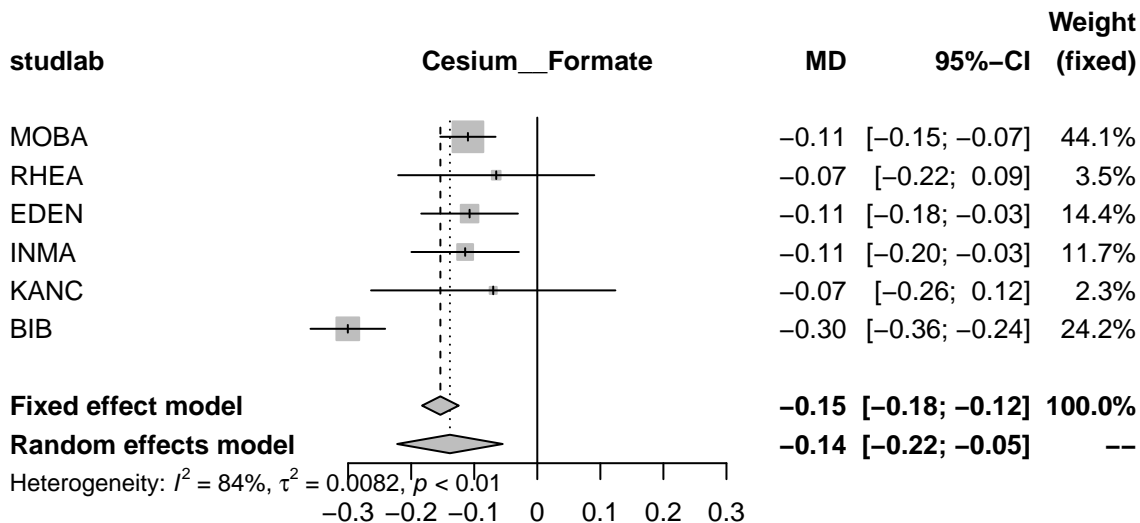

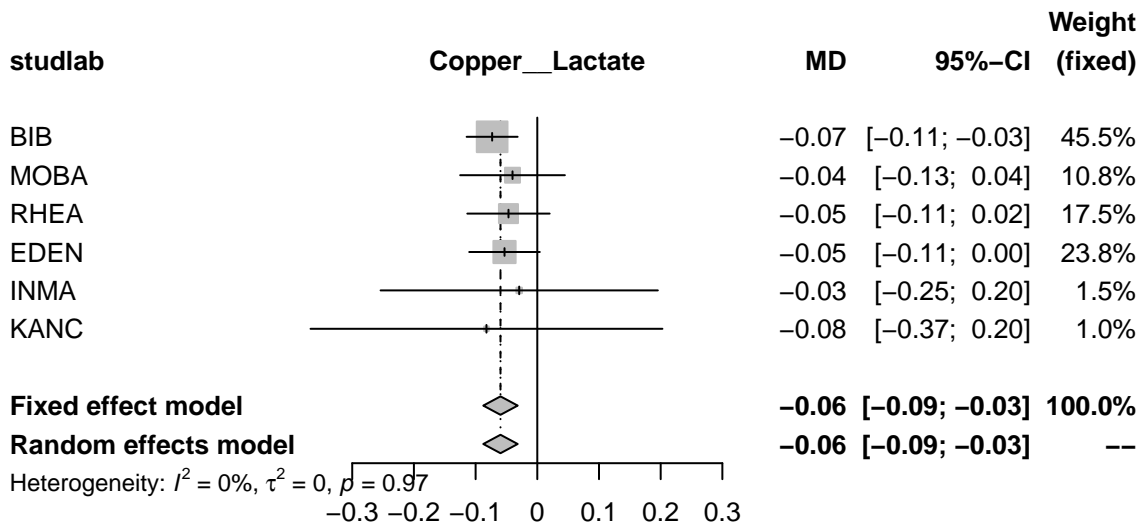

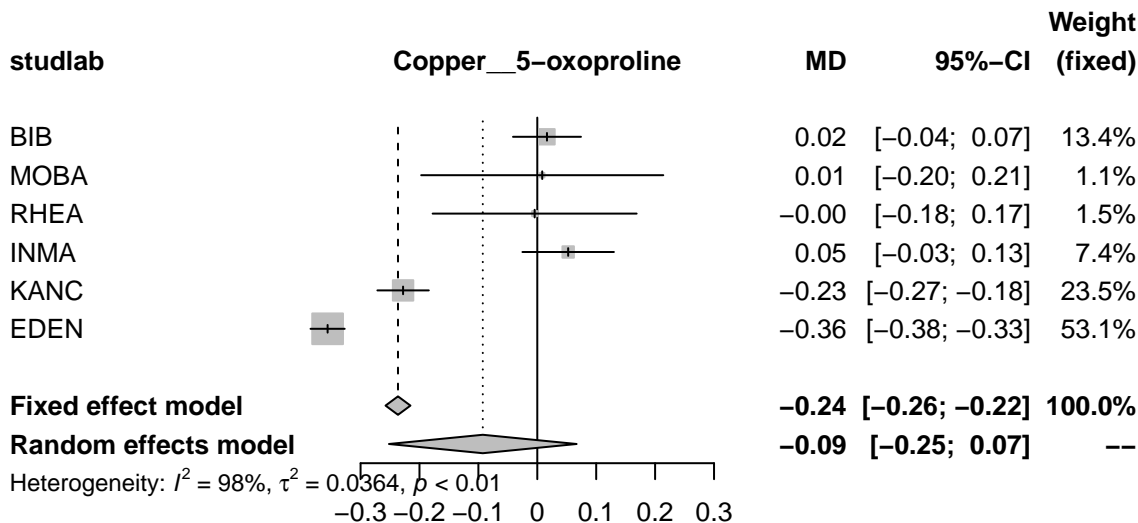

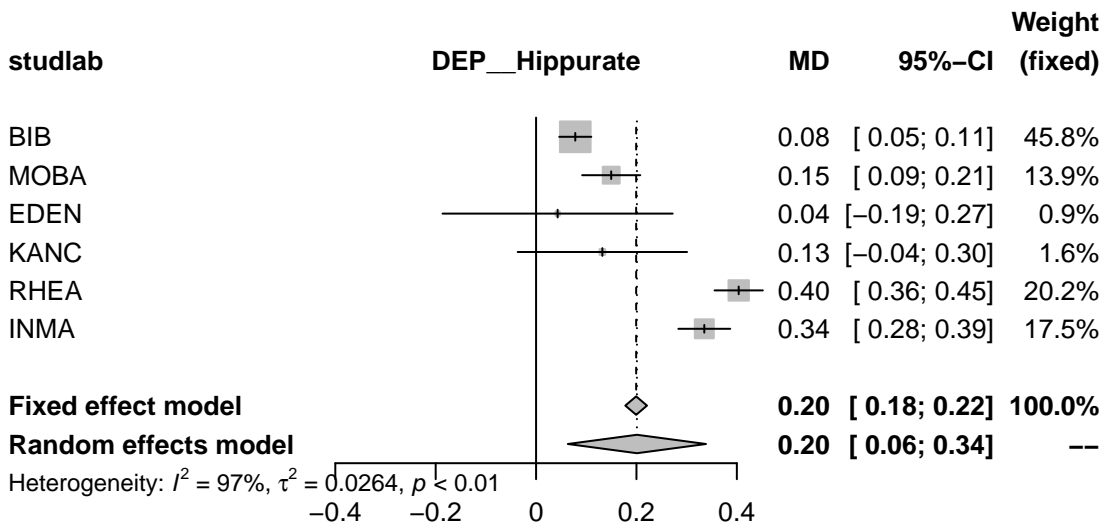

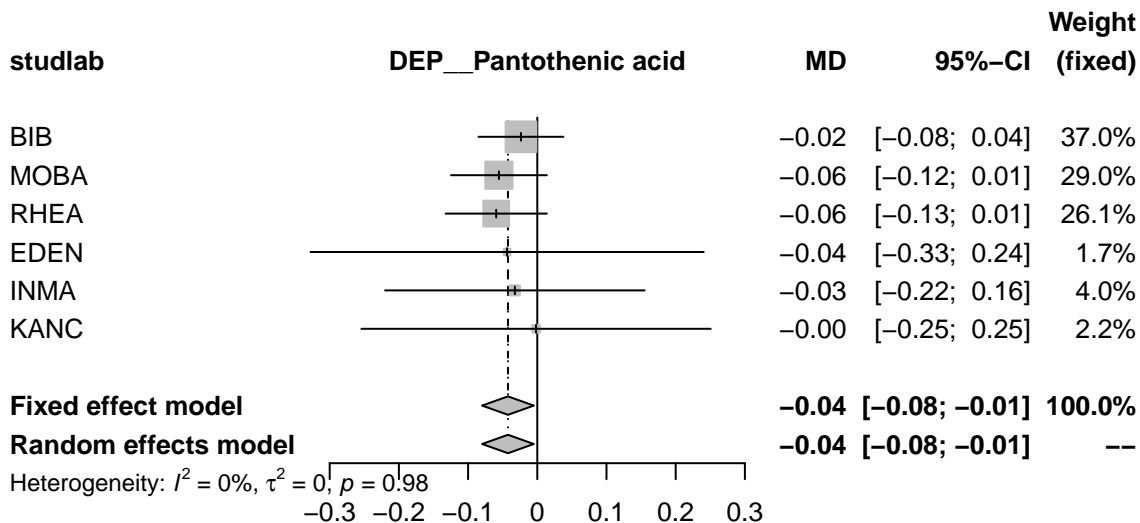

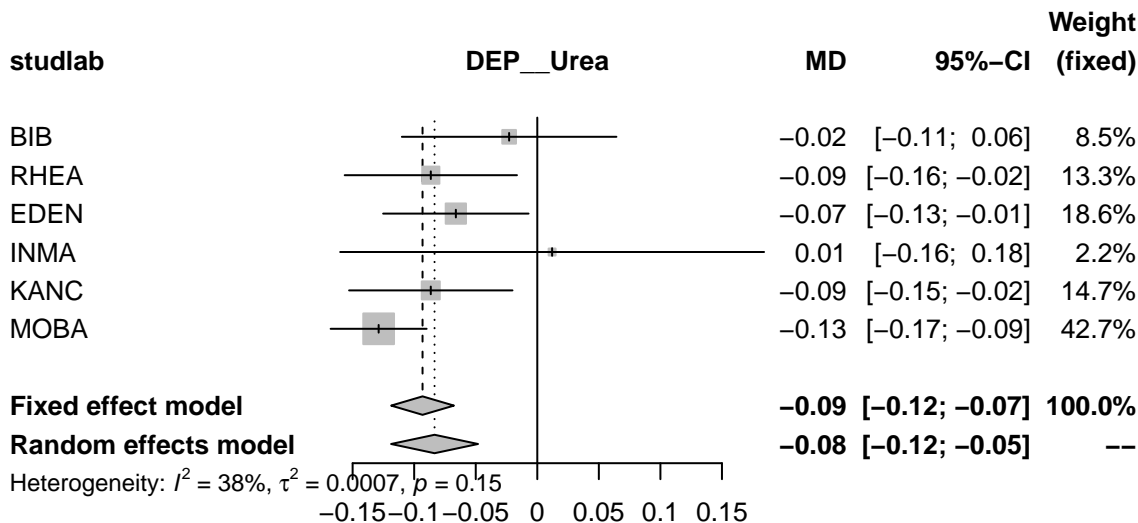

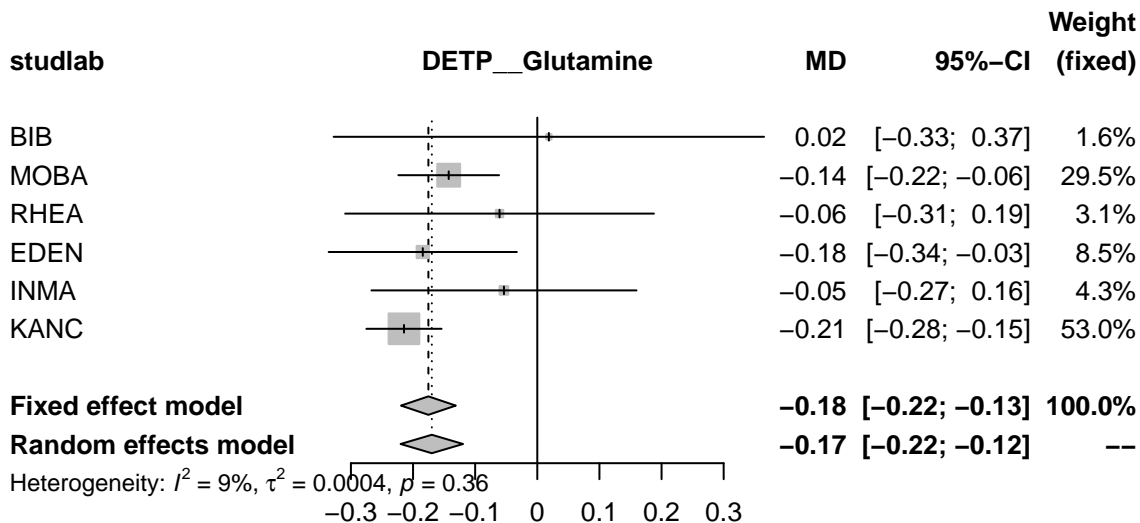

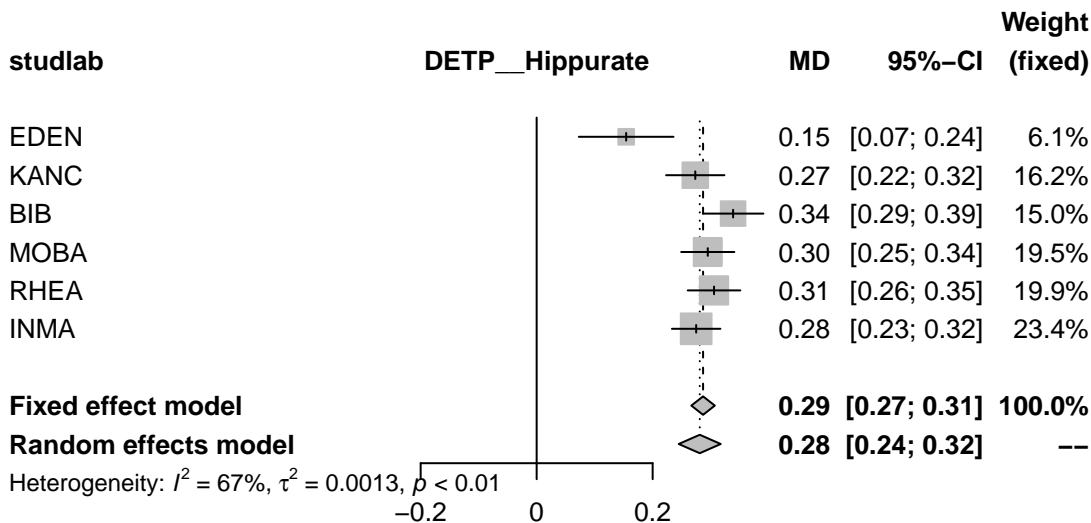

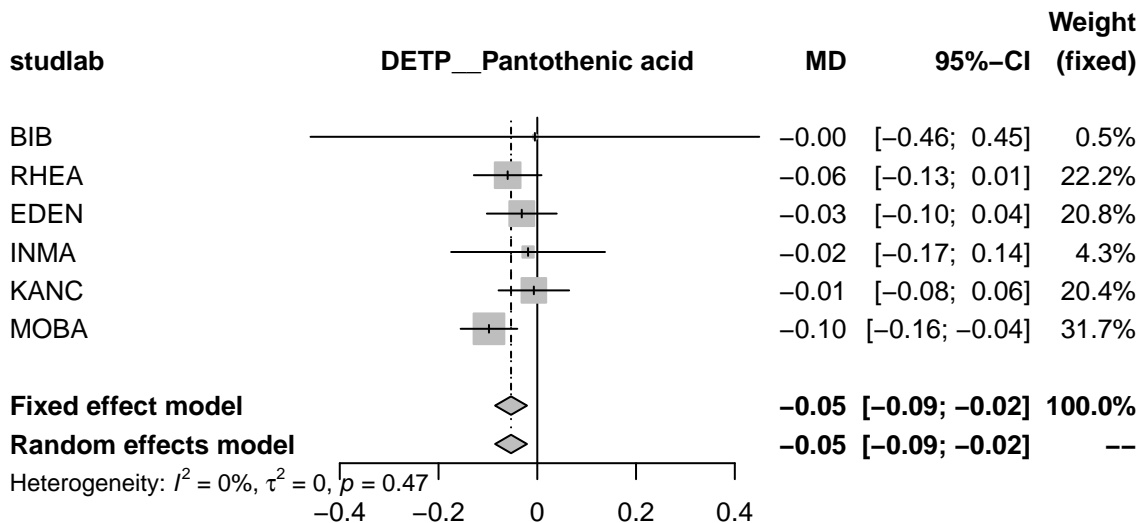

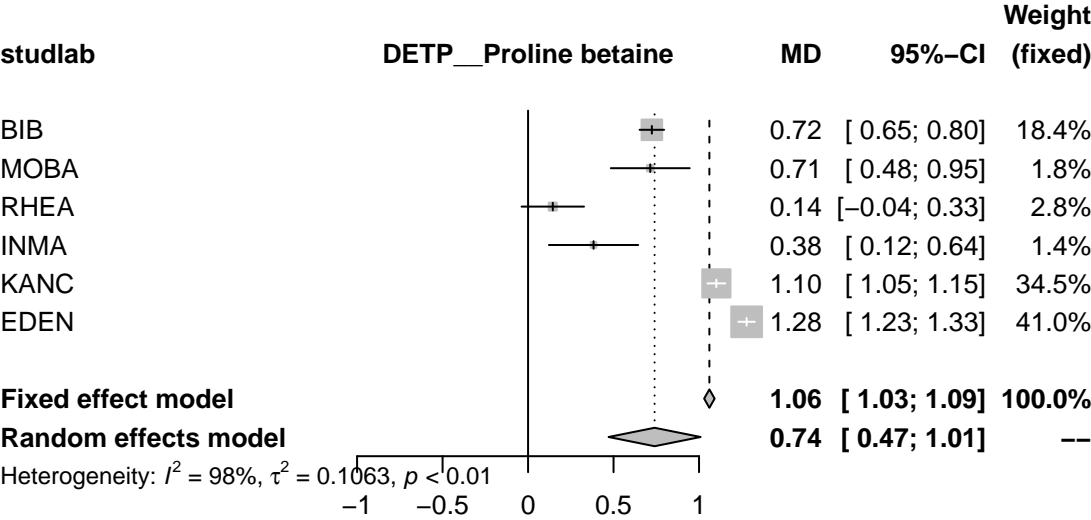

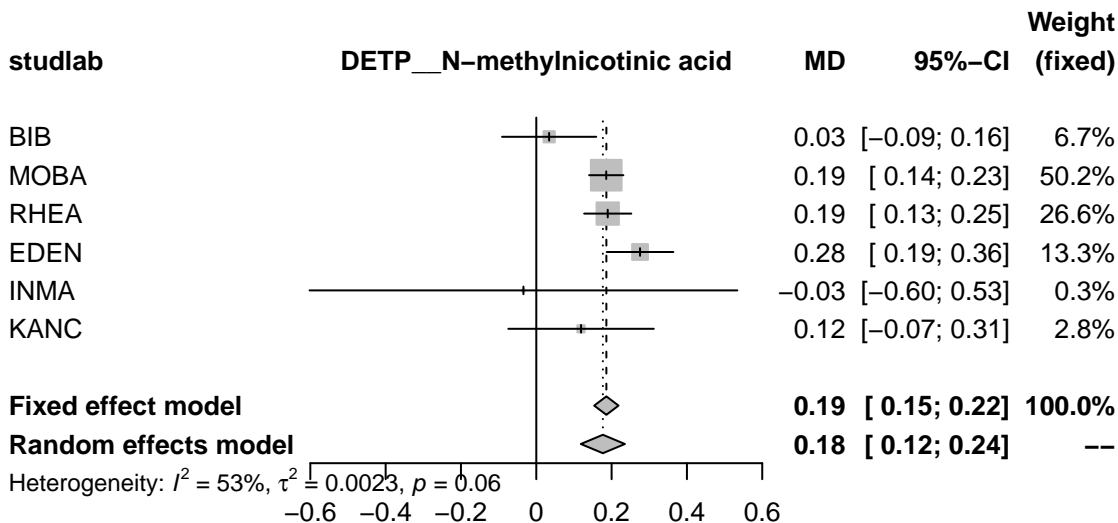

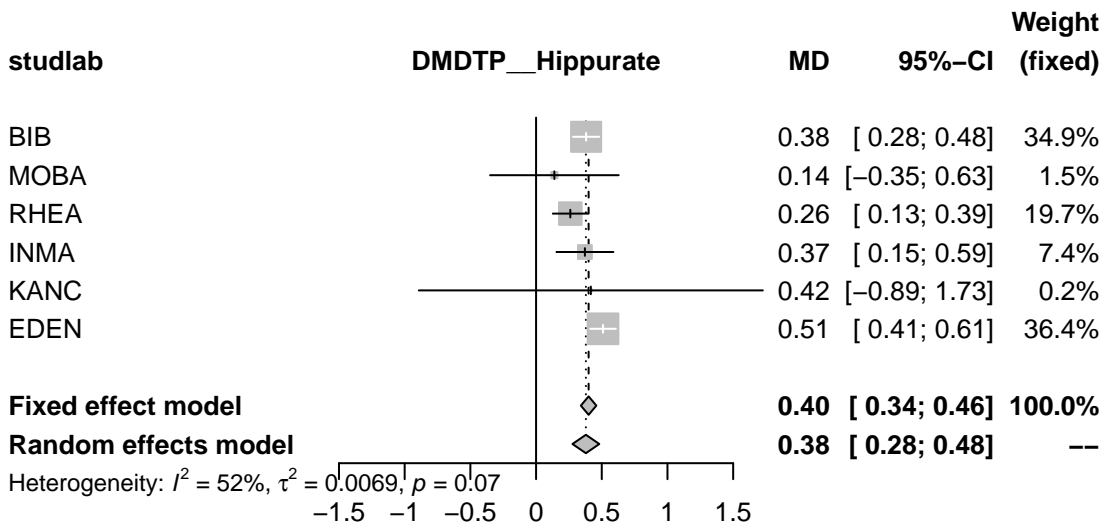

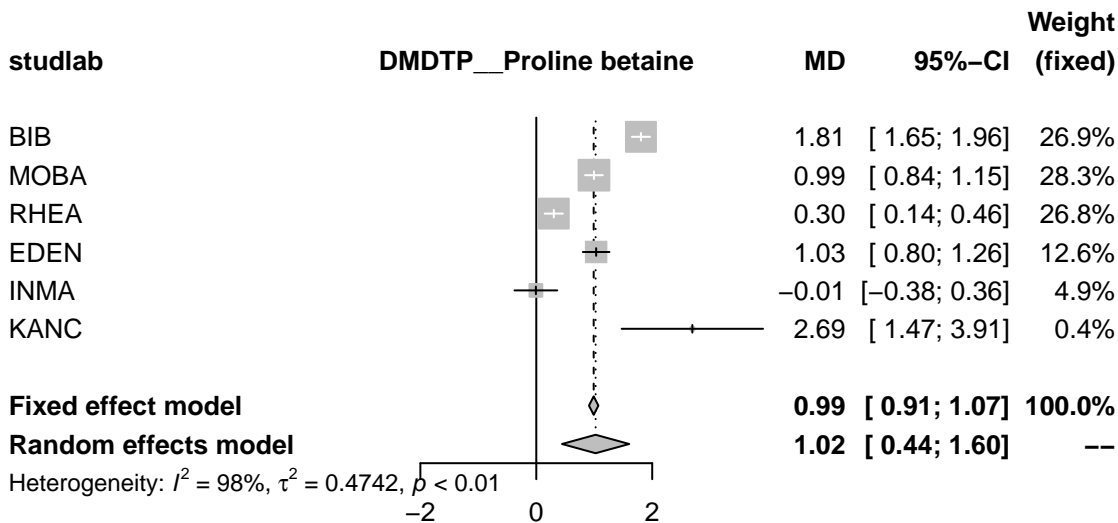

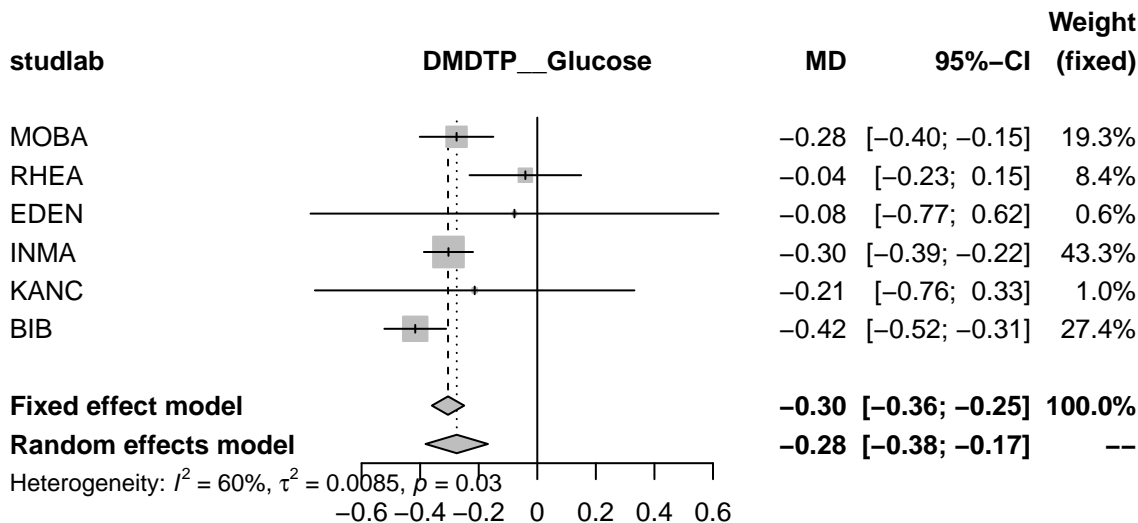

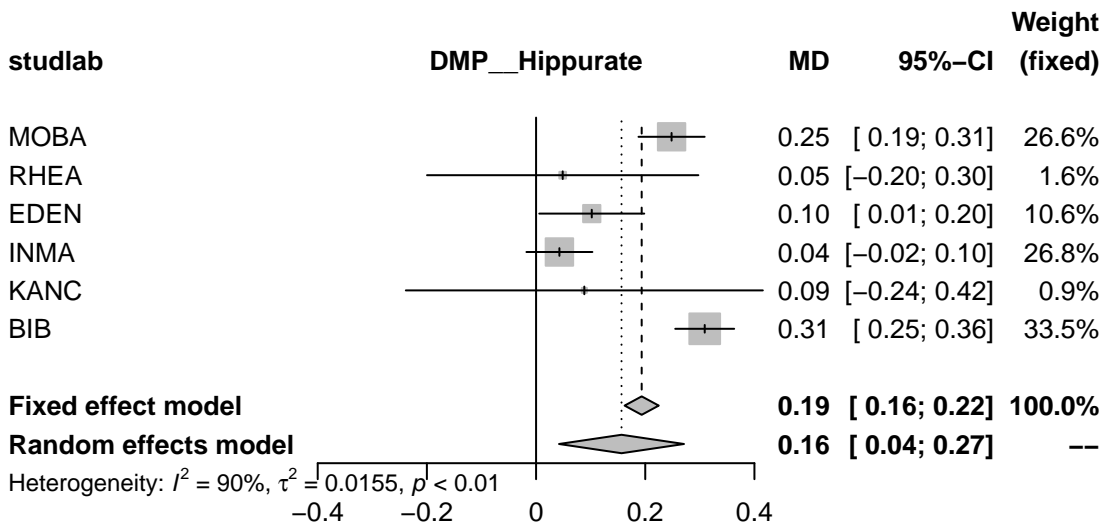

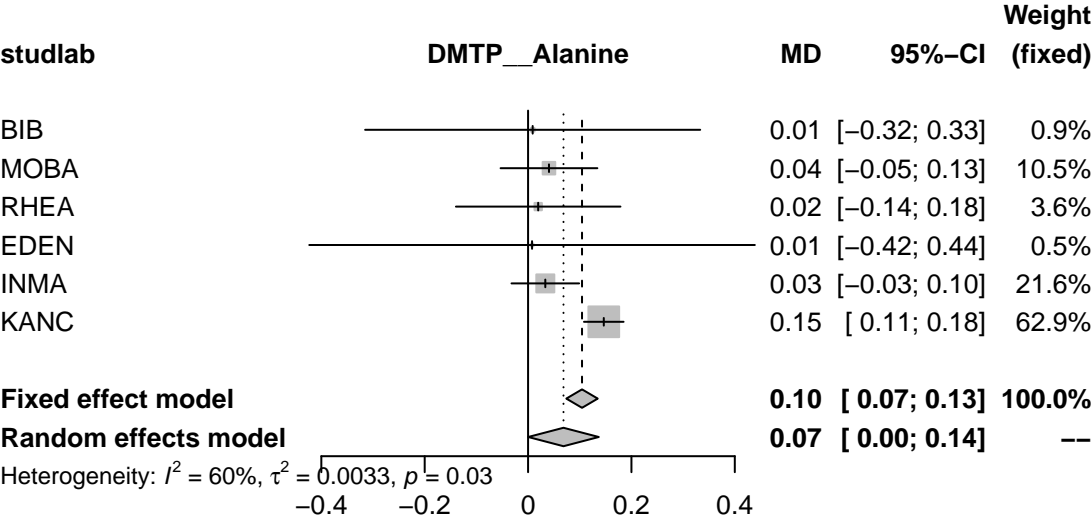

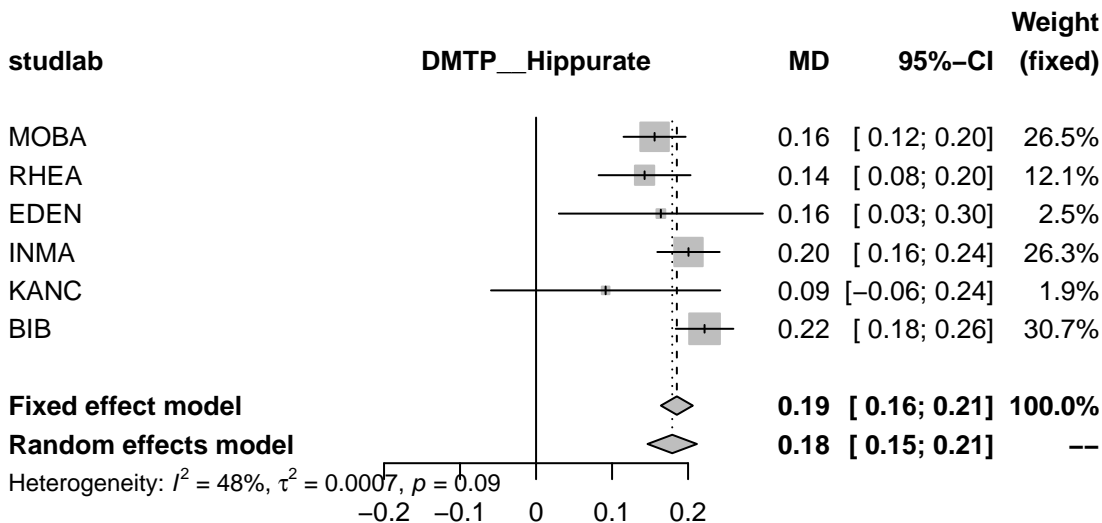

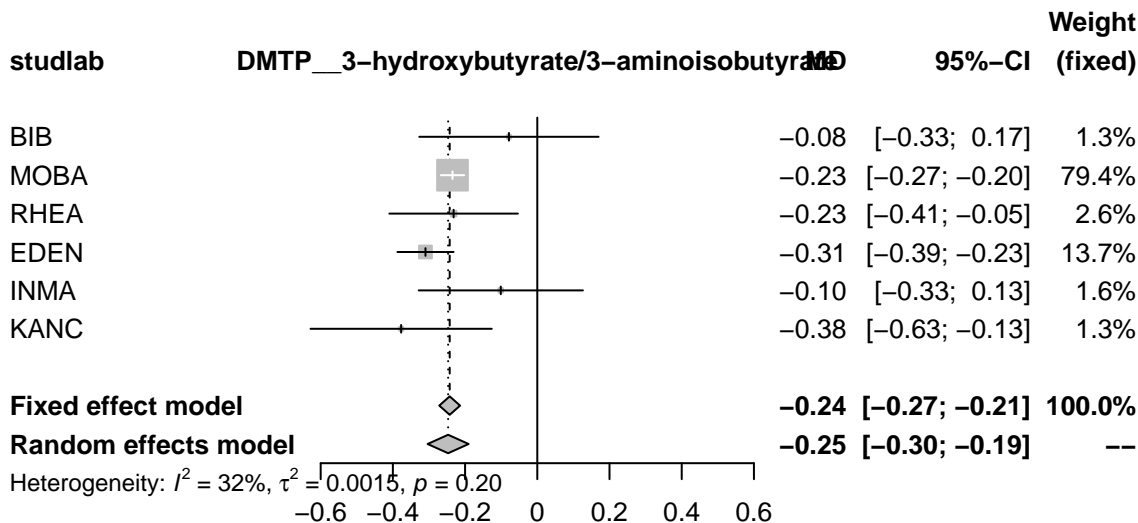

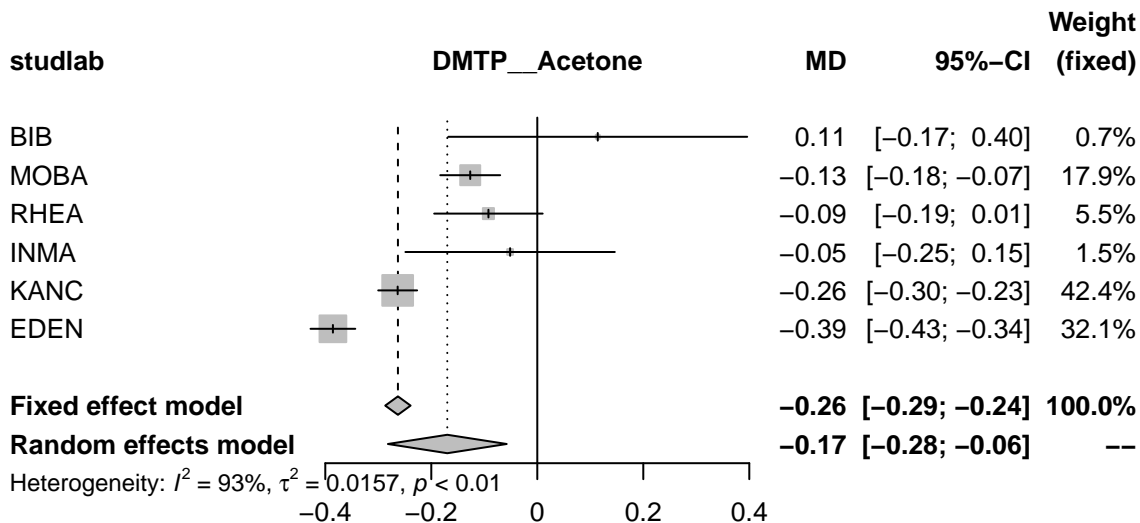

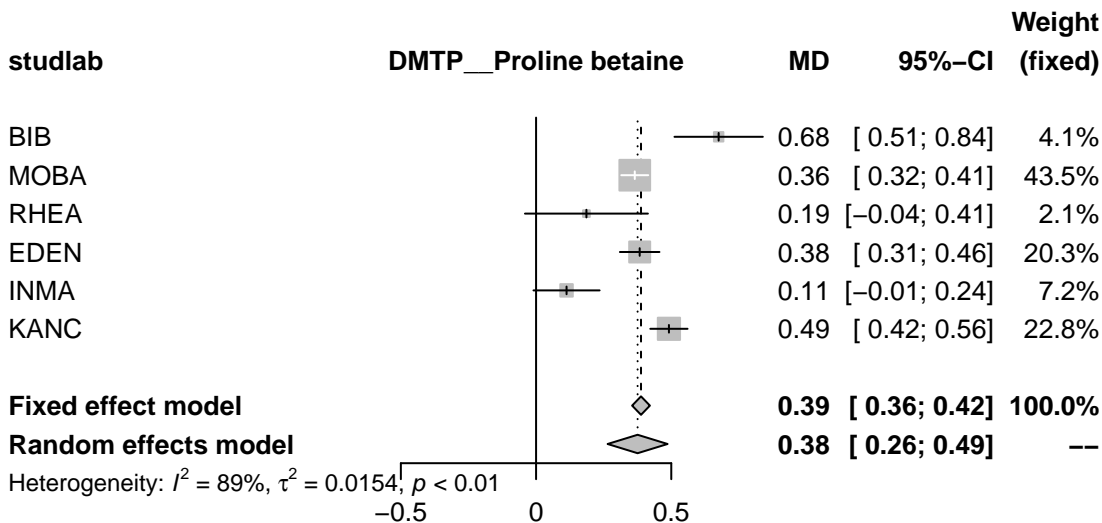

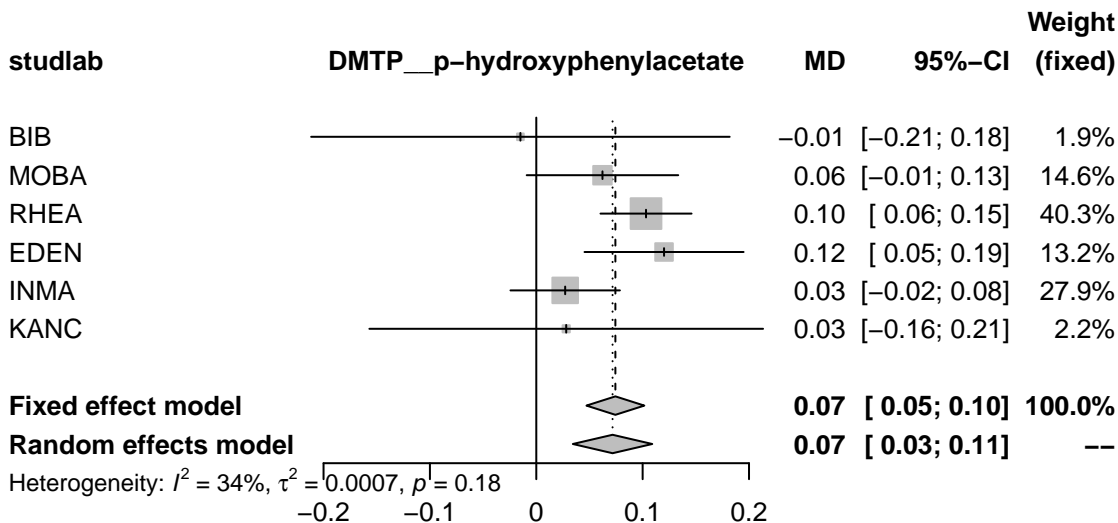

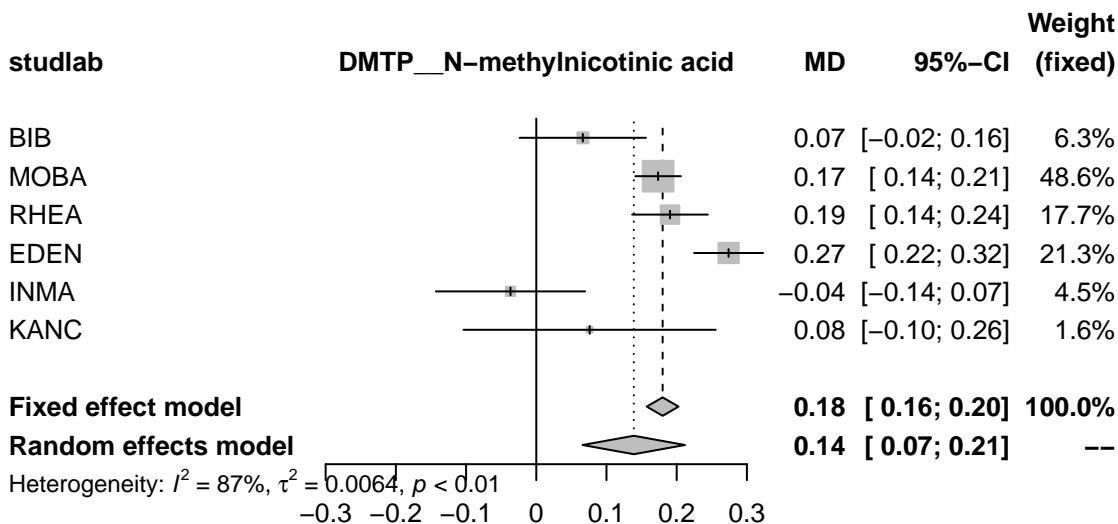

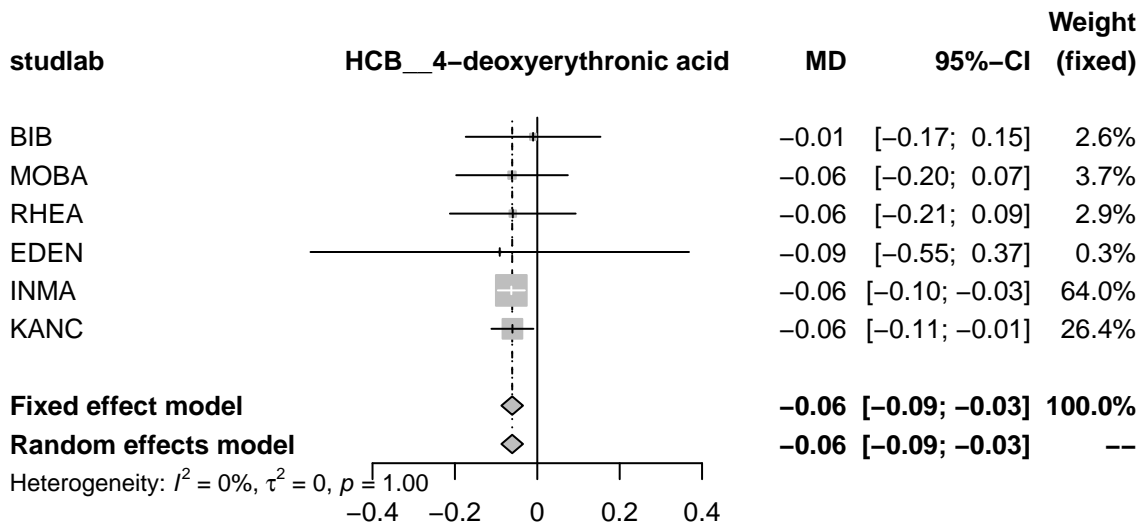

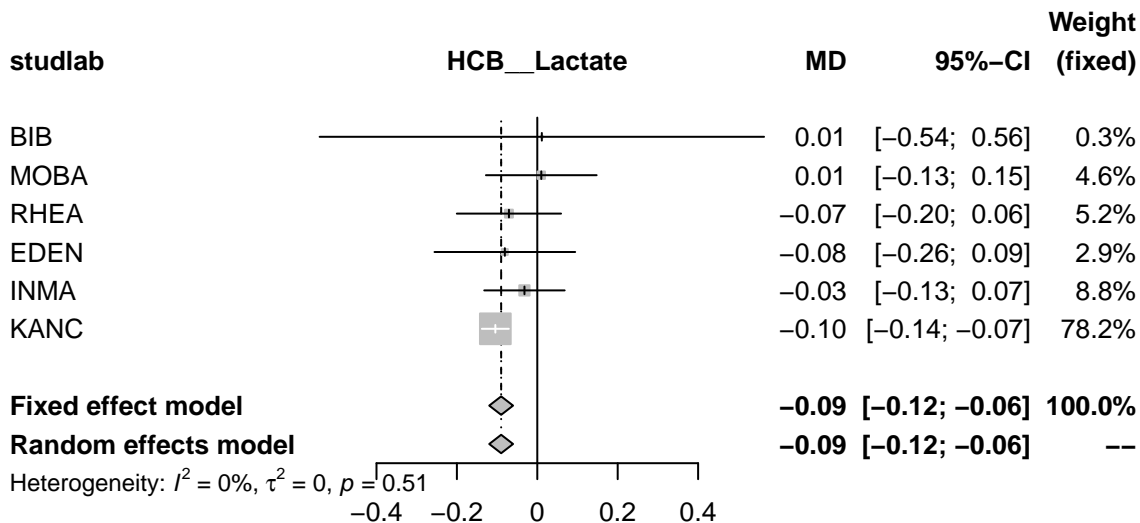

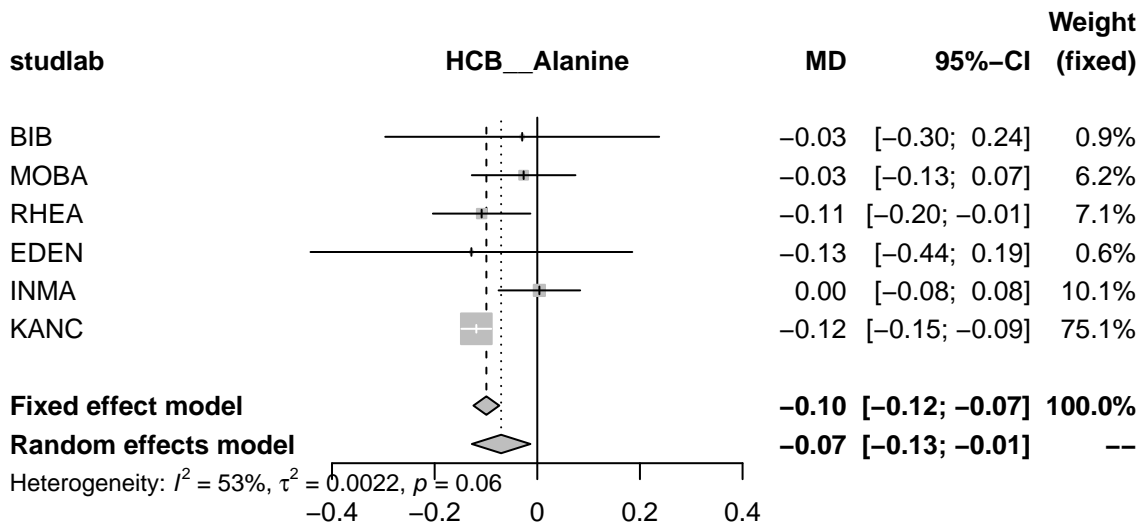

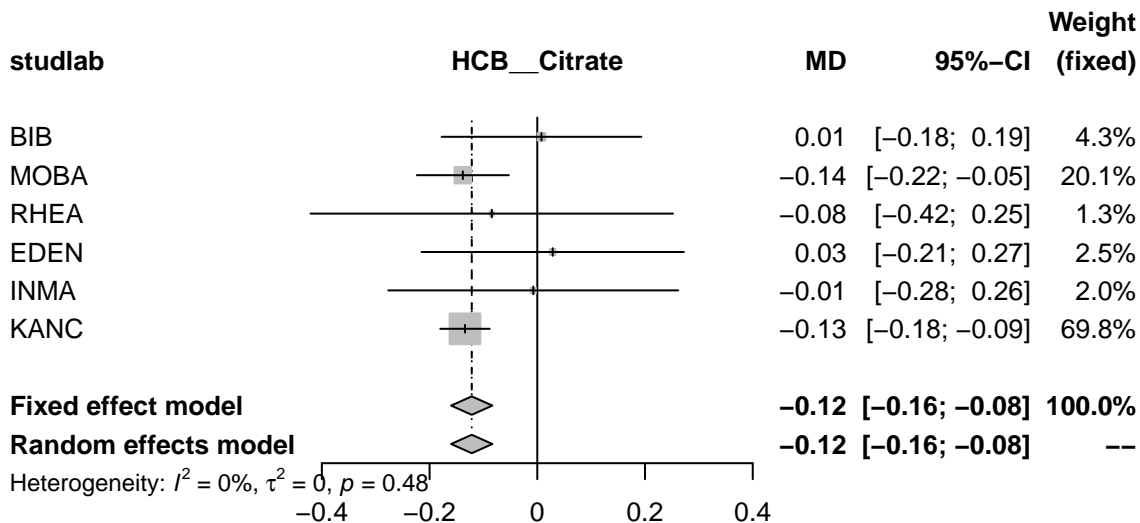

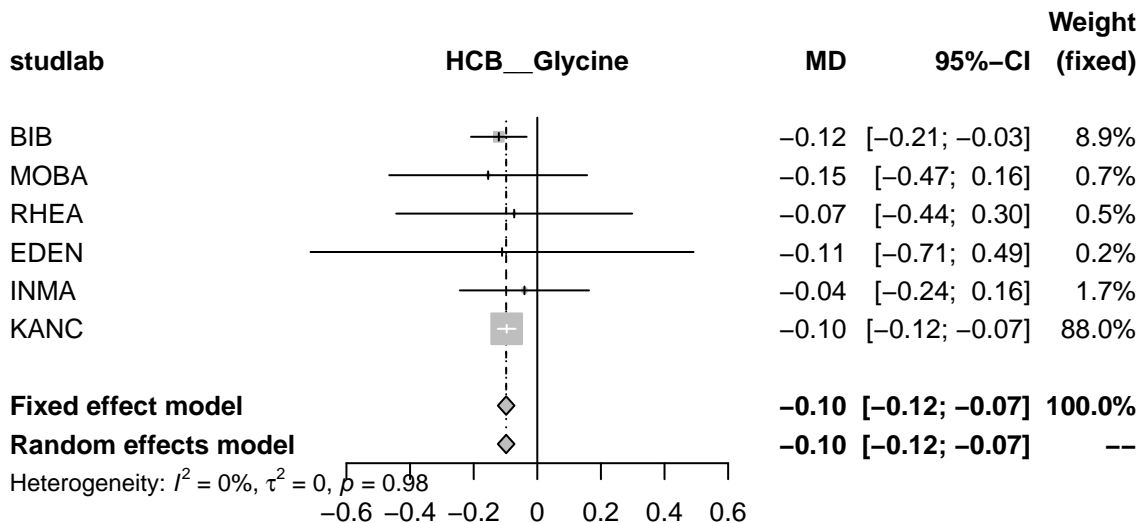

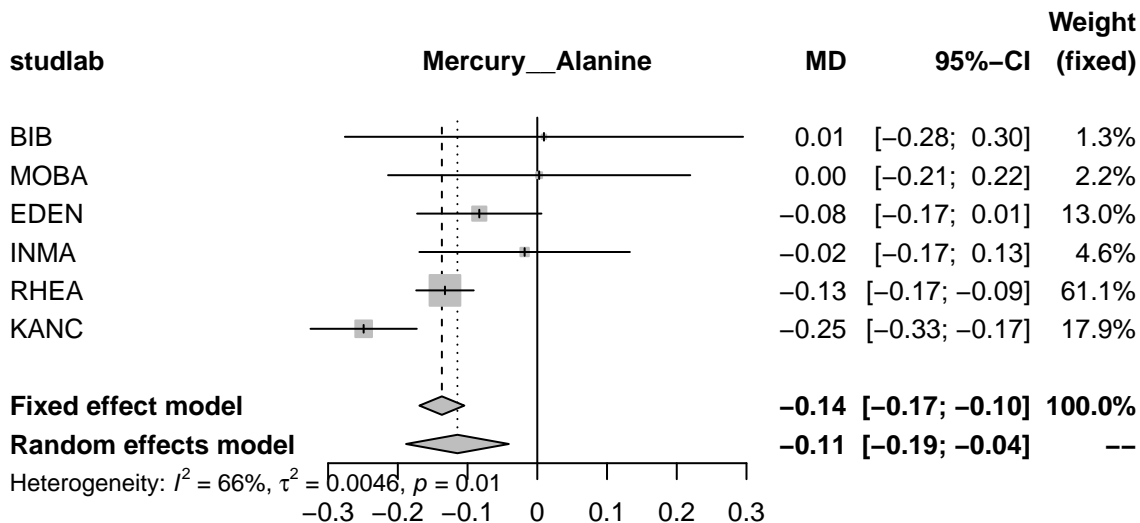

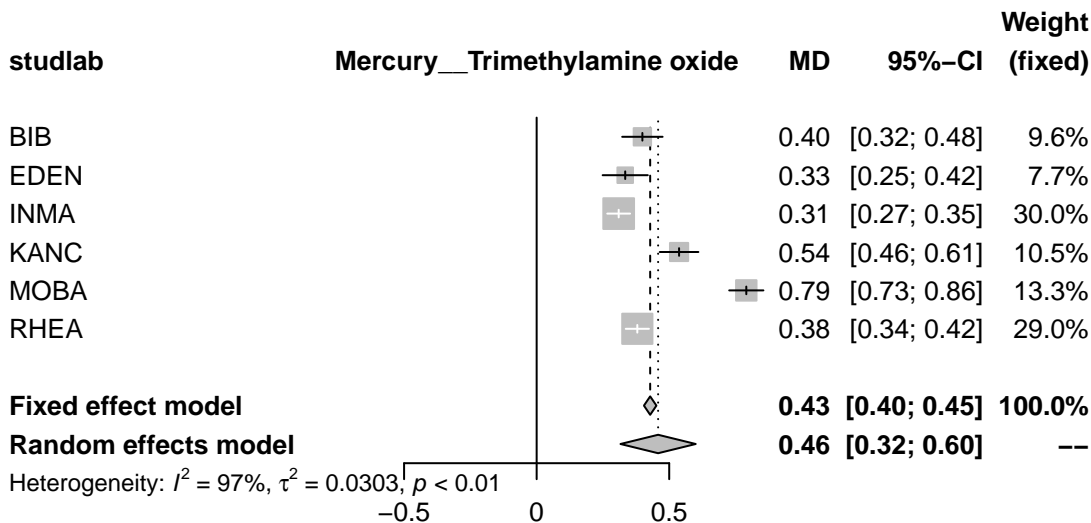

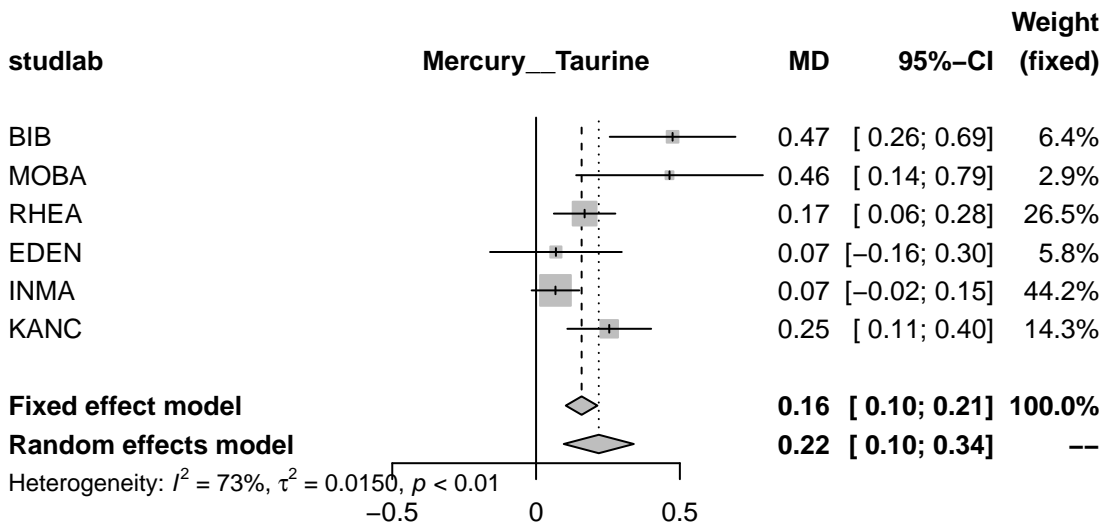

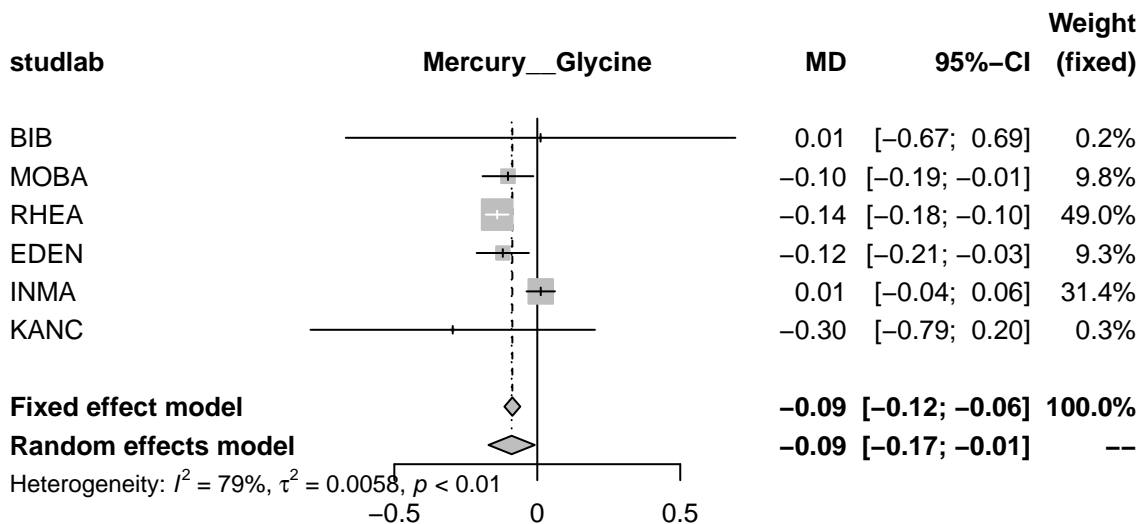

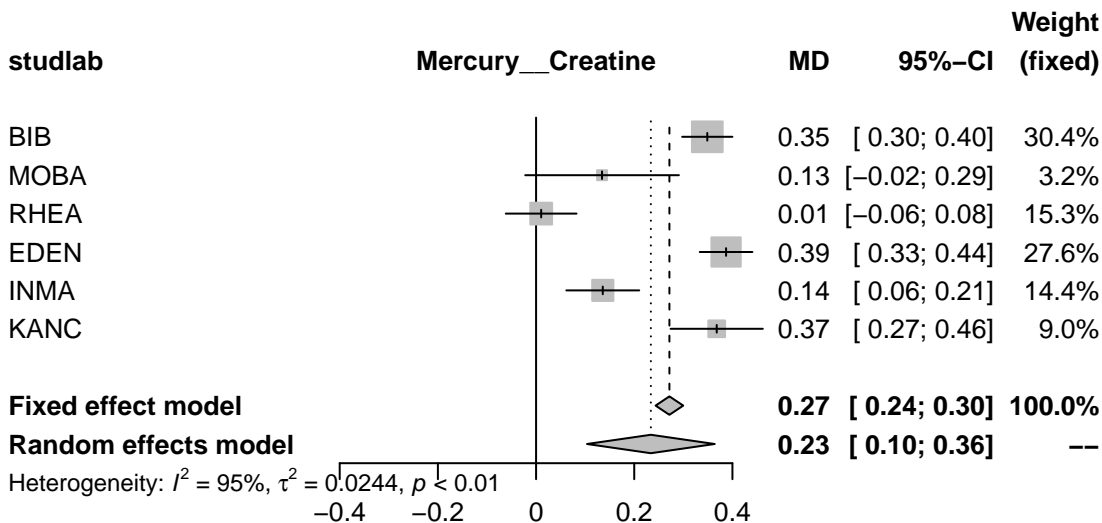

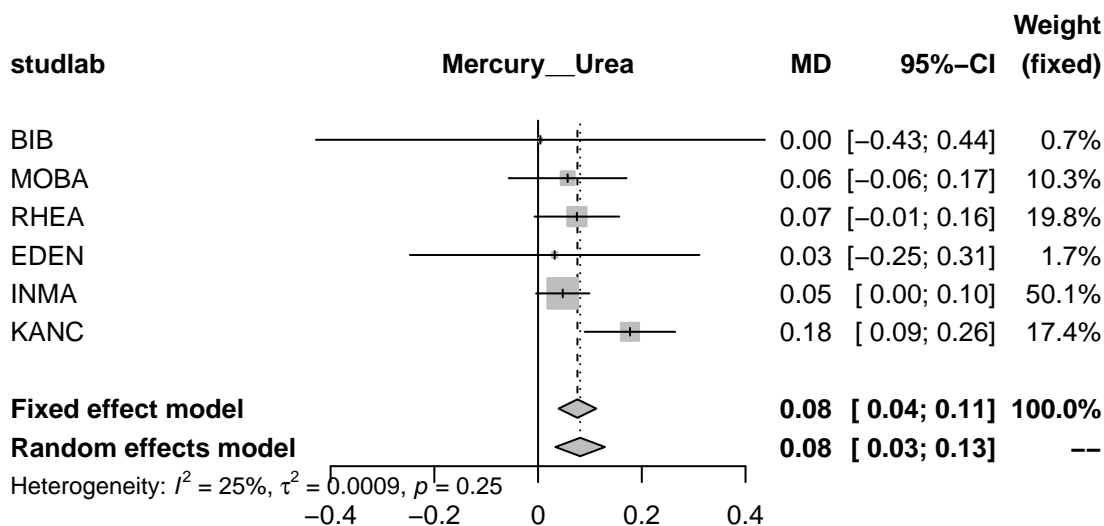

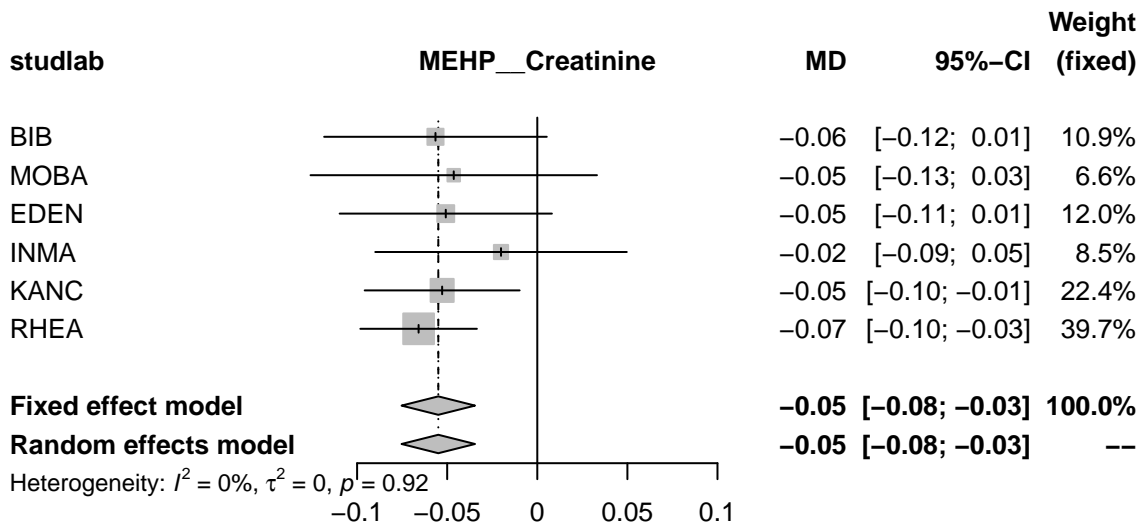

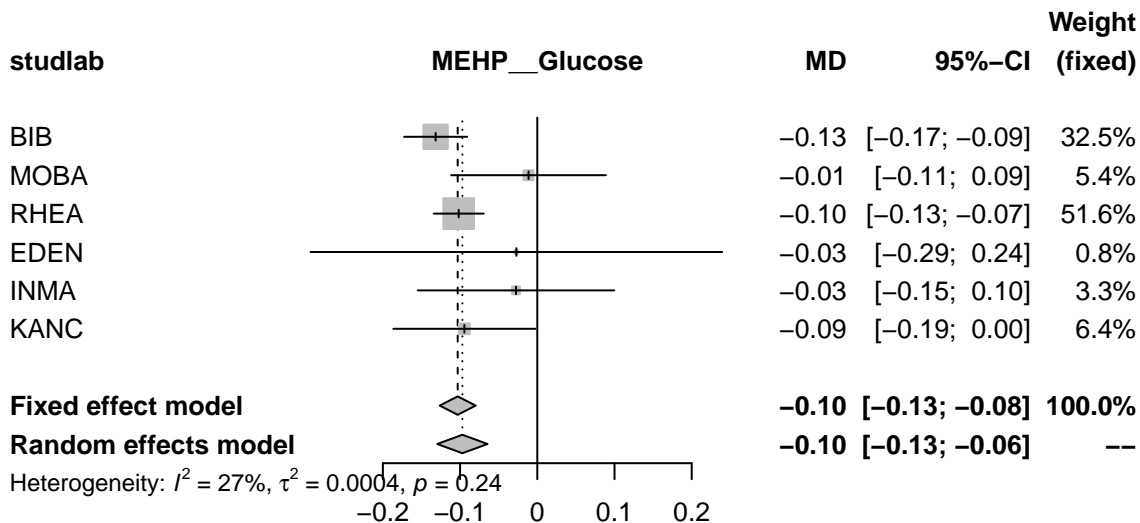

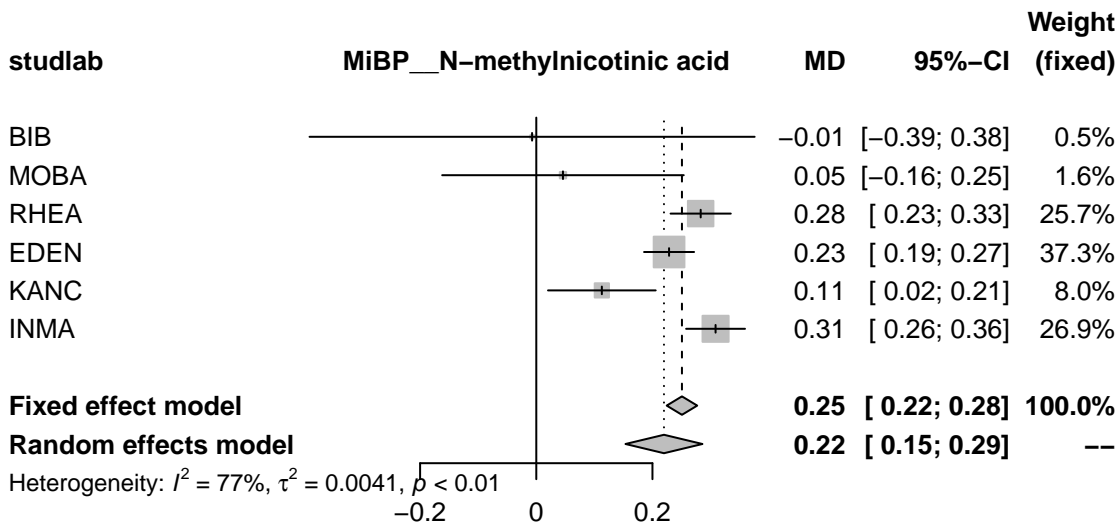

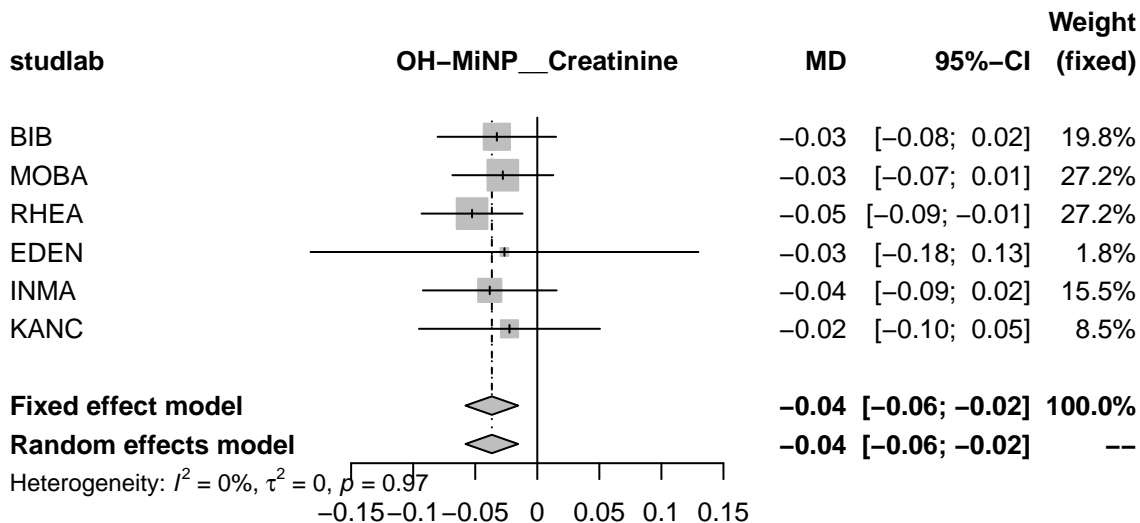

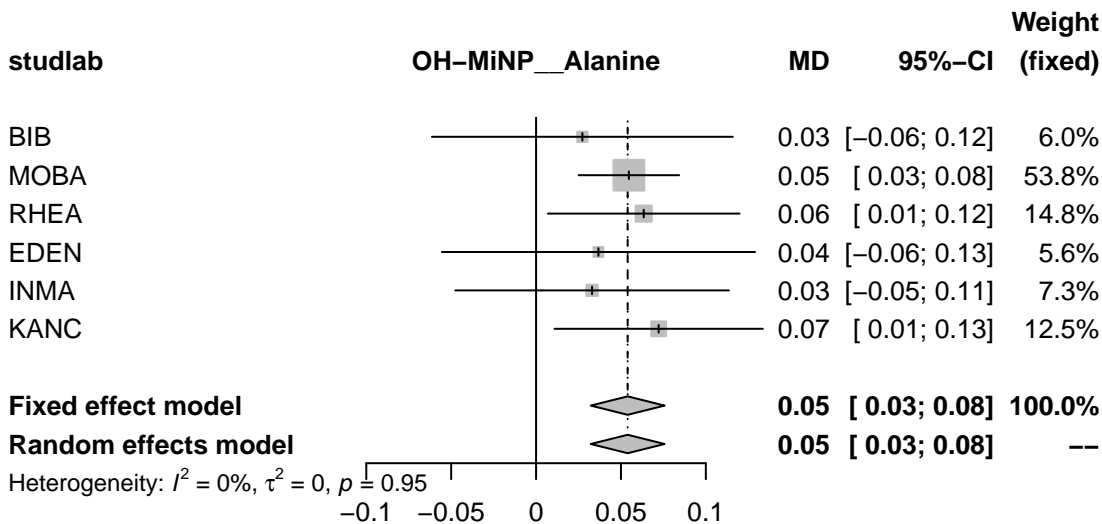

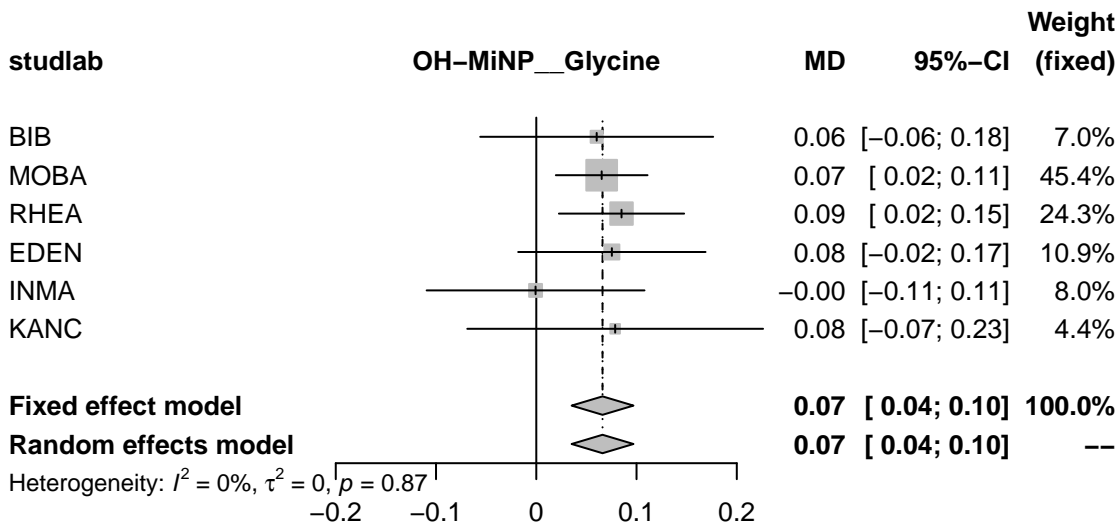

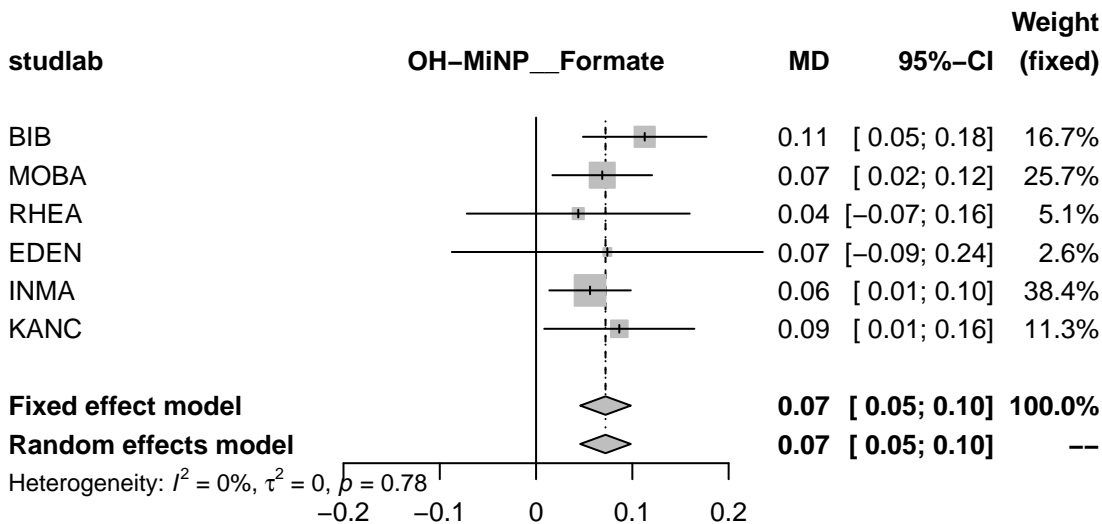

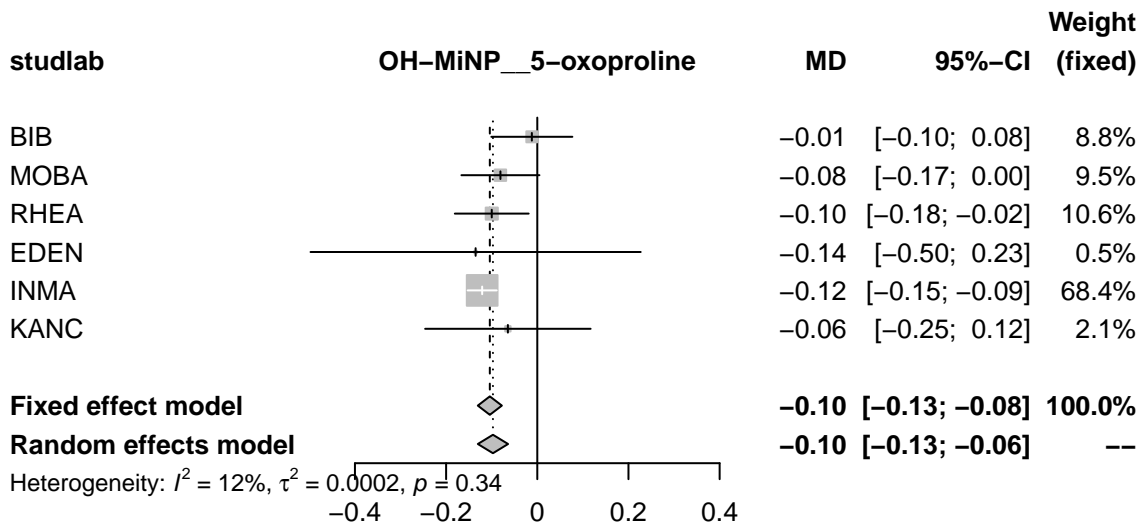

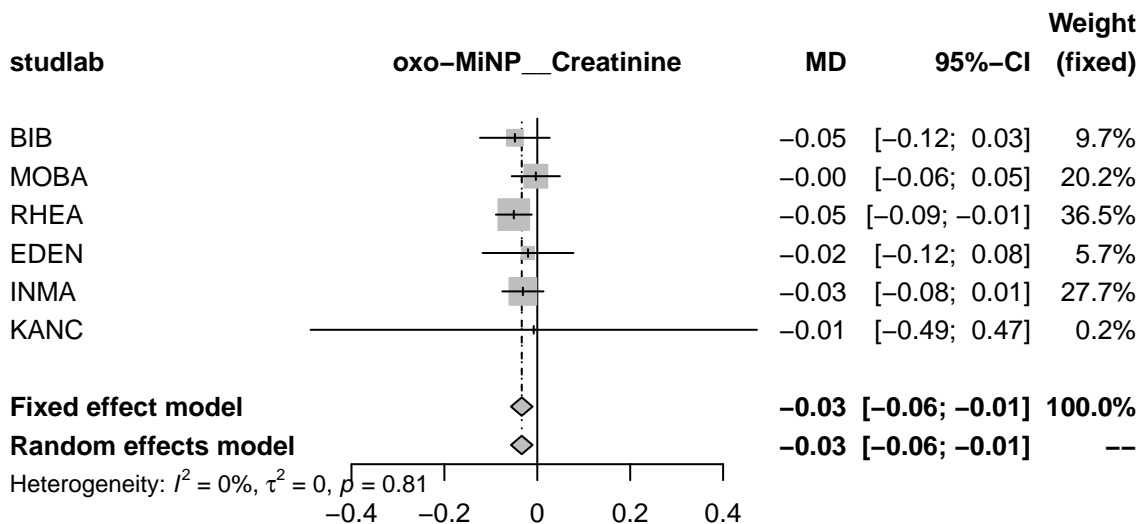

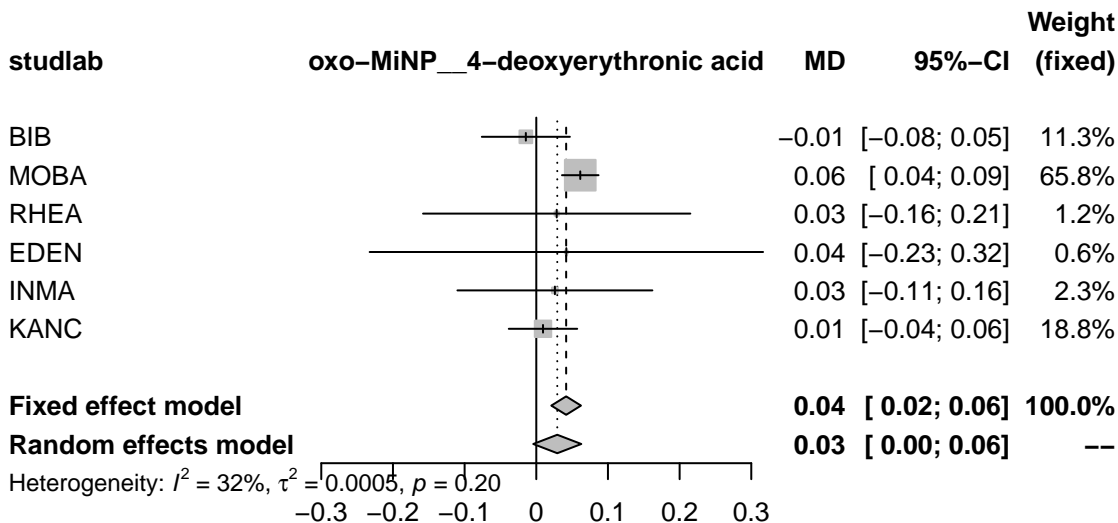

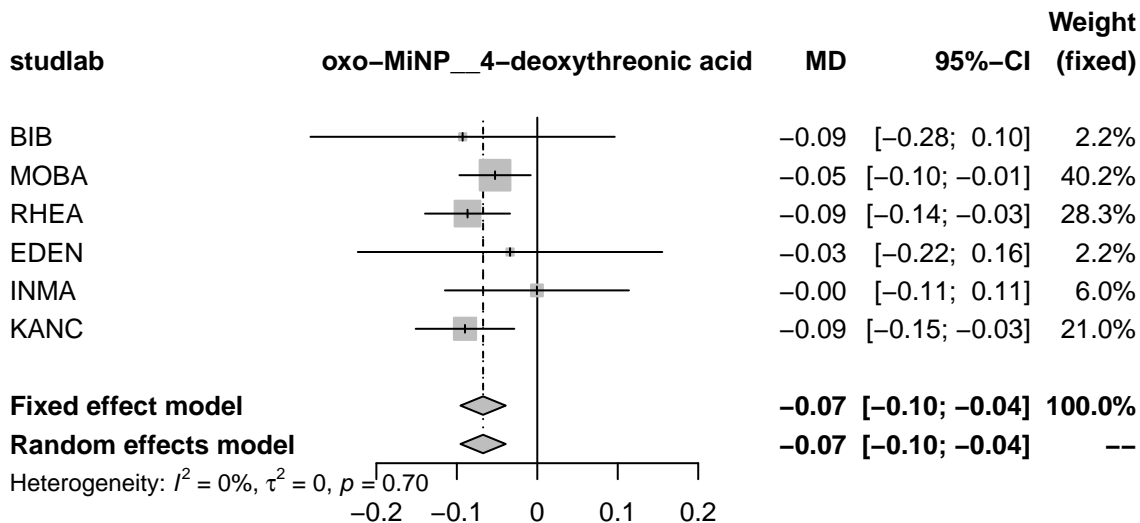

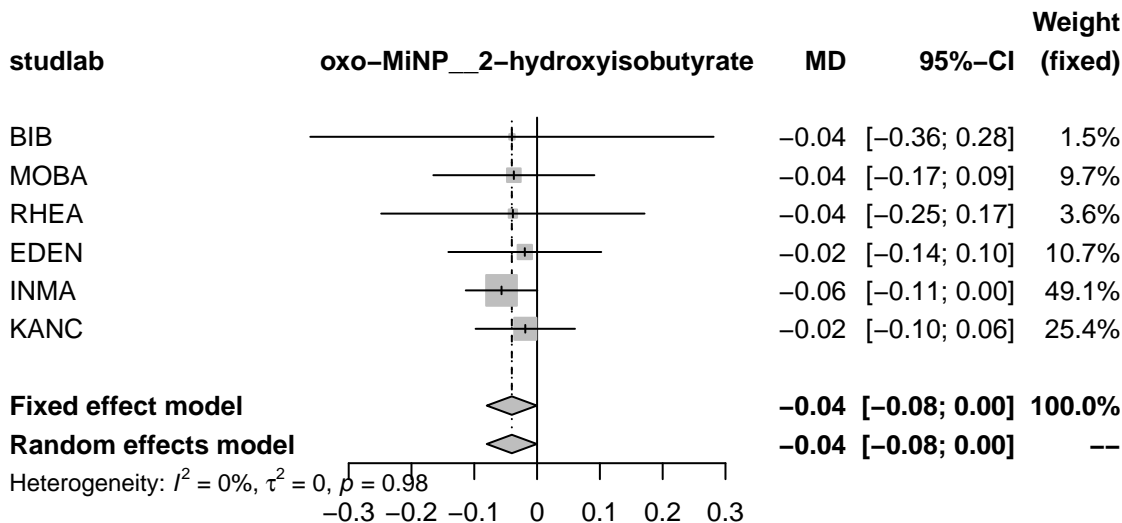

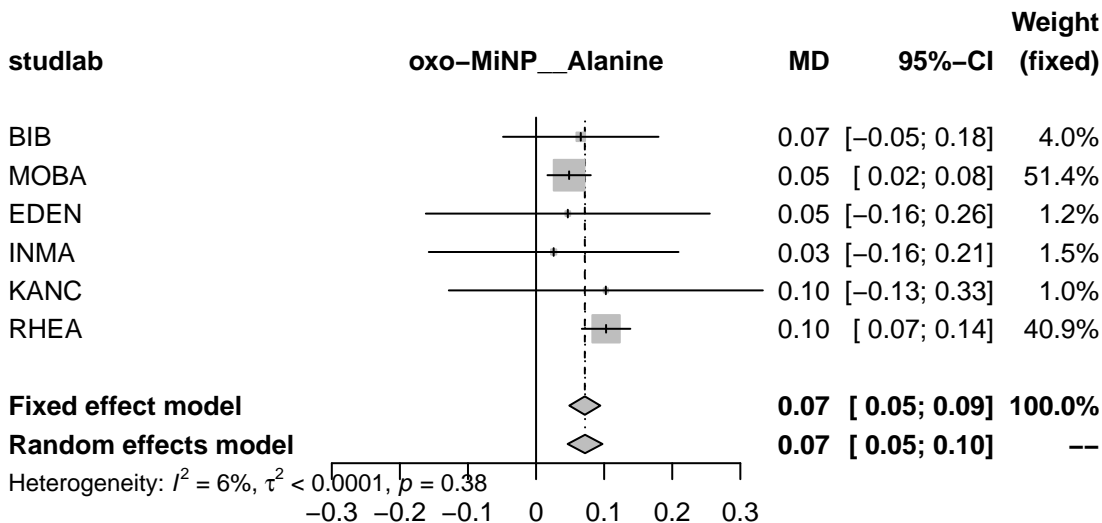

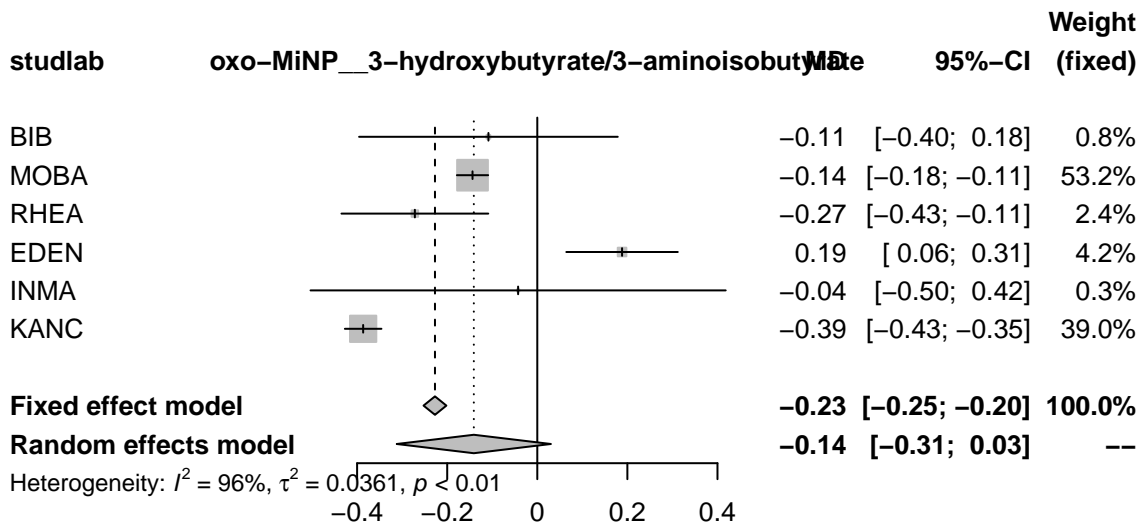

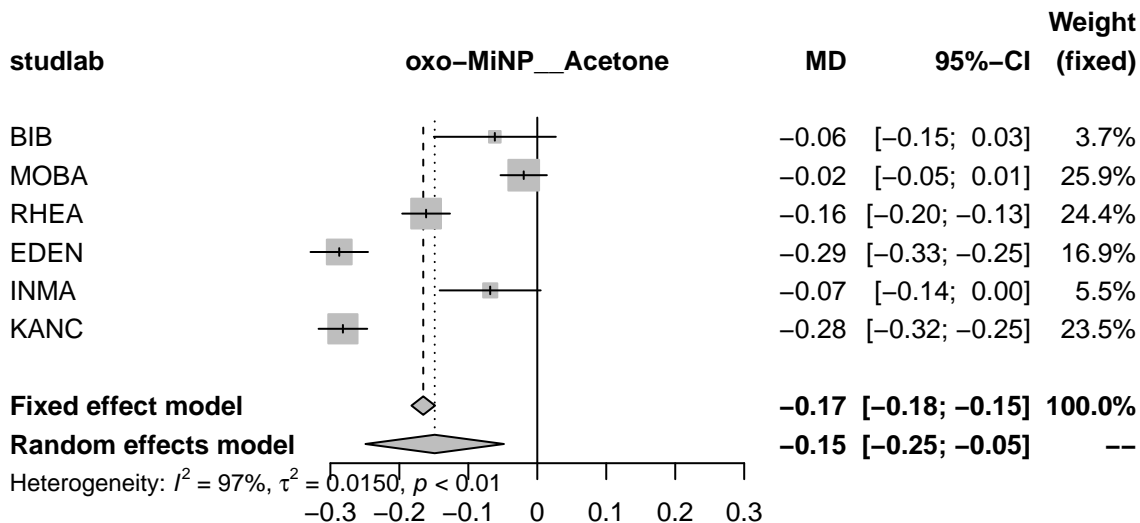

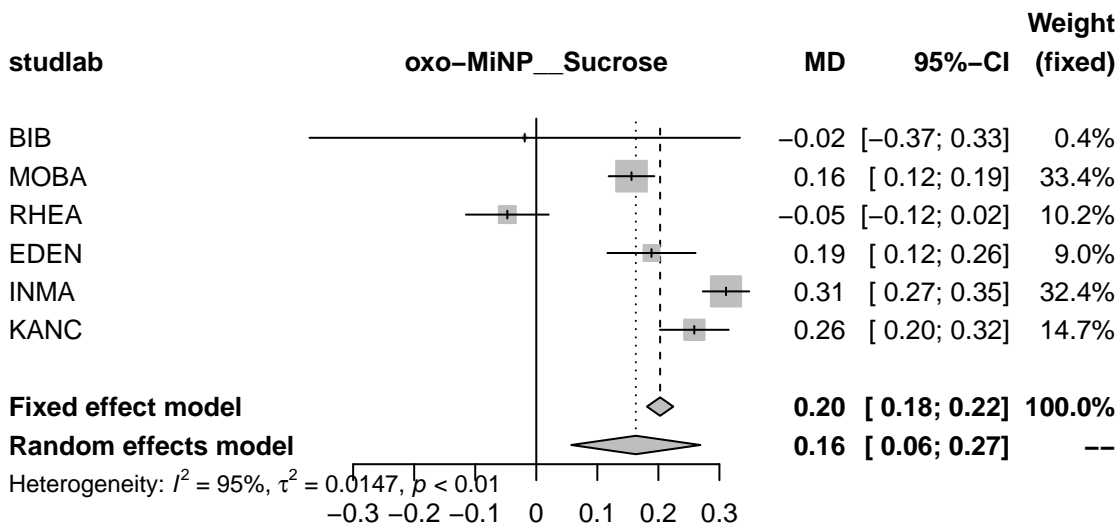

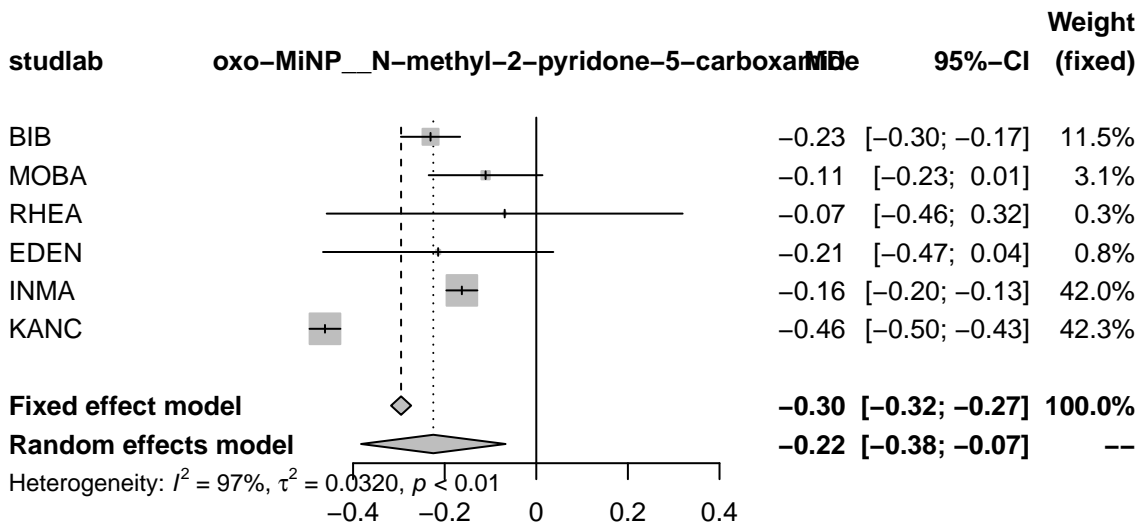

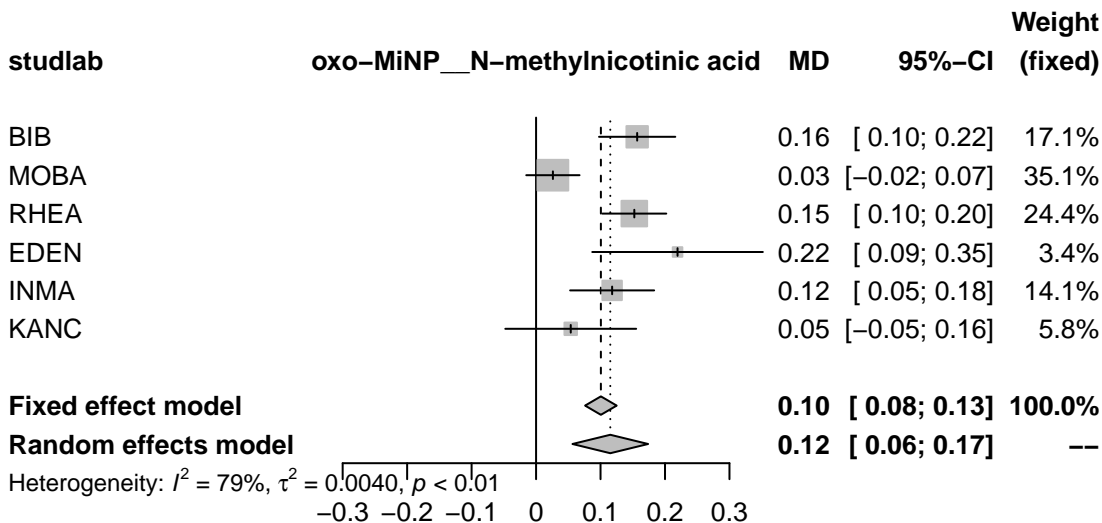

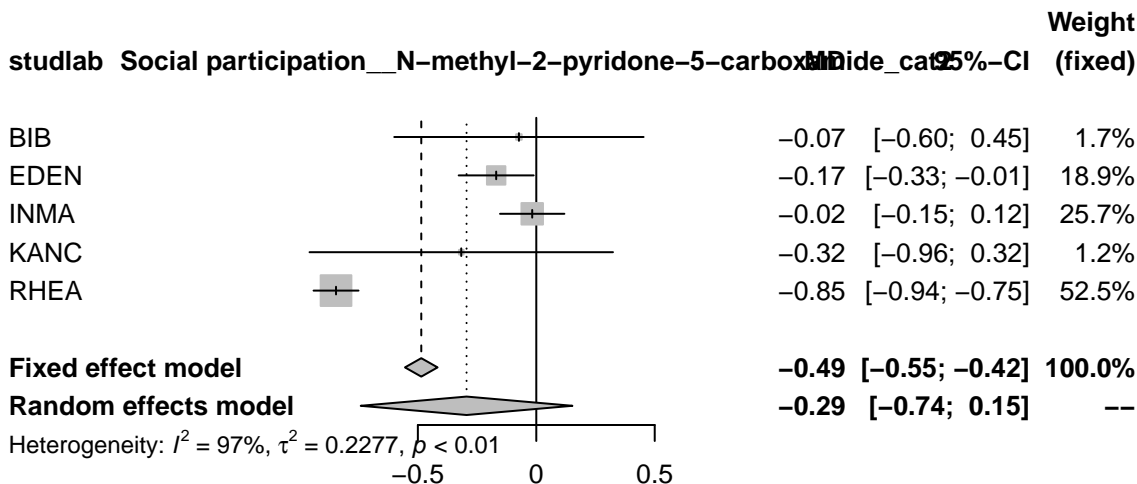

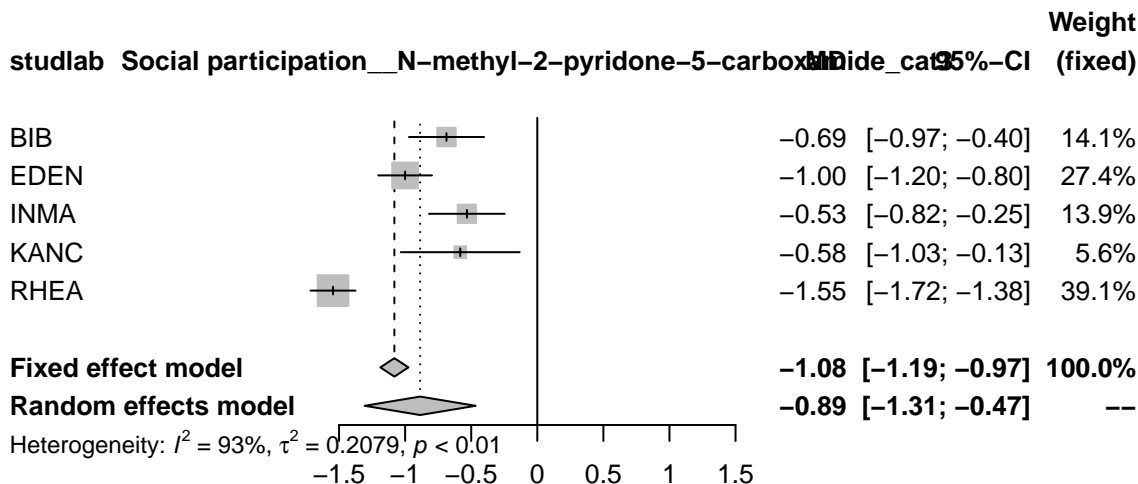

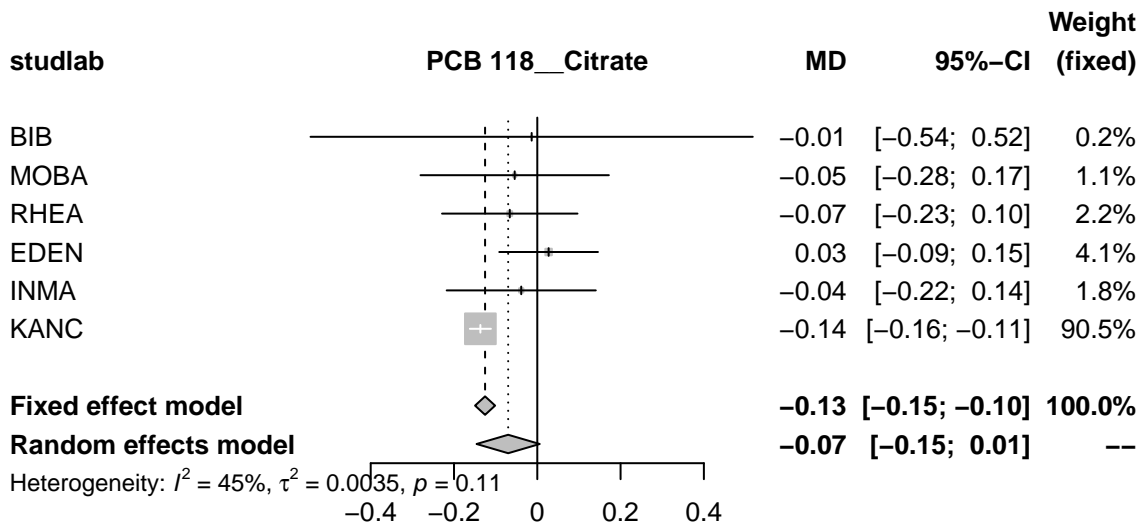

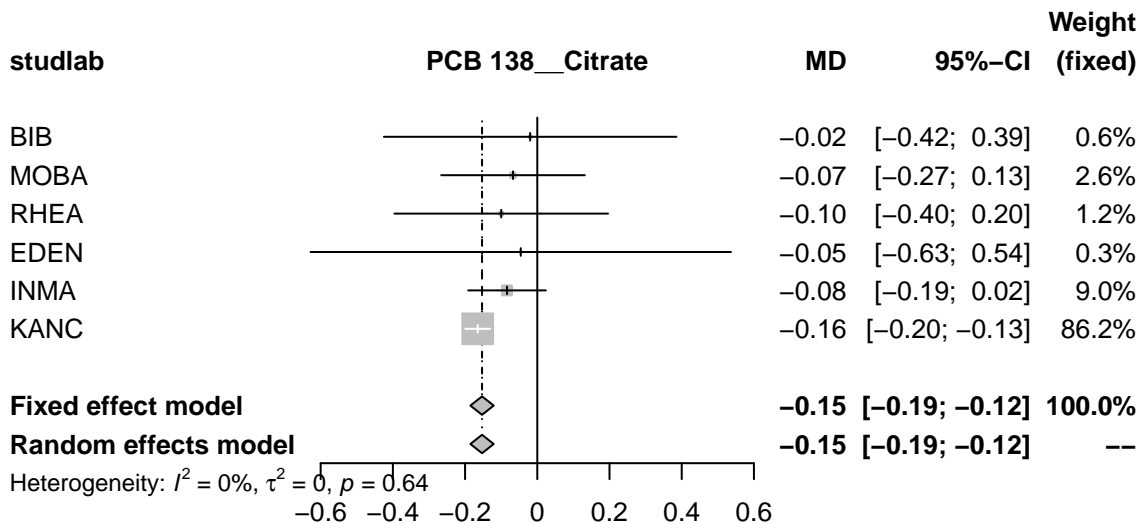

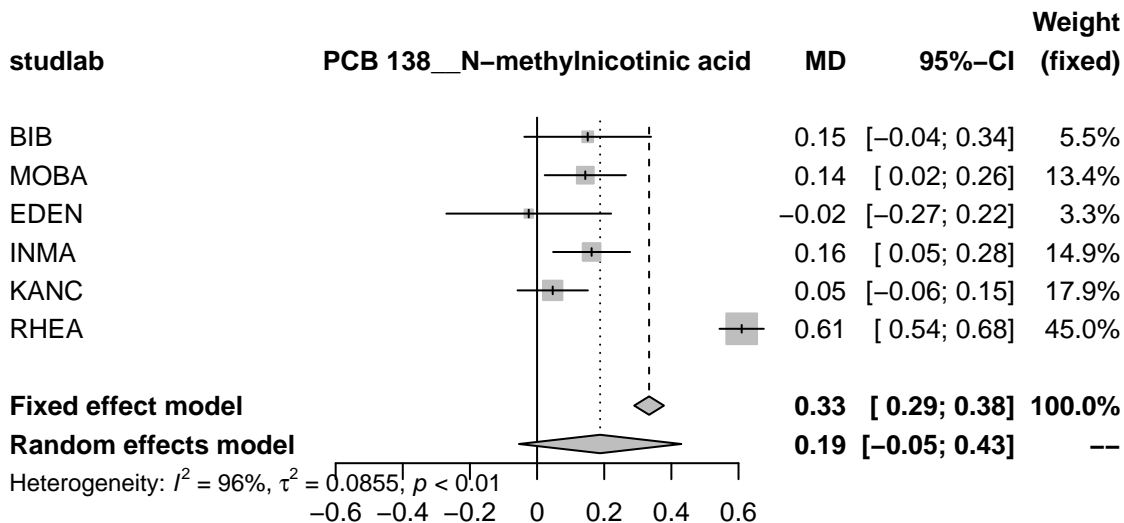

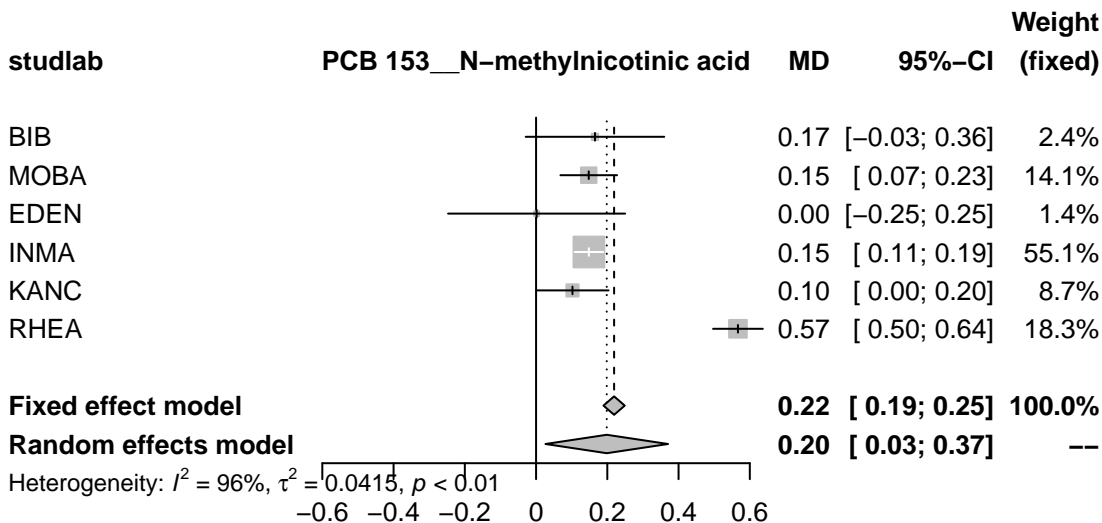

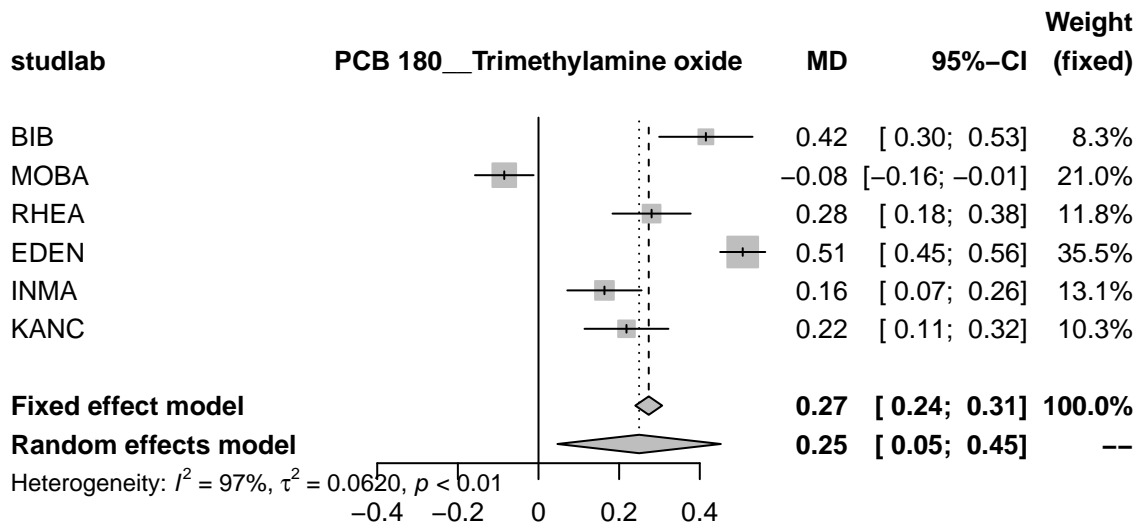

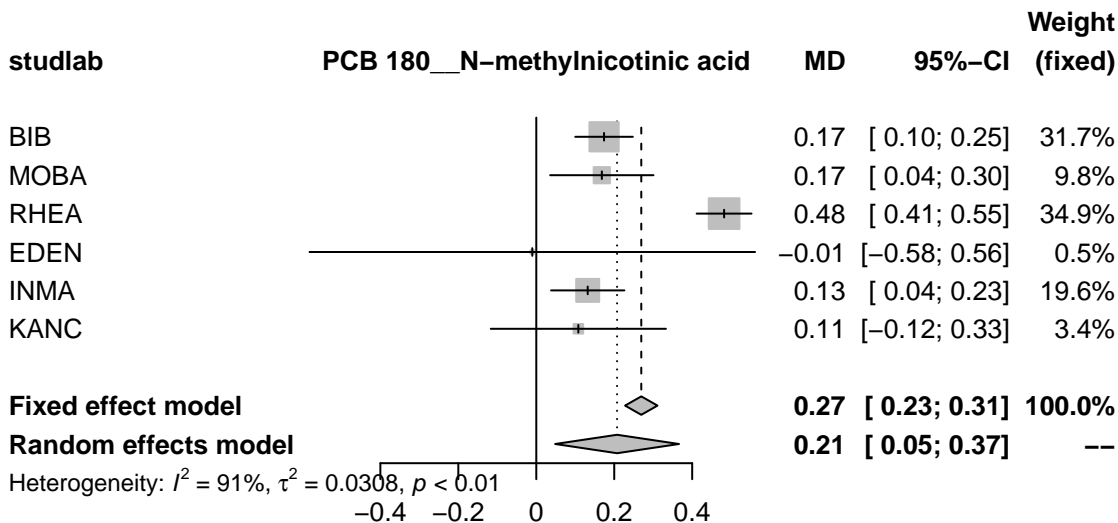

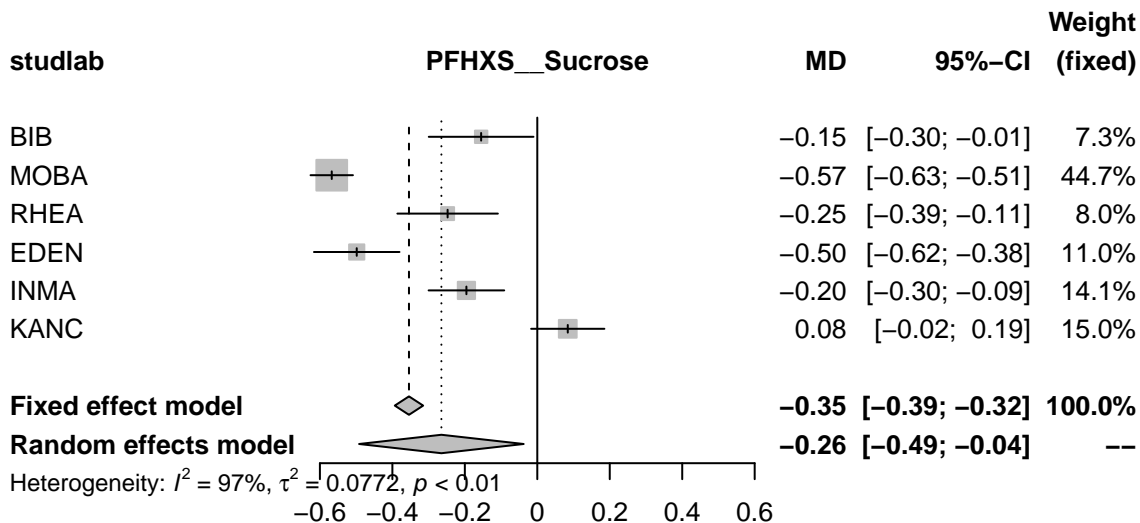

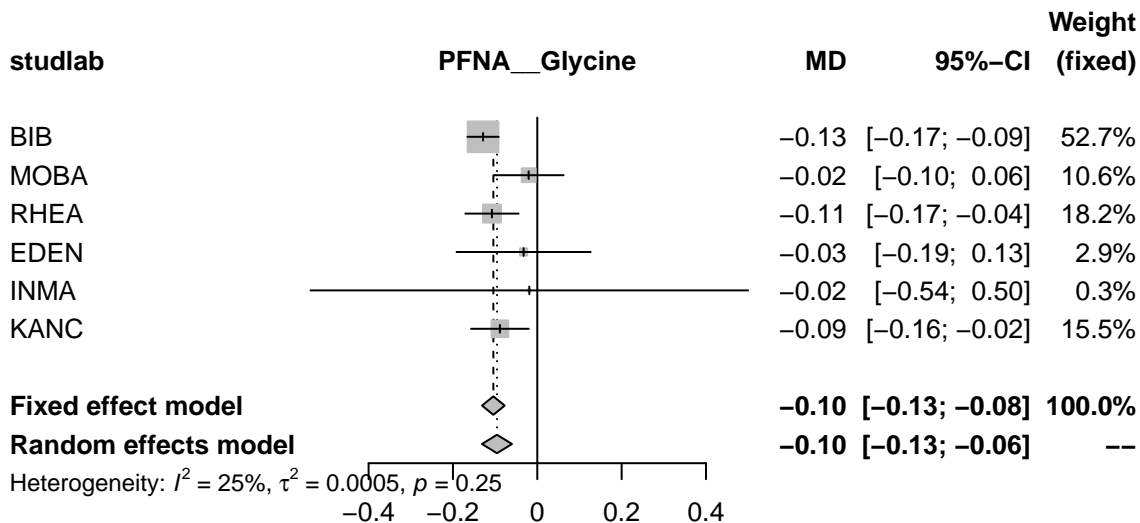

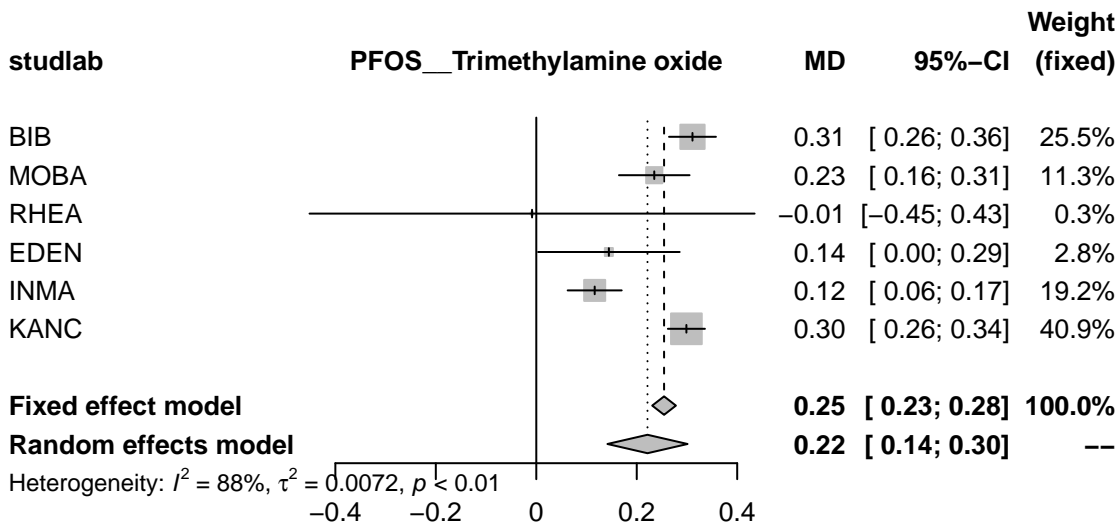

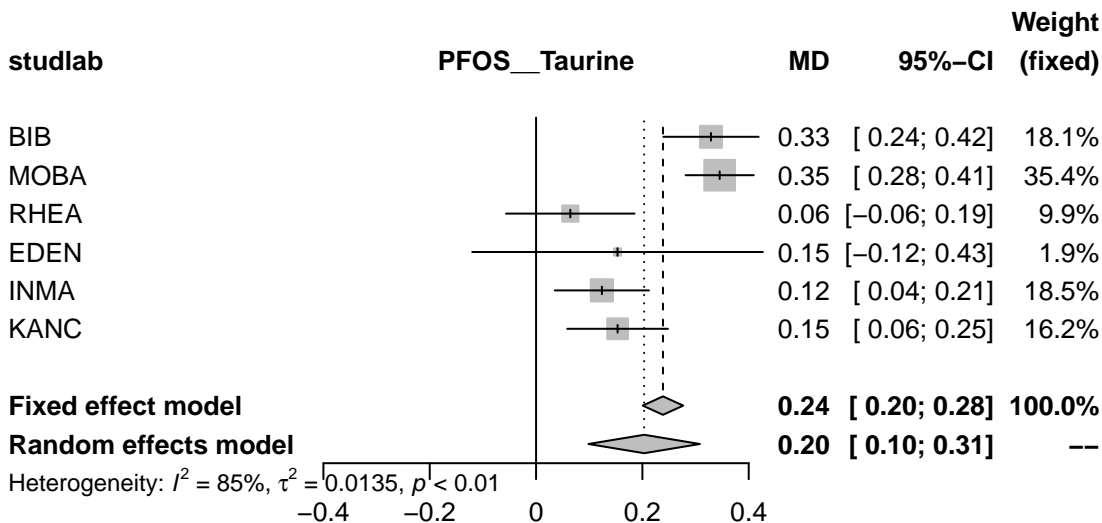

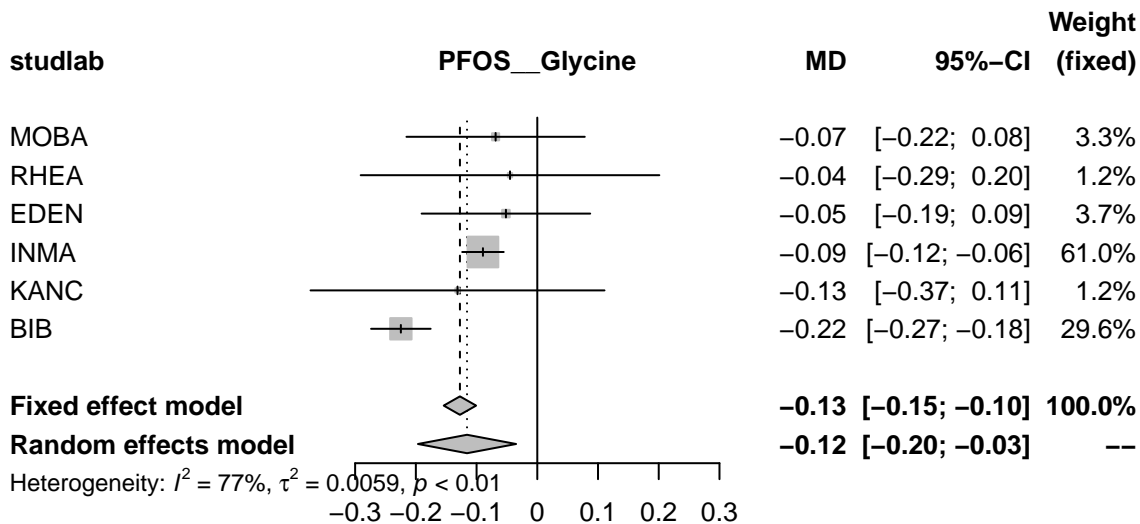

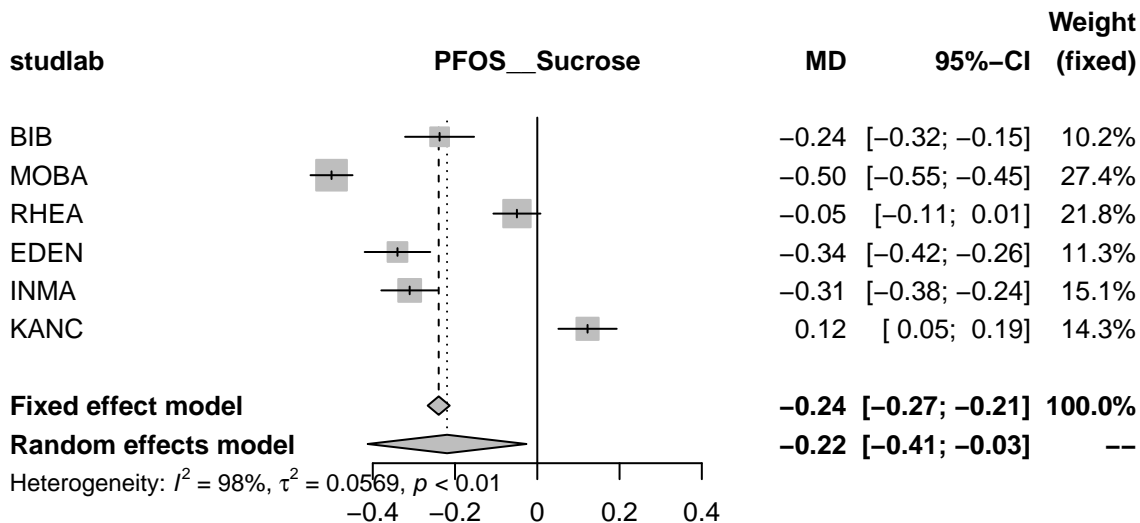

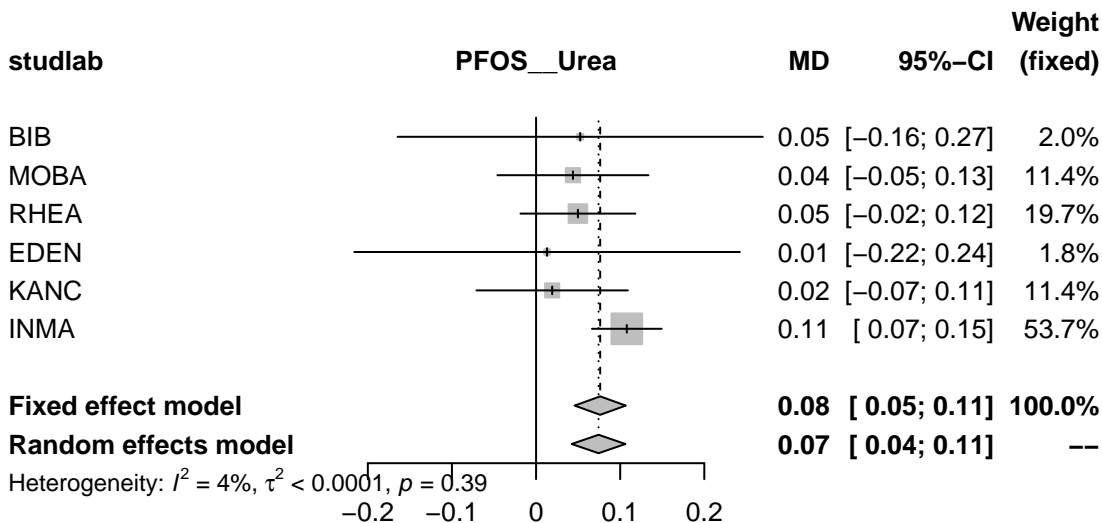

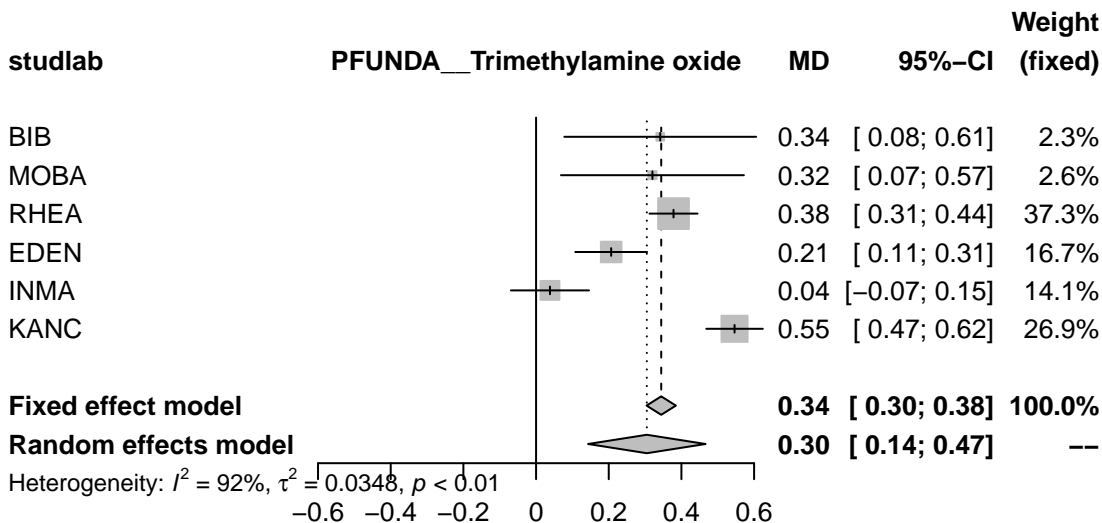

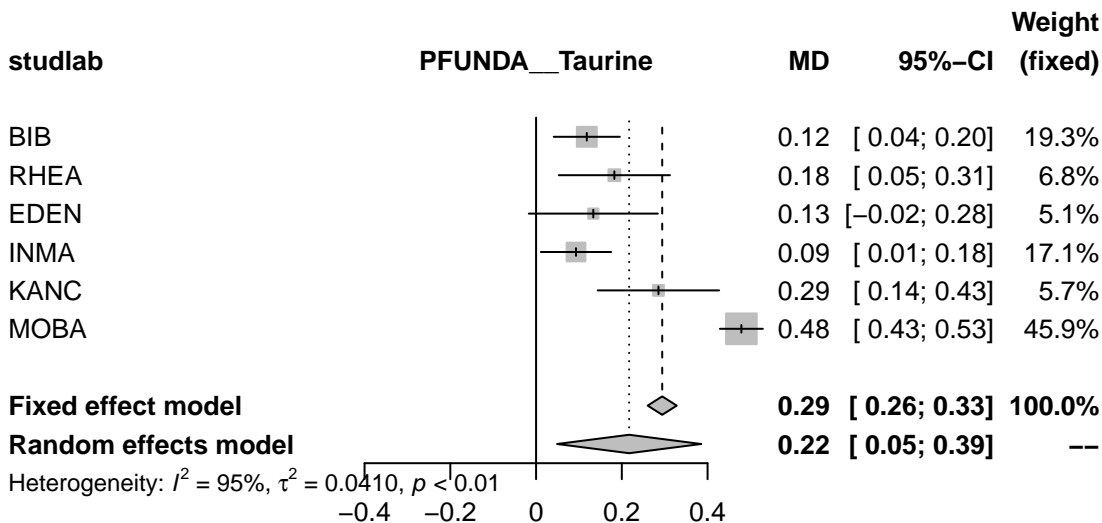

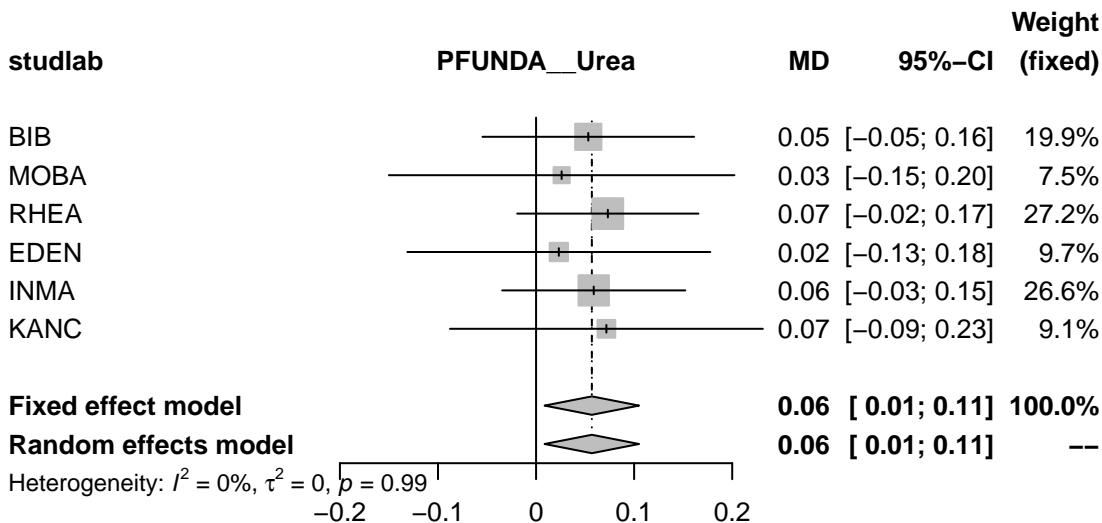

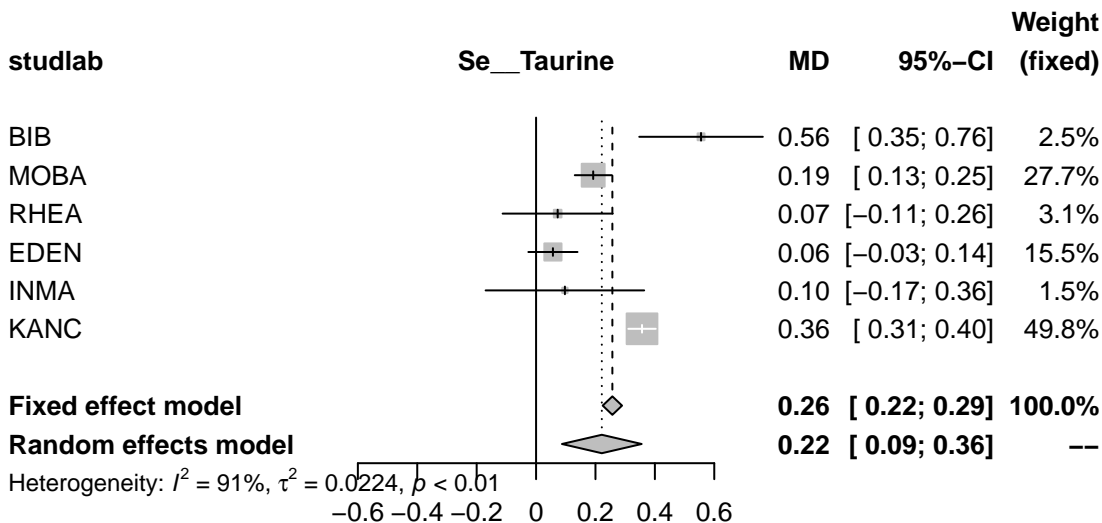

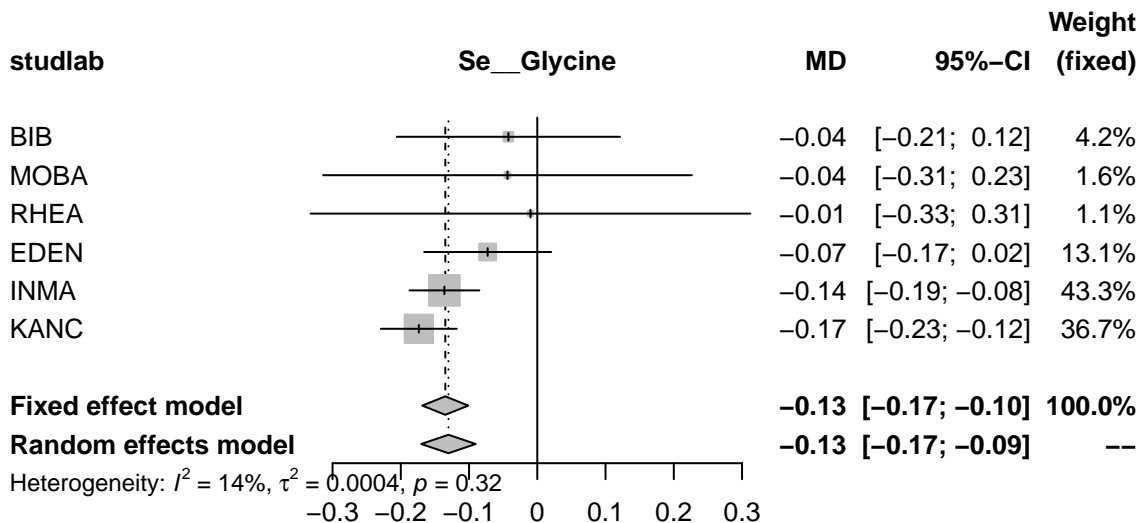

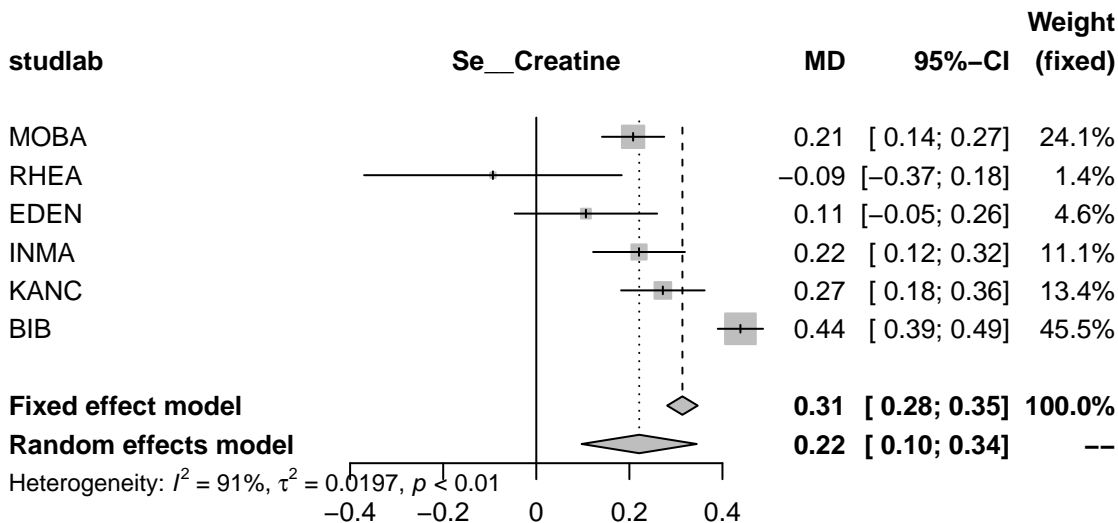

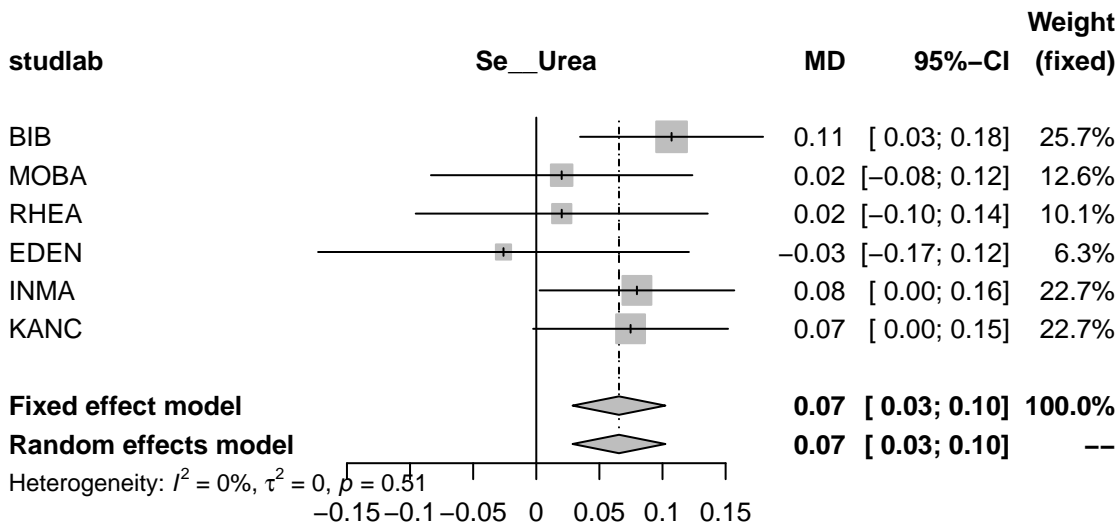

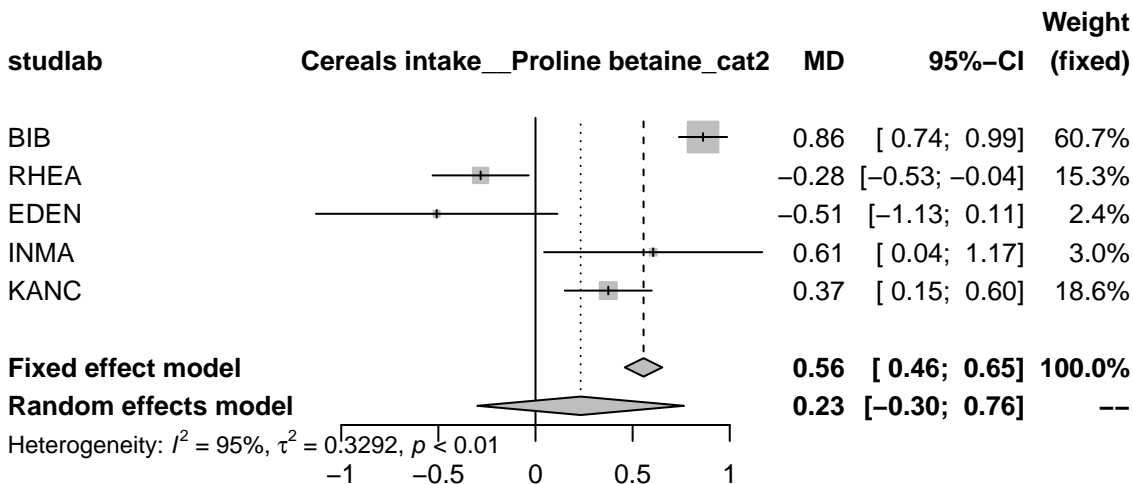

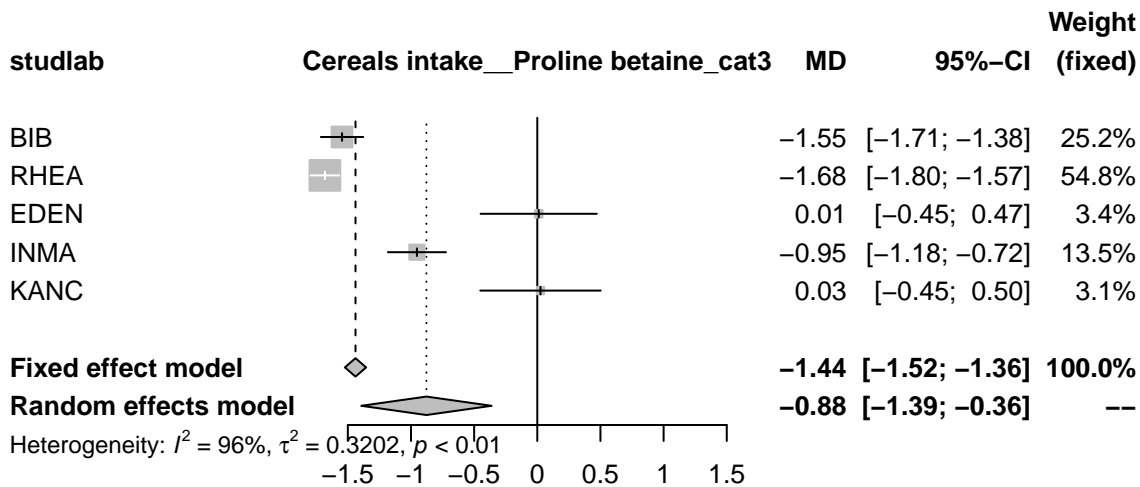

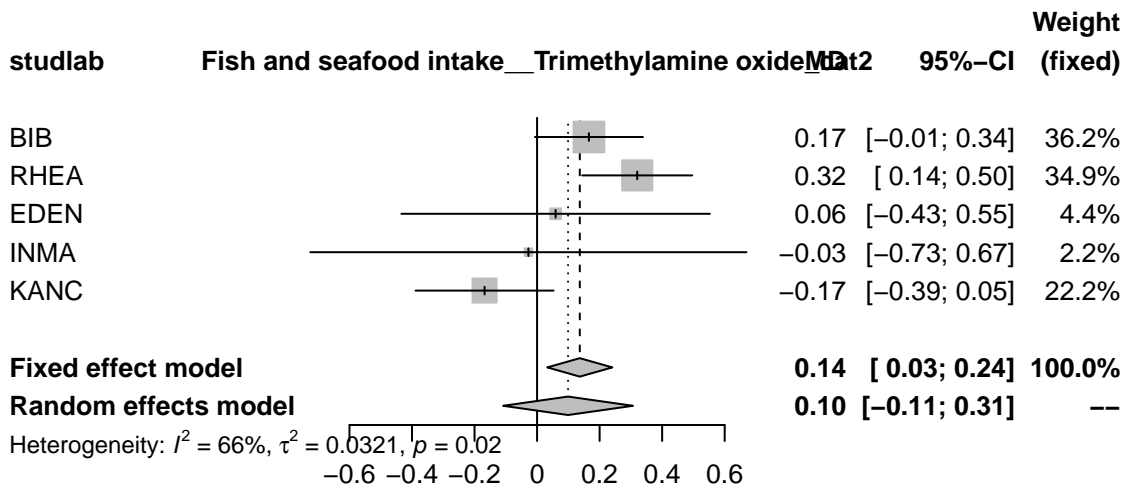

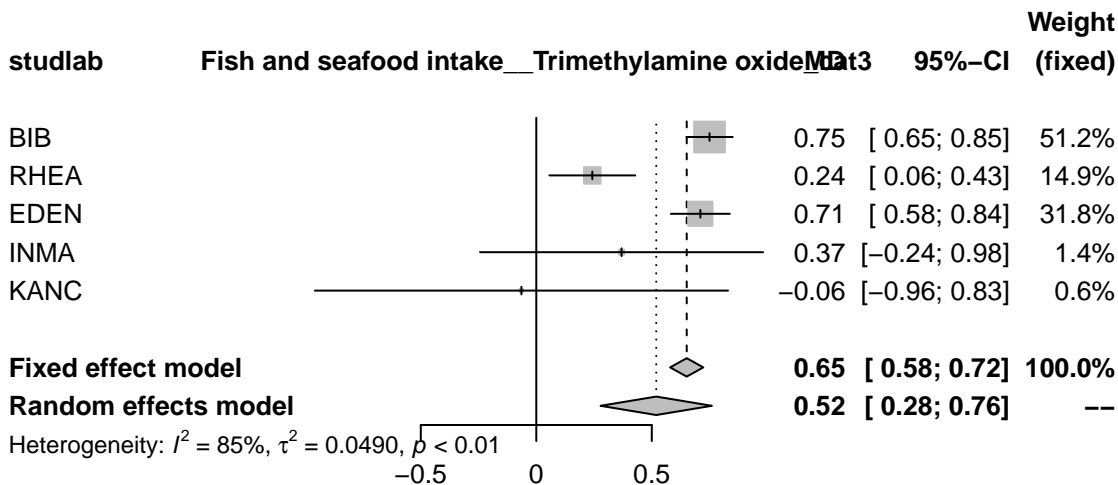

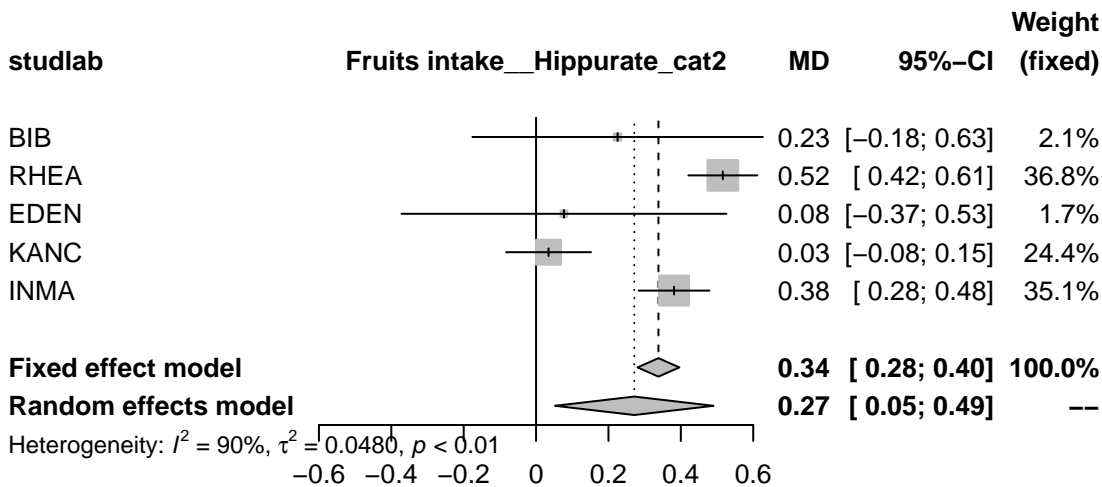

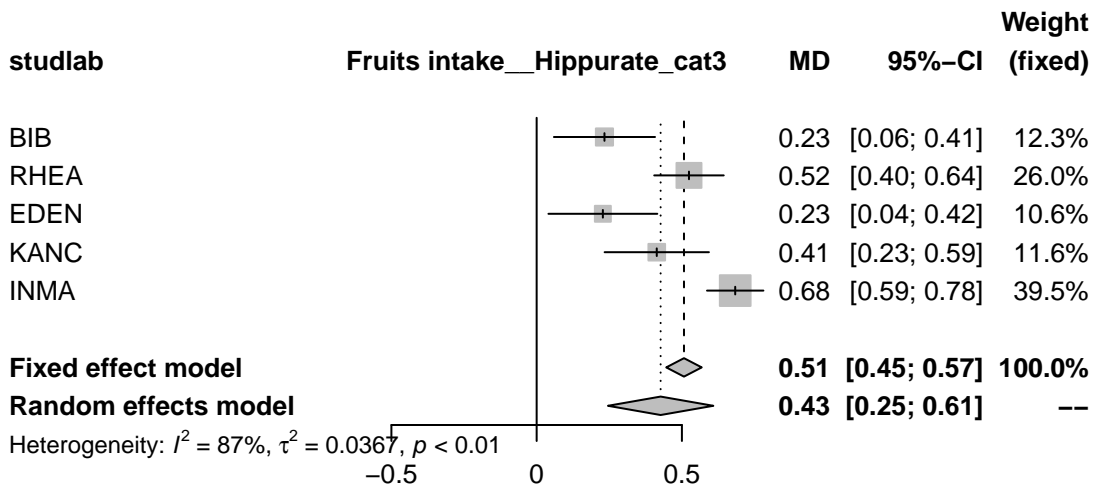

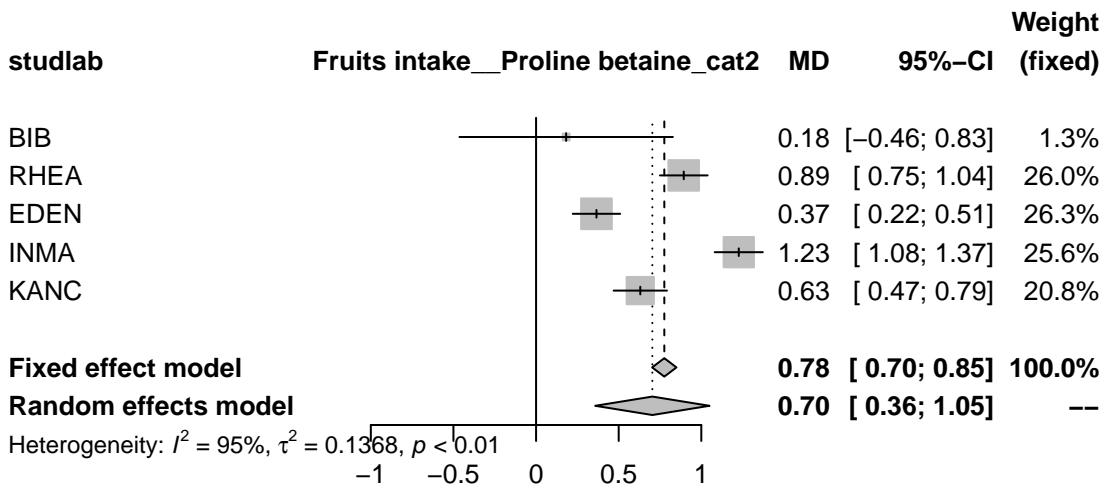

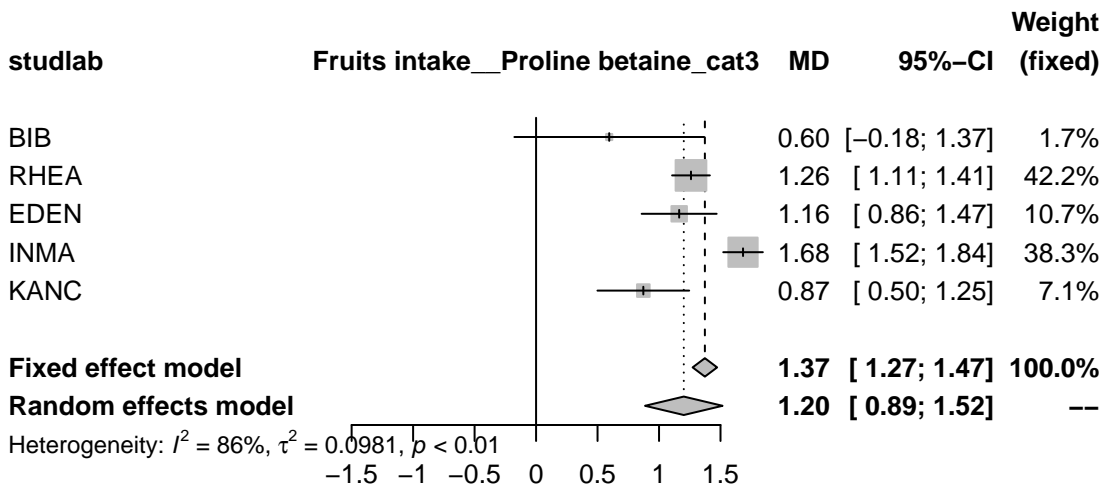

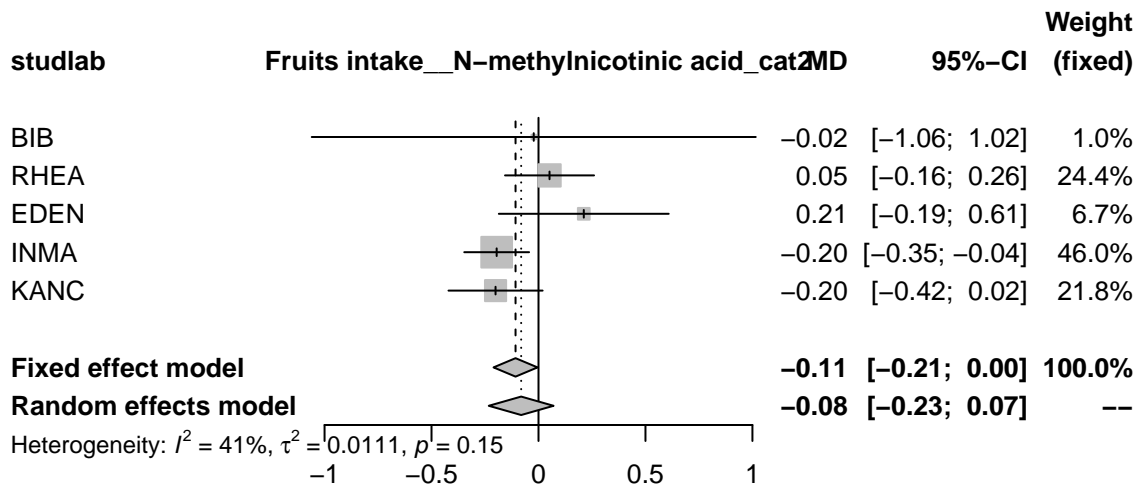

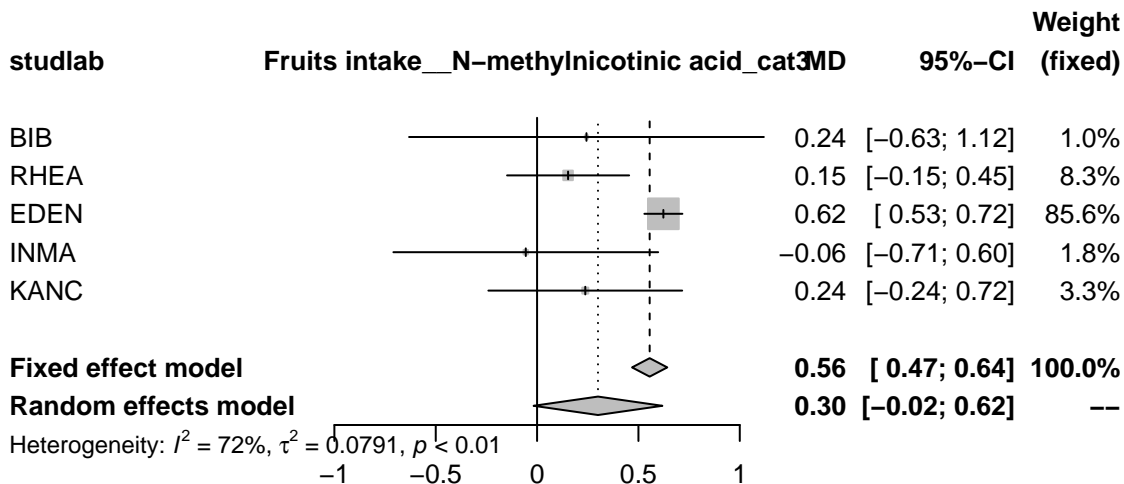

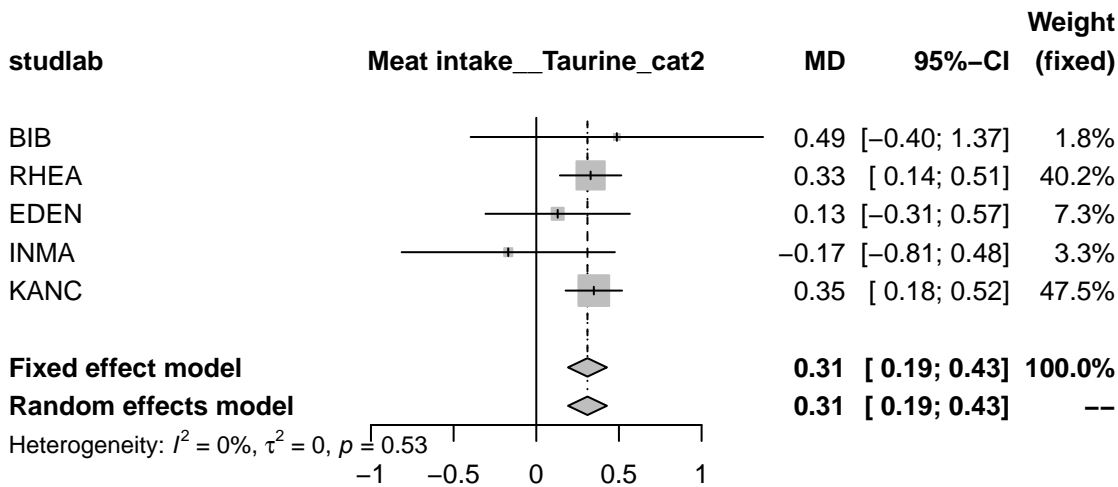

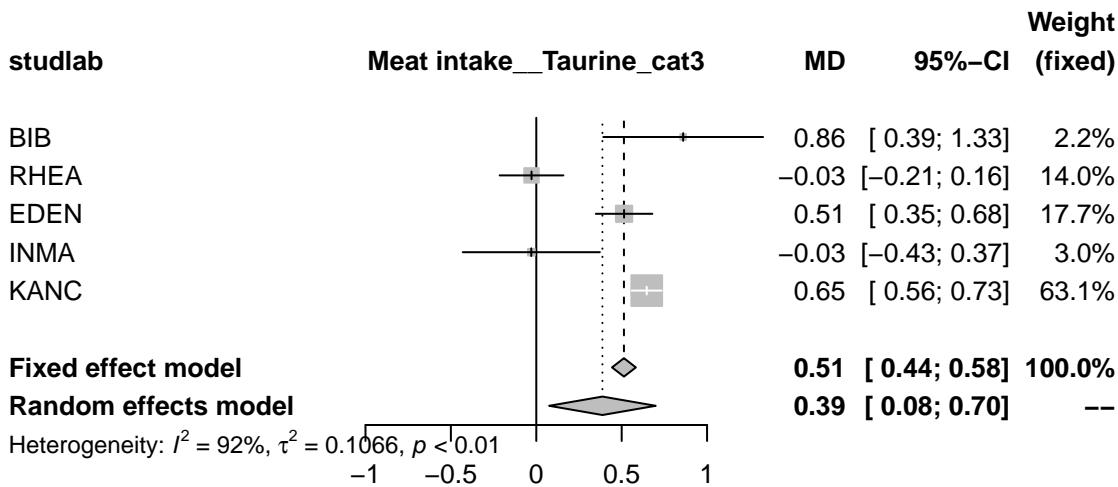

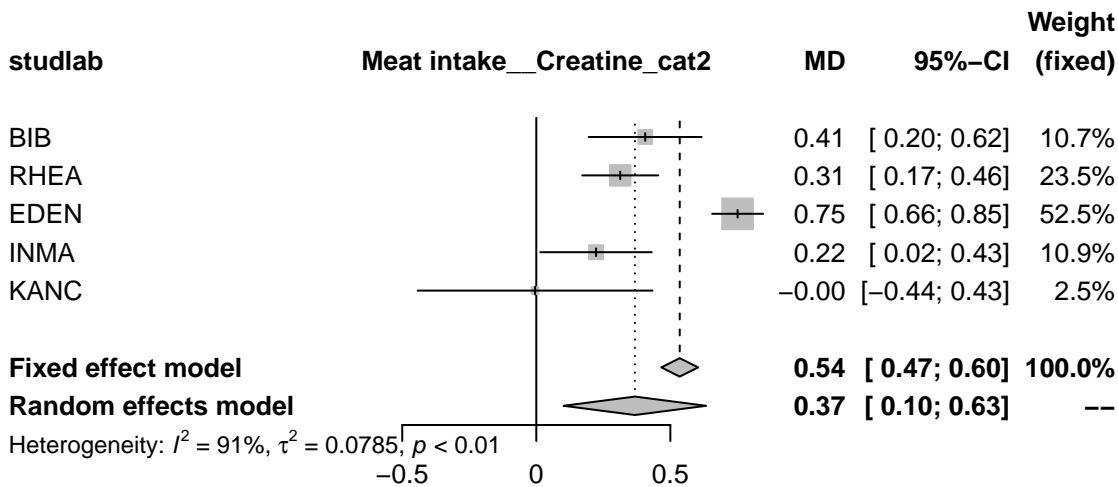

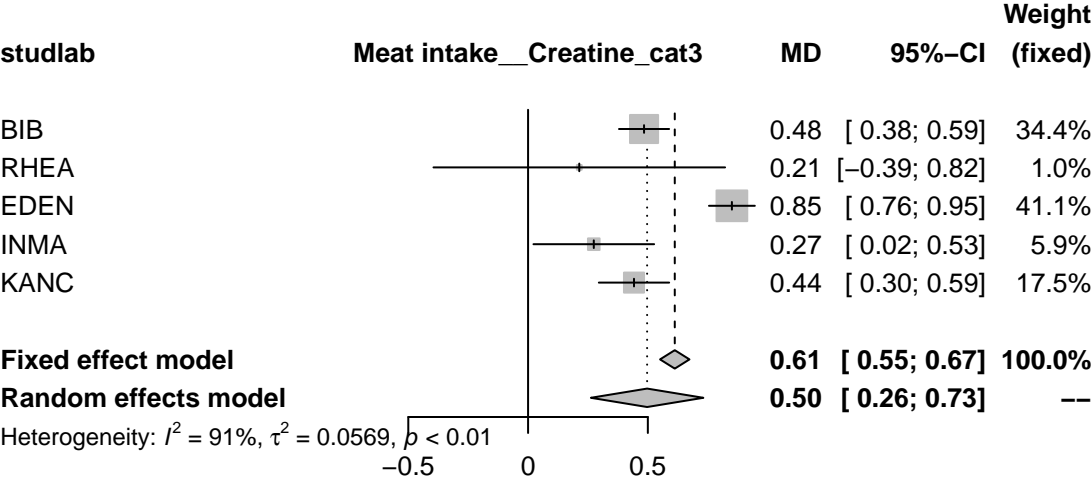

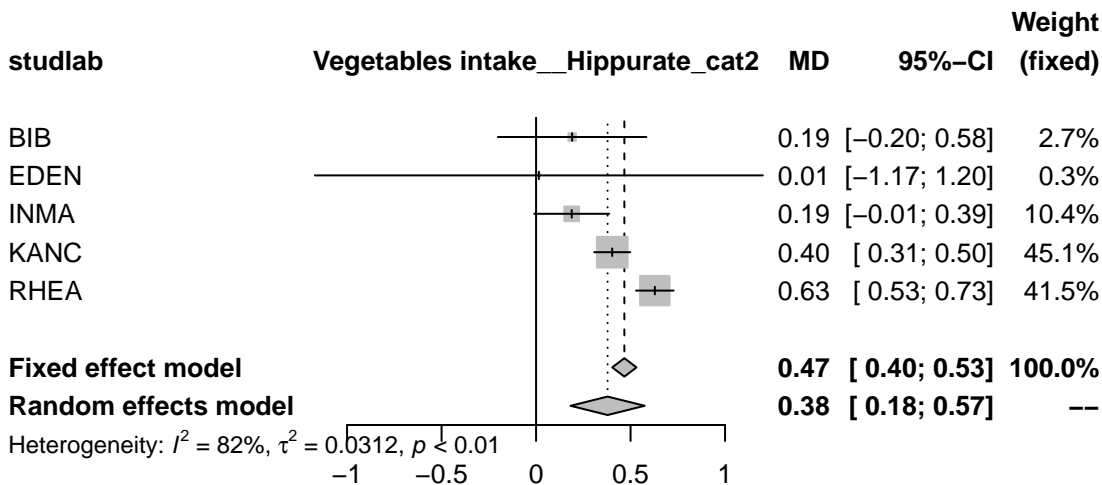

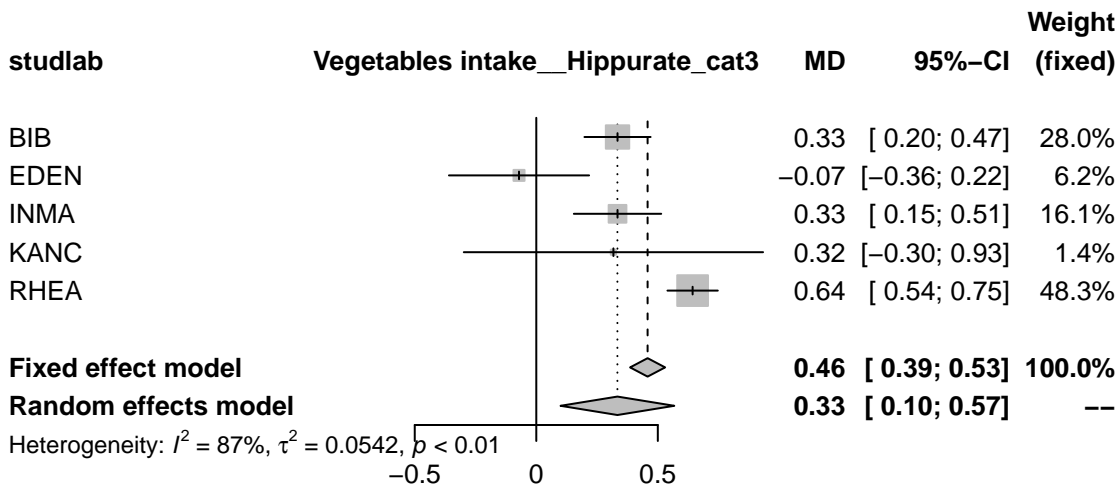

Supplement: Supplementary file 14 — Supplementary Dataset 11 [file 41467_2022_34422_MOESM14_ESM.zip › HELIX_ExpOmics_FigS2_Forestplots/HELIX_ExpOmics_FigS2L_met_u_post.pdf]

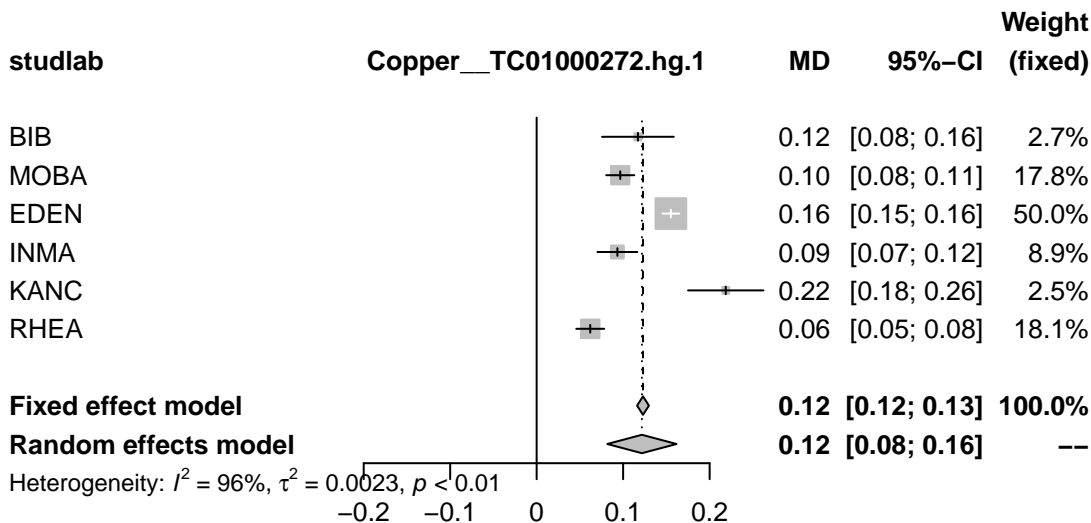

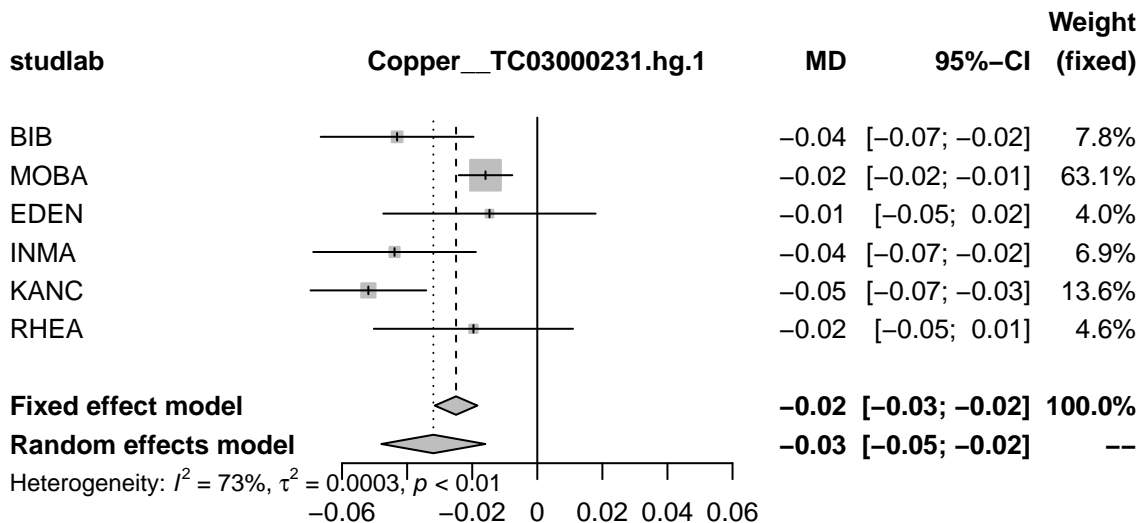

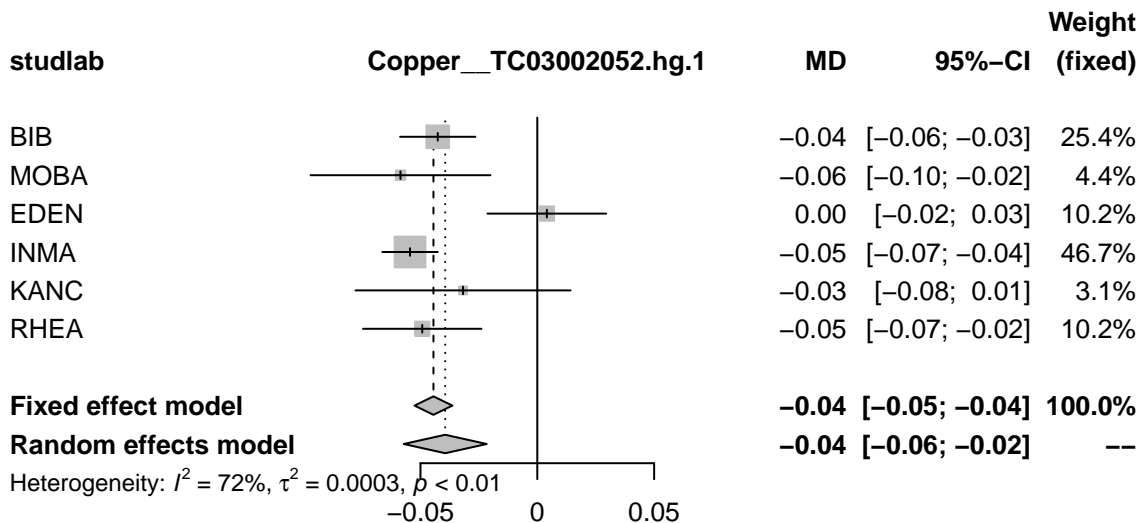

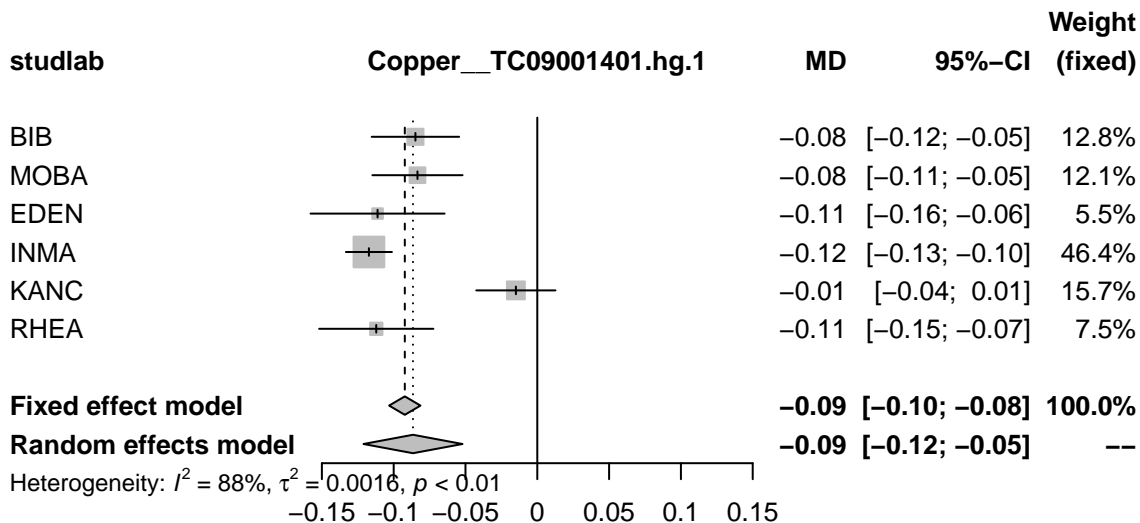

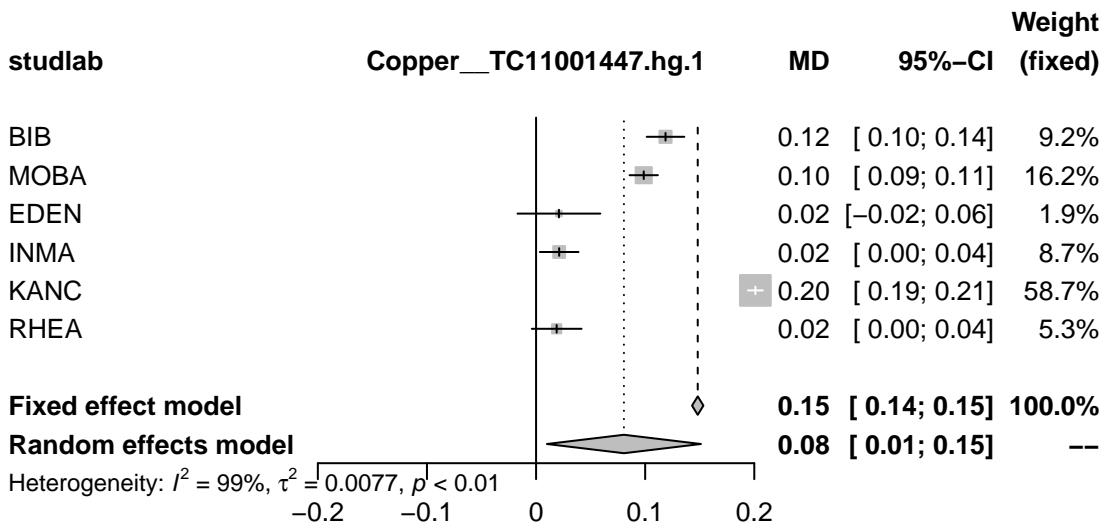

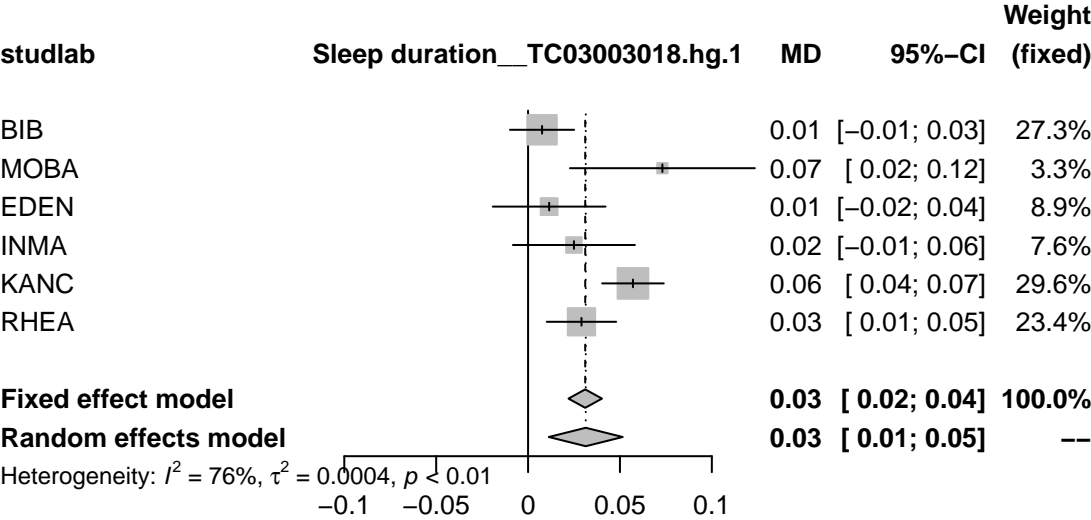

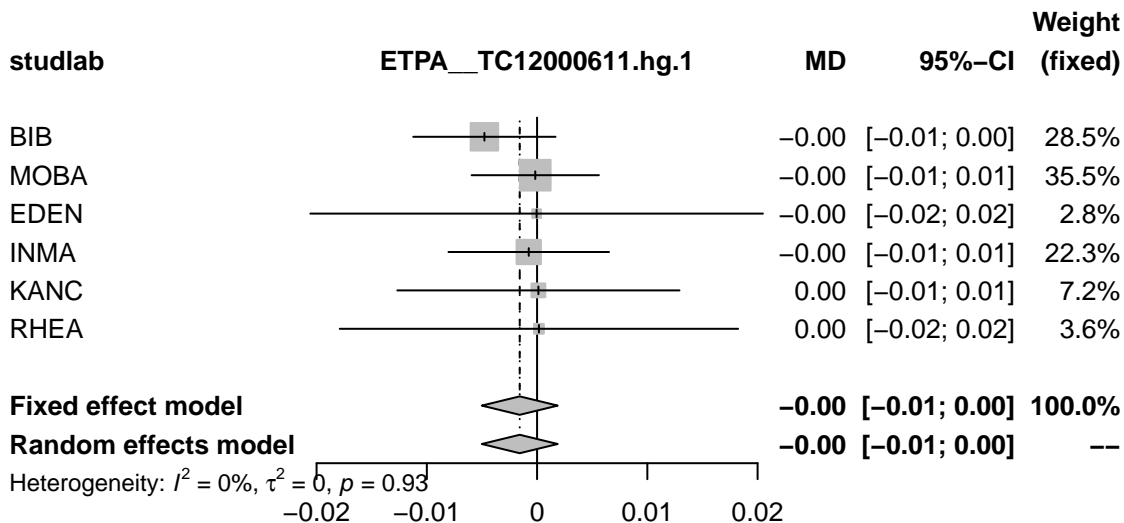

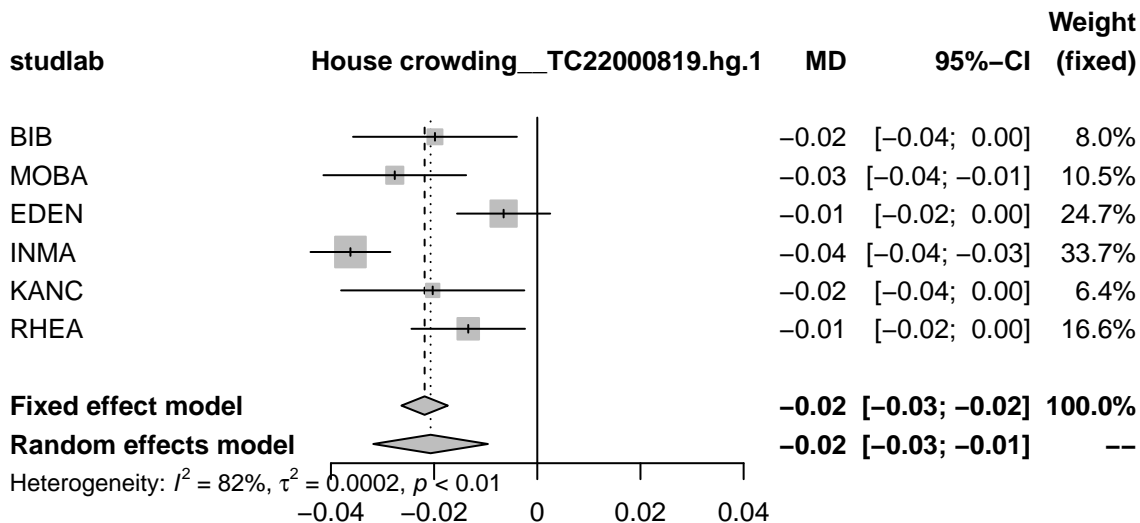

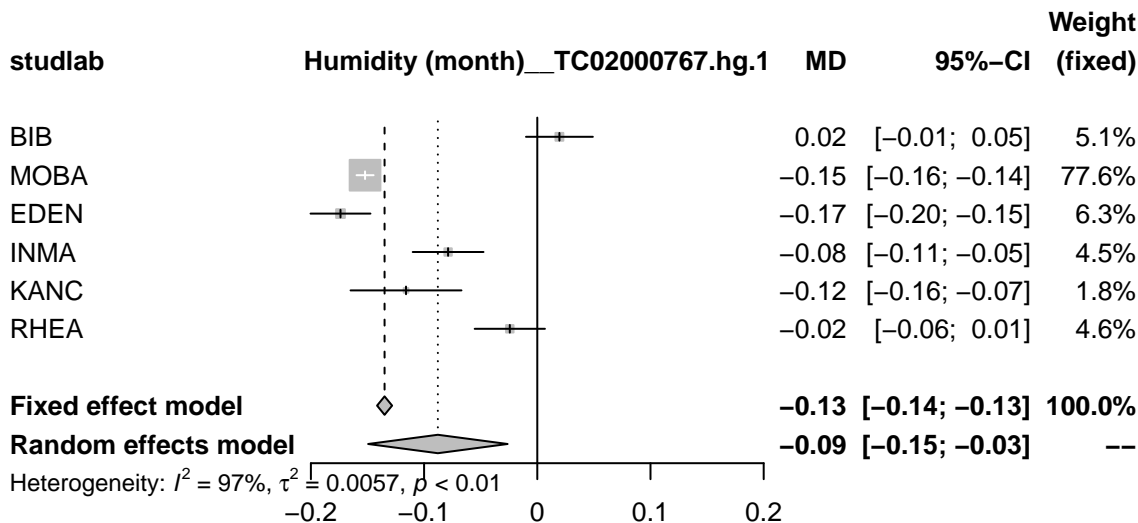

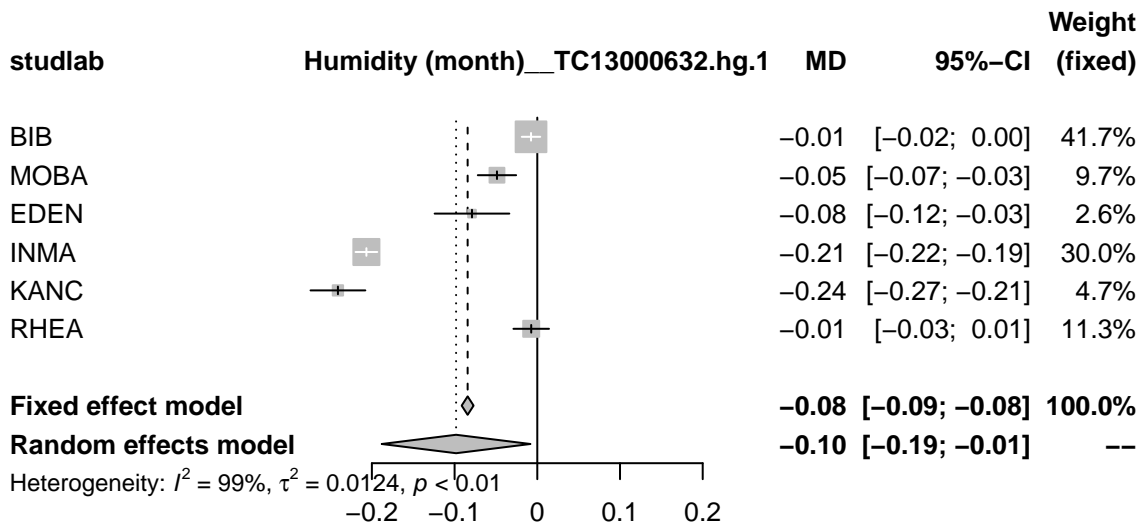

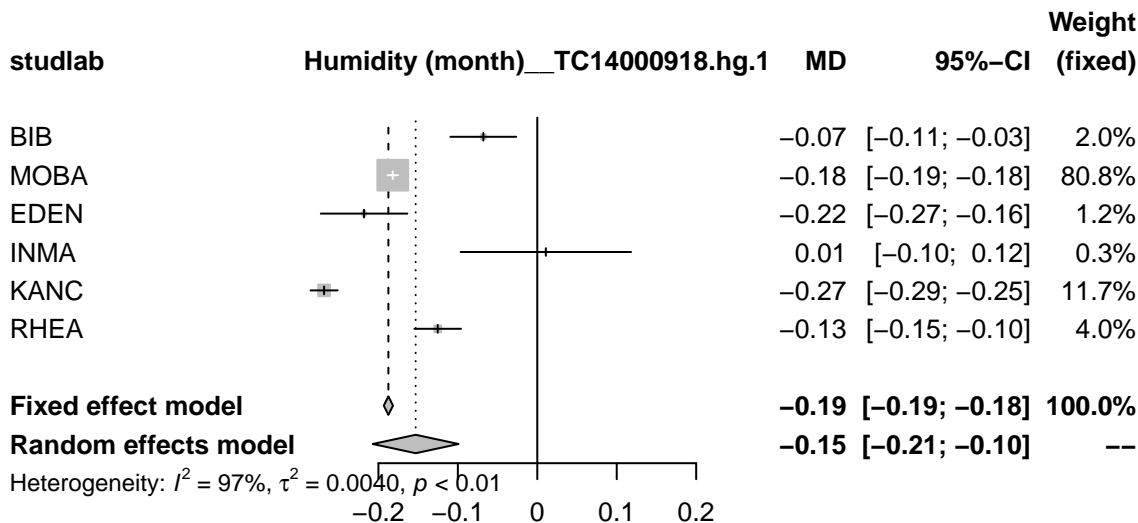

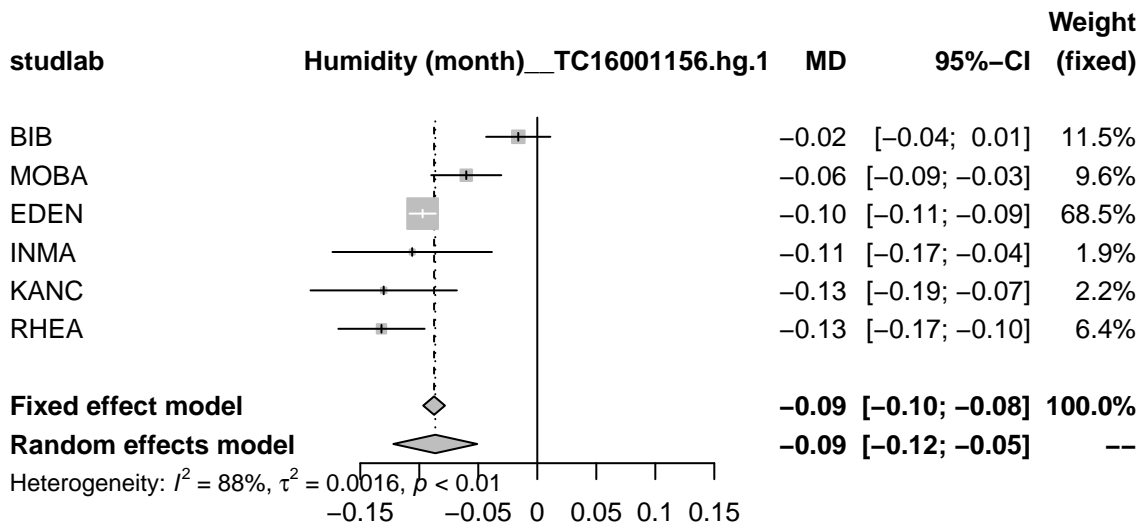

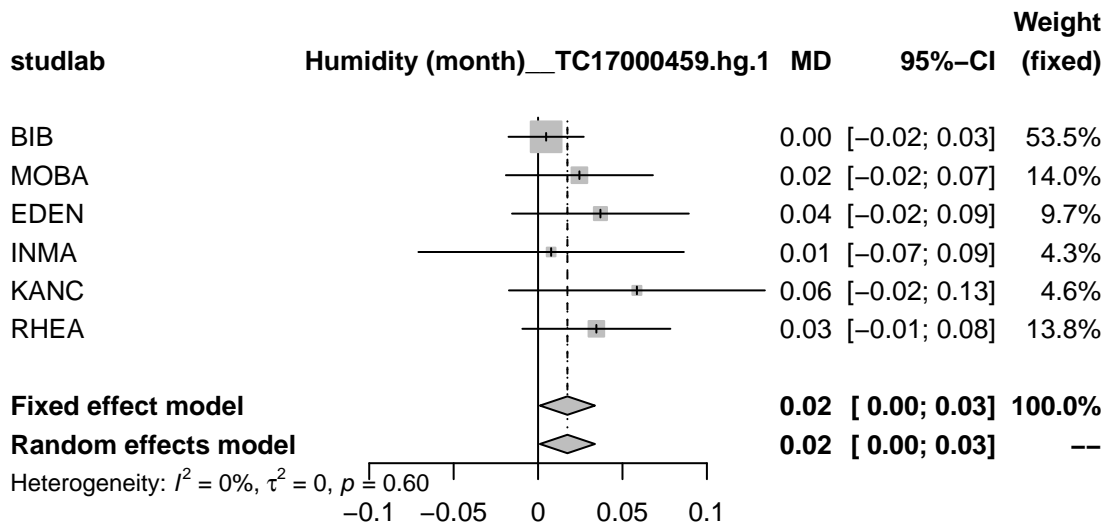

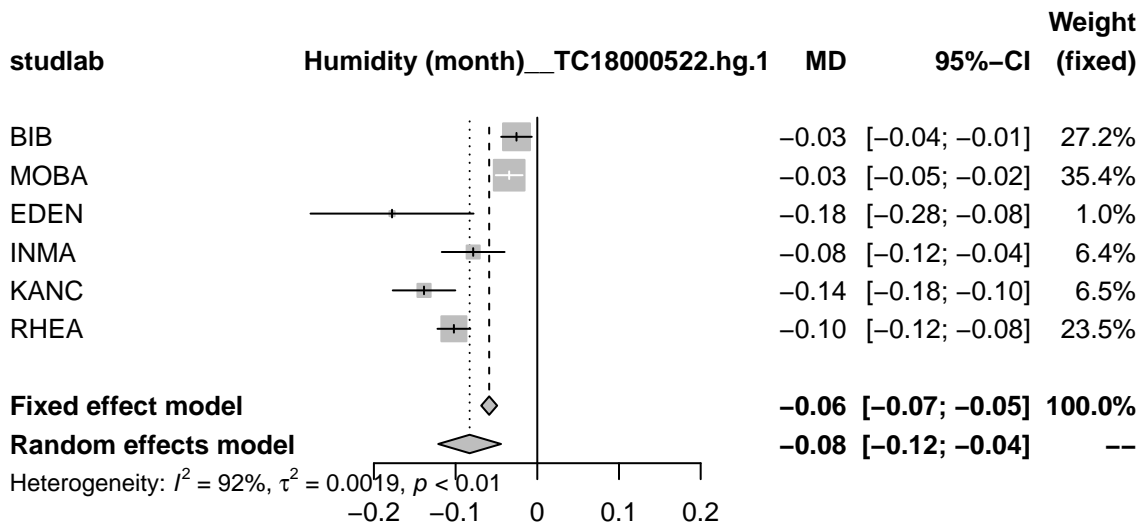

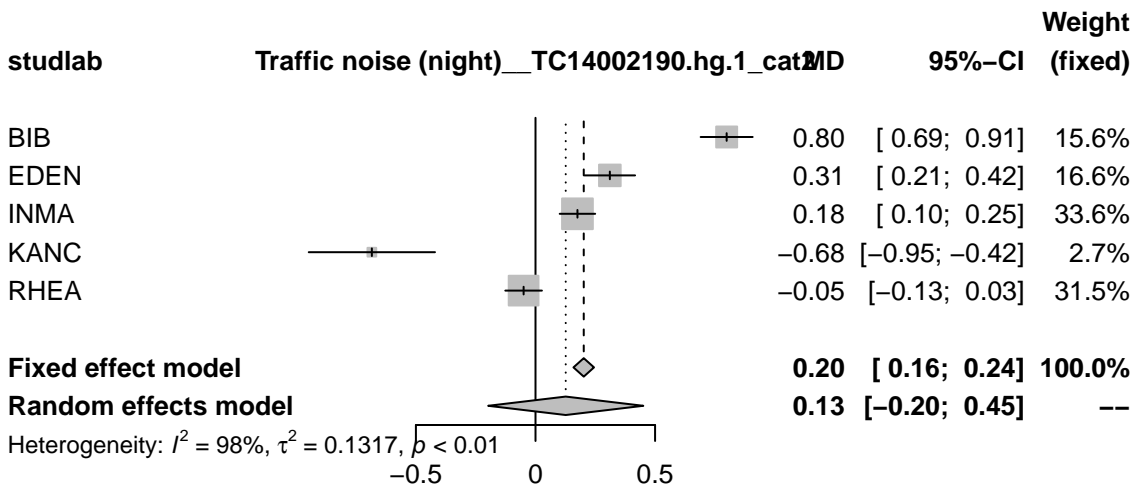

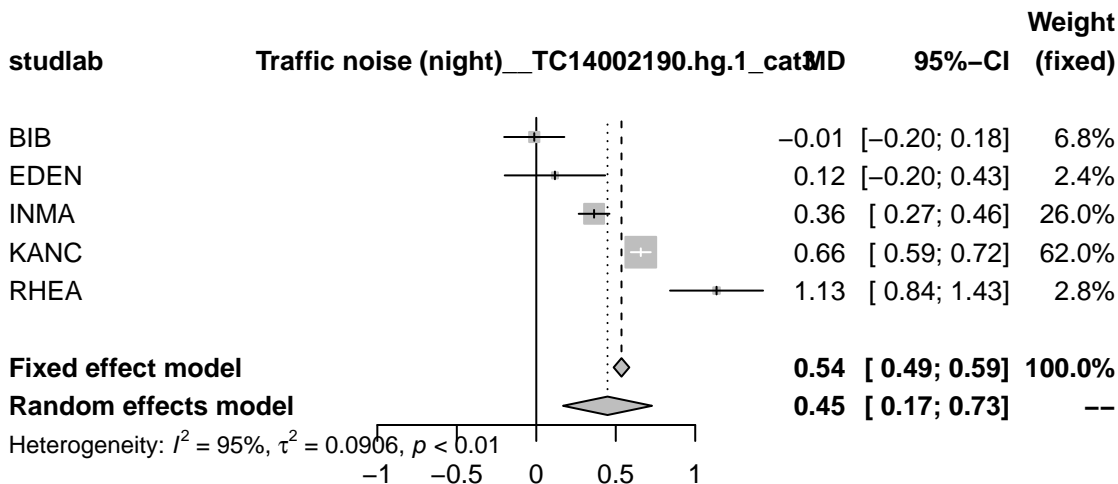

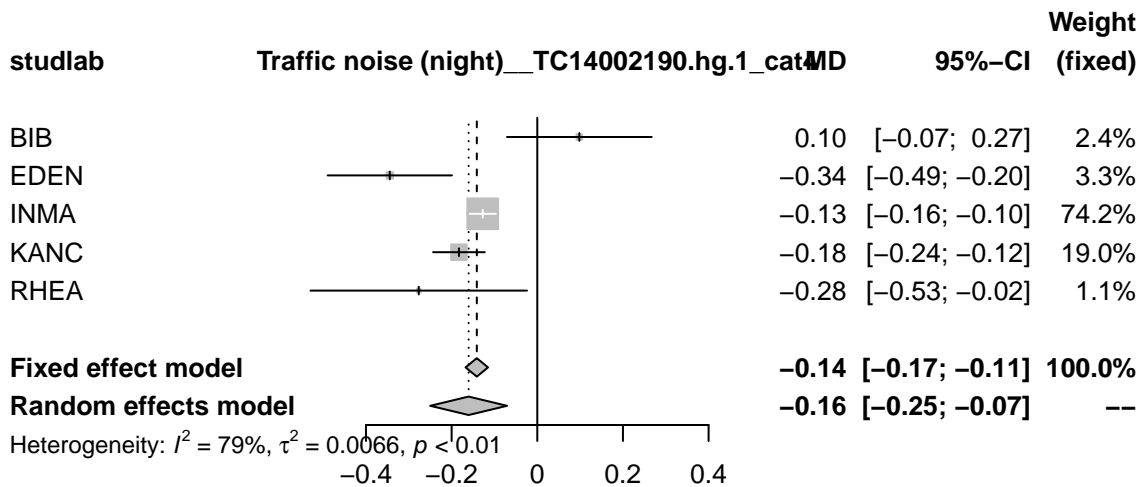

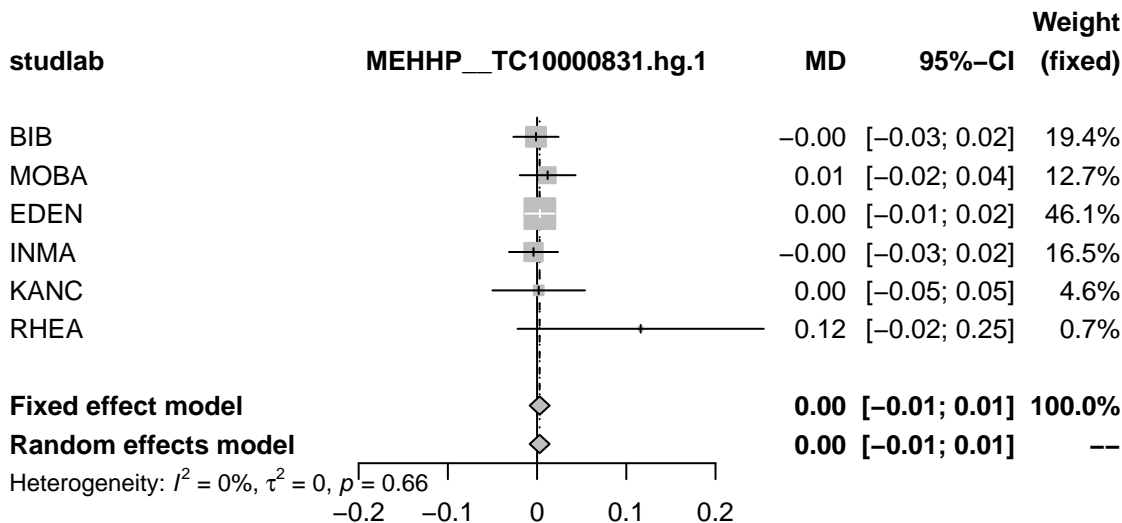

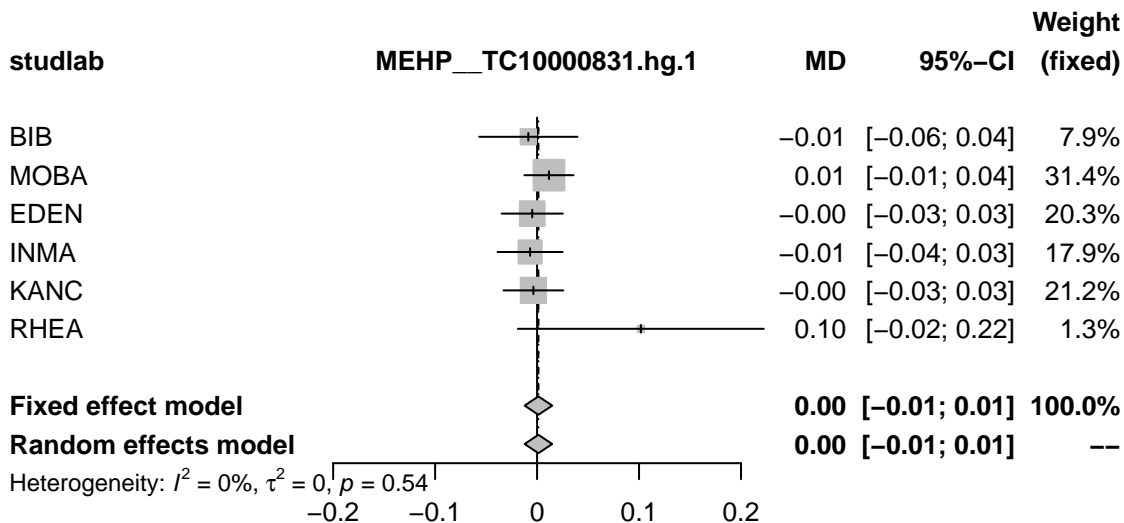

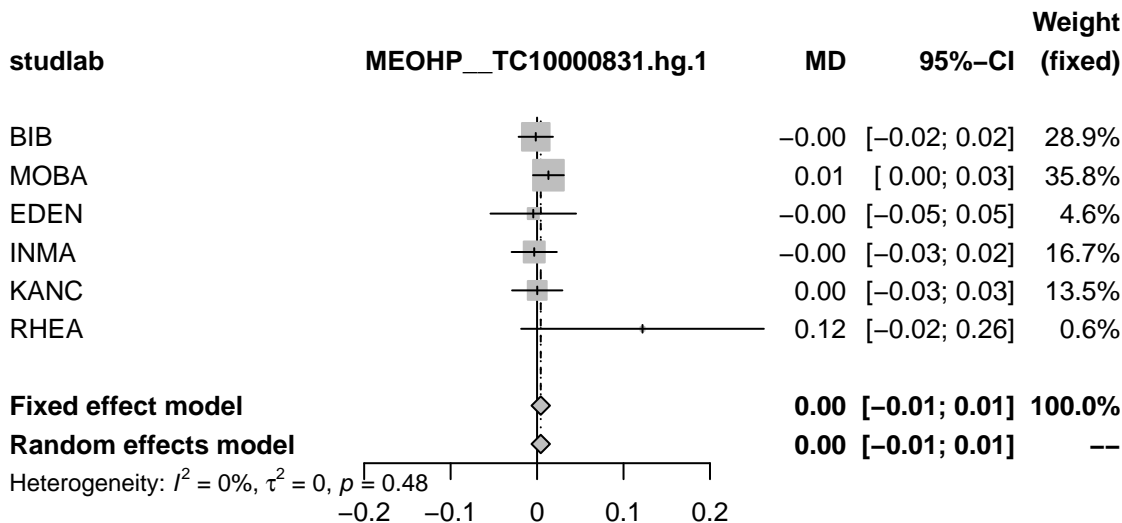

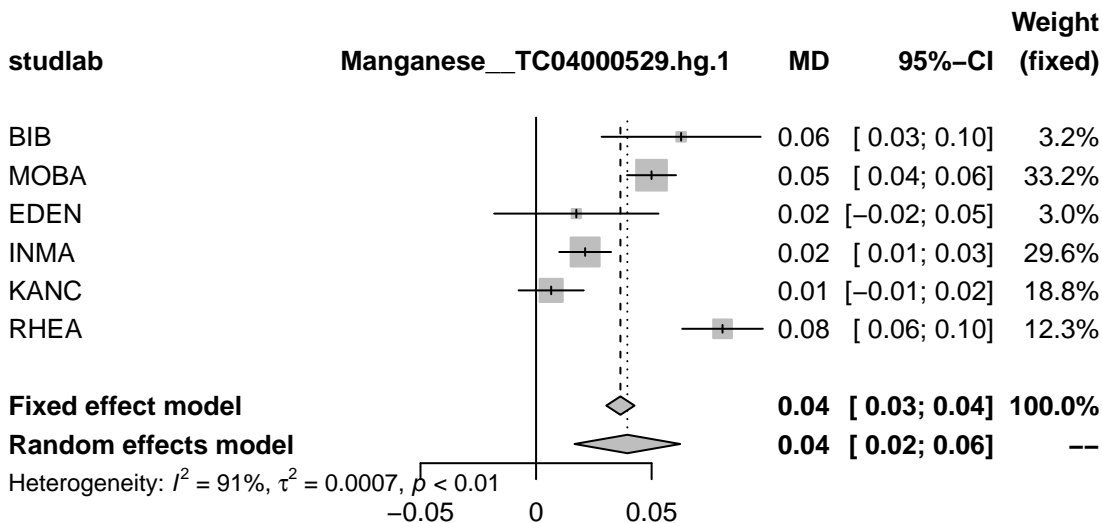

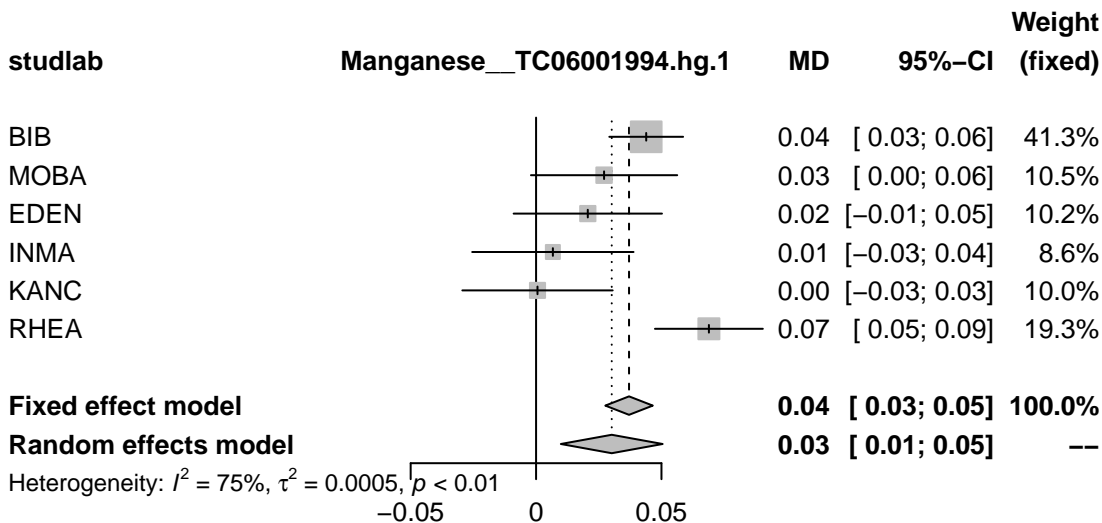

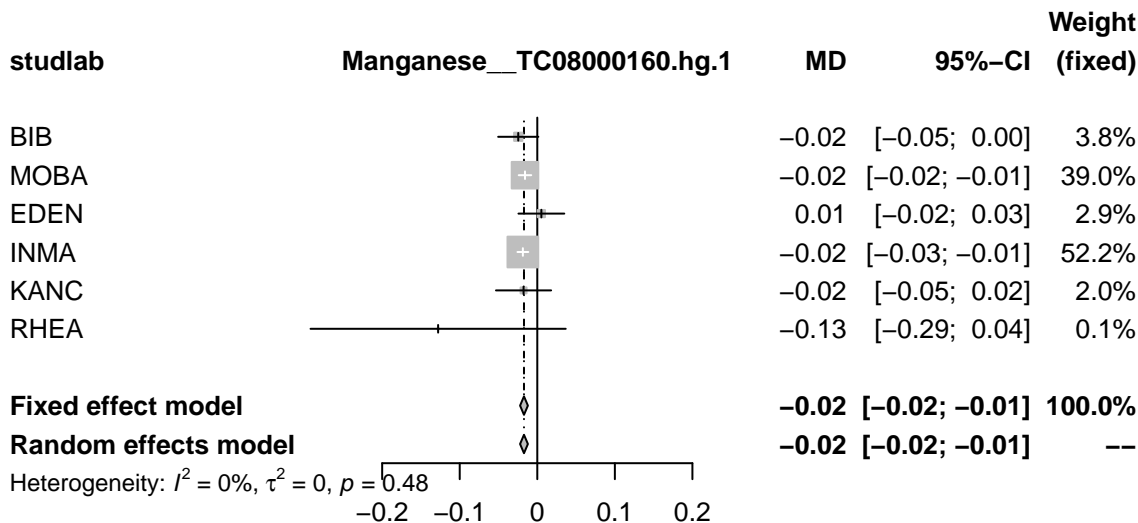

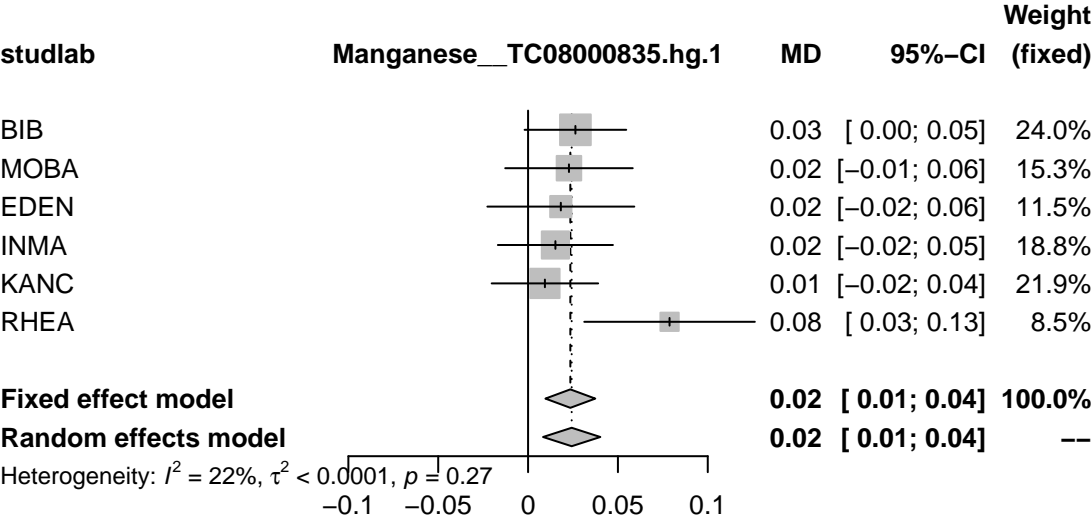

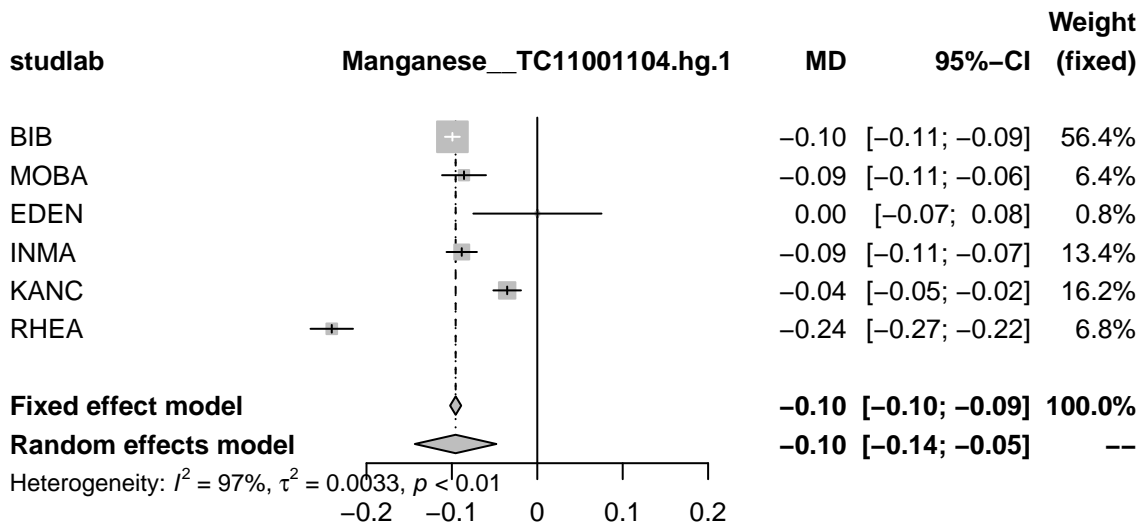

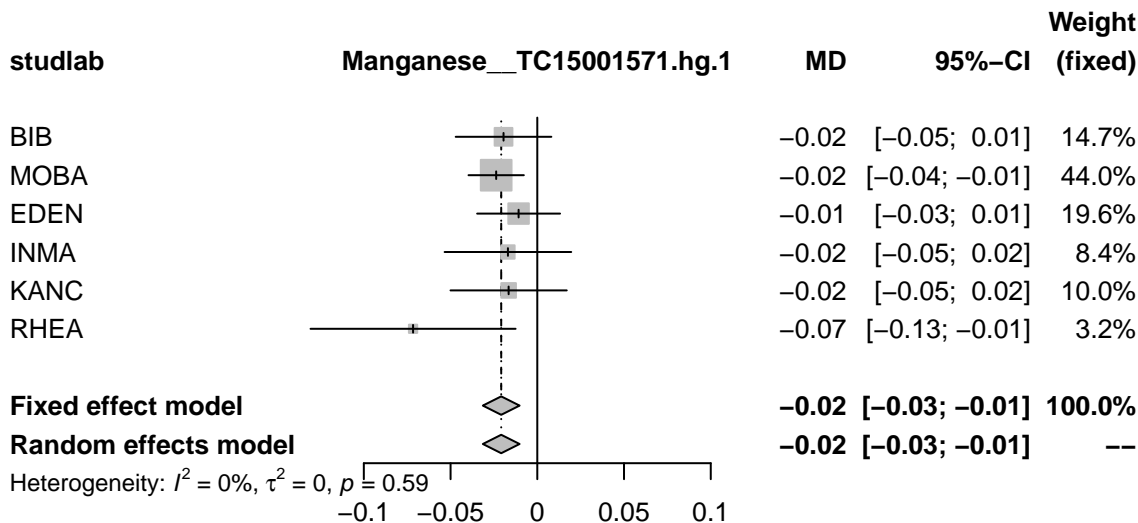

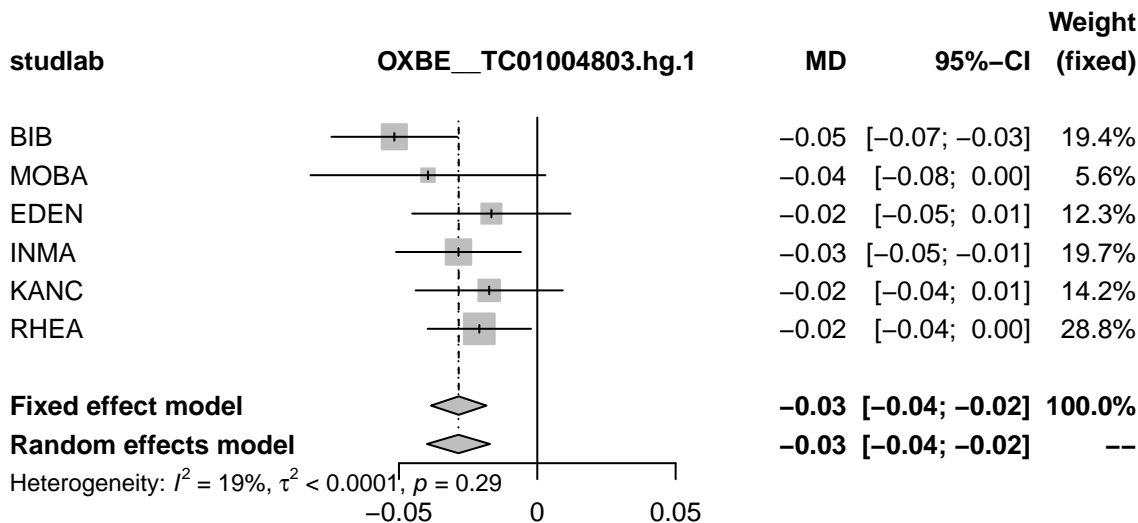

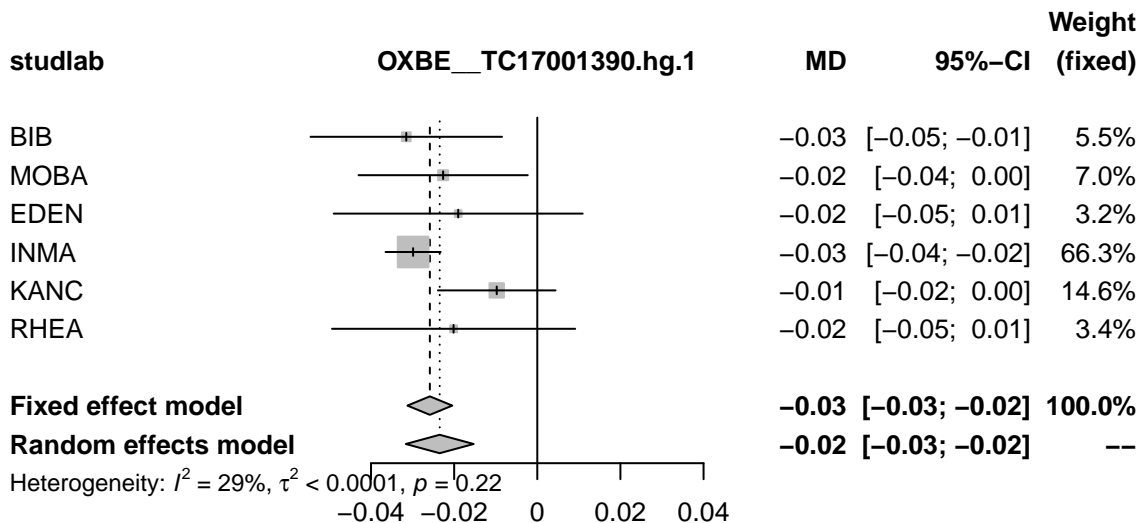

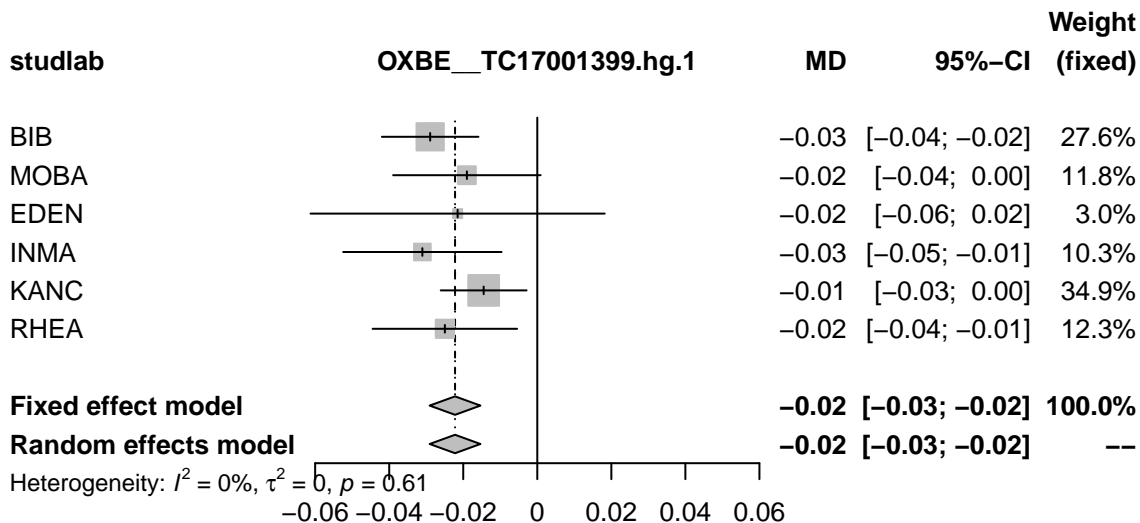

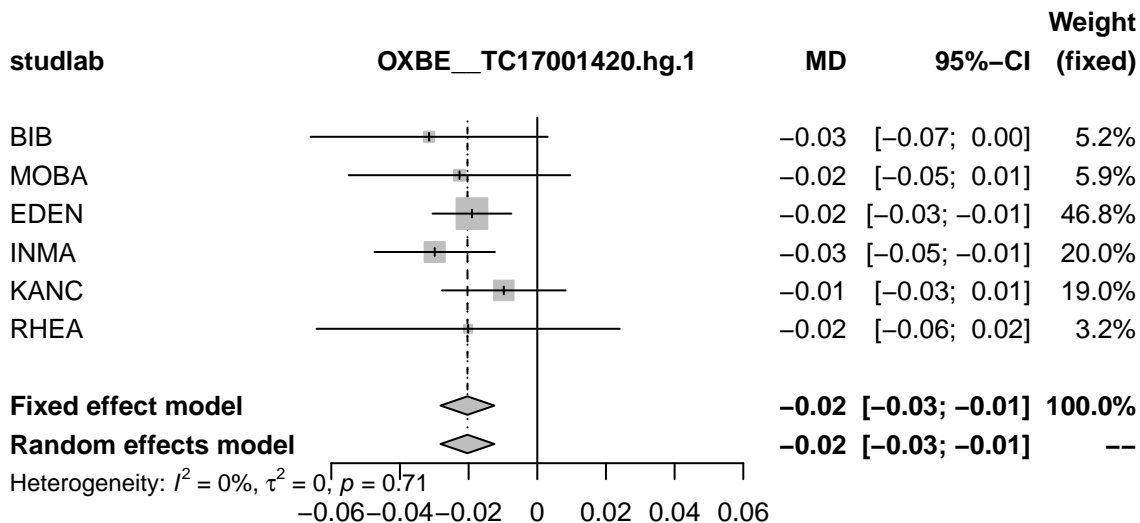

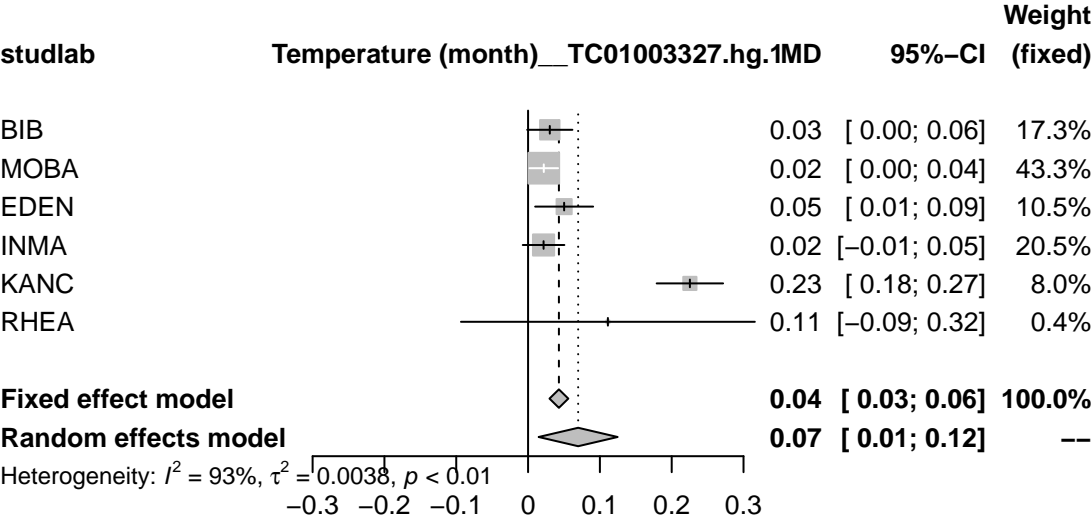

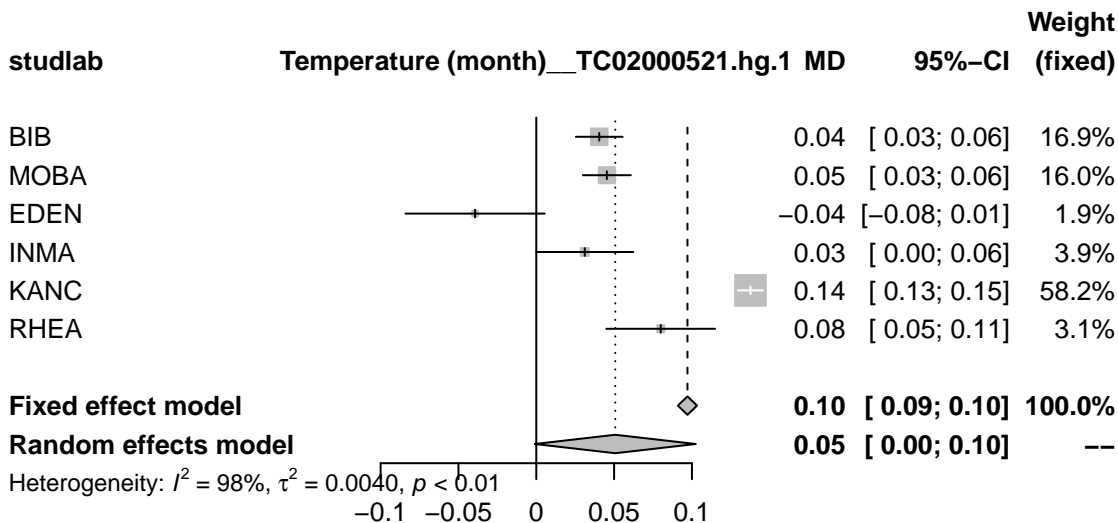

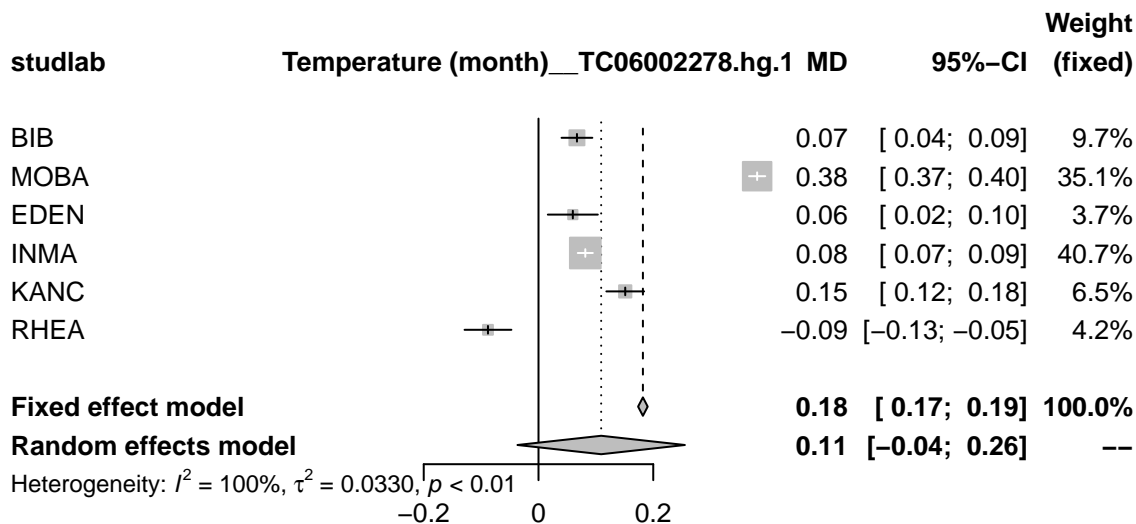

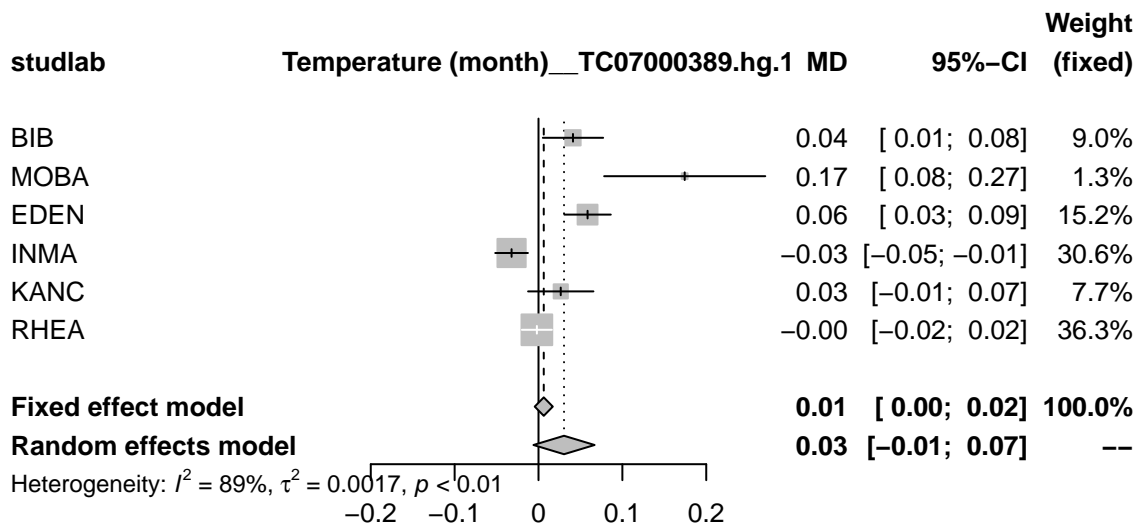

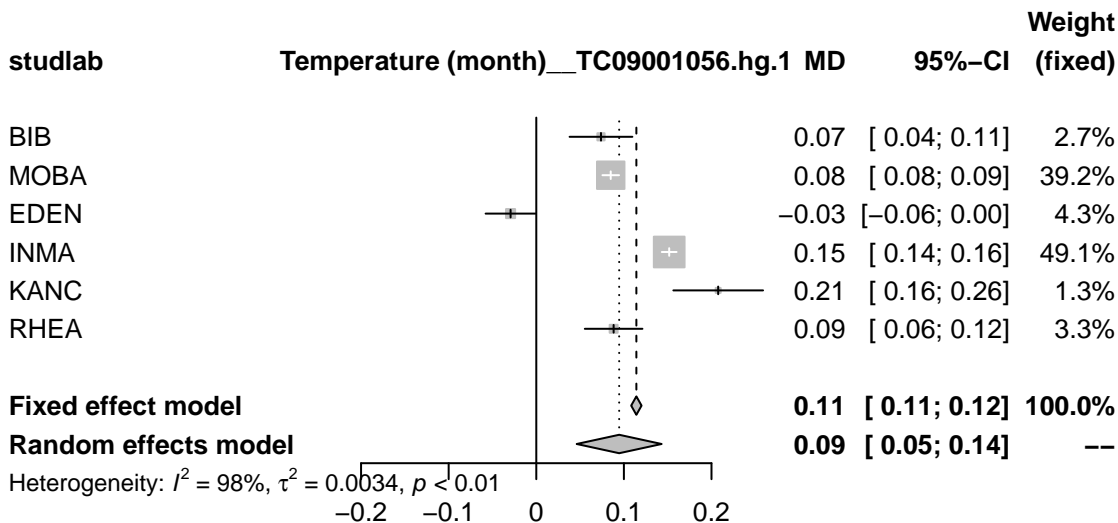

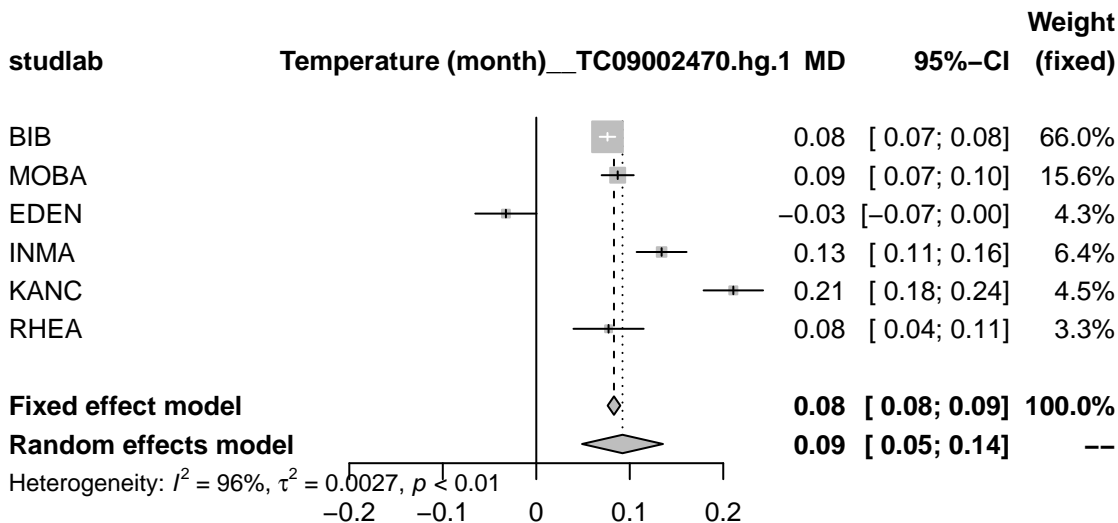

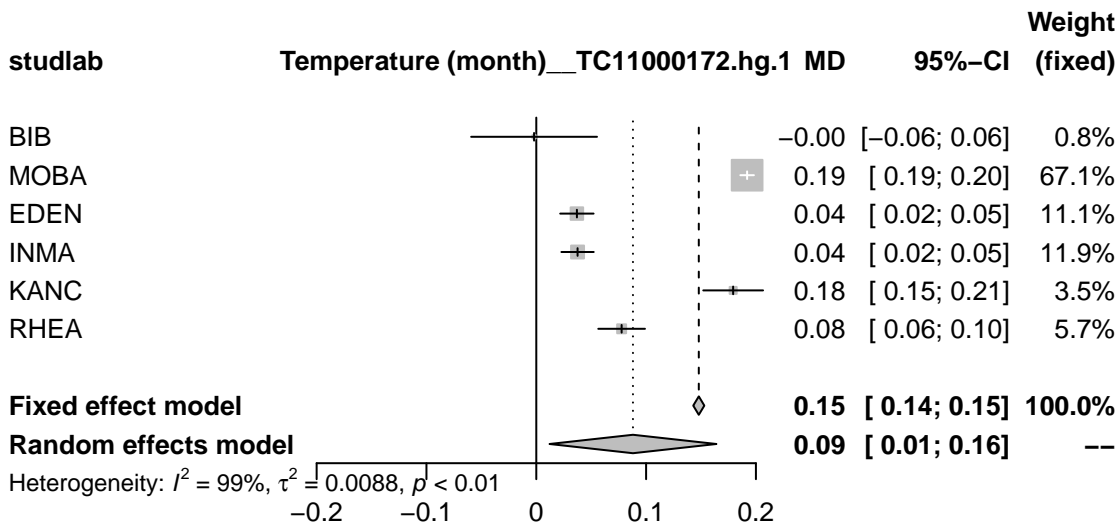

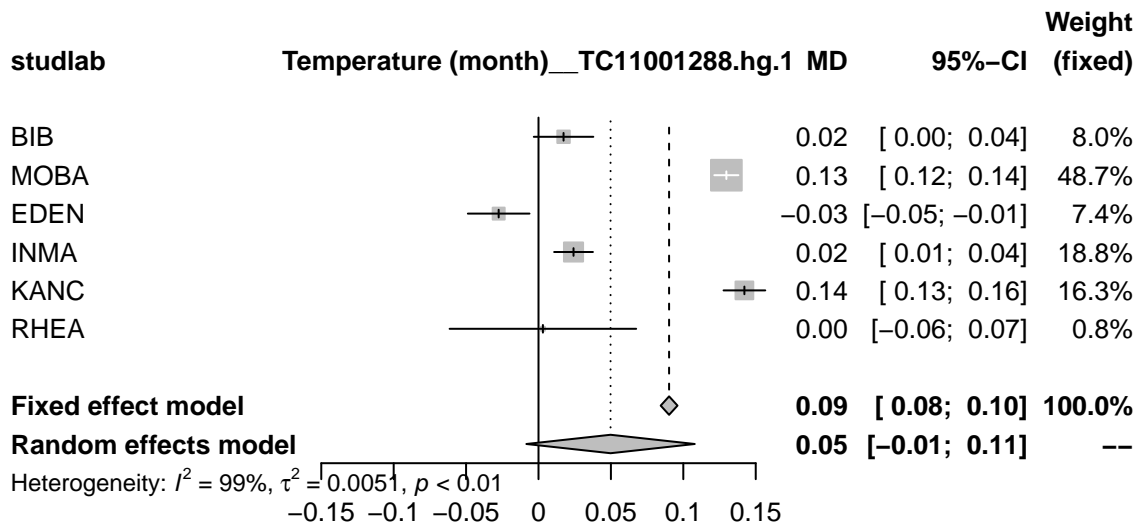

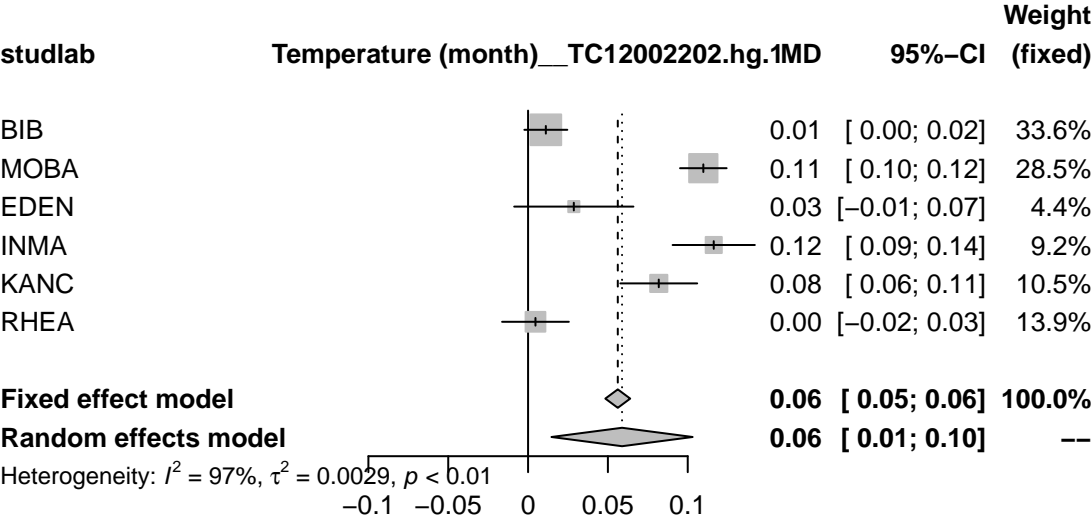

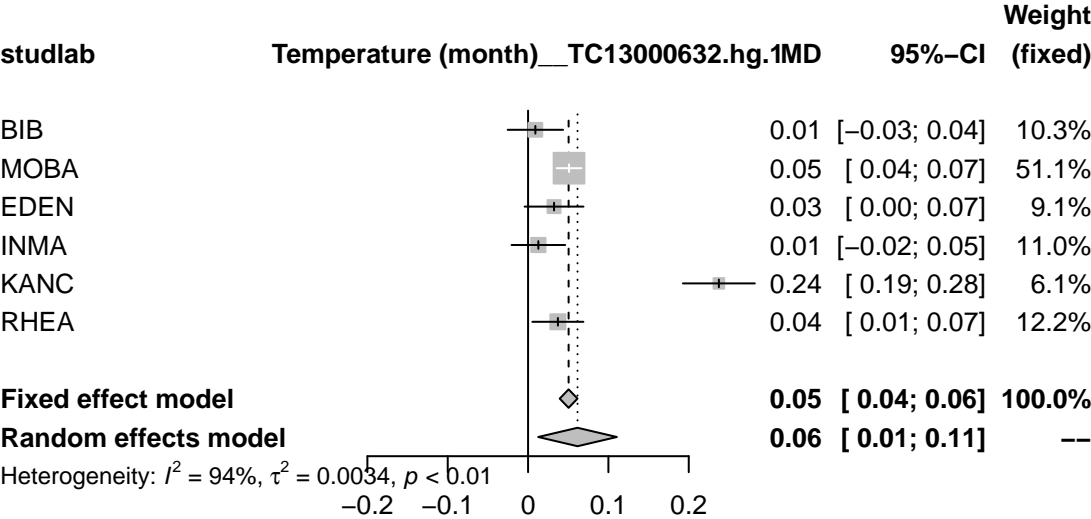

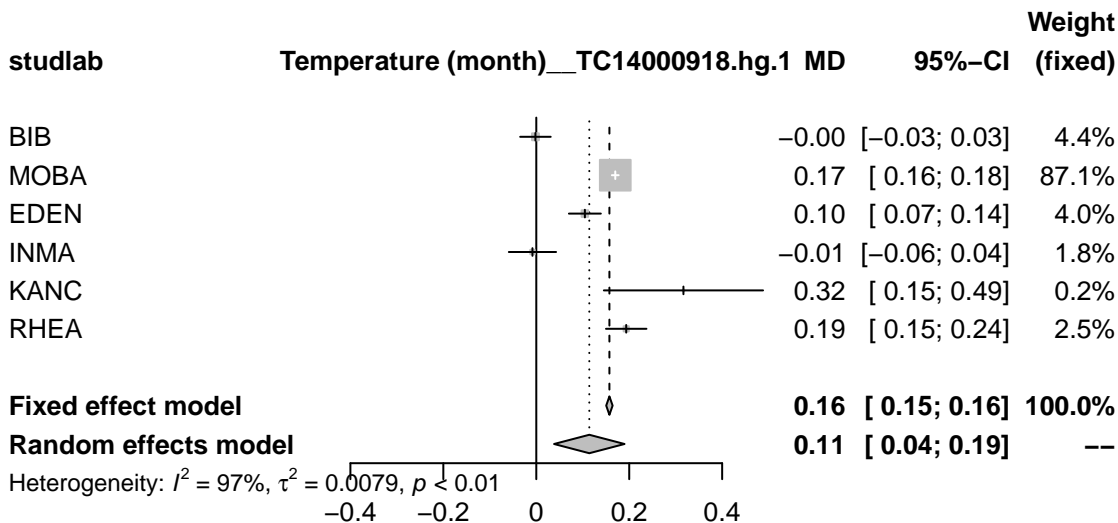

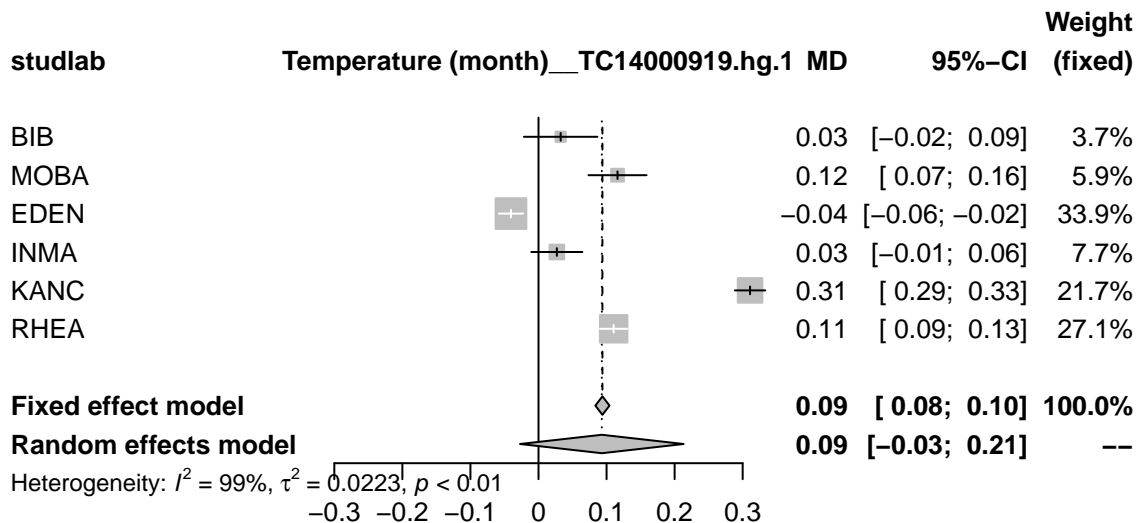

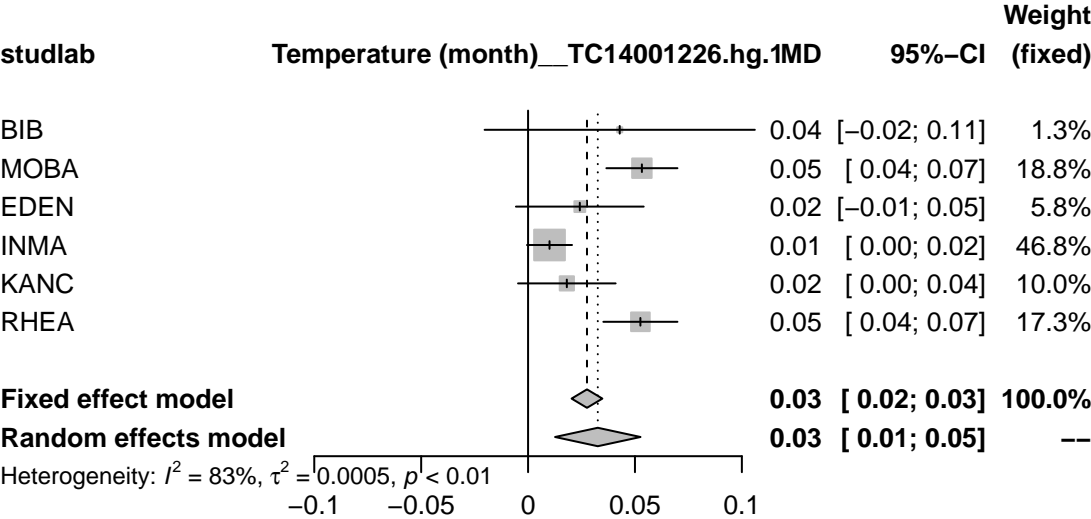

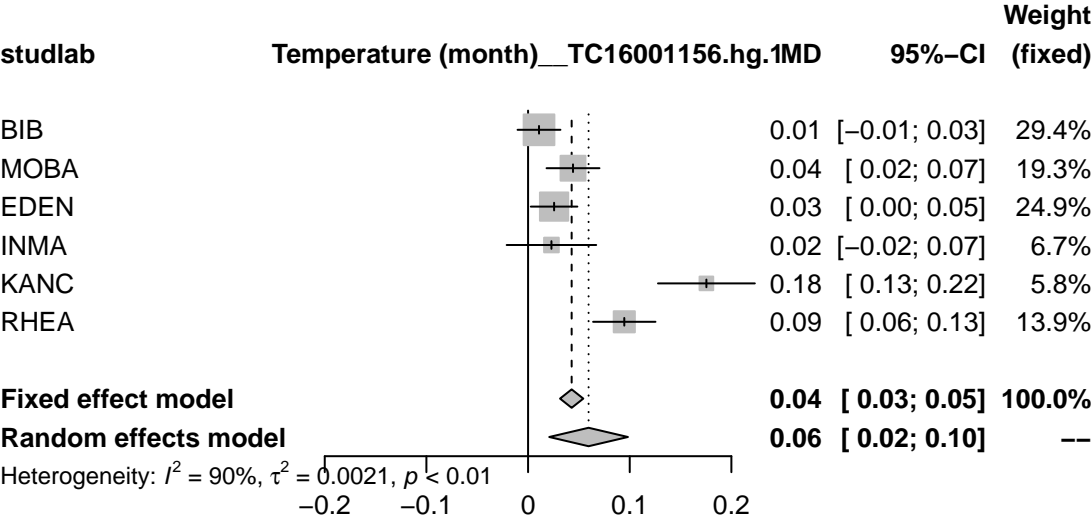

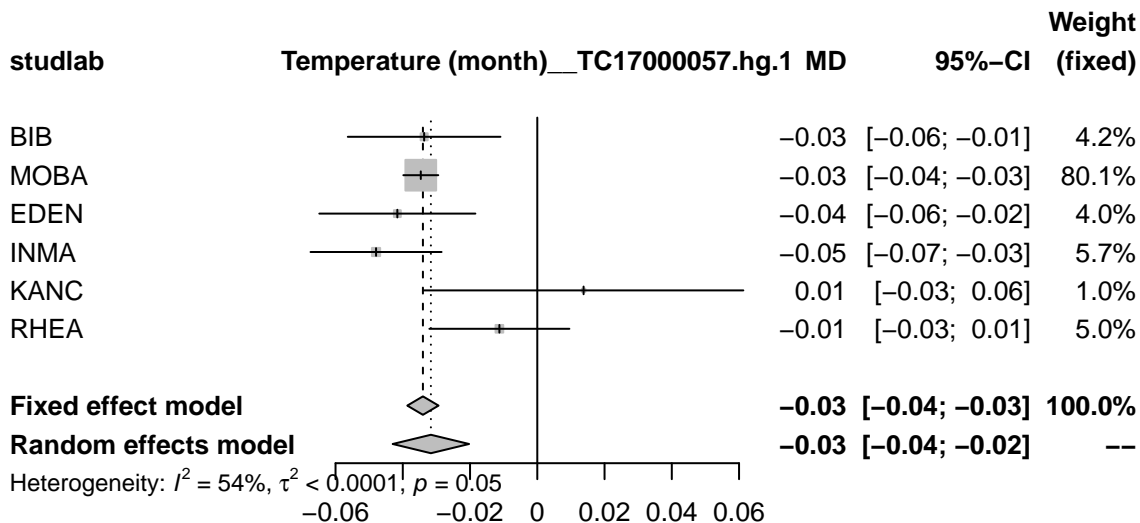

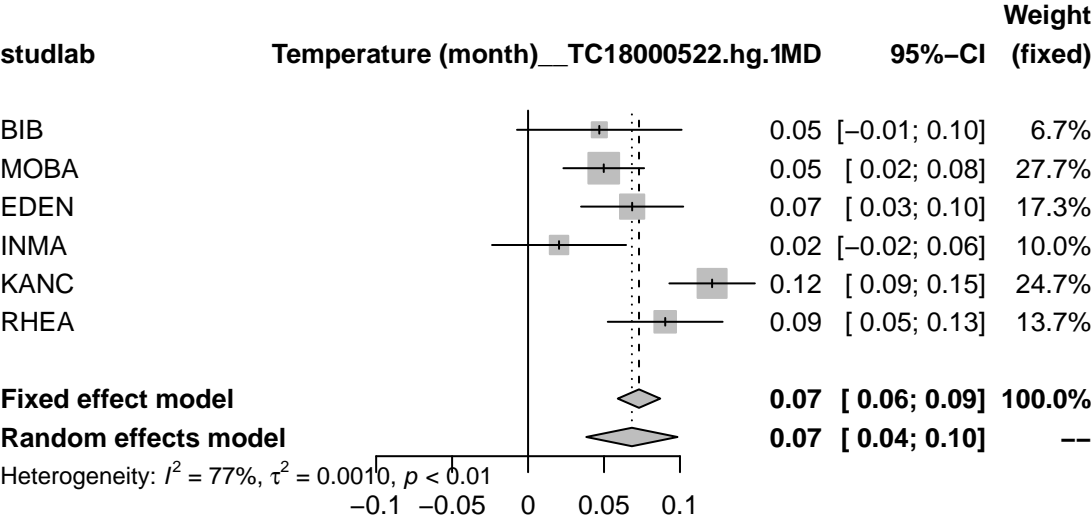

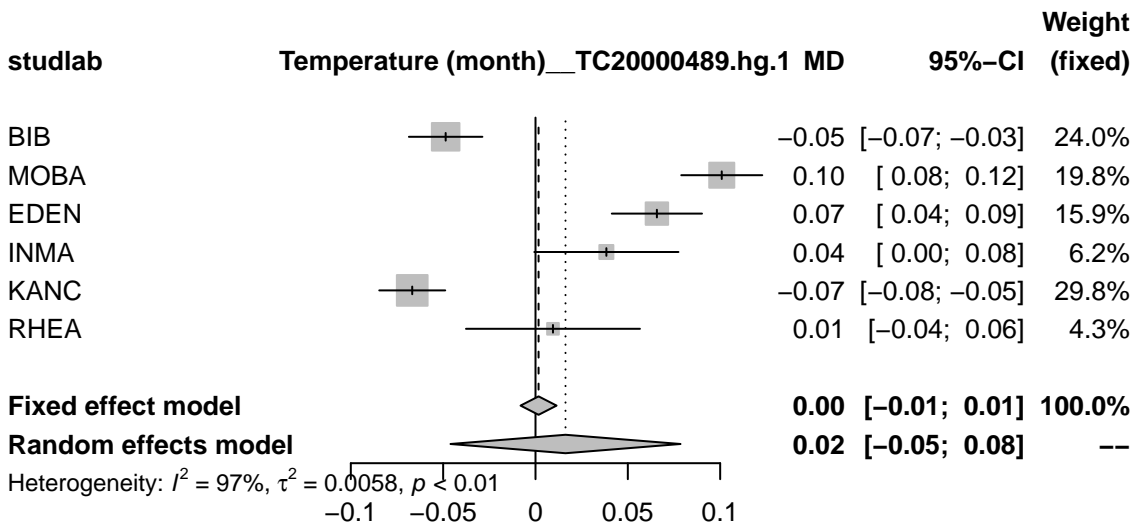

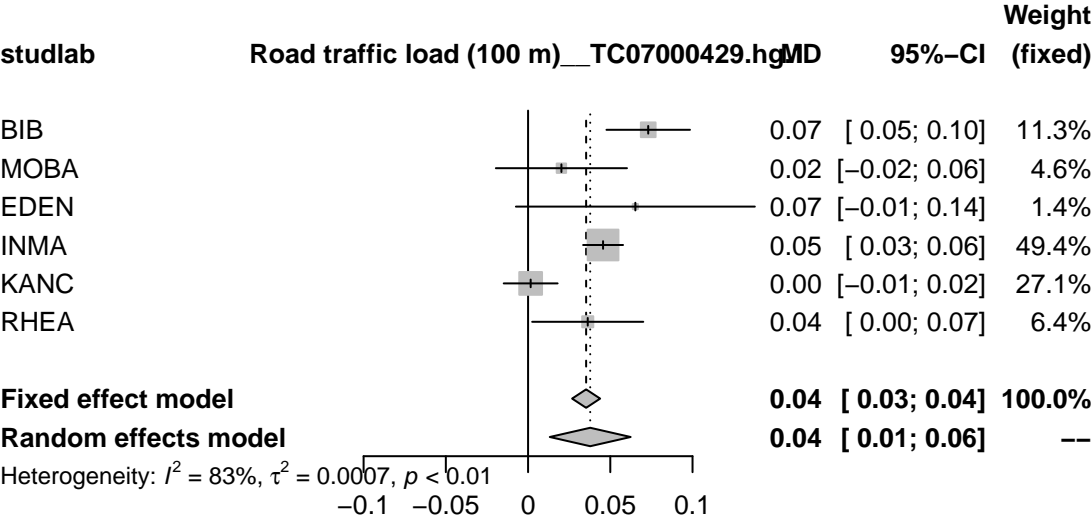

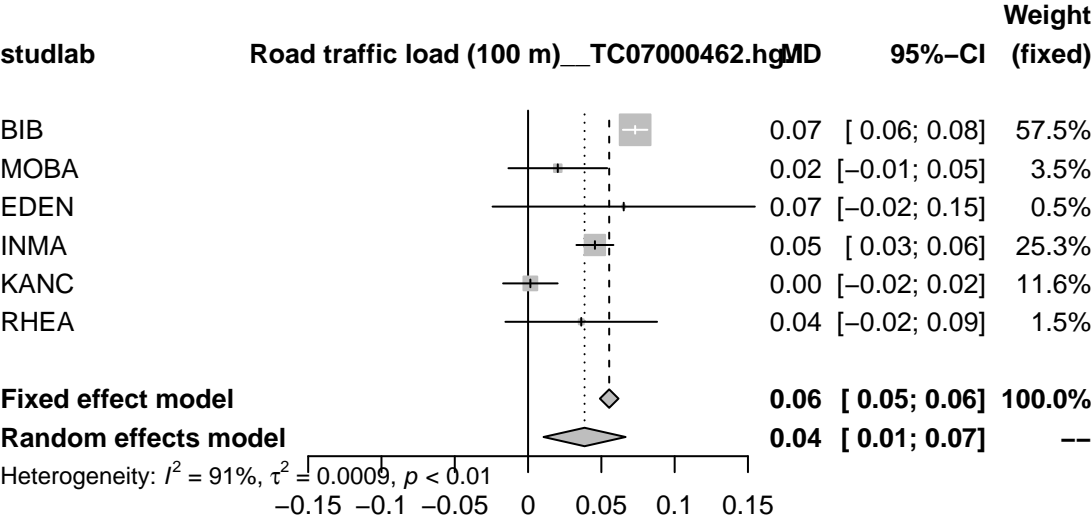

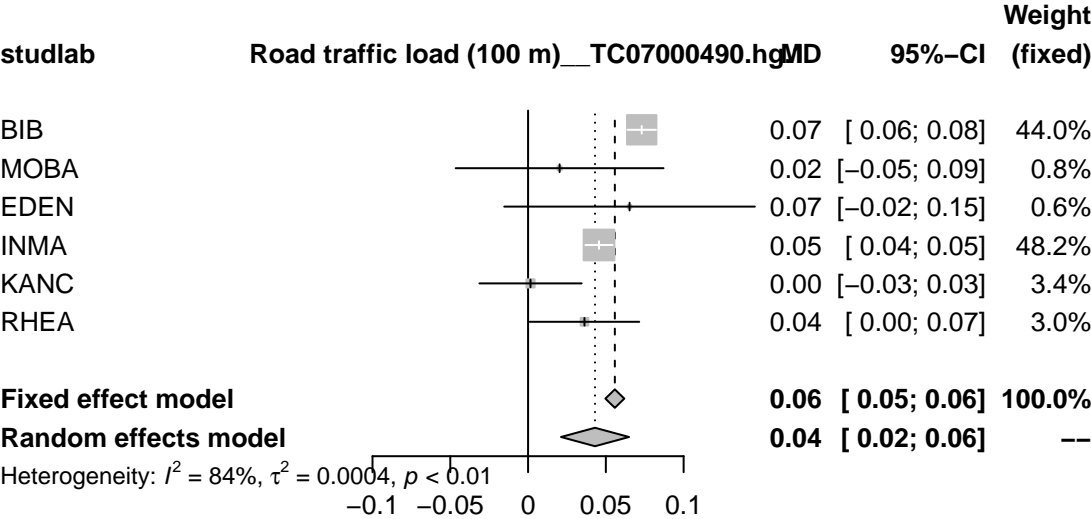

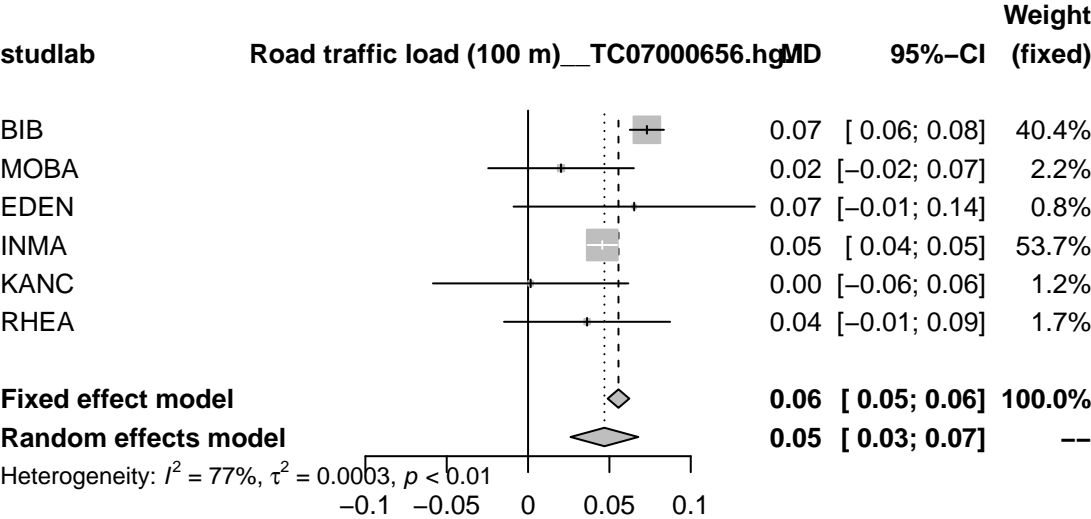

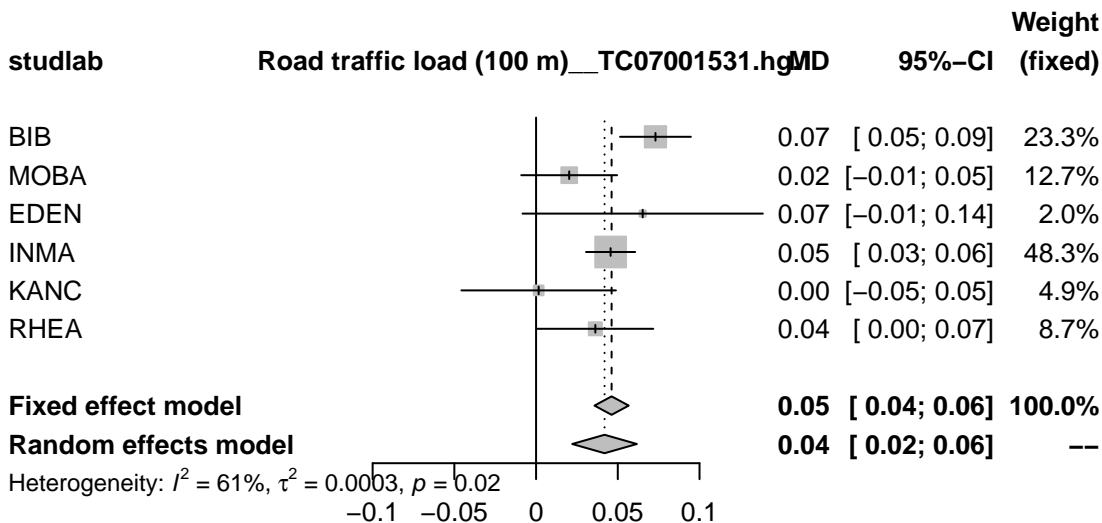

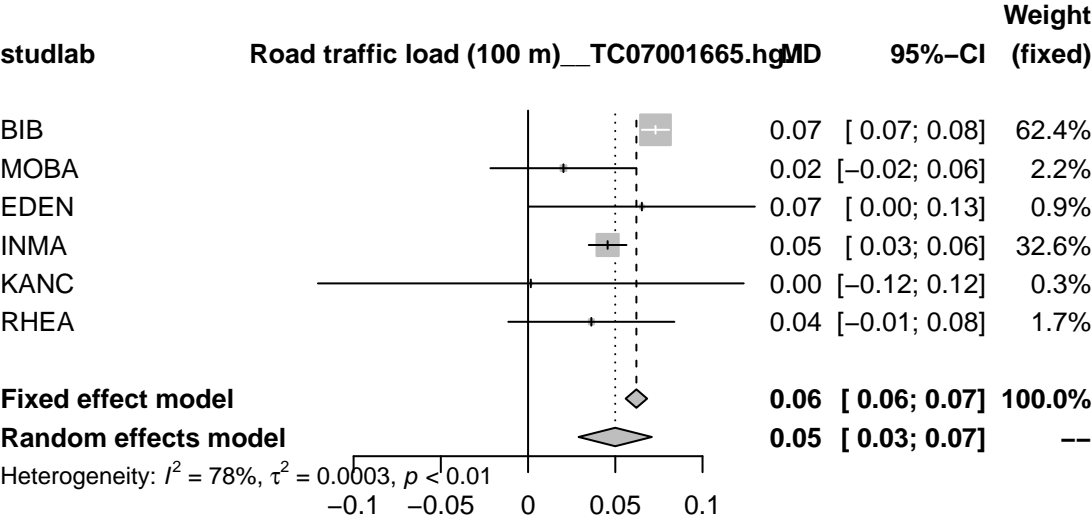

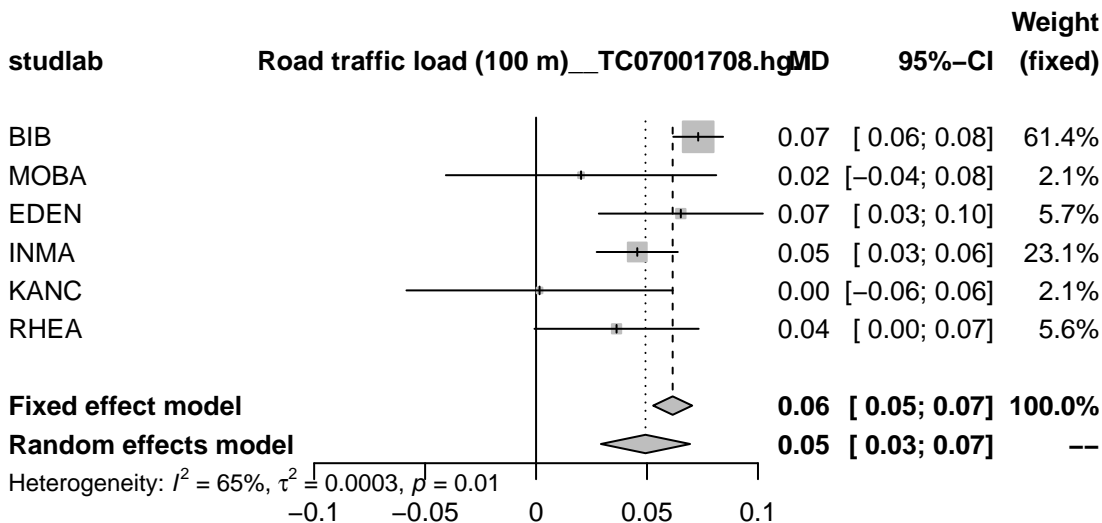

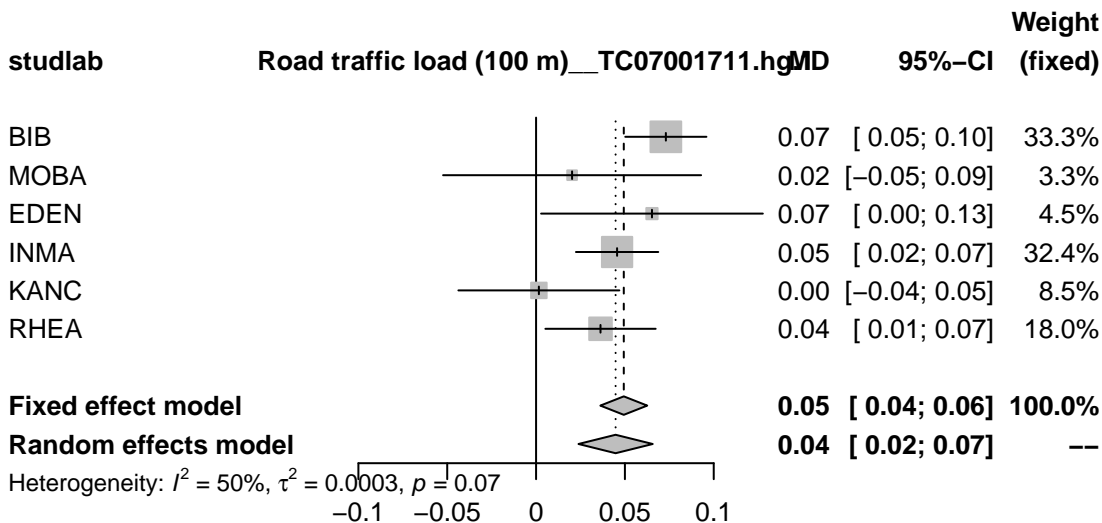

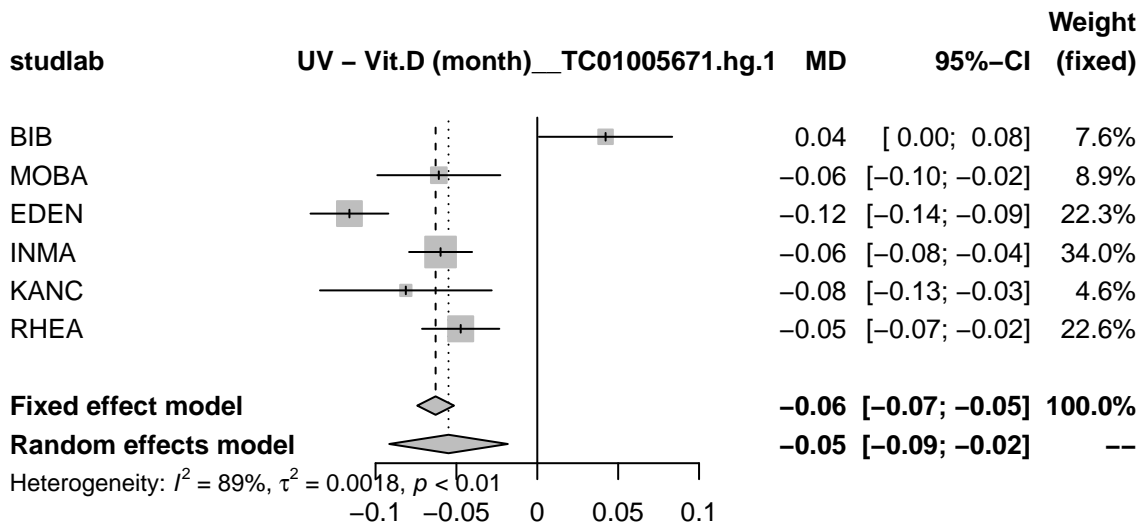

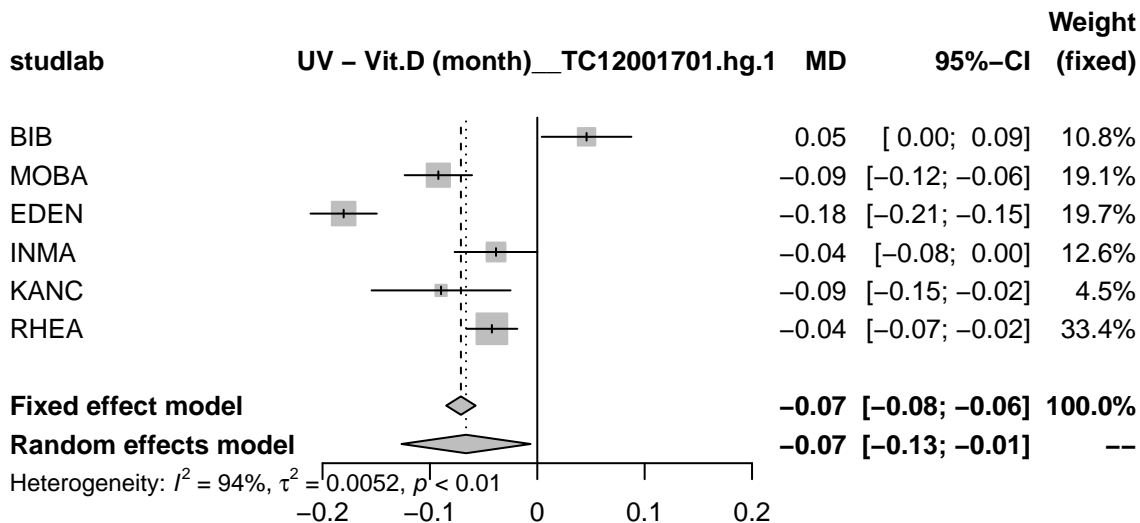

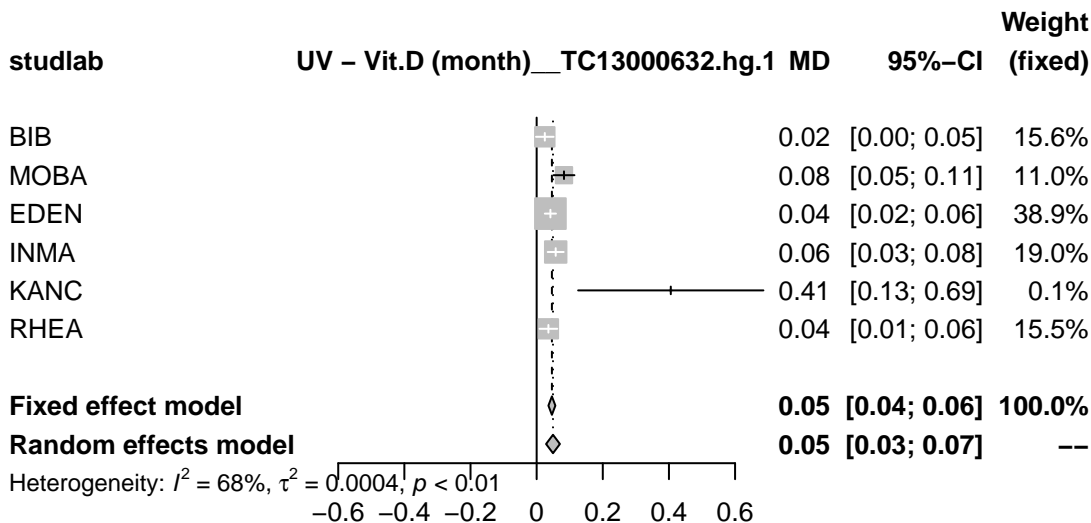

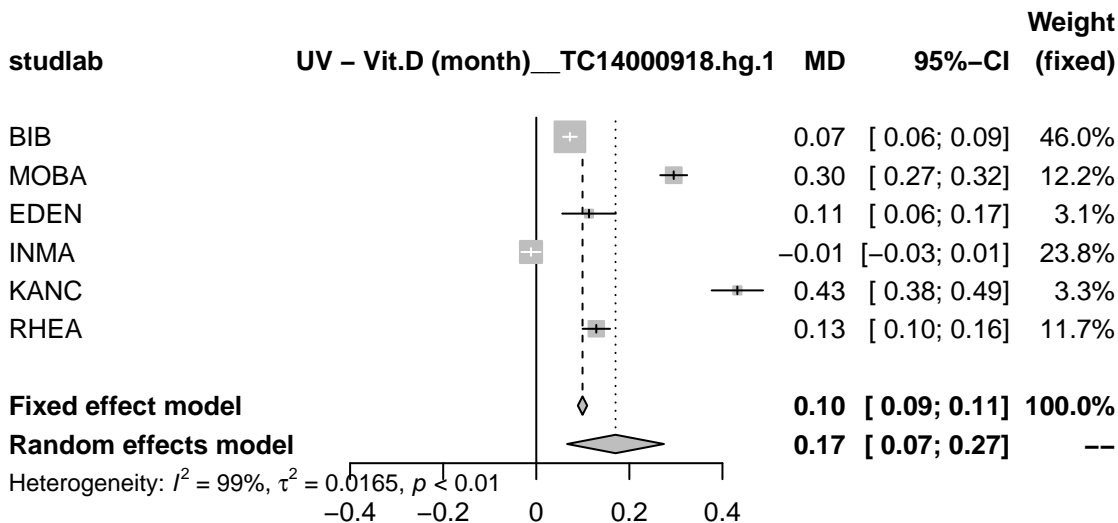

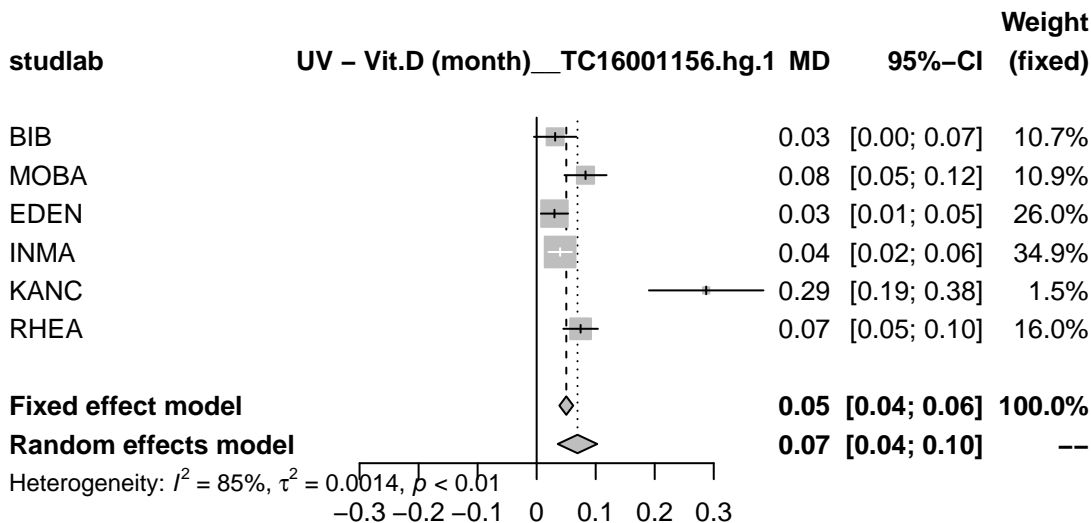

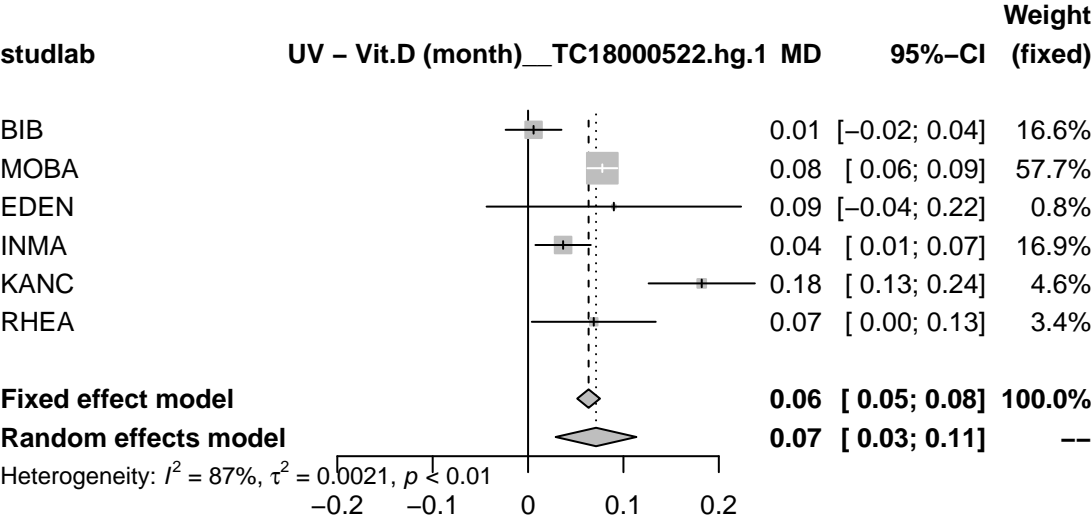

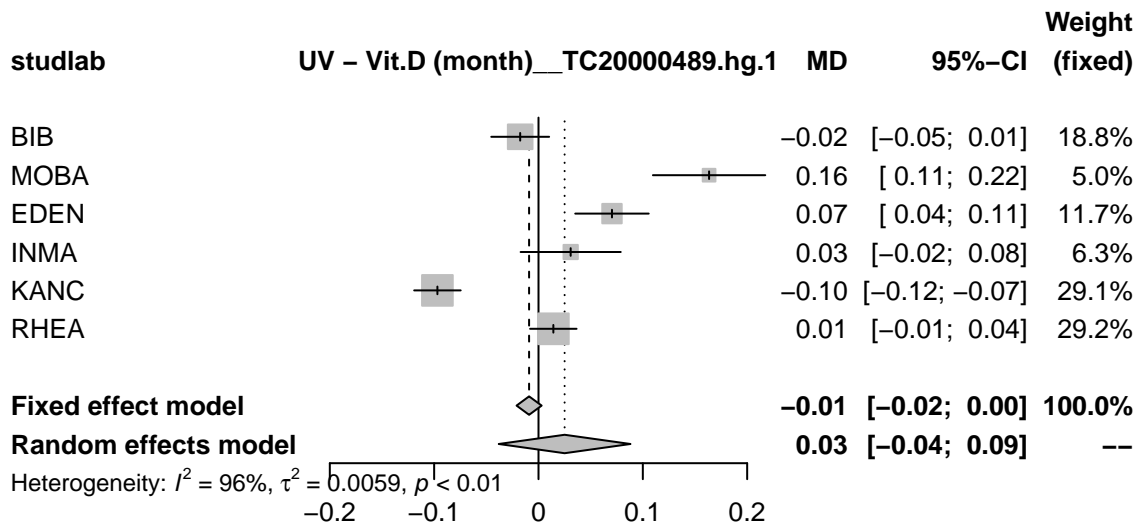

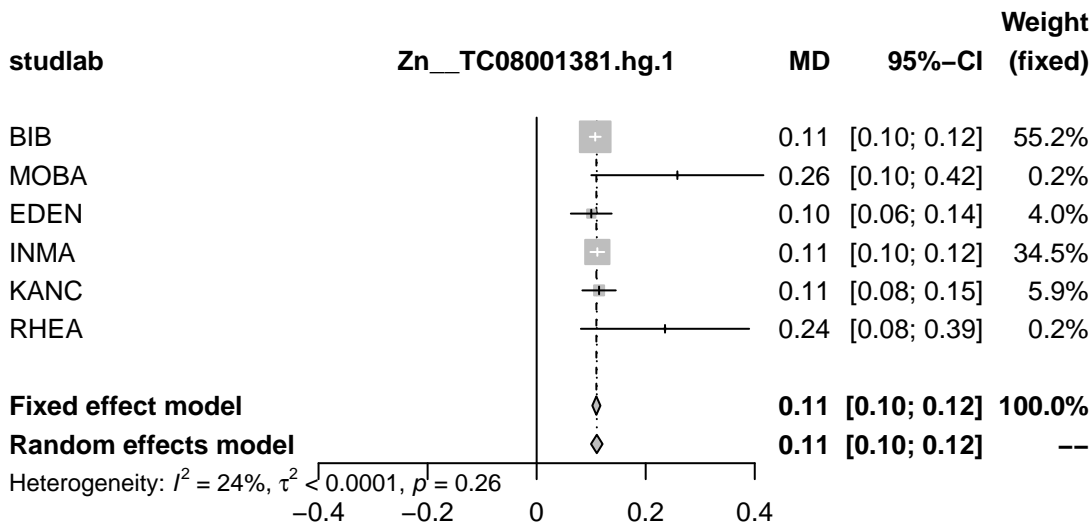

Supplement: Supplementary file 14 — Supplementary Dataset 11 [file 41467_2022_34422_MOESM14_ESM.zip › HELIX_ExpOmics_FigS2_Forestplots/HELIX_ExpOmics_FigS2H_trans_post.pdf]

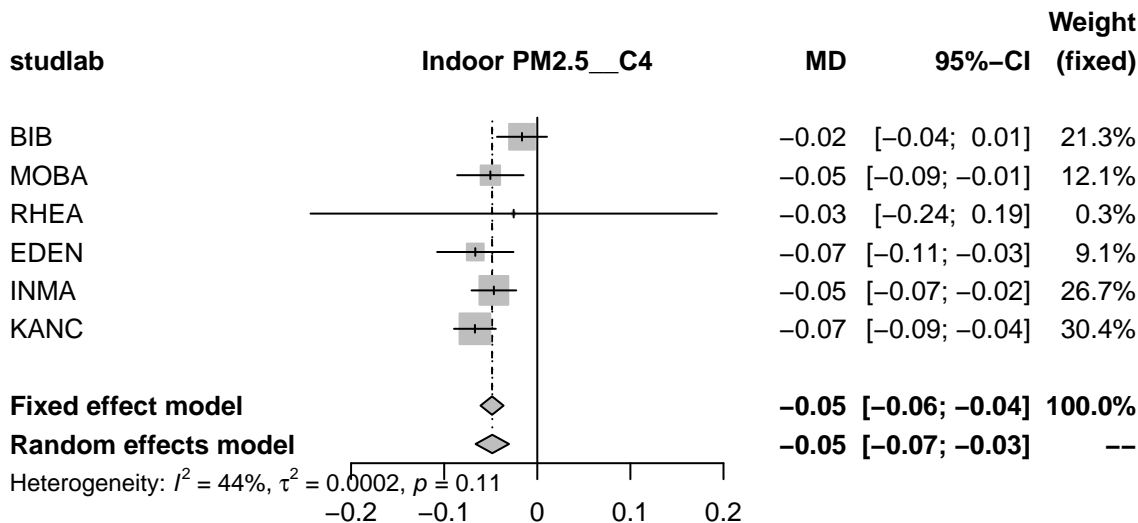

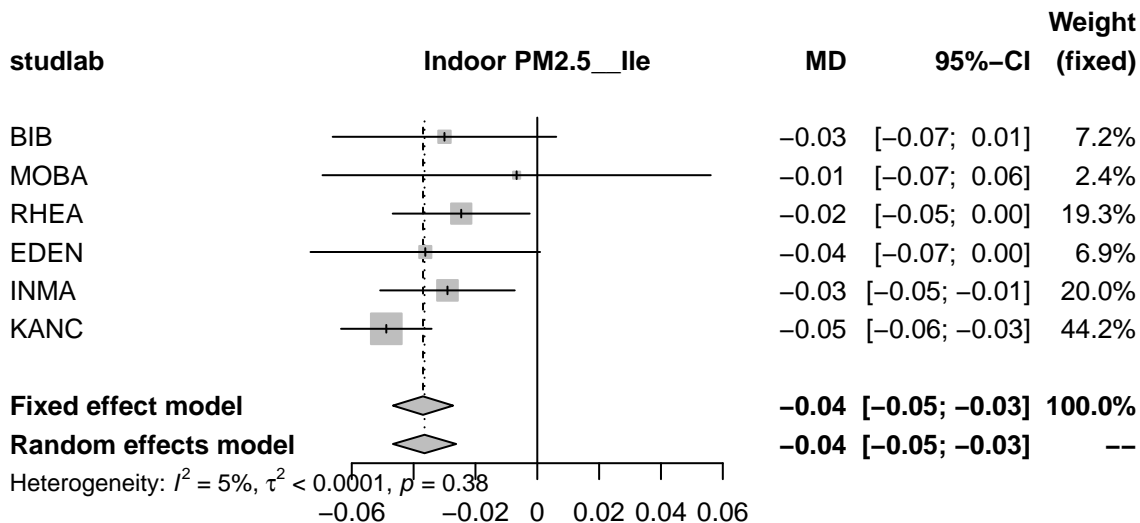

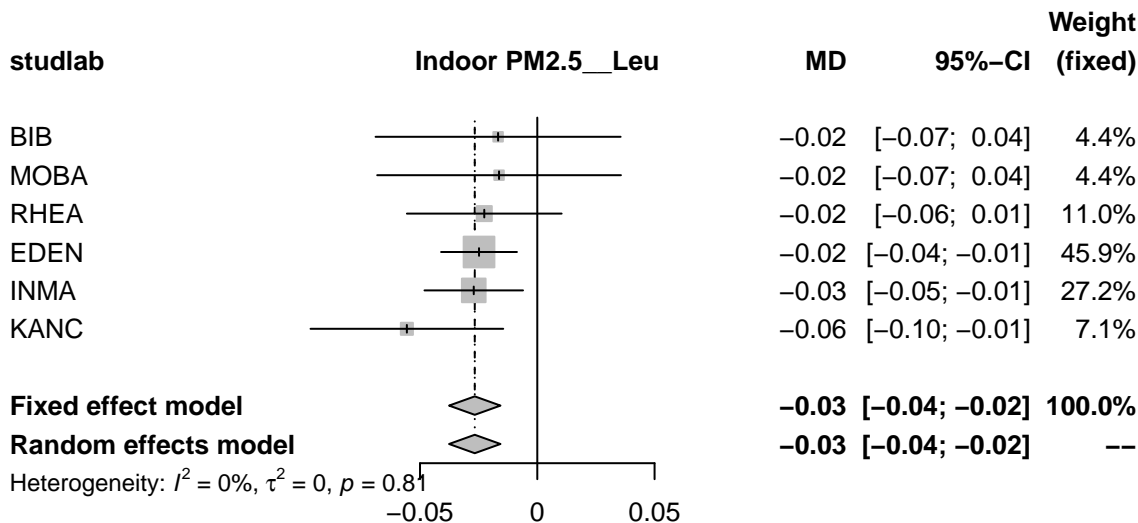

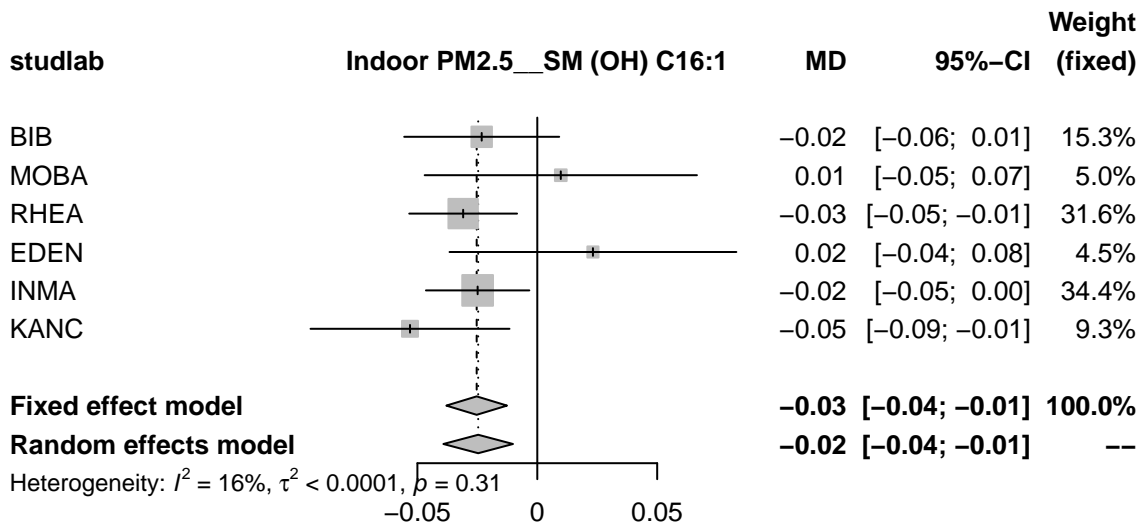

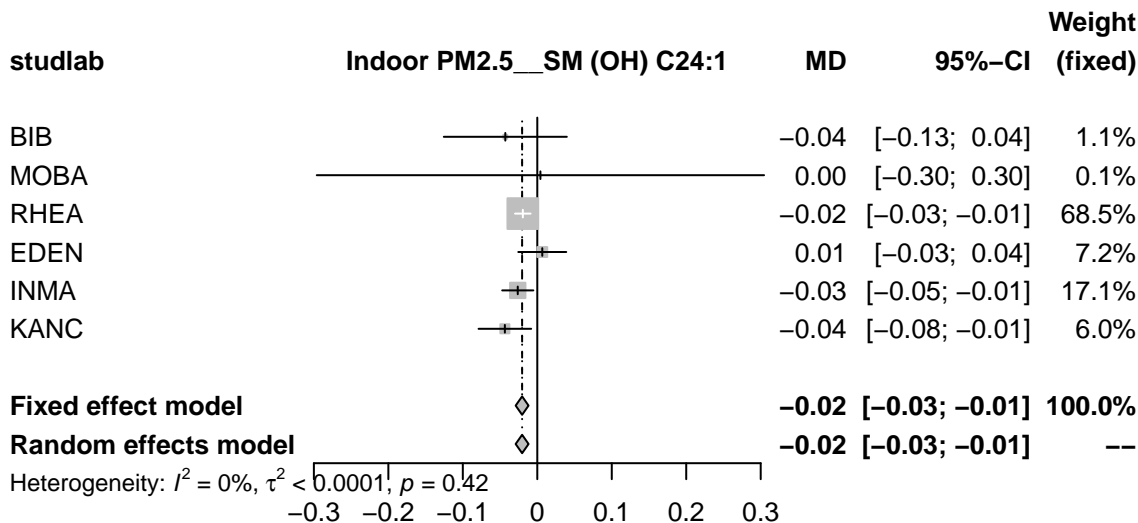

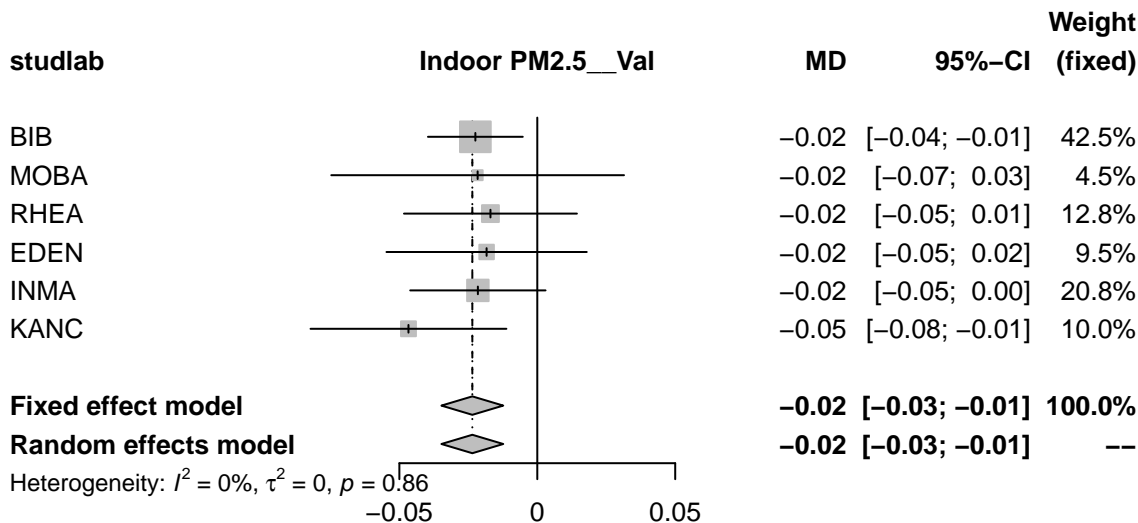

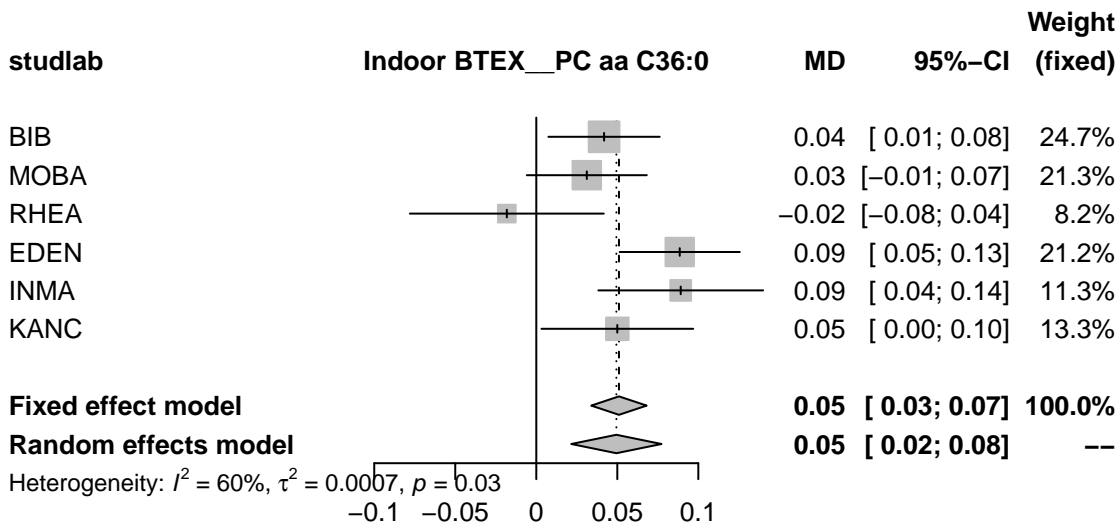

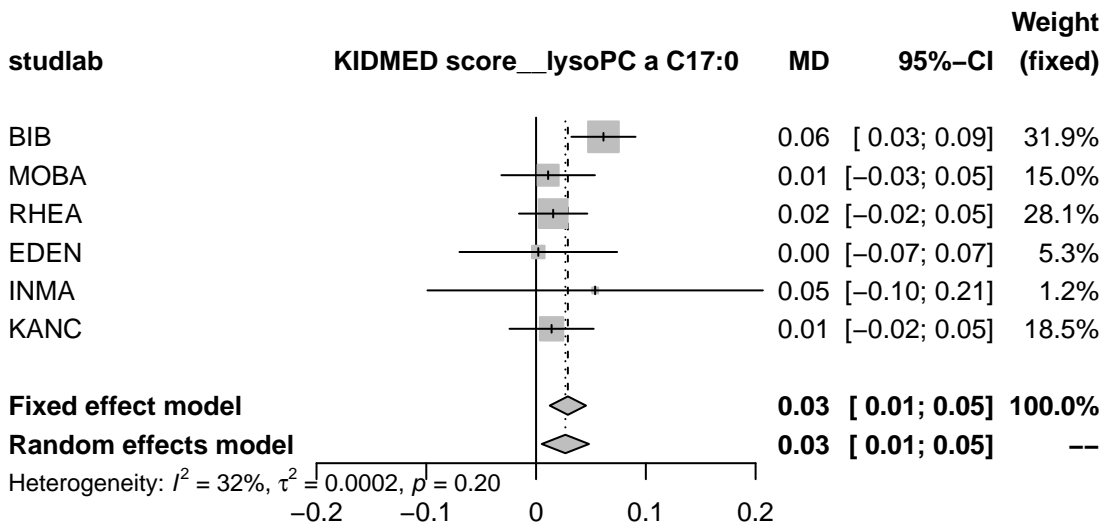

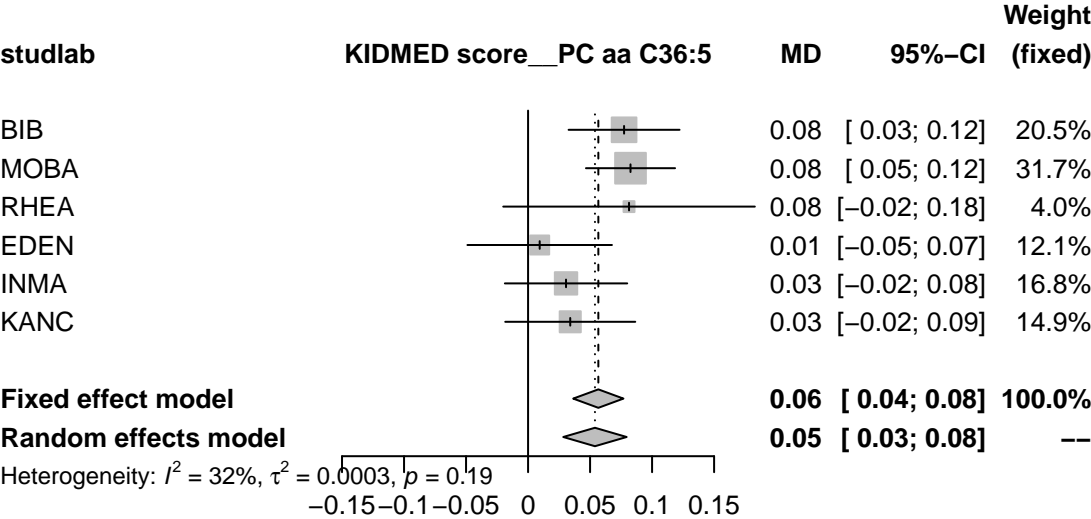

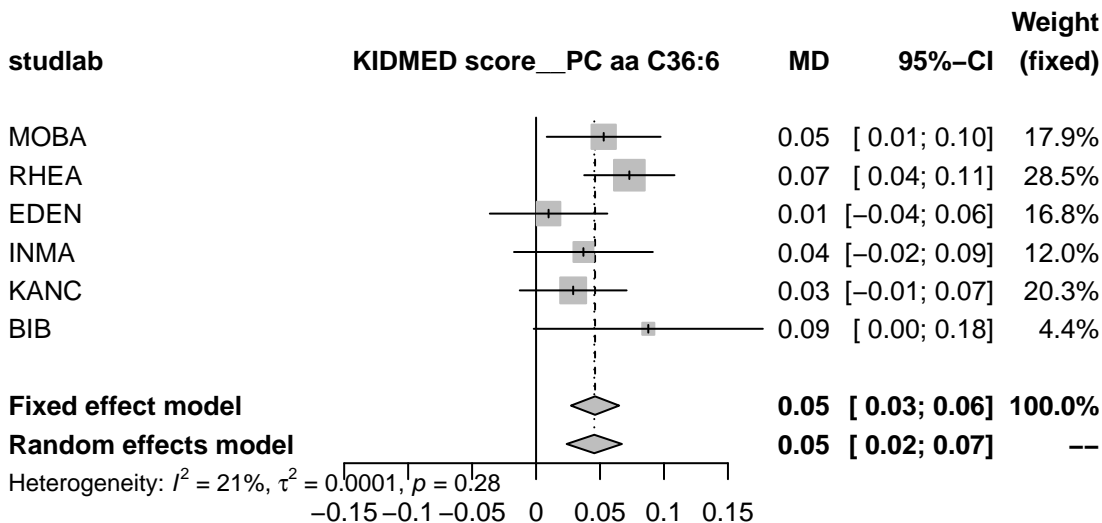

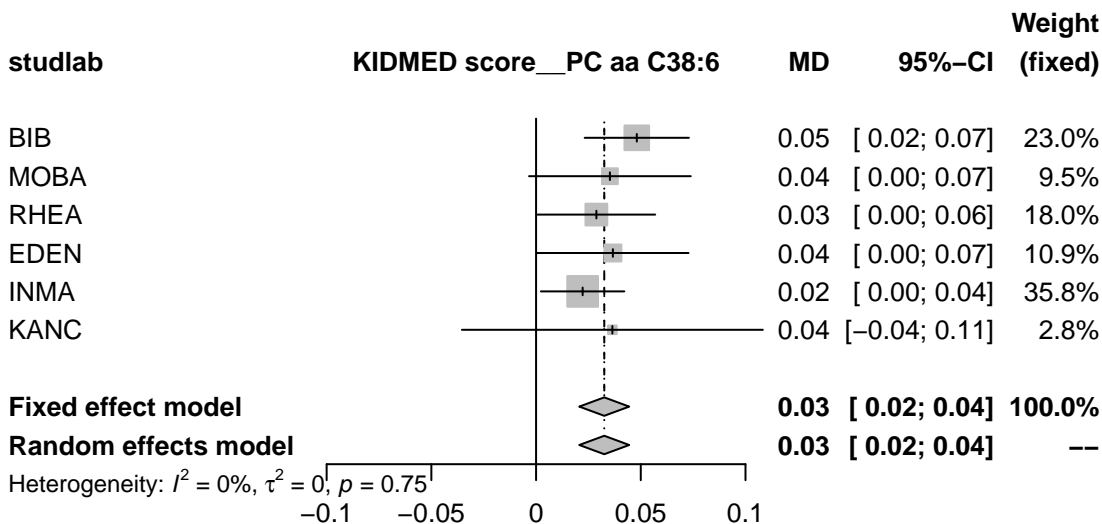

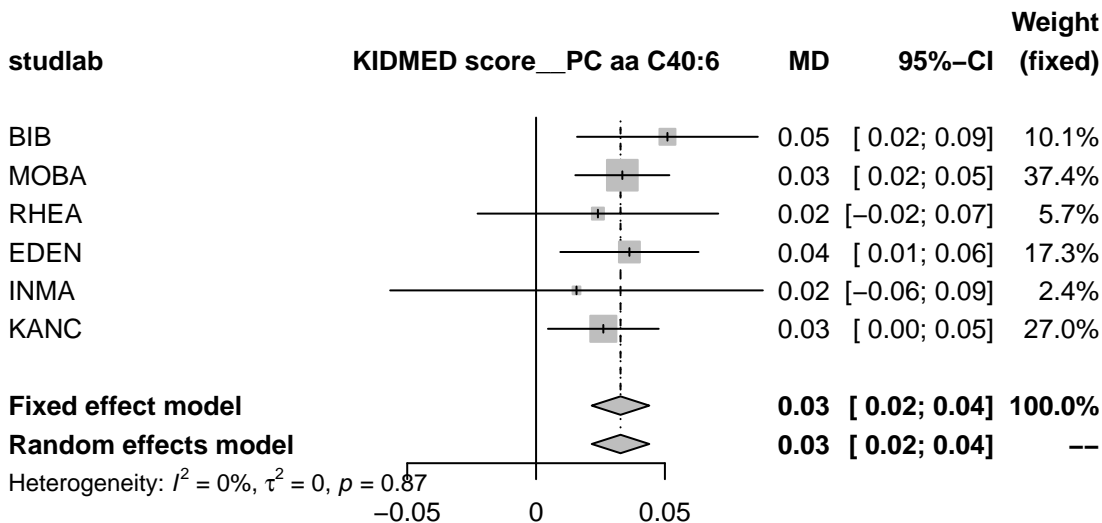

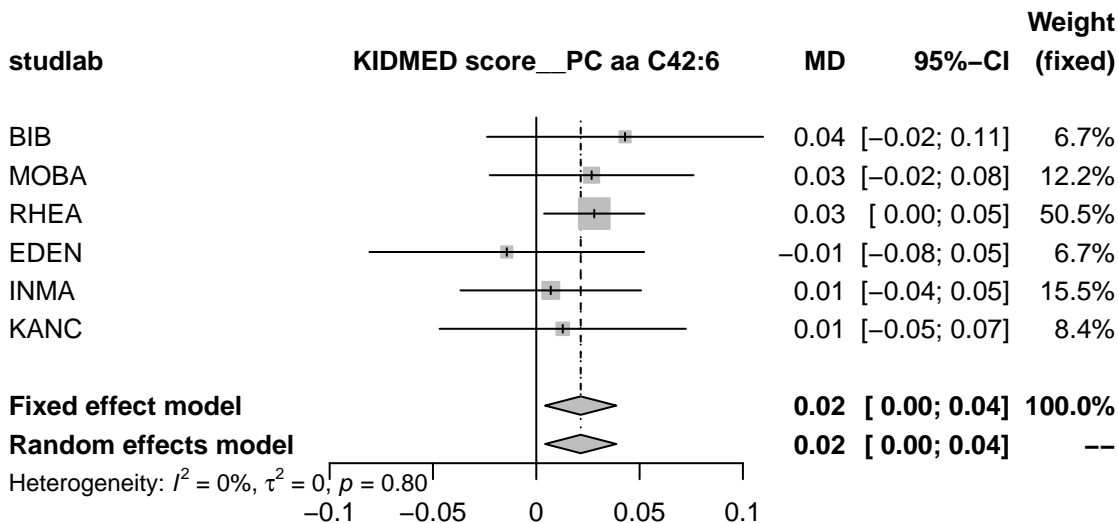

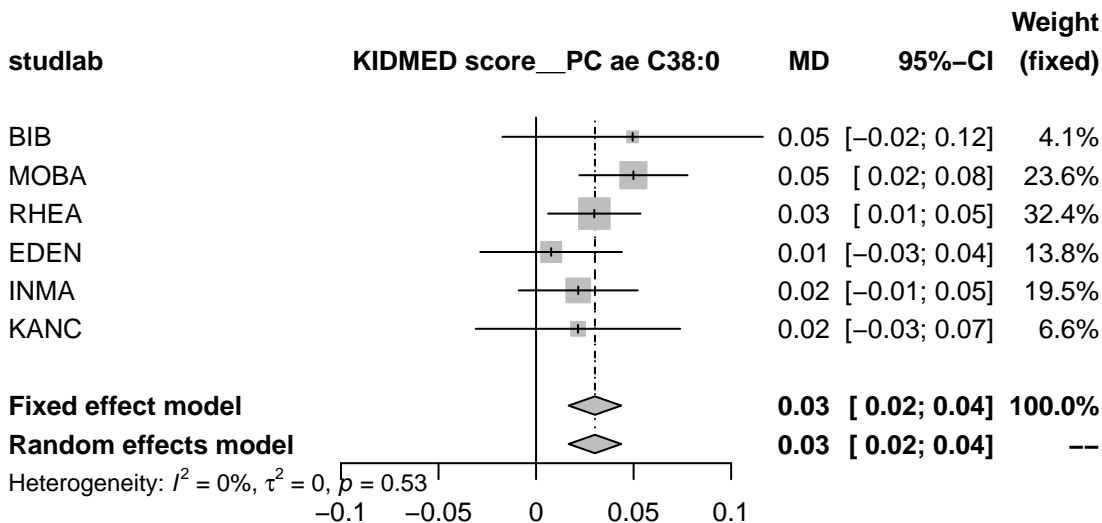

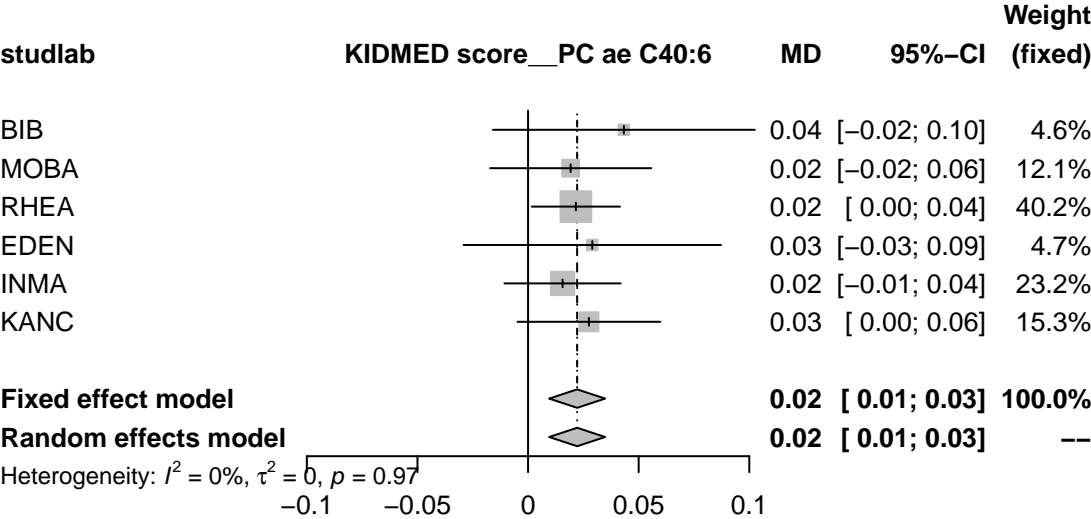

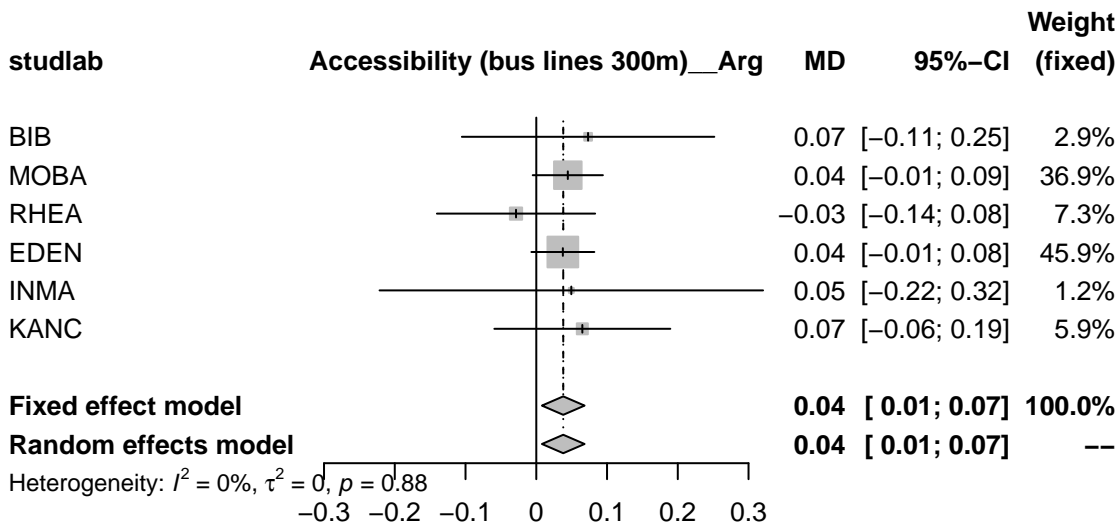

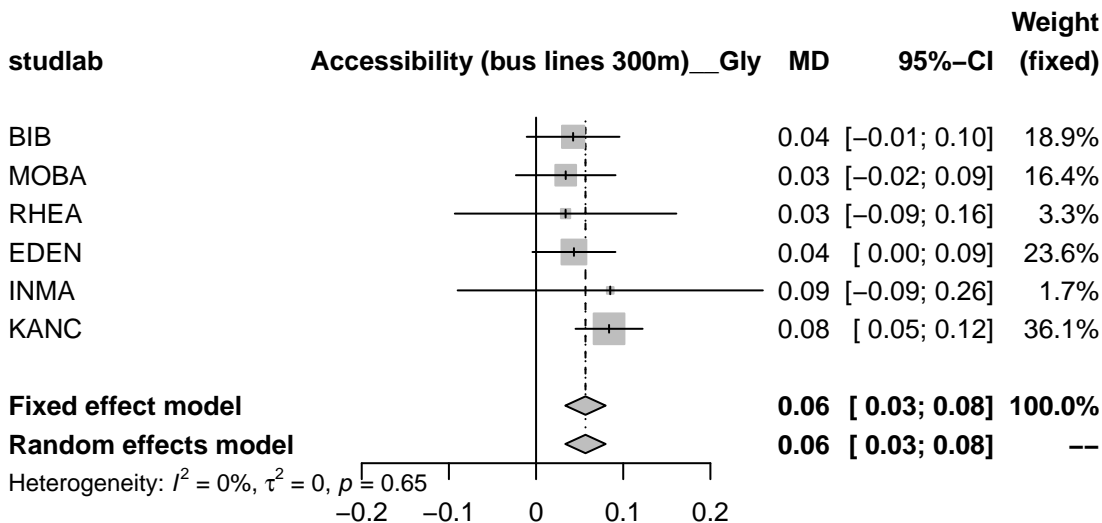

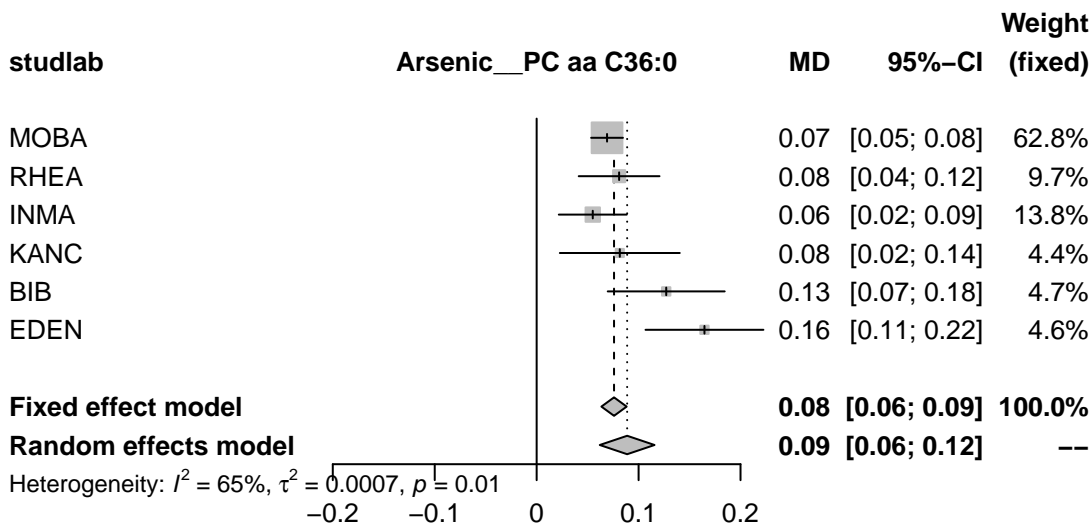

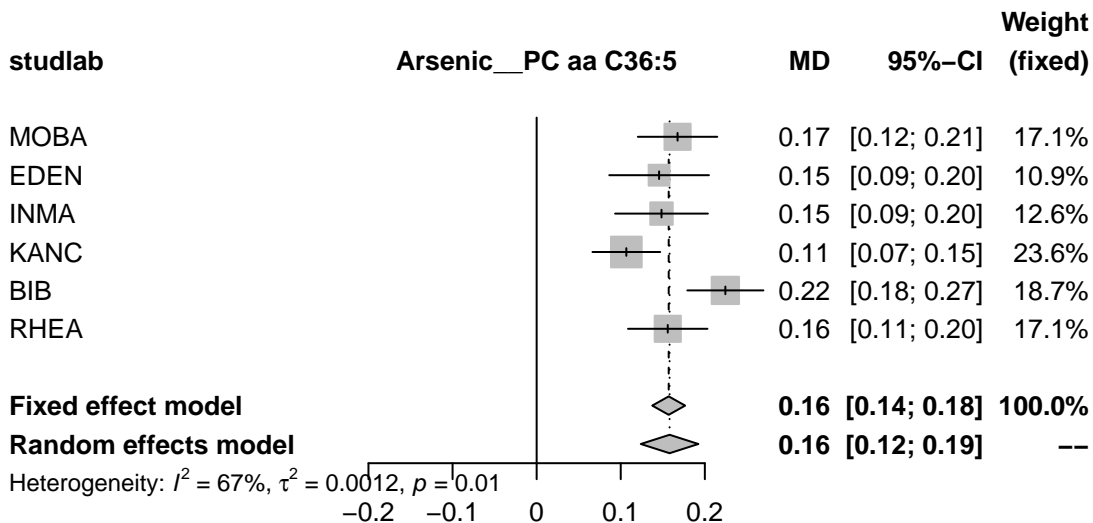

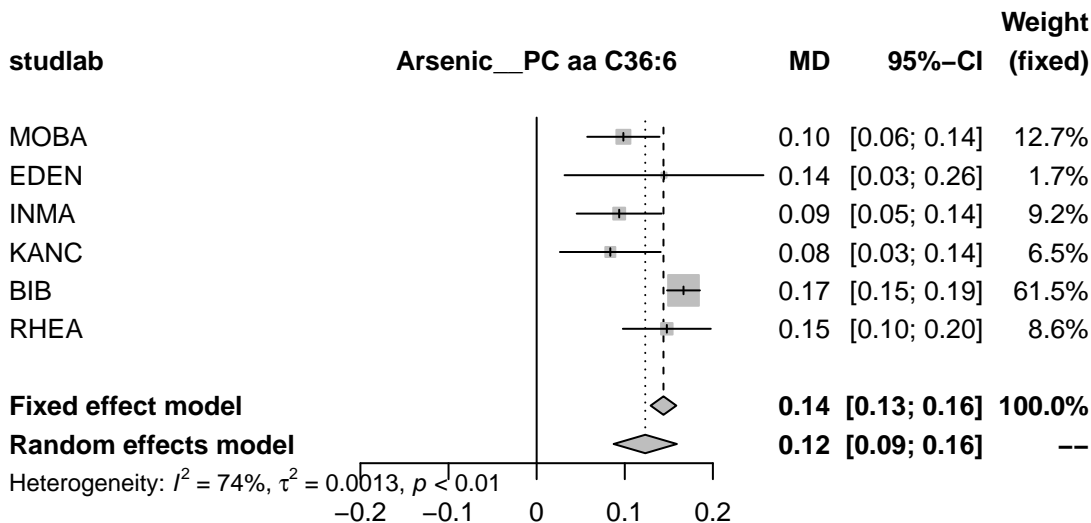

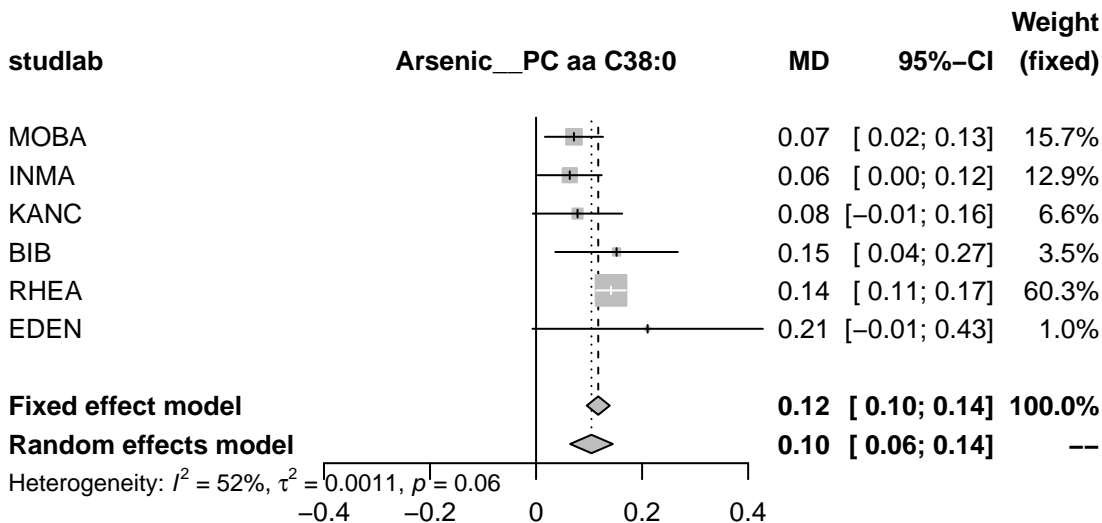

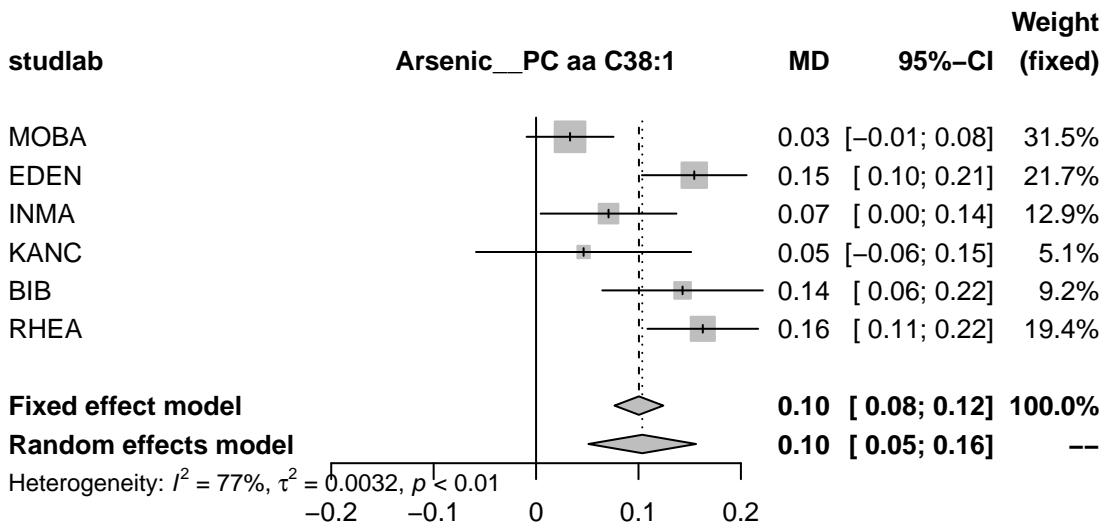

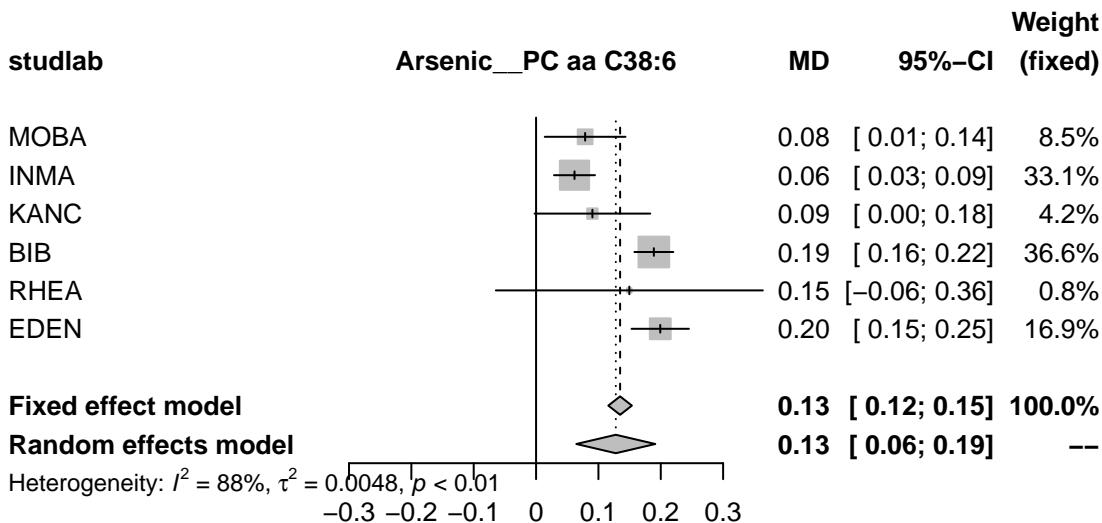

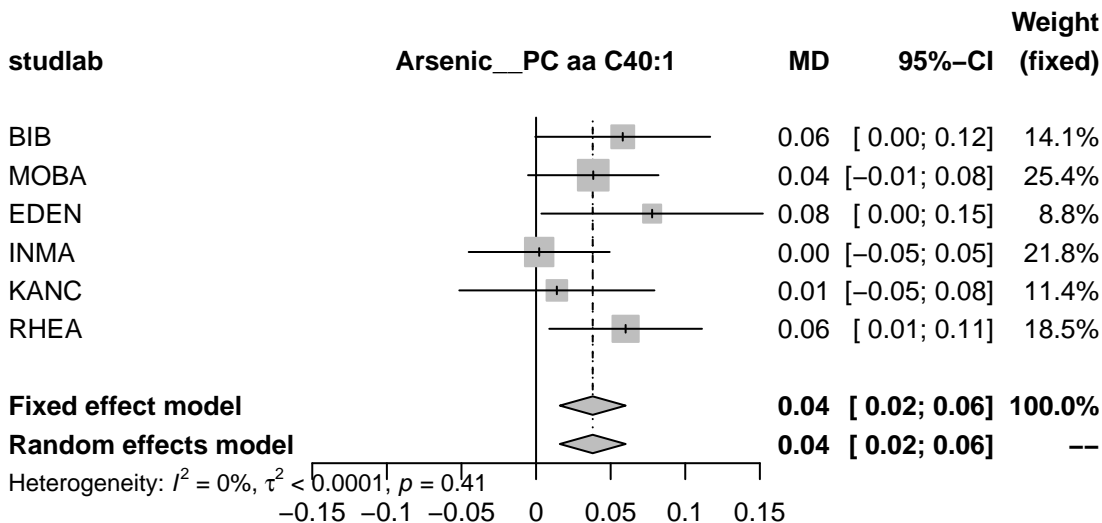

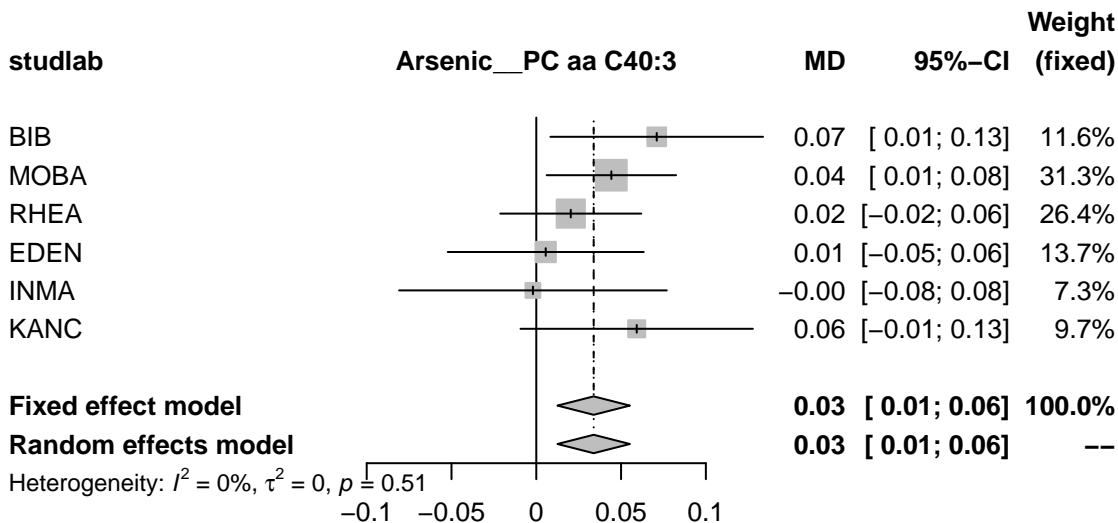

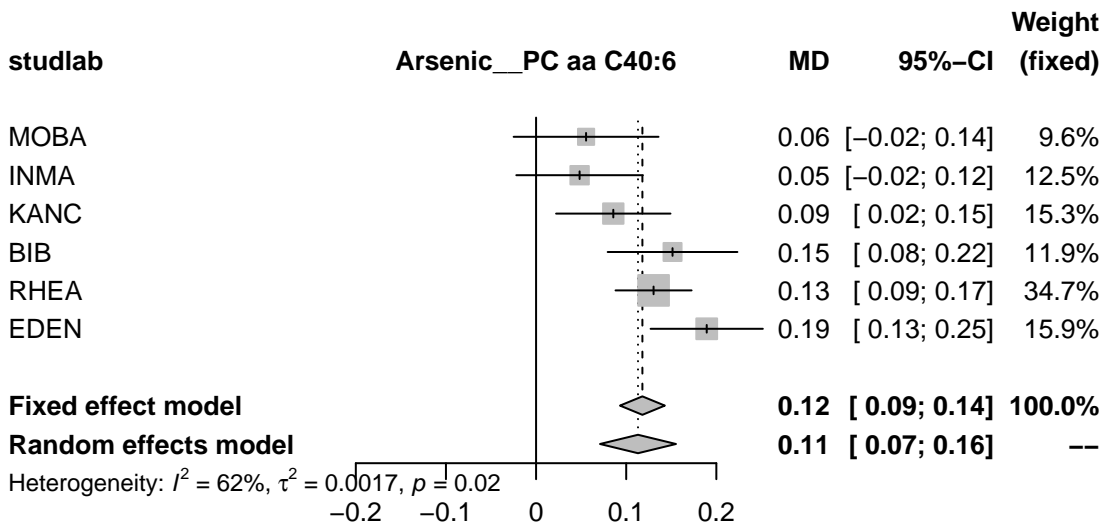

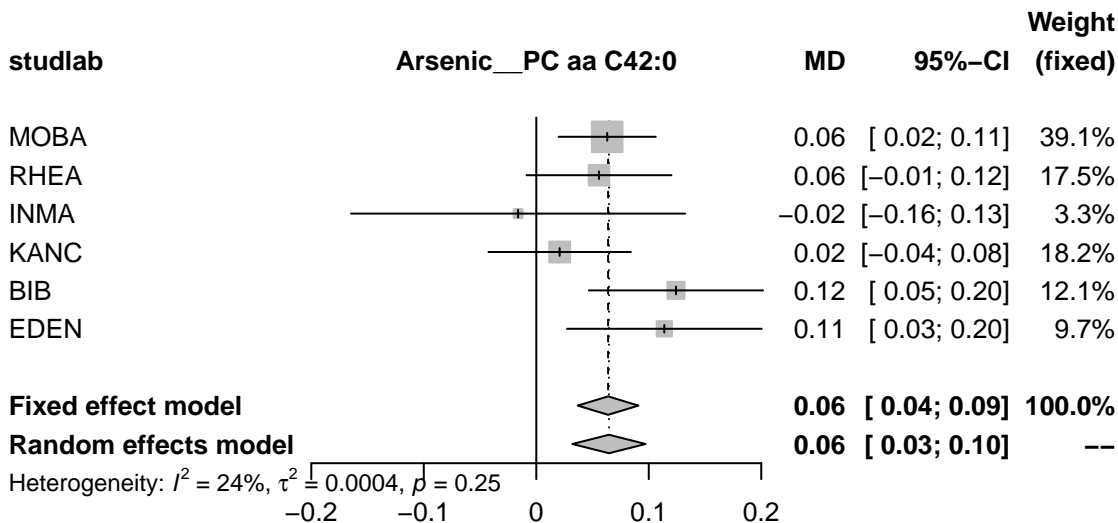

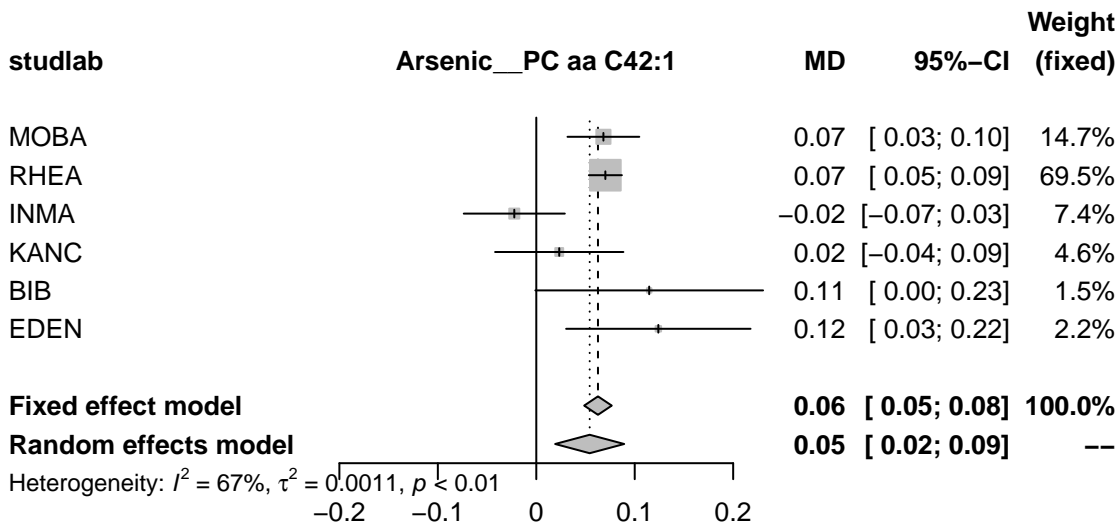

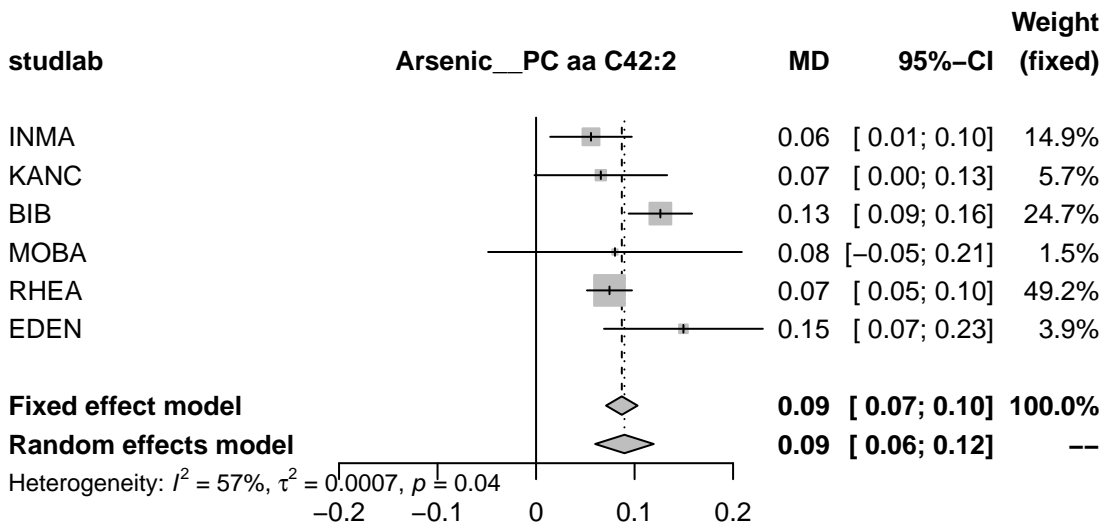

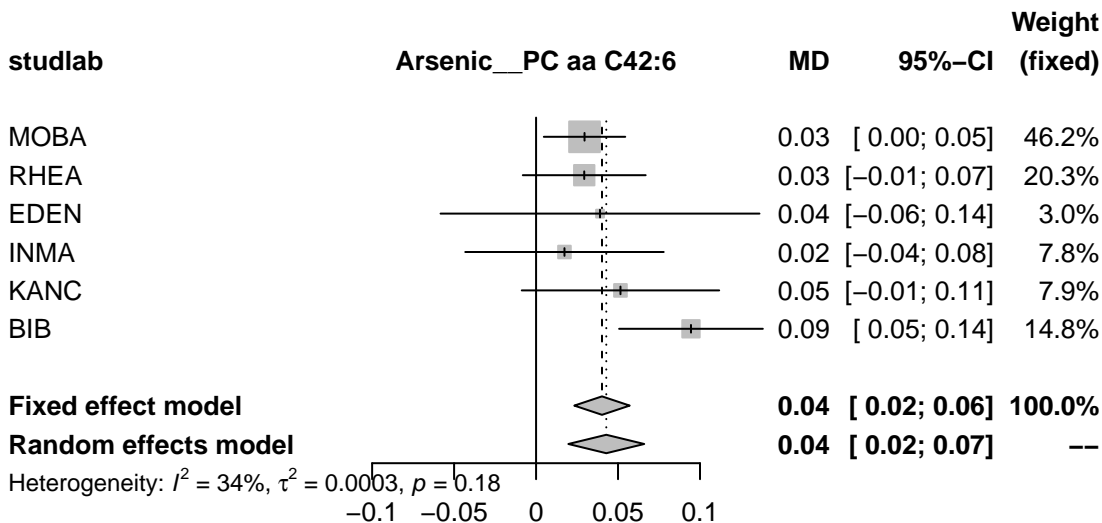

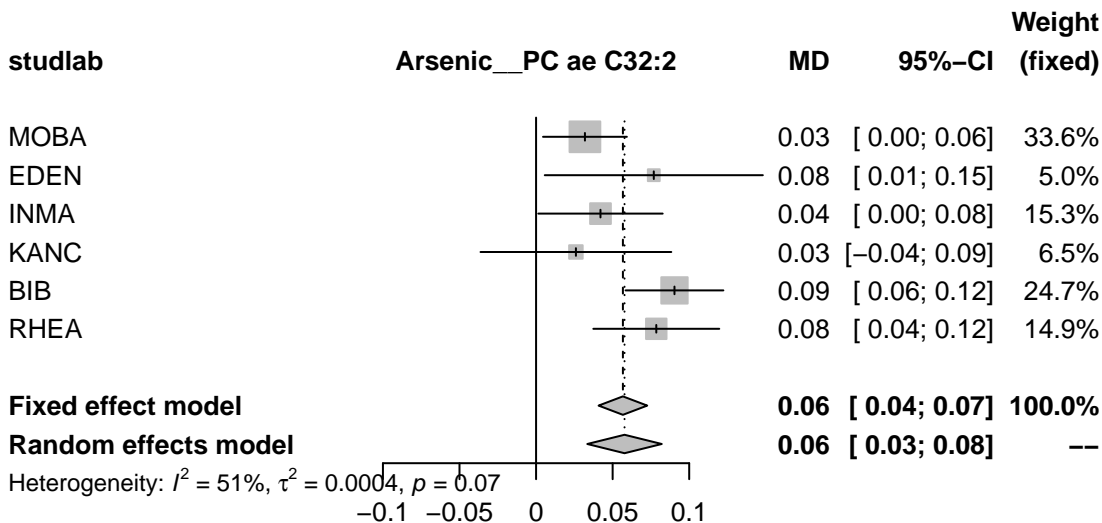

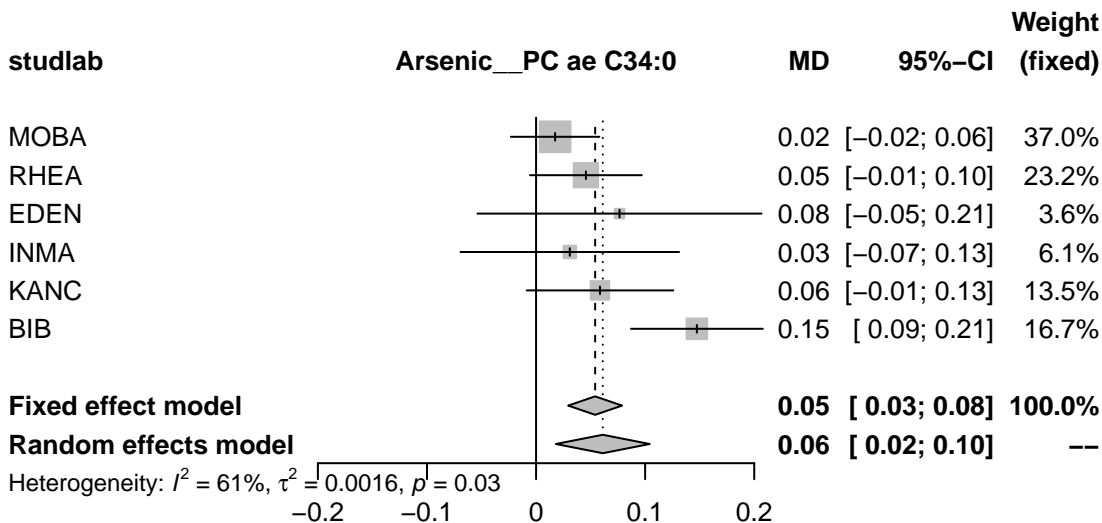

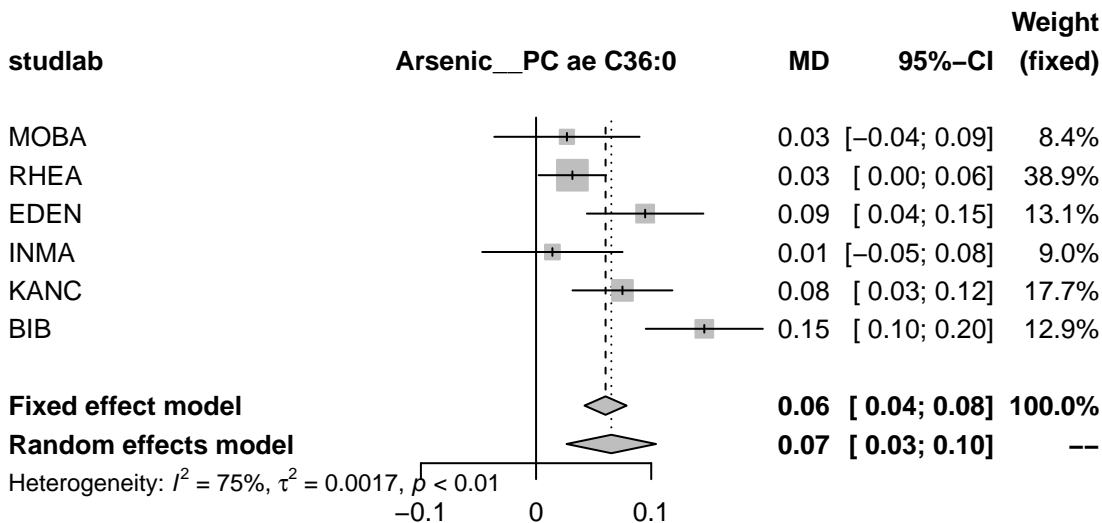

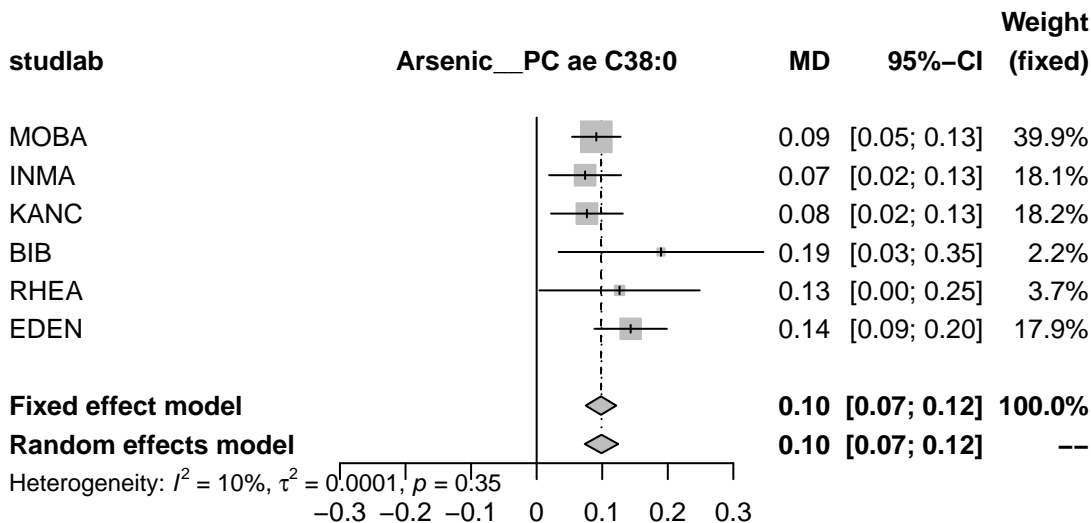

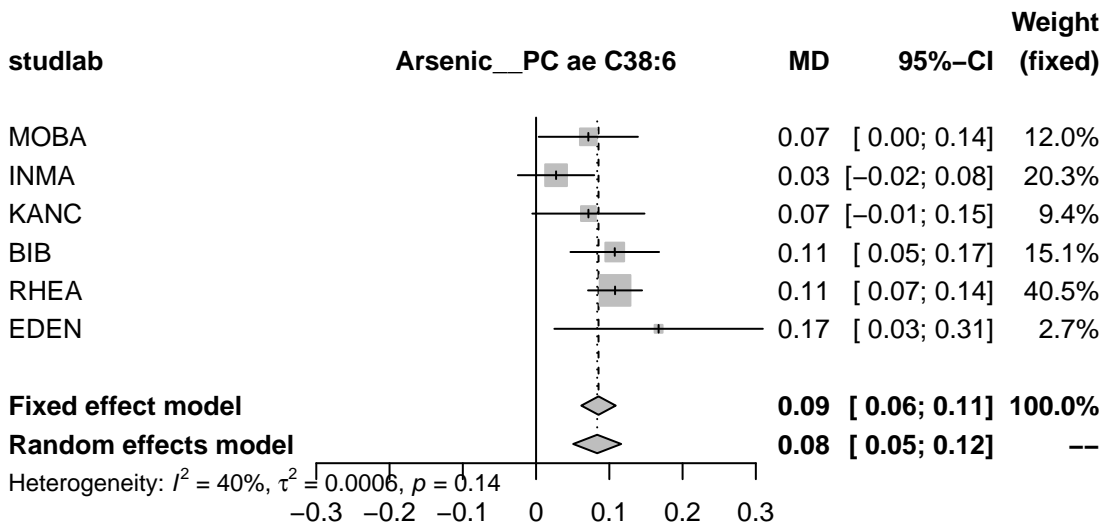

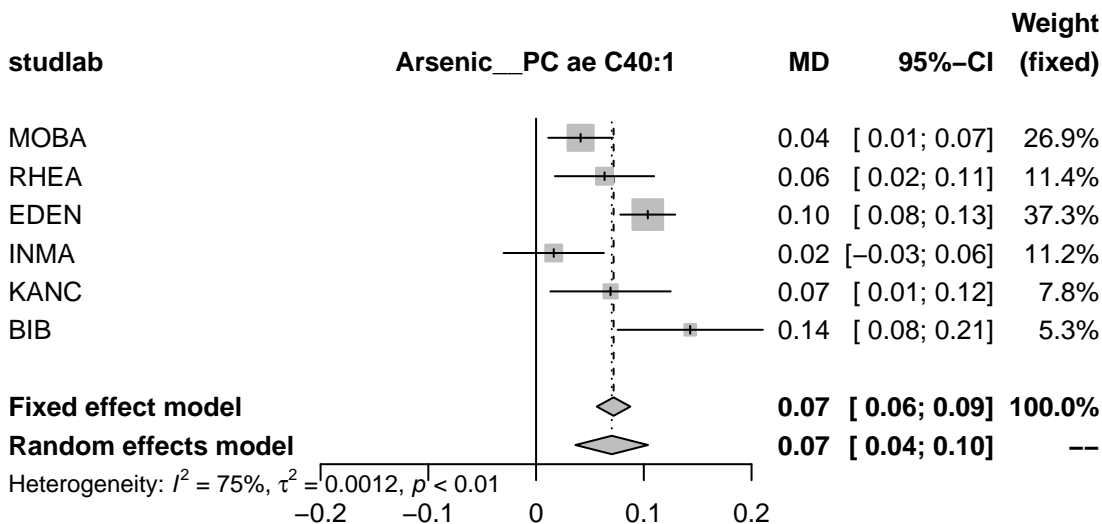

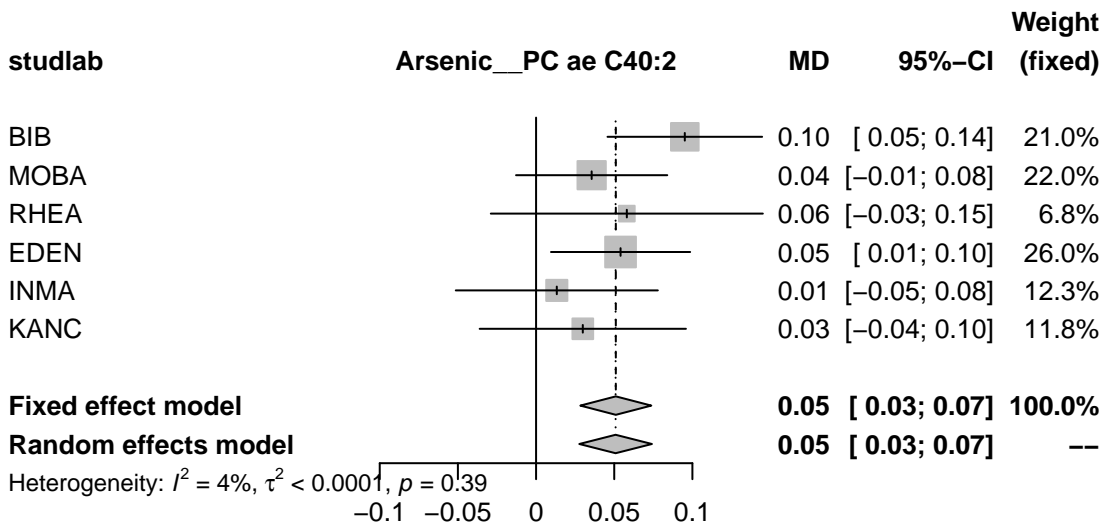

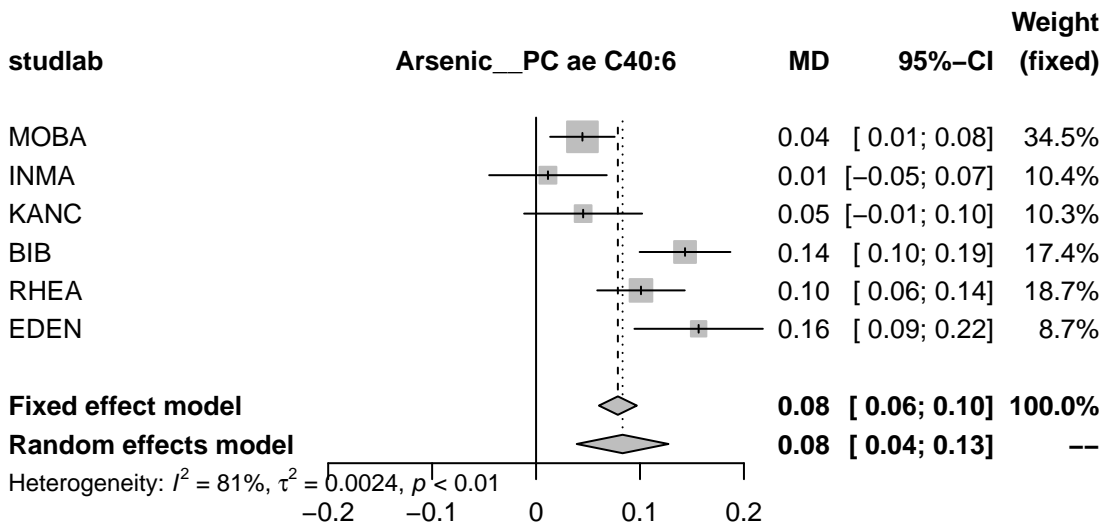

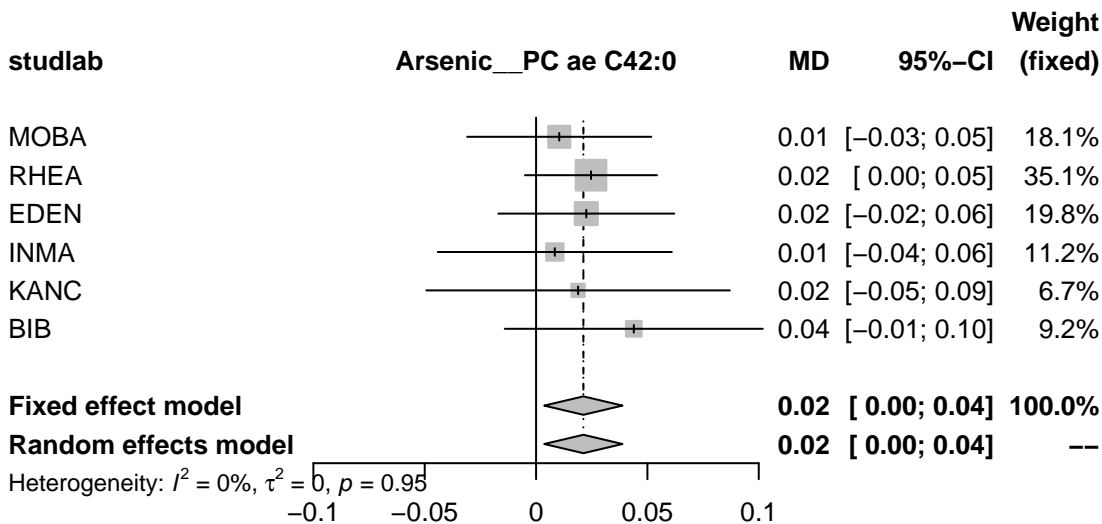

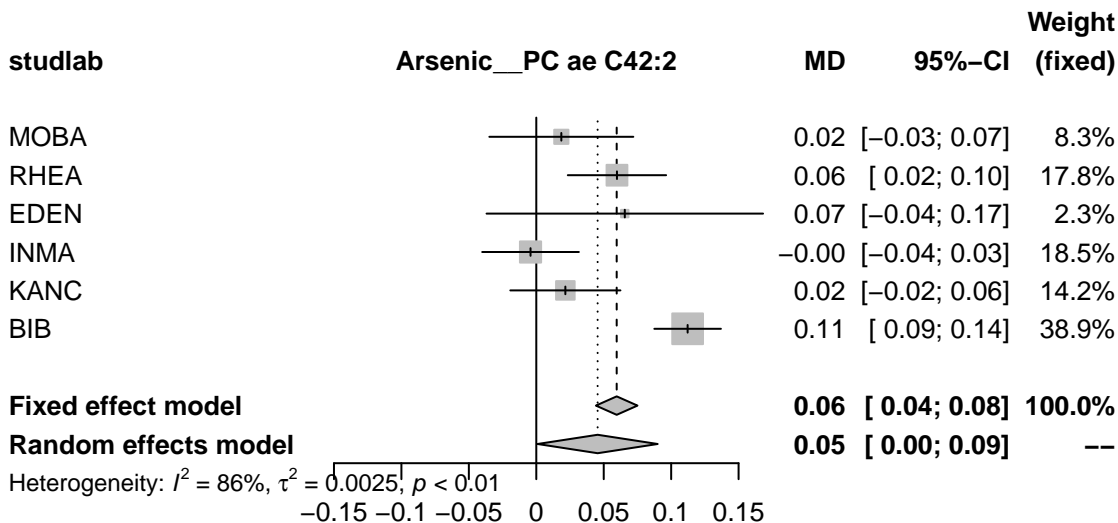

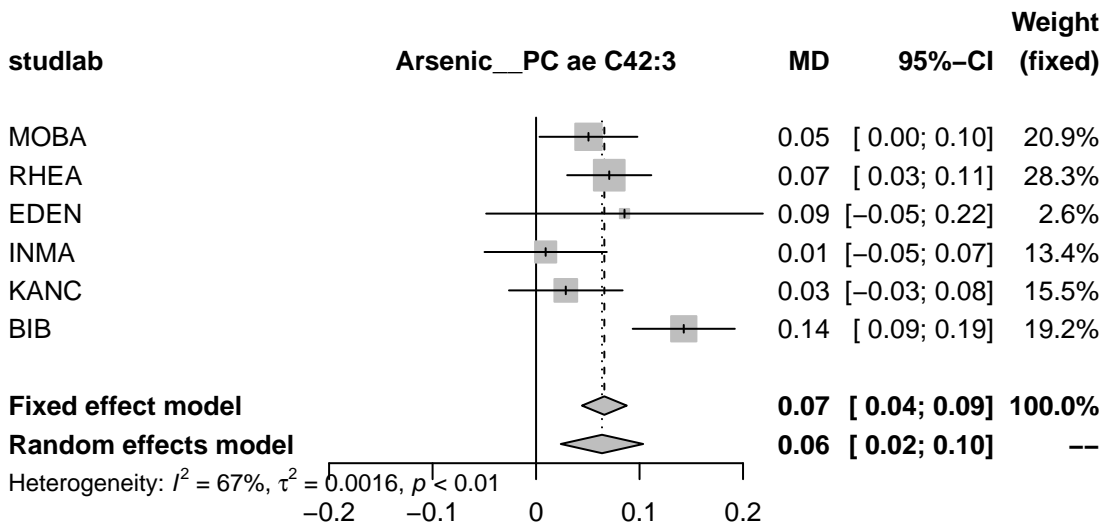

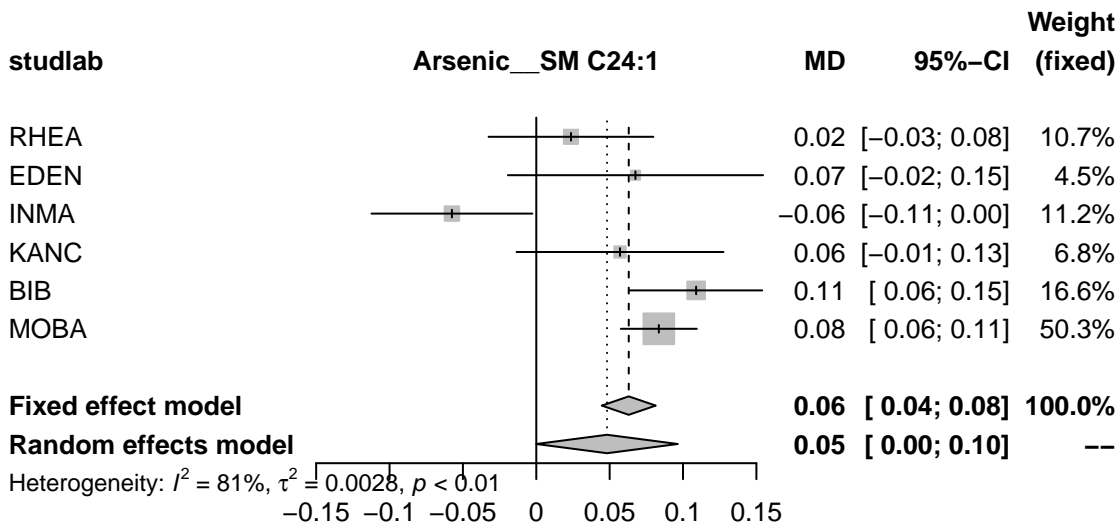

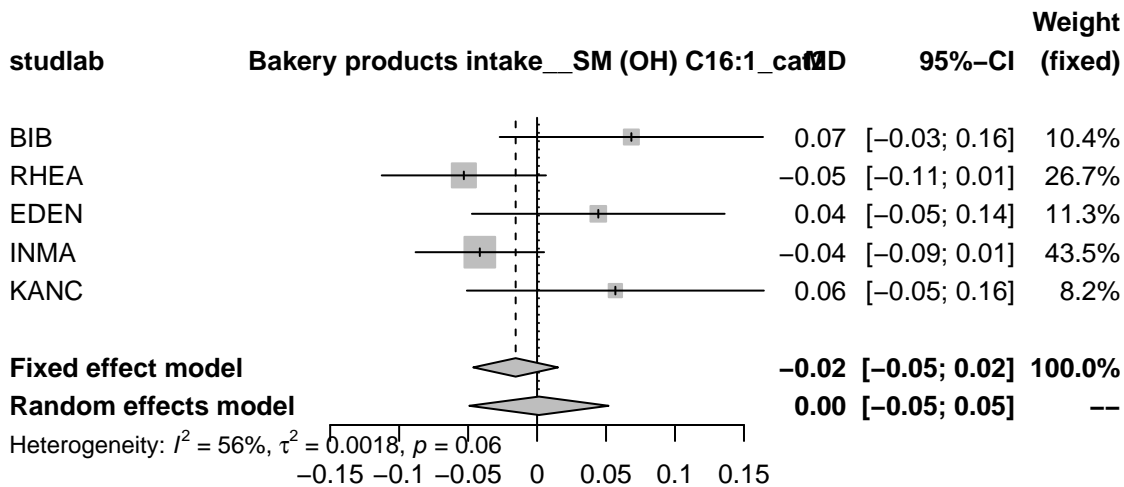

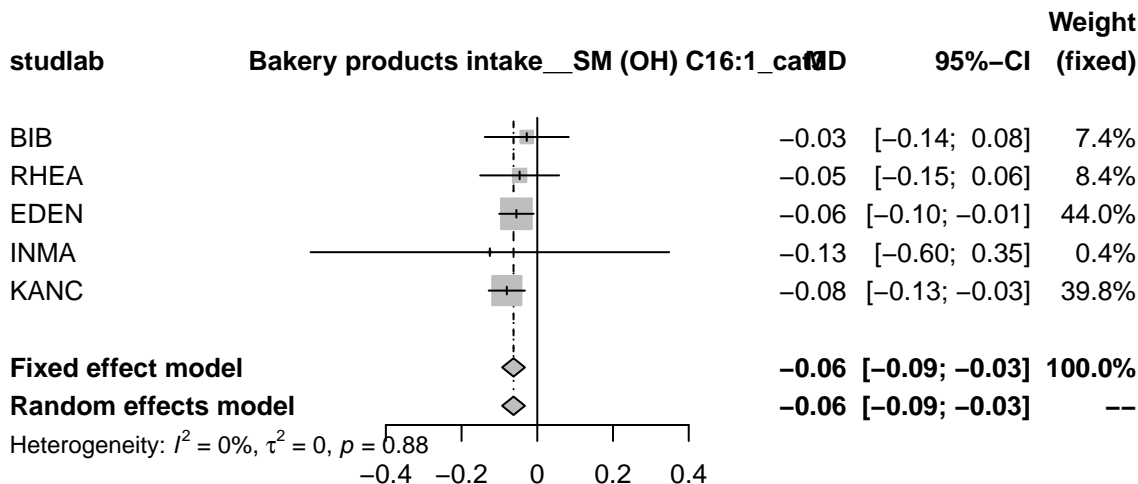

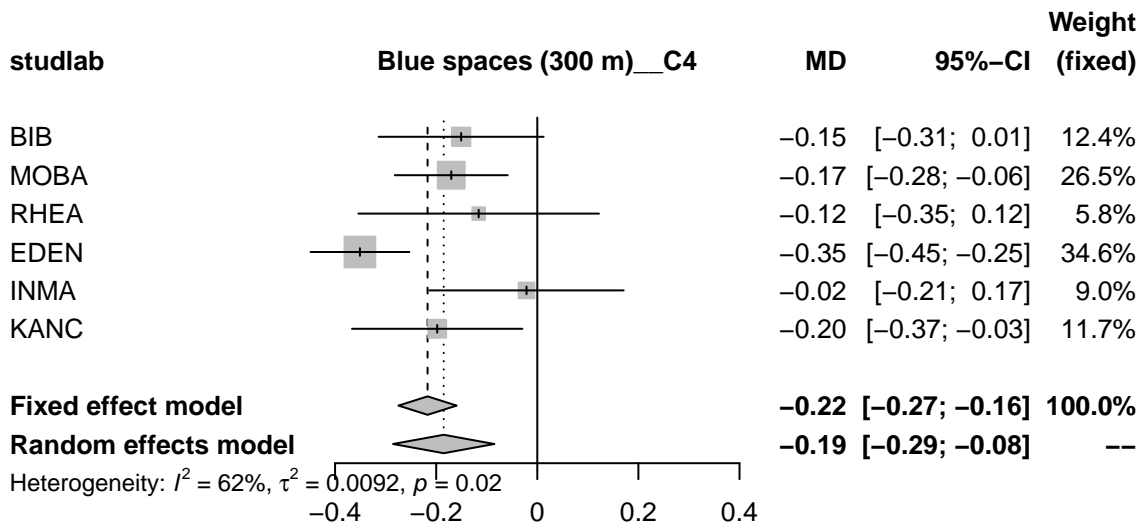

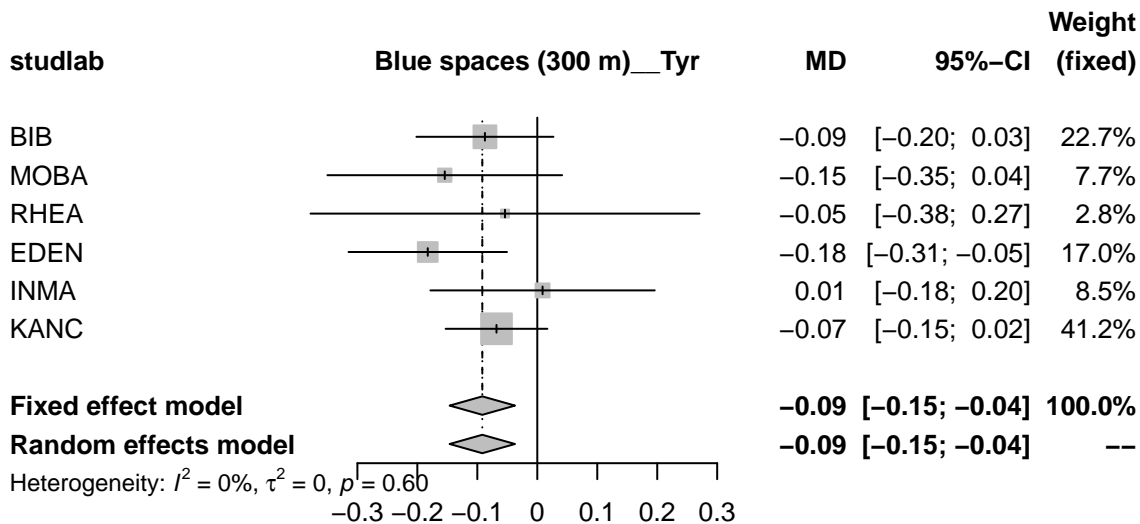

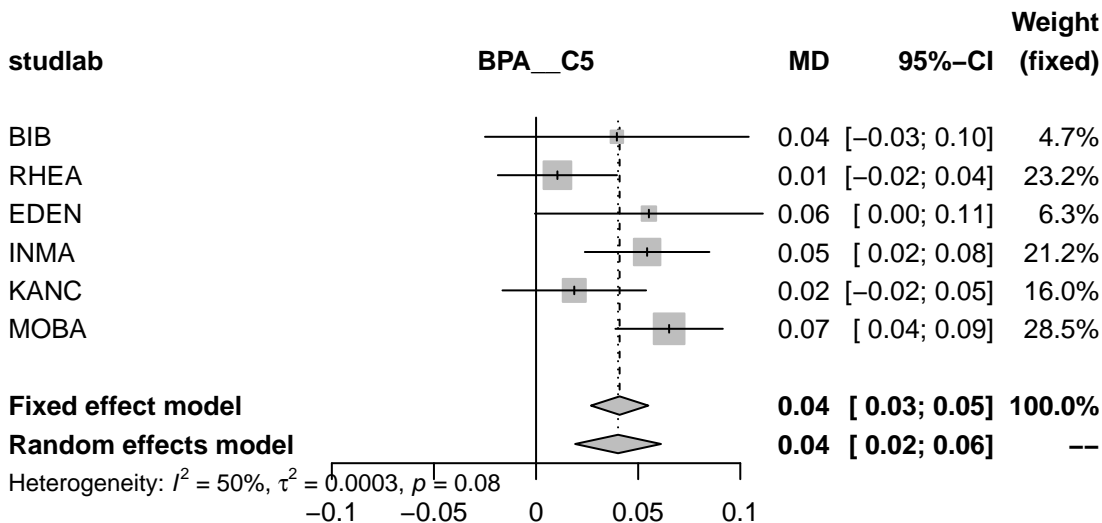

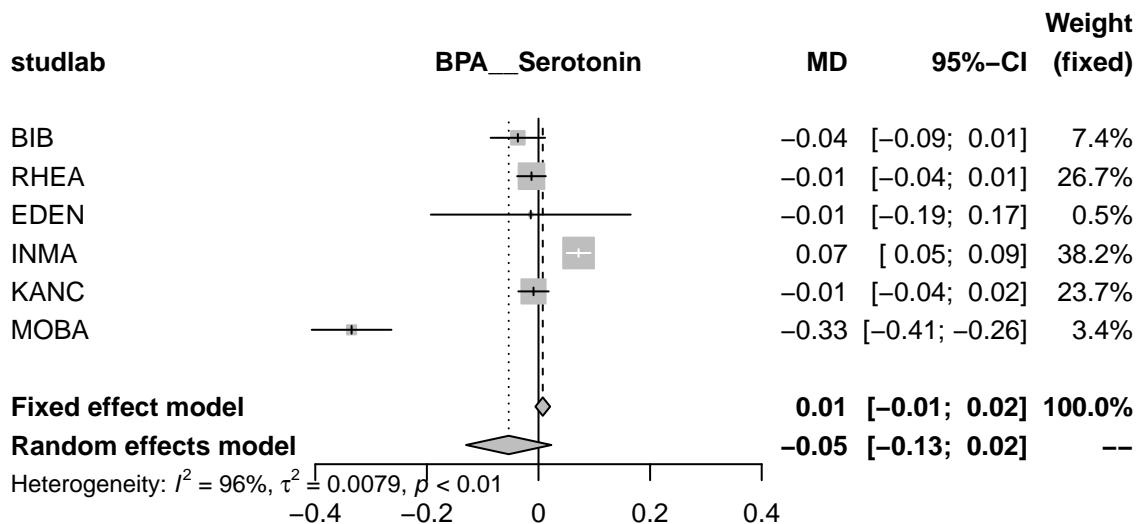

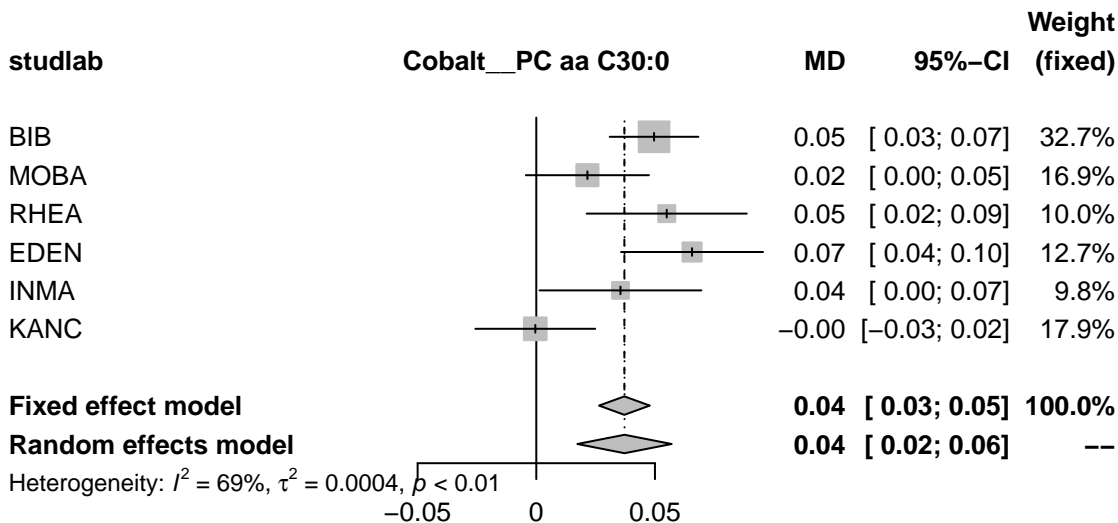

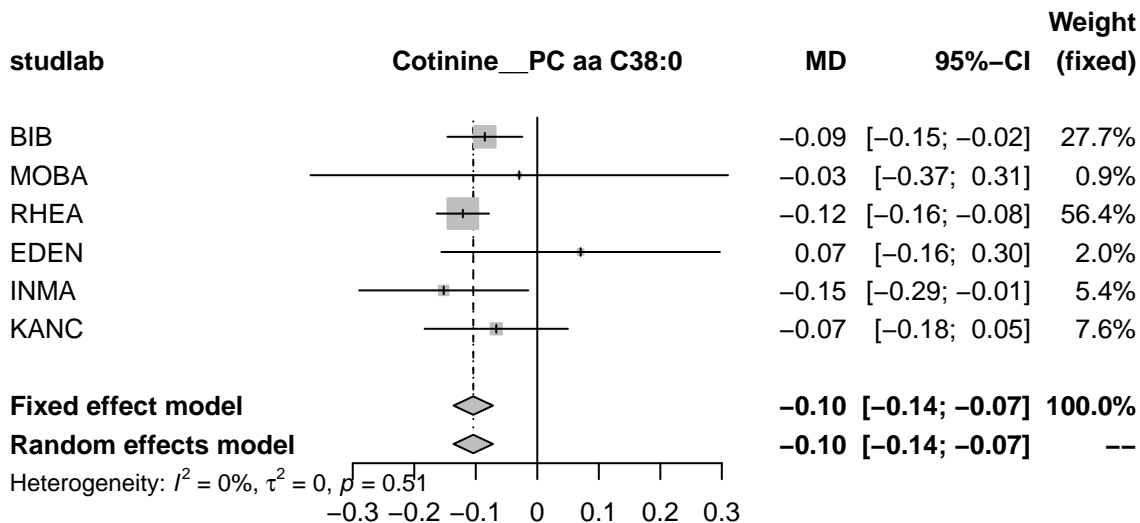

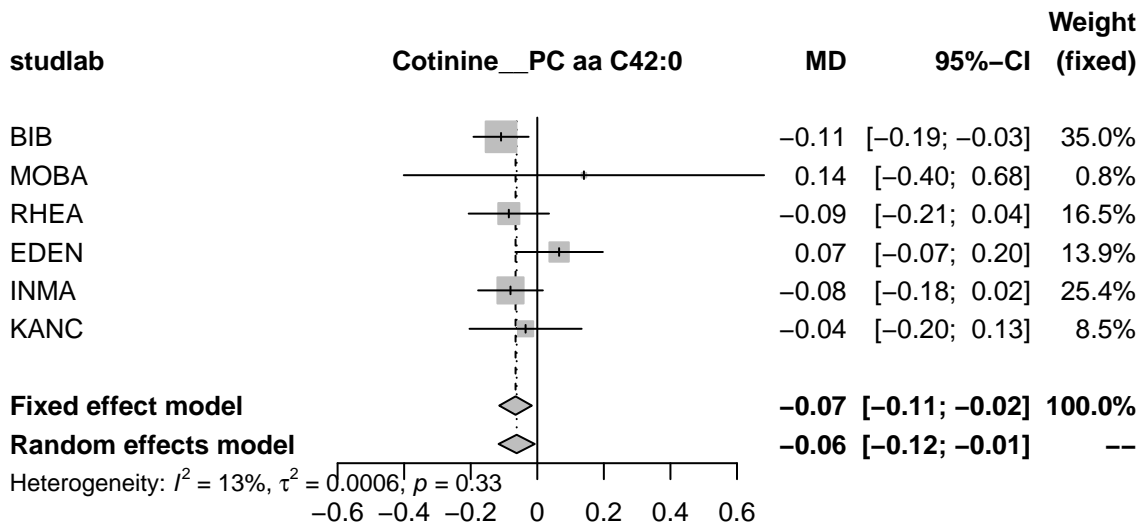

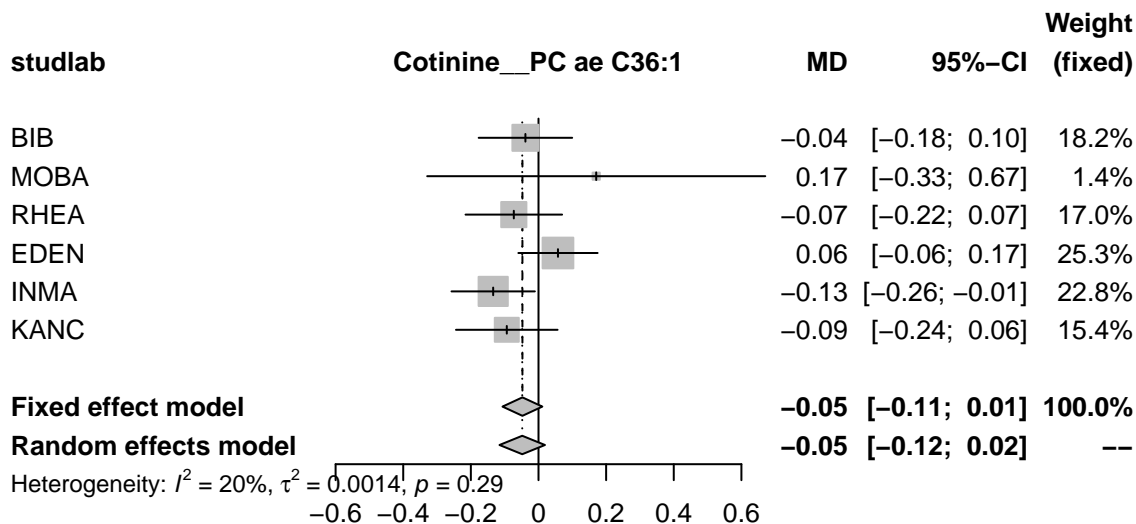

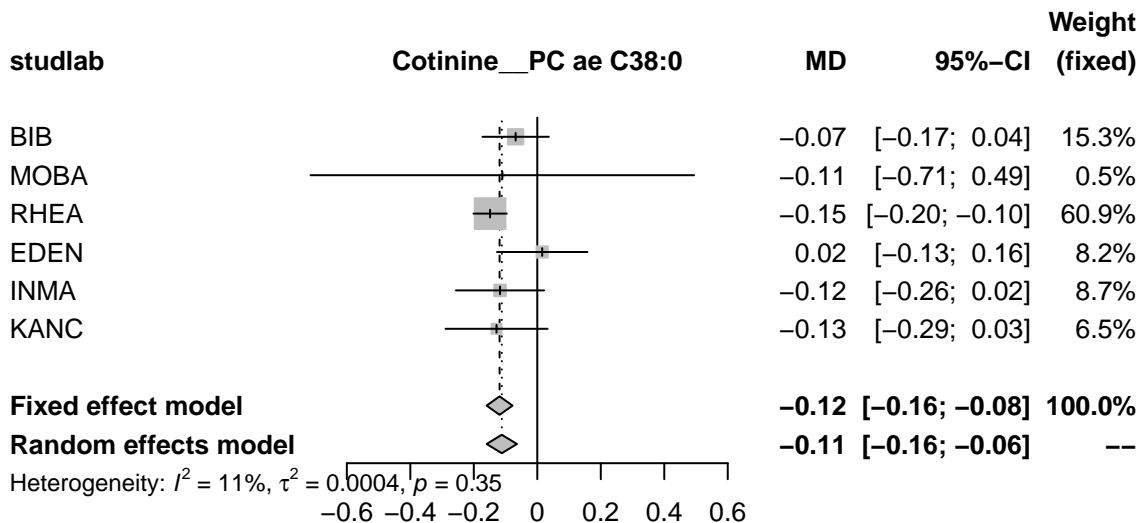

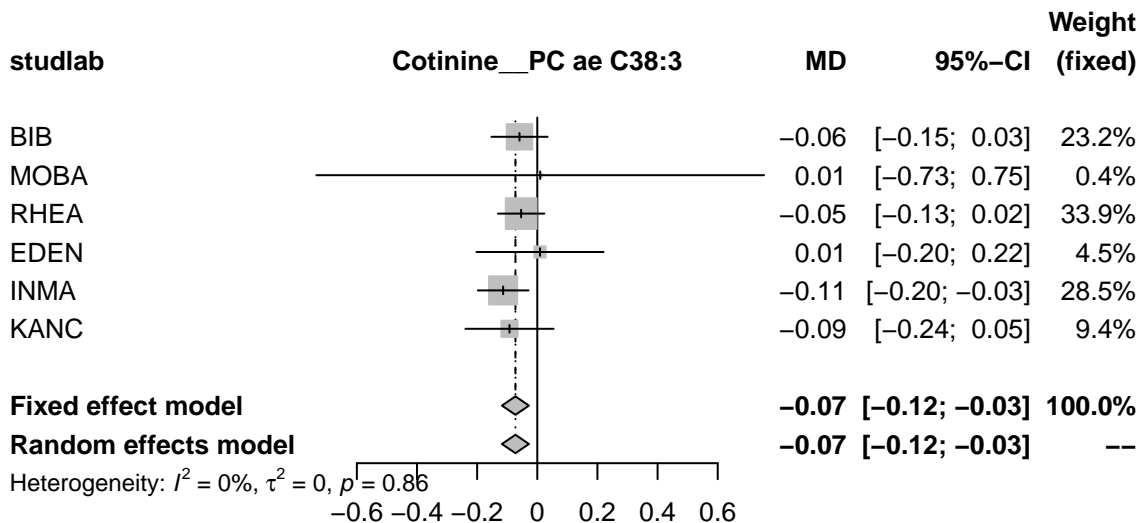

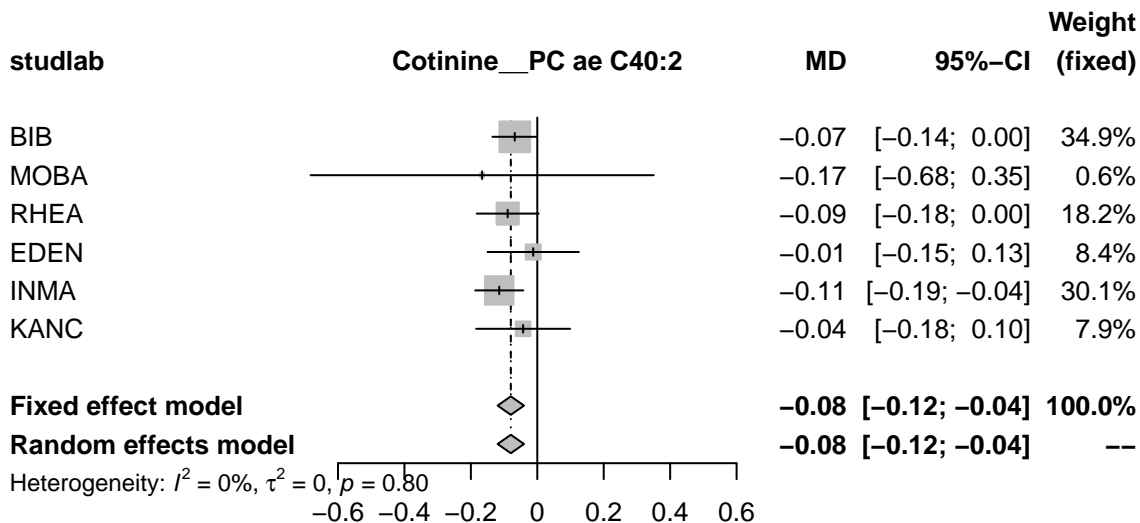

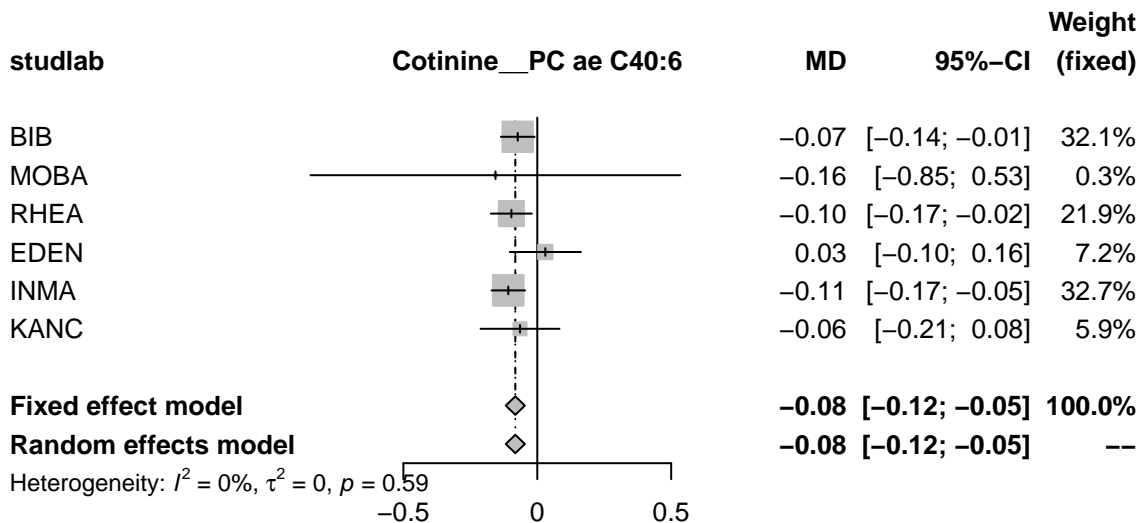

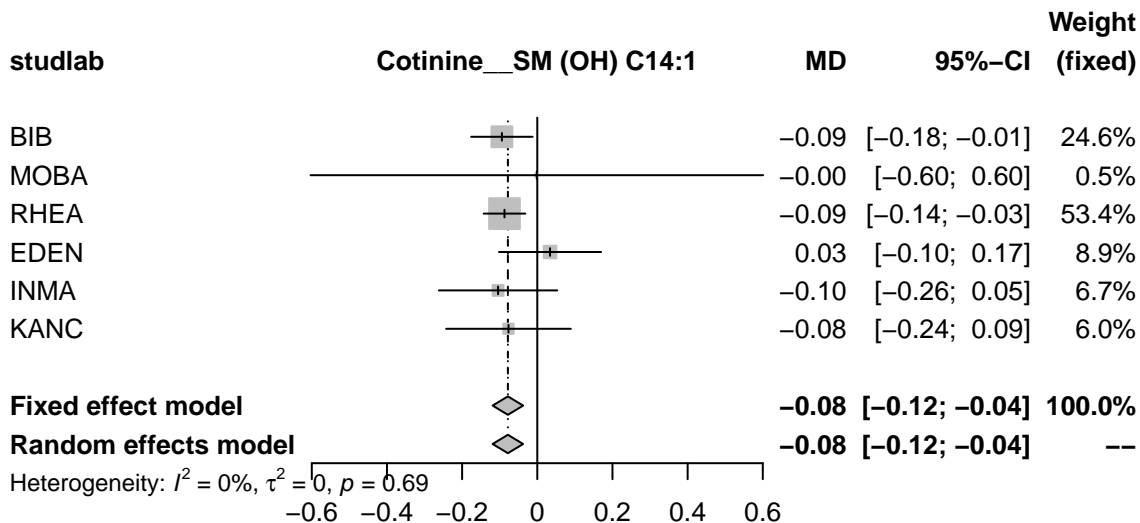

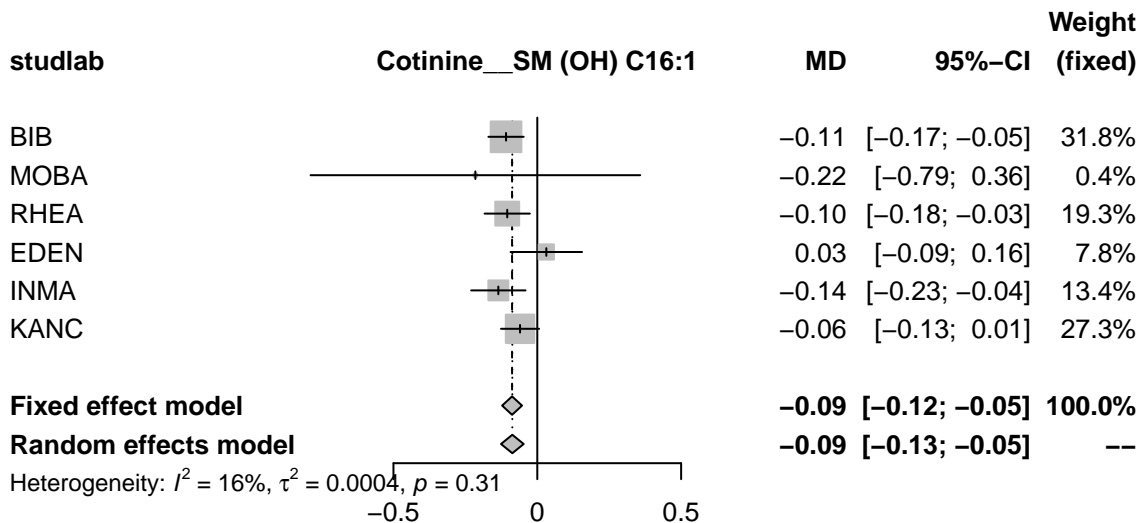

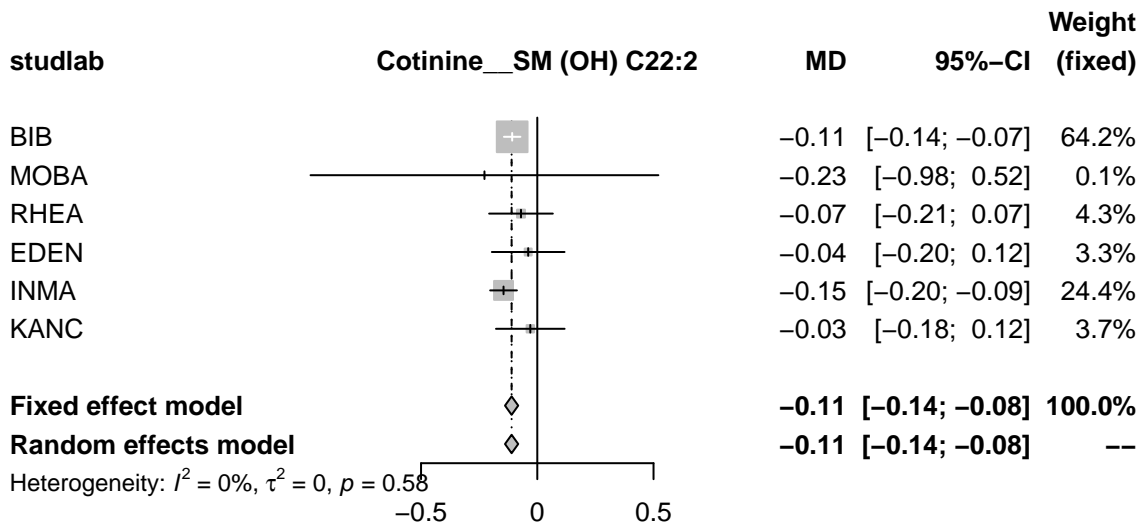

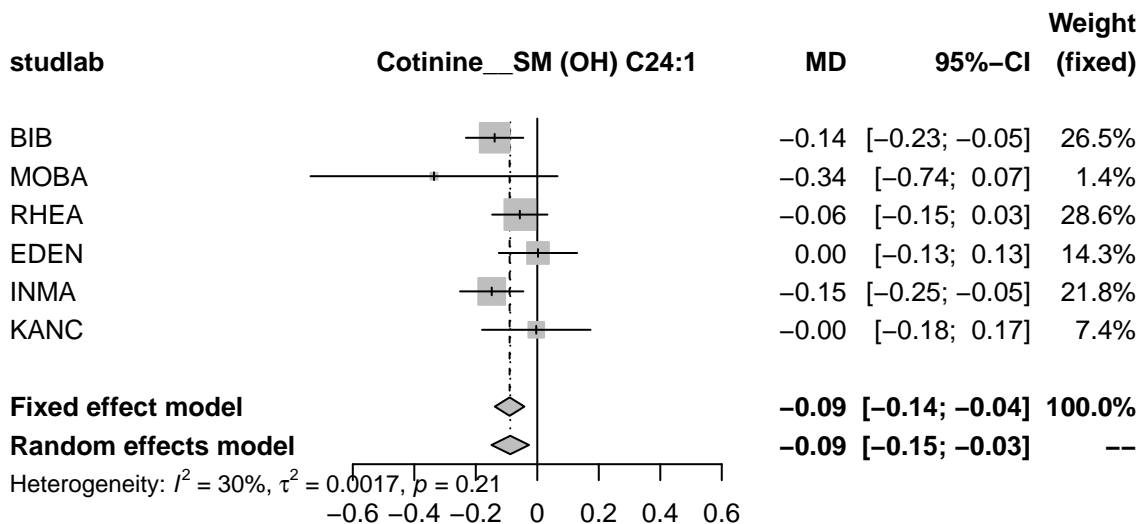

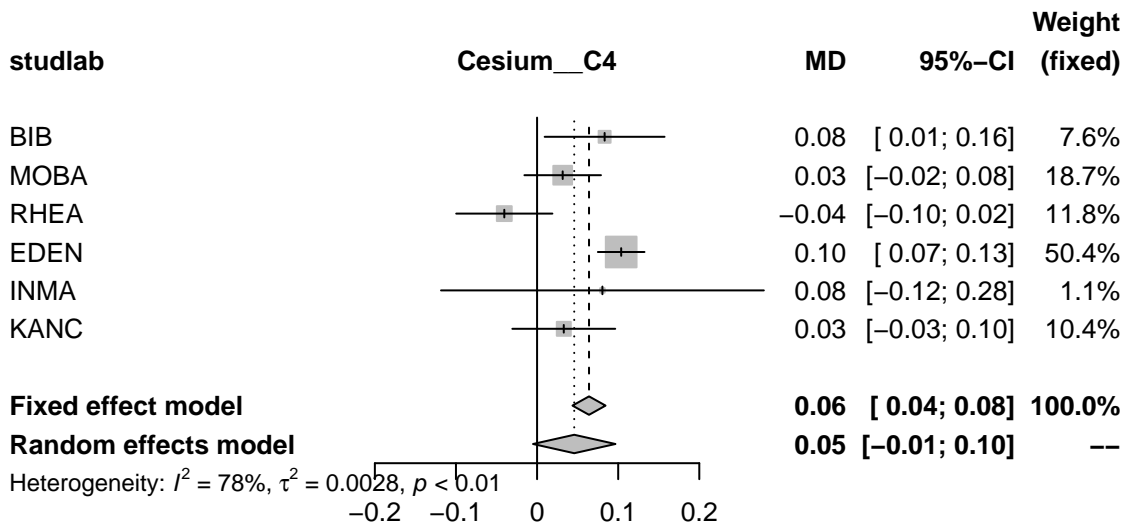

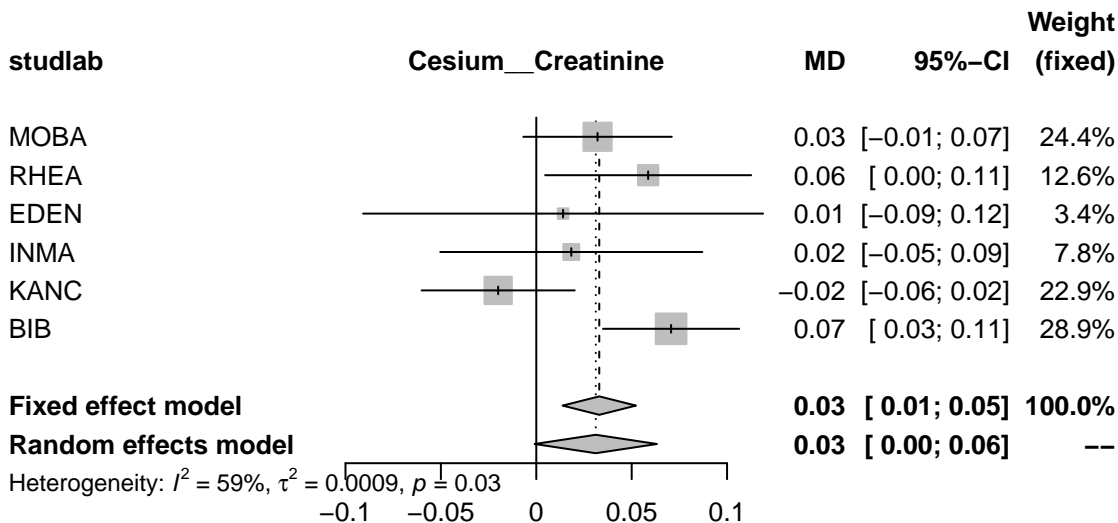

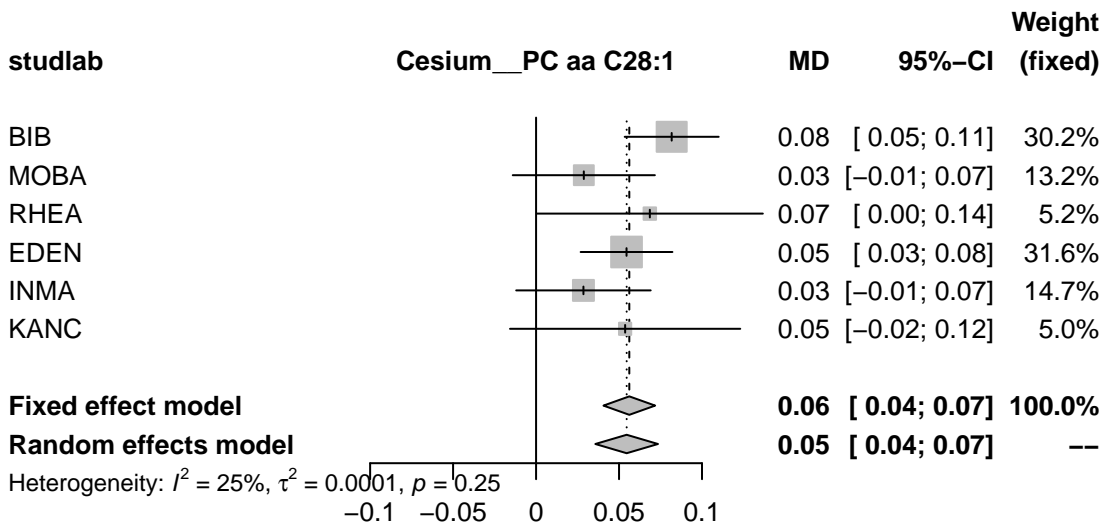

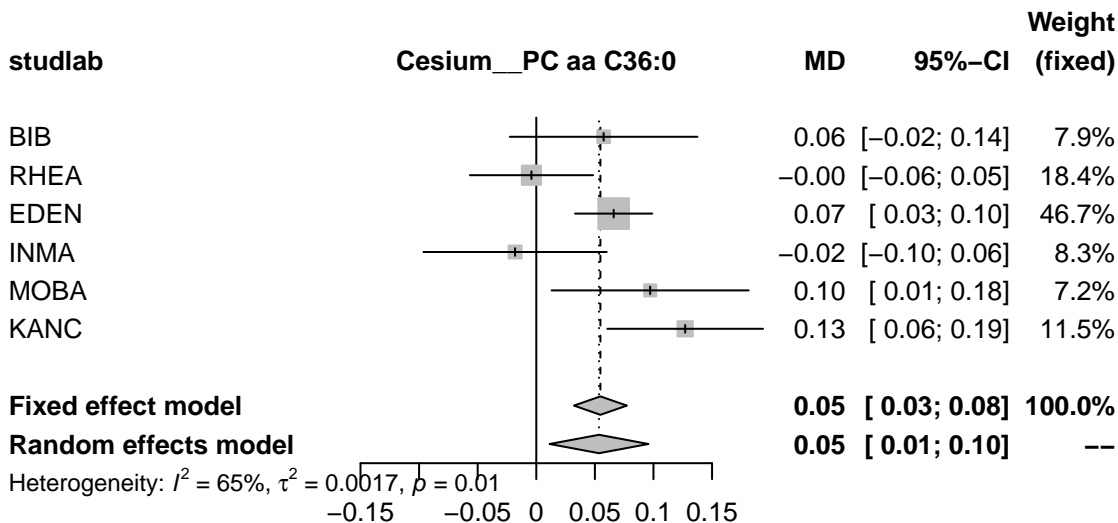

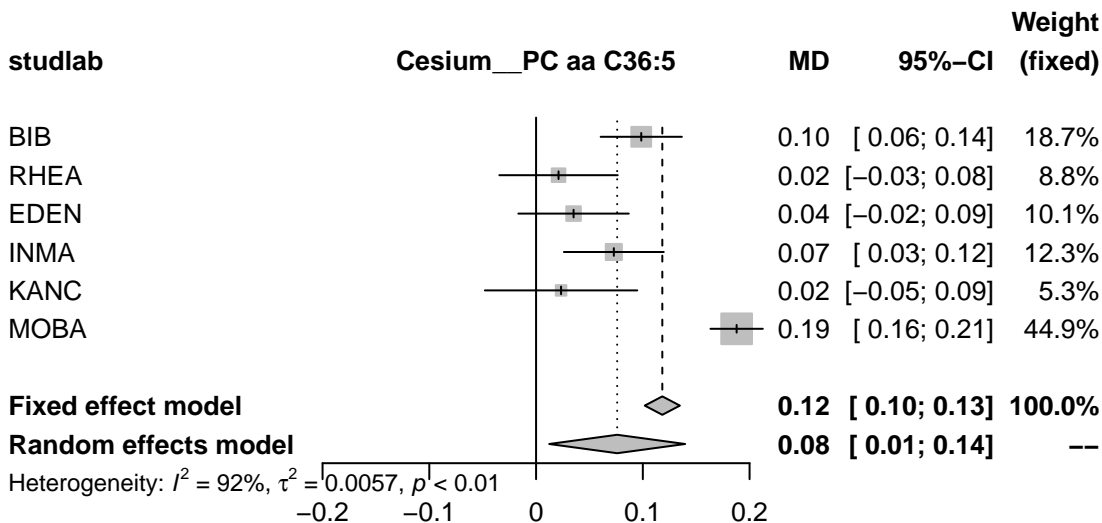

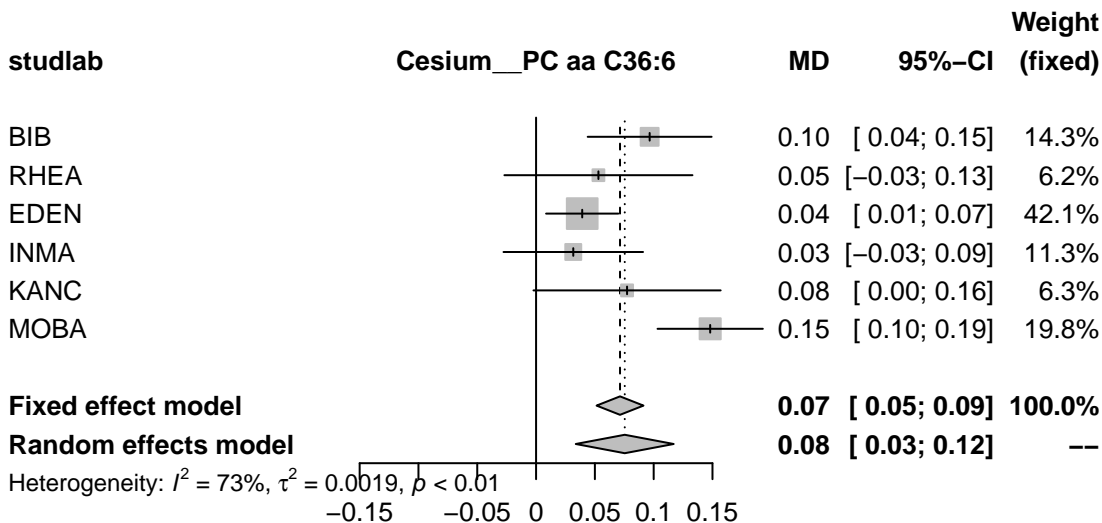

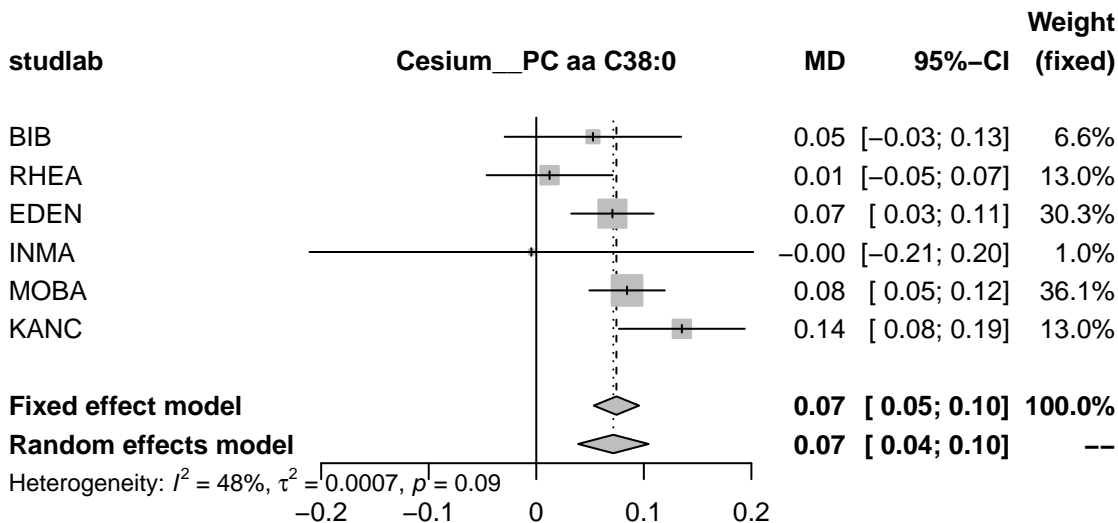

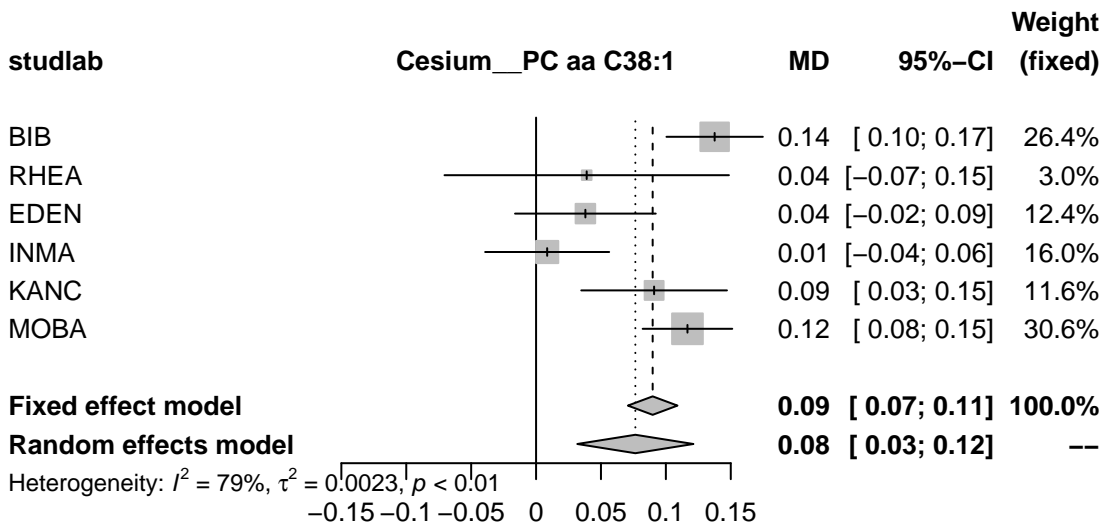

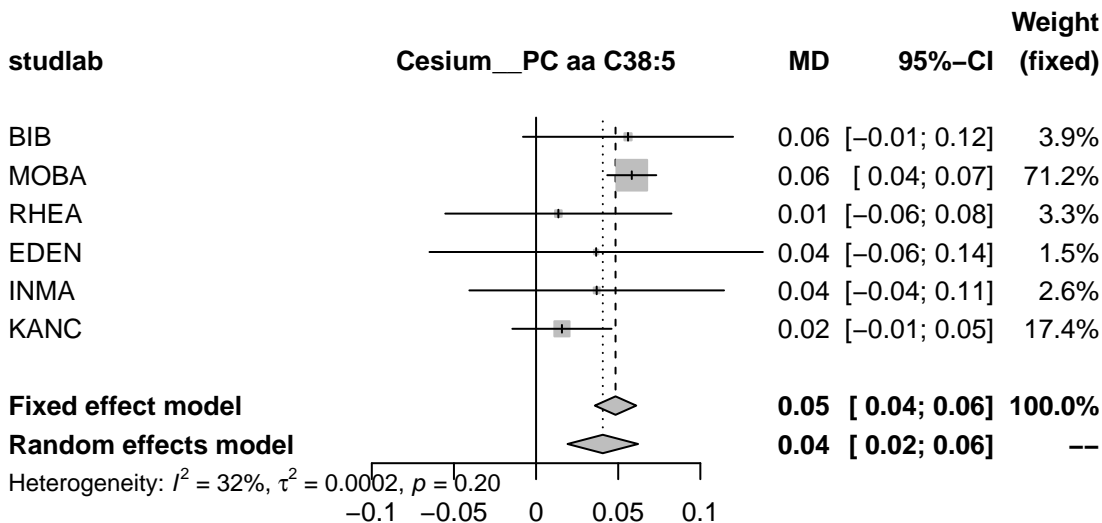

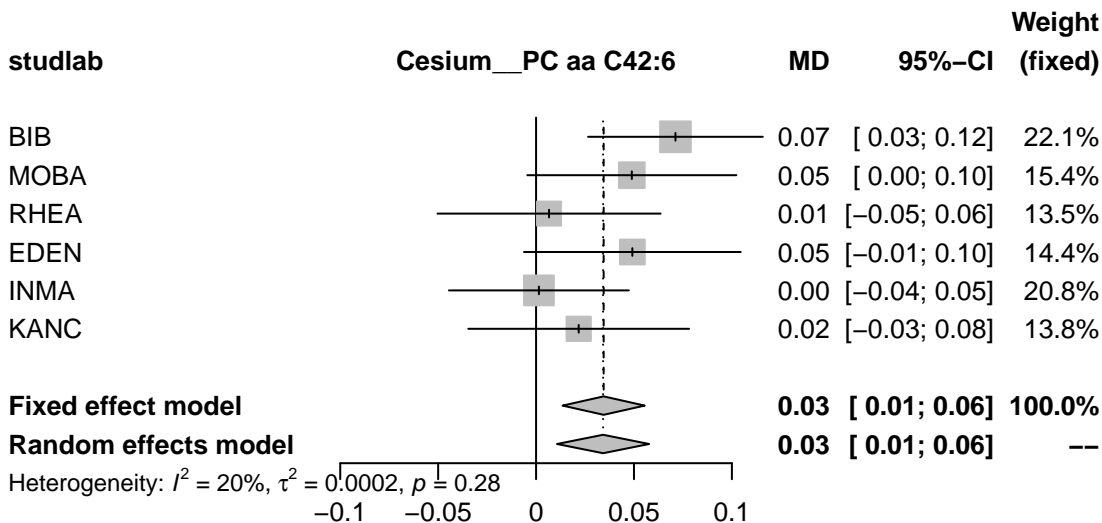

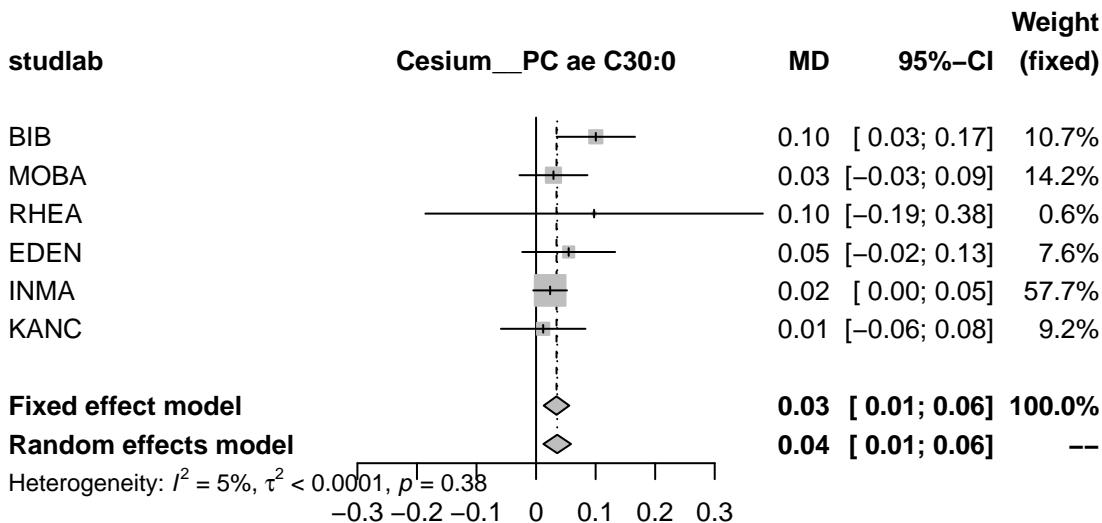

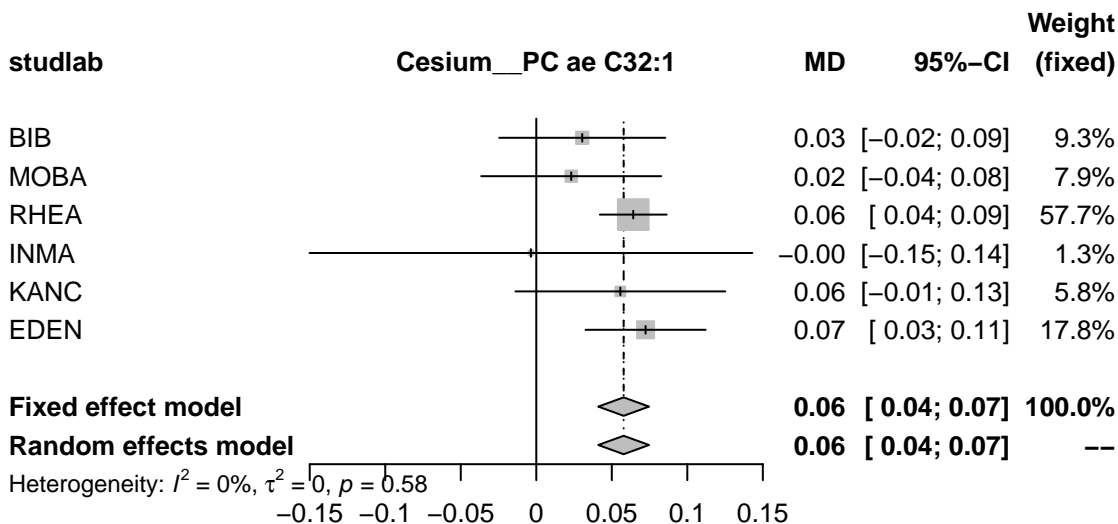

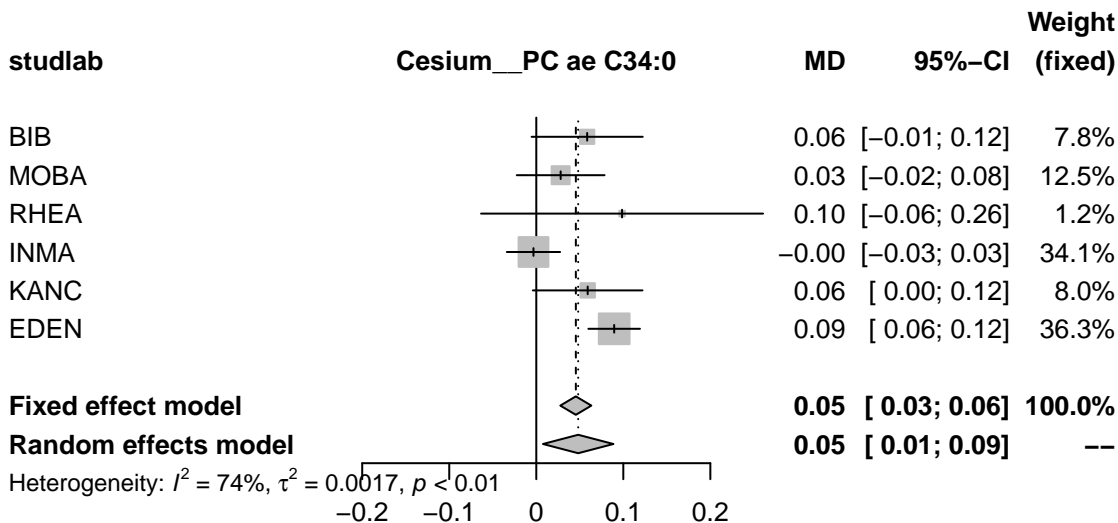

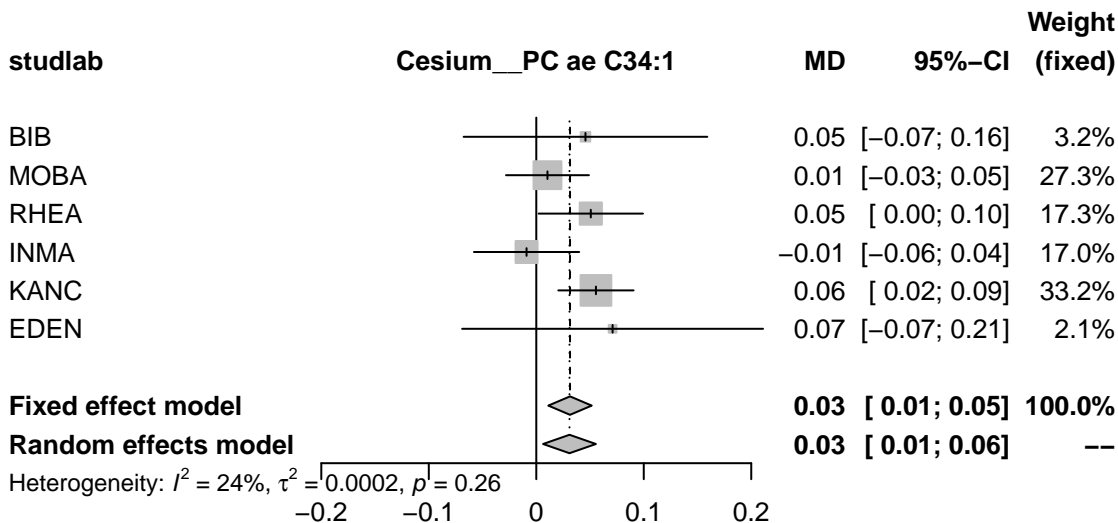

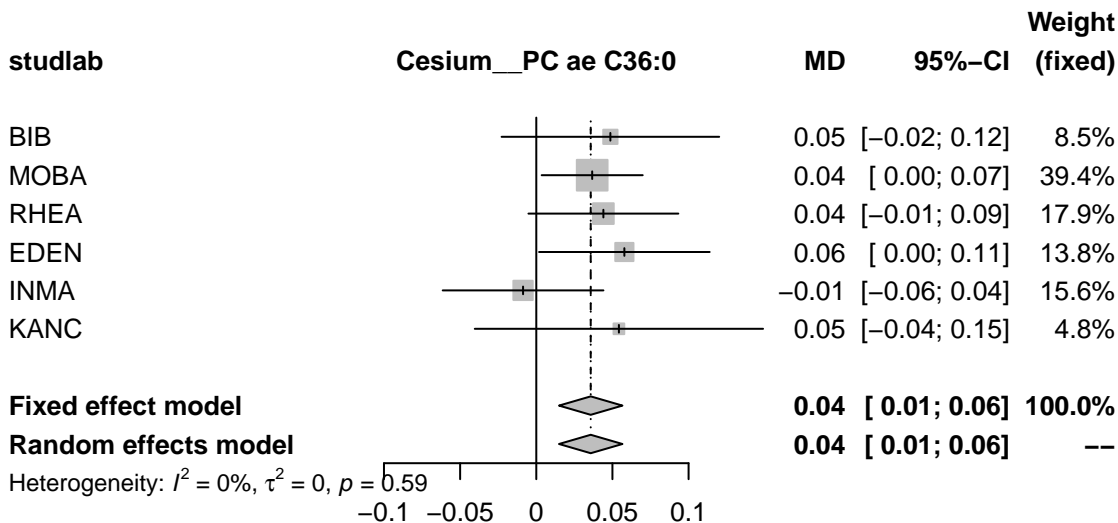

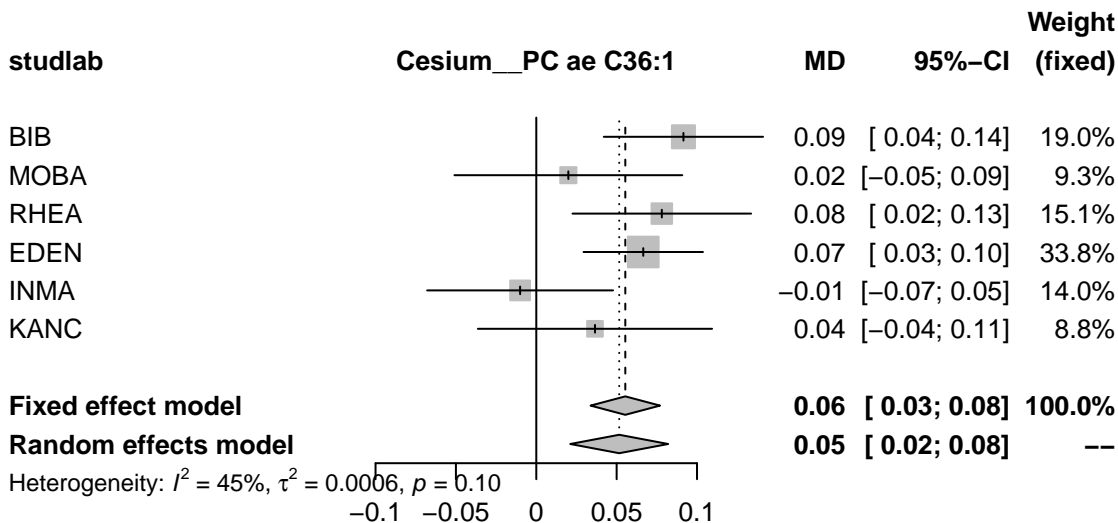

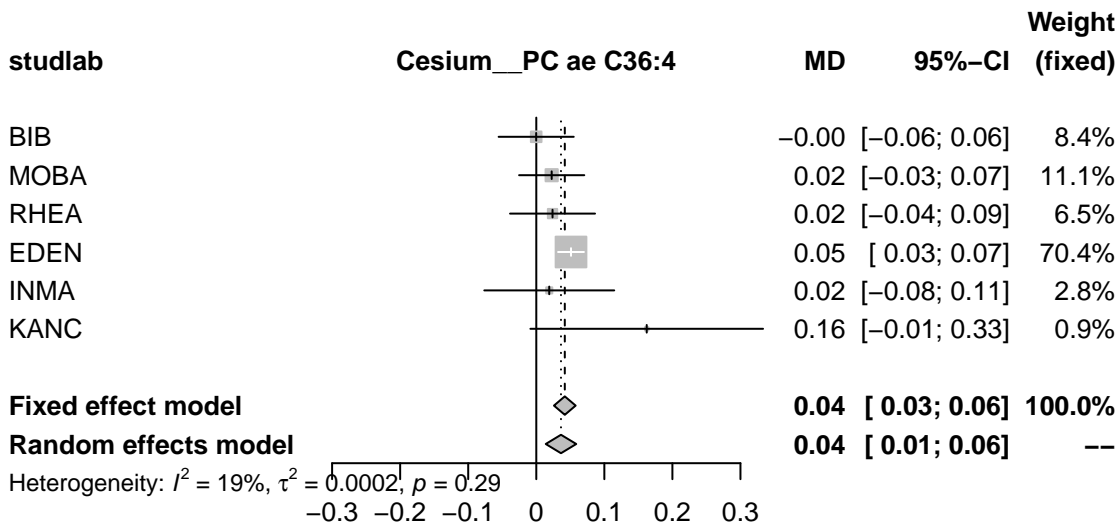

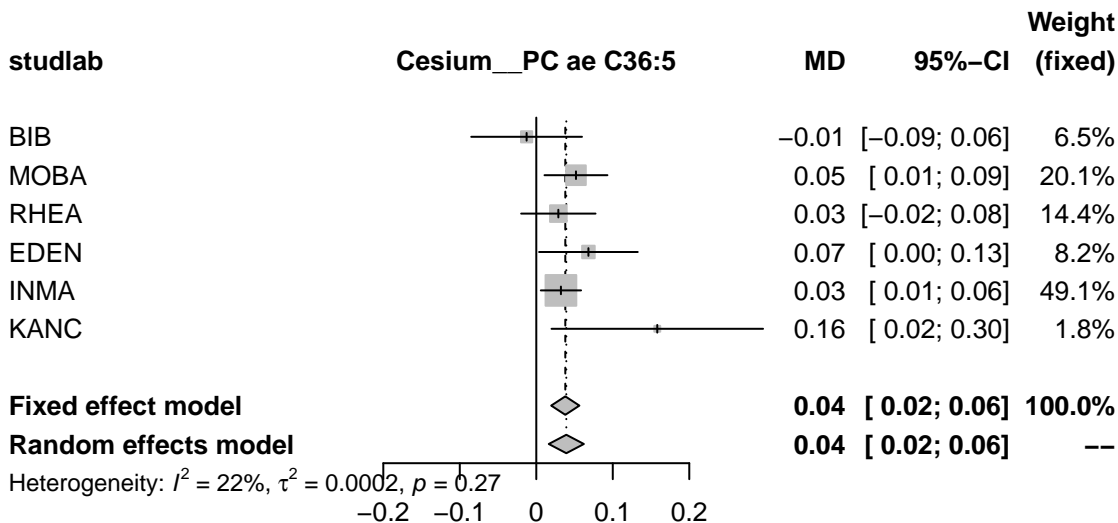

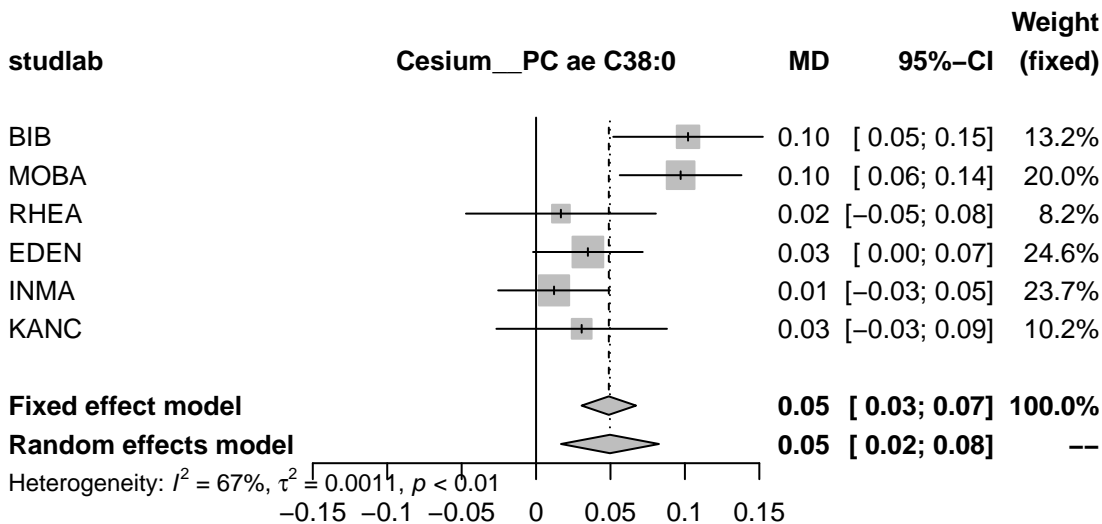

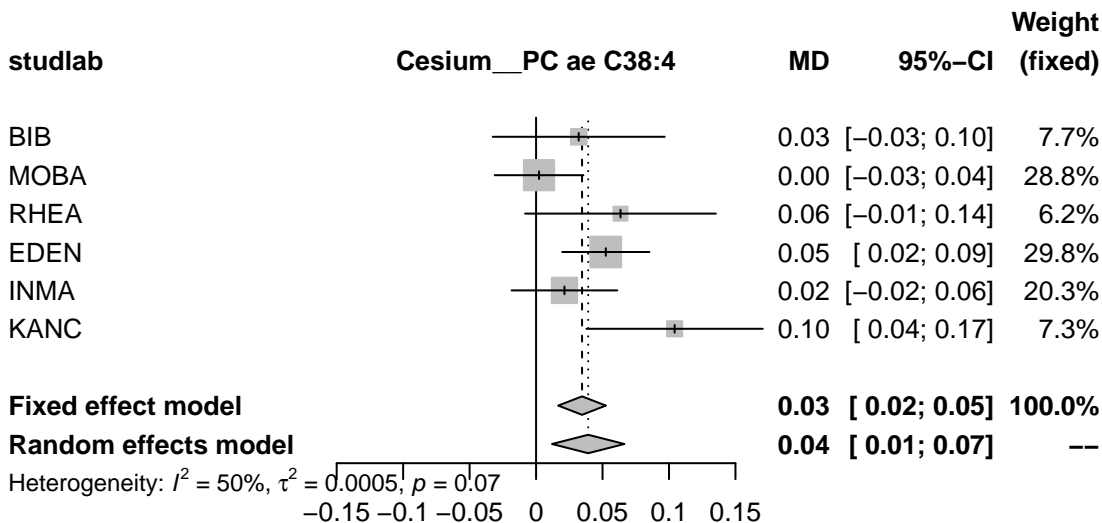

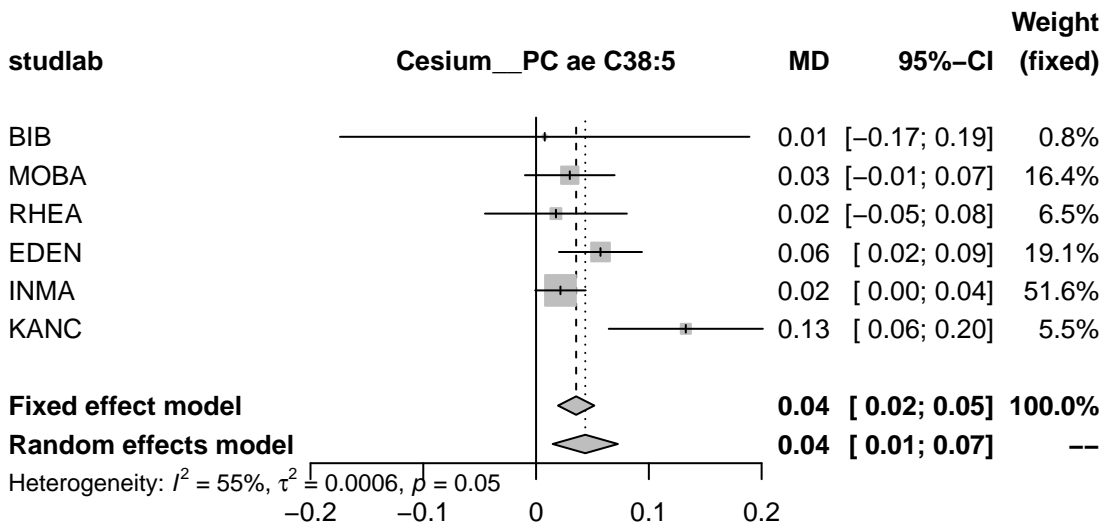

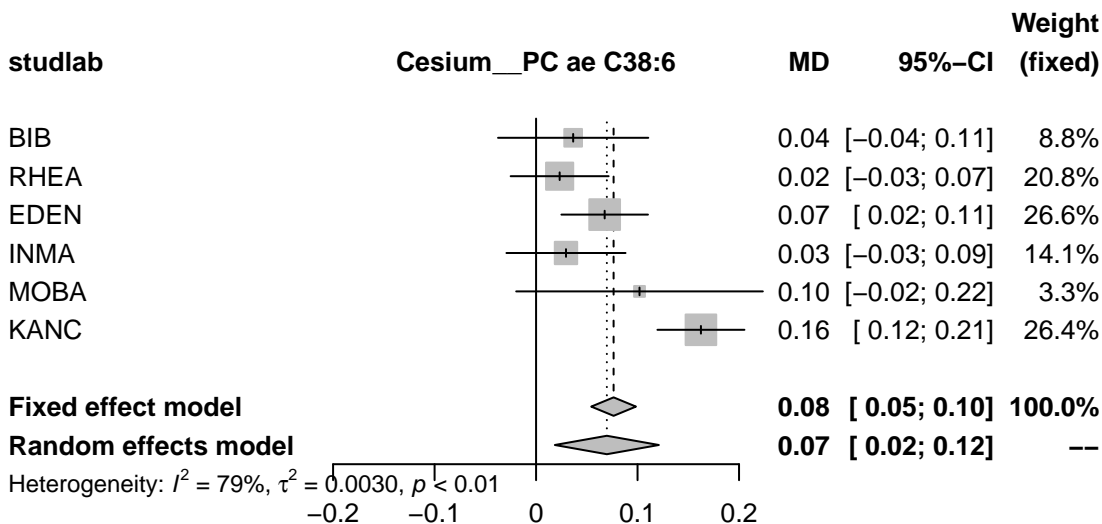

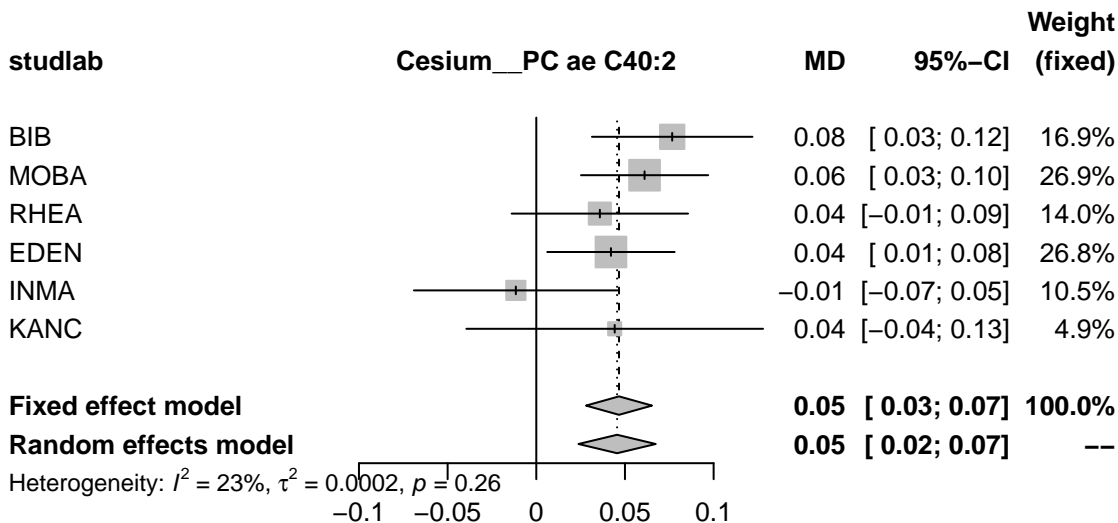

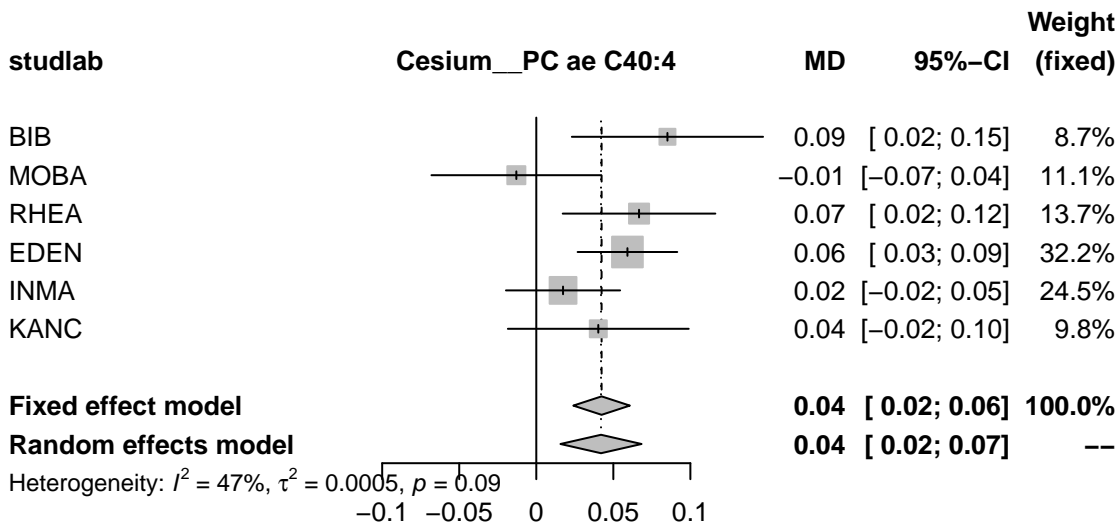

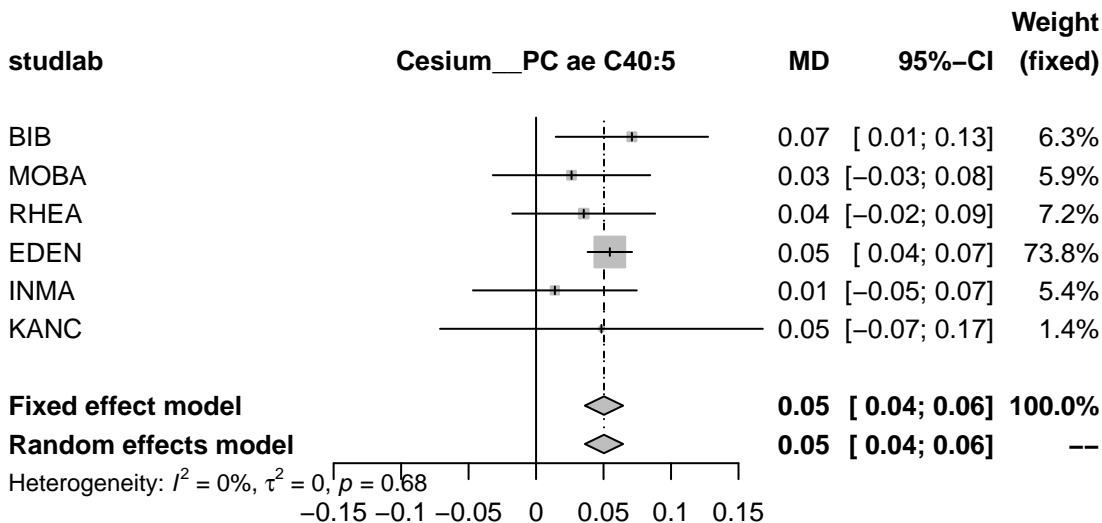

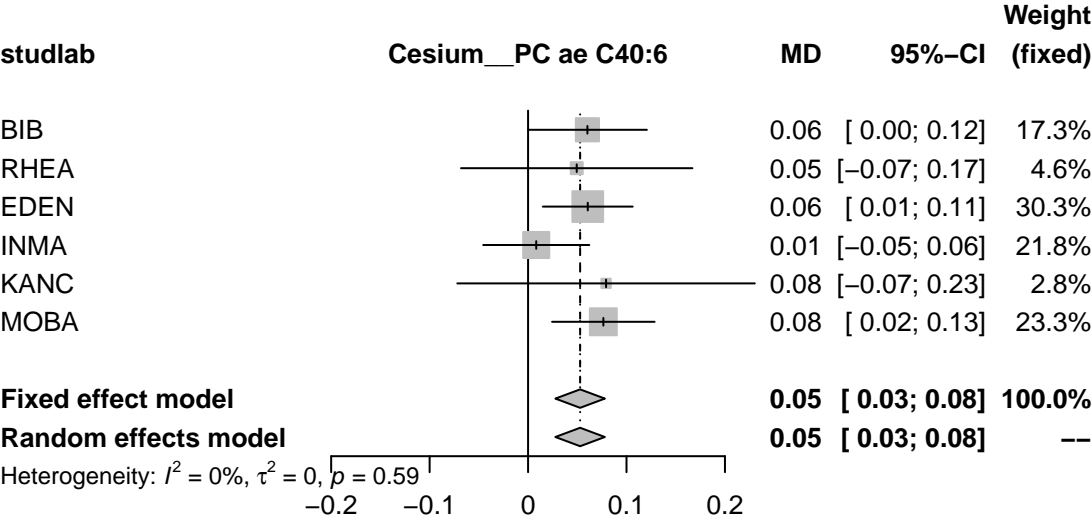

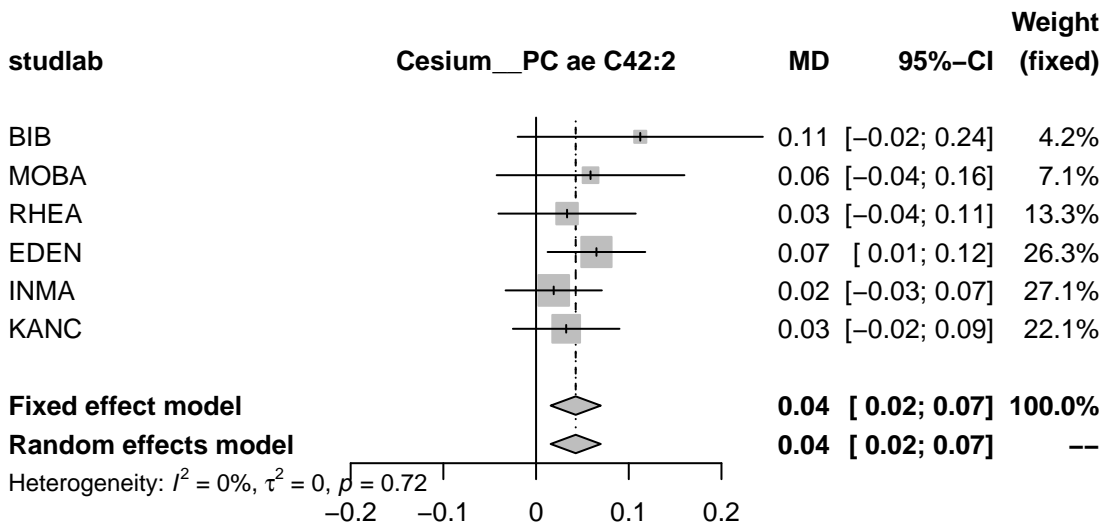

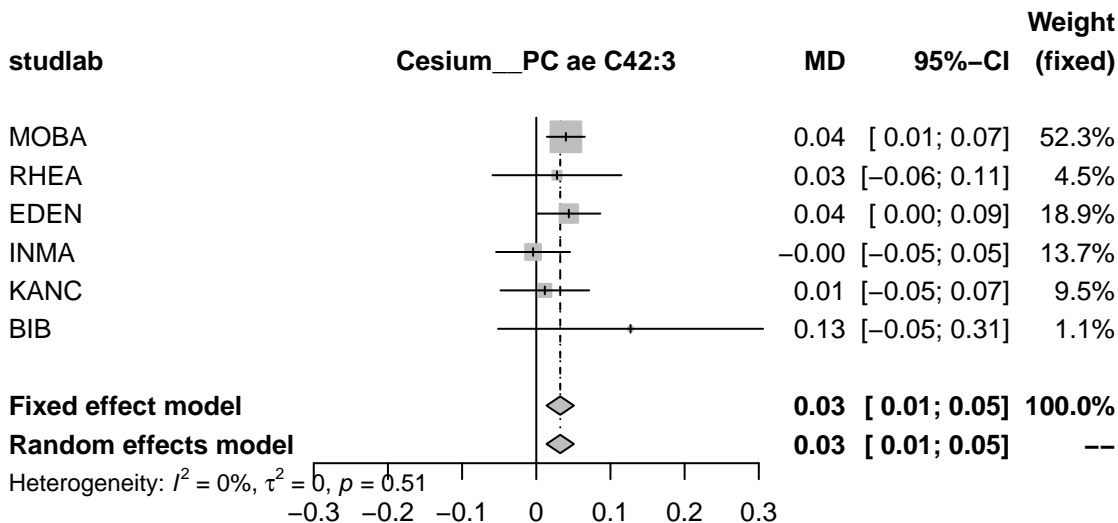

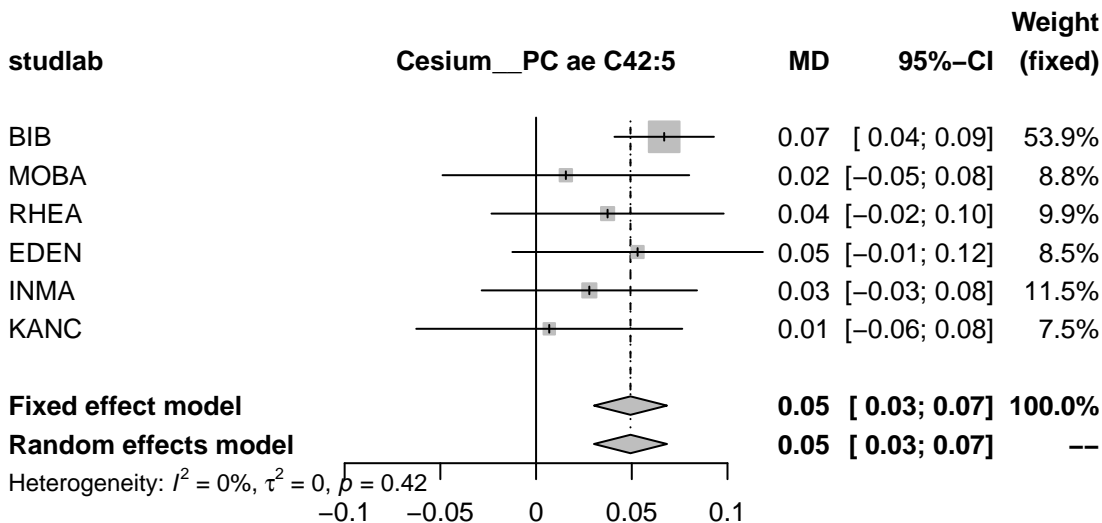

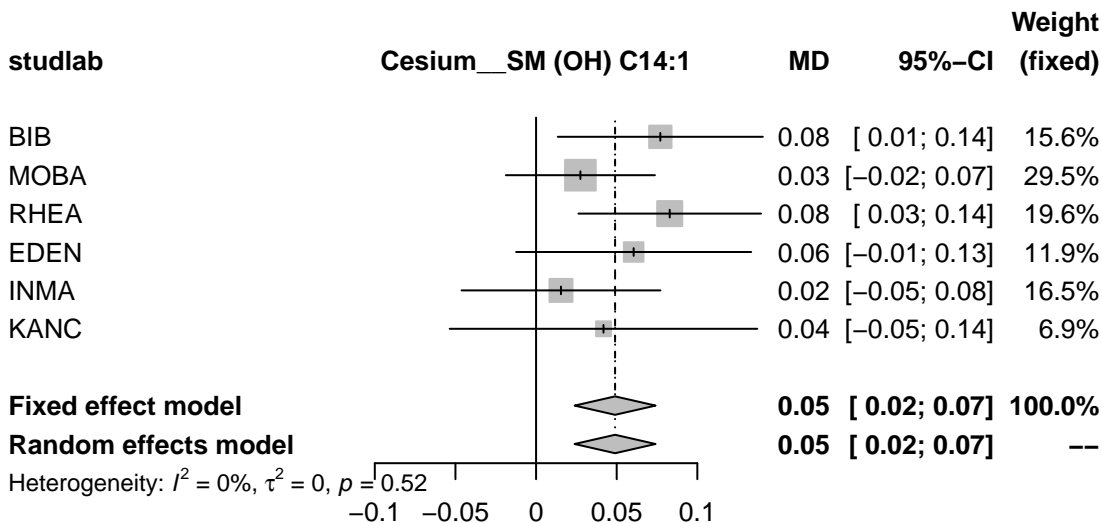

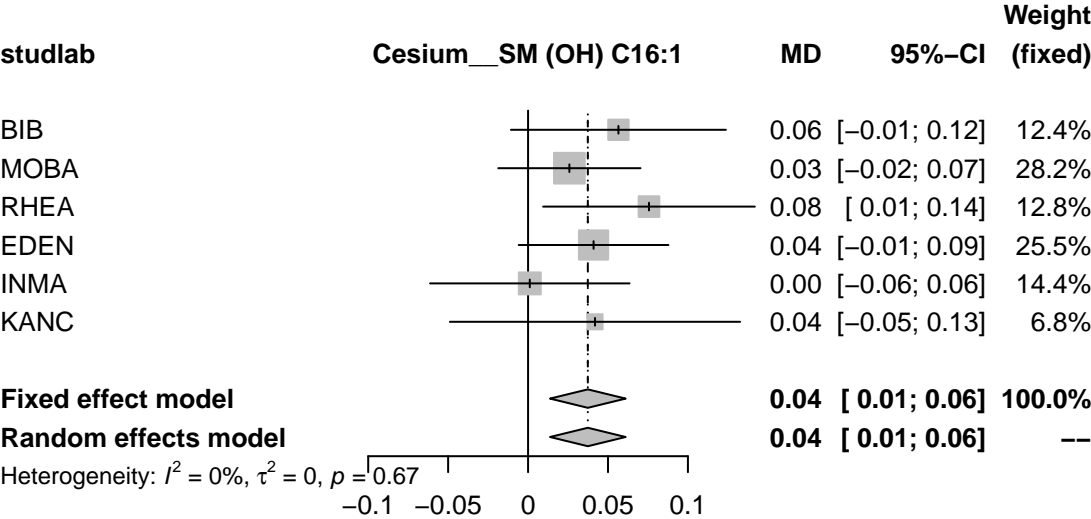

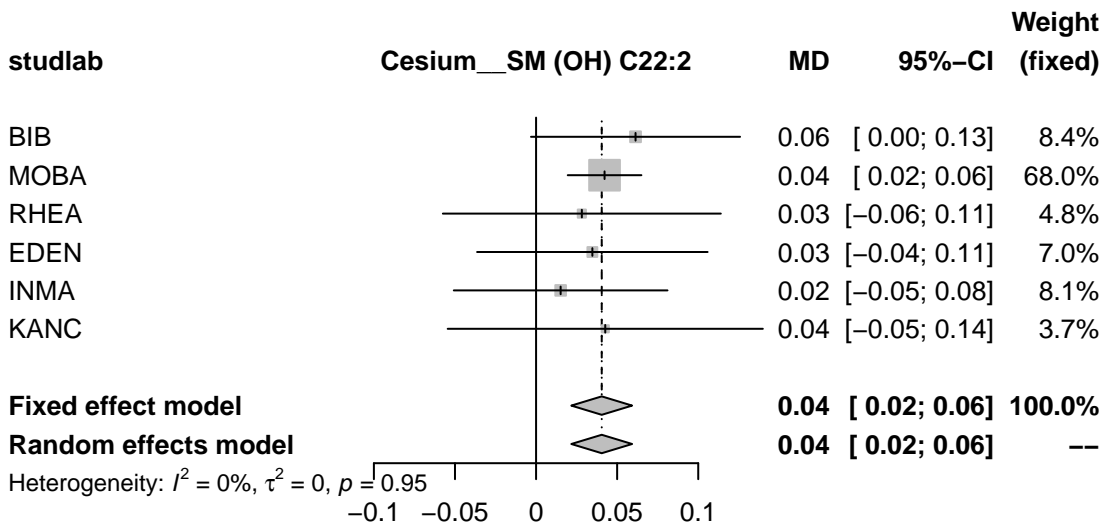

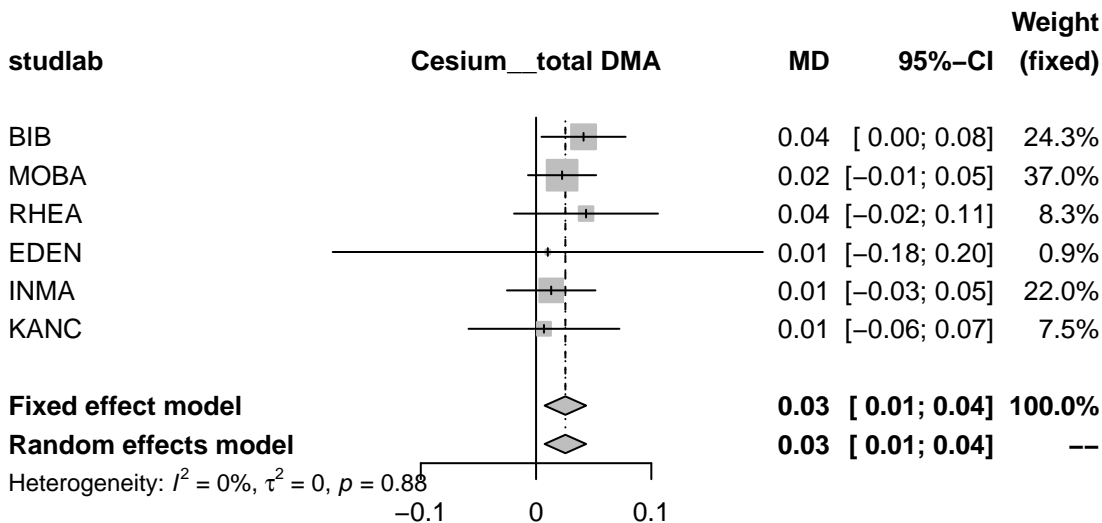

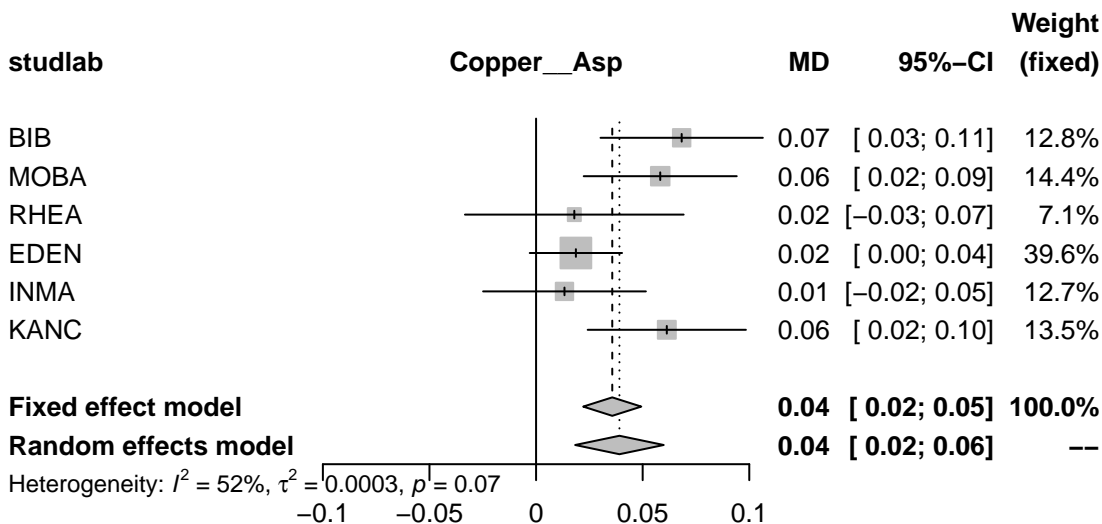

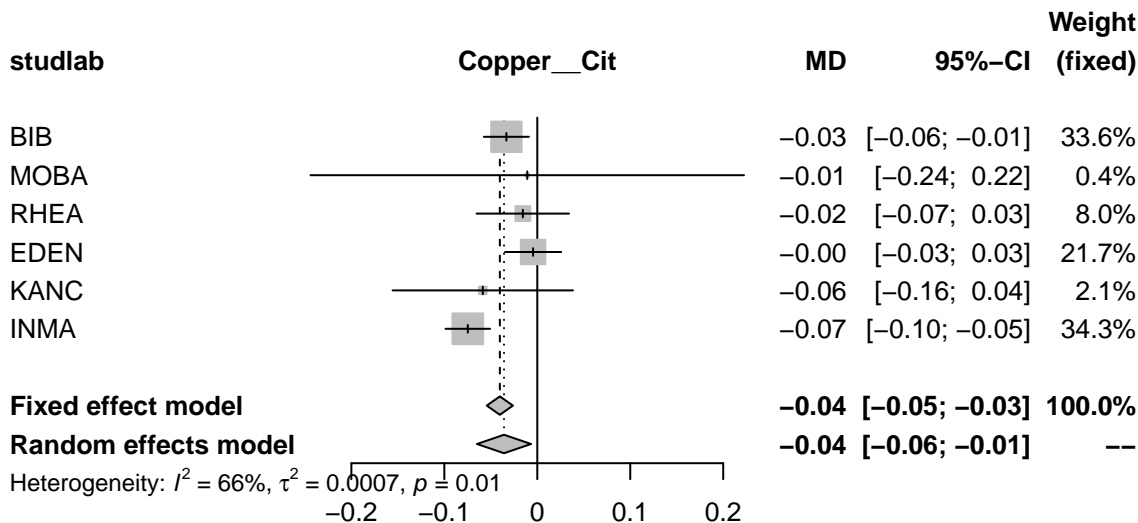

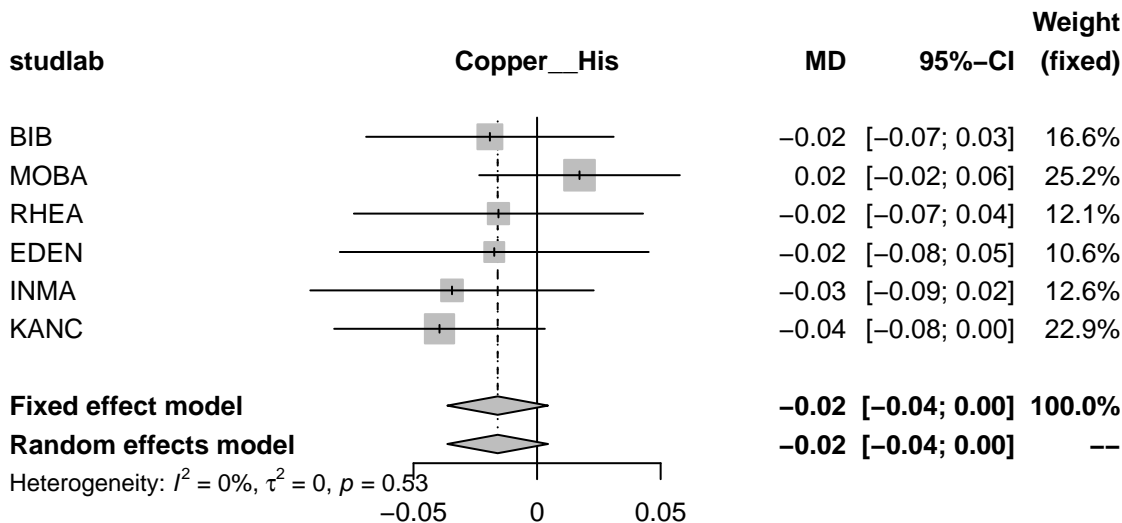

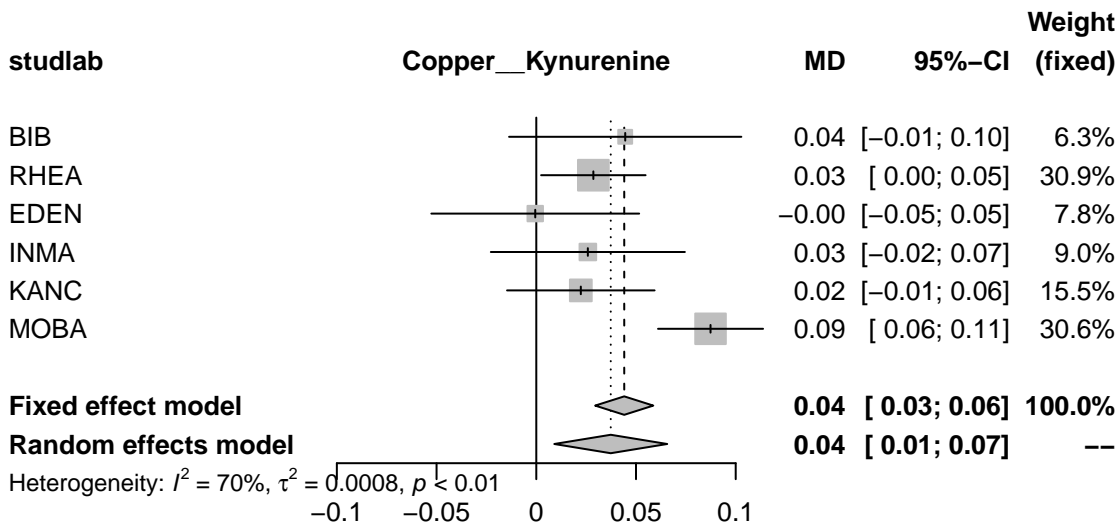

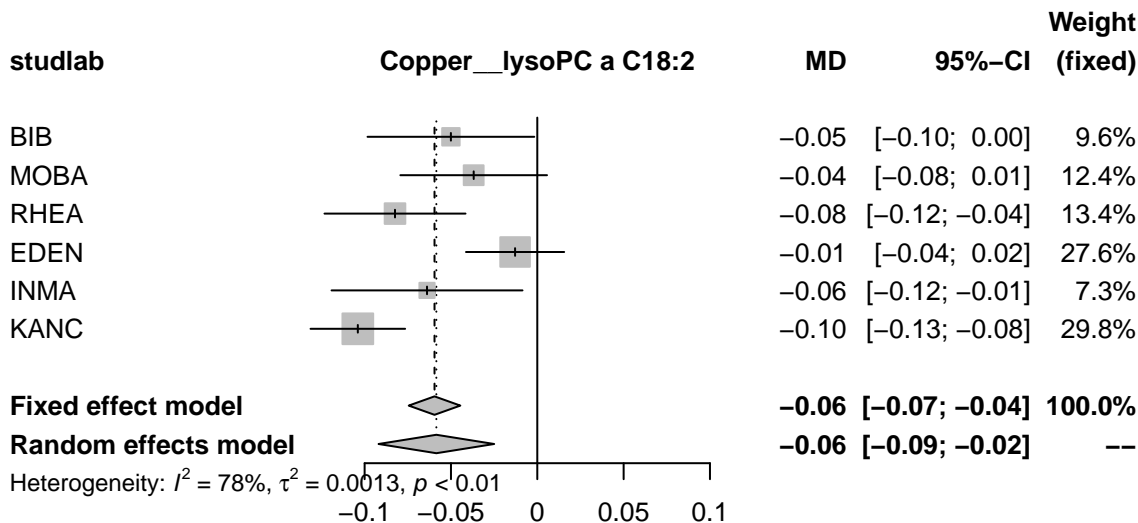

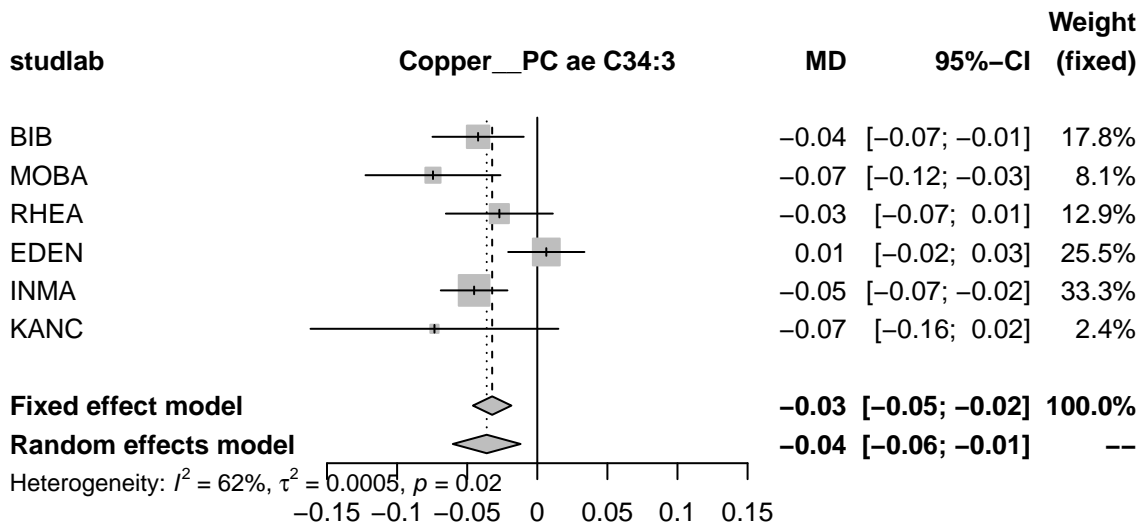

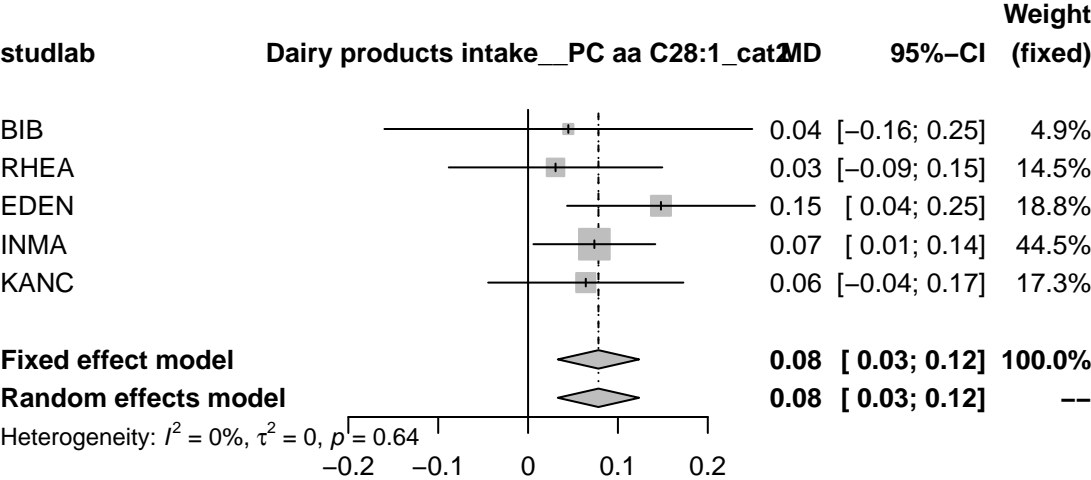

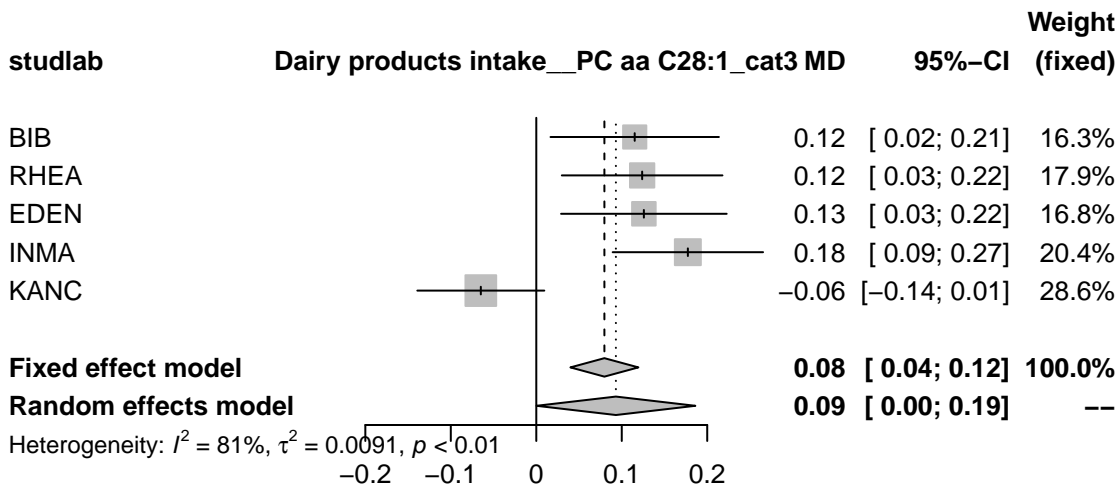

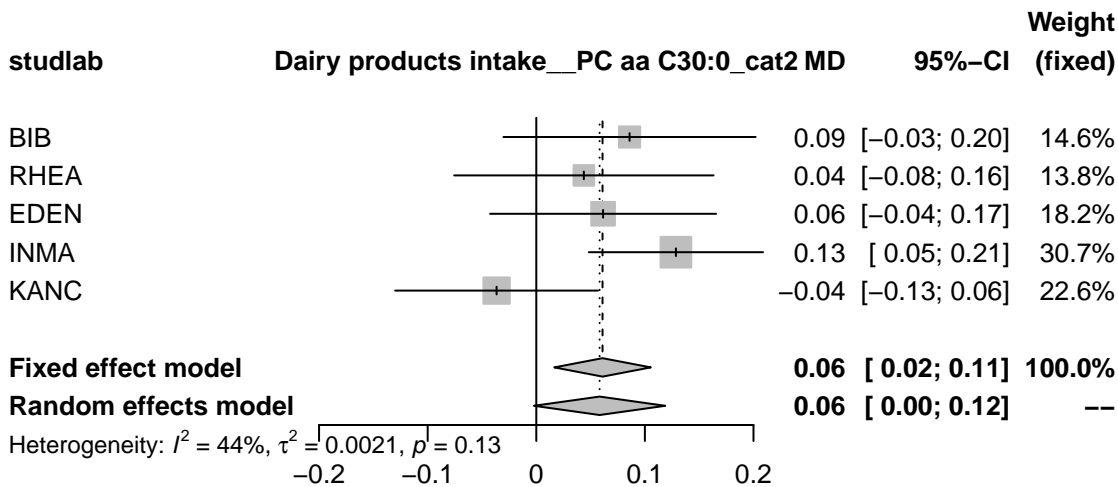

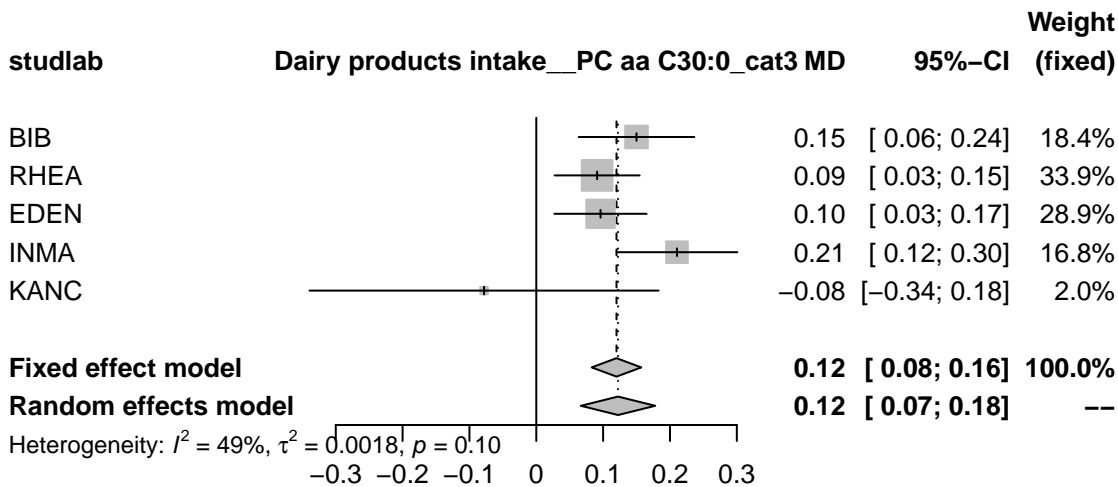

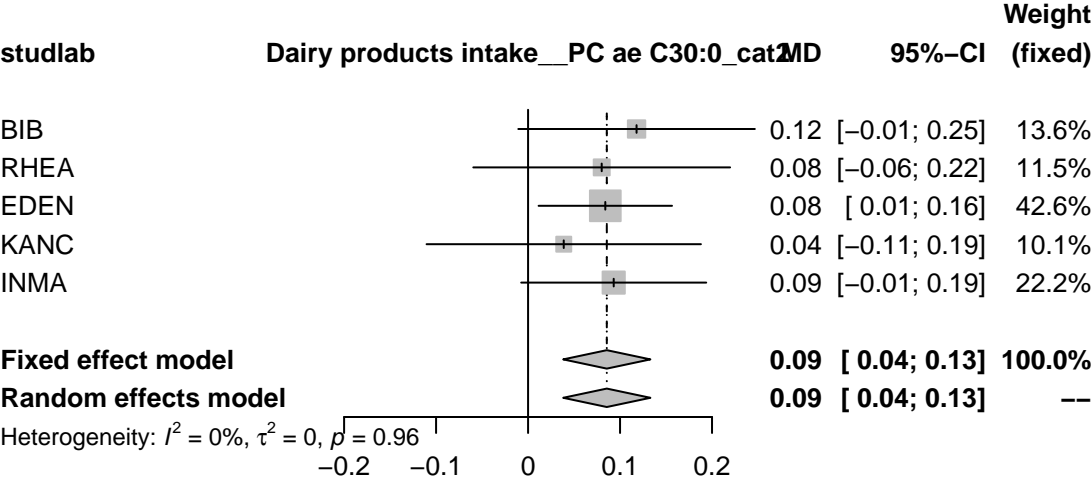

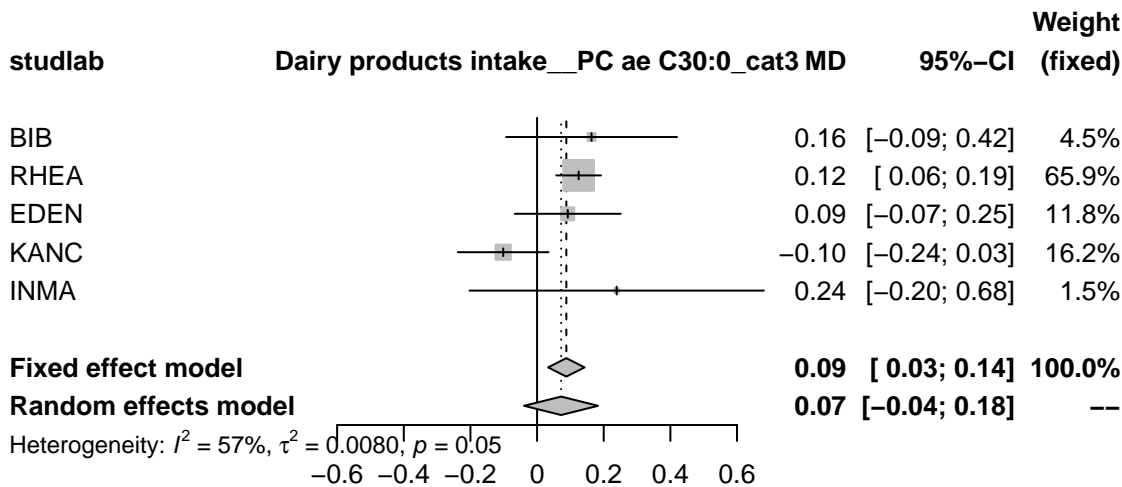

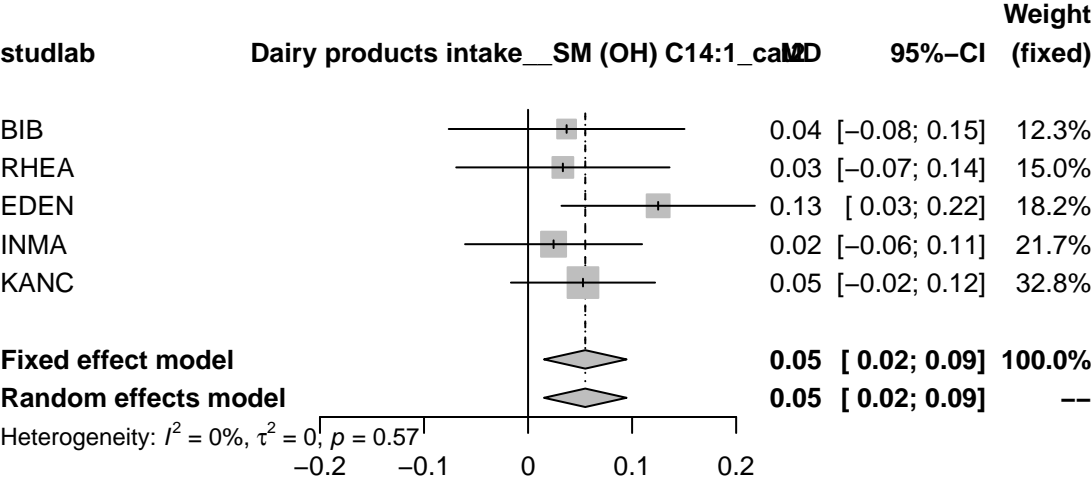

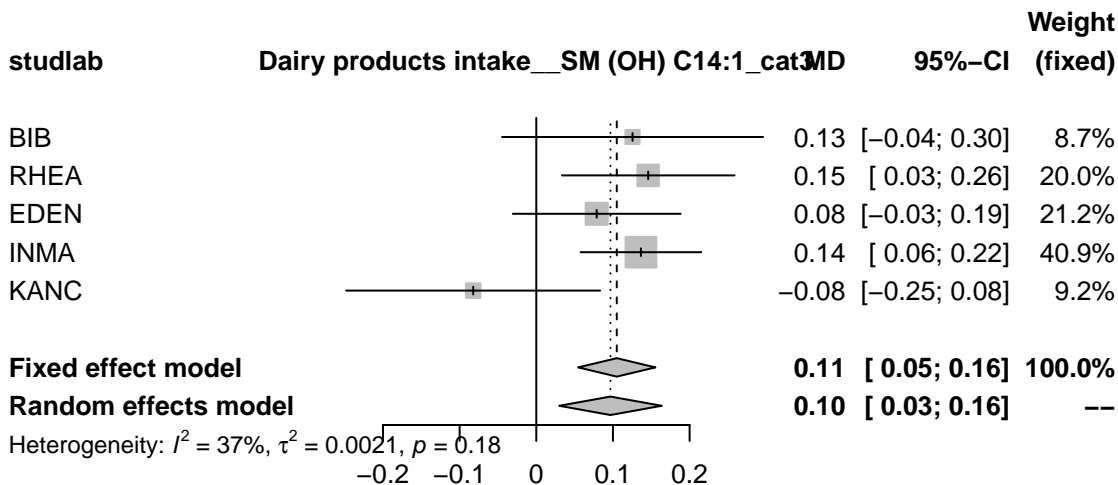

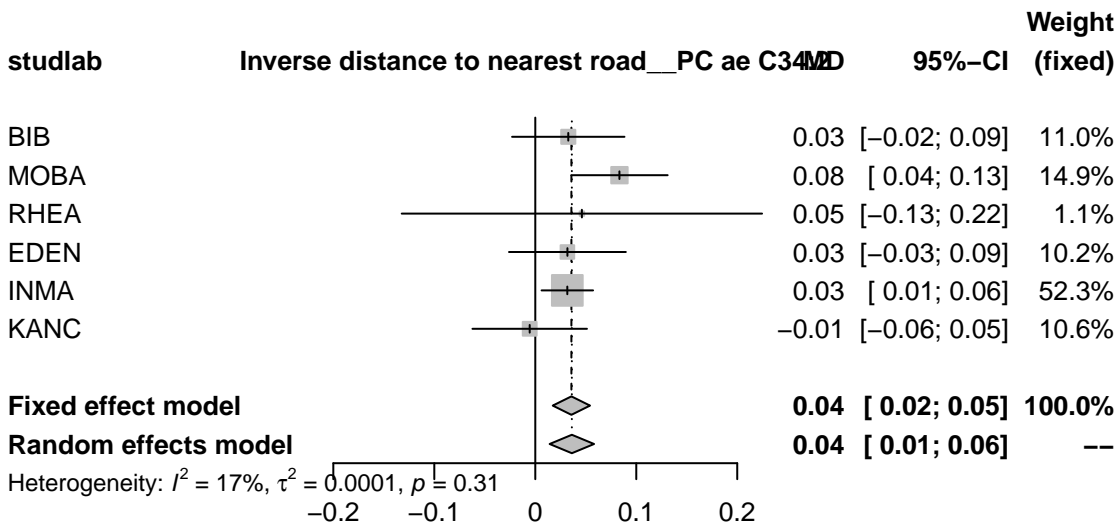

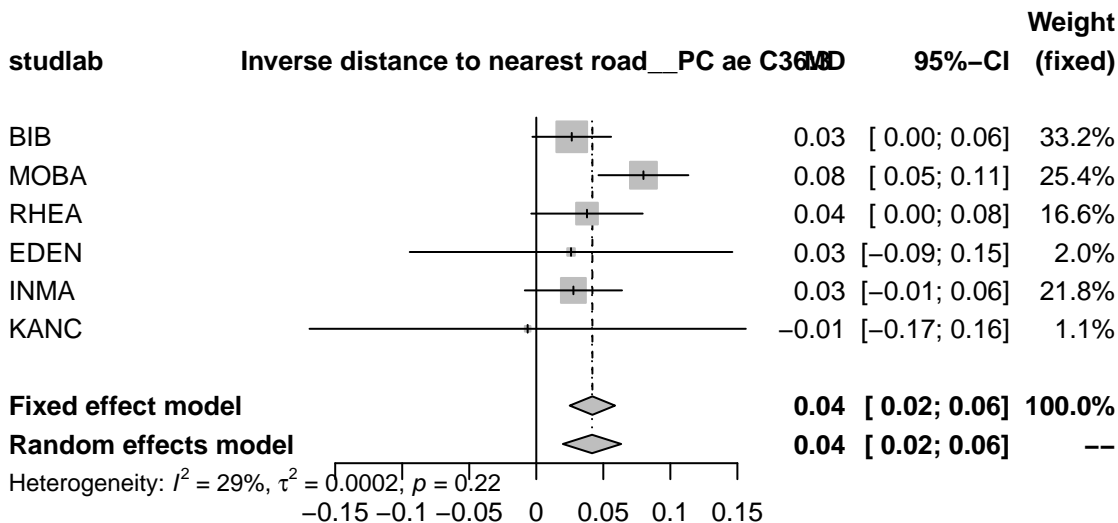

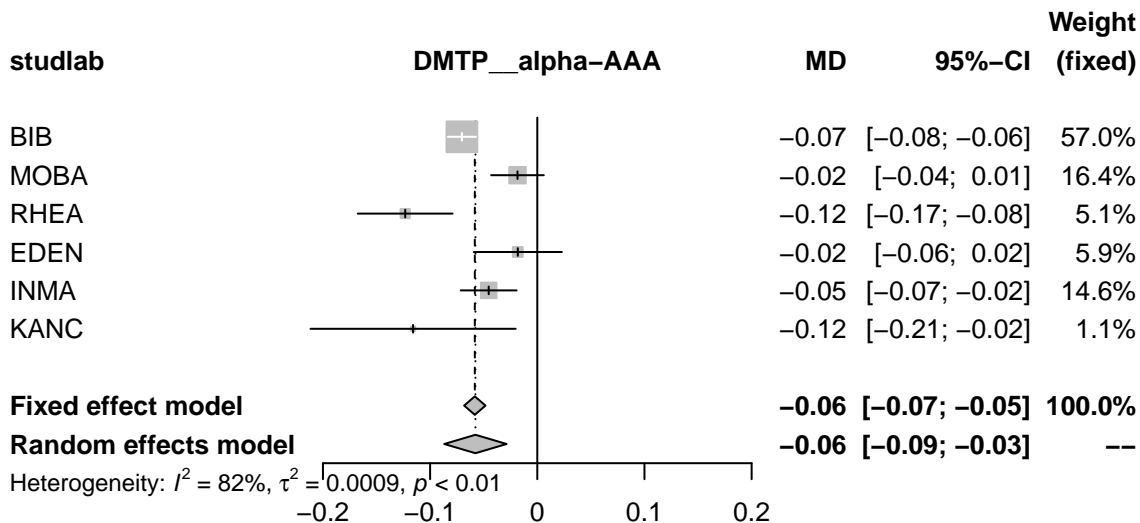

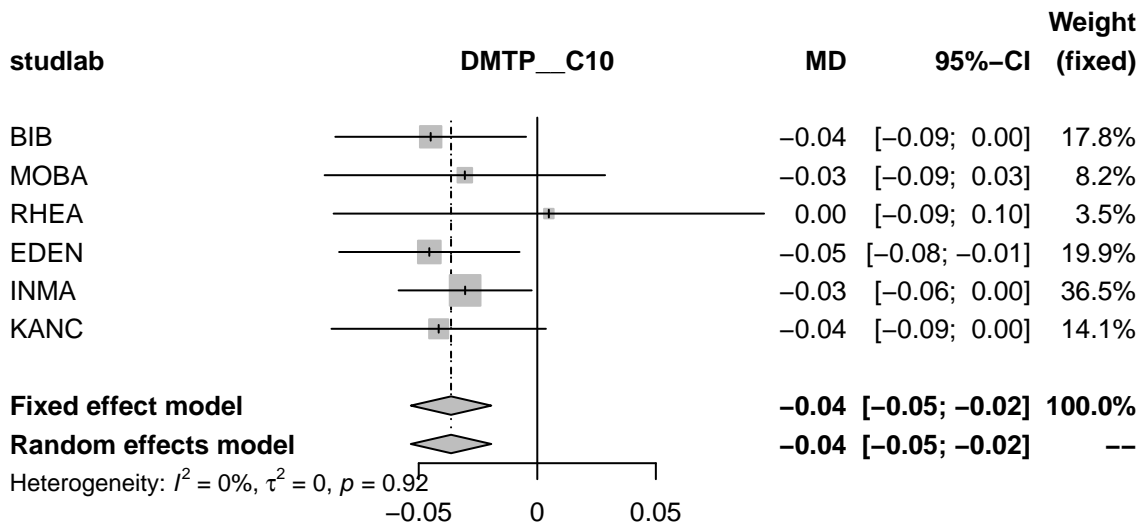

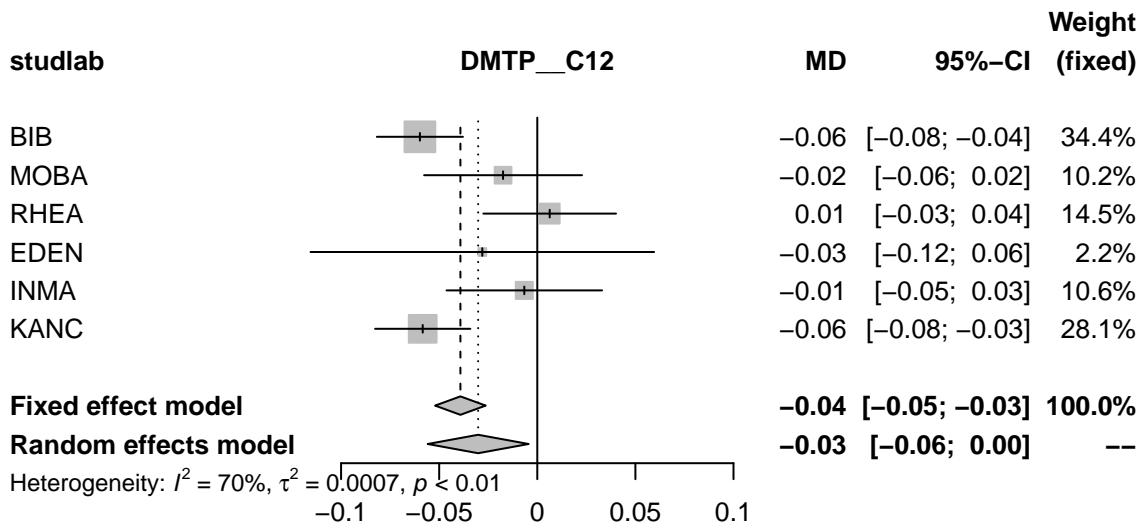

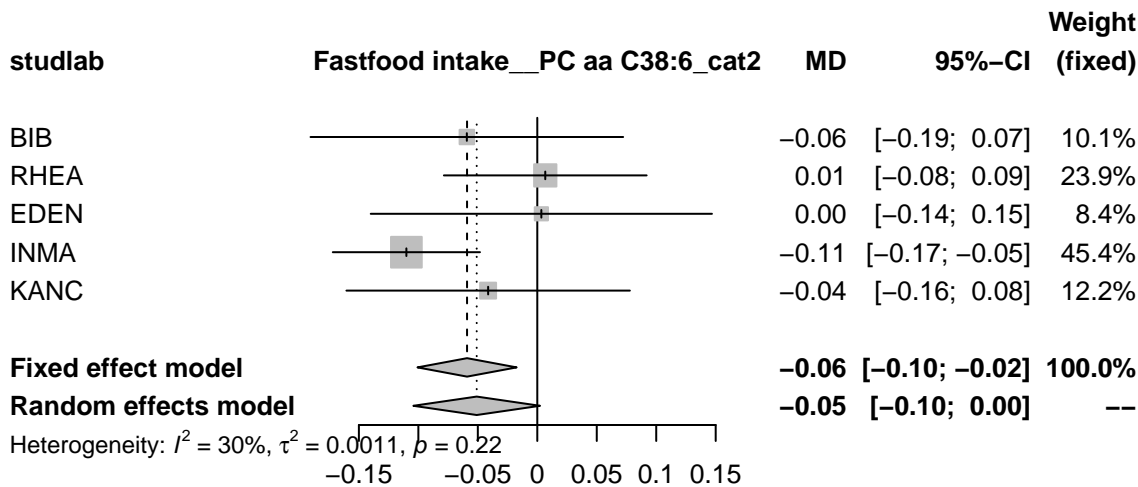

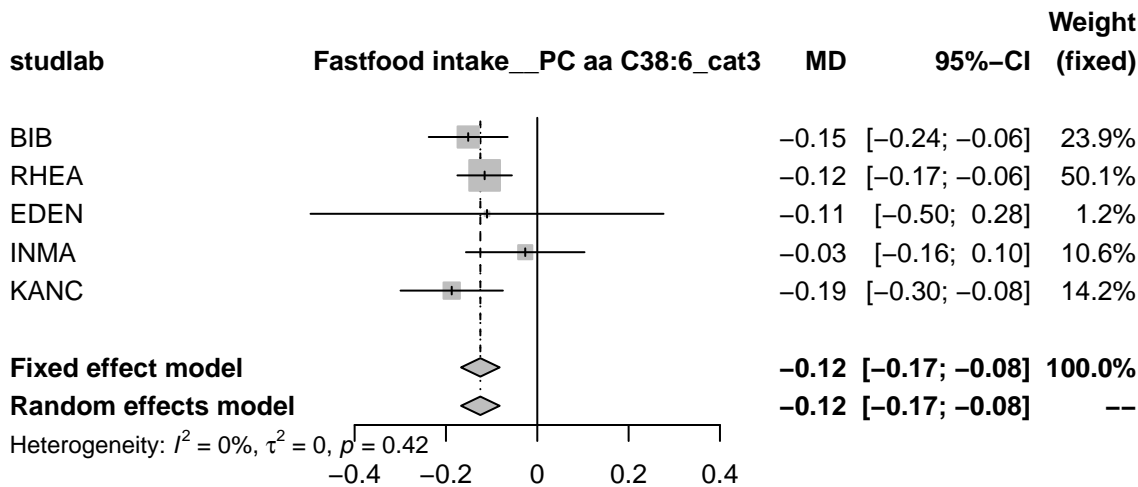

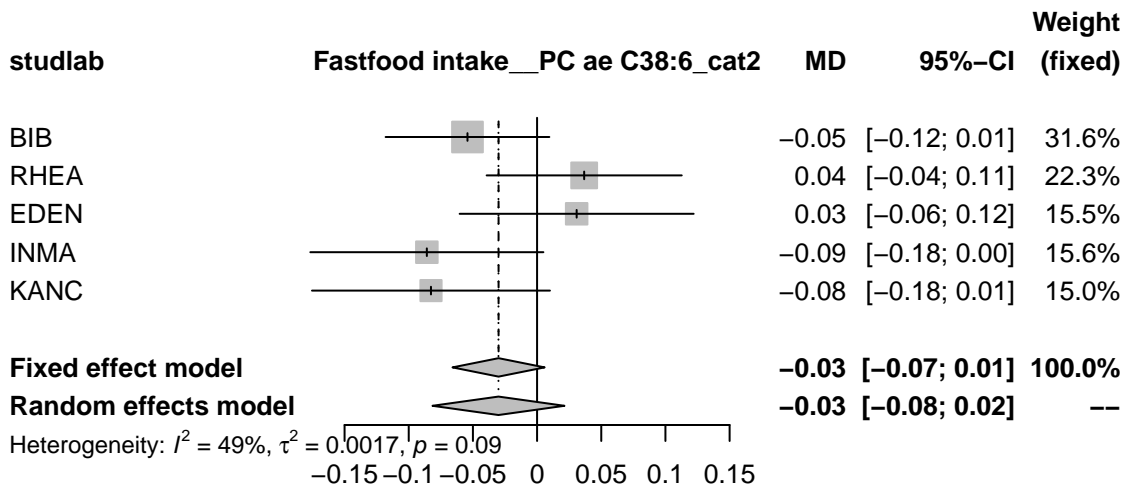

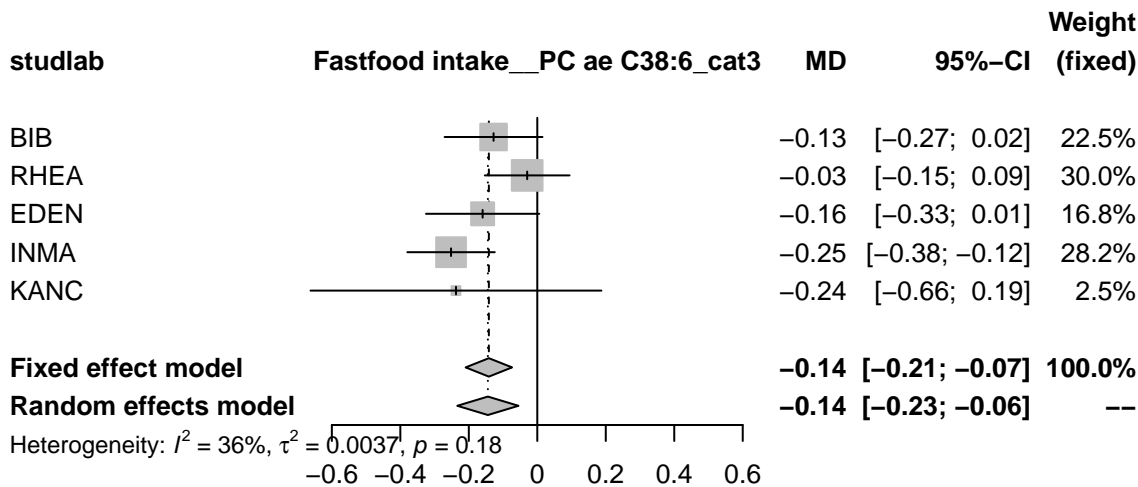

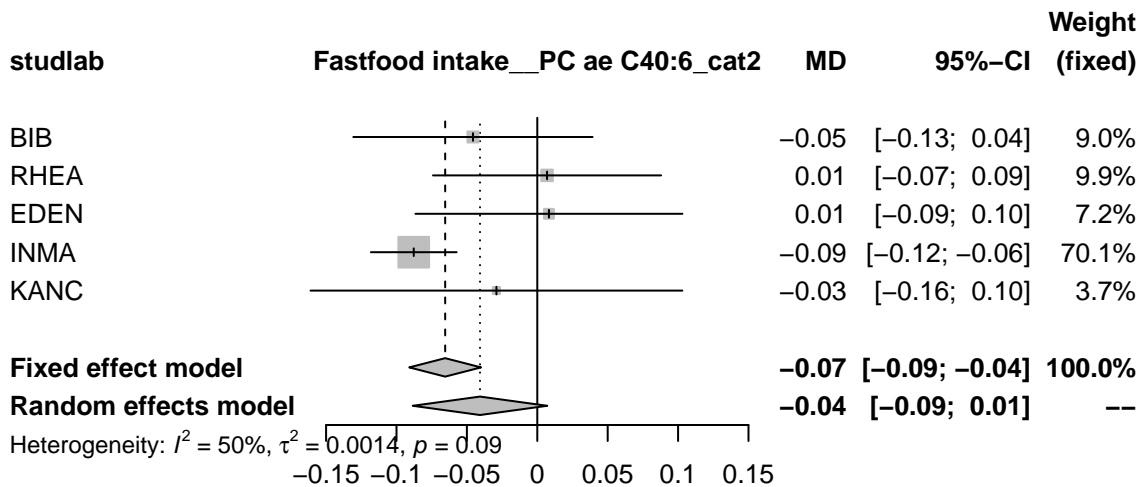

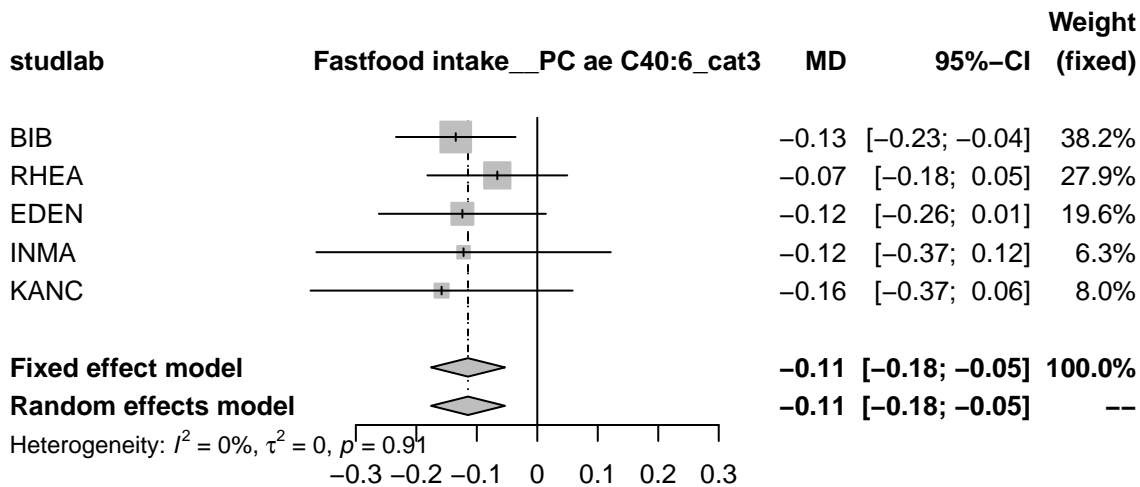

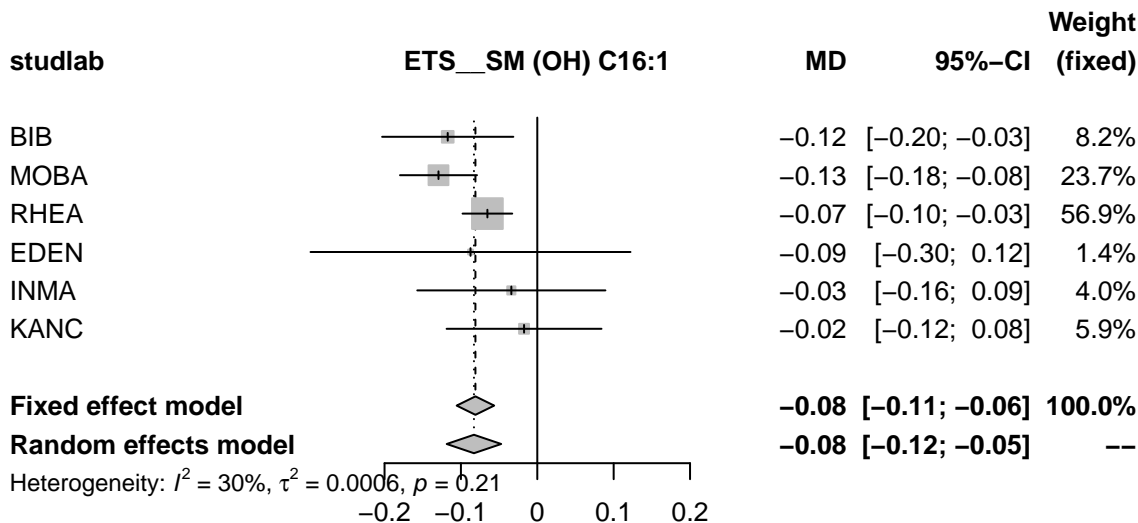

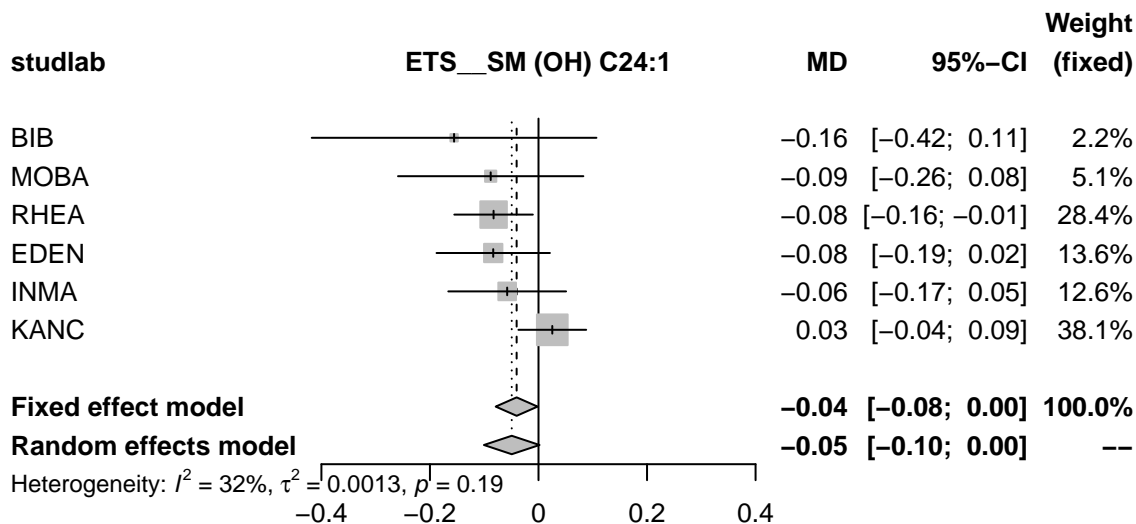

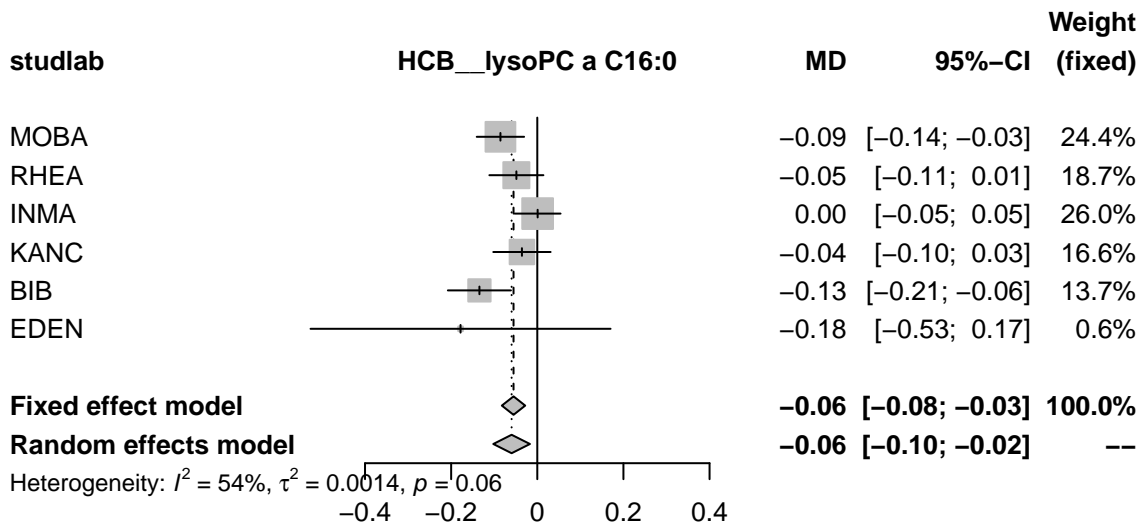

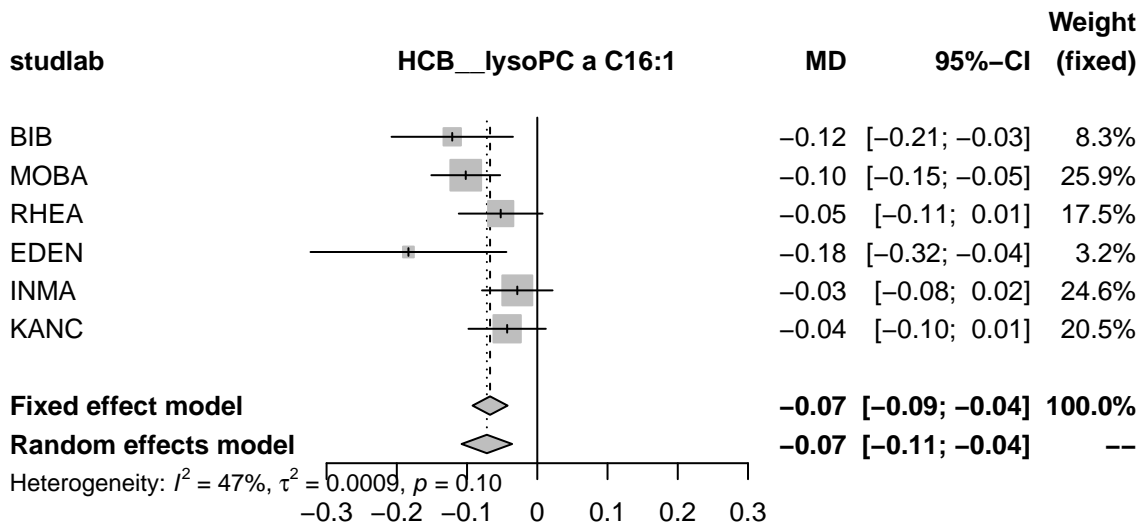

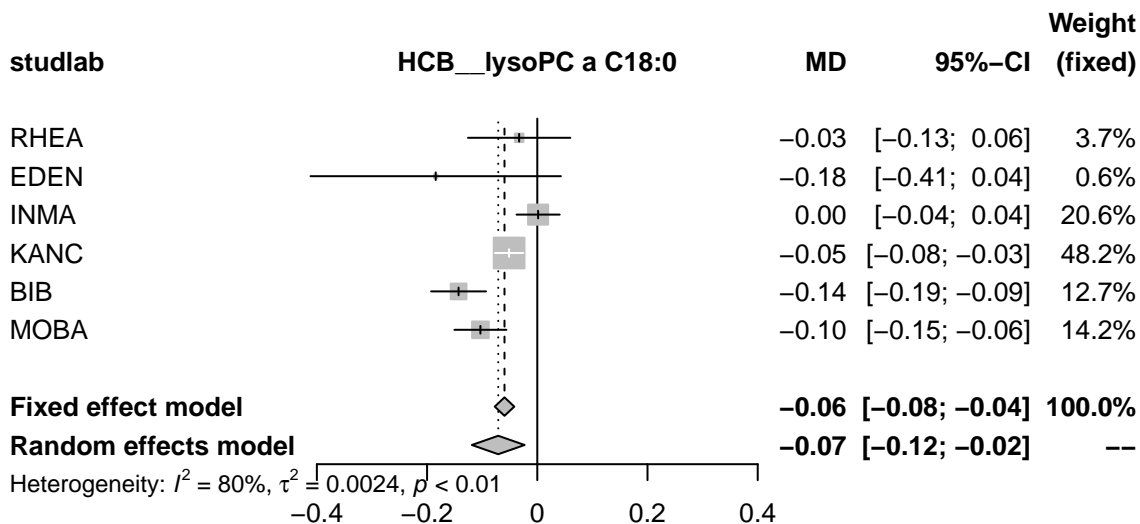

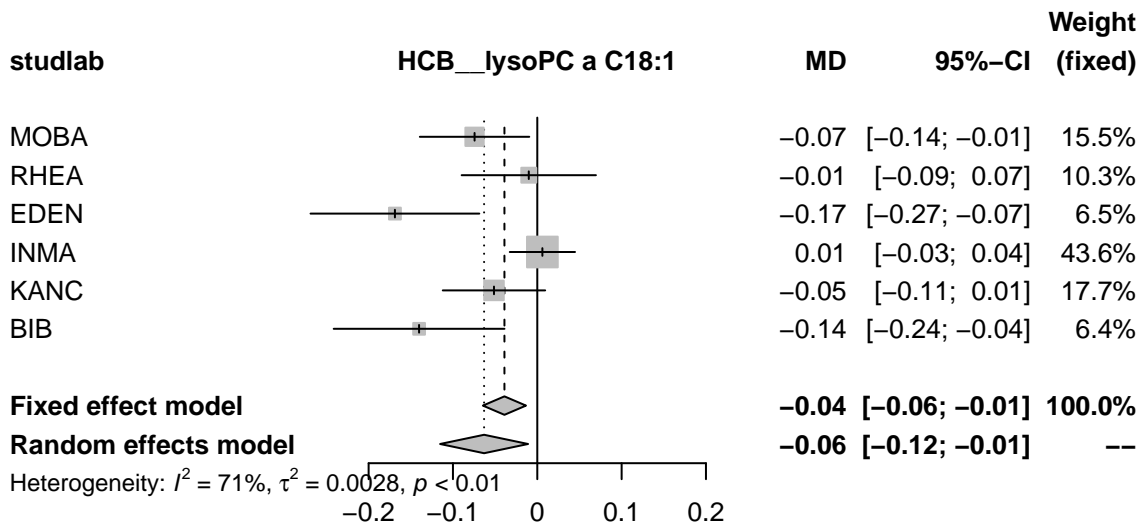

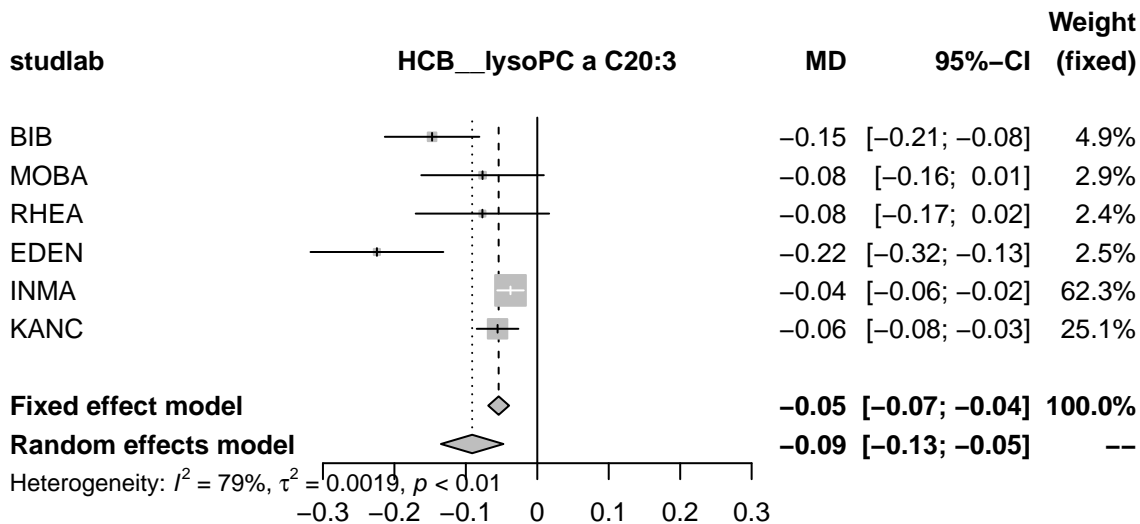

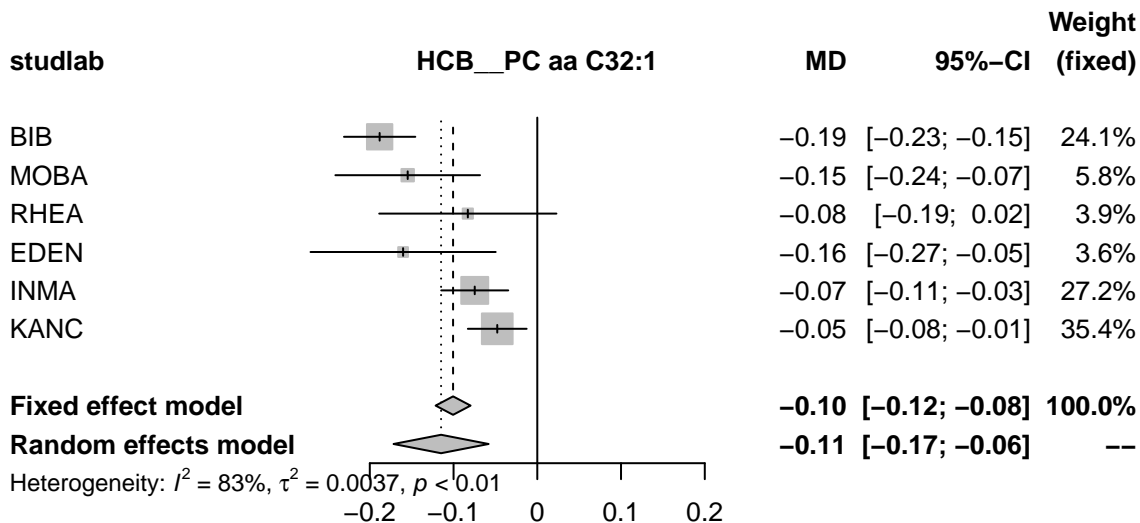

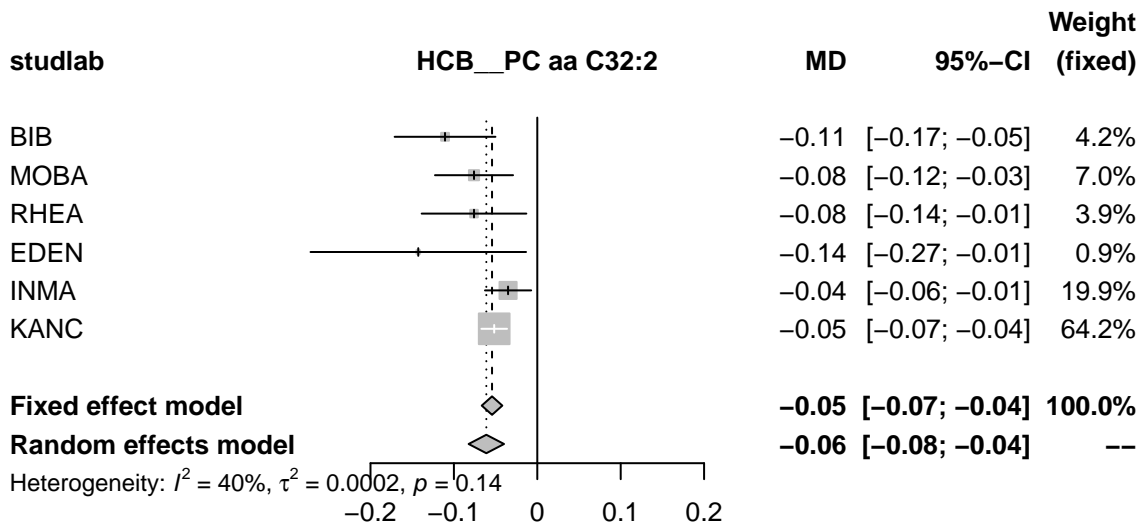

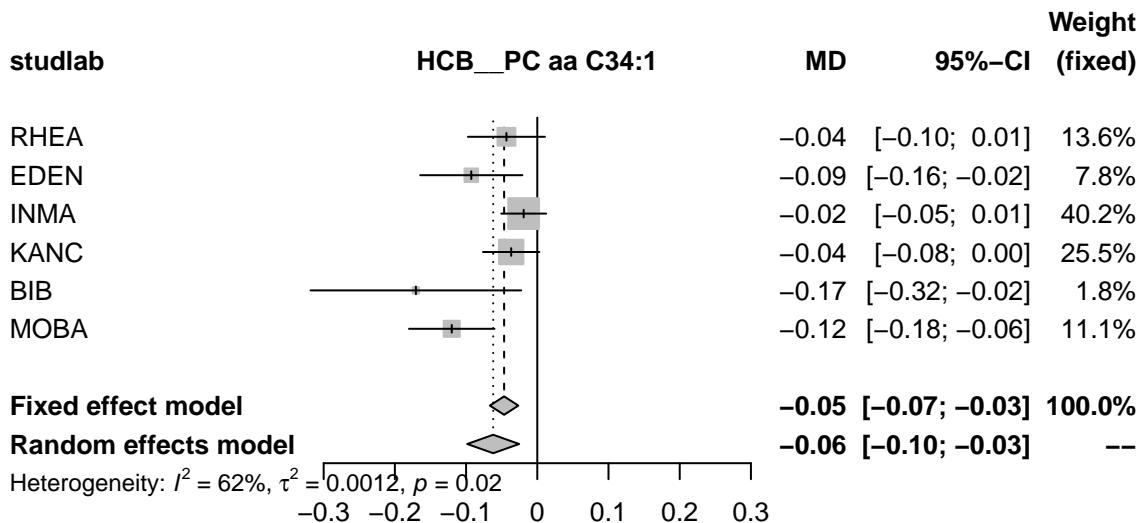

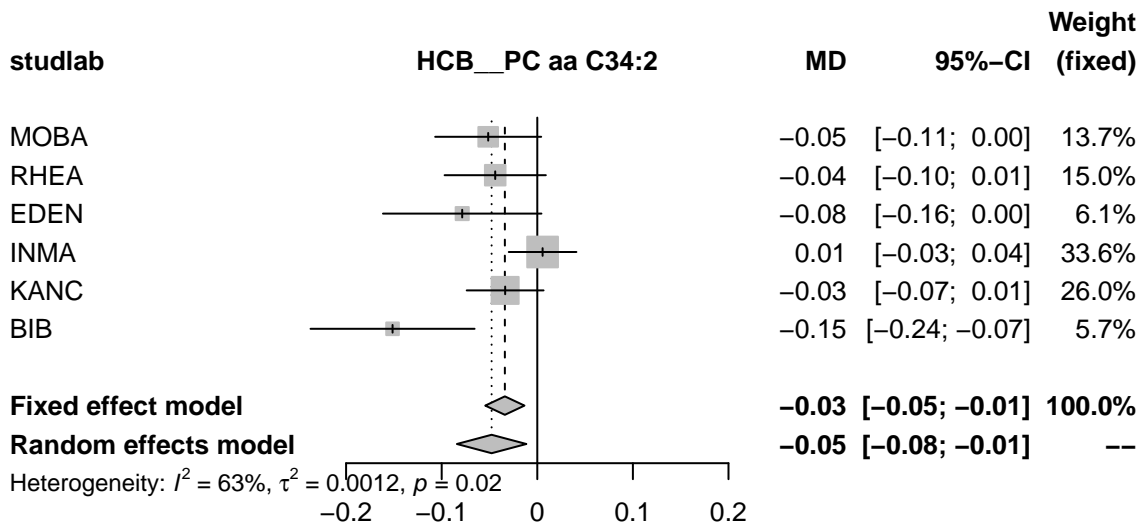

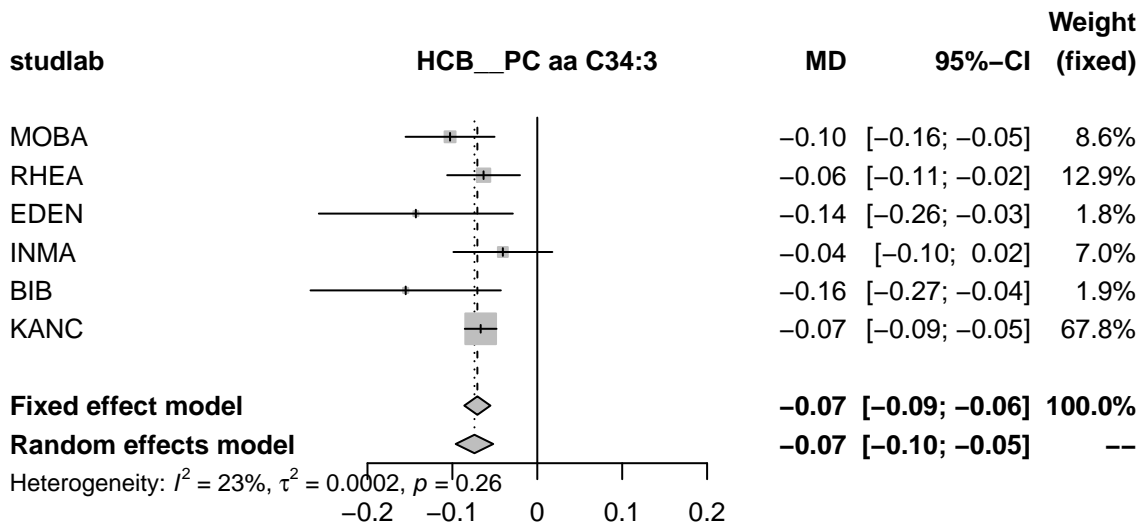

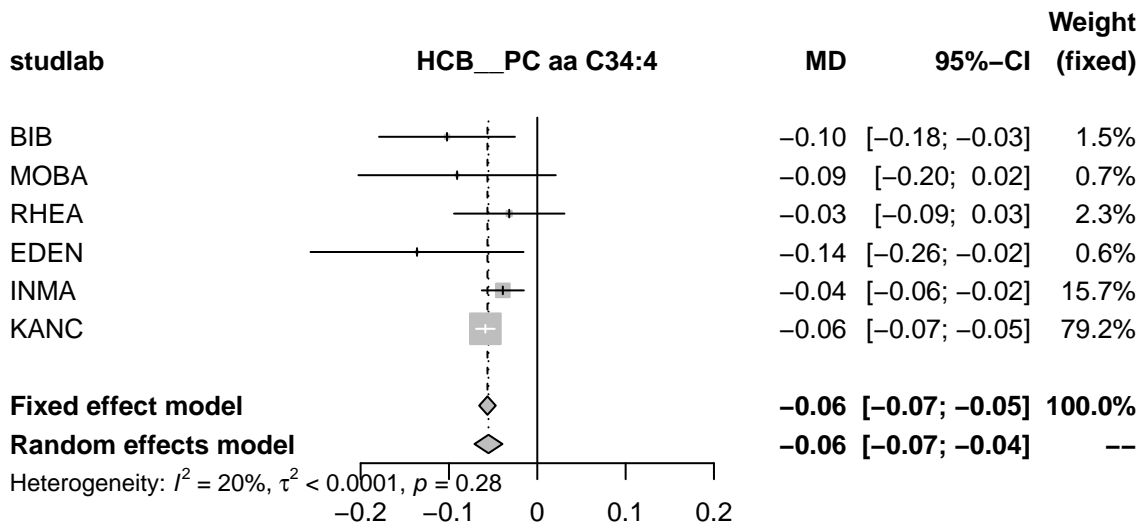

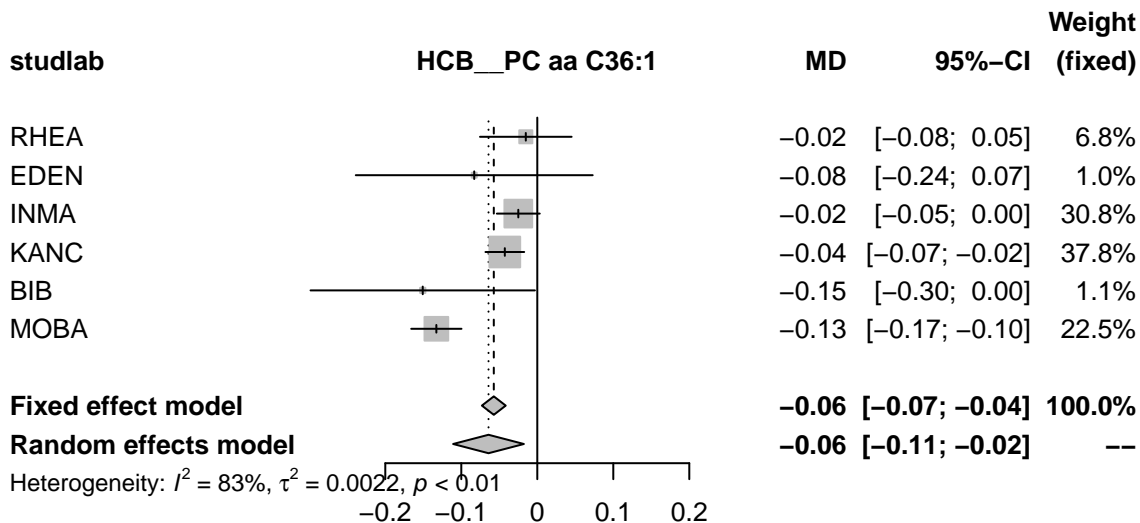

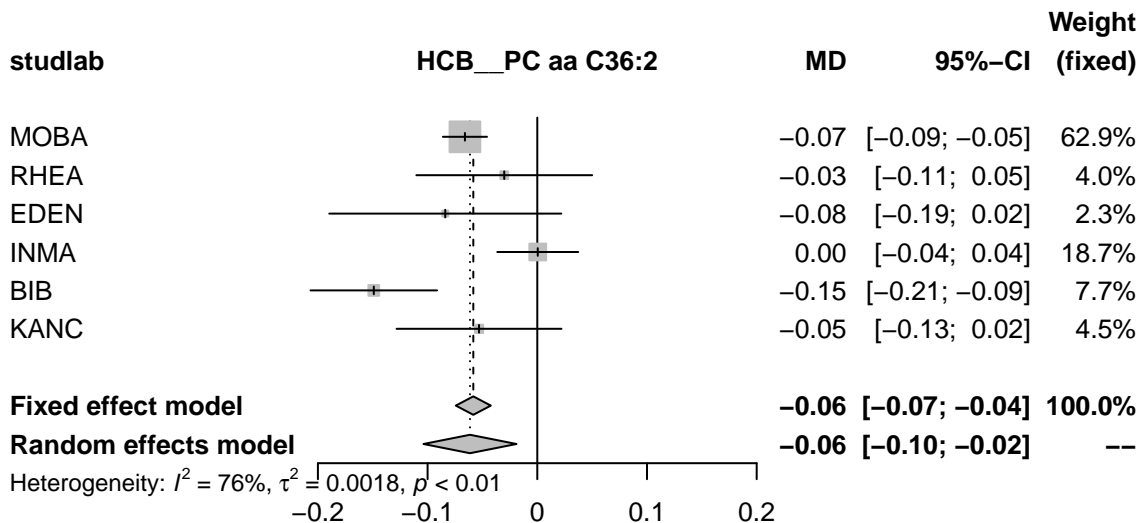

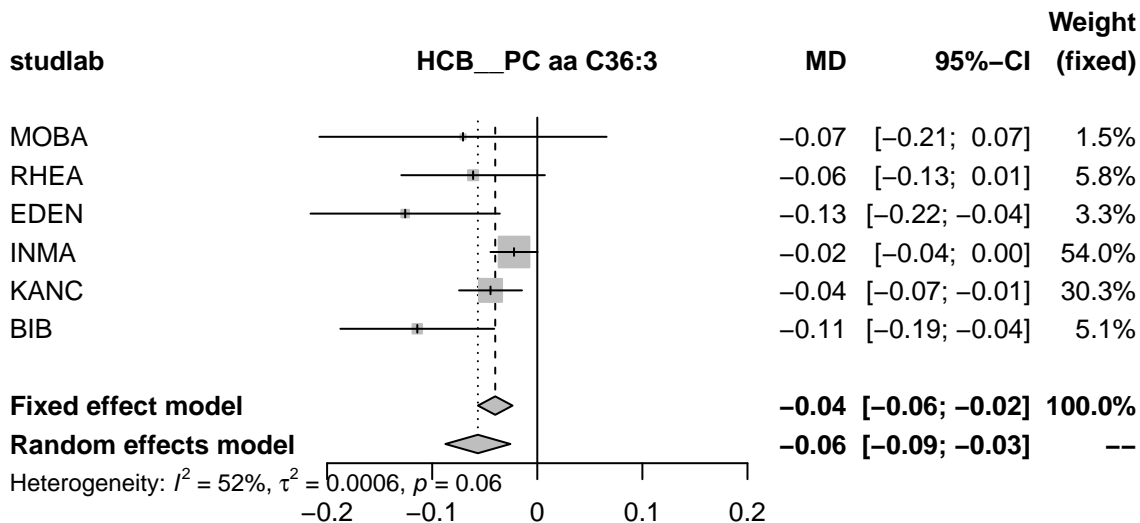

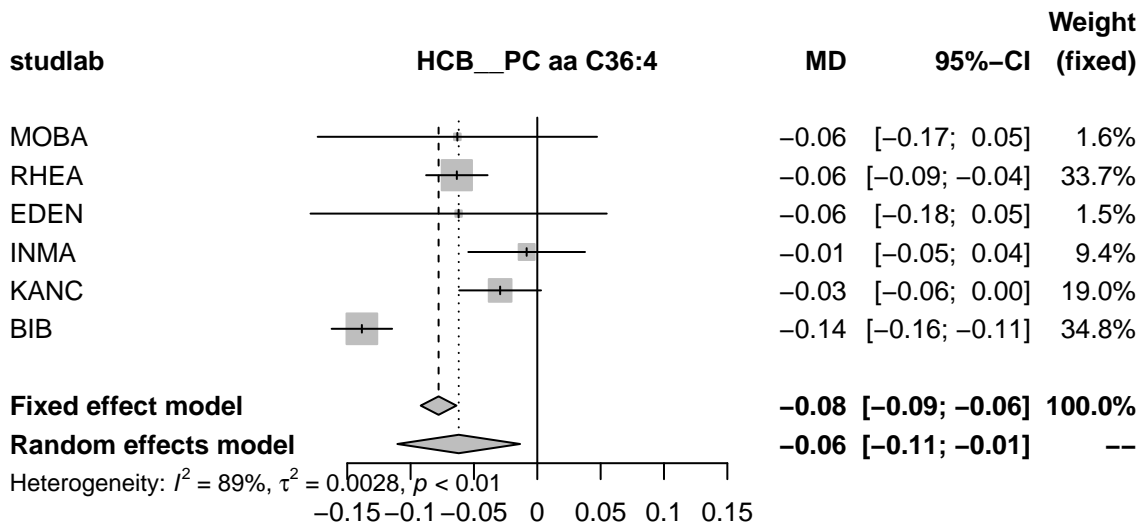

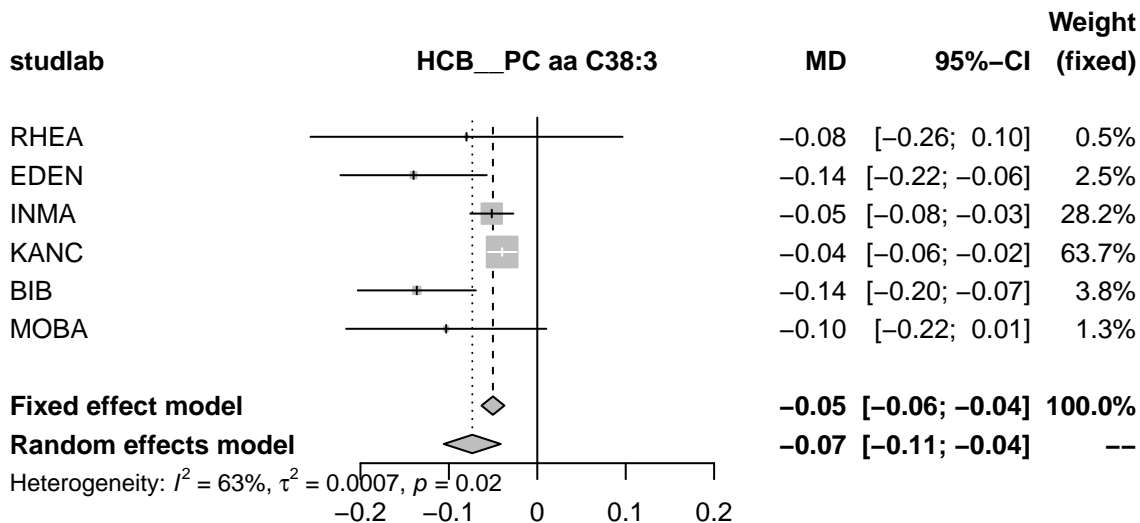

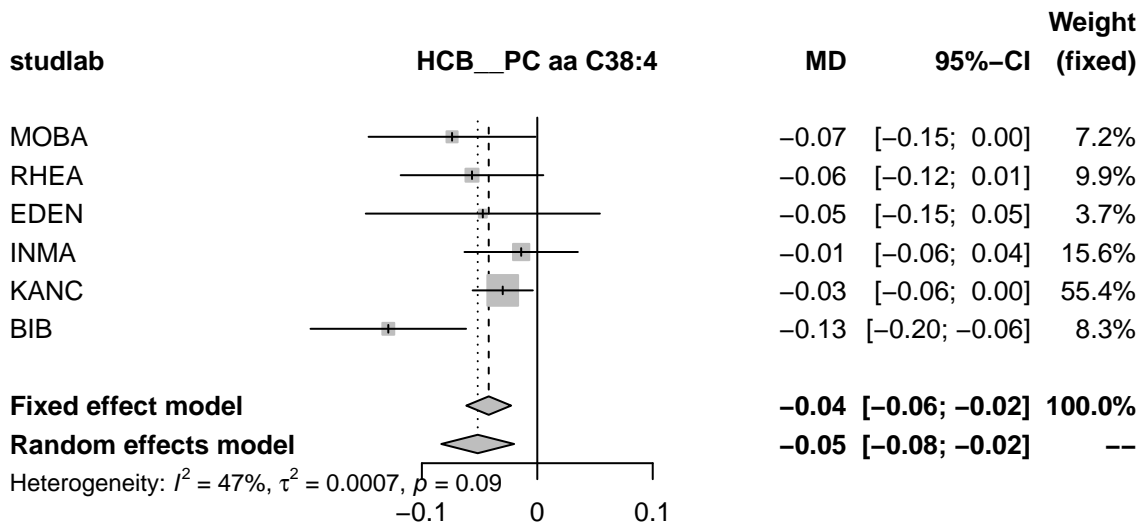

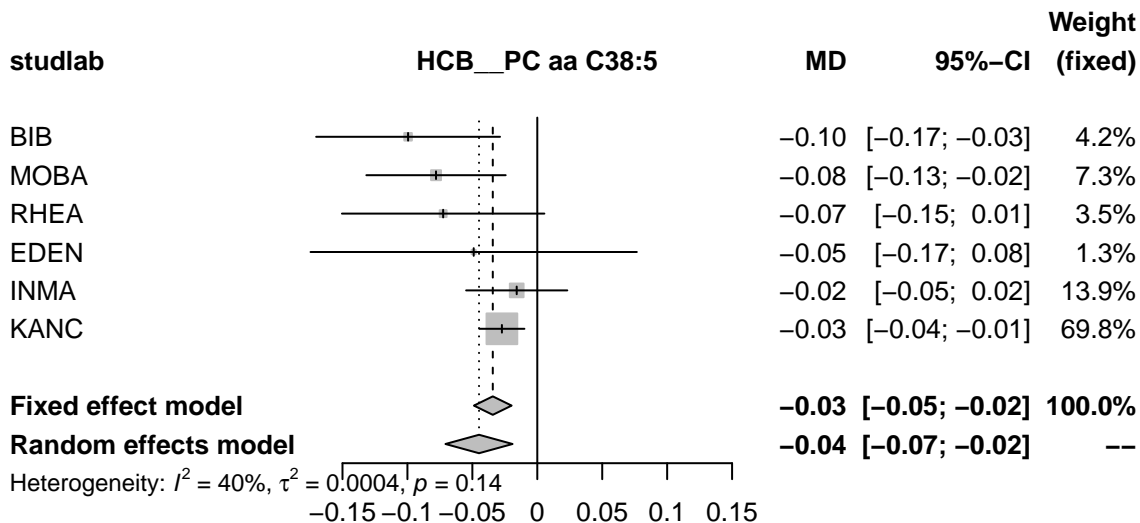

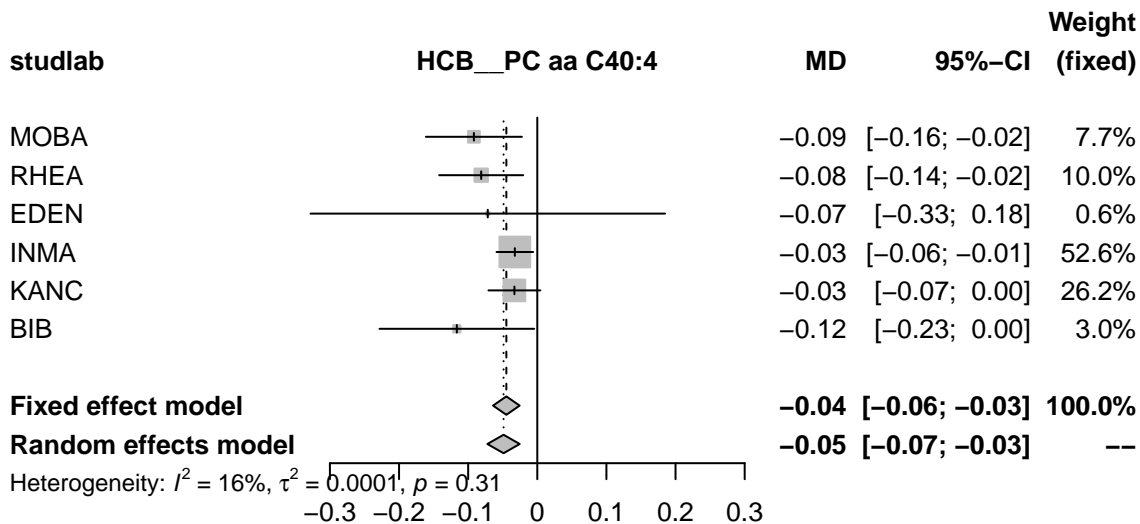

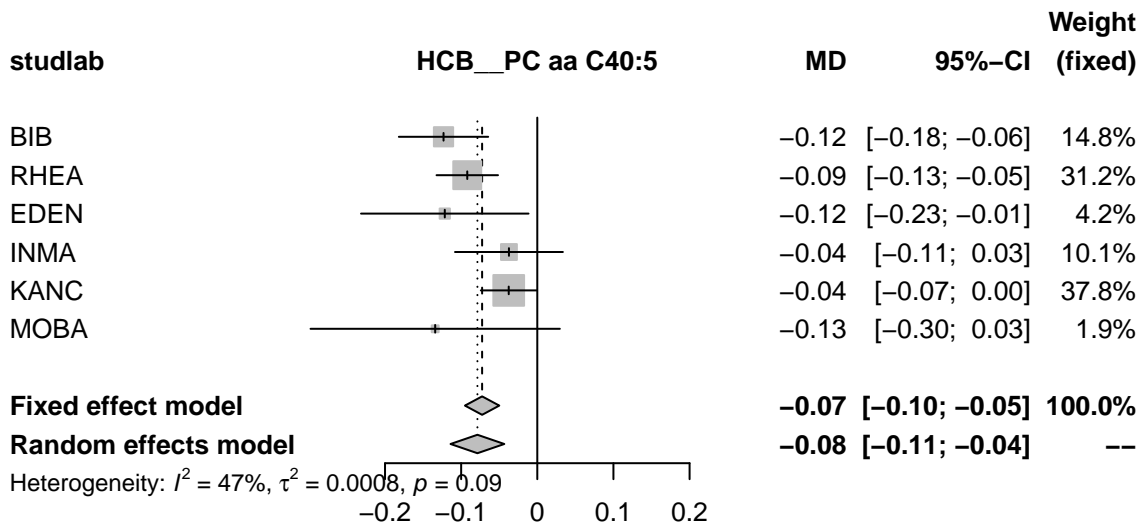

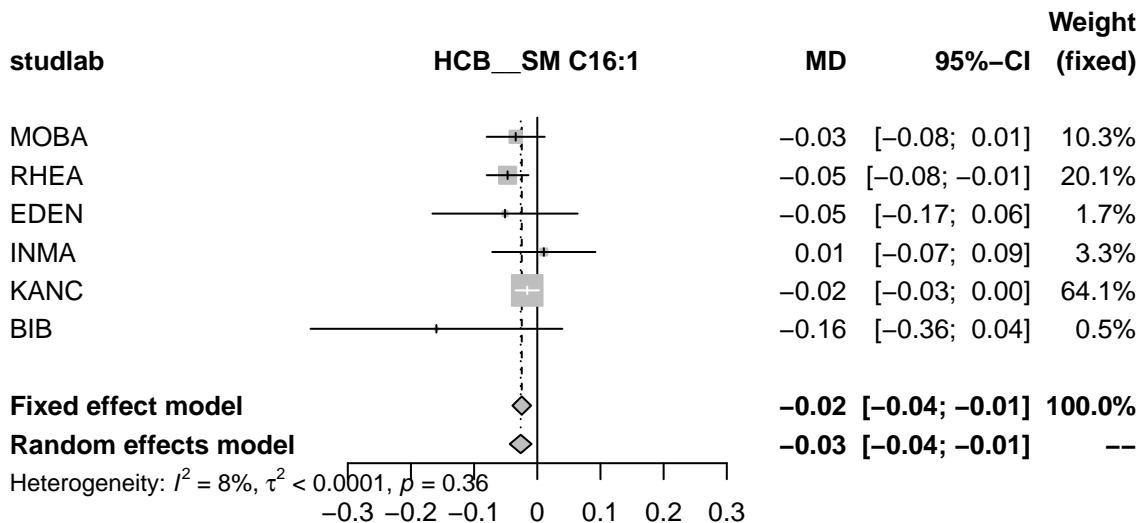

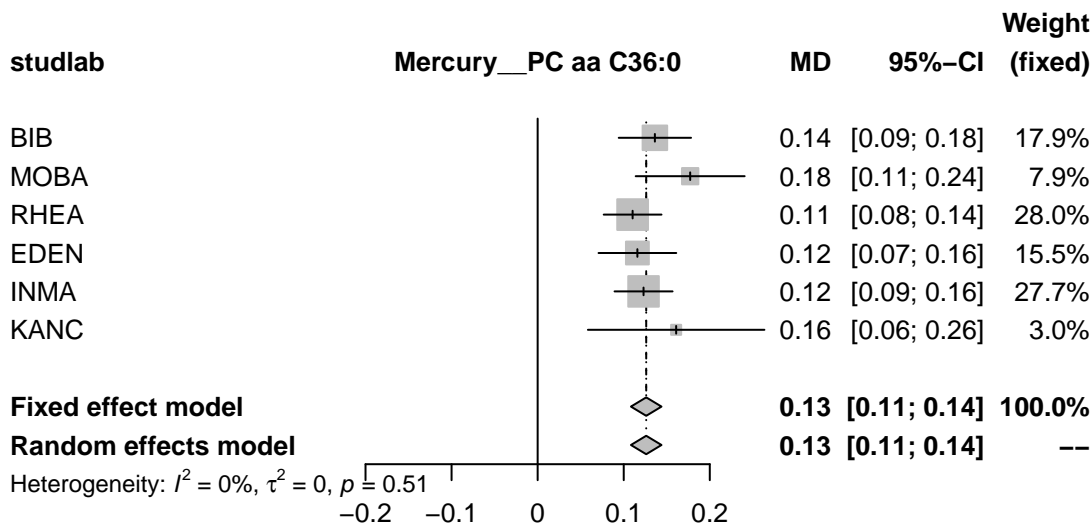

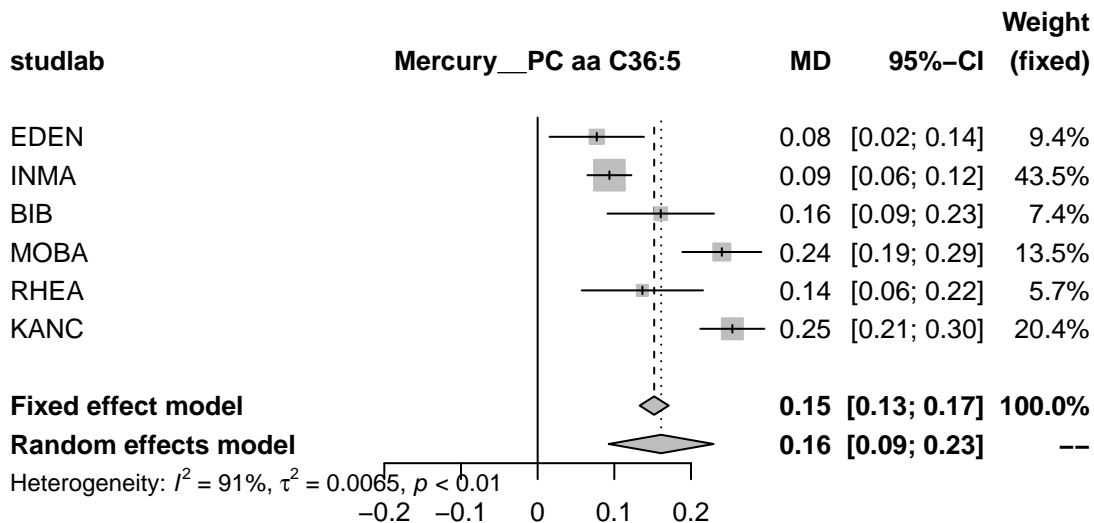

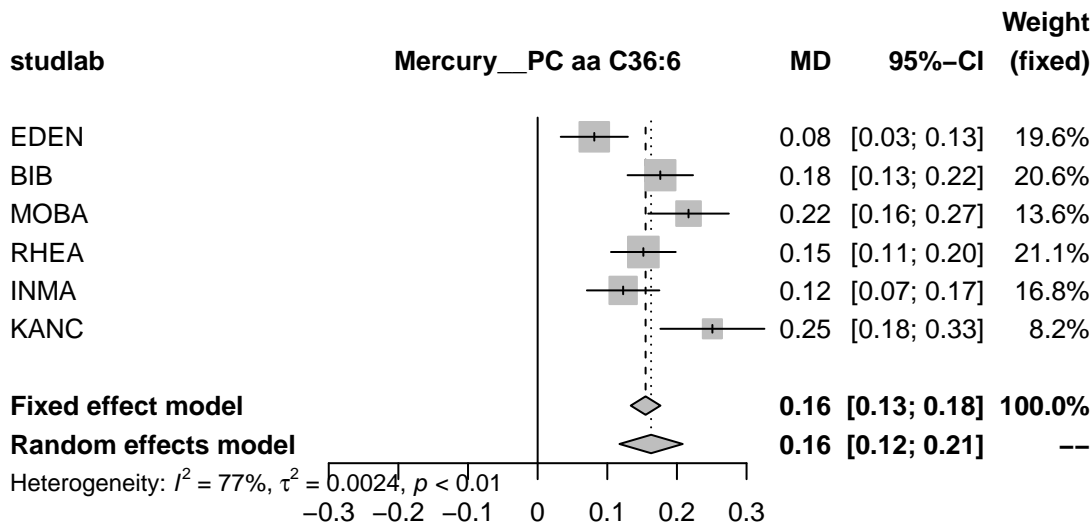

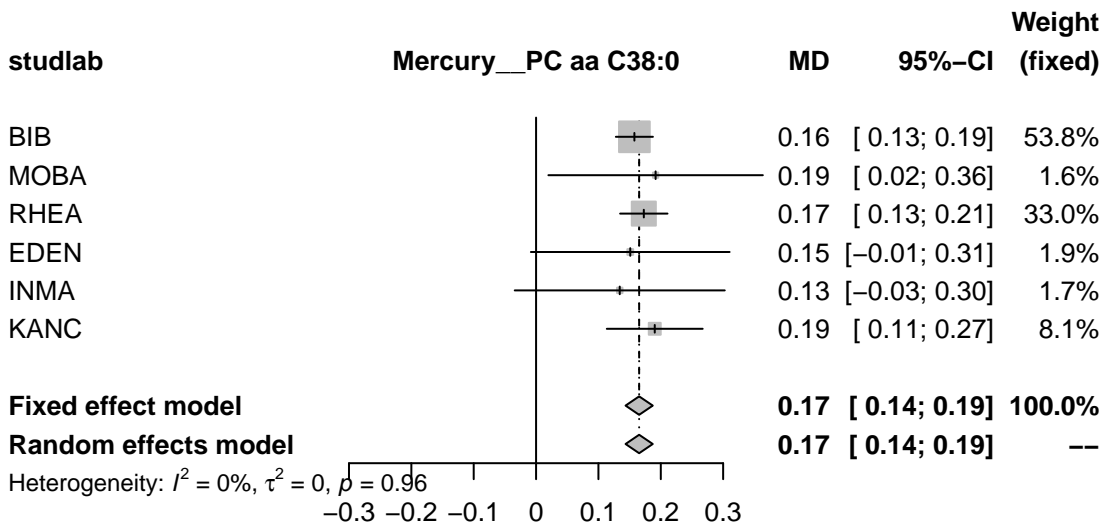

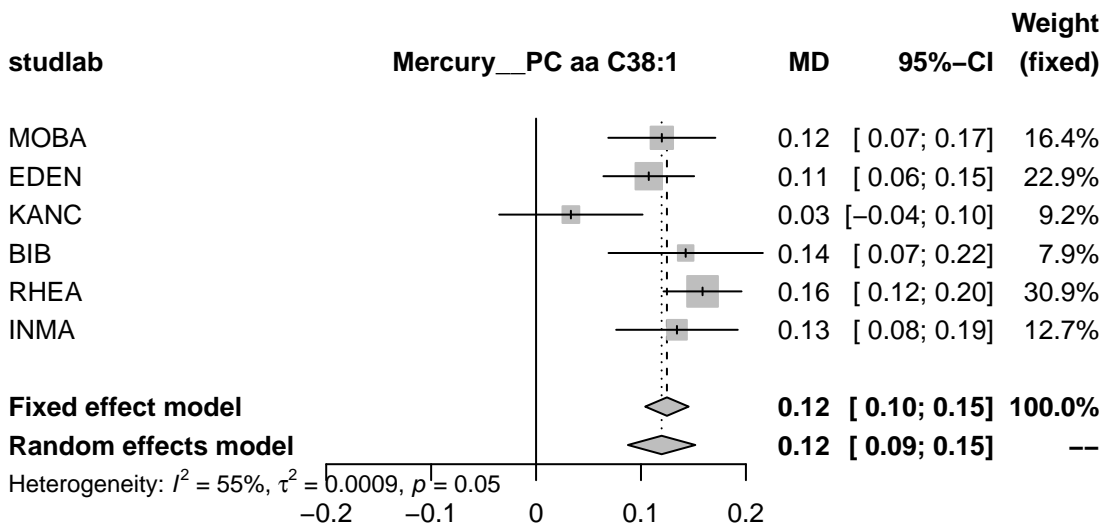

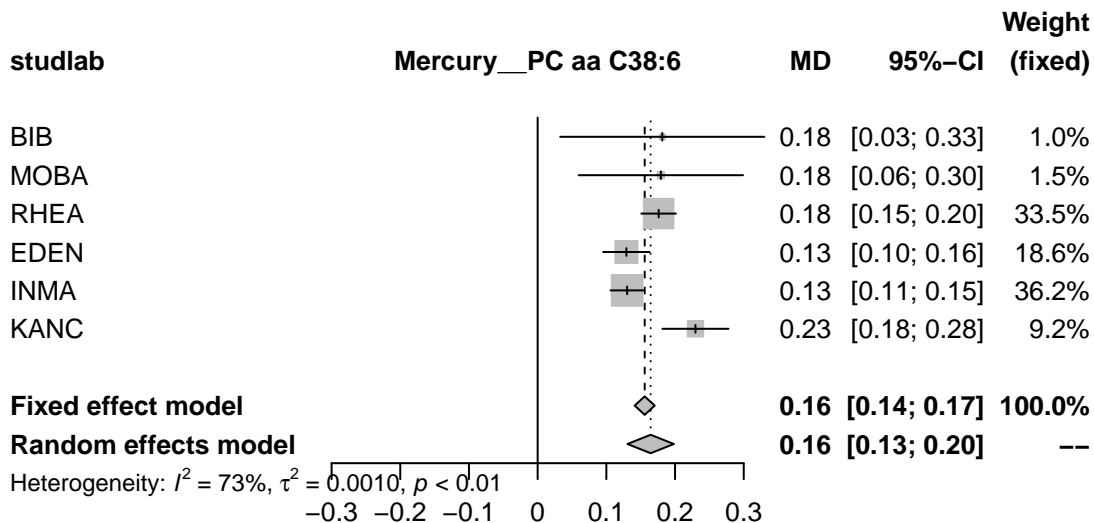

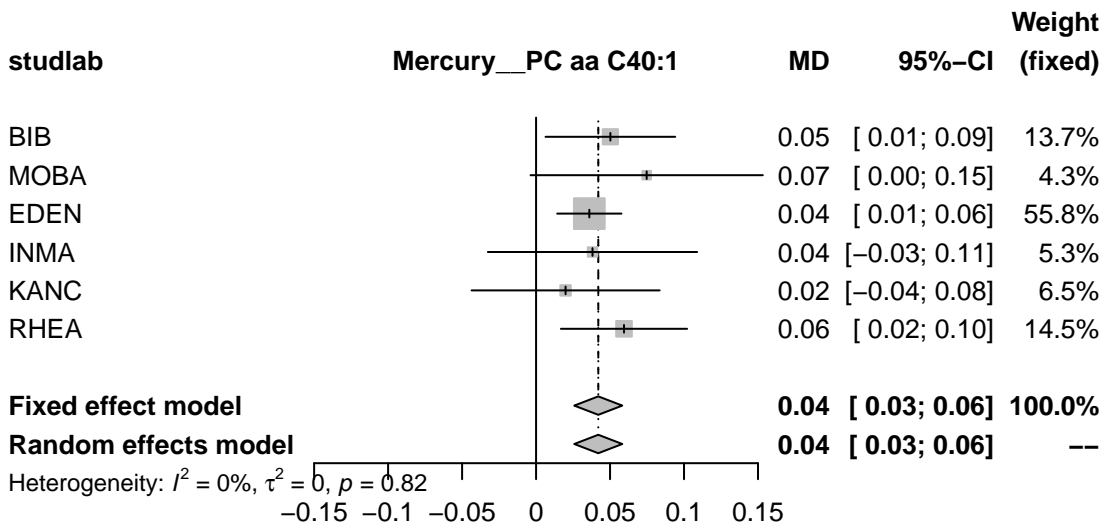

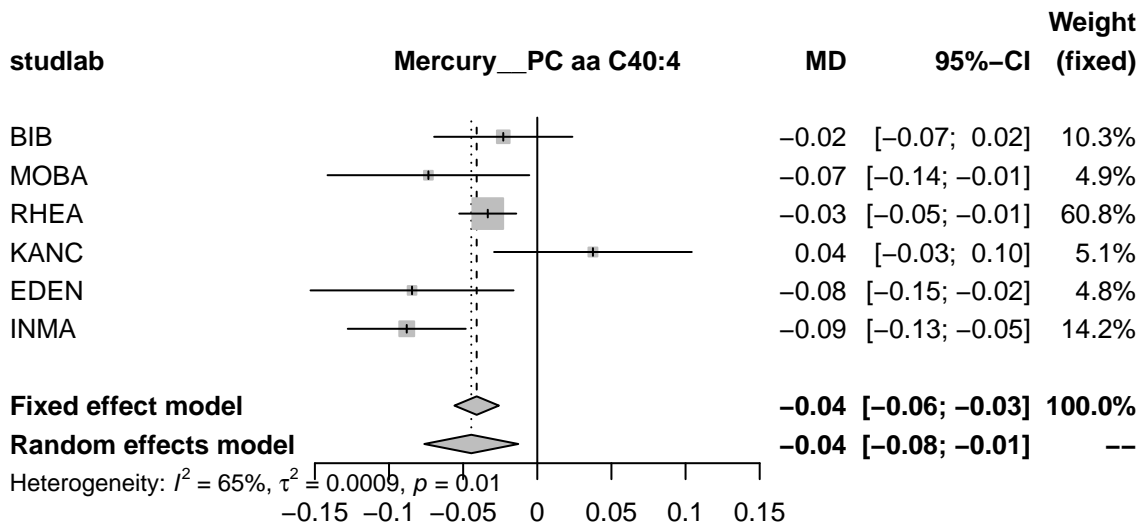

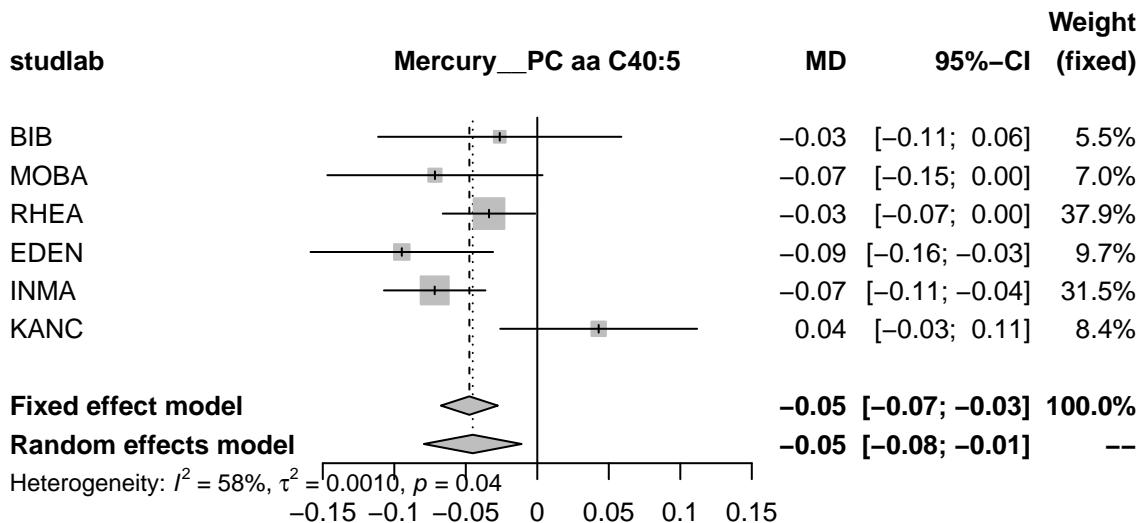

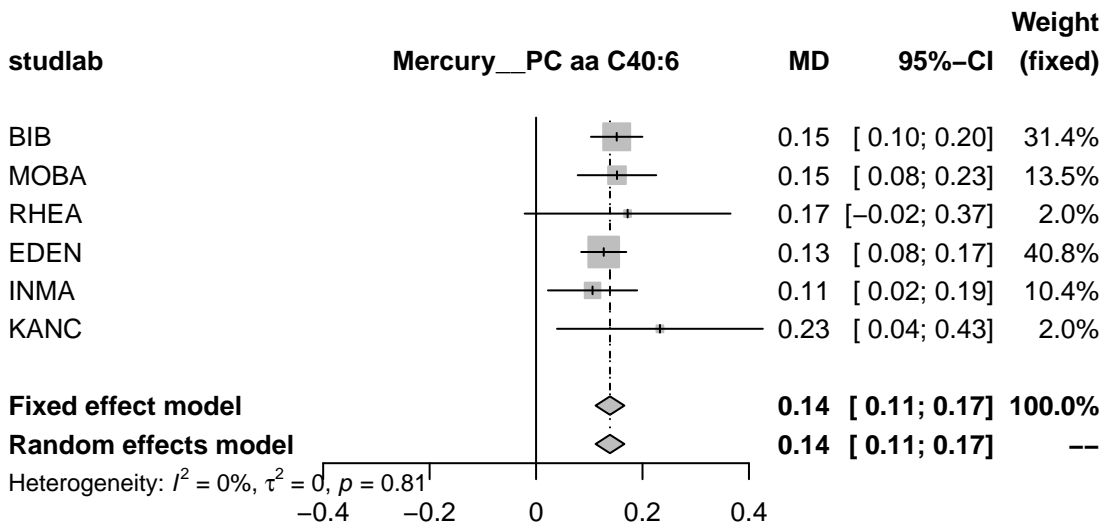

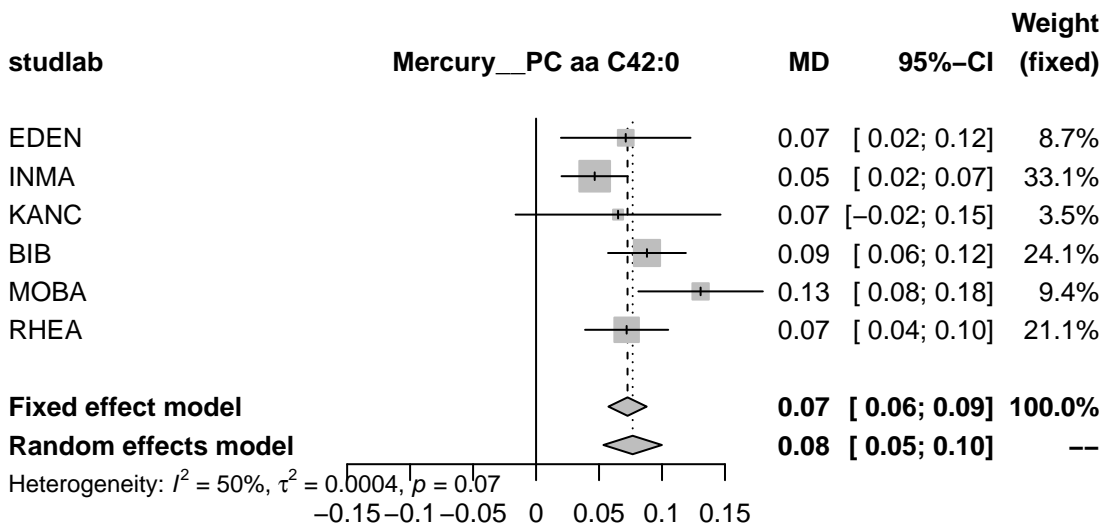

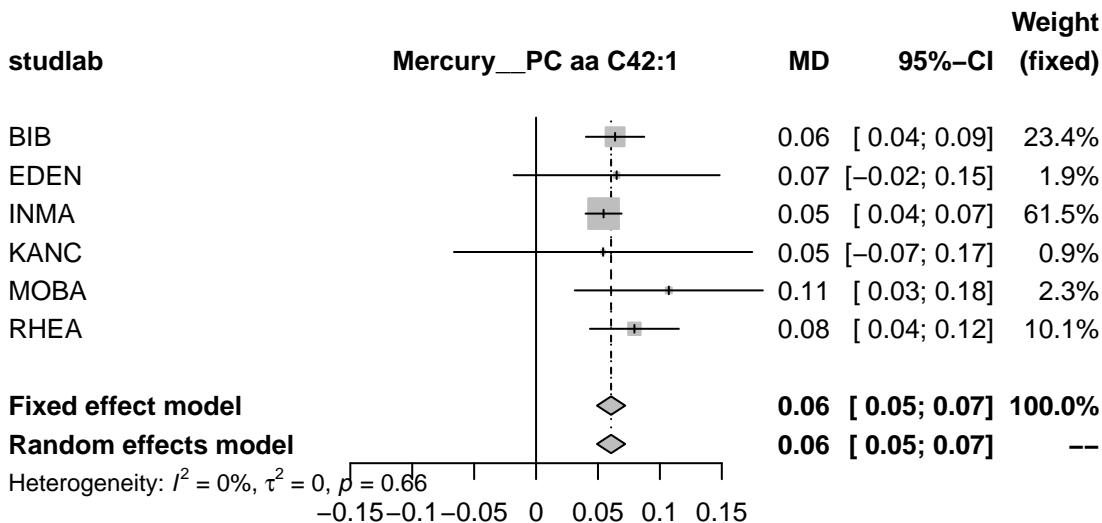

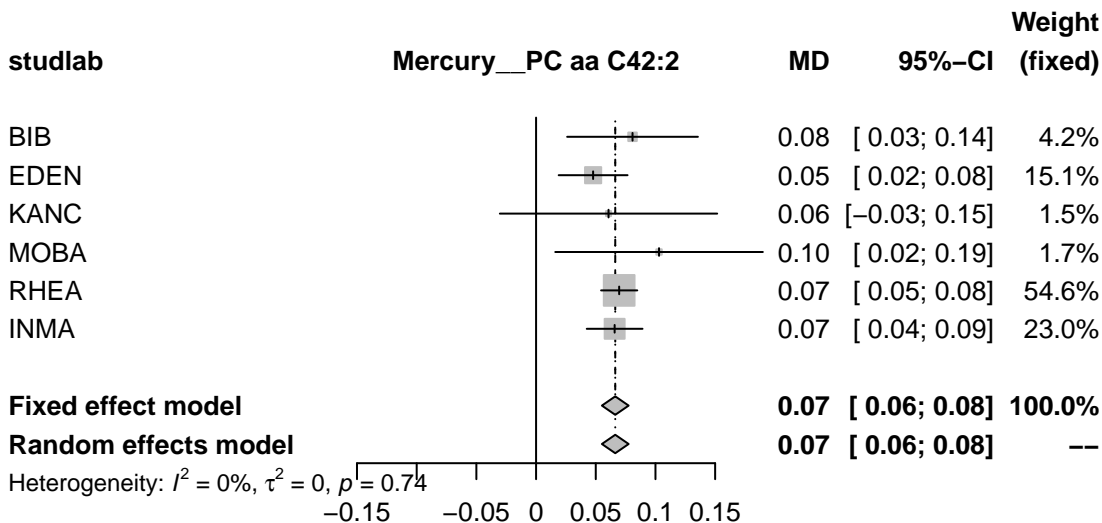

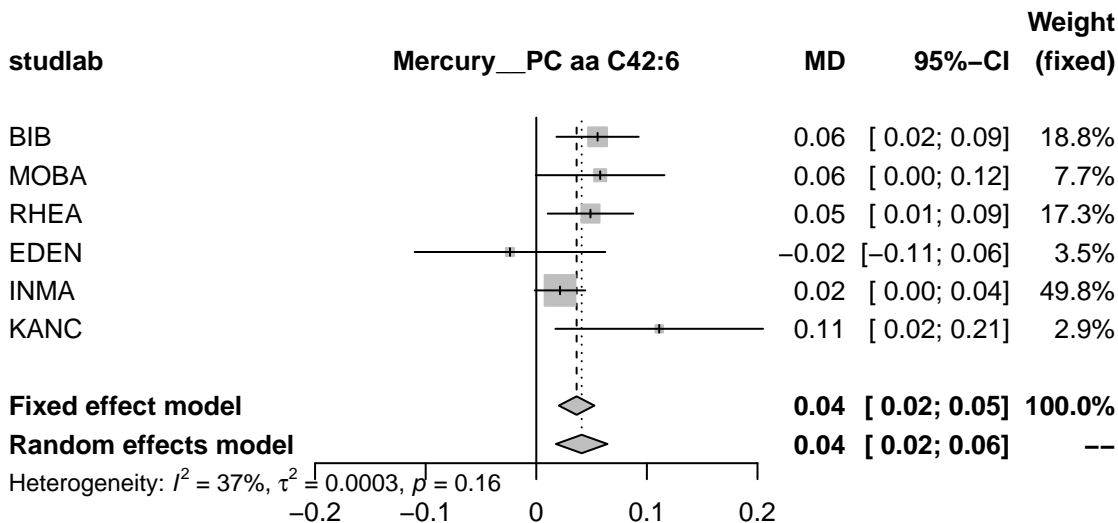

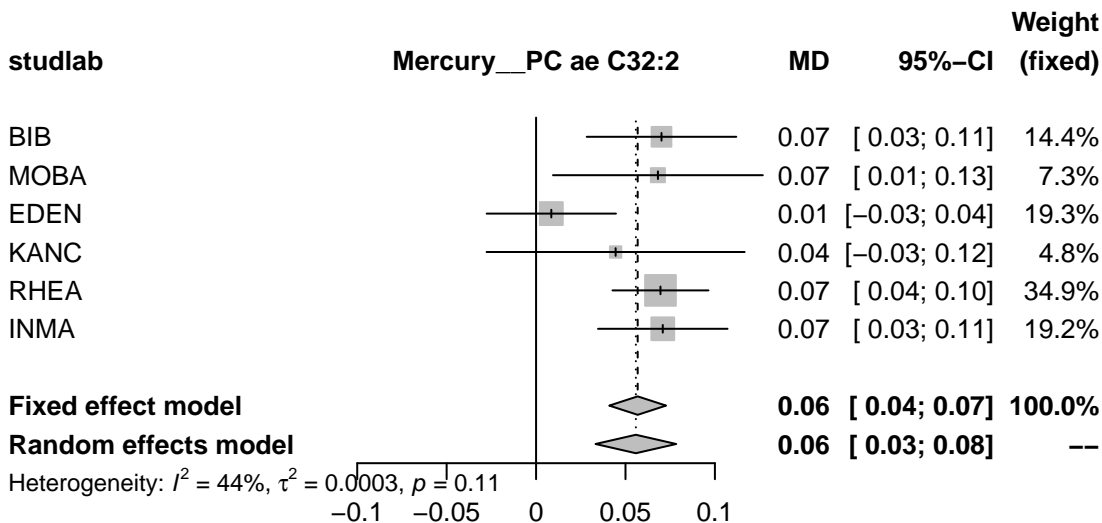

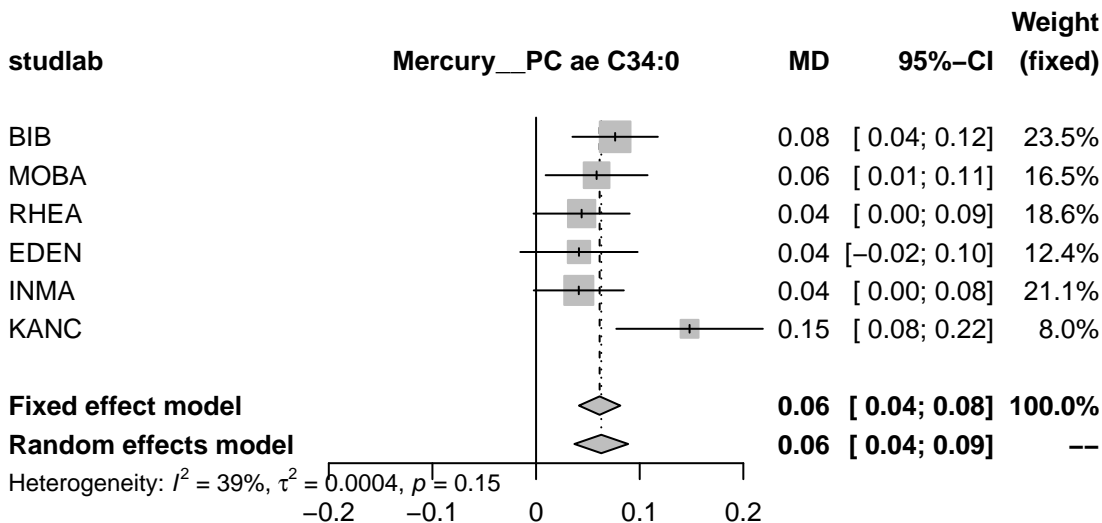

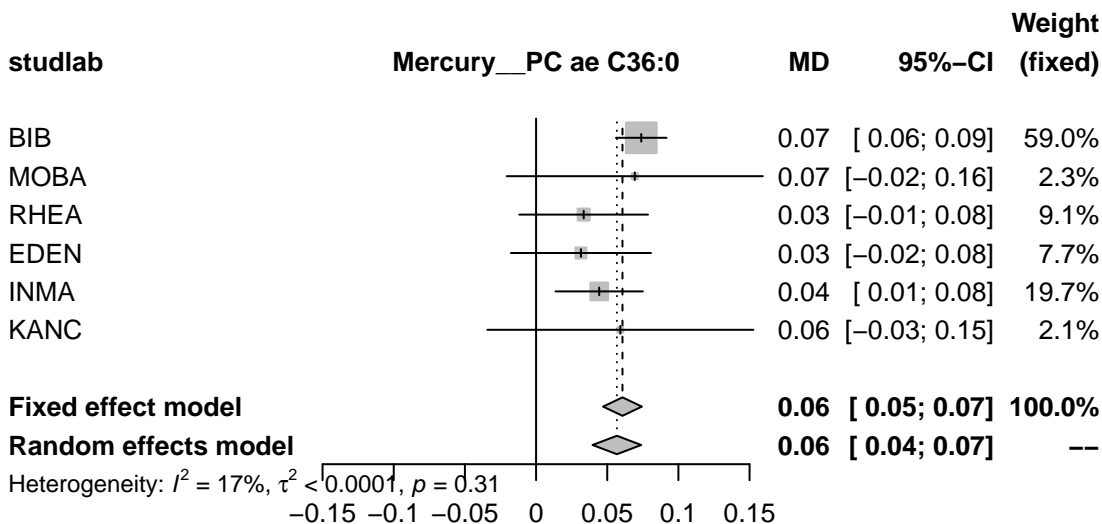

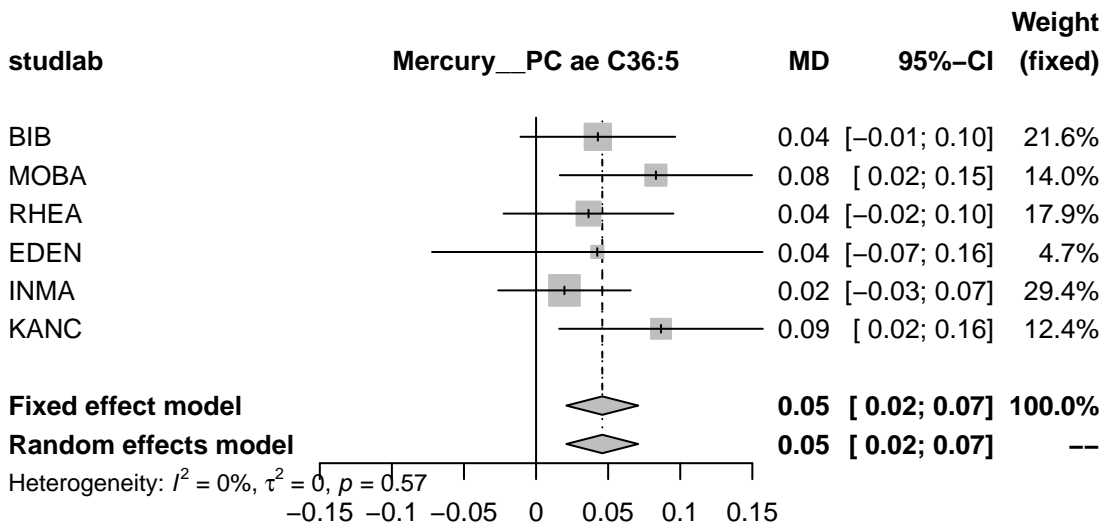

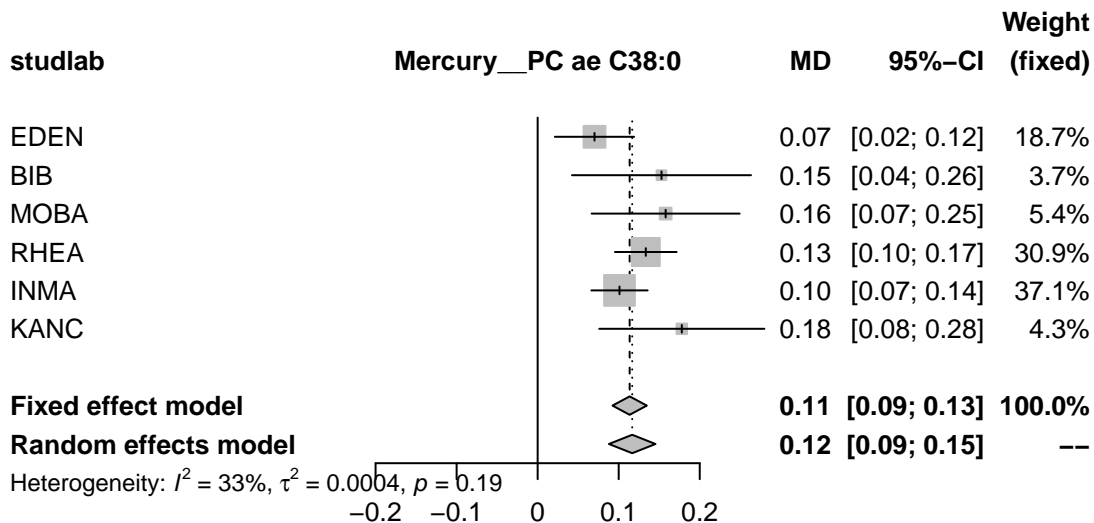

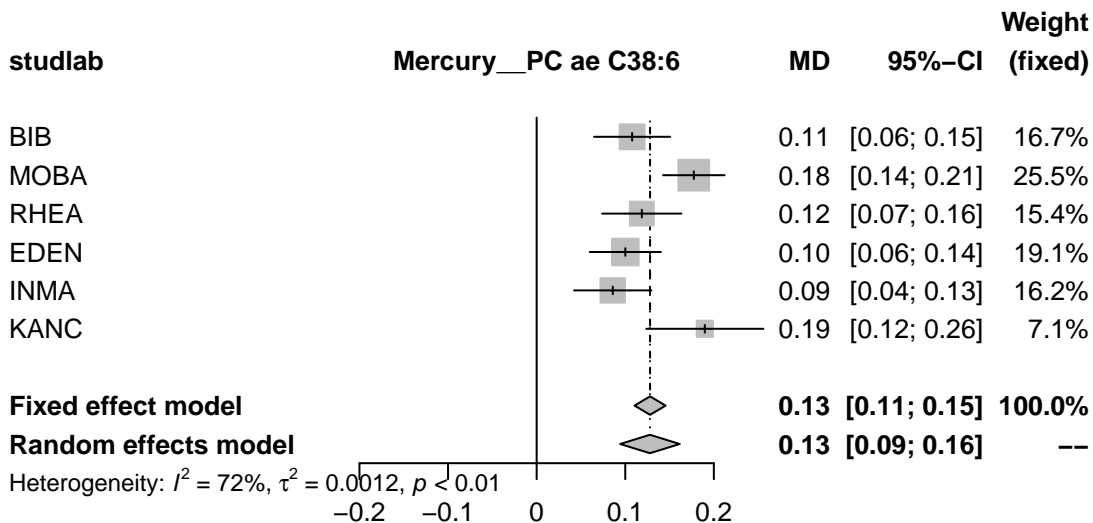

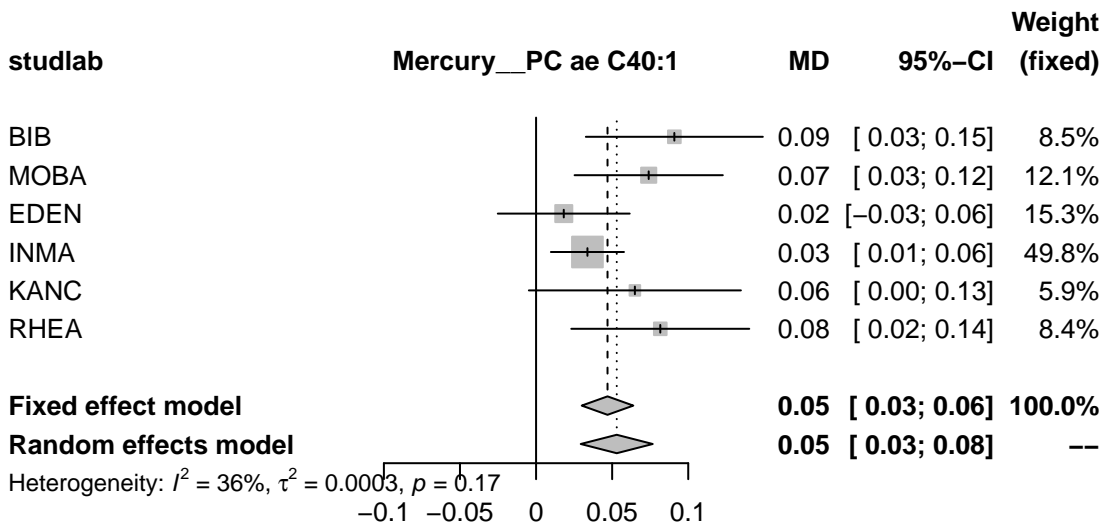

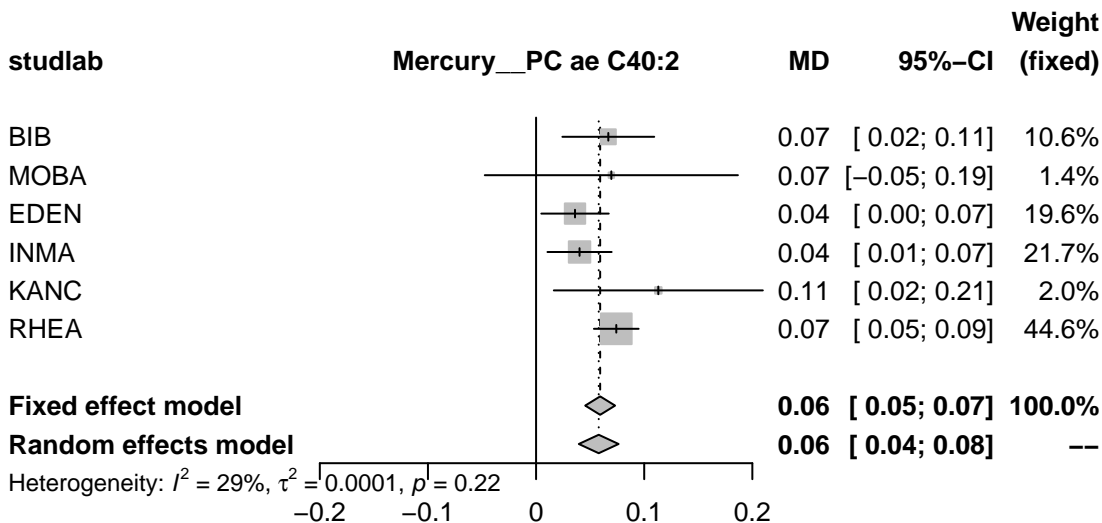

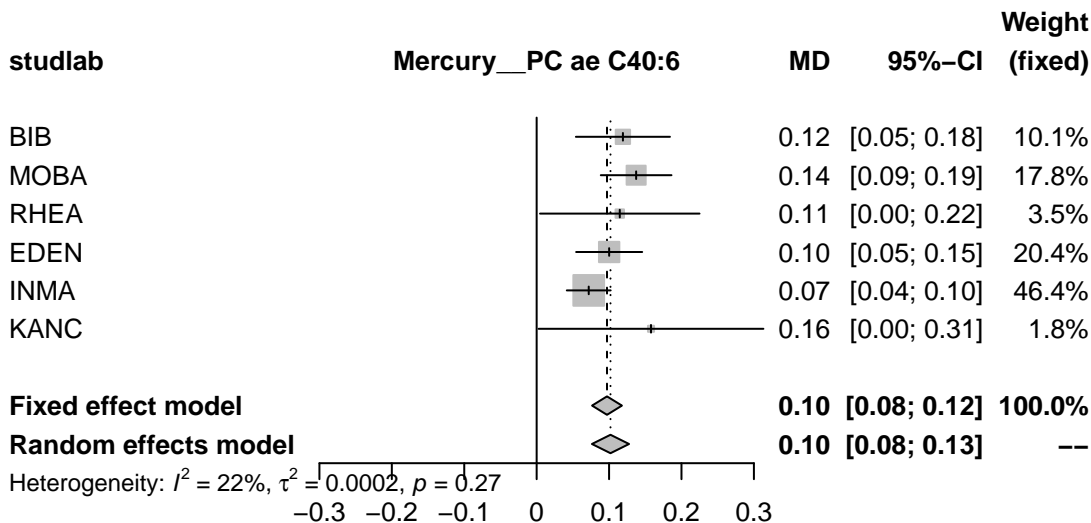

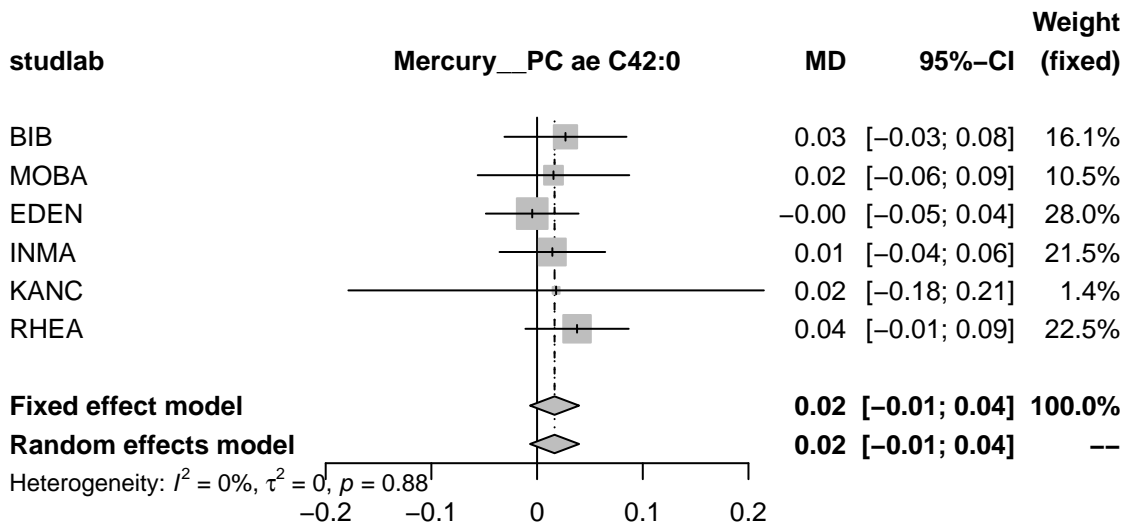

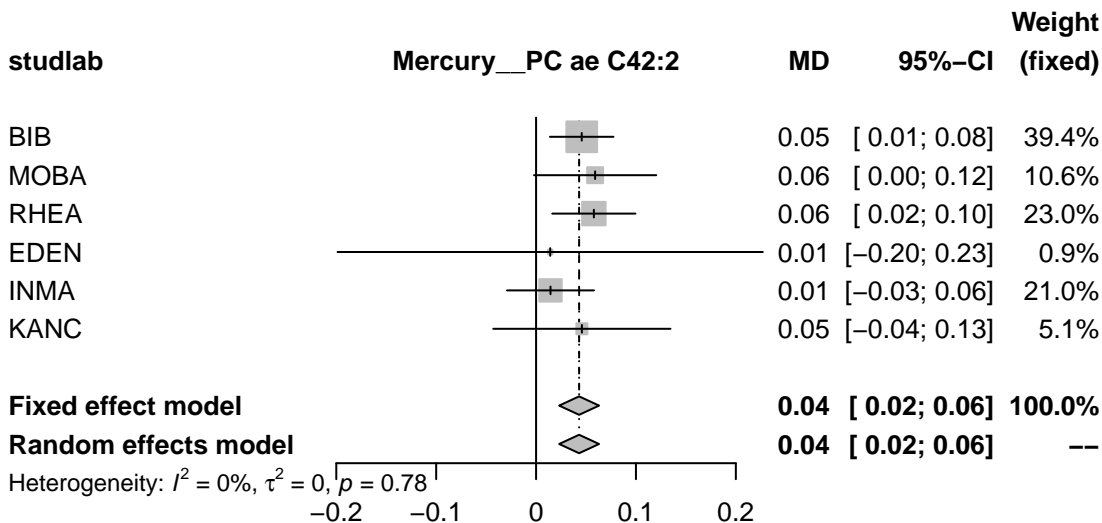

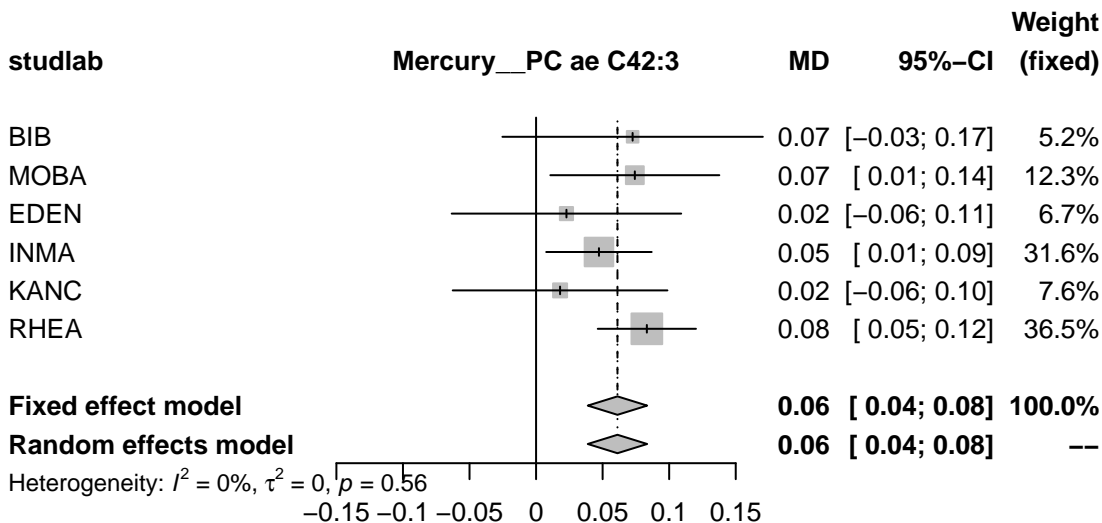

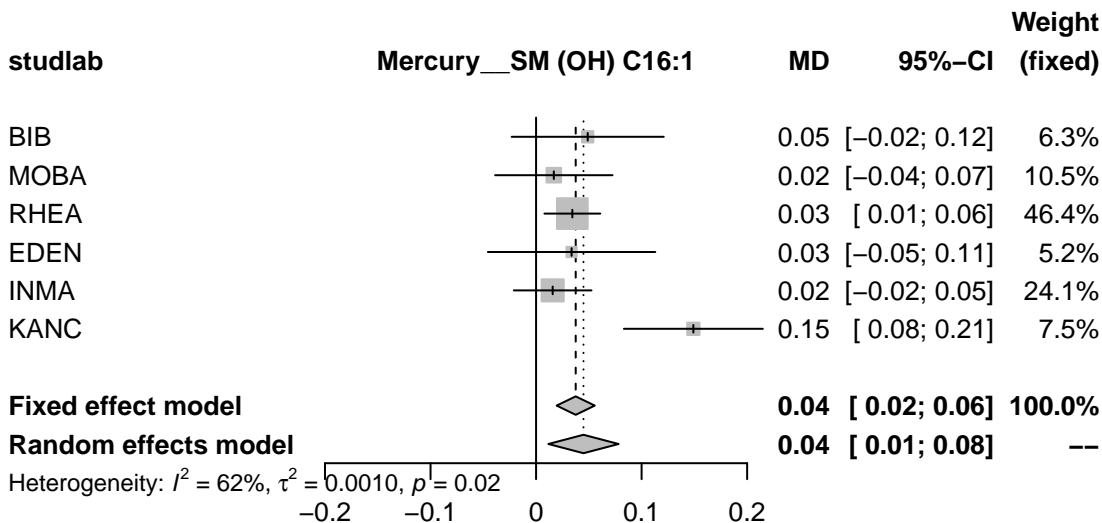

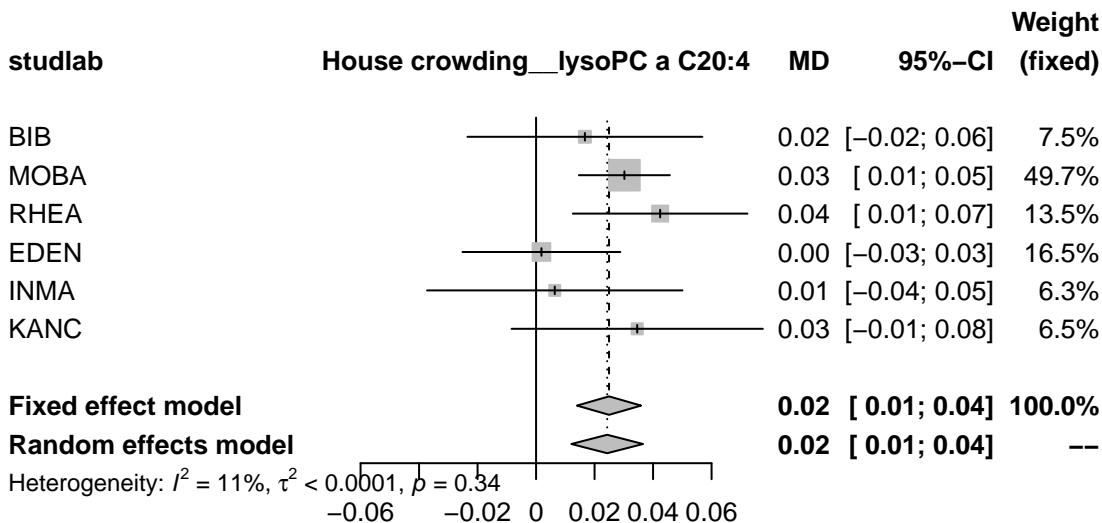

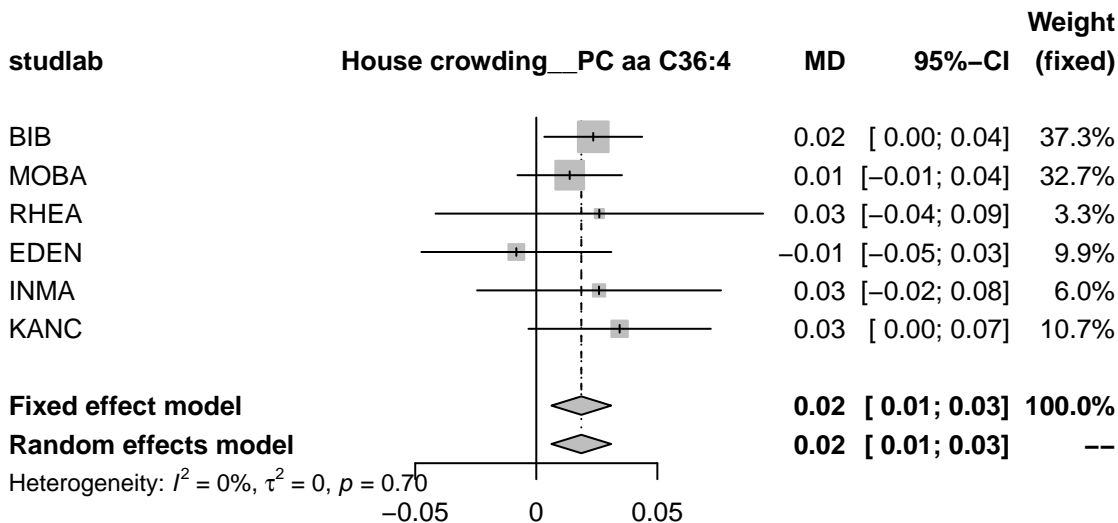

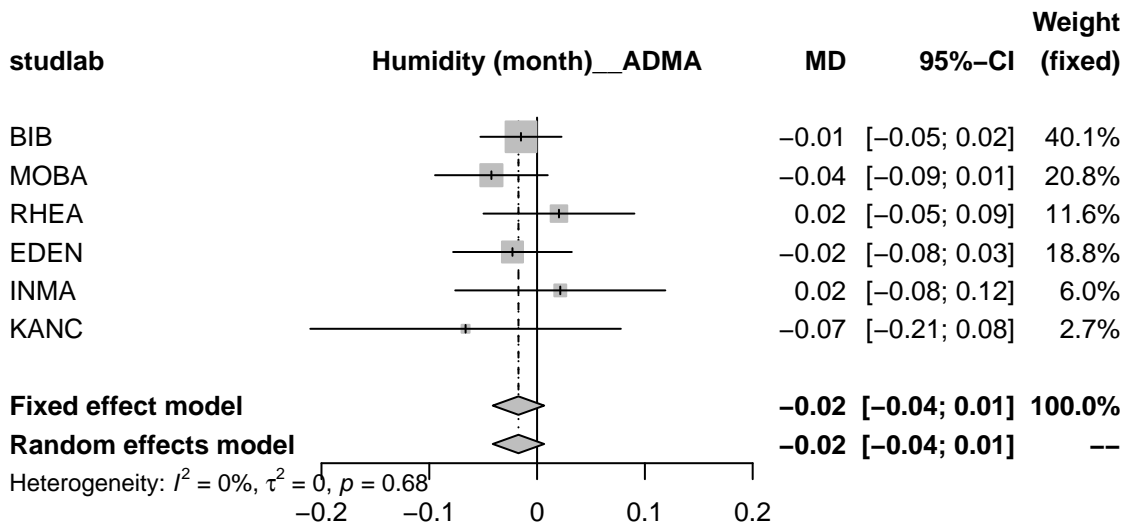

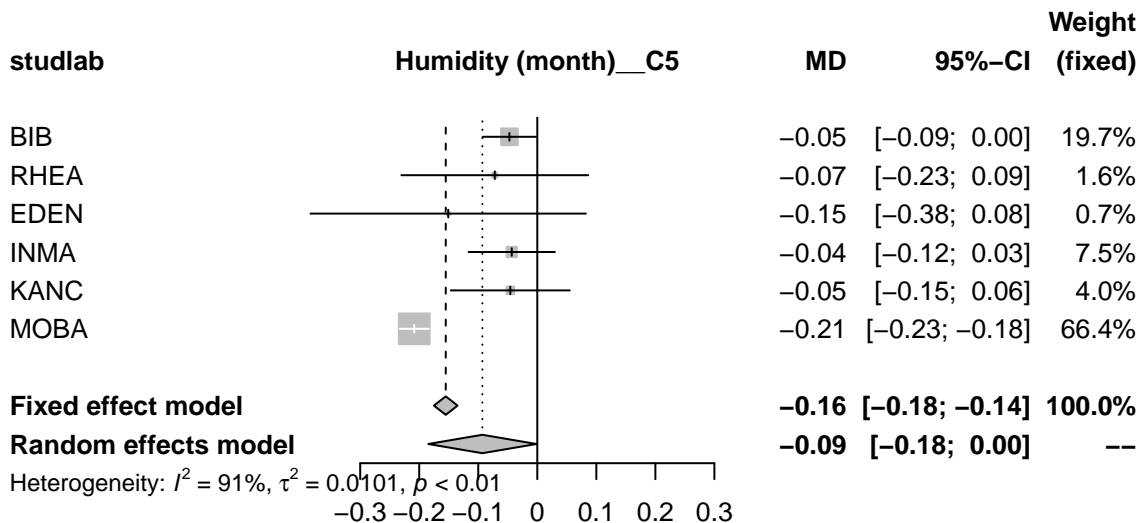

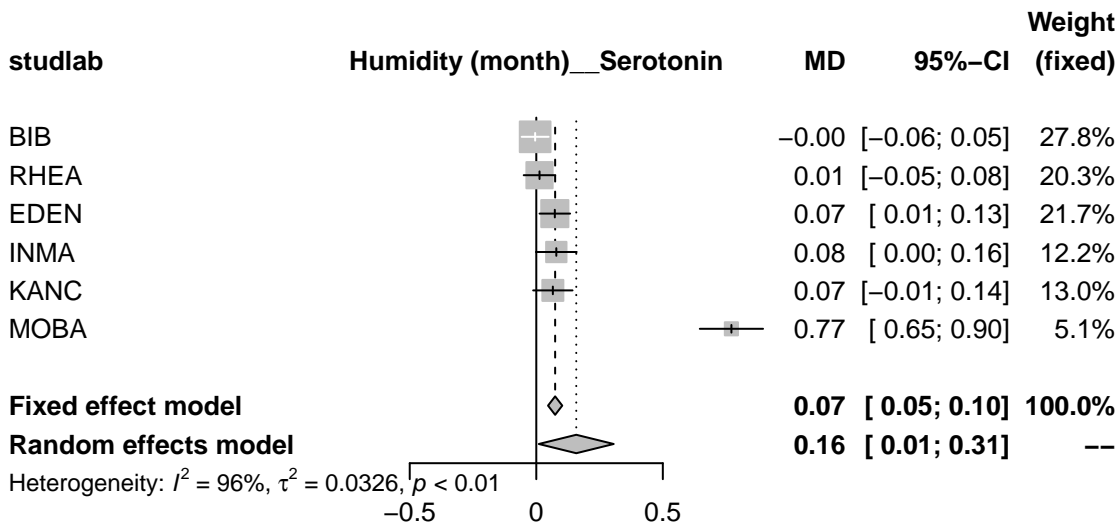

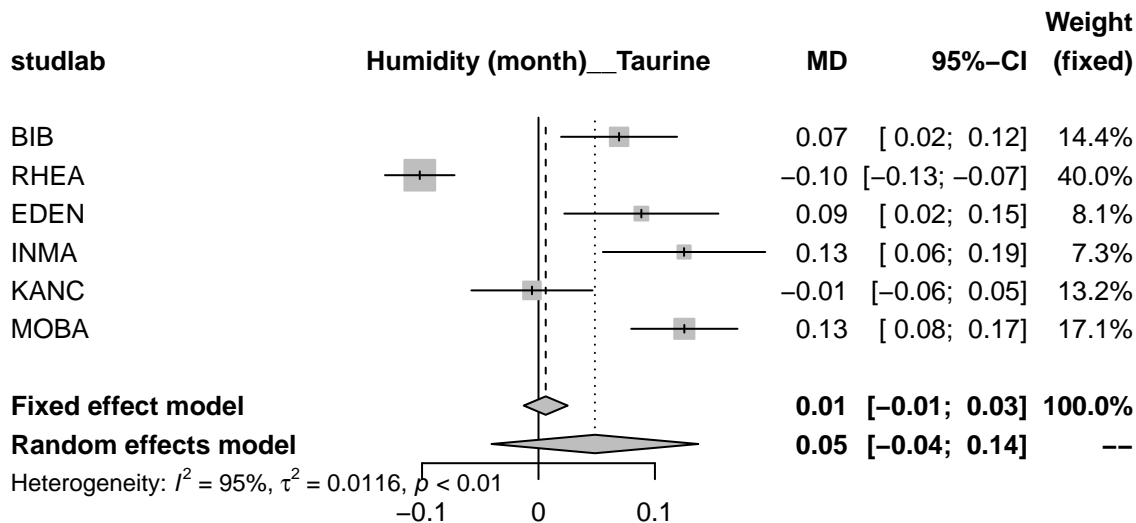

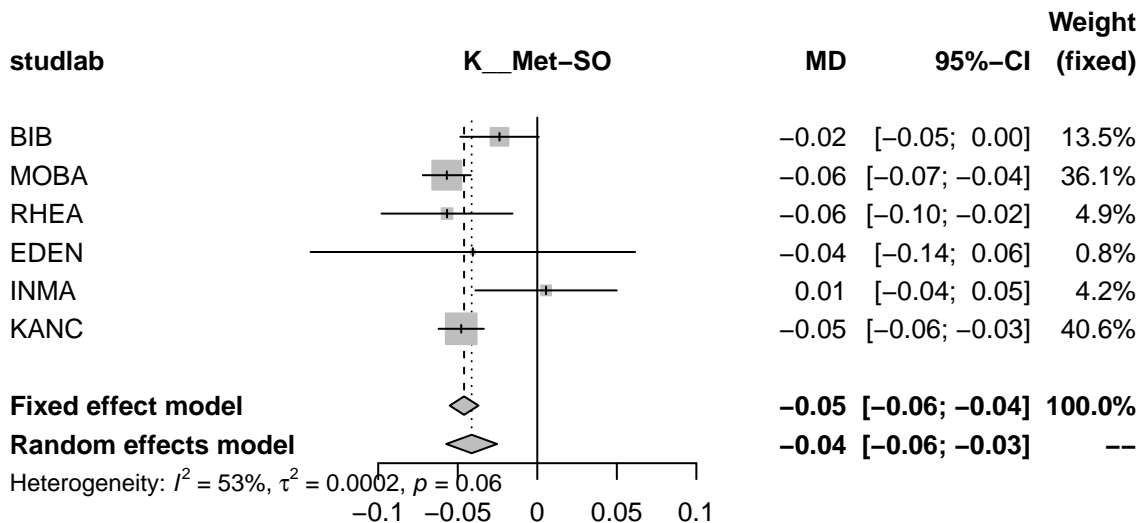

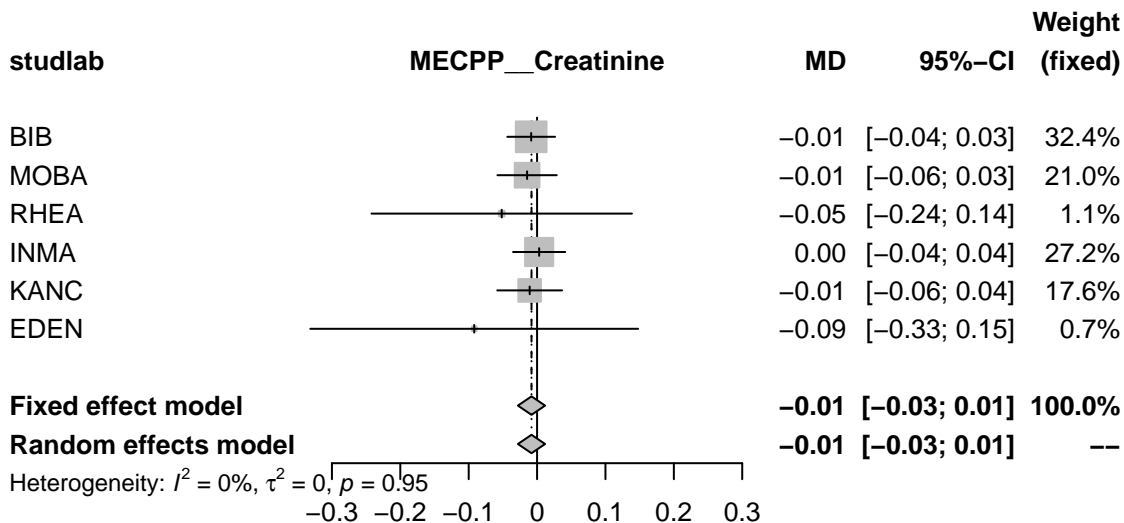

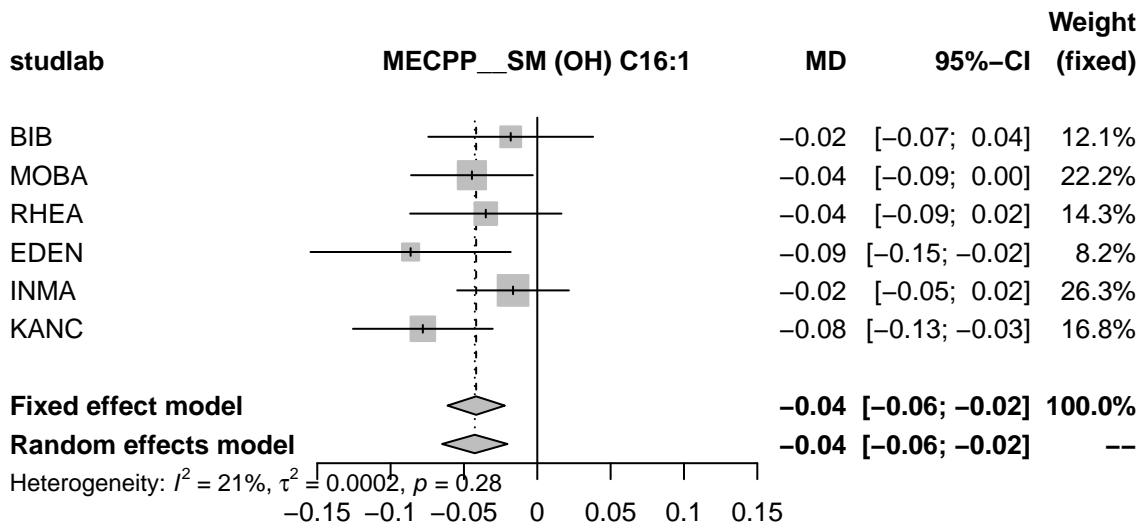

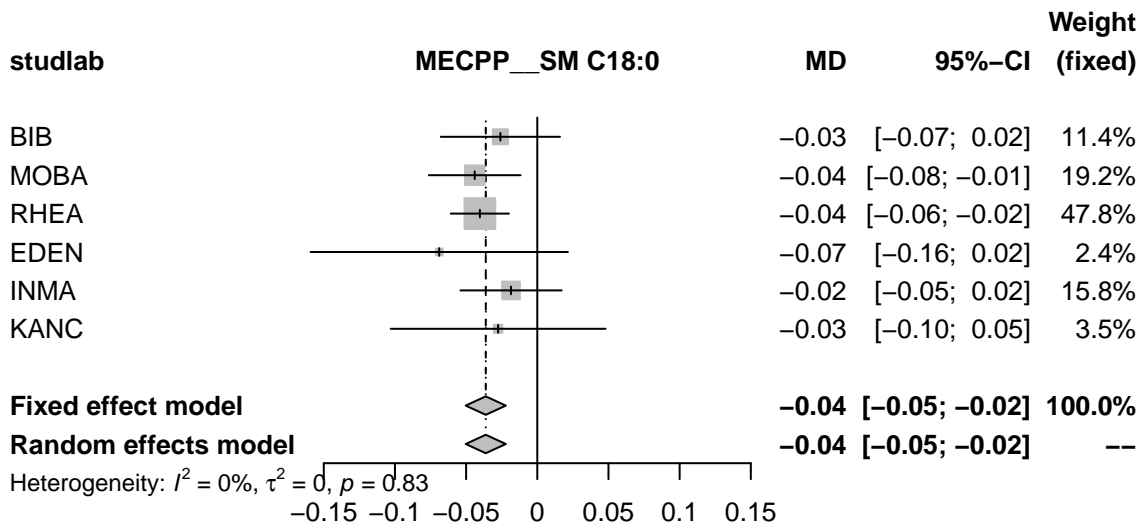

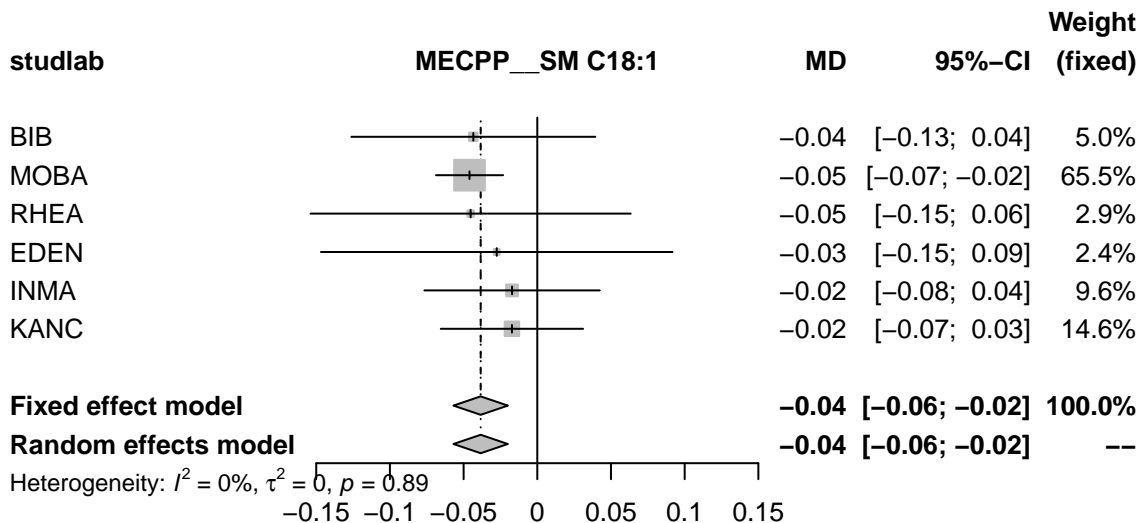

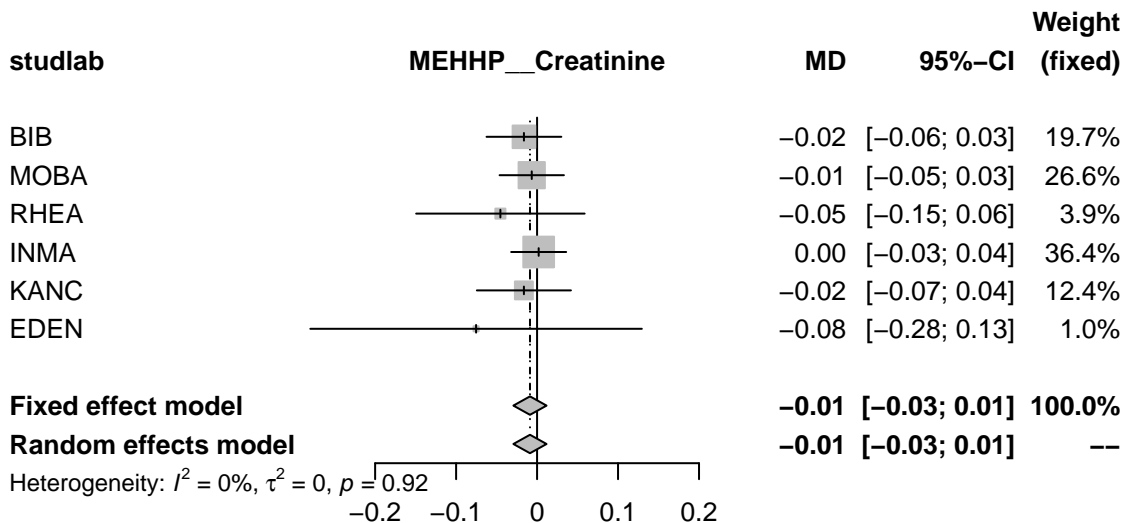

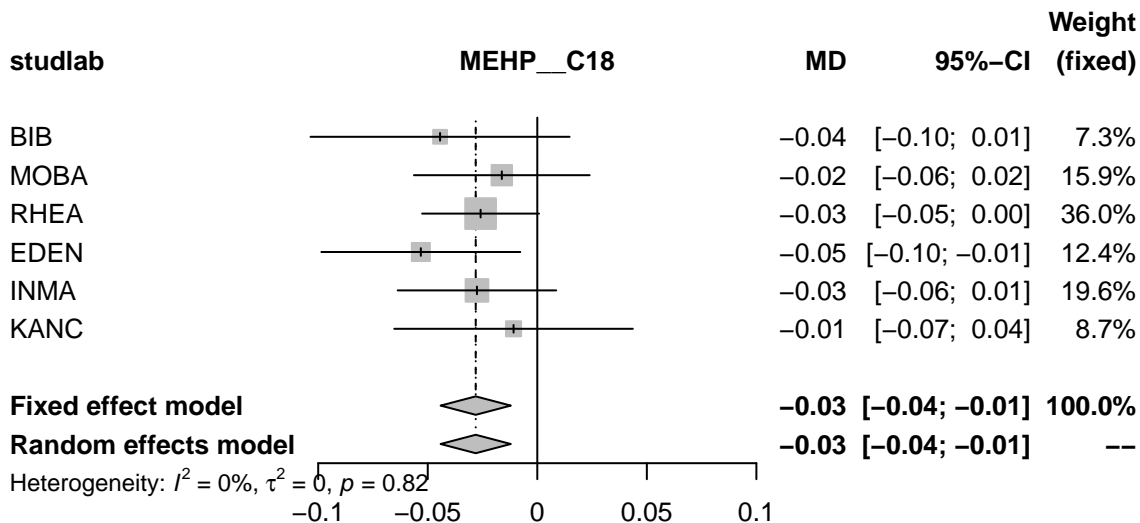

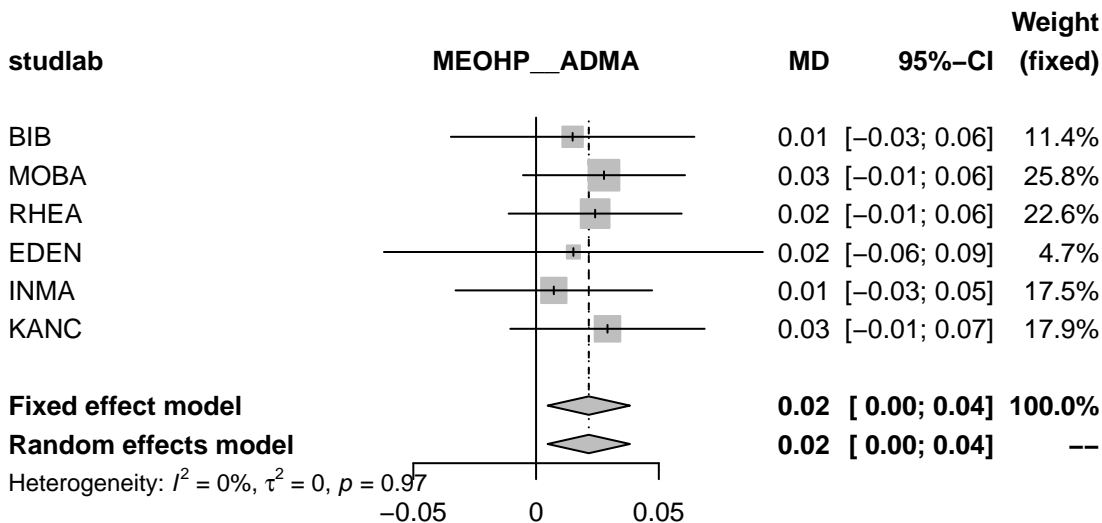

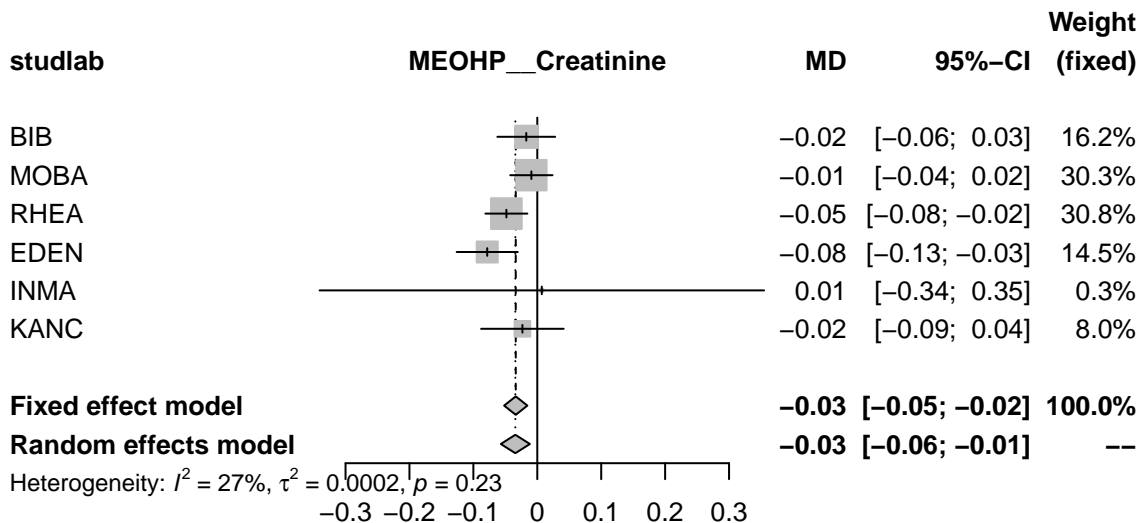

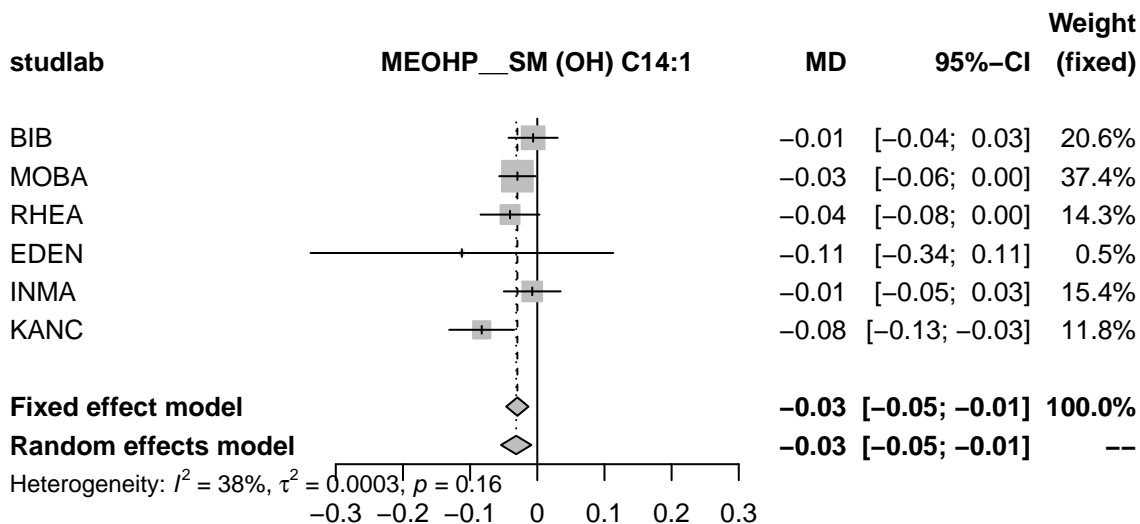

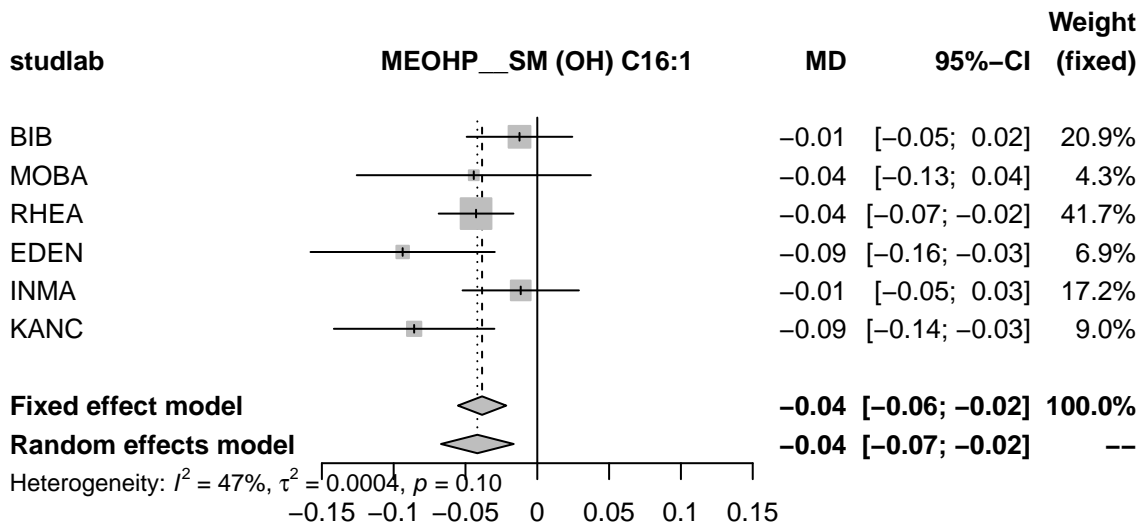

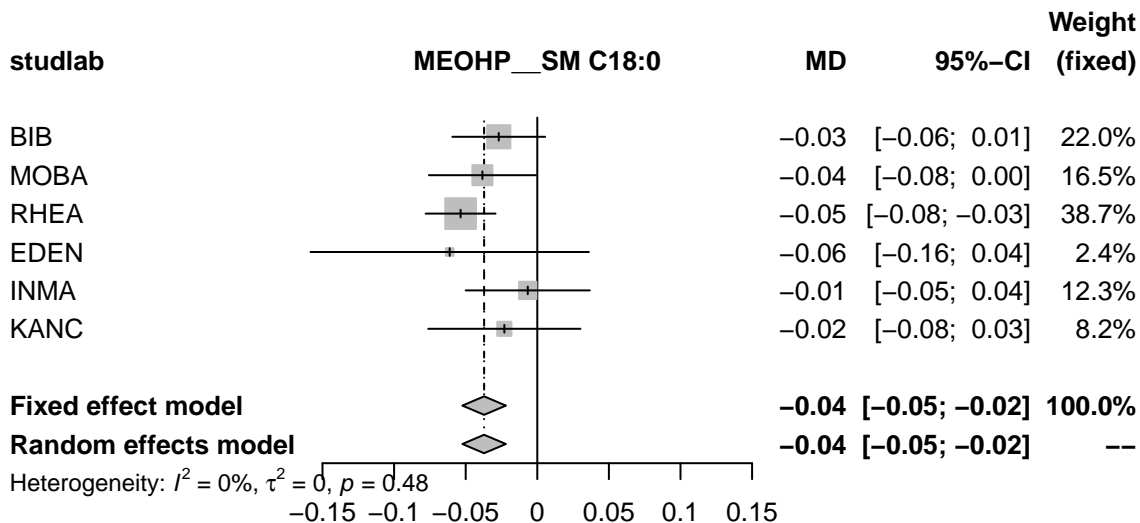

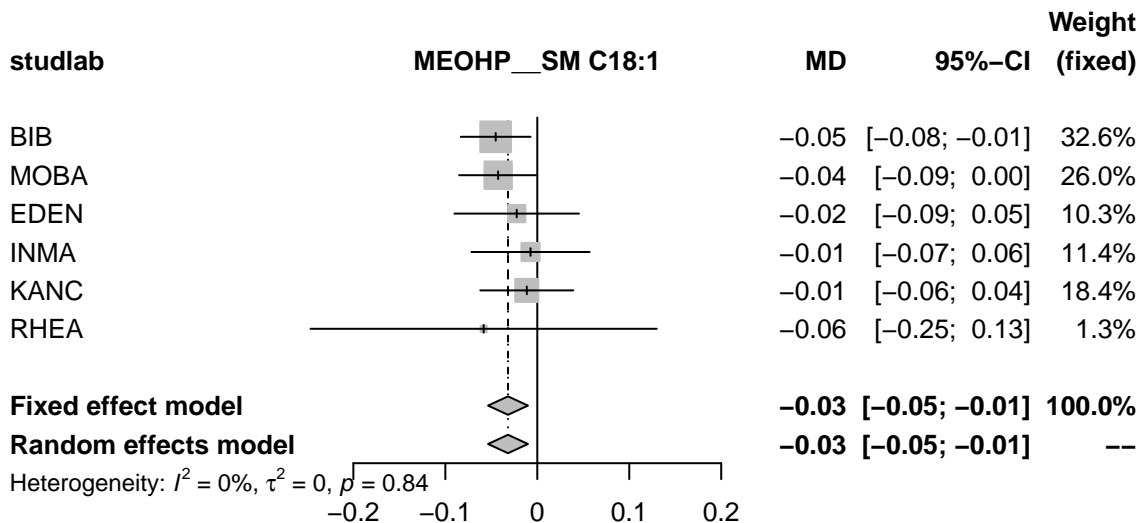

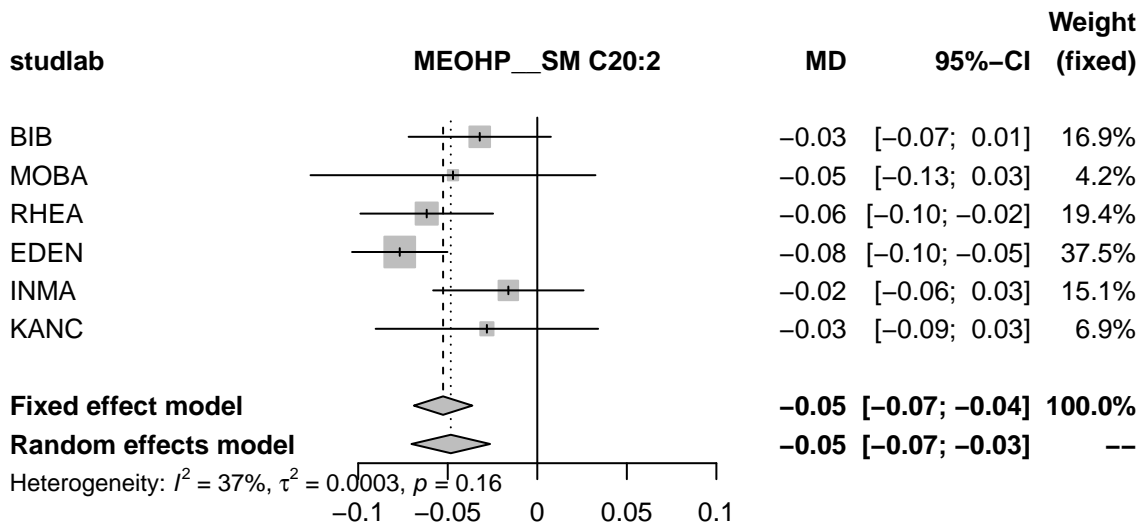

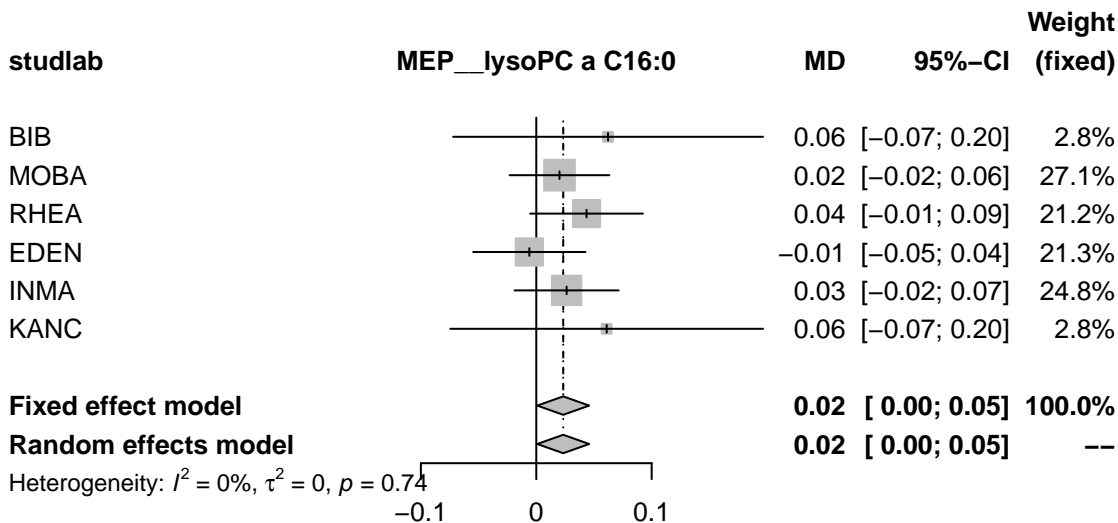

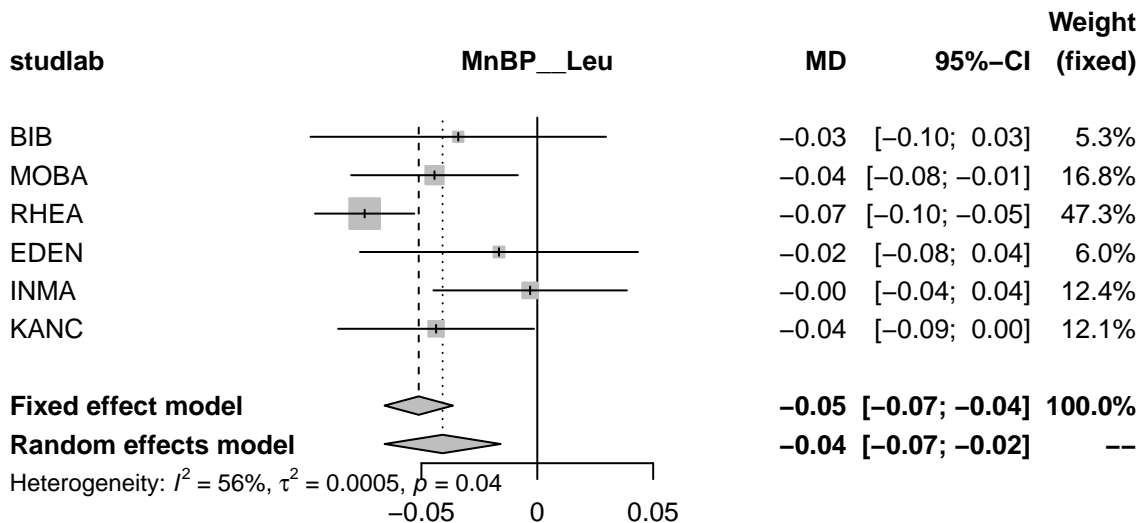

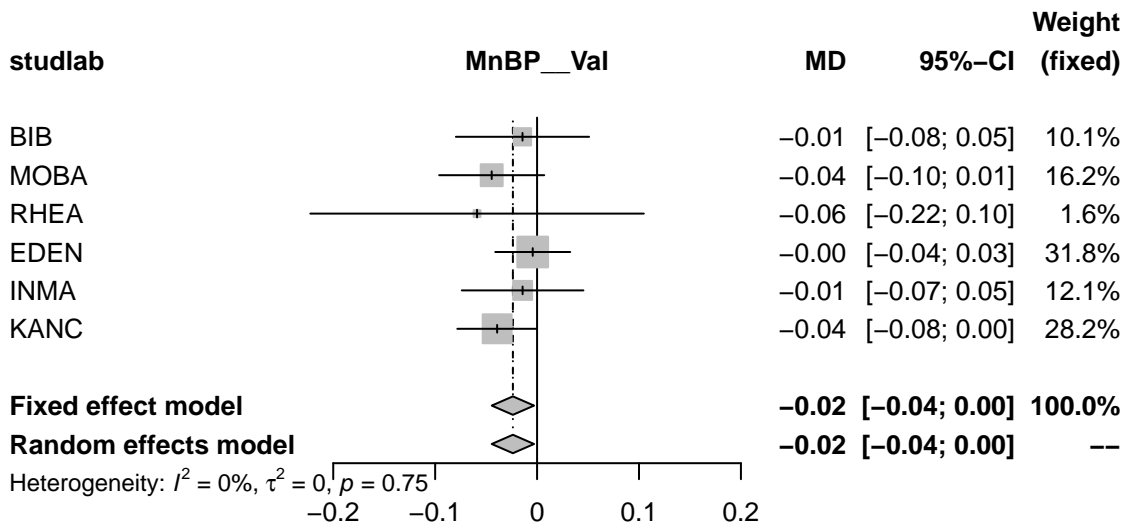

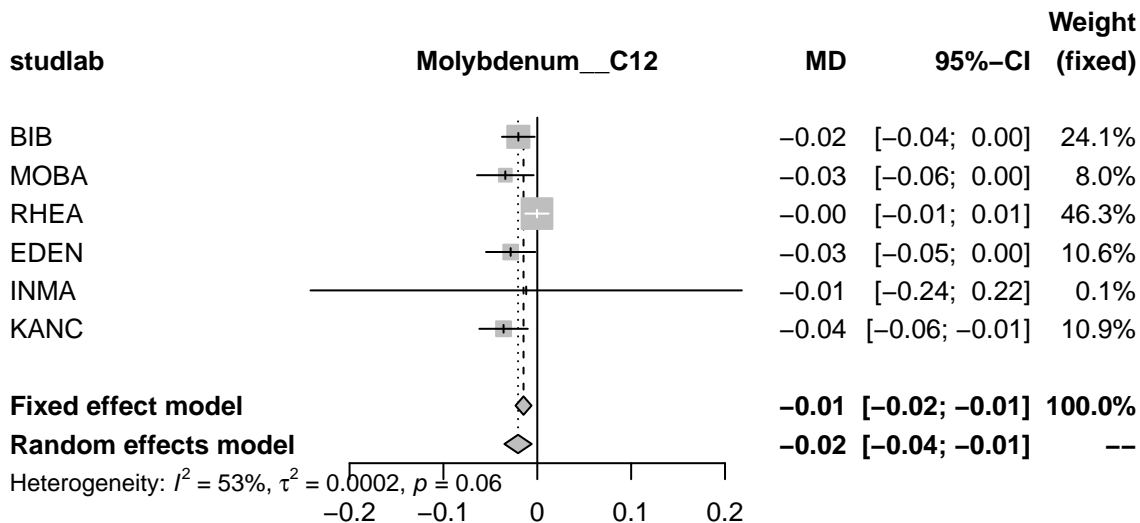

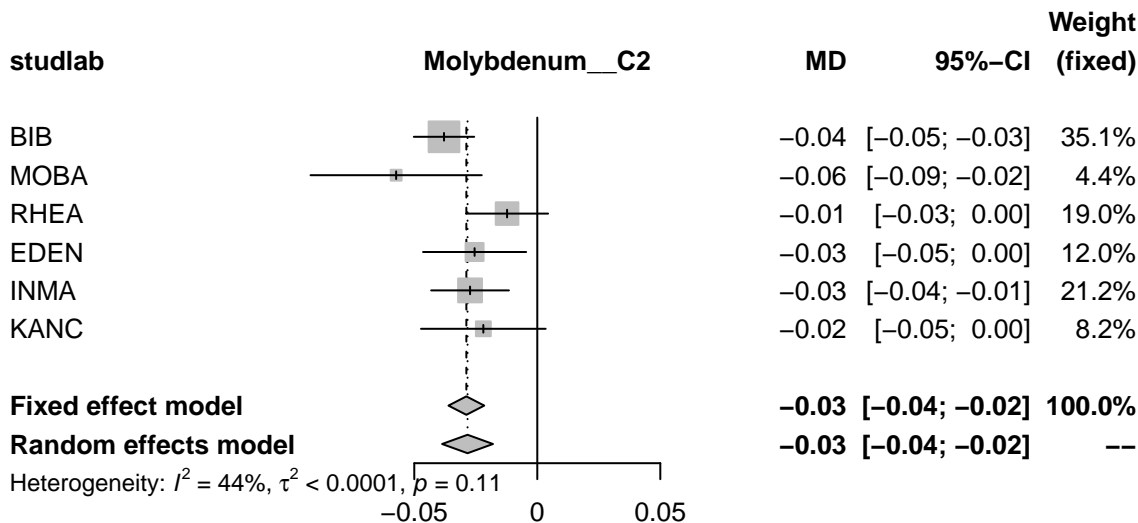

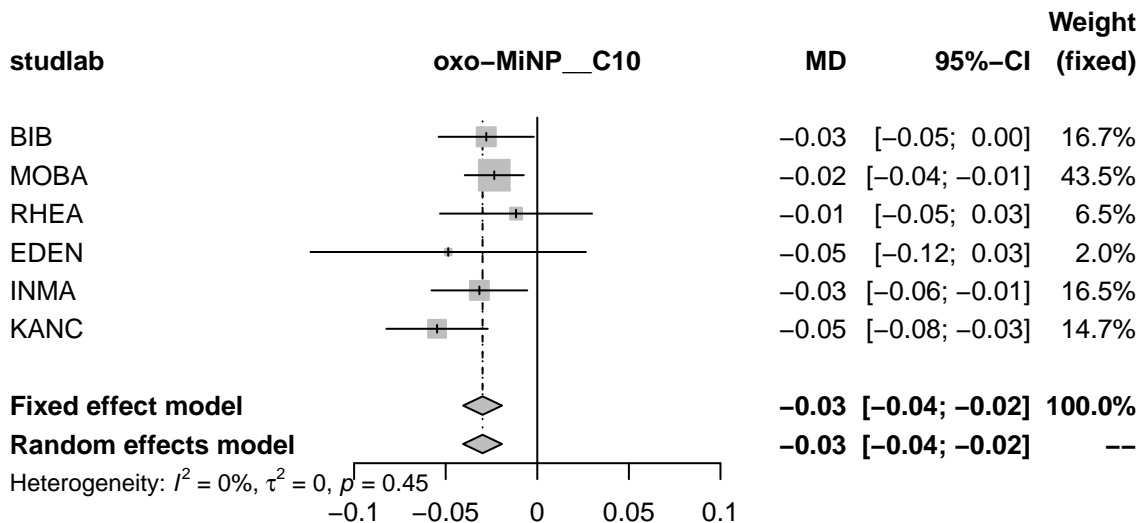

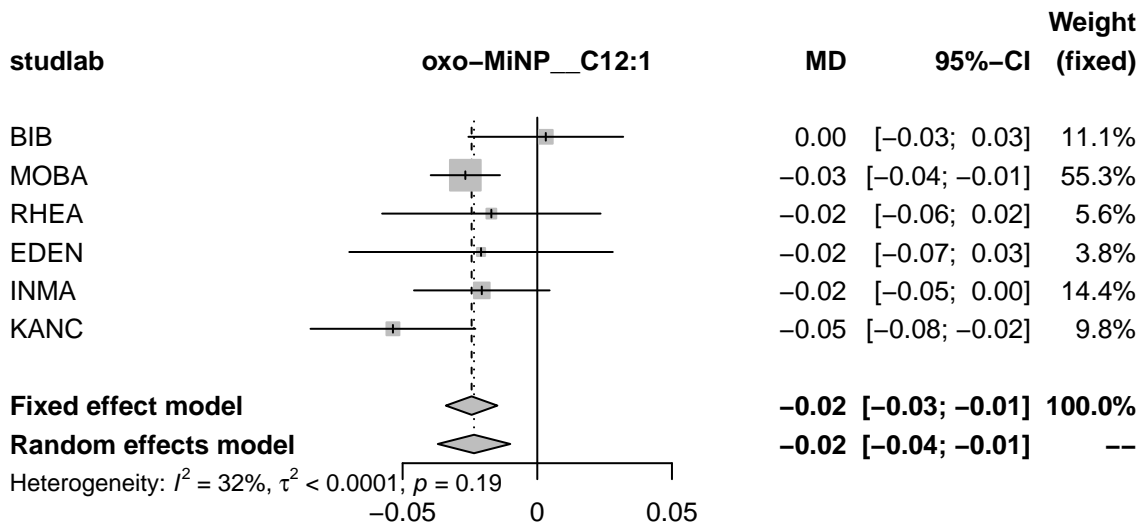

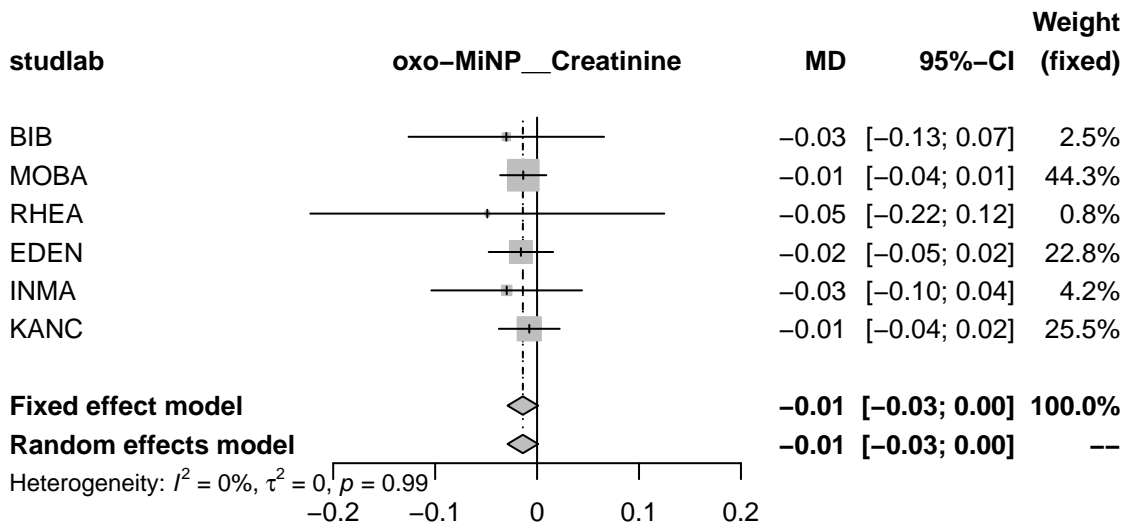

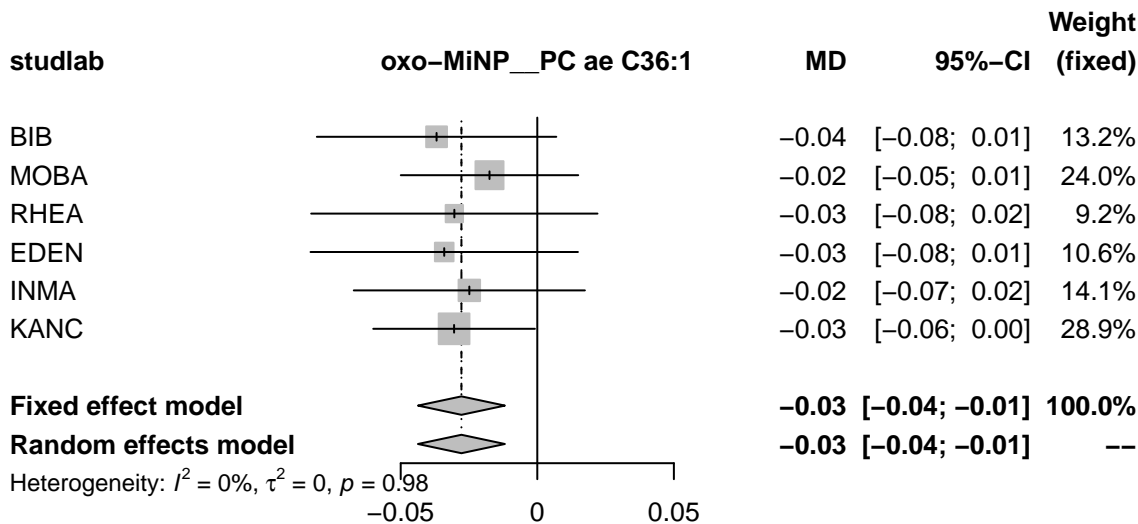

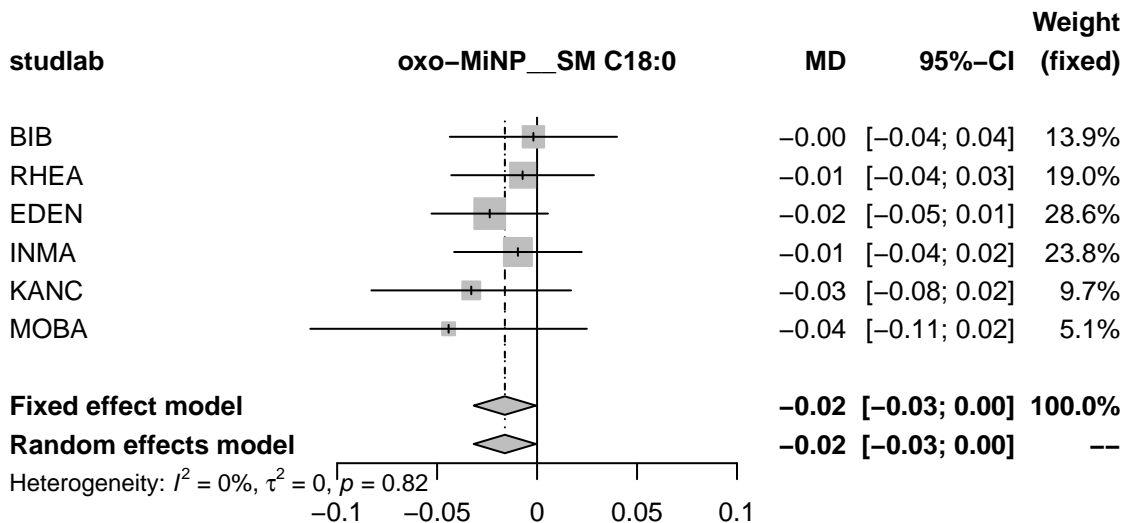

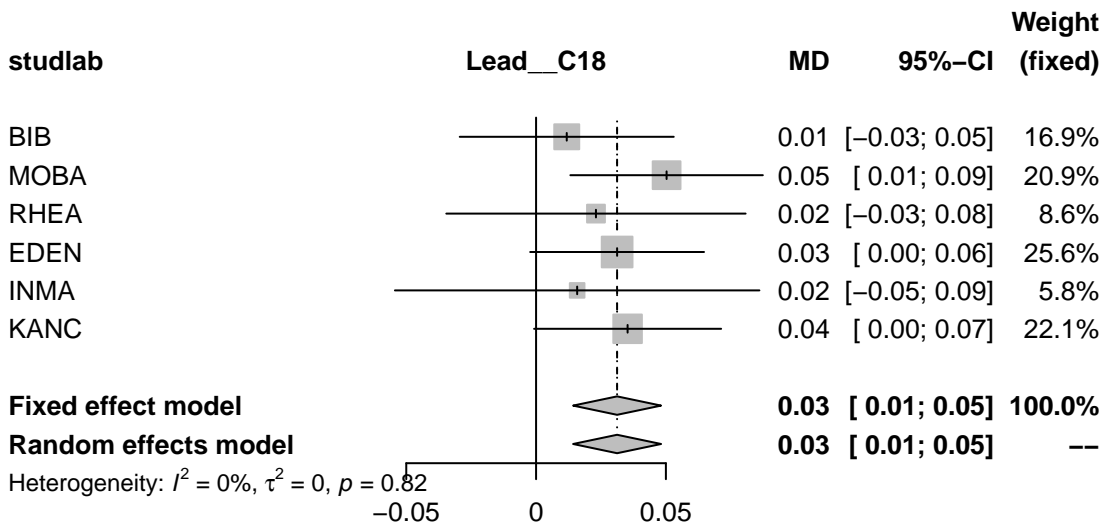

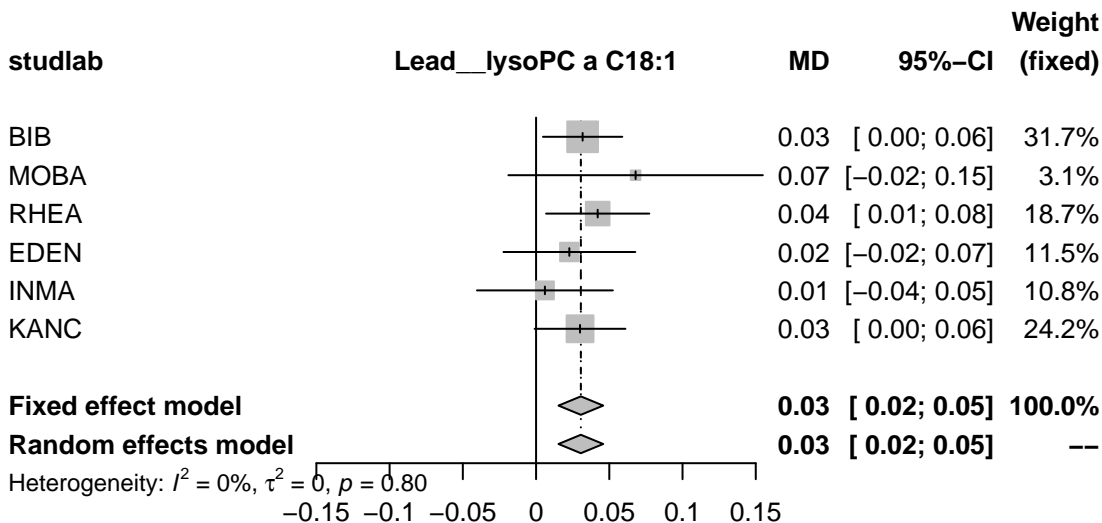

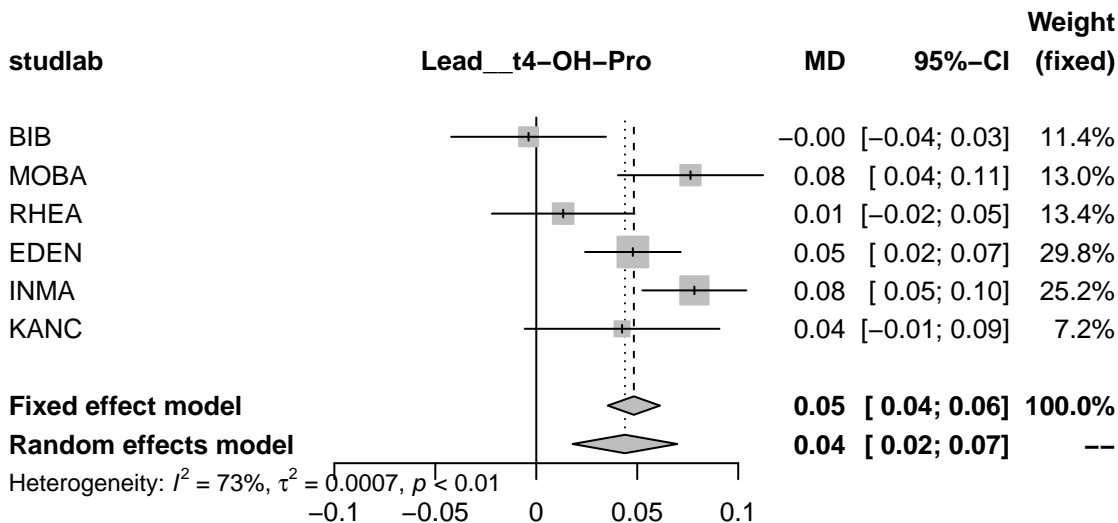

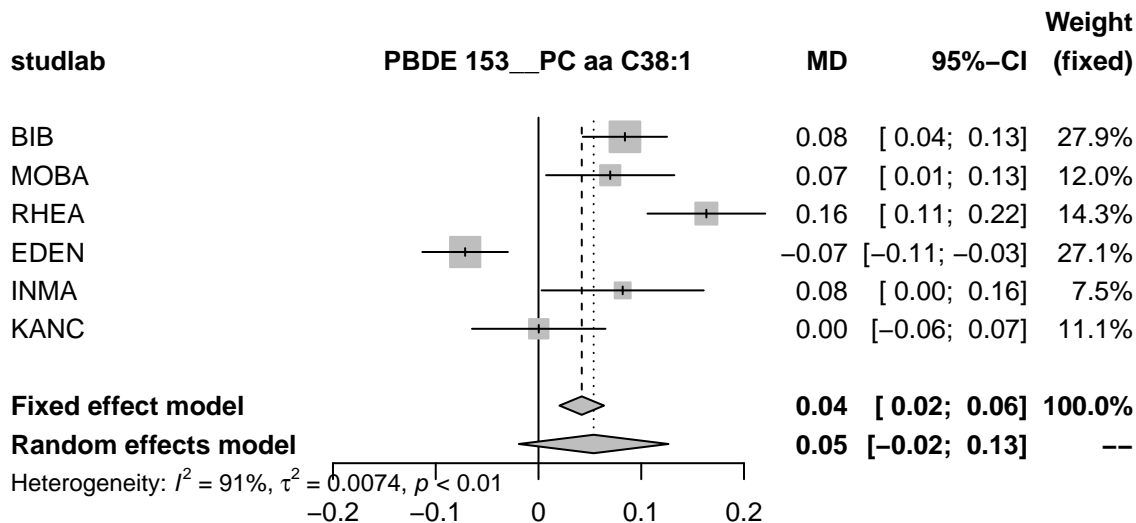

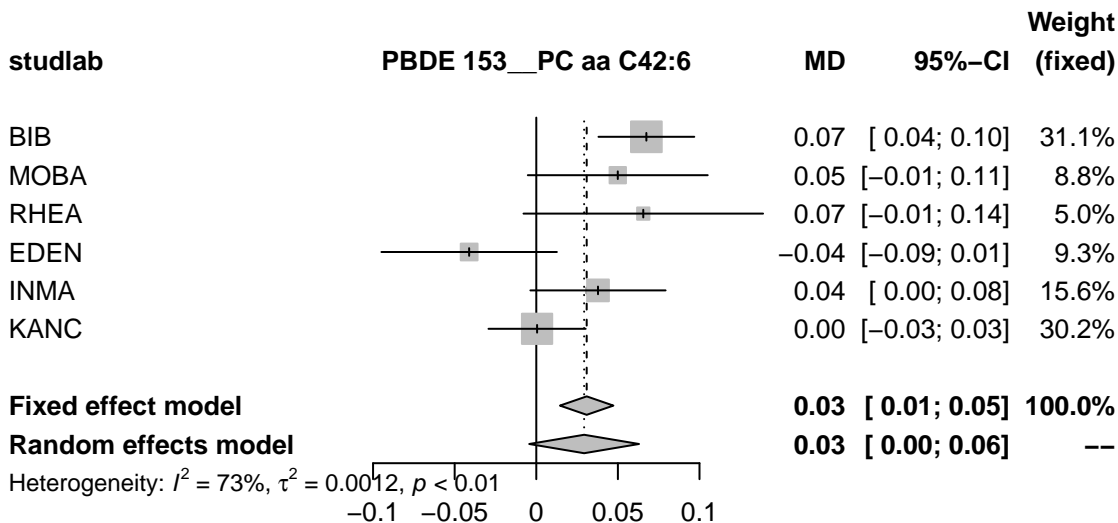

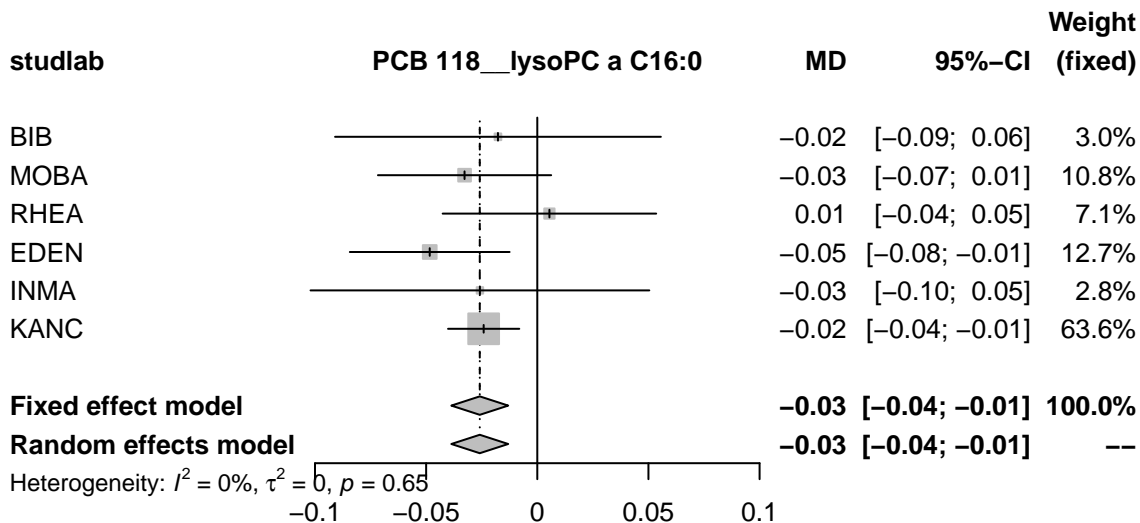

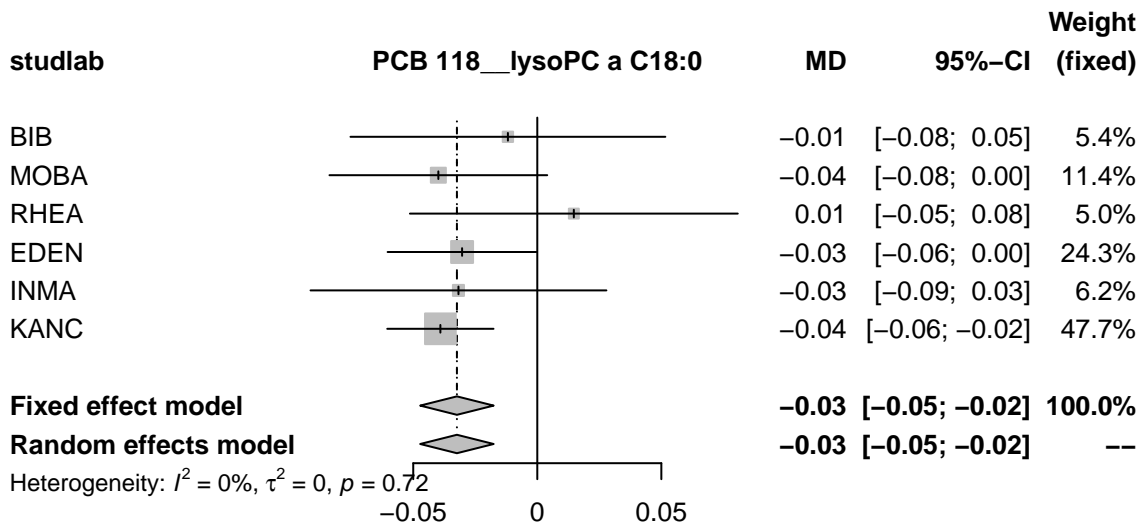

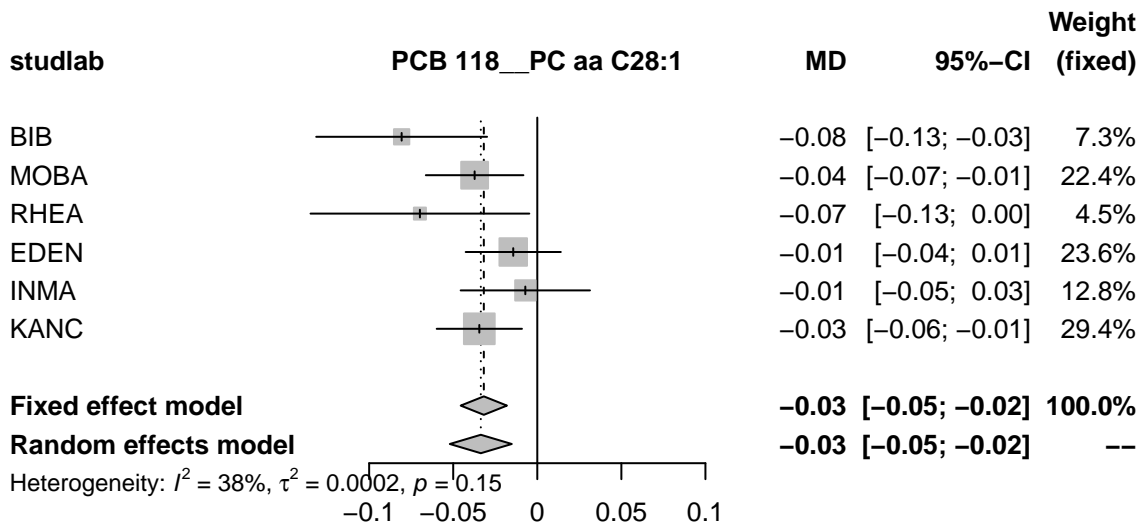

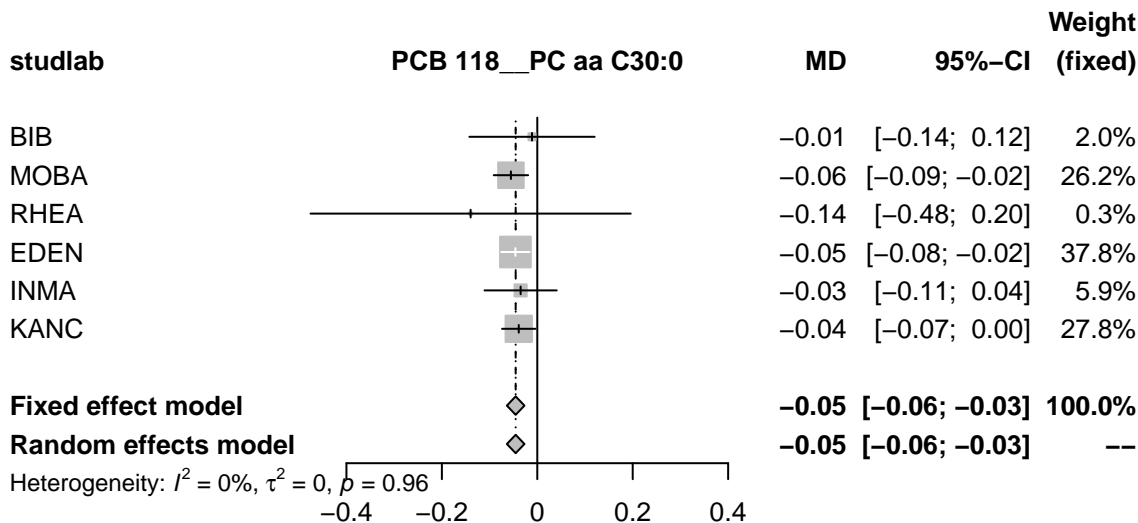

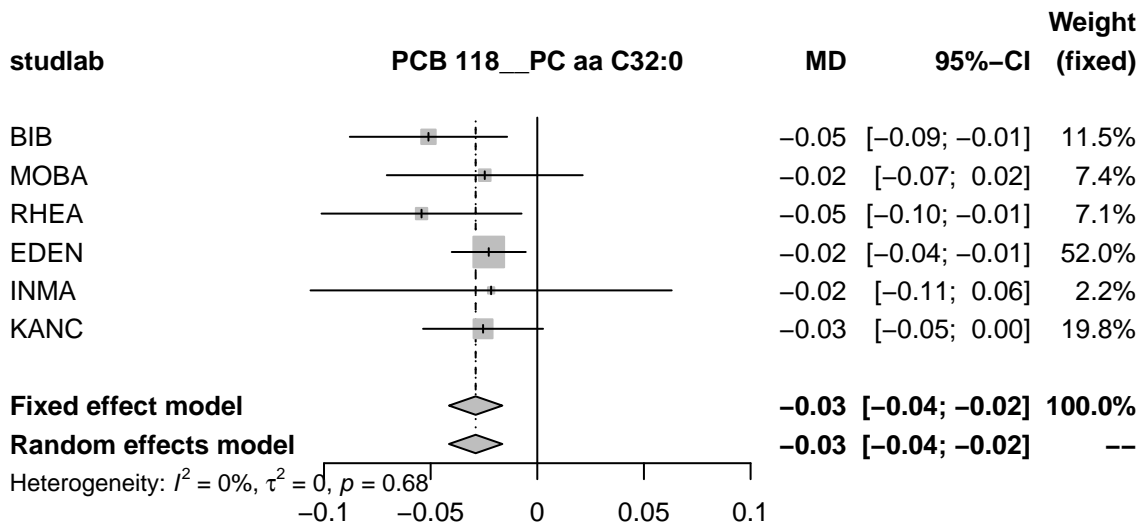

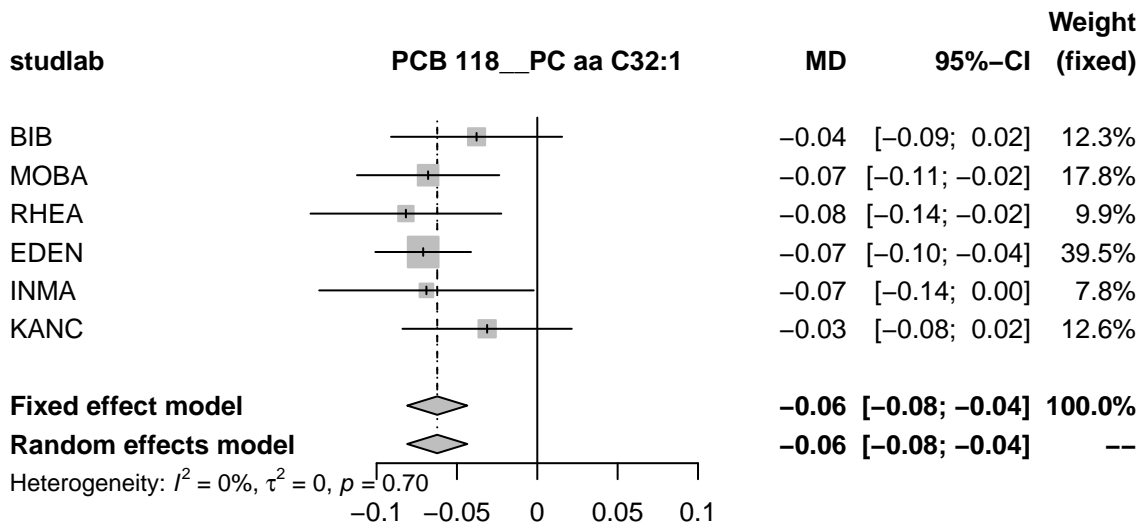

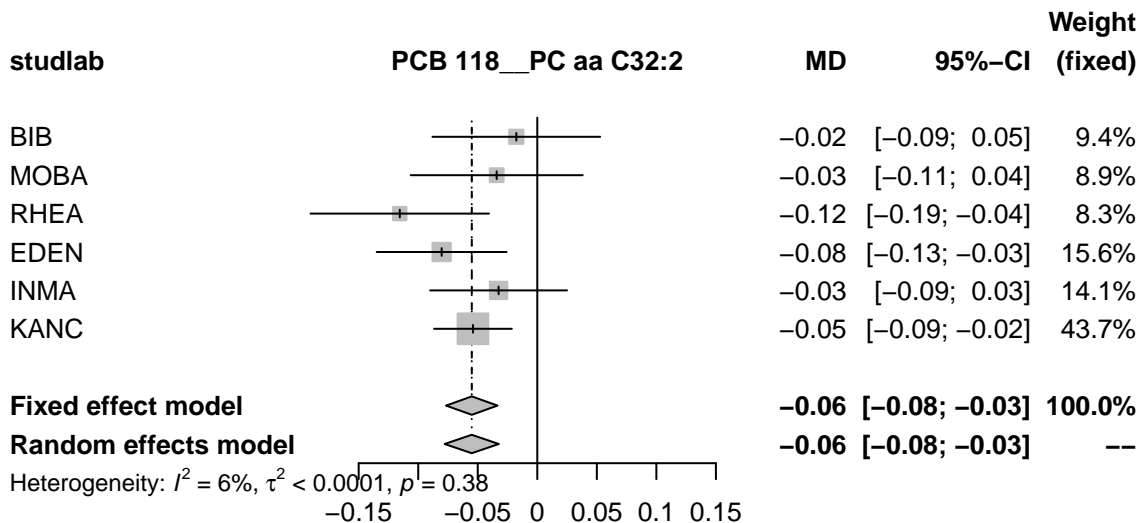

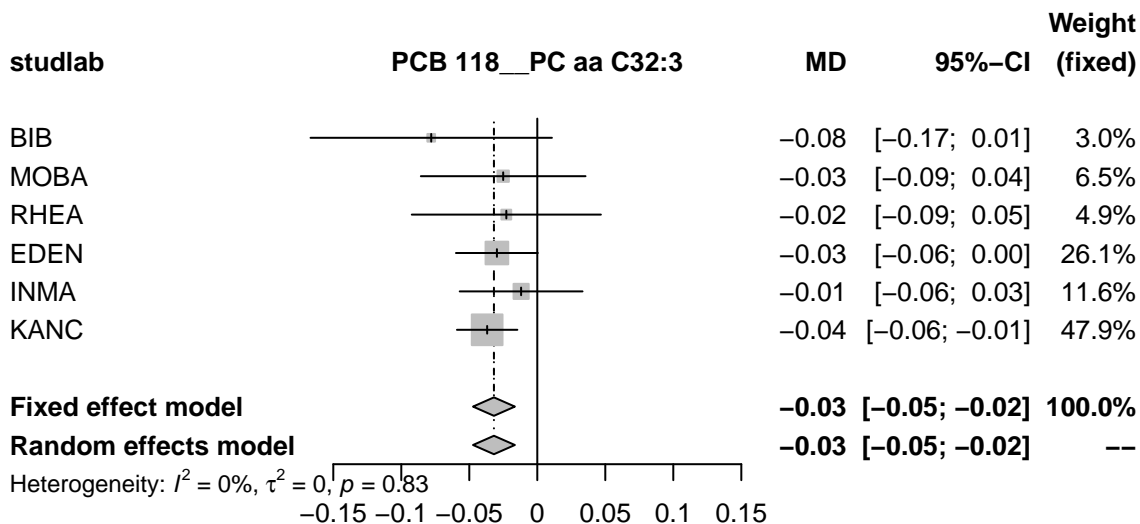

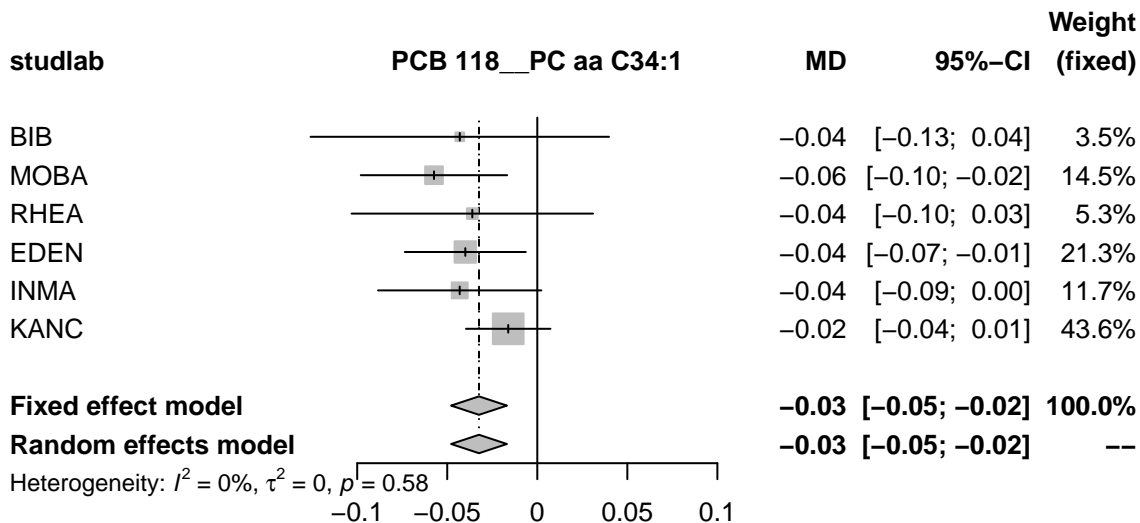

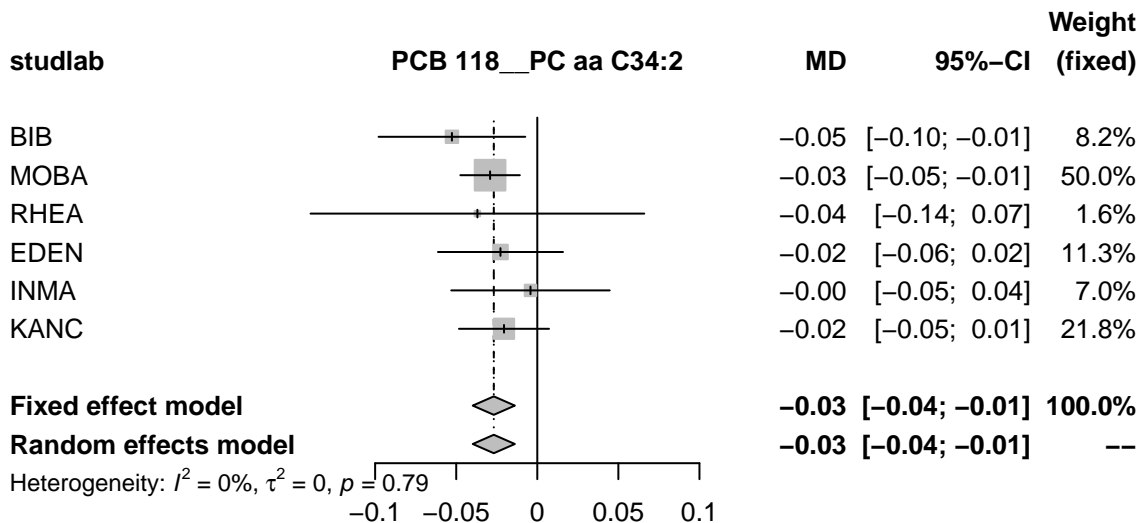

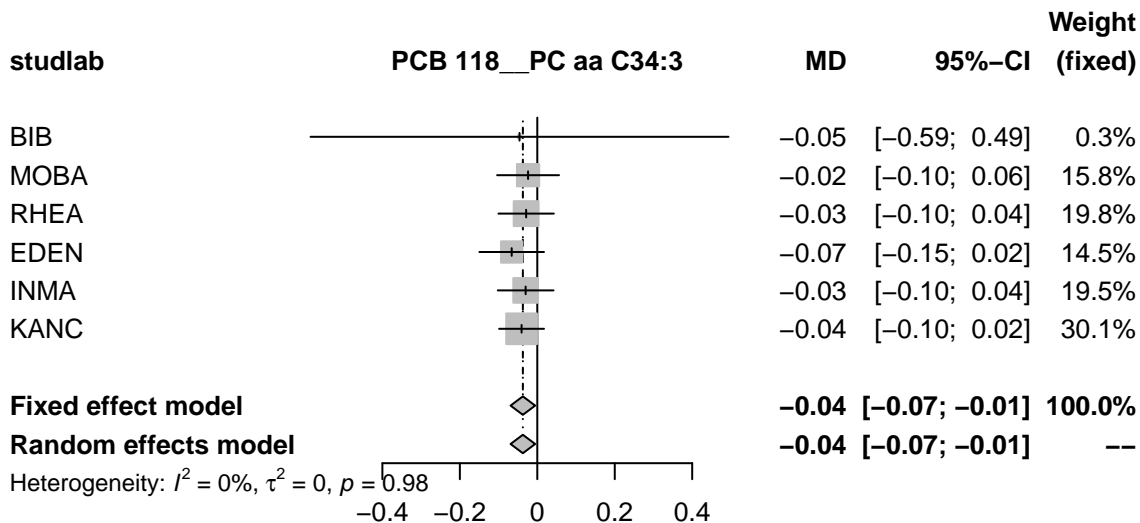

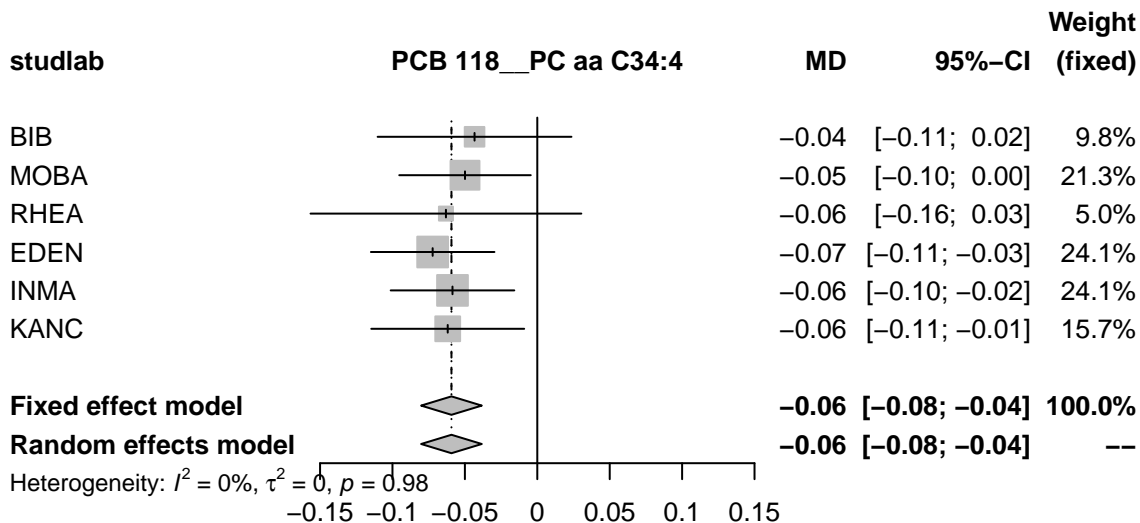

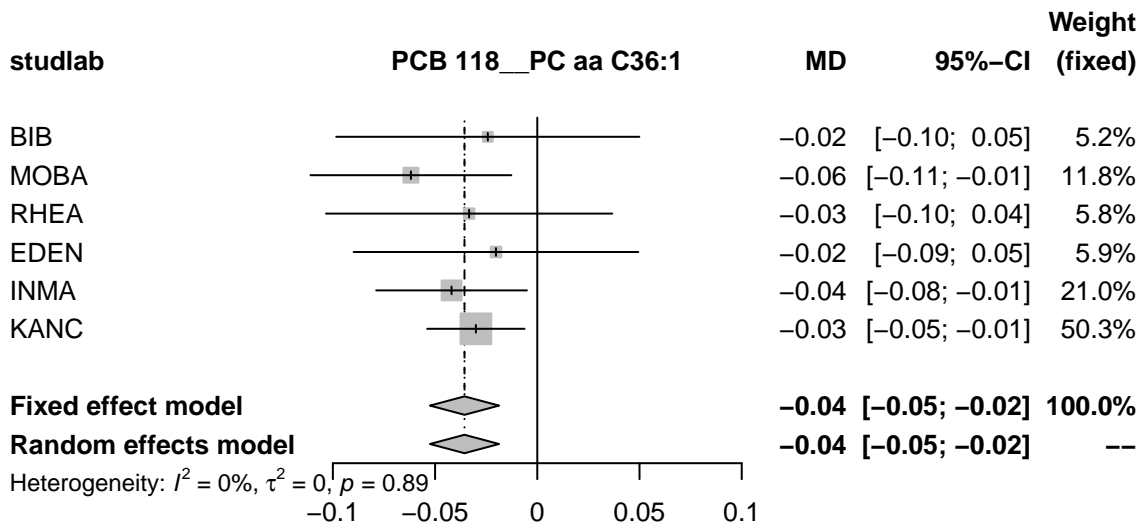

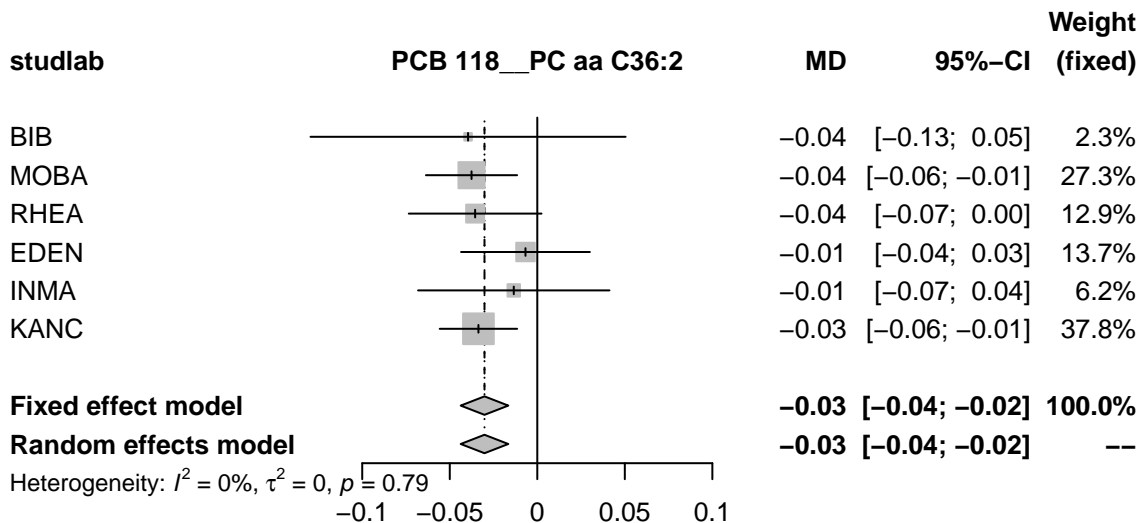

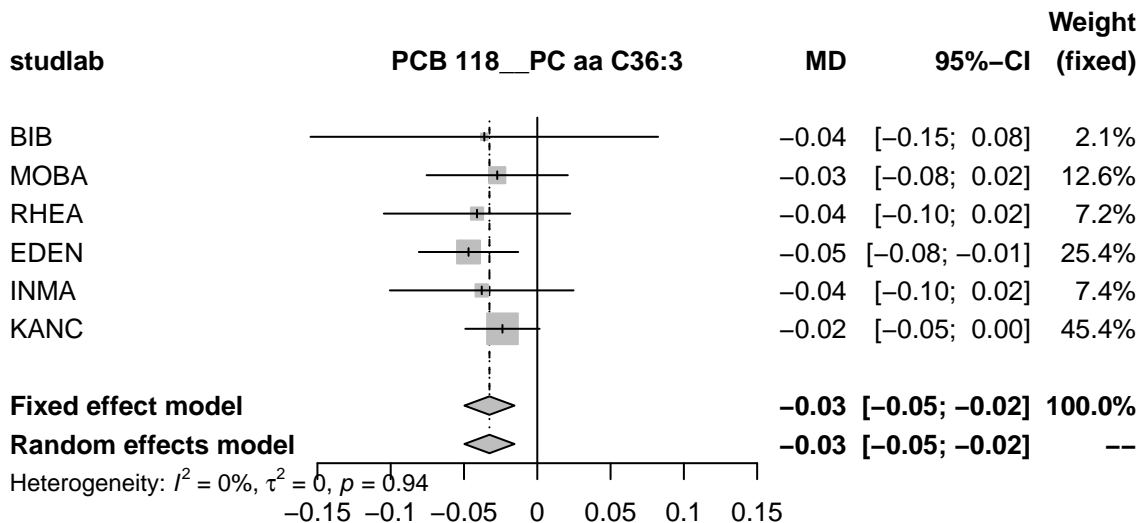

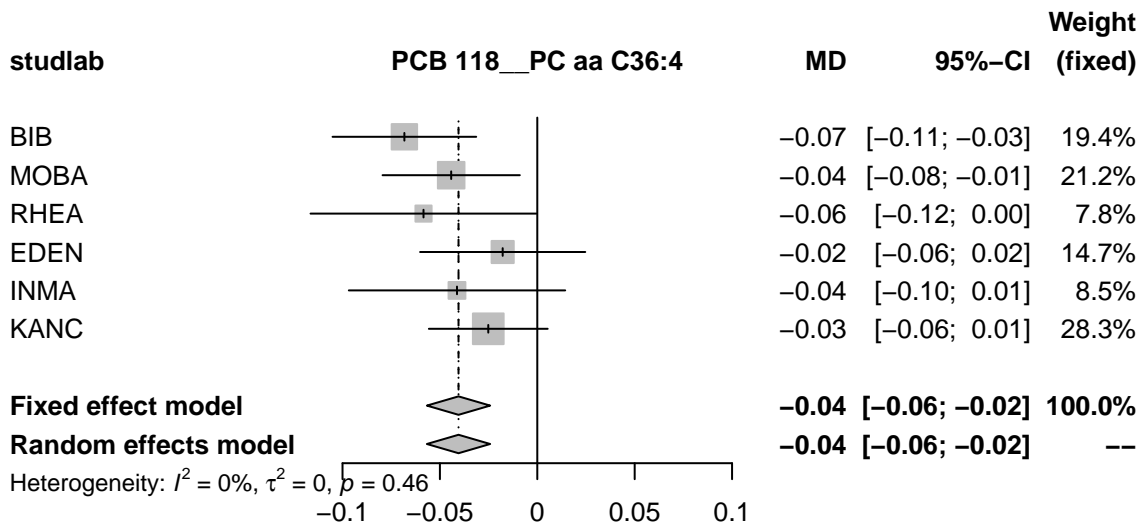

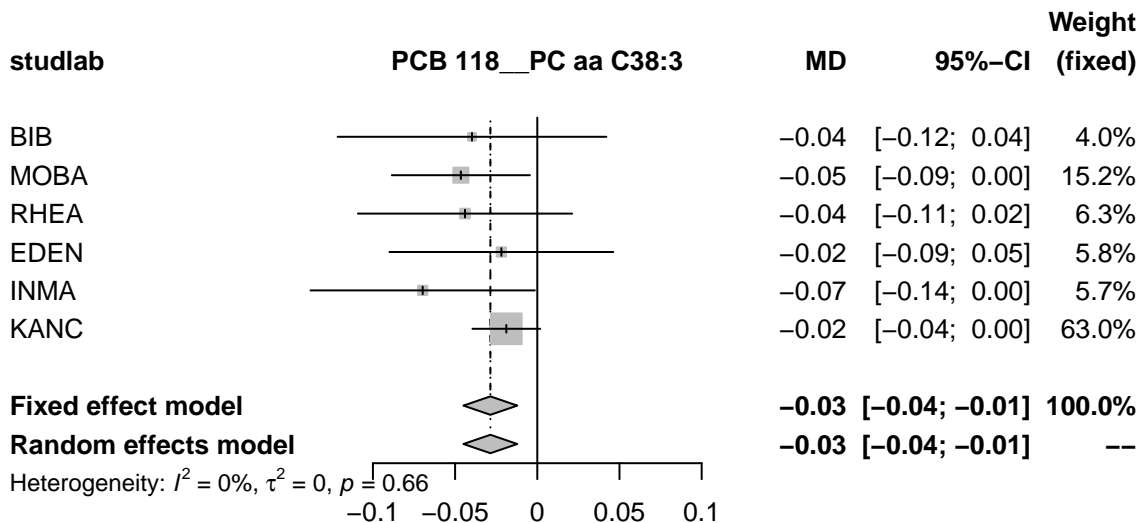

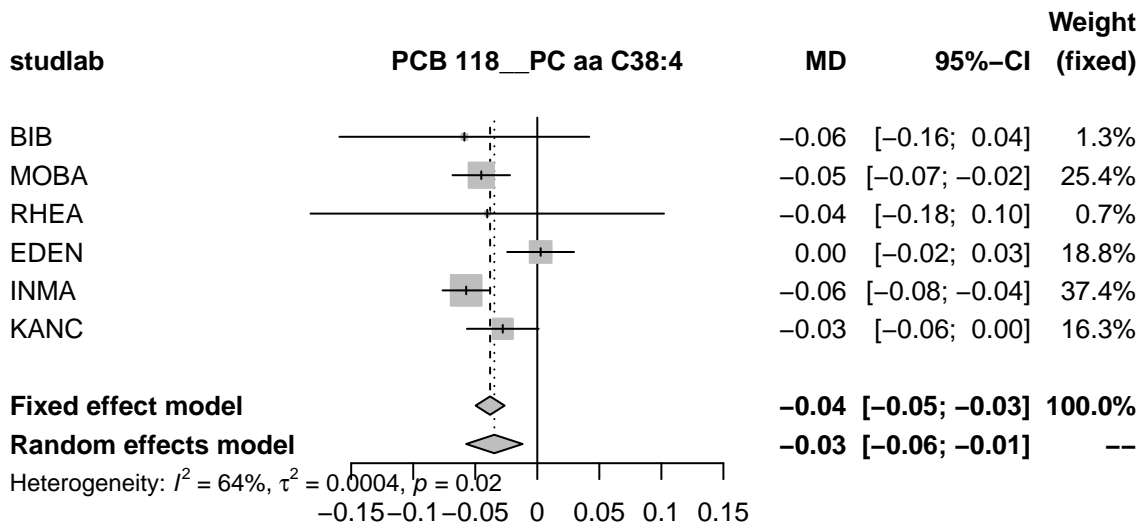

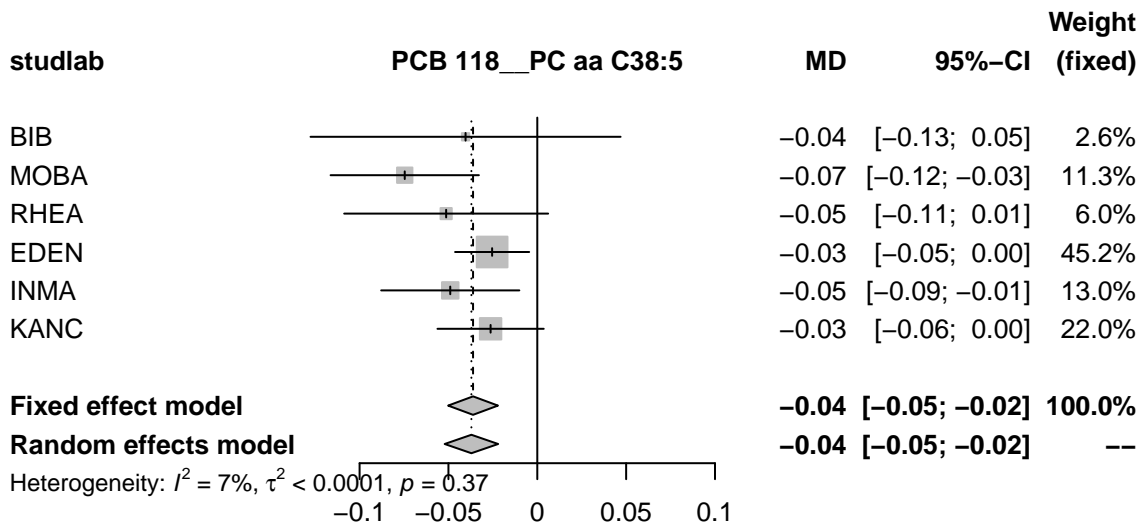

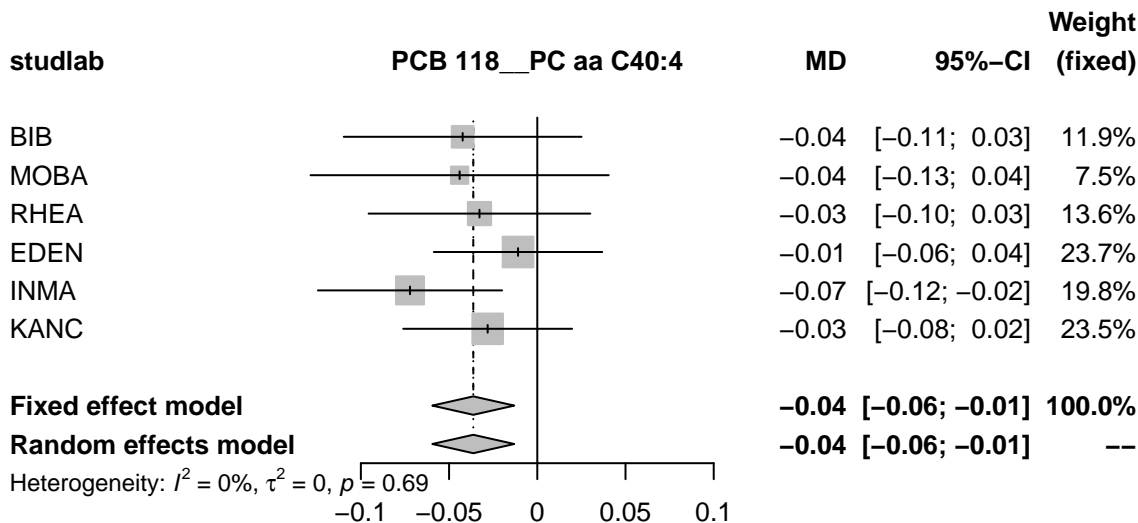

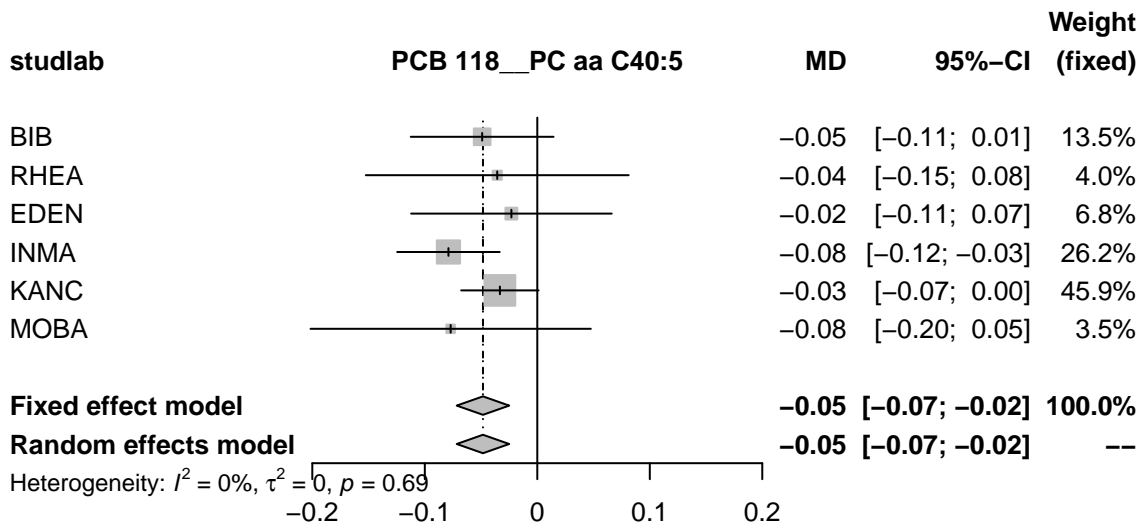

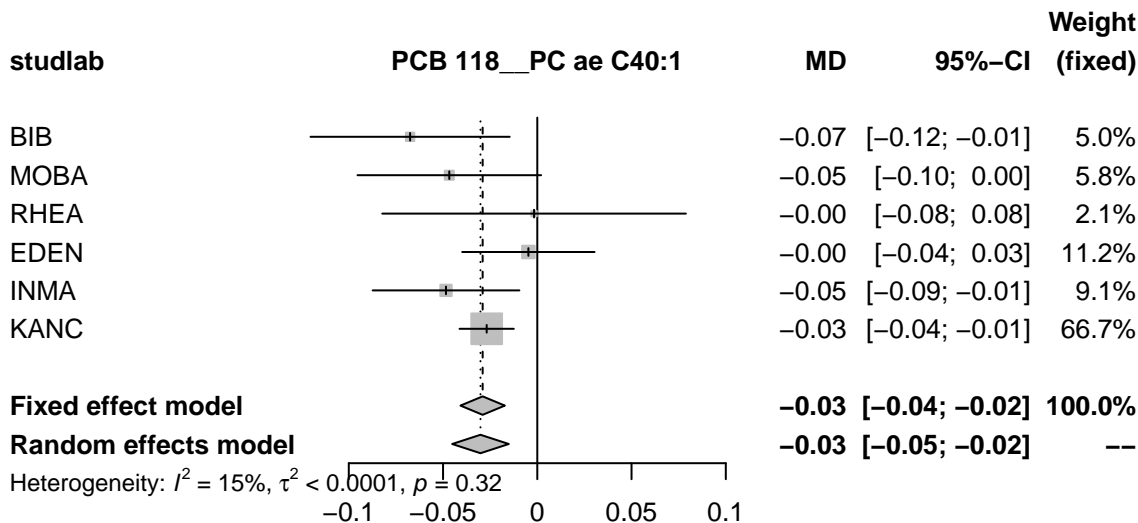

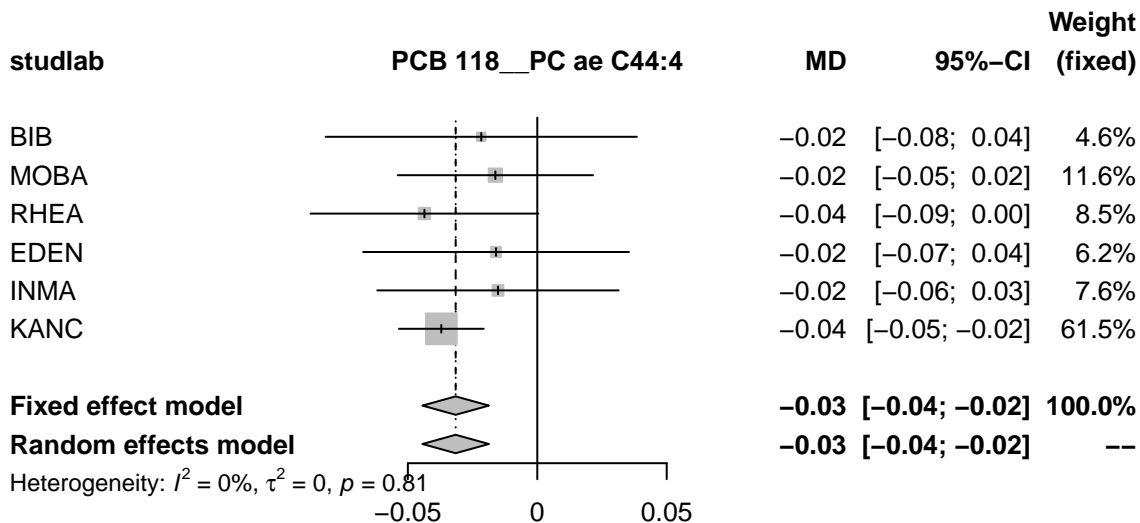

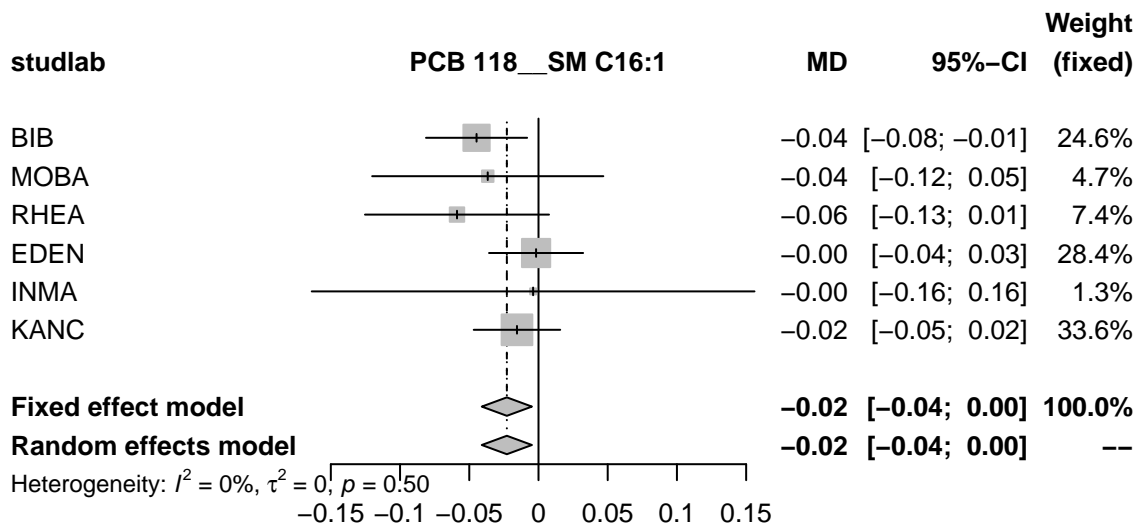

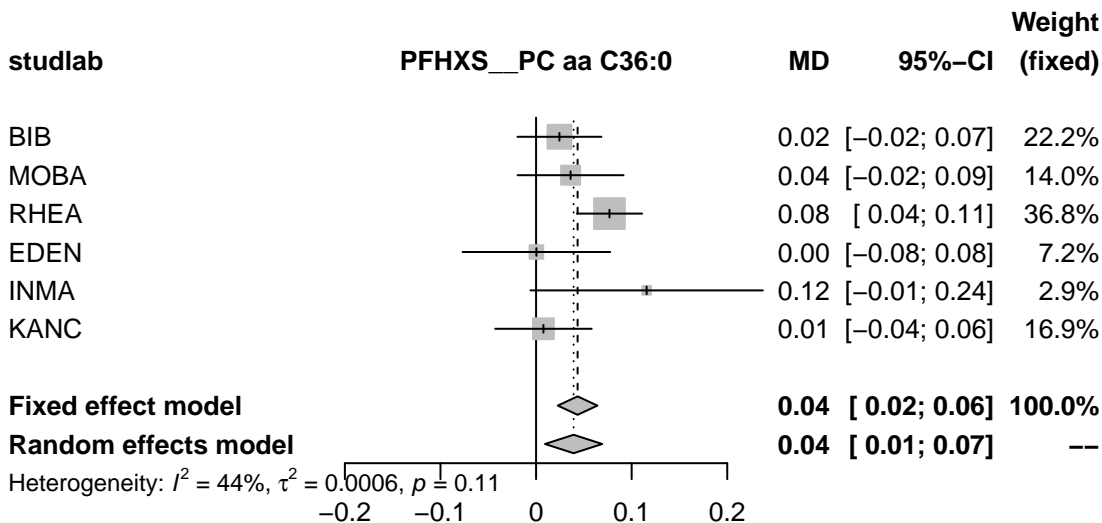

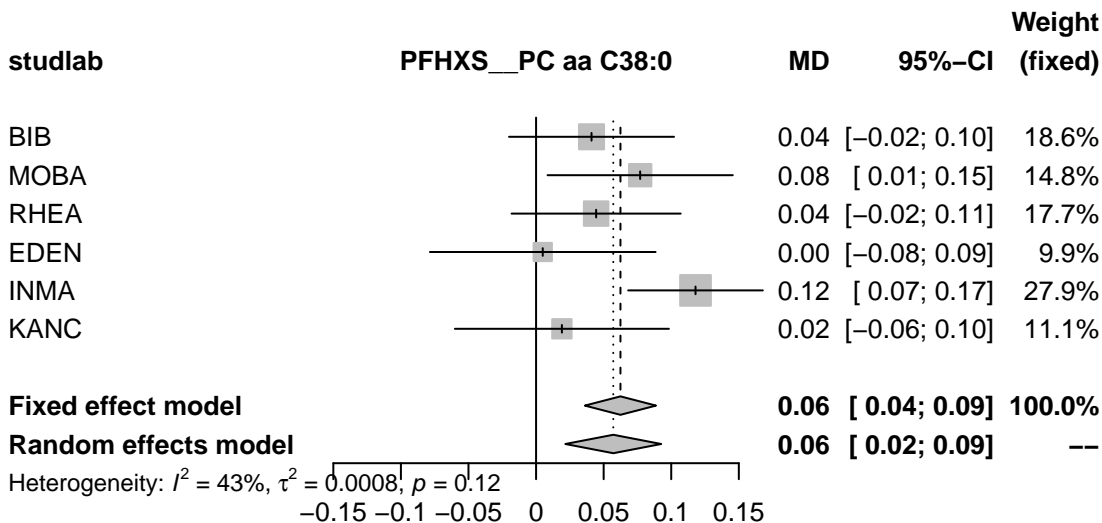

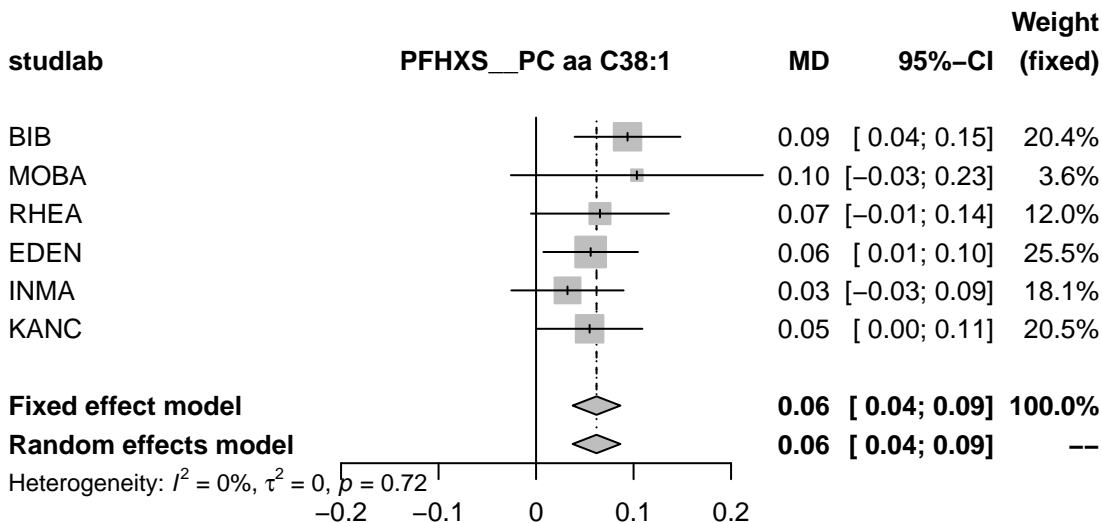

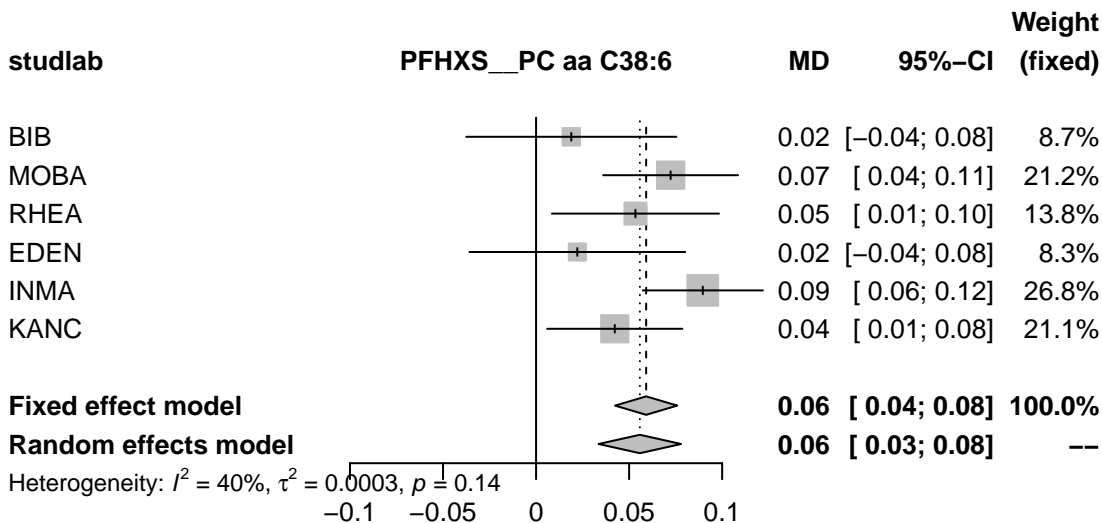

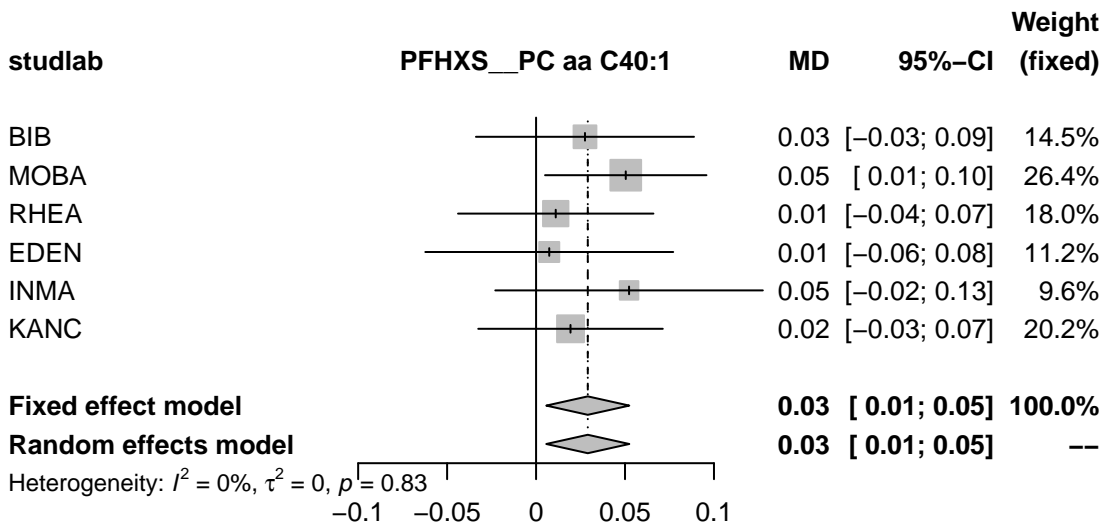

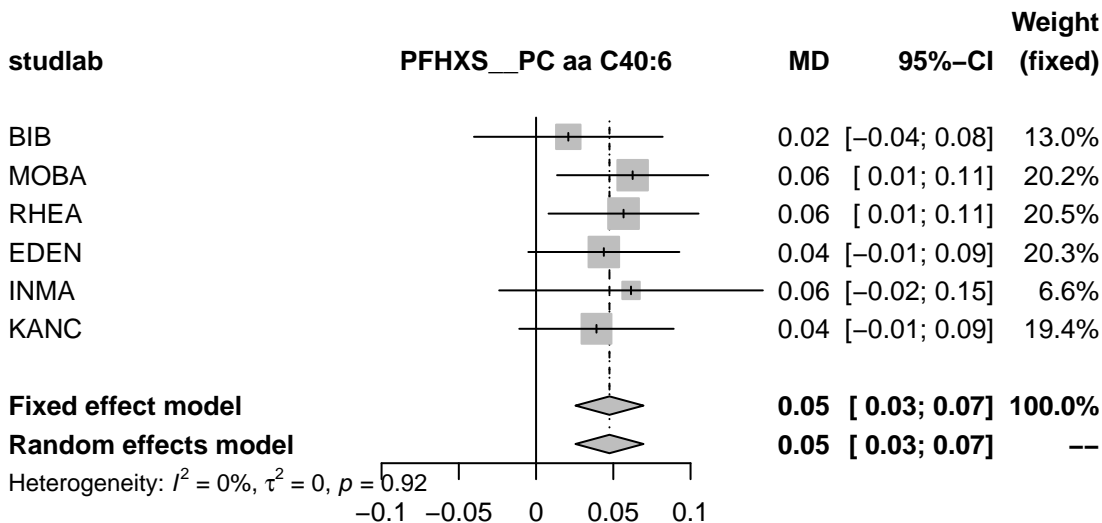

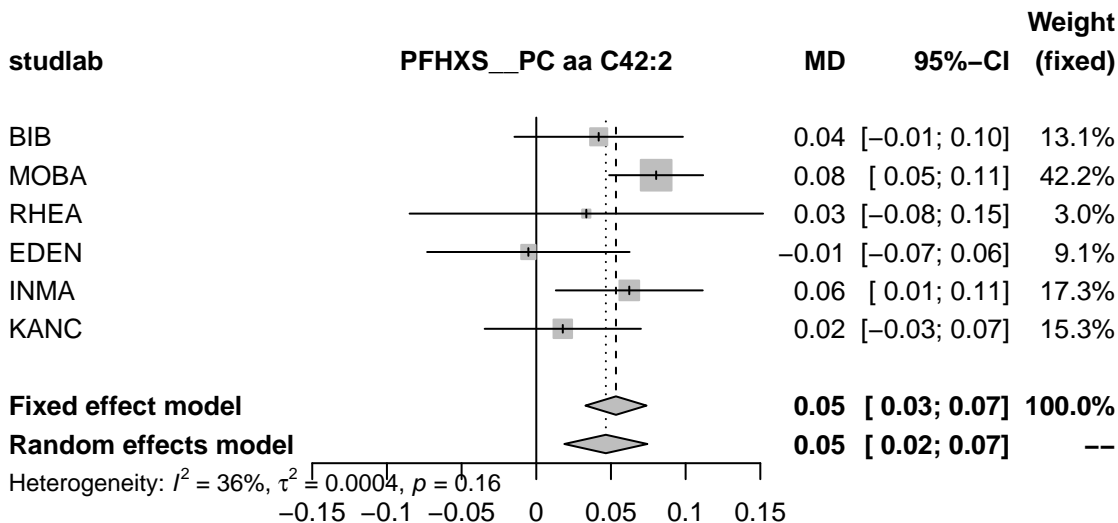

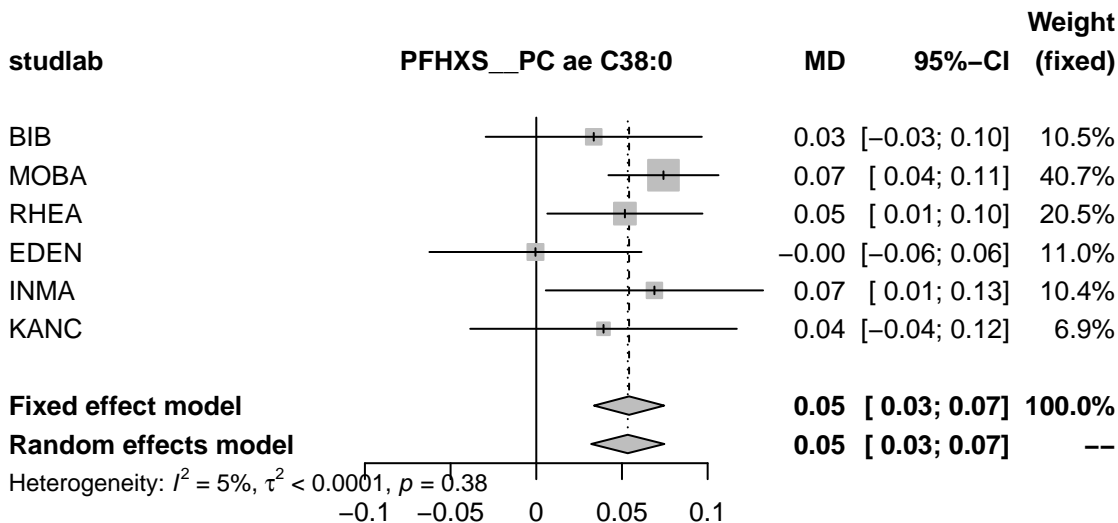

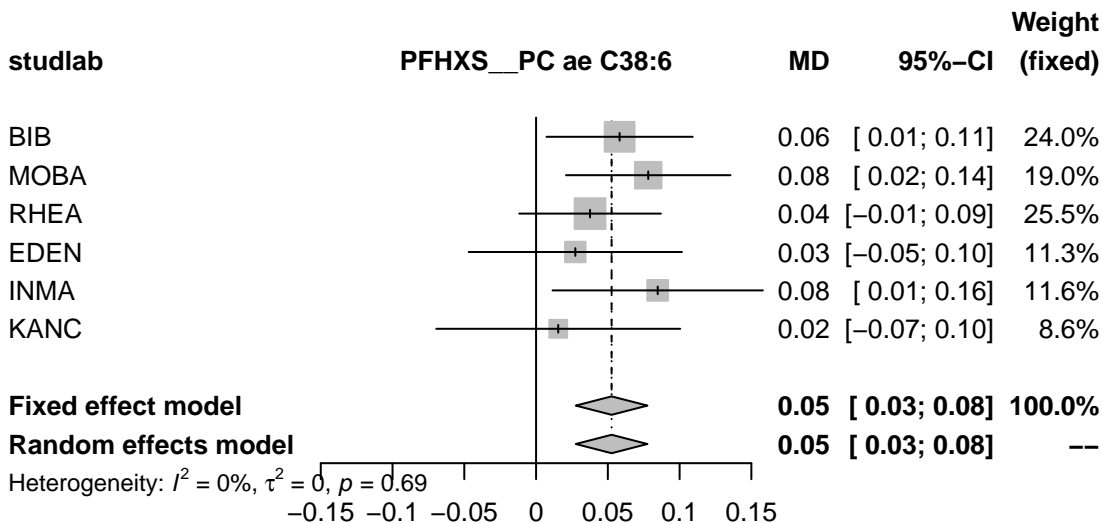

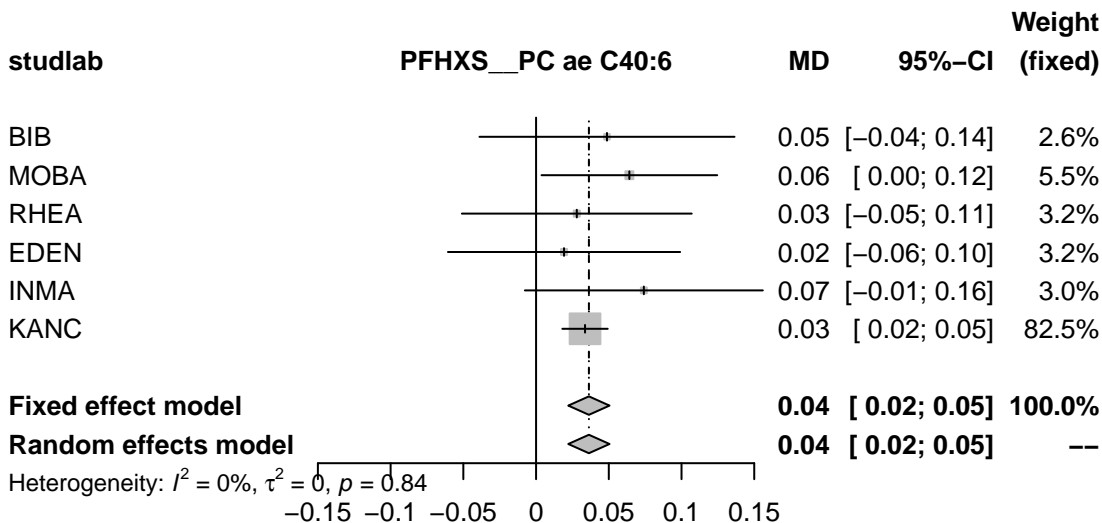

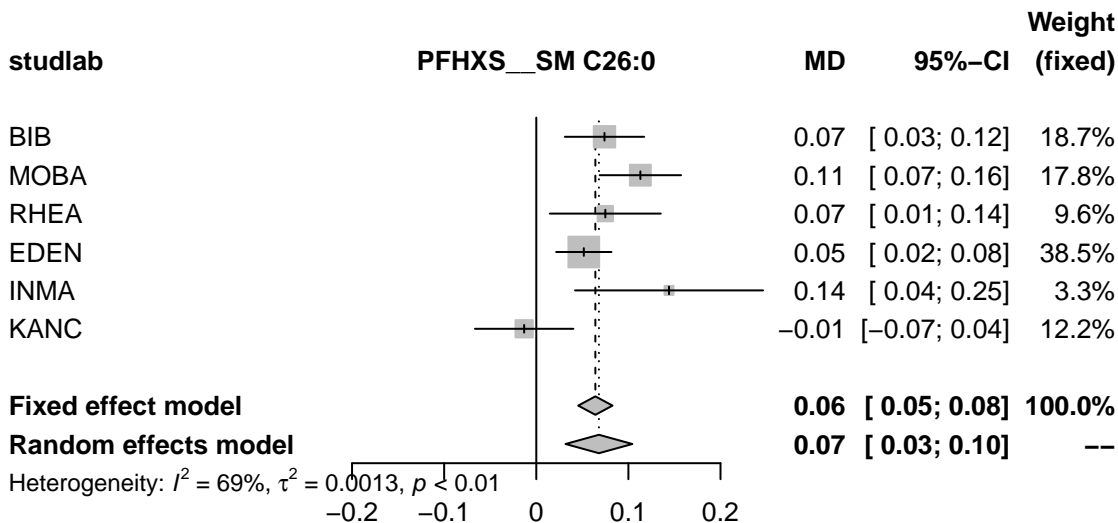

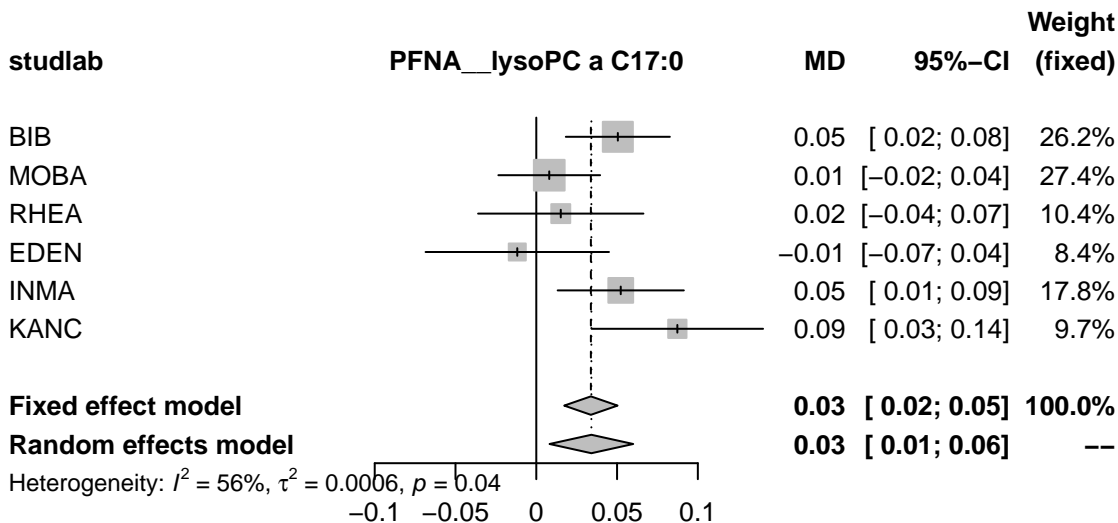

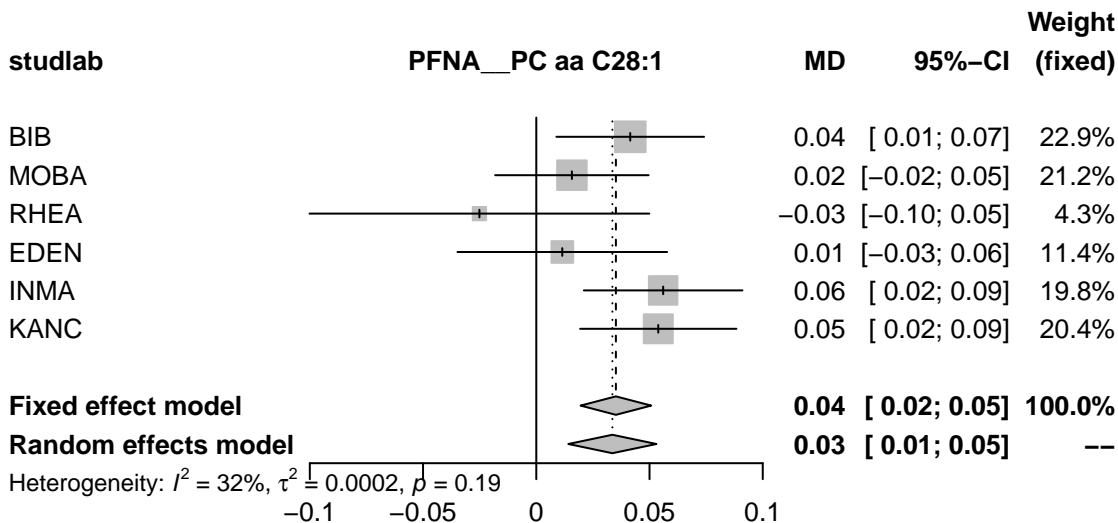

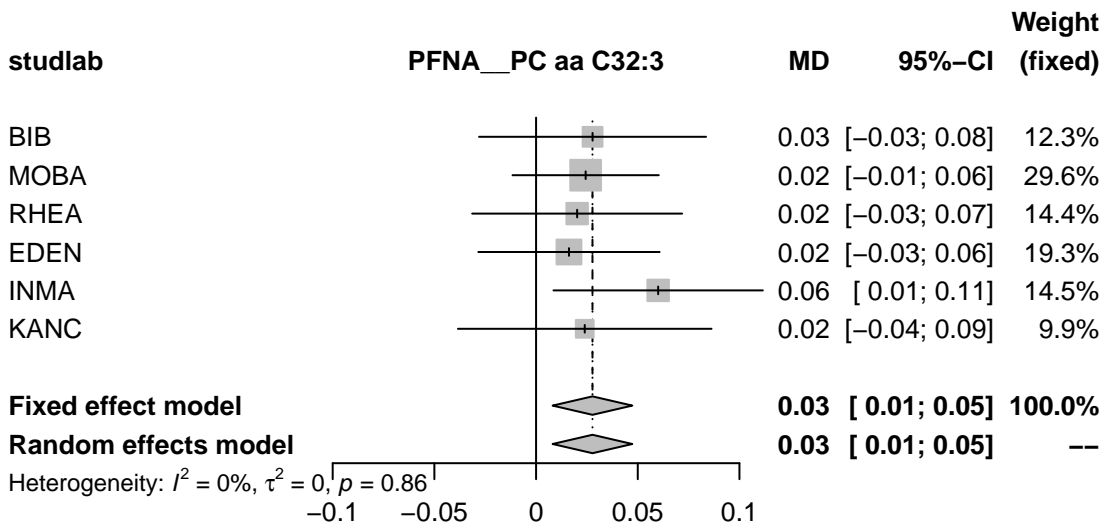

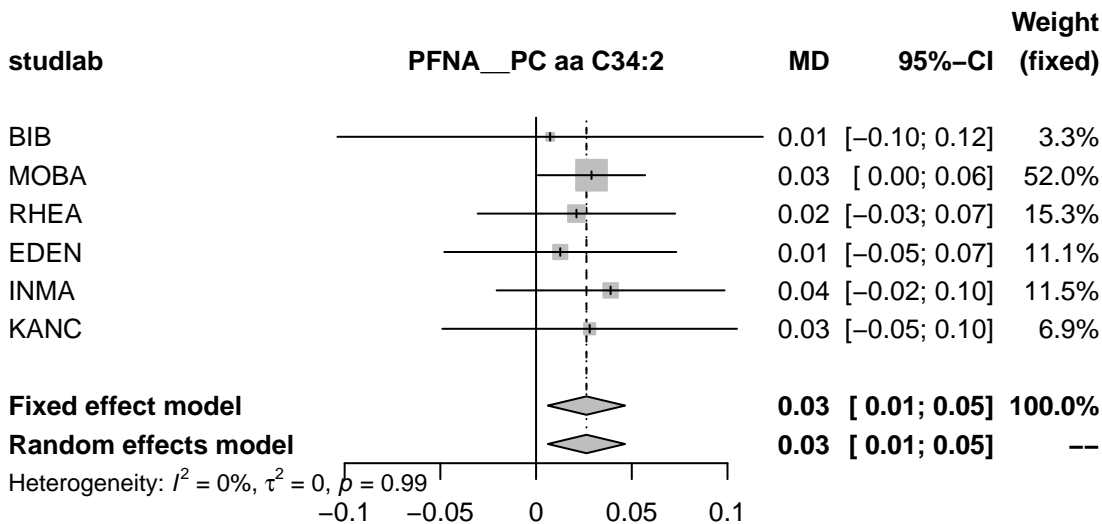

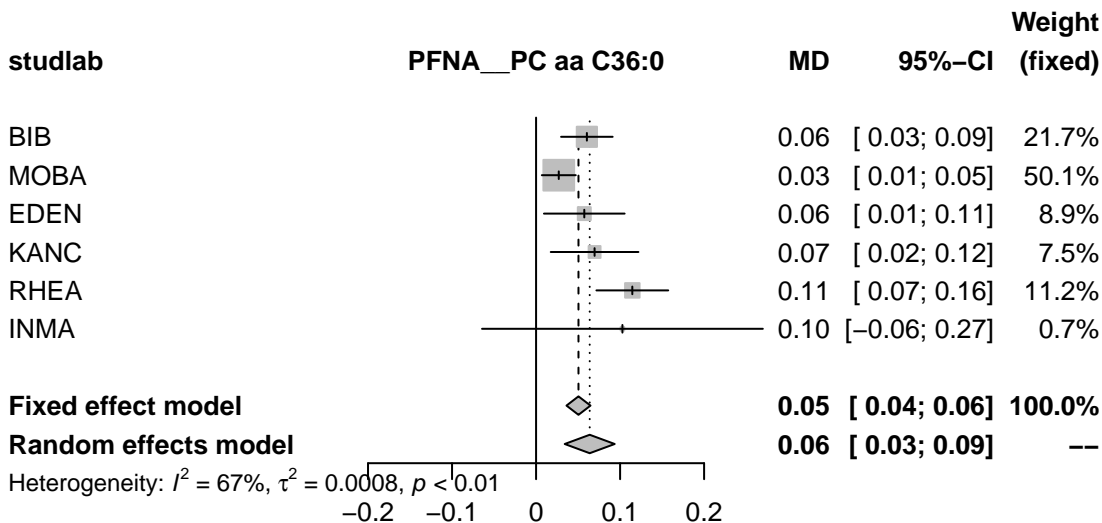

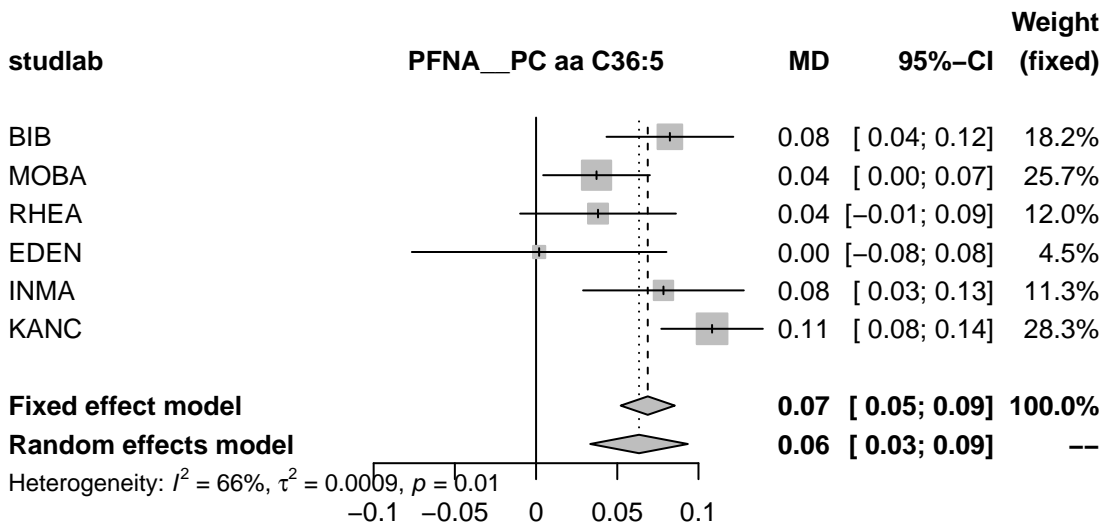

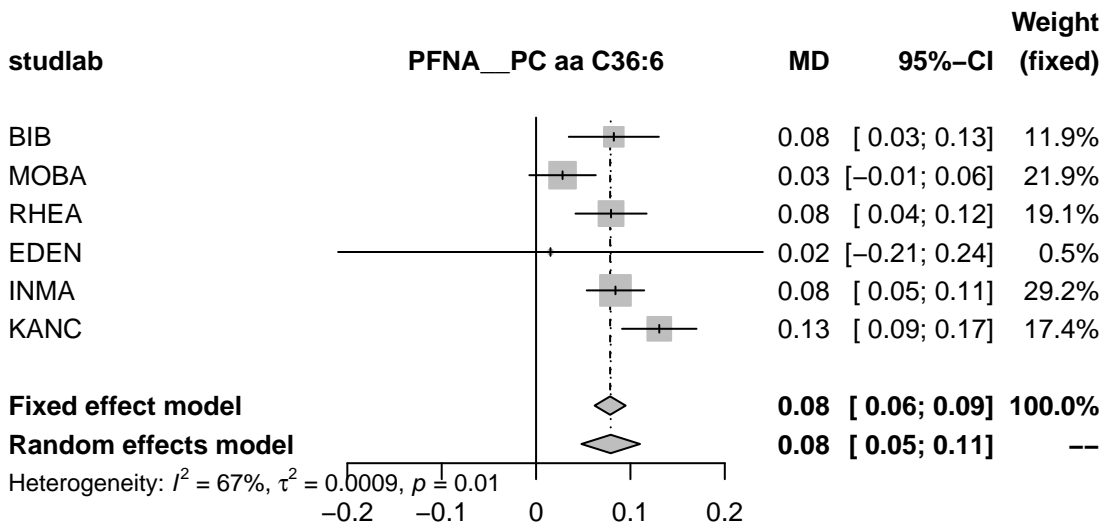

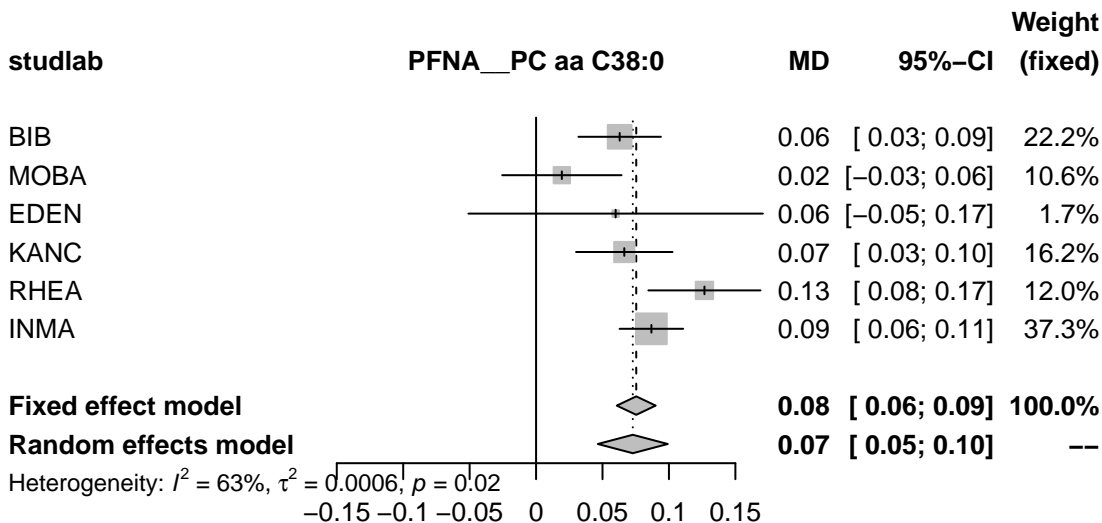

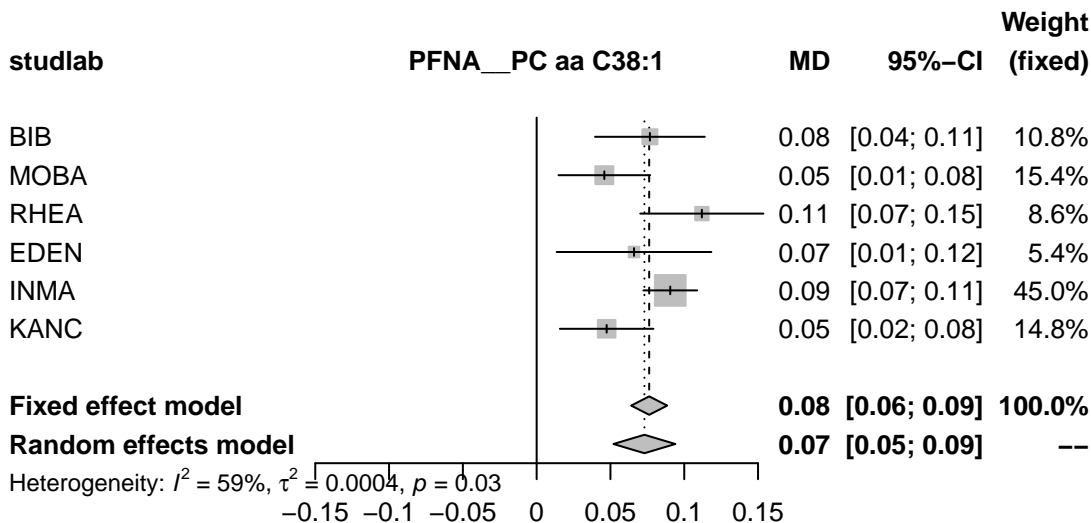

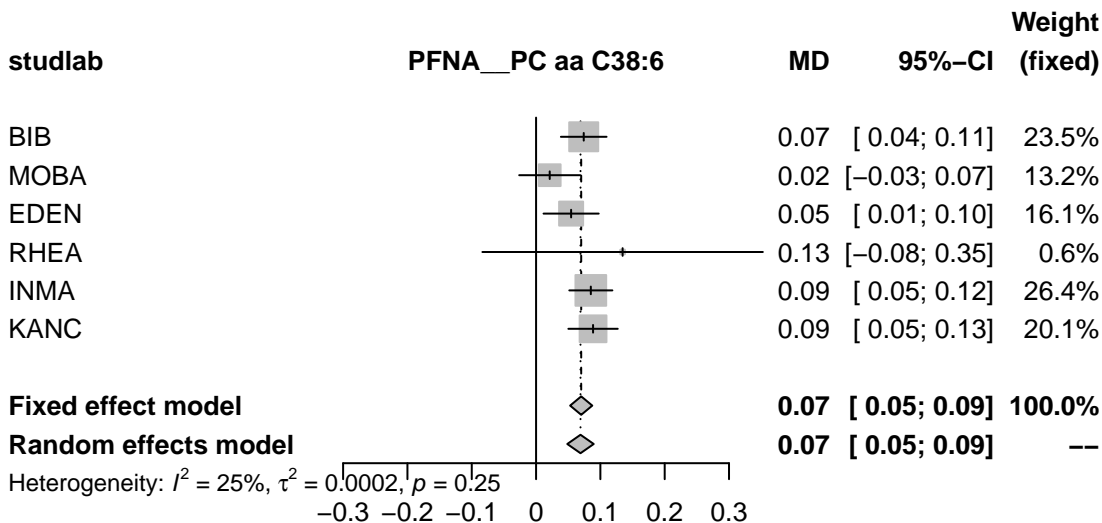

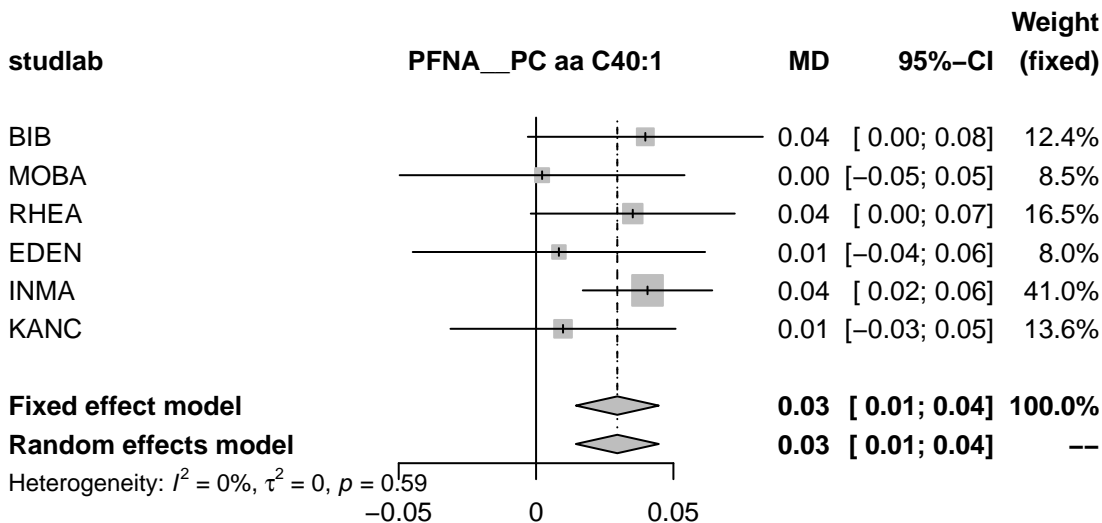

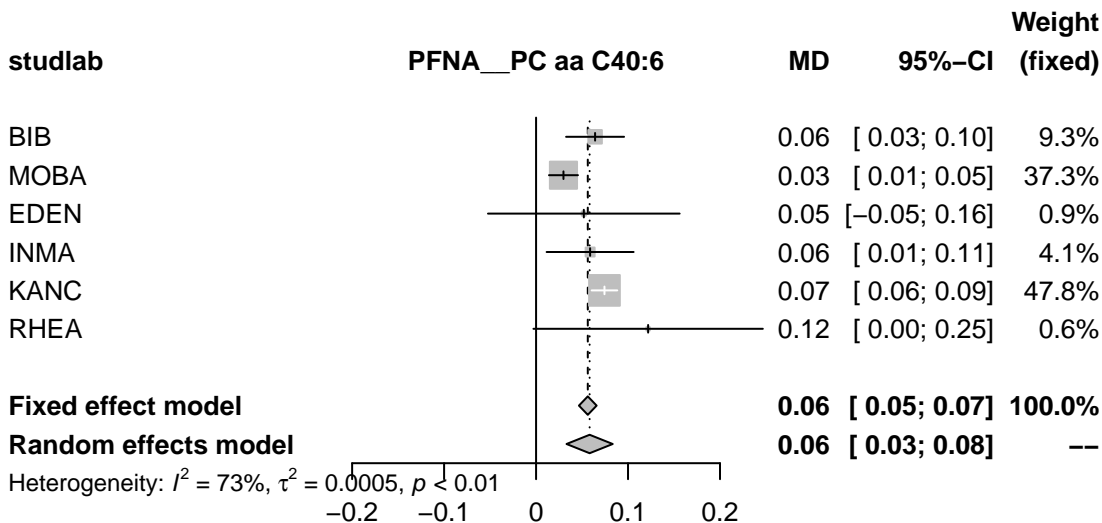

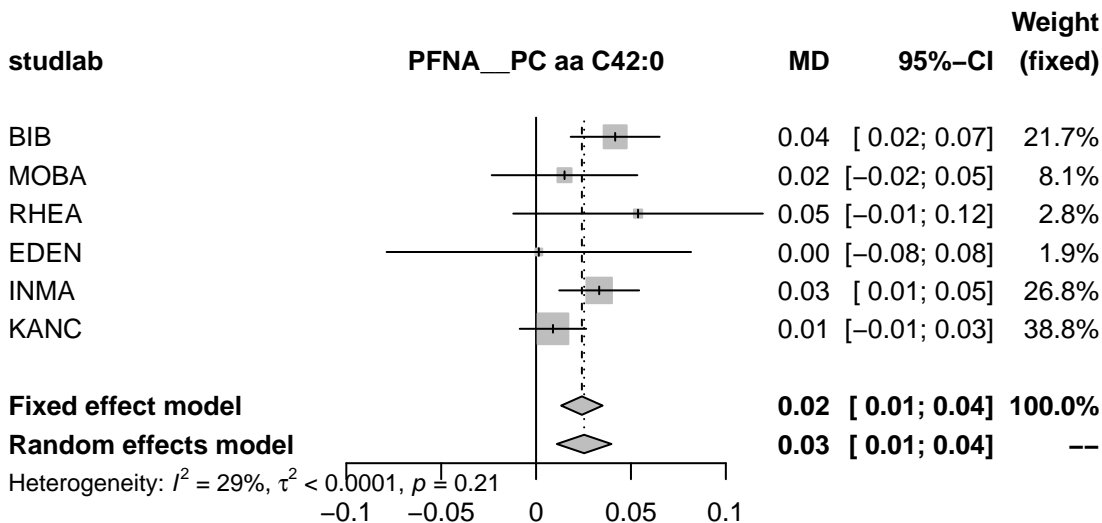

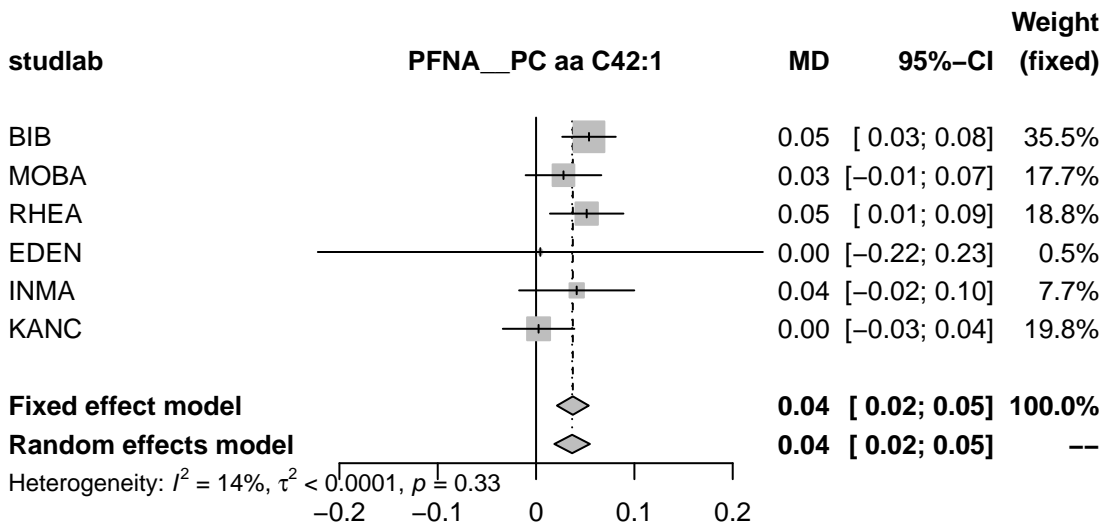

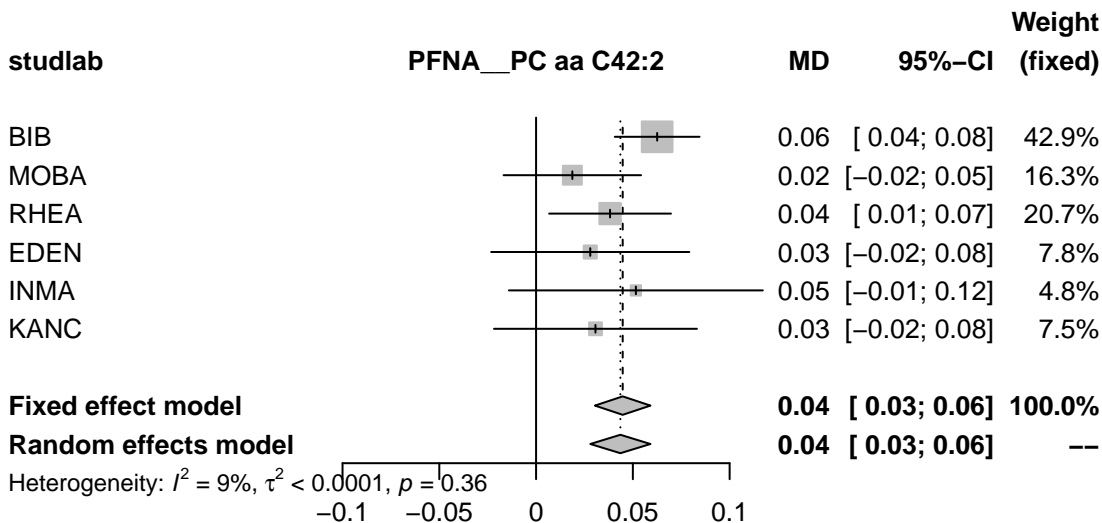

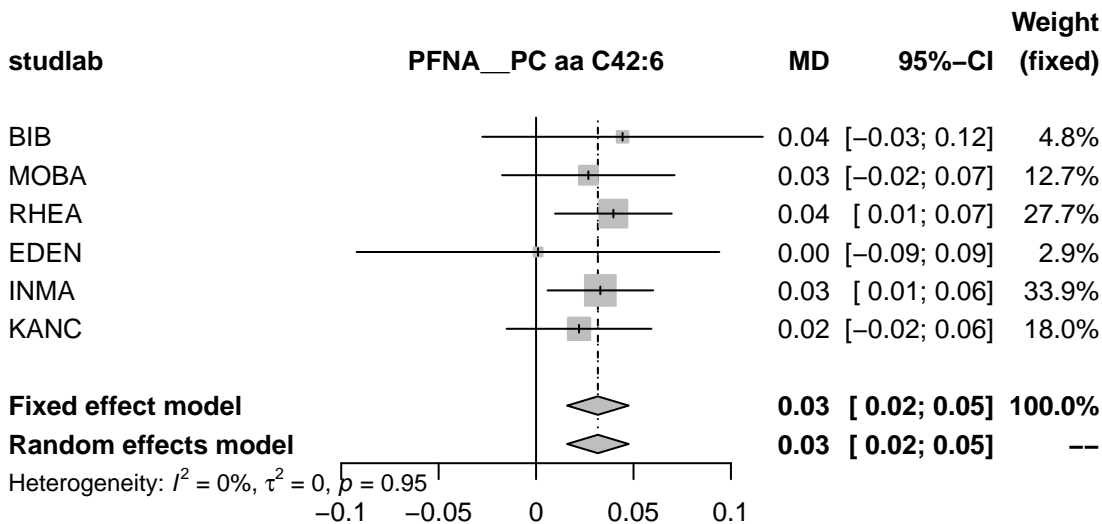

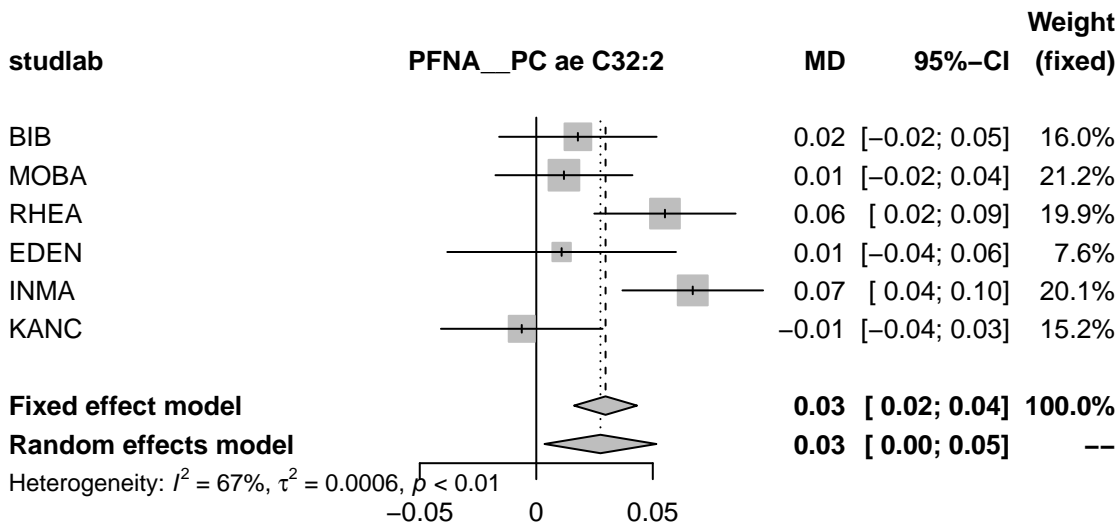

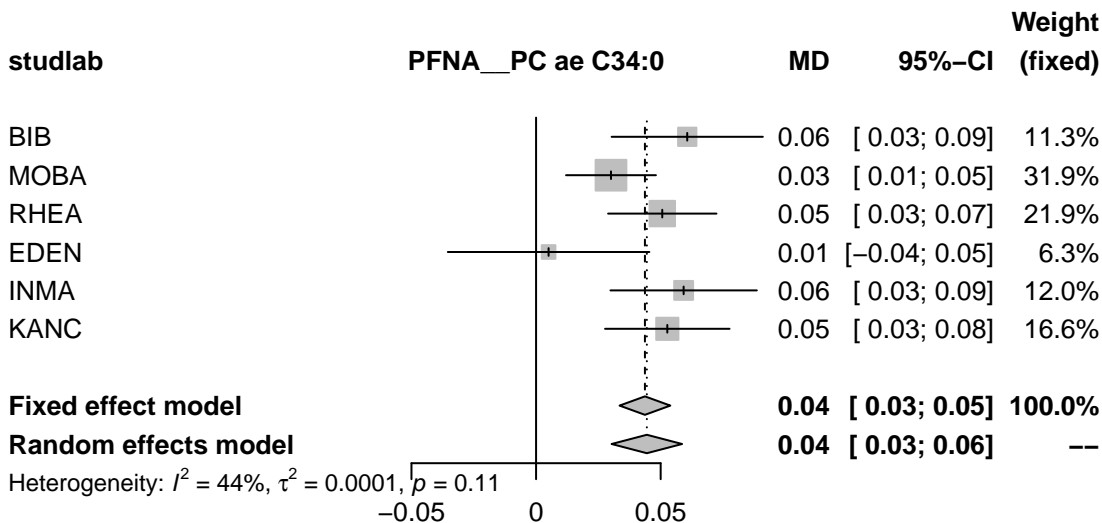

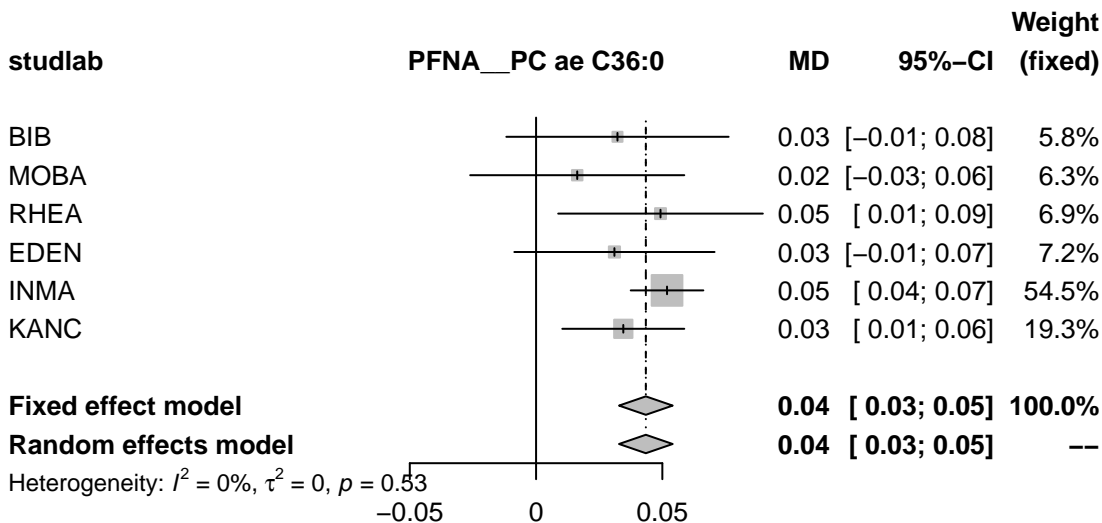

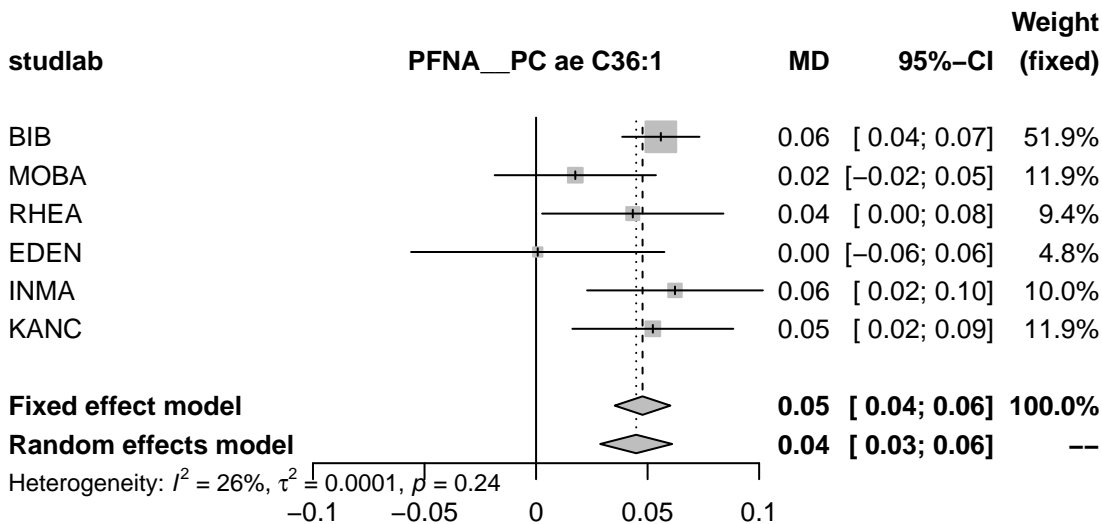

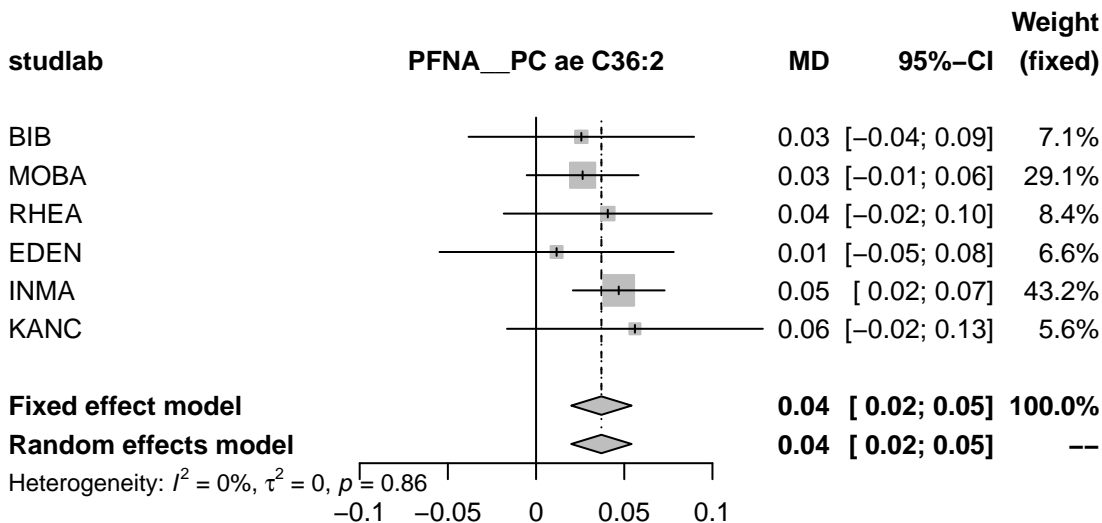

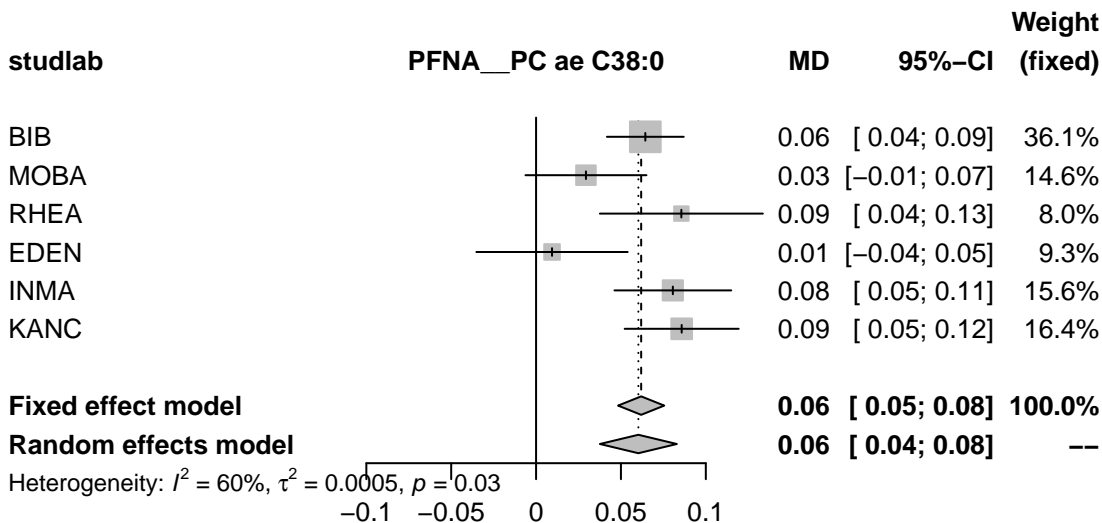

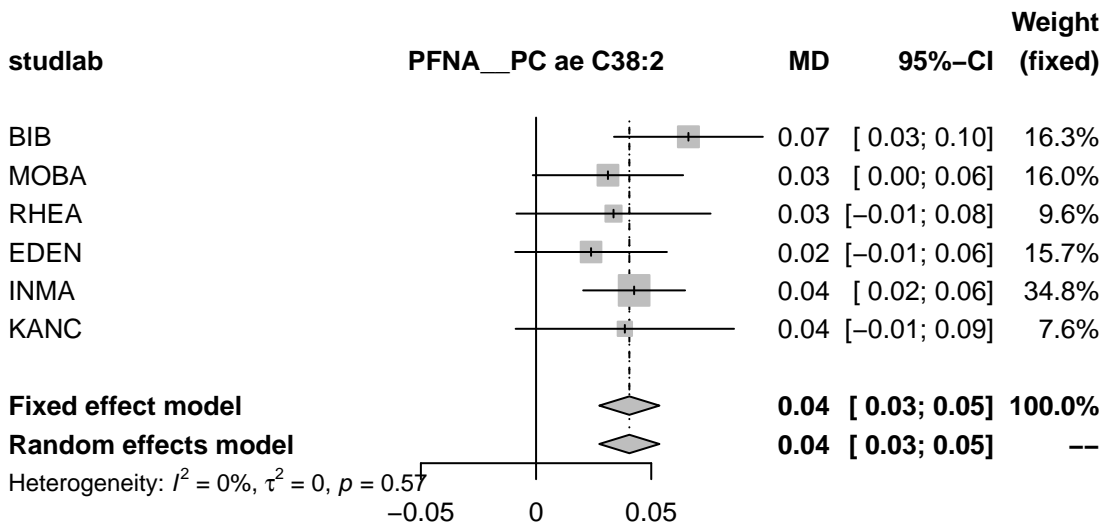

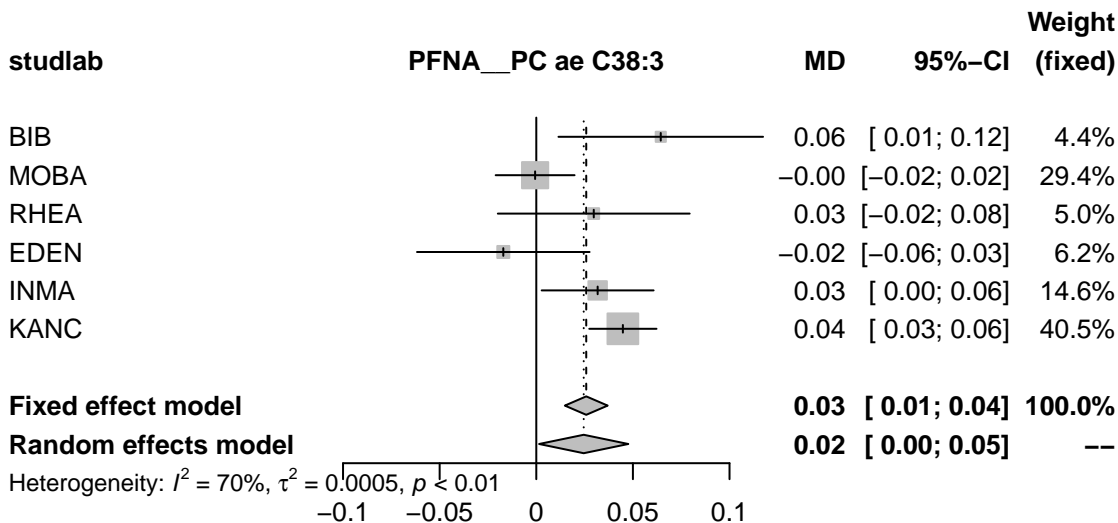

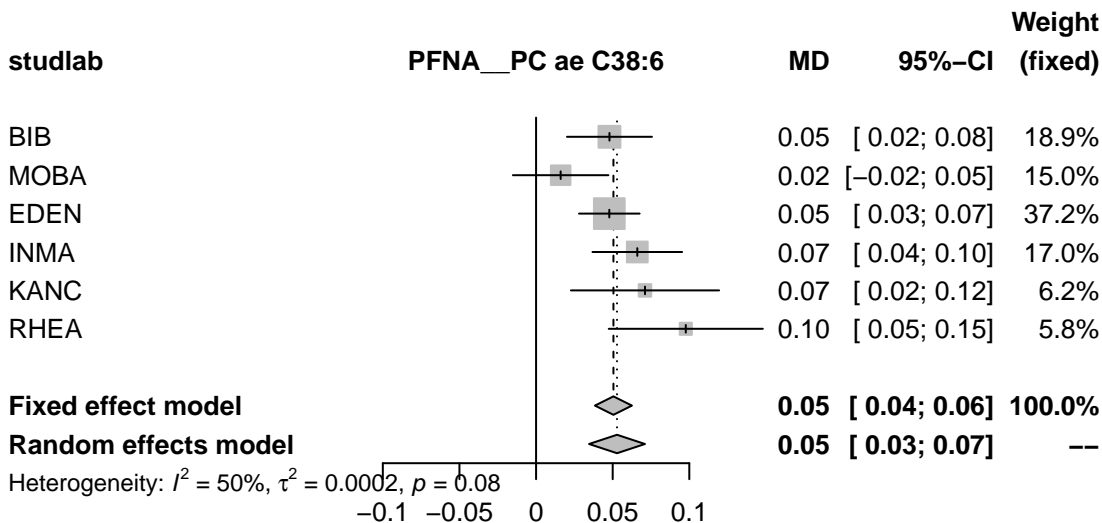

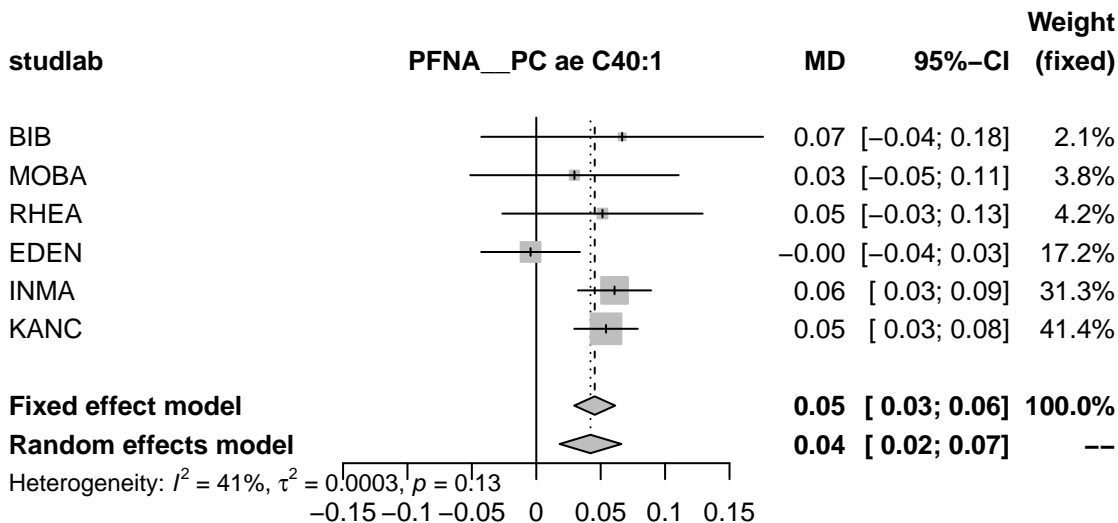

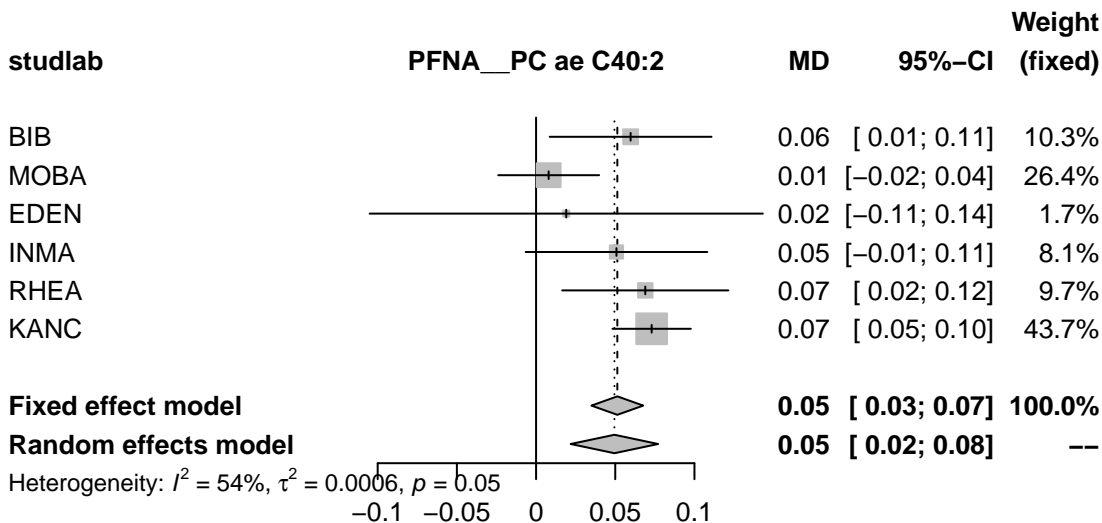

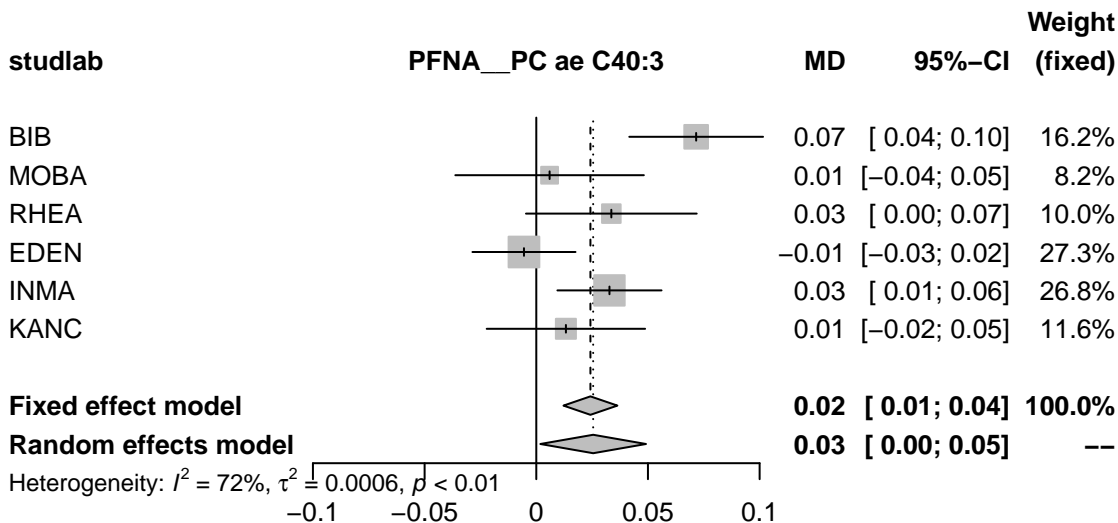

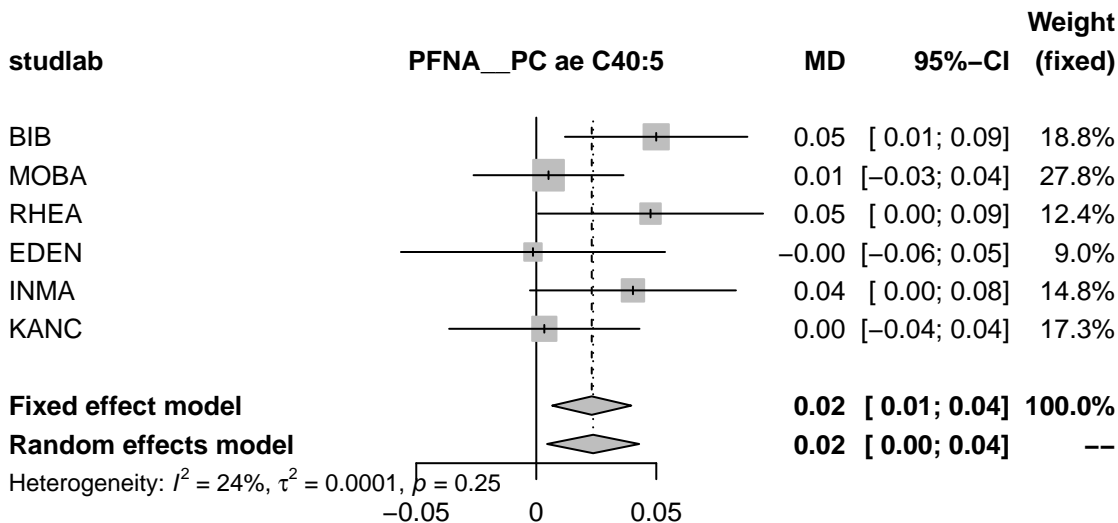

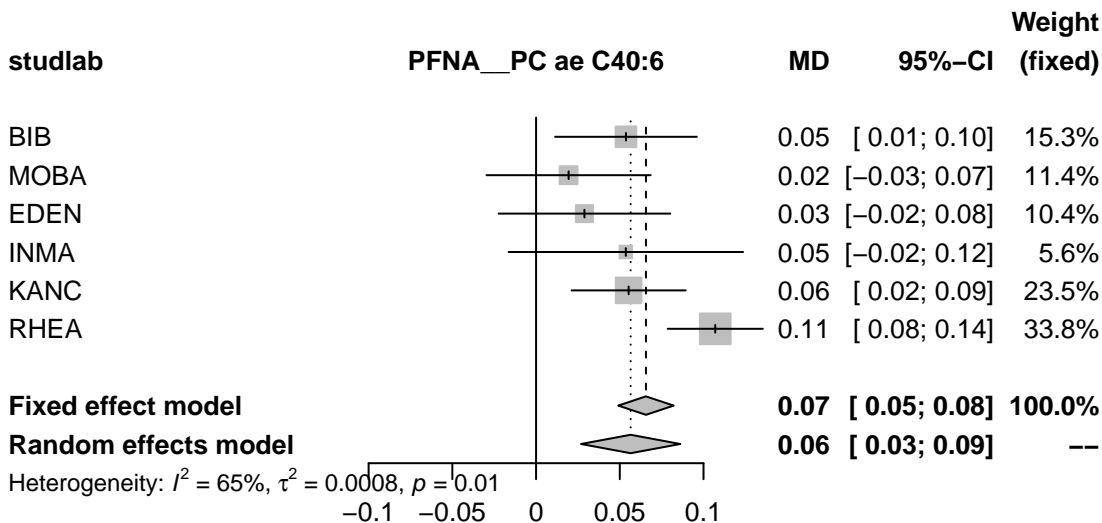

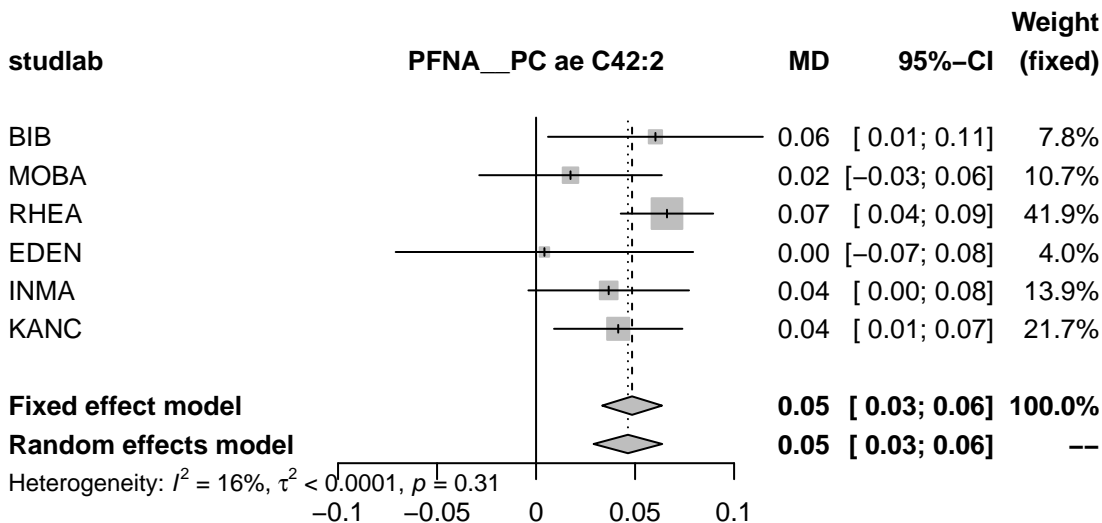

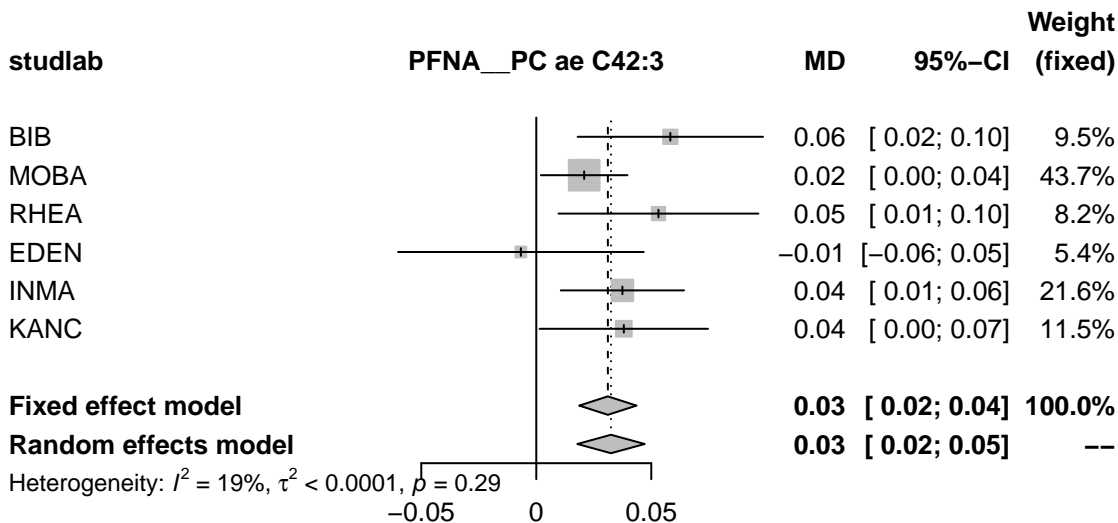

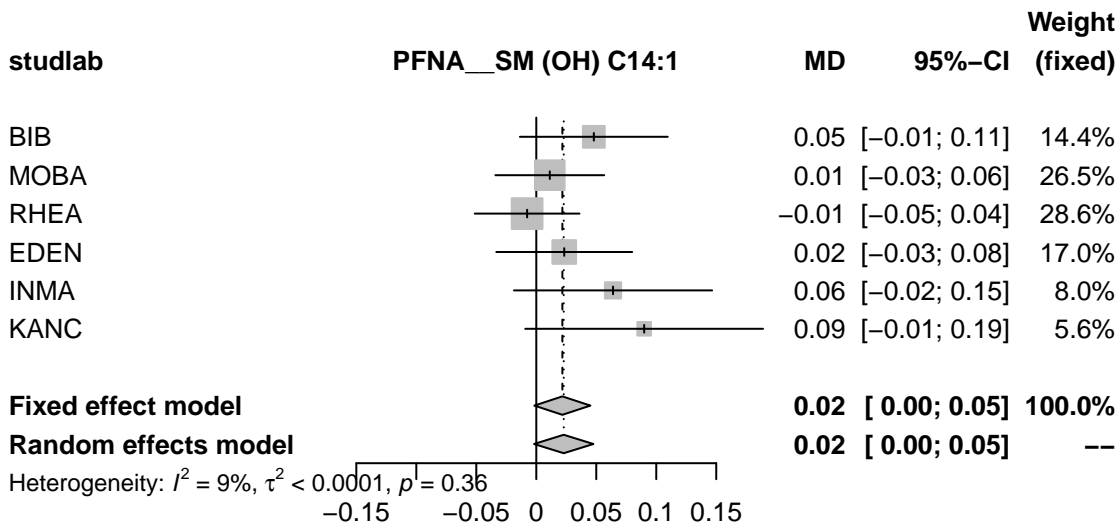

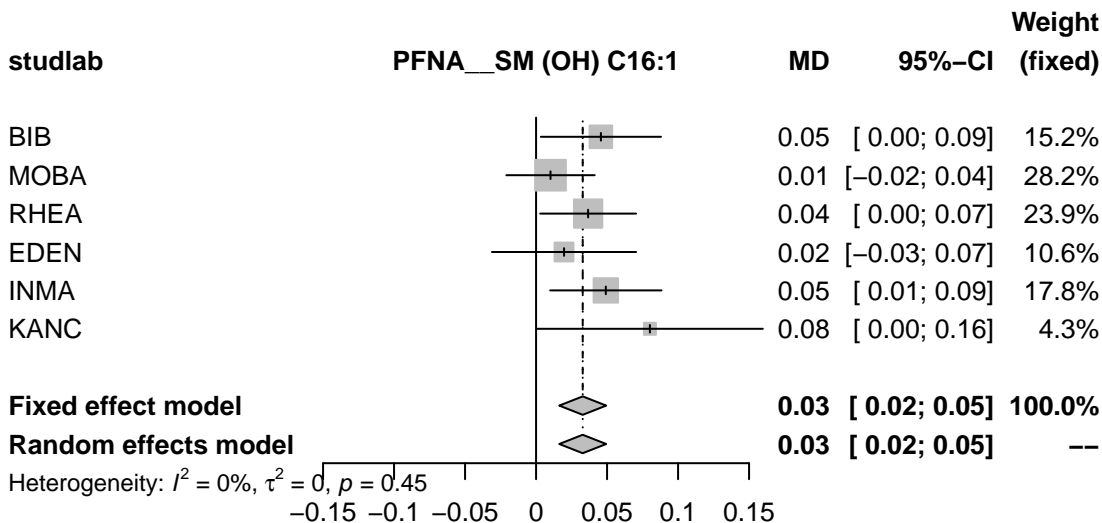

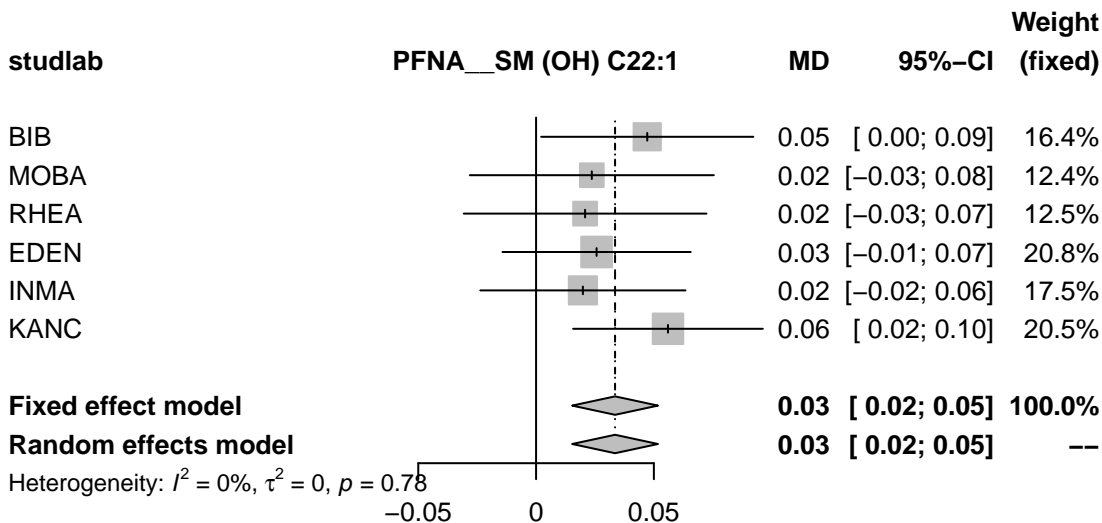

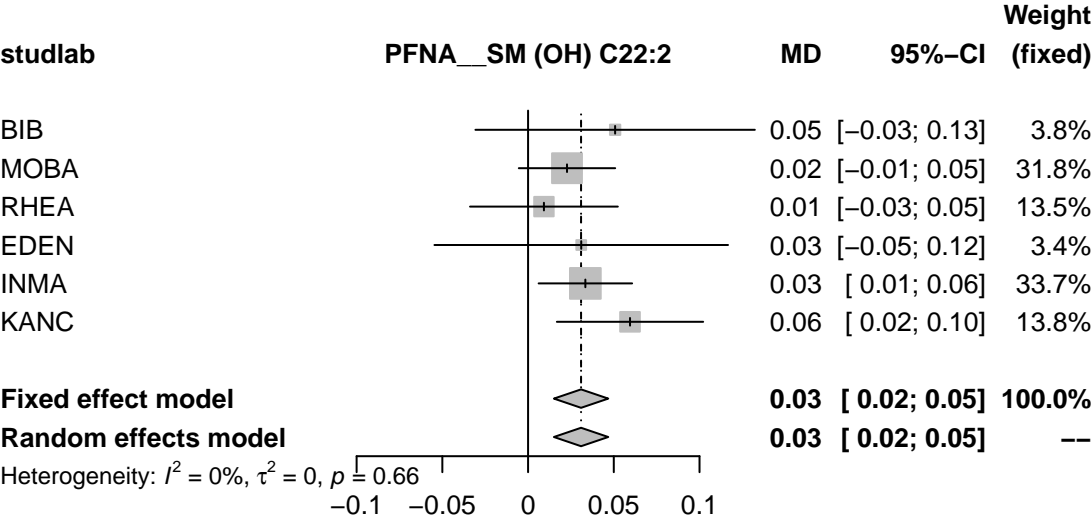

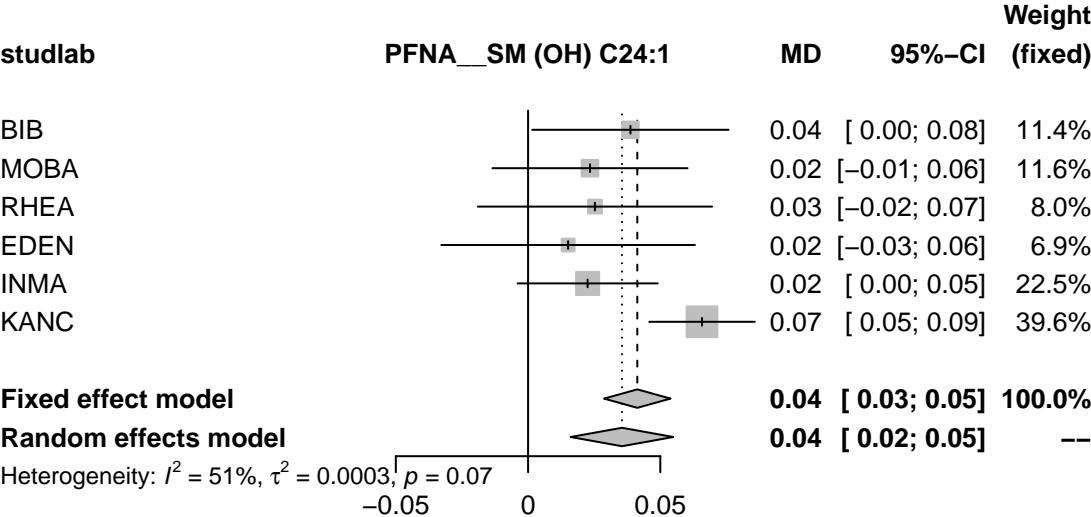

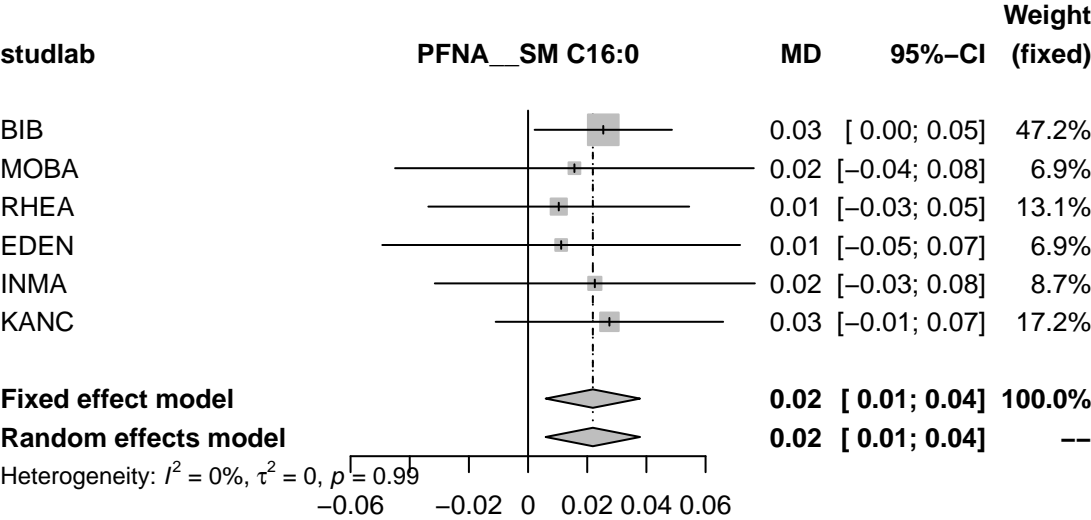

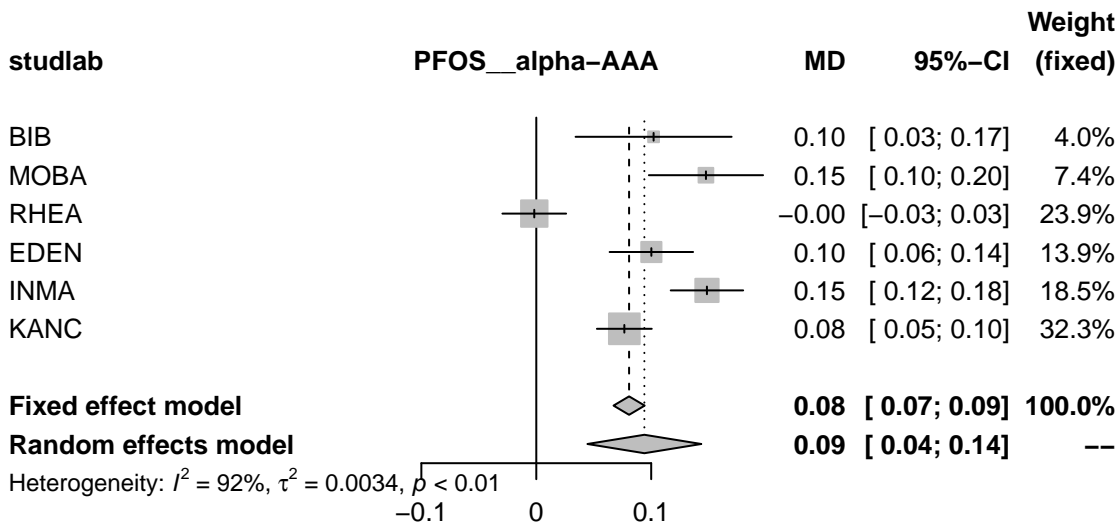

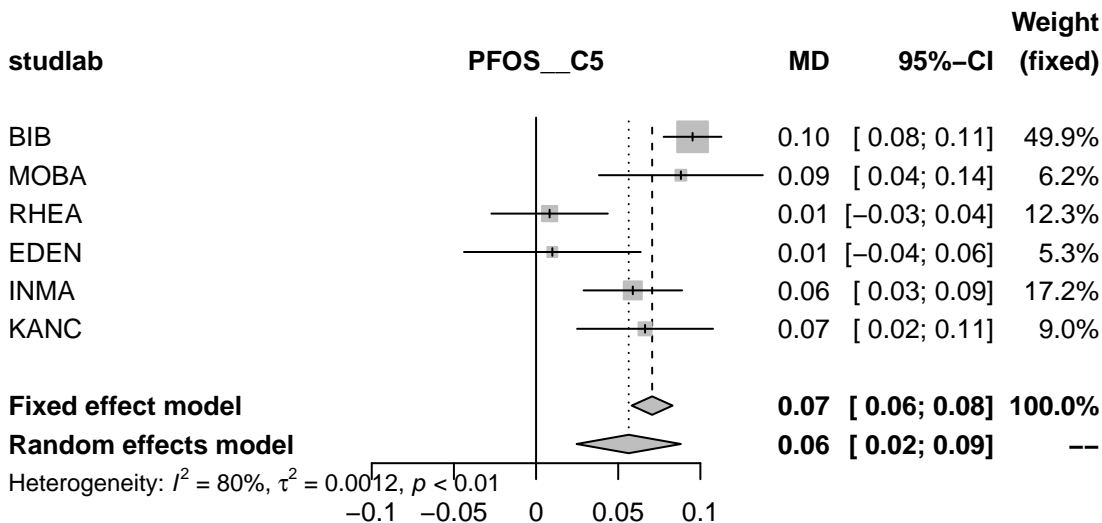

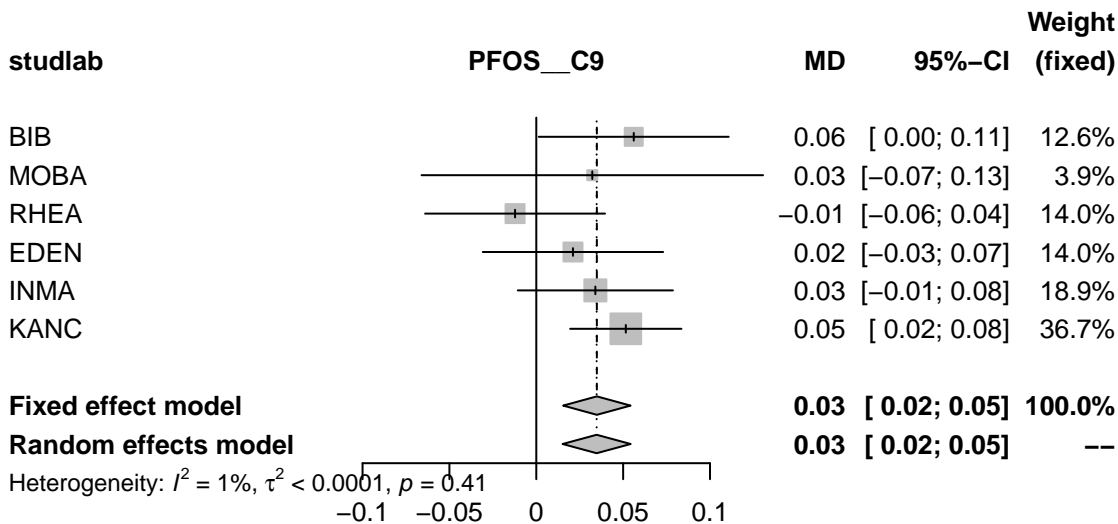

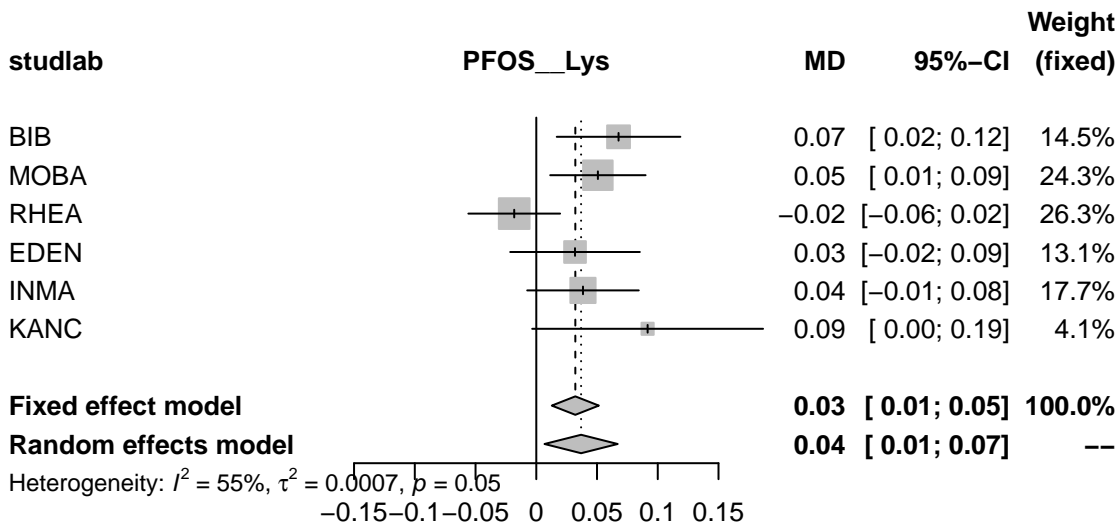

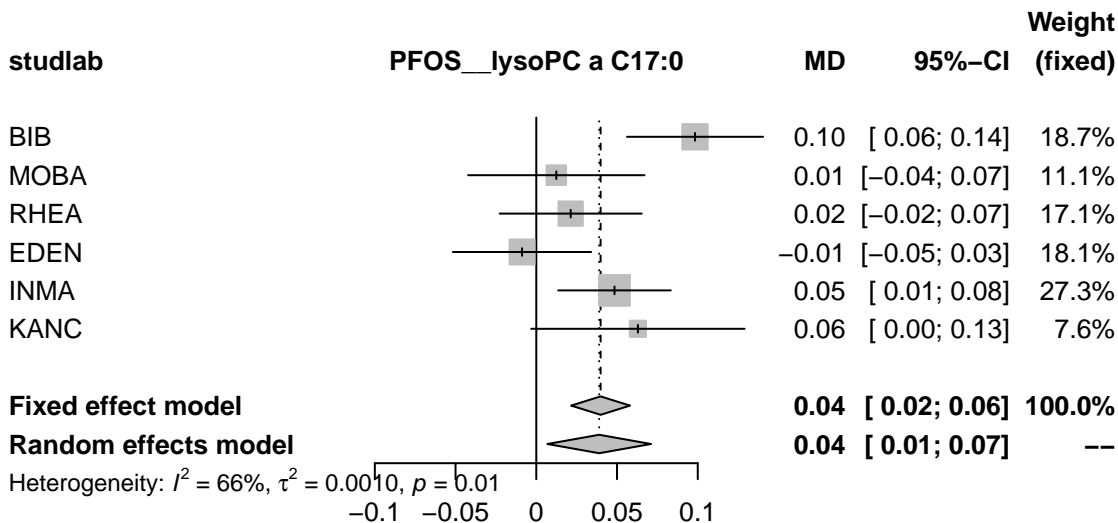

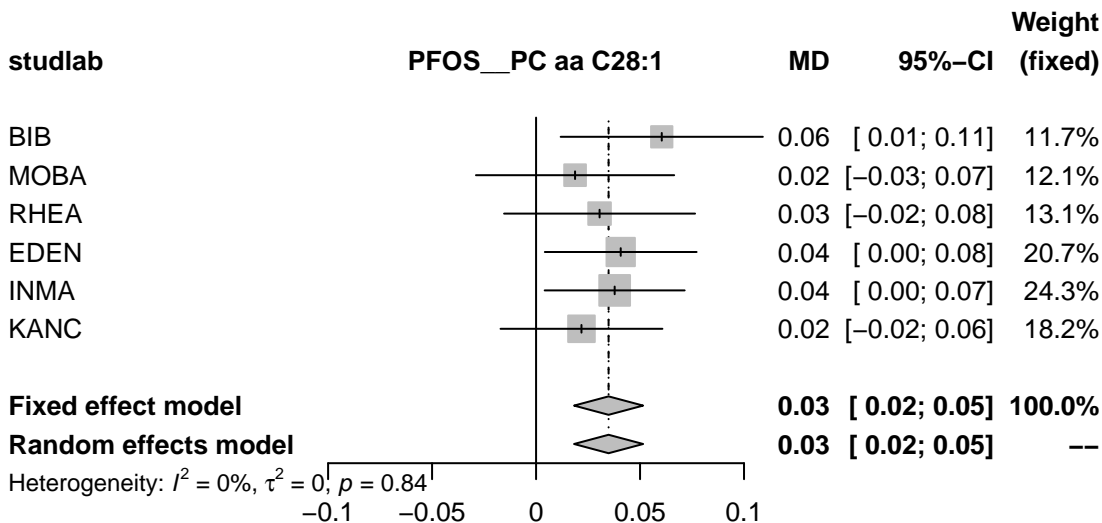

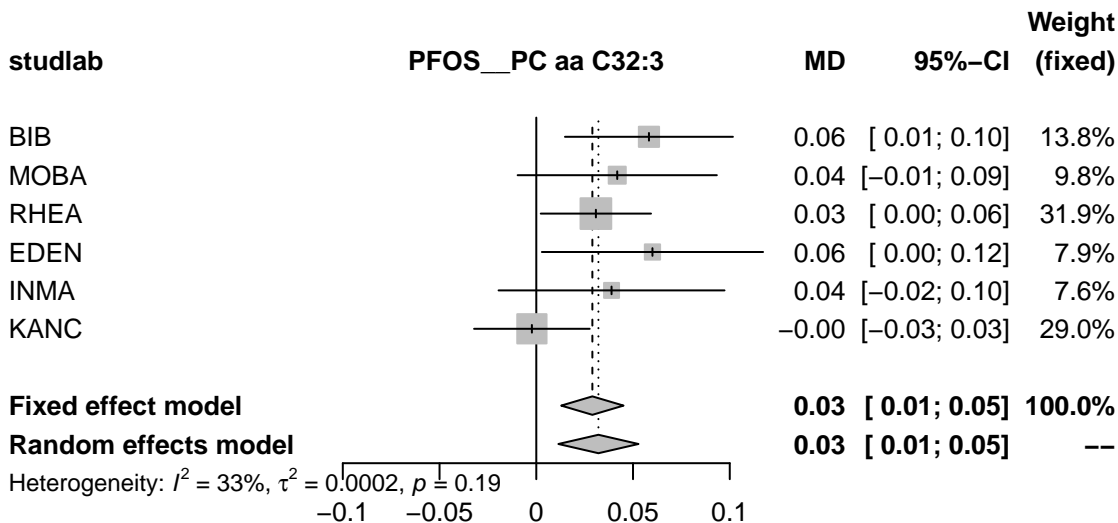

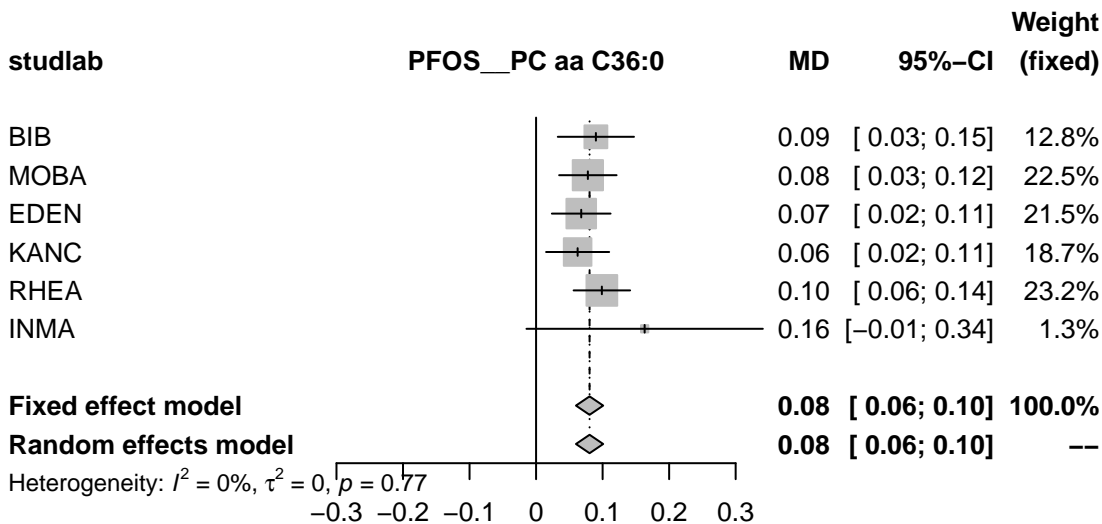

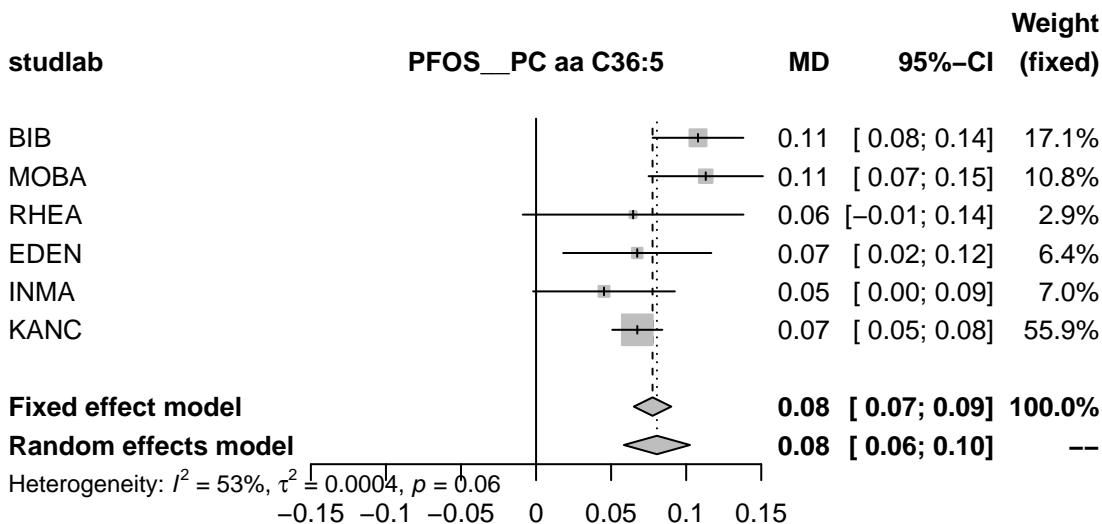

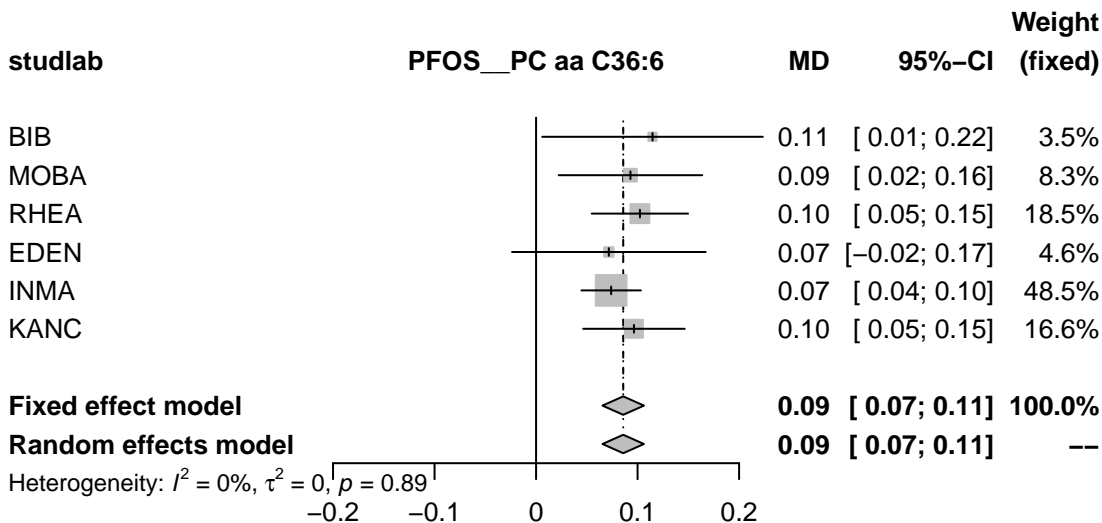

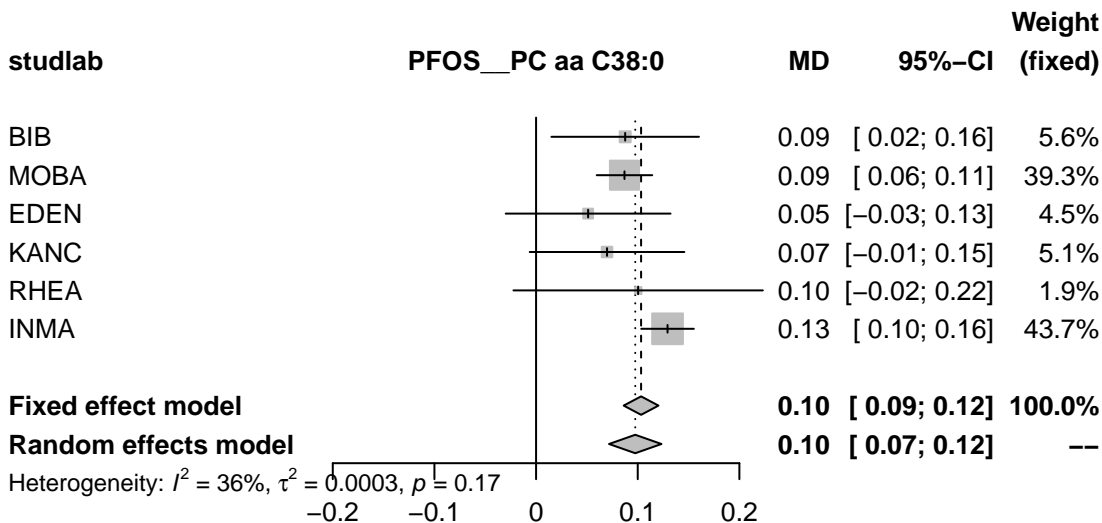

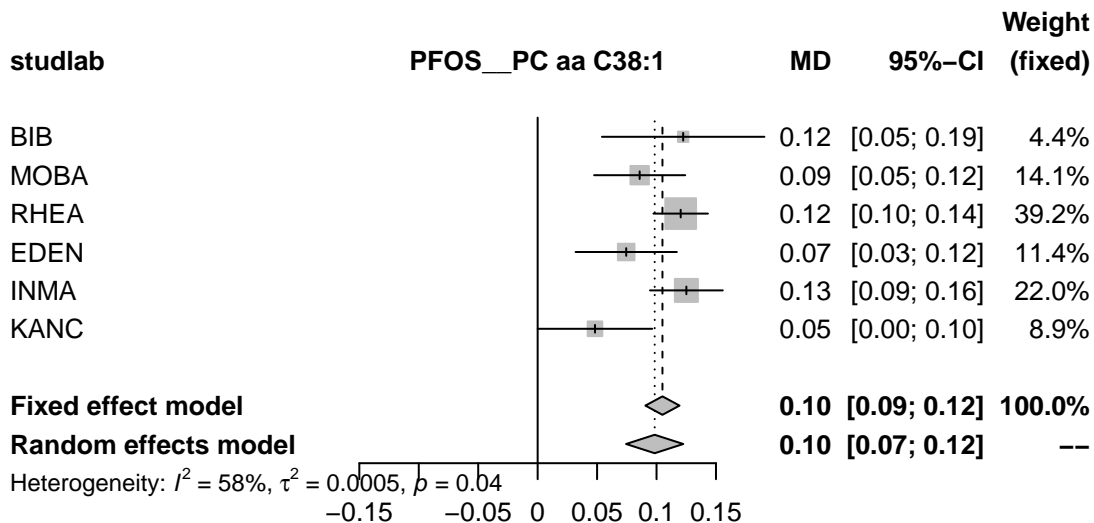

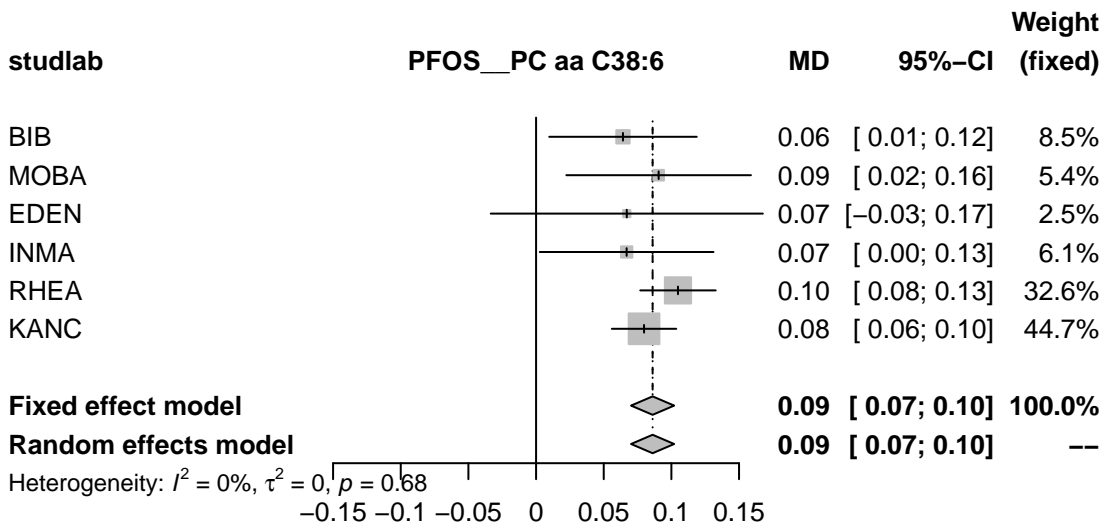

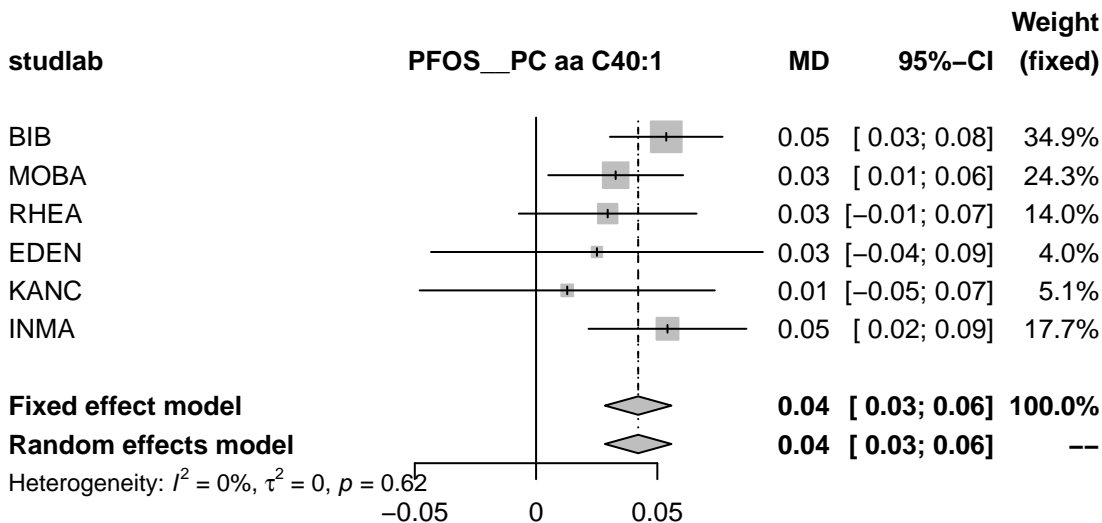

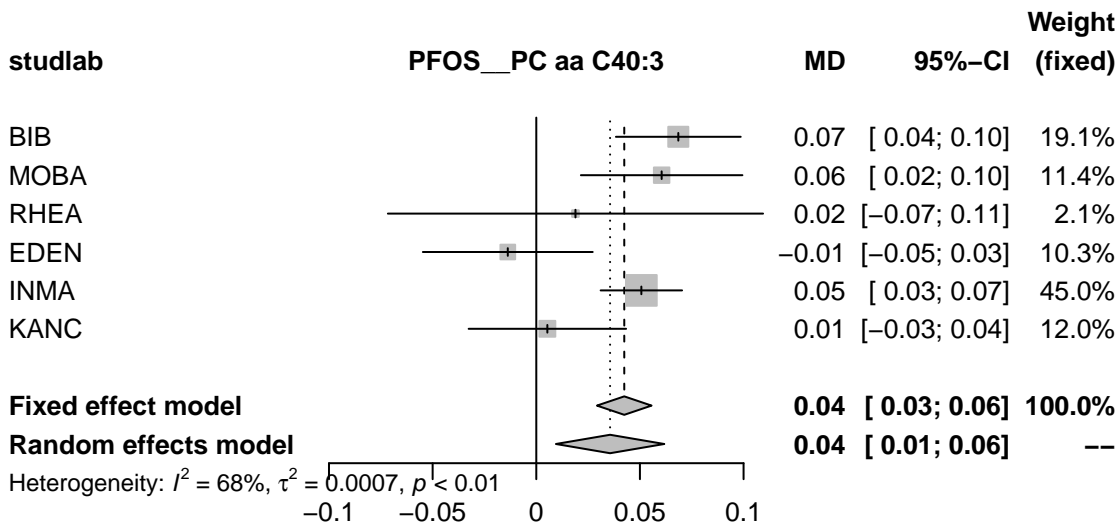

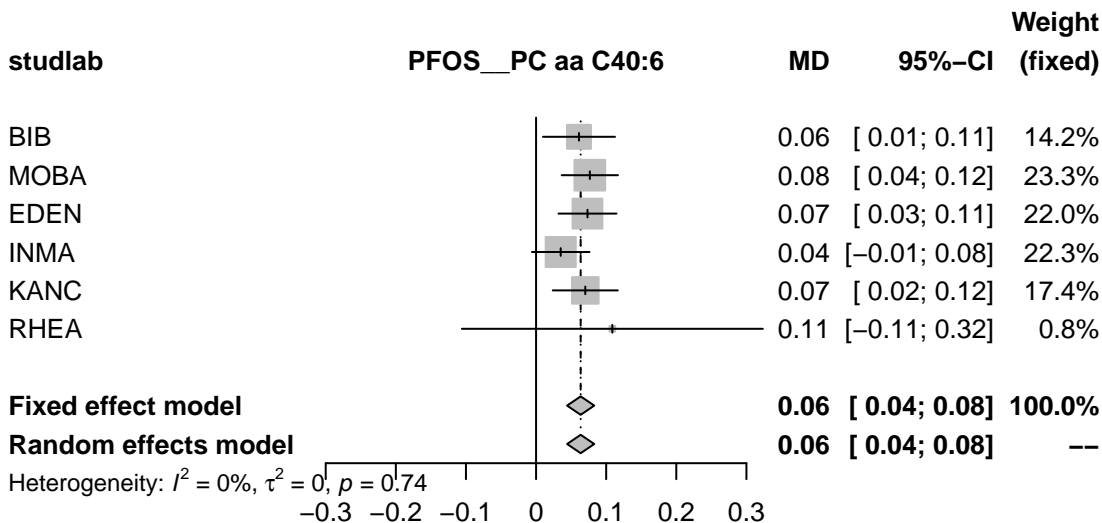

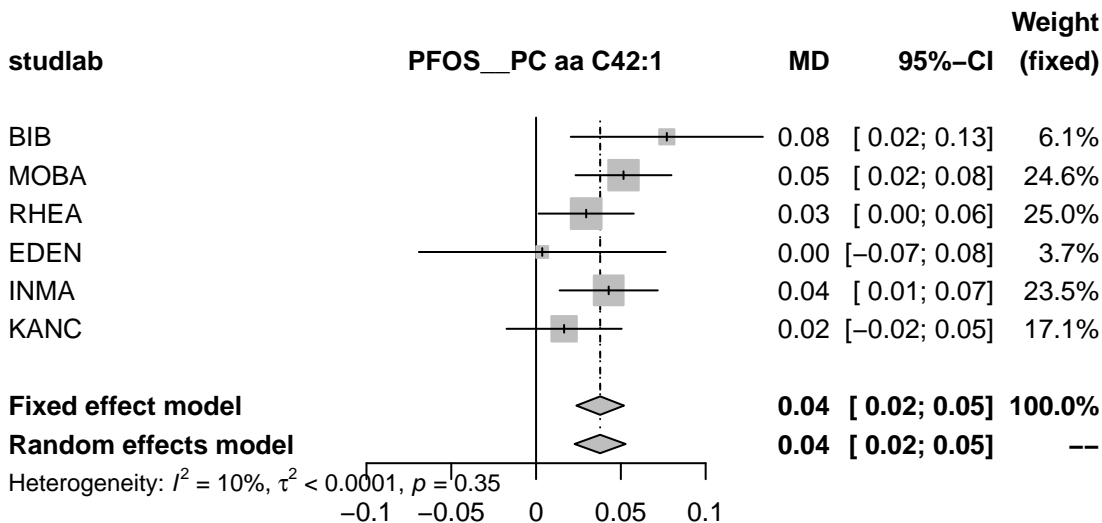

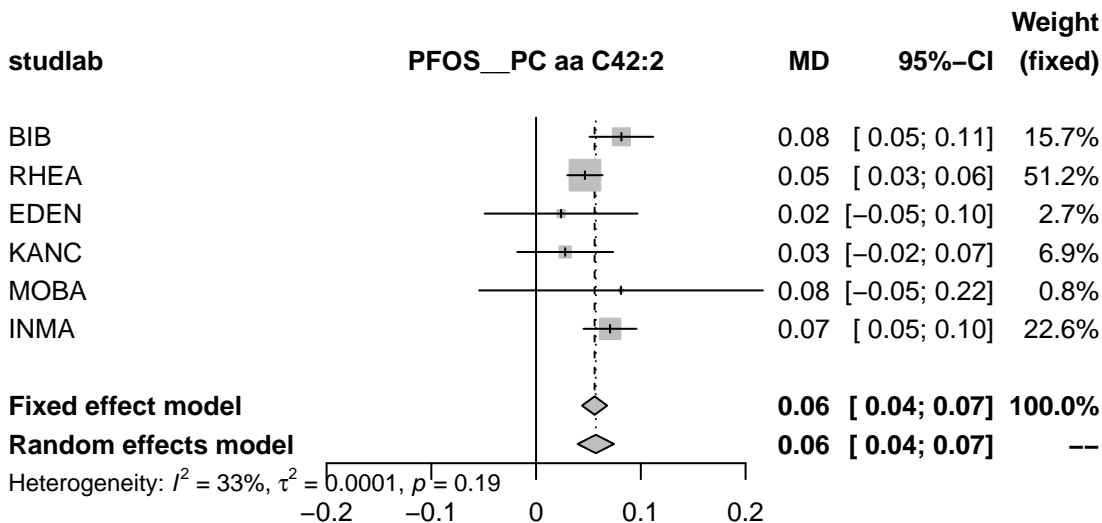

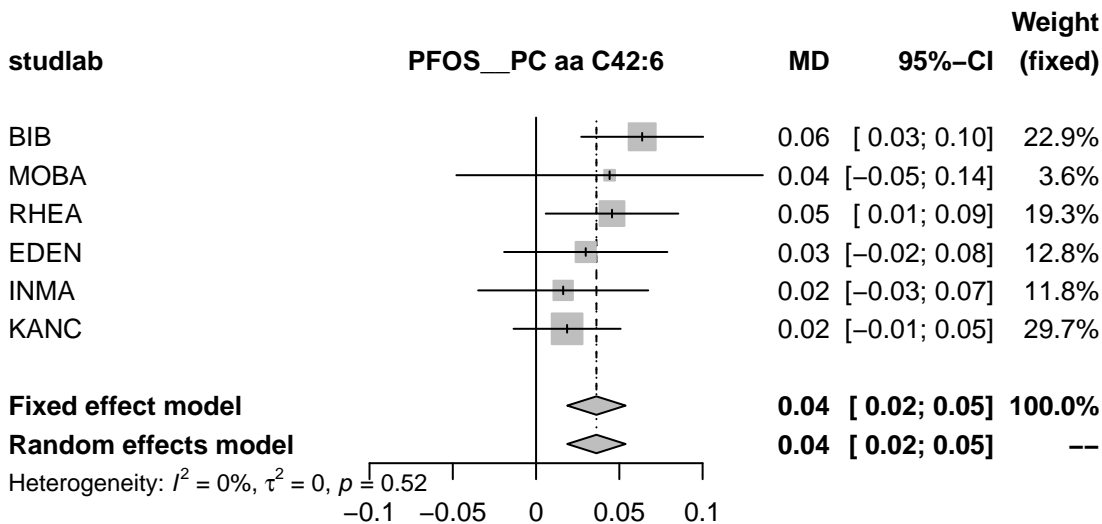

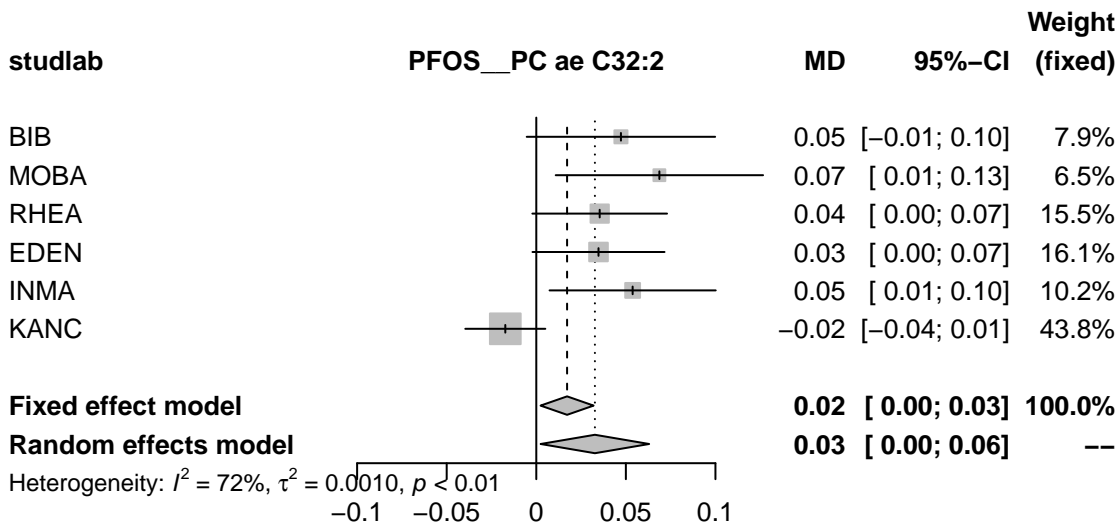

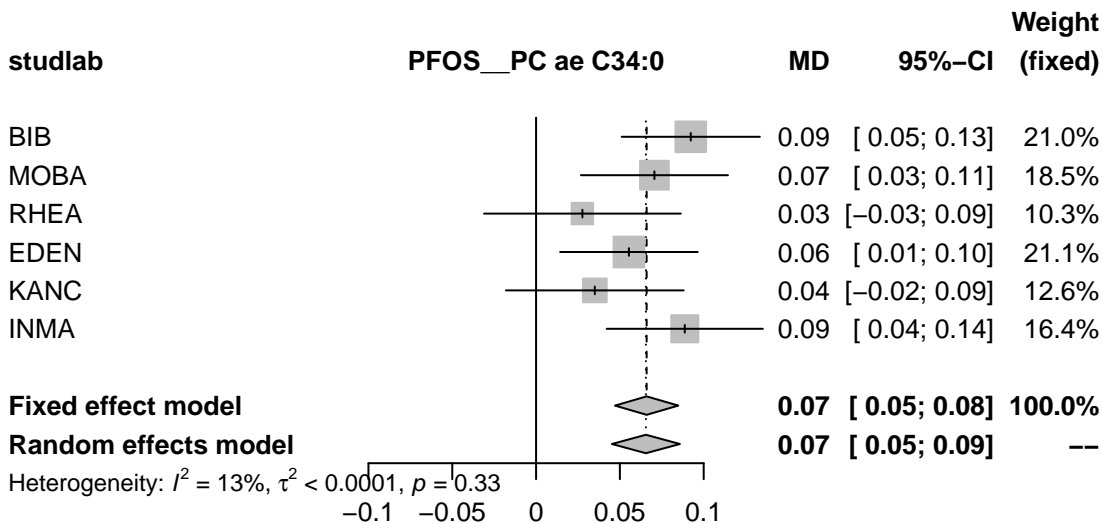

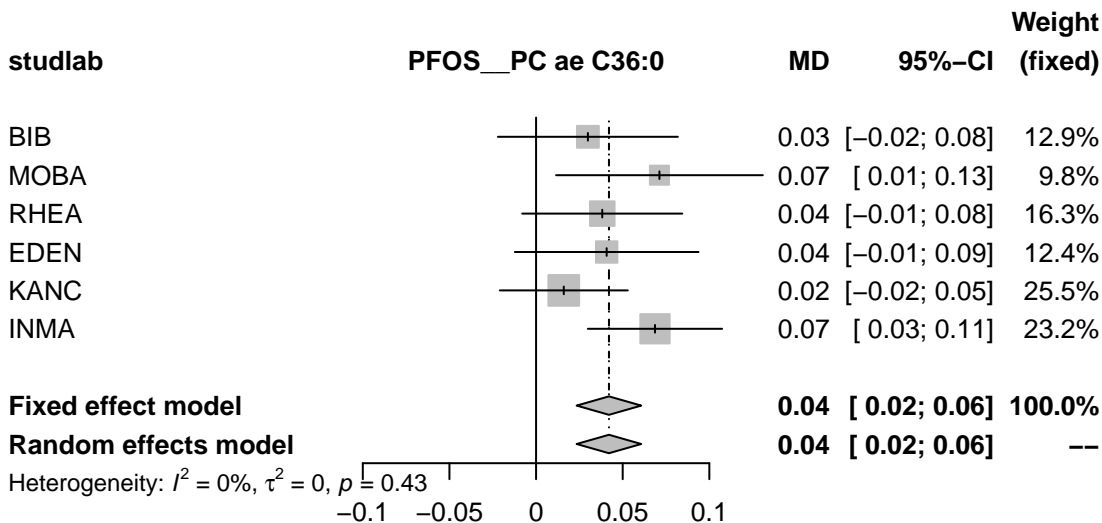

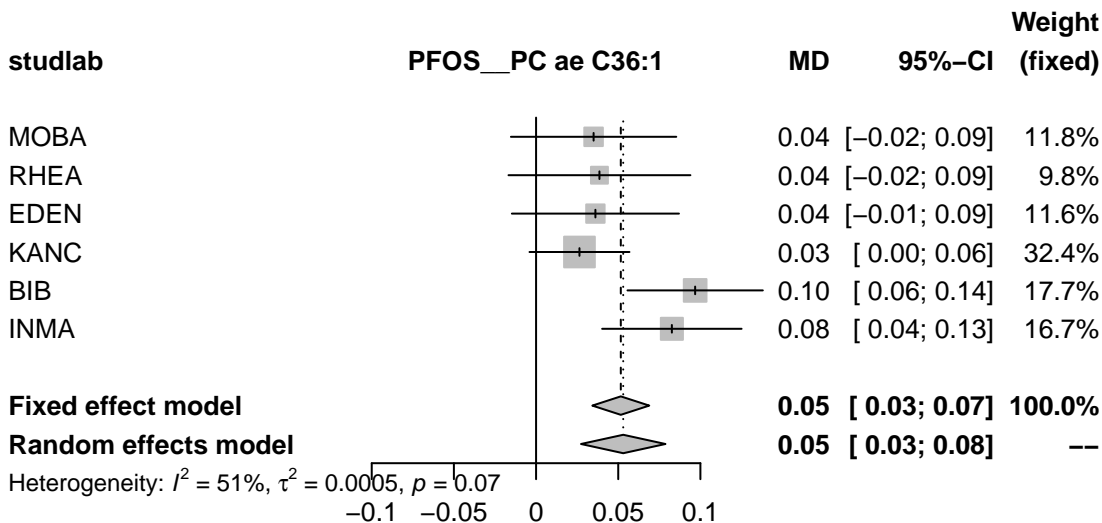

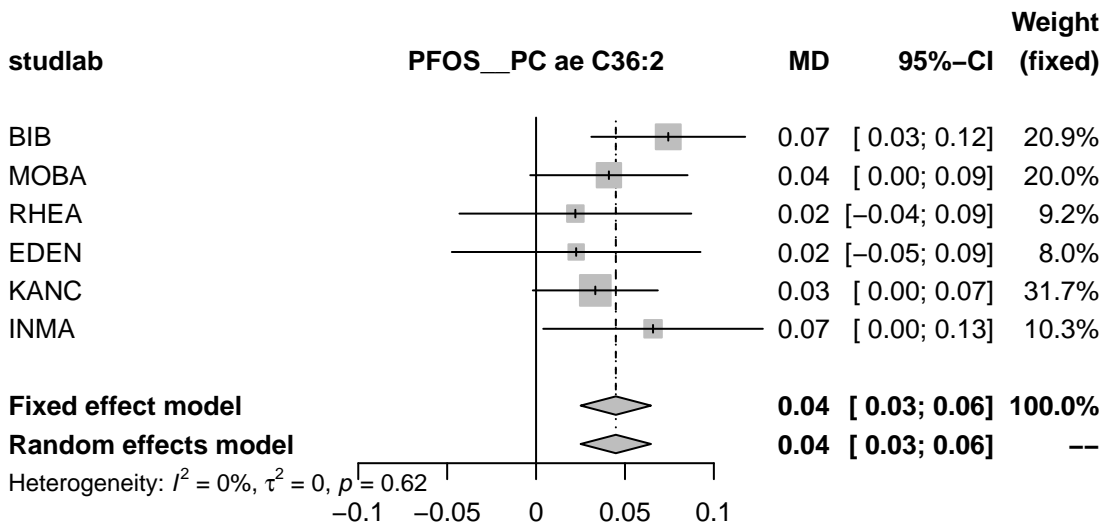

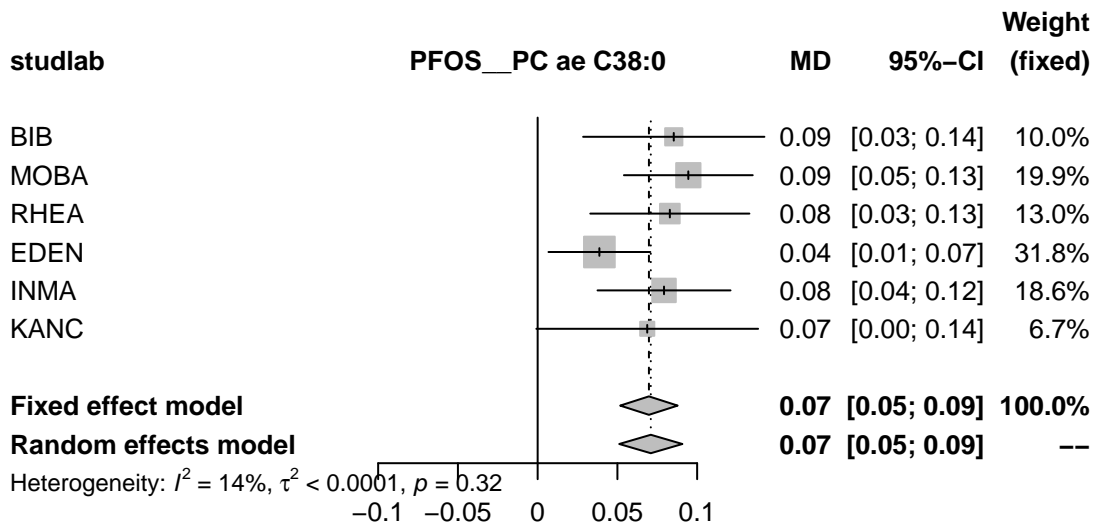

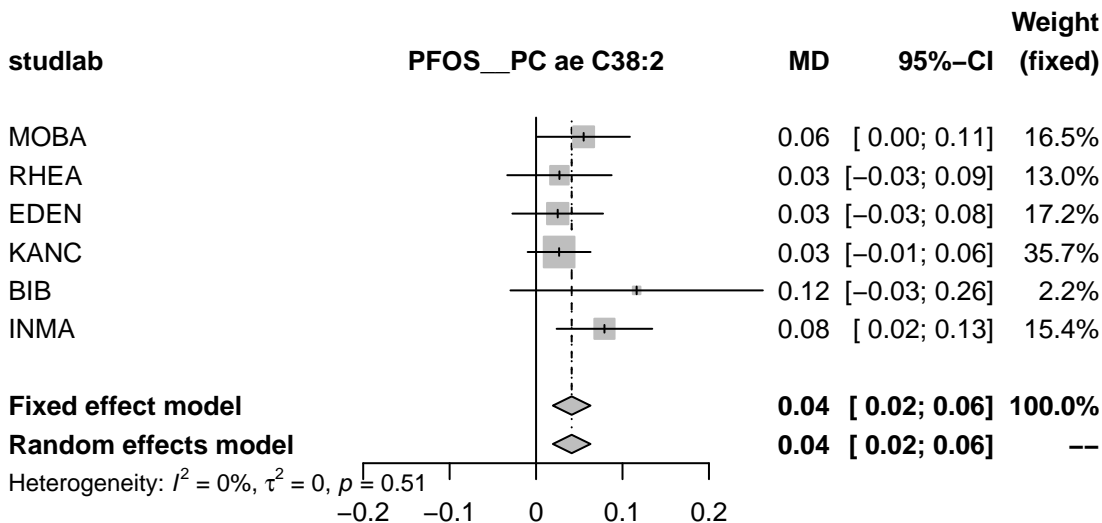

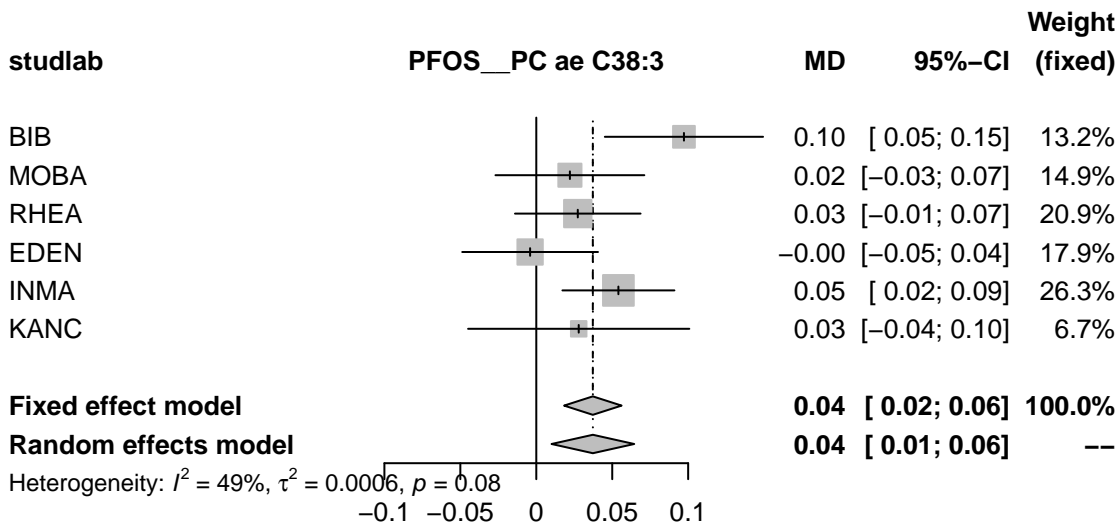

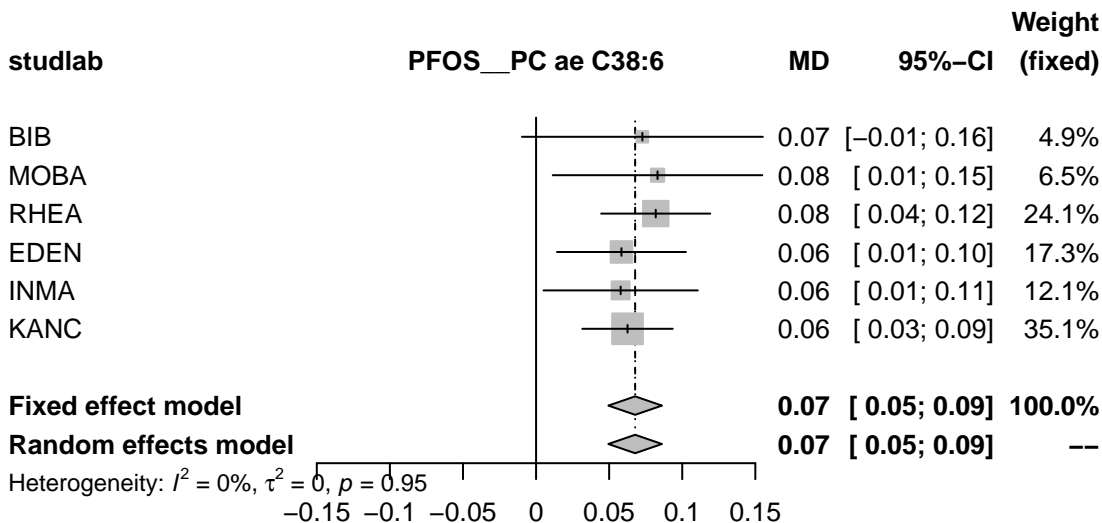

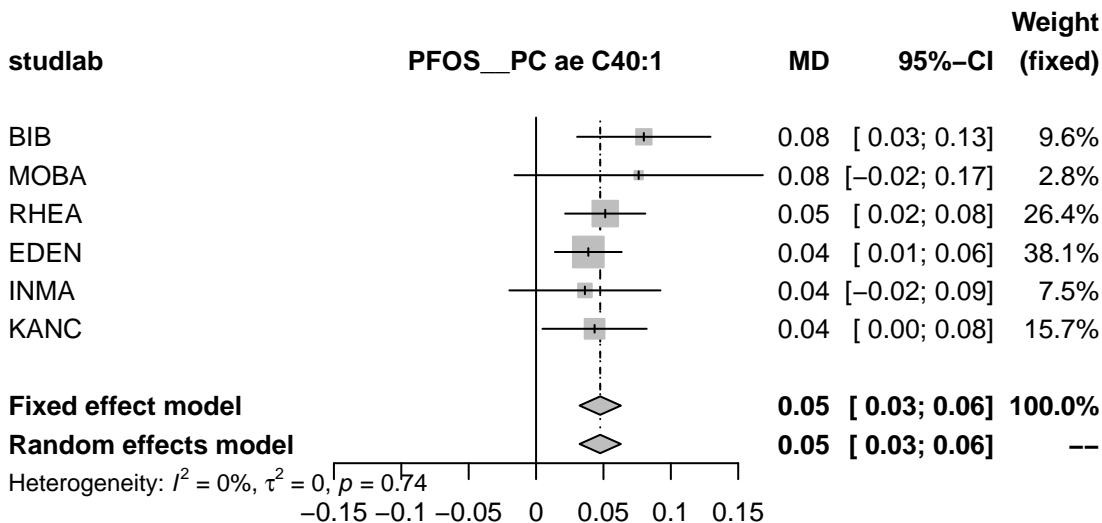

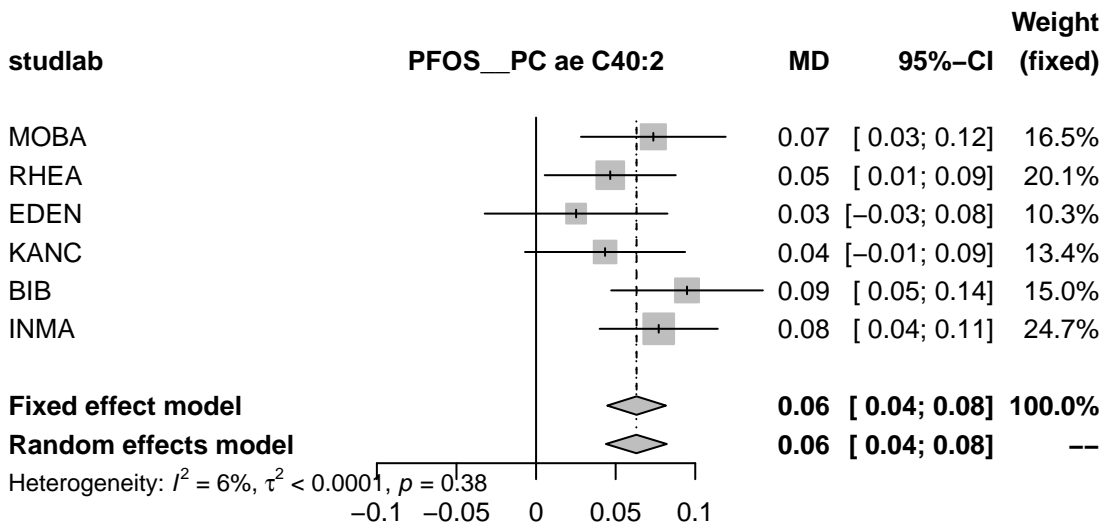

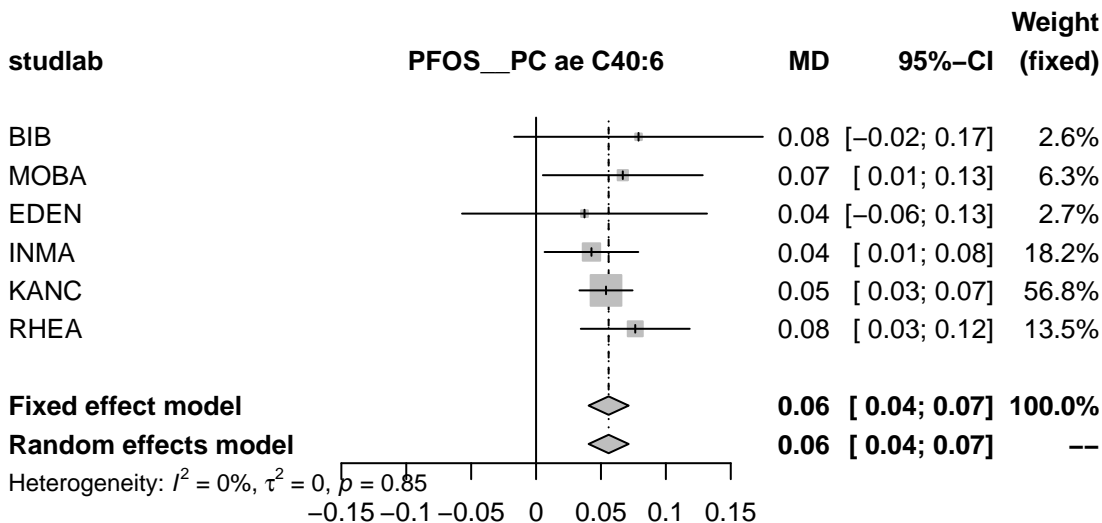

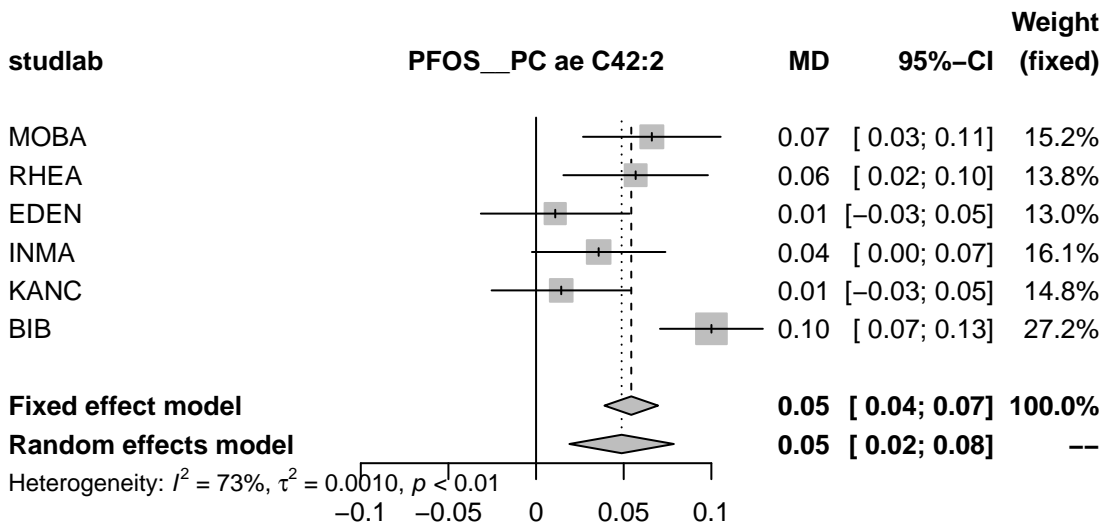

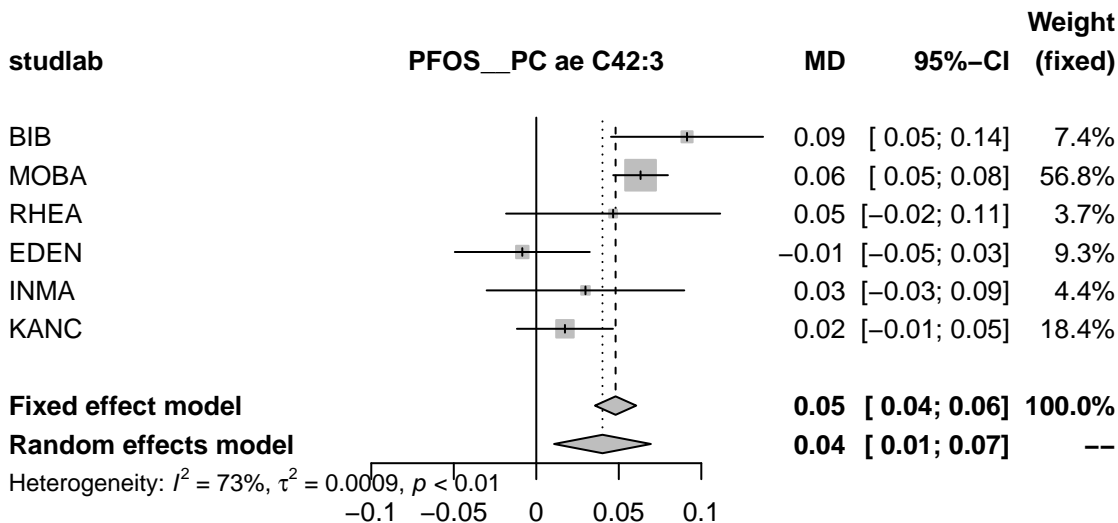

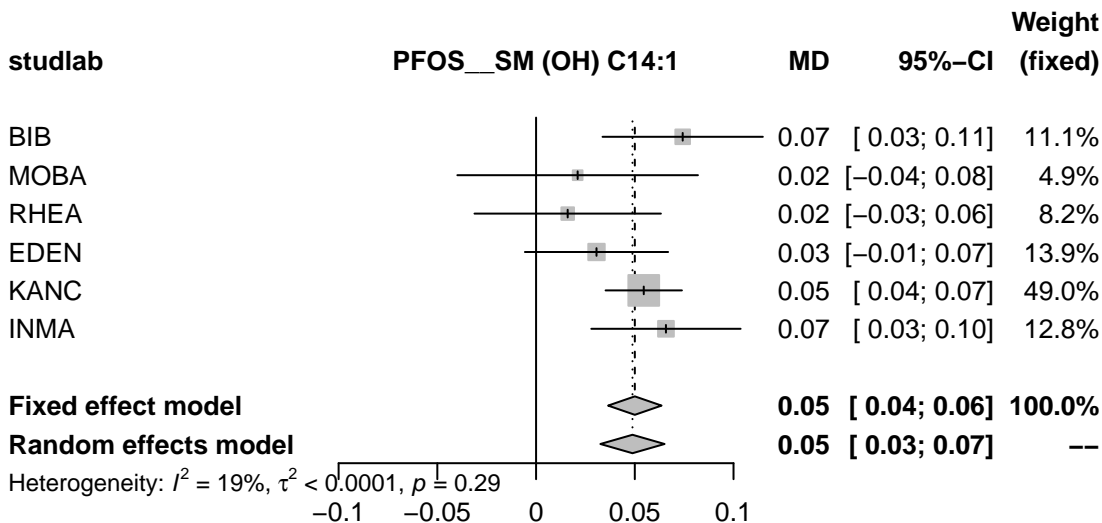

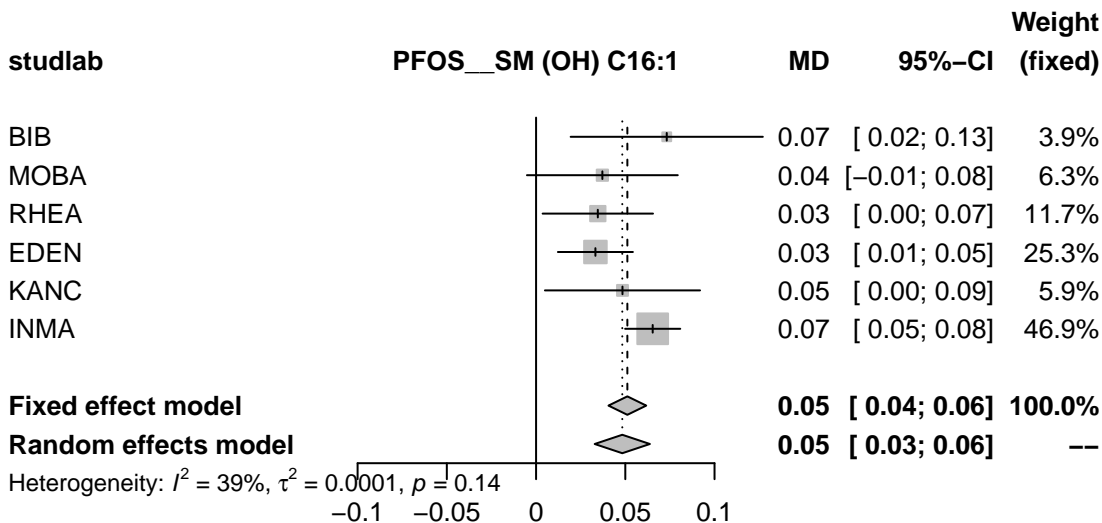

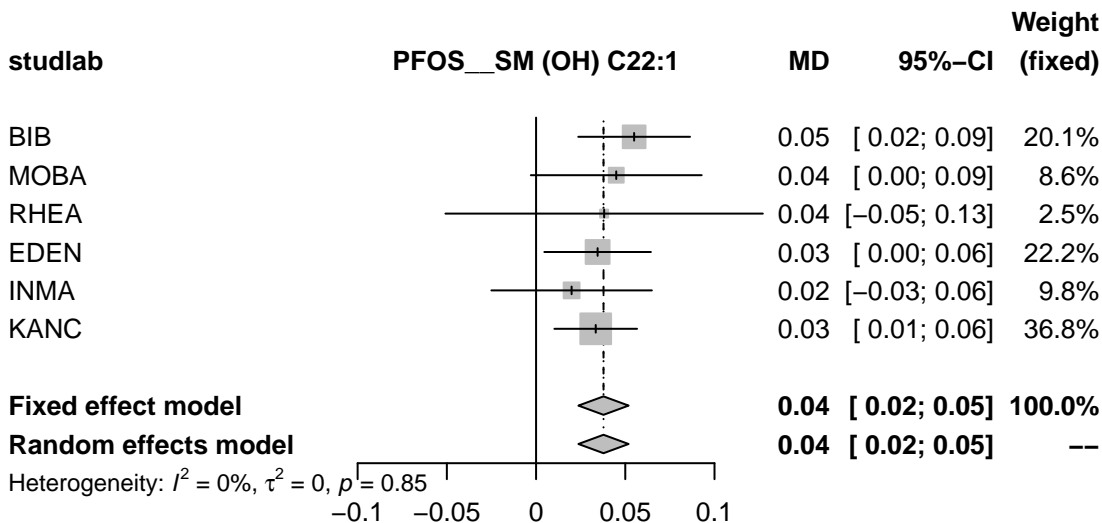

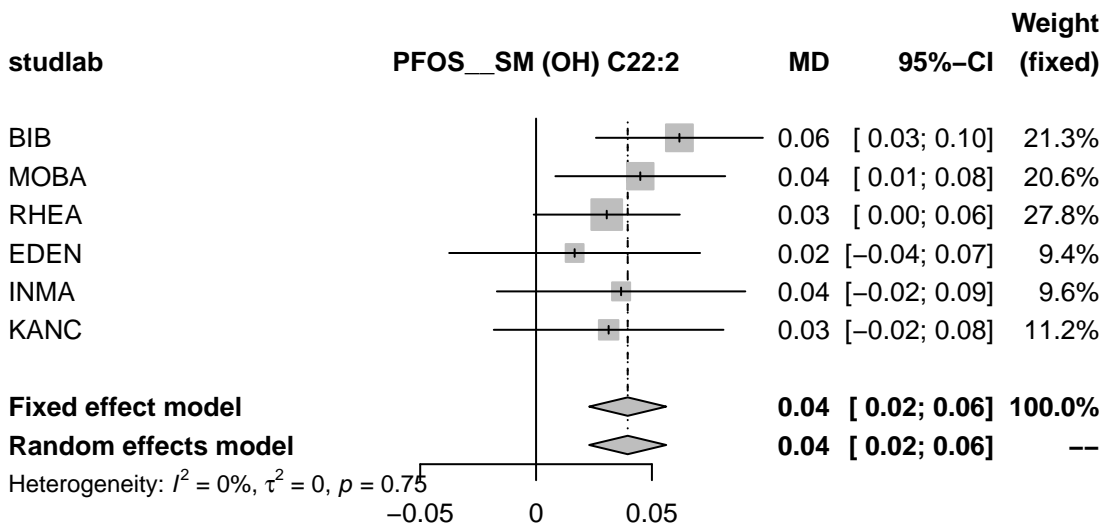

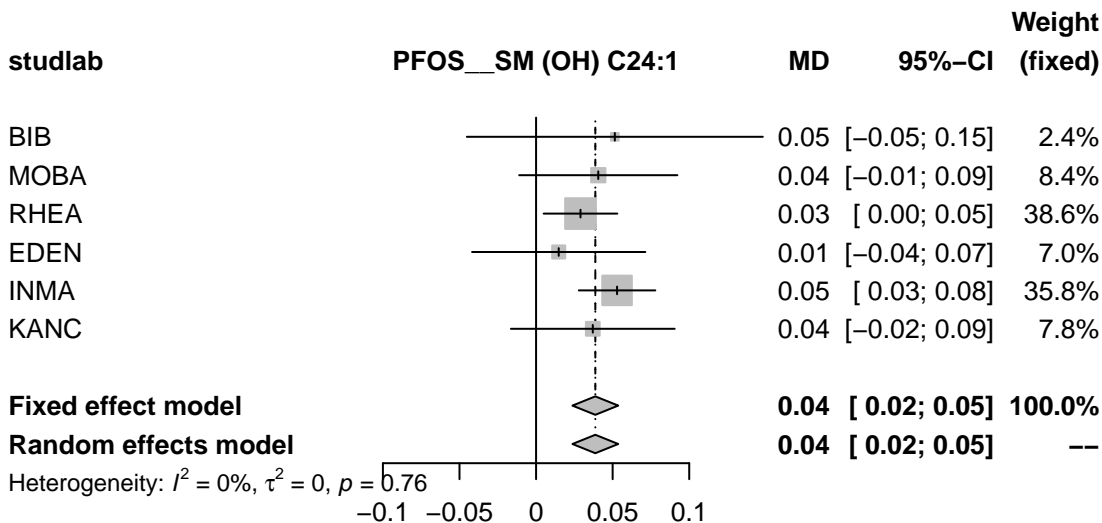

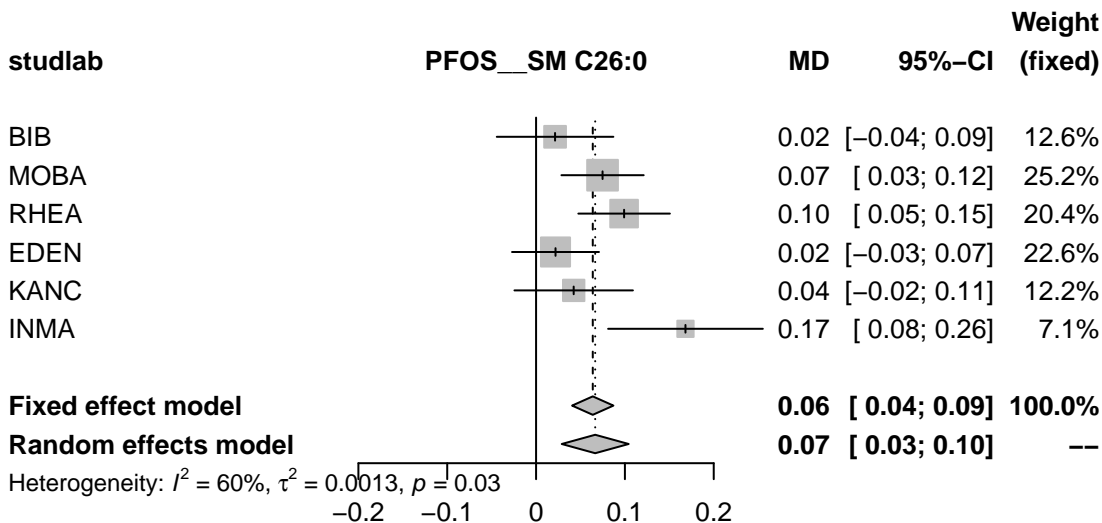

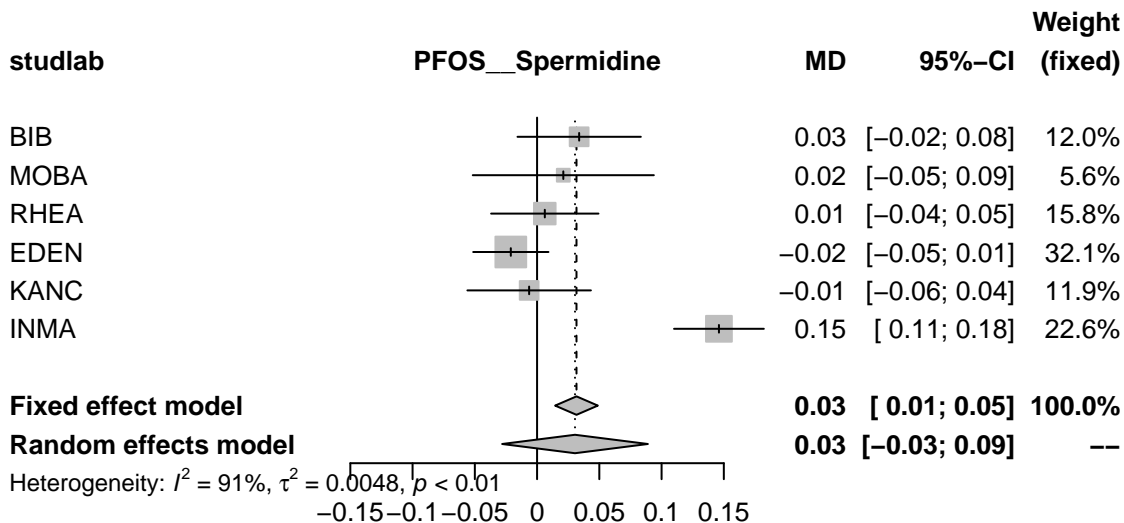

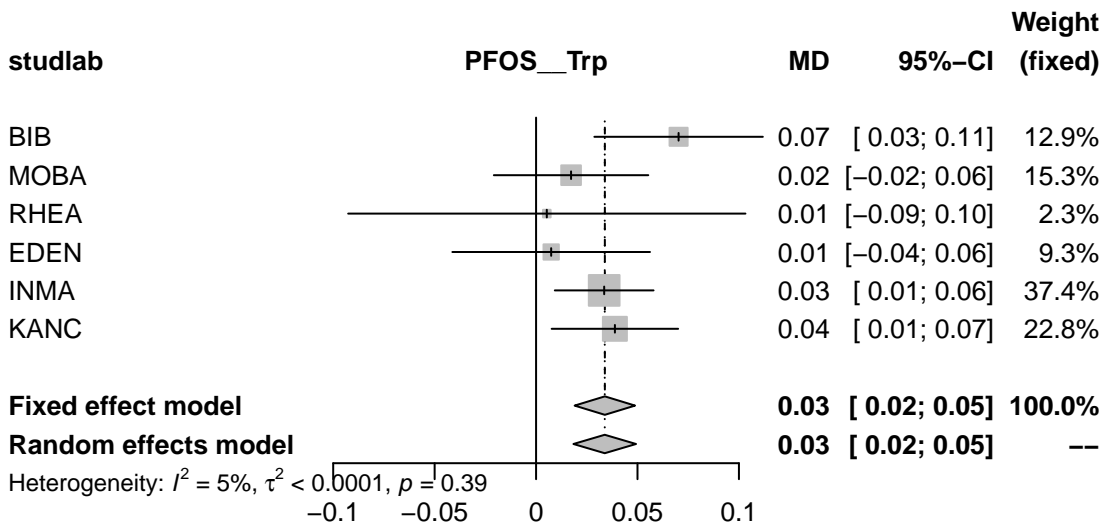

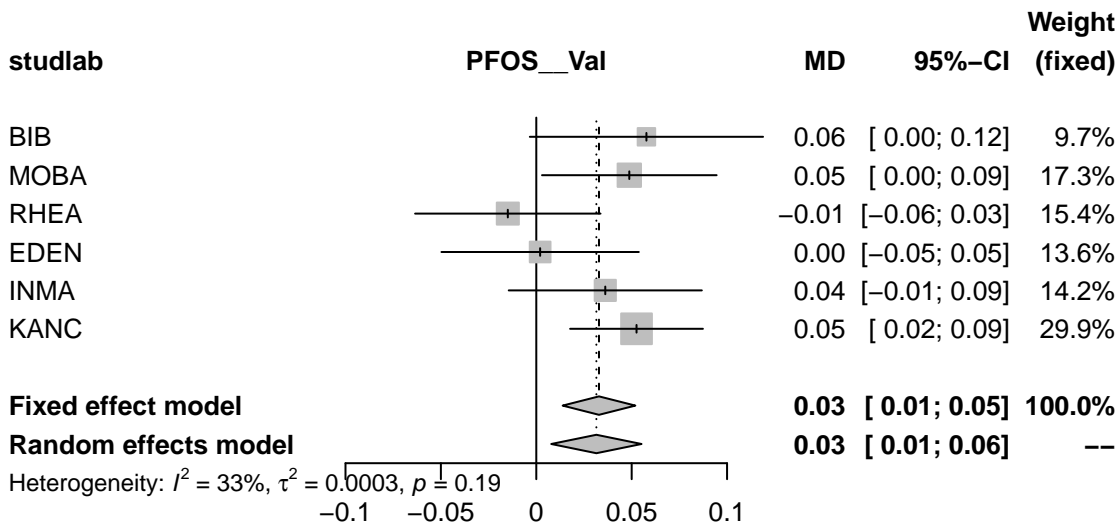

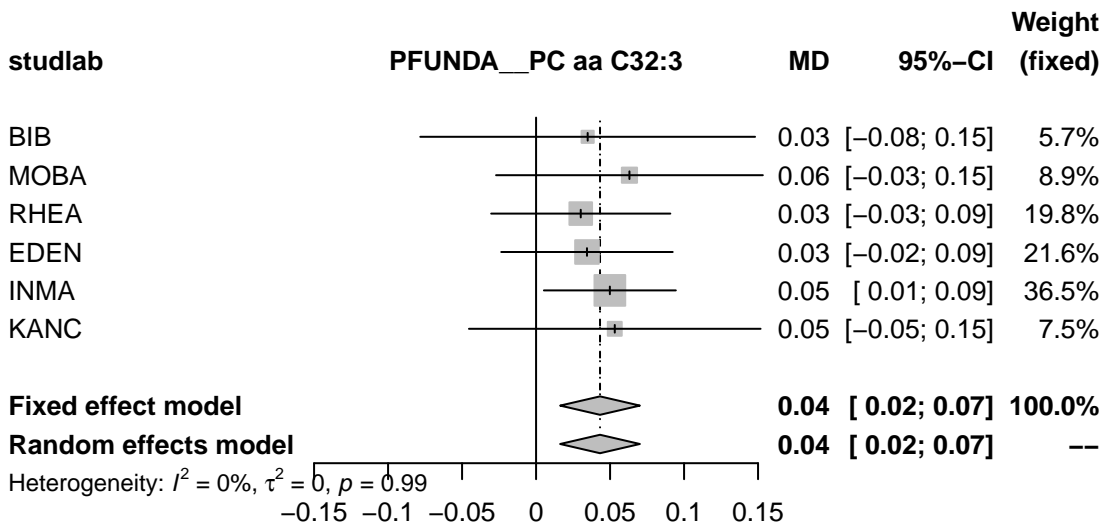

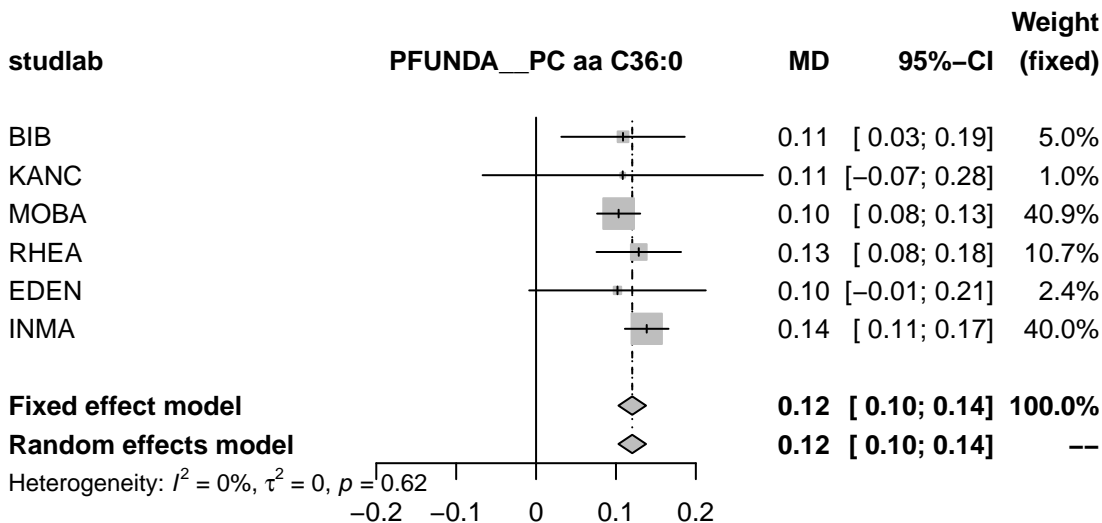

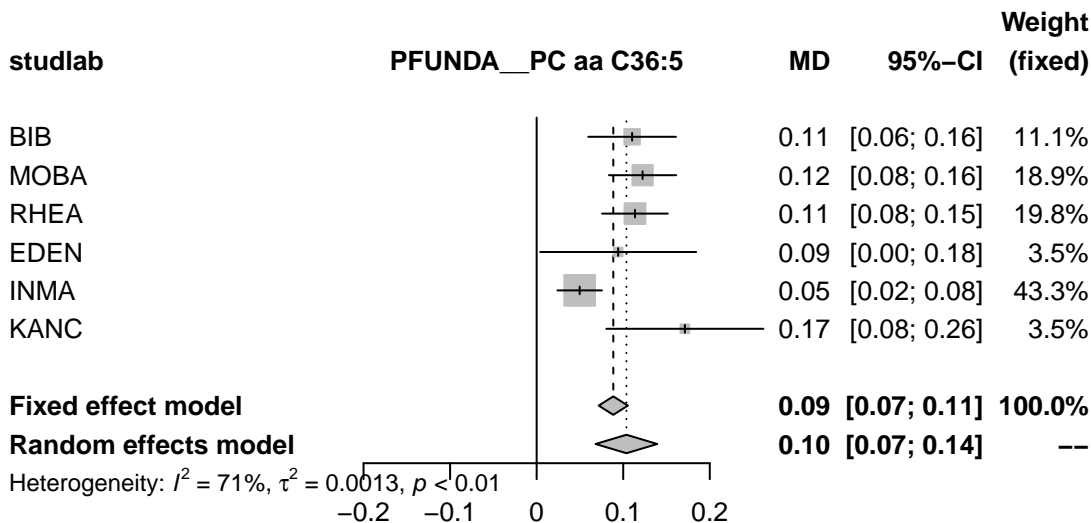

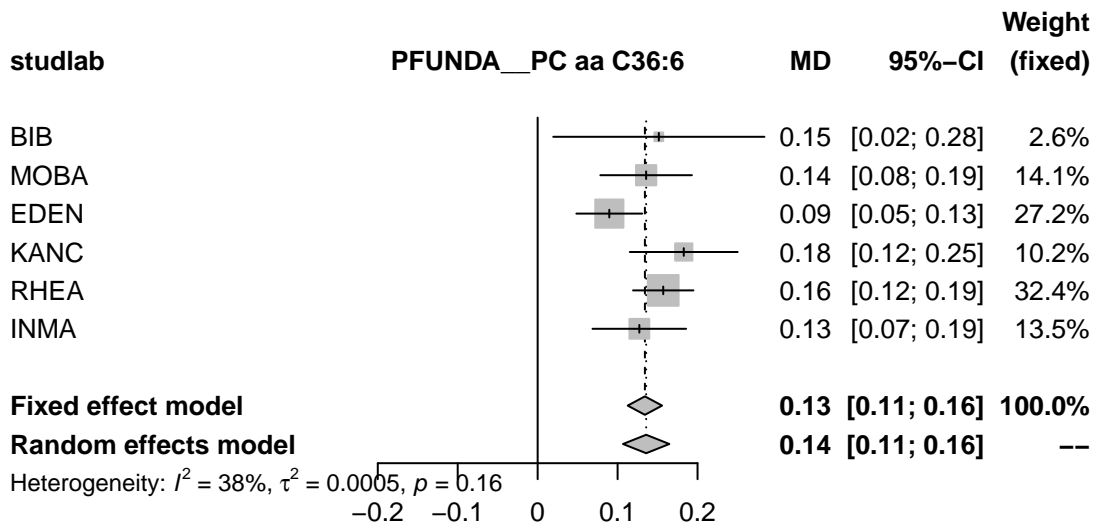

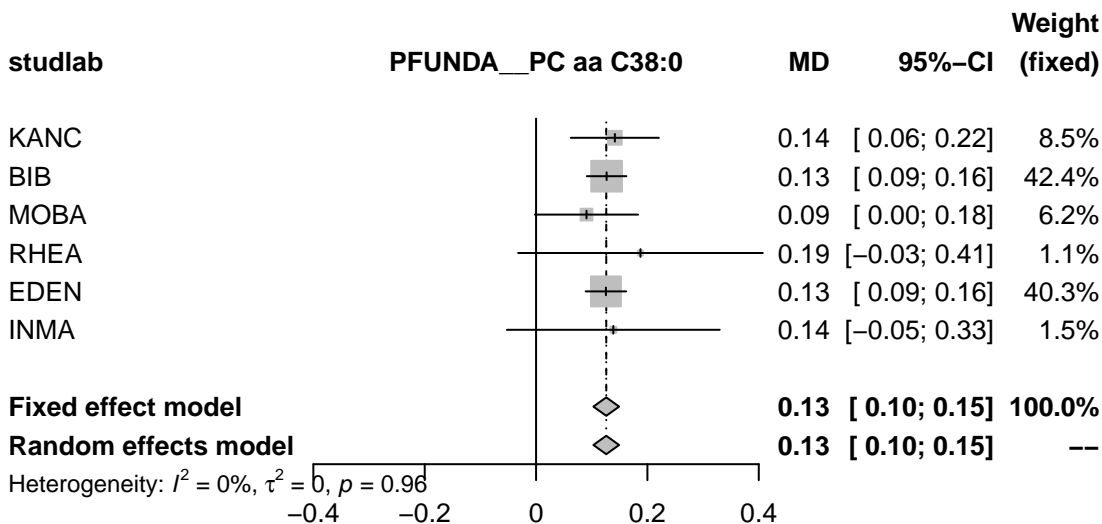

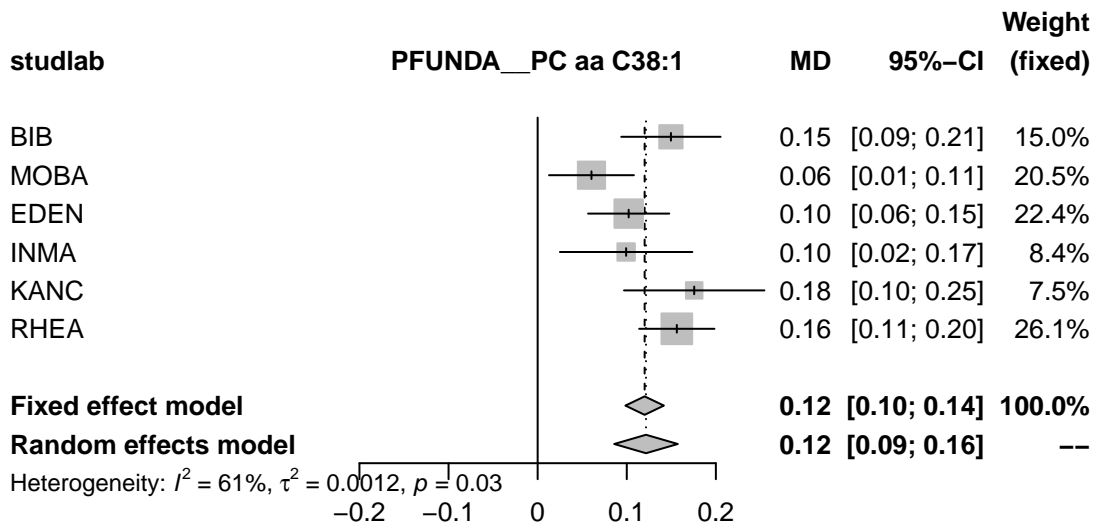

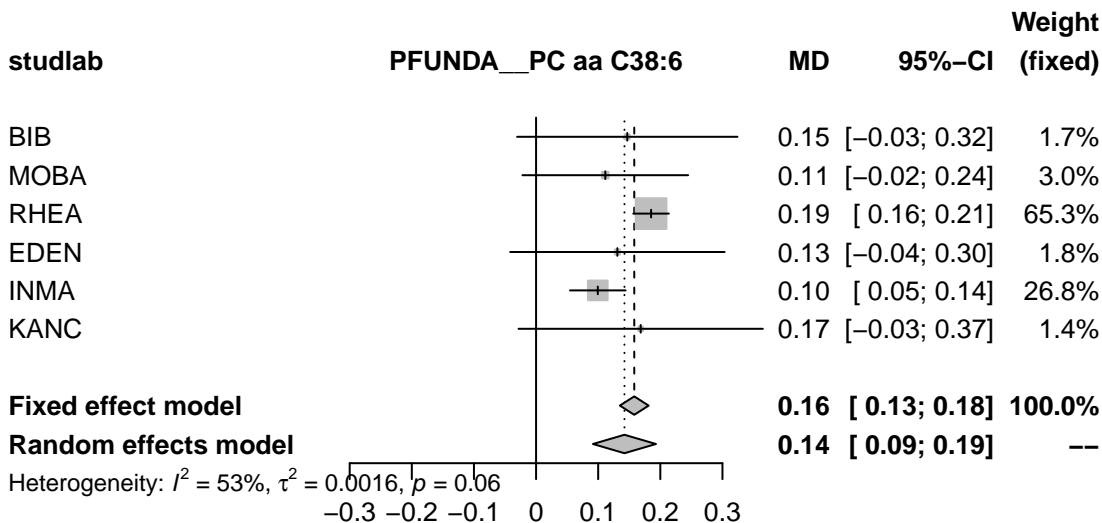

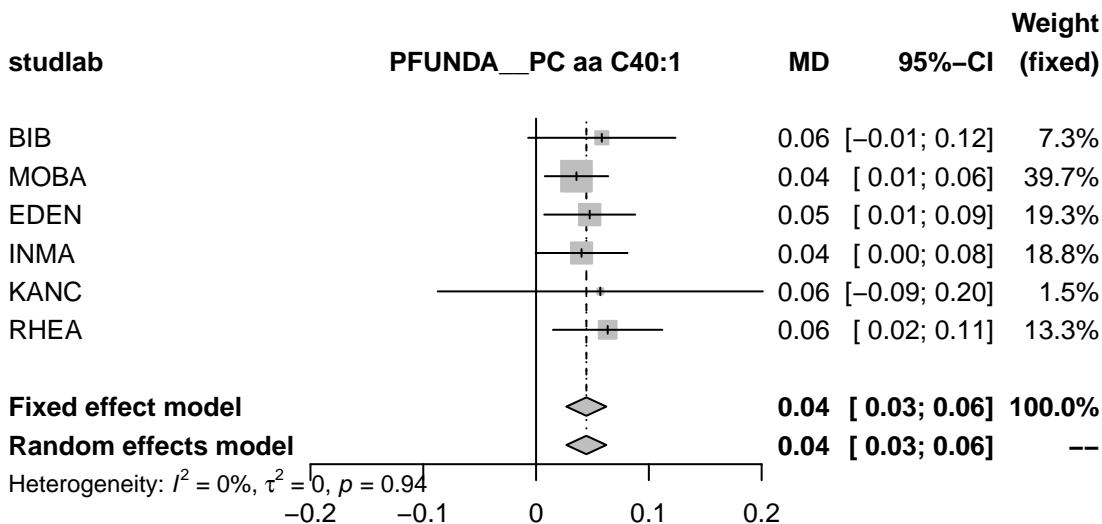

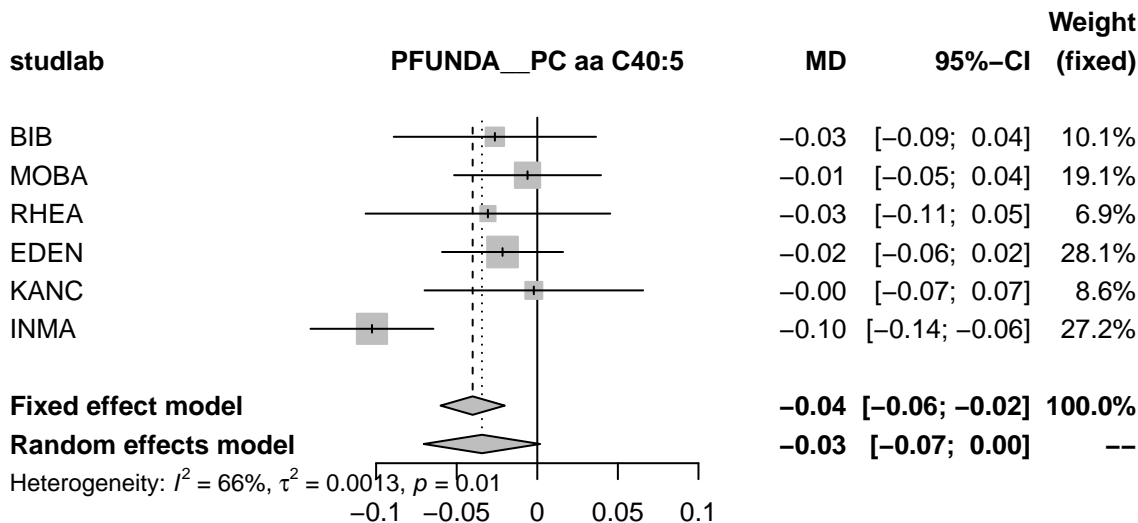

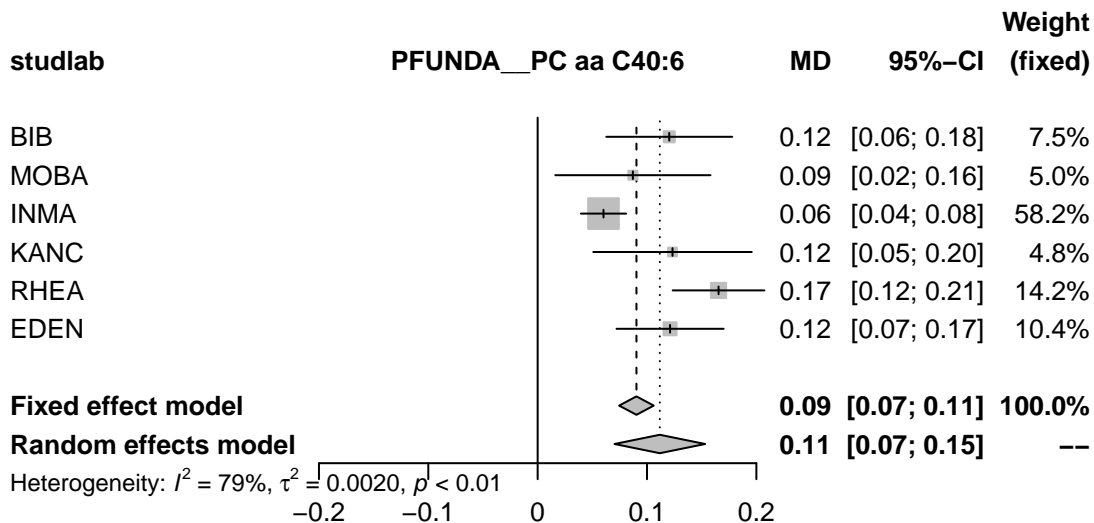

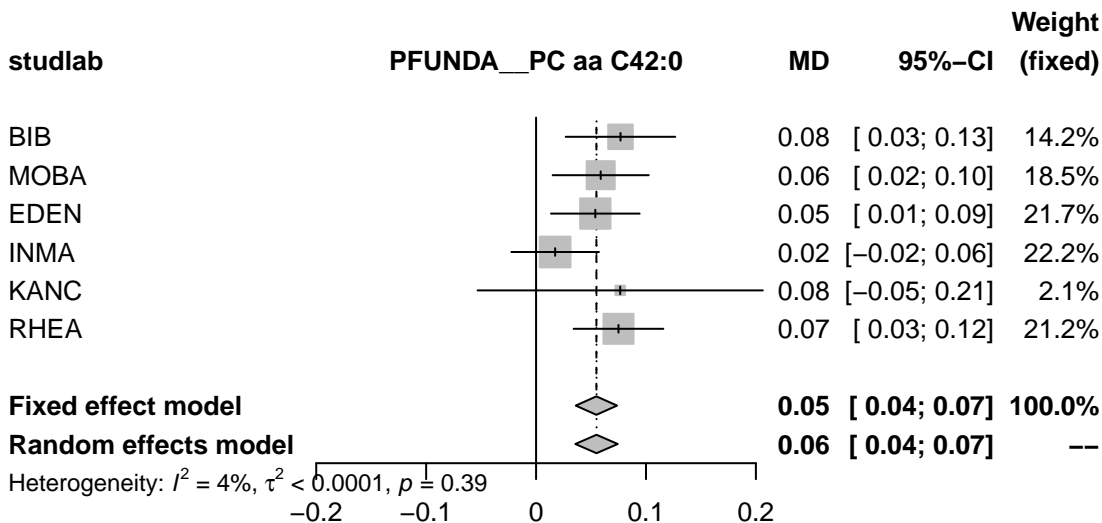

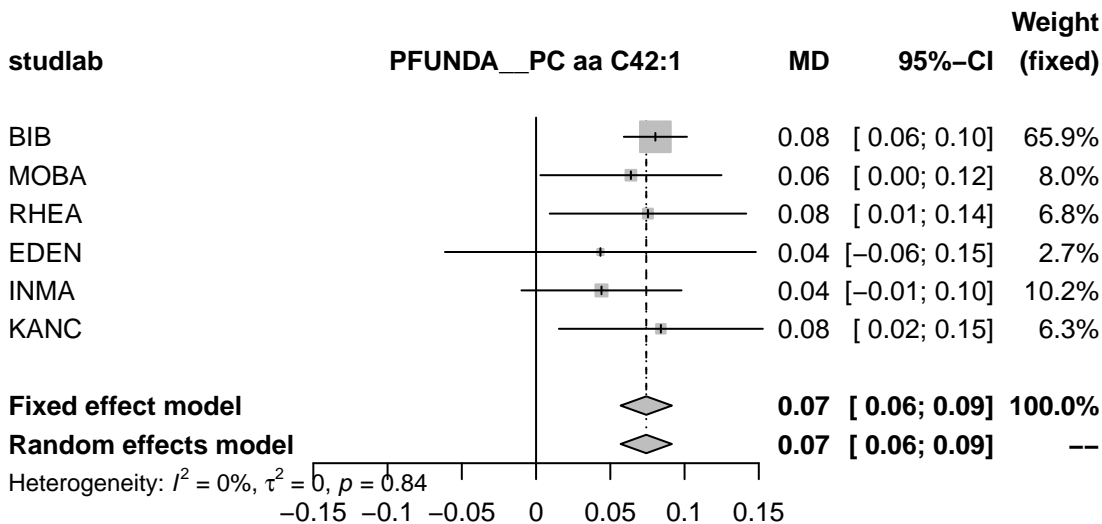

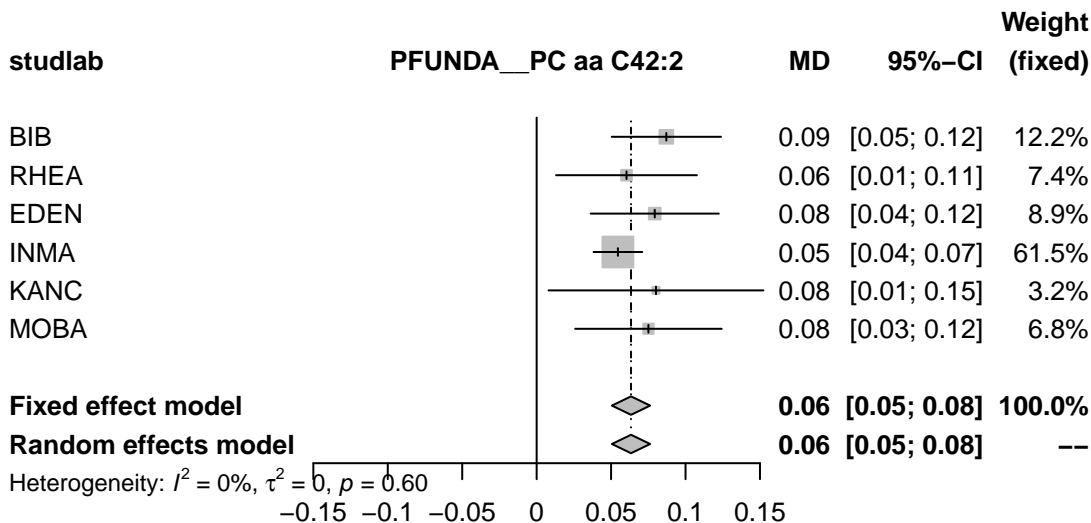

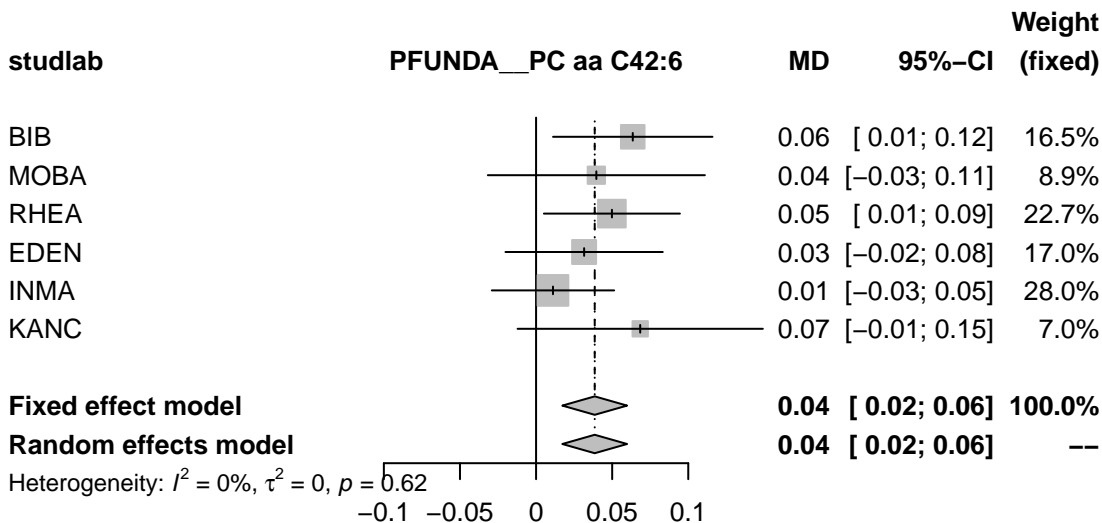

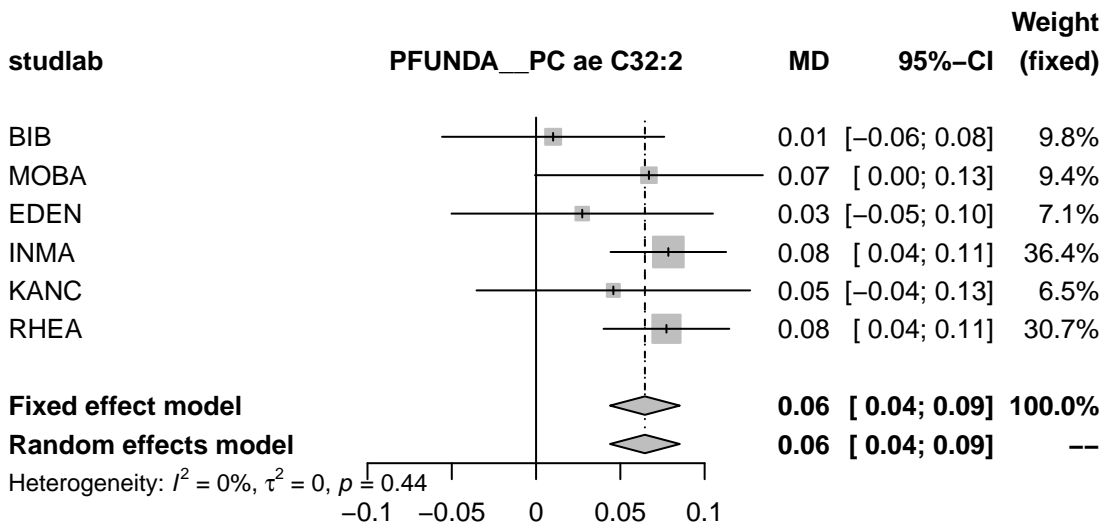

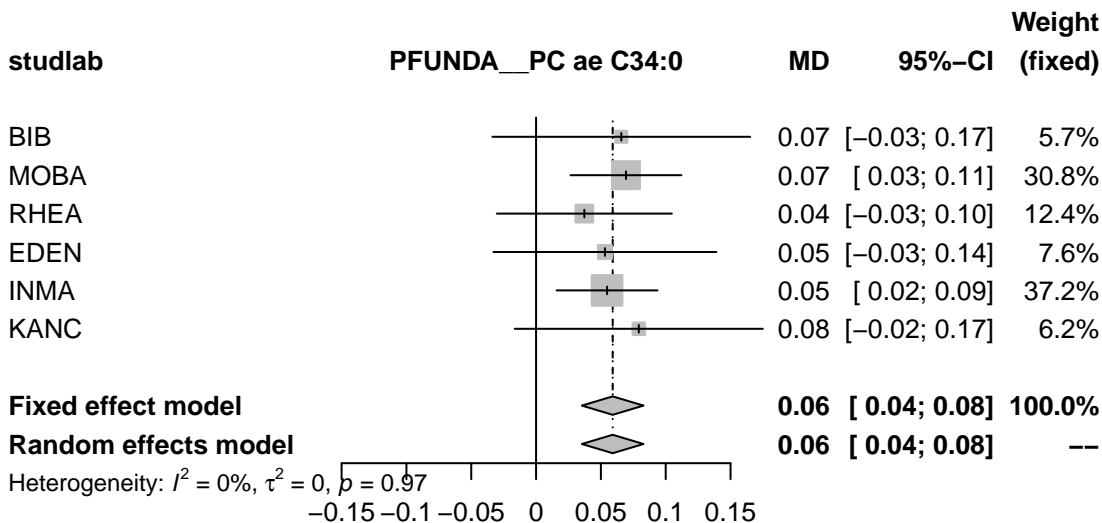

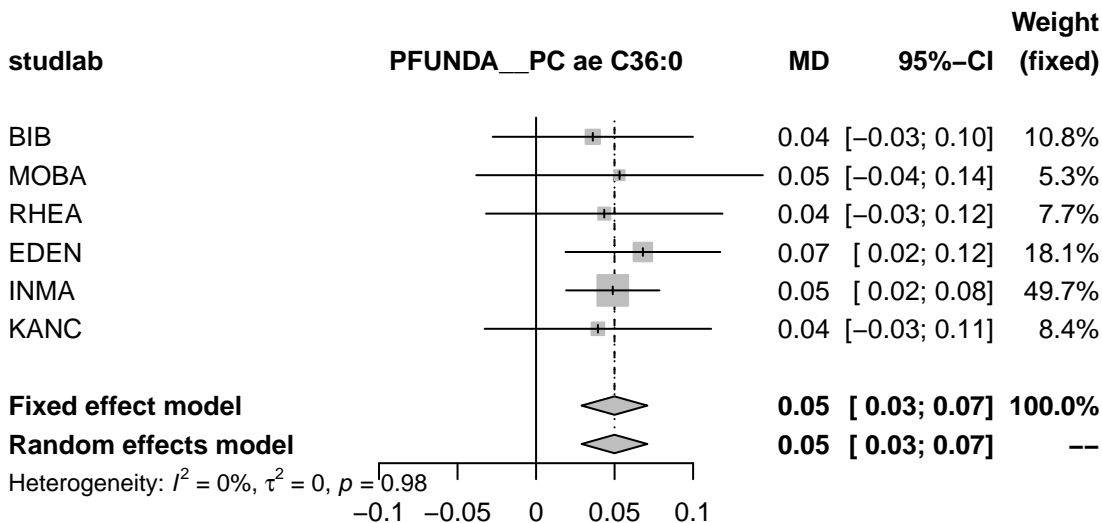

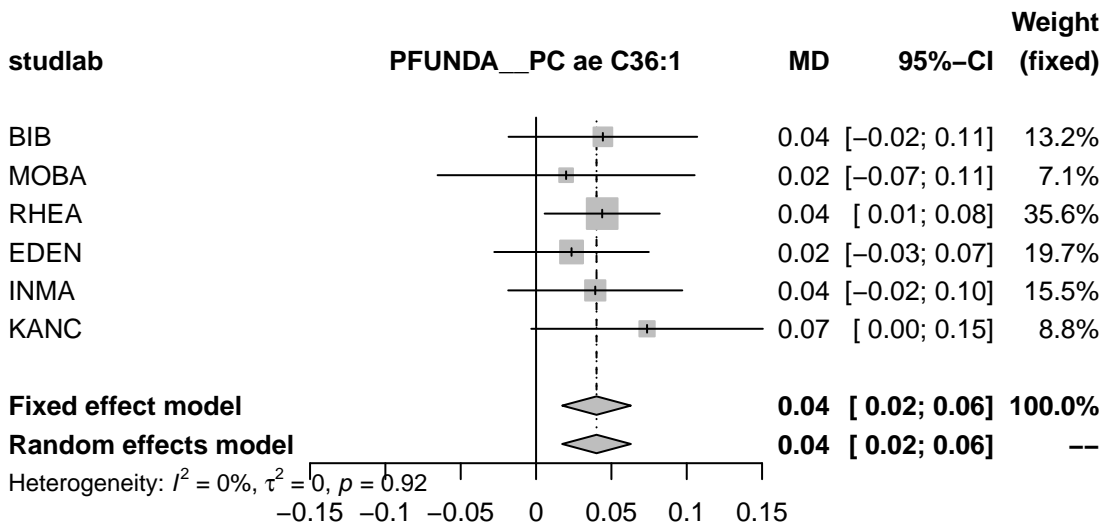

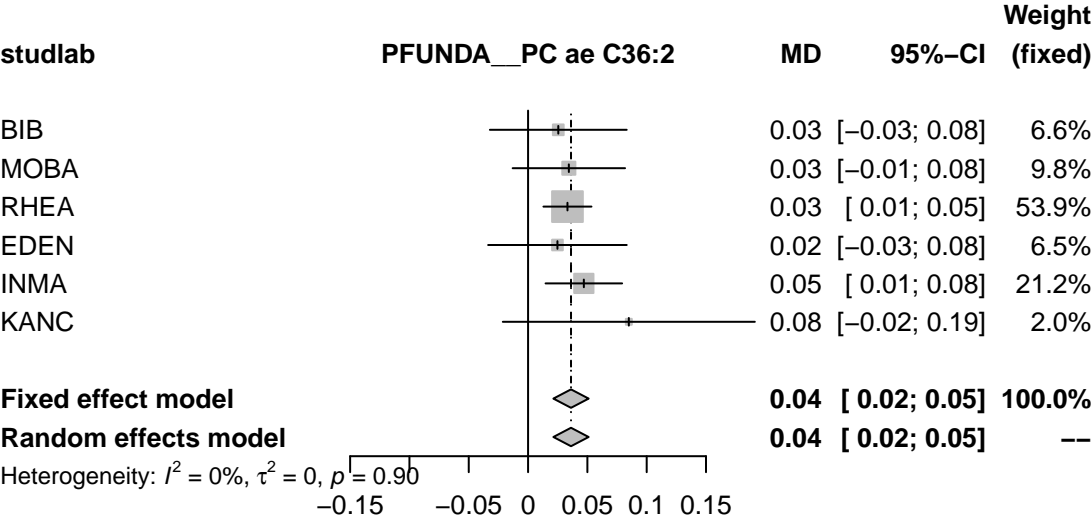

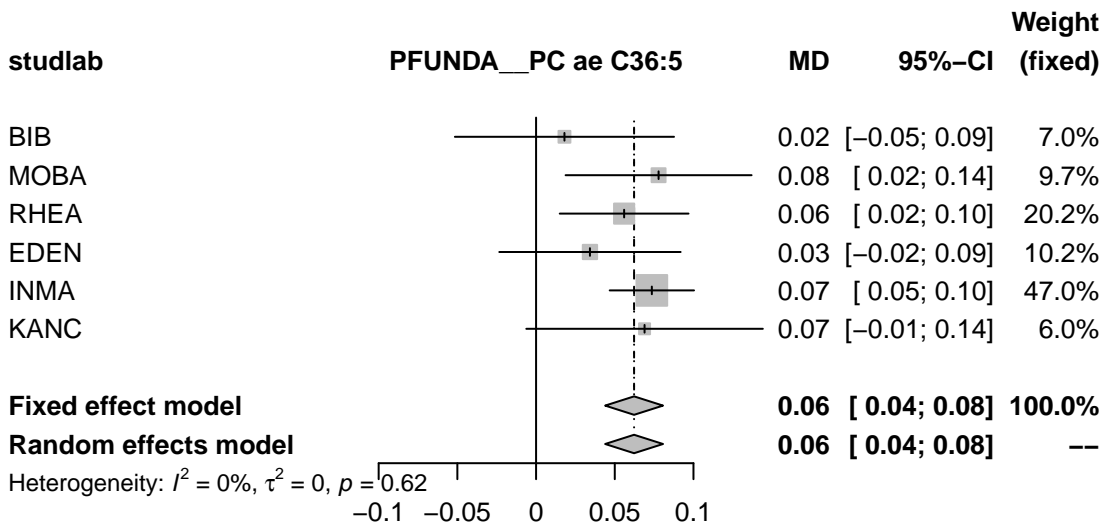

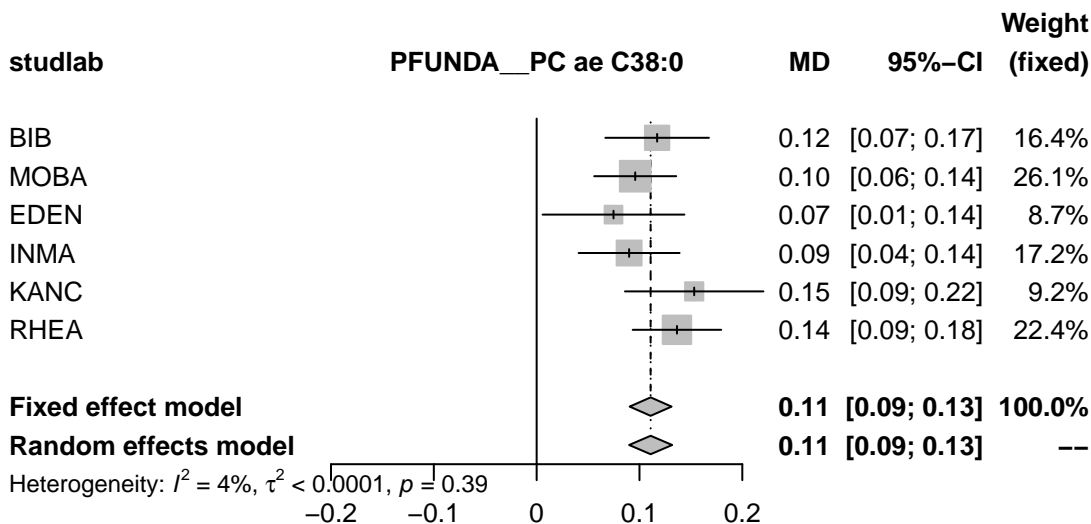

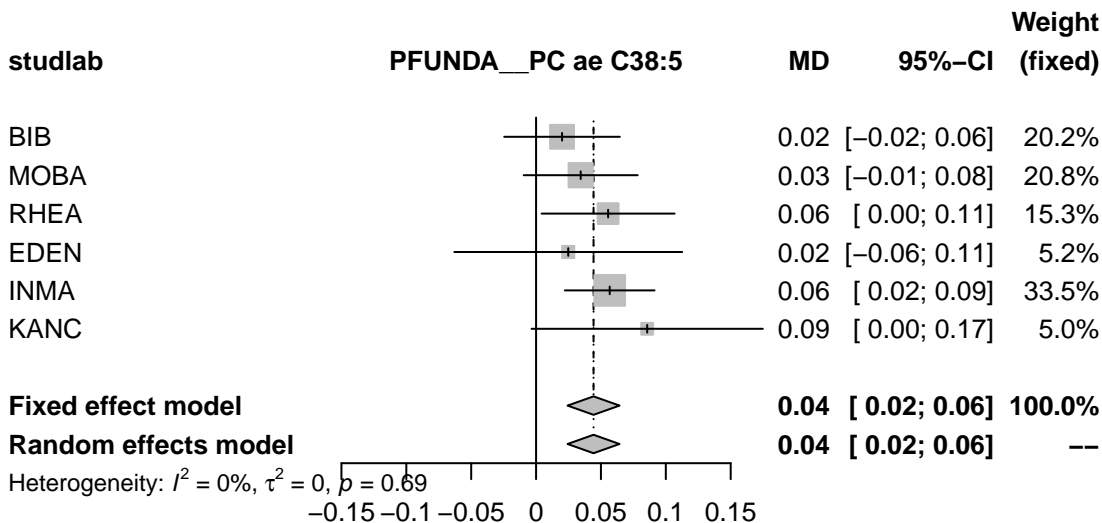

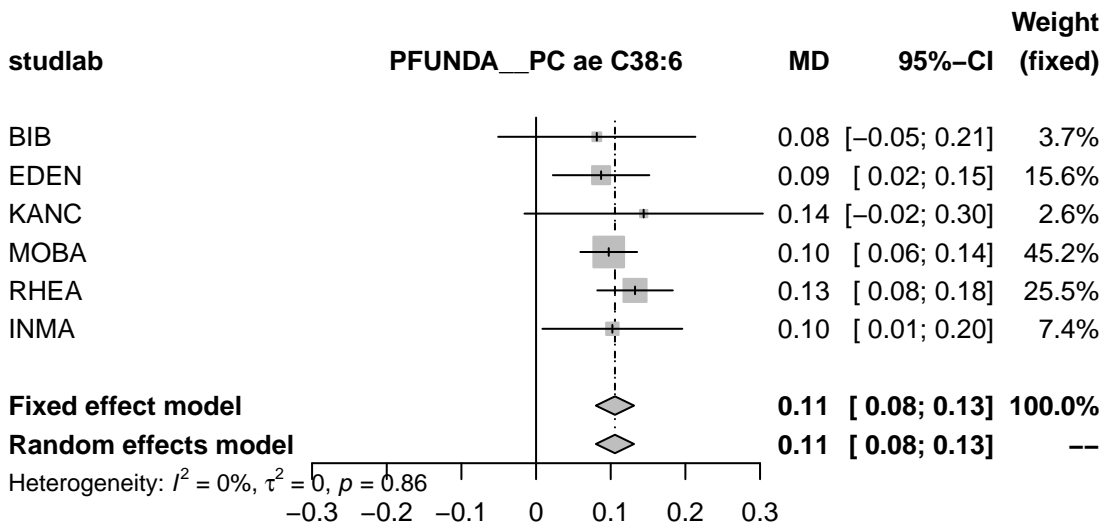

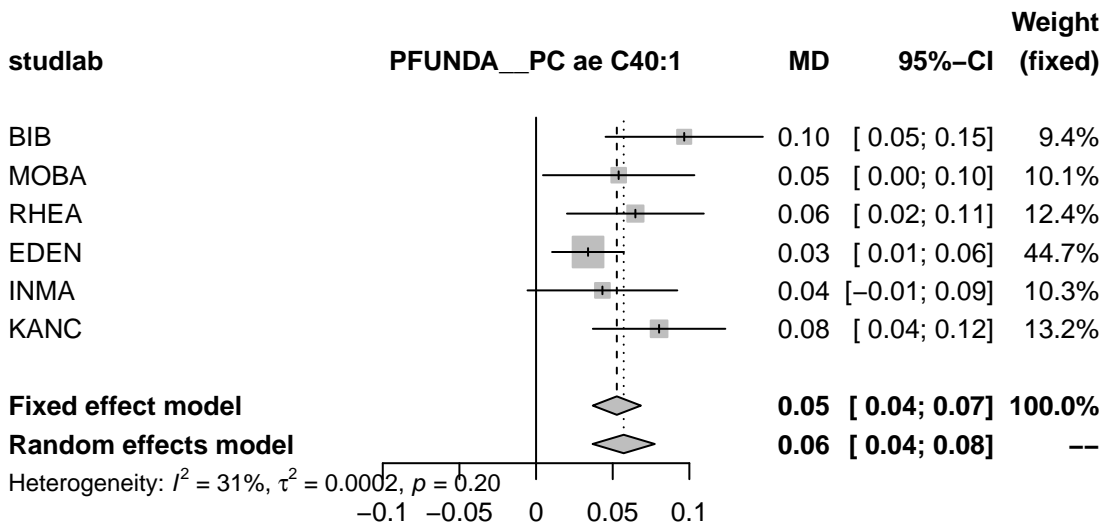

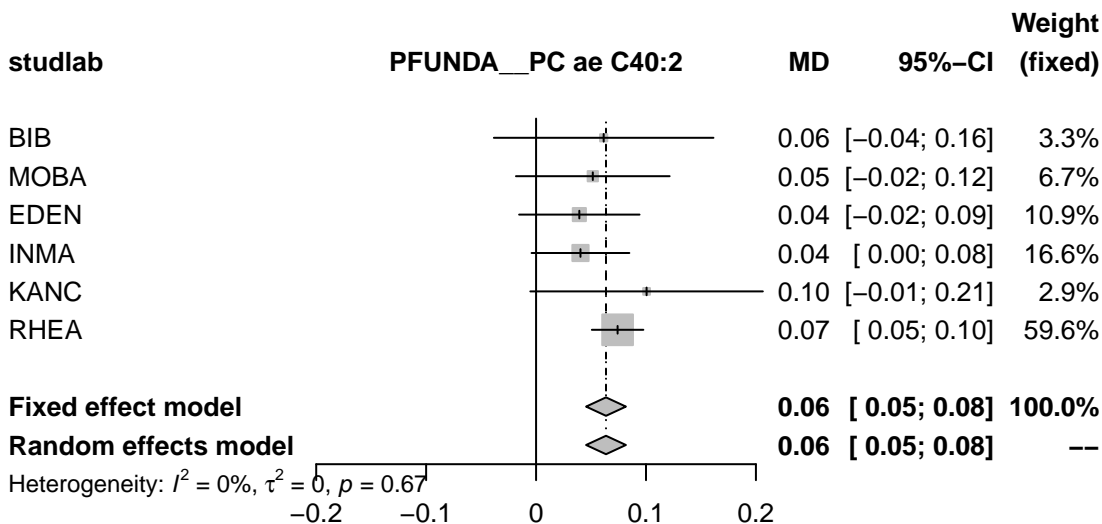

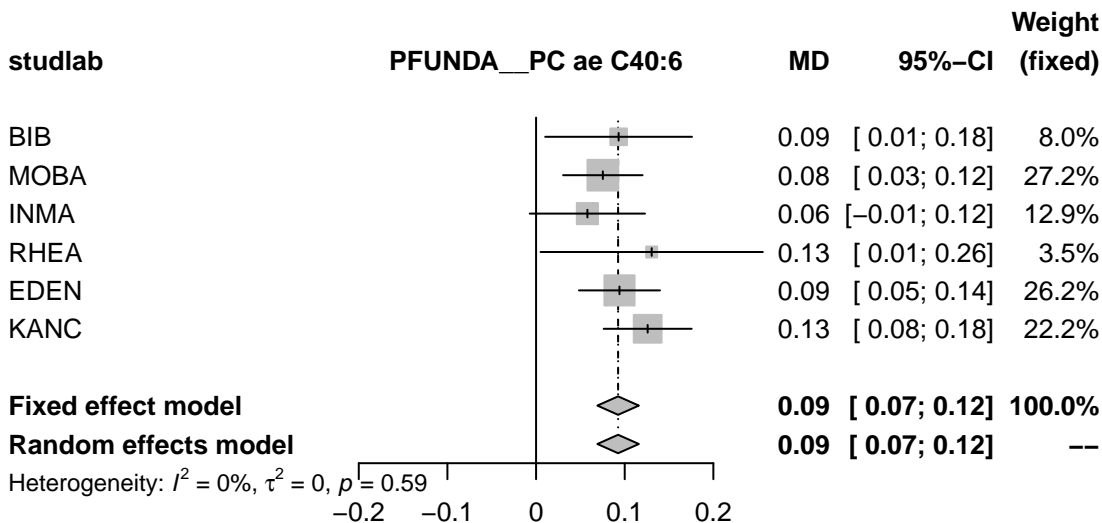

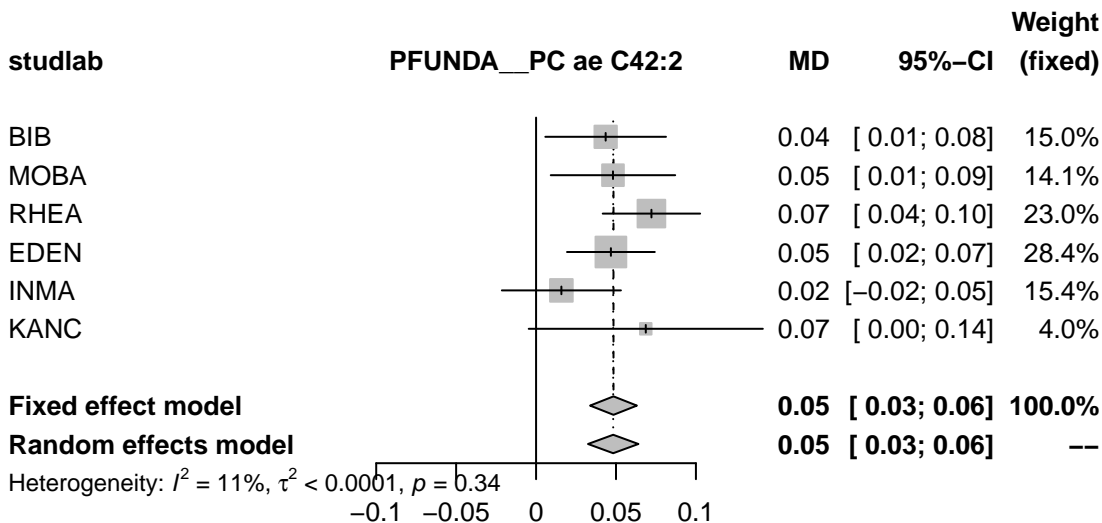

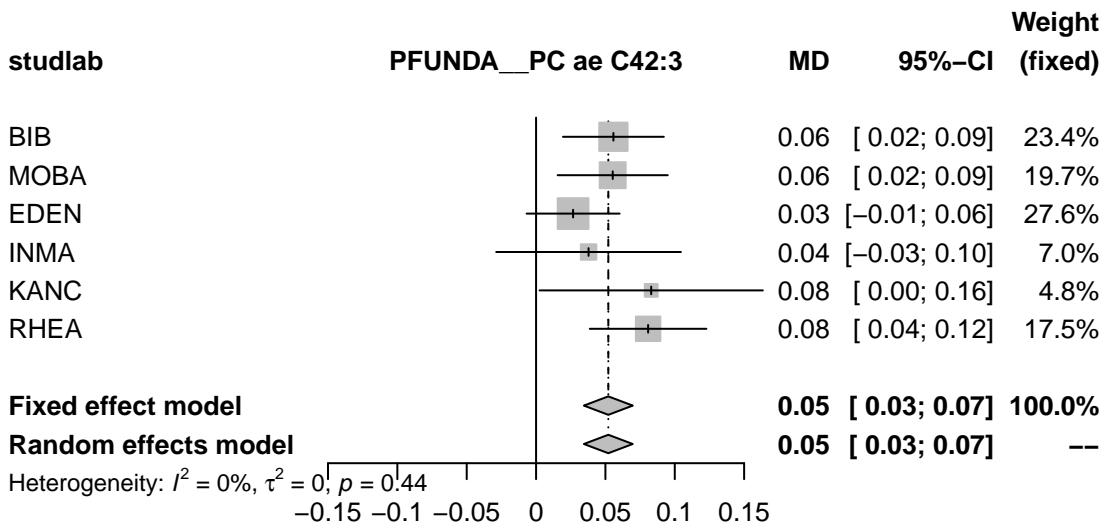

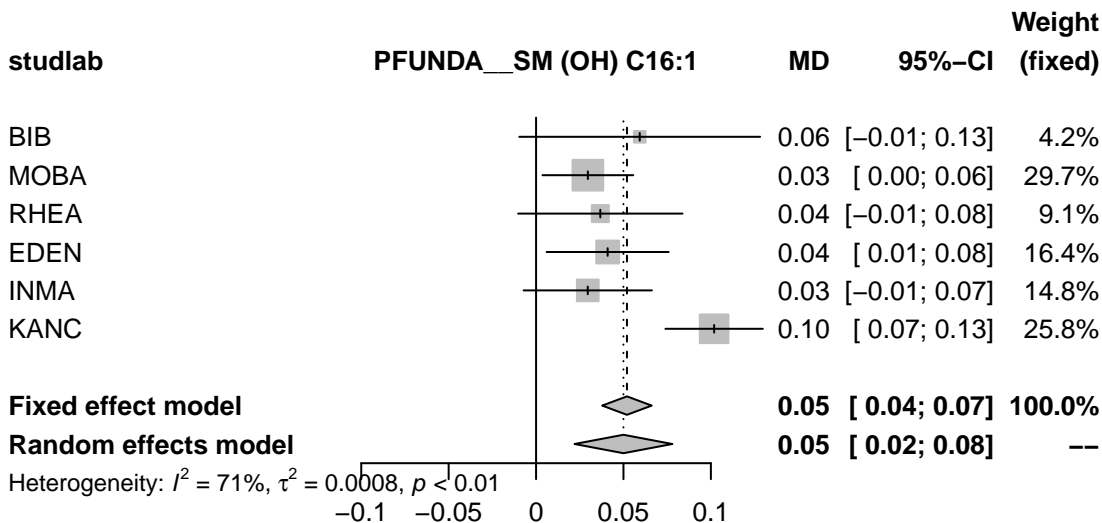

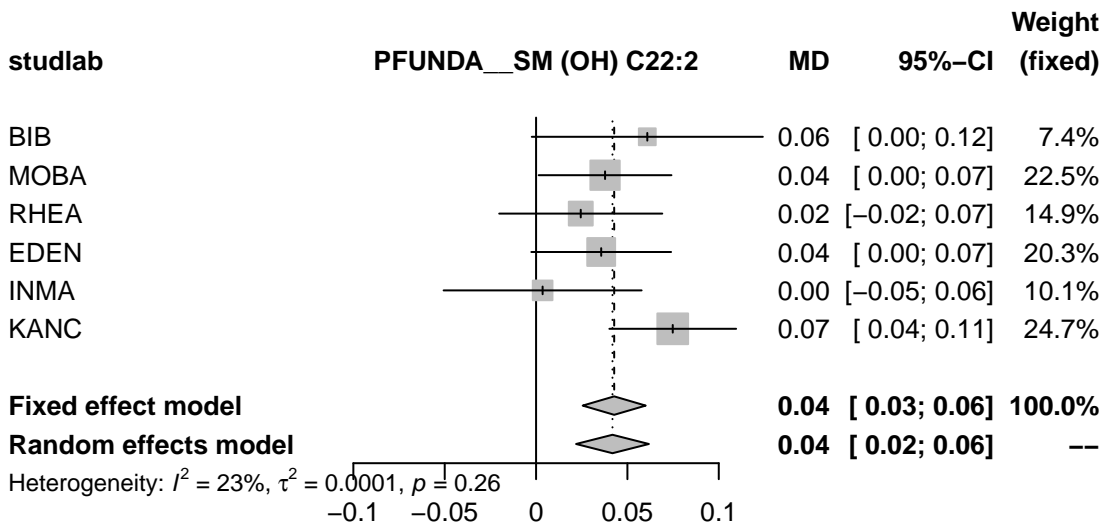

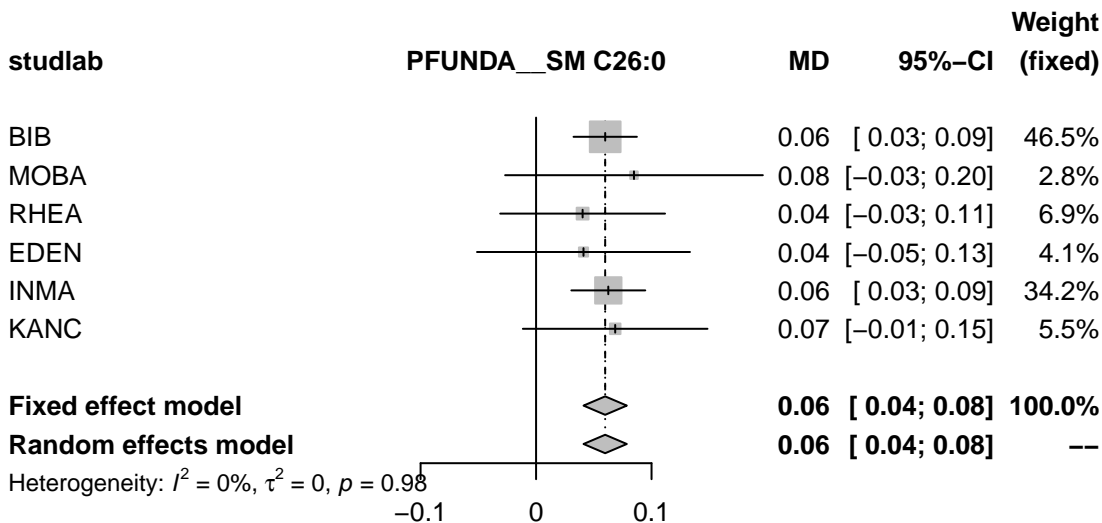

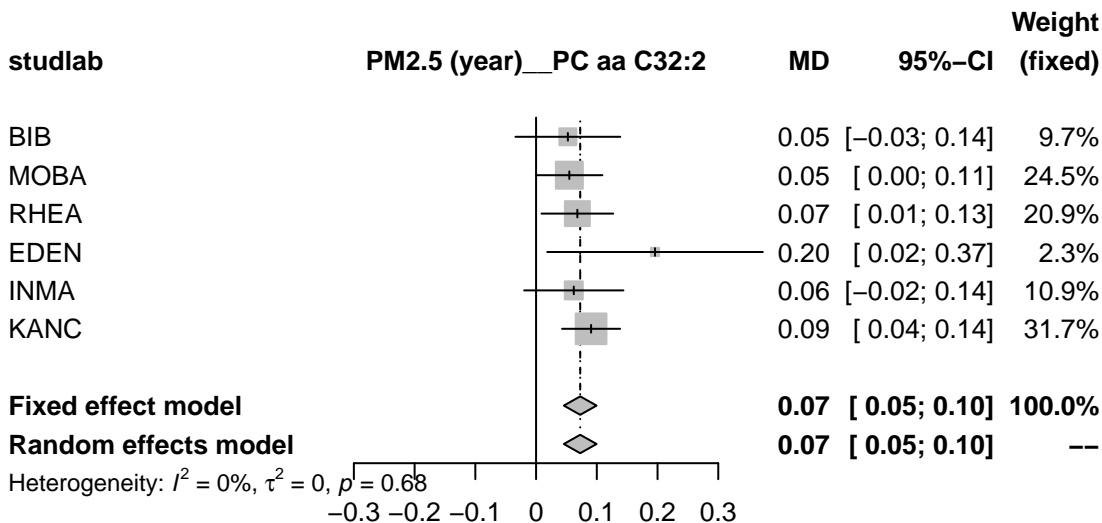

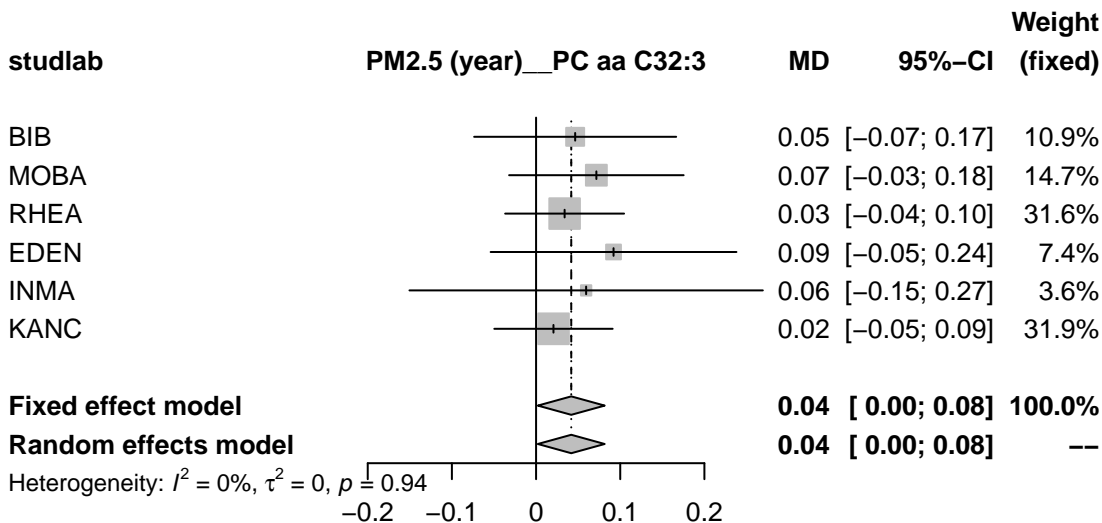

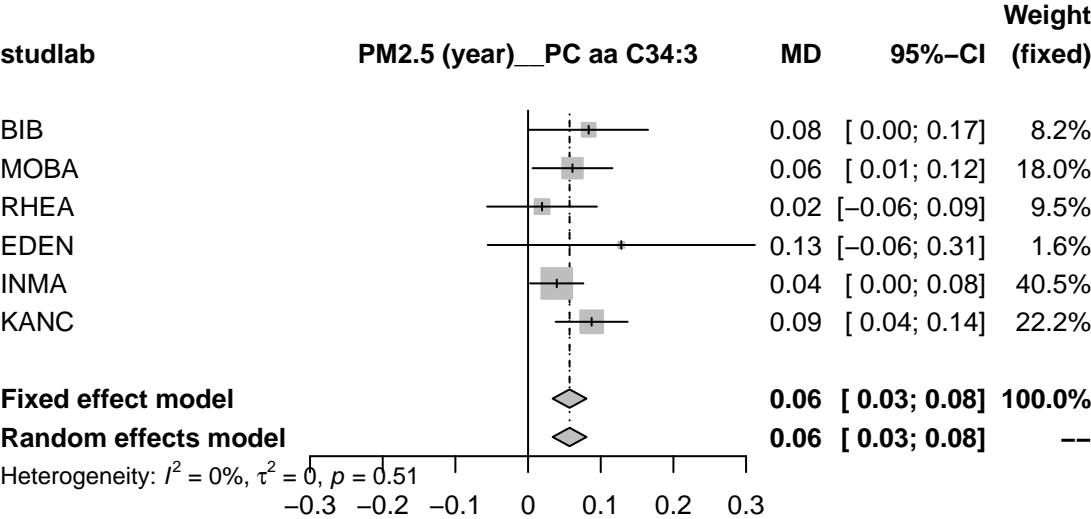

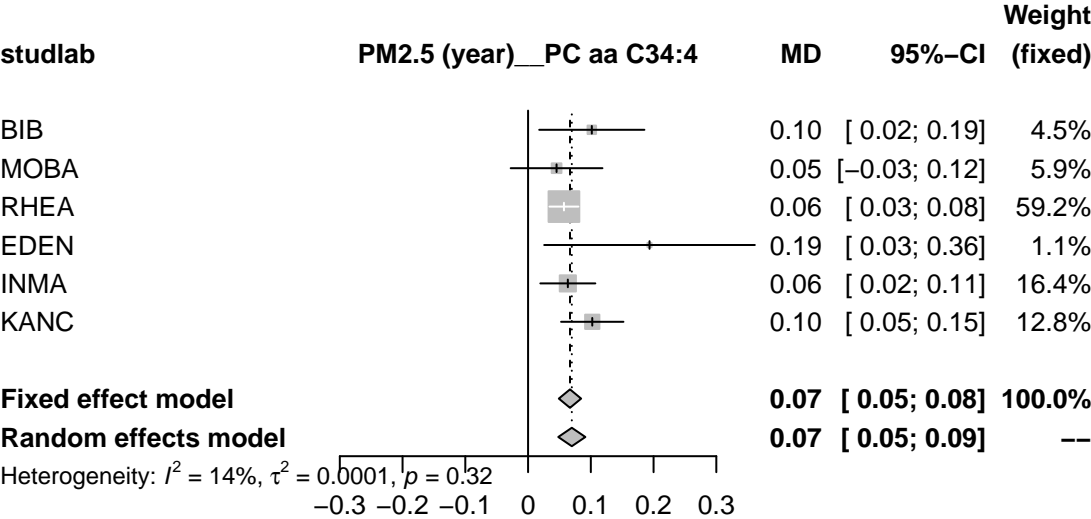

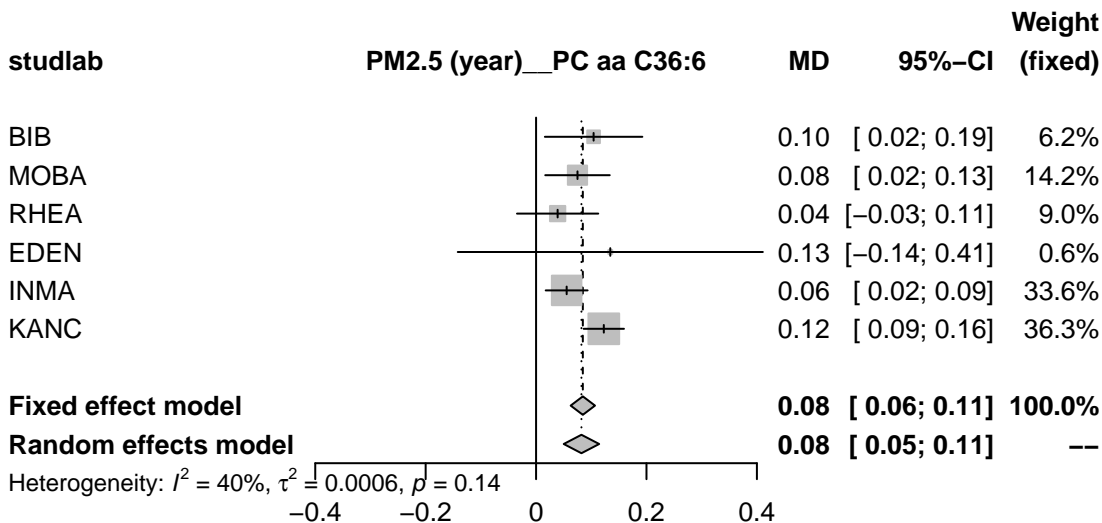

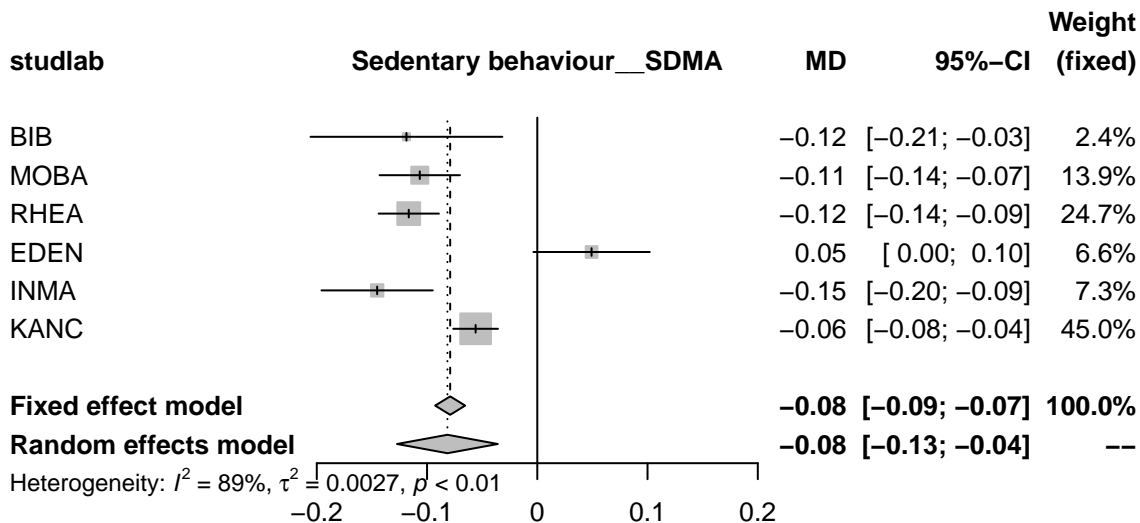

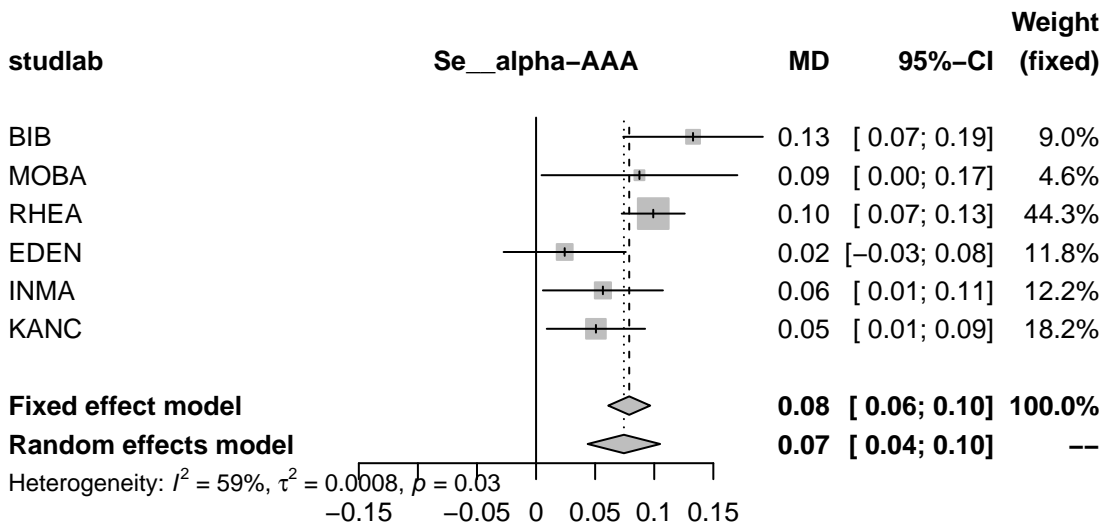

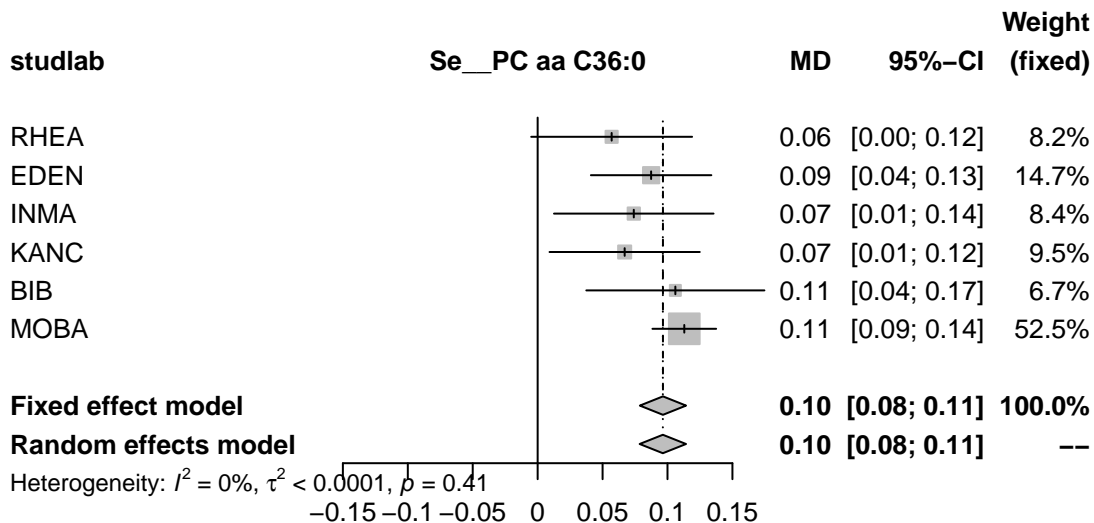

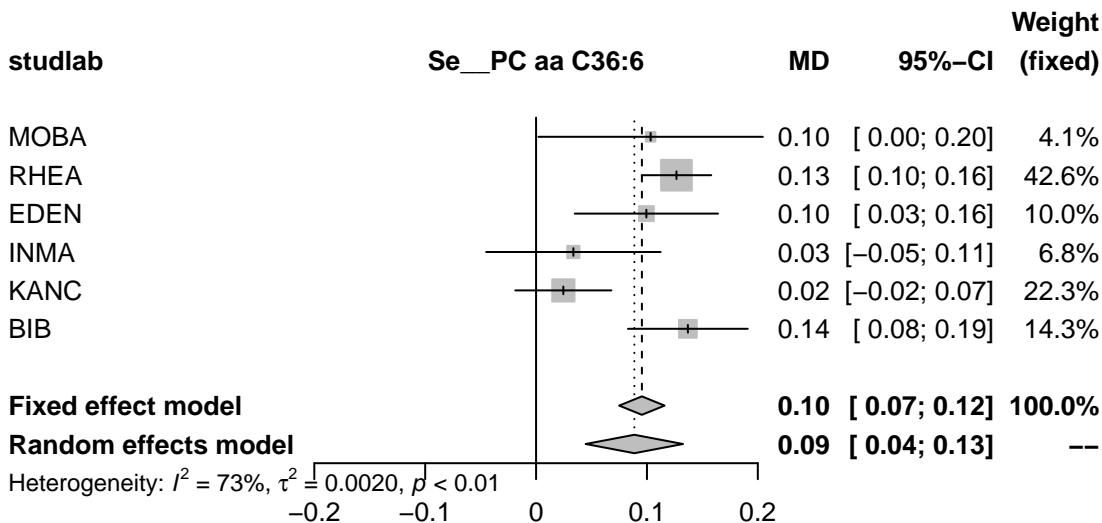

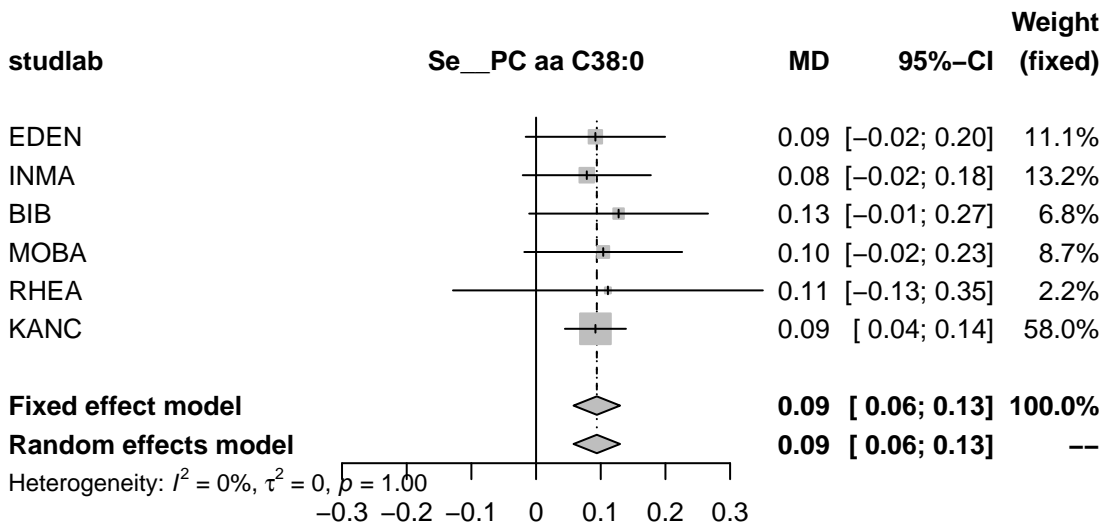

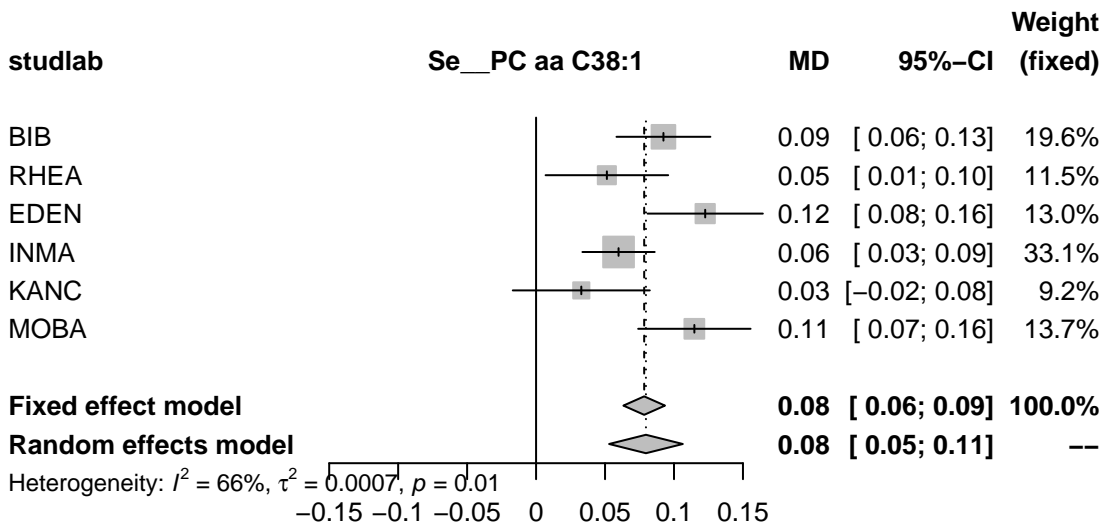

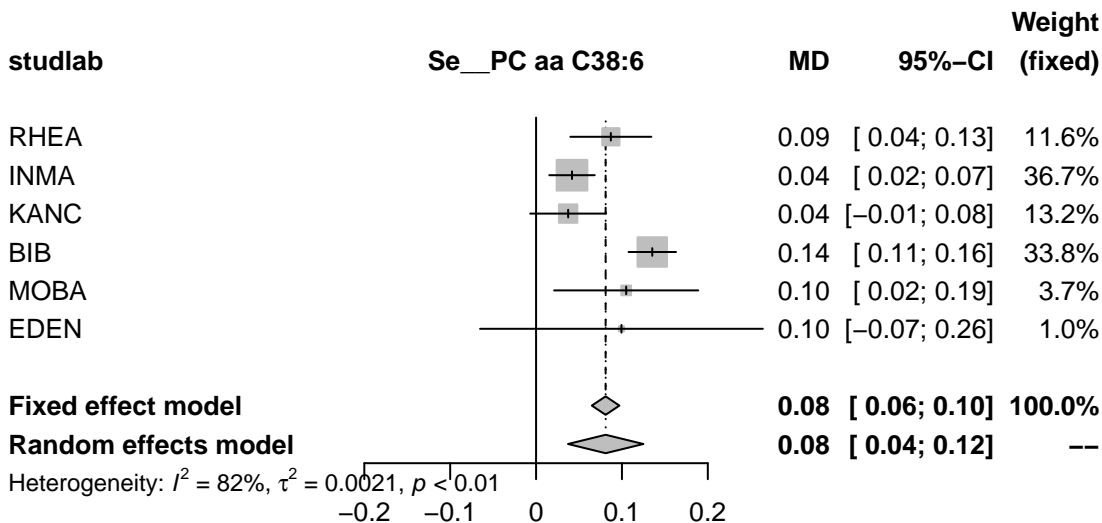

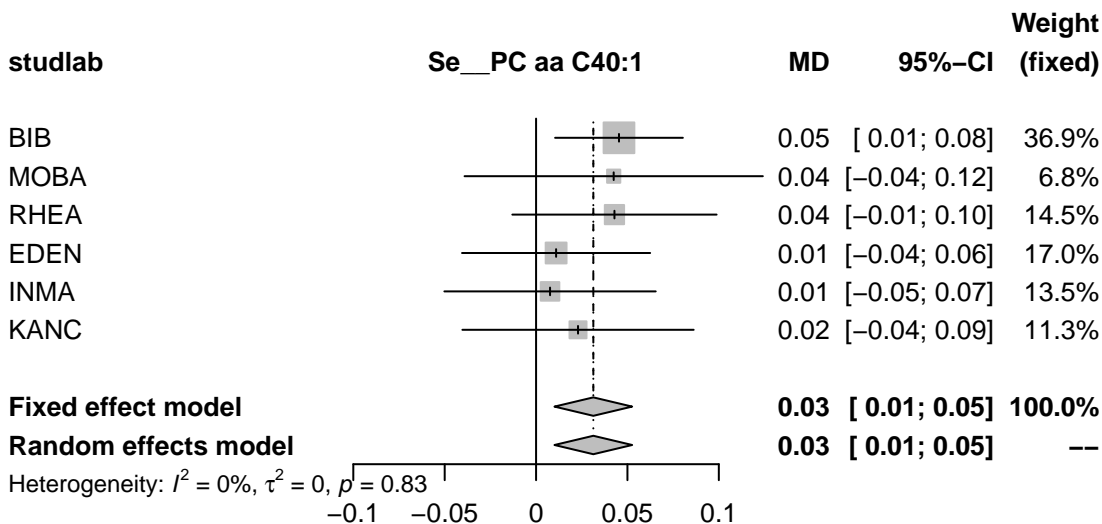

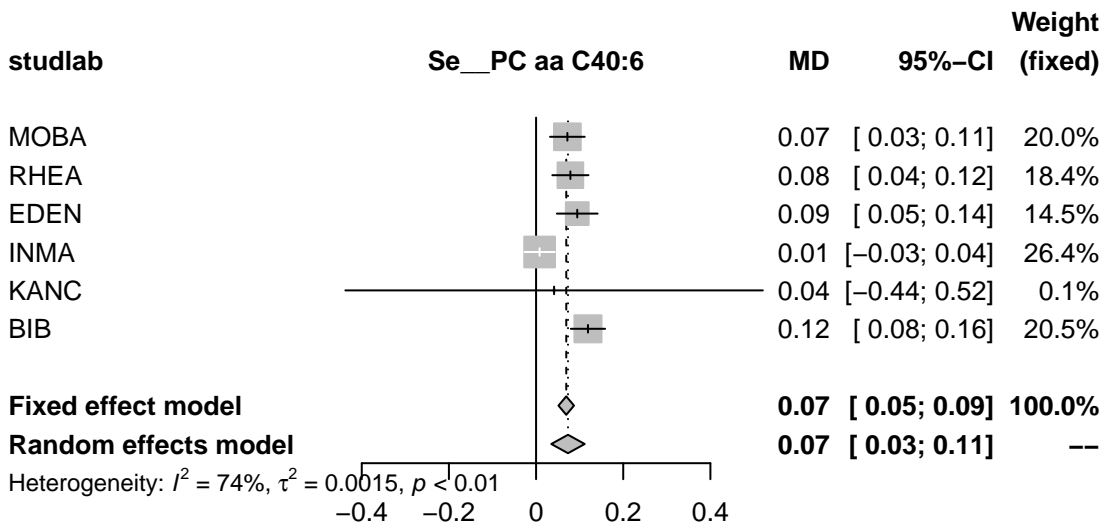

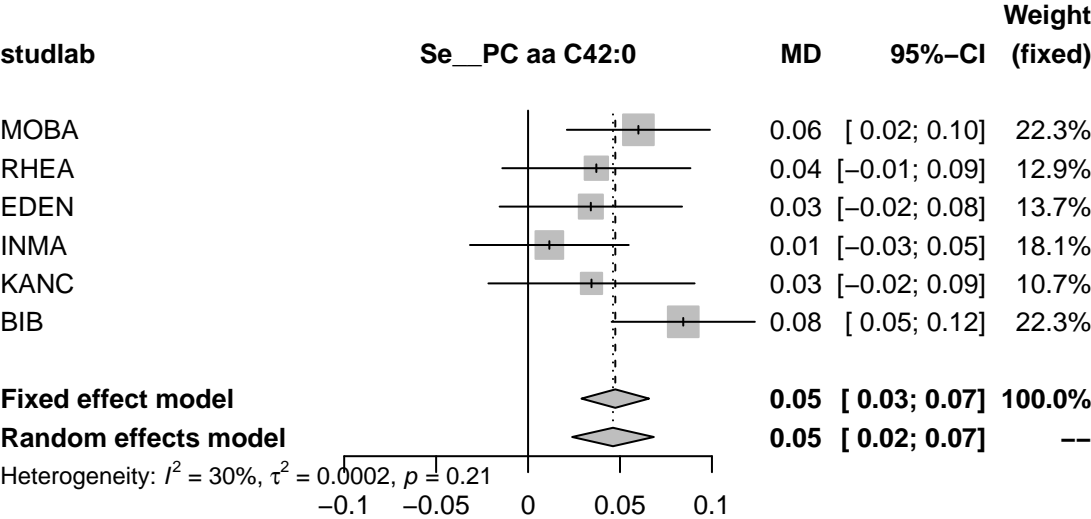

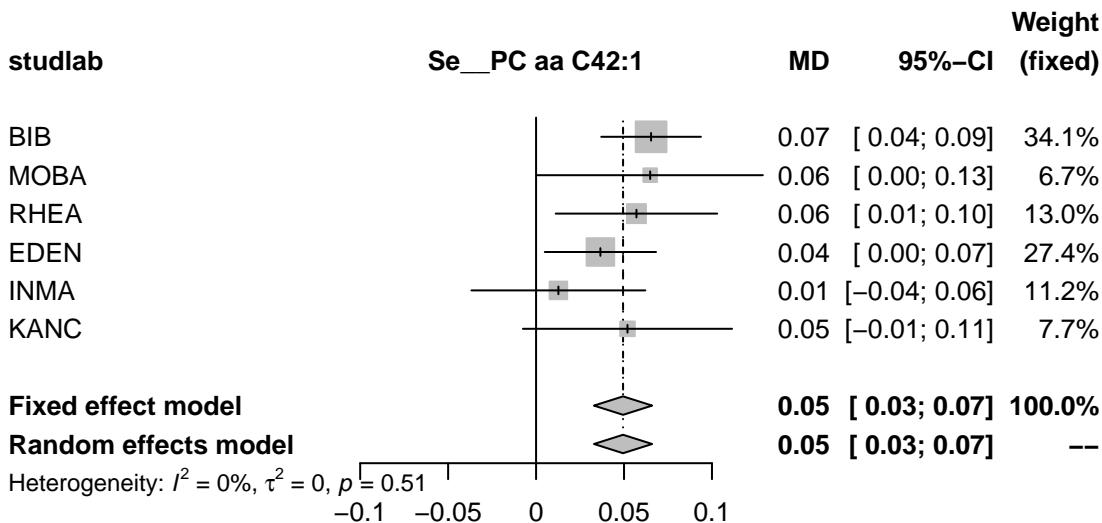

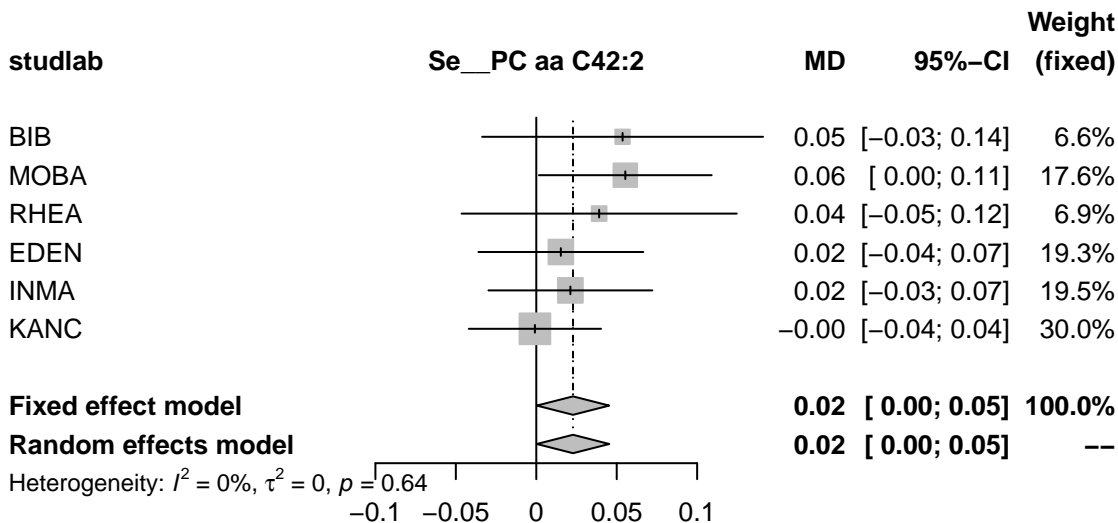

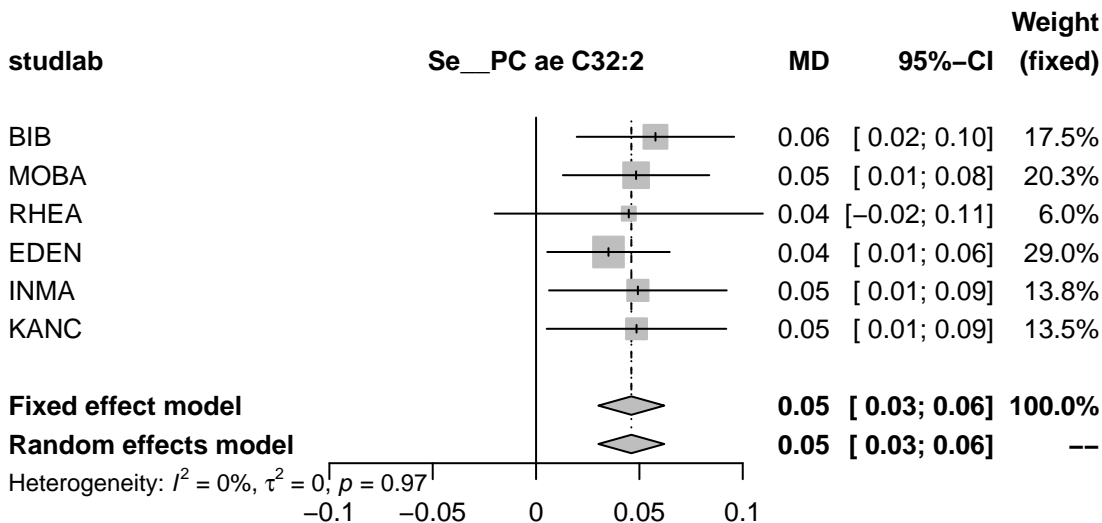

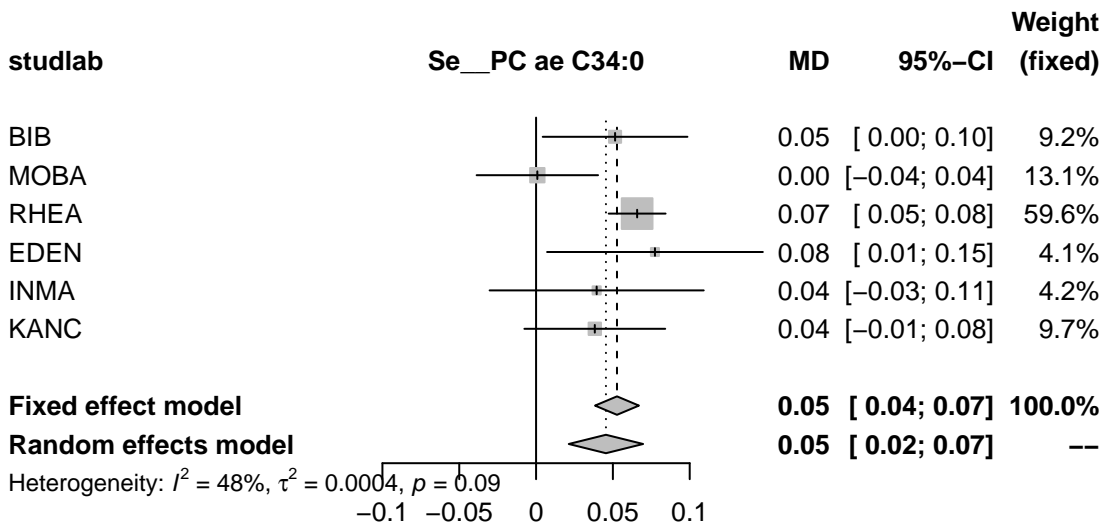

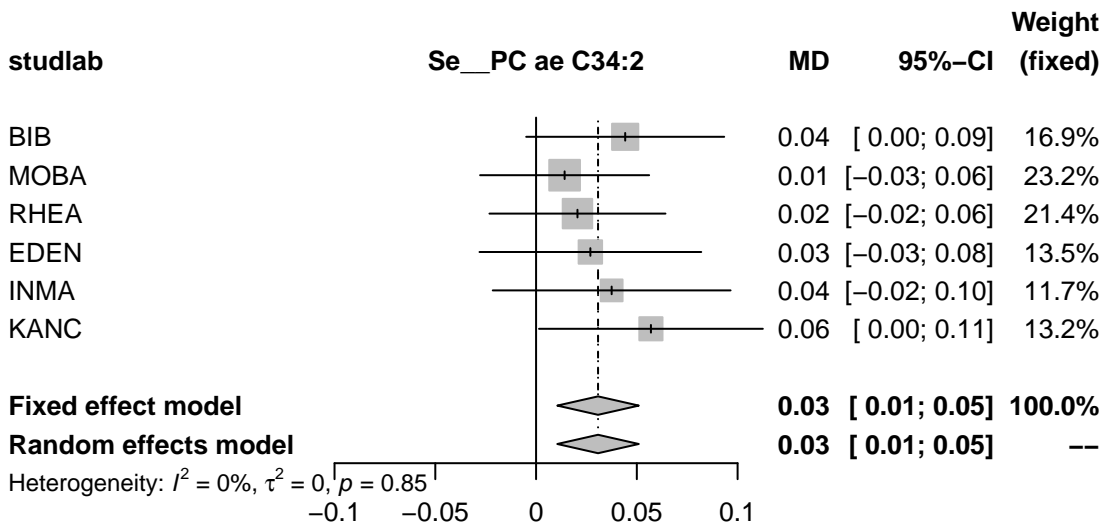

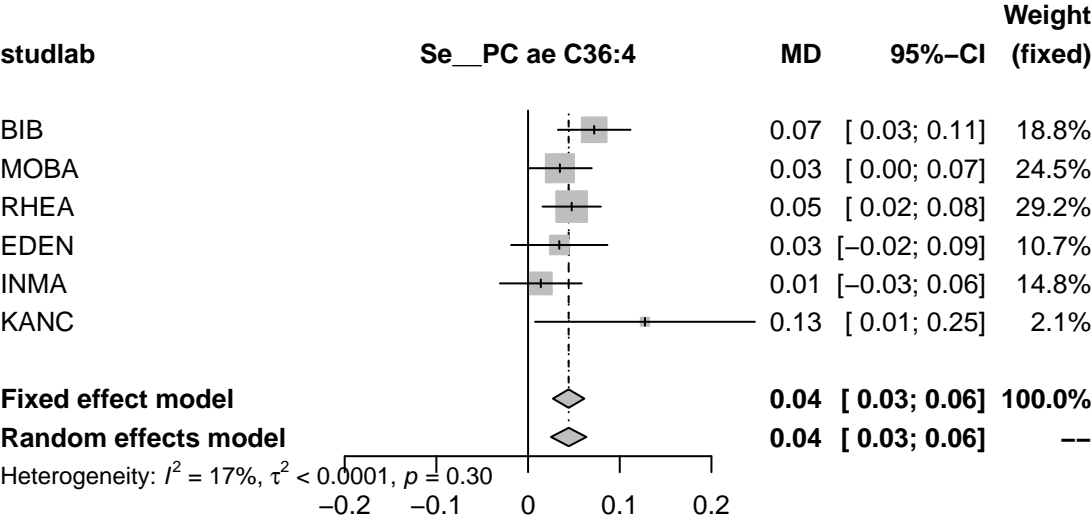

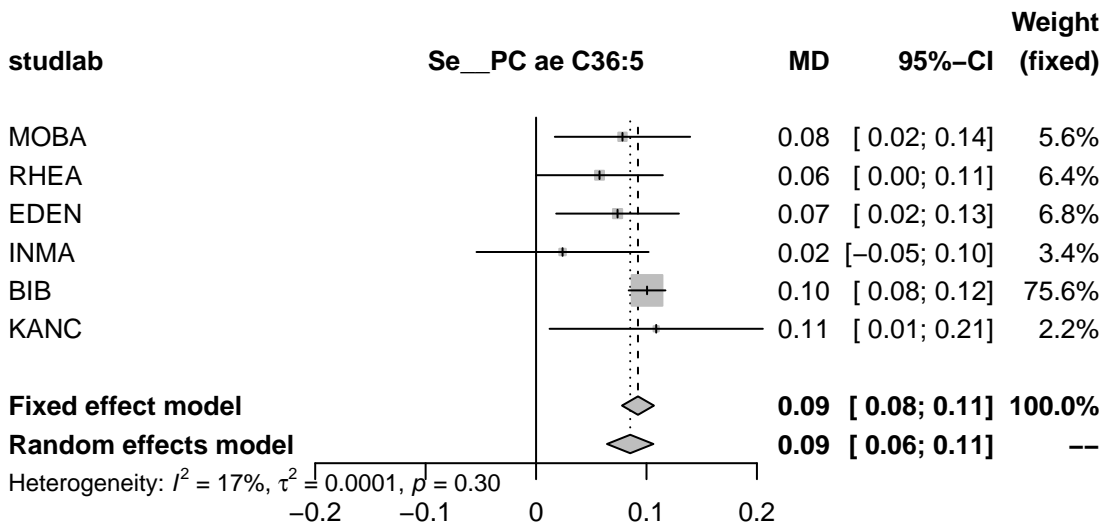

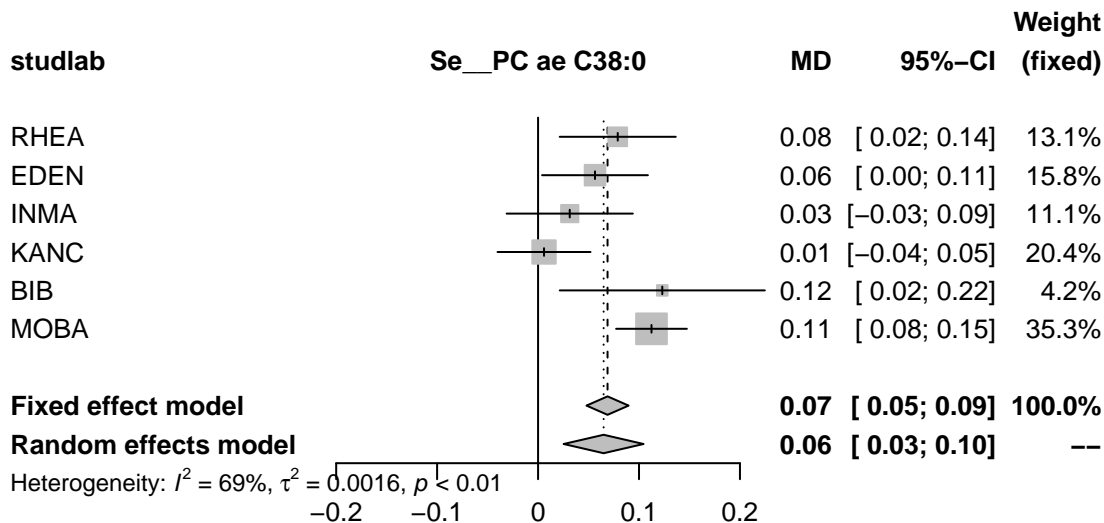

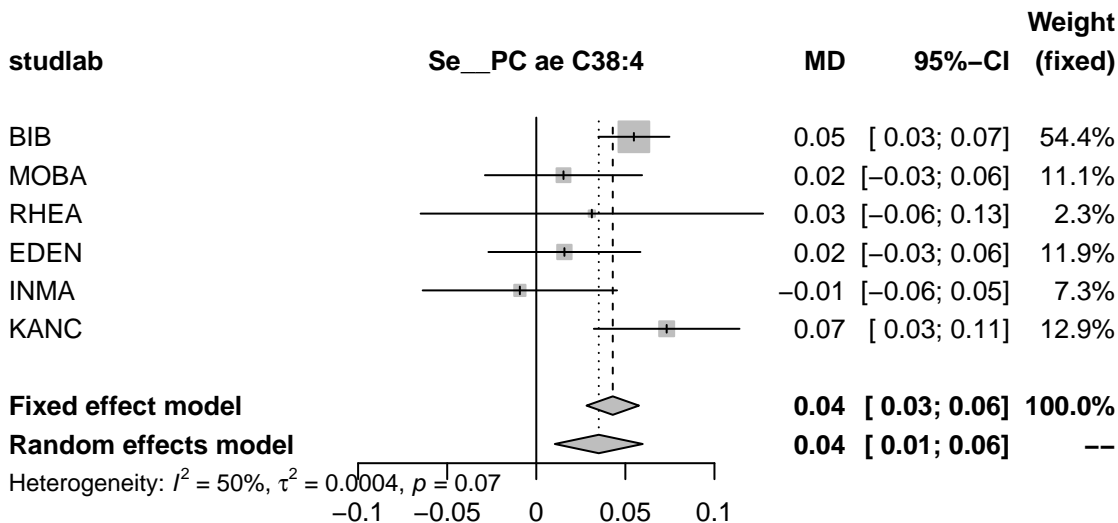

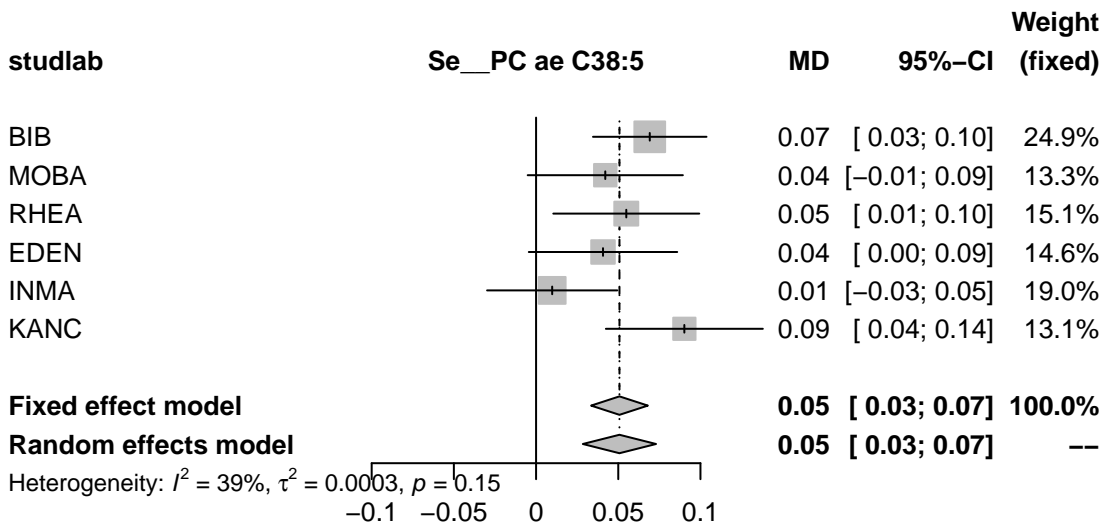

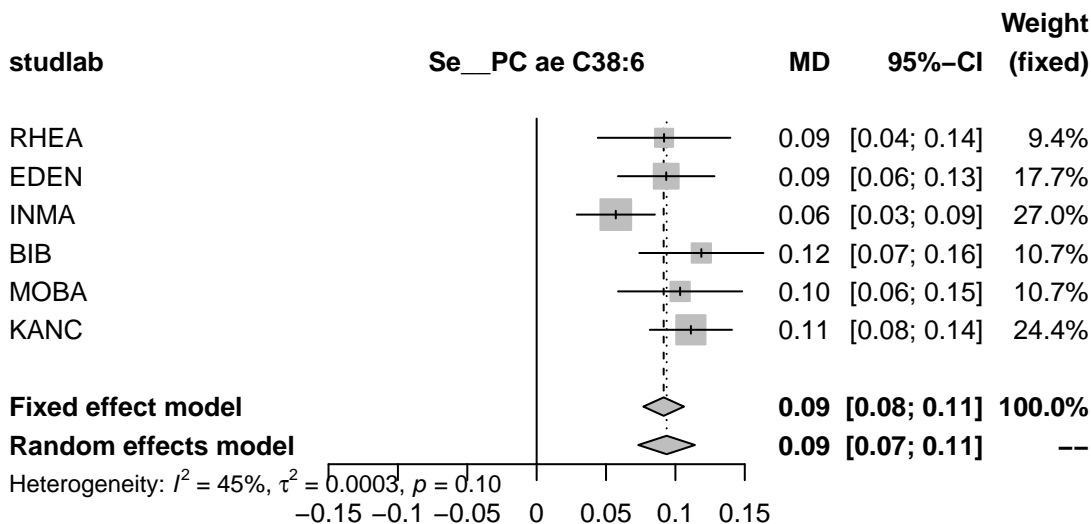

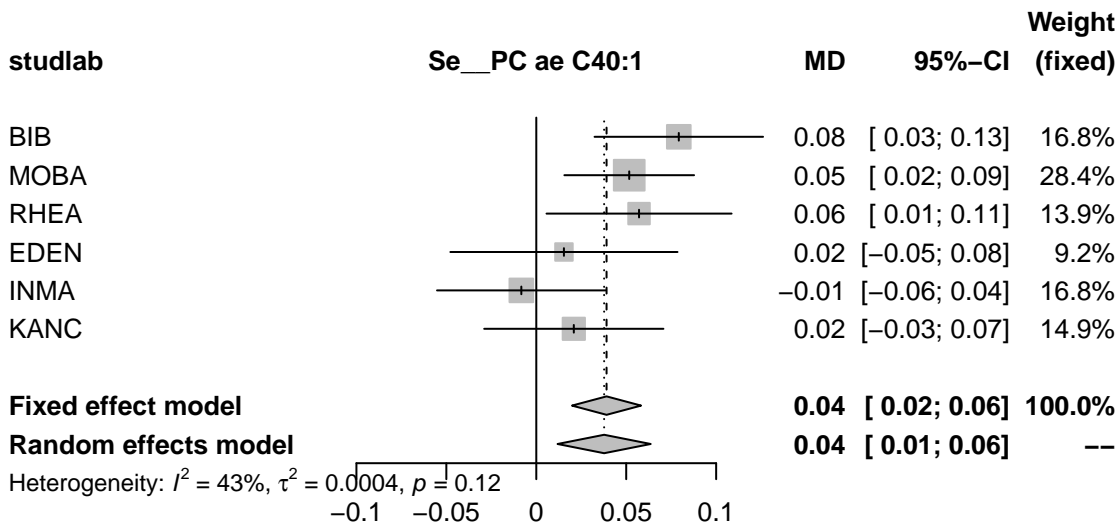

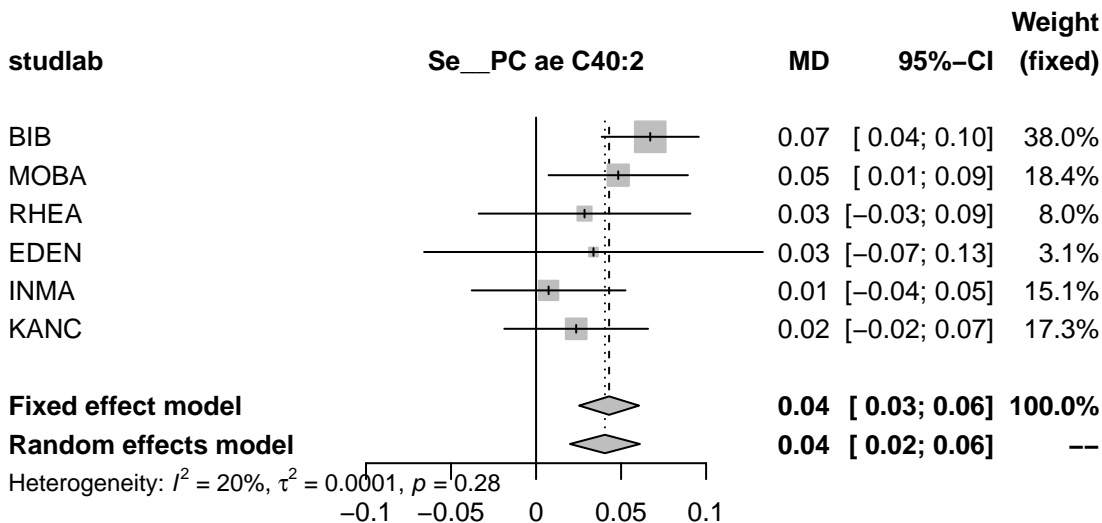

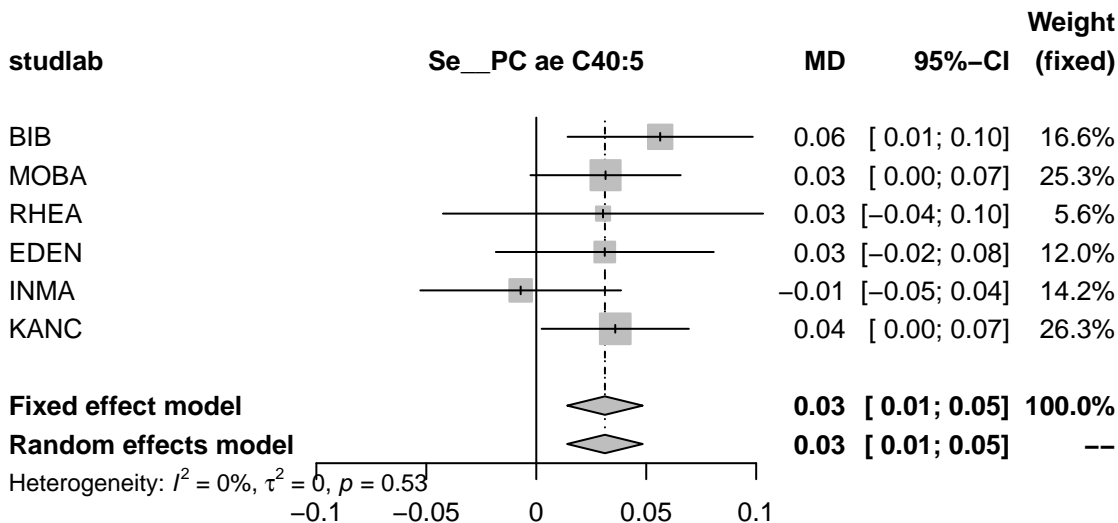

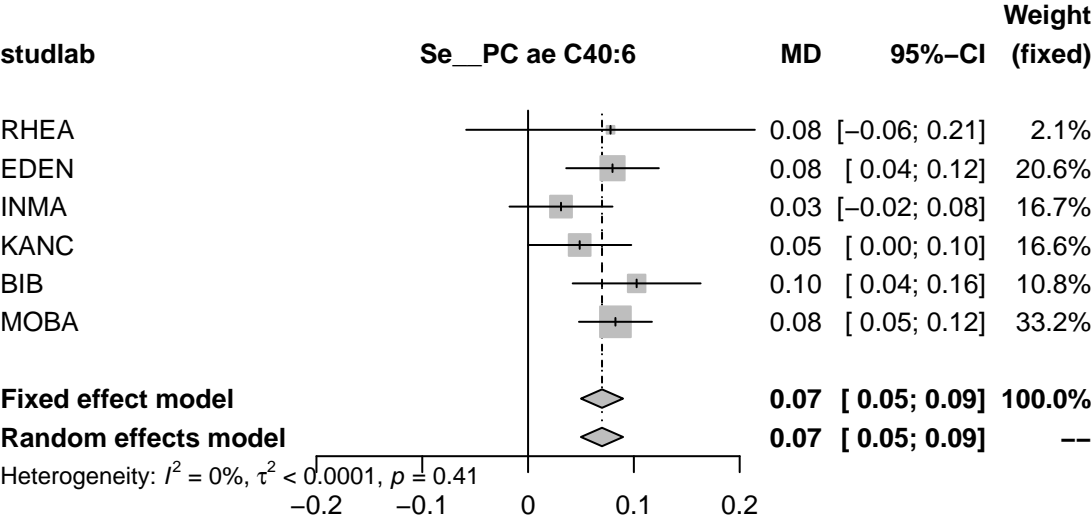

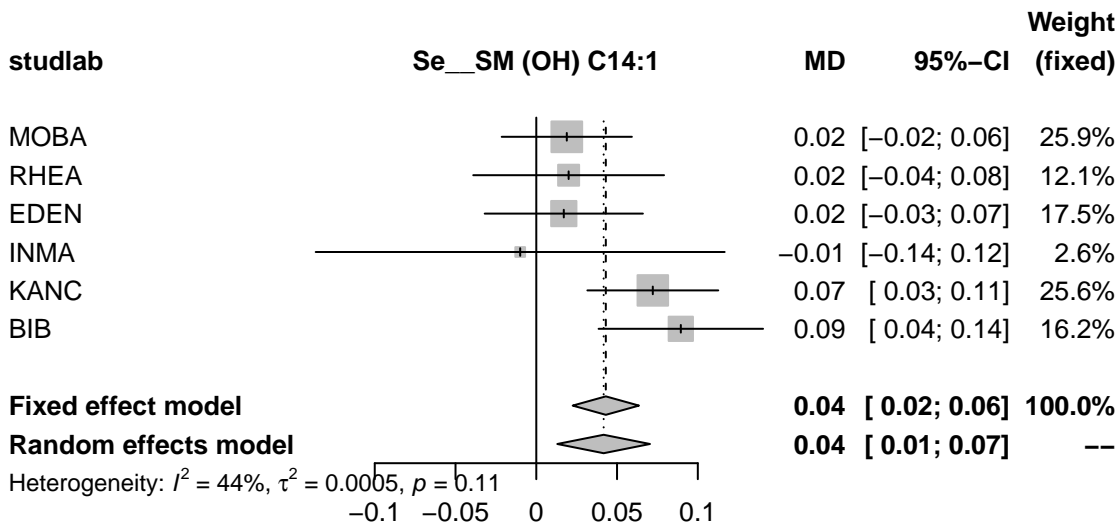

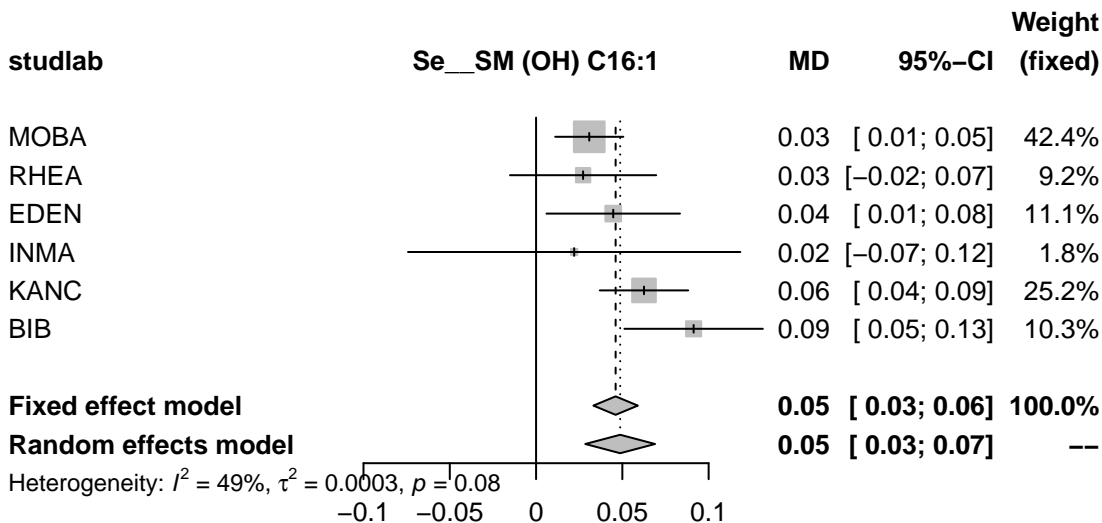

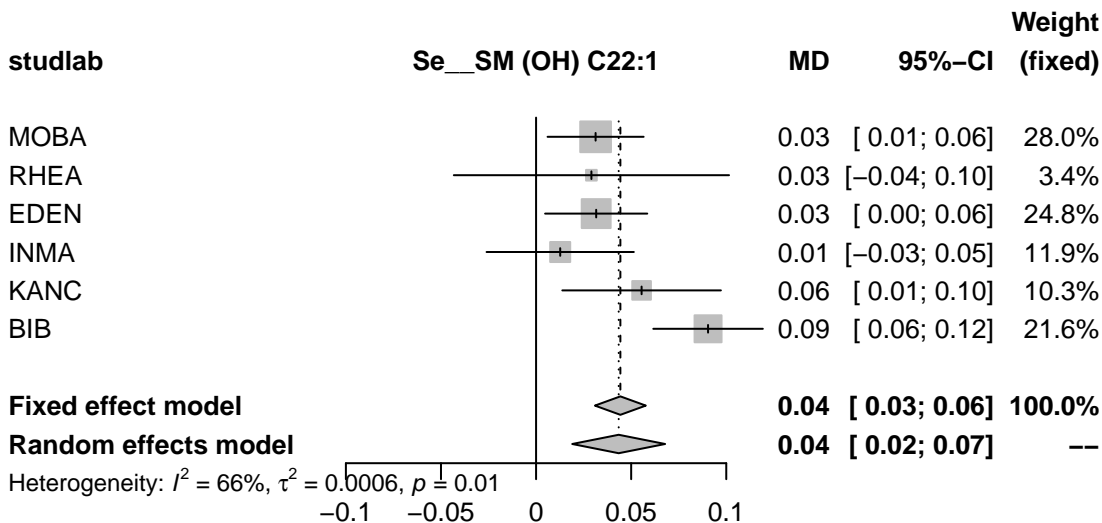

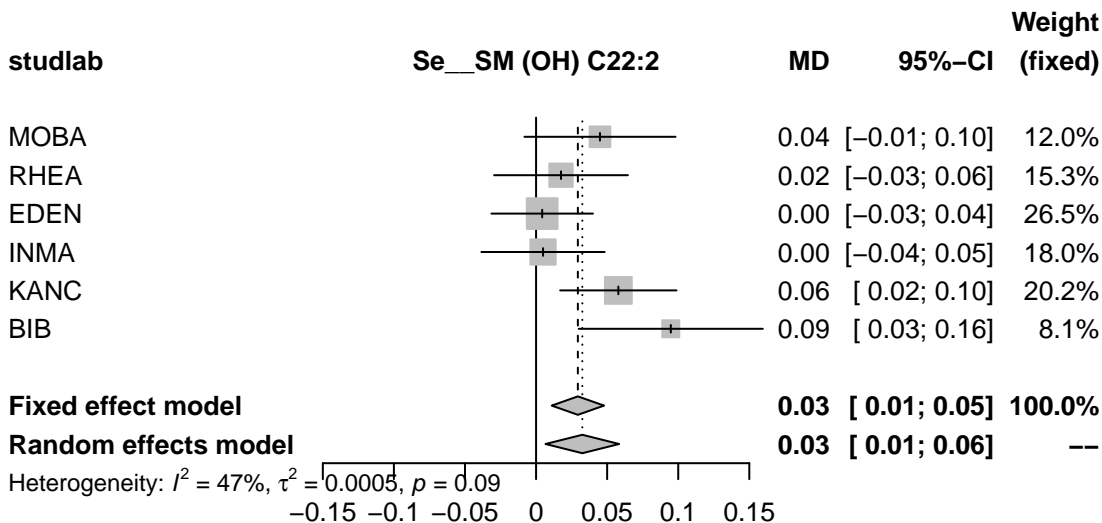

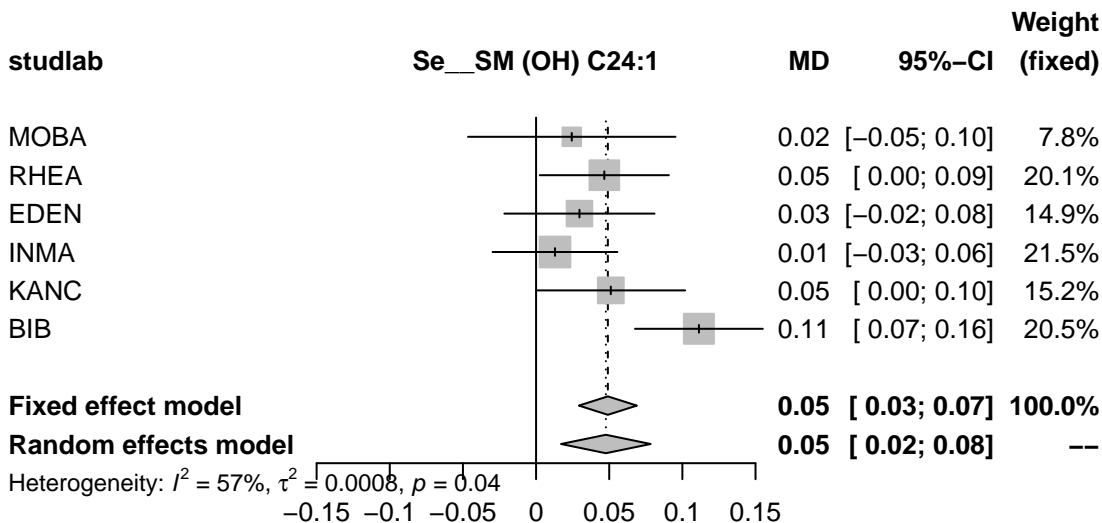

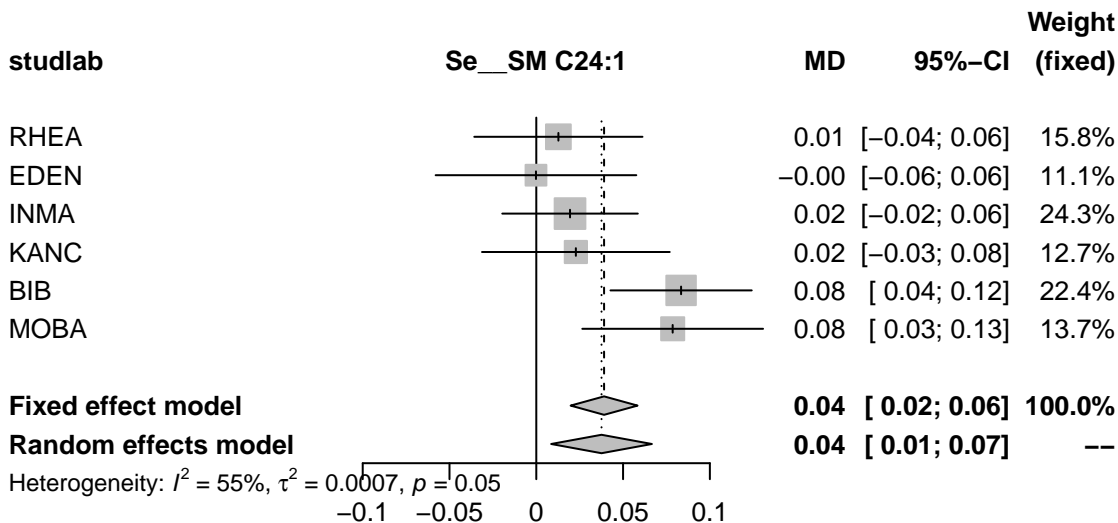

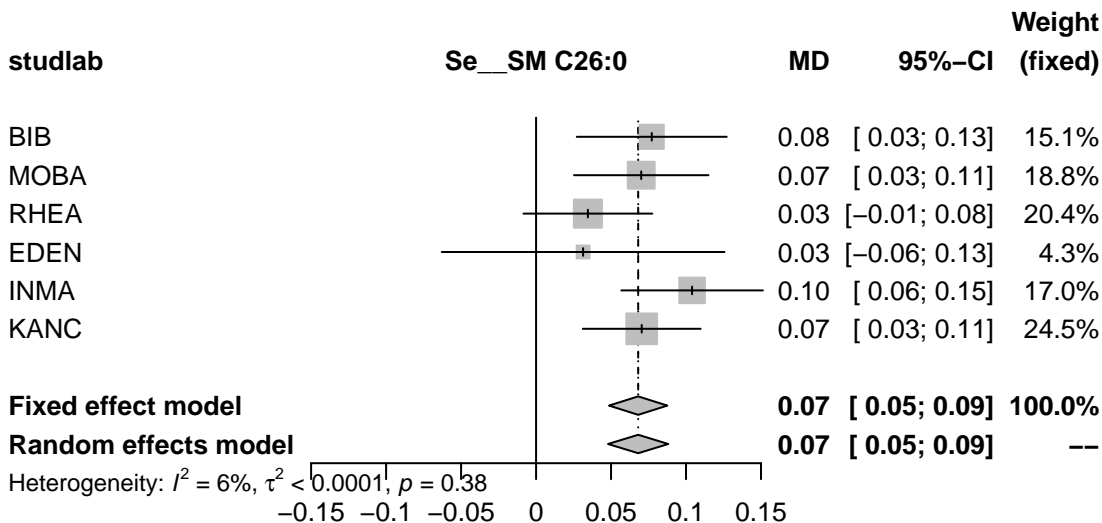

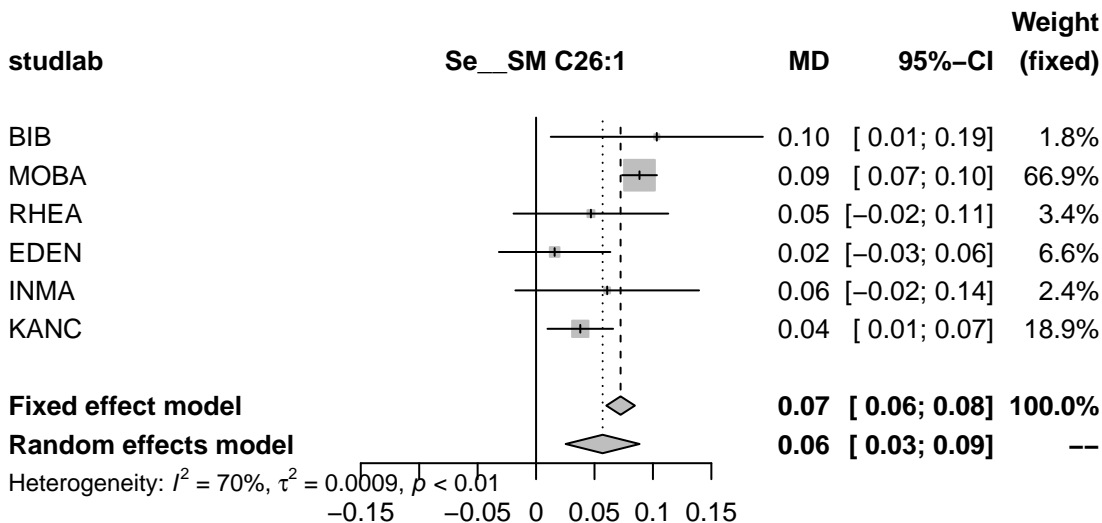

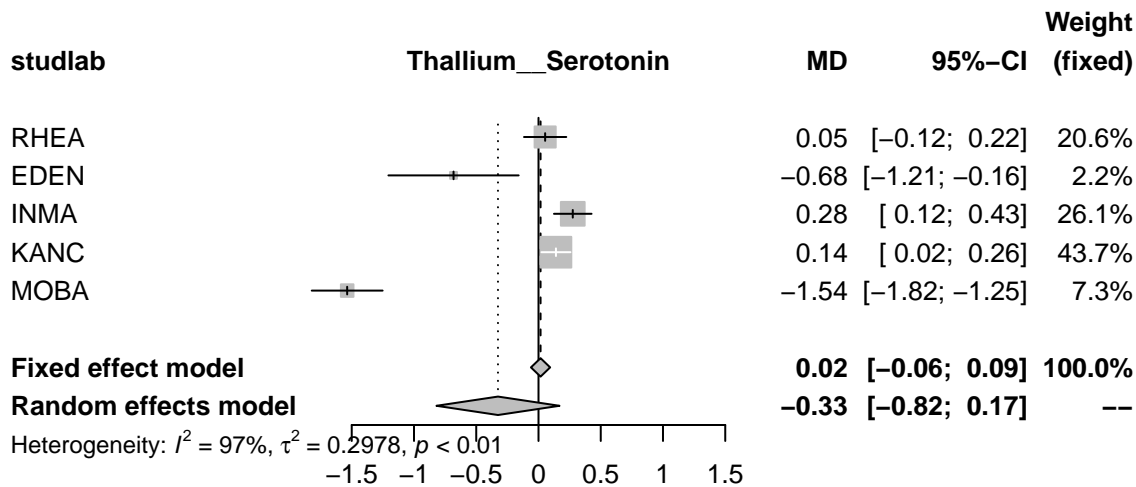

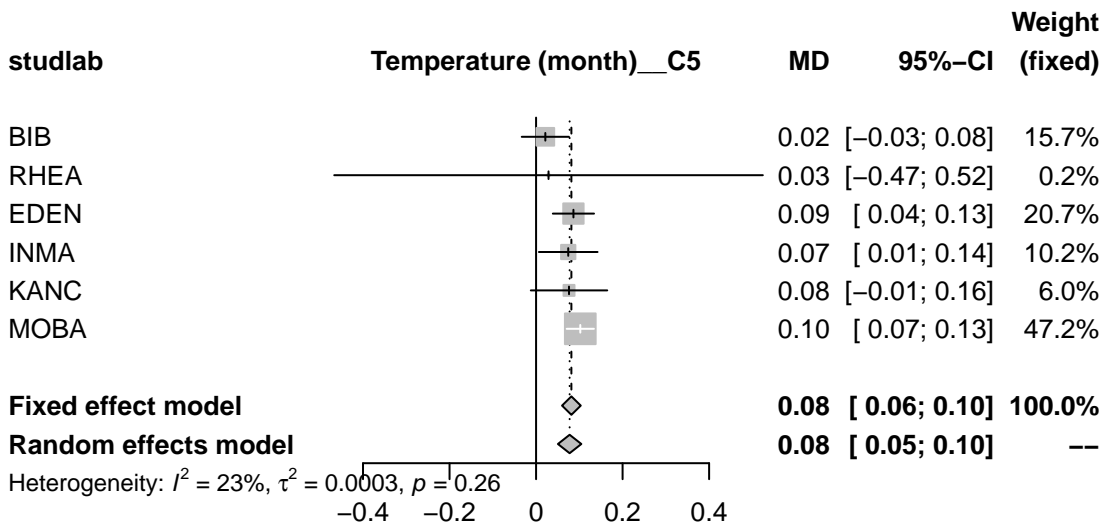

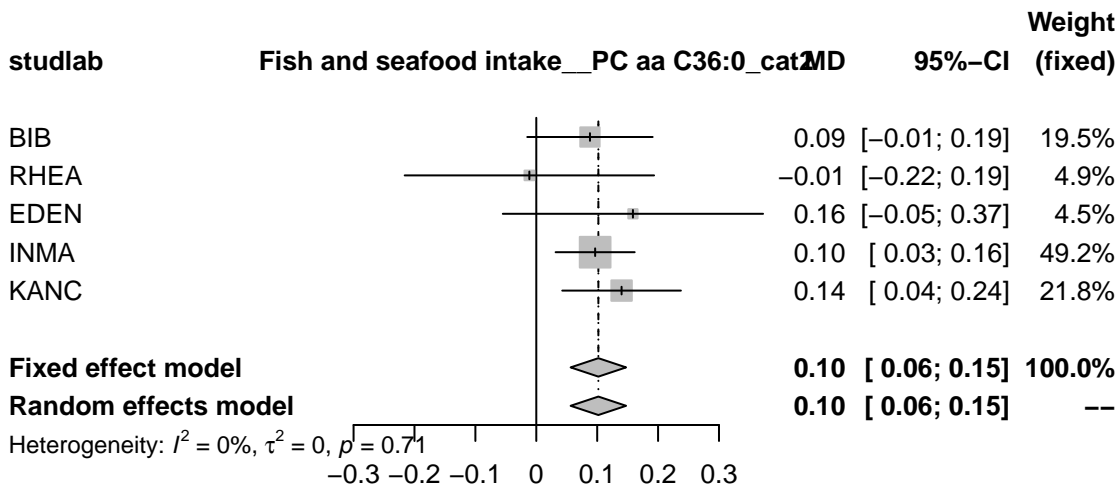

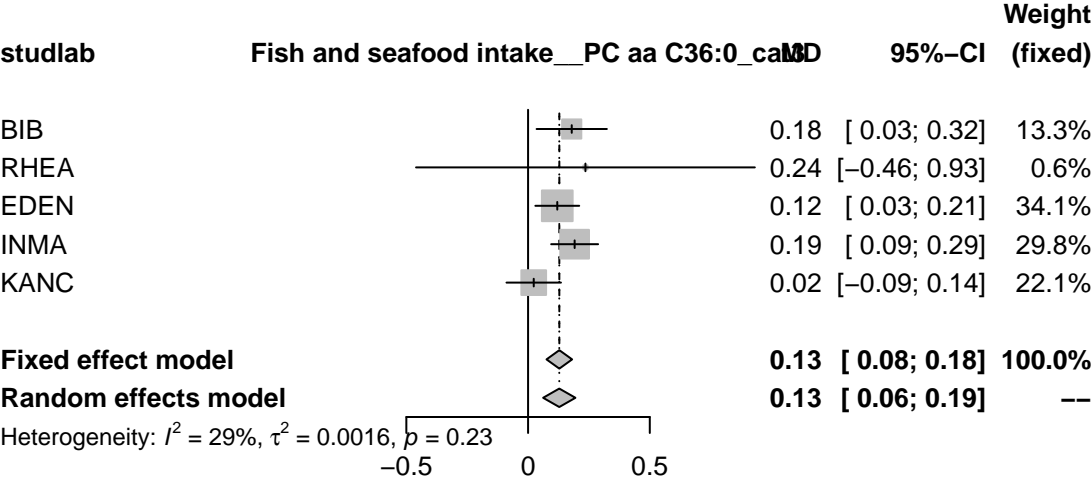

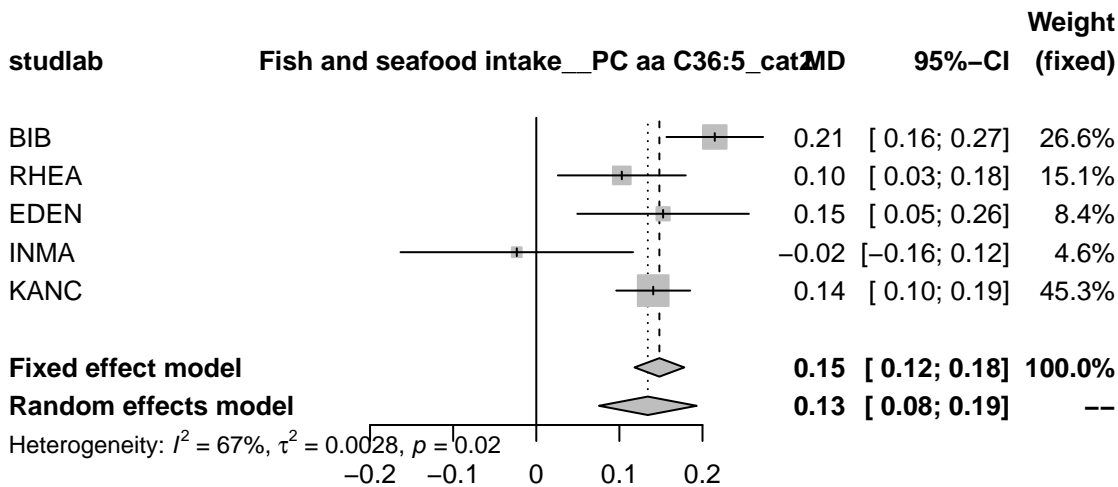

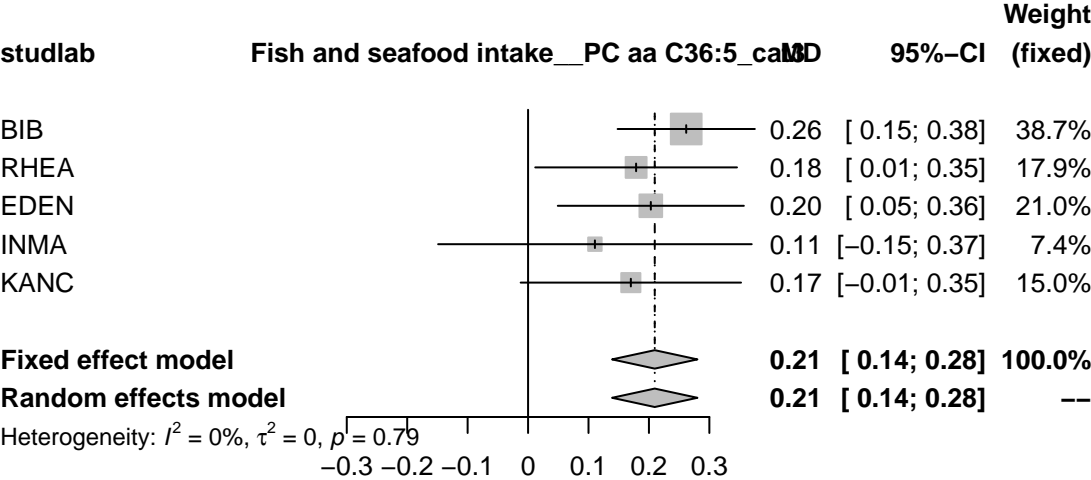

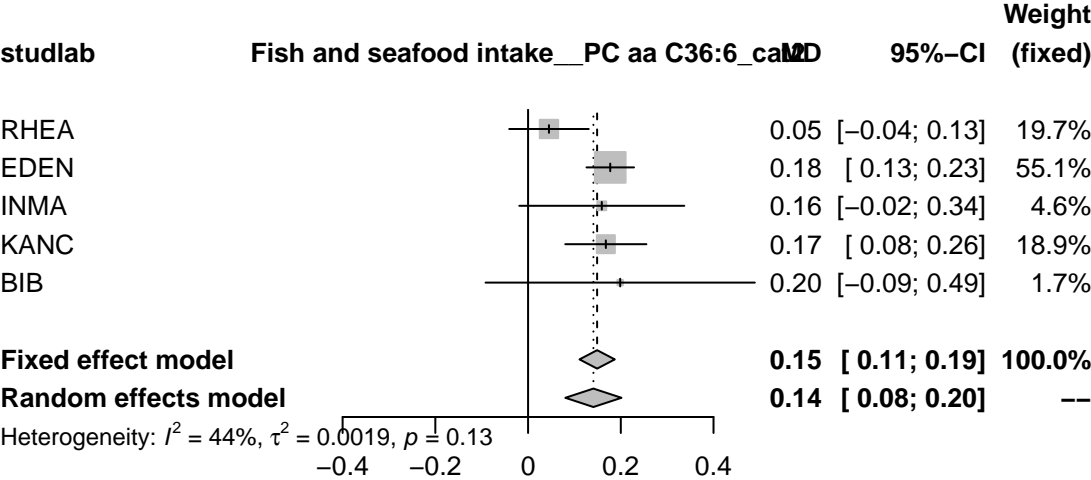

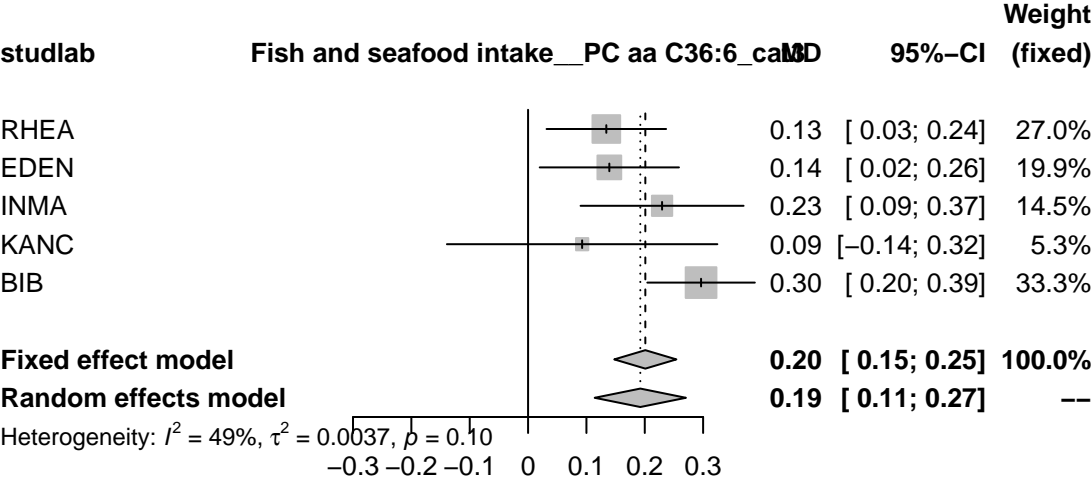

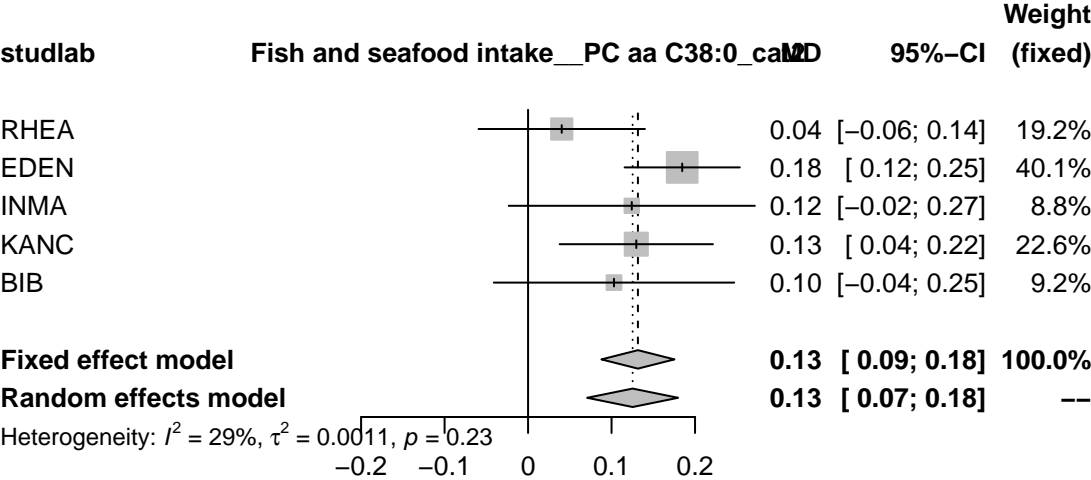

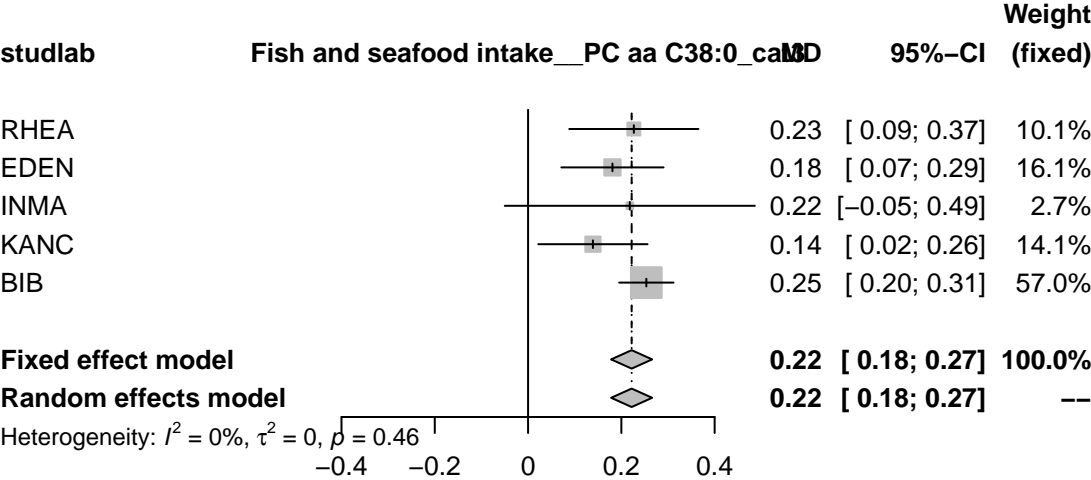

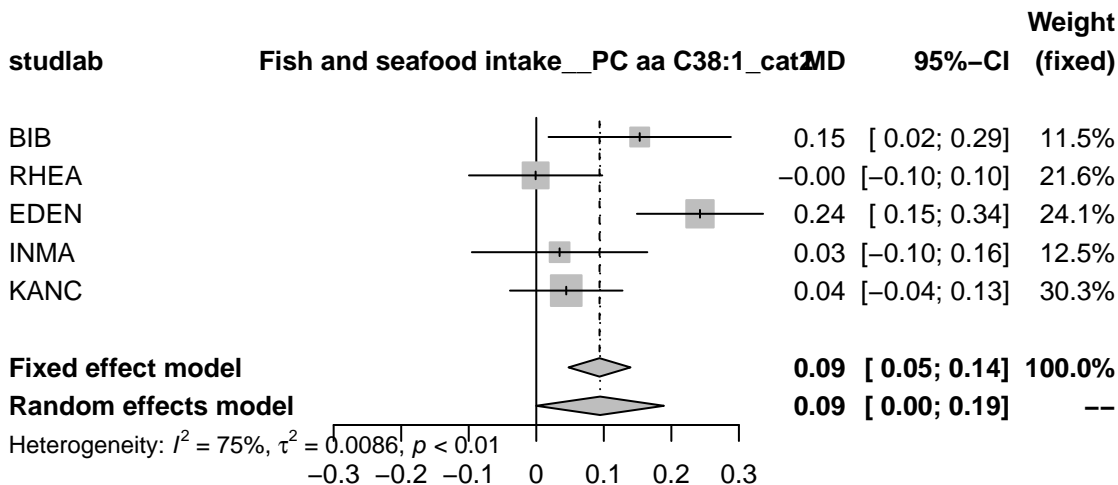

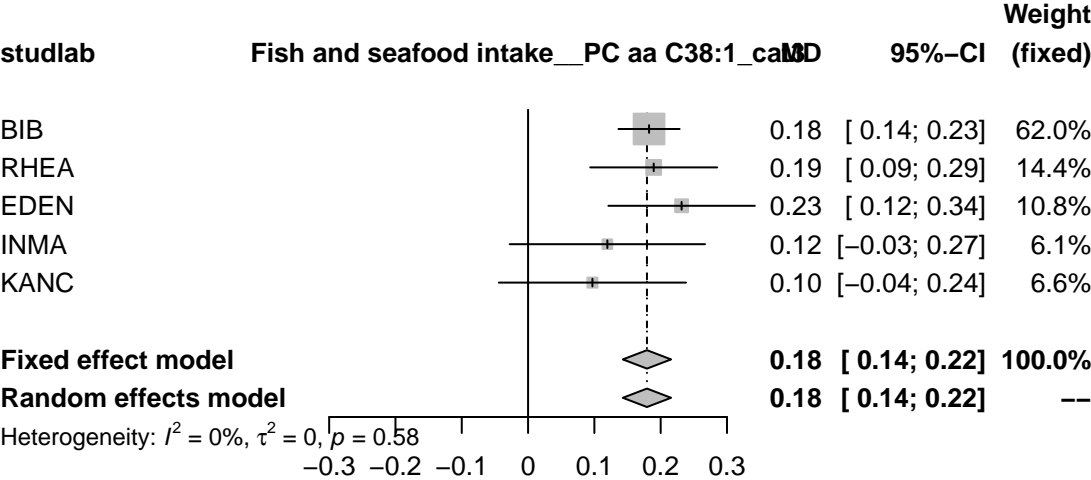

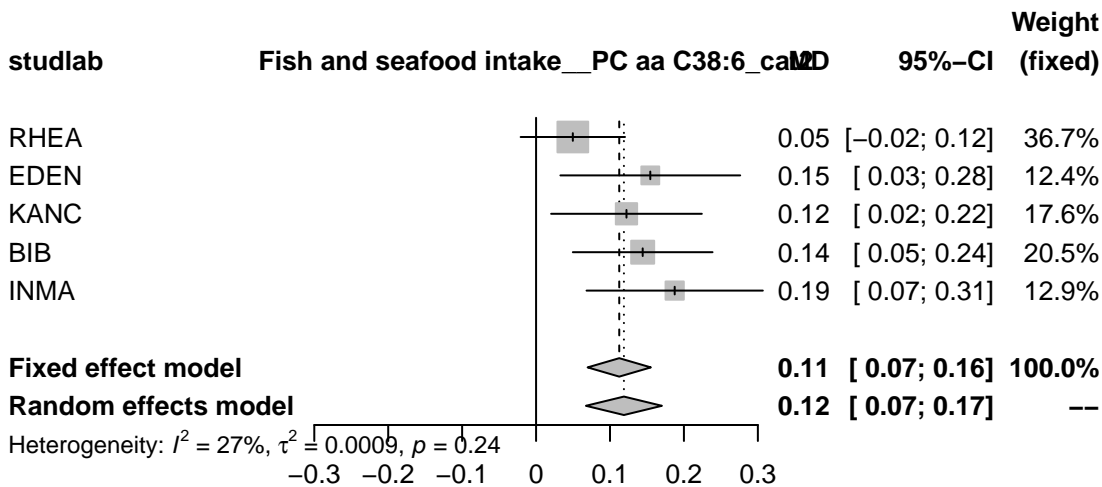

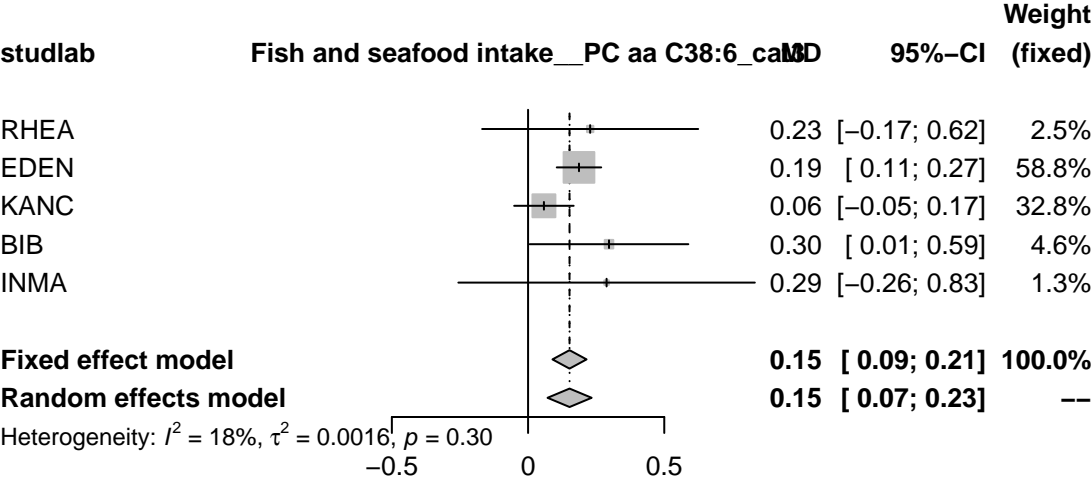

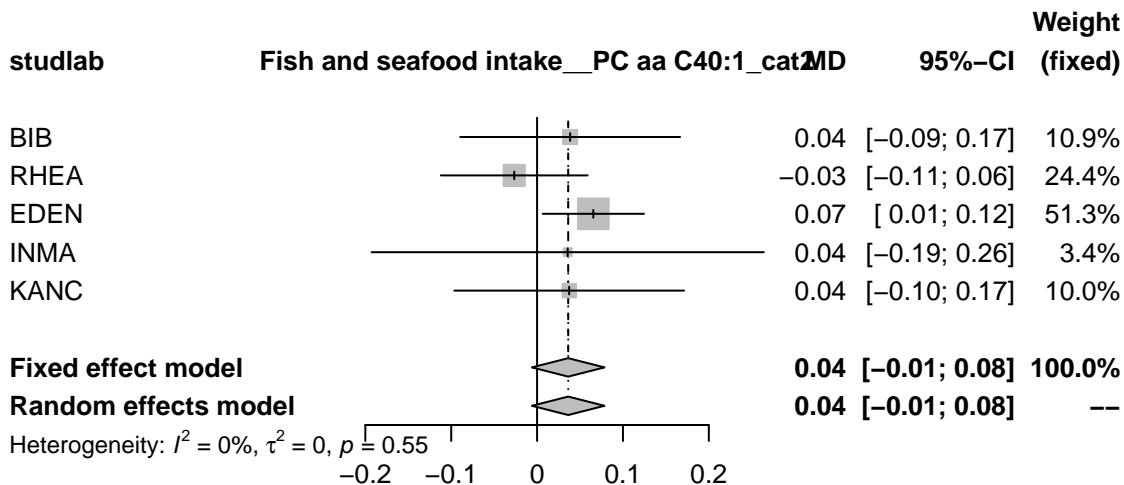

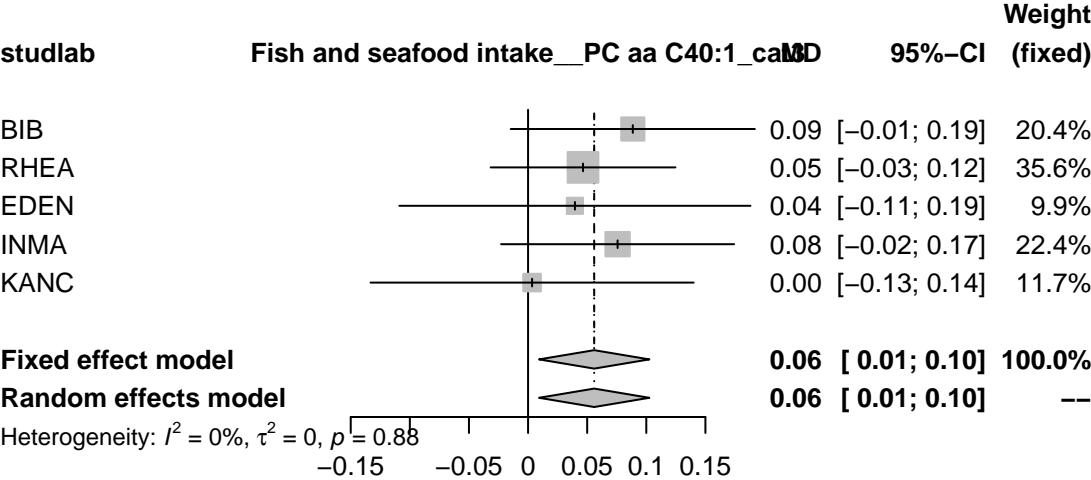

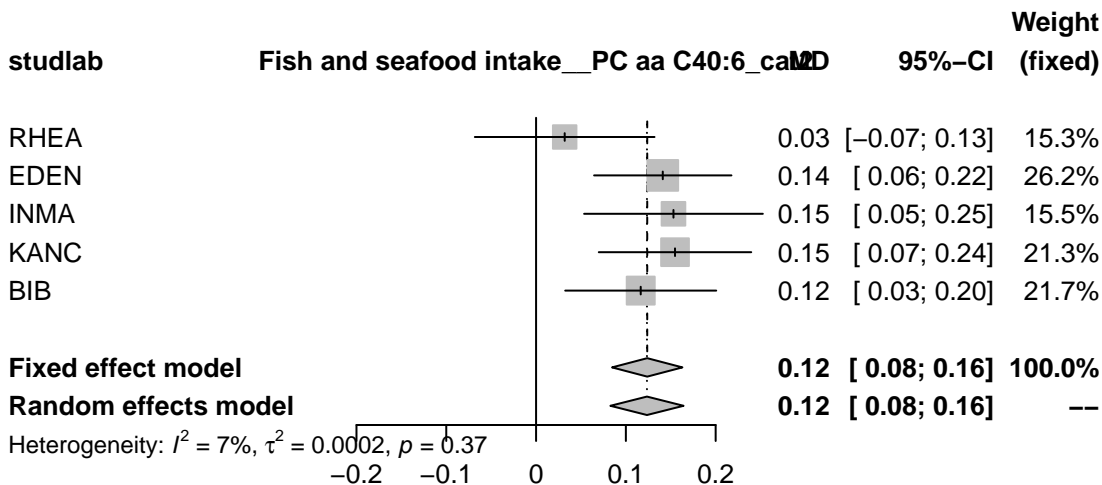

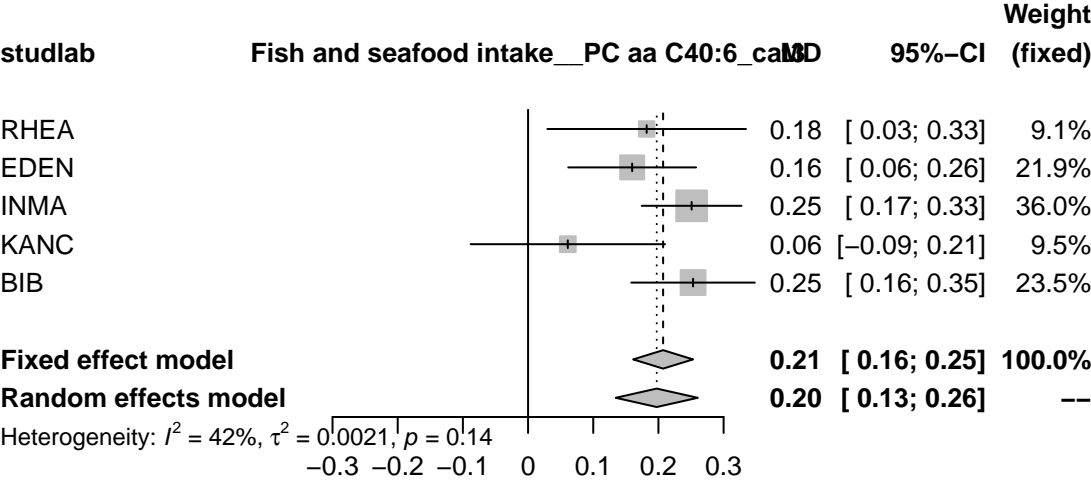

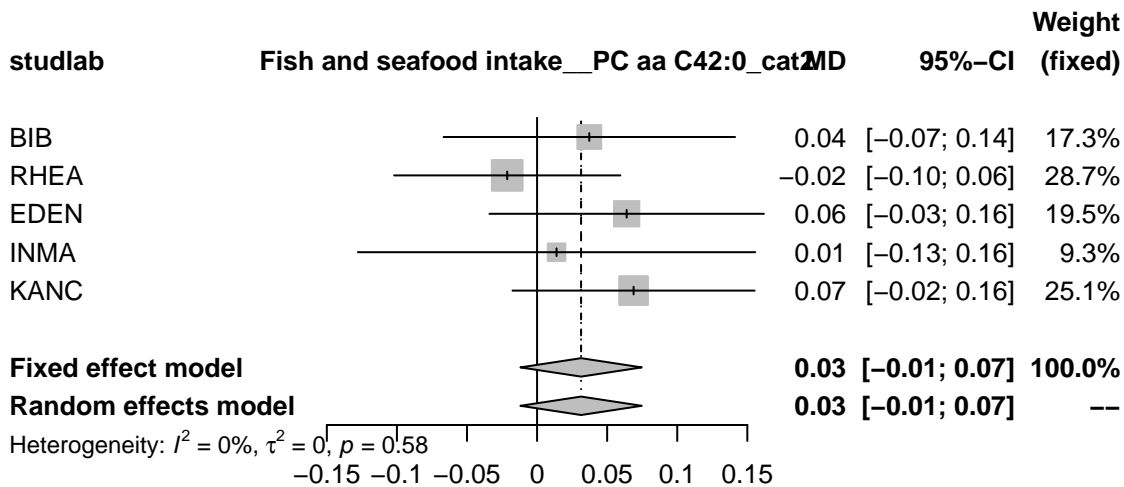

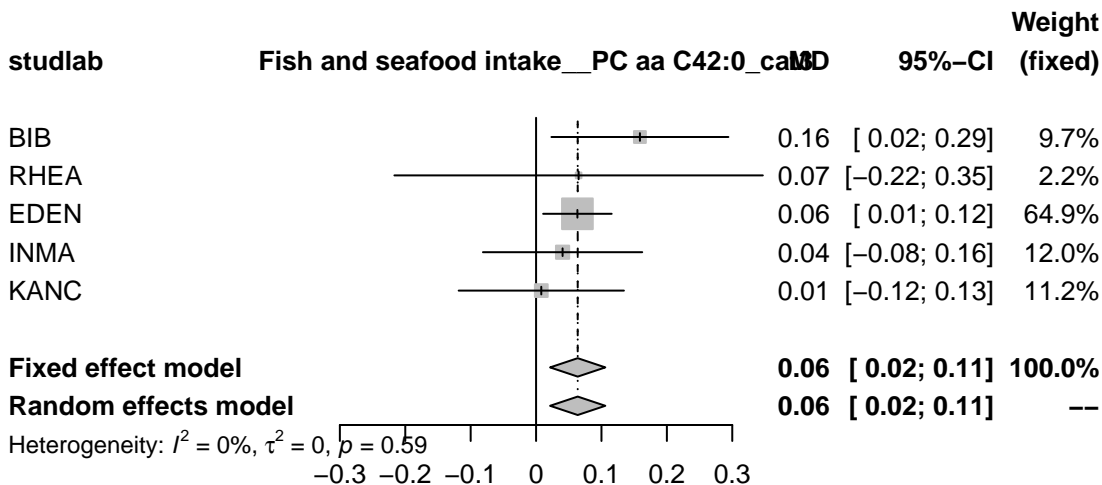

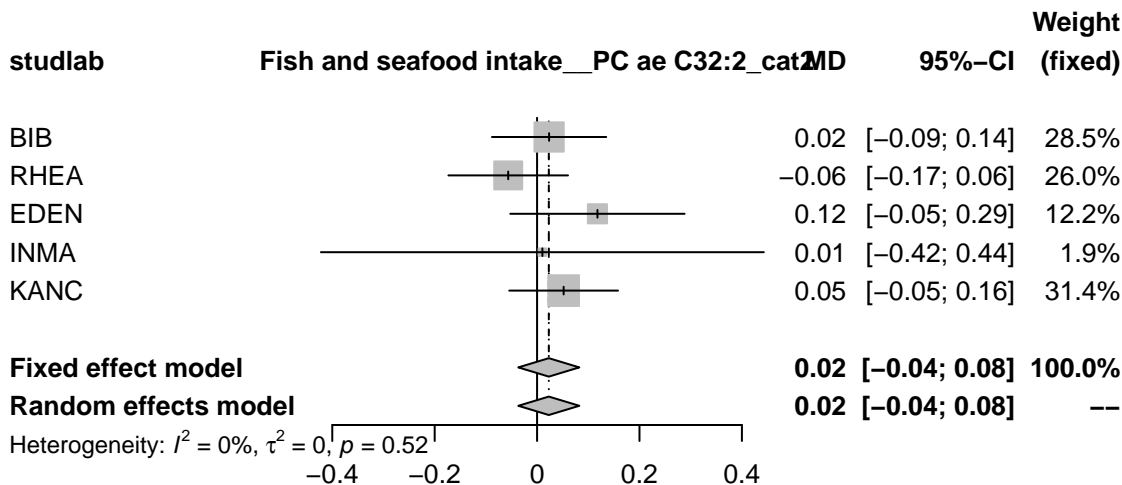

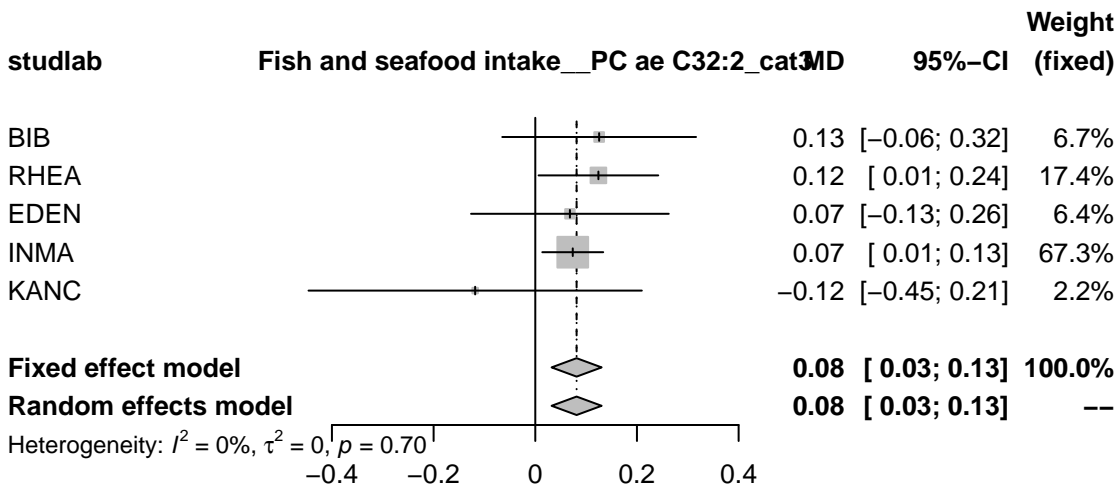

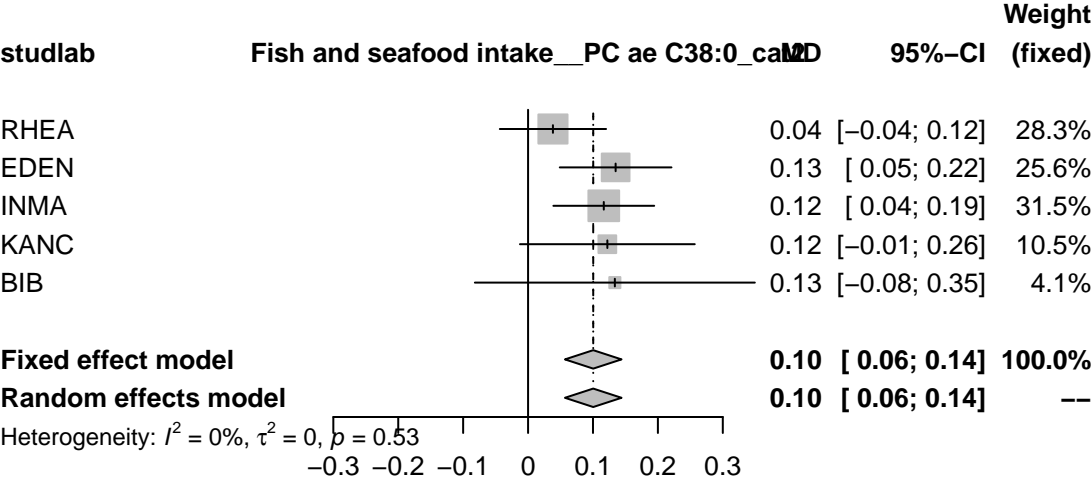

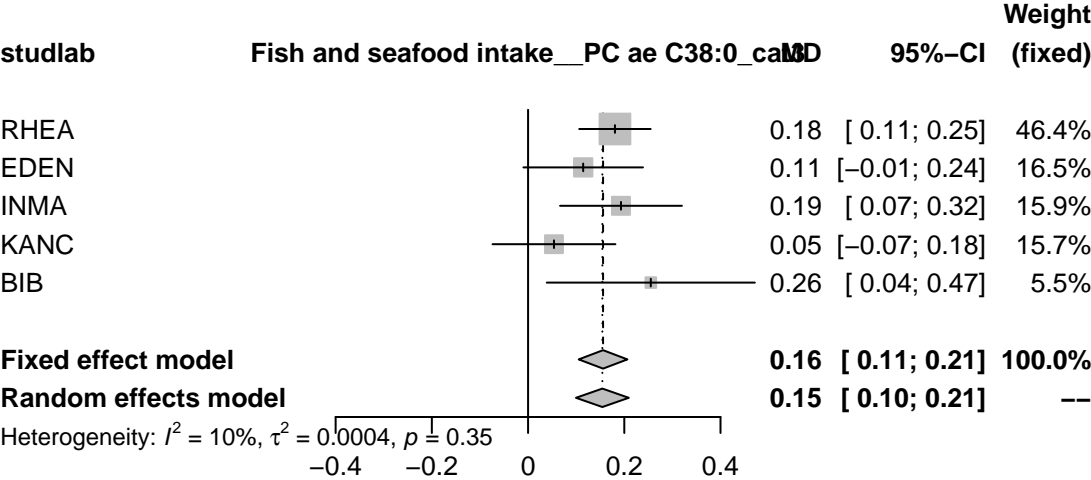

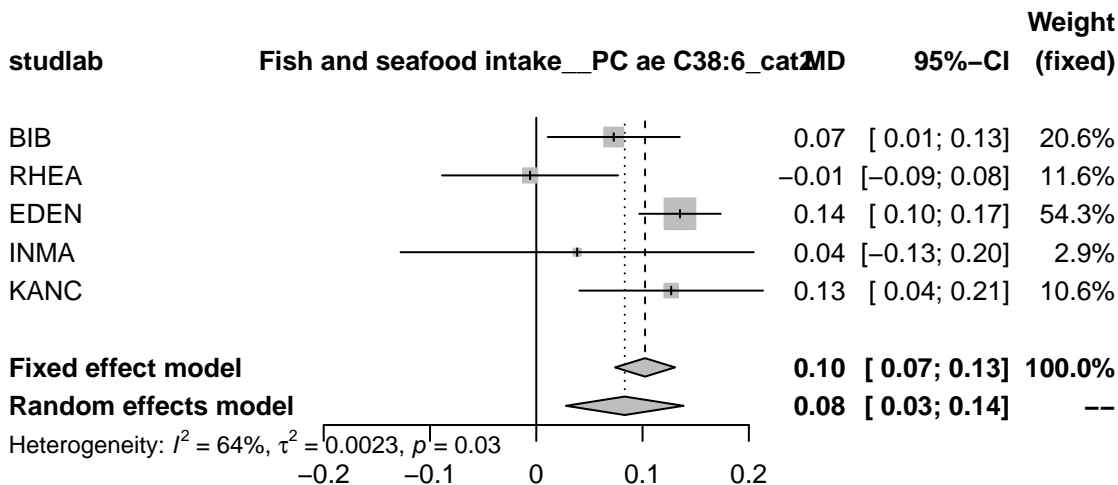

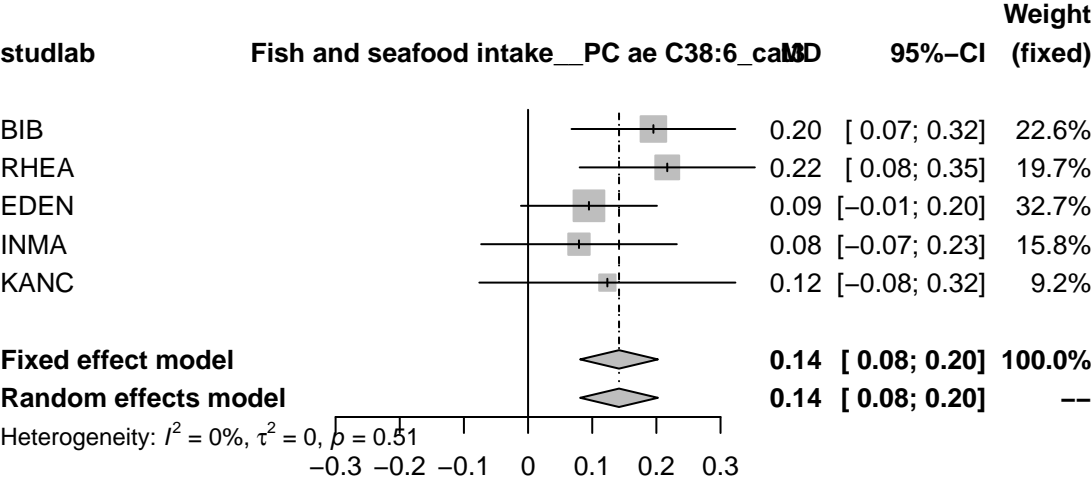

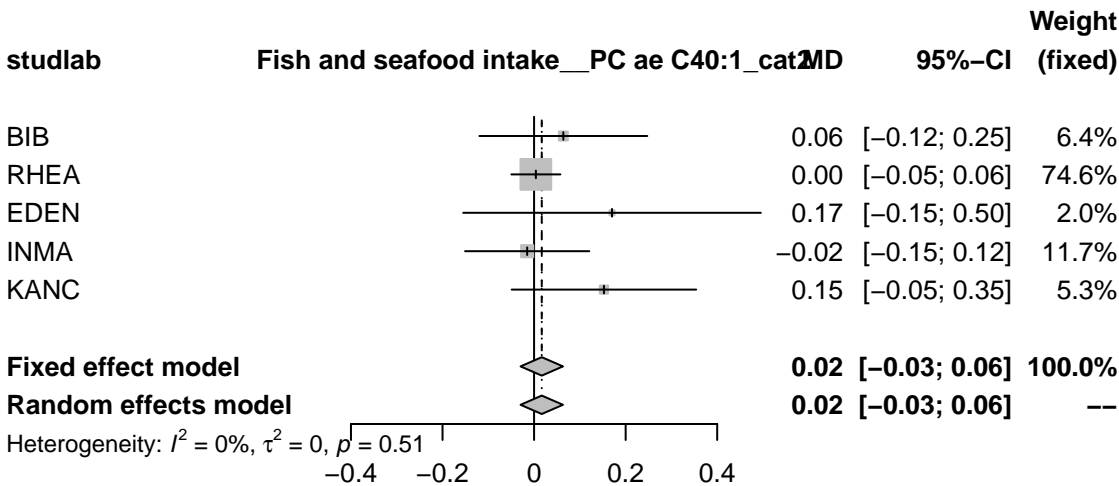

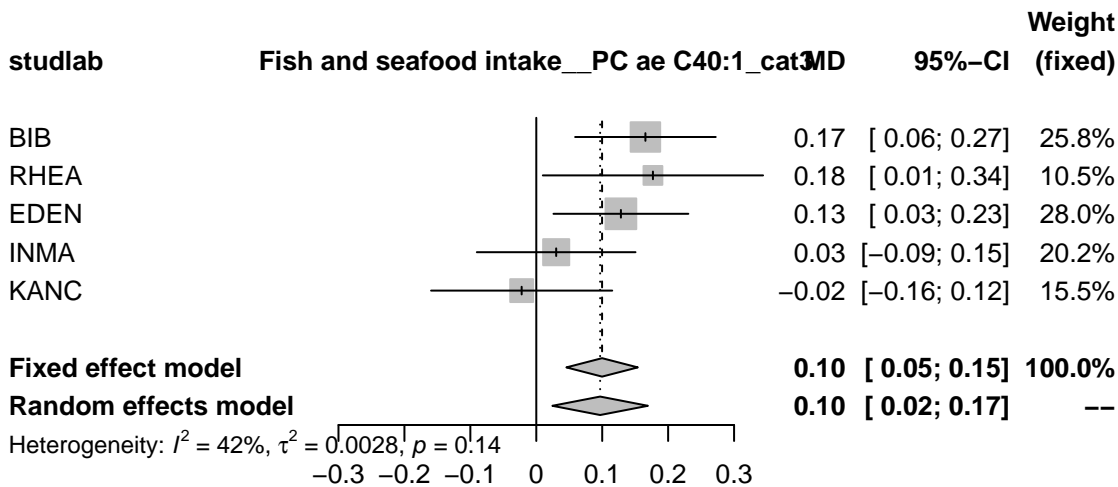

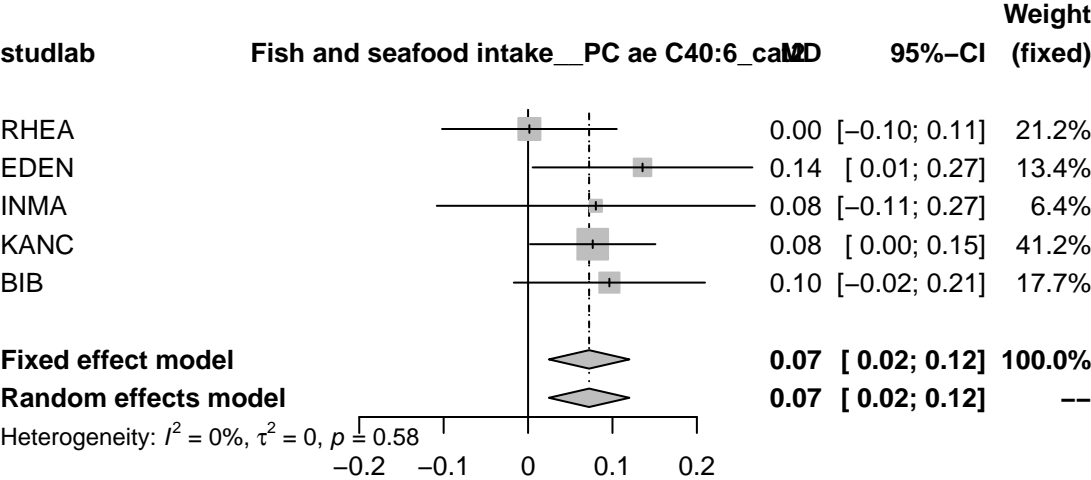

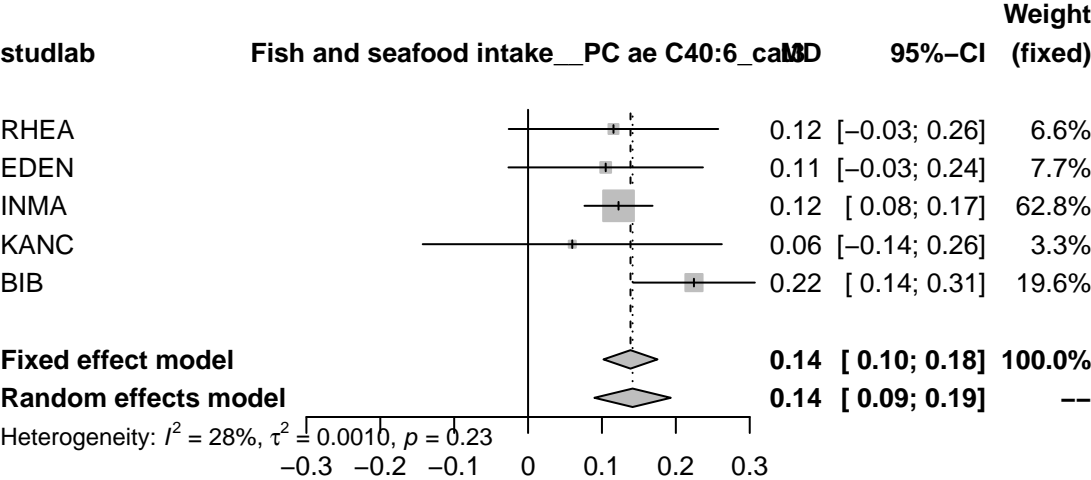

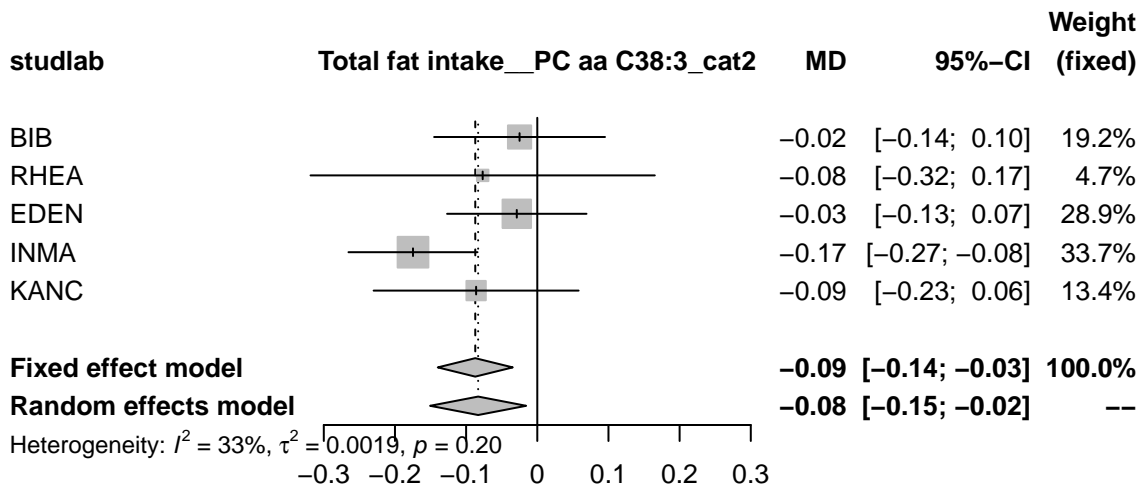

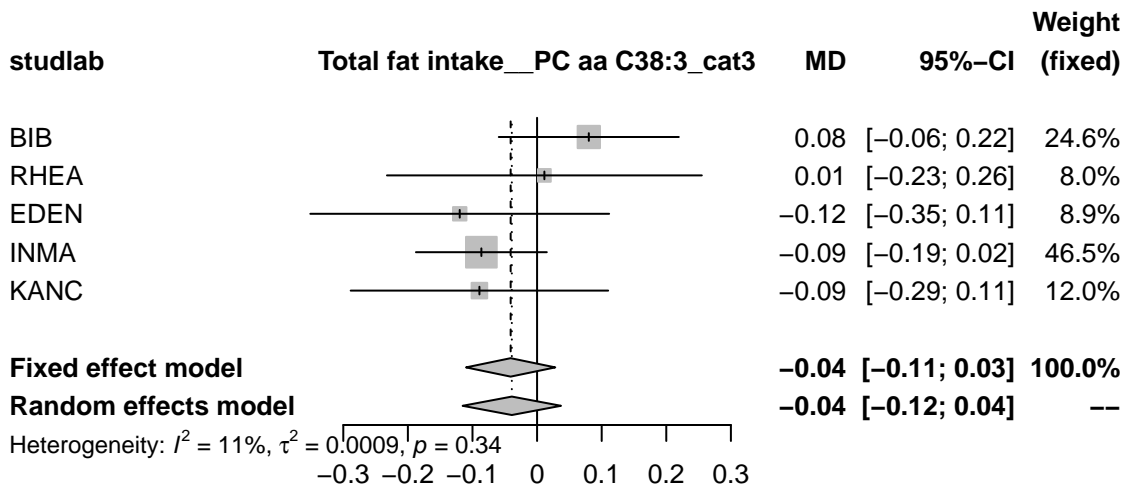

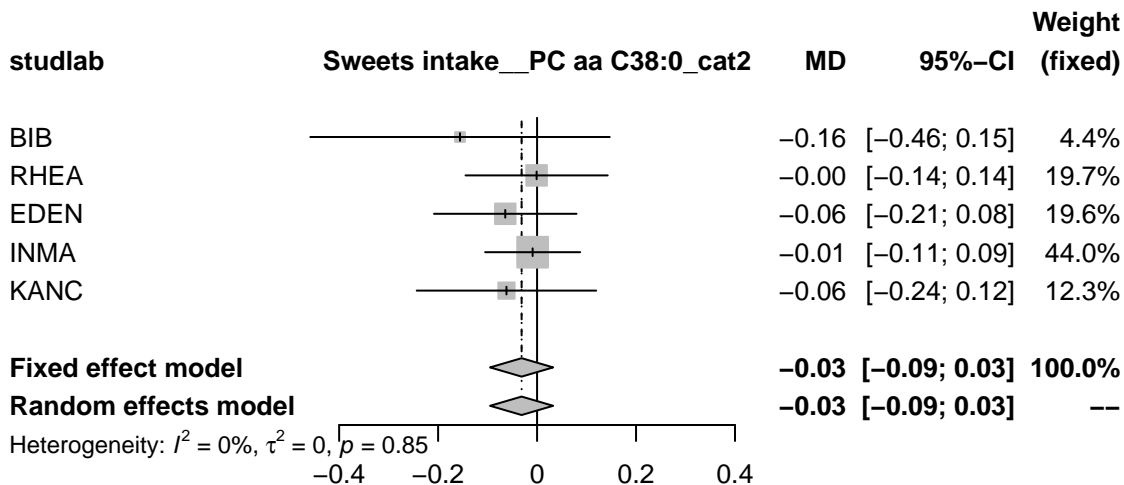

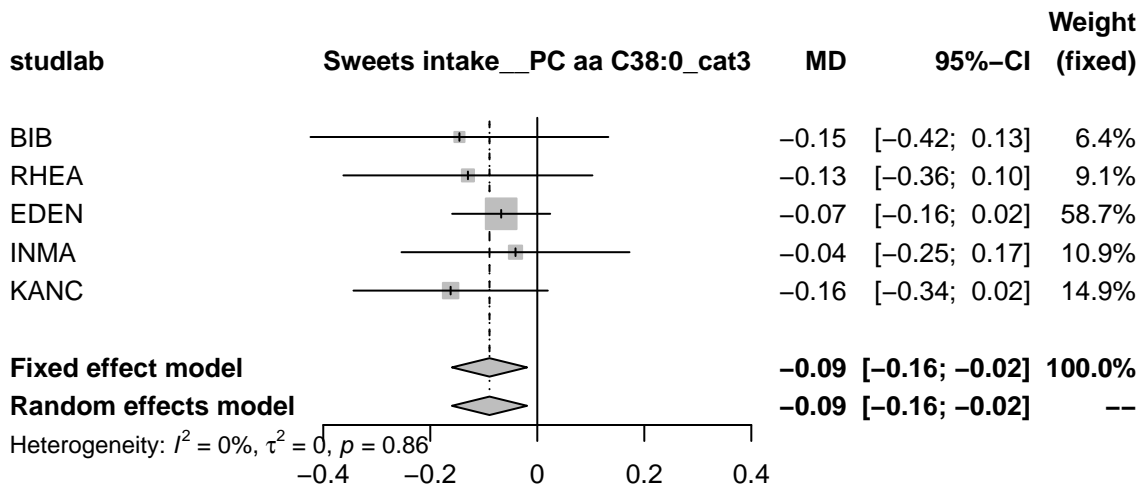

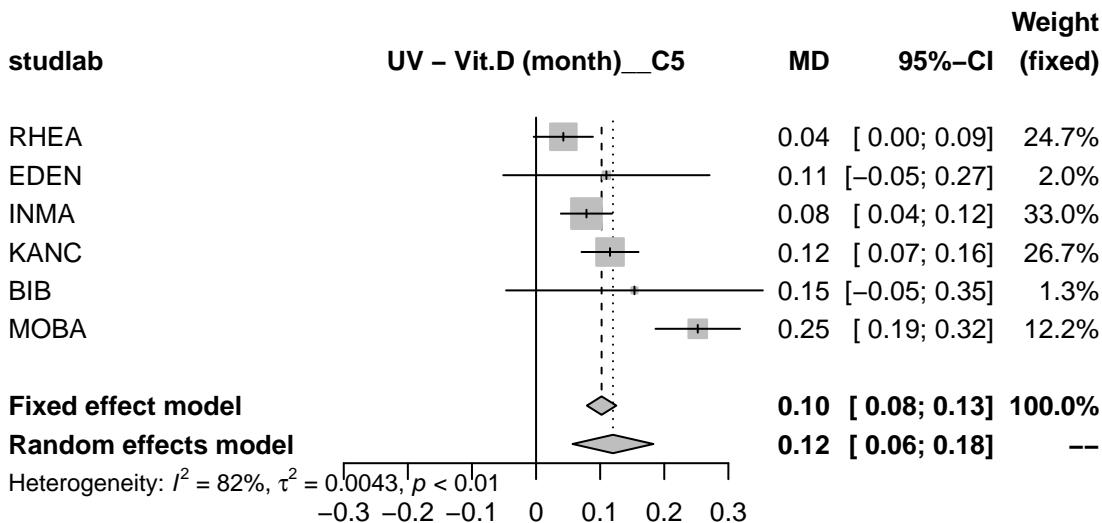

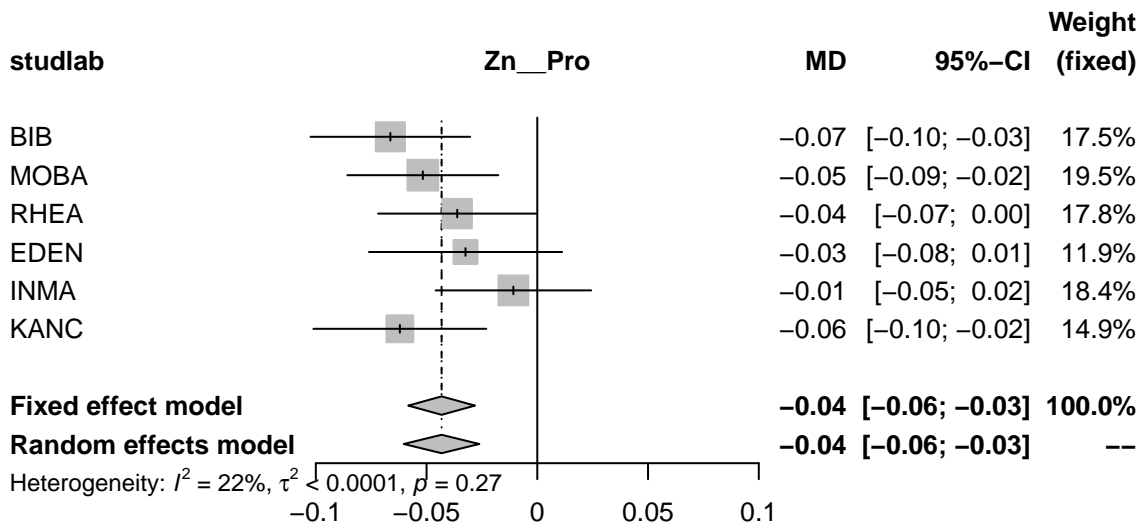

Supplement: Supplementary file 14 — Supplementary Dataset 11 [file 41467_2022_34422_MOESM14_ESM.zip › HELIX_ExpOmics_FigS2_Forestplots/HELIX_ExpOmics_FigS2K_met_s_post.pdf]

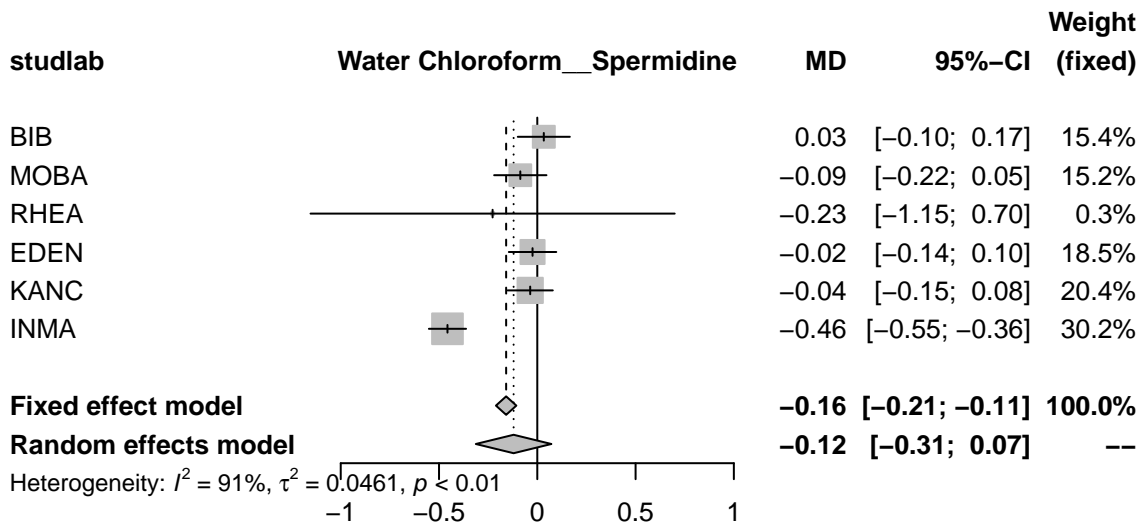

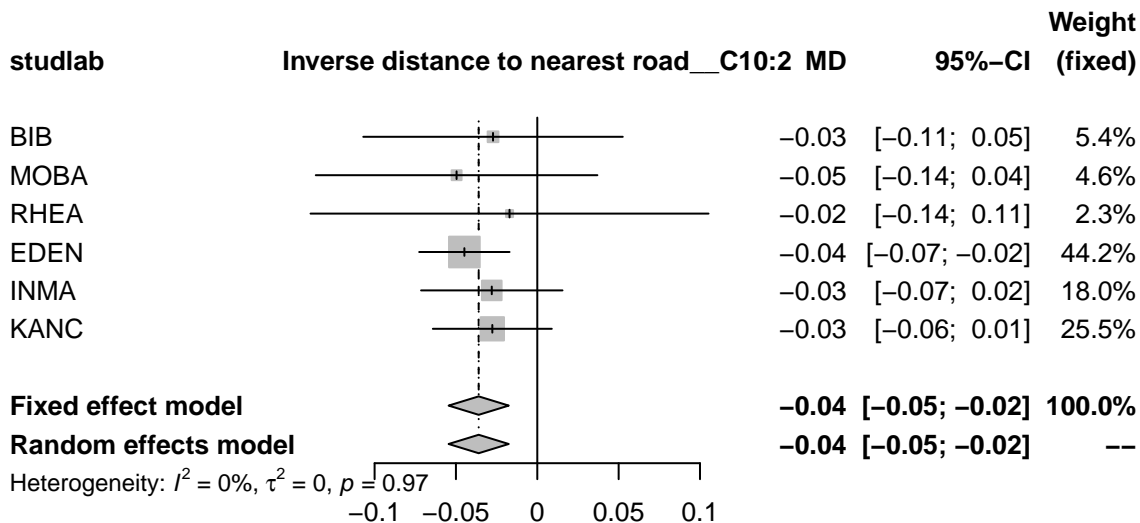

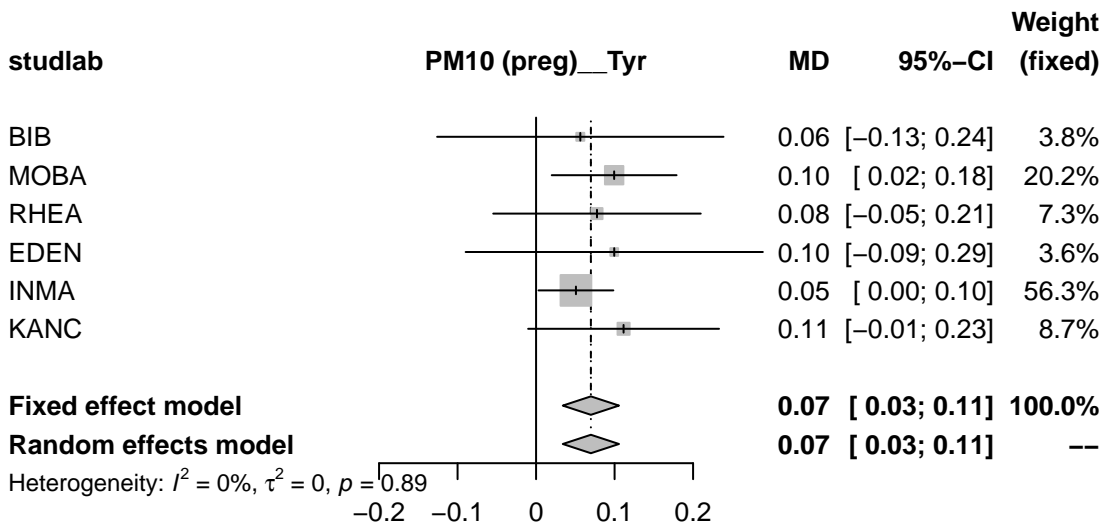

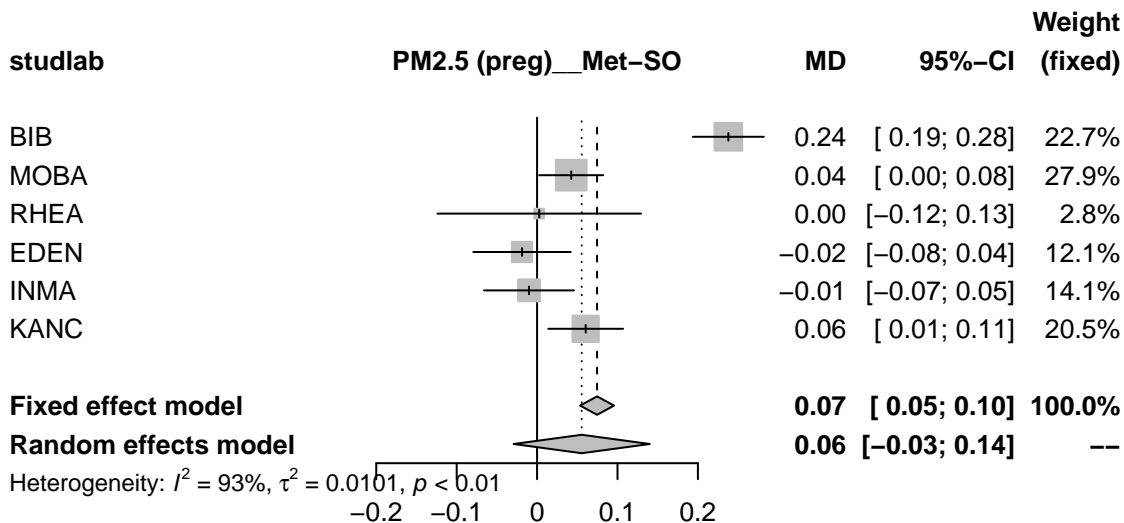

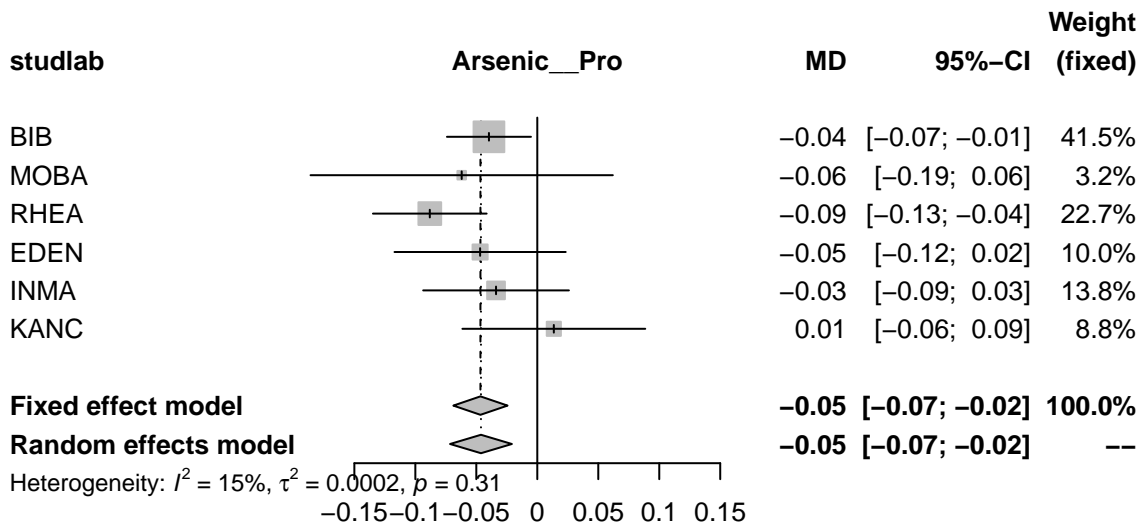

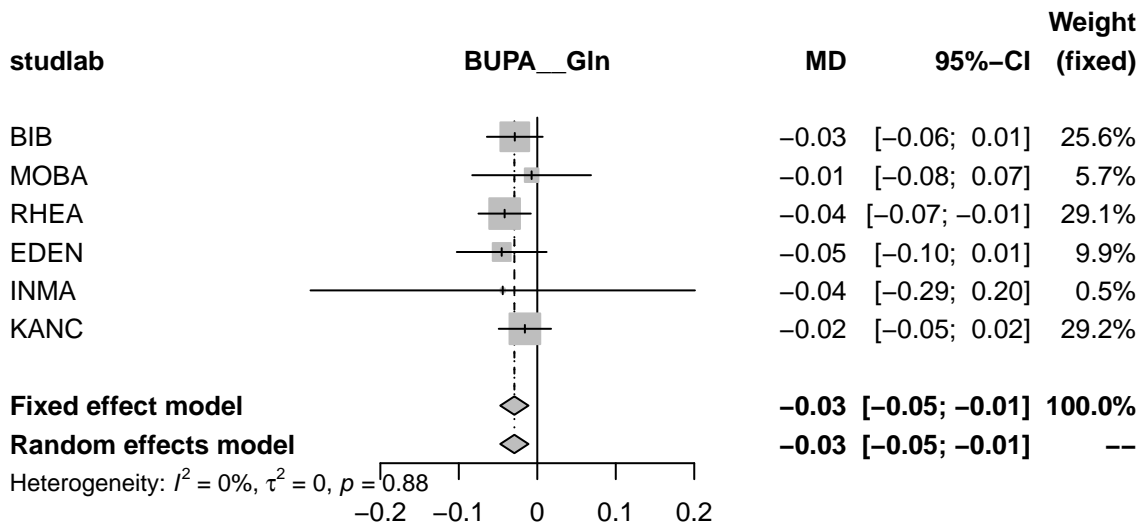

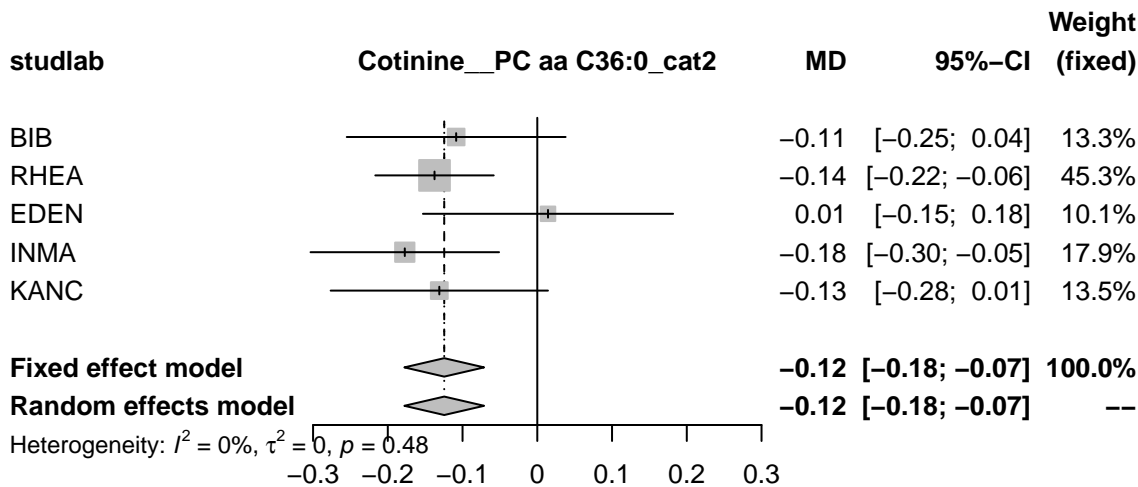

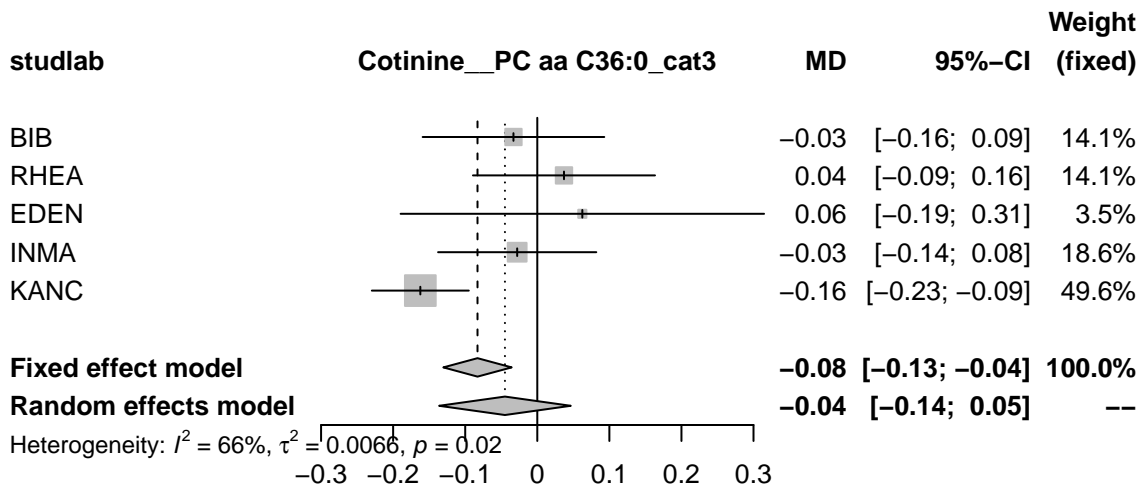

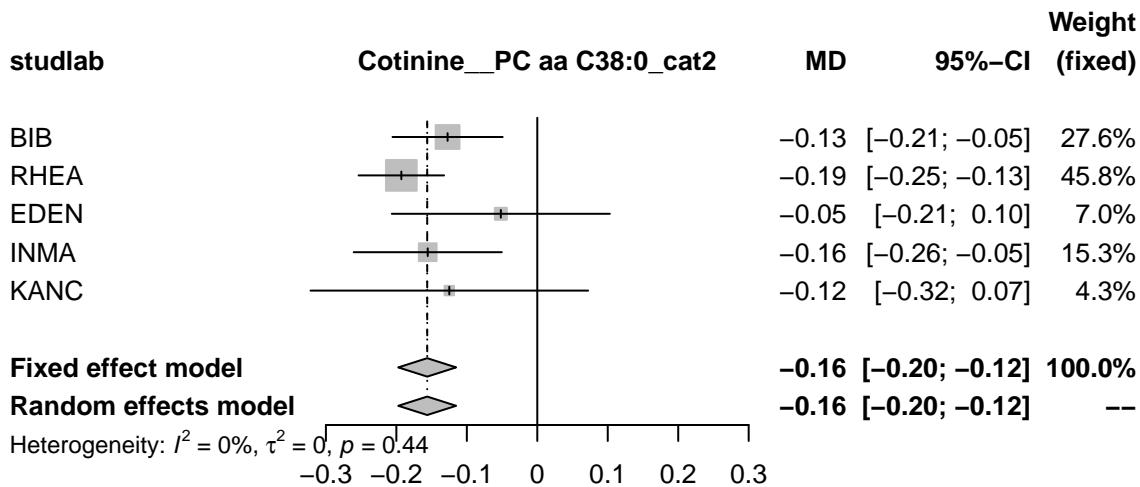

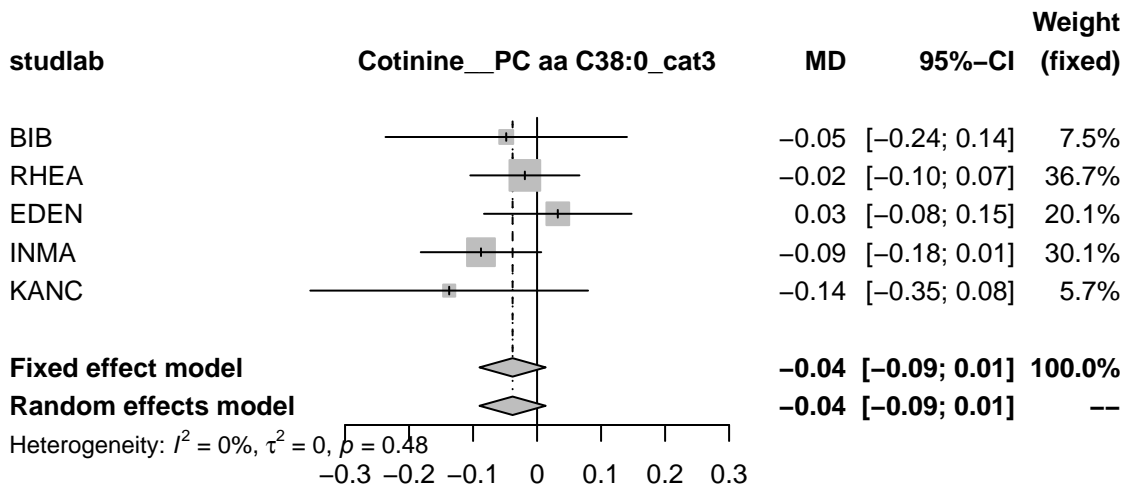

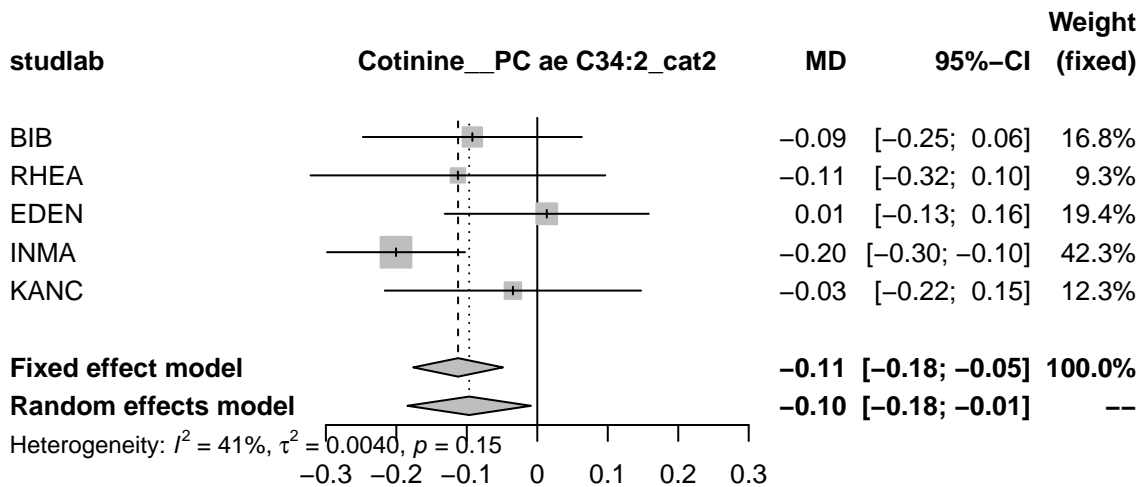

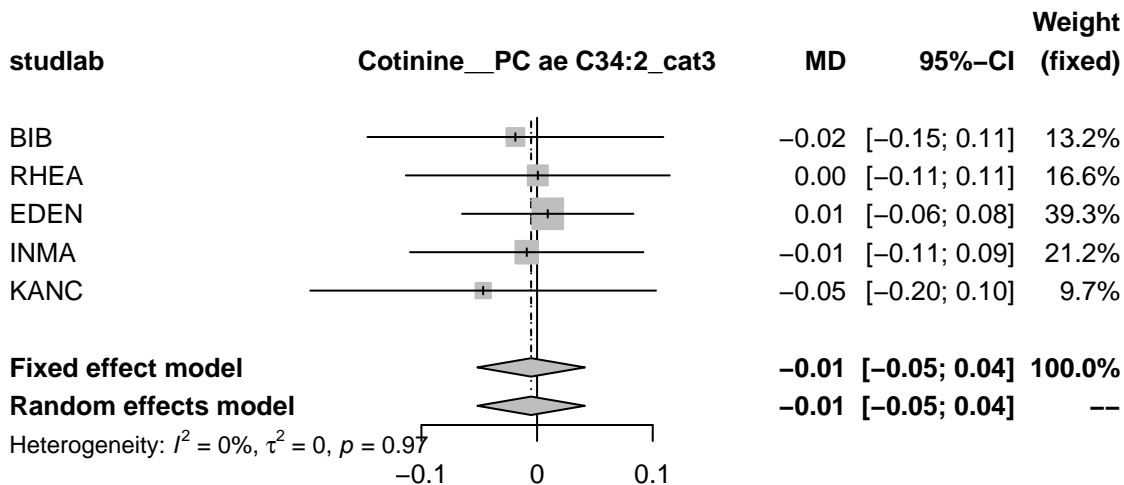

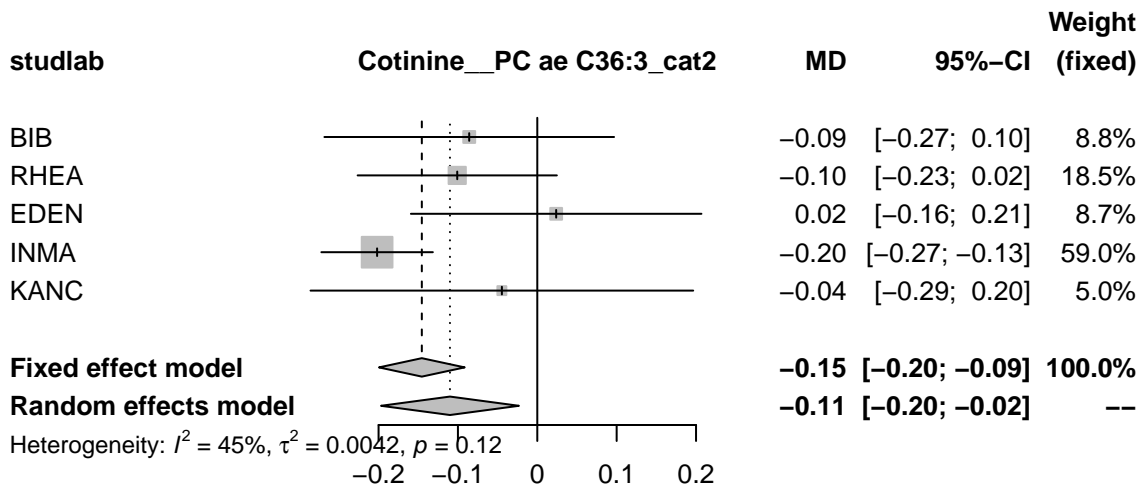

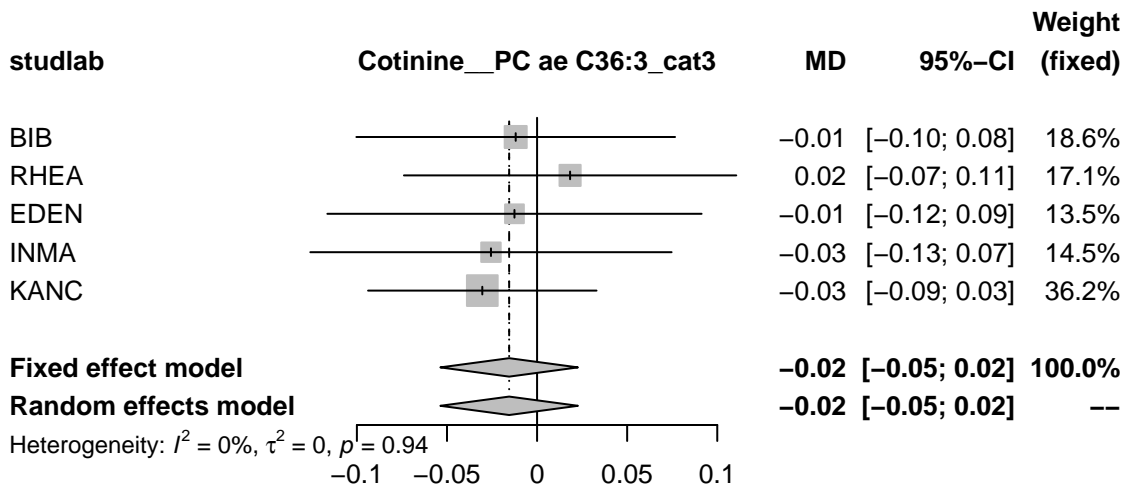

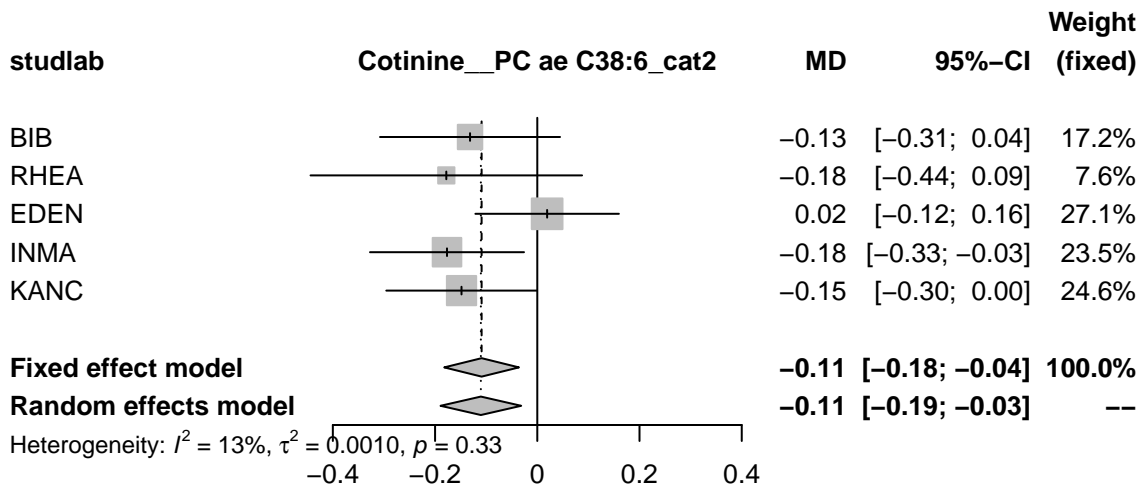

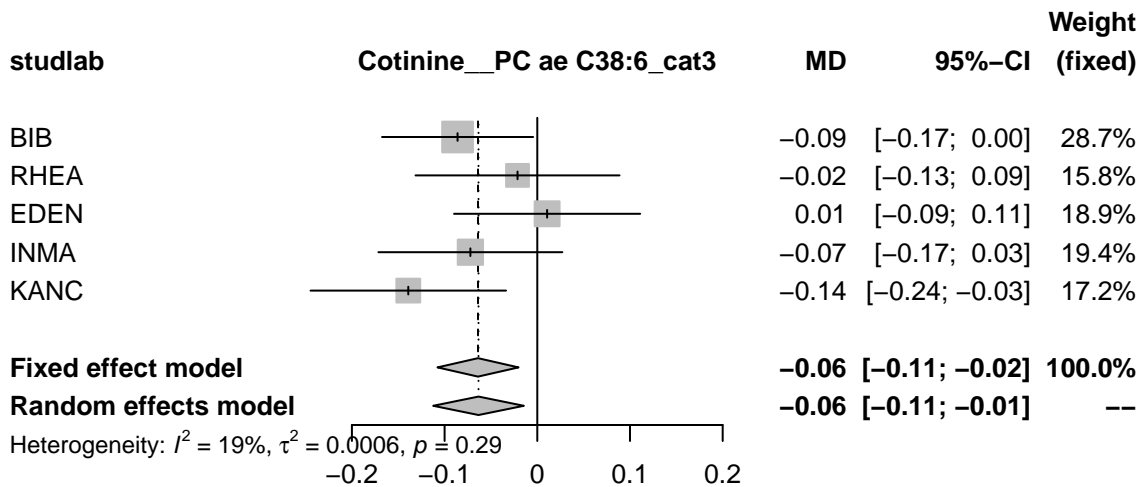

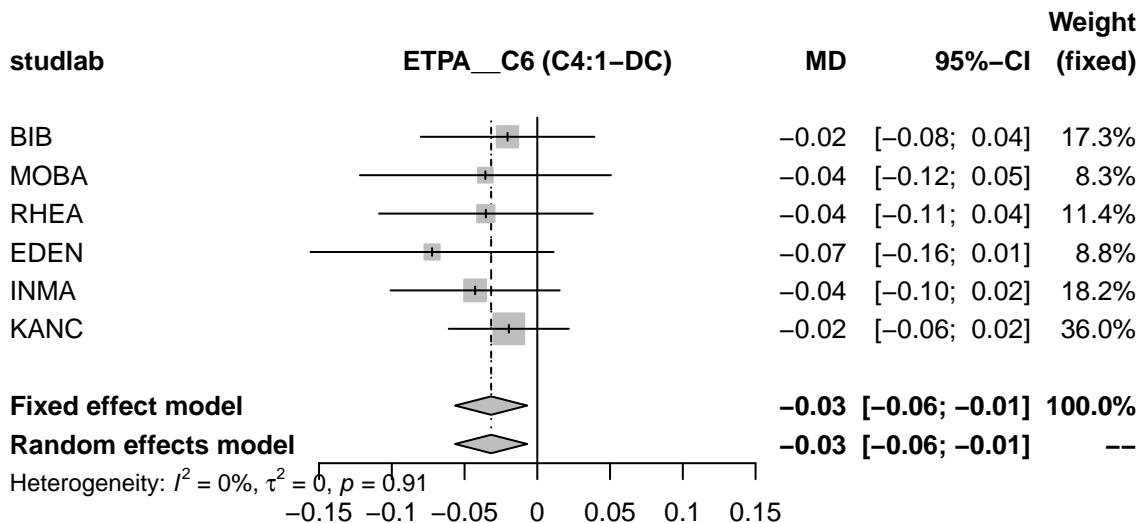

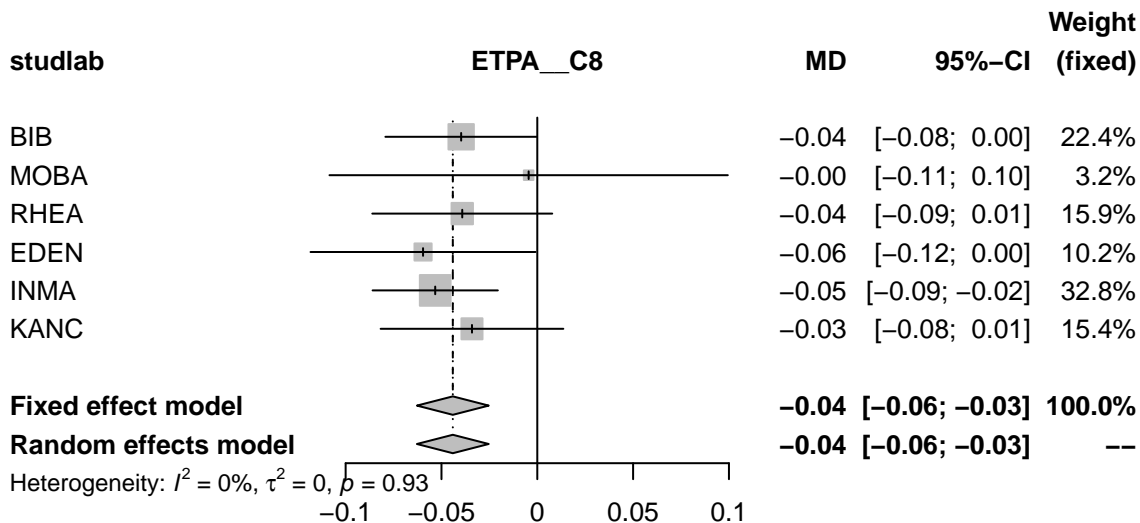

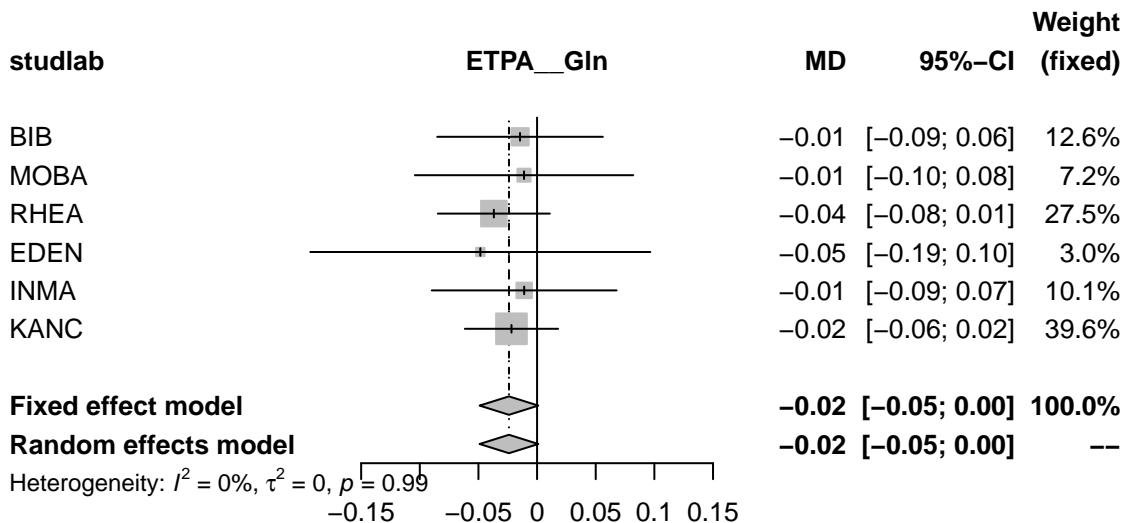

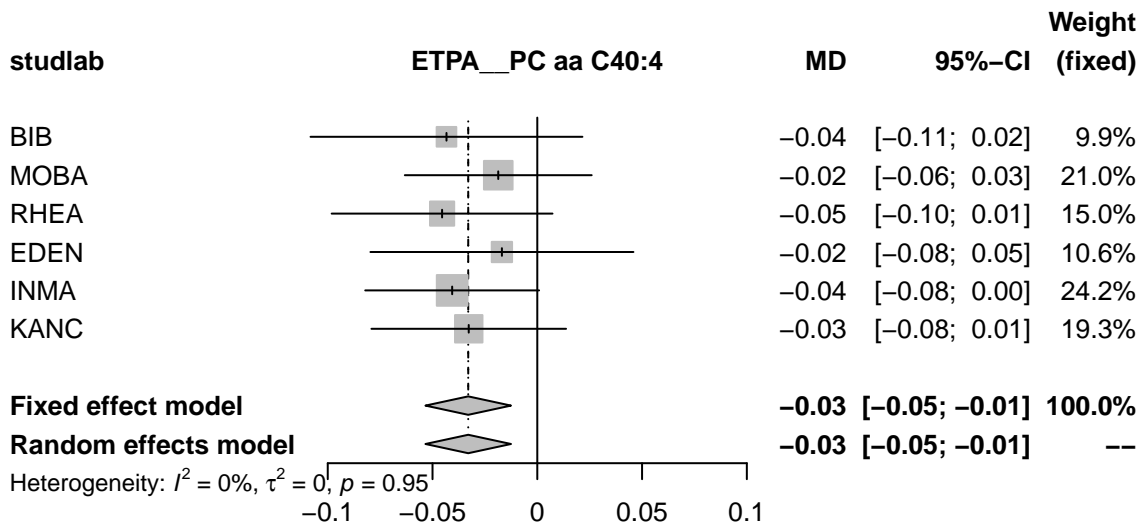

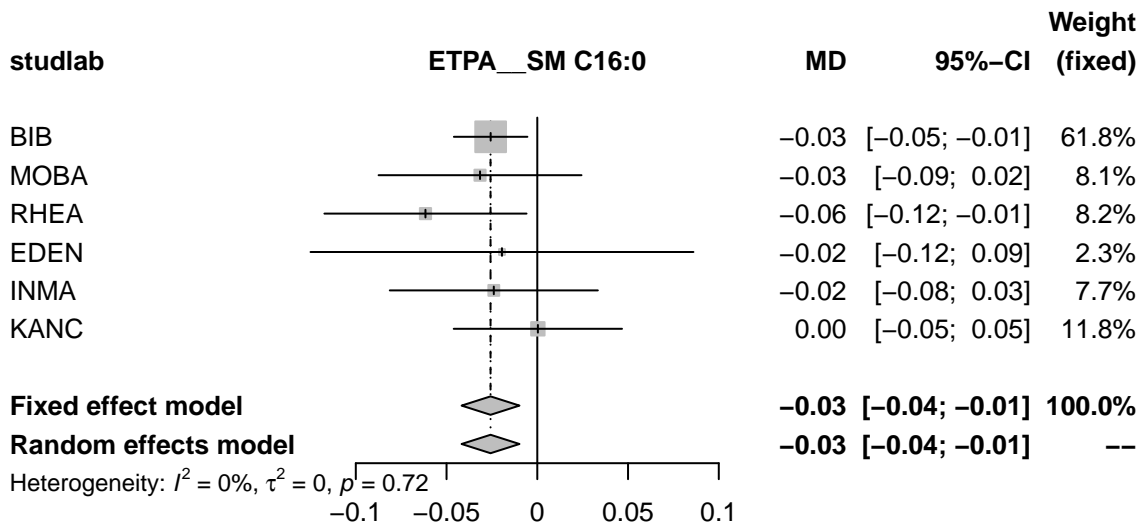

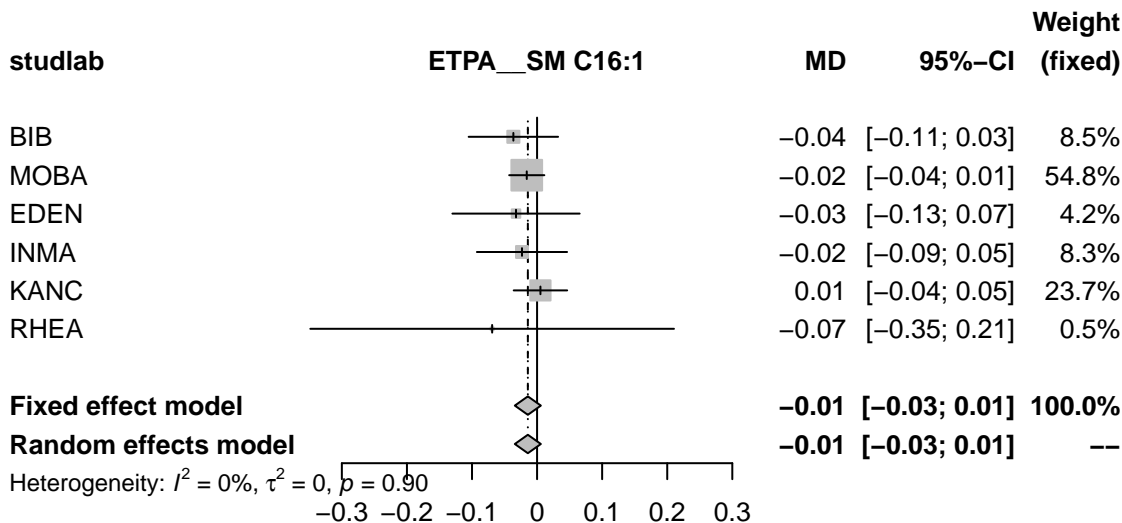

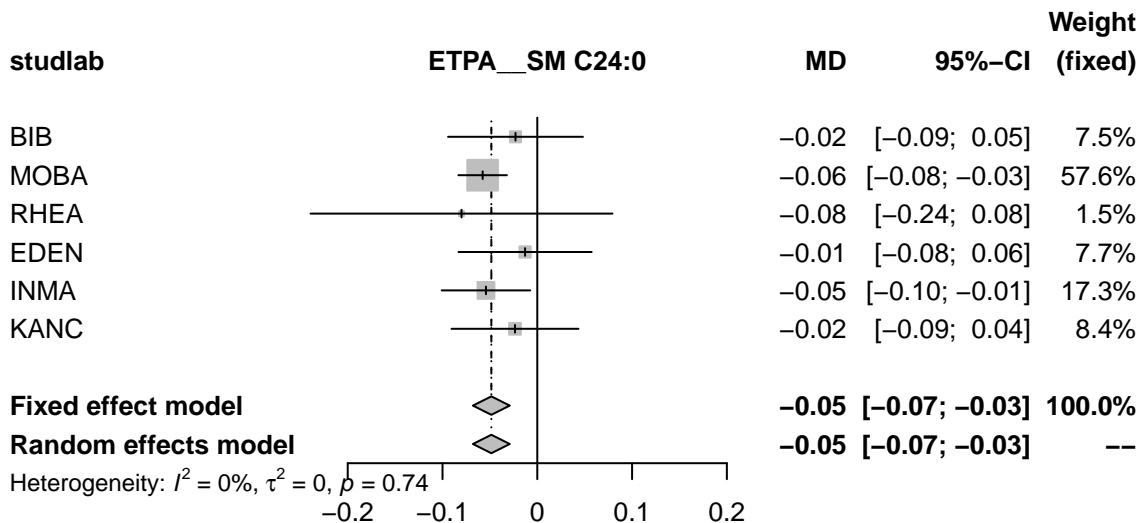

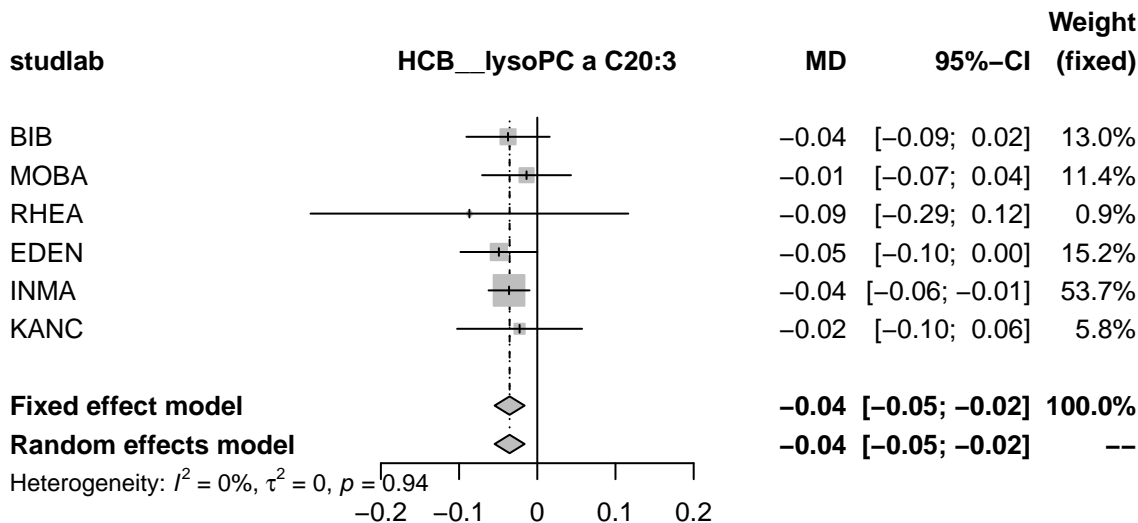

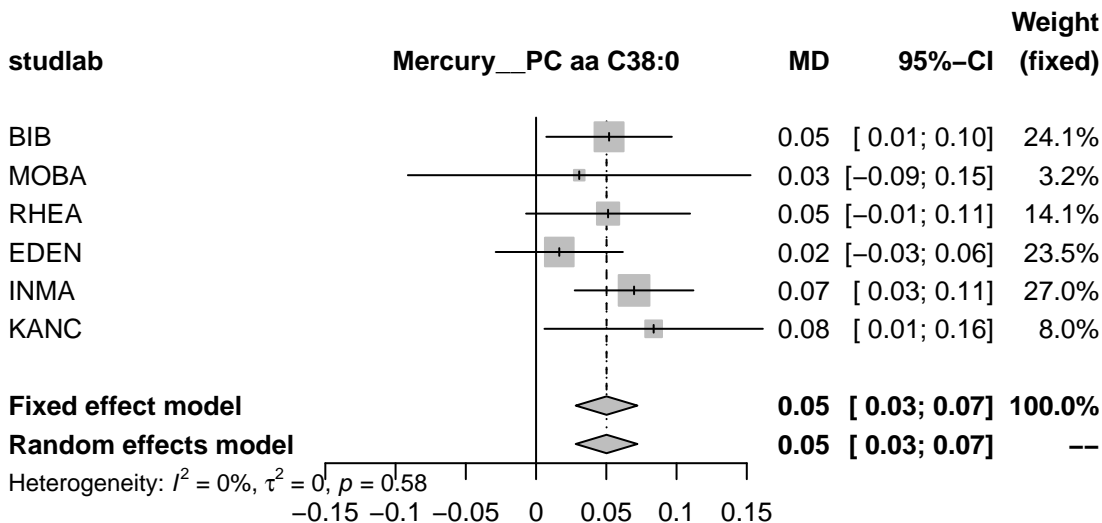

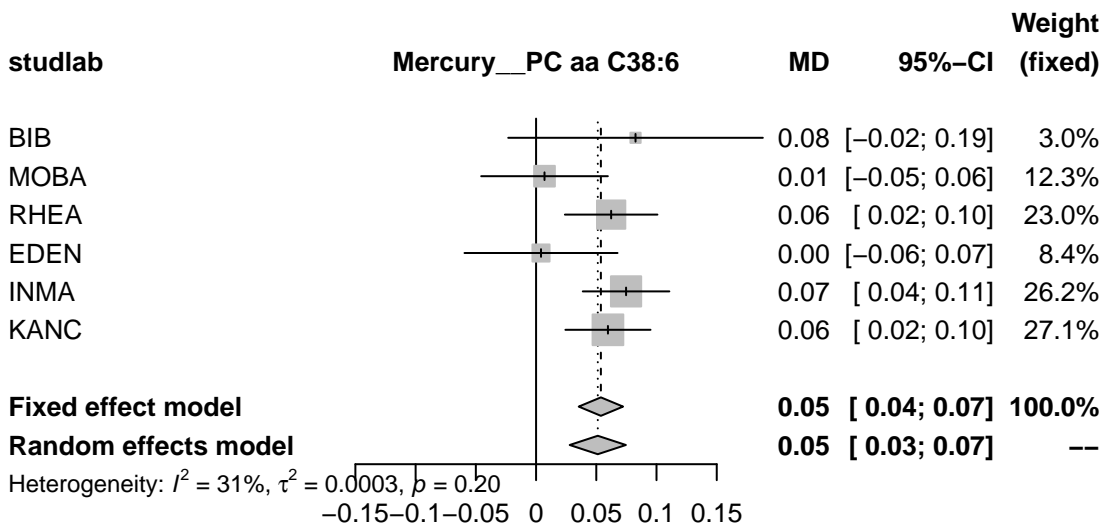

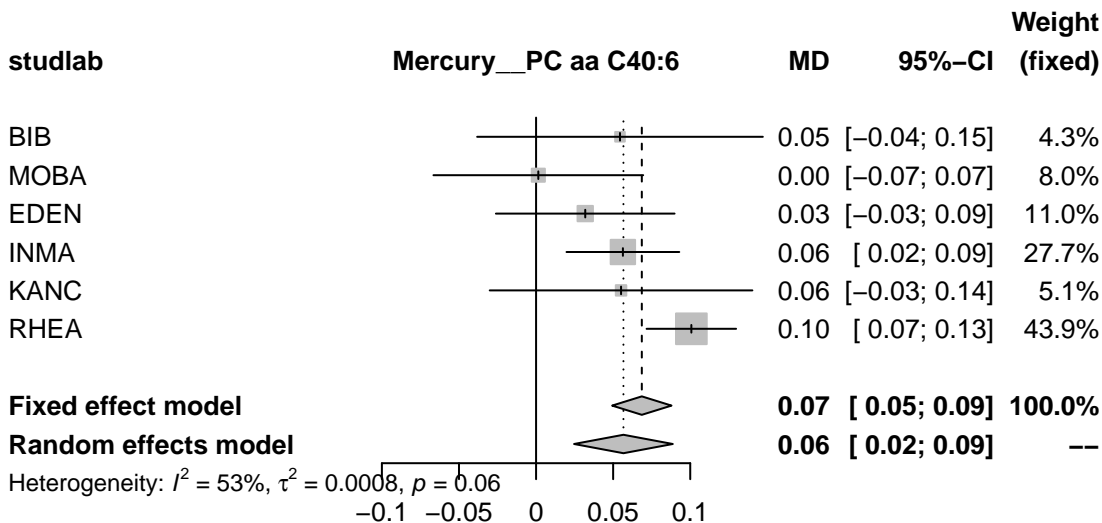

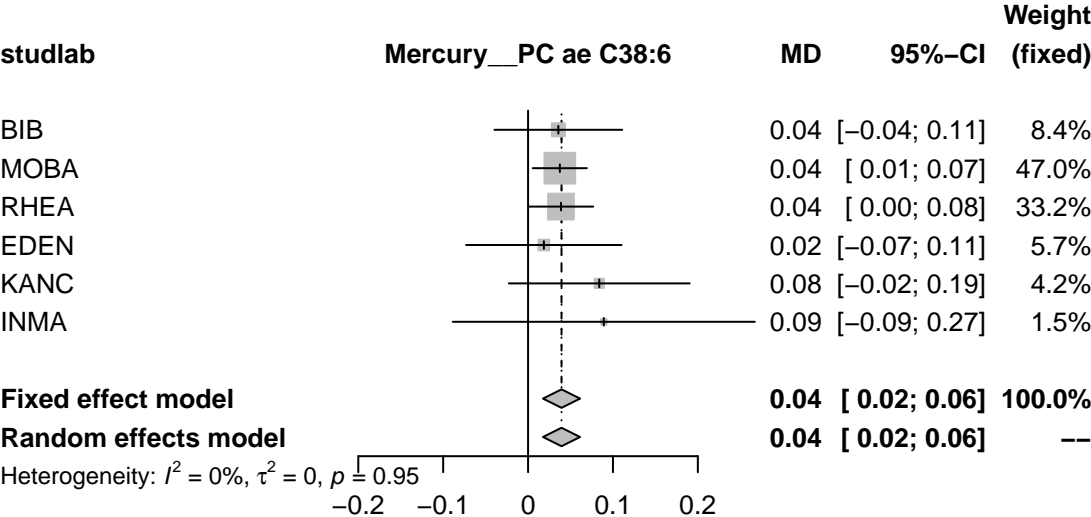

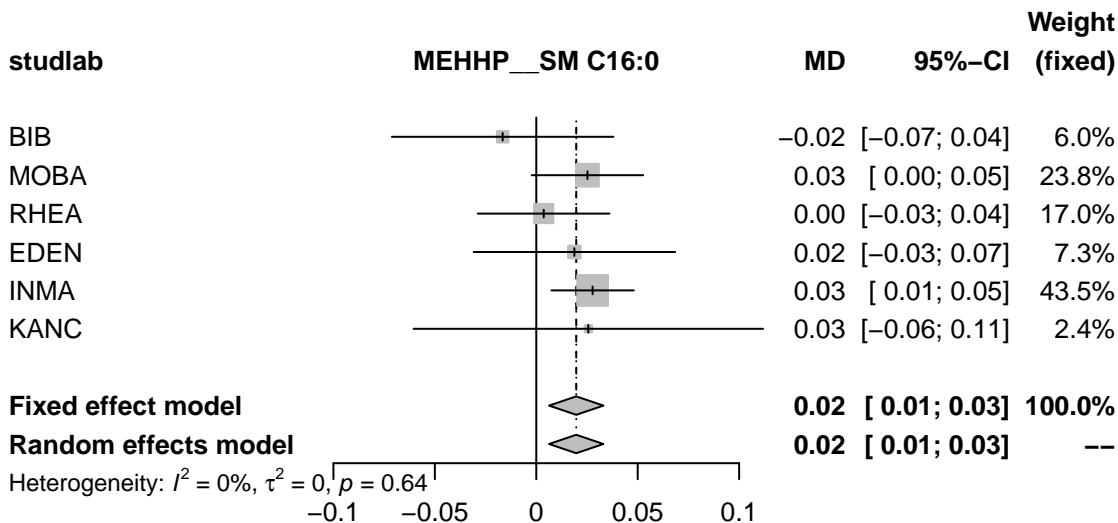

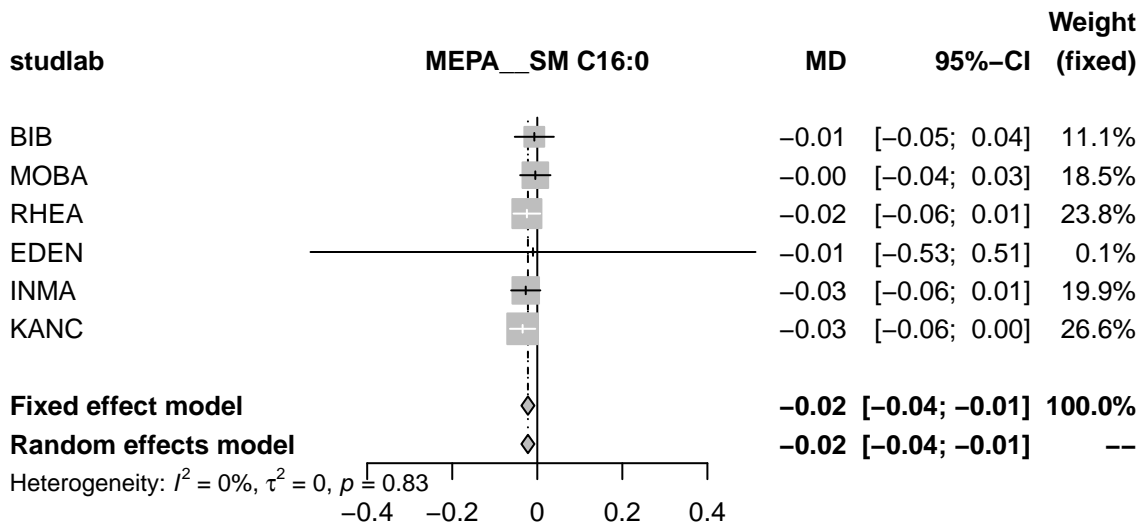

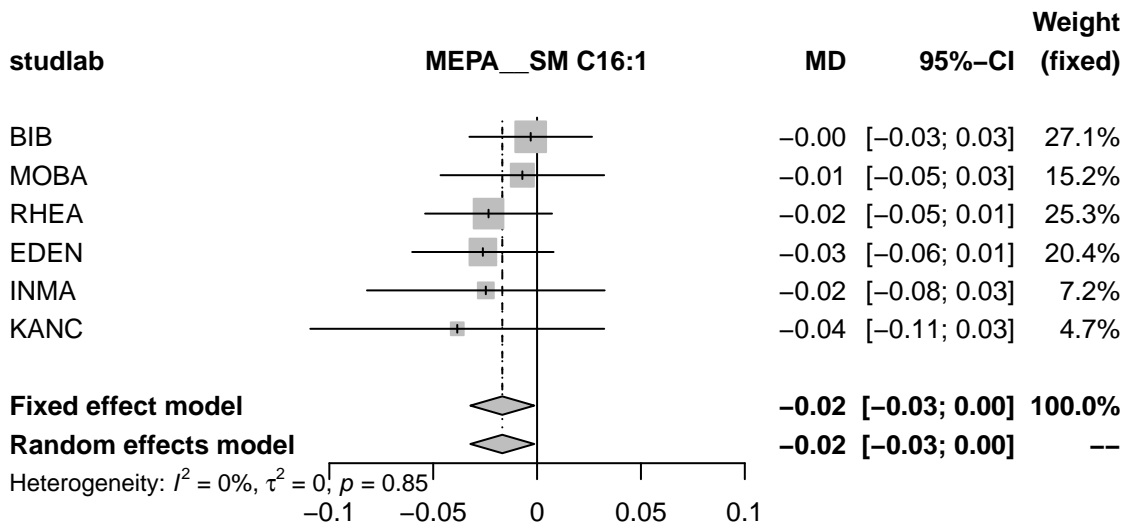

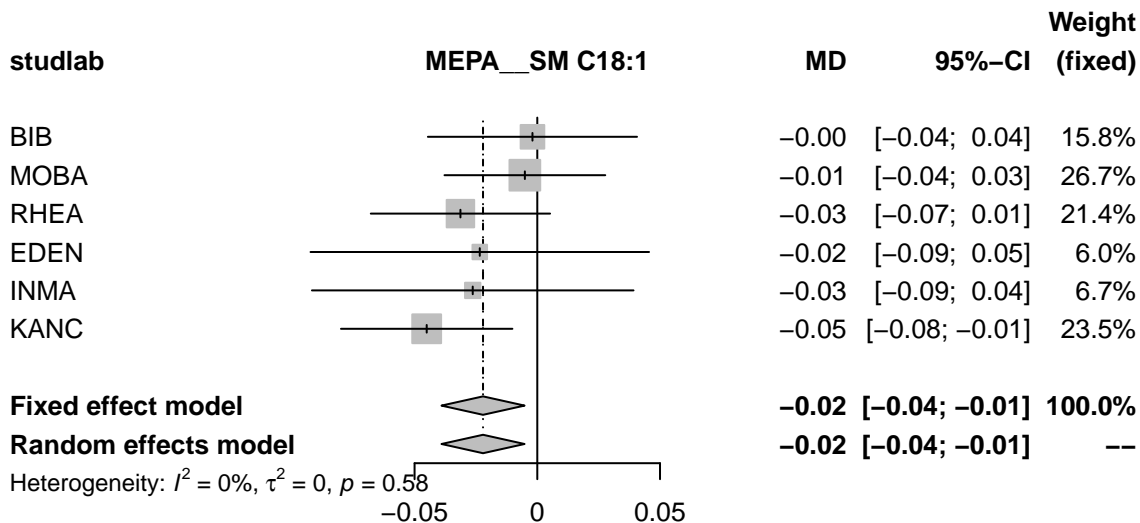

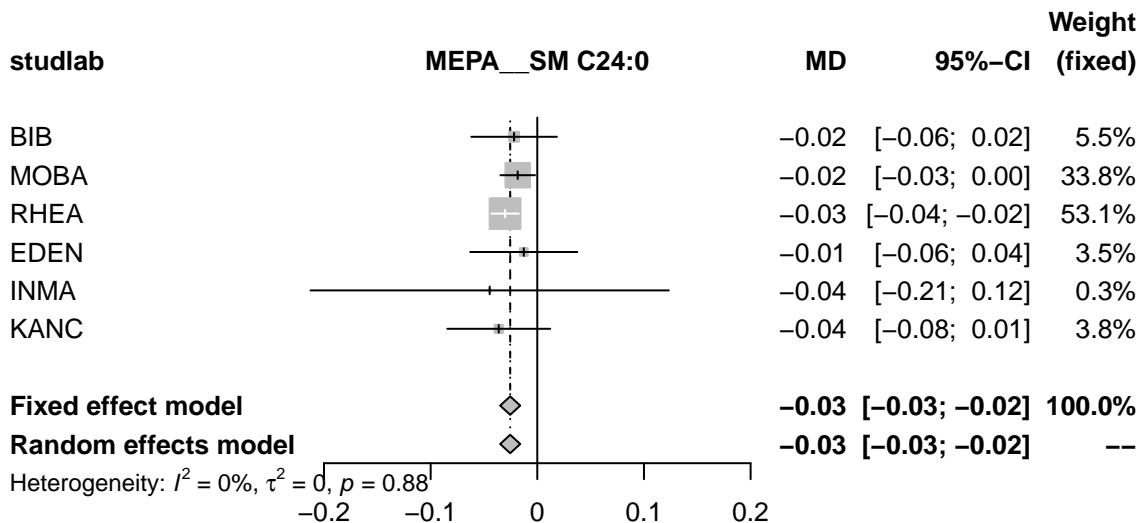

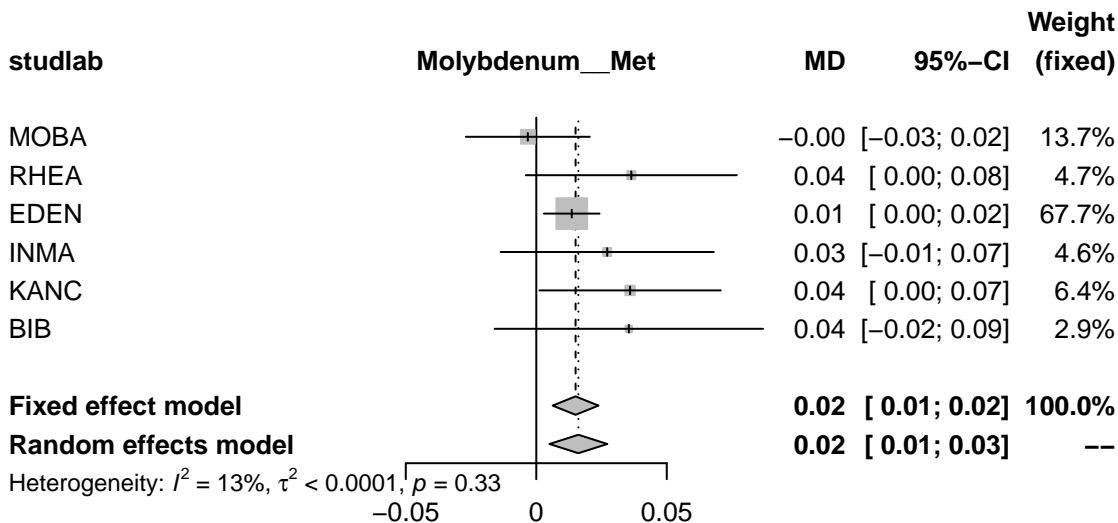

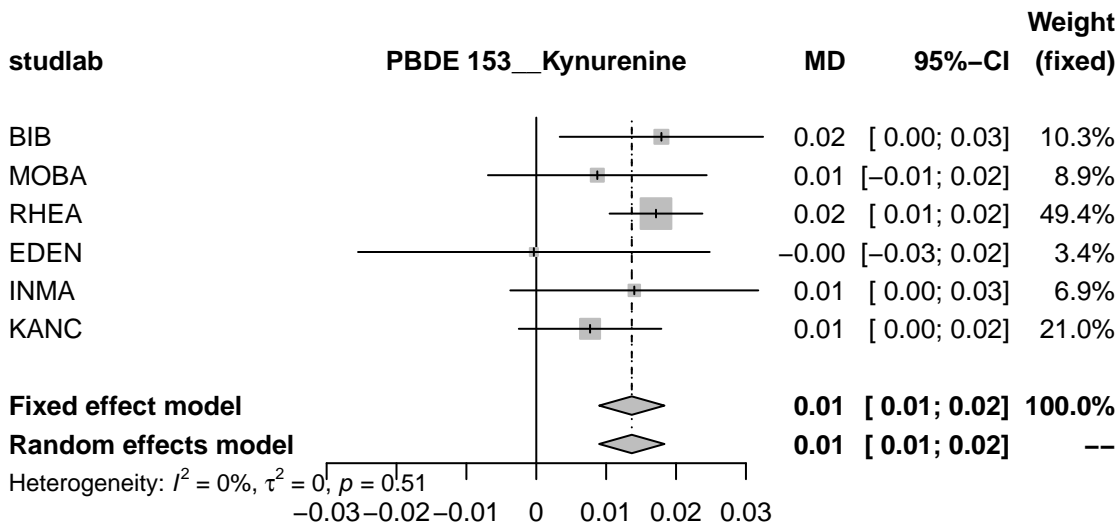

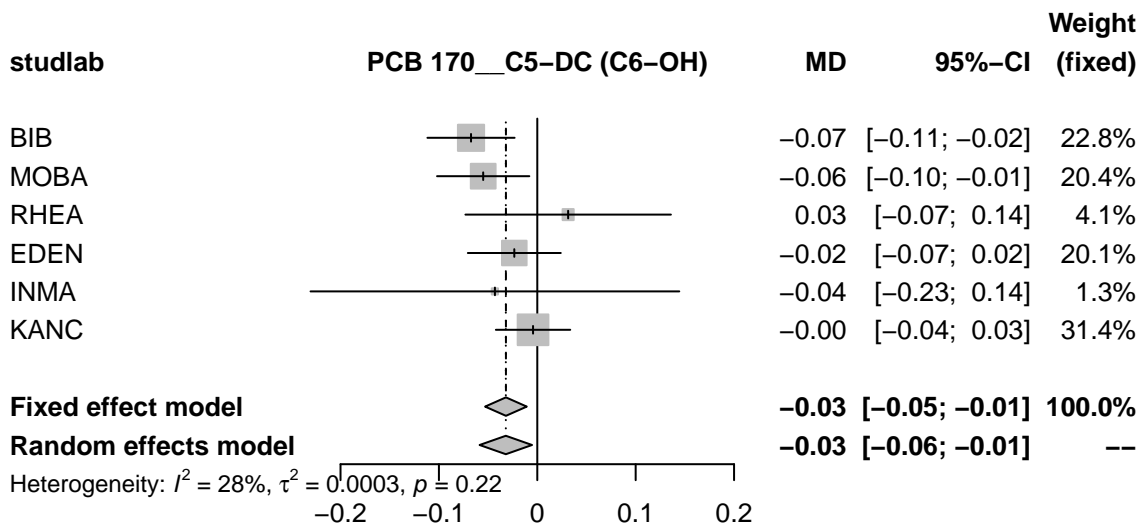

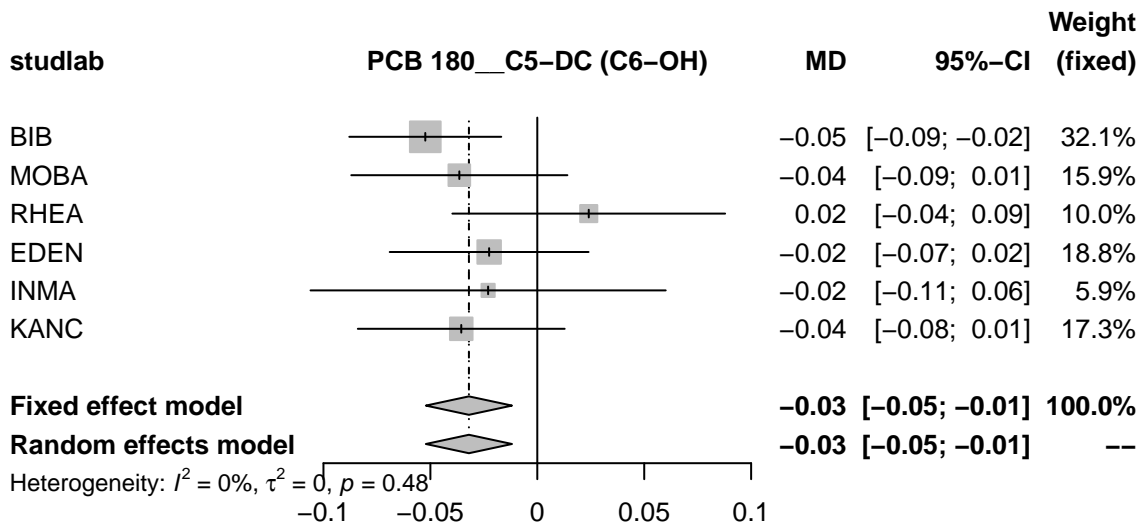

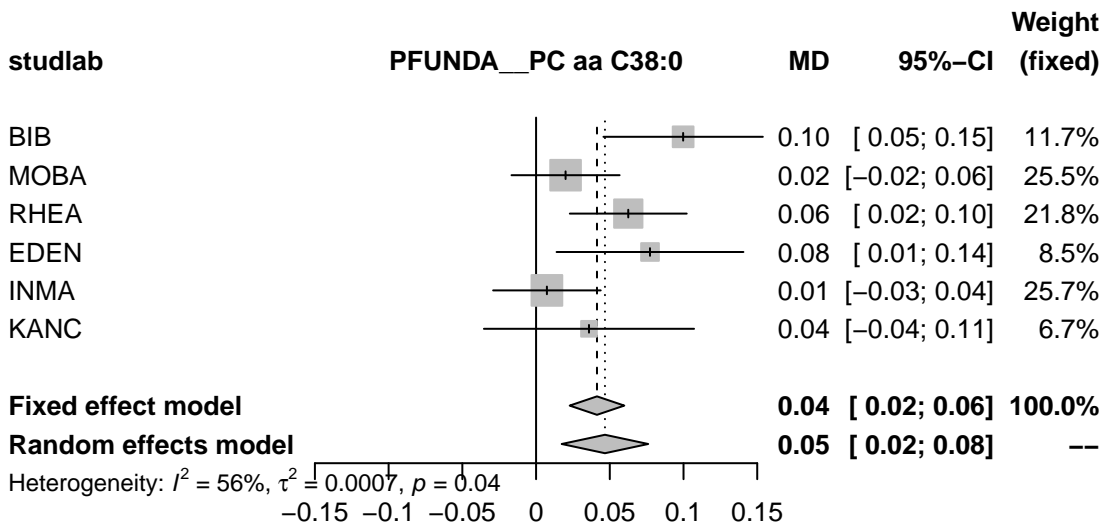

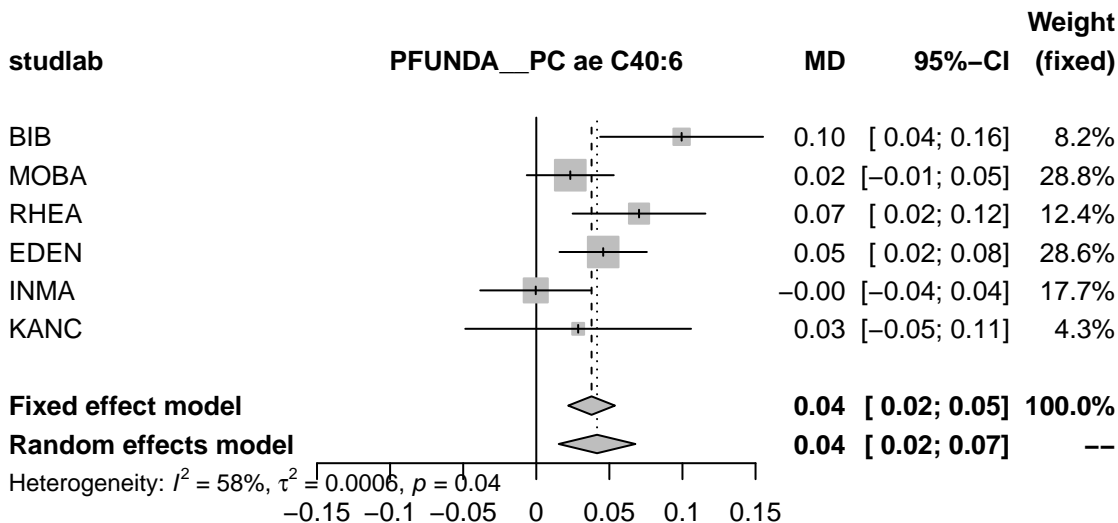

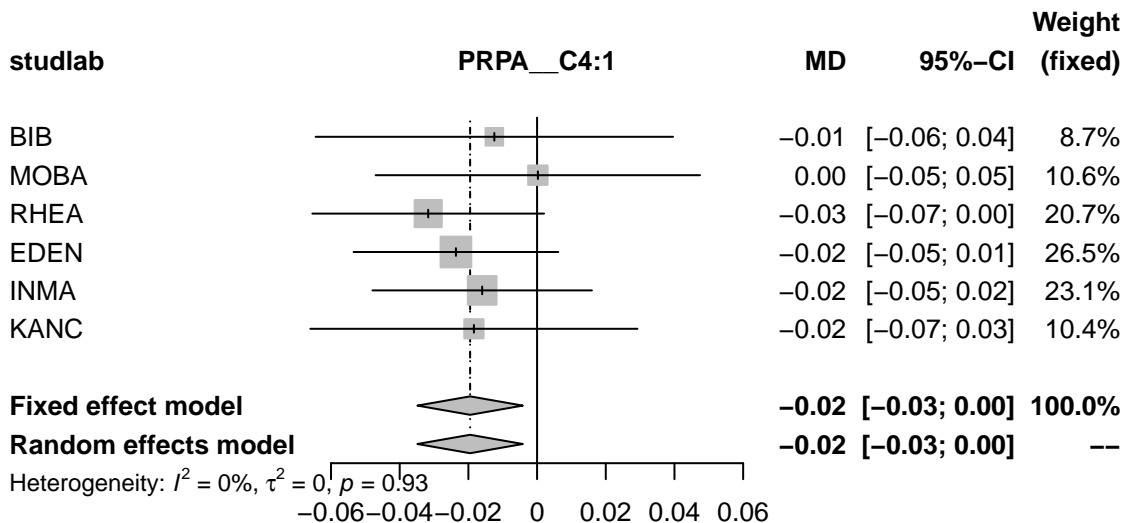

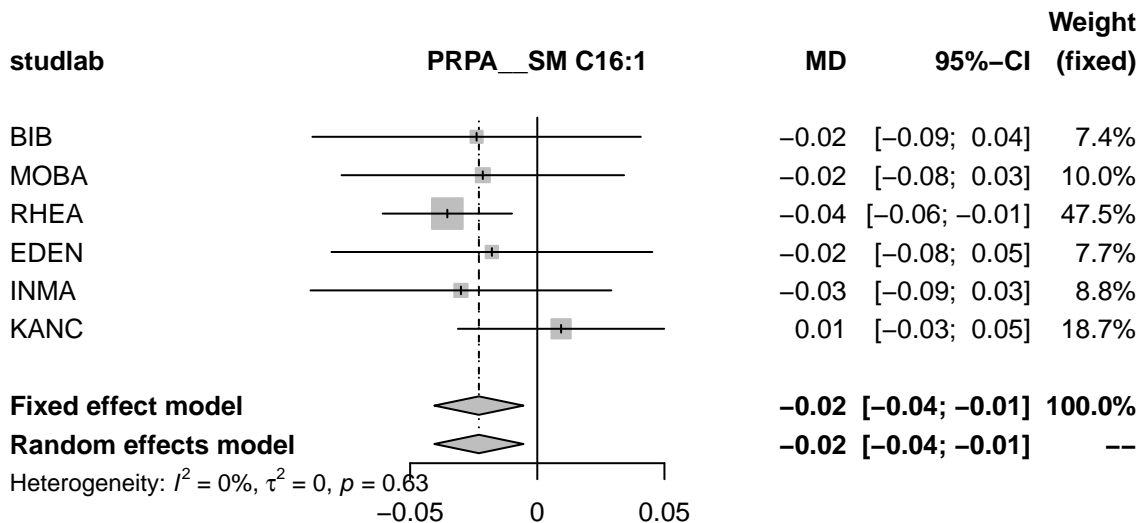

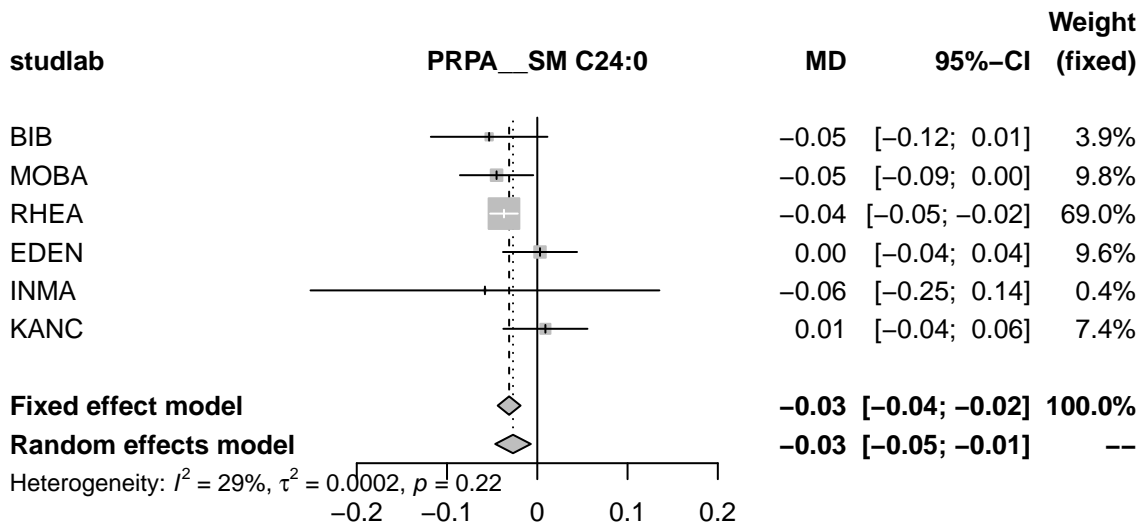

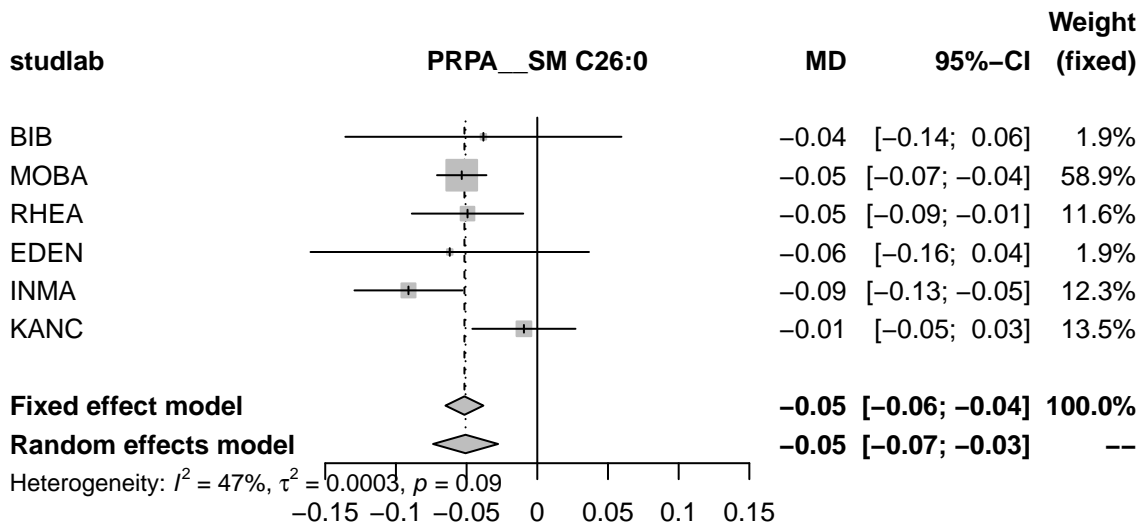

Supplement: Supplementary file 14 — Supplementary Dataset 11 [file 41467_2022_34422_MOESM14_ESM.zip › HELIX_ExpOmics_FigS2_Forestplots/HELIX_ExpOmics_FigS2E_met_s_preg.pdf]

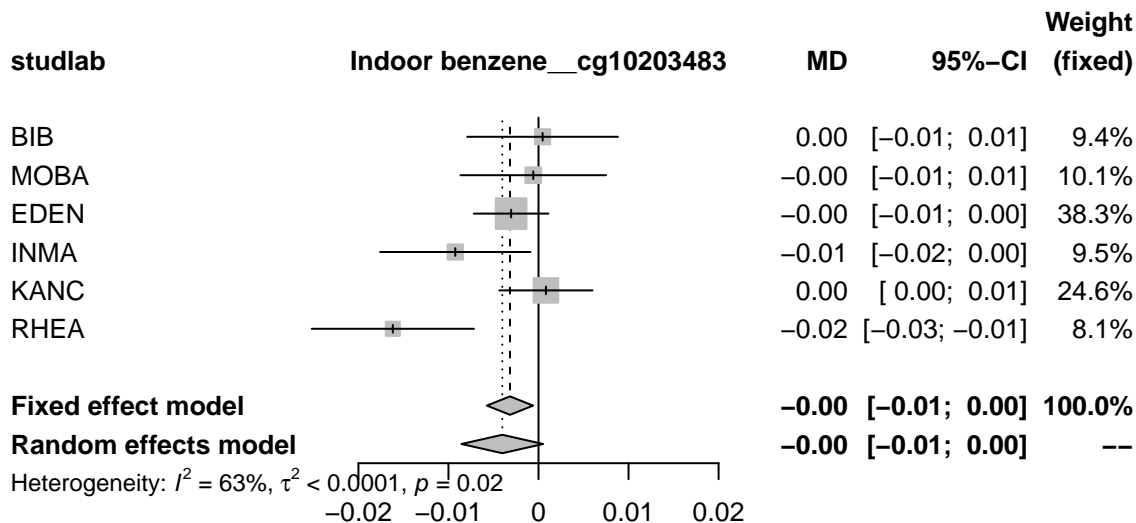

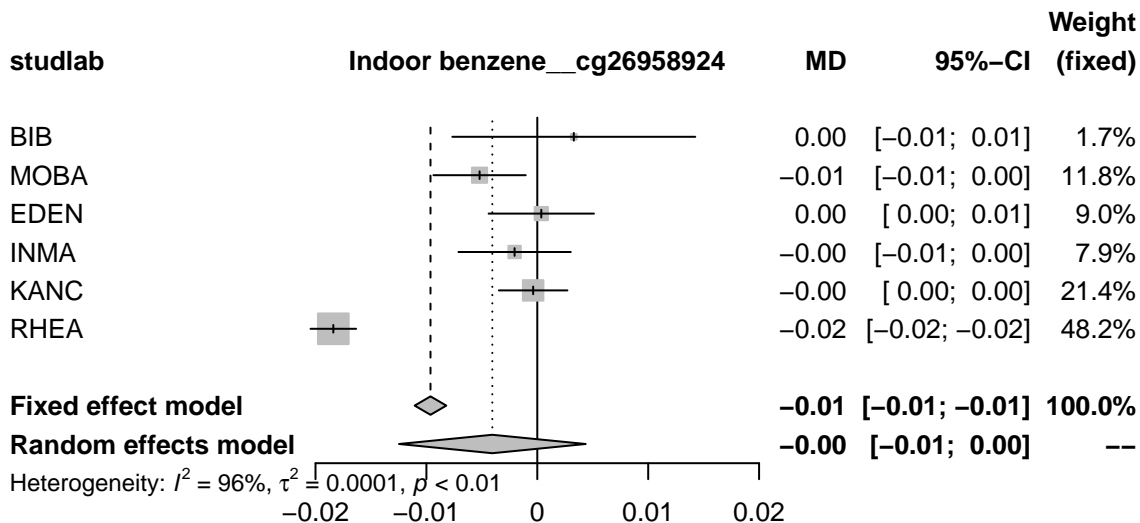

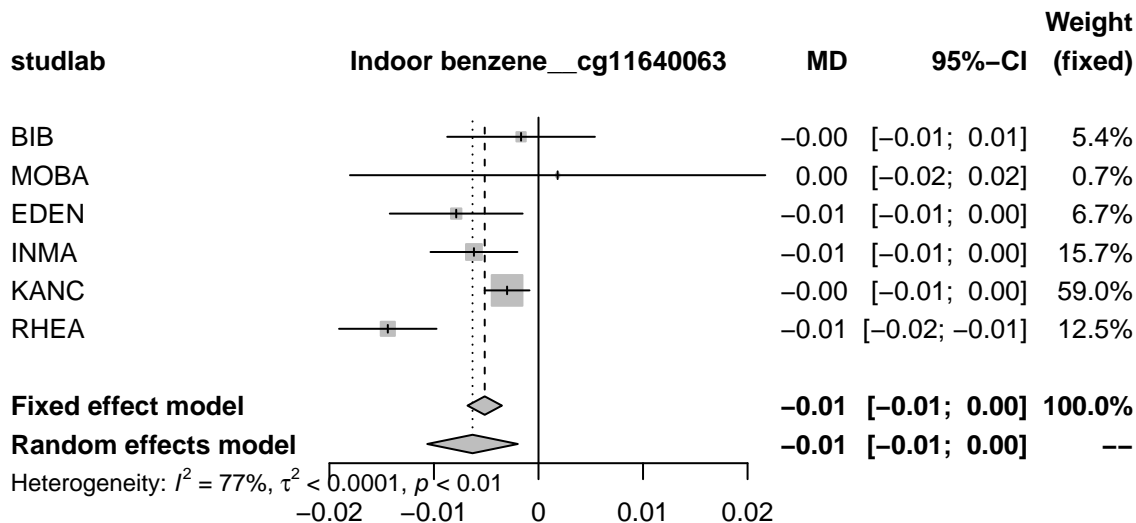

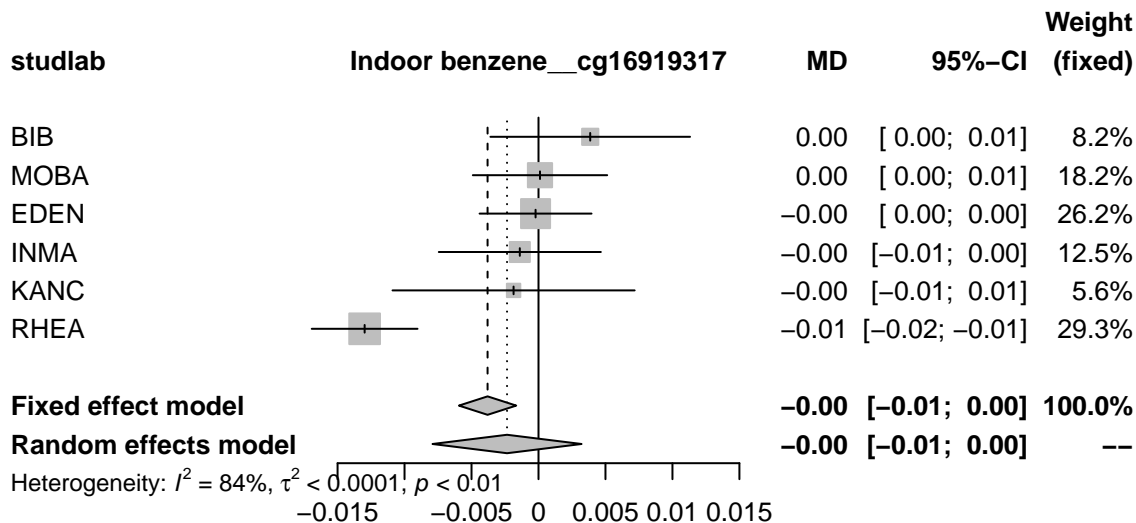

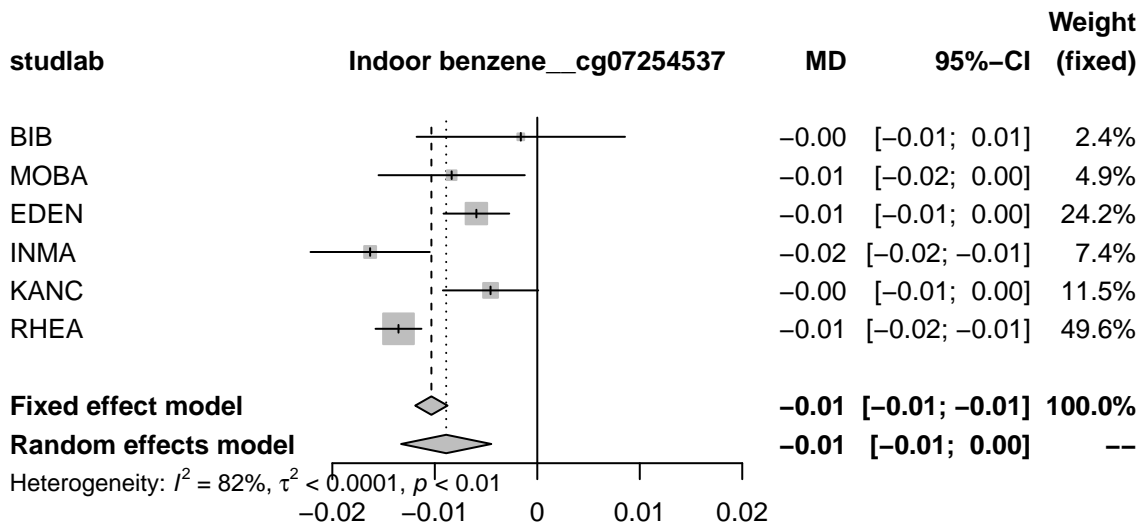

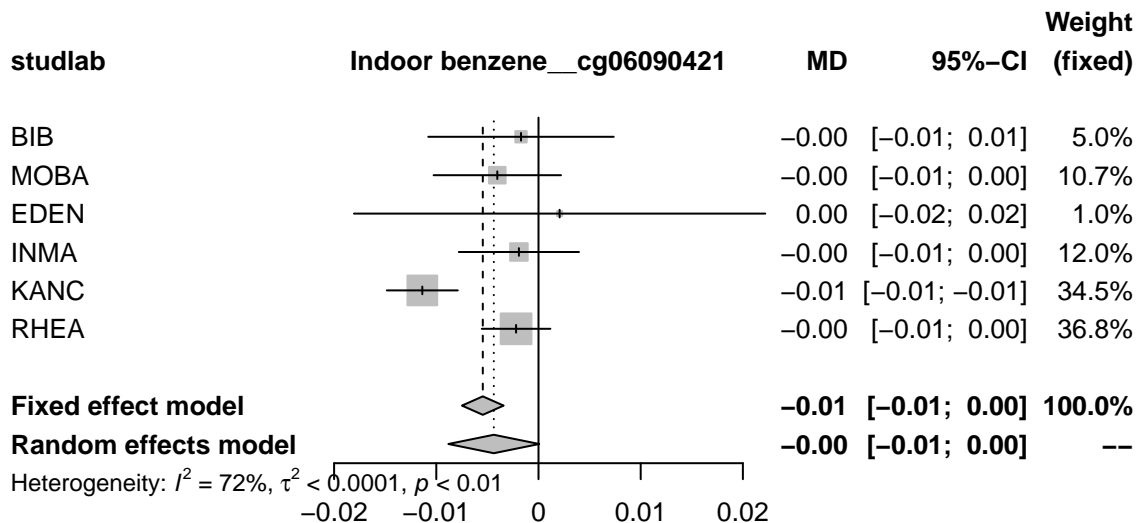

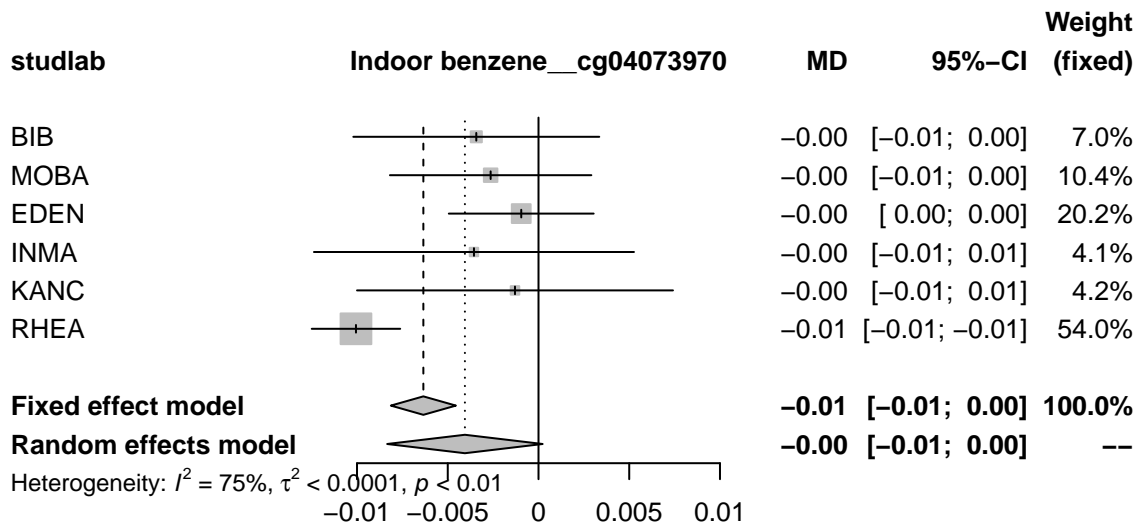

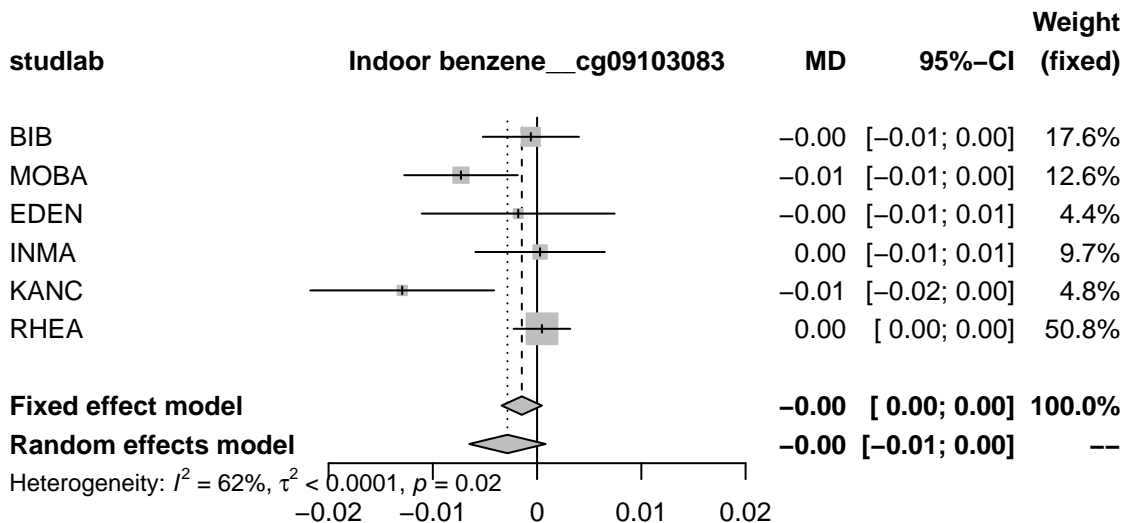

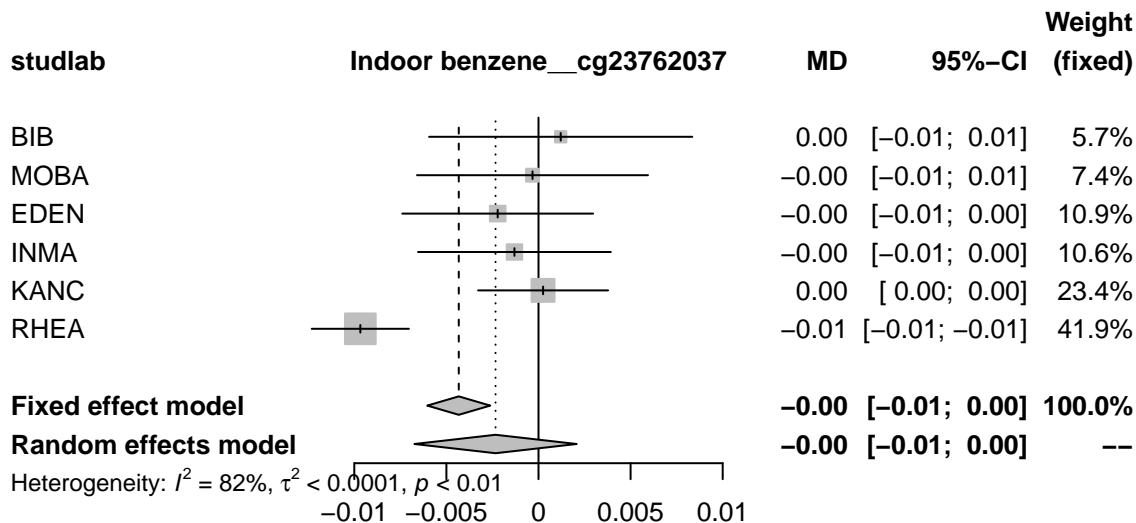

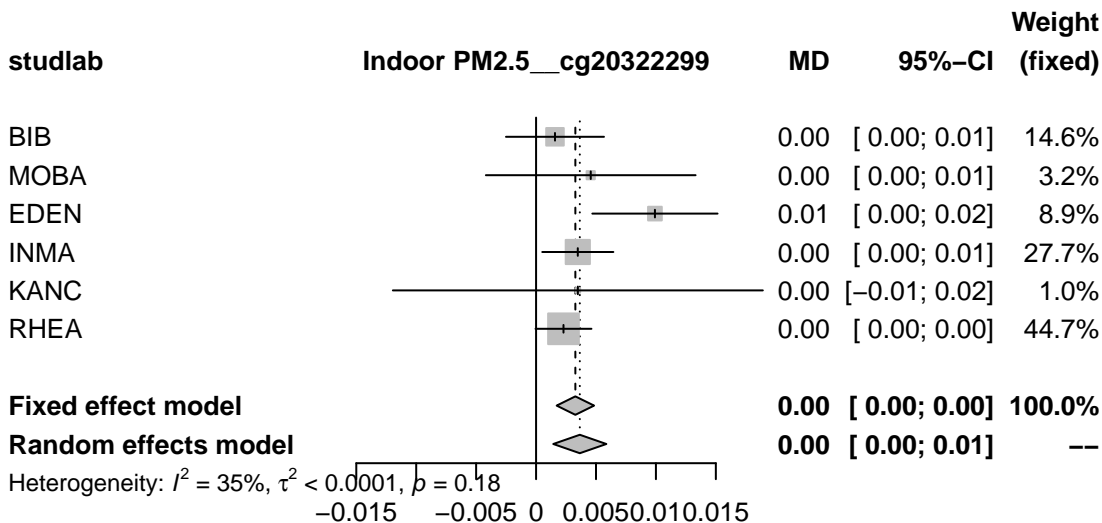

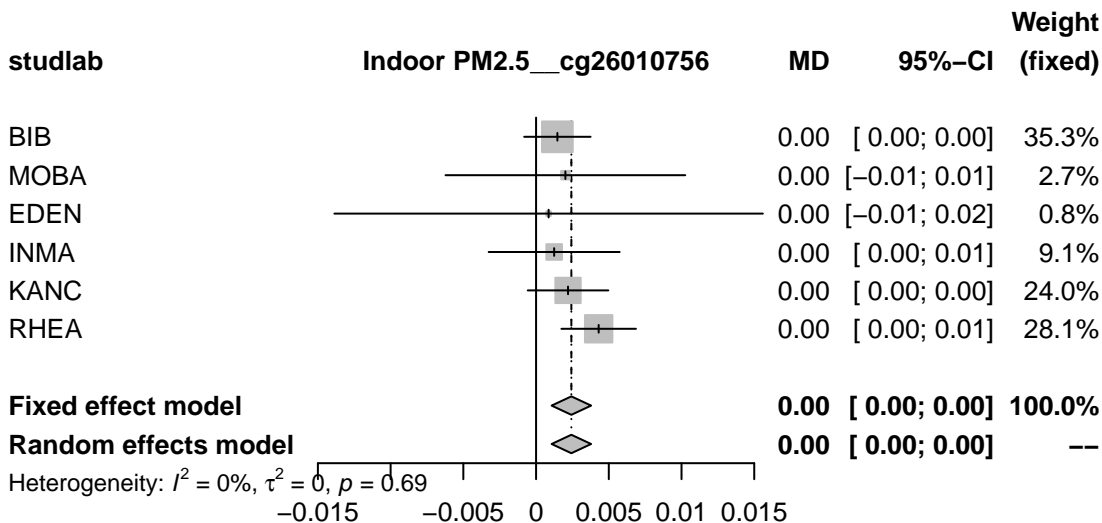

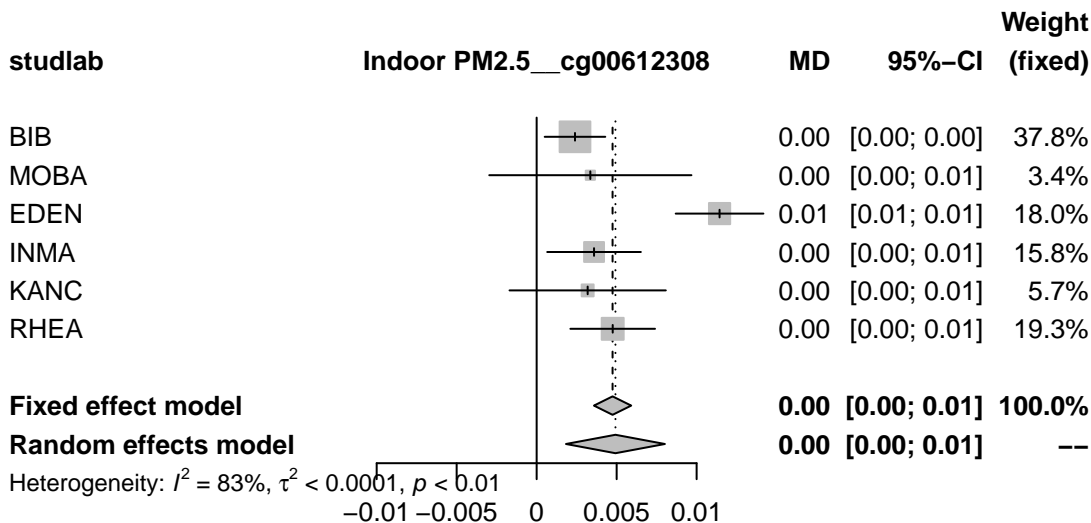

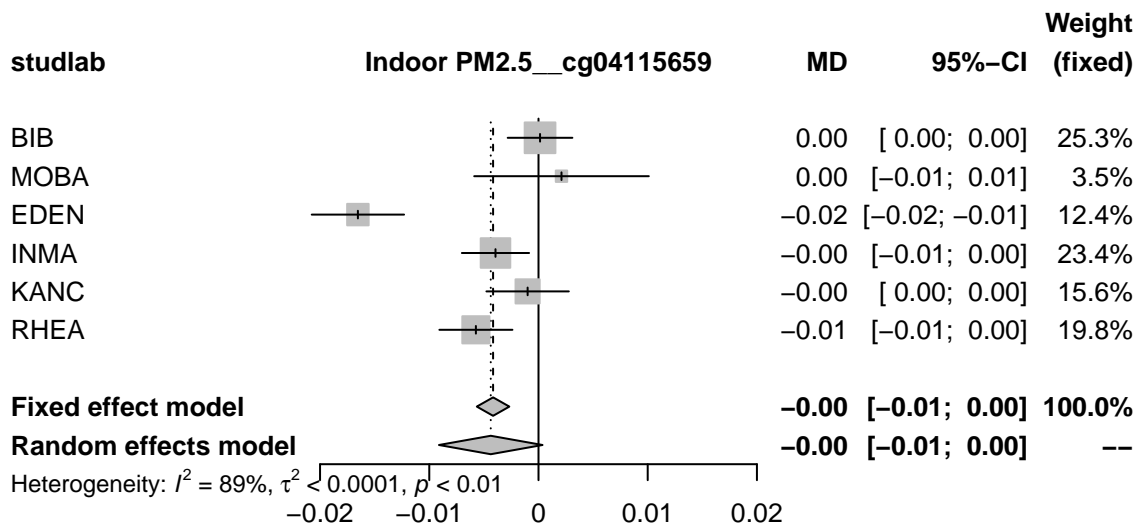

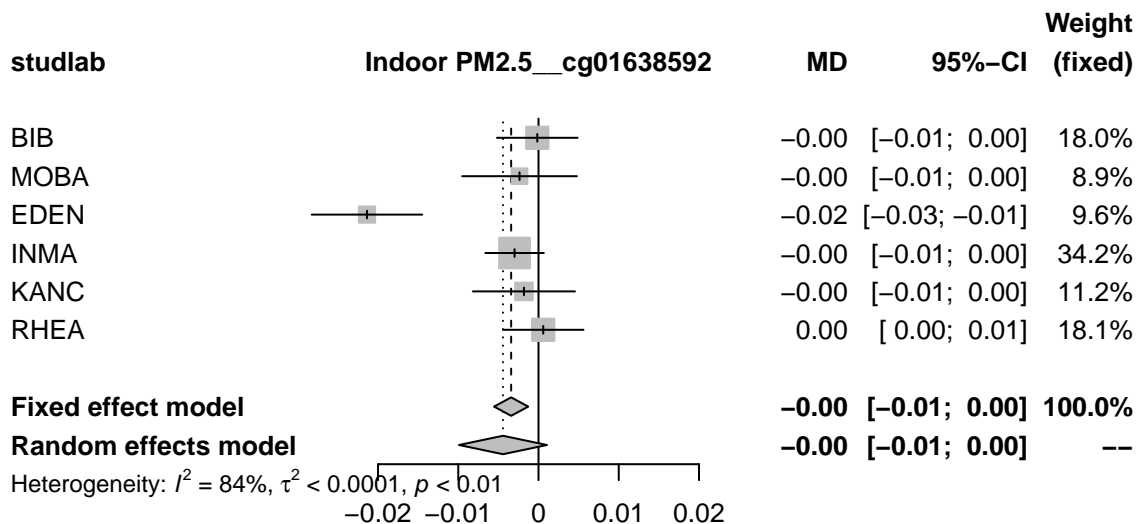

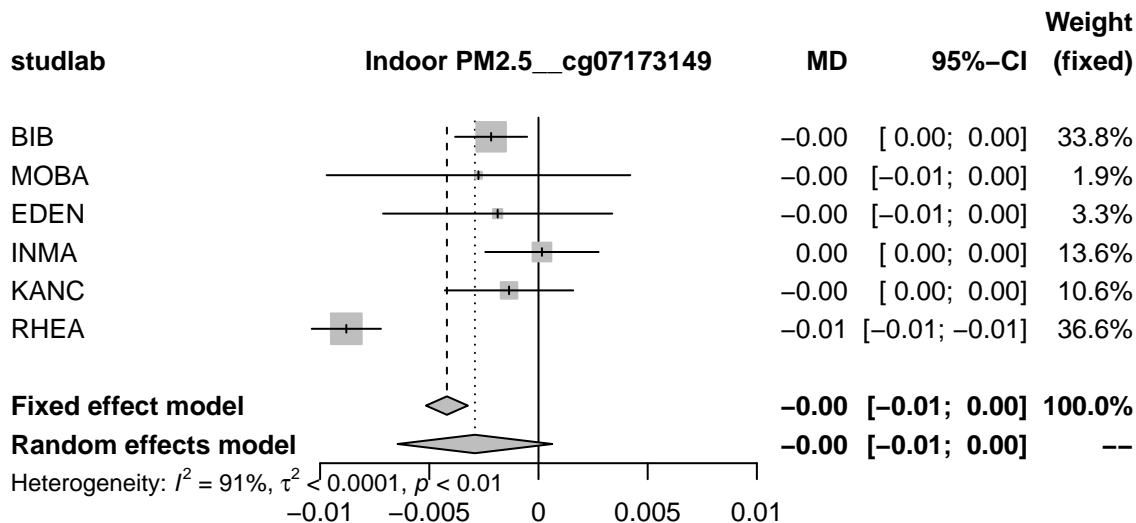

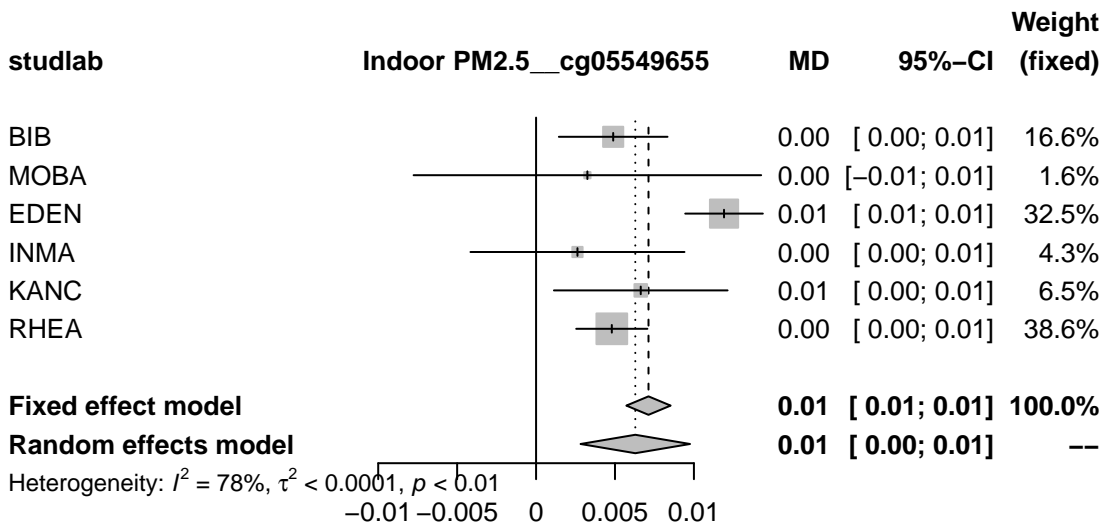

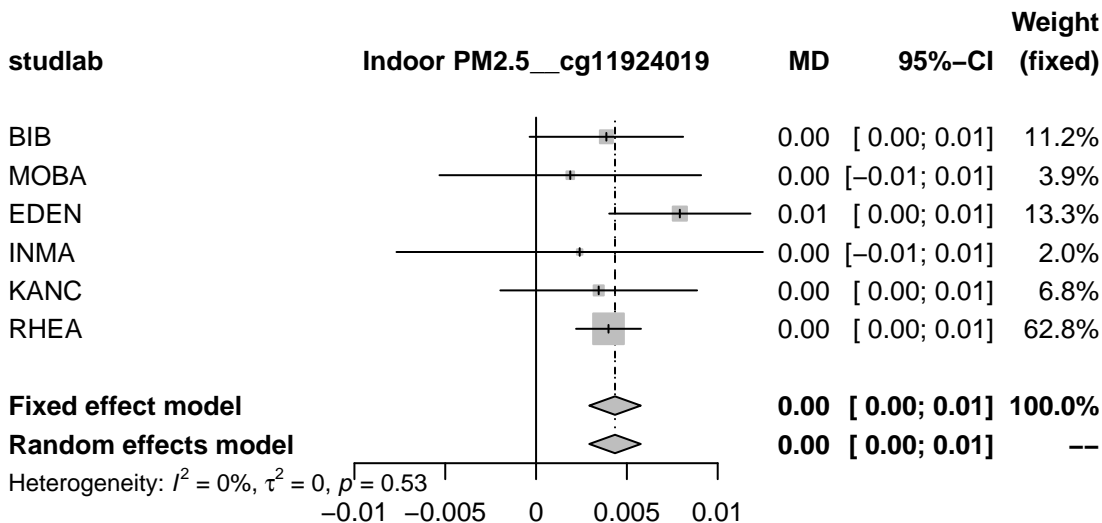

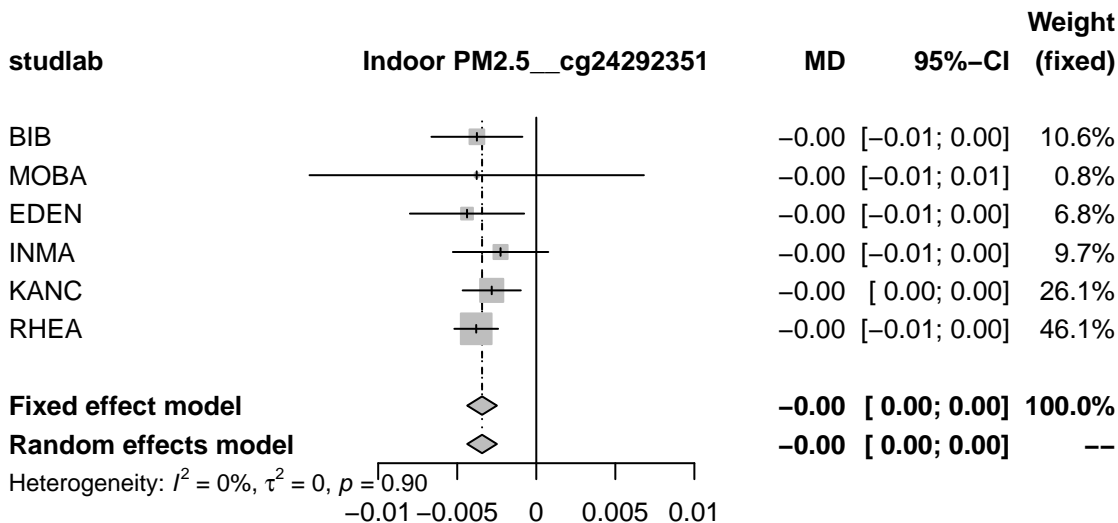

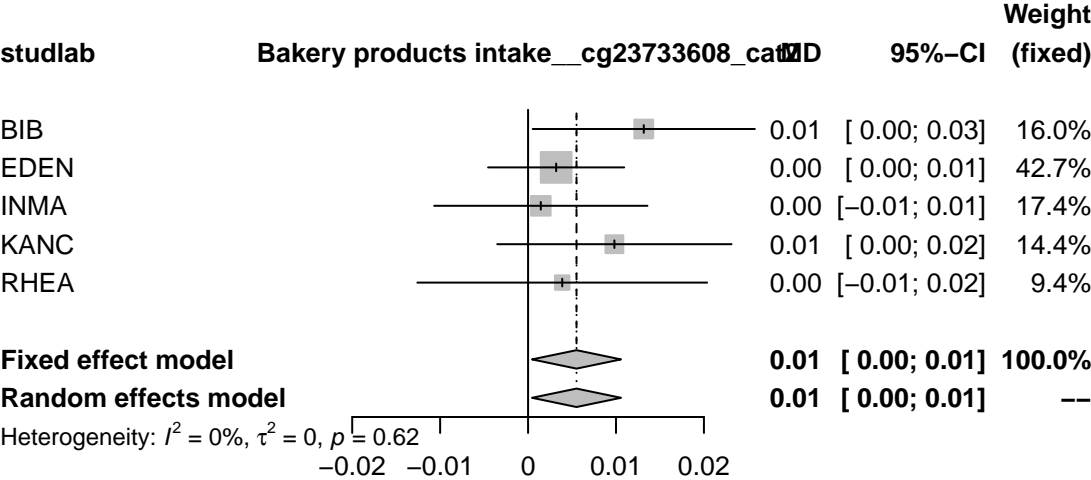

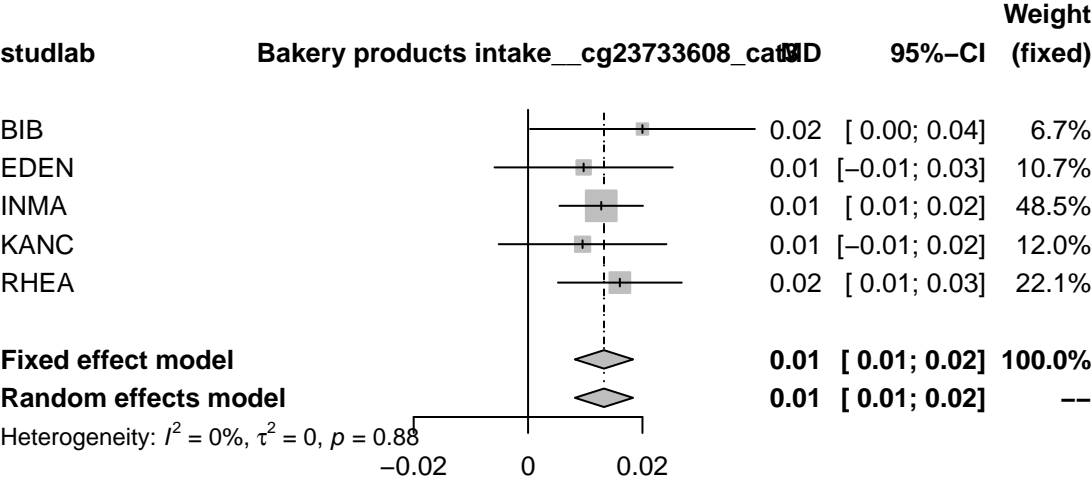

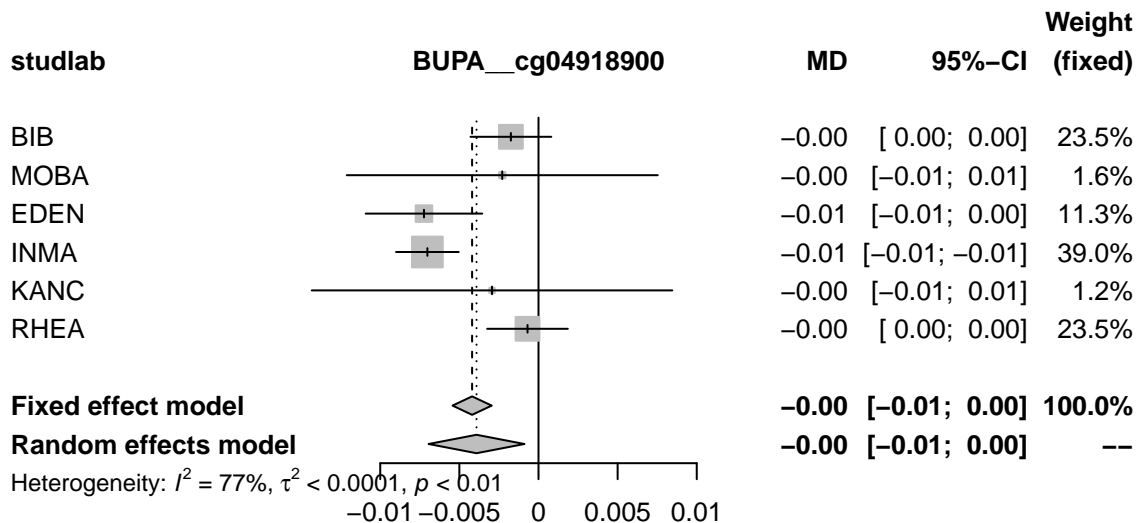

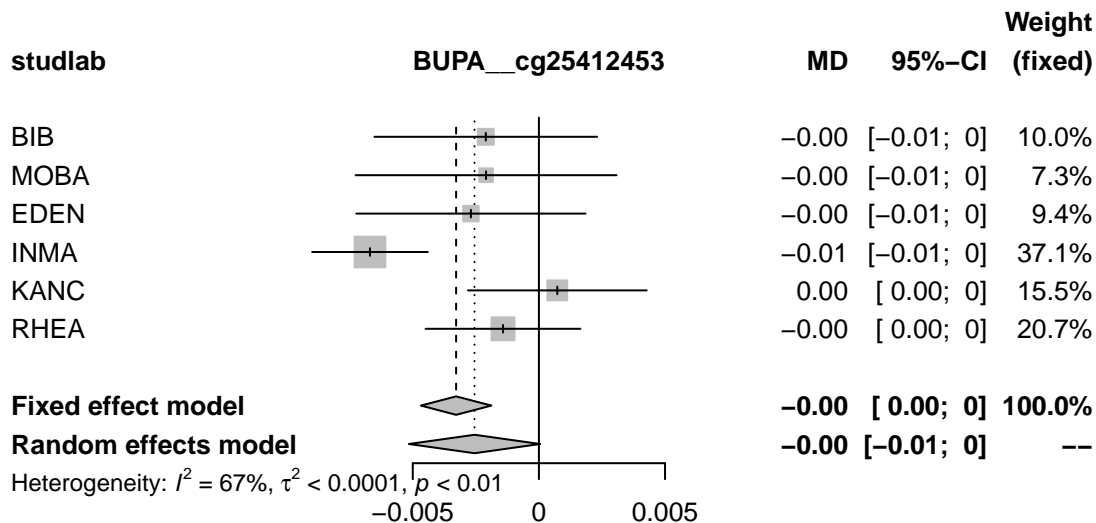

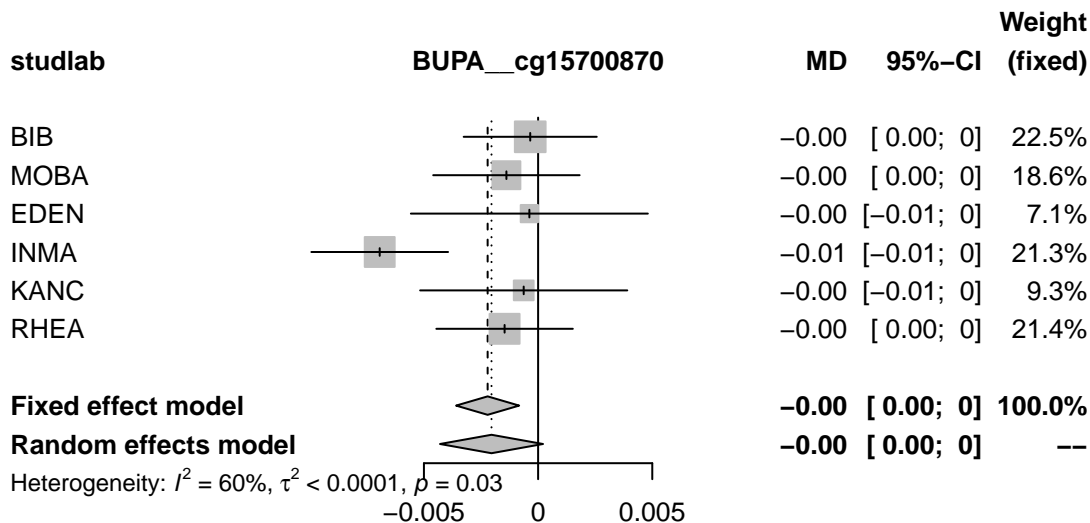

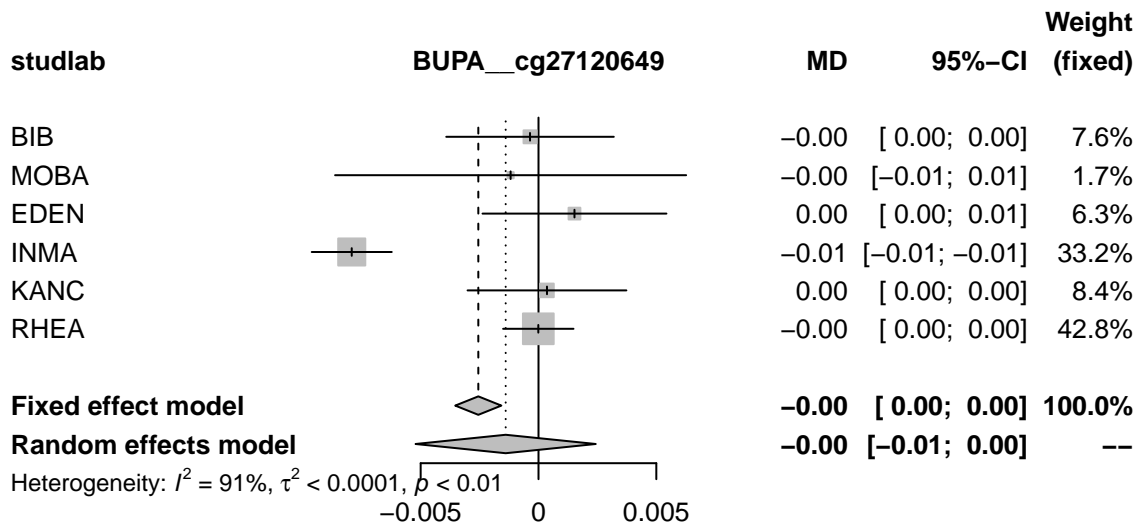

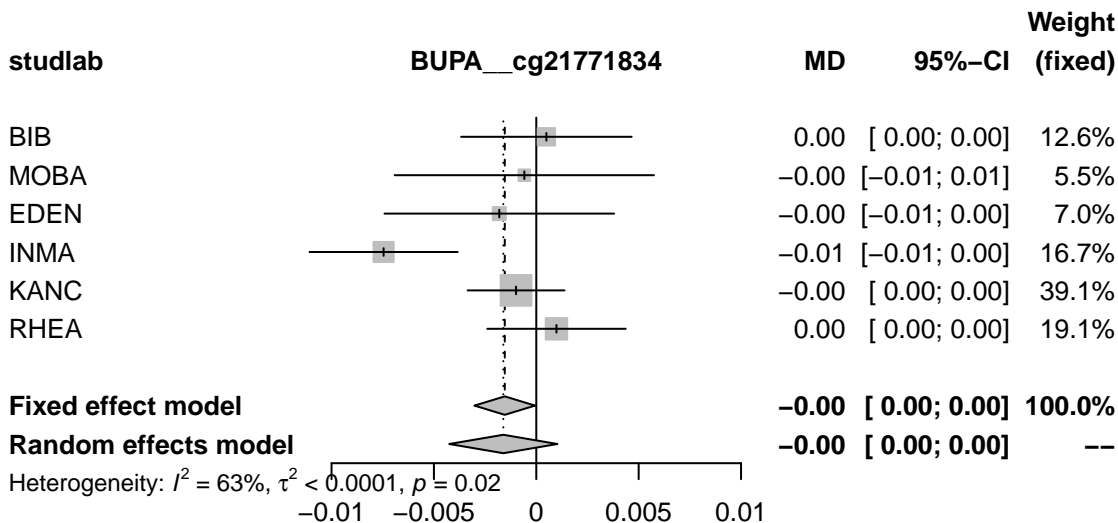

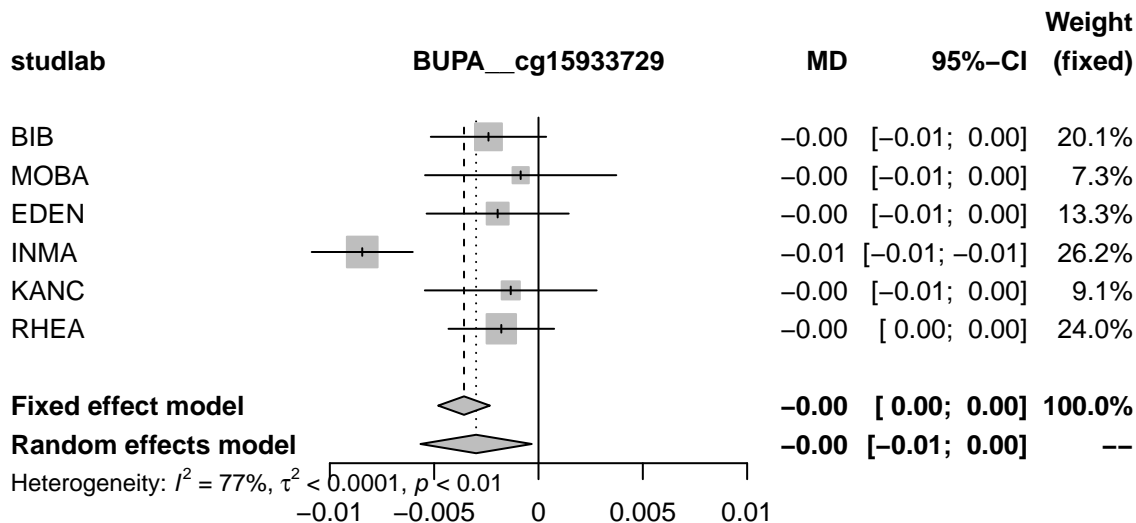

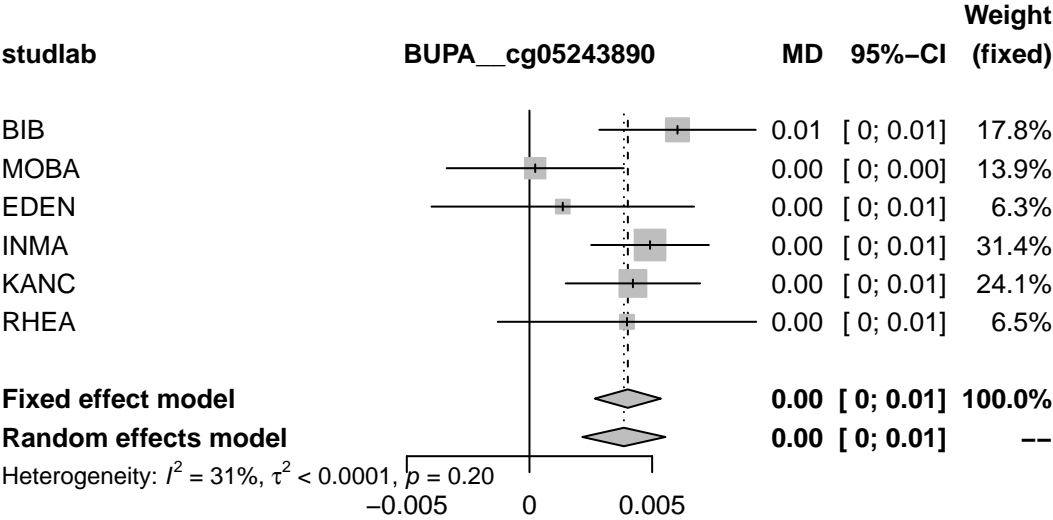

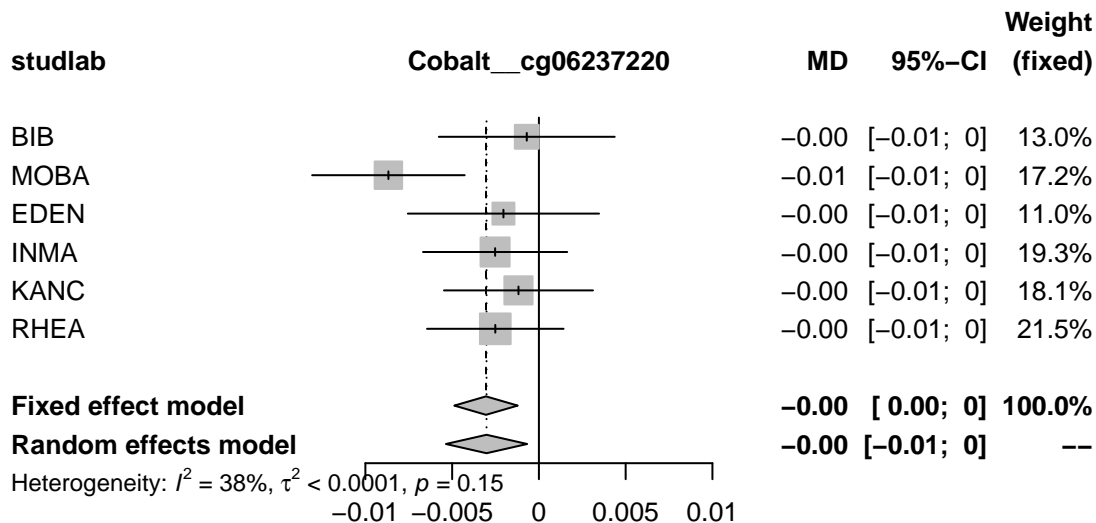

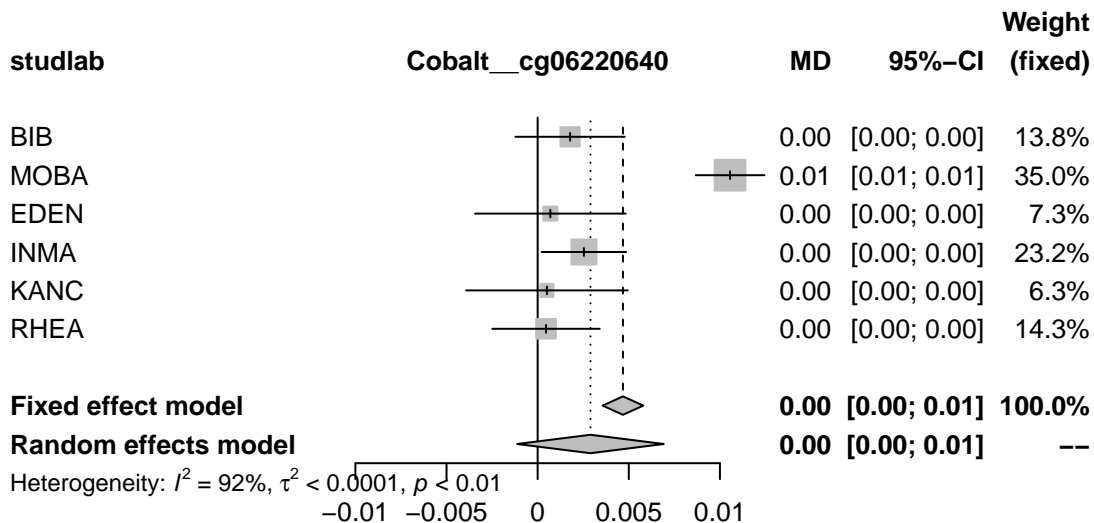

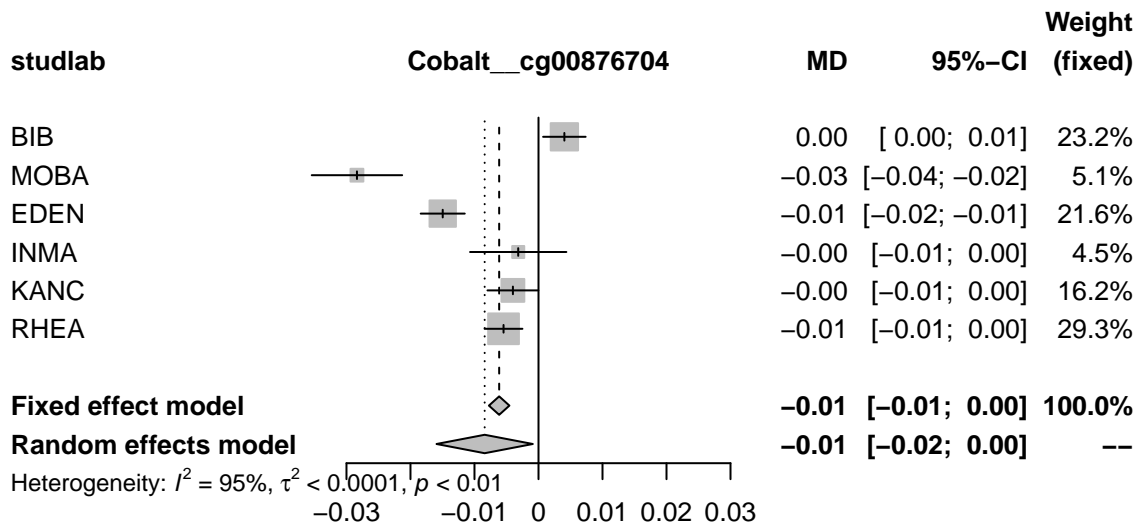

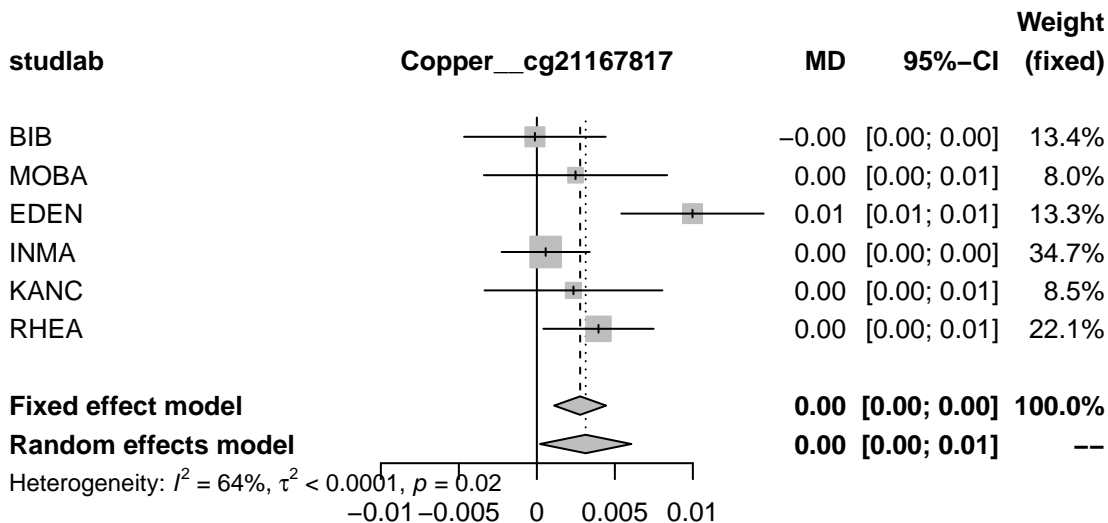

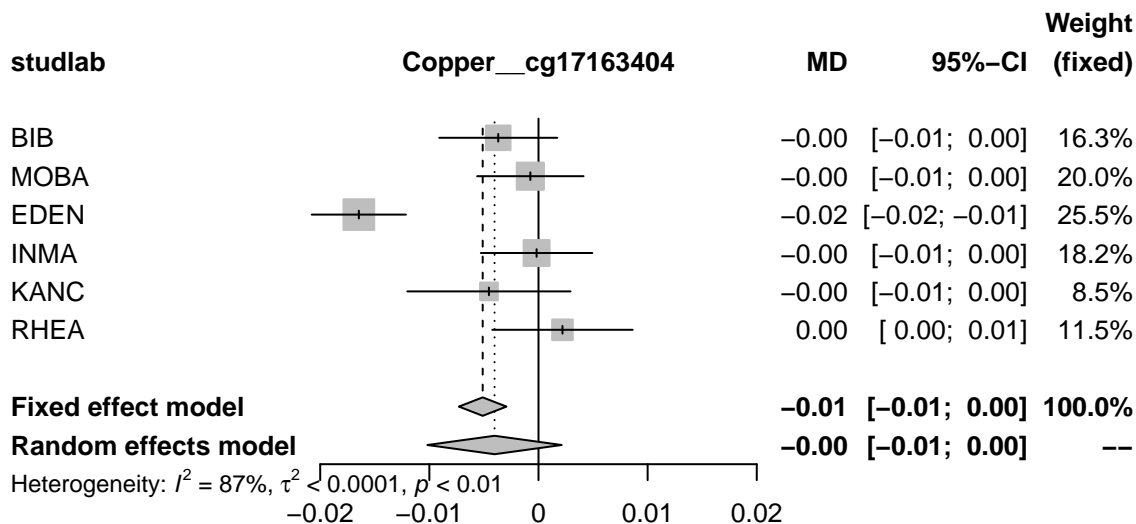

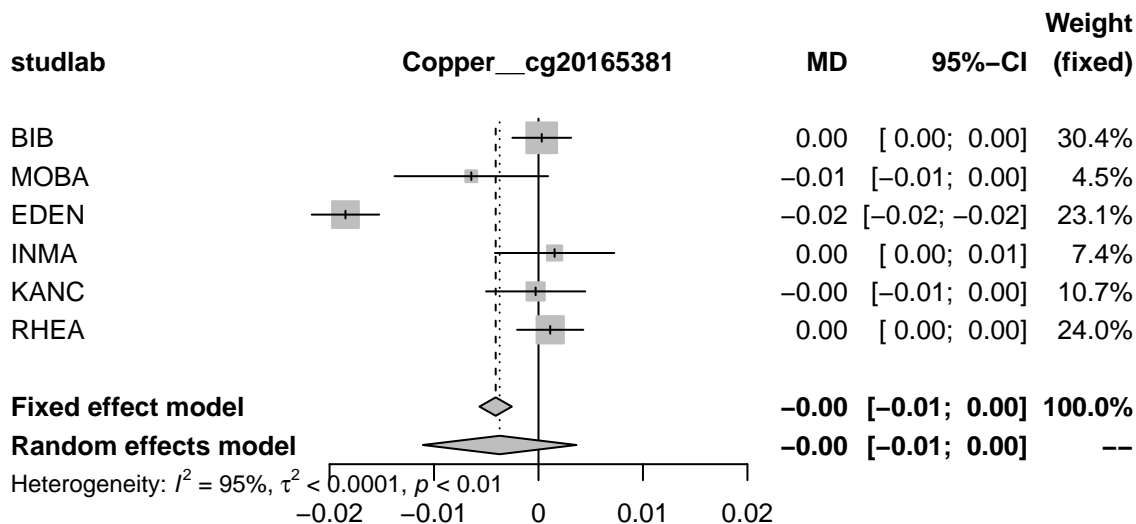

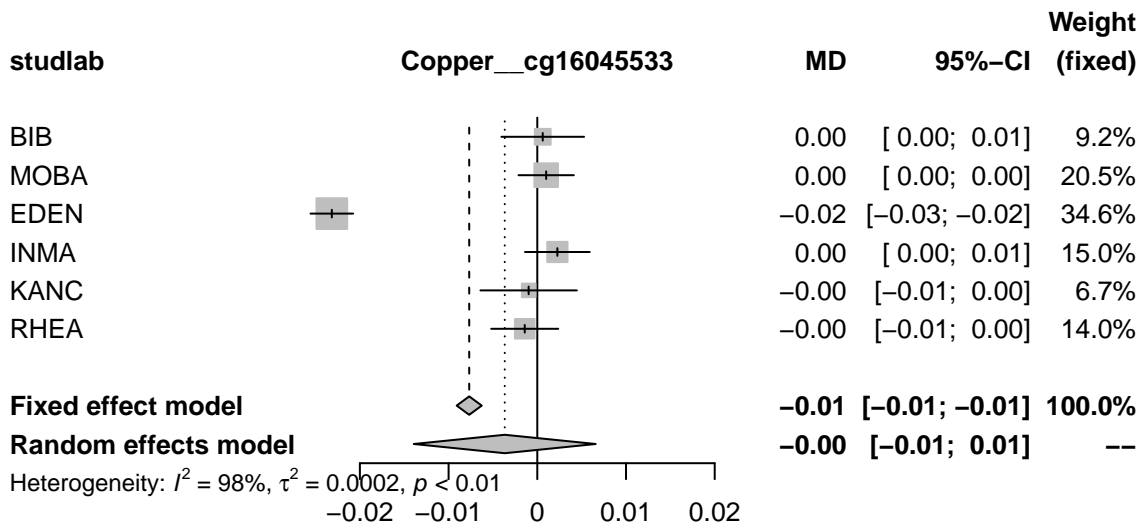

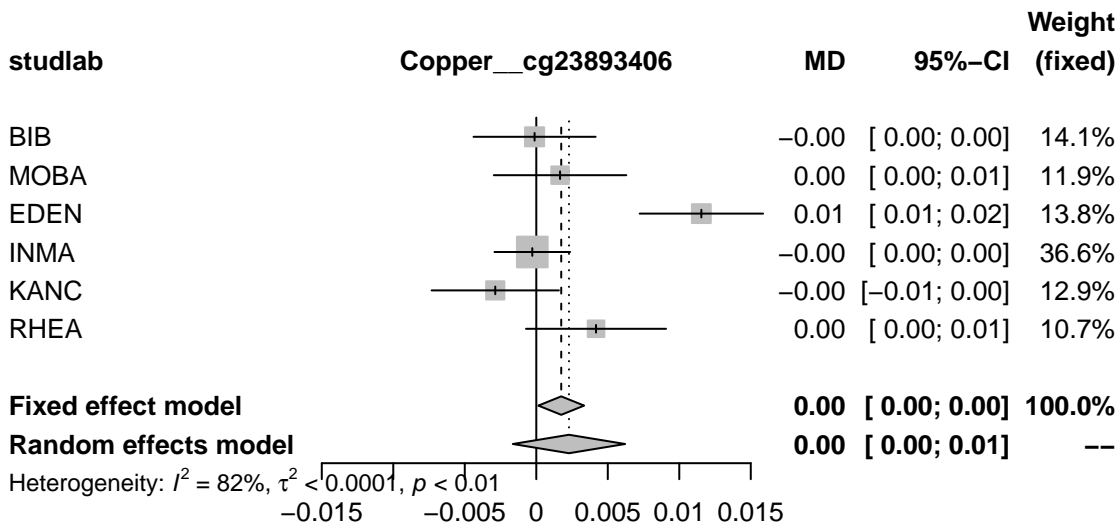

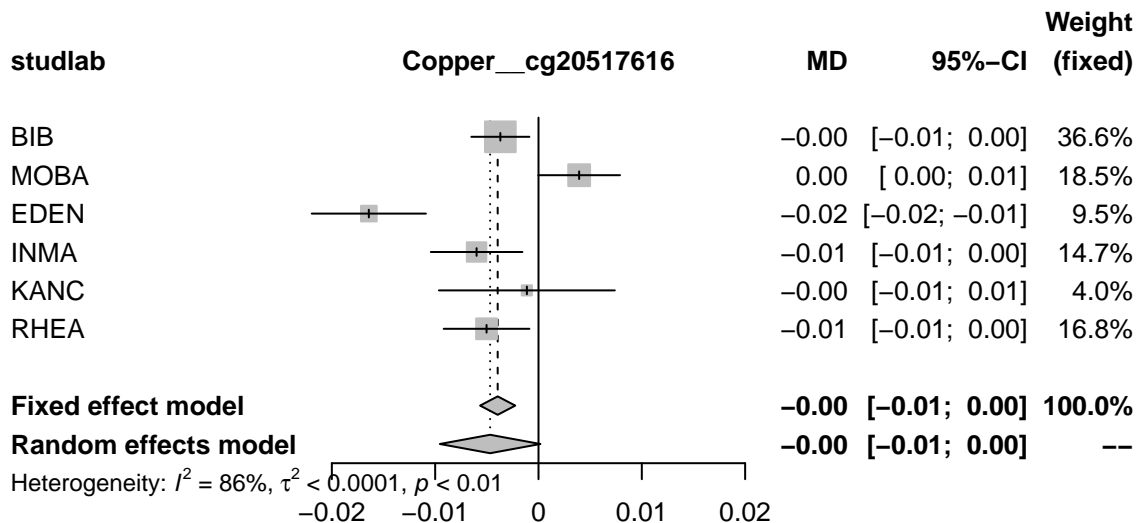

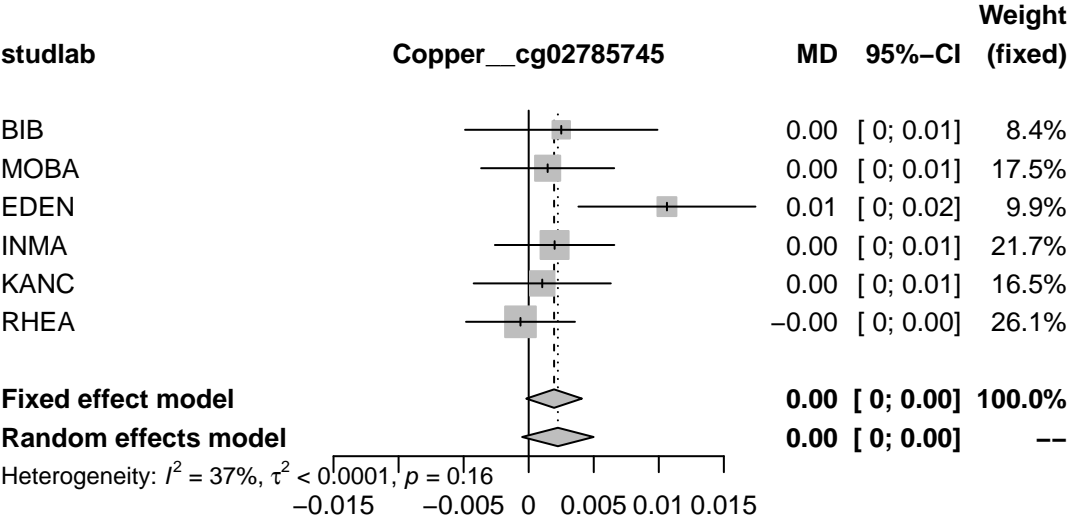

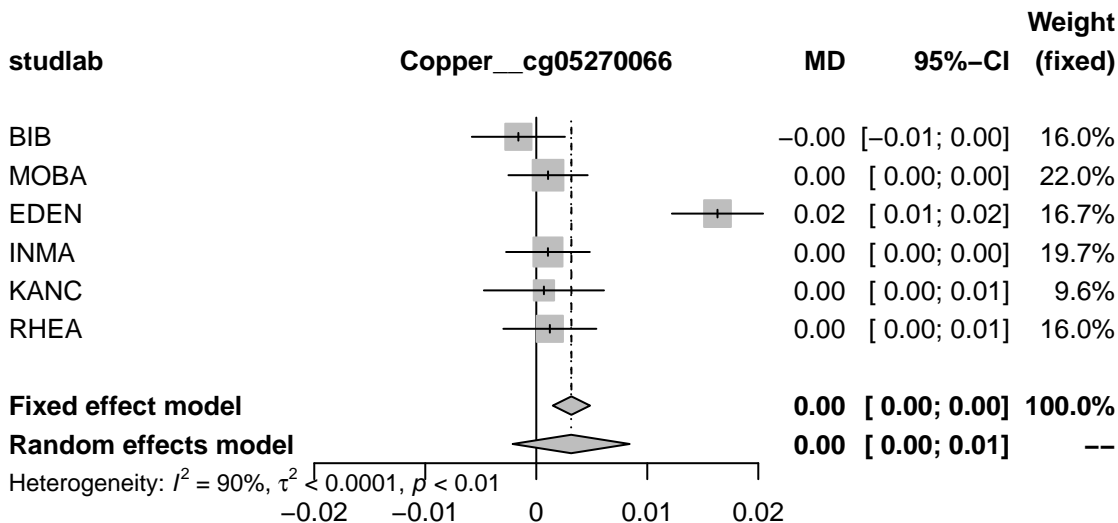

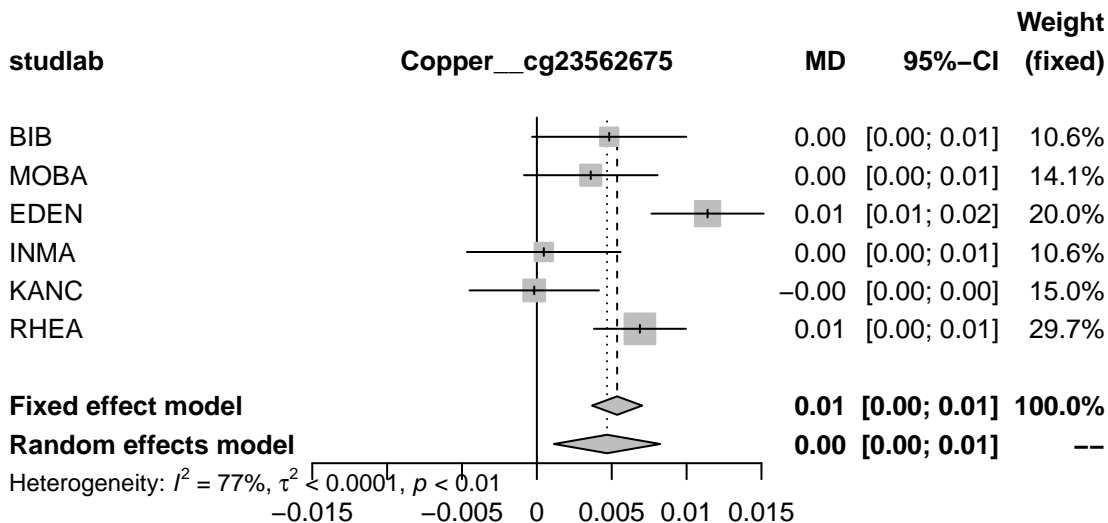

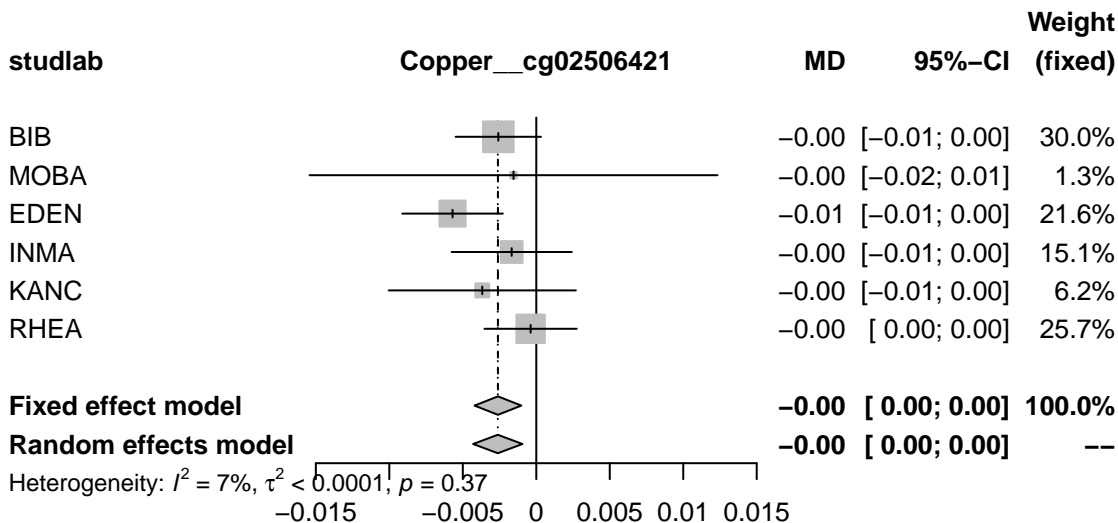

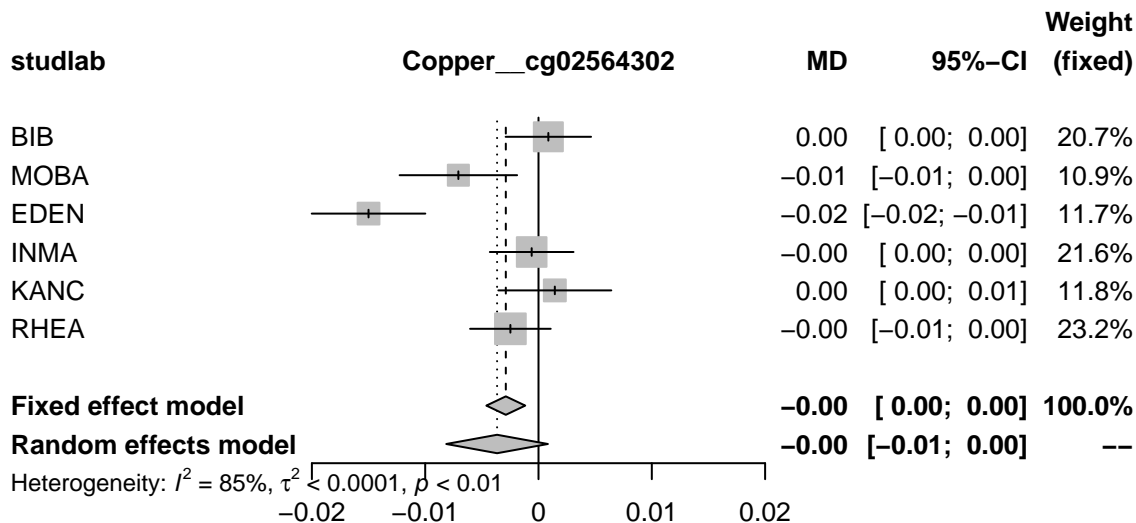

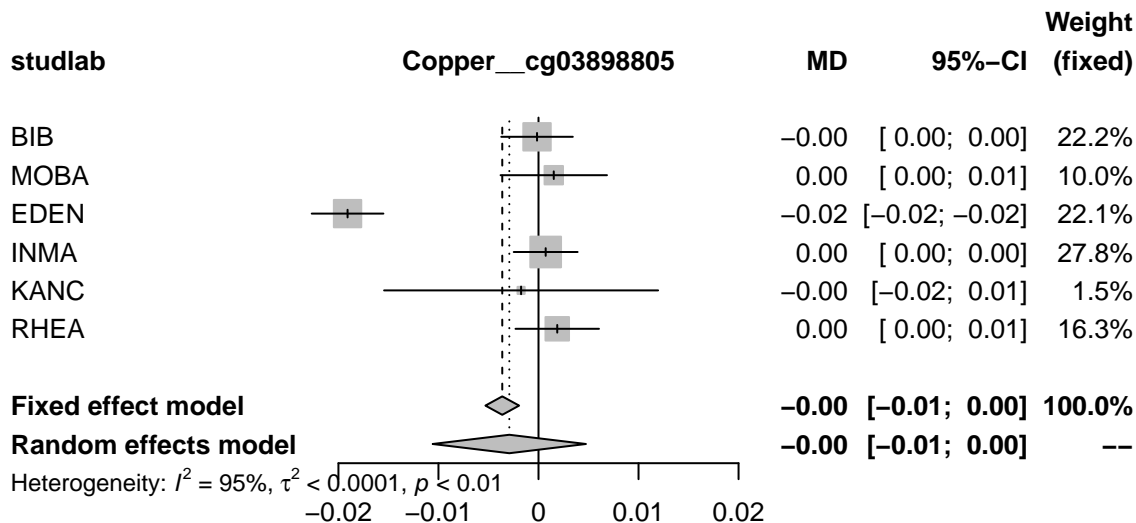

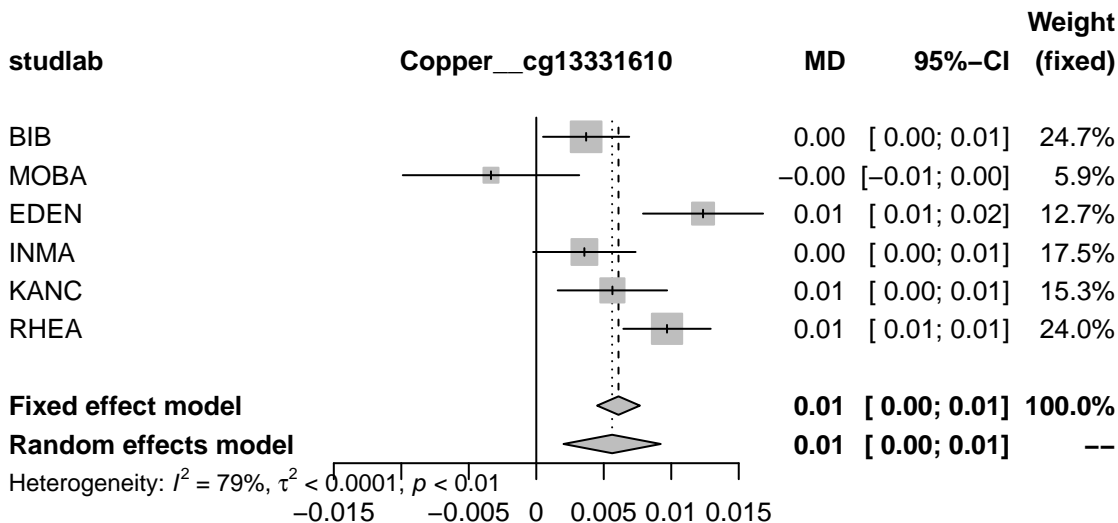

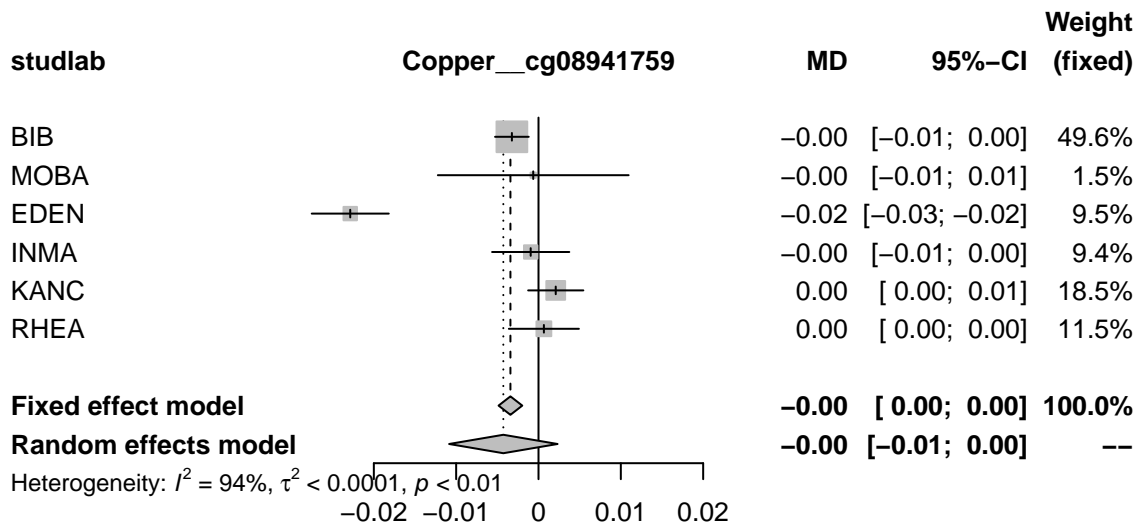

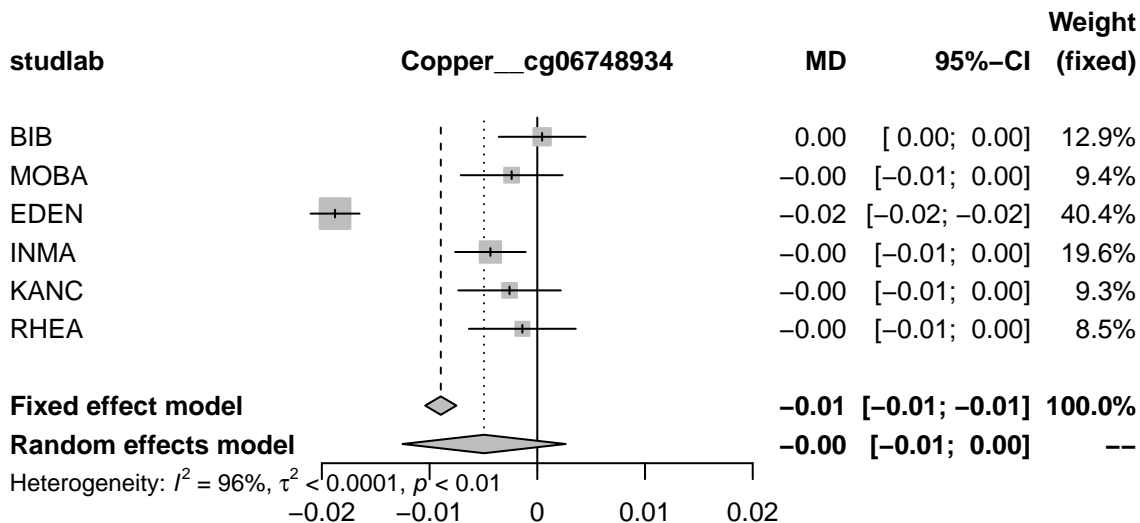

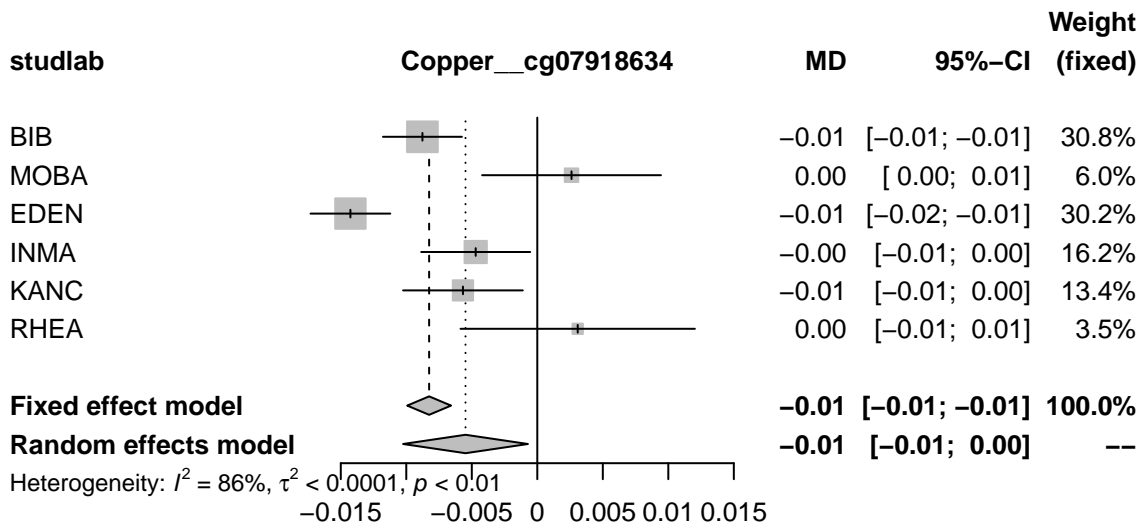

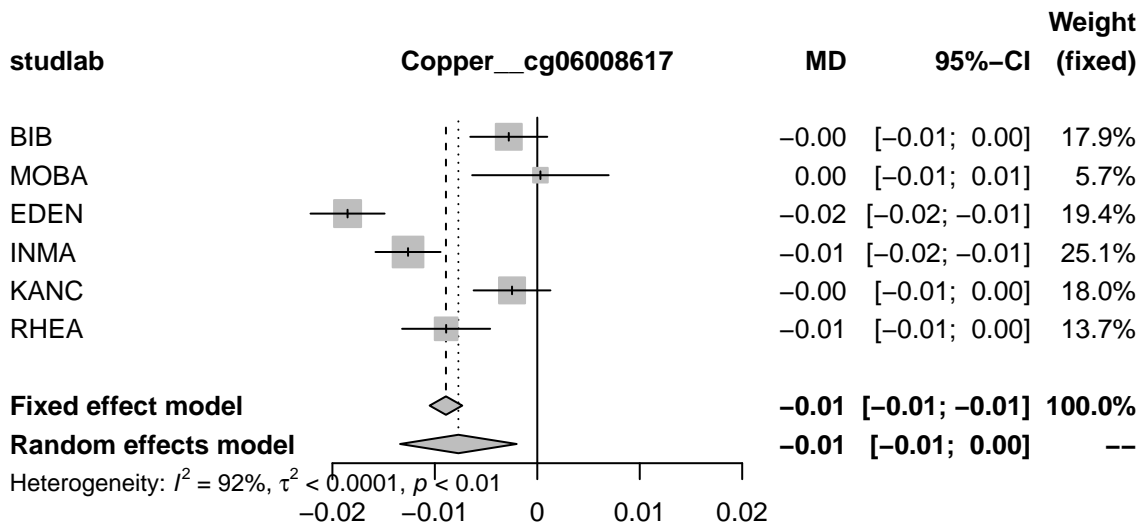

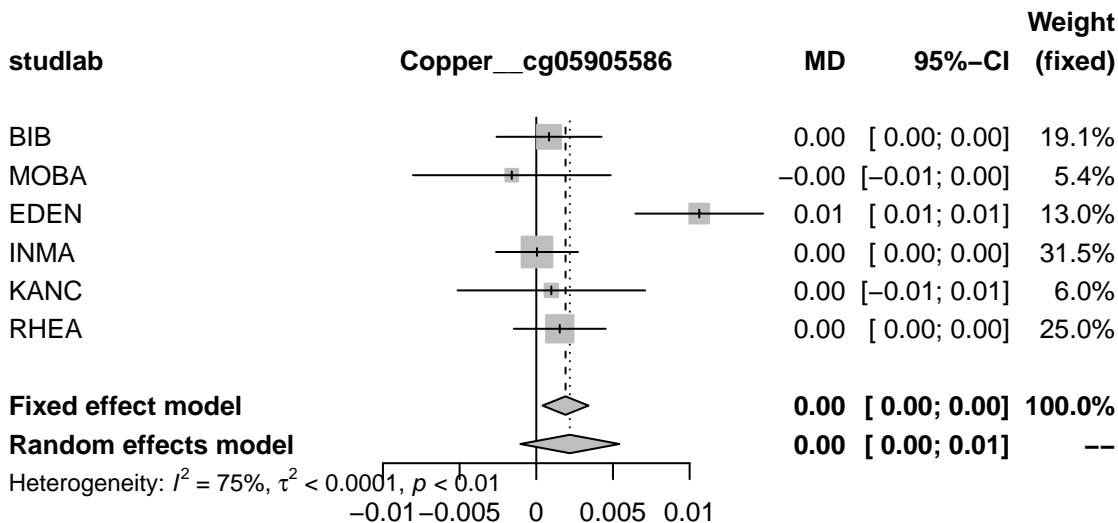

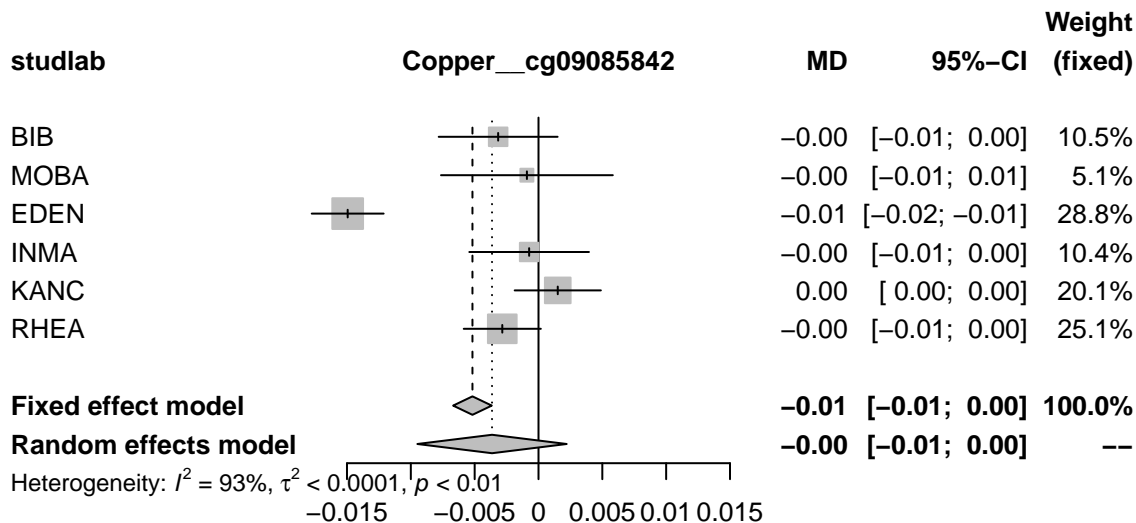

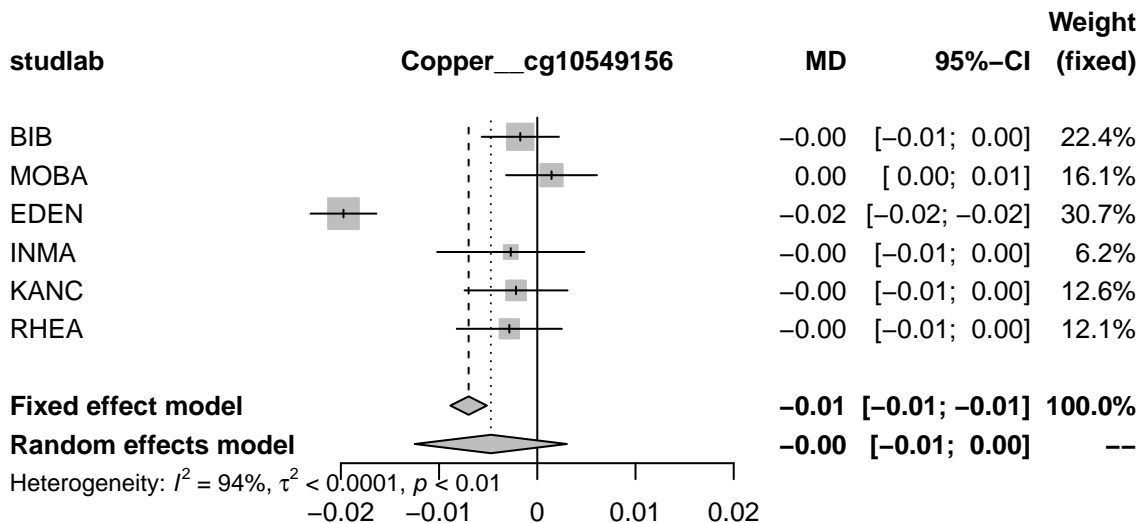

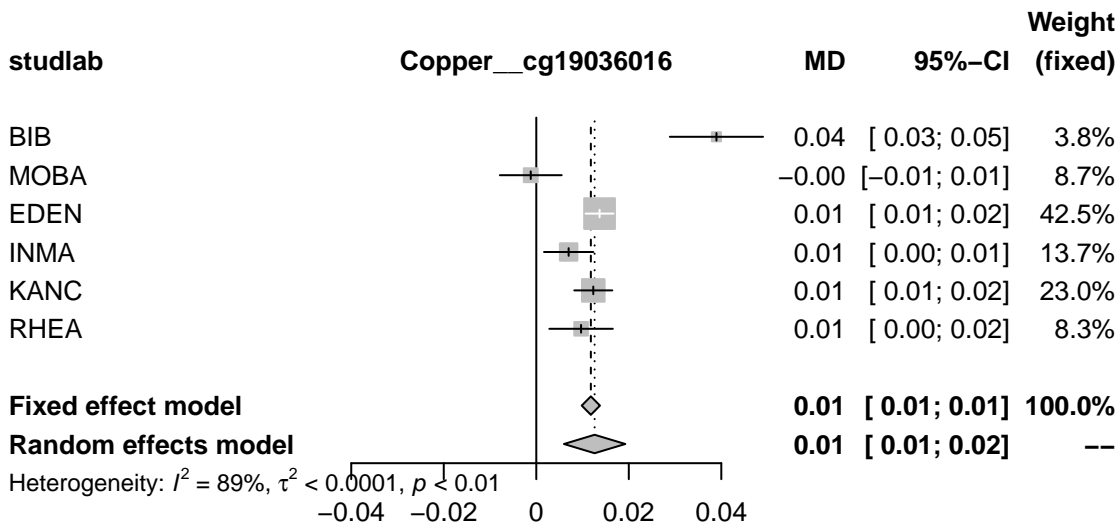

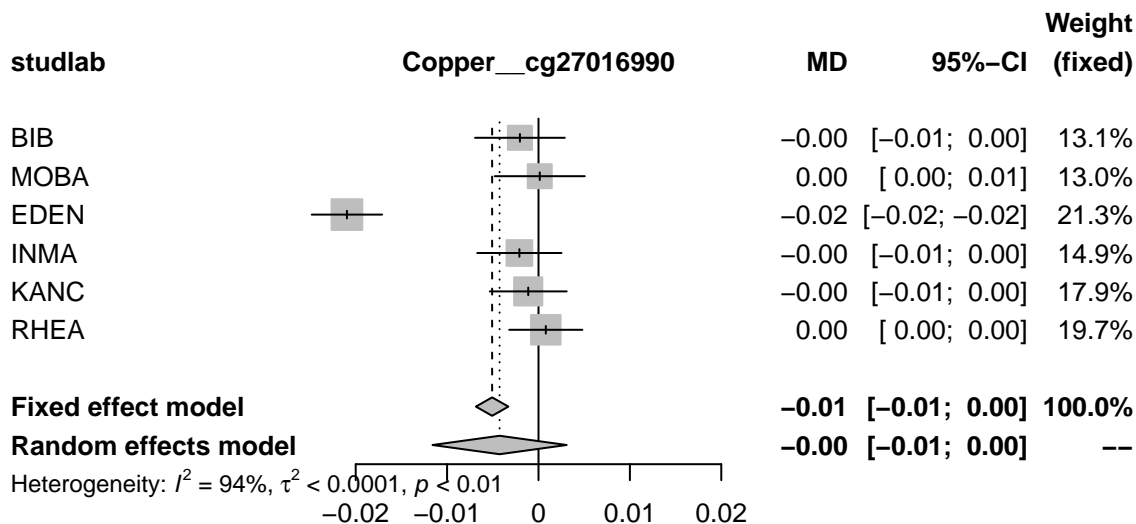

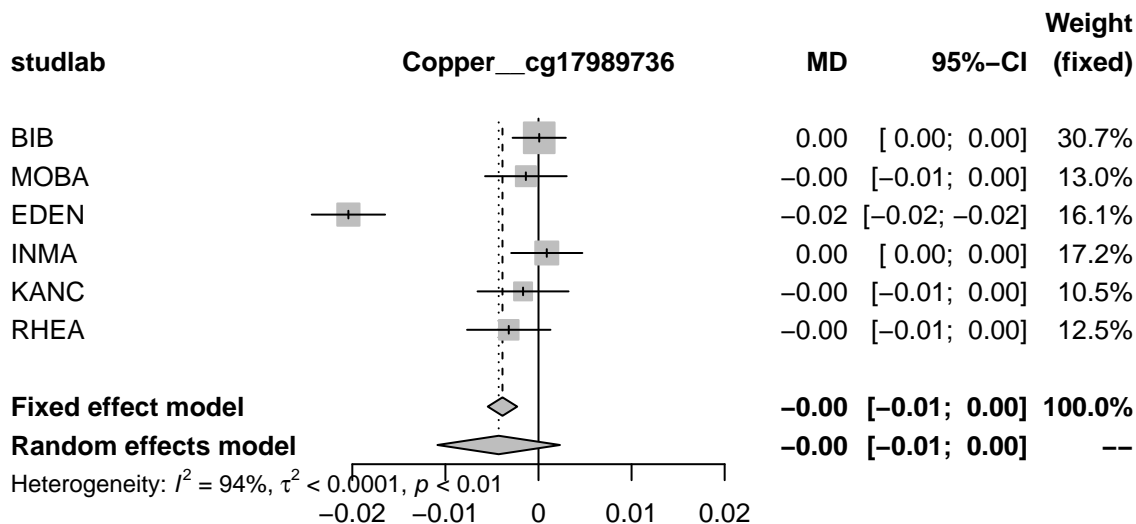

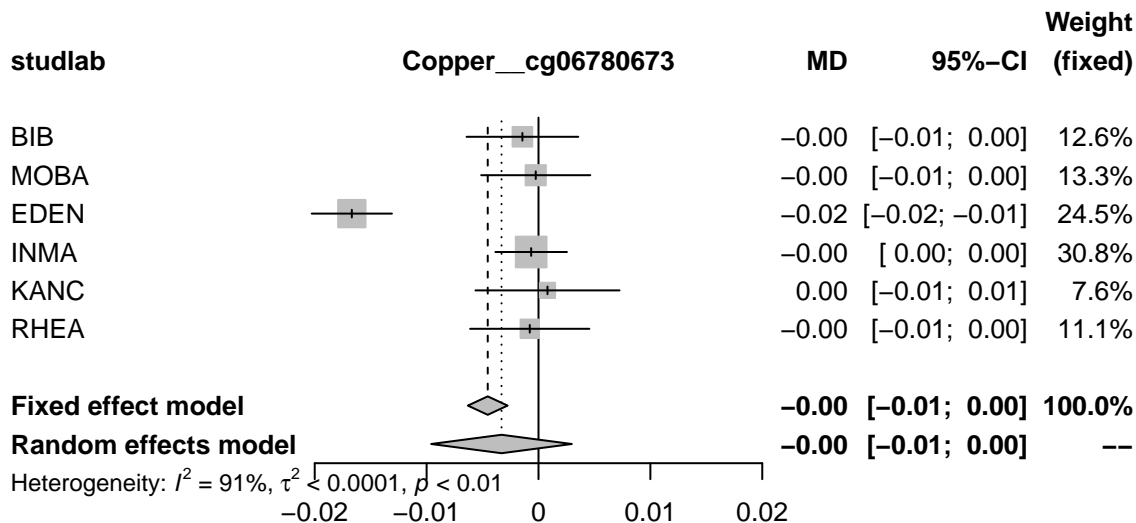

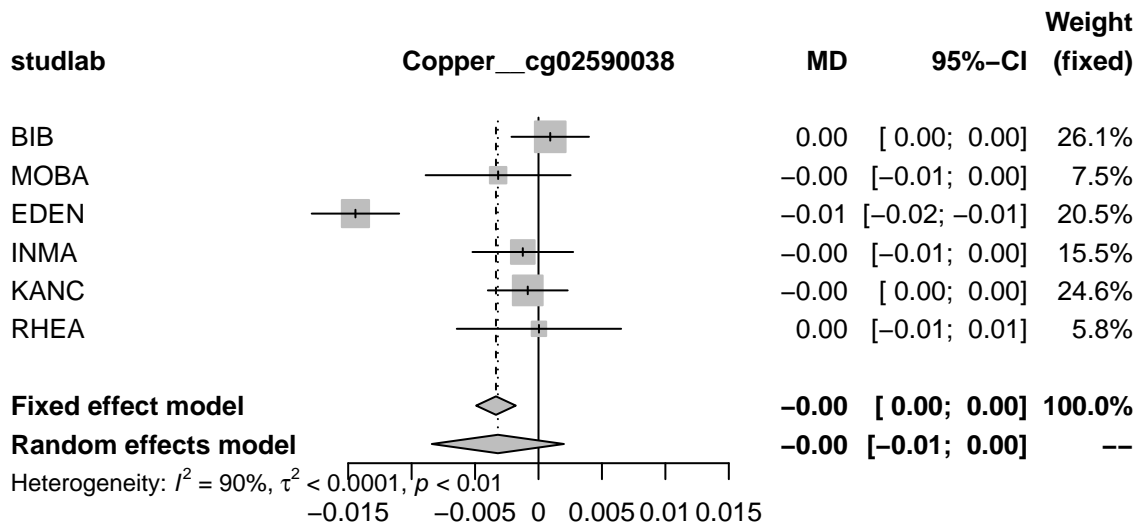

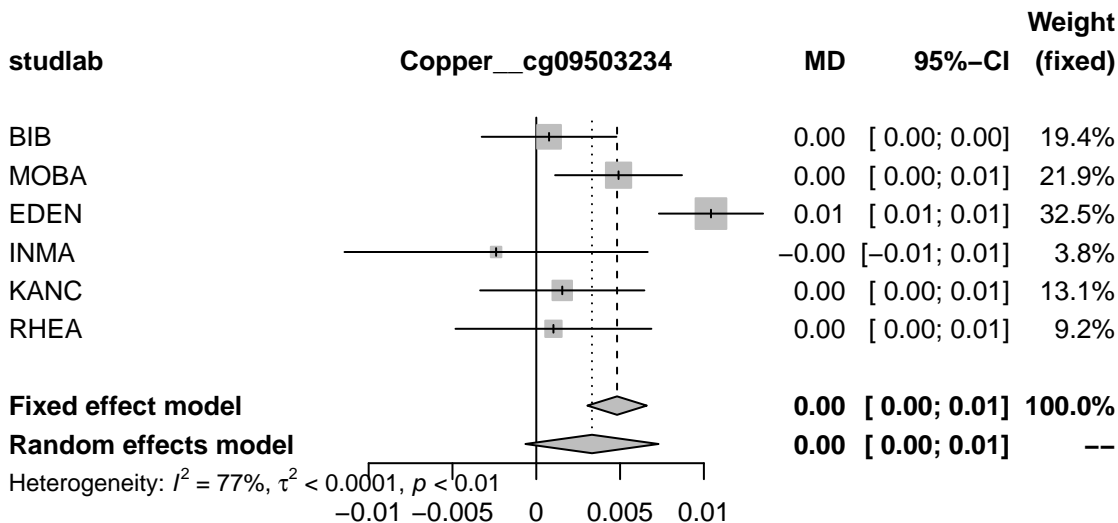

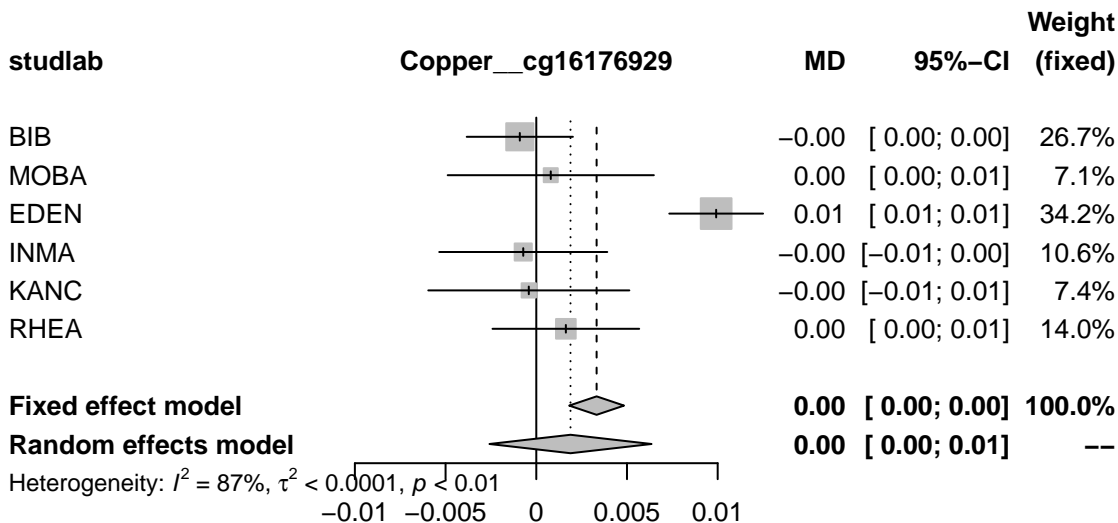

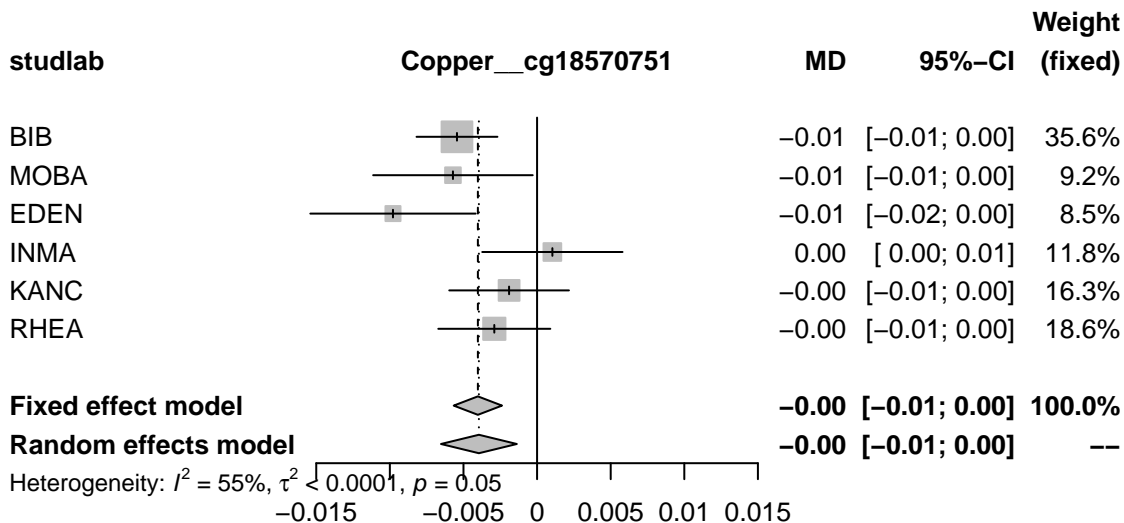

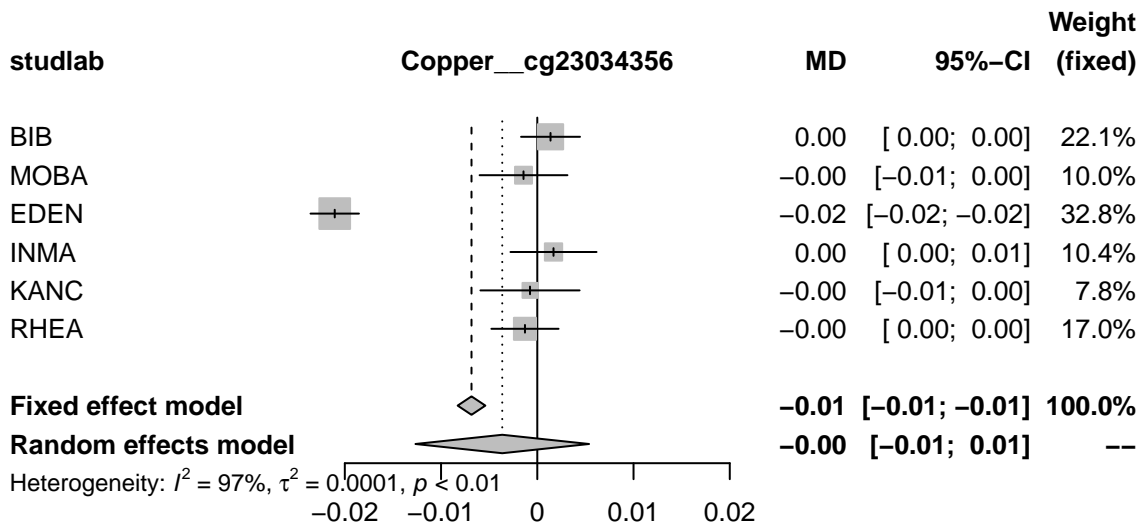

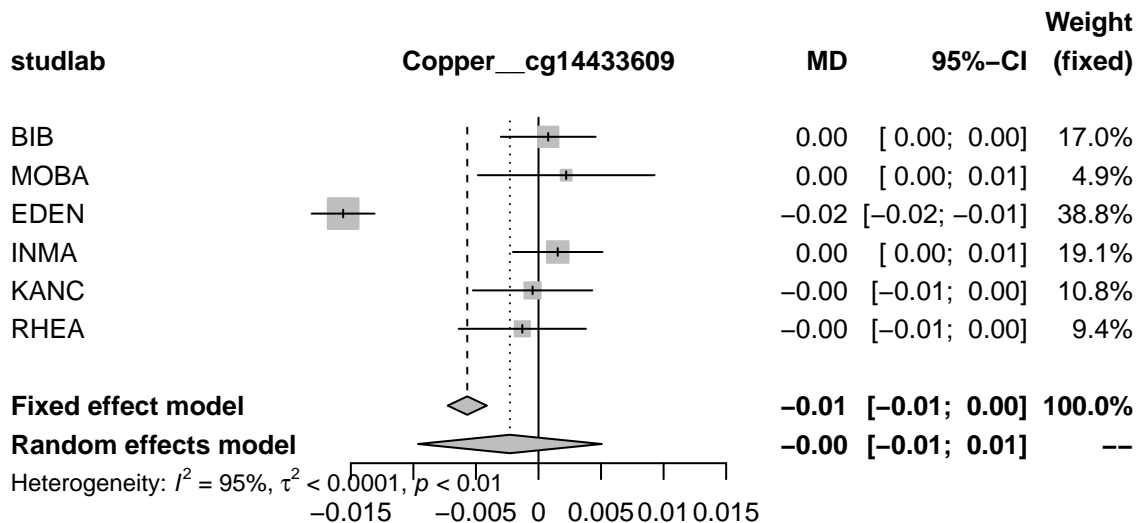

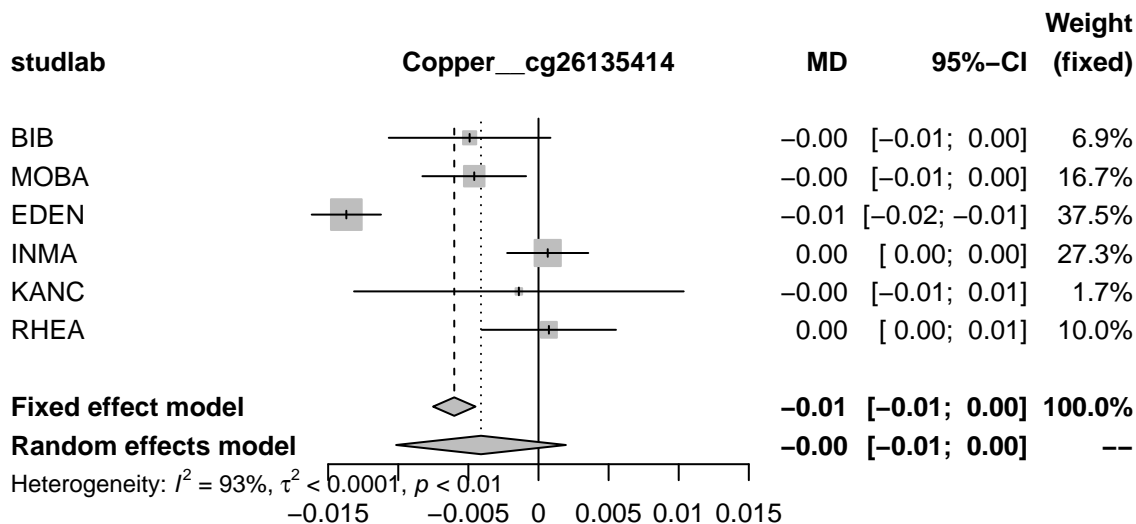

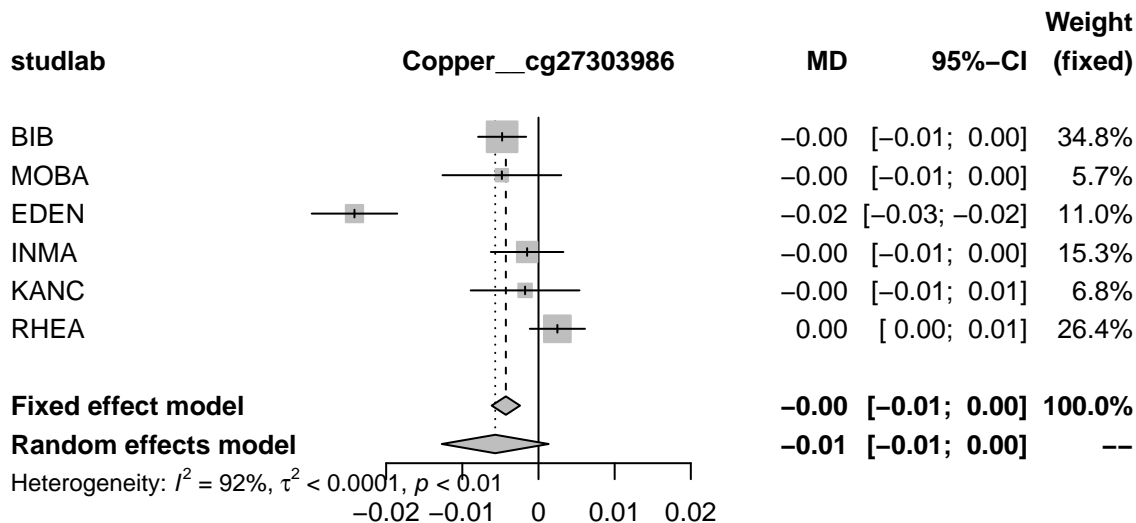

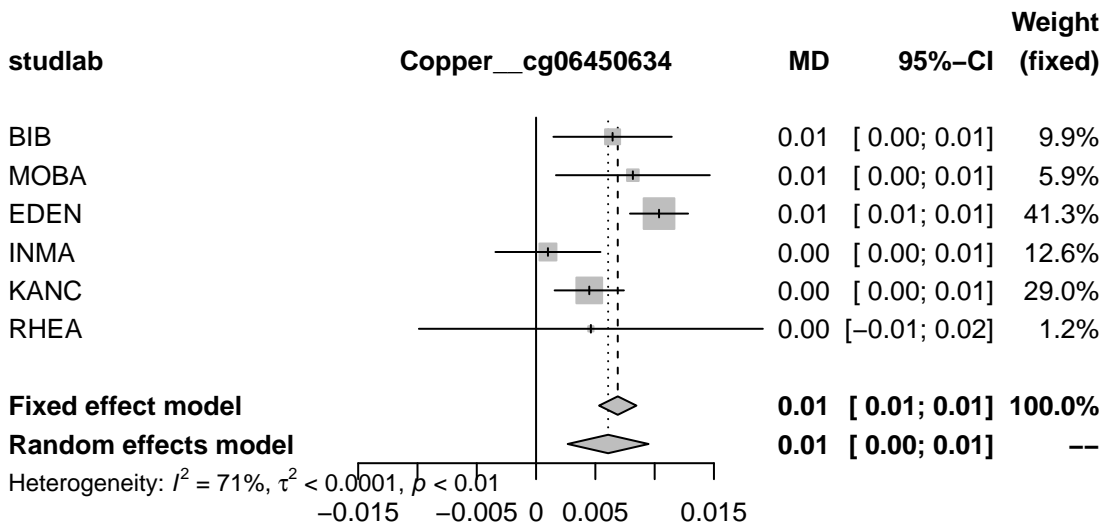

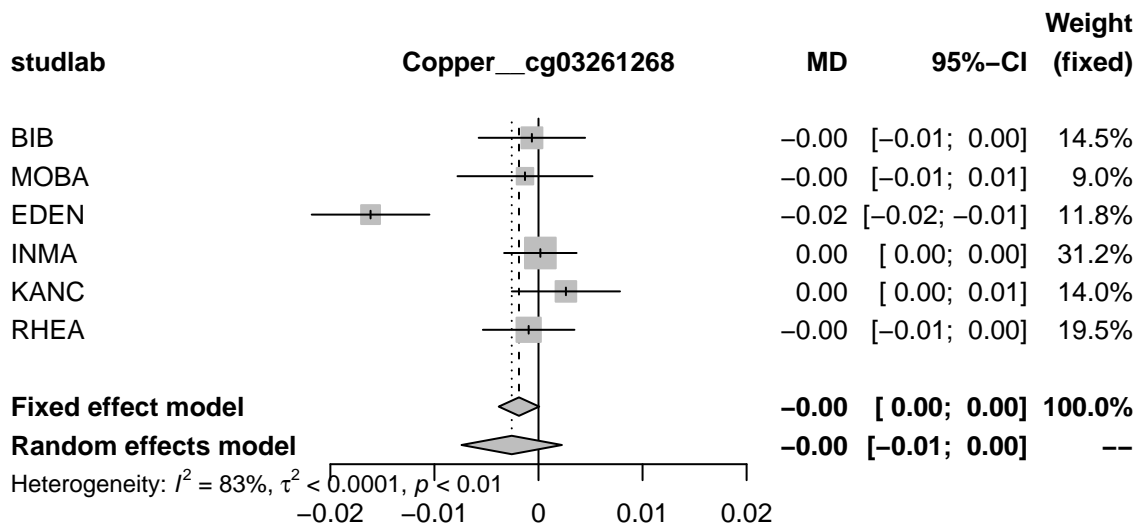

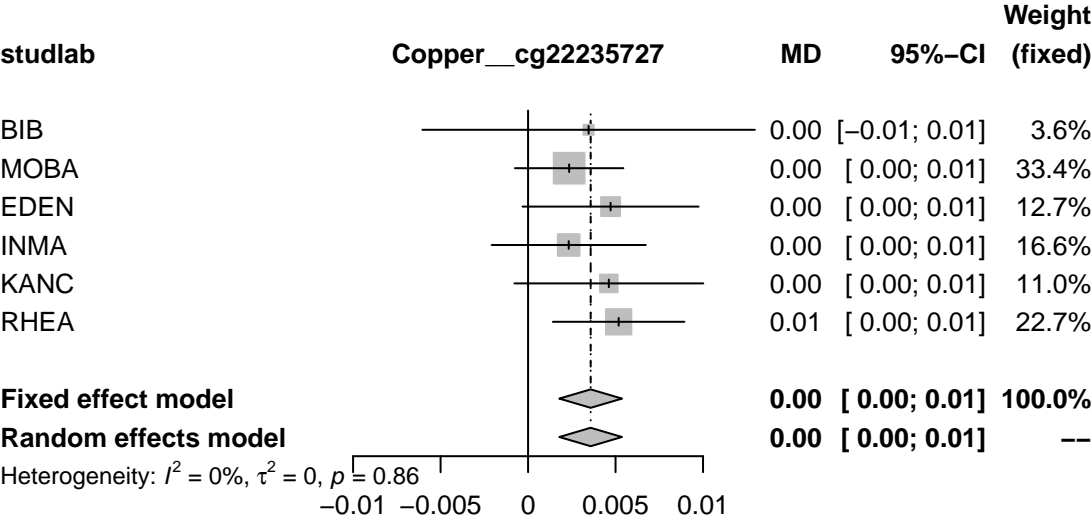

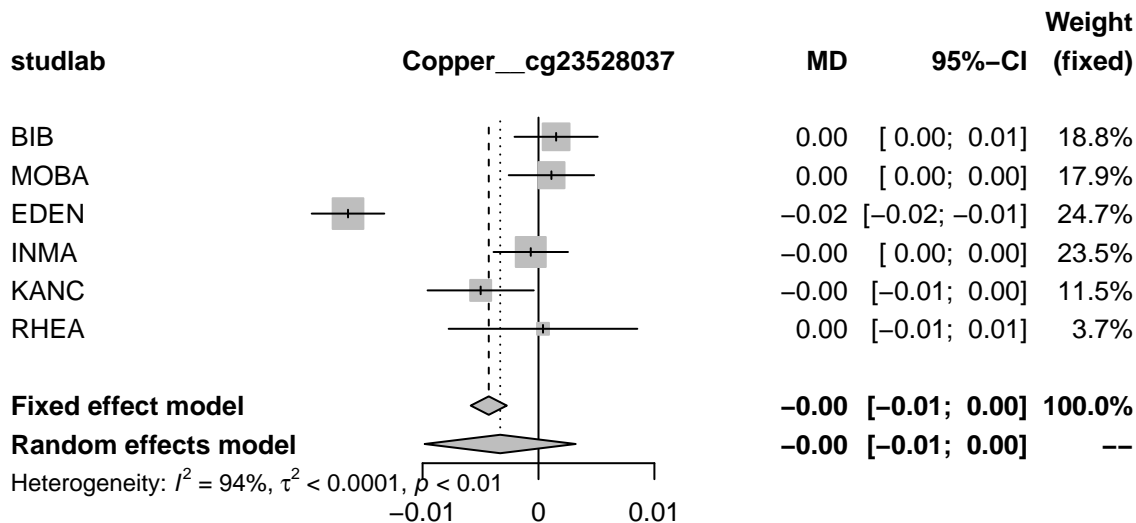

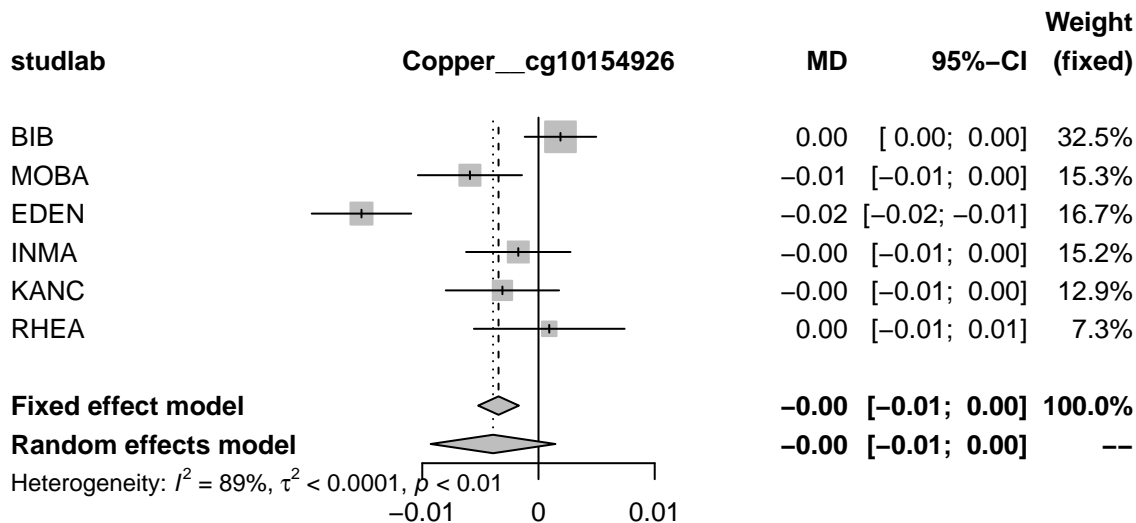

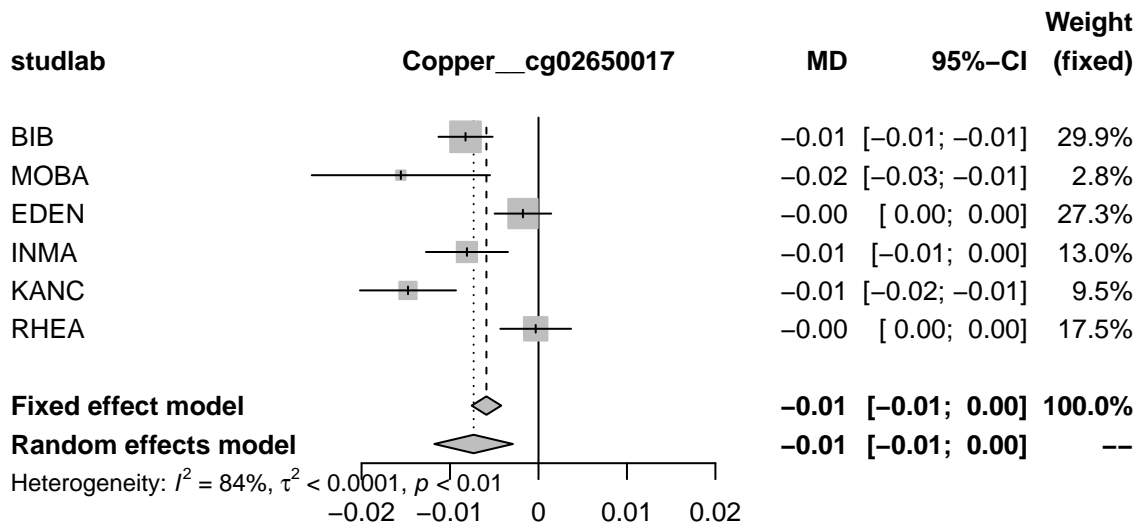

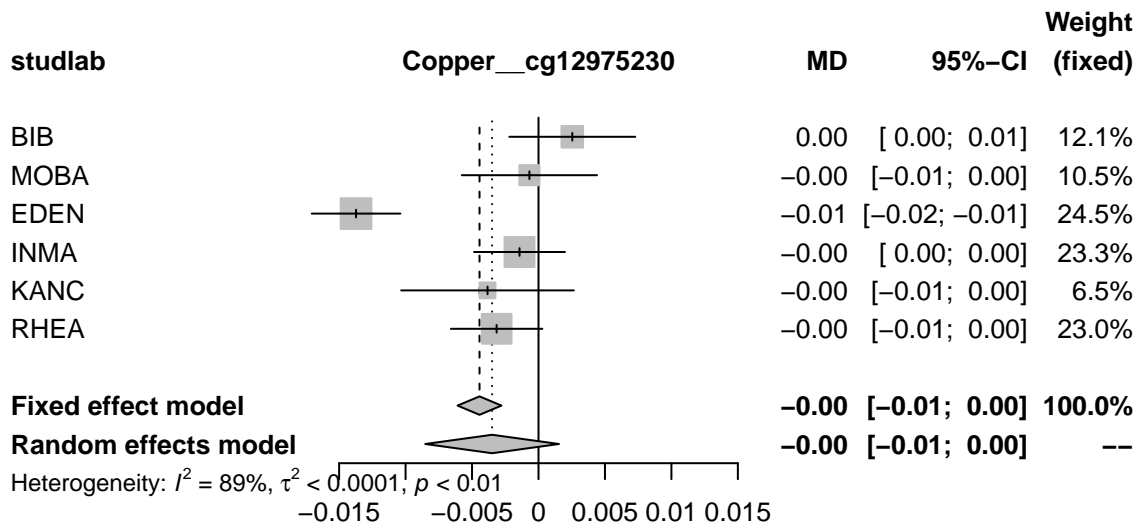

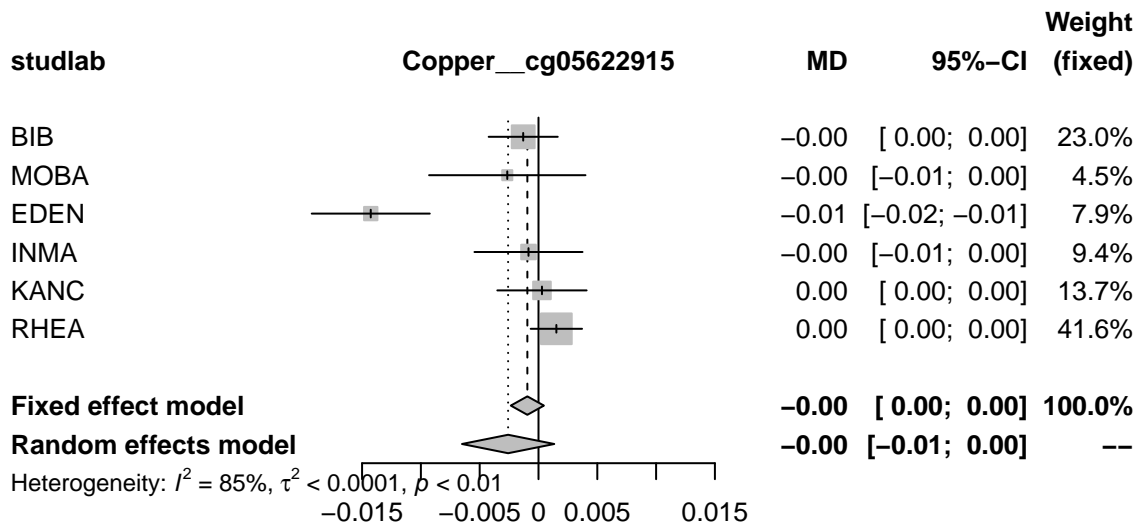

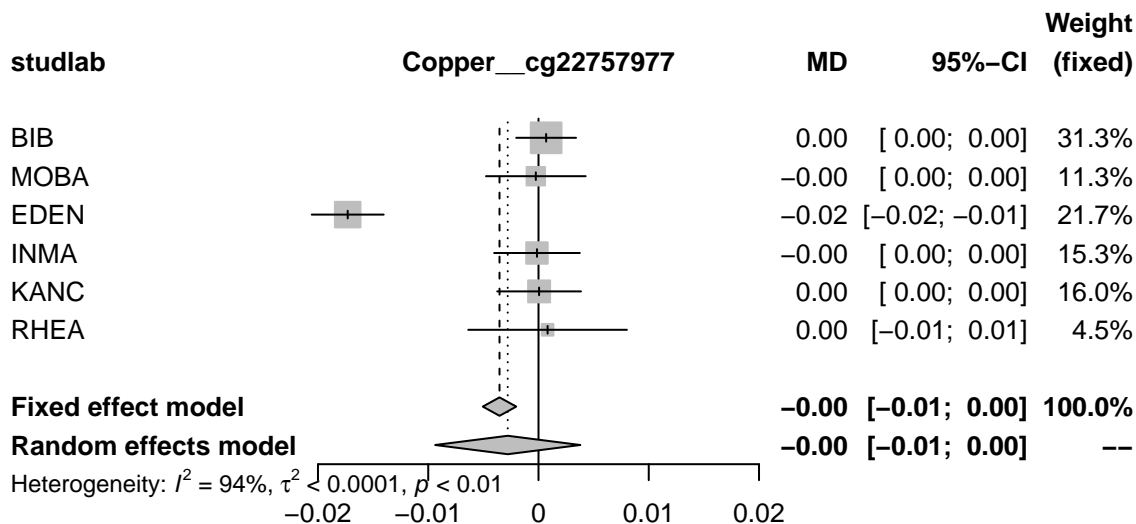

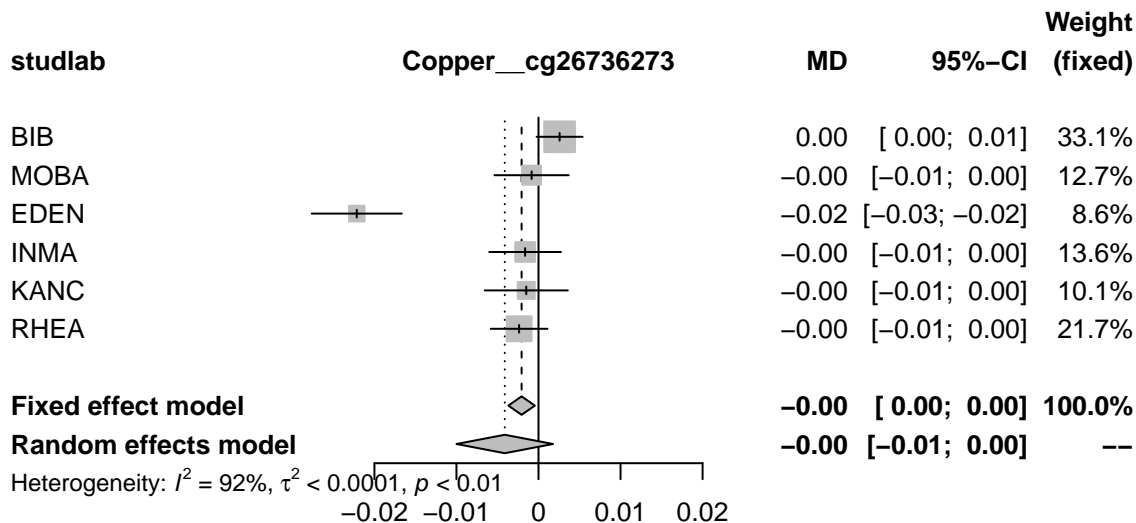

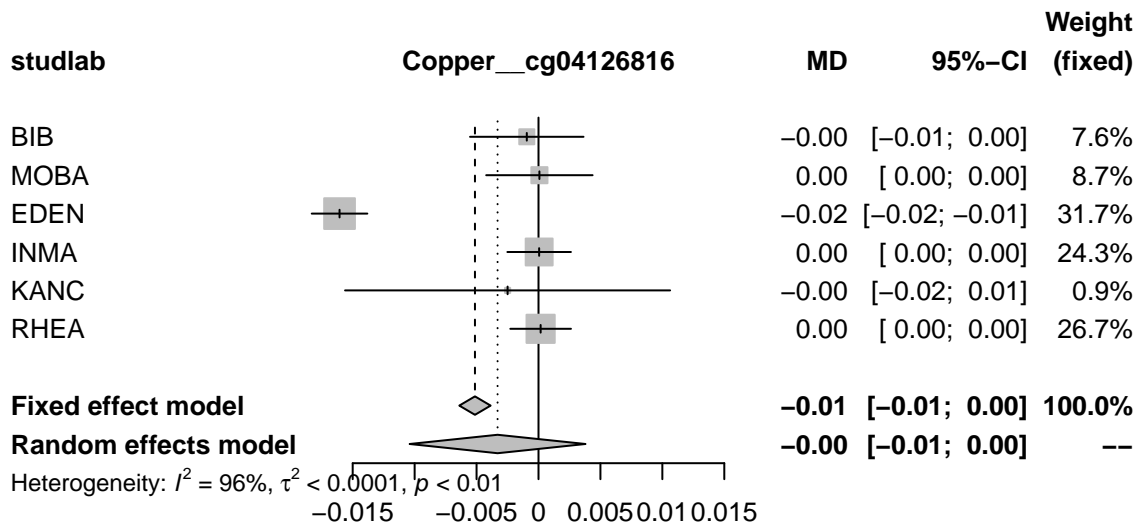

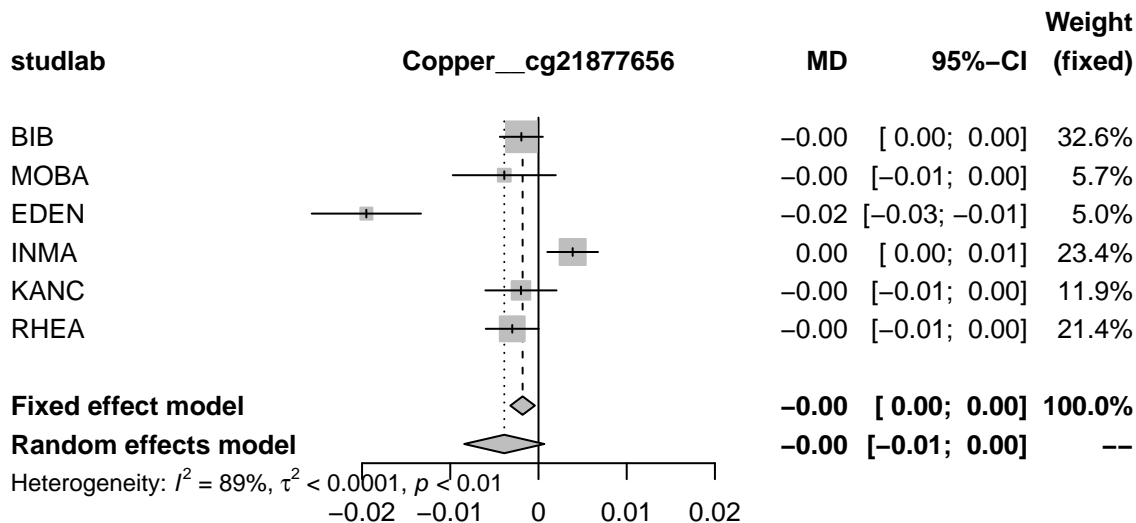

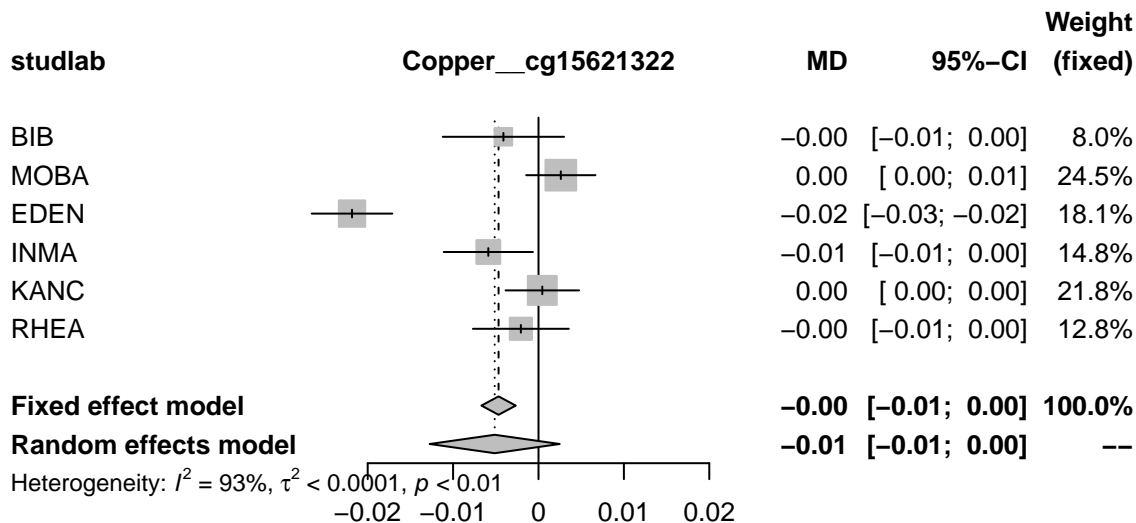

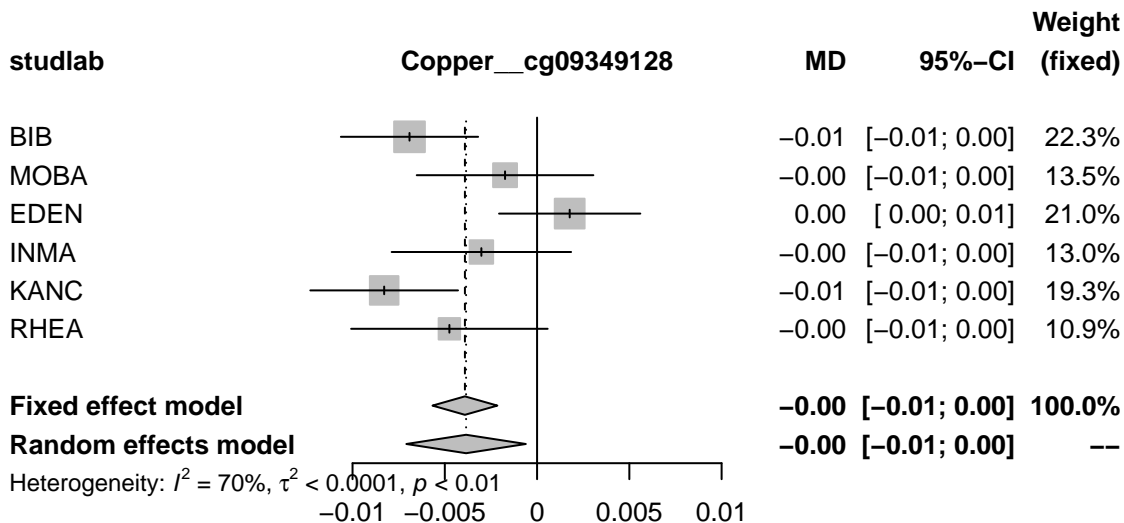

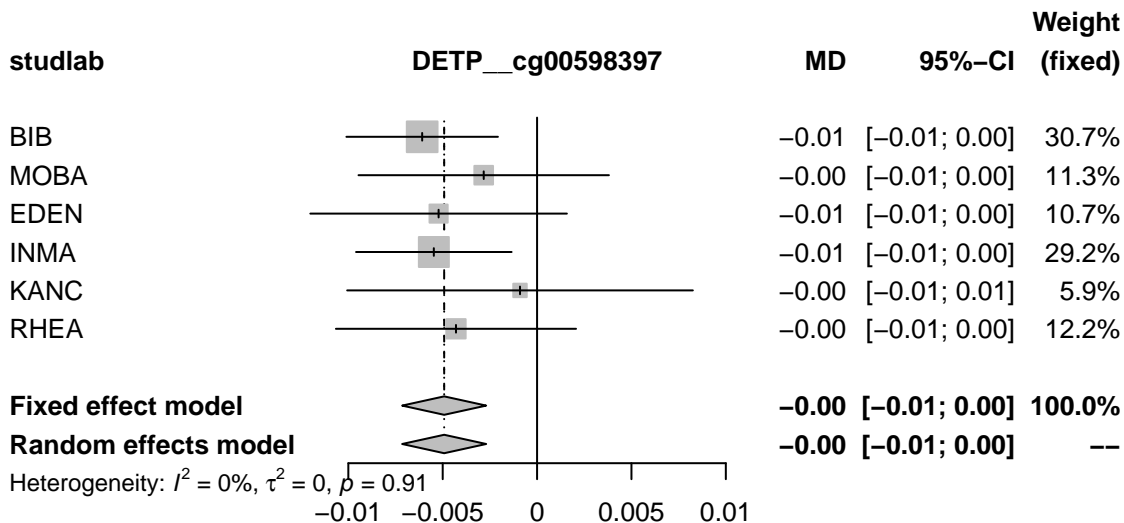

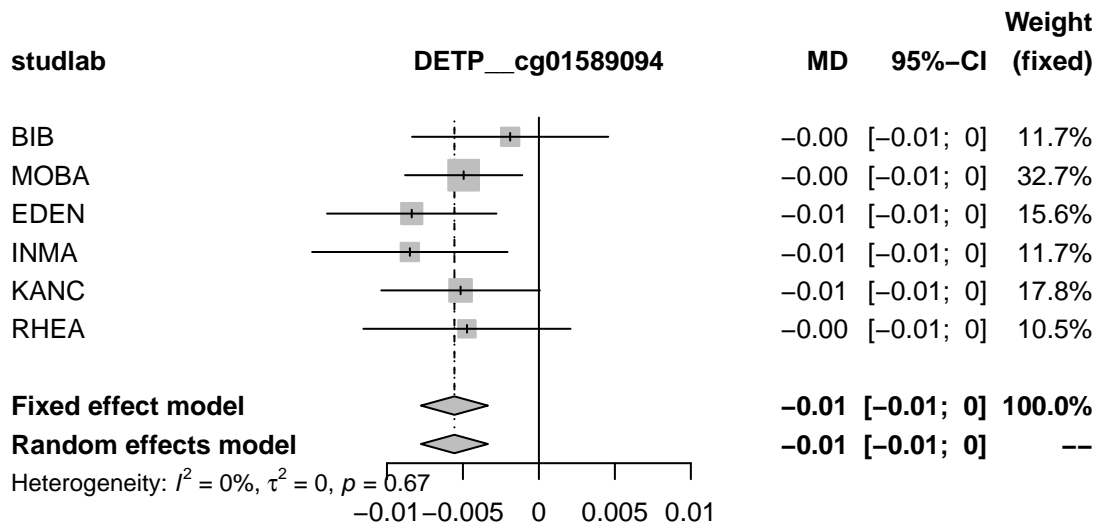

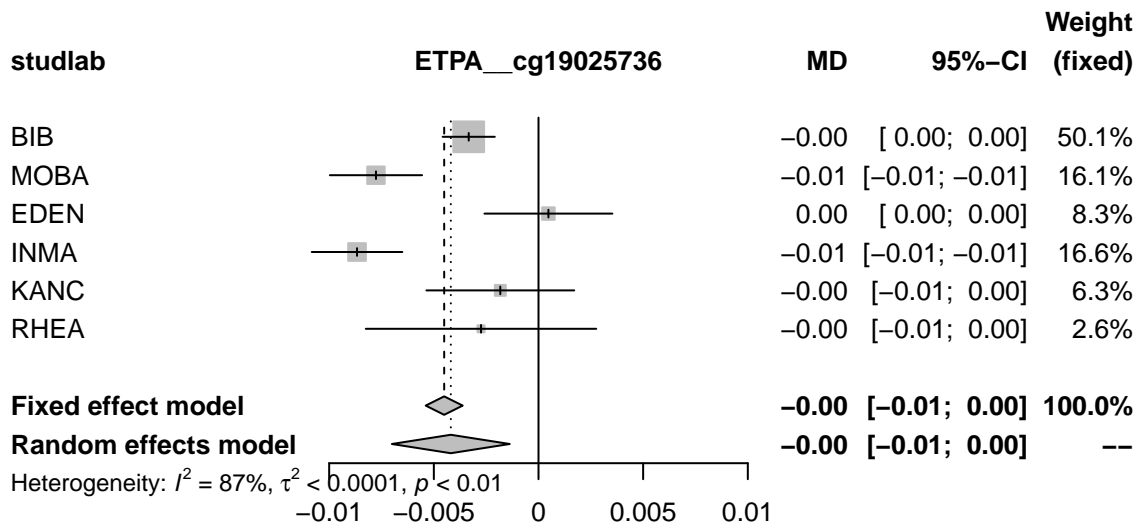

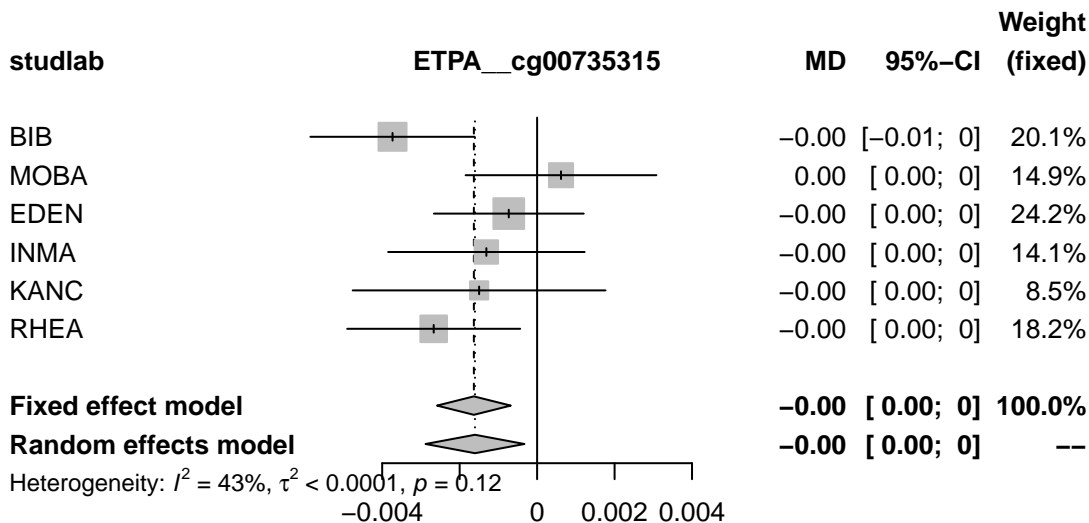

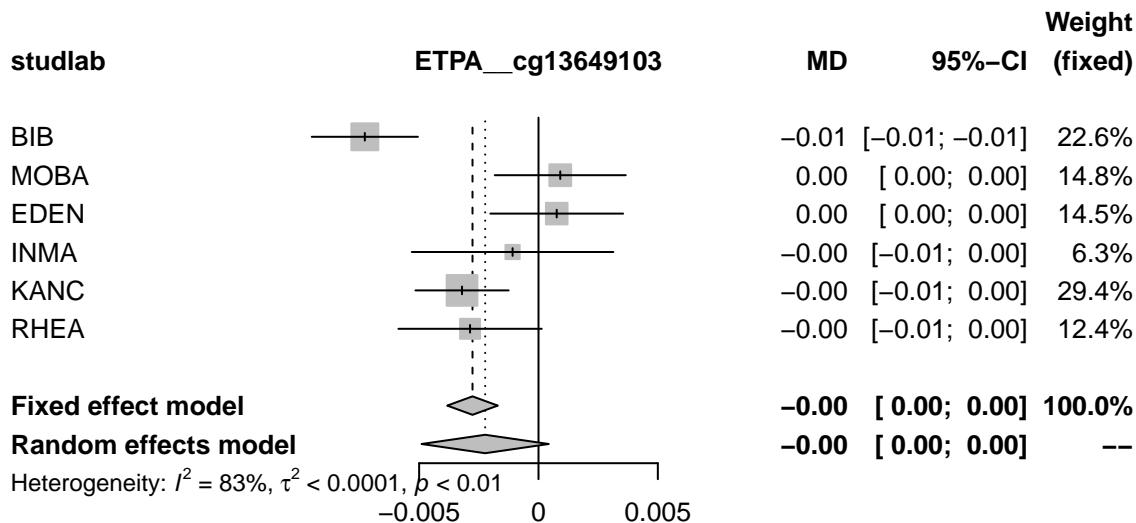

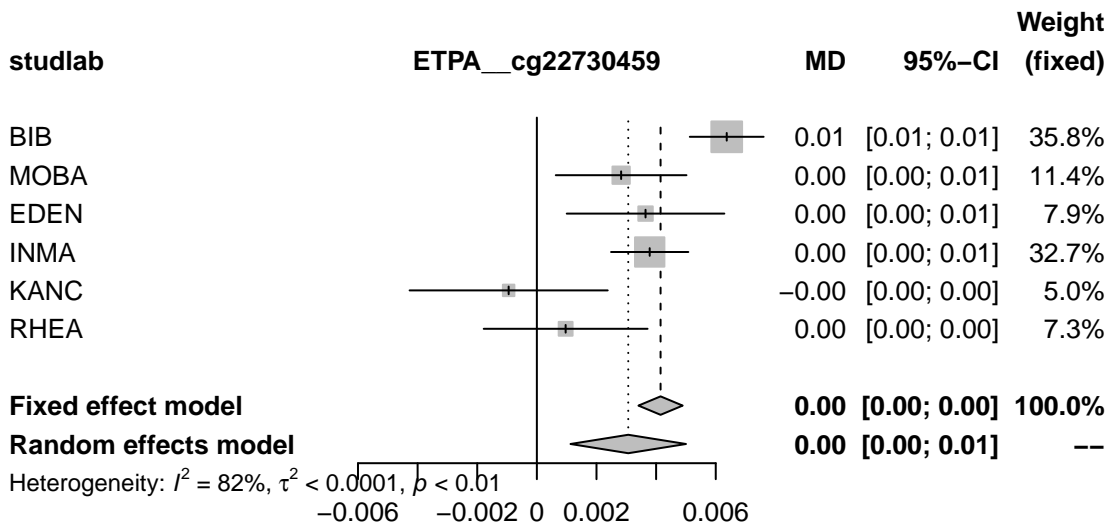

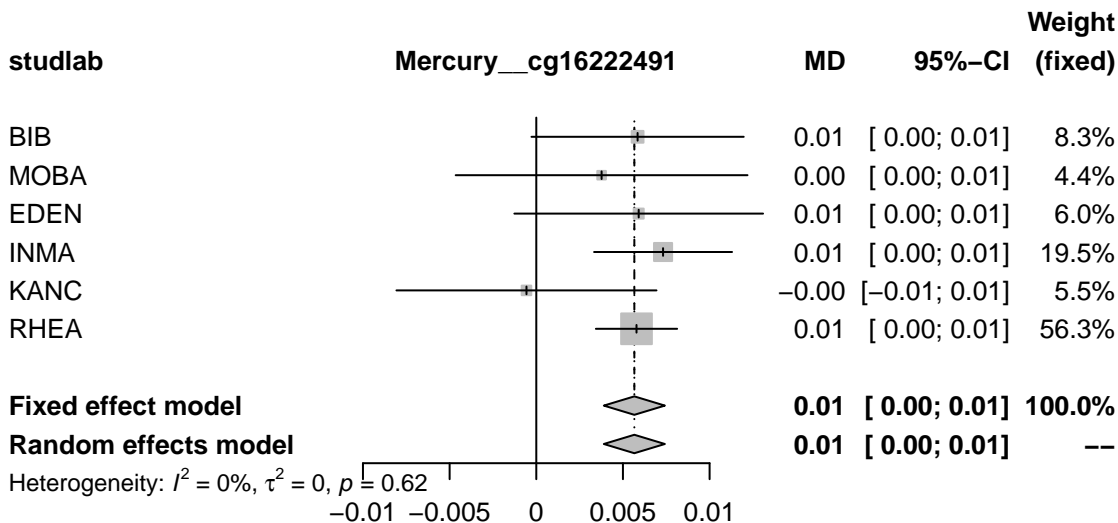

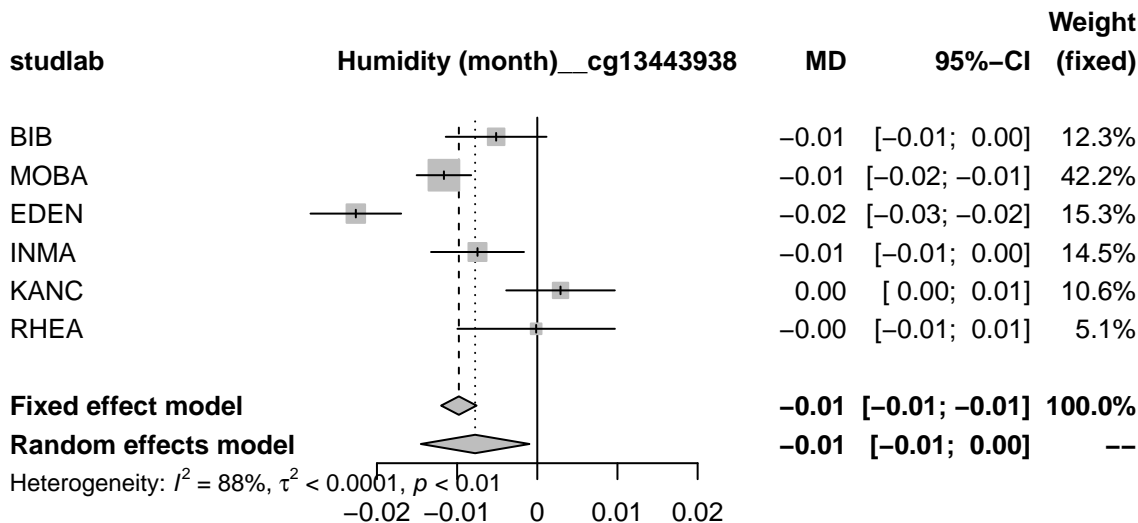

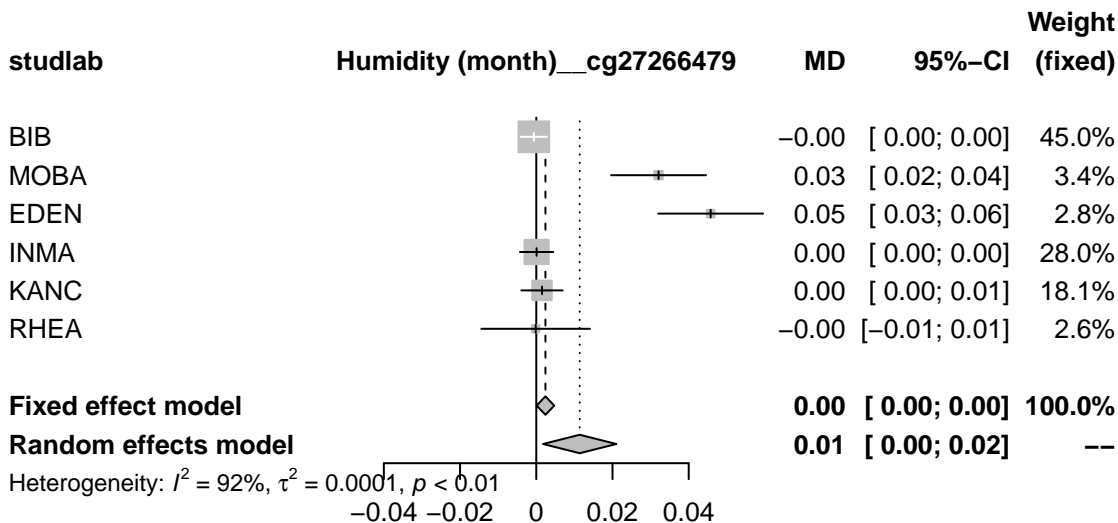

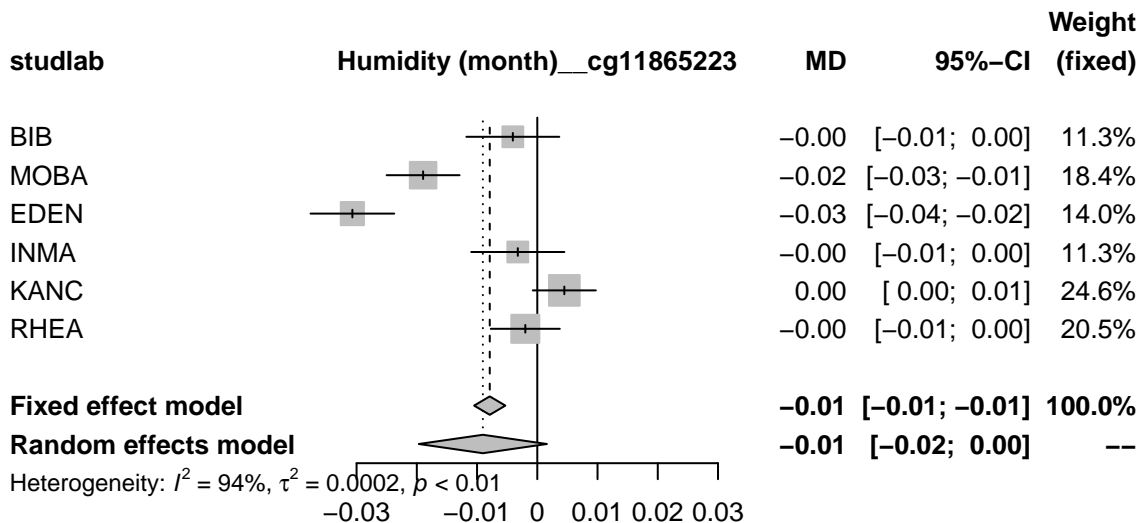

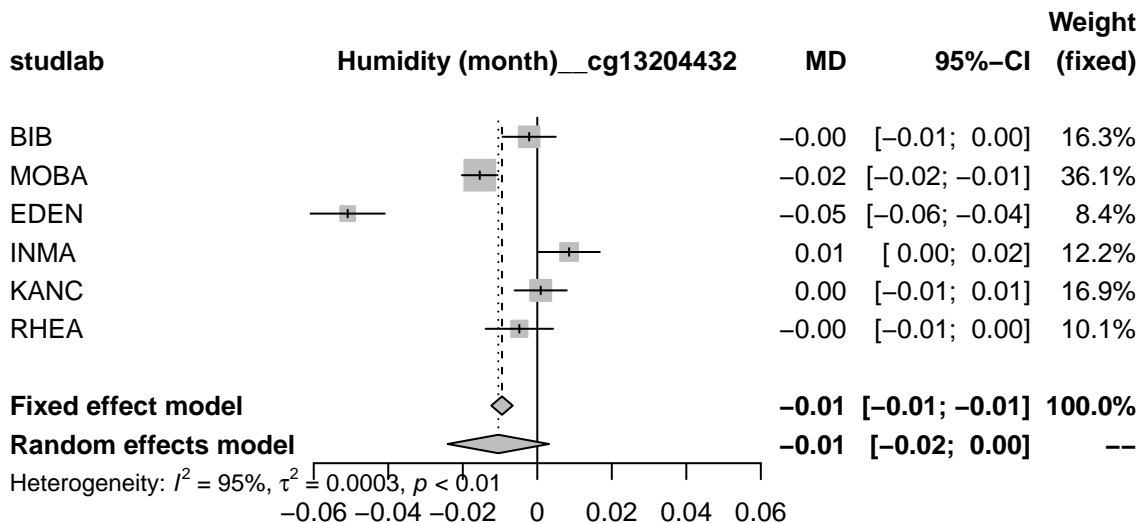

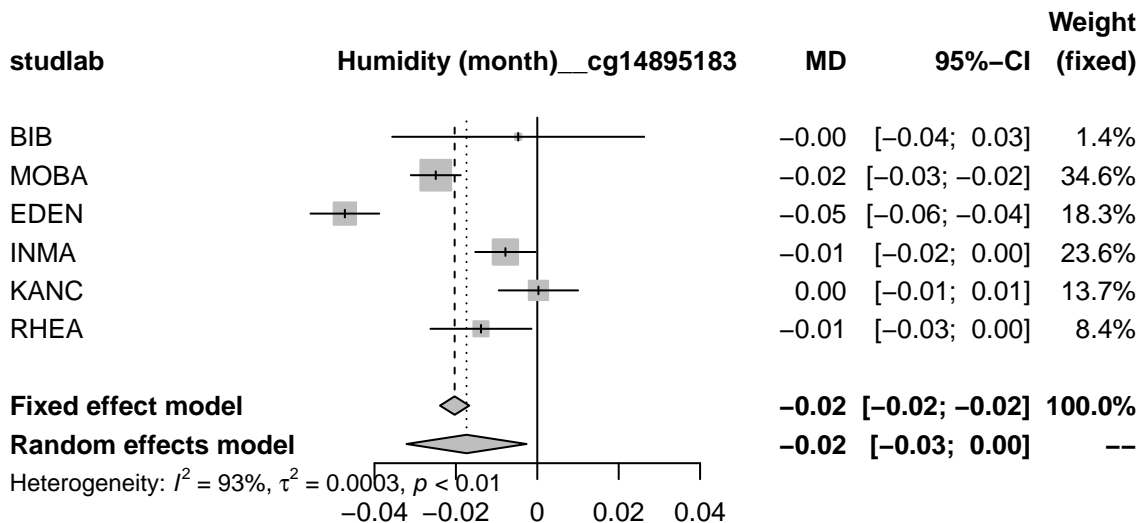

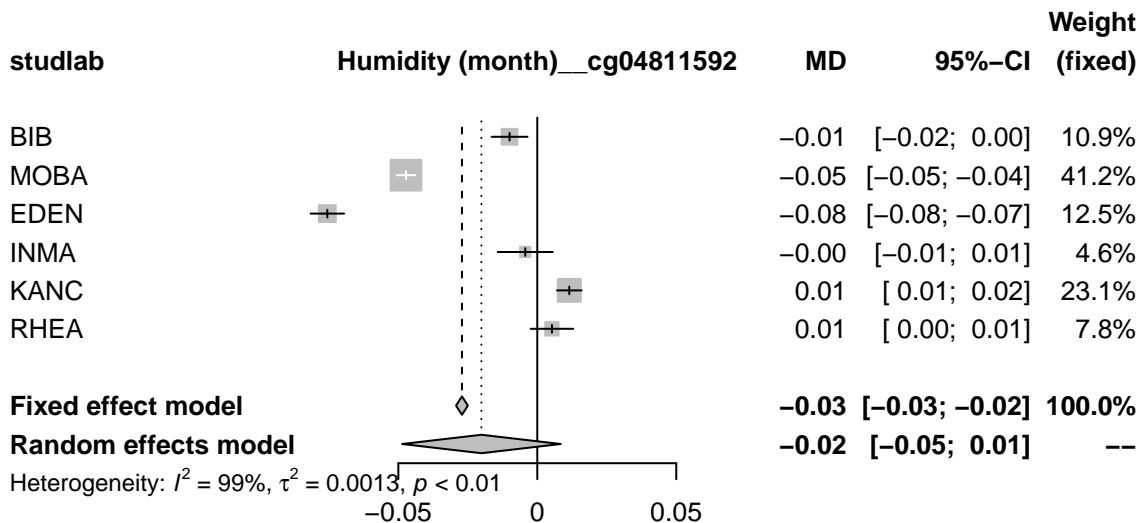

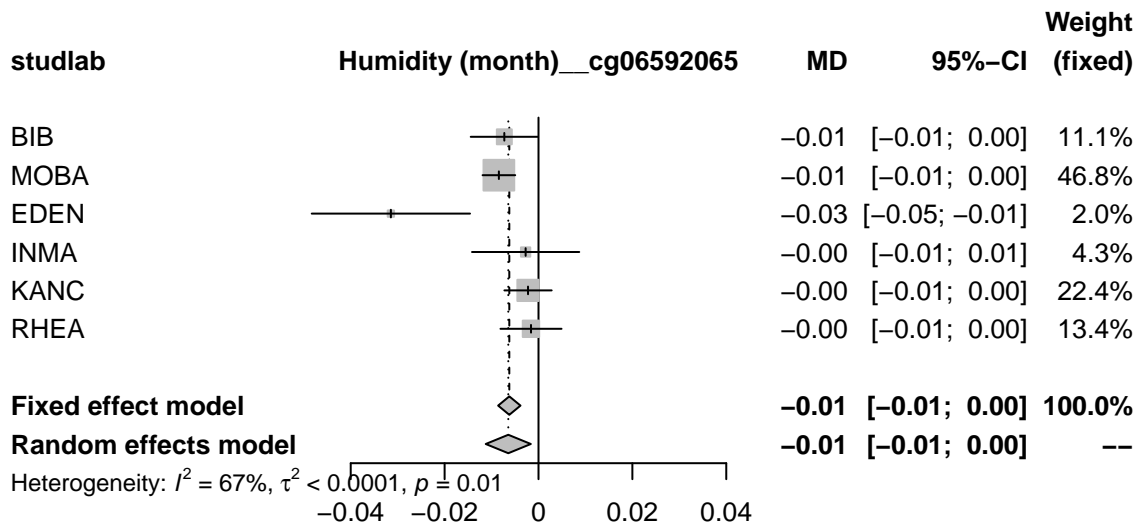

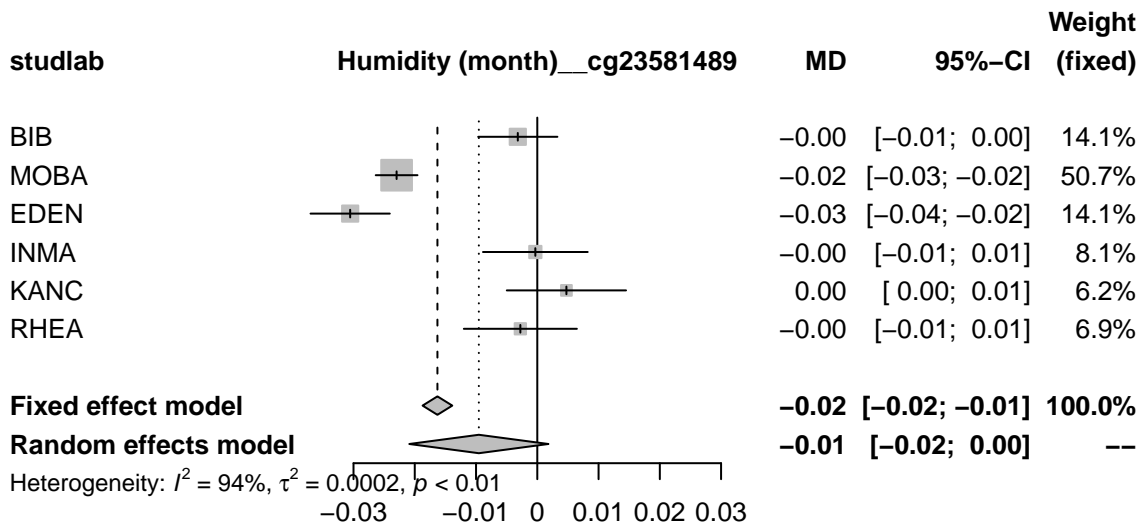

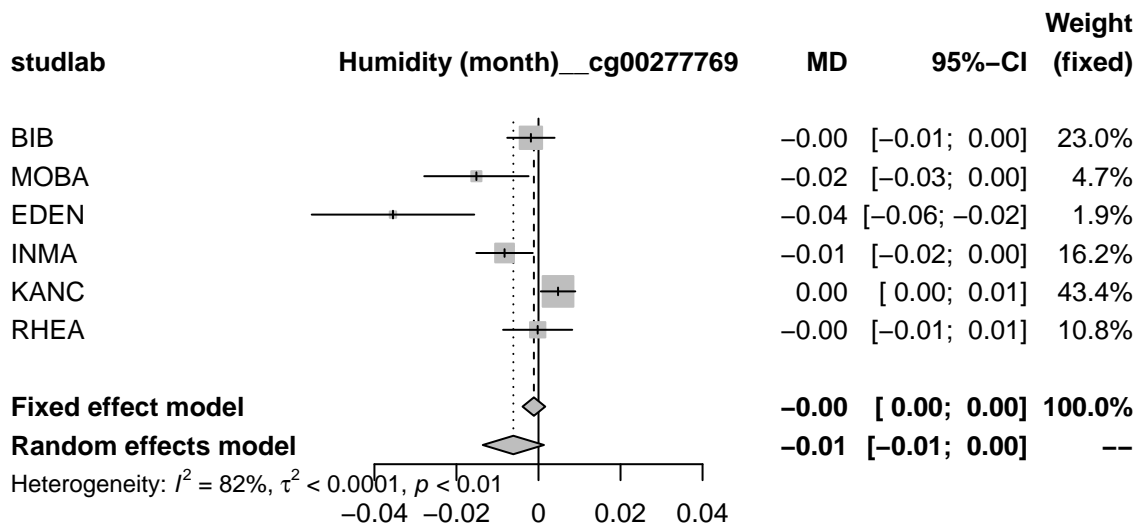

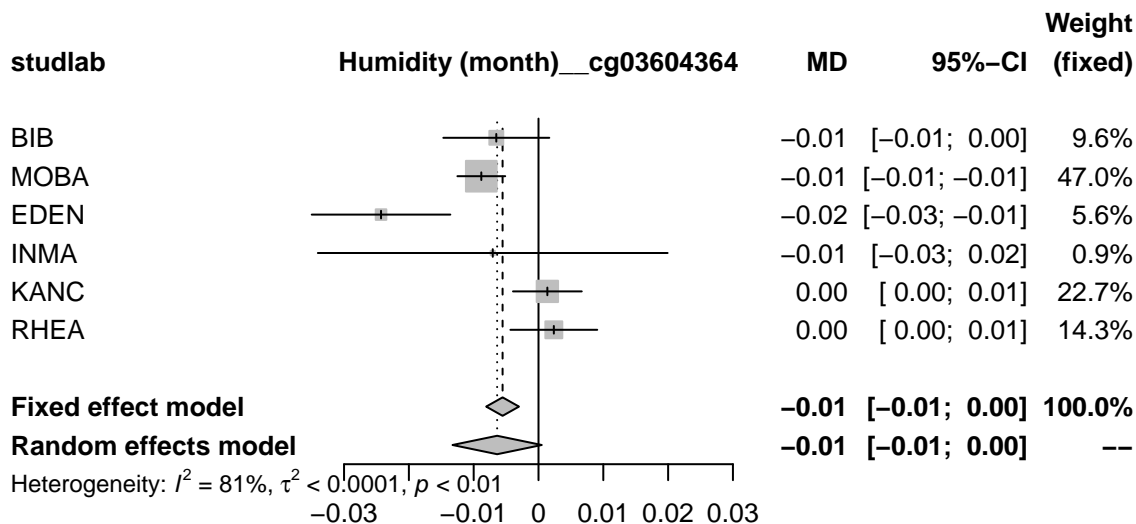

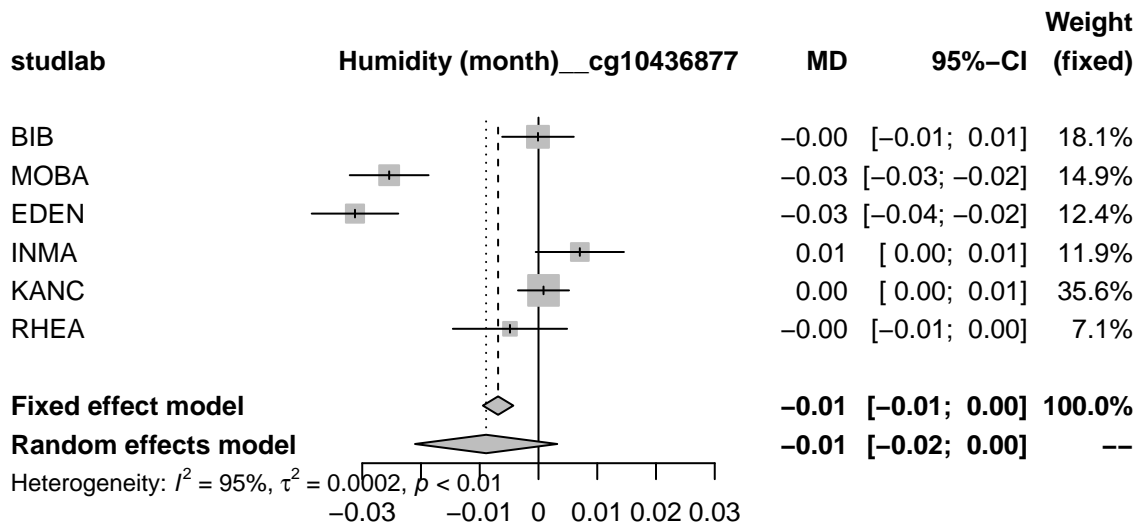

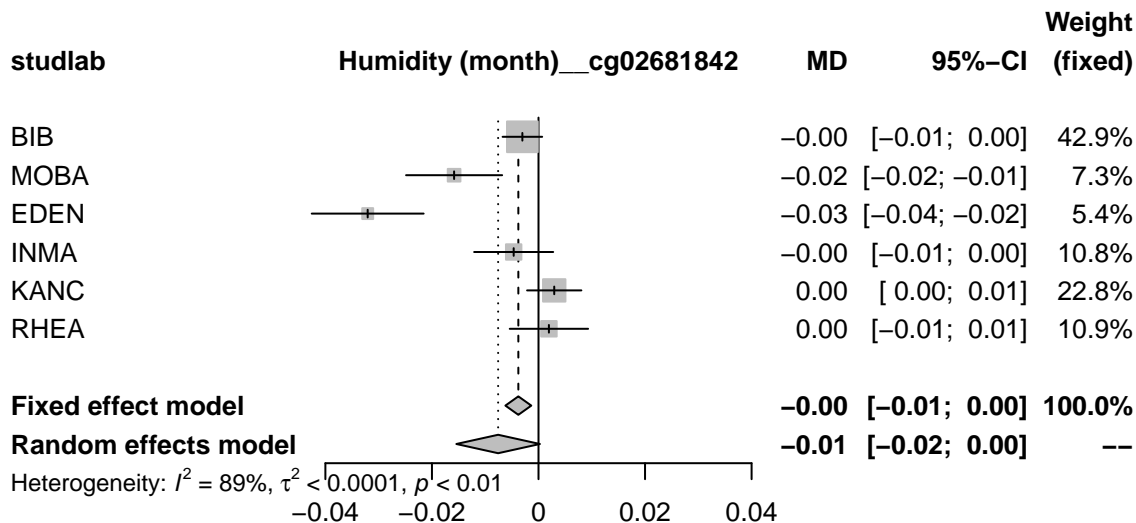

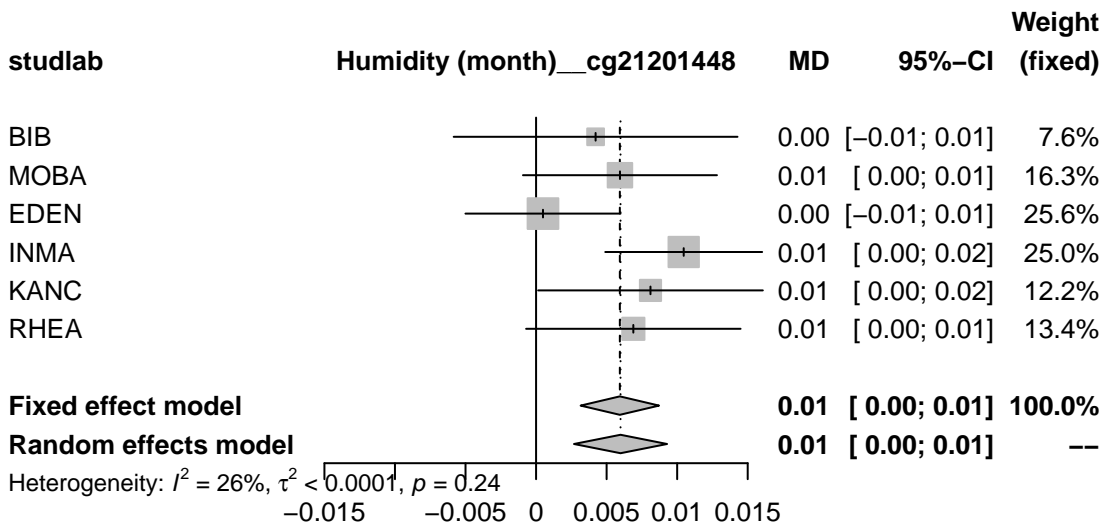

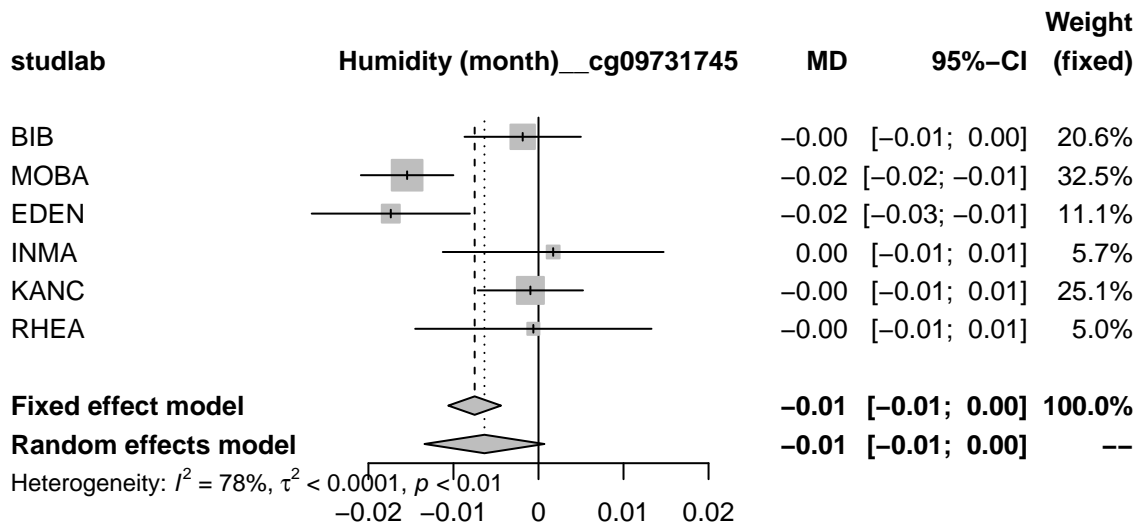

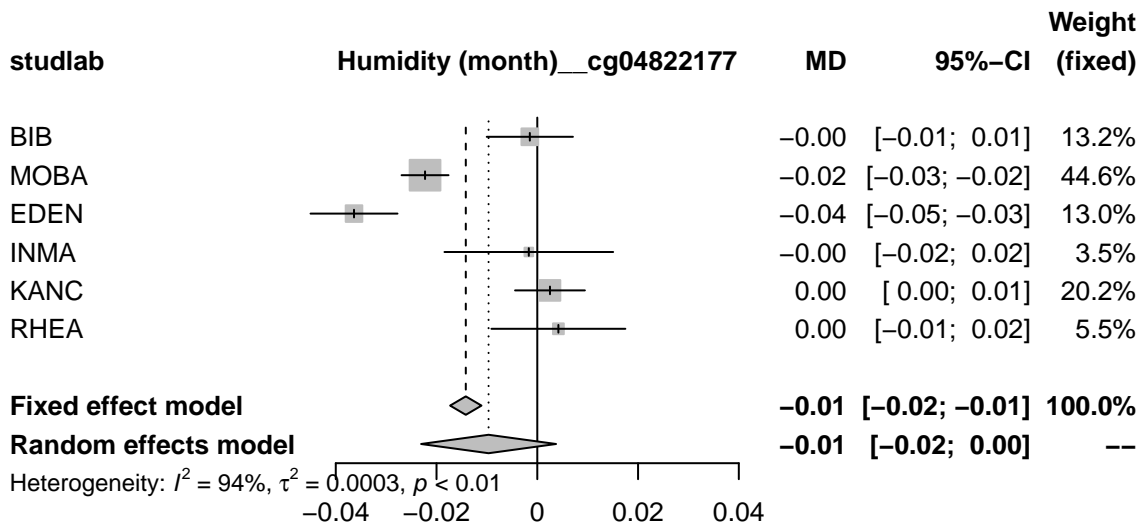

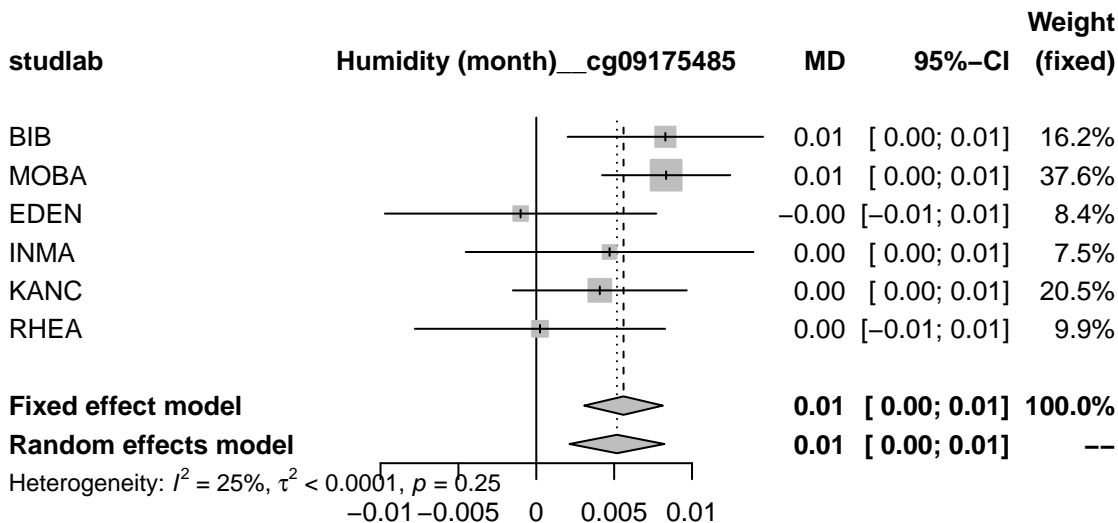

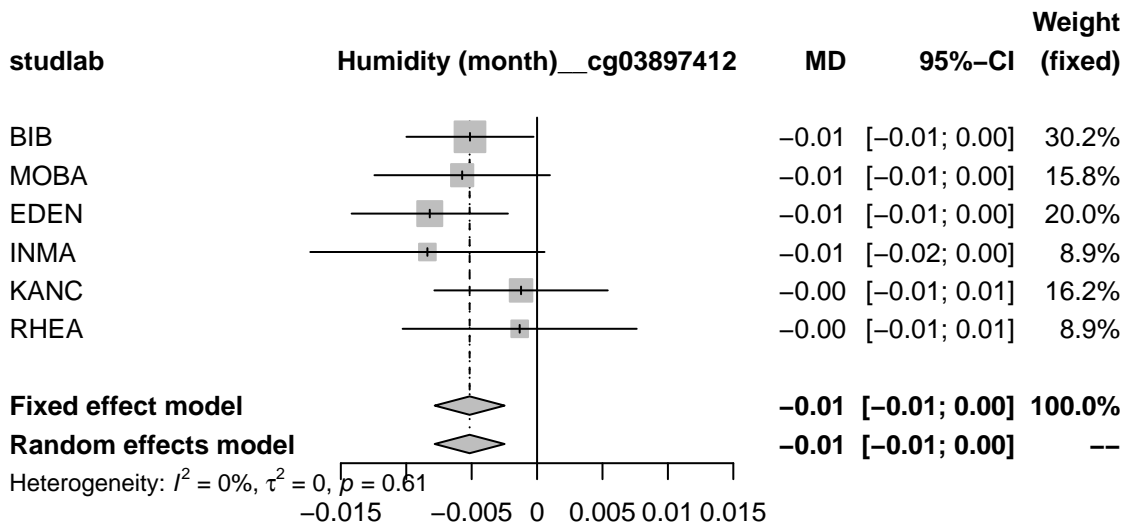

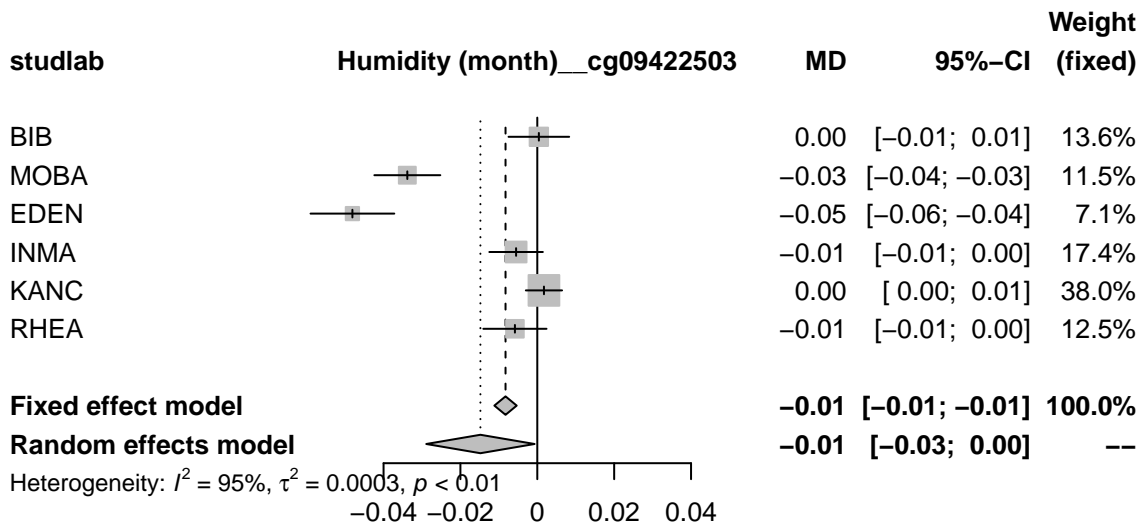

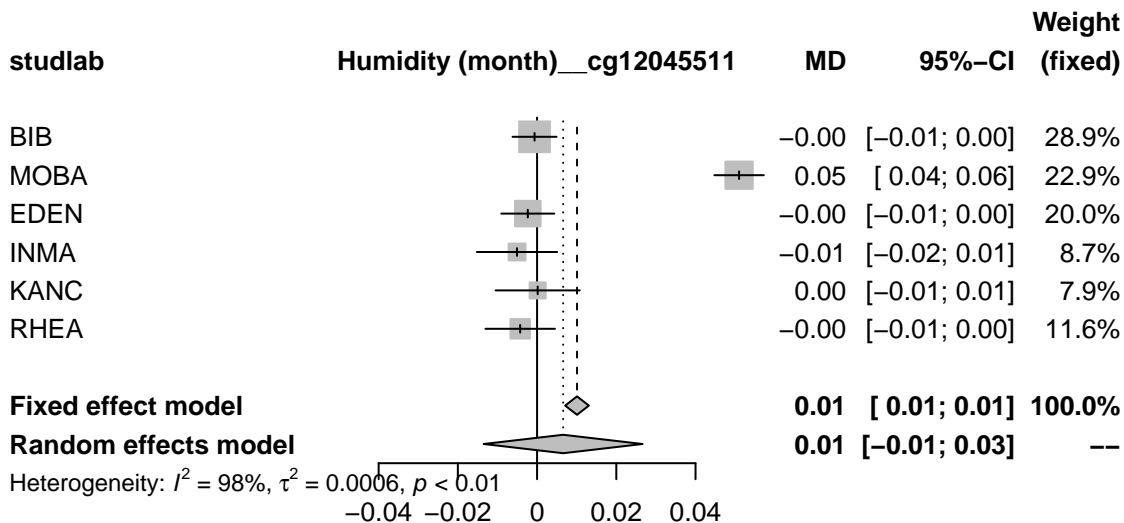

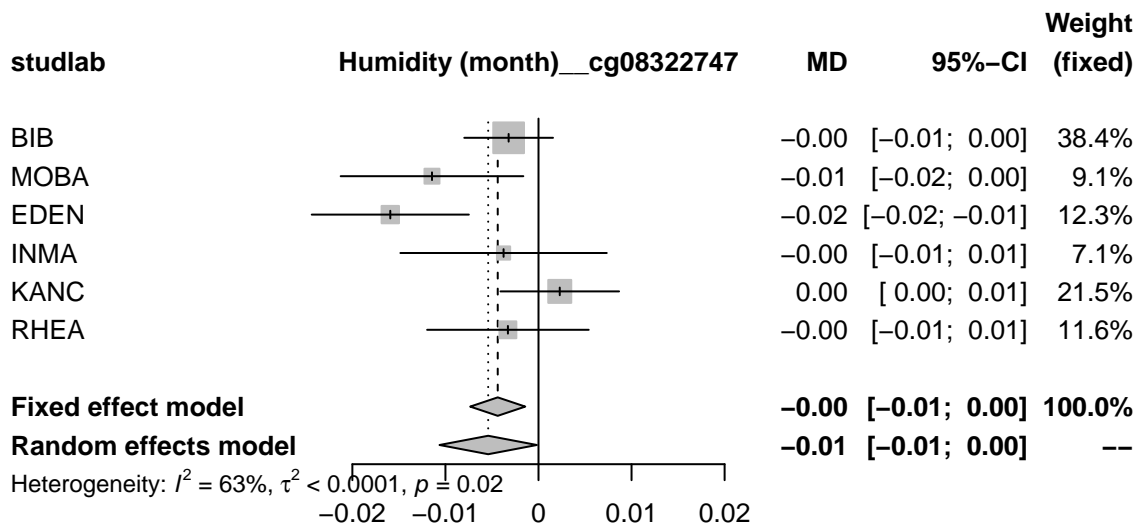

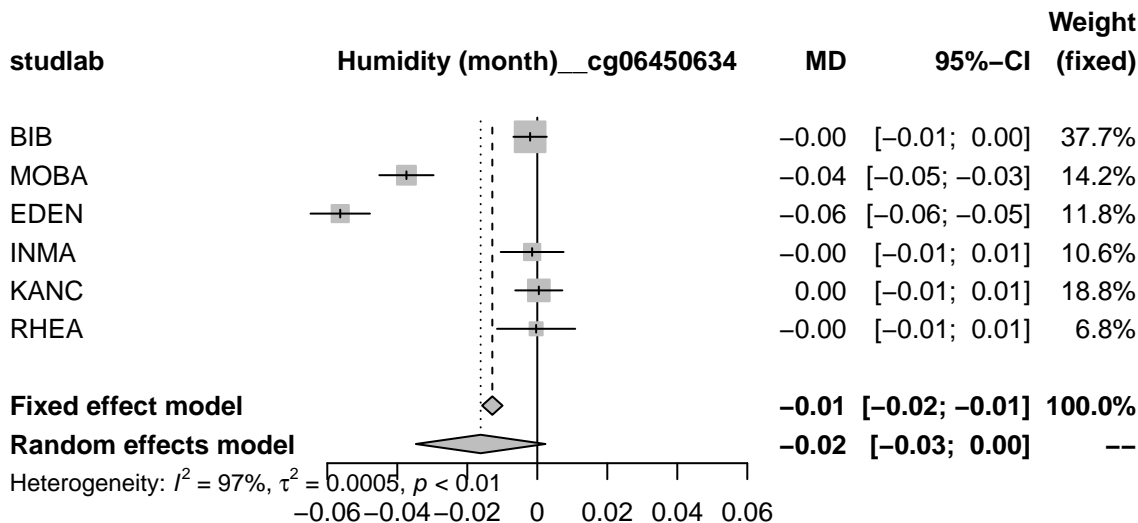

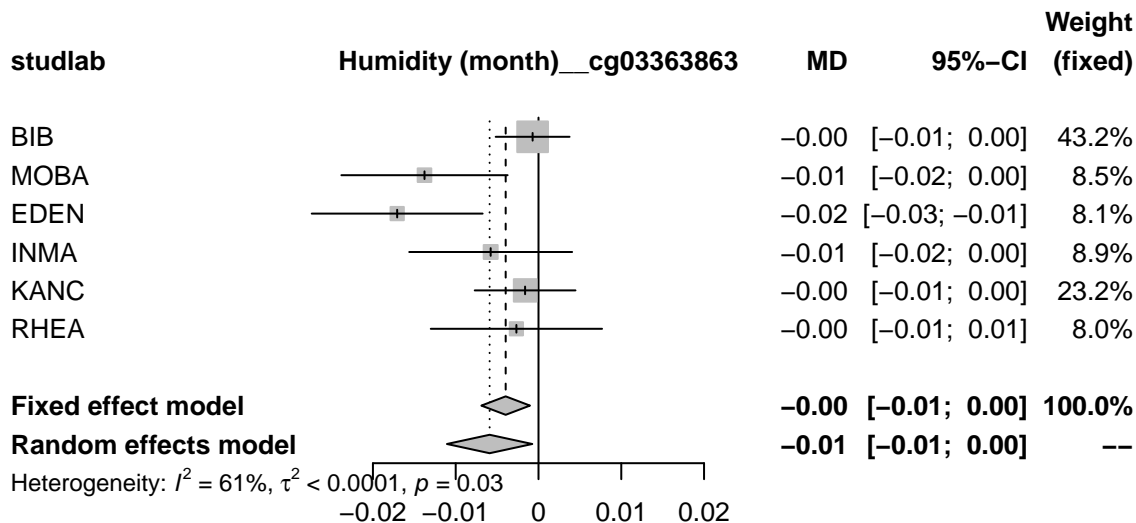

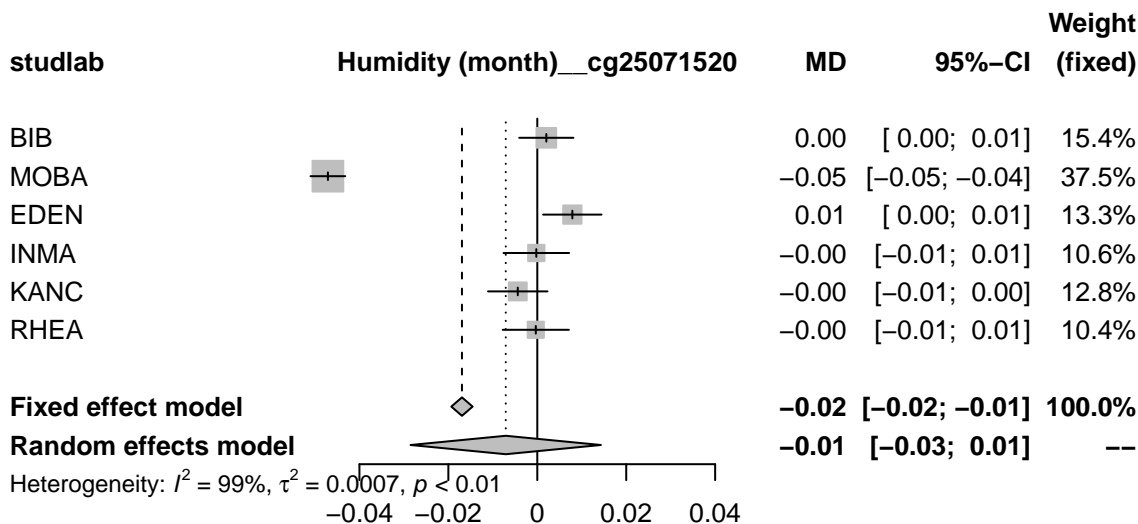

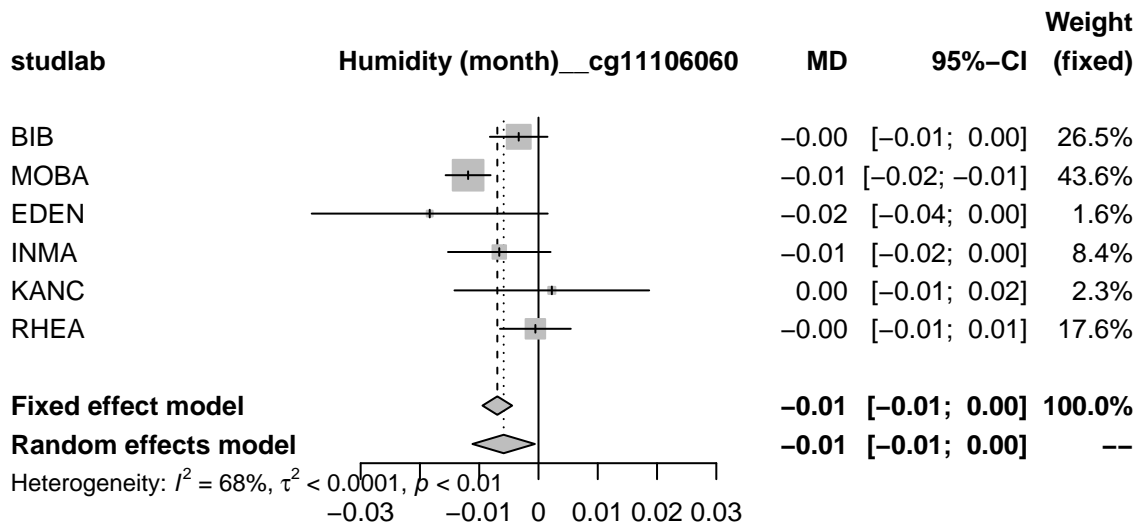

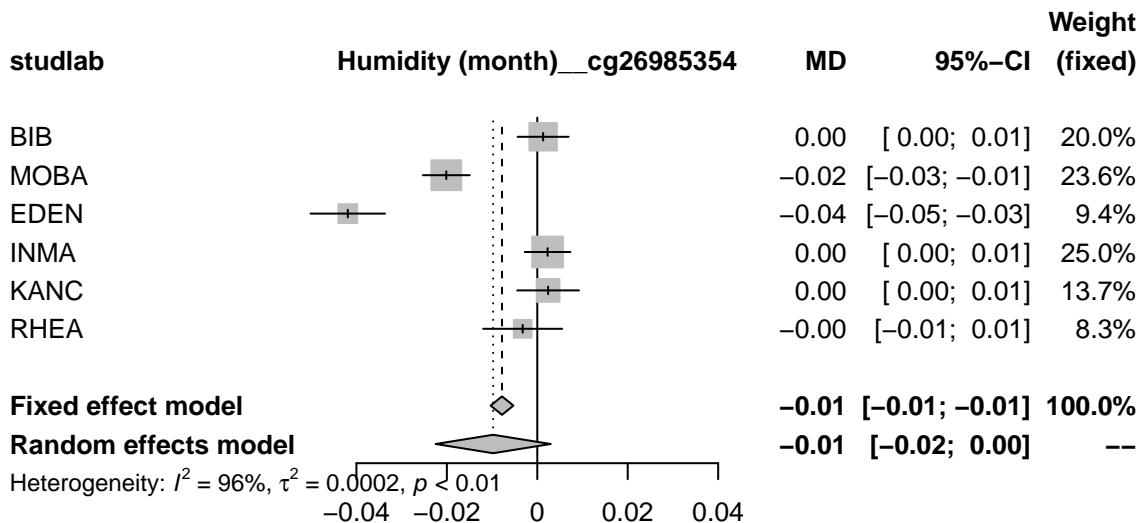

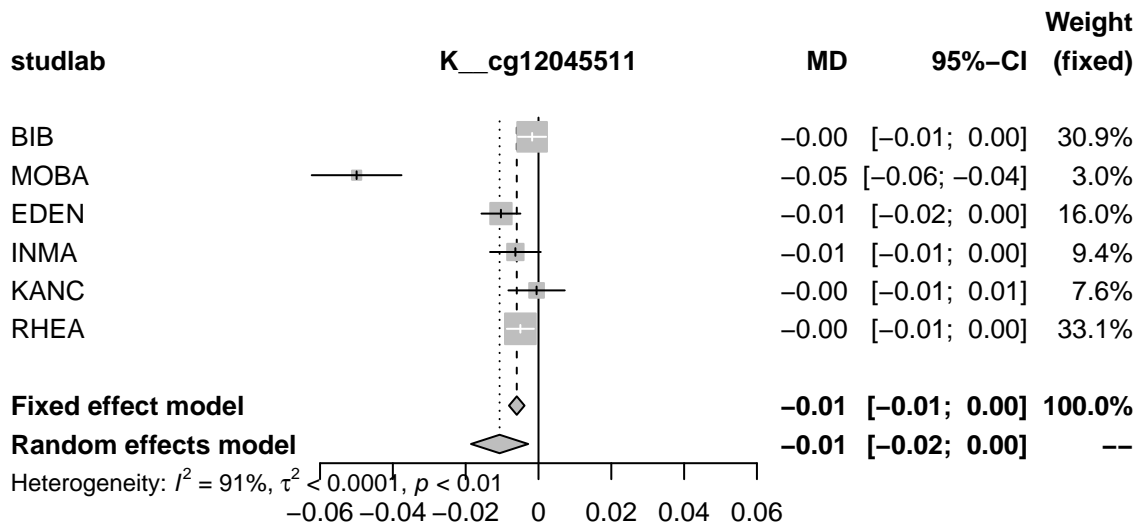

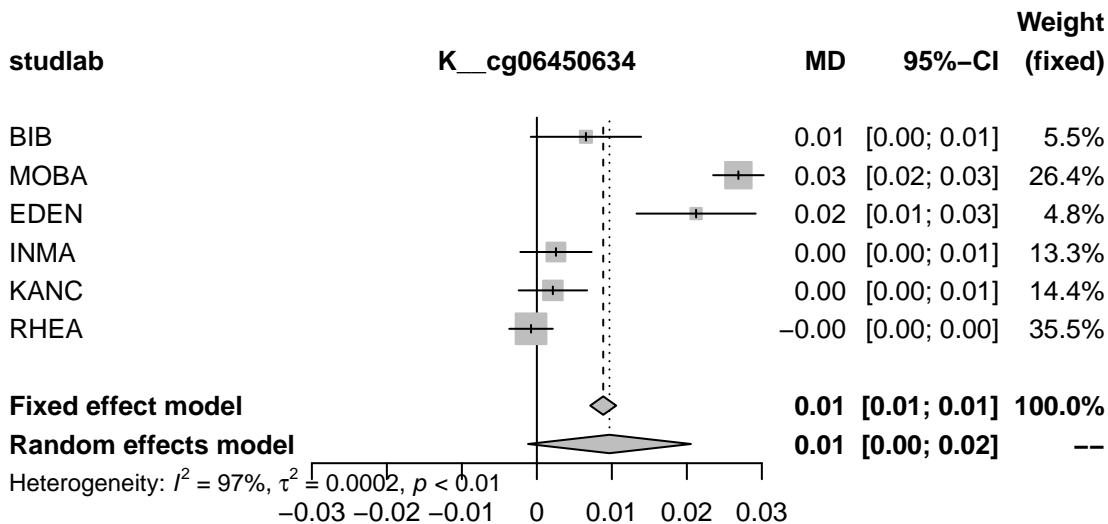

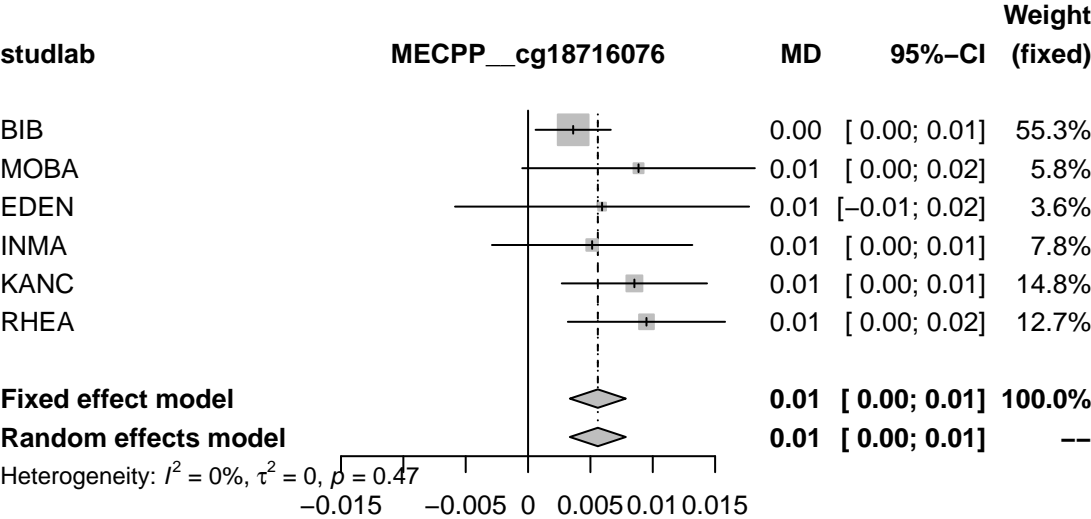

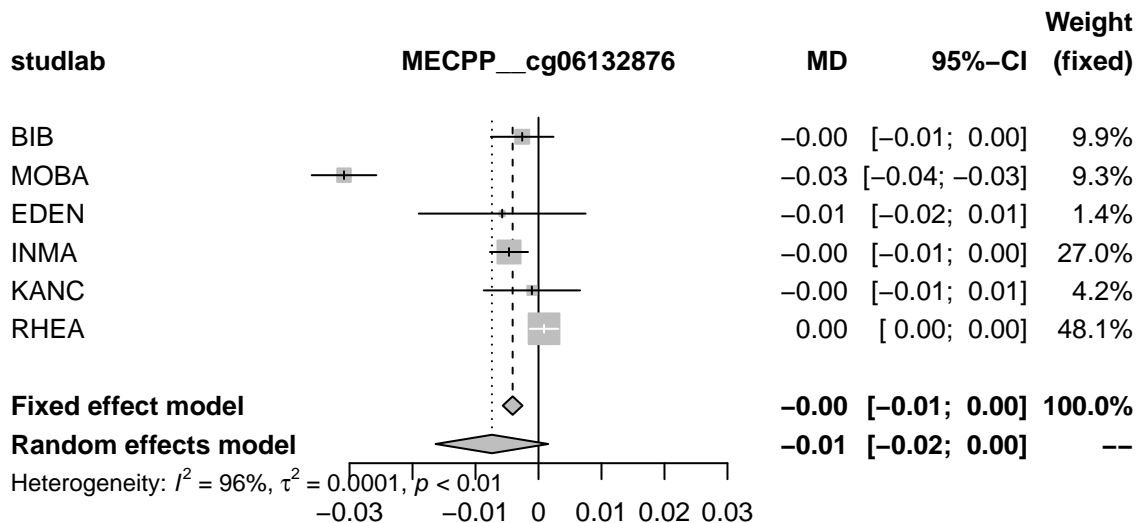

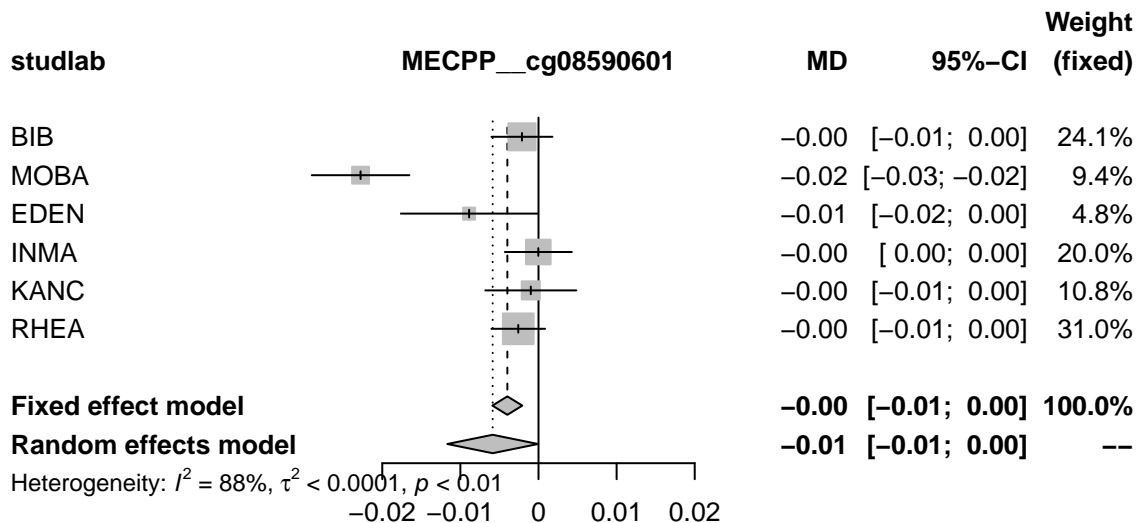

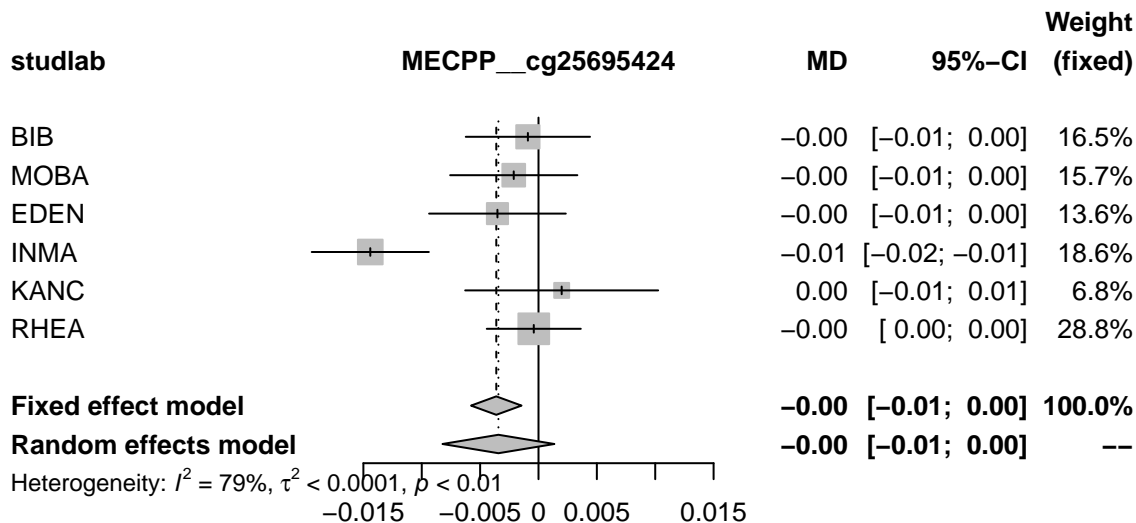

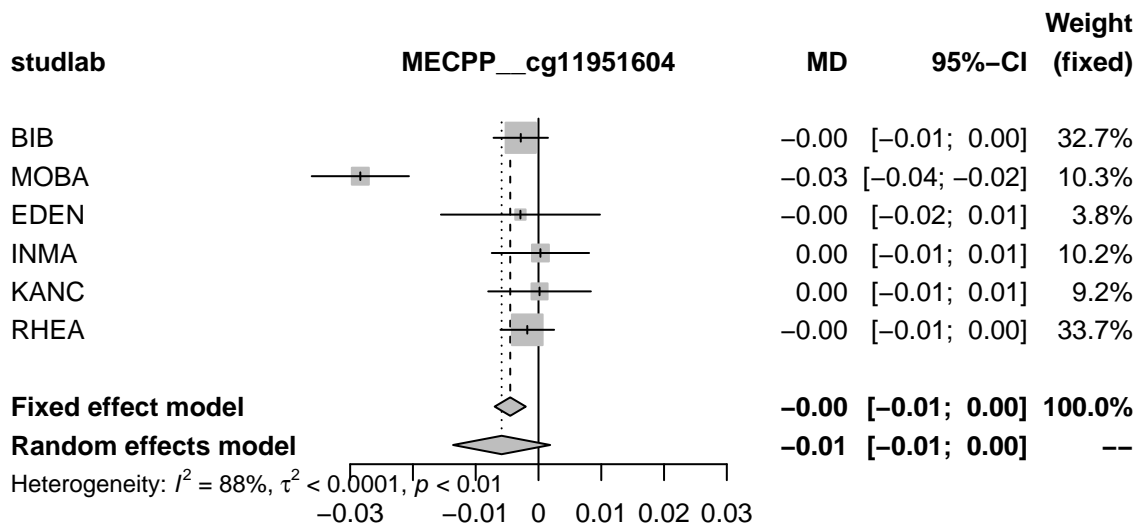

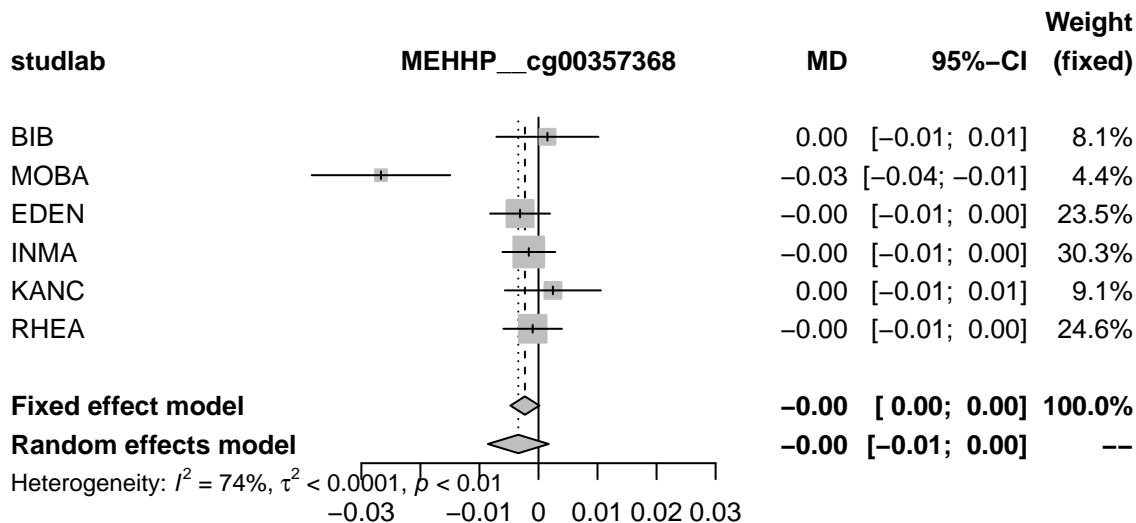

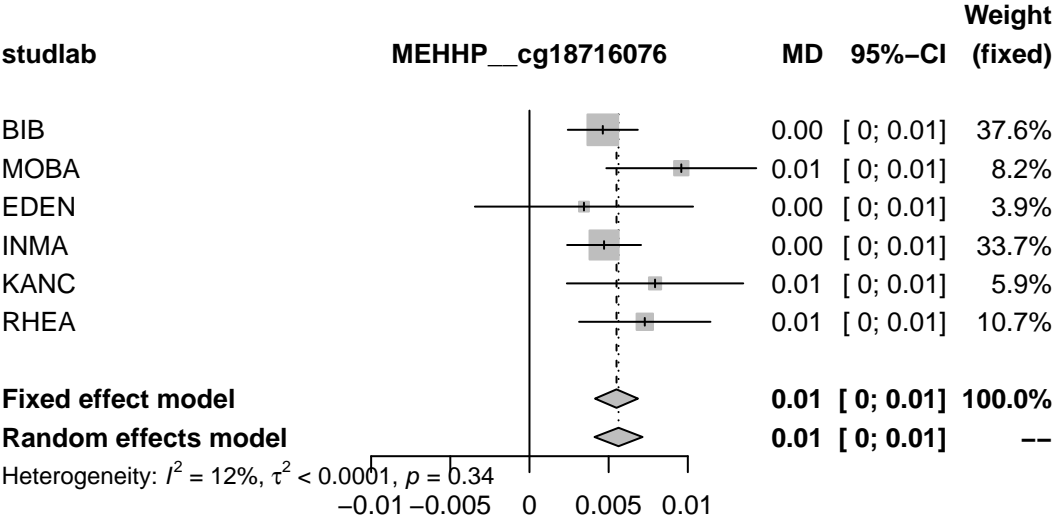

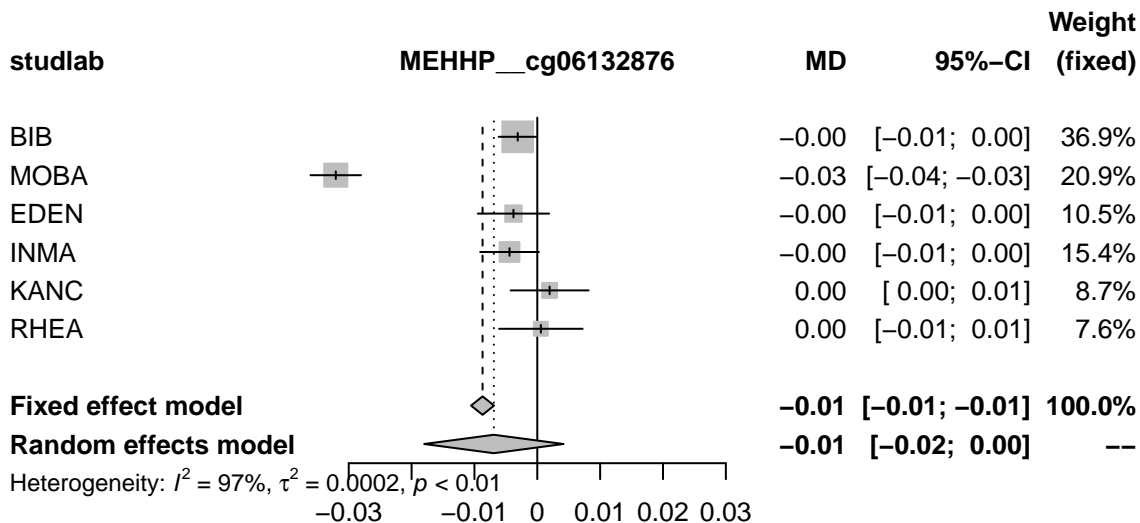

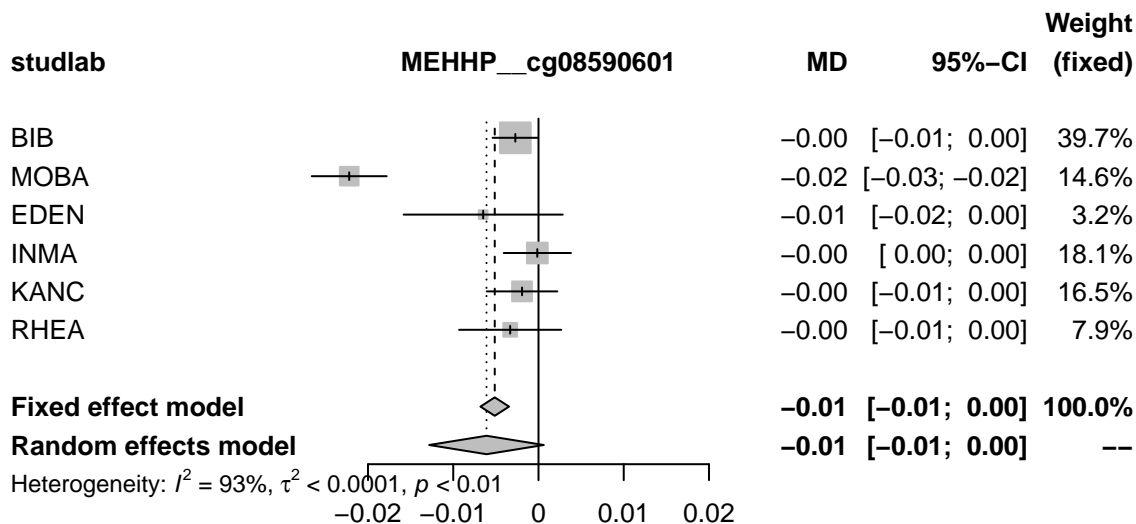

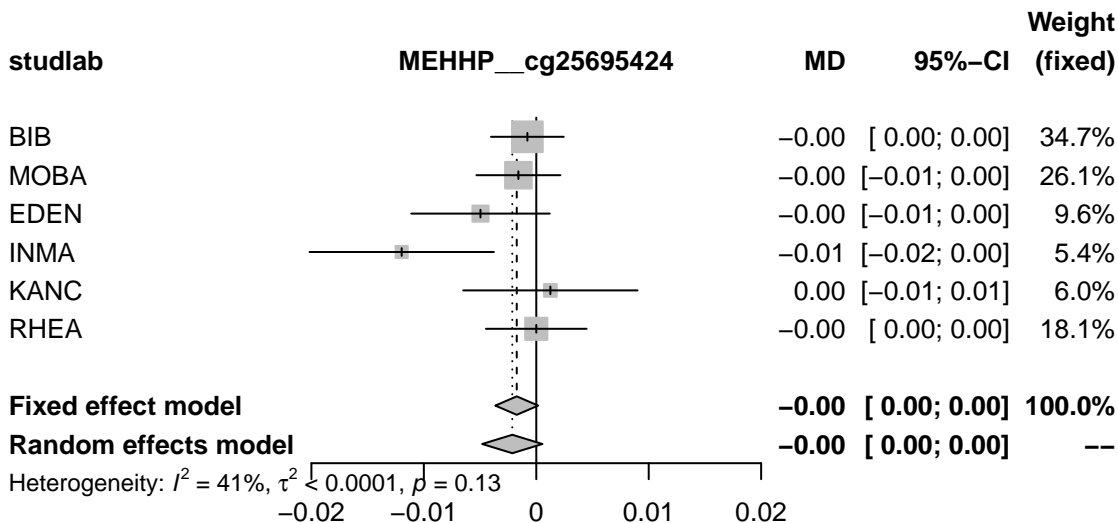

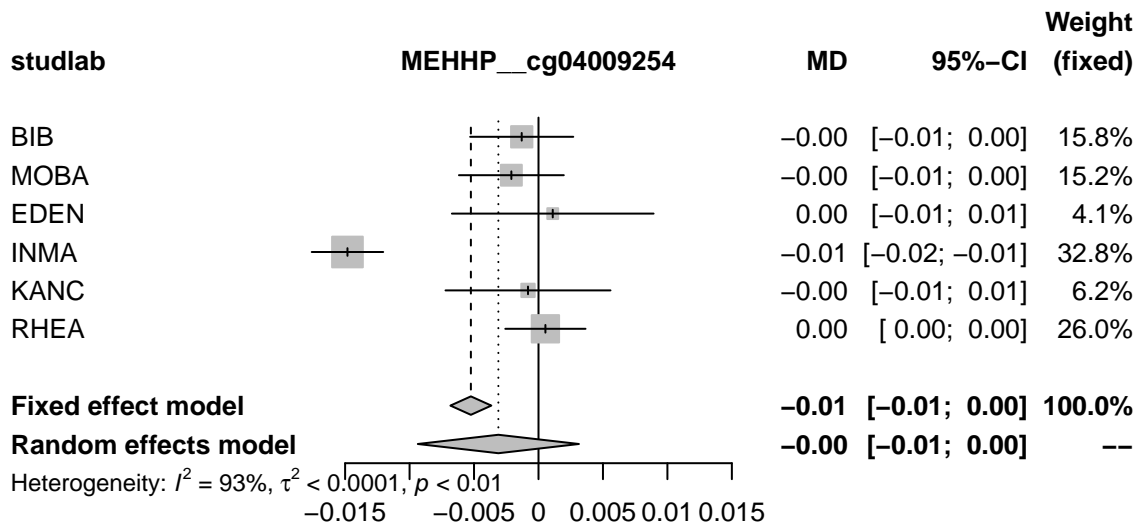

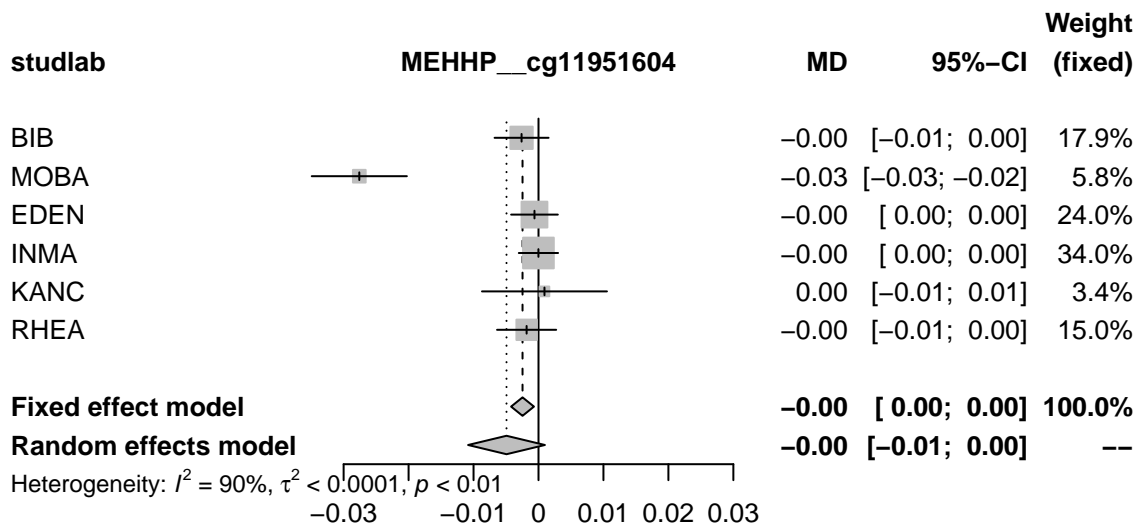

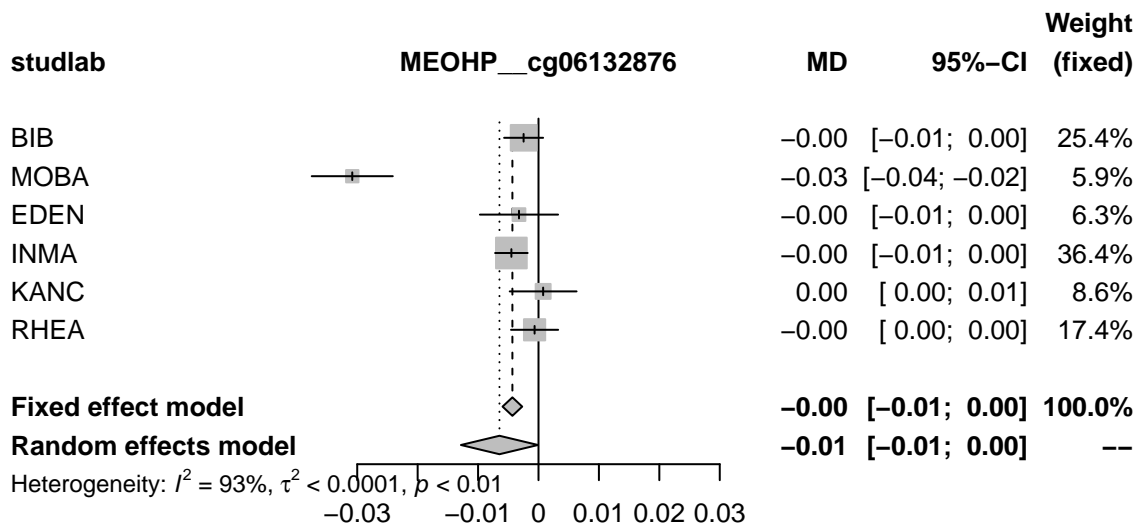

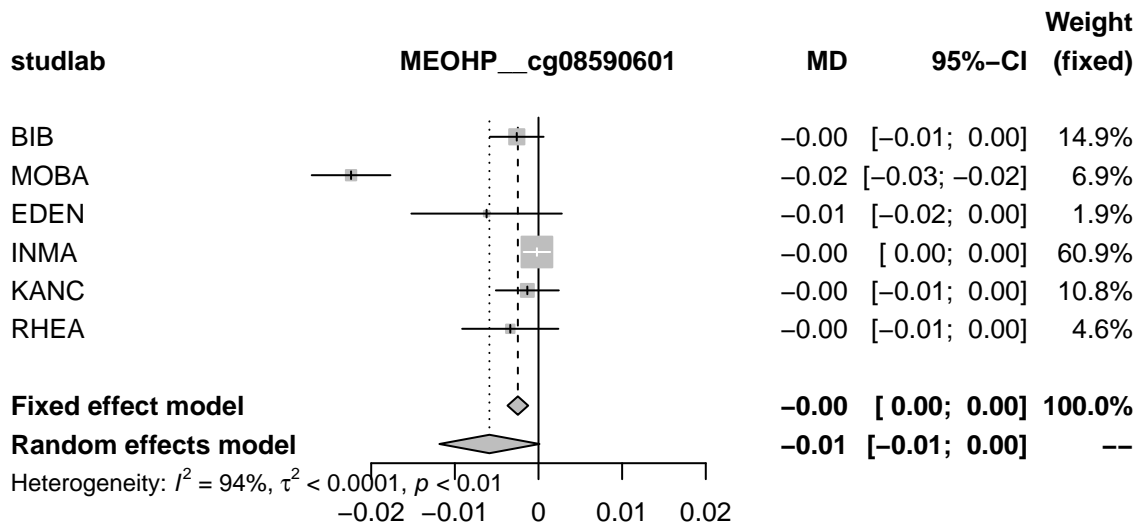

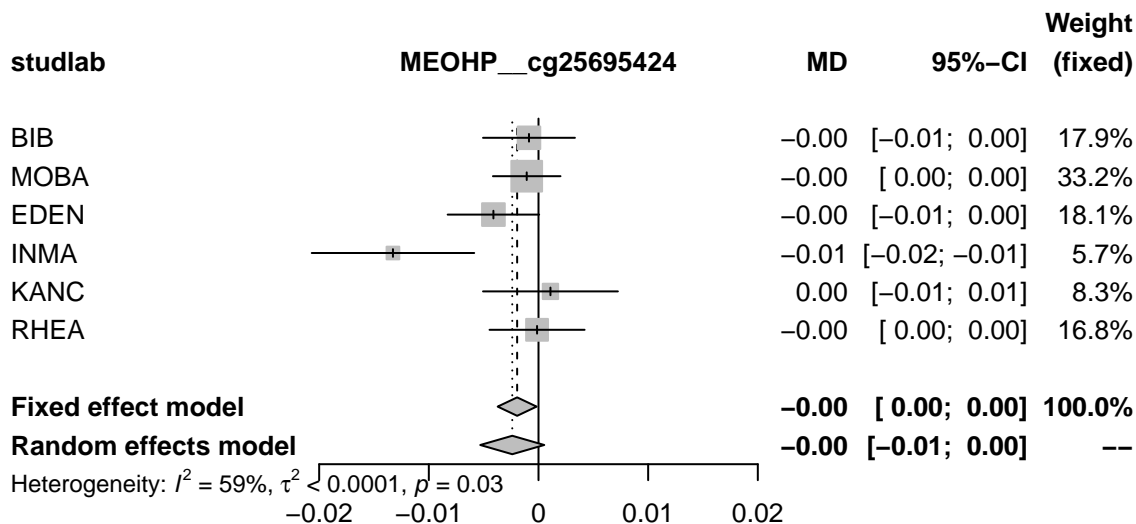

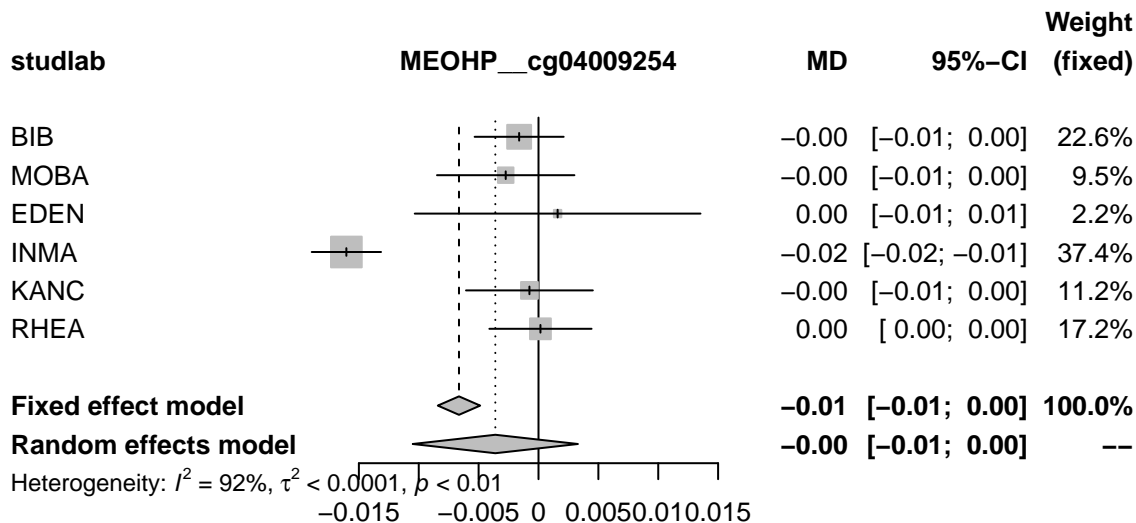

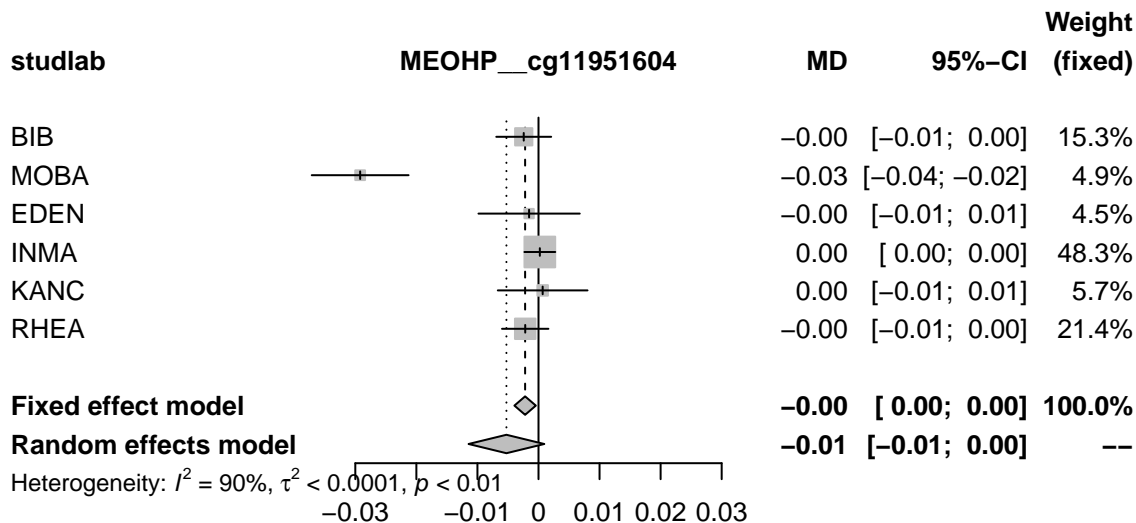

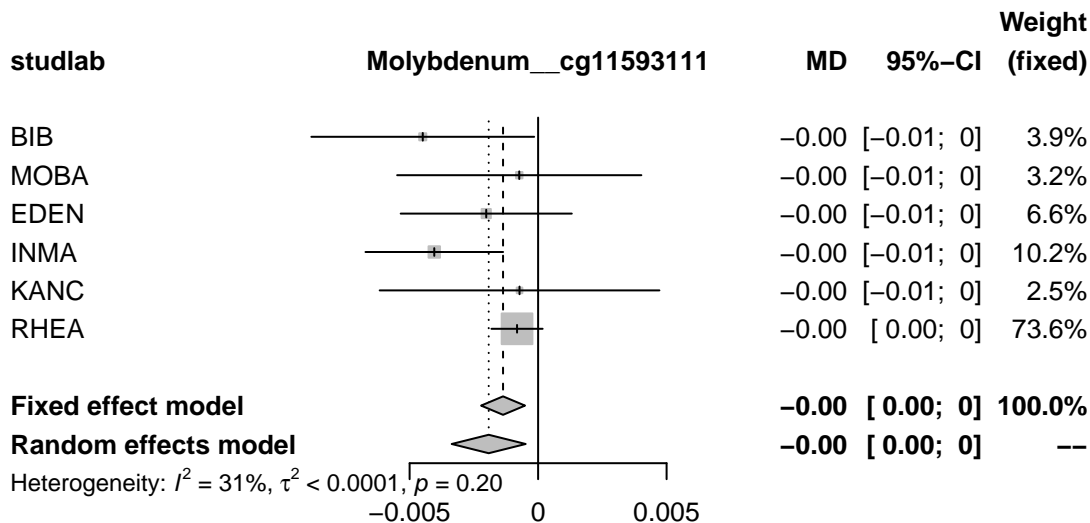

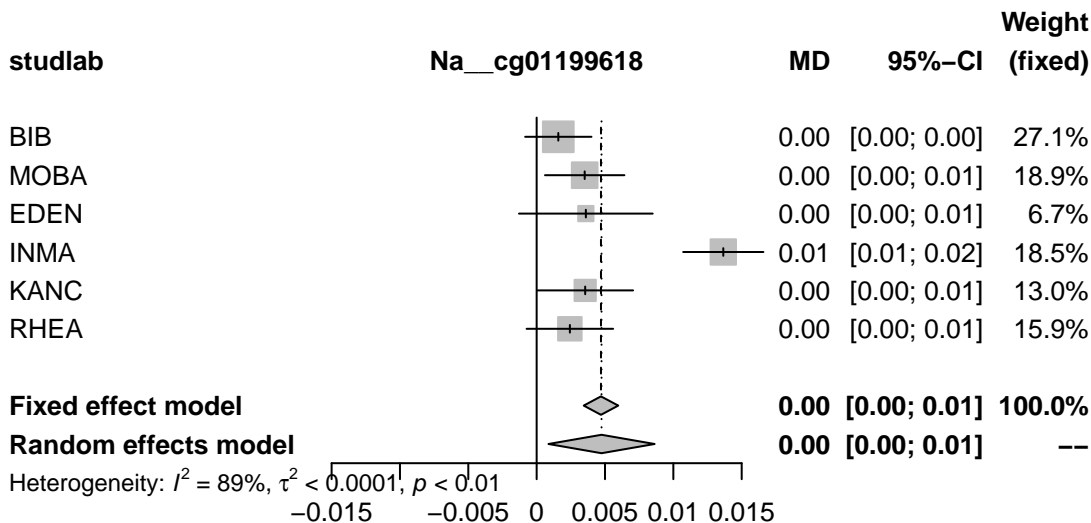

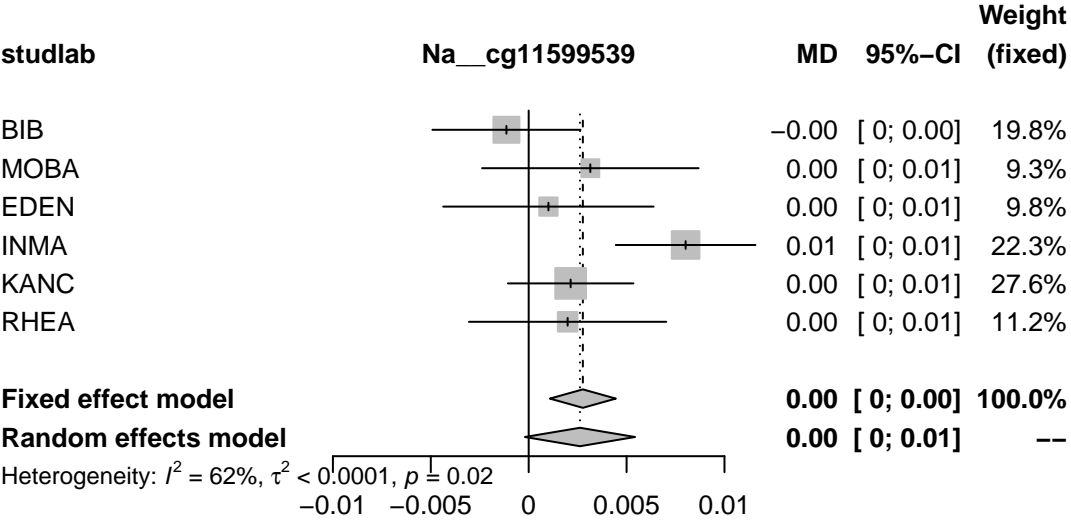

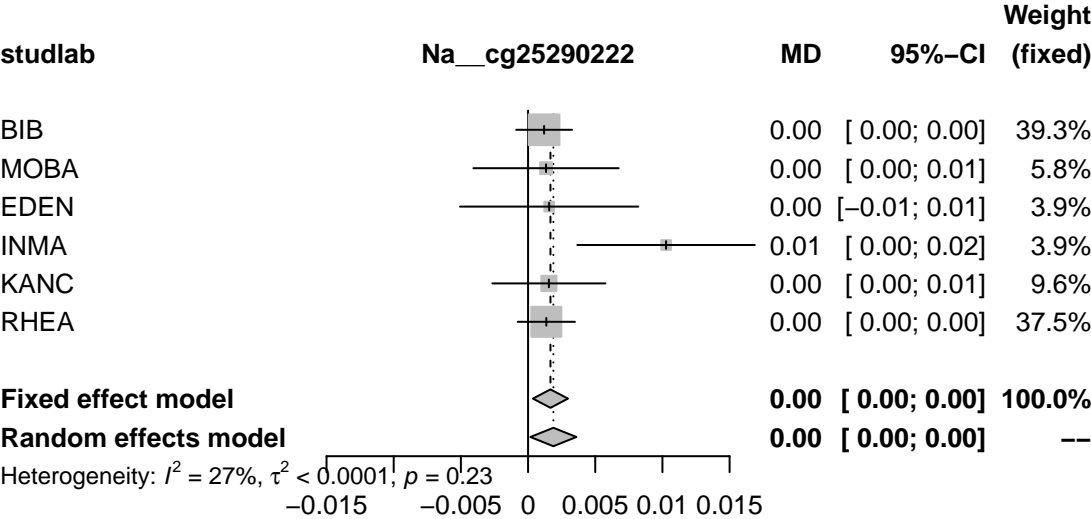

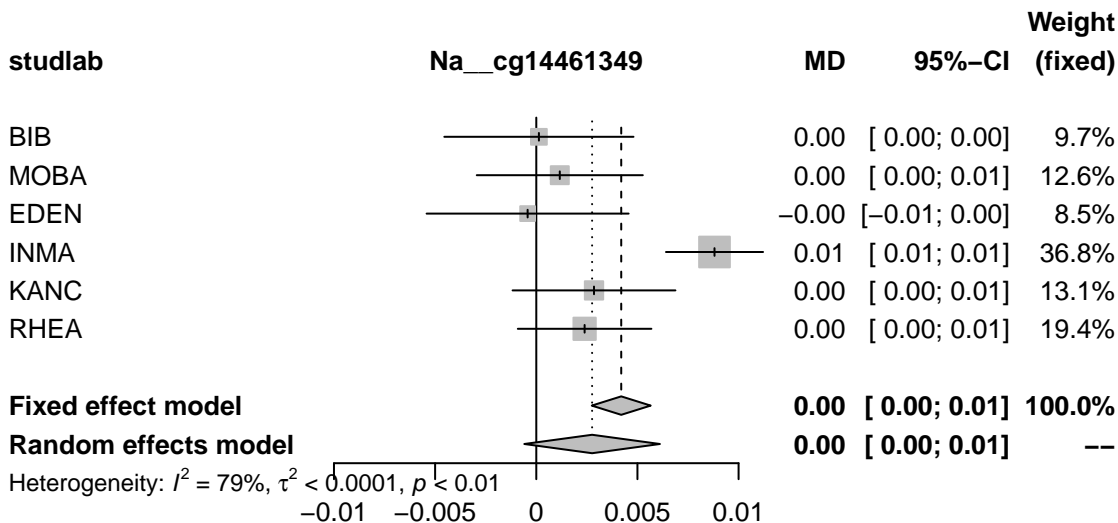

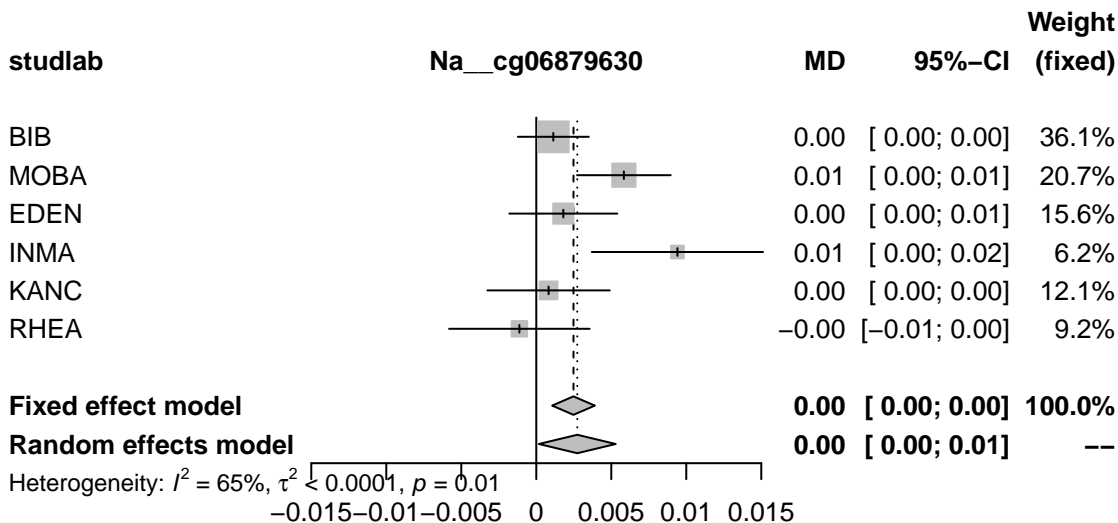

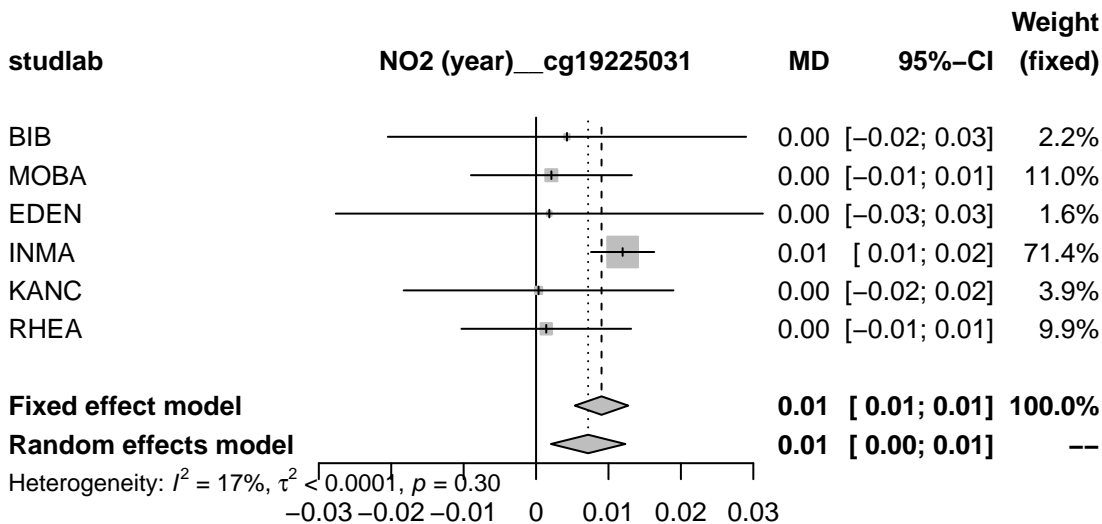

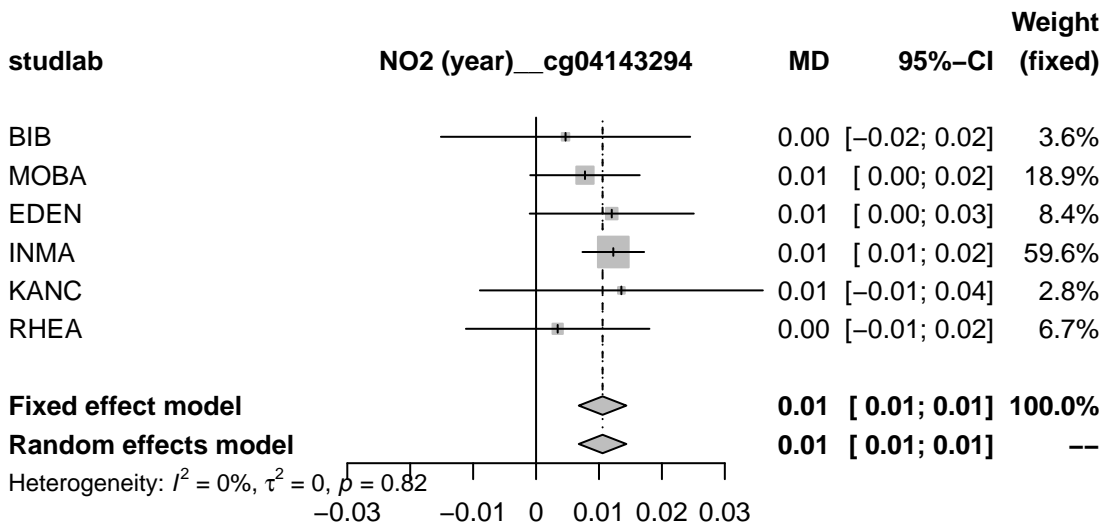

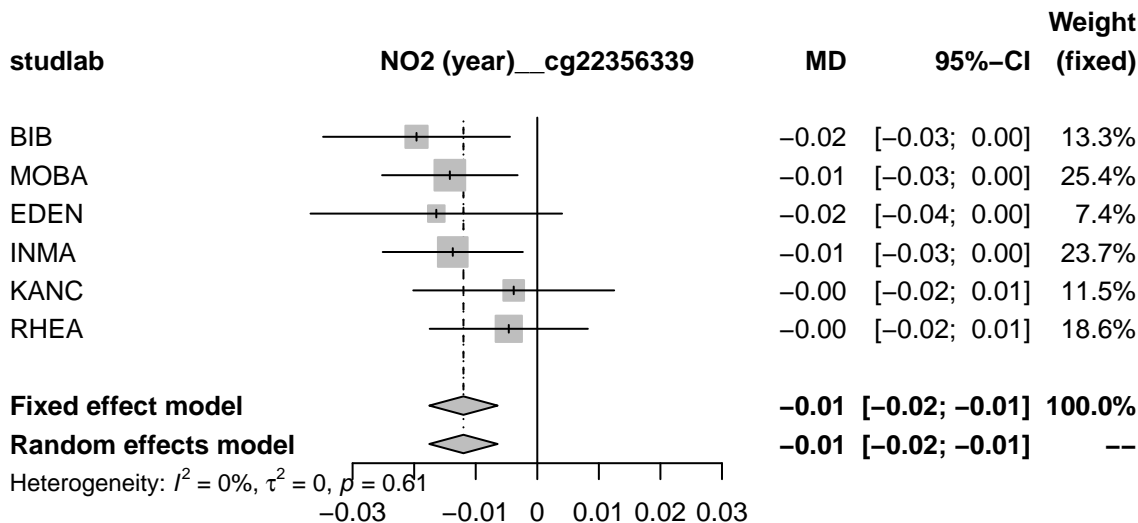

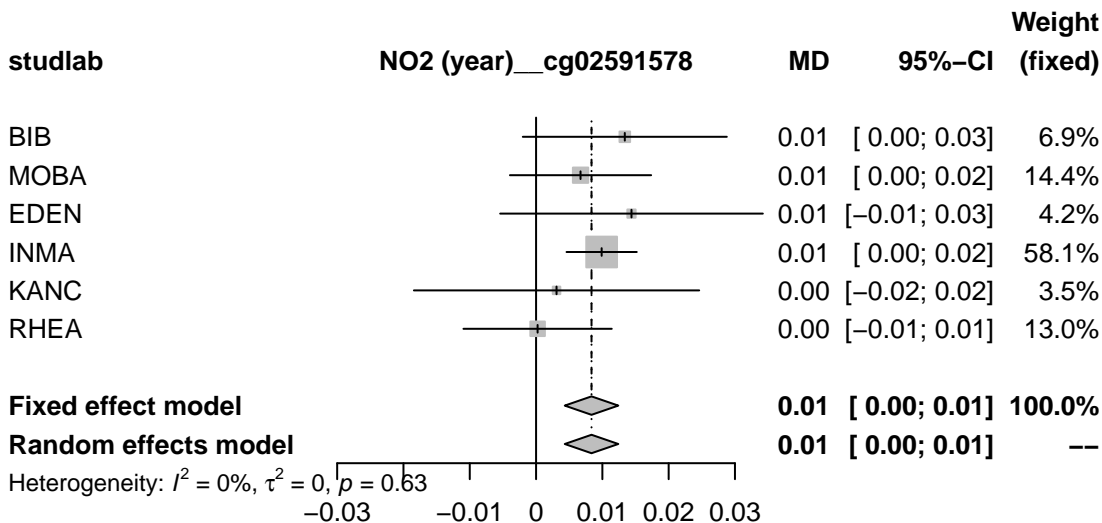

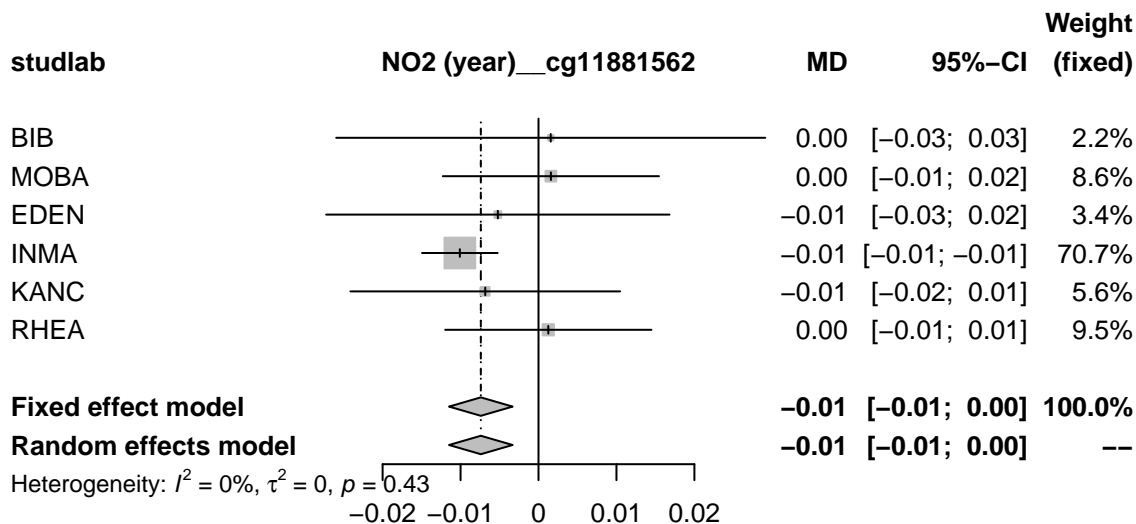

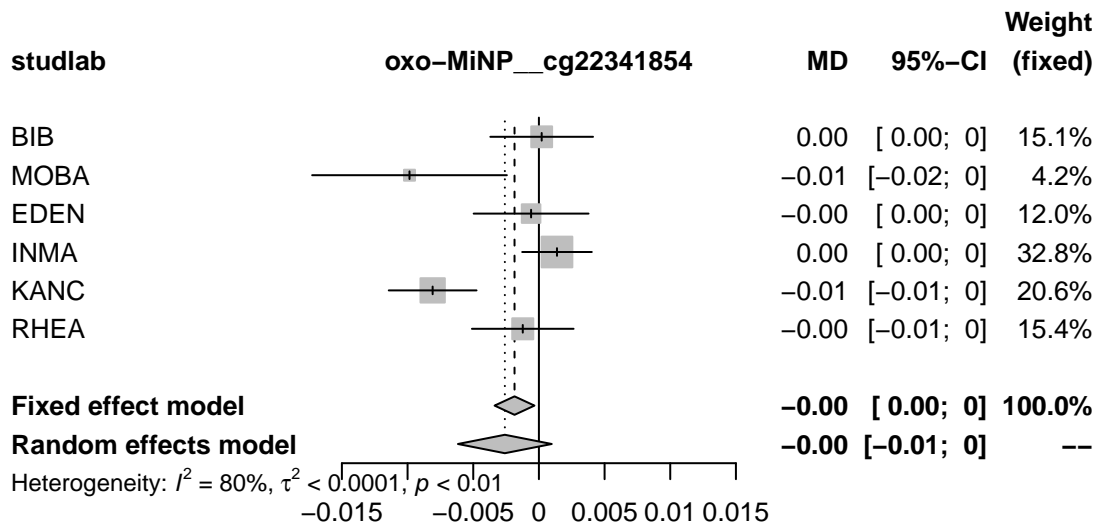

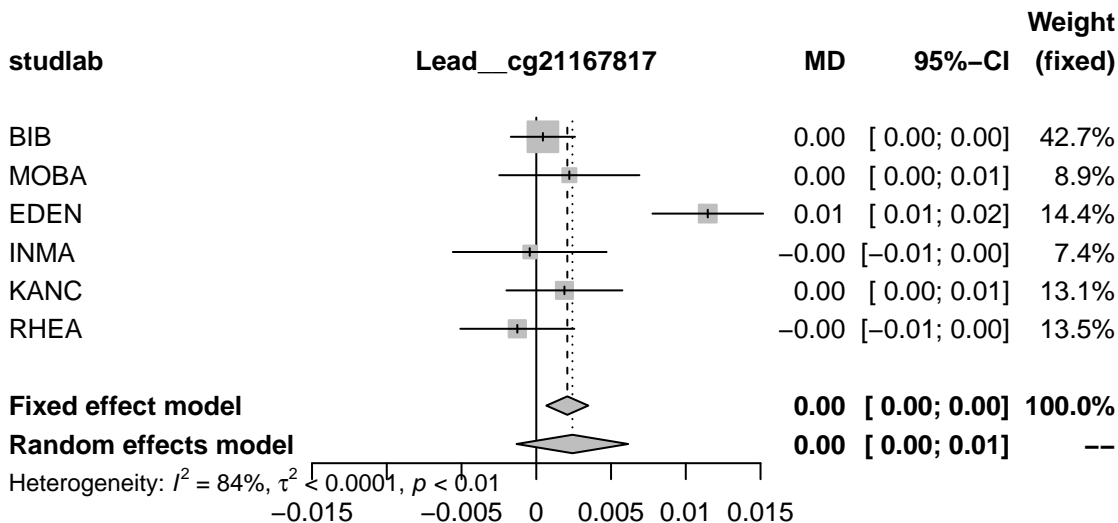

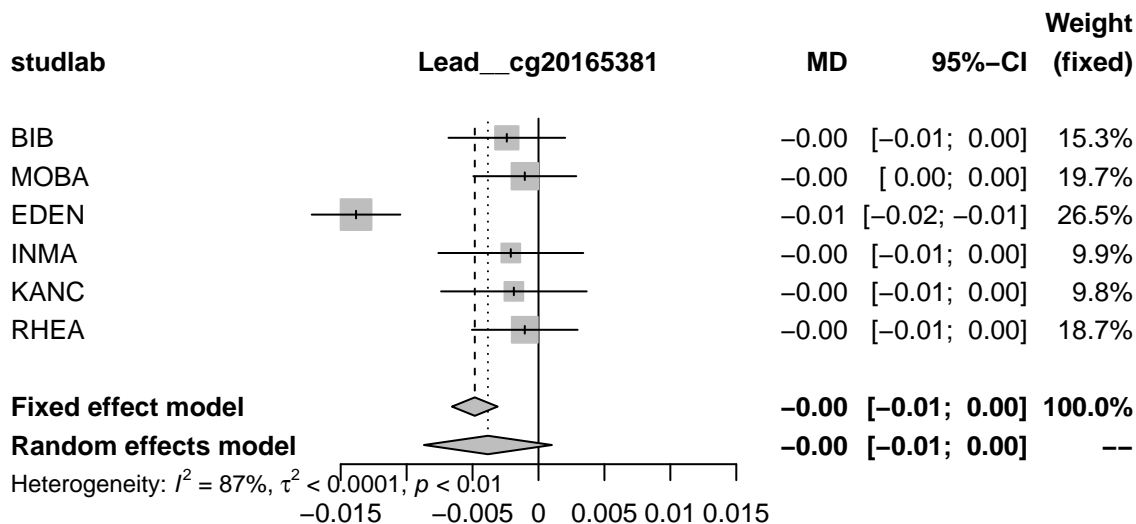

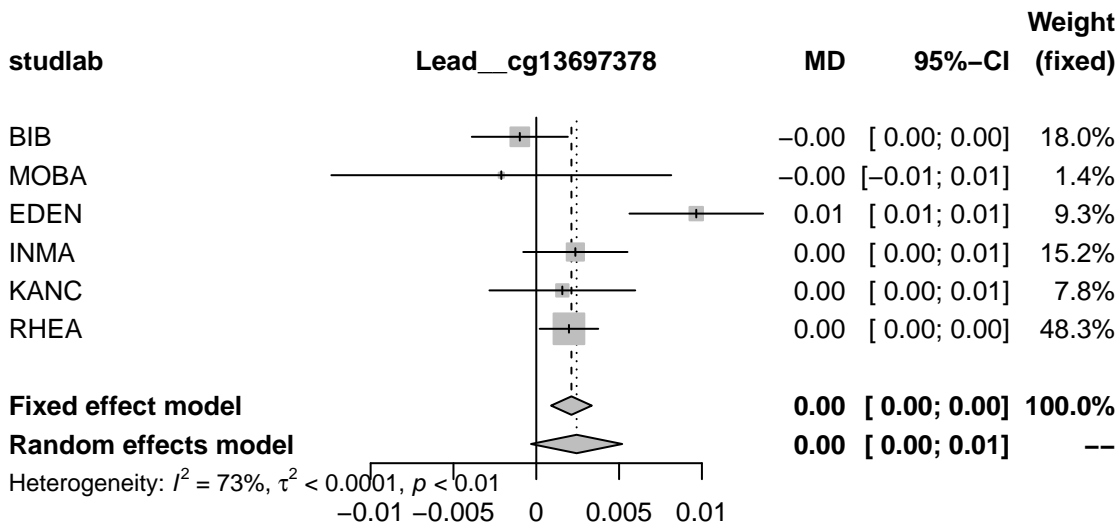

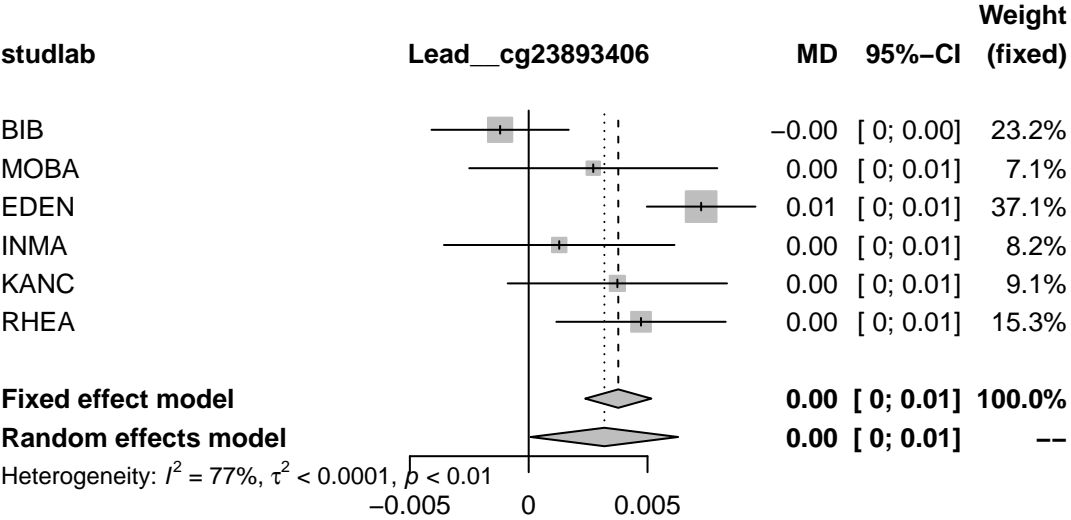

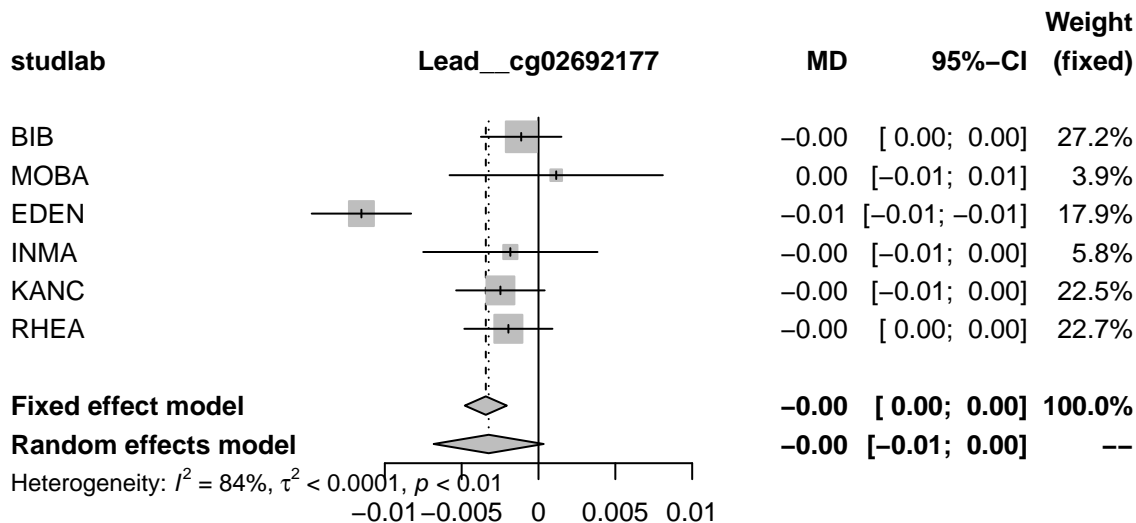

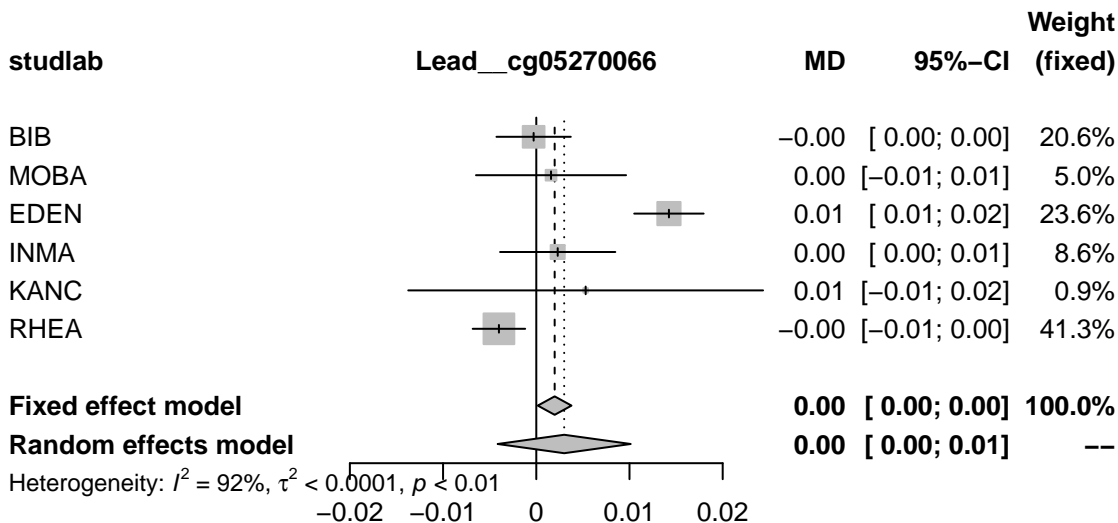

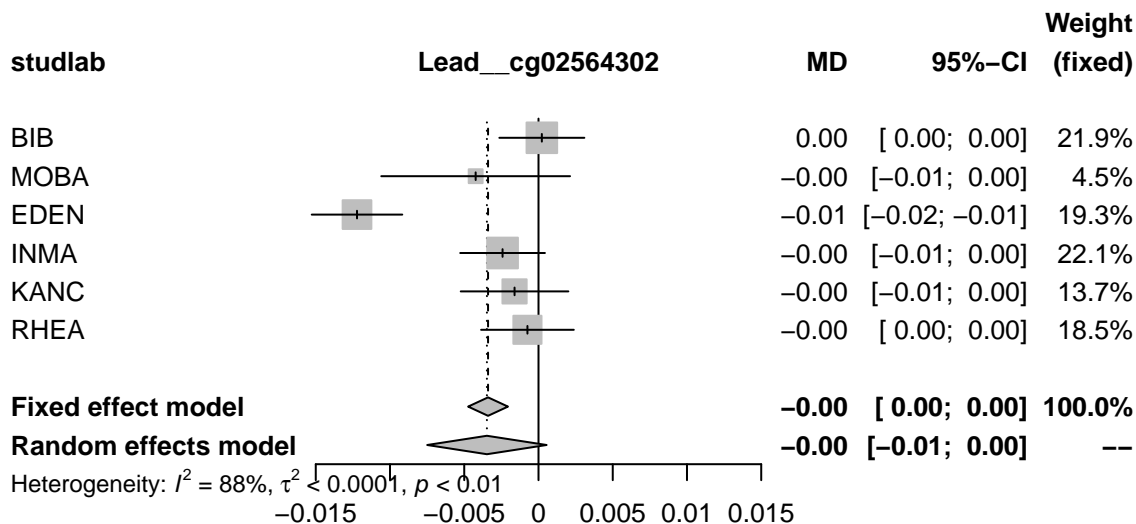

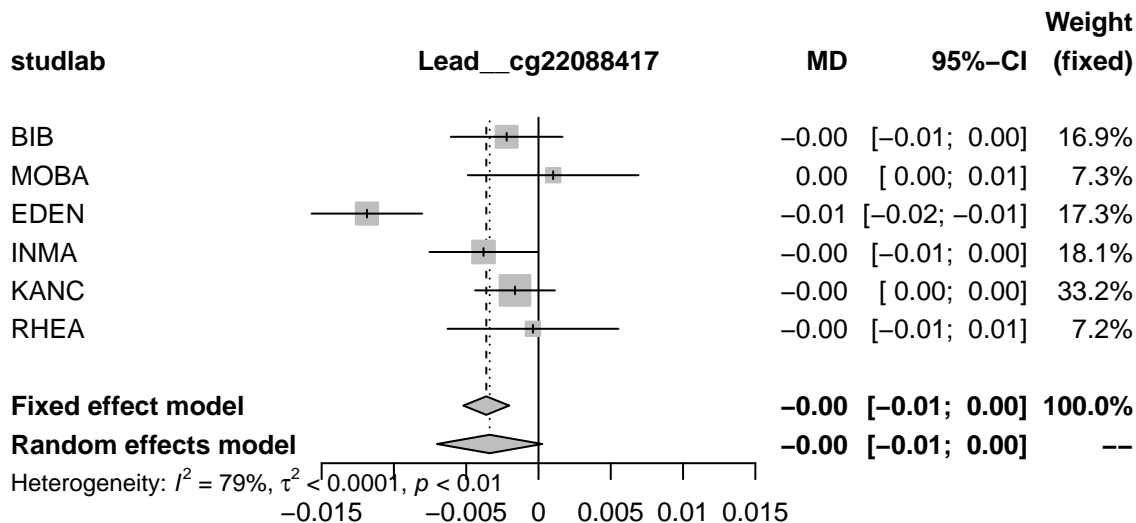

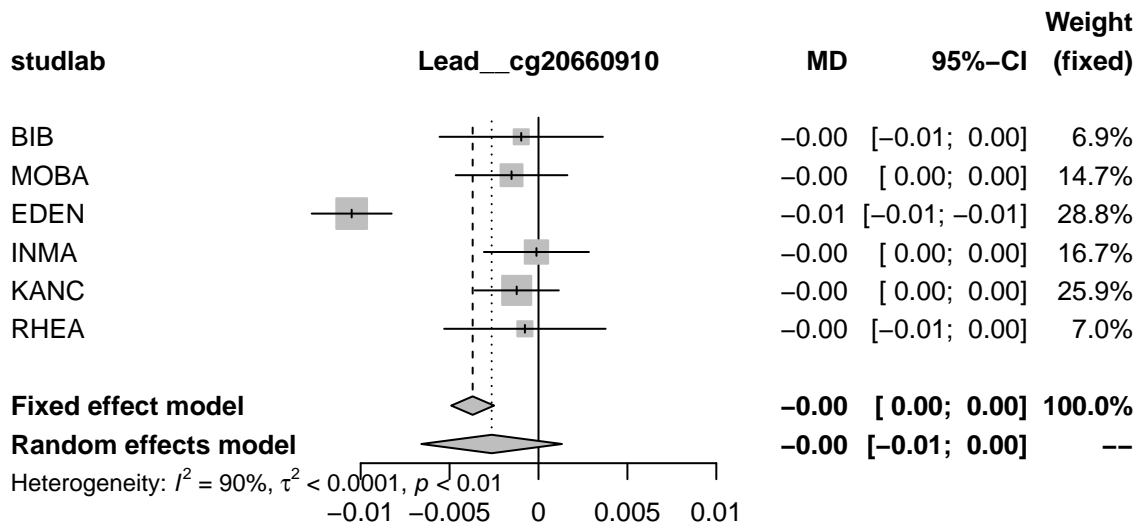

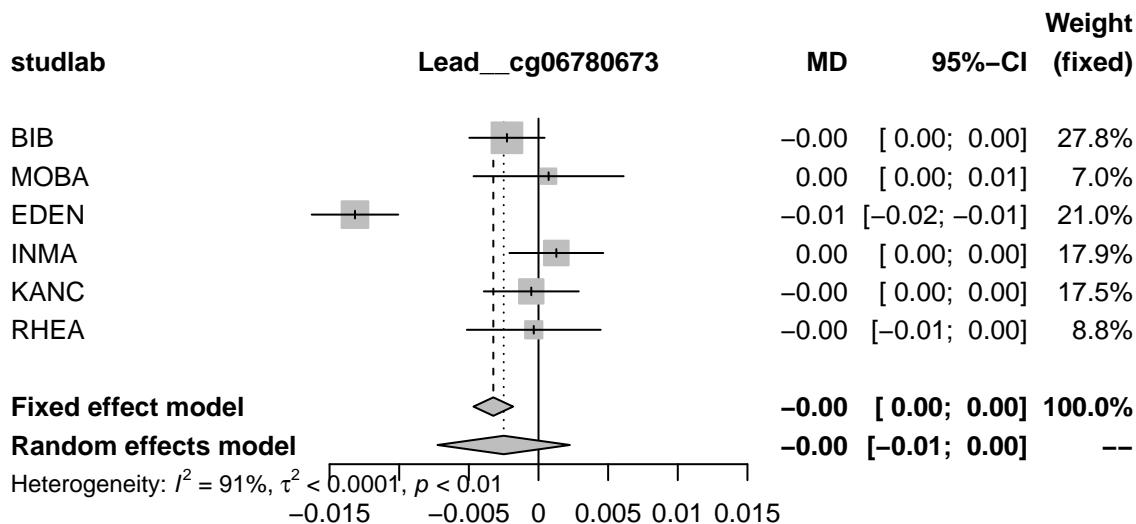

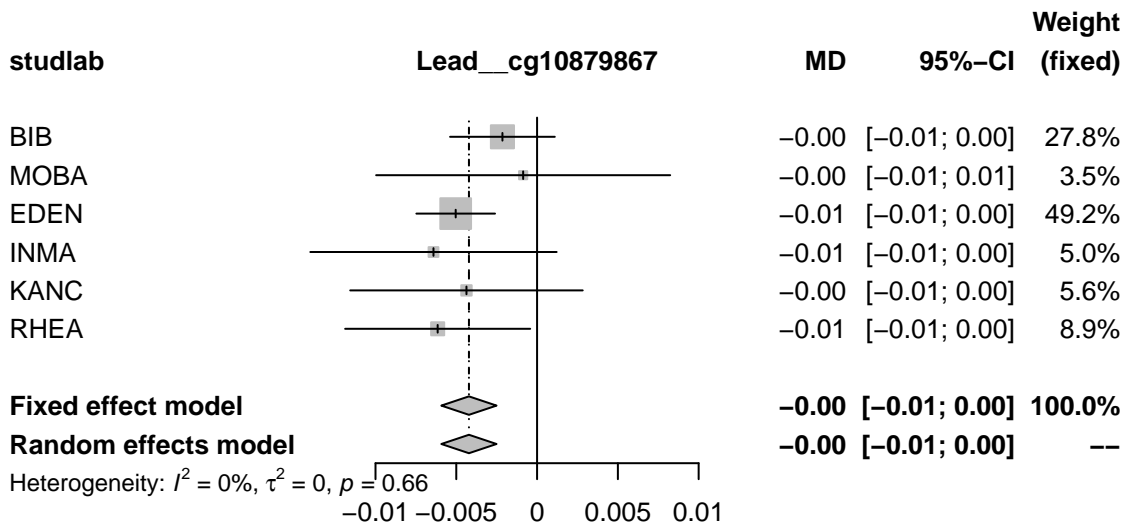

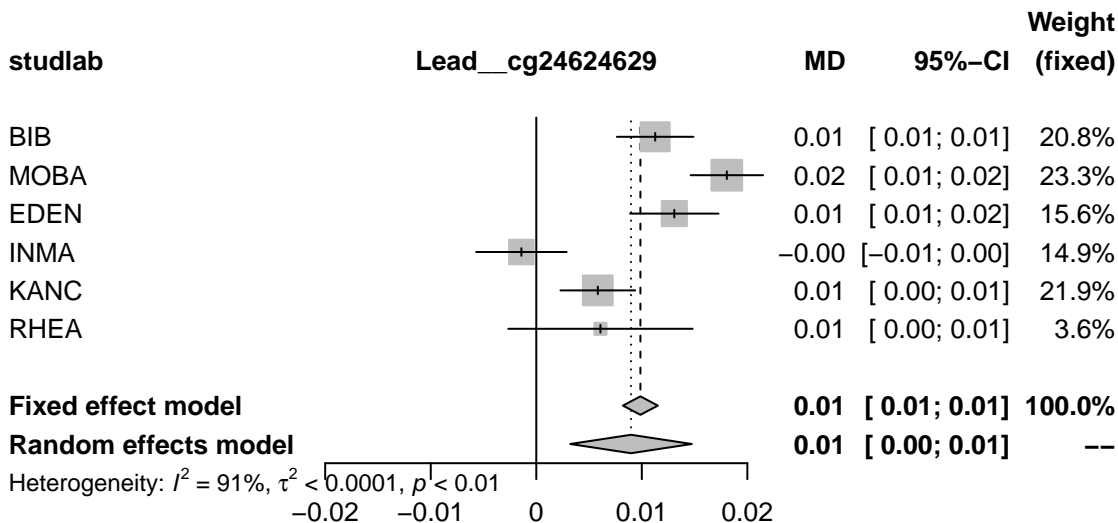

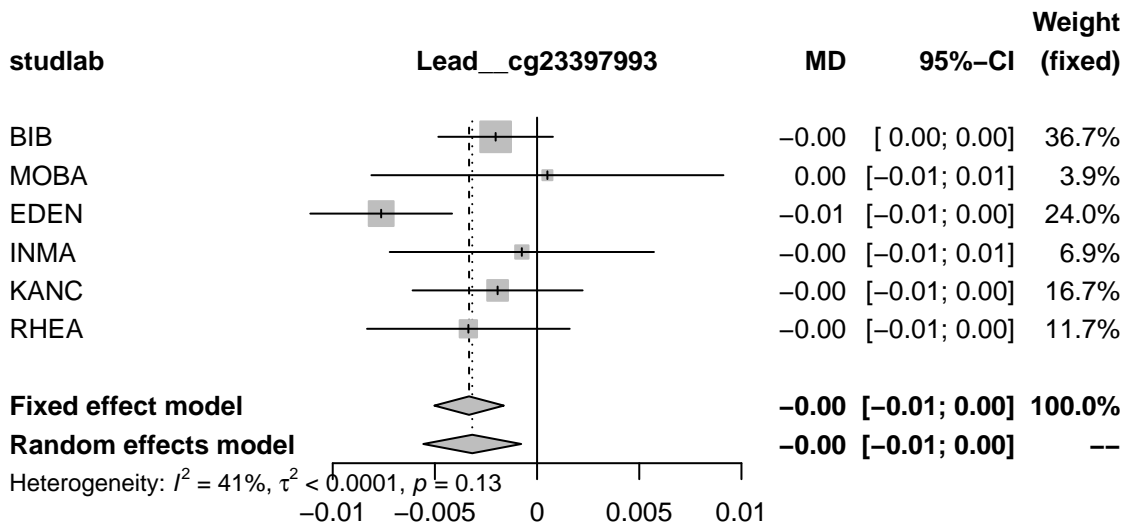

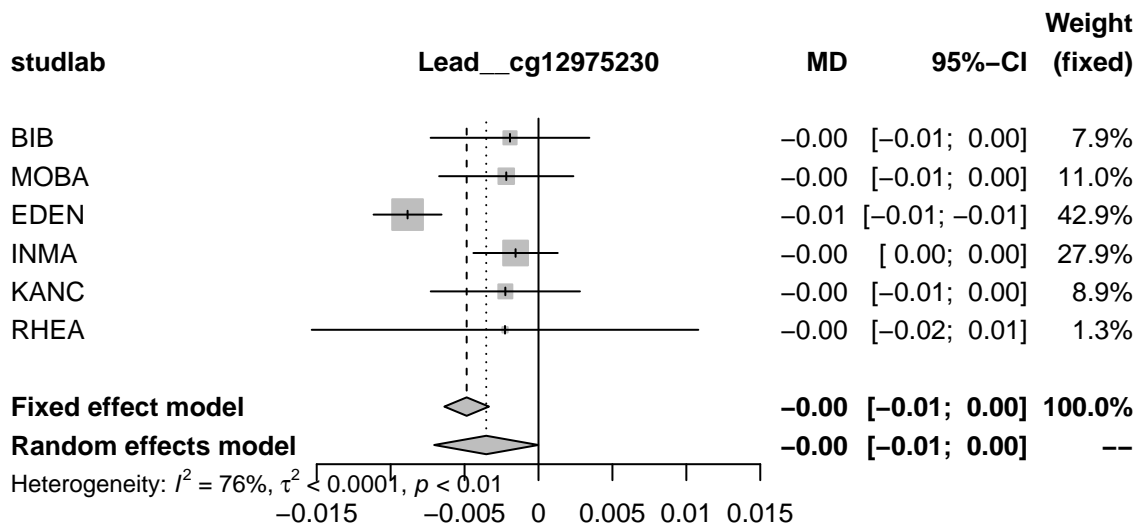

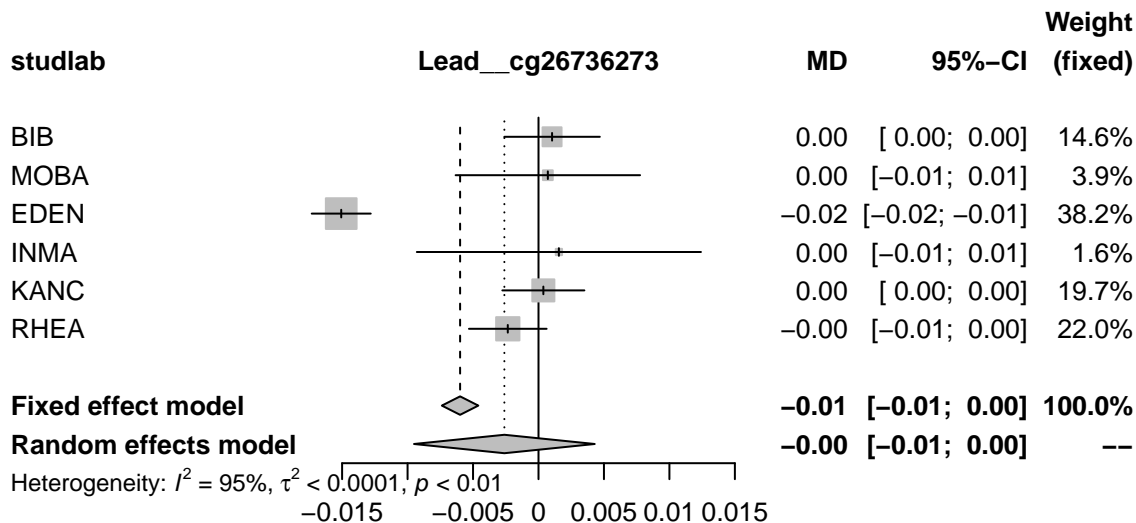

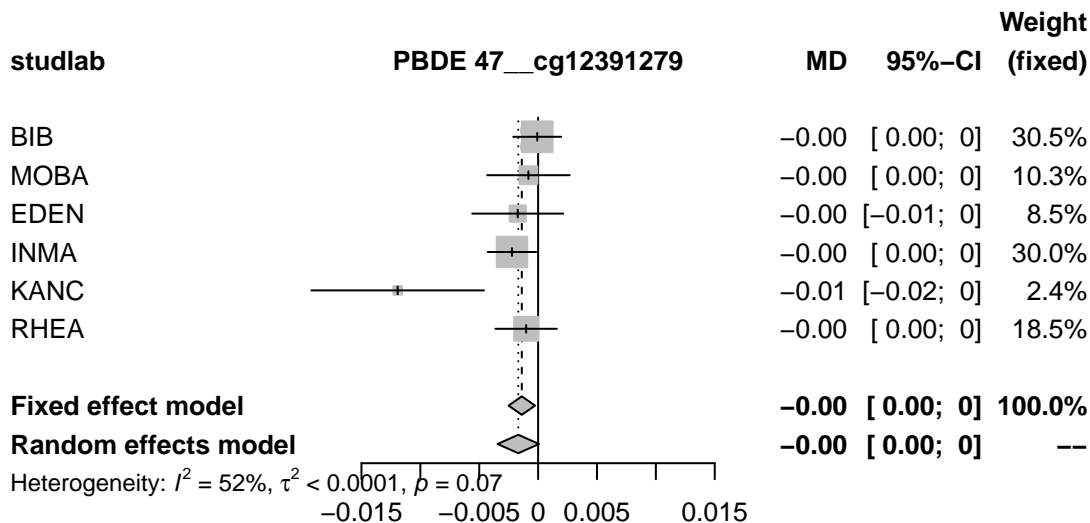

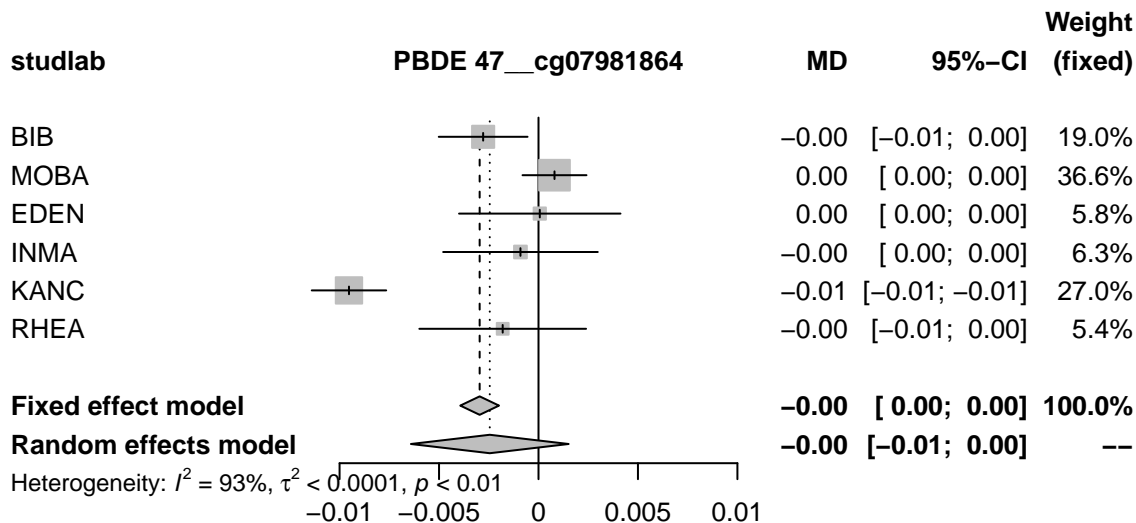

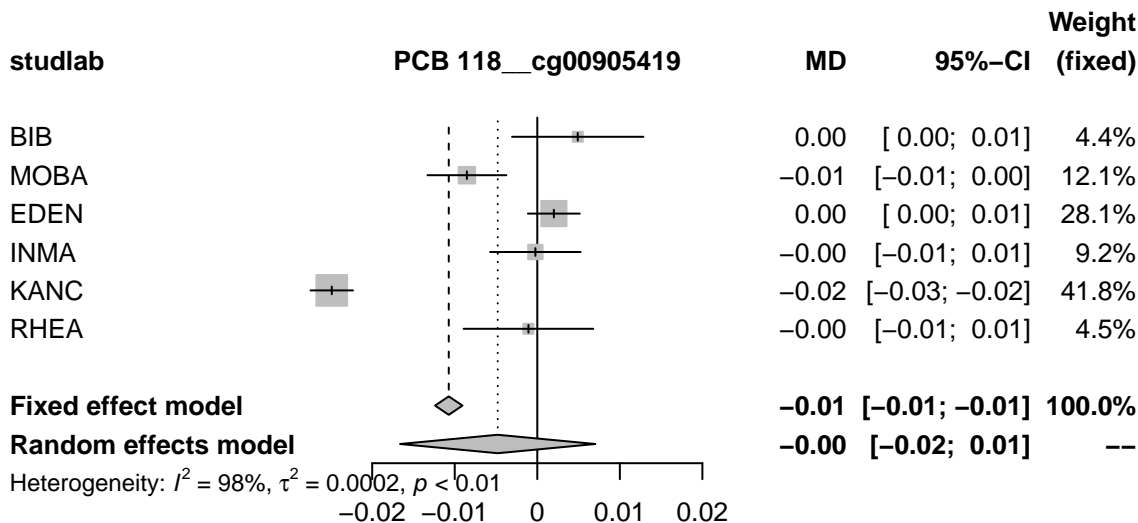

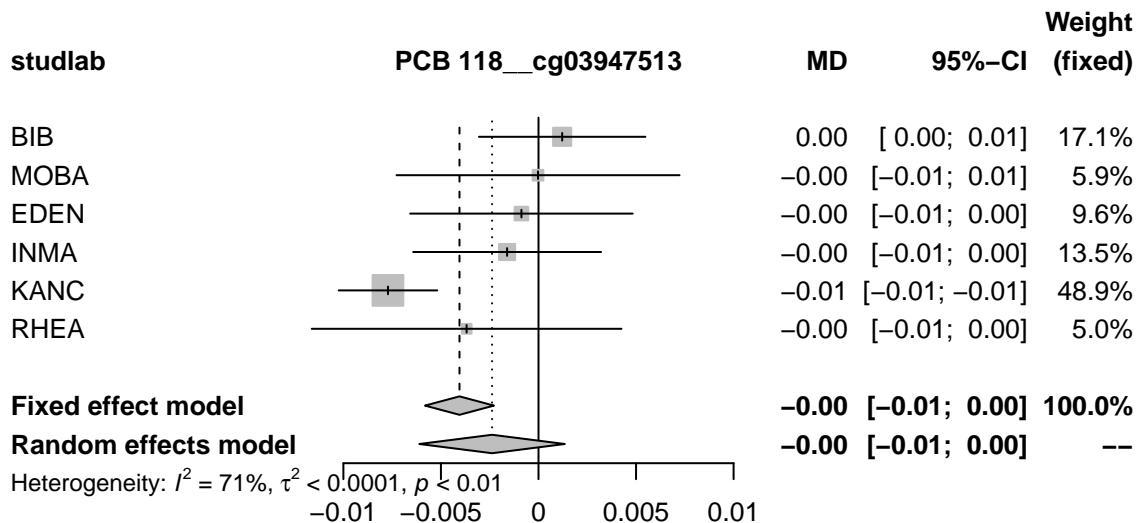

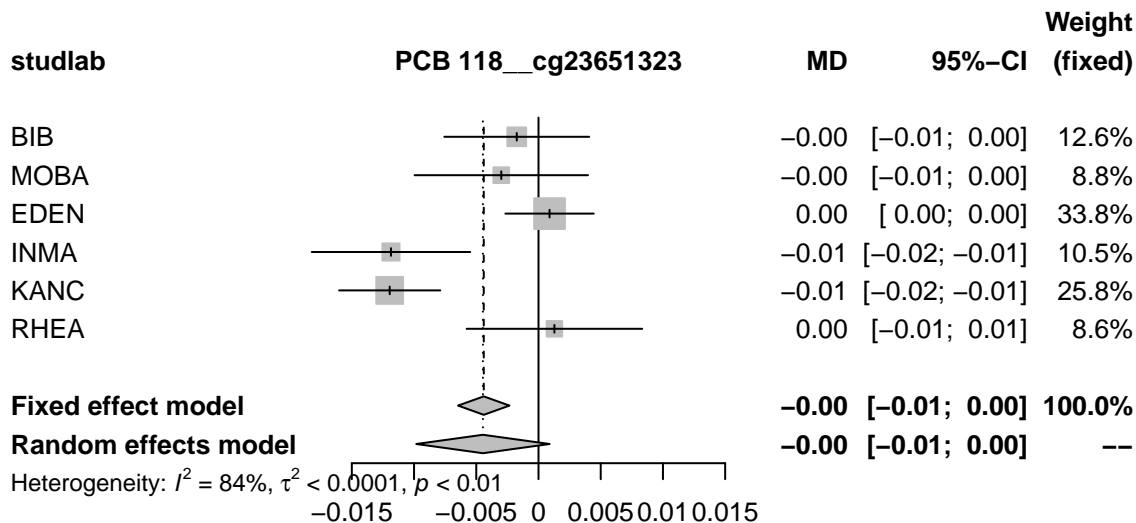

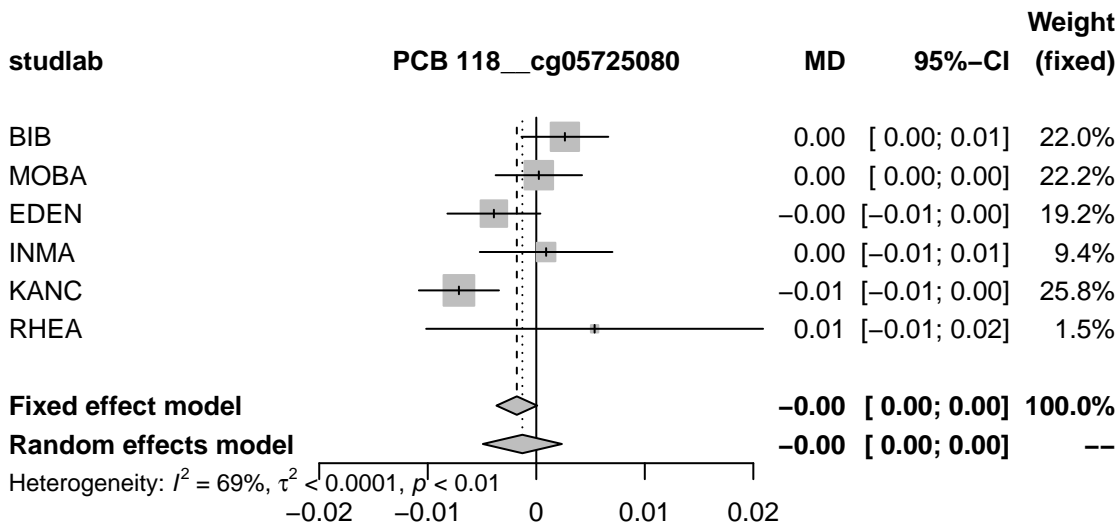

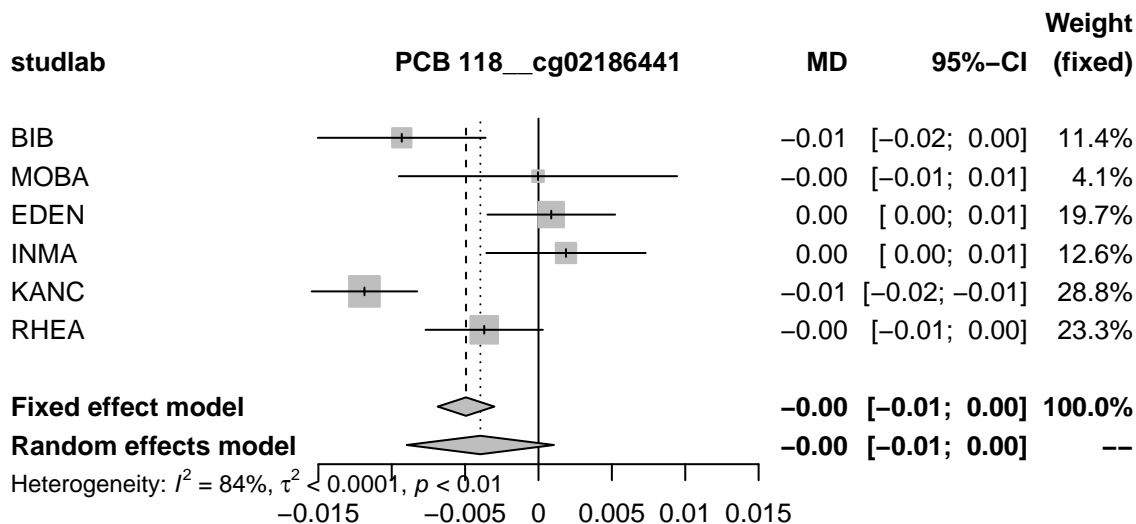

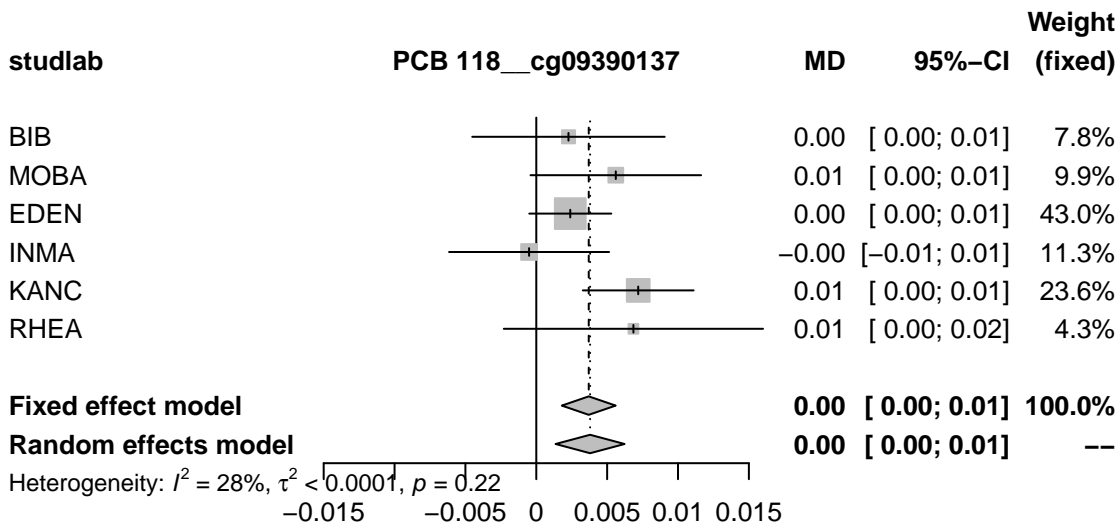

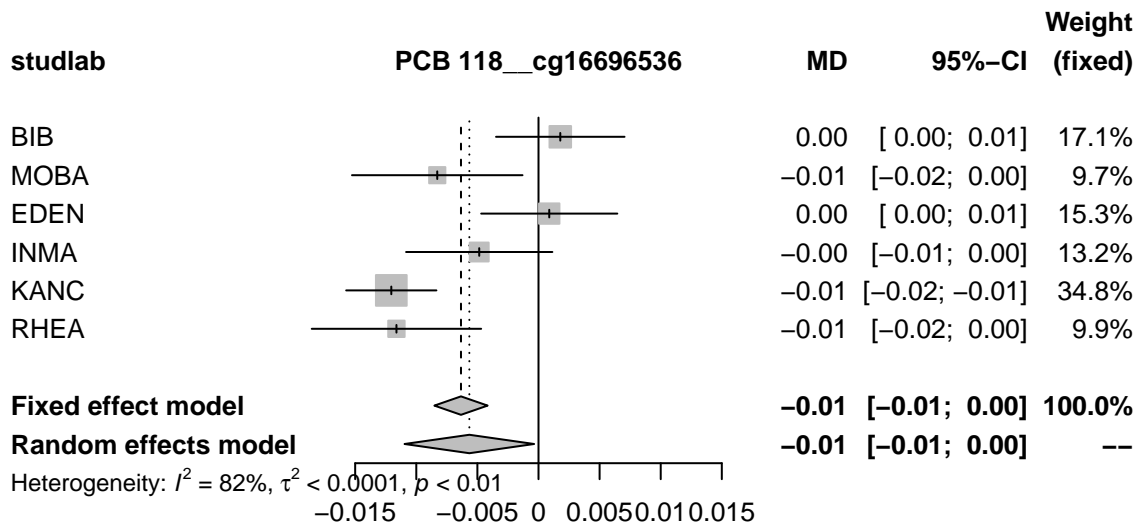

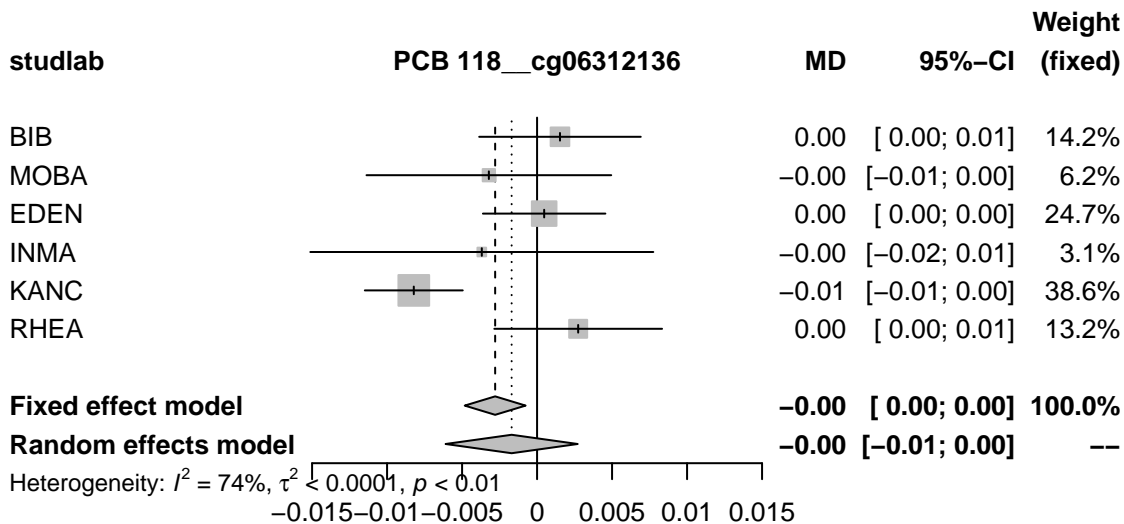

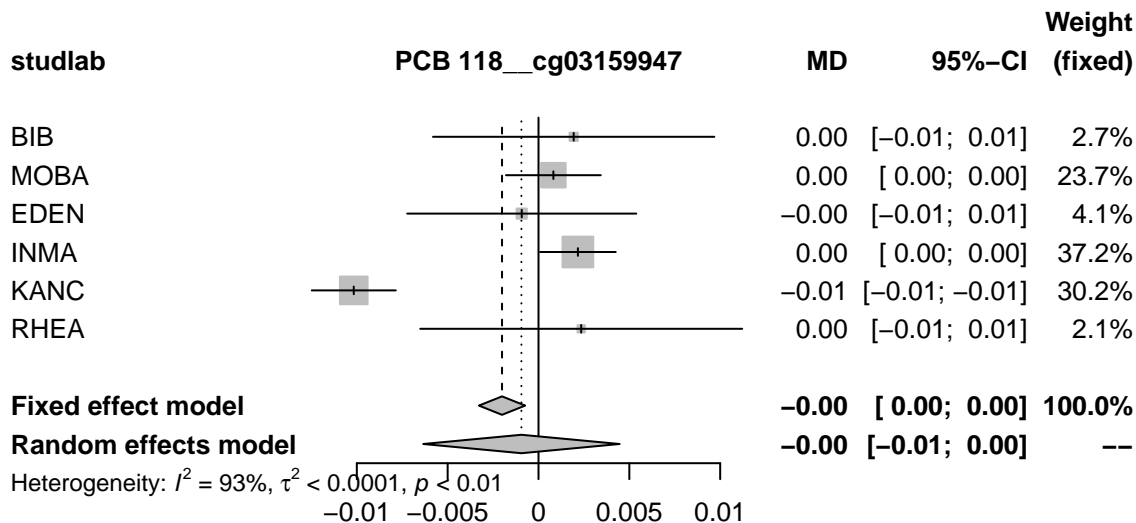

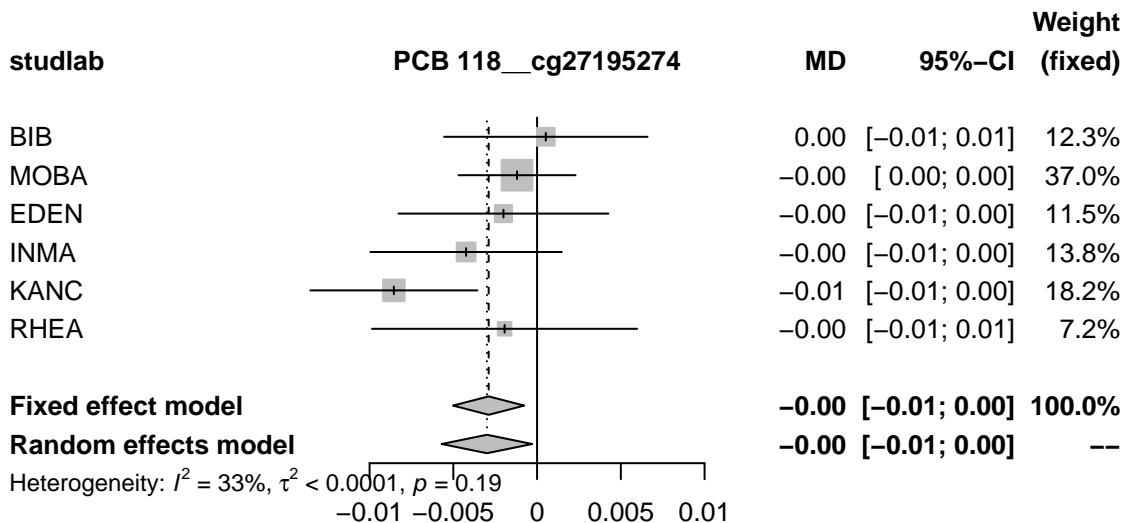

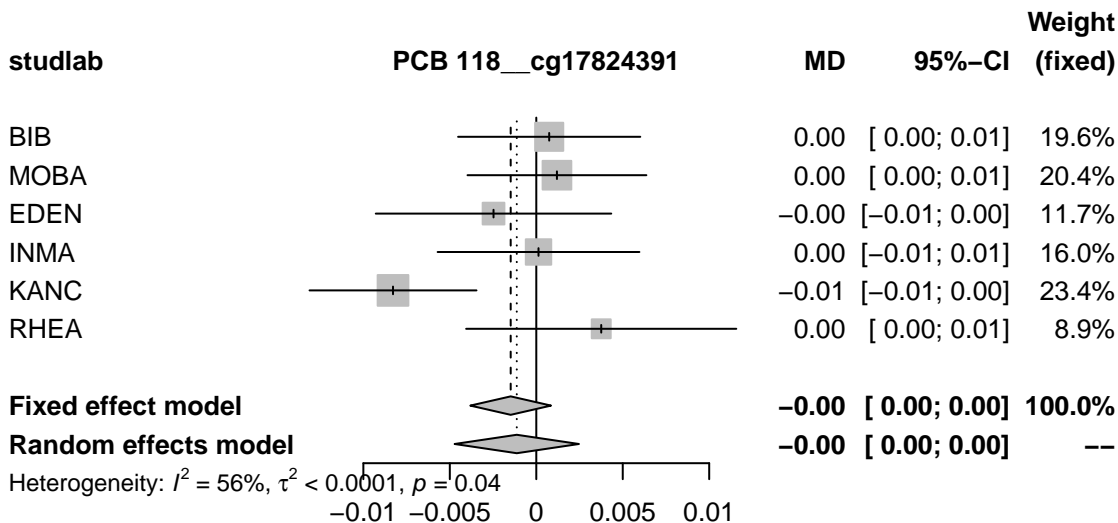

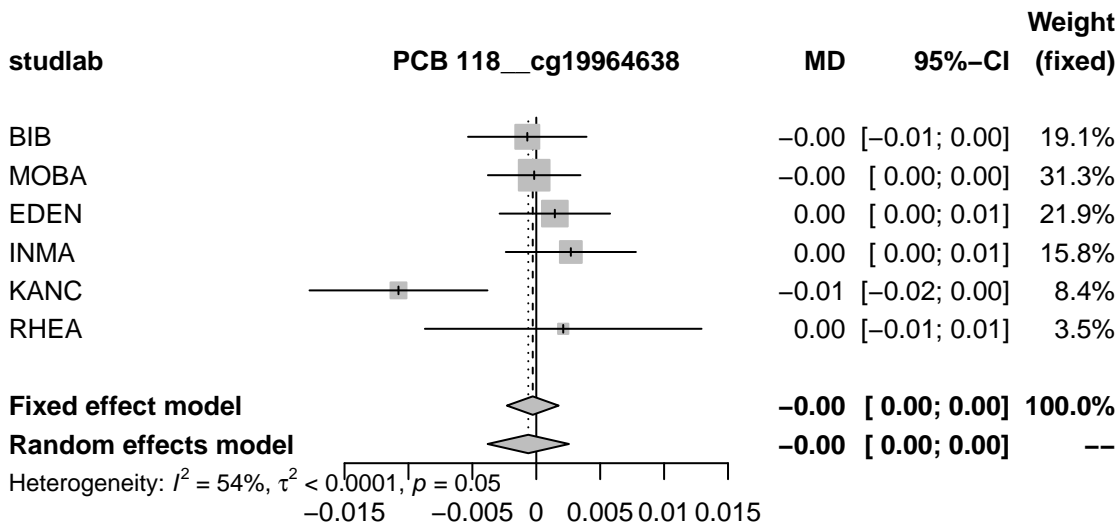

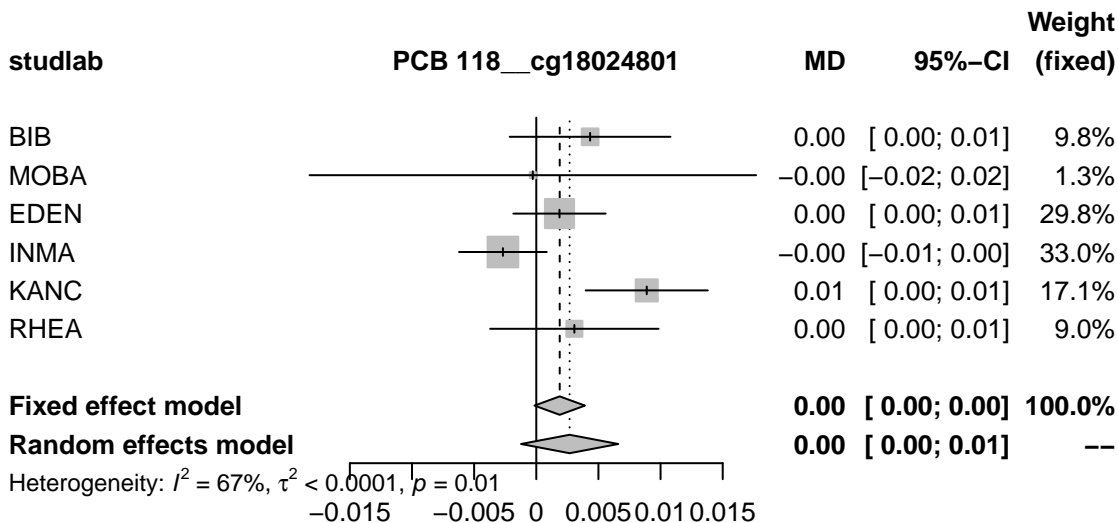

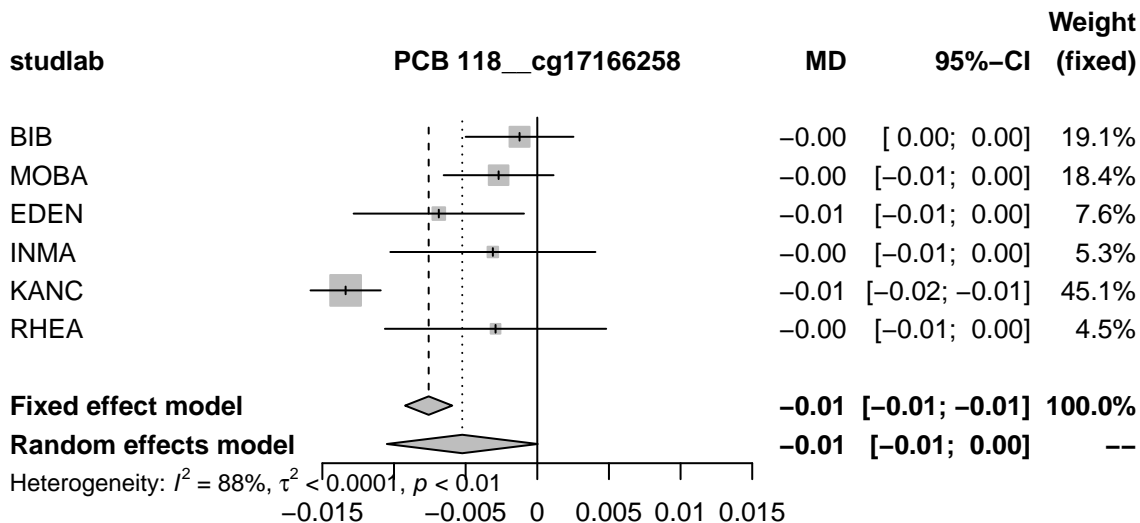

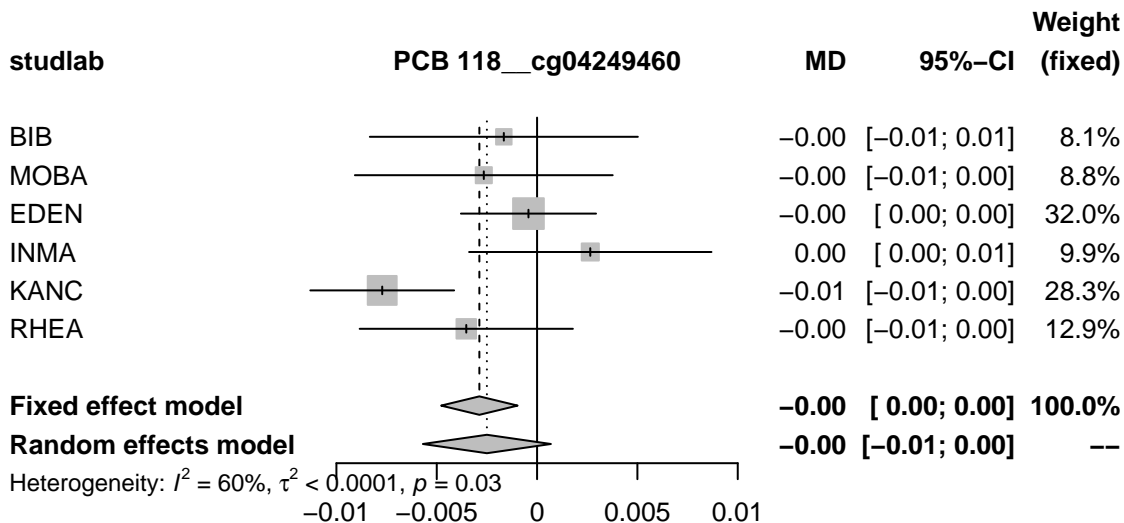

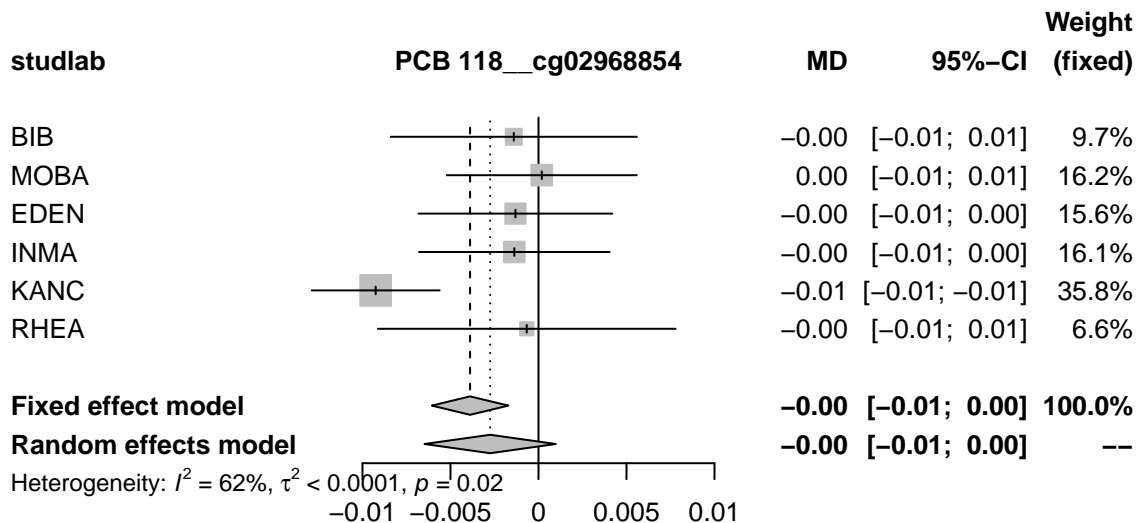

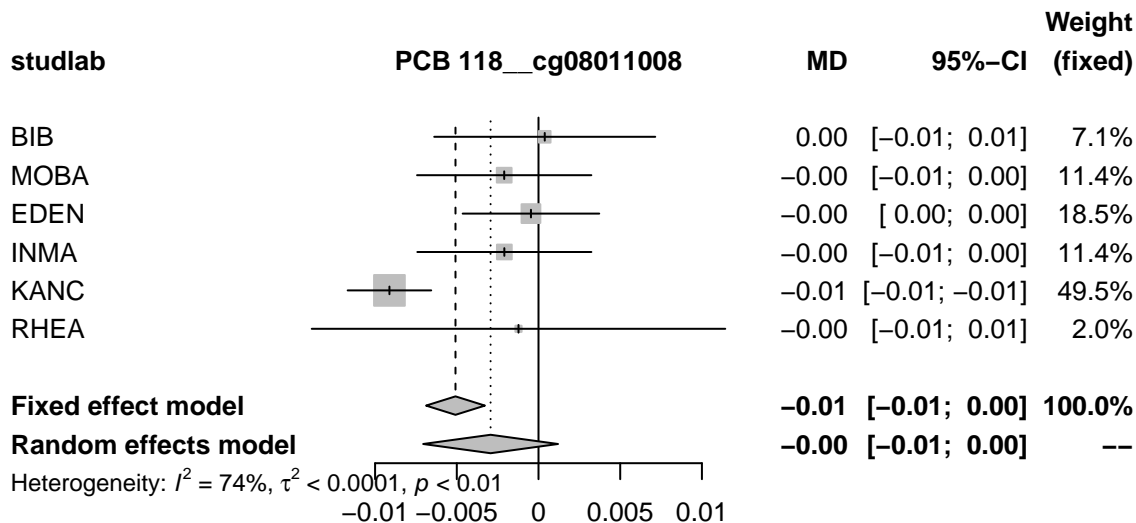

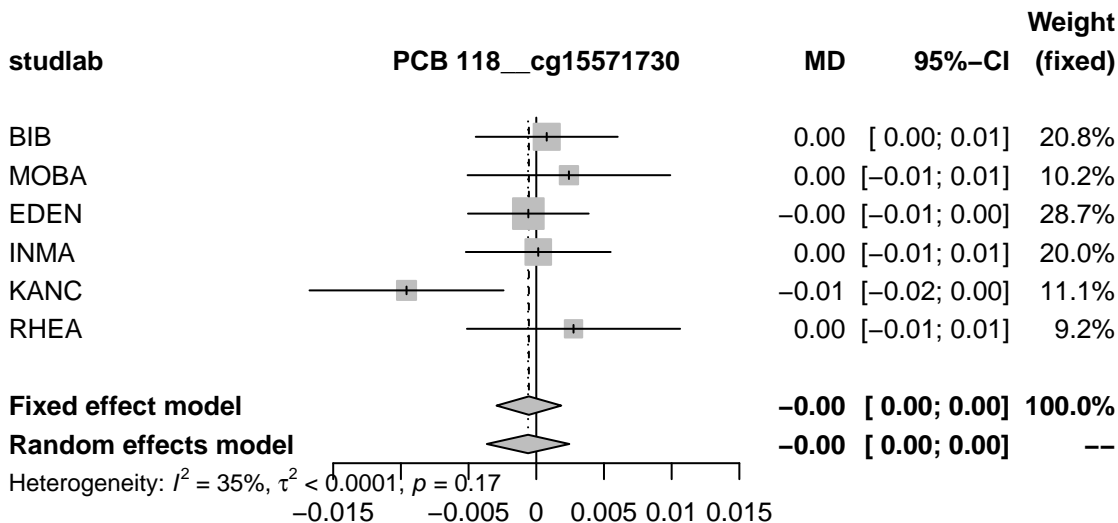

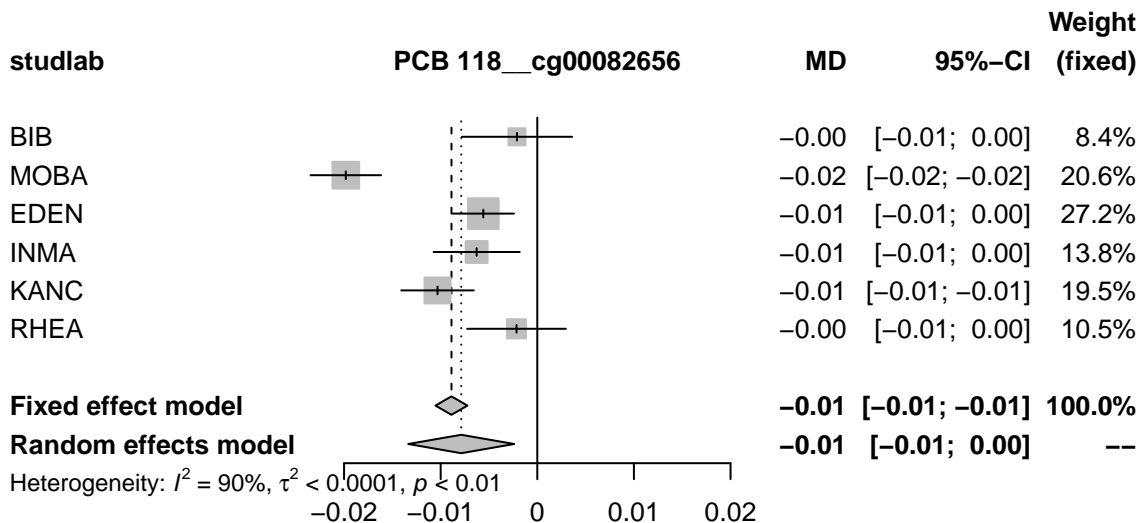

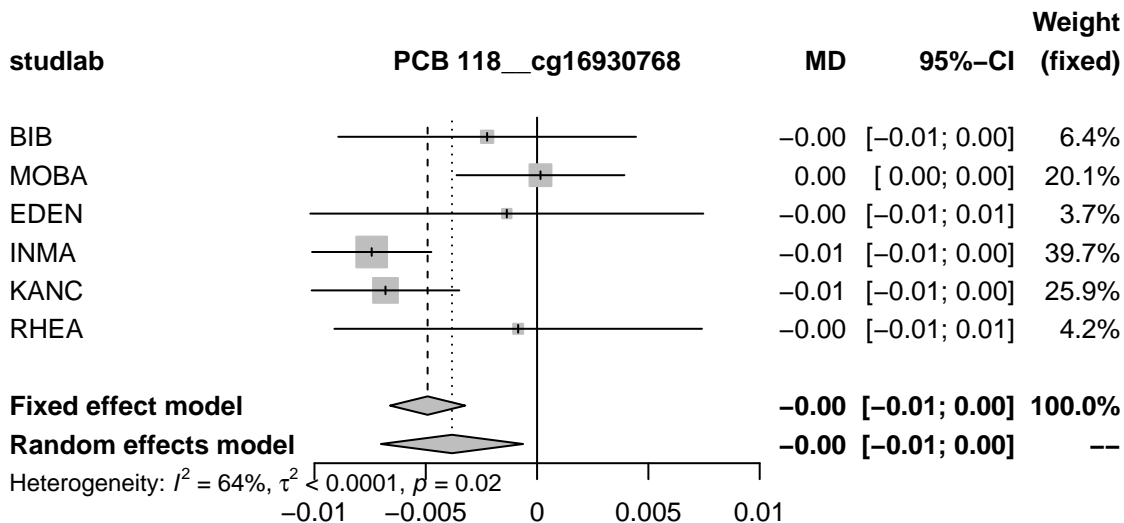

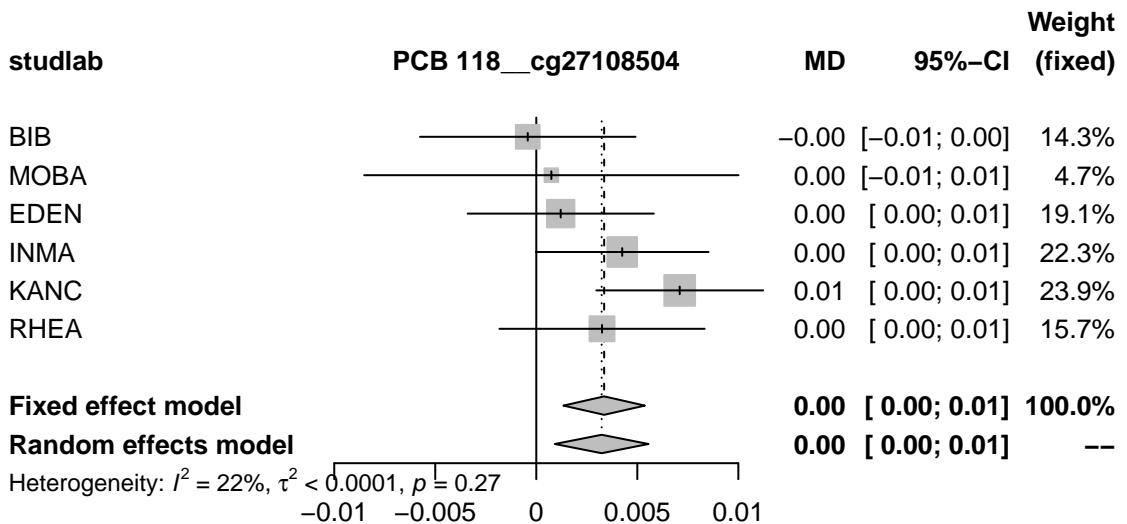

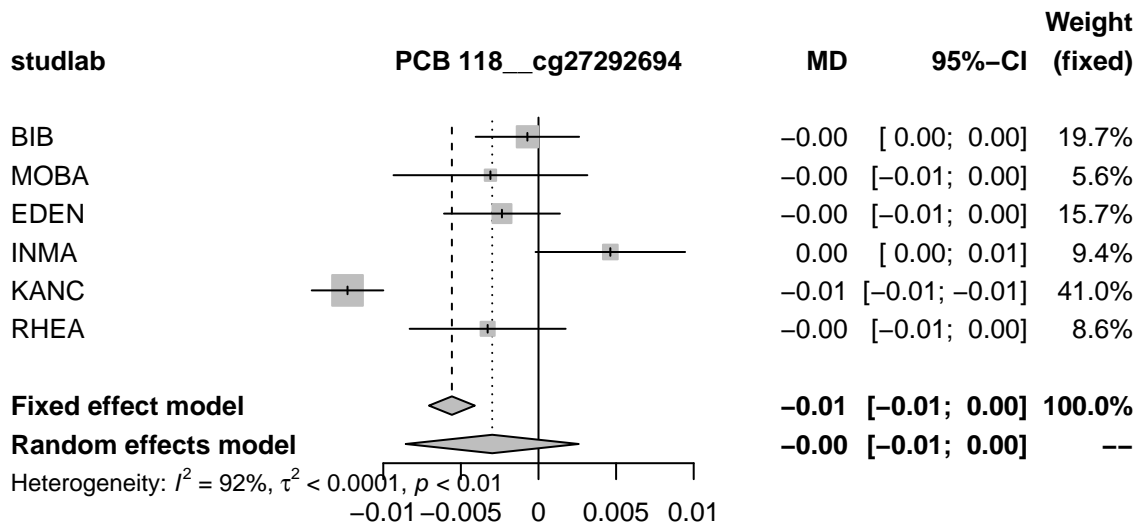

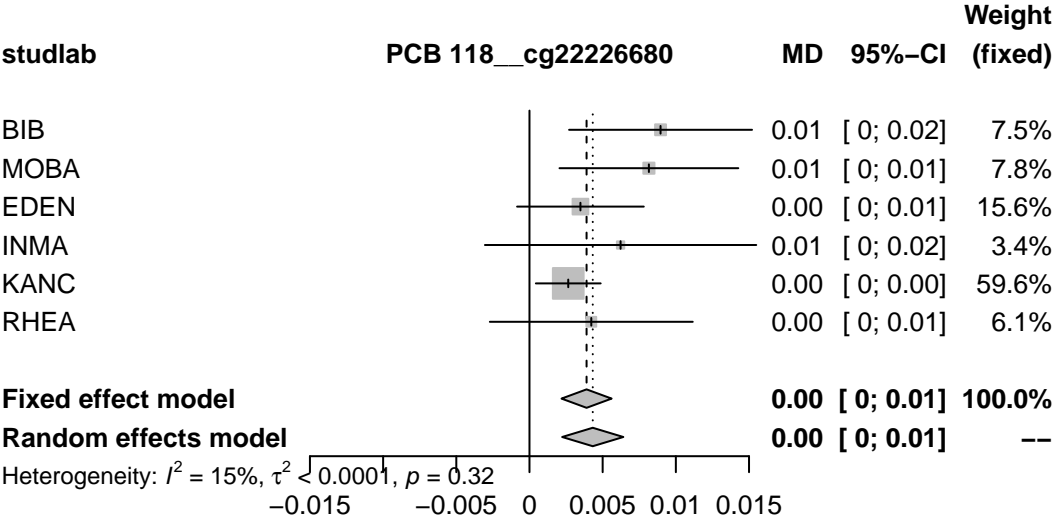

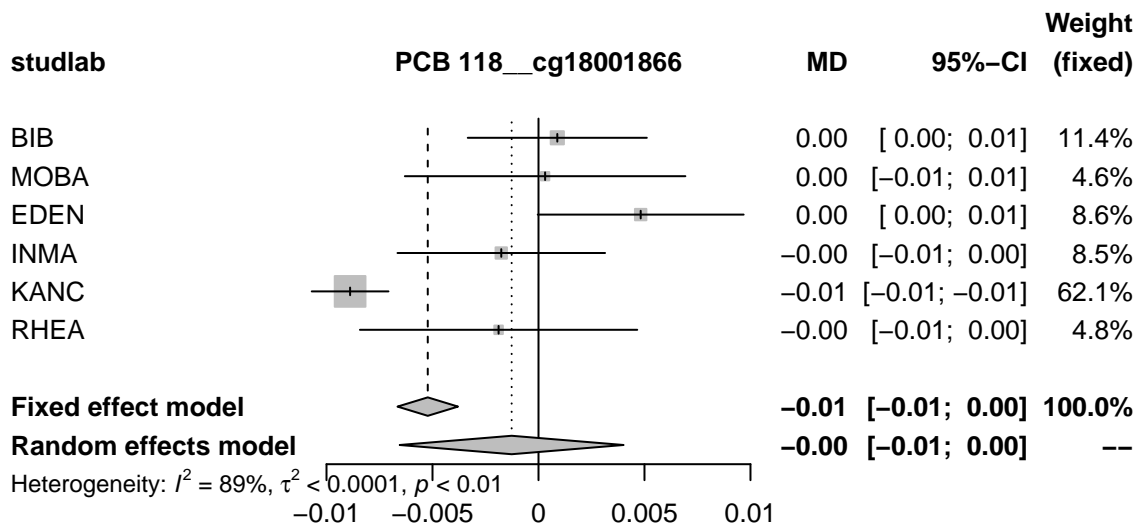

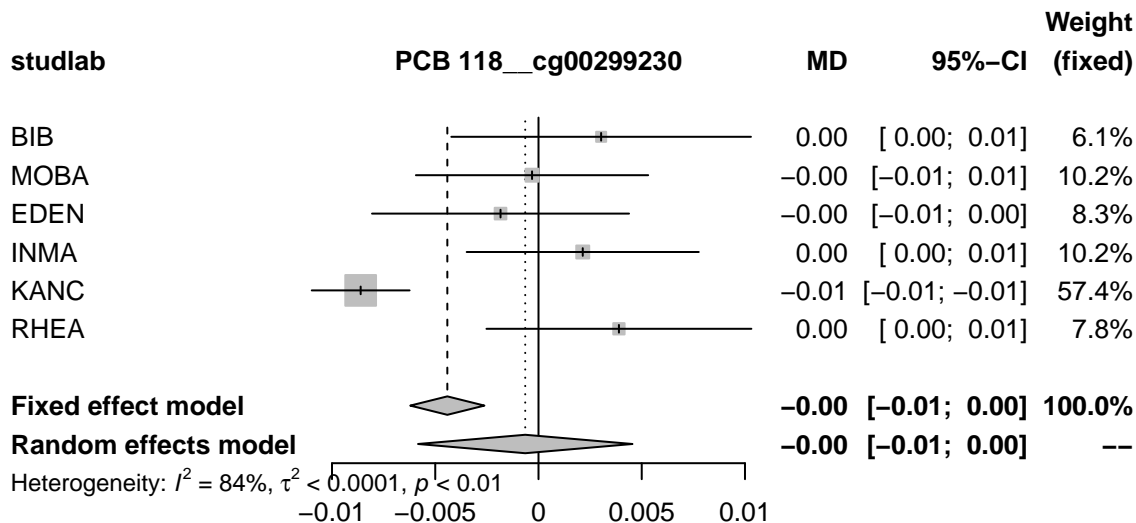

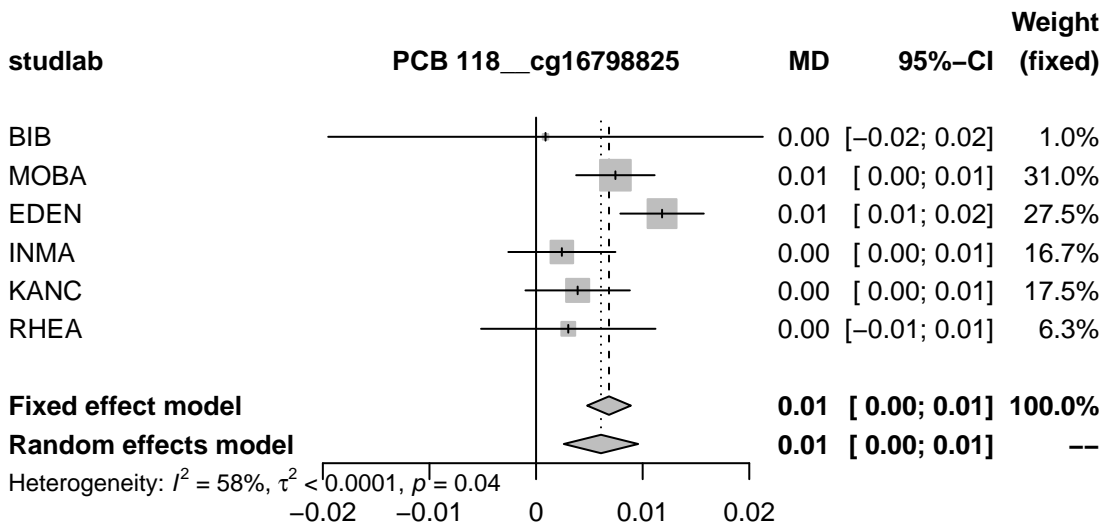

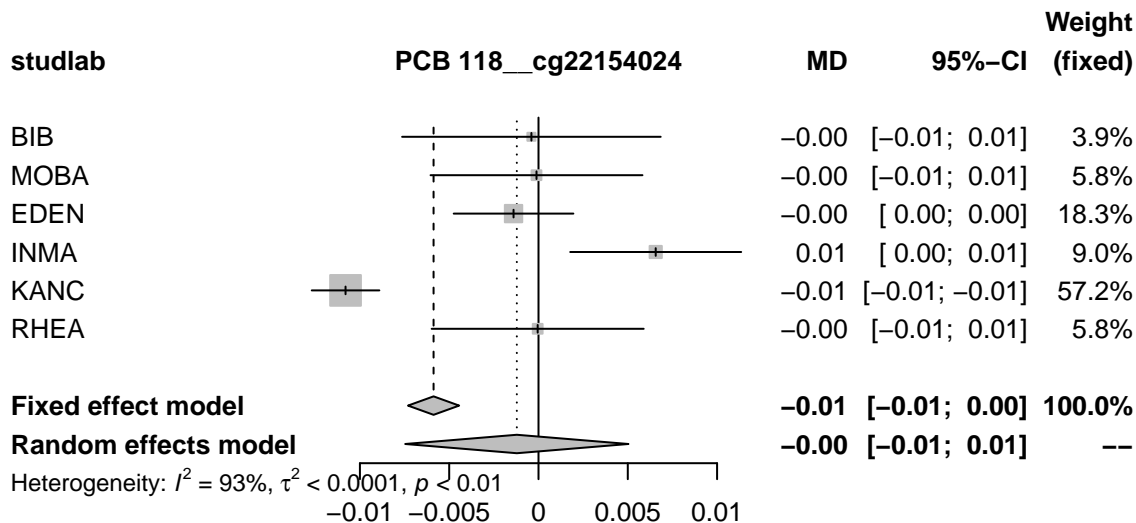

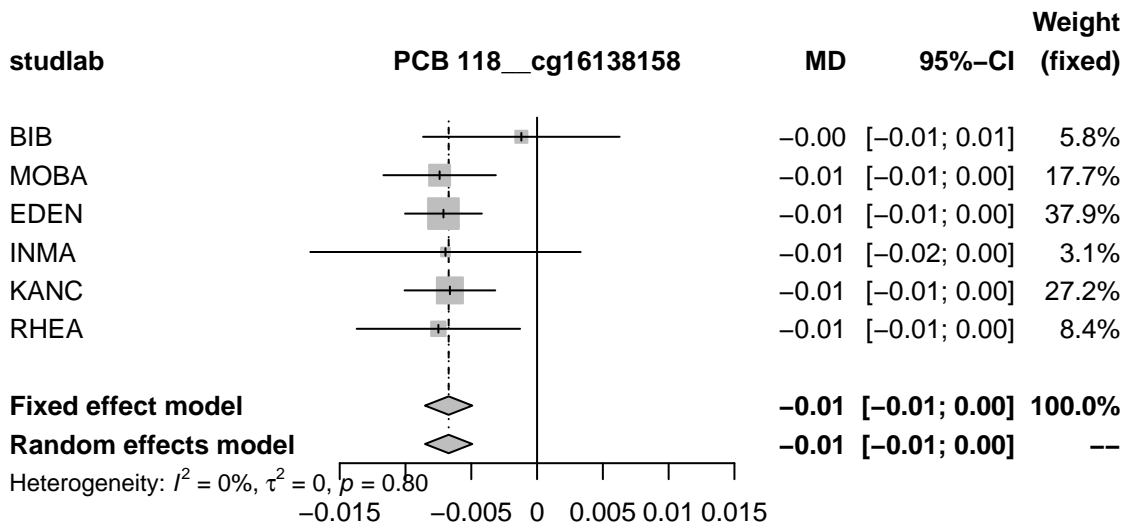

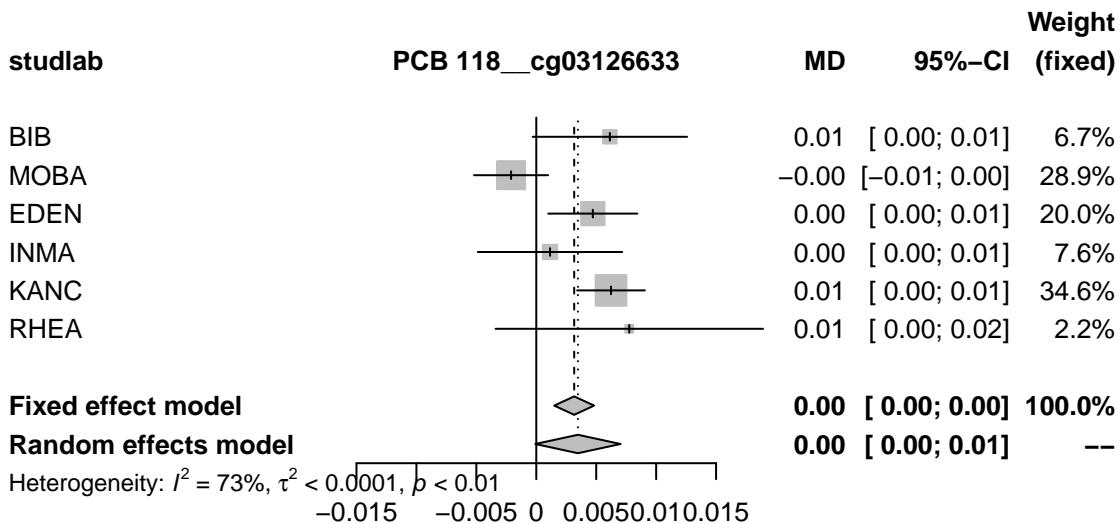

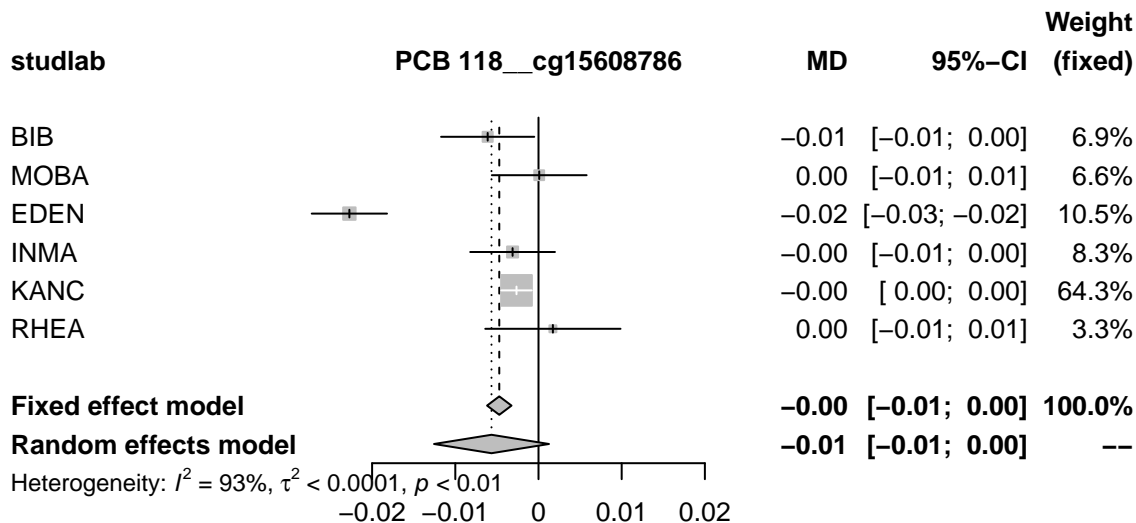

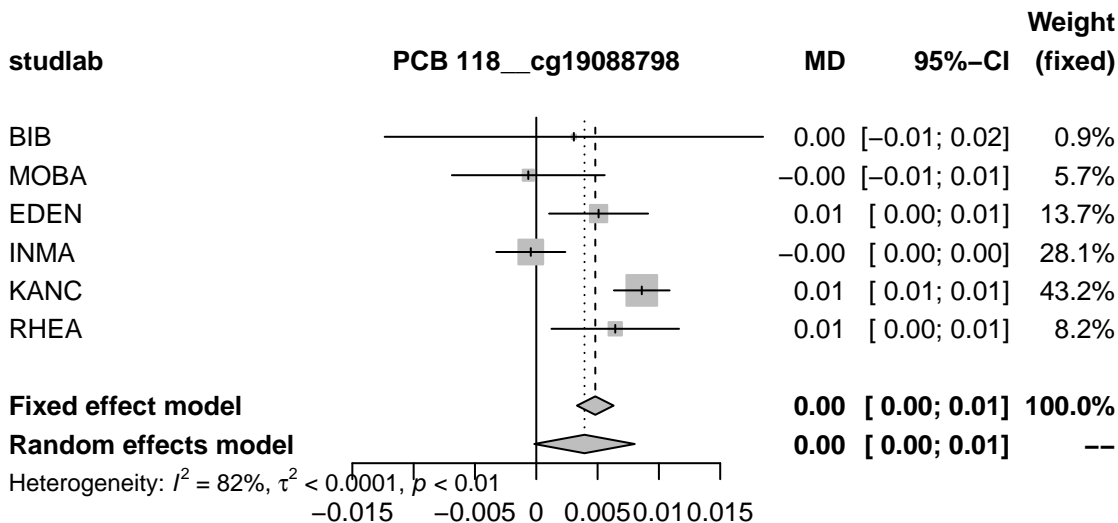

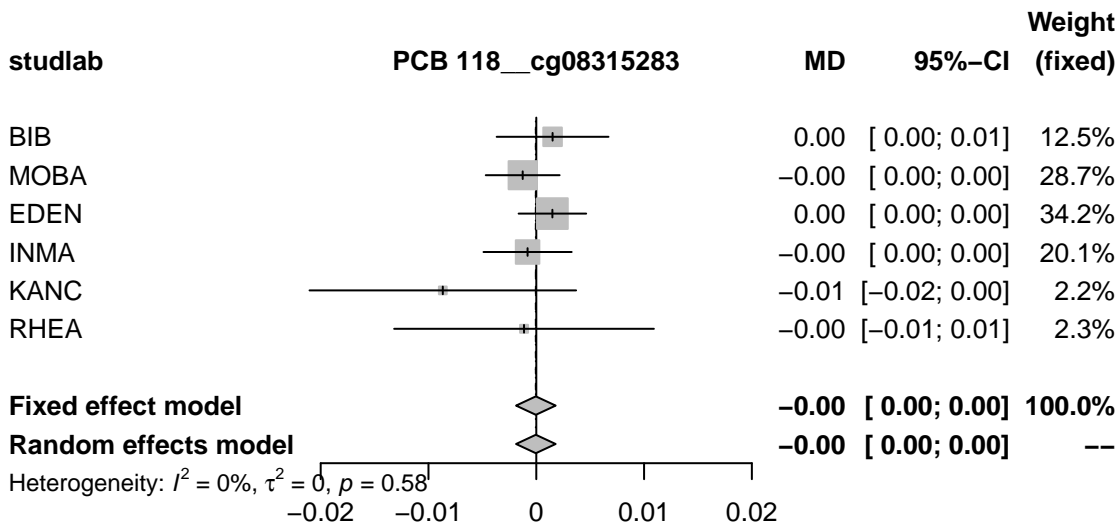

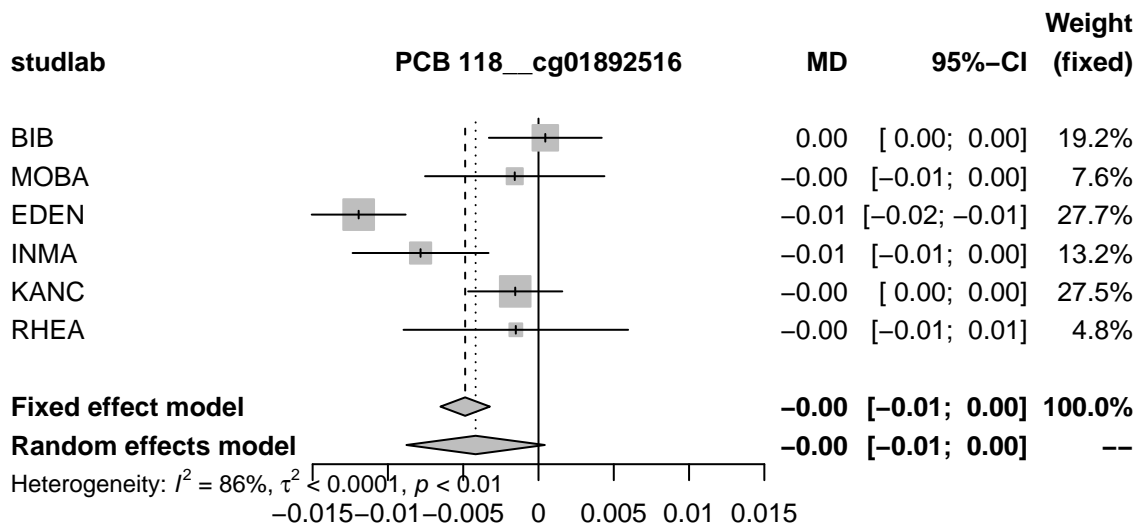

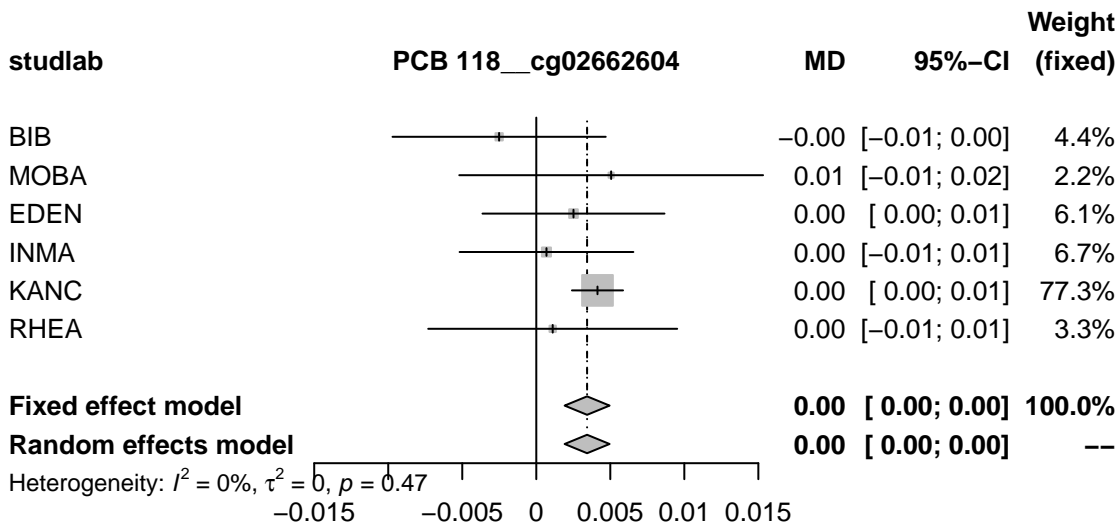

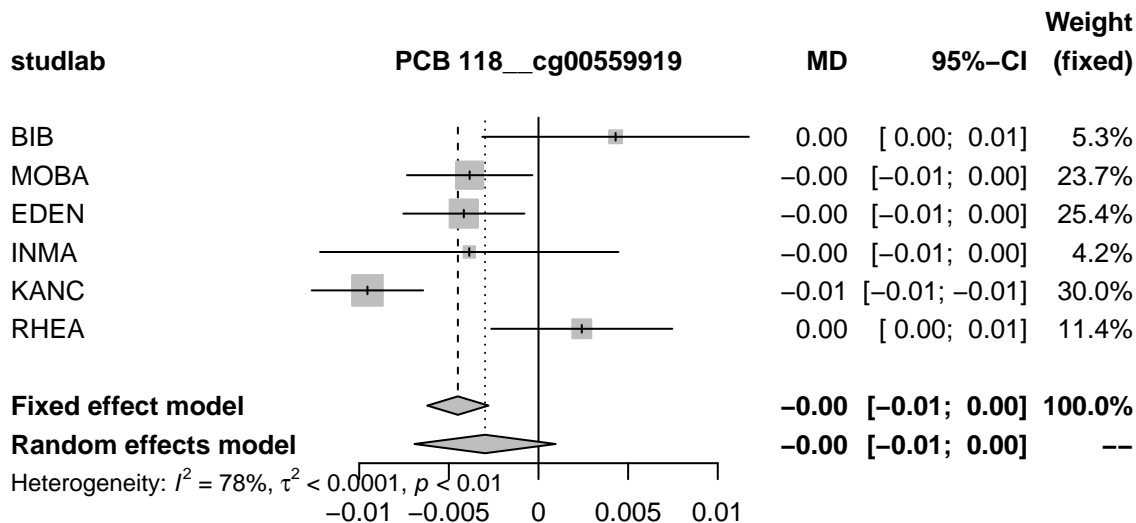

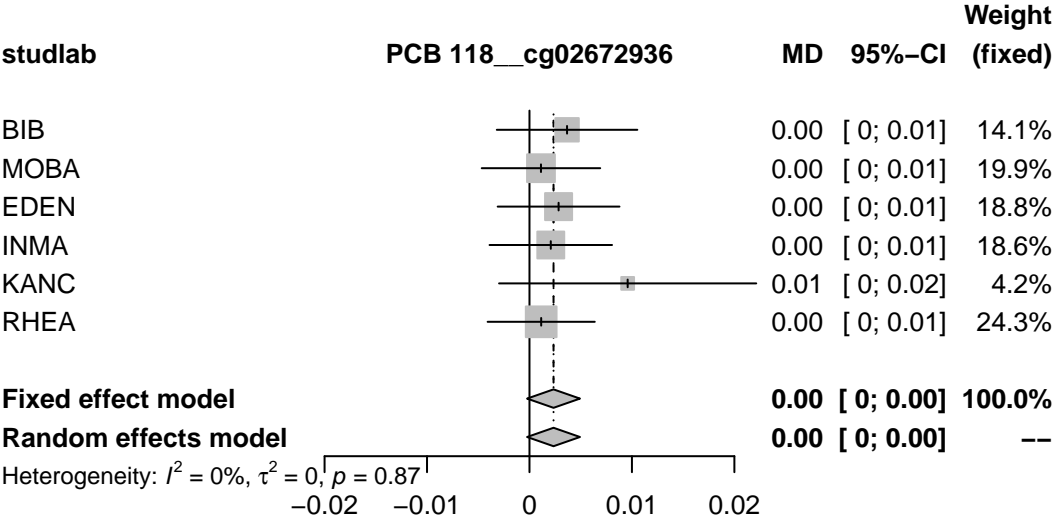

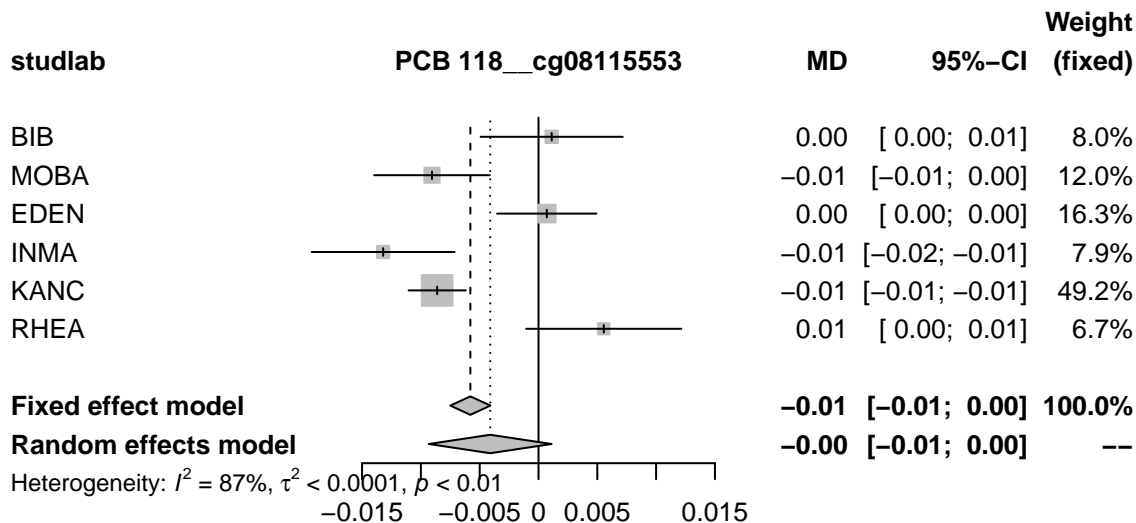

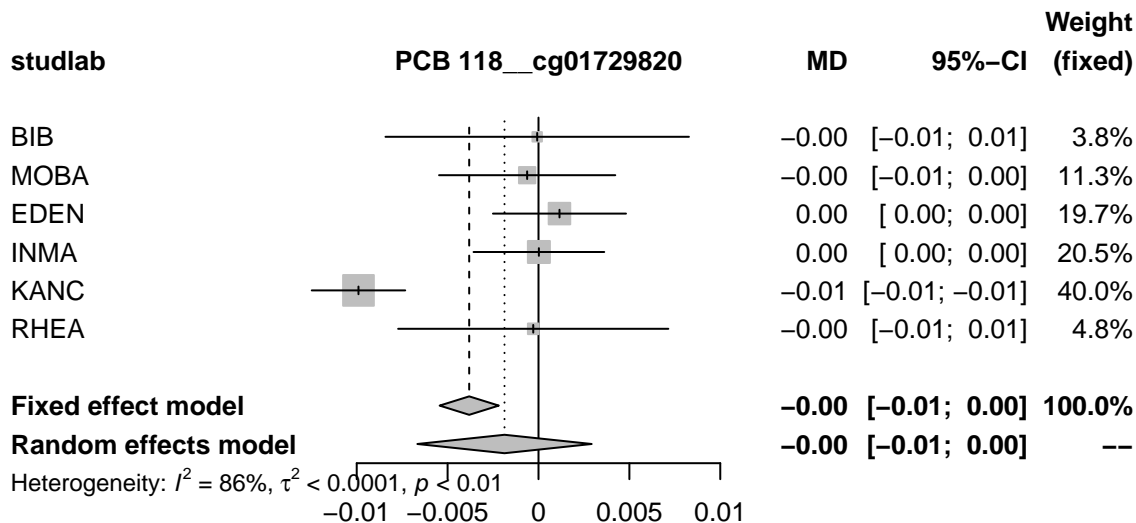

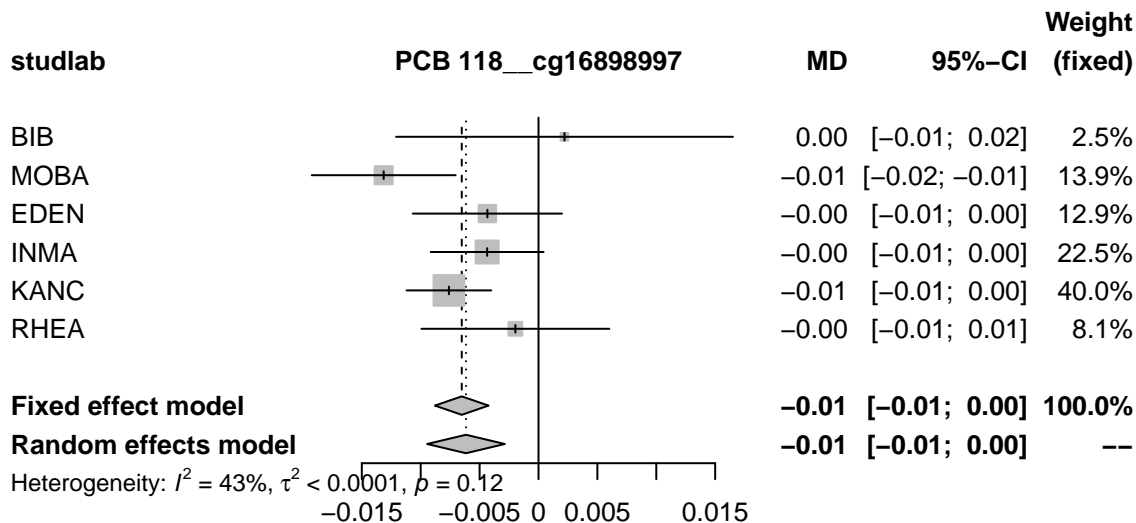

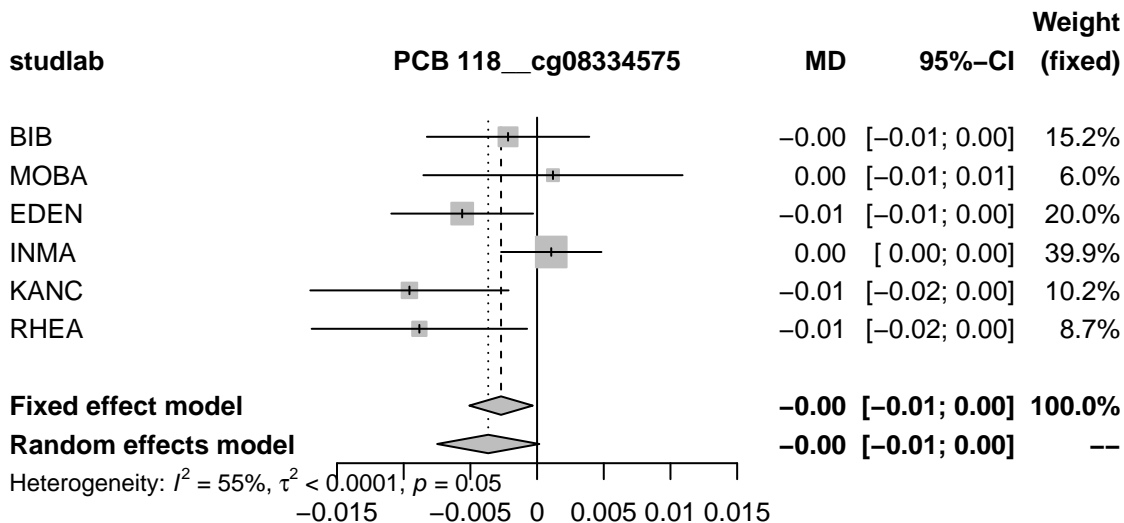

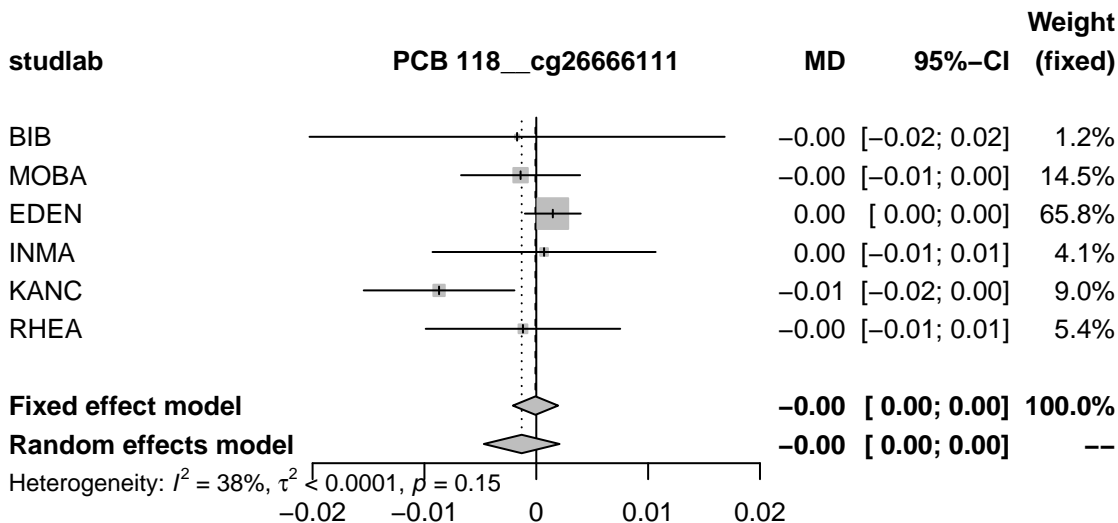

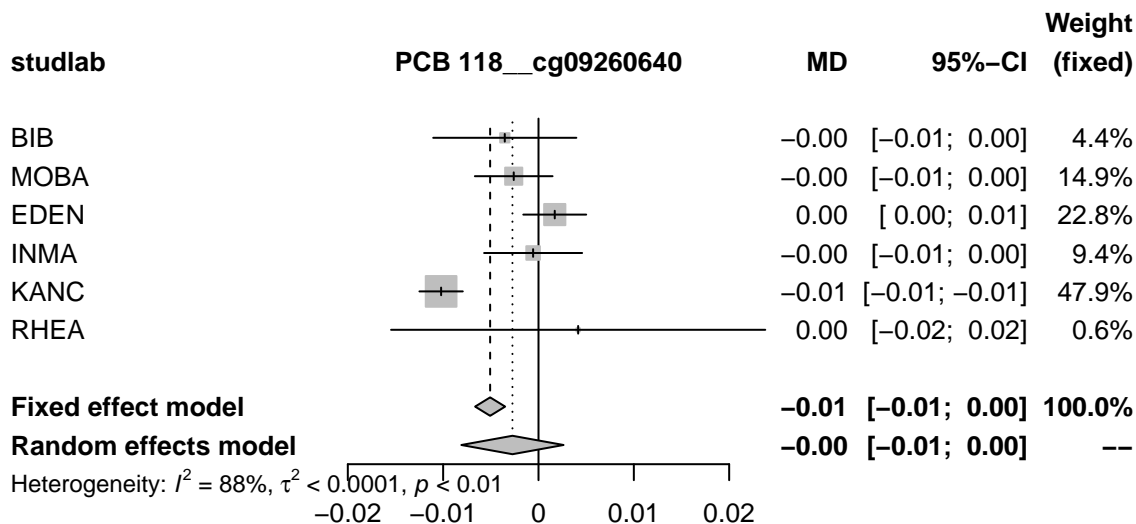

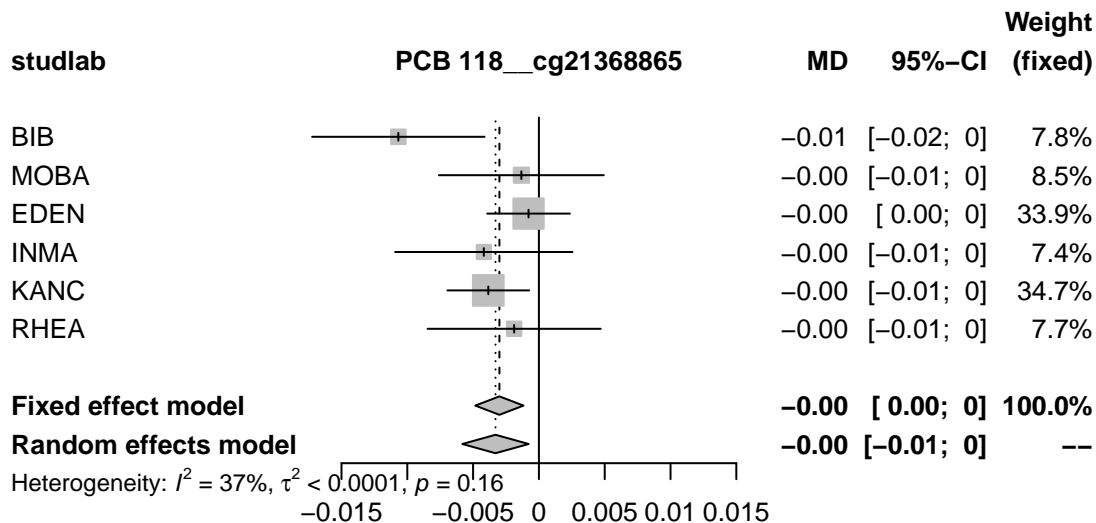

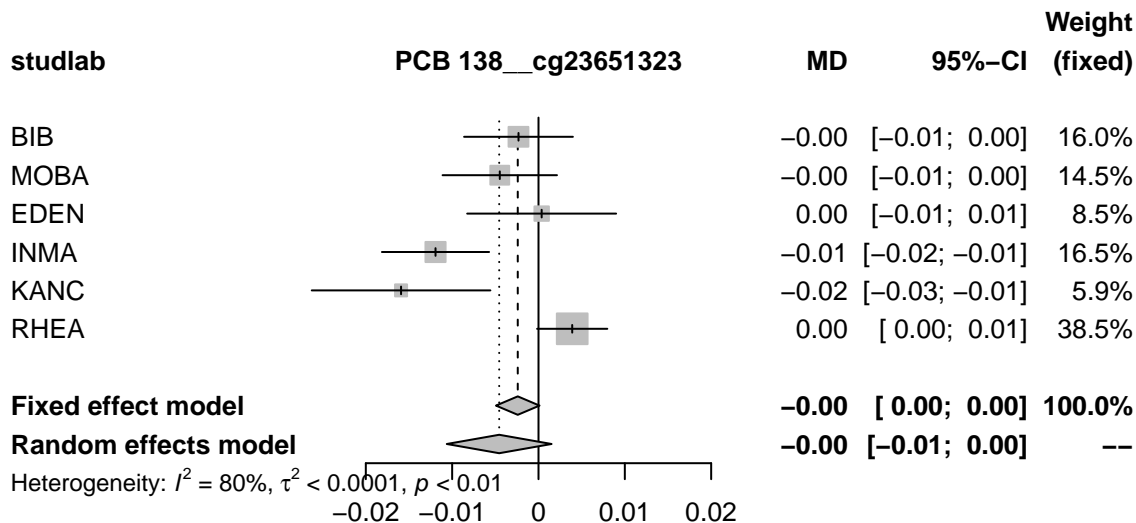

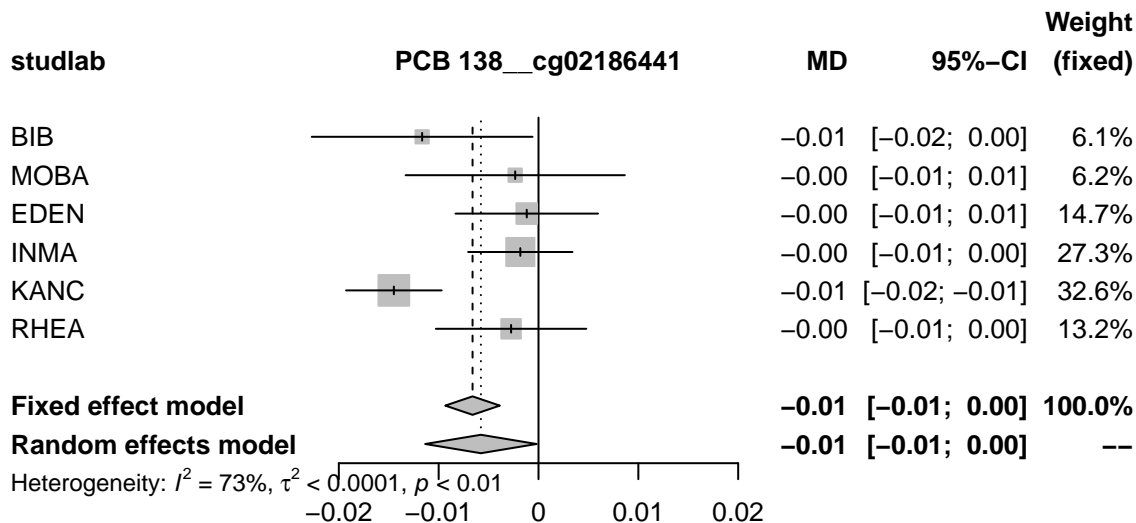

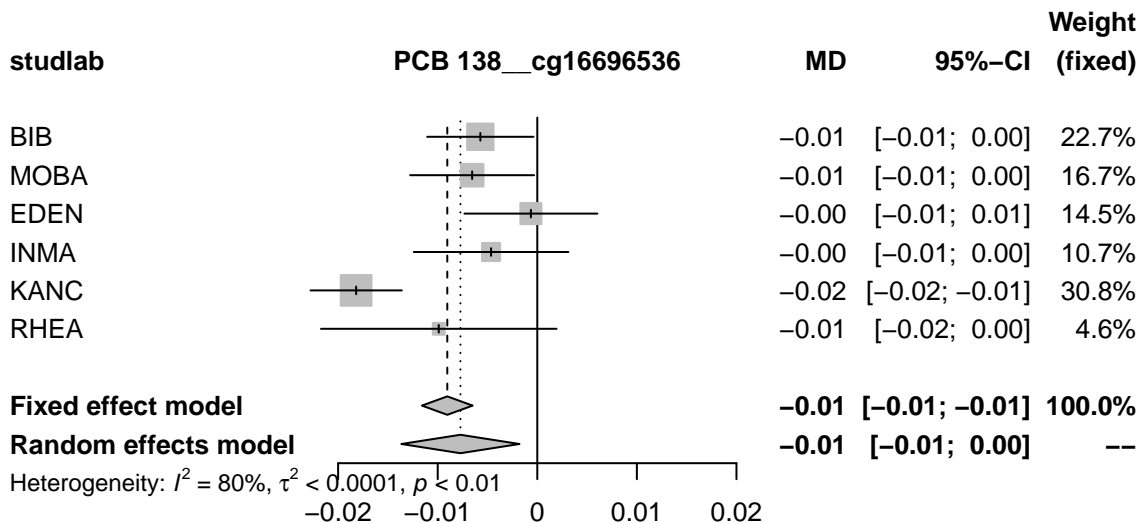

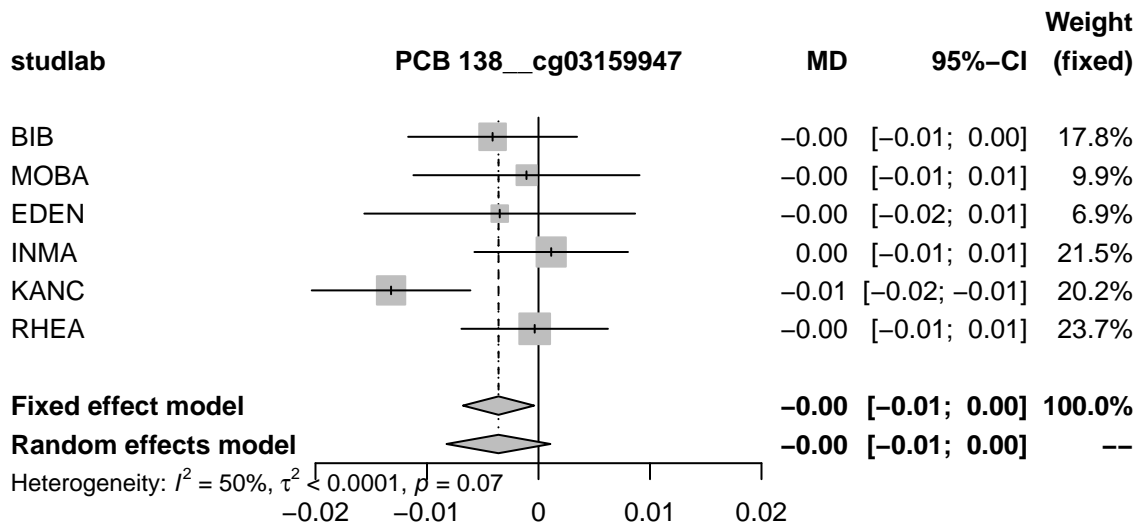

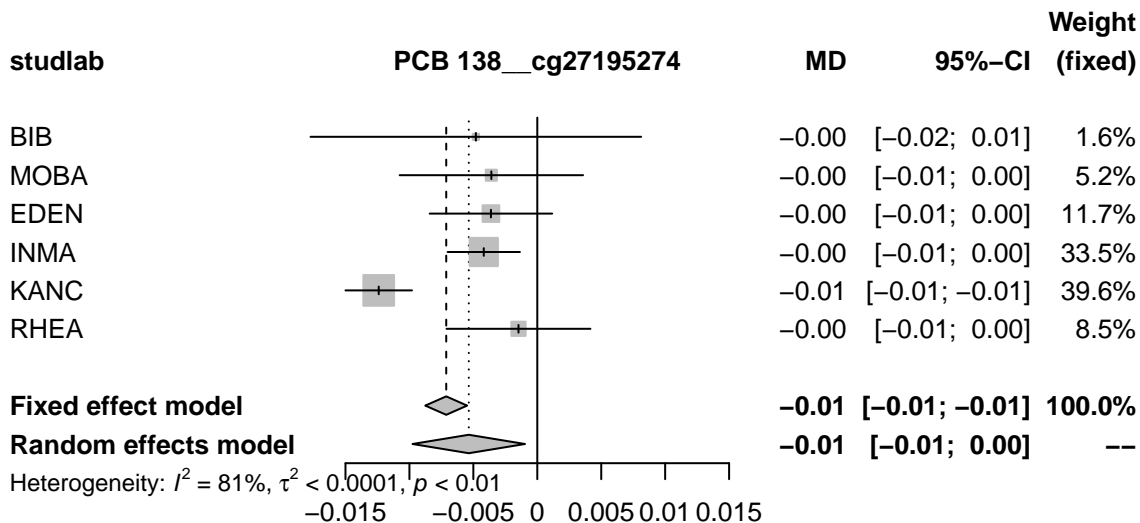

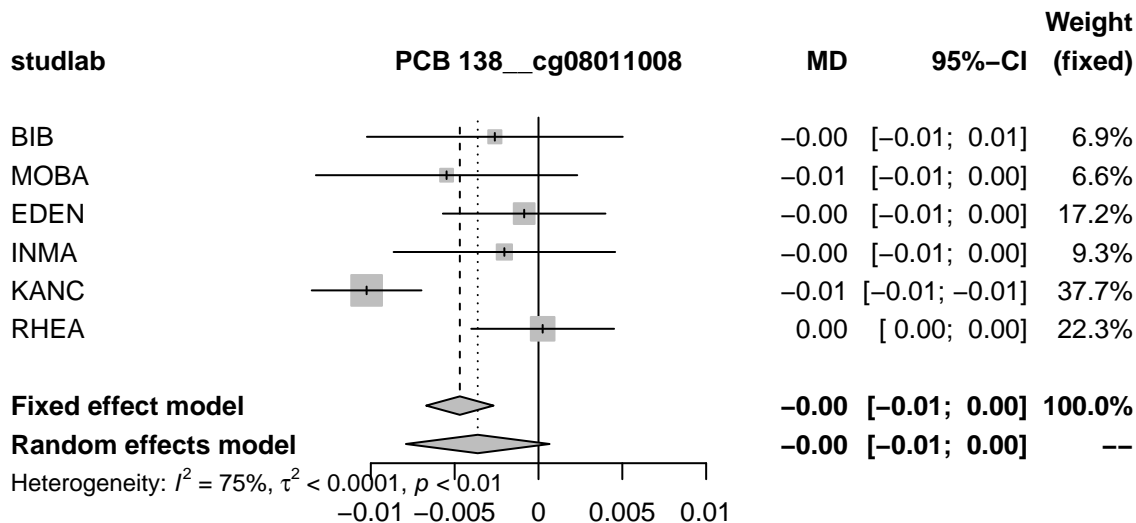

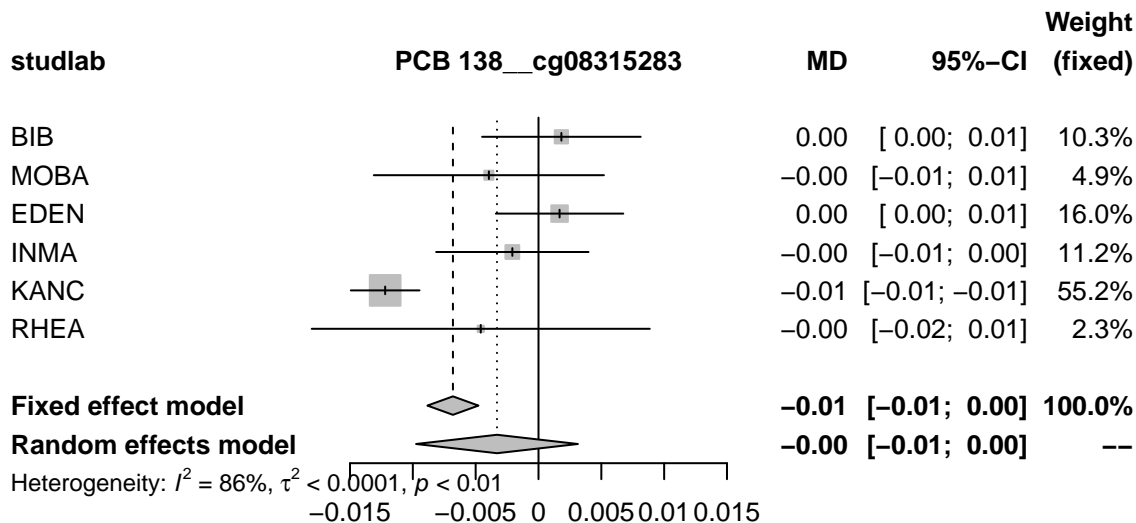

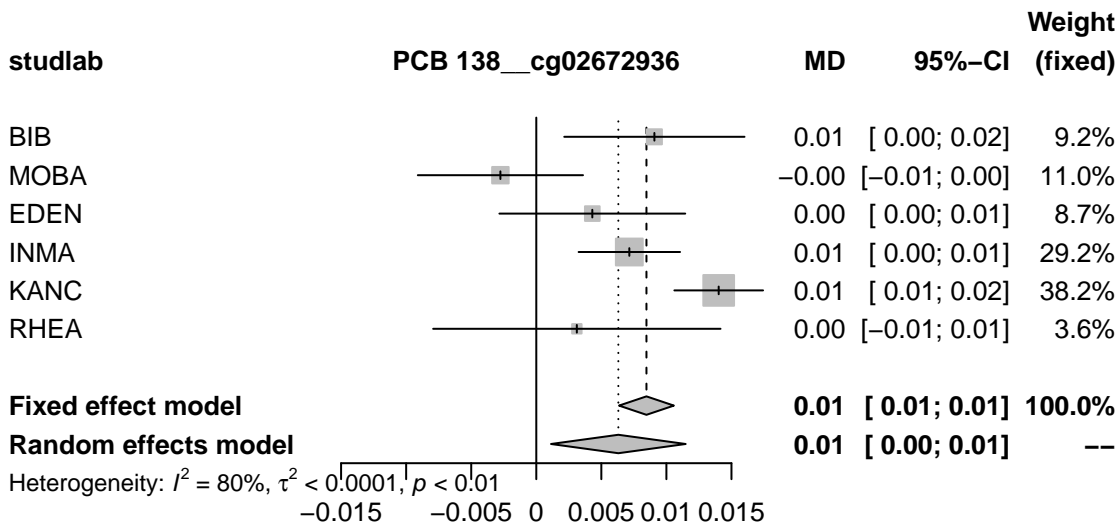

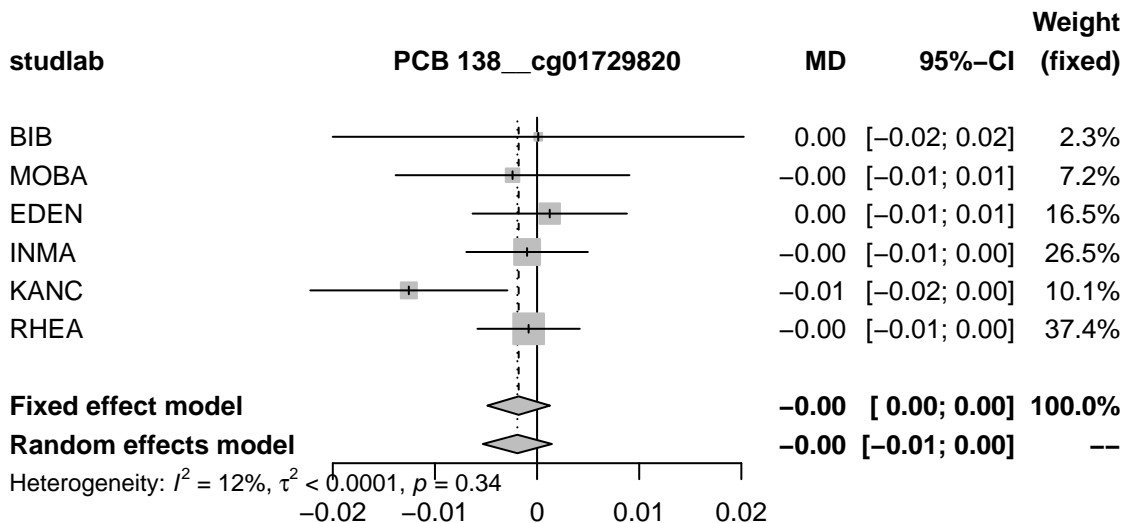

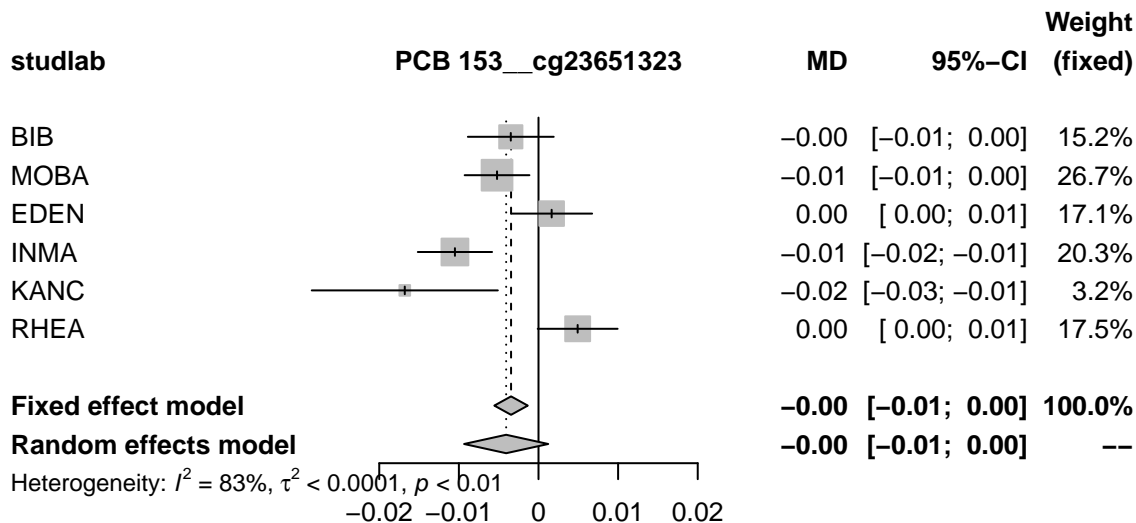

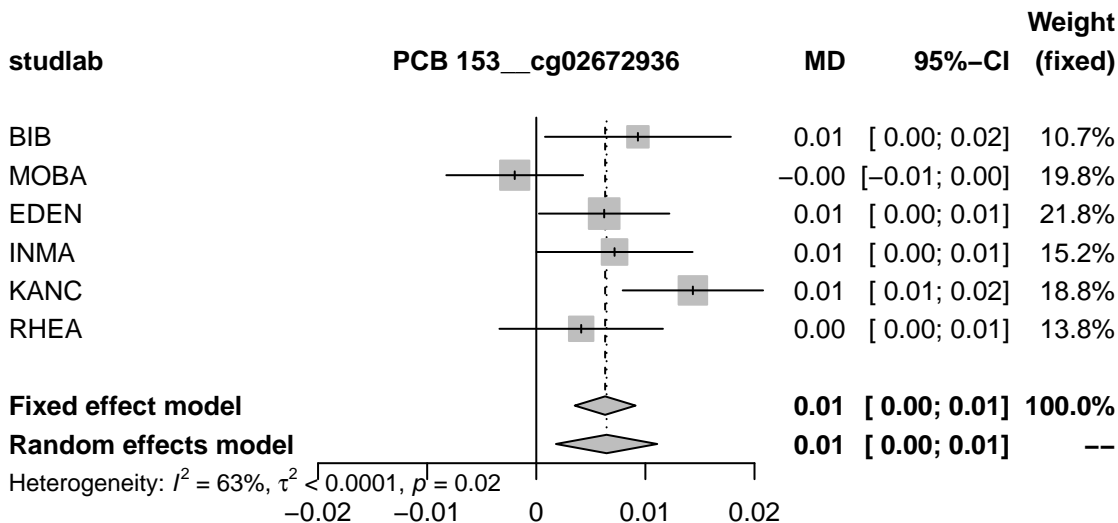

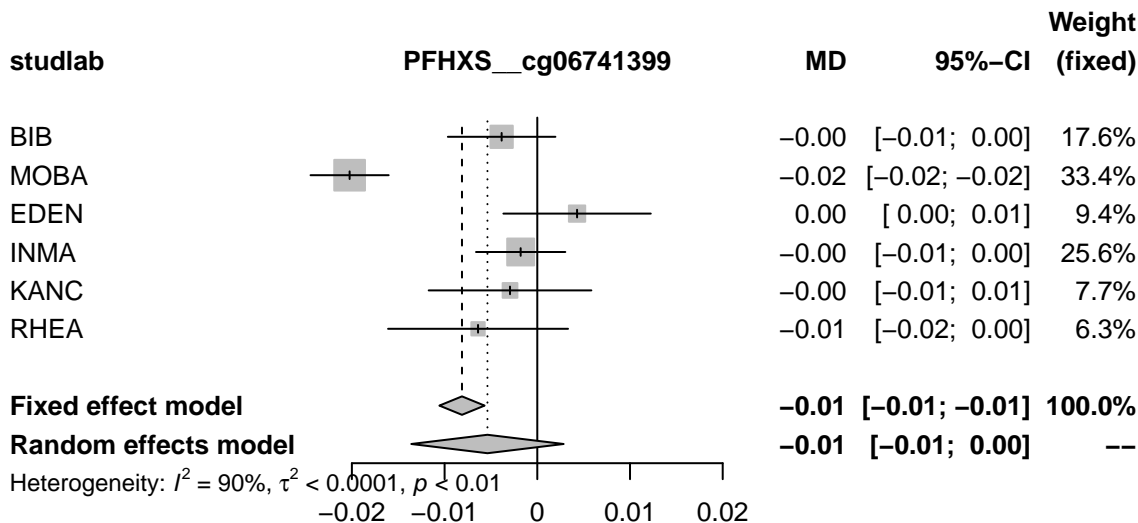

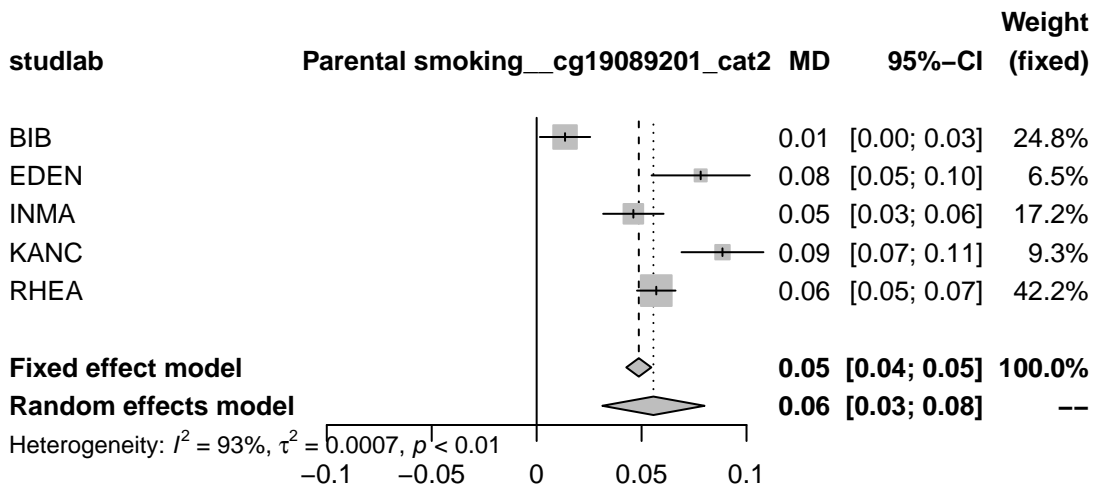

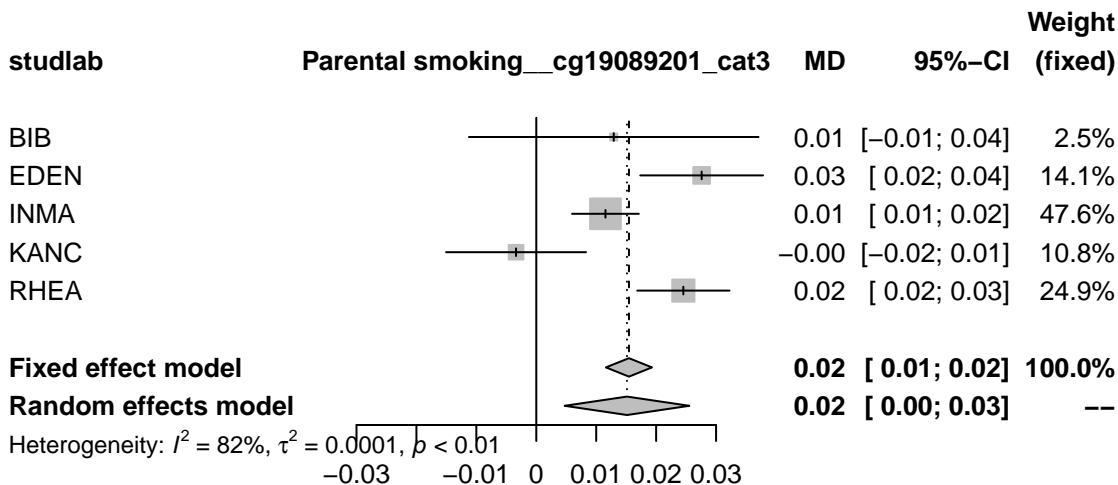

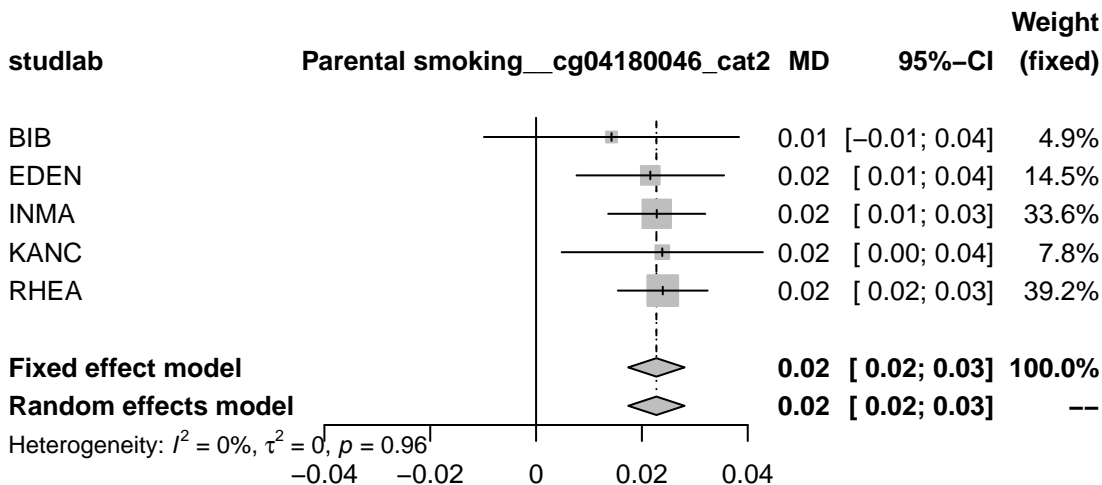

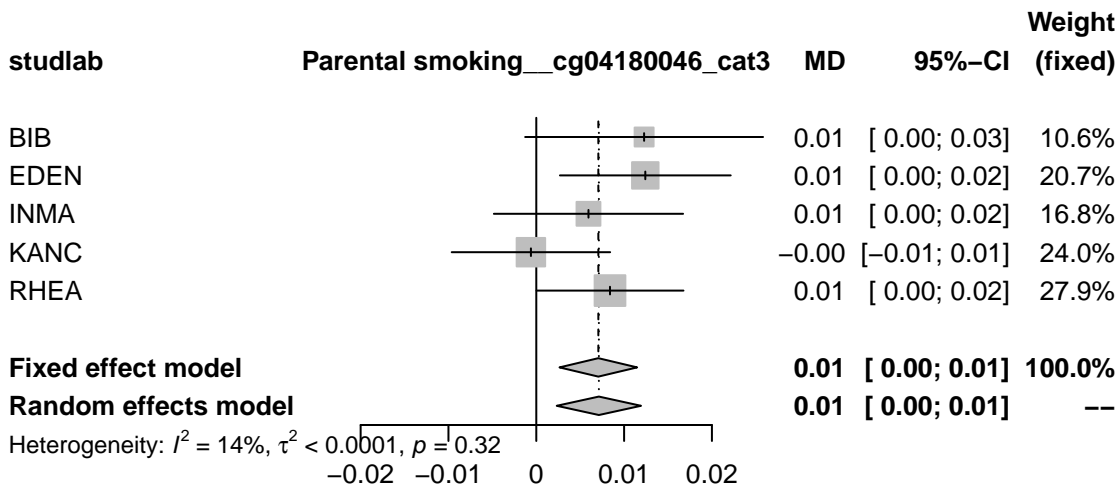

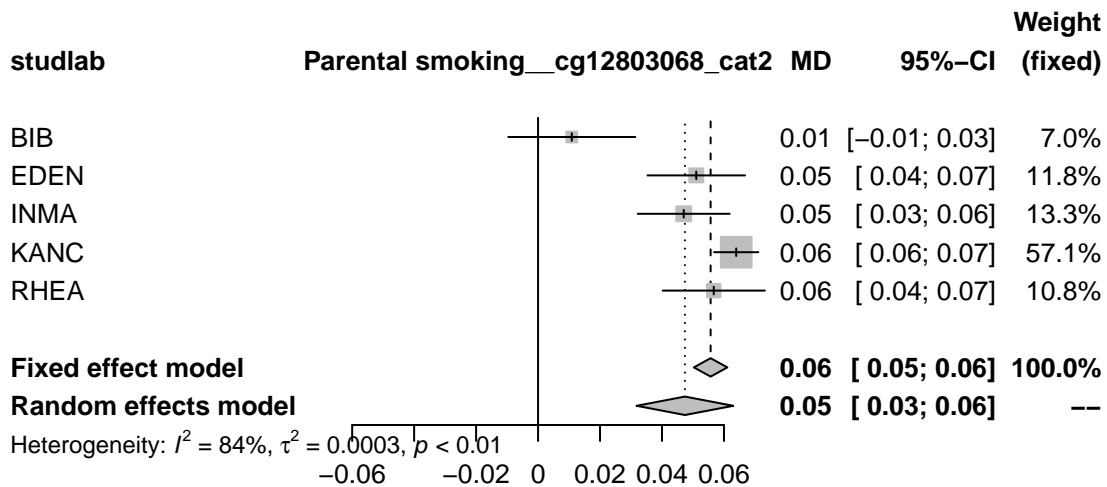

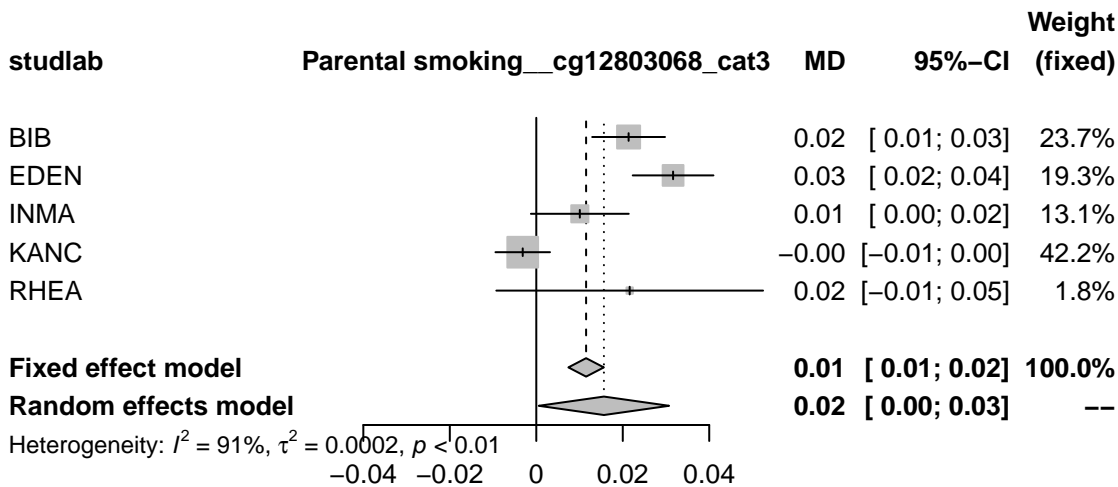

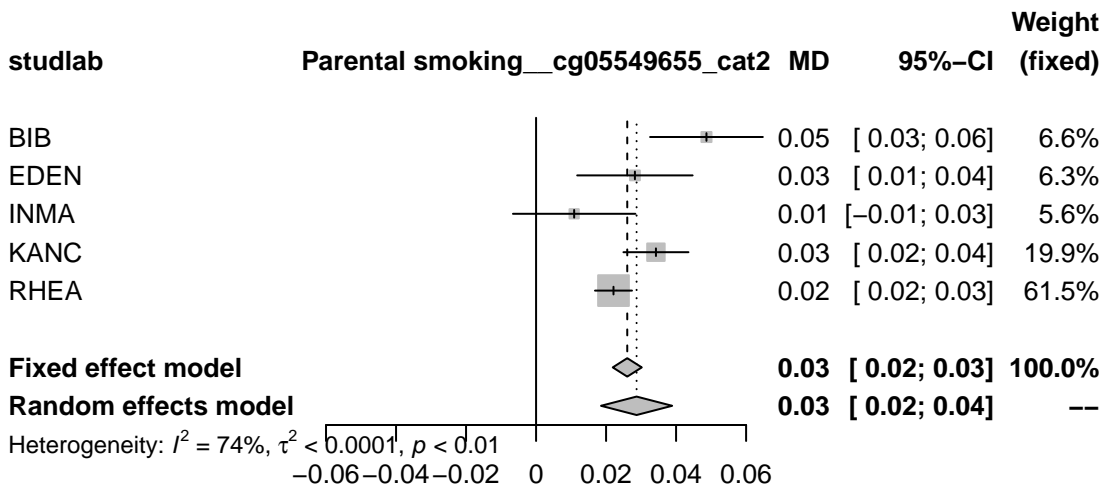

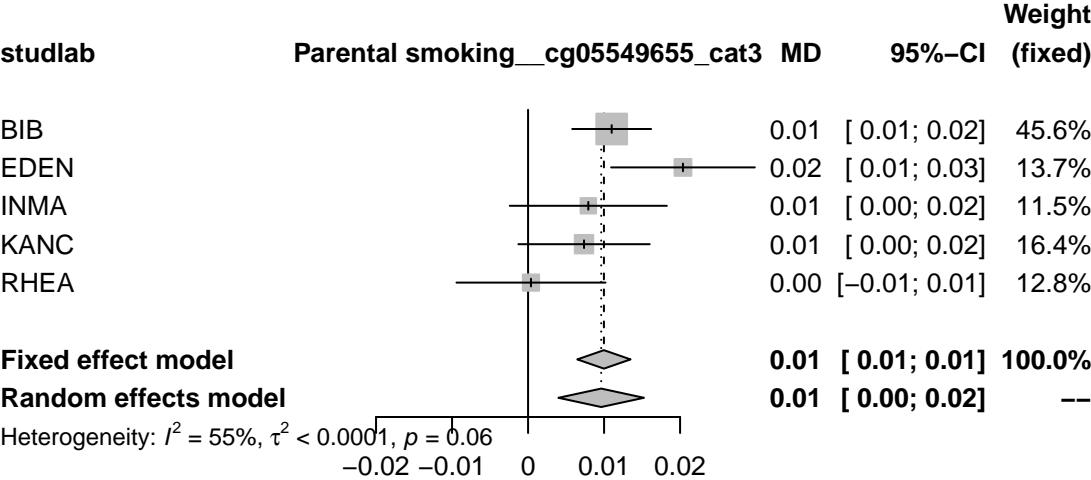

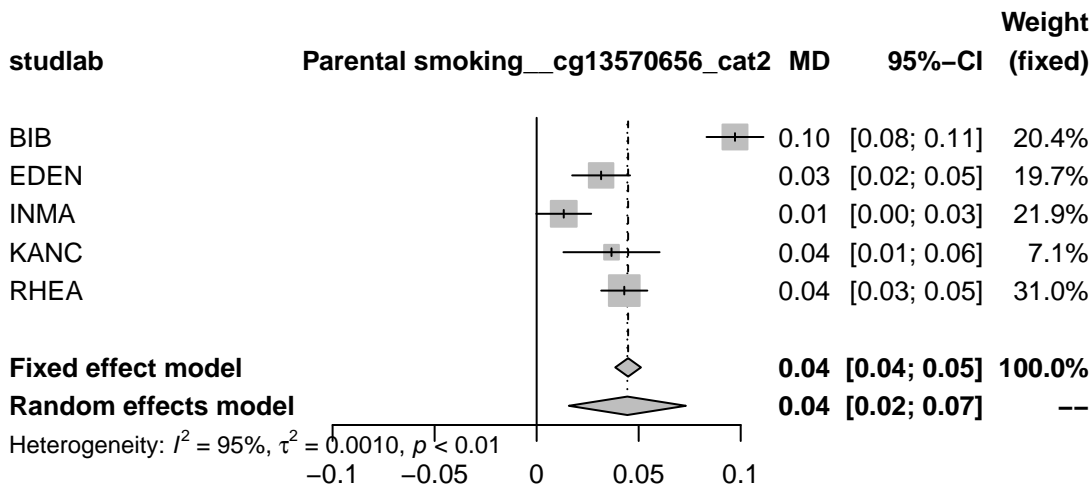

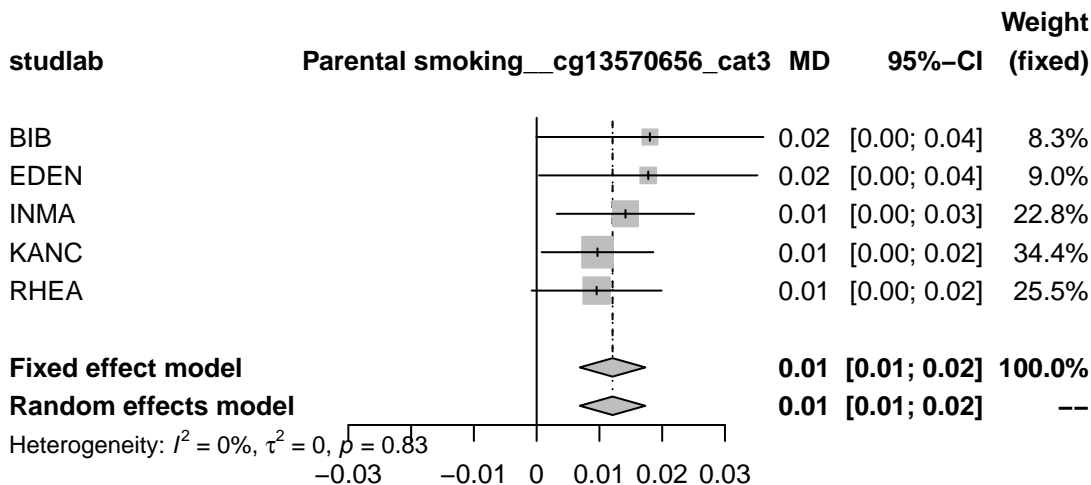

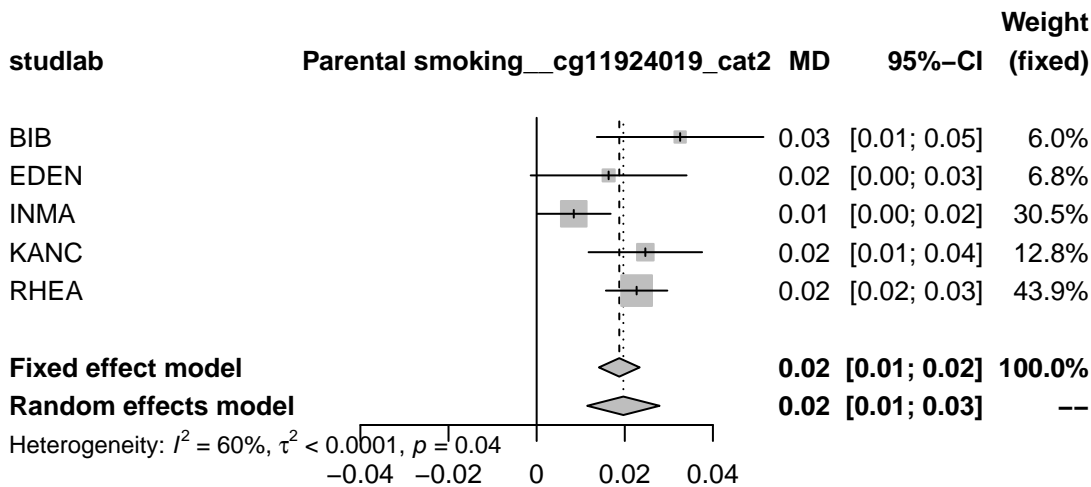

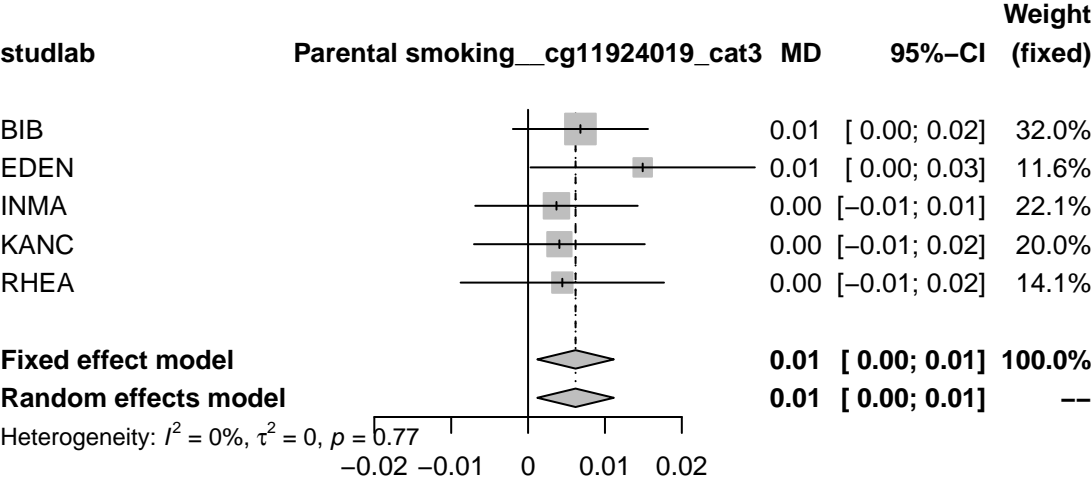

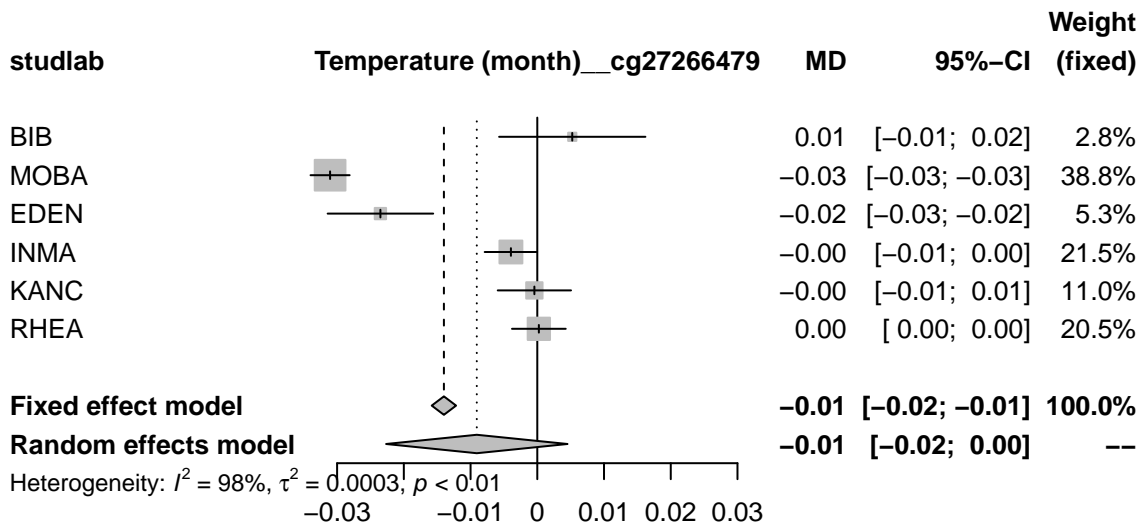

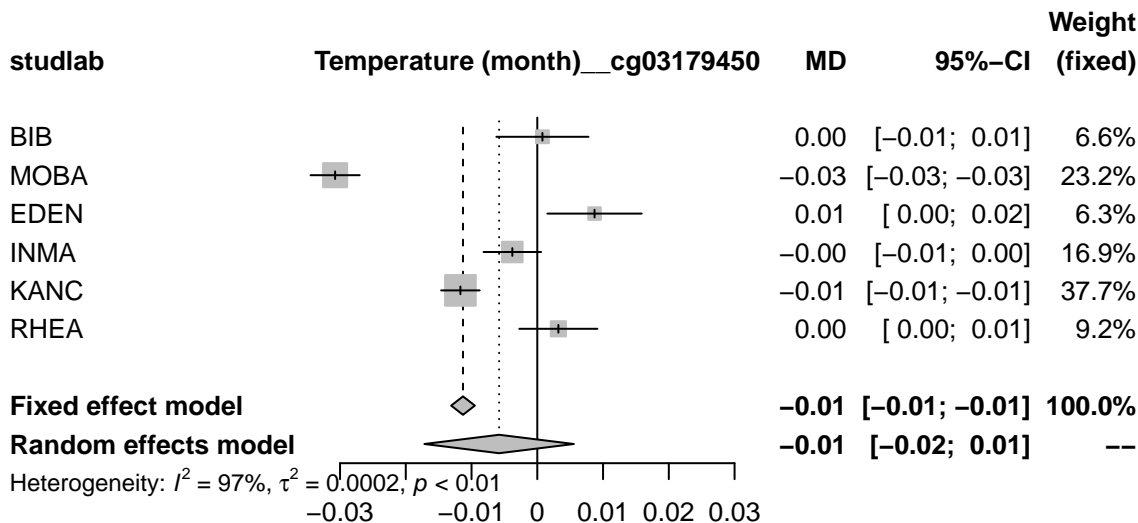

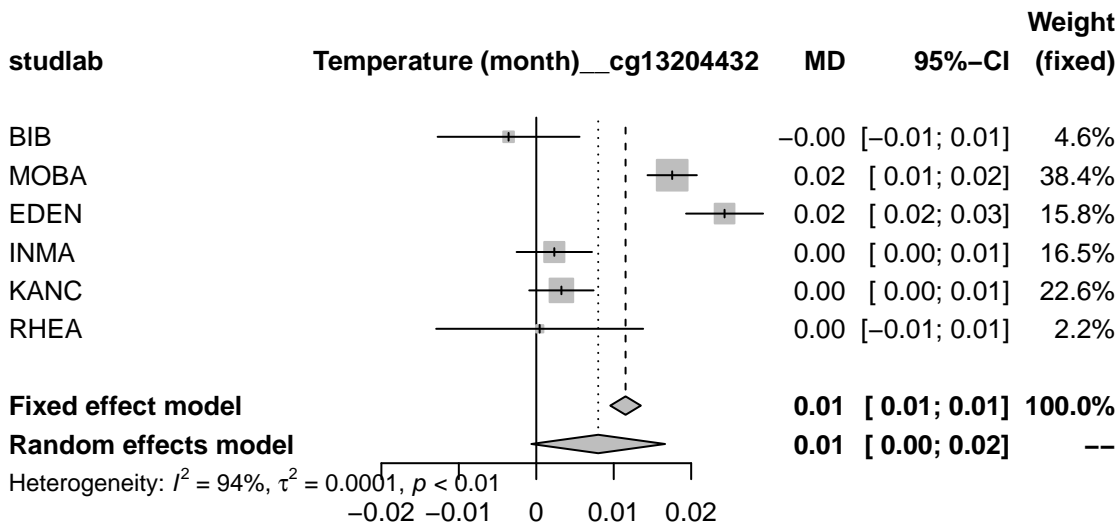

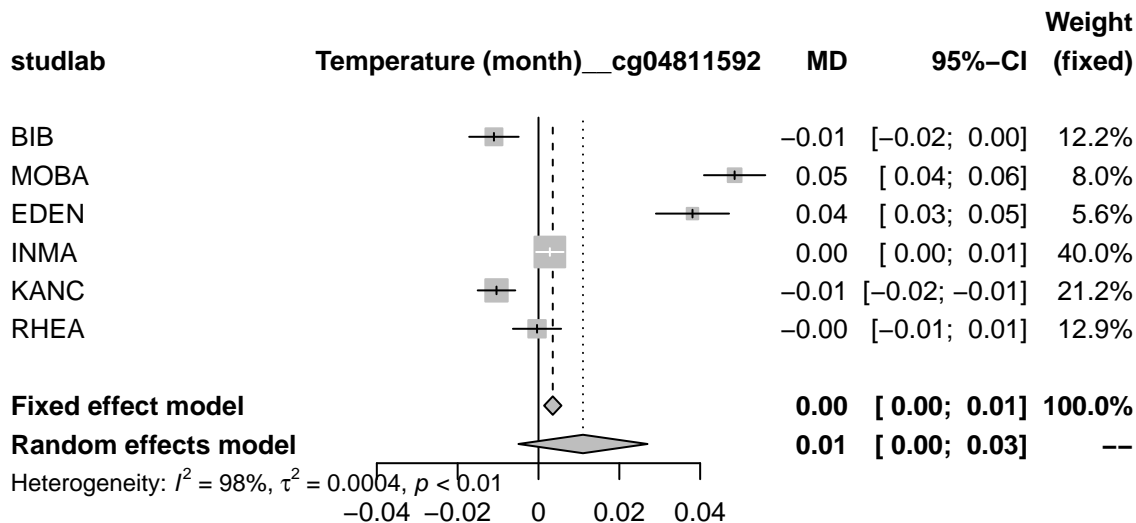

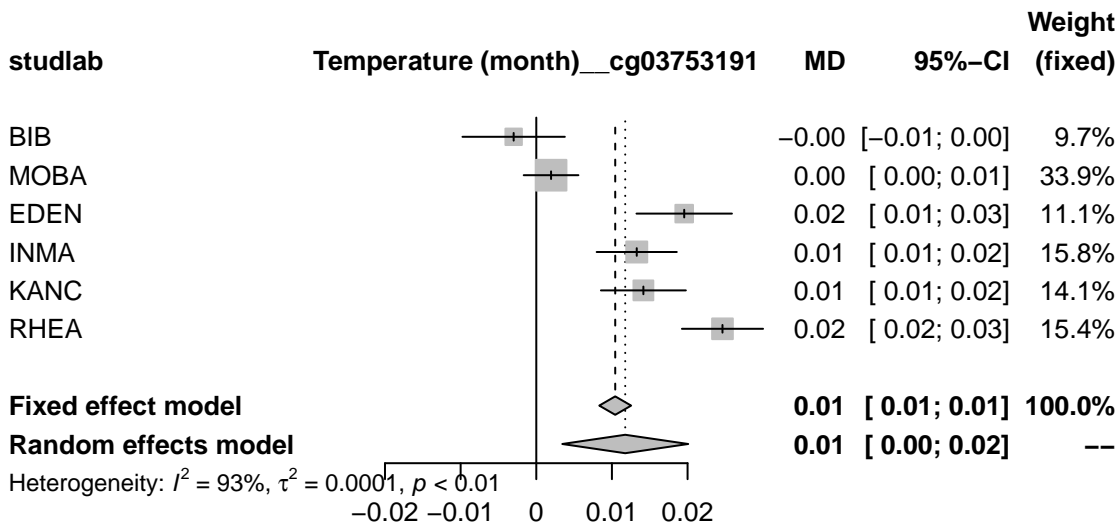

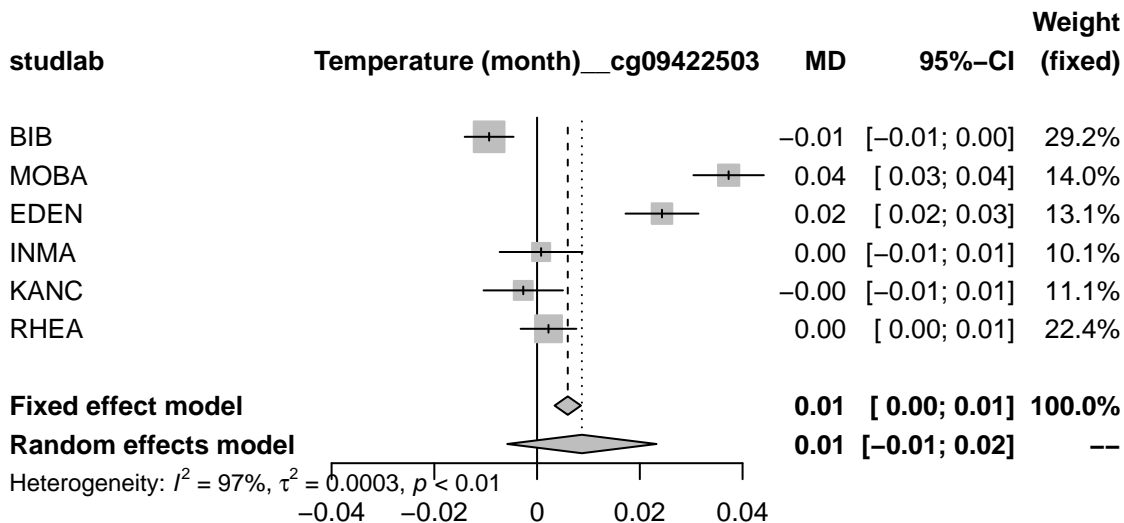

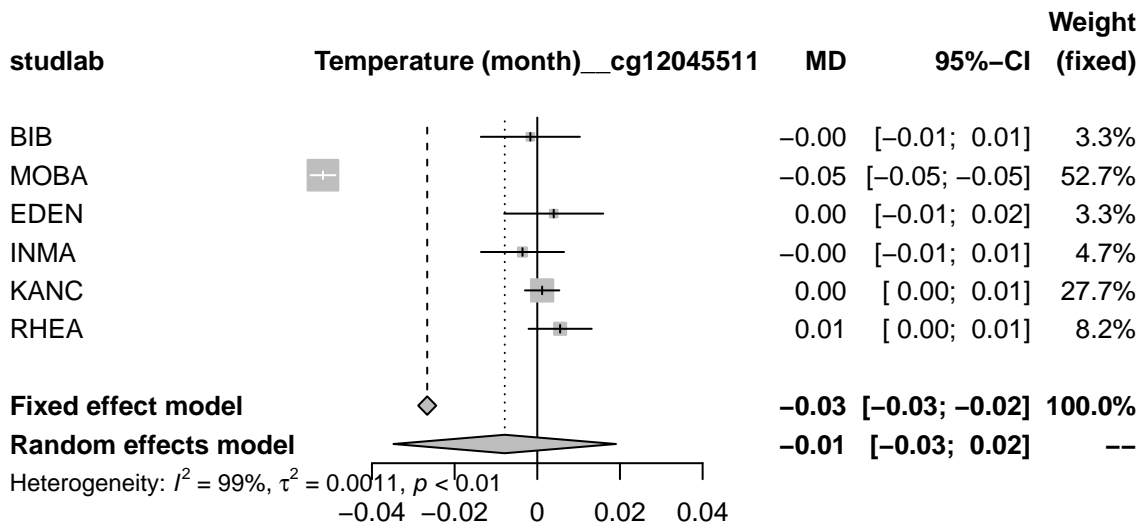

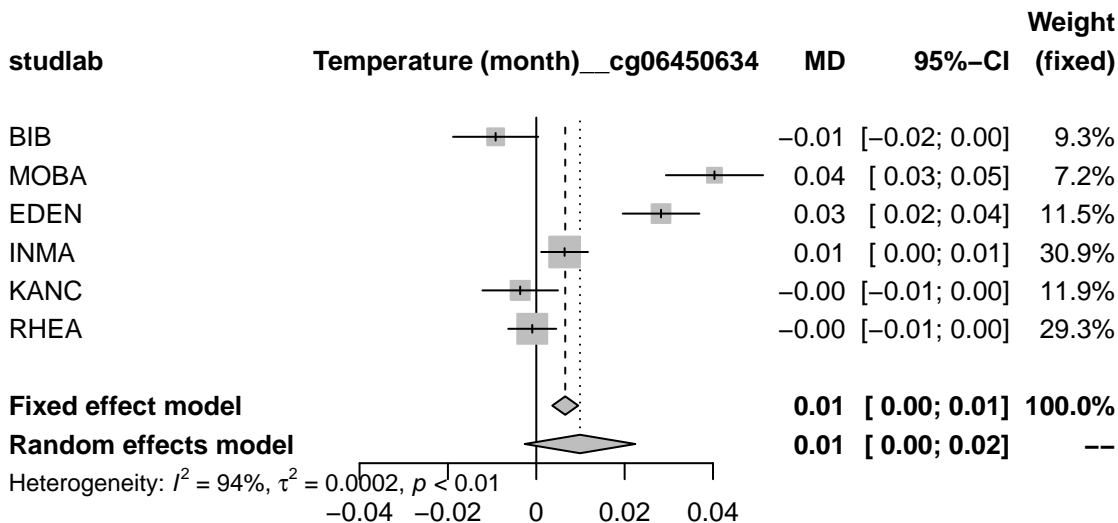

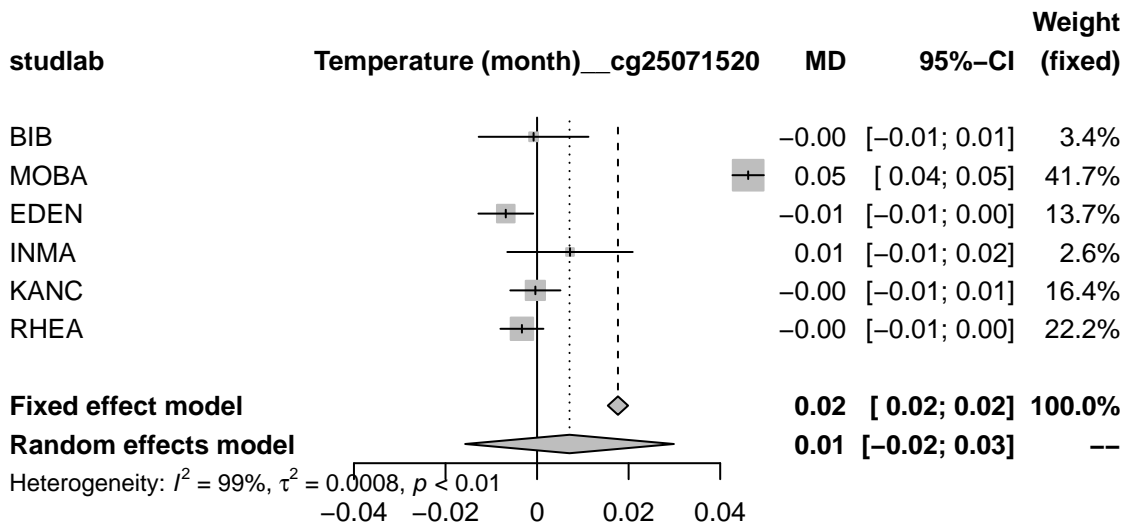

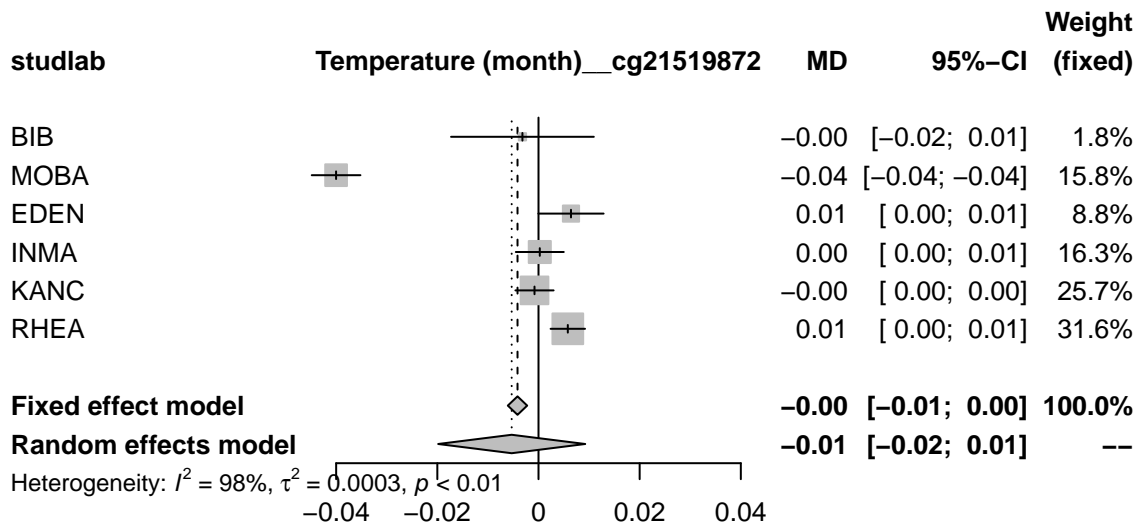

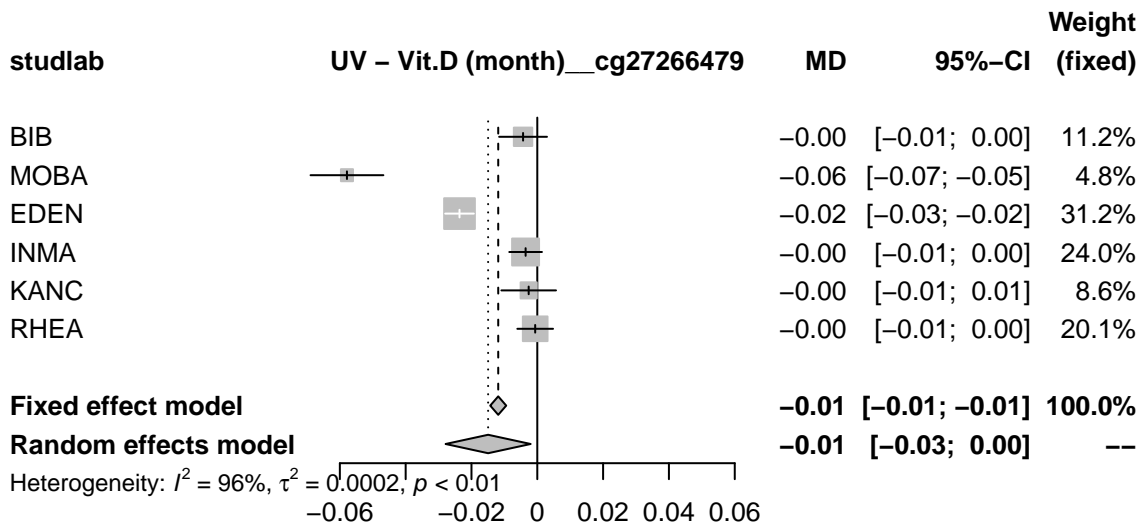

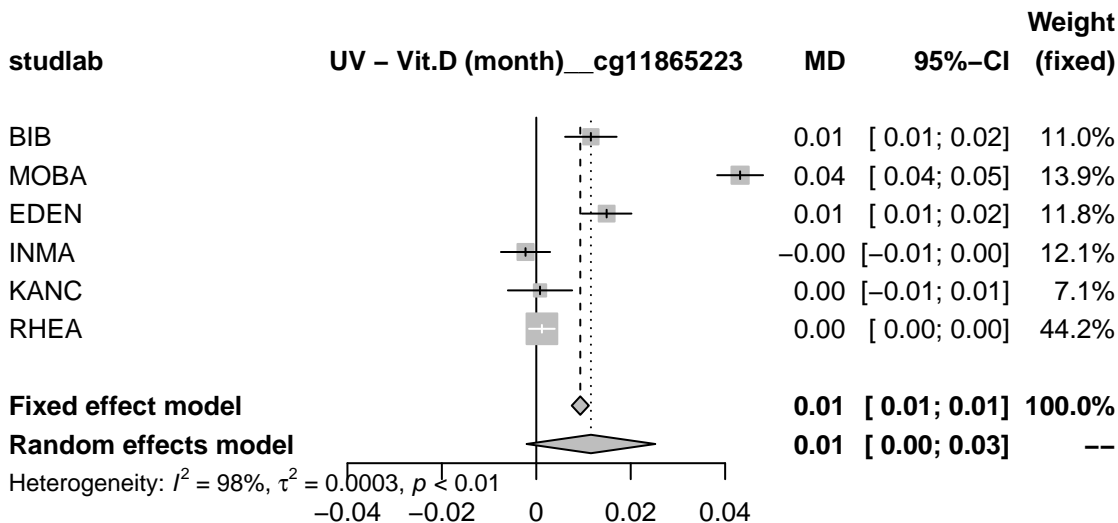

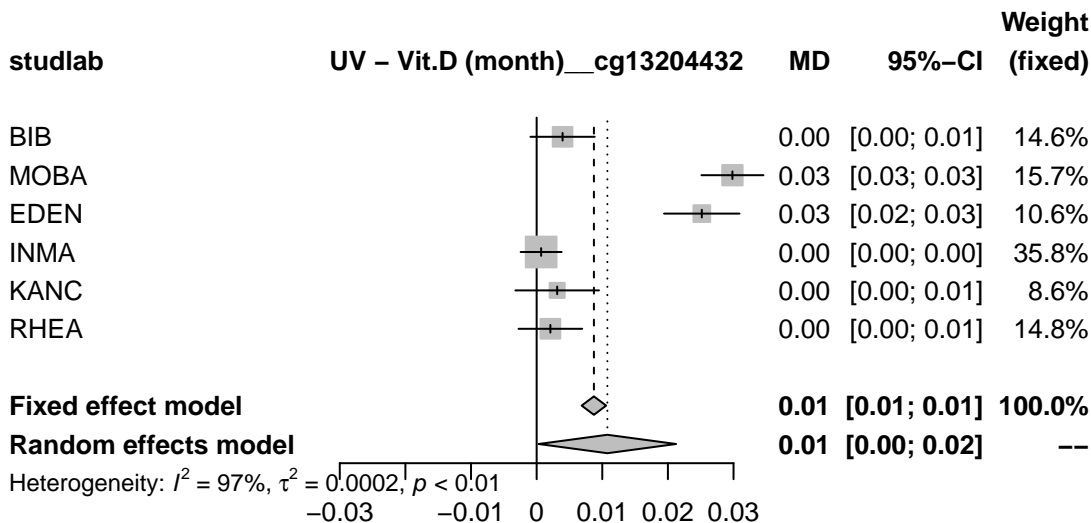

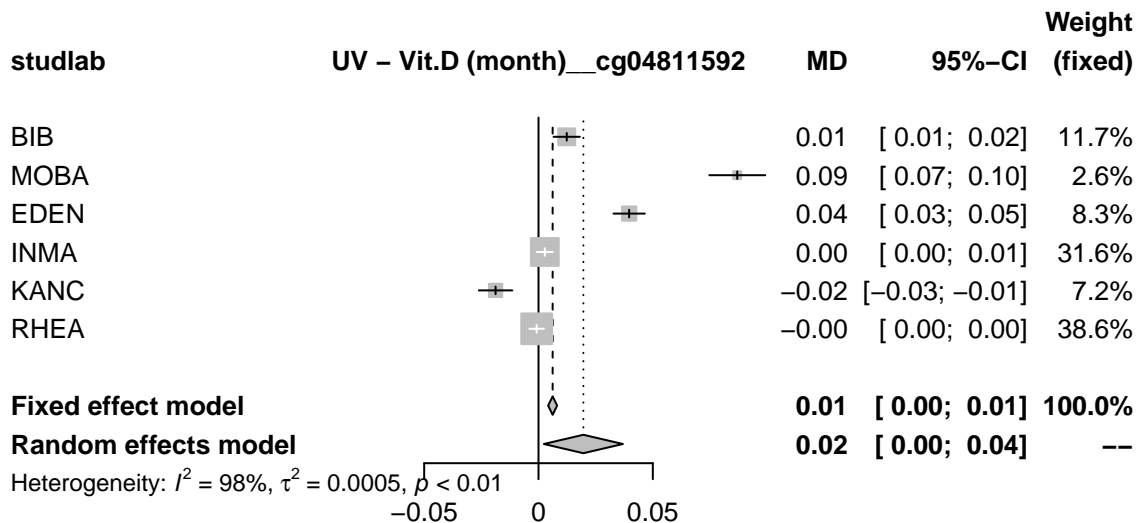

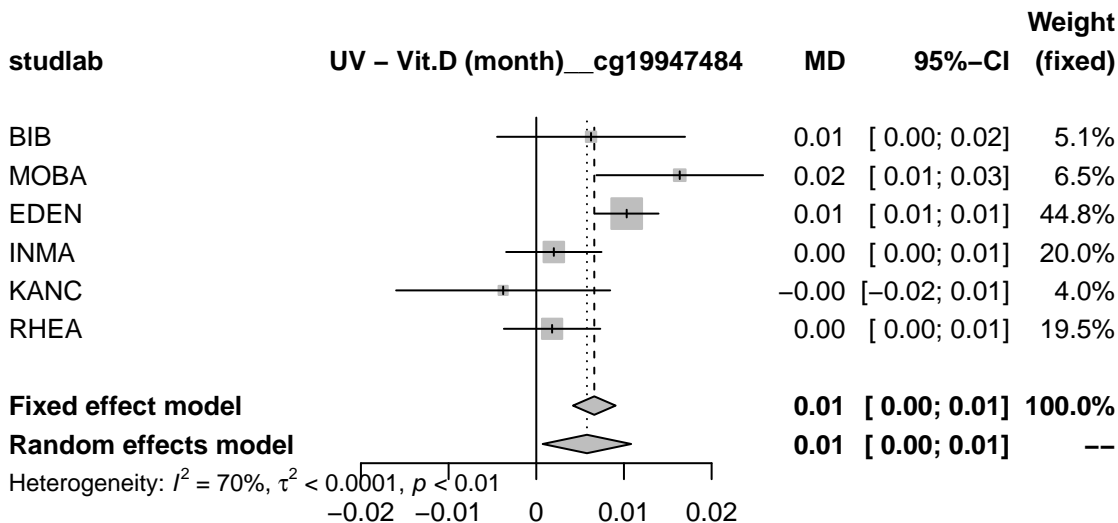

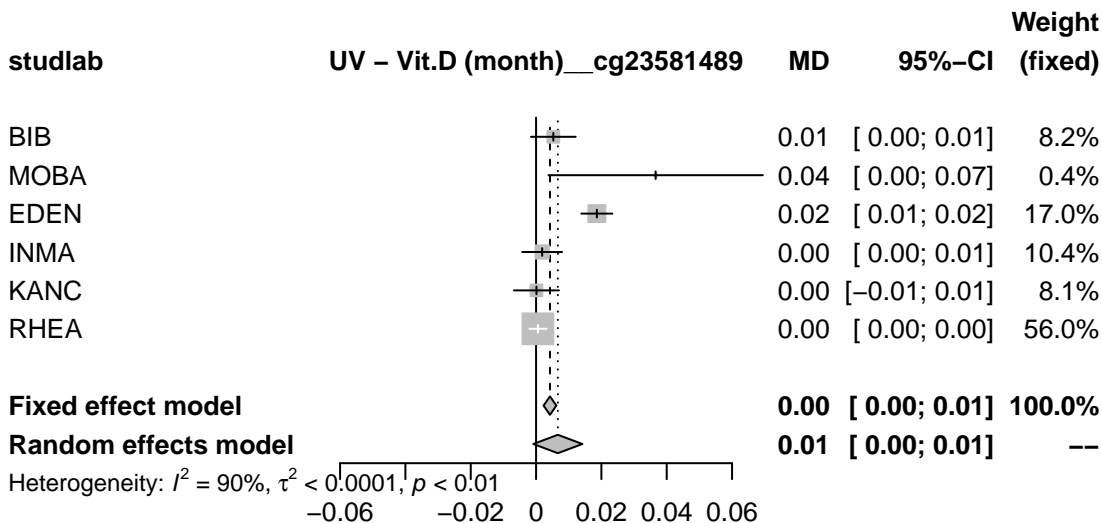

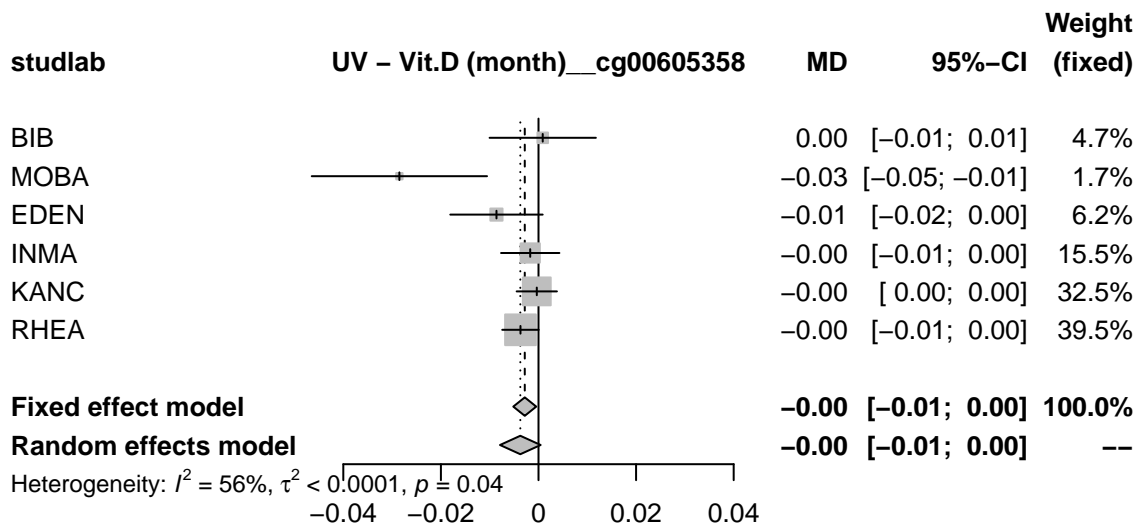

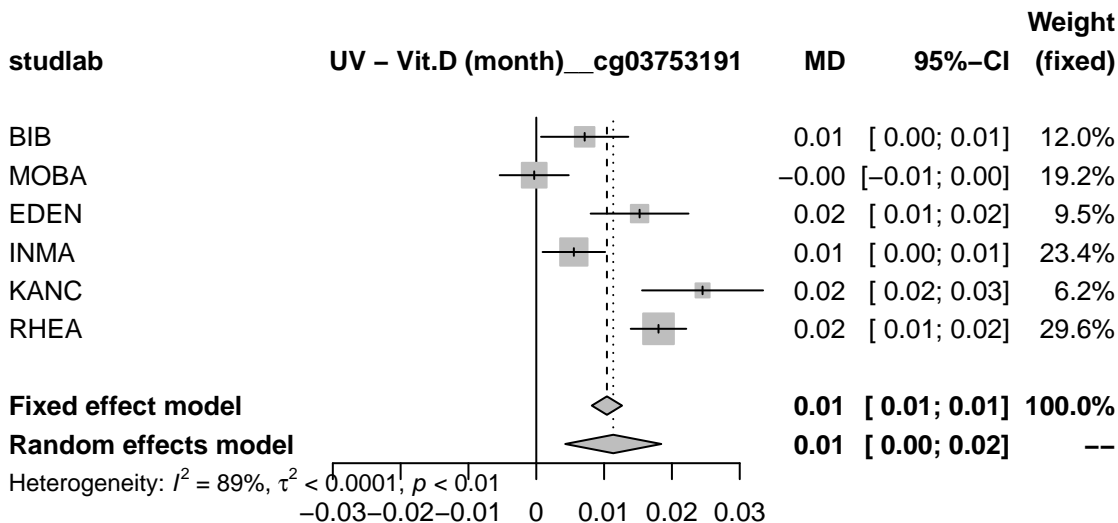

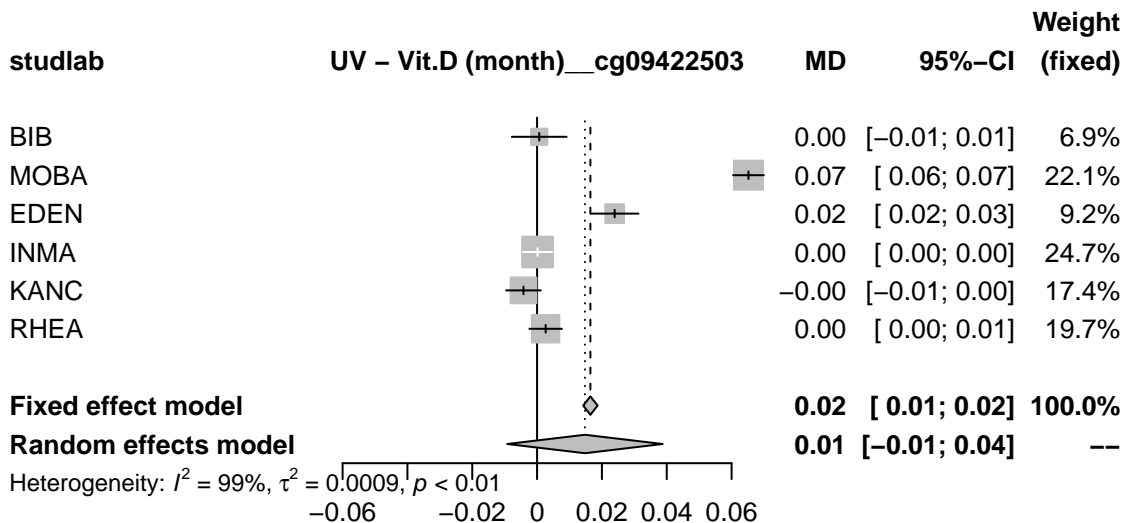

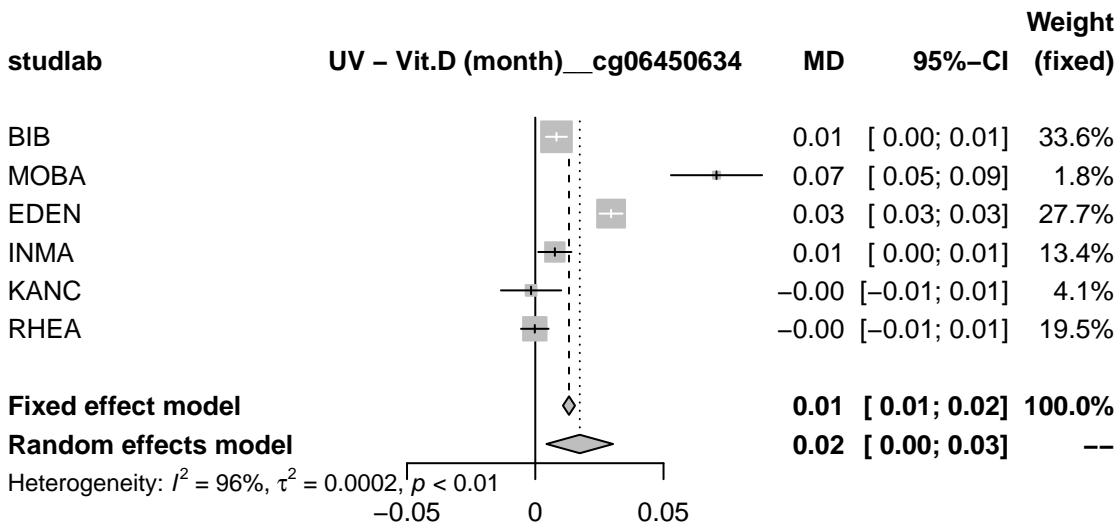

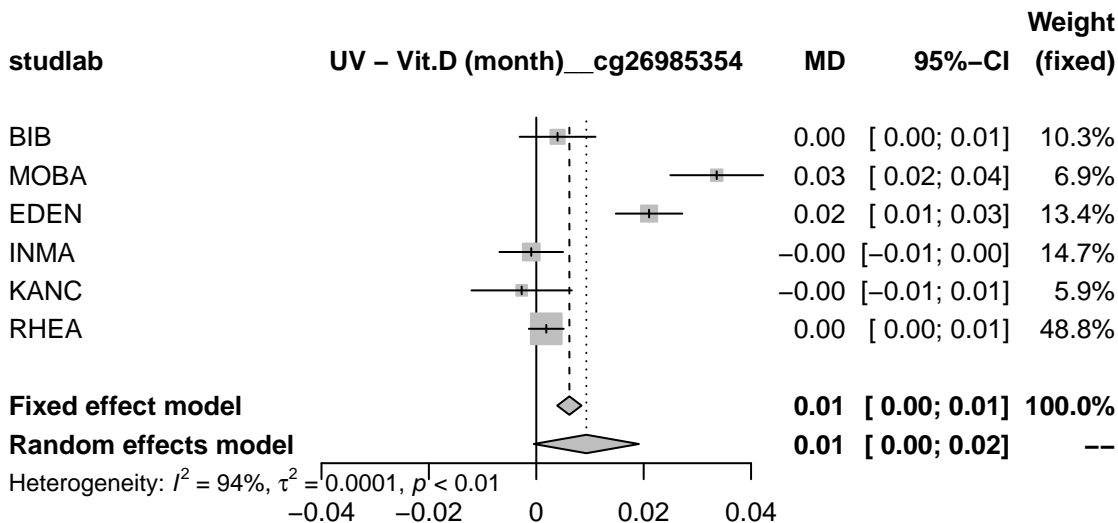

Supplement: Supplementary file 14 — Supplementary Dataset 11 [file 41467_2022_34422_MOESM14_ESM.zip › HELIX_ExpOmics_FigS2_Forestplots/HELIX_ExpOmics_FigS2G_meth_post.pdf]

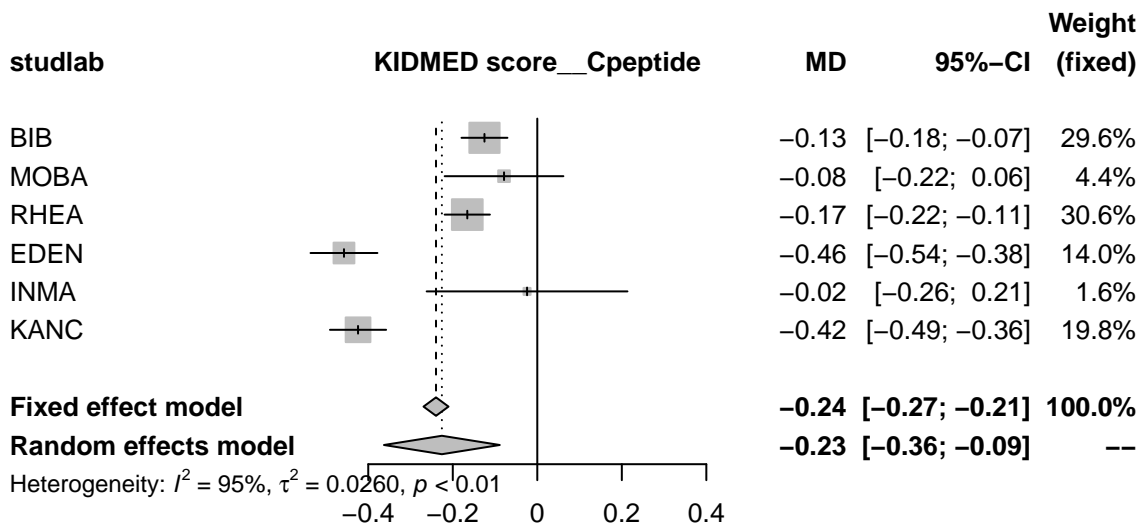

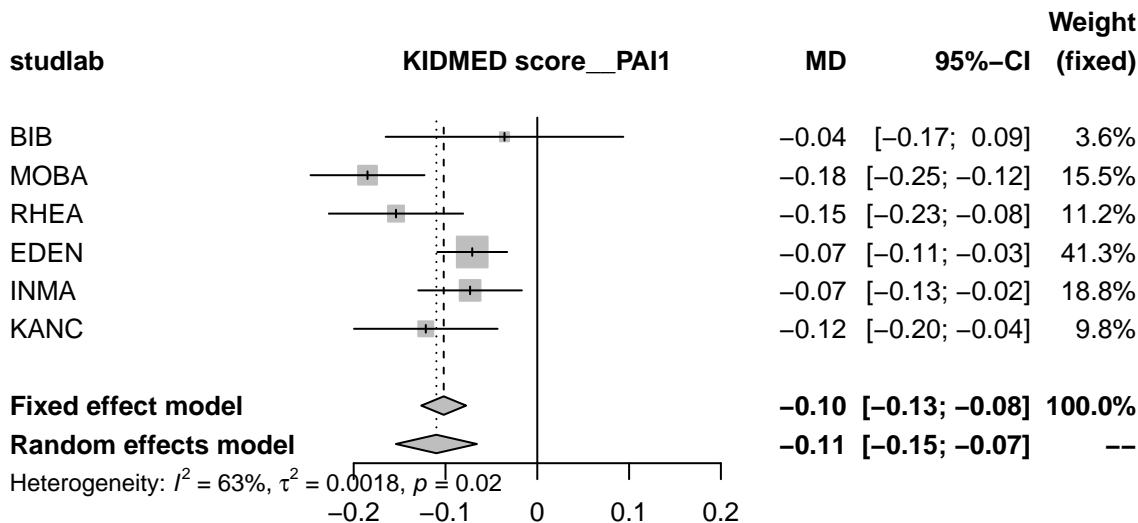

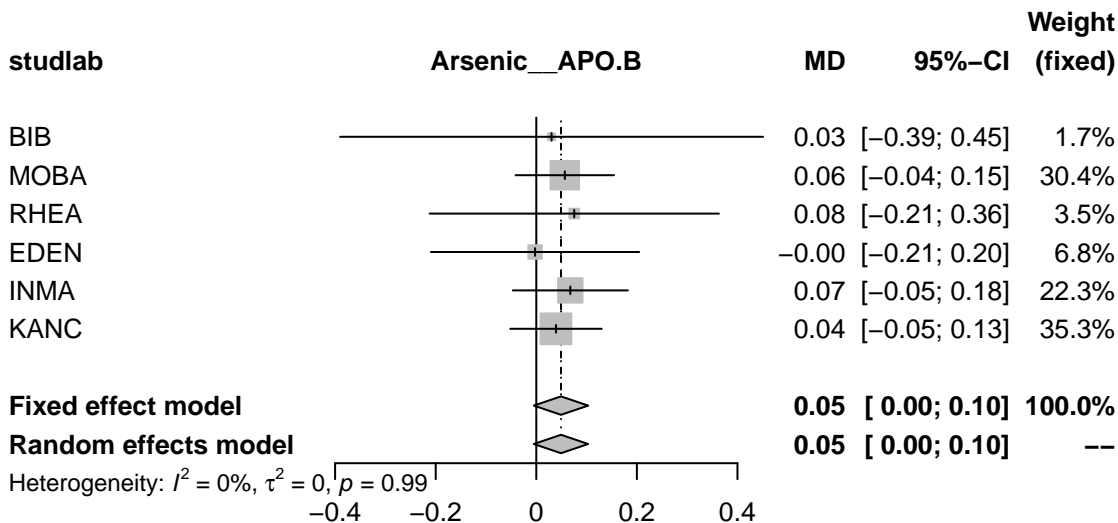

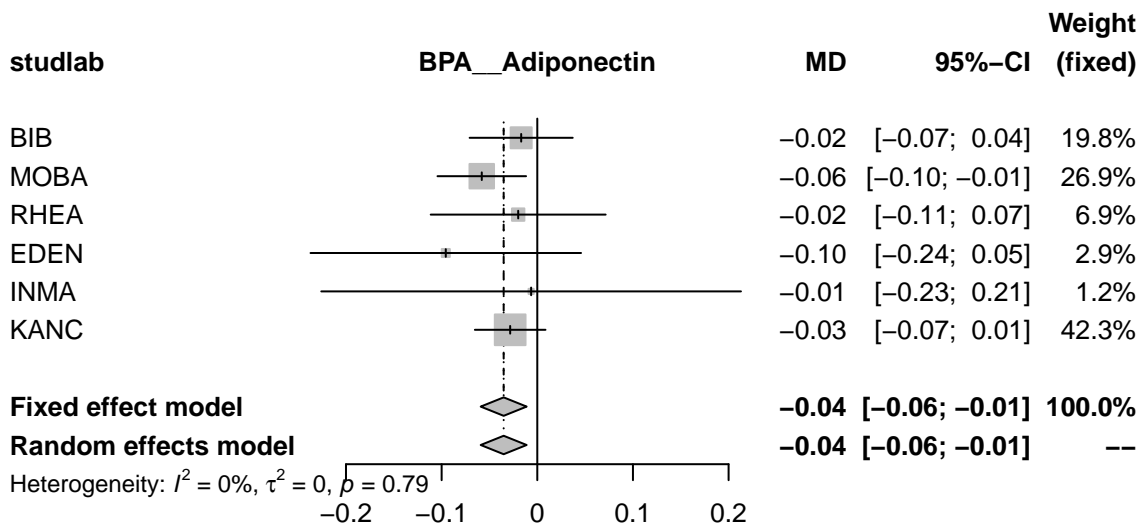

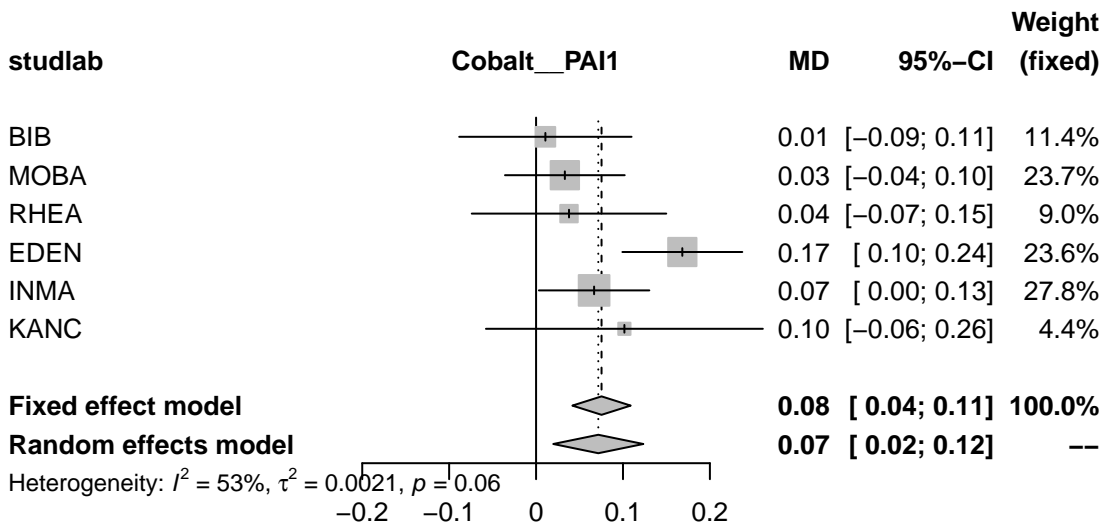

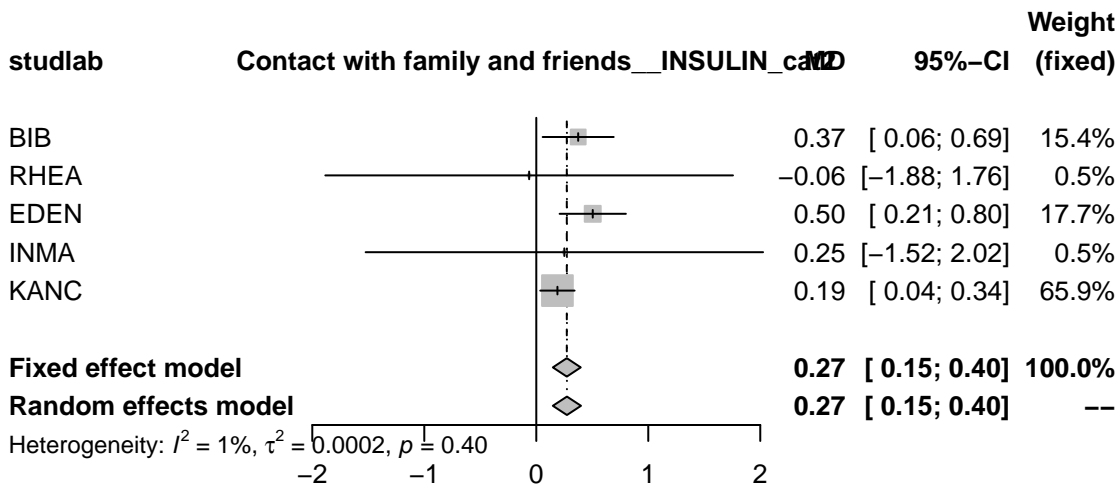

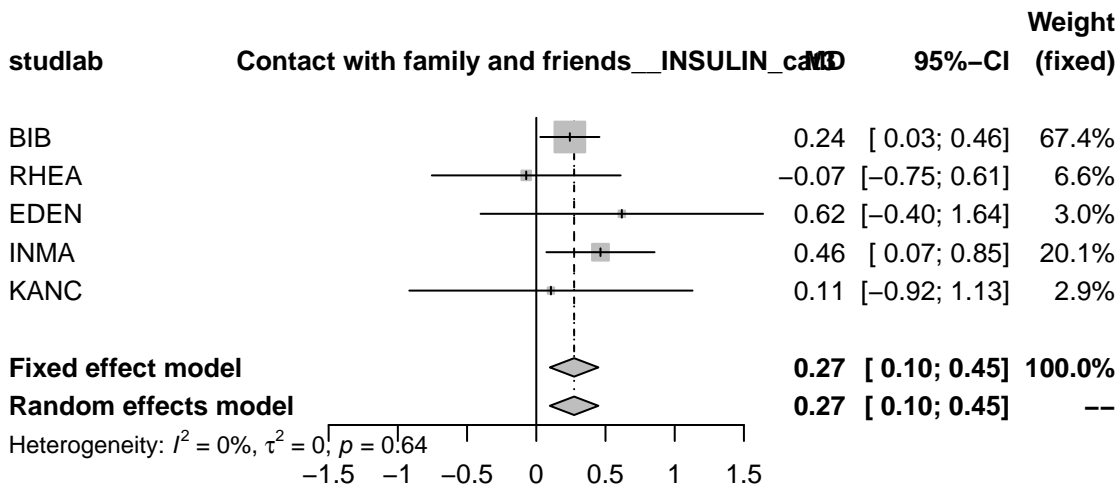

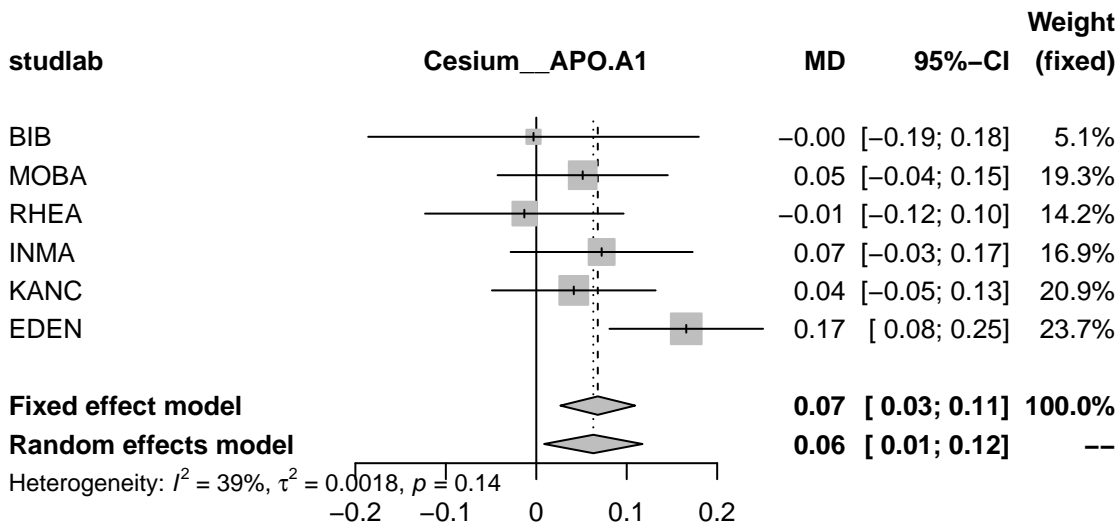

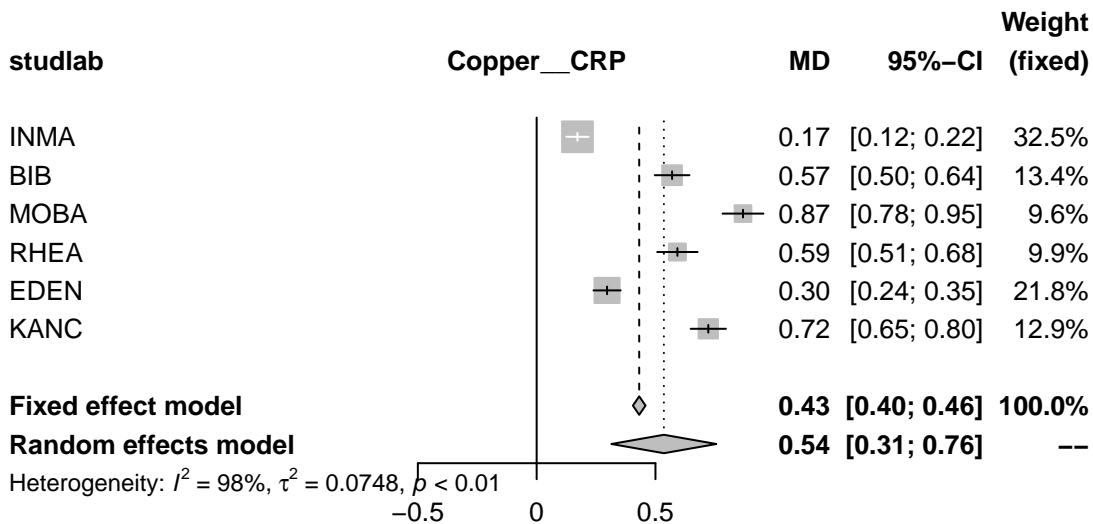

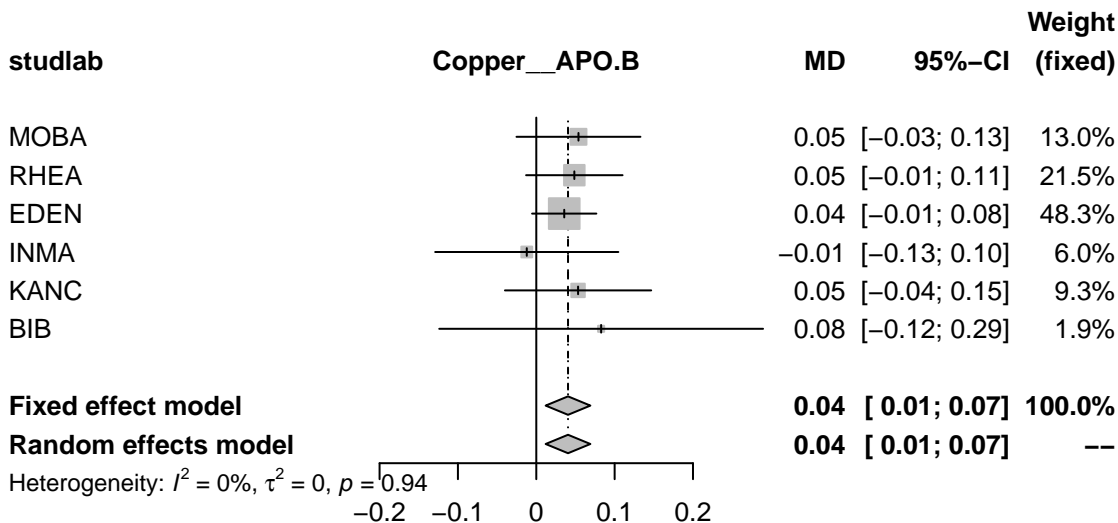

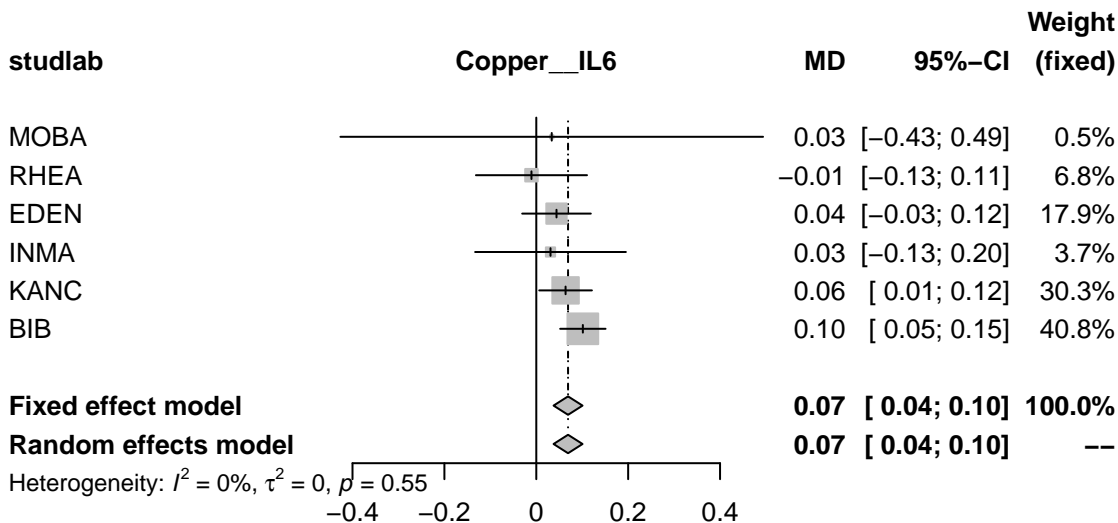

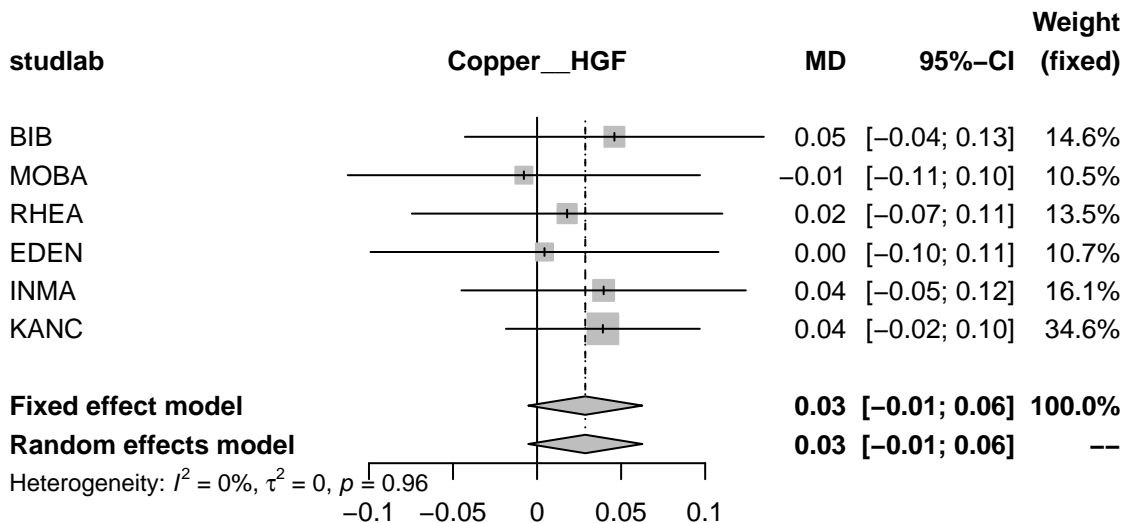

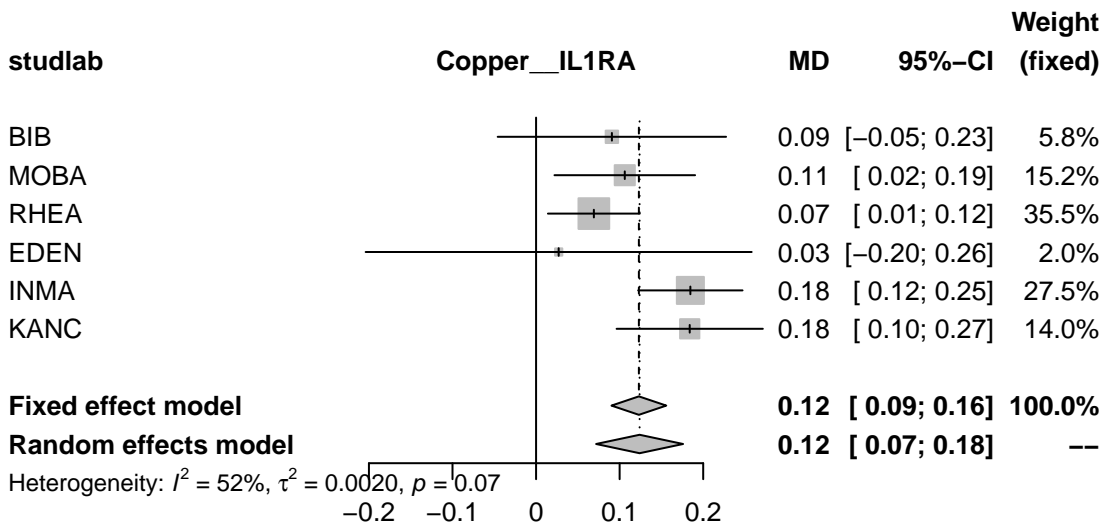

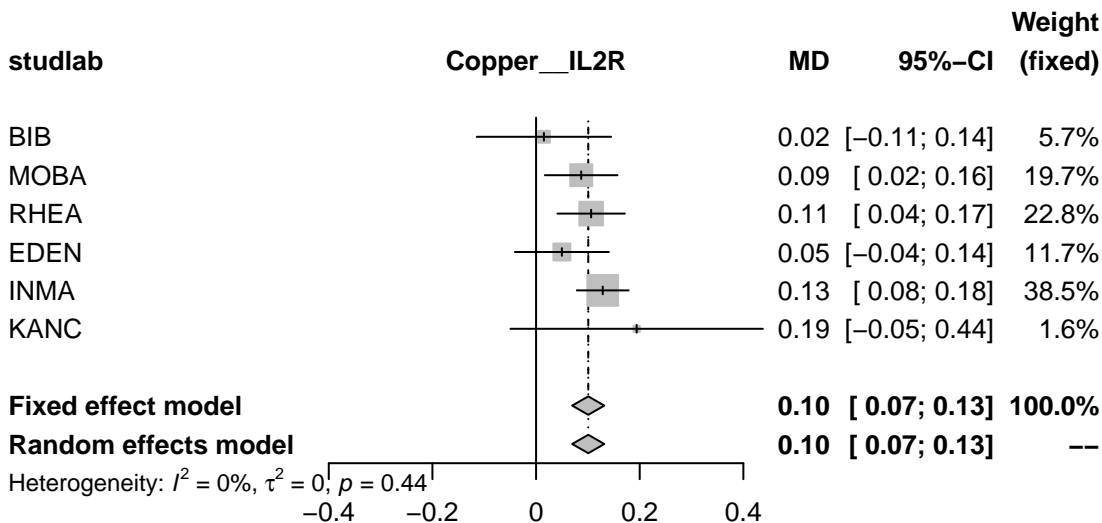

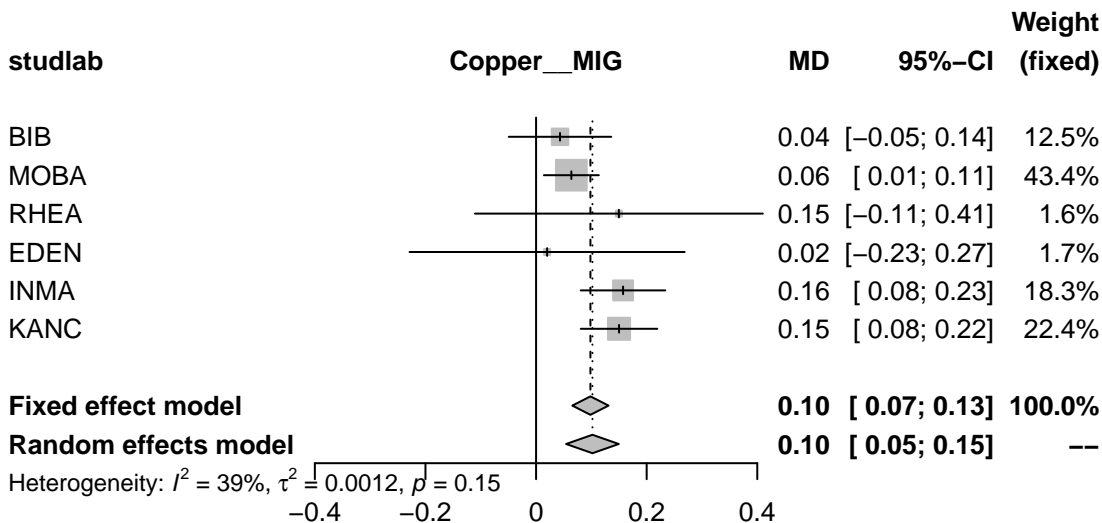

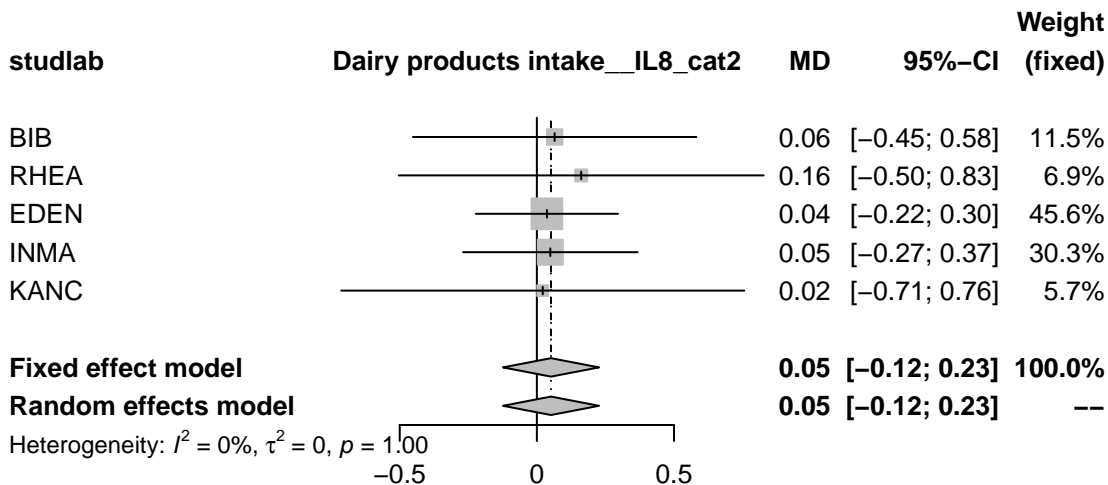

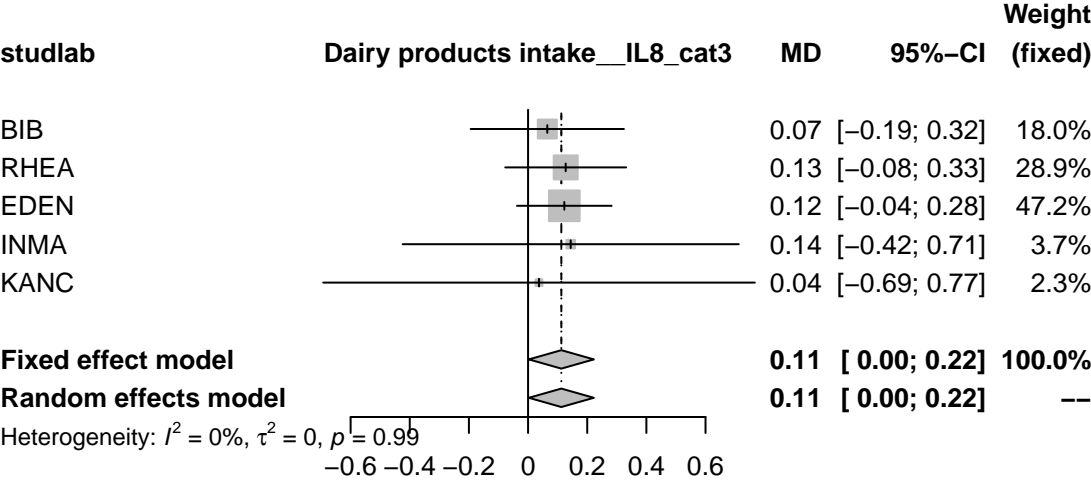

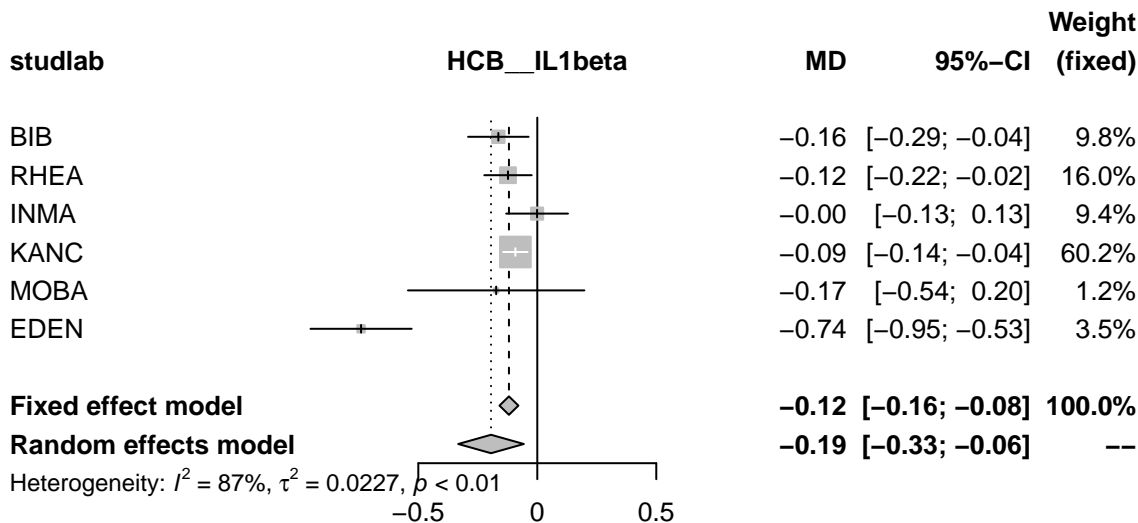

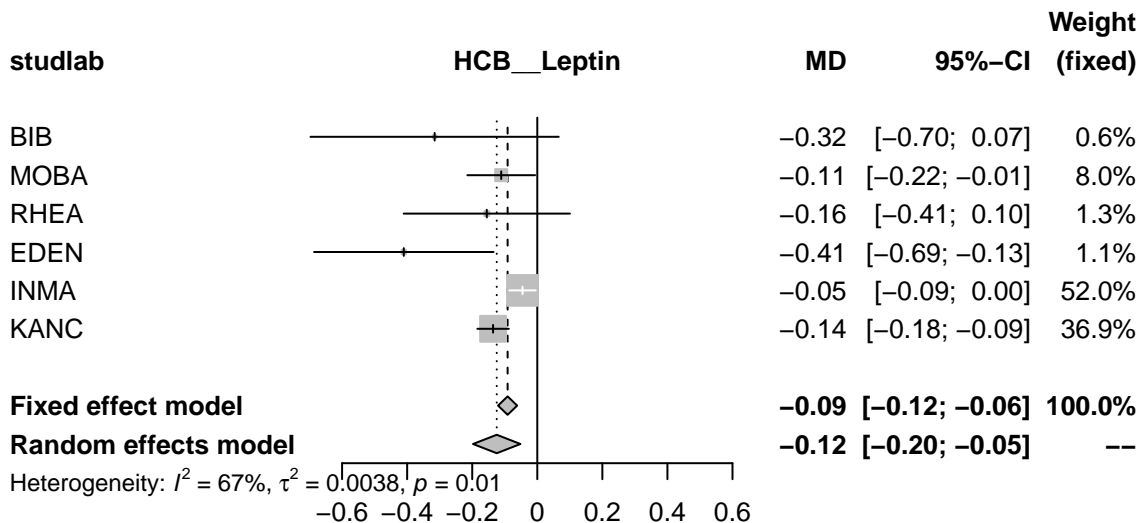

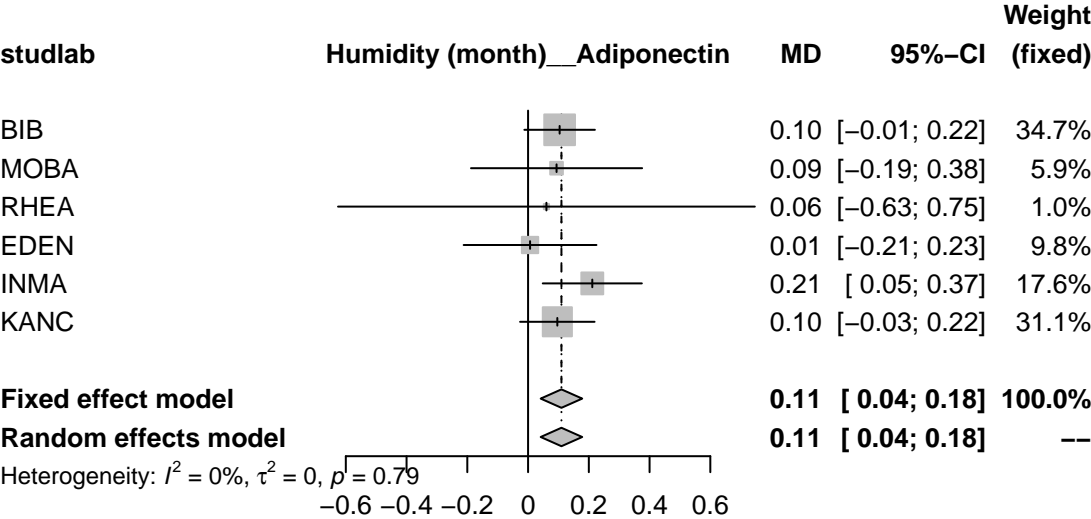

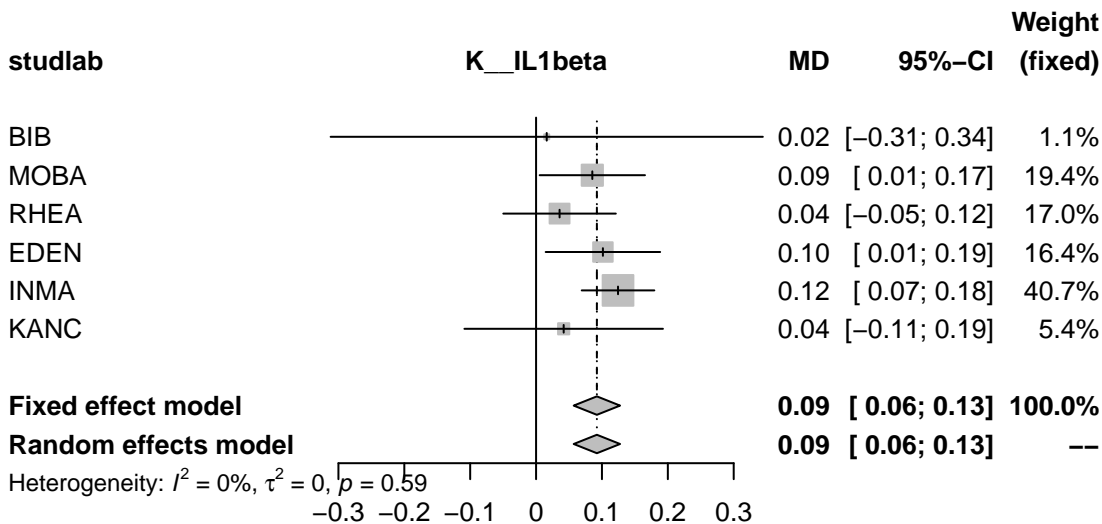

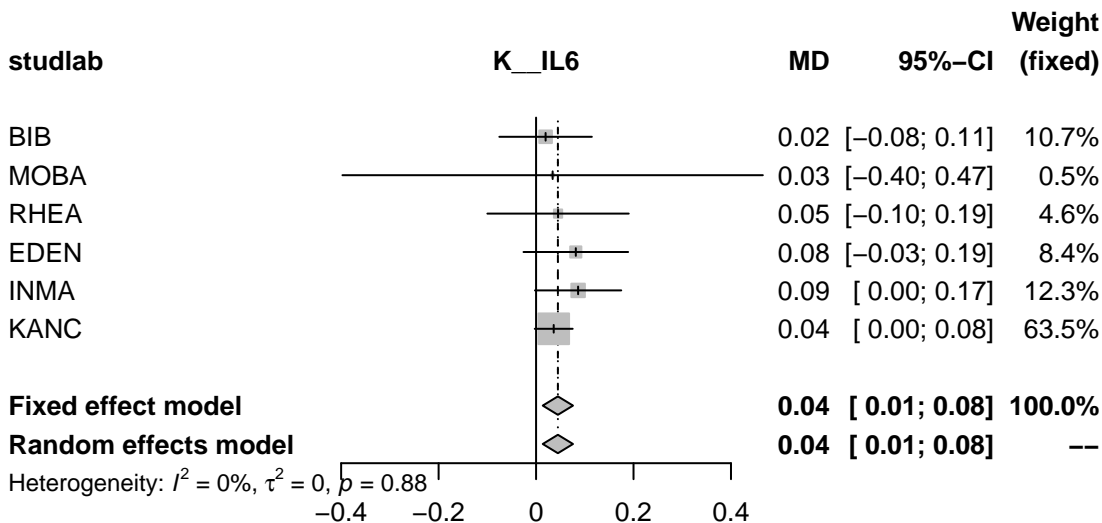

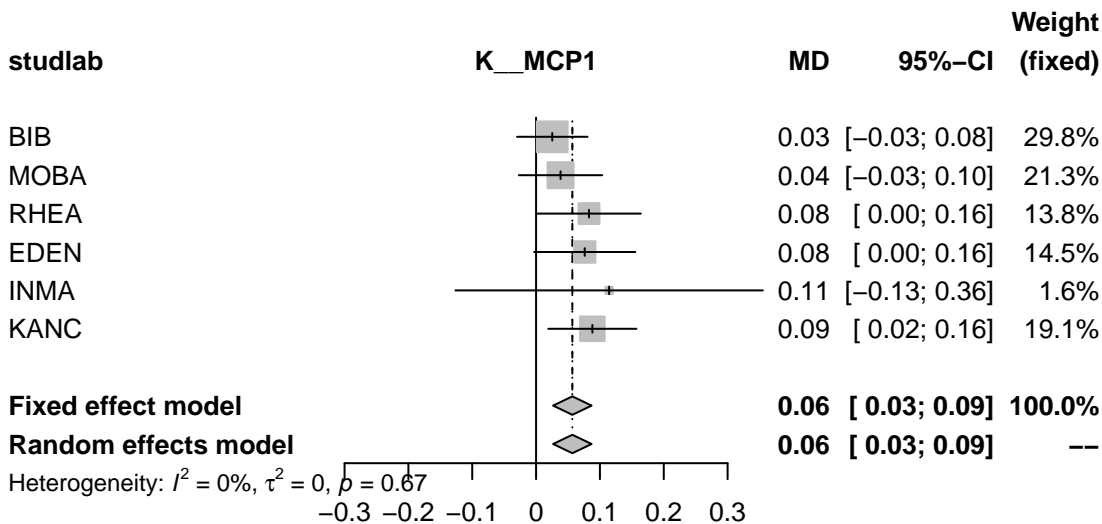

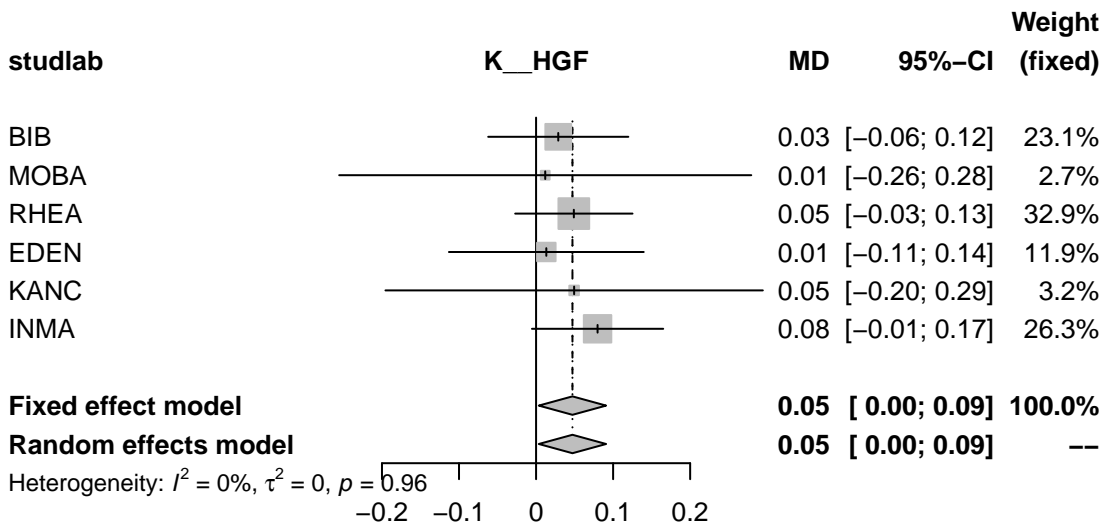

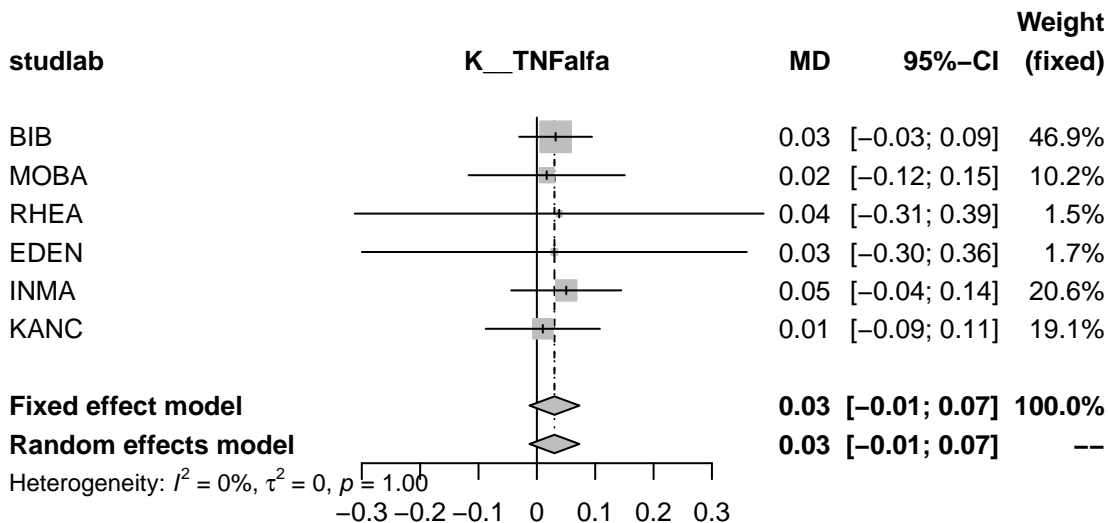

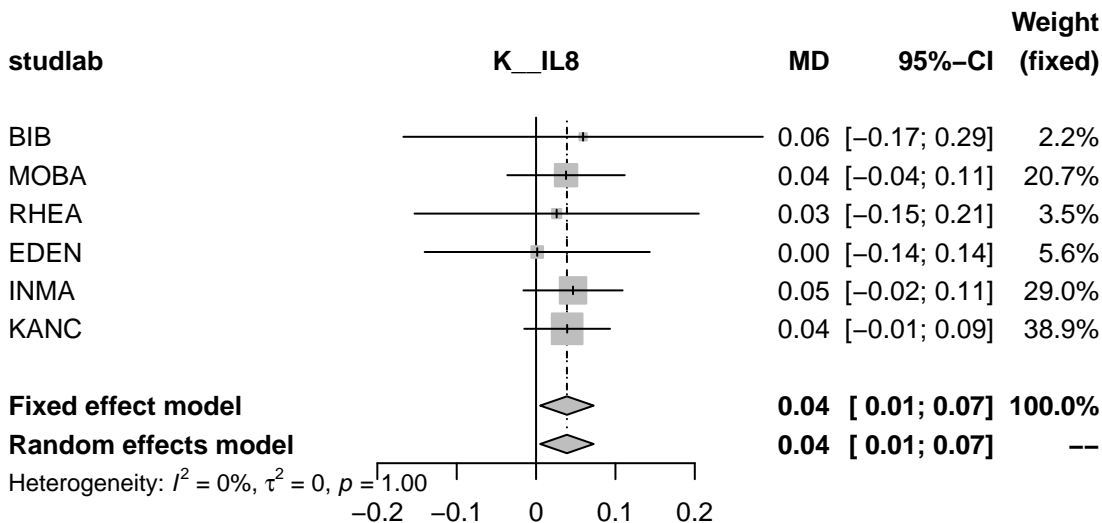

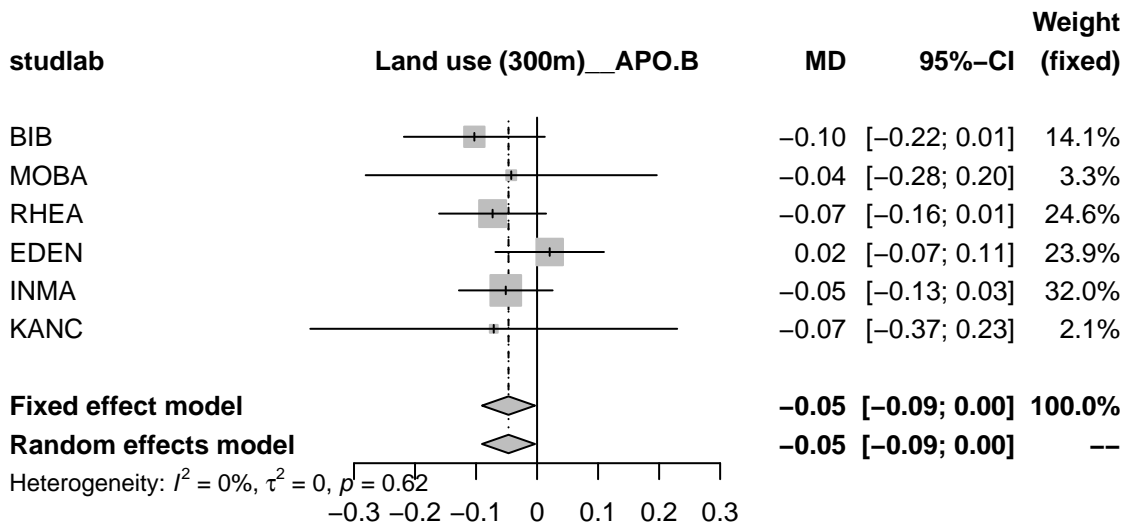

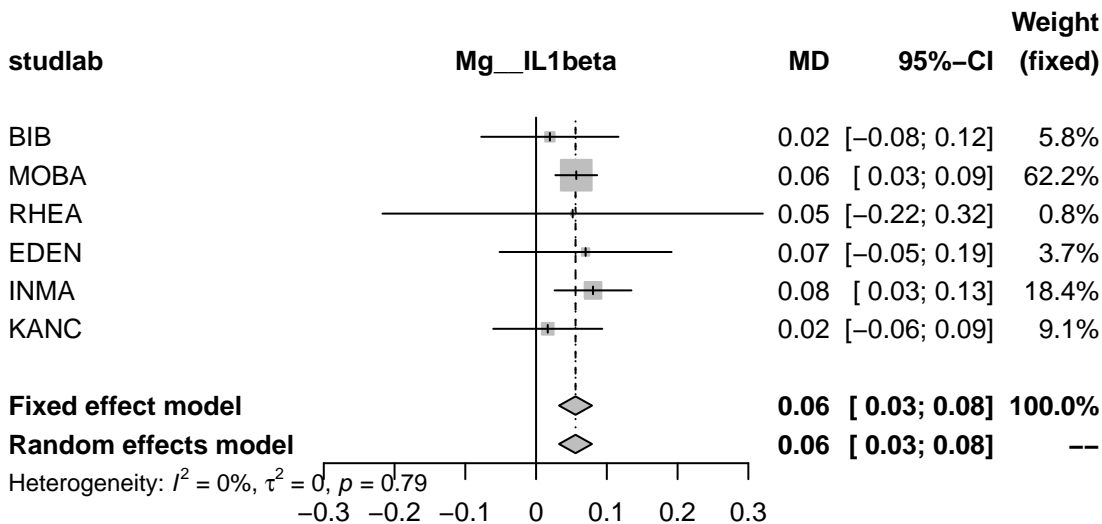

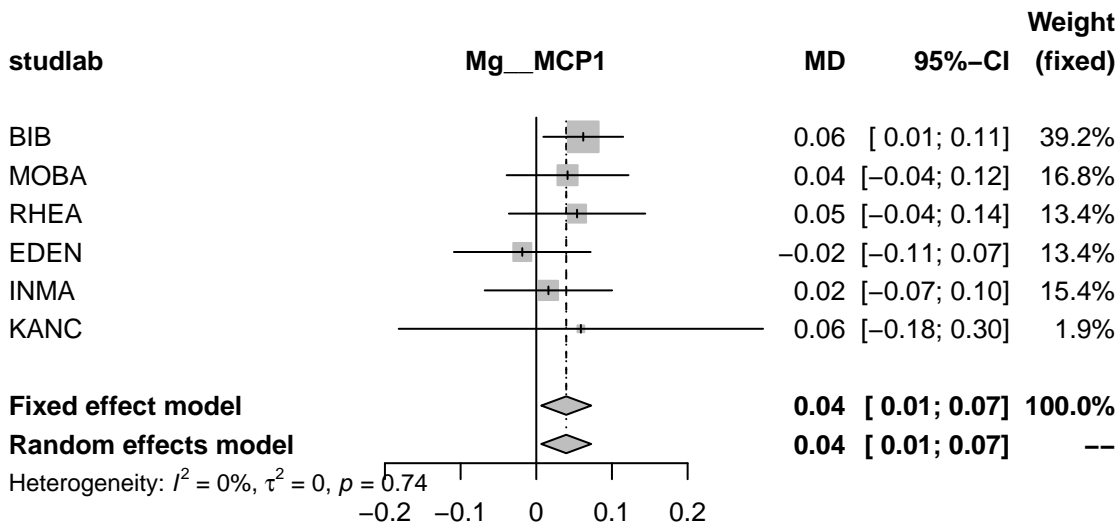

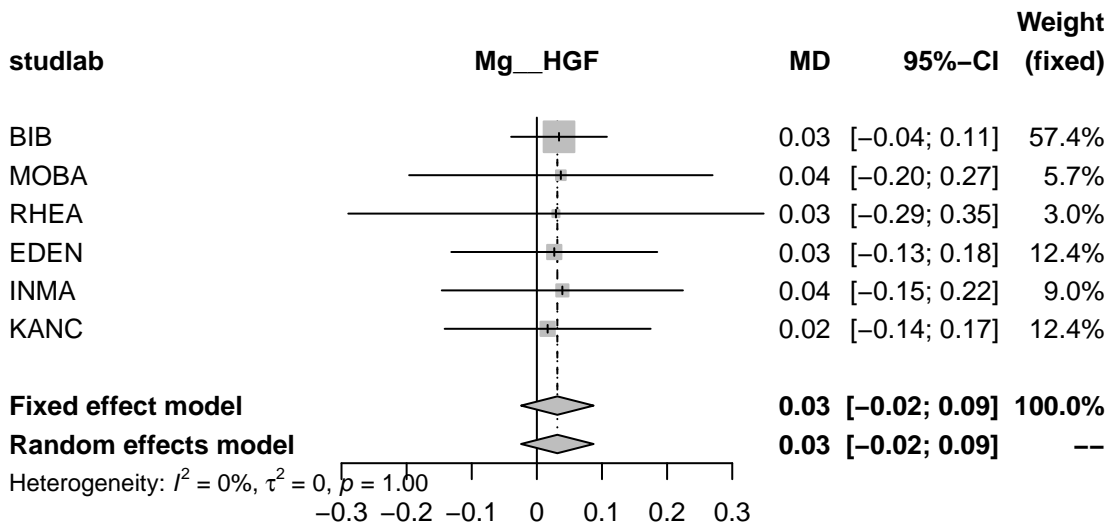

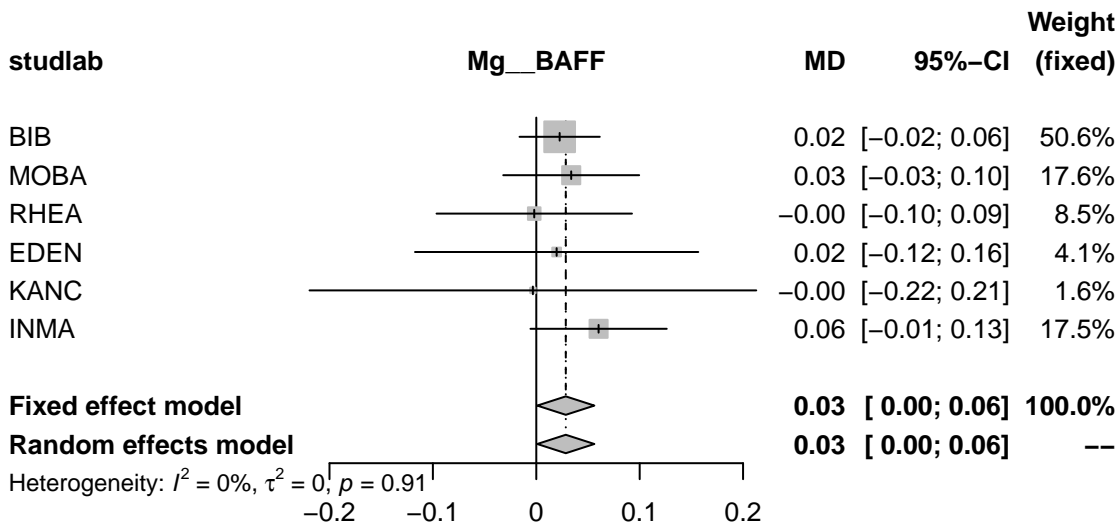

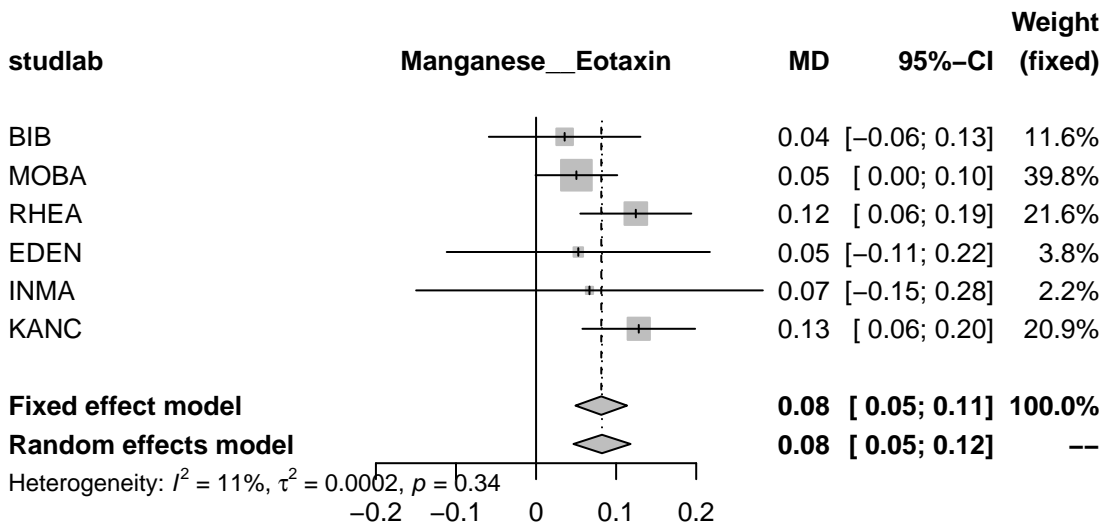

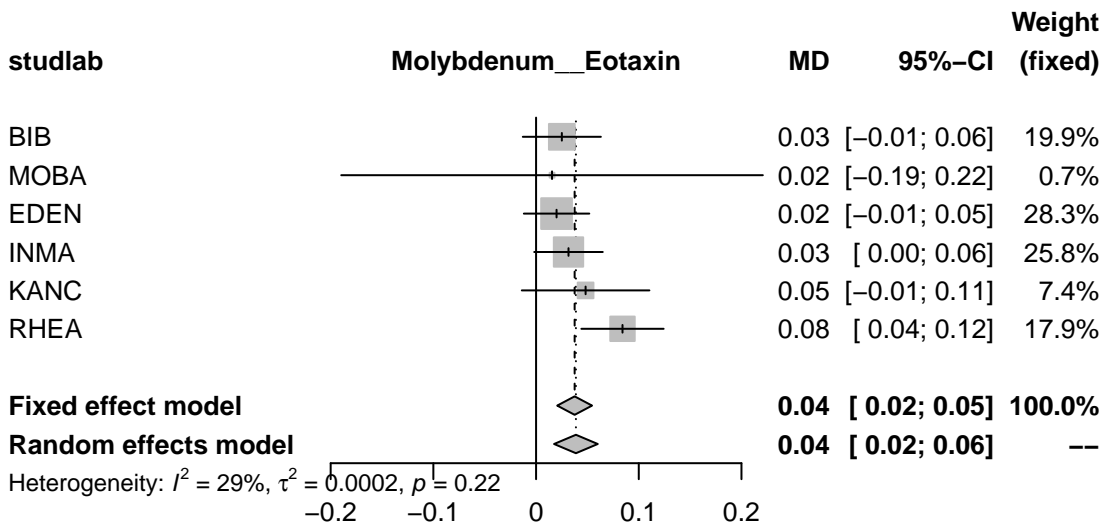

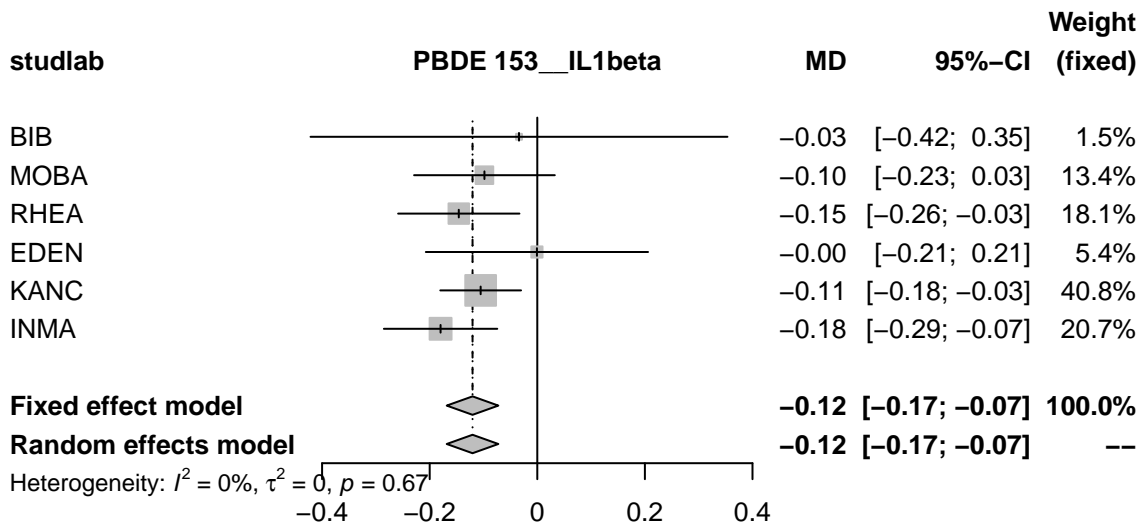

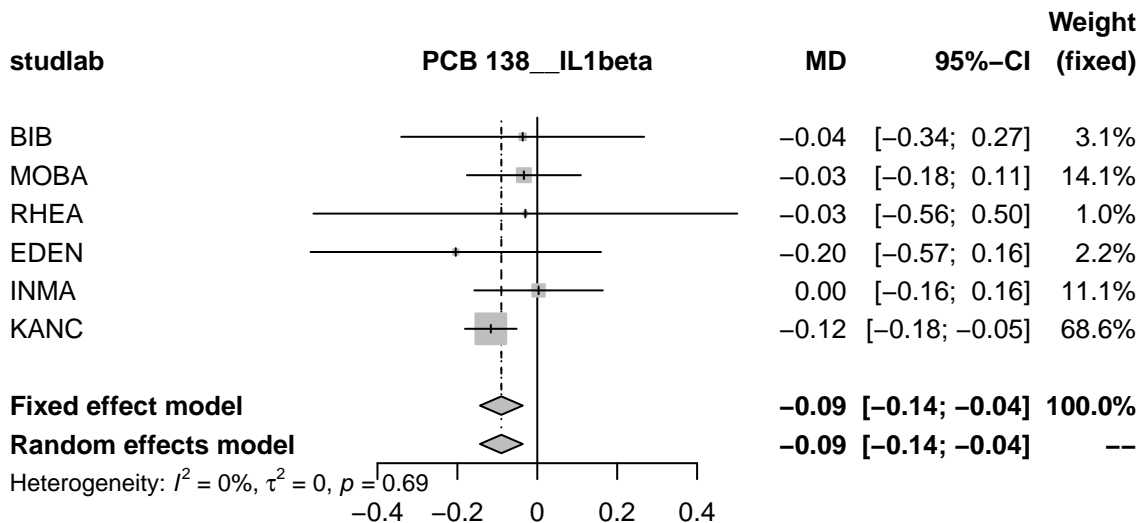

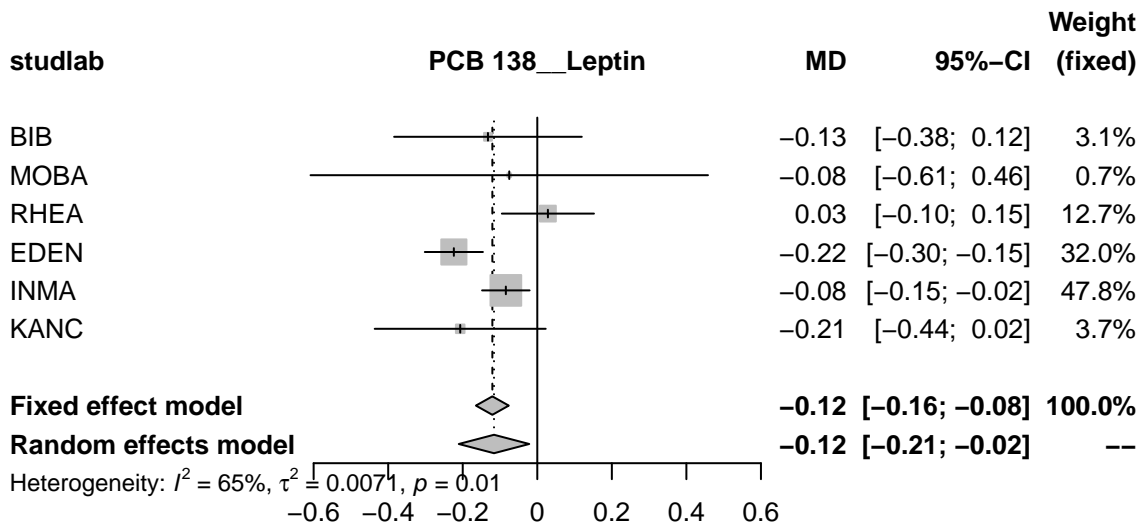

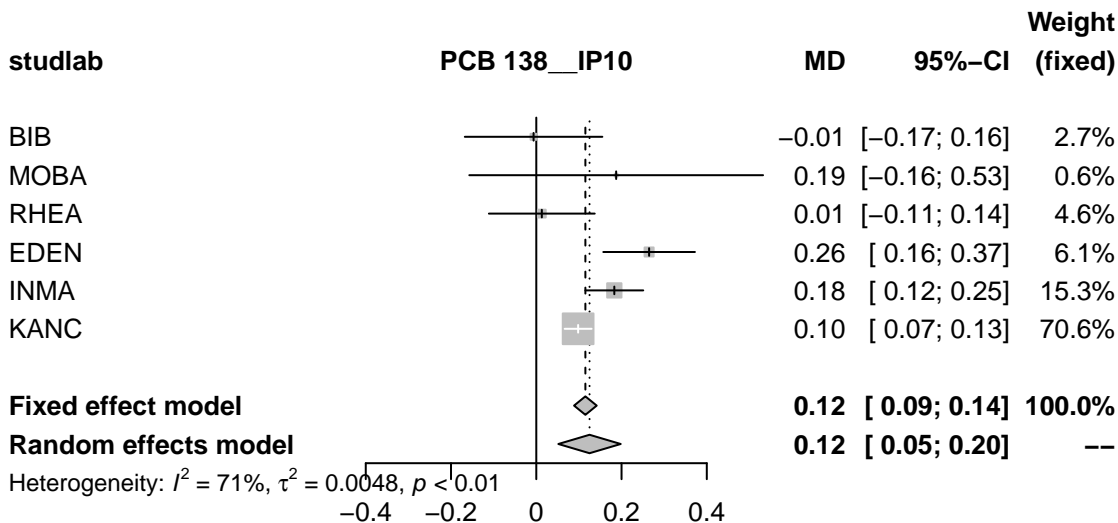

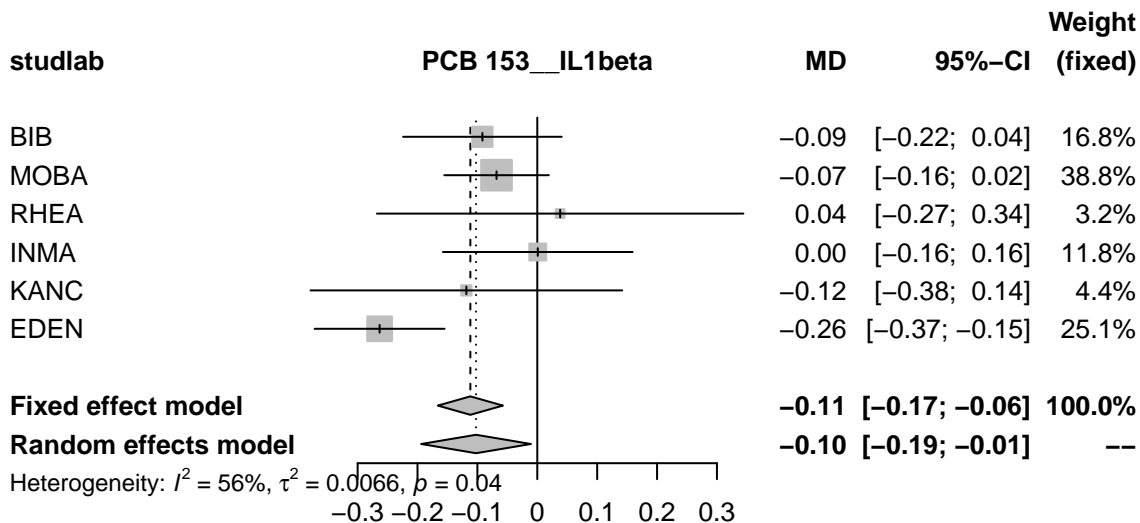

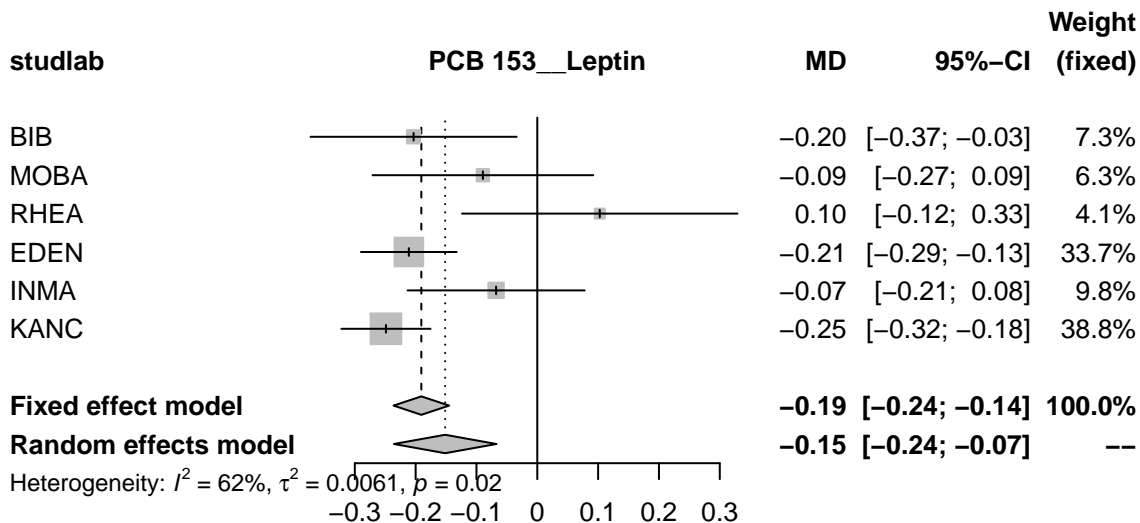

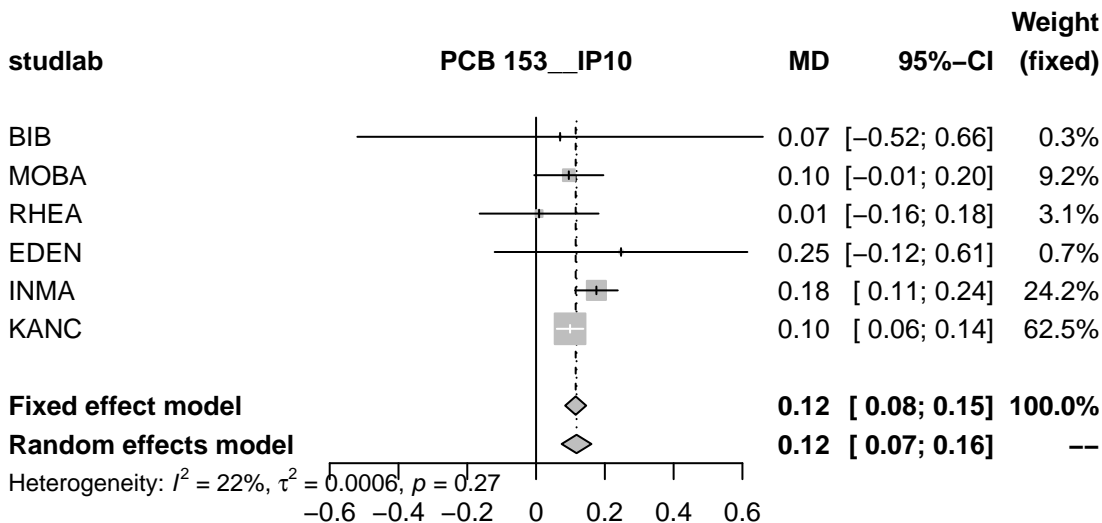

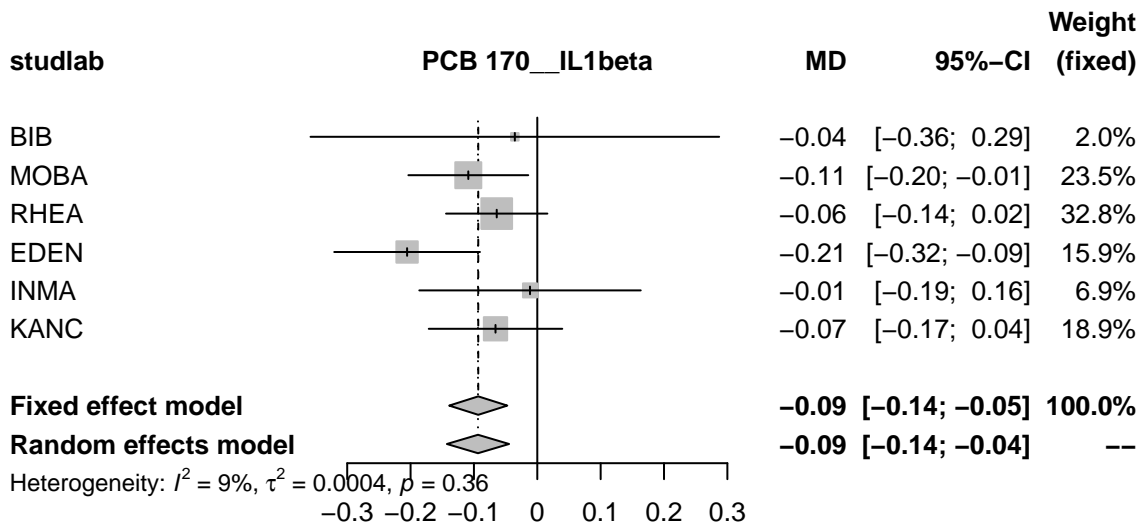

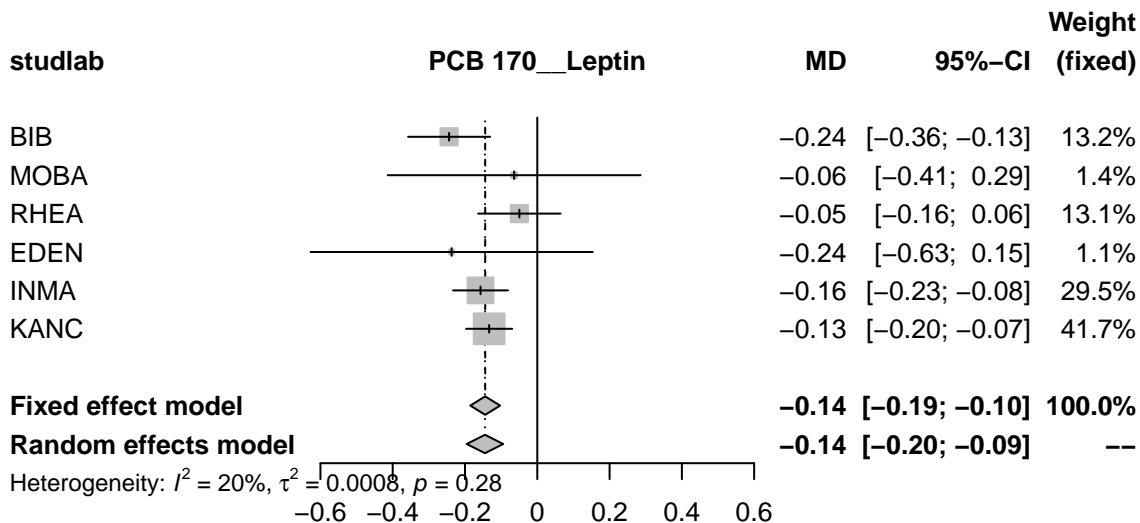

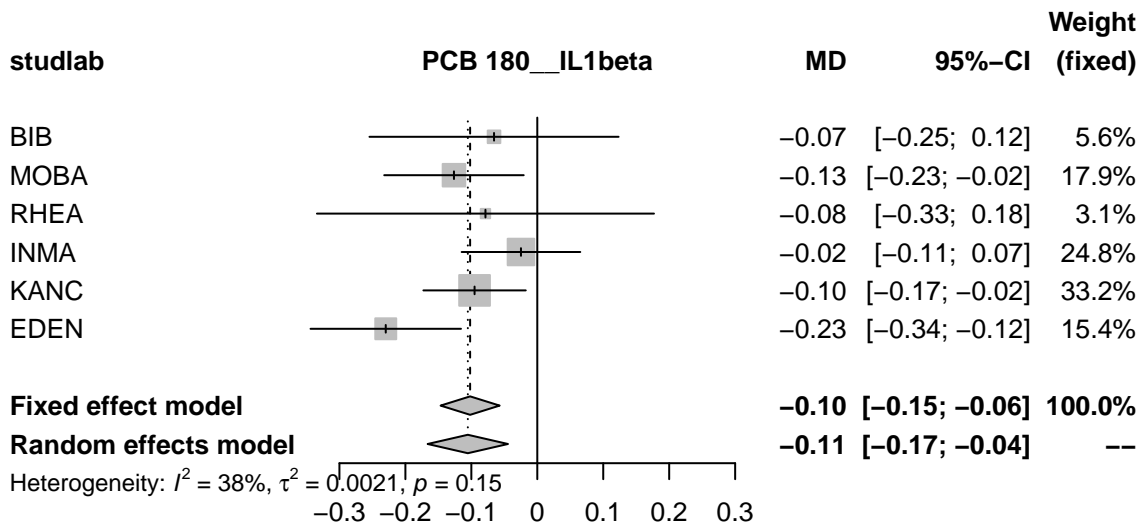

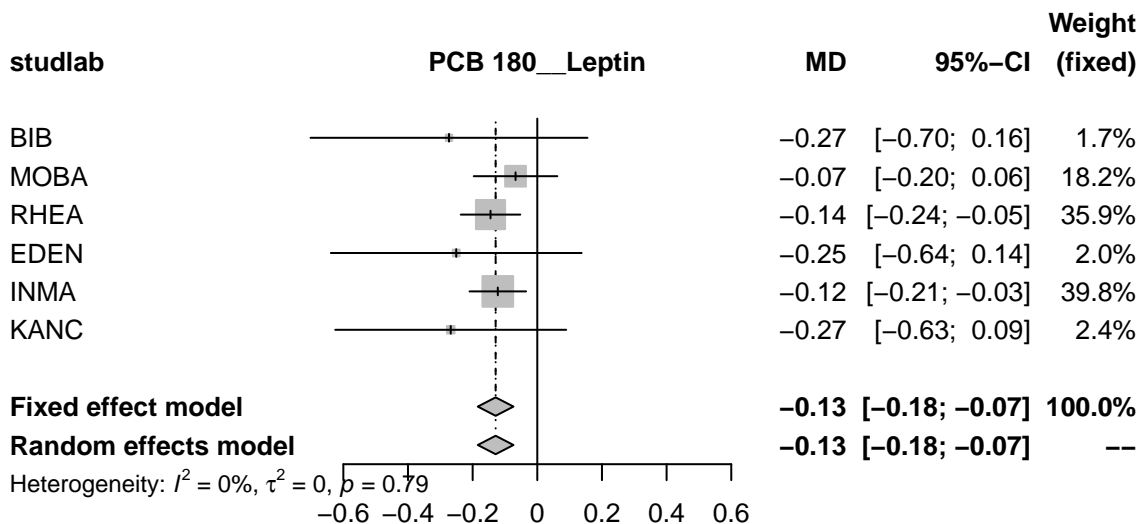

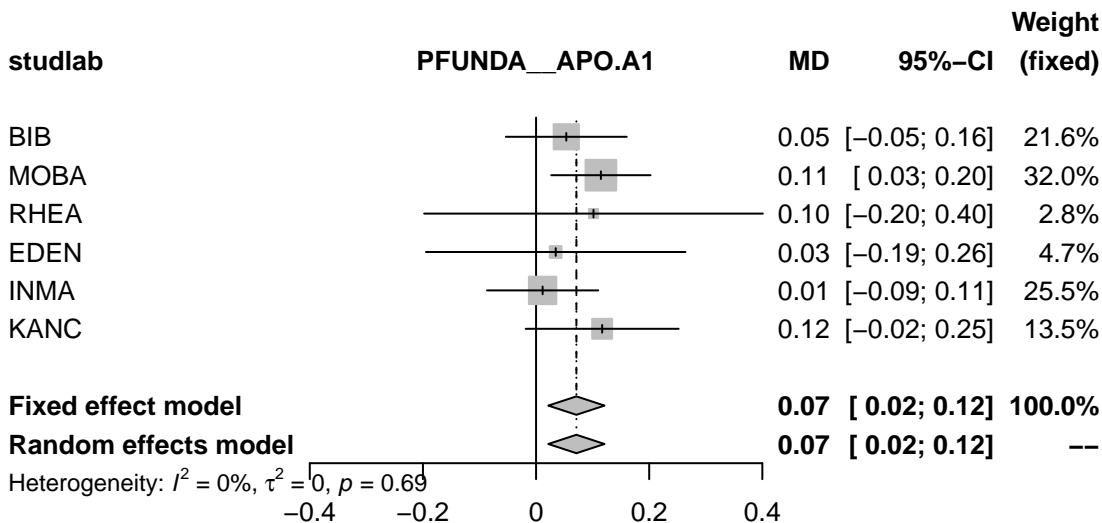

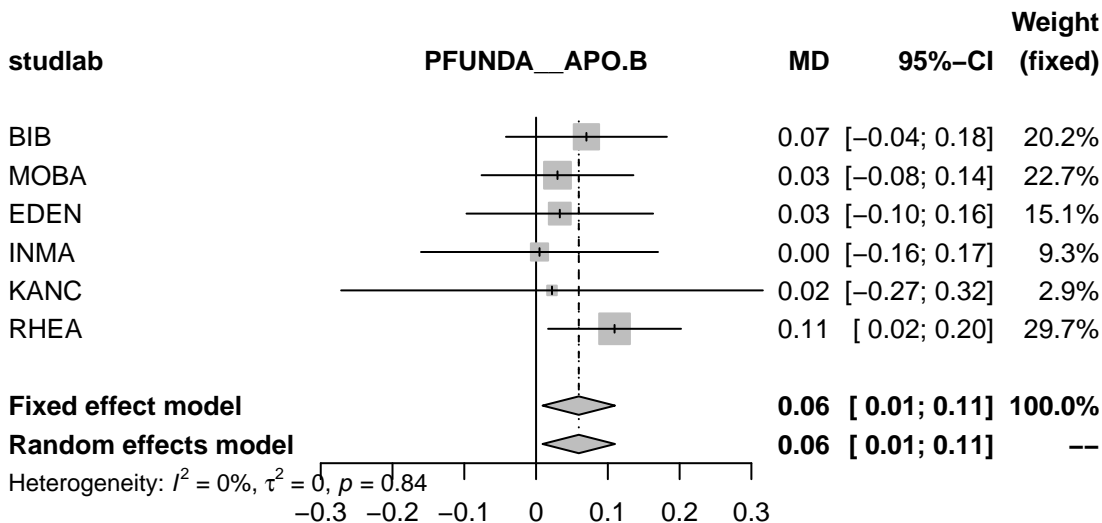

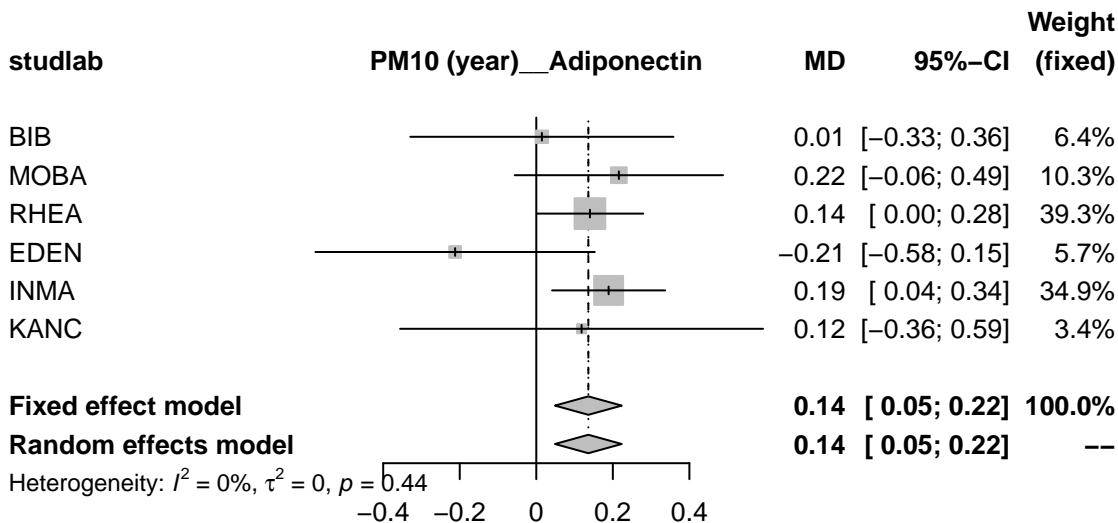

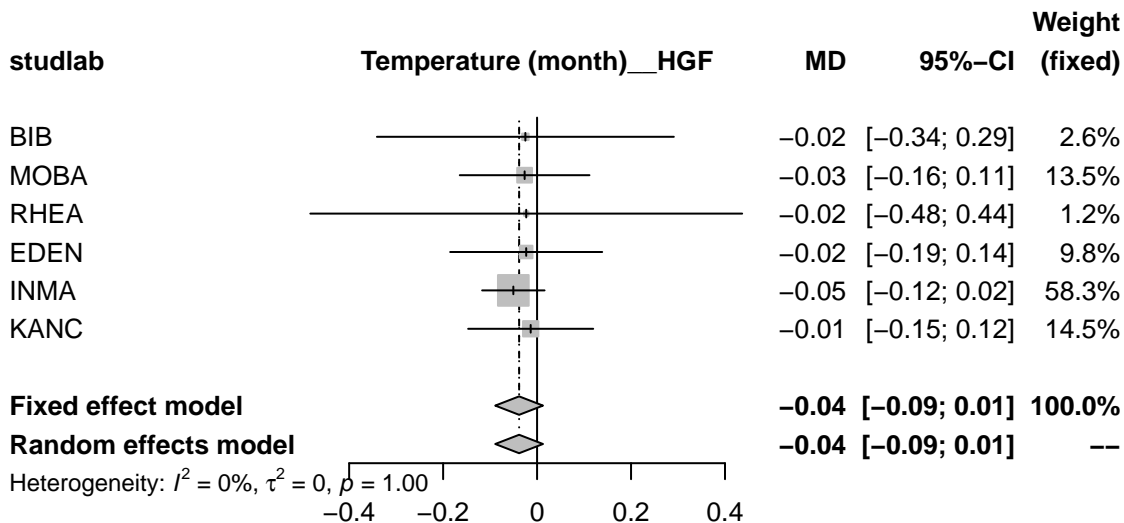

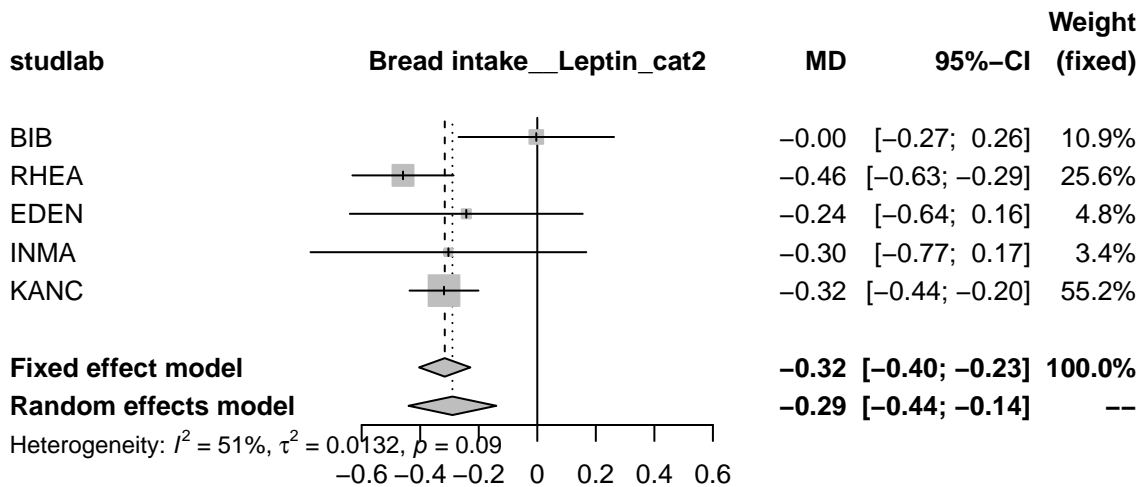

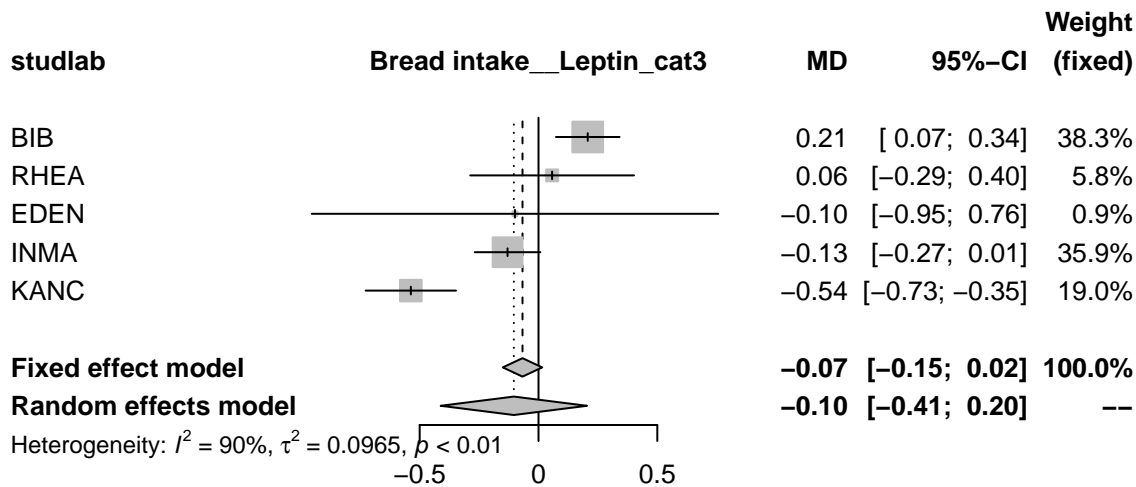

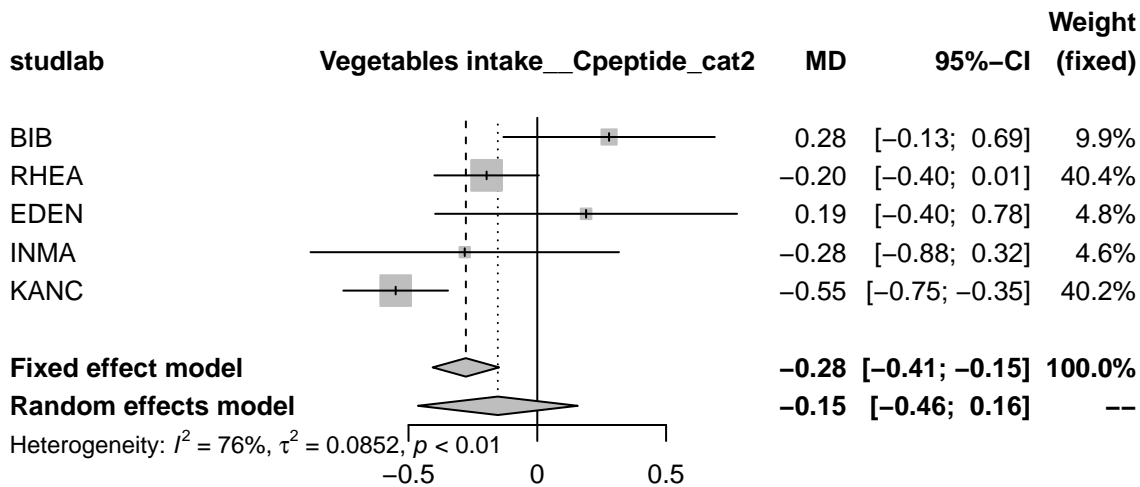

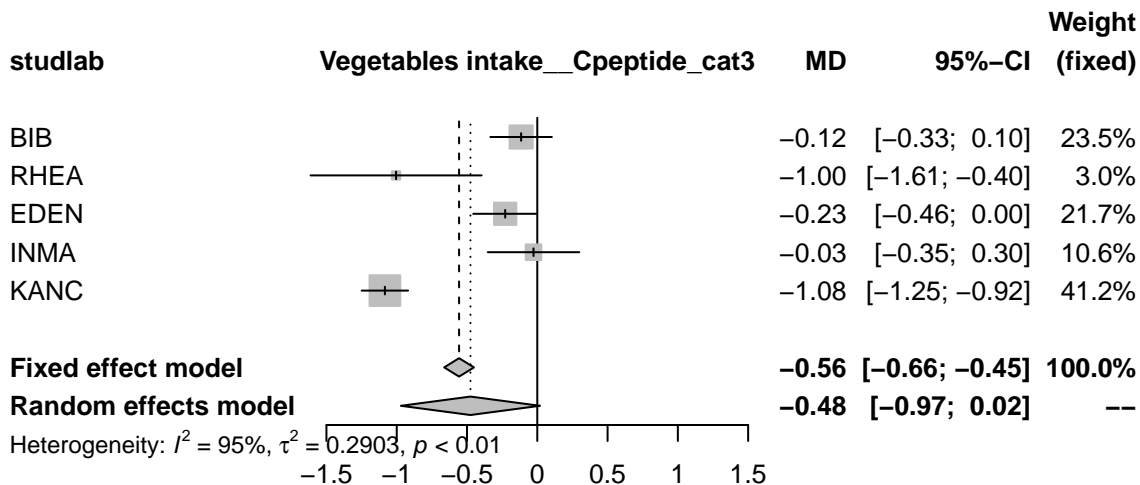

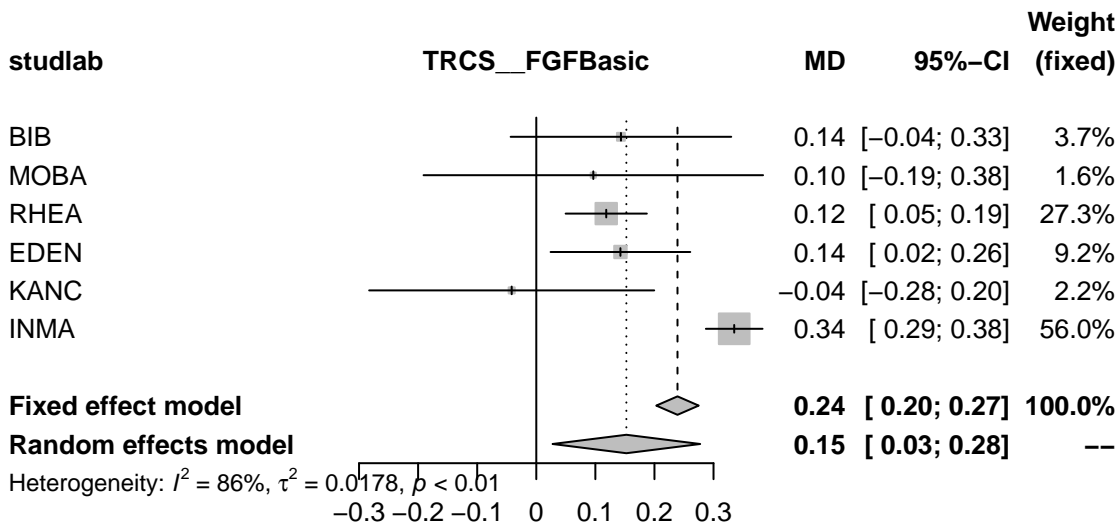

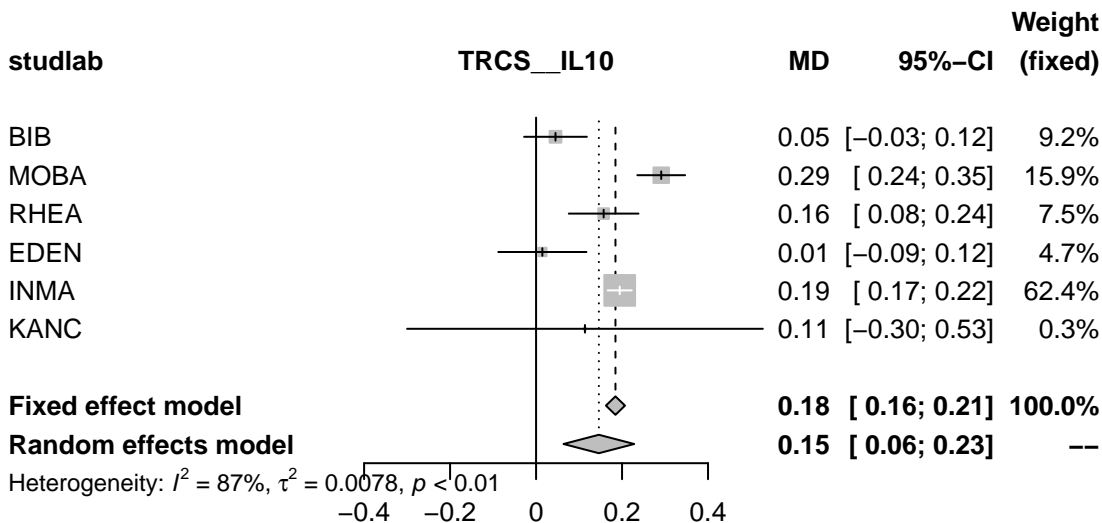

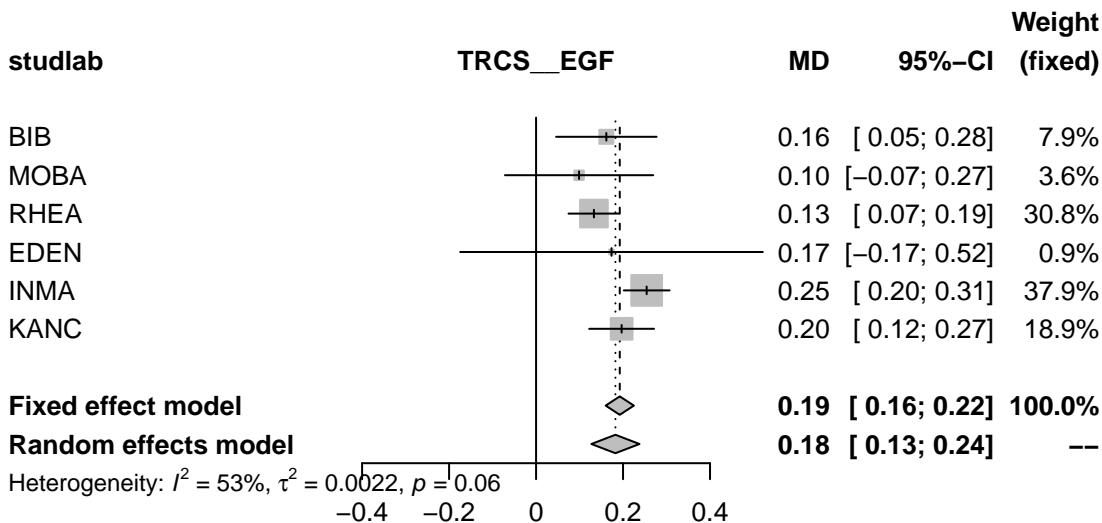

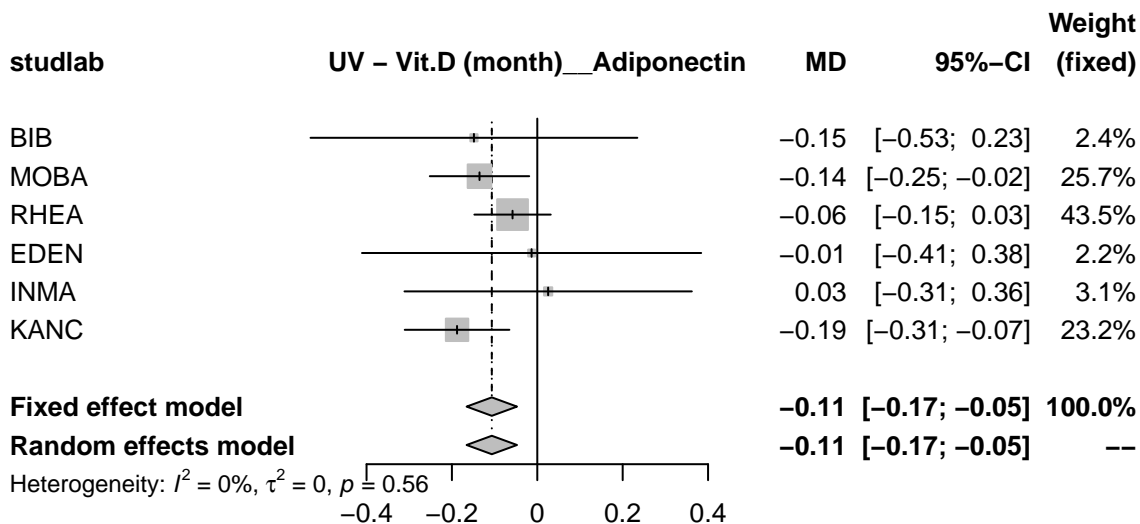

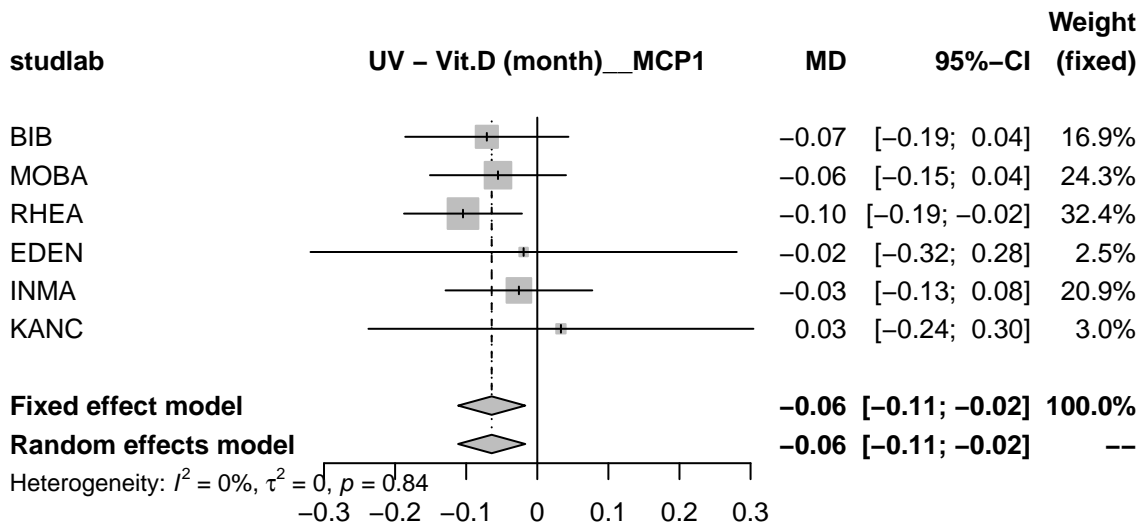

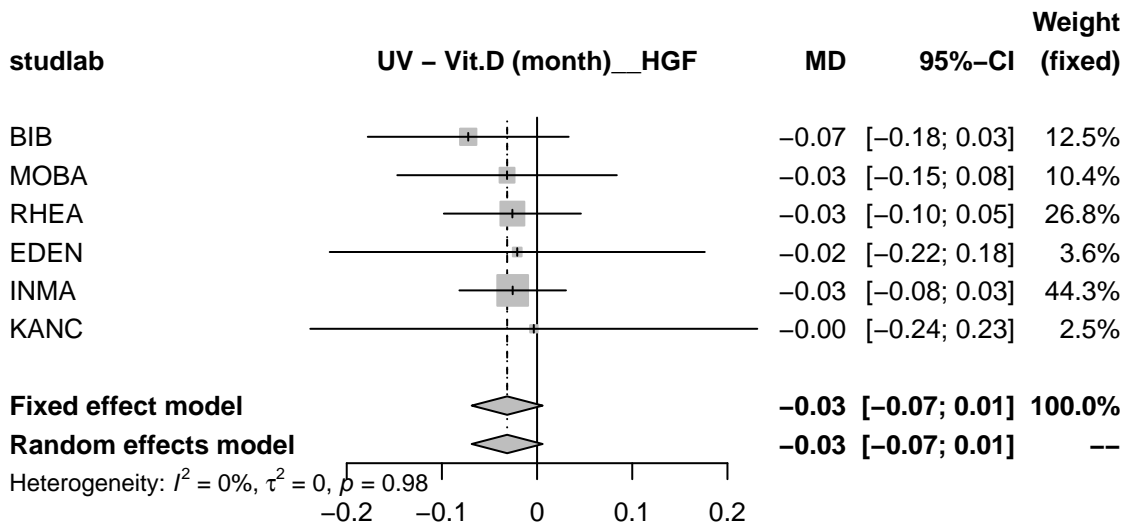

Supplement: Supplementary file 14 — Supplementary Dataset 11 [file 41467_2022_34422_MOESM14_ESM.zip › HELIX_ExpOmics_FigS2_Forestplots/HELIX_ExpOmics_FigS2J_prot_post.pdf]
