# Supplementary material for: Multi-omics signatures of the human early life exposome
Source: Nat Commun. 2022 Nov 21;13:7024. doi: 10.1038/s41467-022-34422-2 (PMC9678903; doi:10.1038/s41467-022-34422-2)
Supplement: Supplementary file 15 — Reporting Summary [file 41467_2022_34422_MOESM15_ESM.pdf]

nature research

Corresponding author(s): Martine Vrijheid

Last updated by author(s): Oct 10, 2022

Reporting Summary

Nature Research wishes to improve the reproducibility of the work that we publish. This form provides structure for consistency and transparency in reporting. For further information on Nature Research policies, see our Editorial Policies and the Editorial Policy Checklist.

Statistics

For all statistical analyses, confirm that the following items are present in the figure legend, table legend, main text, or Methods section.

n/a	Confirmed	
☐	☒	The exact sample size (*n*) for each experimental group/condition, given as a discrete number and unit of measurement
☐	☒	A statement on whether measurements were taken from distinct samples or whether the same sample was measured repeatedly
☐	☒	The statistical test(s) used AND whether they are one- or two-sided<br>*Only common tests should be described solely by name; describe more complex techniques in the Methods section.*
☐	☒	A description of all covariates tested
☐	☒	A description of any assumptions or corrections, such as tests of normality and adjustment for multiple comparisons
☐	☒	A full description of the statistical parameters including central tendency (e.g. means) or other basic estimates (e.g. regression coefficient) AND variation (e.g. standard deviation) or associated estimates of uncertainty (e.g. confidence intervals)
☐	☒	For null hypothesis testing, the test statistic (e.g. F, t, r) with confidence intervals, effect sizes, degrees of freedom and P value noted<br>*Give P values as exact values whenever suitable.*
☒	☐	For Bayesian analysis, information on the choice of priors and Markov chain Monte Carlo settings
☐	☒	For hierarchical and complex designs, identification of the appropriate level for tests and full reporting of outcomes
☐	☒	Estimates of effect sizes (e.g. Cohen's *d*, Pearson's *r*), indicating how they were calculated

Our web collection on statistics for biologists contains articles on many of the points above.

Software and code

Policy information about availability of computer code

Data collection

Missing exposome data were imputed using a chained equations method implemented in the mice v3.4.0 R package (Buuren et al. 2011).
Several tools were used for the quality control of omics data. They are described in Supplemental Information.

References:
Buuren, S. van & Groothuis-Oudshoorn, K. mice : Multivariate Imputation by Chained Equations in R. J. Stat. Softw. 45, 1–67 (2011).

Data analysis

To test the relationship between the pregnancy and childhood exposomes and molecular features, we fitted linear regressions between each exposure variable and each molecular feature adjusting for covariates, using the limma v3.46.0 R package (Ritchie et al., 2015) implemented in omicRexposome v1.12.1 (Hernandez-Ferrer et al., 2019). Multiple testing correction was applied for each exposure and within each omics layer. For methylation, gene expression and miRNAs we used the False Discovery Rate (FDR) - Benjamini Hochberg (BH) method (Benjamini & Hochberg, 1995). For other omics, proteins, urine and serum metabolites, we calculated the effective number of tests (ENT) which is based on the correlation structure of the data (Li et al., 2012), and divided the nominal p-value (0.05) by that number.
Top hit associations were run by cohort and combined through fixed- and random-effects inverse variance weighted meta-analyses using the meta v4.16-1 R package (Schwarzer et al., 2007), and forest-plots were visually inspected. I2 was used to evaluate heterogeneity in the results across cohorts.

Networks visualization was carried out using Cytoscape v3.9.0 (http://cytoscape.org) and were automatically arranged using the Cytoscape force-directed layout which aims to highlight the underlying topology of the graph (Shannon et al., 2012). The association effect size was set as the numeric edge column to use as a weight for the length of the edges. In order to find densely connected regions in the network, clustering of the childhood network was done based on Community Clustering (GLay) using clusterMaker2 v2.0 (Newman et al, 2003; Su et al., 2010).

ClusterProfiler v3.8.0 R package (Yu et al., 2018) was used to check whether this list of genes was enriched for gene-sets (Gene Ontology (GO) Biological Processes terms, KEGG, Molecular Signatures Database - C2 curated gene-sets), diseases (DisGeNET), and transcription factor and

miRNA binding motifs (Molecular Signatures Database - C3 motifs and transcription factors motifs). To identify experimentally validated target genes for miRNAs we used miRwalk v3 (Sticht et al., 2018).

Additional tools were used for visualization of the data which are reported in Supplemental Information.

References:
Benjamini, Y. & Hochberg, Y. Controlling the False Discovery Rate: A Practical and Powerful Approach to Multiple Testing. Journal of the Royal Statistical Society. Series B (Methodological) vol. 57 289–300 (1995).
Hernandez-Ferrer, C., Wellenius, G.A., Tamayo, I., Basagaña, X., Sunyer, J., Vrijheid, M., and Gonzalez, J.R. (2019). Comprehensive study of the exposome and omic data using rexposome Bioconductor packages. Bioinformatics.
Li, M.-X., Yeung, J. M. Y., Cherny, S. S. & Sham, P. C. Evaluating the effective numbers of independent tests and significant p-value thresholds in commercial genotyping arrays and public imputation reference datasets. Hum. Genet. 131, 747–56 (2012).
Newman, M.E.J., and Girvan, M. (2003). Finding and evaluating community structure in networks.
Ritchie, M.E., Phipson, B., Wu, D., Hu, Y., Law, C.W., Shi, W., and Smyth, G.K. (2015). limma powers differential expression analyses for RNA-sequencing and microarray studies. Nucleic Acids Res. 43, e47.
Schwarzer, G. Package ' meta '. R News (2007). doi:10.1007/978-3-319-21416-0>.
Shannon, P., Markiel, A., Ozier, O., Baliga, N.S., Wang, J.T., Ramage, D., Amin, N., Schwikowski, B., and Ideker, T. (2003). Cytoscape: a software environment for integrated models of biomolecular interaction networks. Genome Res. 13, 2498–2504.
Sticht, C., De La Torre, C., Parveen, A. & Gretz, N. Mirwalk: An online resource for prediction of microrna binding sites. PLoS One (2018) doi:10.1371/journal.pone.0206239.
Su, G., Kuchinsky, A., Morris, J.H., States, D.J., and Meng, F. (2010). GLay: community structure analysis of biological networks. Bioinformatics 26, 3135–3137.
Yu, G. clusterProfiler: universal enrichment tool for functional and comparative study. bioRxiv (2018) doi:10.1101/256784.

For manuscripts utilizing custom algorithms or software that are central to the research but not yet described in published literature, software must be made available to editors and reviewers. We strongly encourage code deposition in a community repository (e.g. GitHub). See the Nature Research guidelines for submitting code & software for further information.

Data

Policy information about availability of data

All manuscripts must include a data availability statement. This statement should provide the following information, where applicable:

- Accession codes, unique identifiers, or web links for publicly available datasets
- A list of figures that have associated raw data
- A description of any restrictions on data availability

The summarized results (exposure, omics biomarker, effect, standard error, p-value) generated during this study are available at https://helixomics.isglobal.org/. The raw data supporting the current study are available from the corresponding author on request subject to ethical and legislative review. The "HELIX Data External Data Request Procedures" are available with the data inventory in this website: http://www.projecthelix.eu/data-inventory.

The following data bases were used for biological interpretion of the findings:
1) Comparison with literature:
EWAS Catalog (http://ewascatalog.org/) (Battram et al. 2021)
EWAS Atlas (http://bigd.big.ac.cn/ewas/index) (Li et al. 2019)
The Exposome Explorer database (http://exposome-explorer.iarc.fr/) (Neveu et al. 2017; Neveu et al. 2020).
2) Enrichment analyses with ClusterProfiler v3.8.0 R package (Yu et al. 2018): Gene Ontology (GO) Biological Processes terms, KEGG, Molecular Signatures Database - C2 curated gene-sets), diseases (DisGeNET), and transcription factor and miRNA binding motifs (Molecular Signatures Database - C3 motifs and transcription factors motifs).
3) miRNA targets: miRwalk v3 (Sticht et al. 2018).
4) Blood cis epression quantitative methylation trait (eQTM): HELIX eQTM catalog, (https://helixomics.isglobal.org/) (Ruiz-Arenas et al. 2022).

References:
Battram, T. et al. The EWAS Catalog: a database of epigenome-wide association studies. OSF Prepr. 4 (2021) doi:10.31219/OSF.IO/837WN.
Li, M. et al. EWAS Atlas: A curated knowledgebase of epigenome-wide association studies. Nucleic Acids Res. (2019) doi:10.1093/nar/gky1027.
Neveu, V. et al. Exposome-Explorer: A manually-curated database on biomarkers of exposure to dietary and environmental factors. Nucleic Acids Res. 45, D979–D984 (2017).
Neveu, V., Nicolas, G., Salek, R. M., Wishart, D. S. & Scalbert, A. Exposome-Explorer 2.0: an update incorporating candidate dietary biomarkers and dietary associations with cancer risk. Nucleic Acids Res. 48, D908–D912 (2020).
Ruiz-Arenas, C. et al. Identification of autosomal cis expression quantitative trait methylation (cis eQTMs) in children's blood. Elife (2022) doi:10.7554/eLife.65310.
Sticht, C., De La Torre, C., Parveen, A. & Gretz, N. Mirwalk: An online resource for prediction of microrna binding sites. PLoS One (2018) doi:10.1371/journal.pone.0206239.
Yu, G. clusterProfiler: universal enrichment tool for functional and comparative study. bioRxiv (2018) doi:10.1101/256784.

Field-specific reporting

Please select the one below that is the best fit for your research. If you are not sure, read the appropriate sections before making your selection.

☒ Life sciences ☐ Behavioural & social sciences ☐ Ecological, evolutionary & environmental sciences

For a reference copy of the document with all sections, see nature.com/documents/nr-reporting-summary-flat.pdf

Life sciences study design

All studies must disclose on these points even when the disclosure is negative.

Sample size	The HELIX subcohort of 1,301 mother–child pairs was nested within the entire cohorts by selection of approximately 200 pairs from each cohort. Eligibility criteria included a) age 6–9 years, 7–8 years, if possible; the age range was as narrow as possible for comparability of omics analyses and exposure-related behavior; b) stored pregnancy blood and urine samples available, and available sample volume sufficient for the analysis of exposure biomarkers 1; c) complete address history available from first to last follow-up point; d) no serious health problems that, in the opinion of a local clinician, may affect the performance of the clinical testing (e.g., spirometry) or affect the volunteer's safety (e.g., renal failure, pneumonia). In addition, the selection considered whether data on important covariates (genetic data, diet, socioeconomic factors) were available. Cohorts with more than the required number of mother–child pairs that meet these criteria invited subjects at random from the eligible pool. The following sample size calculation for the HELIX subcohort was made as follow: With a sample size of 1,200 (subcohort, which was lower than the finally reached number of chidlren of 1,300), the agnostic EWAS analysis with control for false discovery rate will have a power of 80% to detect a 3-point difference in a continuous outcome variable with a standard deviation of 15 (as in common neurodevelopment indexes), considering that 15% of the tested exposures will show an association (Liu and Hwang 2007).<br><br>Reference:<br>Liu P, Hwang JT. Quick calculation for sample size while controlling false discovery rate with application to microarray analysis. Bioinformatics. 2007;23(6):739–746
Data exclusions	The study includes complete cases for each omics layer. First, we selected 1301 mother-child pairs with complete questionnaire and clinical examination data, and urine and blood samples. Then, we did the quality control of each omics dataset and we end up with the following numbers: 1173 for DNA methylation, 1007 for gene expression, 941 for miRNAs, 1170 for plasma proteins, 1198 for serum metabolites and 1198 for urinary metabolites (Supplemental Data 1E).
Replication	No other studies have the rich data available in HELIX, thus replication is difficult. We compared our results with previous associations reported in EWAS and exposure-metabolomics databases and literature.
Randomization	Participants were not allocated to groups. This is an observational study.
Blinding	Participants were not allocated to groups. This is an observational study.

Reporting for specific materials, systems and methods

We require information from authors about some types of materials, experimental systems and methods used in many studies. Here, indicate whether each material, system or method listed is relevant to your study. If you are not sure if a list item applies to your research, read the appropriate section before selecting a response.

Materials & experimental systems

n/a	Involved in the study	
☒	☐	Antibodies
☒	☐	Eukaryotic cell lines
☒	☐	Palaeontology and archaeology
☒	☐	Animals and other organisms
☐	☒	Human research participants
☒	☐	Clinical data
☒	☐	Dual use research of concern

Methods

n/a	Involved in the study	
☒	☐	ChIP-seq
☒	☐	Flow cytometry
☒	☐	MRI-based neuroimaging

Human research participants

Policy information about studies involving human research participants

Population characteristics	See above
Recruitment	The study population for the entire HELIX cohort includes 31472 women who had singleton deliveries between 1999 and 2010, and for whom exposure to ambient air pollution during pregnancy had been estimated as part of the European Study of Cohorts for Air Pollution Effects (ESCAPE) project.<br><br>A subcohort of 1301 children from the entire cohort were followed up as part of the HELIX project at the mean age of 7.98 (standard deviation - SD: 1.6). They were representative of the 6 cohorts participating in HELIX and were balanced by sex (female 45.3%). 90% of them were of White European ancestry, 7.8% Pakistani or Asian and 2.2% from other ancestries. Around 28% of the children were overweight or obese and half of them (51.8%) were born from mother with a university degree or higher.<br><br>Basic characteristics of the subcohort were somewhat different to those of the entire cohort, probably reflecting selective participation of families in the intensive subcohort follow-up visit and data completeness requirements. Compared with the

entire cohort, the subcohort contained a greater percentage of boys, fewer children whose parents were born abroad (in particular in INMA and RHEA), a lower percentage of mothers with low education (in particular in BiB), a lower percentage of primiparous mothers (mainly in MoBa) and older mothers.

More information can be found in the HELIX cohort profile article:
Maitre L, de Bont J, Casas M, et alHuman Early Life Exposome (HELIX) study: a European population-based exposome cohortBMJ Open 2018;8:e021311. doi: 10.1136/bmjopen-2017-021311

Ethics oversight

Local ethical committees approved the studies that were conducted according to the guidelines laid down in the Declaration of Helsinki. The ethical committees for each cohort were the following: BIB: Bradford Teaching Hospitals NHS Foundation Trust, EDEN: Agence nationale de sécurité du médicament et des produits de santé, INMA: Comité Ético de Inverticación Clínica Parc de Salut MAR, KANC: LIETUVOS BIOETIKOS KOMITETAS, MoBa: Regional komité for medisinsk og helsefaglig forskningsetikk, Rhea: Ethical committee of the general university hospital of Heraklion, Crete. Informed consent was obtained from a parent and/or legal guardian of all participants in the study.

Note that full information on the approval of the study protocol must also be provided in the manuscript.
